# The murine MHC-E molecule Qa-1^b^ is surface displayed in a peptide-free conformation in homeostasis

**DOI:** 10.3389/fimmu.2026.1743362

**Published:** 2026-03-09

**Authors:** Gaby Schaap, Soroush Ghaffari, Jim Middelburg, Marjolein Sluijter, Lisa Griffioen, Tom A. W. Schoufour, Ruud H. M. Wijdeven, Jacques Neefjes, Ramon Arens, Jon Weidanz, Thorbald van Hall

**Affiliations:** 1 Department of Medical Oncology, Oncode Institute, Leiden University Medical Center, Leiden, Netherlands; 2 College of Nursing and Health Innovation, University of Texas at Arlington, Arlington, TX, United States; 3 Department of Cell and Chemical Biology, Oncode Institute, Leiden University Medical Center, Leiden, Netherlands; 4 Department of Immunology, Leiden University Medical Center, Leiden, Netherlands

**Keywords:** antibody, CRISPR/Cas9 genome-wide screen, non-classical MHC-E, peptide-free conformation, Qa-1^b^

## Abstract

Qa-1^b^, the murine ortholog of the nonclassical MHC-E family, contains minimal polymorphism and exhibits reduced surface stability compared with classical MHC class I molecules. To investigate Qa-1^b^ conformations and their immunological relevance, we employed two antibodies: EXX-1, which selectively recognizes Qa-1^b^ bound to the canonical leader peptide Qdm, and 6A8.6F10, a broadly used Qa-1^b^-reactive antibody. Genome-wide CRISPR screens revealed that Qdm presentation was induced by interferon-γ and required the components of the peptide-loading complex (PLC) and endoplasmic reticulum quality control. EXX-1 binding thus reflected broad cellular integrity and mirrored CD94/NKG2x receptor engagement. In contrast, 6A8.6F10 staining occurred independently of PLC components such as ERAP1 and tapasin, and intriguingly increased in their absence. Accordingly, exogenous pulsing with Qa-1^b^-binding peptides markedly reduced 6A8.6F10 antibody binding and resonance shift assays revealed that 6A8.6F10 selectively recognizes peptide-deficient Qa-1^b^ complexes. These findings suggest an additional layer of regulation beyond the immune checkpoint NKG2x/CD94, involving peptide-free MHC-E.

## Introduction

MHC-E molecules are non-polymorphic MHC class I proteins in mammalian species. In human, only two alleles of HLA-E exist world-wide and outbred mice similarly encode two prototypic alleles, Qa-1^a^ and Qa-1^b^ (1, 2). Although additional allelic variants have been detected in some non-human primates, the overall genetic diversity of MHC-E remains minimal compared to the extensive polymorphism of classical class I loci (2, 3). Interestingly, the canonical leader peptides accommodated in MHC-E are also remarkably conserved, even across mammalian species (4). These dominant peptides, referred to as ‘Qdm’ in mice (for Qa-1 determinant modifier) and ‘VL9’ in humans, stabilize MHC-E complexes. The fold of this class I complex of MHC-E, β2m and peptide serves as a ligand for NKG2x/CD94 innate receptors, which are expressed on a majority of NK cells and other cytolytic lymphocytes. Although germline encoded, the NKG2x/CD94 receptors interact with residues of the leader peptides and are in that respect peptide-specific (5–7). We and others showed that the inhibitory variant NKG2A/CD94 is an immune checkpoint in cancer immunity (8, 9, 10) and blocking antibodies are now being evaluated in a dozen clinical trials with promising results (11).

Interestingly, next to its very conserved role as ligand for the innate receptors NKG2x/CD94, MHC-E also serves as an antigen-presenting molecule for T cell responses. During the past few years, seminal studies on effective vaccination in Rhesus macaques sparked a growing interest to induce protective T cell responses via MHC-E in the context of infectious diseases and cancer (2, 12–16). Due to its conserved nature, such MHC-E restricted T cell responses might represent a universal approach for vaccination, and induce immunity independent from the individualized responses that are shaped by conventional highly polymorphic MHC molecules. However, the biology and antigen-presentation capacity of MHC-E is not fully understood yet, as illustrated by the observation that MHC-E molecules are very instable at the surface of cells (2, 17–19). Molecular studies also reported on the possibility of peptide-free forms of HLA-E (20, 21), pointing at the necessity to better characterize the biology of MHC-E molecules.

Here, we studied the murine Qa-1^b^ member of the MHC-E family using two antibodies: the recently developed EXX-1 antibody that selectively targets Qa-1^b^ molecules when the Qdm leader peptide is accommodated (18, 22) and the generally employed 6A8.6F10 antibody that was raised to a linear range from the alpha 2 helix of Qa-1^b^ (aa 161-179) (23). We surprisingly found that the well-known antibody 6A8.6F10 binds to a peptide-free configuration of Qa-1^b^ and that these conformations, rather than Qdm containing Qa-1^b^, are present at the surface of cells in homeostasis.

## Results

### The 6A8 antibody binds to a linear stretch of the alpha 2 helix of Qa-1^b^, whereas the EXX-1 antibody interacts with residues of the Qdm peptide in Qa-1^b^

**Figure 1:**
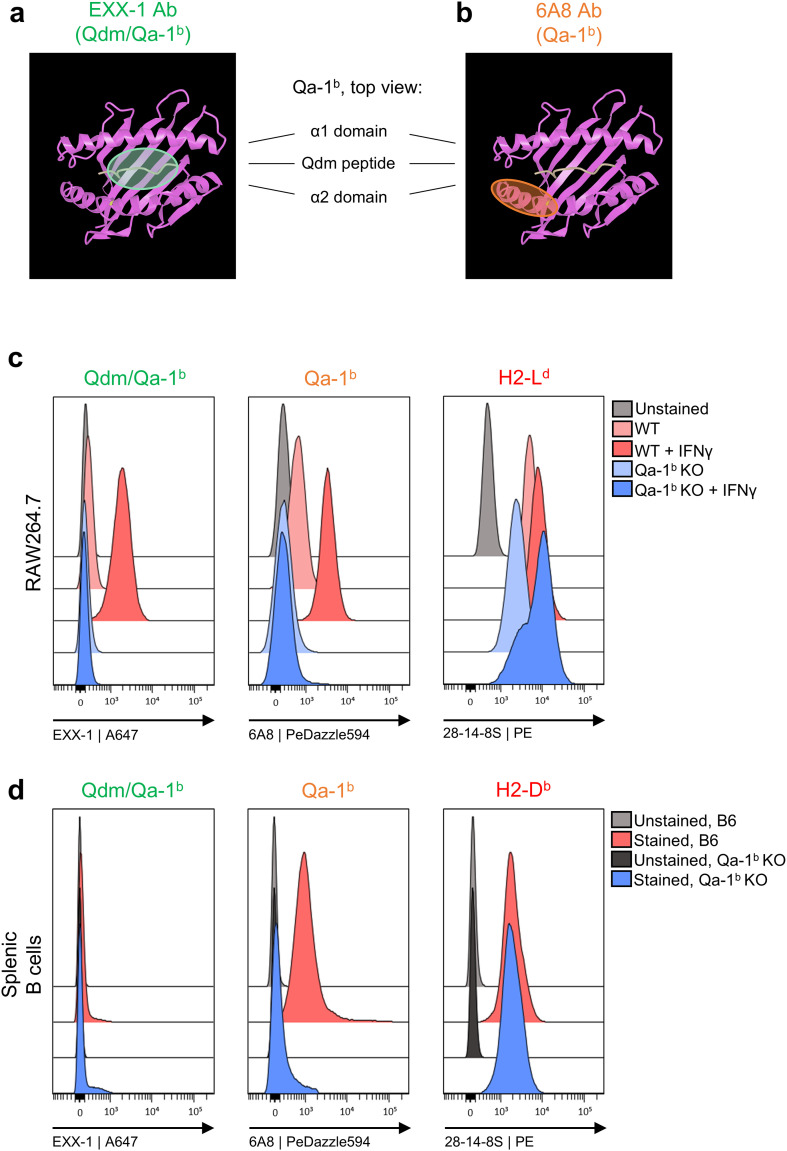
Two Qa-1^b^-specific antibodies recognize distinct epitopes. **(A, B)** Top view of the crystal structure of Qa-1^b^ (in pink) with Qdm peptide (in yellow) (24). The EXX-1 antibody recognizes Qa-1^b^ in a Qdm peptide-dependent way (green) (22) **(A)** and the 6A8 antibody binds to a linear stretch on the alpha 2 domain of Qa-1^b^ (orange) (23) **(B, C)** Flow cytometry histograms of IFNγ treated RAW264.7 macrophages and their Qa-1^b^ KO variants stained with EXX-1, 6A8 and 28-14-8S antibodies. **(D)** Flow cytometry histograms of *ex vivo* splenic B cells from C57BL/6 mice and Qa-1^b^ KO mice, stained with the same antibodies. Pre-gating of the B cells is shown in **Supplementary Figure 1**.

We recently isolated the ‘EXX-1’ VHH nanobody from a lama immunized with recombinant Qdm/Qa-1^b^ complexes and cloned this nanobody in a mouse IgG2a format (22). EXX-1 was shown to bind Qa-1^b^ molecules in a Qdm peptide-dependent manner and alanine scan revealed interaction with amino acids 3, 6, 7 and 8 of the AMAPRTLLL sequence (22) (represented in the crystal structure in **Figure 1A**). In contrast, the widely used monoclonal antibody 6A8.6F10 (abbreviated in this paper as ‘6A8’) was raised to a linear peptide from the alpha 2 helix of Qa-1^b^ (aa 161-179) (23) (**Figure 1B**). The 6A8 antibody has been applied in denatured western blotting and protein pull down studies, underscoring its binding to a linear epitope (23). EXX-1 and 6A8 both selectively bind Qa-1^b^ molecules as demonstrated by flow cytometry staining of RAW264.7 macrophages and their Qa-1^b^-knockdown counterparts (**Figure 1C**) and splenic CD19^+^ B cells from C57BL/6 mice and Qa-1^b^-knockout mice (**Figure 1D**; **Supplementary Figure 1**). The 6A8 antibody stained both cell types, whereas EXX-1 staining required treatment with IFNγ, confirming our earlier observation that loading of the Qdm peptide depends on IFNγ, despite the availability of Qa-1^b^ molecules (18). The Qdm peptide derives from the leader sequence of the class I H2-L^d^ and H2-D^b^ proteins (25), which were expressed in these cells (**Figures 1C, D**, respectively). These data argue that the Qa-1^b^ molecules detected on RAW264.7 macrophages and splenic B cells in the absence of IFNγ are devoid of Qdm peptides and that the 6A8 antibody binds to other peptide/Qa-1^b^ complexes or alternative conformations.

### Genome-wide CRISPR screen reveals members of the peptide loading complex as inhibitors for 6A8 binding

**Figure 2:**
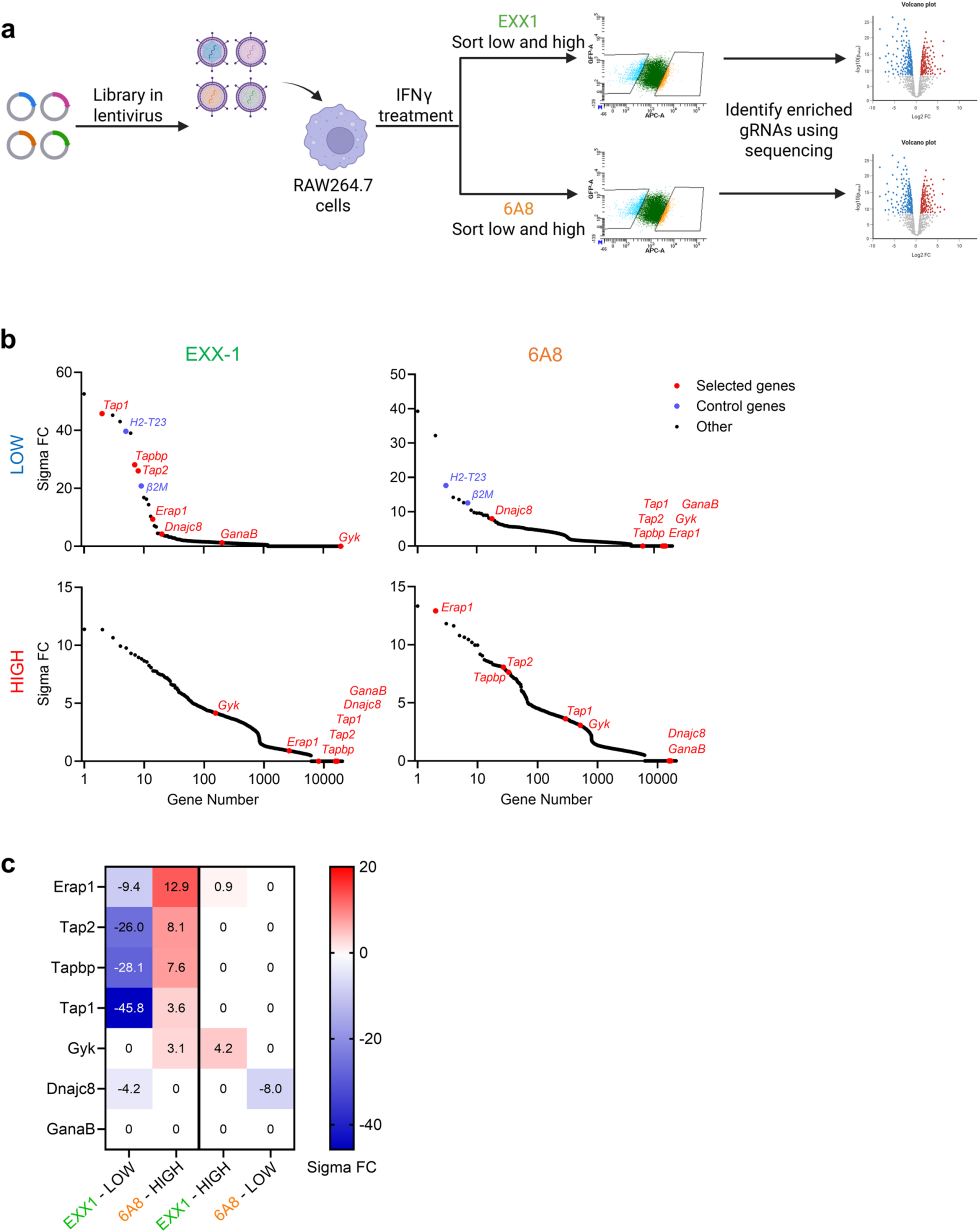
The binding of the 6A8 antibody increases in the absence of components of the peptide loading complex. **(A)** Schematic overview of the genome-wide CRISPR-Cas9 screen in RAW264.7 macrophages to identify genes that impact surface expression of Qdm/Qa-1^b^ complexes (EXX-1) or Qa-1^b^ heavy chains (6A8). The macrophages were treated with 5 IU/mL IFNγ for 2 days to induce Qdm/Qa-1^b^ expression. **(B)** Genetic hits identified from the screens based on antibody binding from the 5% lowest and 5% highest sorted cells for EXX-1 and 6A8. **(C)** Heatmaps of the sigma fold change scores for the selected genes.

We previously conducted a genome-wide clustered regularly interspaced short palindromic repeats (CRISPR)-Cas9 screen using the Brie library in the context of EXX-1 antibody specificity (18, 26). RAW264.7 macrophages were transduced with the gRNA viruses and pretreated with IFNγ to increase levels of Qa-1^b^. The lowest 5% and the highest 5% of cells stained with the EXX-1 and 6A8 antibodies were sorted, resulting in four separate populations of RAW264.7 knockout cells (**Figure 2A**). The gRNAs of these four populations were sequenced and compared to input. The hits of EXX-1-low and 6A8-low screens included genes important for IFNγ signal transduction (*Stat1*, *Jak1*, *Jak2* and both IFNγ receptors *Ifngr1* and *Ifngr2*) and genes for the Qa-1^b^ protein (*H2-T23* and *β2m*) (**Figure 2B**; **Supplementary Table 1**), illustrating the validity of the screen (18). In addition, components of the peptide loading complex (PLC) were yielded from the EXX-1-low screen, including the genes for tapasin, TAP1, TAP2, ERp57 and ERAAP, indicating the strict dependency of the Qdm peptide on a functional antigen processing machinery (**Figure 2B**). Furthermore, we found genes for heat shock protein DNAJC8 and for the alpha subunit of glucosidase II (*GanaB*) in the EXX-1-low screen. Remarkably, the hits of the 6A8-low population did not yield any of the PLC members, rather ERAAP, tapasin and TAP were surprisingly enriched in the 6A8-high population, indicating that staining with the 6A8 antibody was increased after abrogation of these genes. The enrichment scores of these selected genes revealed a clear opposite pattern of the two antibodies (**Figures 2B, C**). Thus, genes essential for Qdm peptide presentation seem to hamper staining with the 6A8 antibody.

**Figure 3:**
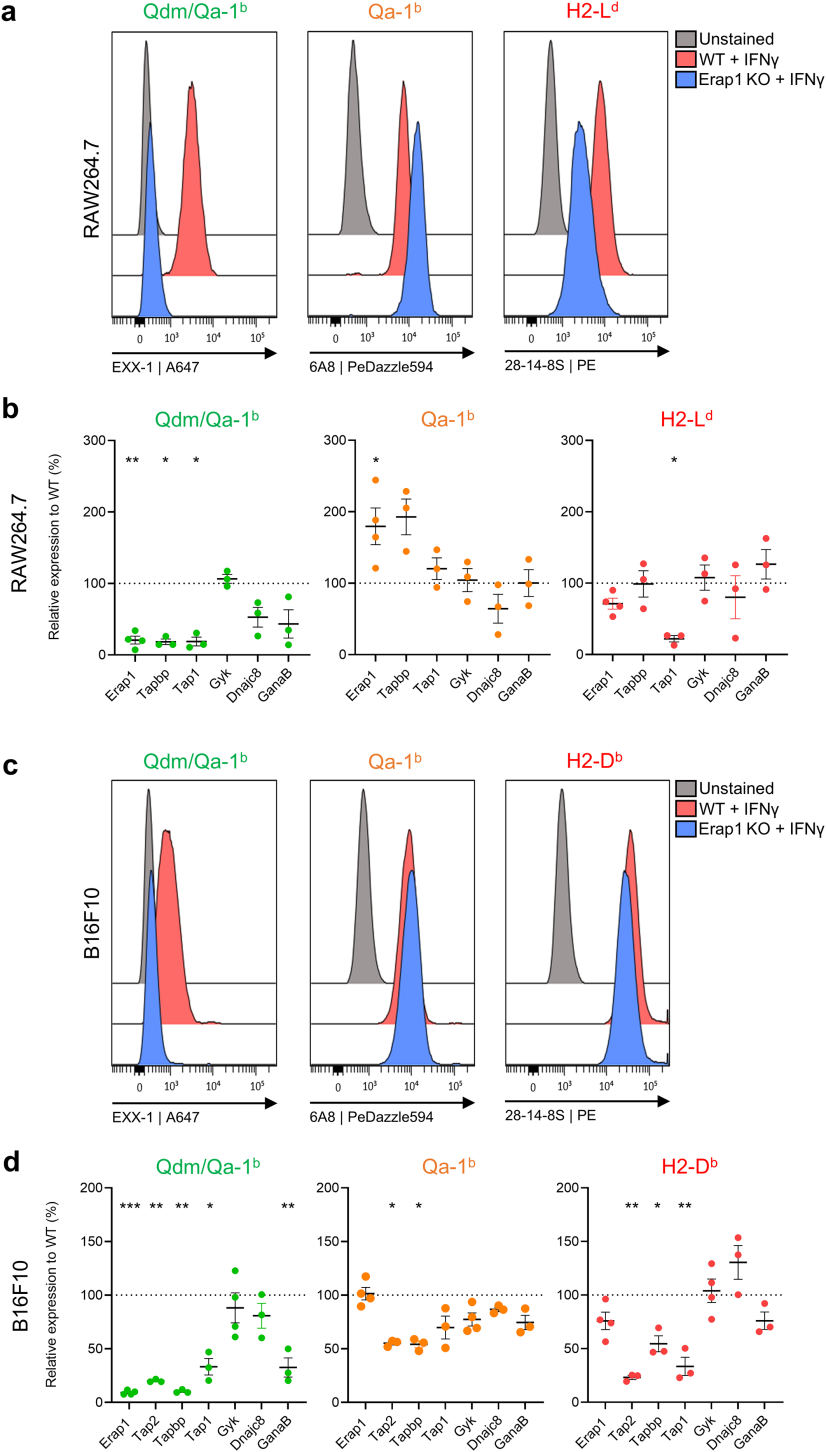
Validation of the screening hits in RAW264.7 macrophages and B16F10 melanoma cells. **(A)** Representative flow cytometry histograms of gene knock down validation in RAW264.7 macrophages for ERAP1. **(B)** Quantified results of EXX-1, 6A8, and 28-14-8S staining of genetic knock down in IFNγ treated RAW264.7 macrophages. Percentages of decreased or increased staining geomean intensities were calculated compared to wild type cells. Means and SD are shown of three independent experiments. Statistical analyses were performed per gene using a paired t-test. **(C, D)** Similar analysis for B16F10 melanoma cells. Statistical significance is shown as *p < 0.05, **p < 0.01 and ***p < 0.001.

The selected genes were then validated in RAW264.7 macrophages and, as a second cell type, B16F10 melanoma cells (**Figure 3**). Guide RNA sequences were designed for each of these seven genes, were cloned into lentiviral CRISPR/Cas9 knockdown vectors and were transduced in these two types of target cells. Knockdown of the ERAAP encoding gene in RAW264.7 macrophages indeed efficiently abolished Qdm presentation, but simultaneously, increased 6A8-binding up to two-fold (**Figures 3A,B**). Similar strong effects were observed for the *tapbp* gene, coding for tapasin (**Figure 3B**). Interestingly, 6A8 binding did not alter after knockdown of the other selected genes, despite a decreased Qdm/Qa-1^b^ presentation. Of note, the control antibody 28.14.8S, which binds the classical H2-L^d^ and H2-D^b^ molecules in a peptide-containing conformation (27), indeed strongly depended on TAP1 for surface display in RAW264.7 macrophages. The strict dependency on PLC components for Qdm/Qa-1^b^ was also apparent in B16F10 melanoma cells (**Figures 3C, D**). Deletion of the ER-resident glucosidase II (*GanaB* gene) reduced Qdm presentation more than fifty percent. This enzyme is important for the quality control of protein folding in the ER, implying that Qdm/Qa-1^b^ surface display not only guarantees a functional antigen processing machinery but also a proper protein folding process of cells (28). In RAW264.7 cells, loss of ERAP1 and tapasin led to increased 6A8 binding. In B16F10 melanoma cells, ERAP1 knockdown did not significantly alter 6A8 staining (**Figure 3D**). Irrespective of these differences between macrophages and melanoma cells, which might have been caused by variances in intracellular recirculation of MHC class I molecules, the overall picture revealed a relative independency of PLC components for 6A8 staining, suggesting peptide-independency of 6A8.

### Exogenous peptide pulsing of Qa-1^b^ decreases staining with the 6A8 antibody

**Figure 4:**
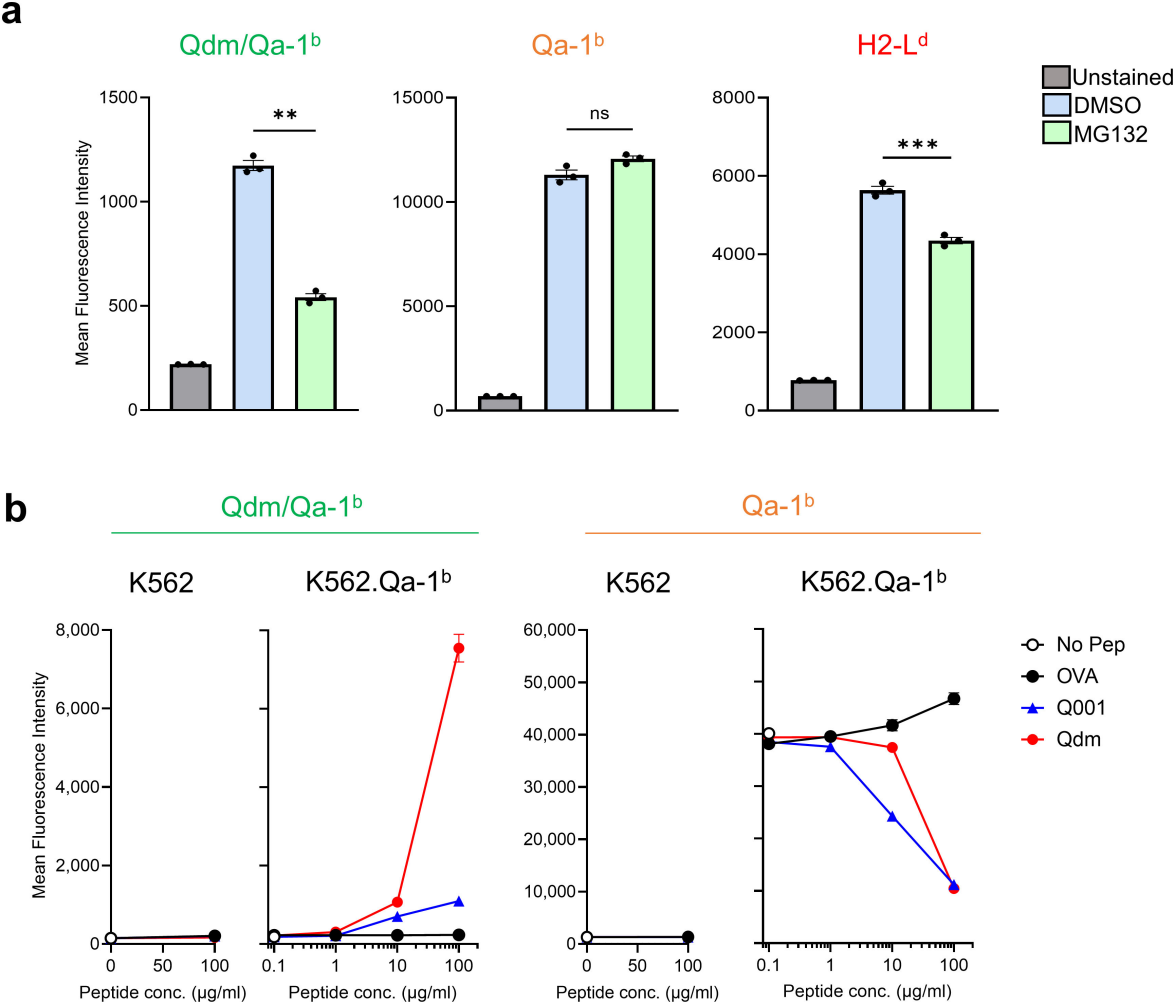
Exogenous loading of Qa-1^b^-binding peptides decrease staining by the 6A8 antibody. **(A)** Effect of proteasome inhibitor MG132 on RAW264.7 macrophages that were pre-treated with IFNγ to induce Qdm presentation. Percentages of geomean intensities of EXX-1, 6A8 and 28.14.8S staining were compared to solvent DMSO treated cells. Means and SD are shown of three independent experiments. Statistical analyses were performed using a paired t-test. **(B)** Flow cytometry analysis of human HLA class I negative K562 cells, transduced with the Qa-1^b^ encoding gene and pulsed with increasing concentrations of peptides Qdm, Q001 or OVA. Statistical significance is shown as **p < 0.01 and ***p < 0.001.

Given the relative independency of peptides for the 6A8 antibody binding, we tested the impact of proteasome inhibition on expression of Qdm/Qa-1^b^ and Qa-1^b^ heavy chains (**Figure 4A**). The proteasome is the major supplier of peptides that are presented in MHC class I molecules and previous reports suggested that liberation of the Qdm peptide also depends on proteasome activity (29). RAW264.7 macrophages were treated with IFNγ to induce surface display of Qdm/Qa-1^b^ complexes and then incubated with MG132, a potent proteasome inhibitor for four hours or with solvent DMSO. Proteasome blockade significantly decreased staining with EXX-1, indicating that Qdm was proteasome dependent. In contrast, 6A8 staining was not influenced, suggesting that this antibody targets Qa-1^b^ molecules independent of peptides (**Figure 4A**).

We directly examined the impact of exogenous peptide pulsing of Qa-1^b^ molecules on binding of the two antibodies. Human HLA class I deficient K562 cells were transduced with the Qa-1^b^ encoding gene H2-T23 and incubated with increasing concentrations of three different synthetic peptides: Qdm, a control Qa-1^b^-binding peptide (Q001) and the K^b^-binding OVA peptide (**Figure 4B**). Peptide pulsing of Qa-1^b^-negative parental K562 remained negative for EXX-1 and 6A8 staining. The EXX-1 antibody strongly bound to K562.Qa-1^b^ cells when loaded with Qdm peptide, and only minimal staining when loaded with Q001 peptide, confirming its specificity. The 6A8 antibody stained K562.Qa-1^b^ cells without addition of peptides and, strikingly, a strongly decreased staining was observed with increasing concentrations of peptides (**Figure 4B**). This decreased staining was only detected for the Qa-1^b^-binding peptides Qdm and Q001, and not for the control K^b^-binding OVA peptide, implying that 6A8 loses its affinity for Qa-1^b^ when a peptide is accommodated in the binding groove.

### The 6A8 antibody selectively binds recombinant peptide-free Qa-1^b^/β2m complexes

We examined binding specificity of the 6A8 antibody to peptide/Qa-1^b^ complexes in a cell-free system. The Qa-1^b^ heavy chain and β2m proteins were produced in bacteria, purified and folded into monomers that incorporated various Qa-1^b^-binding peptides: Qdm, Q001 and GroEL from *Salmonella typhimurium* (30). The folded monomers were purified by size exclusion chromatography and subsequently characterized and analyzed using SDS-PAGE and mass spectrometry (**Supplementary Figures 2A-D**). Interestingly, the yield of monomers folded with the GroEL-derived peptide was comparable to those obtained with the other peptides Qdm and Q001, however no peptide was detected in mass spectrometry analysis of the final product. In contrast, Qa-1^b^/β2m/Qdm and Qa-1^b^/β2m/Q001 complexes did contain peptides (**Supplementary Figure 2C**). Therefore, we concluded that the final monomer of Qa-1^b^/β2m/GroEL was devoid of peptide and was thus labelled as Qa-1^b^/β2m/NP for ‘no peptide’.

**Figure 5:**
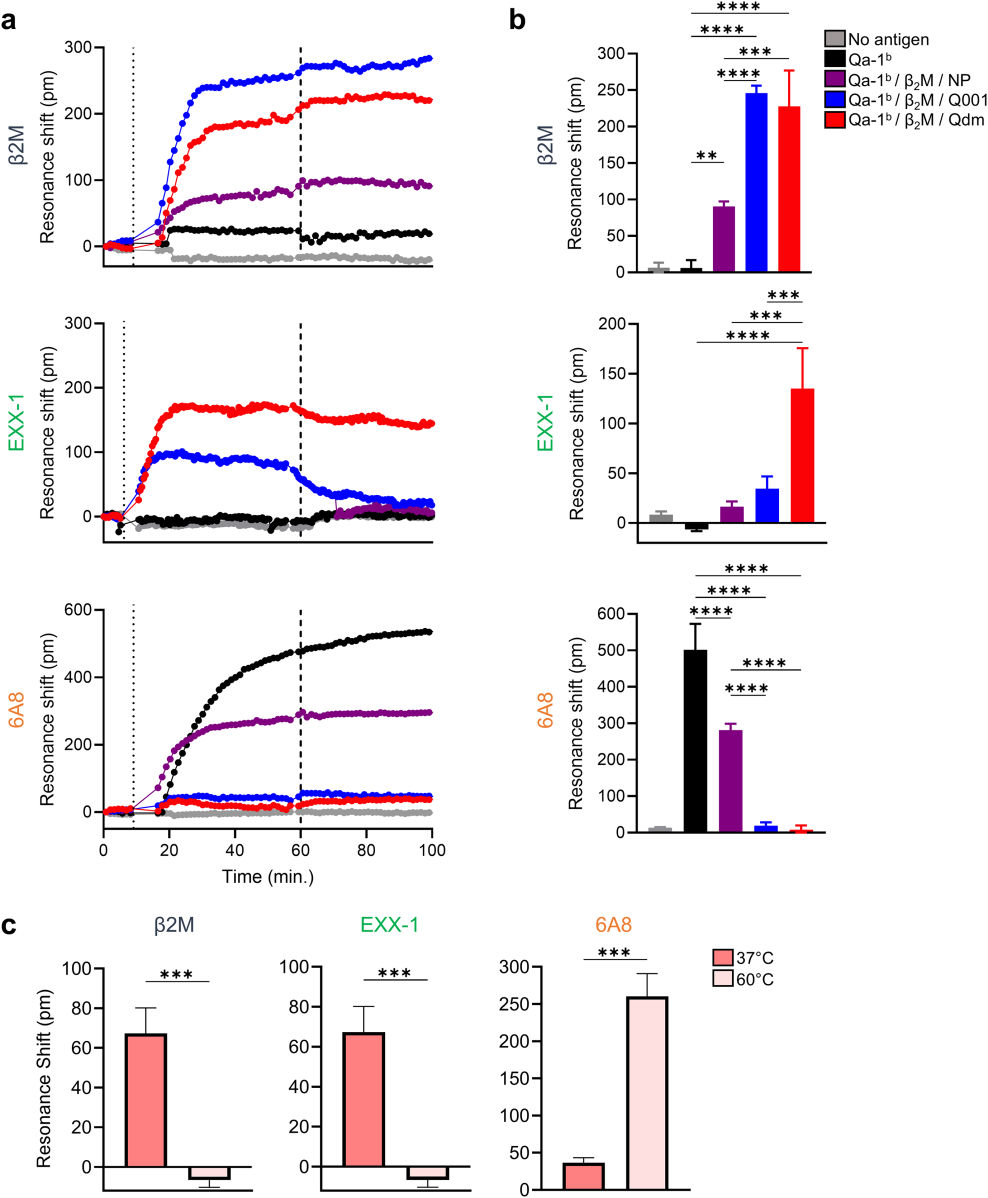
6A8 antibody is selective for peptide-free Qa-1^b^ as determined in the label-free bioassay. **(A-B)** Monomeric Qa-1^b^/β2m/peptide complexes immobilized on label-free microarray plates were incubated with antibodies (anti-β2m, EXX-1, and 6A8) at 5 min (first dashed line), followed by removal of unbound antibodies at 60 min (second dashed line). **(A)** Representative sensorgram depicting resonance shifts over time from one of three independent experiments. **(B)** Quantification of antibody binding, represented as mean resonance shifts at 20 min post-wash (80 min total). **(C)** Stability and retention of antibody epitopes following thermal challenge. Qa-1b/β2m/Qdm complexes were pre-incubated at 37 °C or 60 °C for 10 min before immobilization. The bar graph shows resonance shift at 20 min post-wash, illustrating differential antibody recognition after heat treatment. Data are represented as means ± SD for n=3. Significance was calculated using One-way ANOVA followed Tukey’s *post hoc* tests **(B)** or unpaired student t-test **(C)**. Quality controls for folded Qa-1^b^ used in these assay are provided in **Supplementary Figure 2**. Statistical significance is shown as **p < 0.01, ***p < 0.001 and ****p < 0.0001.

Next, we immobilized the various Qa-1^b^ complexes in a label-free ResoSens bioassay to measure the resonance shift induced by binding of 6A8, EXX-1 and anti-β2m antibodies (**Figures 5A–C**). After 60 minutes the plates were washed to determine the decay of the signals in the absence of antibody excess. The β2m antibody detected all monomer complexes, except for the Qa-1^b^ heavy chain, underscoring proper folding of the monomeric complexes (**Figures 5A, B**). The EXX-1 antibody was selective for Qa-1^b^/β2m/Qdm complexes especially after removal of the excess of antibody, illustrating its specificity. Surprisingly, the 6A8 antibody did not induce a resonance shift with either the Qa-1^b^/β2m/Qdm or Qa-1^b^/β2m/Q001 complexes, whereas it showed a strong positive signal with the Qa-1^b^ free heavy chain and the peptide-free Qa-1^b^/β2m/NP complexes. These results indicate that the 6A8 antibody exclusively binds peptide-free molecules. Finally, denaturation of Qa-1^b^/β2m/Qdm complexes by incubation at 60 °C resulted in loss of binding by anti-β2m and EXX-1 antibodies but led to a substantial increase of resonance shift by the 6A8 antibody, underscoring its specificity for peptide-free Qa-1^b^ molecules (**Figure 5C**).

### Peptide-free Qa-1^b^ molecules are abundantly present *in vivo*

**Figure 6:**
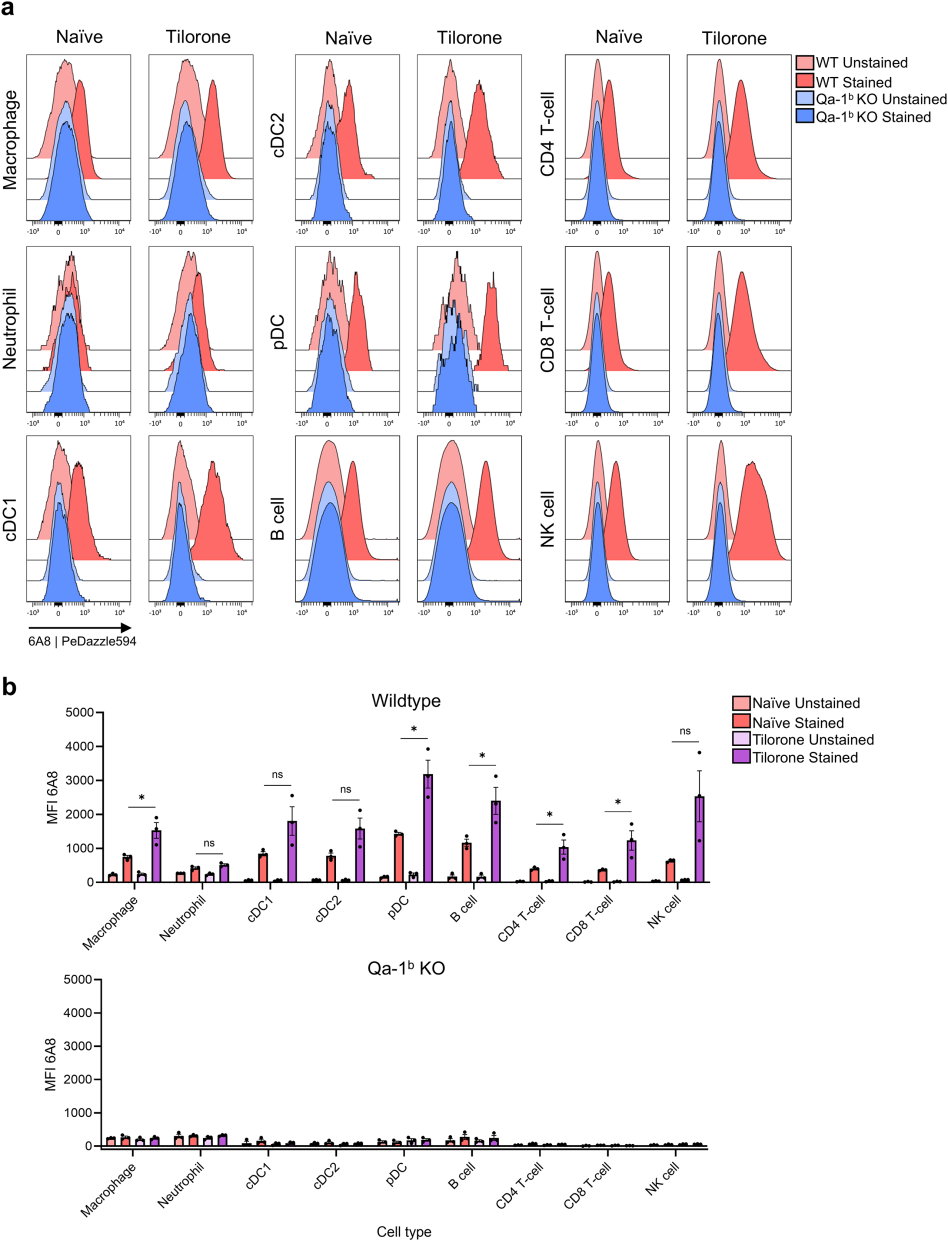
Peptide-free Qa-1^b^ molecules are predominantly displayed by antigen-presenting cells. Splenocytes from B6 mice and Qa-1^b^ knockout animals were stained with an antibody panel of lineage markers and 6A8. Naïve and tilorone-injected mice were tested for peptide-free Qa-1^b^ expression on several myeloid and lymphoid cell types, as defined in **Supplementary Figure 3**. Flow cytometry histograms **(A)** contain compiled events from separate staining of 3 animals and bar graphs **(B)** depict geometric means with SD. Unpaired t-tests of staining intensity of cells from B6 versus Qa-1^b^ knockout mice was used for statistics. Statistical significance is shown as *p < 0.05.

Finally, we performed flow cytometry assays to determine the cell populations that display peptide-free Qa-1^b^ molecules *in vivo*. Splenocytes were stained with a panel of antibodies to discern immune cell lineages, such as dendritic cells, B cells and T cell subsets (**Figure 6**; **Supplementary Figure 3**). 6A8 binding was highest on plasmacytoid DC (pDC) and other antigen-presenting cell types, including conventional DC (cDC), macrophages and B cells, whereas expression on T cell lineages and NK cells was much lower and granulocytes were negative (**Figures 6A, B**). Injection of tilorone, a potent interferon inducer, resulted in increased staining on all subsets, except for granulocytes. These findings implied that empty Qa-1^b^ molecules are exhibited on antigen-presenting cells *in vivo*. Previously, infection with murine cytomegalovirus (MCMV) was shown to affect the peptide loading machinery, resulting in loading of alternate peptides in Qa-1^b^ molecules (31). RAW264.7 cells infected with GFP-encoding MCMV virus were still negative for Qdm/Qa-1^b^ complexes and presented more peptide-free Qa-1^b^ (**Supplementary Figure 4**). Altogether, these data suggested that the widely used 6A8 antibody specifically binds peptide-free Qa-1^b^ molecules, which exist on the surface of multiple cell types in homeostasis and are enhanced after viral infection or interferon stimulation. In contrast, Qdm-presentation by Qa-1^b^ is absent in homeostasis and strictly requires induction by interferon.

## Discussion

Intracellular loading of the Qdm peptide in Qa-1^b^ molecules is critical for functional interaction with NKG2x/CD94 immune receptors (32) and here we show that the loading of this peptide requires the core members of the peptide loading complex, including TAP, tapasin and ERAAP. In addition, we found that Qdm loading also depends on endoplasmic reticulum (ER) quality control mechanisms for glycoproteins as knockdown of glucosidase II, encoded by the *GanaB* gene, resulted in dramatic decrease in surface Qdm/Qa-1^b^ complexes. The glucosidase II catalyzes the removal of terminal glucose groups from nascent *N*-linked glycoproteins in the ER, thereby preventing prolonged engagement with folding chaperones, like calreticulin, and thus promotes transport out of the ER (28, 33). Together with previous observations (18, 34–36), a clear picture emerges concerning the display of Qdm/Qa-1^b^ complexes by somatic cells: they are induced by interferons and reflect the functional integrity of the antigen-processing and glycoprotein folding machinery. High expression of Qdm/Qa-1^b^ complexes during inflammation thus serves as a ‘do not kill me’ signal to cytotoxic lymphocytes through engagement by NKG2A/CD94. These insights were enabled by our recently developed monoclonal antibody EXX-1, which binds Qa-1^b^ in a Qdm peptide-dependent manner (22).

In contrast to the EXX-1, we here uncovered that the widely used antibody 6A8 specifically recognizes peptide-free Qa-1^b^ molecules. We found that 6A8 failed to bind recombinant monomers of Qdm/Qa-1^b^, whereas it did bind to denatured Qdm/Qa-1^b^ complexes and peptide-free Qa-1^b^ monomers (**Figure 5**). These peptide-free monomers were initially folded with recombinant Qa-1^b^, β2m and a bacterial GroEL derived peptide (30), but mass spectrometry confirmed the absence of peptide in the final product. Importantly, β2m free heavy chains of Qa-1^b^ were apparently not present on cell surfaces, as genetic loss of β2m gene prevented 6A8 binding (**Figure 2**). These observations on 6A8 specificity have important ramifications. First, functional blockade of Qdm/Qa-1^b^ complexes in cellular assays should henceforth be performed using the EXX-1 antibody rather than 6A8, as has erroneously been executed in the past. Indeed, we previously demonstrated that Qdm/Qa-1^b^ tetramer staining in flow cytometry can be blocked by EXX-1 but not 6A8 (18). Second, the field still lacks a reagent capable of detecting all Qa-1^b^ conformations. Beyond peptide-free and Qdm-bound conformations described here, additional Qa-1^b^ forms exist that present viral- and tumor-derived peptides at the cell surface, emphasizing the need for new antibodies to reveal the complete conformational landscape of Qa-1^b^. Third, peptide-free Qa-1^b^ molecules appear to be naturally expressed *in vivo* under homeostatic conditions, predominantly on antigen-presenting cells like dendritic cell subsets and B cells. The modest increase of 6A8 staining with high concentrations of OVA peptide likely results from generation of peptide-empty Qa-1^b^ due to competition with low-affinity binding endogenous peptides (**Figure 4B**). In our previous study we found that empty Qa-1^b^ have a short half-life of approximately 30 min at the cell surface, indicating a high disintegration rate or fast internalization rates (18). After internalization, the molecules might be loaded with peptides in endolysosomal vesicles to present exogenous antigens. The dated findings on efficient presentation of a peptide derived from the exogenous insulin protein suggest that recirculation and endolysosomal loading is a realistic hypothesis (23, 37, 38). Indeed, macrophages have been shown to transport HLA-E, the human counterparts of Qa-1, predominantly to lysosomal vesicles (39). Importantly, peptide-free Qa-1^b^ molecules were still present in inflammatory conditions in our study, indicating that the emergence of empty Qa-1^b^ is independent of Qdm loading. So, the different folds co-exist on cells.

Although we did not directly evaluate the existence of peptide-free HLA-E molecules *in vivo* in this study, recent structural studies provide compelling support for their existence. Small-angle X-ray scatter (SAXS) analyses demonstrated that the peptide content affects the gross conformation of HLA-E molecules (40). The Qdm equivalent in humans, known as ‘VL9’ peptide, consists in a more concise form than HLA-E complexes in which pathogen-derived peptides were loaded, implying that different conformations do exist for HLA-E. In addition, recombinant HLA-E and β2m proteins were found to readily form dimers in the absence of exogenously added peptide, a rather unusual finding for HLA class I molecules (19, 20). These peptide-free complexes could be visualized on blue-native PAGE gels and discerned from peptide-containing HLA-E complexes. Moreover, the HLA-E specific antibody 4D12 was found to preferentially bind to peptide-free HLA-E on cells, as exogenous loading with the ‘VL9’ peptide decreased staining by this antibody (21). Together, these observations suggest that peptide-free HLA-E molecules also exist *in vivo*, although a dedicated antibody to specifically detect this conformation, equivalent to the 6A8 antibody, is not available yet.

In contrast to Qdm/’VL9’ loaded MHC-E, we hypothesize that empty MHC-E molecules are widely present in homeostasis and might serve as ligands for undefined immune receptors. We previously demonstrated that the interaction of LILRB1 and LILRB2 receptors, which are expressed throughout the immune cell lineages, to HLA-E tetramers is indifferent to the peptide content (18), and even binds to peptide-free HLA-E tetramers, which were generated by addition of an extra disulfide bridge (41). Furthermore, genome-wide screens identified stabilin-1 and -2 as immune scavenger receptors binding empty HLA-E molecules (41). Stabilins, also known as Clever, are expressed on myeloid cells and scavenge multiple degraded proteins from the circulation (42–44). Even more immune receptors were found to interact with MHC-E, e.g. CD8αα homodimers that are expressed by intraepithelial lymphocytes (45) and the broadly expressed VISTA (46). At this stage, it remains to be elucidated to which forms of MHC-E these receptors bind and the biological responses of their interactions. So, the function of MHC-E is clearly diversified within immunology, from exhibiting cellular integrity under inflammatory circumstances via the checkpoint NKG2x/CD94 and damping immune reactivity via LILRB1 and -2, to presenting antigens derived from pathogens to T cells.

## Materials and methods

All used materials are listed in the Resource Table (**Supplementary Table 2**).

### Mice

C57BL/6J mice were purchased from Charles River, the Netherlands. Qa-1^b^ deficient C57BL/6J mice were initially obtained from Jackson Laboratories (B6.129S6-H2-T23tm1Cant/J, stock number 007907) and bred at the Leiden University Medical Center (LUMC). Mice were housed at the animal facility of the LUMC and experiments were approved by the Dutch animal ethics committee (CCD) and the local Animal Welfare body on the permit number AVD11600202010004. The health status of the animals was monitored over time and all animals were tested negative for agents listed in the FELASA (Federation of European Laboratory Animal Science Associations) guidelines for specific-pathogen free (SPF) mouse colonies (47). Experiments were performed in accordance with the Dutch Act on Animal Experimentation and EU Directive 2010/63/EU (‘On the protection of animals used for scientific purposes’).

### Injection of tilorone

Naïve C57BL/6 mice were i.p. injected with 30 mg/kg tilorone dihydrochloride (Sigma-Aldrich). After 24 hours, mice were sacrificed and spleen were isolated for analysis by flow cytometry.

### Cell preparation and flow cytometry

Single cell suspensions of the cell lines or mouse tissues were generated. Spleens were dissociated into a single-cell suspension using a 70 µM cell strainer (BD Biosciences). Splenocytes were incubated with lysis buffer (in house pharmacy) for 3 minutes at RT to remove all red blood cells before use.

Mouse Fc-receptors were blocked by Rat Anti-Mouse CD16/CD32 (Clone 2.4.G2, BD) for 15 min at 4 °C in PBS. Viability was assessed with the LIVE/DEAD Fixable Aqua Dead Cell Stain Kit (Biolegend) in PBS before surface staining. Surface markers were stained in FACS buffer (PBS with 0.5% BSA (Sigma-Aldrich) and 0.002% sodium azide (in house pharmacy)) for 20 min at 4 °C. Finally, cells were resuspended in FACS buffer and measured on a Fortessa cytometer (BD Biosciences) and analyzed with FlowJo software v10.8.1 (Treestar) or OMIQ software. An overview of all the antibodies used for flow cytometry is shown in **Supplementary Table 2**.

### Cell lines

The B16F10 melanoma cell line (RRID: CVCL_0159) was purchased from the American Type Culture Collection (ATCC). The BALB/c macrophage cell line RAW264.7 (RRID: CVCL_0493) was kindly provided by Dr. F. Ossendorp (LUMC, Leiden, the Netherlands). The K562 lymphoblast cell line (RRID: CVCL_0004) was isolated from the bone marrow of a patient with chronic myelogenous leukemia and was obtained from Yvonne Zoet (LUMC, Leiden, the Netherlands). Finally, HEK293T cells (RRID: CVCL_0063) were purchased from the ATCC. Where indicated, cell lines were stimulated with recombinant 30 IU/mL (B16F10) or 5 IU/mL (RAW264.7) IFNγ (BioLegend) for two days. Unless indicated otherwise, all cell lines were cultured in Iscove’s modified Dulbecco’s medium (IMDM, Invitrogen) supplemented with 8% heat-inactivated FBS (Serana), 2% penicillin/streptomycin (Gibco) and 2mM glutamine (Gibco) at 37 °C and 5% CO_2_. All cell lines were frequently tested negative for mycoplasma by PCR test.

### Generation of knockout and overexpression cell lines

Knockout cell lines were generated using CRISPR/Cas9 vector LentiCRISPRv2 generated by the Zhang lab (48). sgRNAs were designed using Benchling and Crispor online software with additional base pairs on each oligonucleotide to allow ligation into the target vector: 5′ CACCG(sgRNA1) 3′ and 3′ C(complementary sequence to sgRNA1)CAAA 5’ to facilitate ligation into BsmBI-digested (NEB) LentiCRISPRv2 puro or blast vector (Addgene) using T4 DNA Ligase (Thermo Scientific). The plasmid constructs were propagated in Stbl3 bacteria (Invitrogen) and isolated using nucleospin plasmid transfection grade kits (Macherey-Nagel) according to manufacturers’ protocol. HEK293T were transfected with the LentiCRISPRv2 knockout constructs and accessory Pax2 and pMD2.G plasmids (both kindly gifted by Dr. Didier Trono) using lipofectamine 3000 (Invitrogen) according to manufacturers’ protocol. Supernatant of the HEK293T cells containing the lentivirus was then used to transduce the target cell lines. After puromycin (ThermoFisher) or blasticidin antibiotic (ThermoFisher) selection, the knockdown cell lines were validated by flow cytometry. Mouse cell lines B16F10 and RAW264.7 were targeted with gRNAs for different genes (**Supplementary Table 2**).

K562.Qa-1^b^ cells were generated by transduction with lentiviral particles from HEK293T cells, which were transfected with a pCDH-CMV-MCS-EF1-Puro plasmid (System Biosciences) encoding the H2-T23 gene and accessory Pax2 and pMD2.G plasmids. Transduced cells were sorted using flow cytometry based on surface Qa-1^b^ expression (6A8 antibody).

### Genome-wide CRISPR knockout screen for 6A8 and EXX-1 antibody binding

A screen with the mouse CRISPR Brie genome-wide knockout library (26), containing 4 gRNAs per gene, was performed in RAW264.7 cells. For virus production, HEK293T cells were transfected with packaging plasmids pMDLg/pRRE (Addgene), pRSV-Rev (Addgene), pCMV-VSV-G (Addgene) together with the Brie plasmid using polyethyleneimine (Polyscience Inc.). Virus was harvested, filtered and 150 million RAW264.7 cells were transduced in the presence of 8 μg/mL polybrene (Millipore) at a multiplicity of infection (MOI) of 0.3. Transduced cells were selected using puromycin (2µg/mL, ThermoFisher), 5 IU/mL IFNγ was added after five days and seven days after transduction 50 million cells were stained for surface Qa-1^b^ with the 6A8 or EXX-1 antibody. The highest 5% and lowest 5% of stained cells were sorted using an Aria cell sorter (BD Biosciences). Cells were expanded and genomic DNA (gDNA) was isolated using an isolate II genomic DNA kit (GC Biotech) for both the unsorted and sorted populations and gDNAs were amplified by PCR using a mix of forward primers and a specific barcoded knockout reverse primer (**Supplementary Table 2**, encoded as NGS-Lib primers) (18). gRNAs were sequenced using a NovaSeq600 system (Illumina) and inserts were mapped to the reference. Analysis of gRNA enrichment was done using PinAPL-Py (49).

### MCMV infection

Wild type and Qa-1^b^ knockout RAW264.7 cells were seeded in a 24-wells plate at a density of 2x10^5^ cells per well. Cells were infected with MCMV-GFP (50) at a multiplicity of infection (MOI) of 1 or stimulated with 5 IU/mL IFNγ. Plates were briefly centrifuged (1 minute) and incubated for two days at 37 °C and 5% CO_2_ atmosphere. After incubation, cells were harvested and stained for flow cytometric analysis.

### Proteasome treatment

RAW264.7 cells were stimulated with 5 IU/mL IFNγ for two days. After harvesting, cells were washed with cold PBS and resuspended in cold culture medium at 1x10^6^ cells/mL in a 15mL tube. 1 µM Proteasome inhibitor MG132 (Sigma-Aldrich) or solvent DMSO (WAK-Chemie Medical) was added for a total of 4 hours at 37 °C and 5% CO_2_ under constant rotation in a HulaMixer (Invitrogen) to keep the cells in suspension. After incubation, cells were washed and analyzed using flow cytometry.

### Exogenous peptide pulsing

K562 and K562.Qa-1^b^ cells were harvested and washed with serum-free medium. Serial dilutions of three different peptides Qdm (AMAPRTLLL, derived from mouse H2-D^b^), Q001 (AQAERTPEL, derived from mouse DENND3) and OVA8 (SIINFEKL, derived from chicken ovalbumin) were prepared in a 96 well V-bottom plate. A total of 2x10^5^ cells in serum-free medium per well were added and incubated for 4 hours at 37 °C and 5% CO_2_. After incubation, cells were stained and analyzed using flow cytometry.

### Production of the EXX-1 antibody

The EXX-1 antibody was produced as mouse IgG2a chimeric molecules (ATUM, Newark, California) by transfection of Expi293F cells (ThermoFisher) with the pFUSE-mouse (m)IgG2A-Fc2 vector (Invivogen) containing the VHH region of EXX-1 (22). Five days post transfection, supernatant was harvested and antibodies were purified using protein A resin (Genscript) according to the manufacturer’s instructions. Antibody purity was confirmed using SDS-PAGE and analytical-grade Superdex 200 columns. For flow cytometry purposes, the EXX-1 and isotype control antibodies were conjugated using the SiteClick antibody azido modification kit (ThermoFisher) and subsequently labeled with Alexa Fluor 647 fluorochrome using the SiteClick sDIBO alkyne kit for antibody labeling (ThermoFisher), both according to the manufacturer’s protocol.

### Qa-1^b^ protein production and refolding

To generate peptide-loaded Qa-1^b^ complexes, the extracellular domain of murine Qa-1^b^, including a C-terminal BirA tag for enzymatic biotinylation, and human β2-microglobulin (β2M) were expressed in Escherichia coli BL21 (DE3) cells (New England Biolabs) using pET21(+) expression vectors (Millipore Sigma). Human β2M was used instead of mouse β2M due to its higher complex stability (51). Recombinant proteins were isolated from inclusion bodies and refolded *in vitro* in the presence of synthetic peptides to assemble stable Qa-1^b^/β2M/peptide complexes (18). Peptides included Qdm (AMAPRTLLL), Q001 (AQAERTPEL) and GroEL (GMQFDRGYL, derived from *Salmonella typhimurium* GroEL) (30) (GenScript Biotech, Piscataway, NJ). Qa-1^b^ heavy chains, free of β2M, were processed the same way as the complexes without β2M or peptides. Refolded complexes were then concentrated and purified by size-exclusion chromatography (SEC) using a Superdex 75 (S75) column (Cytiva, MA). A subset of monomeric complexes was enzymatically biotinylated using BirA biotin ligase (Avidity, CO) and subjected to a second round of SEC for final purification. Mass spectrometry analysis (Sciex X500B QTOF) confirmed the incorporation of Qa-1^b^ heavy chain, β2M, and either the Qdm or Q001 peptides in the tested complexes. In contrast, no GroEL peptide was detected in the corresponding samples, validating its use as a “no peptide” (NP) complex.

### Binding specificity using label-free bioassay system

The binding specificity of EXX-1, 6A8, and anti-β2M antibodies was quantified using the ResoSens Ultra Mab-Pro instrument (Resonant Sensors, TX), with data acquisition and statistical analysis performed via Integrated ResoVu software. Binding specificity was assessed using biotinylated Qa-1^b^ monomer complexes diluted in assay buffer (PBS supplemented with 0.1% BSA and 0.05% Tween-20) and immobilized onto NeutrAvidin-coated Bionetics label-free microarray plates (Millipore-Sigma) at a concentration of 5 µg/mL until equilibrium was reached. Plates were washed three times with assay buffer prior to antibody incubation. Separate samples containing EXX-1, 6A8, and anti-β2M antibodies were each diluted to 5 µg/mL in assay buffer and added to designated wells containing immobilized Qa-1^b^ monomers. Following the binding phase, the unbound antibodies were removed and the plates were washed three times with assay buffer. The wells were aspirated and fresh assay buffer was replaced in each well and plates were placed in the reader for approximately 30 minutes to monitor post-wash kinetics. Quantification of binding specificity of antibodies to Qa-1^b^ monomers was analyzed by subtracting the post-wash reading at 20 min from that of the baseline at 5 min using ResoVu software. For graphical representation, dissociation data corresponding to the 20-minute post-wash timepoint were extracted and used to generate final bar graphs.

### Statistical analysis

All experiments were performed with a minimum of three biological replicates. *In vitro* experiments were at least performed two times. Statistical tests are described in the figure legend, and calculated between two groups using an unpaired two-tailed Student’s t-test and between more than two groups using an ANOVA with Tukey’s *post-hoc* test, unless otherwise indicated. GraphPad Prism (V10.2.3) was used for all statistical testing. Data are represented as mean ± SD unless indicated otherwise. Statistical significance is shown as *p < 0.05, **p < 0.01, ***p < 0.001 and ****p < 0.0001.

## Data Availability

The data presented in the study are deposited in the NCBI SRA under BioProject accession number PRJNA1431763.

## Supplementary material

Supplementary Table 1 Gene hits.

Supplementary Table 2 Resource Table.

Supplementary Figure 1 Flow cytometry gating strategy. Flow cytometry pre-gating of the splenic B cells shown in **Figure 1D**.

Supplementary Figure 2 Quality controls of protein production and formation of Qa-1^b^ monomers. **(A)** Representative size-exclusion chromatography (SEC) profiles using Fast Performance Liquid Chromatography (FPLC) for purification of Qa-1^b^ protein complexes. **(B**) SDS-PAGE analysis of purified correctly folded Qa-1^b^ complexes under reducing conditions, confirming the presence of Qa-1^b^ heavy chain (~34 kDa) and β2-microglobulin (~11 kDa) subunitsz. (C) Liquid chromatography (LC) chromatograms showing detection of peptides Qdm and Q001, but no detectable GroEL-derived peptide (NP). (D) Mass spectrometry analysis of selected LC fractions from (C), confirming the molecular masses corresponding to Qdm and Q001 peptides, validating their incorporation into Qa-1^b^ complexes.

Supplementary Figure 3 Flow cytometry gating strategy. Flow cytometry pre-gating of the several splenocyte populations shown in **Figure 6**. **(A)** Lymphocyte staining panel and **(B)** myeloid cell staining panel, which included antibodies to CD3, CD19 and NK1.1 in the dump channel.

Supplementary Figure 4 Infection with MCMV increases staining with 6A8 antibody.RAW264.7 macrophages and their Qa-1^b^ KO variants were infected by GFP-expressing recombinant MCMV at an MOI of 1 or incubated with 5 IU/mL IFNγ for 2 days. **(B)** Flow cytometry histograms of the RAW264.7 macrophages stained with EXX-1, 6A8 and 28-14-8S antibodies.
