# Supplementary material for: The murine MHC-E molecule Qa-1b is surface displayed in a peptide-free conformation in homeostasis
Source: Front Immunol. 2026 Mar 9;17:1743362. doi: 10.3389/fimmu.2026.1743362 (PMC13006831; doi:10.3389/fimmu.2026.1743362)
Supplement: Supplementary Table 1 — Gene hits. [file DataSheet1.pdf]

Supplementary Table 1. Scores of genome wide CRISPR/Cas9 screen in RAW264.7 cells

6A8 low		6A8 high		EXX-1 low		EXX-1 high	
**Gene**	**SigmaFC**	**Gene**	**SigmaFC**	**Gene**	**SigmaFC**	**Gene**	**SigmaFC**
Stat1	39,25	Dscam	13,33	Stat1	52,57	Tmem131	11,39
Jak1	32,17	Erap1	12,92	Tap1	45,80	Acvr2a	11,35
H2-T23	17,65	Grin2a	11,81	Ifngr2	45,21	Gabre	10,66
Jak2	14,20	Stmn1	11,63	Jak1	43,04	Hsd3b7	9,93
Gamt	13,55	Cul7	10,79	H2-T23	39,68	Susd6	9,77
Ifngr2	12,65	Twf1	10,64	Ifngr1	39,01	Setd6	9,30
B2m	12,57	Tusc5	10,45	Tapbp	28,14	Sycp1	9,17
Lysmd2	10,43	Rab11fip3	10,21	Tap2	26,02	Dhrs4	8,96
Car5a	9,79	Chst4	9,97	B2m	20,83	Ugdh	8,83
Nup62cl	9,62	1700015G11Rik	9,97	H13	16,80	Scoc	8,66
Ssb	9,60	Zfp395	9,18	Sec62	16,29	Opn1sw	8,57
Adck3	9,44	Cdh15	9,06	Jak2	14,37	Prdm5	8,26
Slc35f6	9,00	Lancl2	8,71	Pdia3	10,35	Fuom	8,08
Msi2	8,97	Il17b	8,62	Erap1	9,38	Zfp319	7,79
Zfp787	8,93	Rusc2	8,57	Spcs2	7,06	Rnf103	7,78
Olfr1394	8,15	Rspo2	8,49	Fst	6,75	Adam6b	7,74
Pcgf3	7,99	Snx17	8,47	Pcif1	4,46	Agbl3	7,58
Dnajc8	7,96	2410004B18Rik	8,31	Zfp59	4,44	Pdp1	7,51
Nagk	7,68	Fastkd3	8,27	Tas2r131	4,28	Ilf2	7,46
Ifngr1	7,45	Scn3a	8,25	Dnajc8	4,21	Furin	7,45
Tmprss12	6,99	Man1a	8,22	Thbs4	3,87	Fto	7,39
Calcoco1	6,90	Rarg	8,19	Creld1	3,60	Rab15	7,26
4930558K02Rik	6,48	Slc7a3	8,18	Sec61b	3,58	Papolb	7,20
Itgb1bp2	6,46	Kat2a	8,15	Gnao1	3,57	Fancd2os	7,19
Ddx26b	6,36	Zbtb46	8,13	Rhd	3,57	Depdc1b	7,09
Slc13a4	6,27	Olfr594	8,09	Isoc2a	3,22	Tek	7,02
Mroh8	6,25	Tap2	8,07	Ifitm2	3,20	Hmg20b	6,92
Snx30	6,13	Ube2t	7,93	Appbp2	3,19	Igfbp2	6,65
Gtpbp2	5,98	Gnao1	7,87	9430016H08Rik	3,11	Olfr1396	6,61
Skiv2l	5,93	Efna4	7,75	Marc2	2,95	Vmn2r84	6,57
Scn9a	5,89	Olfr74	7,74	Dyrk1a	2,89	Blk	6,57
Adam1a	5,88	Olfr1502	7,66	Mtmr6	2,85	Adgrg6	6,54
Opn5	5,85	Tapbp	7,63	Ncaph	2,83	Bfsp1	6,33
Fpr2	5,71	Ms4a3	7,62	BRDN0000738036	2,63	Prss43	6,33
Xrcc3	5,70	Glipr1l2	7,51	Scrt1	2,55	Olfr619	6,31
Rdx	5,67	Dock8	7,35	Gm5741	2,42	Hephl1	6,17
Btaf1	5,65	Gm9733	7,29	Wwp2	2,41	Cdnf	6,13
Olfr342	5,64	Ndufa4	7,26	Grm4	2,38	Fam63a	5,96
Abcd3	5,63	Rab1	7,20	Gm8909	2,35	Lnx1	5,93
Gm5868	5,62	Pltp	7,13	Apobec2	2,32	Gpr19	5,90
Olfr1199	5,62	Olfr535	7,13	Fam78b	2,28	Zscan18	5,89
Pik3cg	5,59	Ptpru	7,09	Ripk2	2,18	Cldn20	5,83
Gstm3	5,59	Akr1d1	7,09	Def8	2,14	Pdk1	5,80
Ajap1	5,58	Emp2	7,04	Tbc1d31	2,07	Taar2	5,76
March10	5,58	Ros1	6,95	H2-T3	2,04	Phc1	5,75
Irf8	5,56	Rhoh	6,83	Gpr35	2,03	Riiad1	5,73
E130311K13Rik	5,55	Mdfi	6,80	Rps27l	2,02	Actl11	5,67
Clec3b	5,53	Evi5	6,73	C2cd4d	2,00	Ugt2b5	5,64
Mff	5,53	Olfr1448	6,73	Slc9c1	1,98	Clec9a	5,60
Nat3	5,51	Tcap	6,71	Sidt1	1,96	Ccr10	5,59
Ift80	5,50	Kif1b	6,60	Zmiz2	1,96	Papd4	5,54
Ambra1	5,47	Insm1	6,58	Chsy3	1,91	Ccser2	5,52
Cyp26b1	5,43	Gm13124	6,39	Txndc16	1,90	C3ar1	5,47
1600012H06Rik	5,43	Klra8	6,08	Hacl1	1,89	Mga	5,41
Tmem45a2	5,43	Tnr	5,97	Dgcr14	1,89	Adra1d	5,33
Iqcd	5,39	Zfp40	5,97	Map2k5	1,88	Taar7f	5,31
Gm3604	5,35	Olfr340	5,97	Kcnmb4	1,87	Cxx1c	5,28
Calm1	5,35	Gsk3b	5,93	Scarb2	1,86	Gm5169	5,23
Susd1	5,33	Hivep2	5,81	2310009B15Rik	1,86	Sstr3	5,23
Xpa	5,33	BC021614	5,78	H2-Q7	1,82	Srsf4	5,19
Gsdma	5,32	Htra4	5,68	Tfb1m	1,82	Bcat2	5,15
Zhx1	5,32	Runx2	5,64	BRDN0000737446	1,80	Stx6	5,09
Rabl2	5,30	Tm4sf4	5,52	Ylpm1	1,80	Tlr9	5,07
Nabp1	5,19	Mroh6	5,49	Cotl1	1,80	Fosb	5,04
Tescl	5,12	Tec	5,41	Ifna1	1,78	Ssna1	5,00
Mettl20	5,10	Pacsin2	5,31	Gm44	1,77	Fgfbp3	4,99
Alkbh3	5,10	Gatsl2	5,28	Aurkb	1,76	Eef2k	4,95
Acadm	5,07	Tas2r113	5,07	Cyp2f2	1,75	Alcam	4,95
Atg9b	5,06	Tmem215	5,02	H2-Q10	1,74	Snhg11	4,94
Olfr1153	5,03	Olfr1133	4,93	Sh3gl1	1,74	Olfr1502	4,94
Ccdc153	5,03	Olfr952	4,93	Abhd17c	1,74	Fsip1	4,92
Vmn1r26	5,00	Gm14420	4,88	Iba57	1,73	Ctse	4,89
Sytl1	5,00	Olfr470	4,87	Crlf2	1,72	Hist1h2aa	4,89
Gm8909	4,99	Nudt19	4,86	Plekha3	1,72	Sis	4,89
Olfr653	4,96	Elovl7	4,85	2310036O22Rik	1,71	1700093K21Rik	4,85
Olfr1223	4,96	Lnx2	4,85	Erg	1,69	Ptpro	4,83
Morf4l1	4,95	Pcdhb17	4,81	Mmp13	1,69	Cnn2	4,82
Fam222b	4,94	Tmtc3	4,81	Rbm12b1	1,68	Igf1r	4,79
Satl1	4,94	Smim7	4,81	Pak2	1,67	Zdhhc4	4,78

Syt9	4,93	Slc12a6	4,77	Mettl15	1,66	Avp	4,77
Nt5c3b	4,93	Vmn2r69	4,77	Cacna1c	1,65	Kcng3	4,76
Prr32	4,93	Hoxd9	4,75	Spink12	1,65	Icam1	4,75
Ecm1	4,92	Timp1	4,71	Tshz3	1,63	Olfr794	4,75
Adssl1	4,89	Olfr282	4,69	Ccne1	1,63	Gap43	4,73
Hist1h4k	4,88	Syt2	4,68	Pcm1	1,63	Synpo	4,71
Lilra5	4,88	Usp2	4,67	Ssbp4	1,62	1700023E05Rik	4,71
Pah	4,87	Hcn1	4,66	Scg3	1,62	Cacna1d	4,68
Oasl1	4,85	Ccbe1	4,64	Plod1	1,62	L3mbtl1	4,67
Scube1	4,84	Rdh14	4,64	Dnajc1	1,61	Camta2	4,67
Olfr175-ps1	4,83	Dhx35	4,62	Prkcb	1,61	Tfpt	4,66
P2rx7	4,82	Trim44	4,60	Hkdc1	1,60	Nrarp	4,64
Ube2v1	4,82	Tmem126b	4,59	C130074G19Rik	1,60	Zswim3	4,63
Snx14	4,82	Cryz	4,57	Supt3	1,59	Lrriq4	4,62
Ctla2b	4,79	Iapp	4,57	Bricd5	1,58	Eif3j1	4,61
Rfxap	4,78	Gdi2	4,56	S100a16	1,58	Olfr704	4,59
Abcb11	4,77	Cd177	4,56	Mpv17	1,58	Grin2a	4,58
Coil	4,76	Tspan18	4,55	Slc35g3	1,58	Magea6	4,57
Vasn	4,75	Stard10	4,55	Gmcl1	1,58	Bhlhe41	4,57
Gm13271	4,74	Impad1	4,53	Ubxn10	1,57	Nefm	4,56
Fam187a	4,74	Ugt2a2	4,53	Hsd3b2	1,57	Lct	4,56
Pinlyp	4,72	Rundc3b	4,52	Cgrrf1	1,56	Clec4a3	4,53
Rpl32	4,71	Pcdh9	4,51	Rbck1	1,56	1700013G24Rik	4,51
Mmp9	4,71	Olfr1136	4,50	Gab3	1,56	Nr2c1	4,49
4921501E09Rik	4,71	Susd6	4,50	Tas2r107	1,55	Msmo1	4,48
Cdhr2	4,70	S1pr2	4,49	Lca5	1,54	Tmem242	4,48
Cul7	4,68	Slc38a8	4,49	Xlr3c	1,54	Asb6	4,47
Scgb1b30	4,65	Hist1h4a	4,48	E330014E10Rik	1,54	Tubal3	4,46
P3h2	4,63	Pdzrn4	4,46	Chrnb2	1,54	AI661453	4,44
Colgalt1	4,63	Zfp553	4,43	Kdm5b	1,54	Mug1	4,42
Myod1	4,63	Olfr190	4,42	4933415F23Rik	1,53	Gpr27	4,40
2810428I15Rik	4,61	Cdh11	4,42	Gm7694	1,53	Zfp560	4,40
Aldh1a7	4,61	Mylip	4,41	Ppp2r3c	1,53	Vmn1r27	4,38
Slc30a1	4,61	Tcp10a	4,40	Mrgprf	1,53	Ftmt	4,38
Melk	4,60	Gli1	4,39	Cblc	1,52	Nlrp4a	4,38
Itpkc	4,60	Dpysl5	4,39	Igtp	1,52	Cyb5r4	4,38
Defb28	4,59	Spatc1	4,38	Hsbp1	1,52	AW209491	4,37
Mill2	4,59	Vopp1	4,36	Itgal	1,51	Kcne1	4,36
Sult2a1	4,59	Krtcap2	4,36	Kctd20	1,51	Ppp1cc	4,36
Lrrk1	4,56	Lmbrd2	4,36	Fcer1g	1,51	Rsph14	4,36
Rasal3	4,56	Tmem204	4,35	Psma6	1,51	Kif24	4,36
Olfr389	4,53	Brd9	4,35	Mepe	1,51	L3mbtl4	4,35
Lifr	4,52	Hes3	4,34	Dgat2l6	1,51	Akr1c19	4,35
Ly86	4,51	Ibtk	4,34	Cntn4	1,50	H2-Q7	4,35
Abcg2	4,51	Slbp	4,34	Paip1	1,49	Ly9	4,35
Fzd2	4,50	Erp44	4,33	Zfp82	1,49	Hbegf	4,34
Rps6kl1	4,50	Tmc2	4,32	Olfr877	1,49	Fcer1g	4,34
Tradd	4,50	Cnih2	4,31	Cpt1a	1,48	Kif27	4,33
Zscan22	4,50	Acox3	4,31	Rfpl4	1,48	Ccr3	4,32
Syde2	4,49	Prss39	4,31	Nfkbiz	1,47	Ak4	4,31
Stac3	4,49	Raly	4,31	Slc16a3	1,47	Slc25a46	4,31
Dnaaf2	4,48	Anapc7	4,30	Zar1l	1,46	Olfr506	4,31
Ublcp1	4,47	Marcks	4,29	Tmem101	1,46	Cpa4	4,30
Pgm5	4,46	Olfr116	4,26	Dock6	1,46	Esp4	4,30
Gbf1	4,46	Zfp385a	4,26	Olfr1098	1,46	Nmt2	4,30
Dkk3	4,46	Gbp2b	4,26	Olfr799	1,45	Gria2	4,30
Procr	4,45	Olfr395	4,25	Olfr365	1,45	Tigd4	4,28
Aass	4,44	Phf2	4,23	Ifi35	1,45	Mprip	4,26
Olfr294	4,44	Adhfe1	4,23	Stpg1	1,44	Psmb8	4,25
Gm11758	4,44	Olfr161	4,23	Dnaic1	1,44	Hsp90b1	4,24
Ttc7	4,44	Rabgef1	4,22	Gamt	1,44	Glud1	4,23
Klra4	4,44	H2afy	4,21	Hpca	1,44	Slc45a2	4,23
C2cd2	4,42	E2f7	4,21	Atp6v0e2	1,43	Lrrc23	4,22
Strn3	4,36	Olfr1412	4,21	Nutm1	1,42	Ankrd66	4,21
Ccdc110	4,35	Dnah5	4,20	Srrm1	1,42	Gpr137	4,21
Rnf170	4,34	Irf8	4,19	Atf3	1,41	Plcb4	4,21
Papln	4,33	Napb	4,19	1810011O10Rik	1,41	Sele	4,20
Olfr92	4,32	Taar5	4,17	Scarf2	1,40	Prl7d1	4,20
Sstr4	4,32	Syne4	4,16	Aqp4	1,39	Dcc	4,19
Trim69	4,32	Ceacam10	4,16	Usp24	1,38	Ap1ar	4,19
Vmn1r214	4,32	Dynlt1a	4,15	Actn1	1,38	Wwox	4,18
Cpn1	4,31	Dtna	4,15	Dcun1d3	1,38	Rpl3l	4,18
Camta1	4,30	Ric8	4,14	Lck	1,37	Fut9	4,18
Phf11b	4,30	Itgb1bp2	4,13	St14	1,37	Creg2	4,17
Gm13043	4,29	Coa7	4,13	L3mbtl2	1,37	Olfr1106	4,17
Csn1s2b	4,27	Lrguk	4,12	Pgr	1,36	Gyk	4,16
Prss21	4,26	Olfr1046	4,12	Ankrd39	1,36	Limk2	4,16
Bbs4	4,26	Bpifb1	4,11	Cypt3	1,36	Adgrb1	4,16
Cyb561d2	4,26	Mrgprb3	4,10	Zfp280b	1,36	Myom1	4,15
Adig	4,26	Dpp4	4,09	Zfp641	1,35	Olfr686	4,14
Slc25a16	4,26	Olfr704	4,09	Tprgl	1,35	Syndig1l	4,13
Gpr88	4,25	Sdhaf2	4,08	Cradd	1,35	Synm	4,13

Vmn1r208	4,25	Trim24	4,08	Sec22b	1,34	Olfr267	4,13
Defb21	4,23	Tmem221	4,08	Npas3	1,34	Zic1	4,13
Xkr5	4,23	Acsm2	4,08	Ier2	1,34	Mpp3	4,12
Olfr619	4,22	Proc	4,08	Uts2r	1,34	Foxi1	4,12
Zfp746	4,21	1700008O03Rik	4,08	Dis3l2	1,33	Rasgef1b	4,12
Mapk1ip1	4,21	Vmn1r222	4,07	Defb39	1,33	Vmn2r55	4,12
Foxb1	4,20	4930544G11Rik	4,06	Sult1c2	1,33	1700021F07Rik	4,11
H2-Q1	4,20	Igfl3	4,06	Hist2h2aa2	1,32	Kcnq1	4,11
4930504O13Rik	4,19	Clec16a	4,06	Marco	1,32	Meis1	4,11
Olfr1128	4,19	Shroom2	4,05	Fxyd7	1,31	Brsk1	4,10
Krtap3-2	4,19	Edem1	4,04	Ctsm	1,31	Cpne7	4,09
Lpin1	4,19	Xlr5b	4,04	Kctd15	1,30	Hsd3b2	4,09
Creb3l3	4,17	Rsc1a1	4,04	Stard6	1,30	Tpbpa	4,08
Ppp6r2	4,16	Olfr980	4,04	Ntn3	1,30	Tas2r115	4,08
Cd302	4,12	Rdh12	4,03	Ypel1	1,29	Zkscan5	4,08
Fpr-rs4	4,11	Tubg2	4,03	Cnn1	1,29	Vmn2r38	4,07
Tubb3	4,11	Dyrk1b	4,03	Avp	1,28	Zfp580	4,06
Olfr502	4,11	Ndn	4,02	Pcdh20	1,28	Ubr7	4,05
Olfr819	4,11	Vps9d1	4,01	Cdh22	1,28	Fcrl1	4,05
Gpalpp1	4,09	Csnk1g2	4,01	Pigx	1,28	Klhl17	4,05
Abcc12	4,09	Olfr1509	4,01	Rsrp1	1,28	Olfr109	4,04
Aoah	4,09	Cyp46a1	4,01	Asb3	1,28	Tdpoz5	4,03
Nhs	4,08	Ncoa7	4,01	Flrt3	1,27	Hoxd1	4,03
Zbtb8a	4,08	Cpm	4,00	Arf5	1,27	Olfr583	4,02
Arl5c	4,06	5830411N06Rik	3,99	Nap1l4	1,27	Zfp553	4,01
Mvp	4,04	Mettl22	3,98	Zfp219	1,27	Cxcl10	4,01
Crtc3	4,03	Mapk1ip1	3,97	B3gat1	1,27	Gm14151	4,01
Helb	4,02	Sag	3,97	Pilra	1,26	Nr0b1	4,01
Arhgef19	4,02	Igf1	3,97	Fam188b	1,26	Cbr2	4,01
Aoc3	4,01	Nudt9	3,97	Cdk5r2	1,26	Usp46	4,01
Chst13	4,01	Serpina3k	3,97	Stx16	1,25	Per2	4,01
Ccdc126	4,00	Dennd5a	3,96	Khdc1c	1,25	Il3ra	4,01
Olfr968	4,00	Ugt2a3	3,96	Fam216b	1,25	Bbx	4,00
Tead2	4,00	Ccdc125	3,95	2310030G06Rik	1,25	Fem1b	3,99
Szt2	3,99	Ranbp10	3,95	Pcbp2	1,25	Vmn2r80	3,99
Loxhd1	3,99	Map7	3,95	Igf2r	1,25	Olfr39	3,99
Agps	3,99	Spata18	3,95	Sycp1	1,24	Cntnap1	3,99
Ces5a	3,99	Fxyd6	3,94	Adcy9	1,24	Dytn	3,99
Arl5a	3,98	2010107G23Rik	3,94	Cgref1	1,24	Synb	3,98
Tfe3	3,97	Usp11	3,94	Fam73b	1,24	Olfr1307	3,98
Dusp9	3,95	Gm6484	3,93	Ganab	1,23	Csrp3	3,97
Usp53	3,95	Tmem41a	3,93	Cabp7	1,23	Fut7	3,97
Kcnd2	3,94	Tssk3	3,91	Srgap1	1,23	Kank2	3,97
Wnt8b	3,94	Drg2	3,91	Cracr2b	1,23	Gigyf2	3,97
Naalad2	3,94	Hist1h2ac	3,90	Atg10	1,23	Srd5a2	3,97
Ppp2r5a	3,93	Pvrl2	3,90	Cort	1,22	Ctsq	3,96
Chn1	3,91	Ccdc80	3,90	Fbxo18	1,22	Klhl24	3,94
Rnf186	3,91	Fam122c	3,89	Cypt14	1,22	Stx7	3,94
Primpol	3,91	Bpnt1	3,89	Dnah1	1,21	Errfi1	3,94
Ofd1	3,90	Asb12	3,89	Enpp7	1,21	Tspan15	3,93
Olfr498	3,88	Zmiz2	3,89	Srsf10	1,21	Olfr1301	3,93
Sema3a	3,87	Ybx2	3,88	Bdkrb1	1,20	Fat1	3,93
Gjb1	3,87	Stx19	3,88	Gm1604b	1,20	Ldhd	3,93
Wbp11	3,87	Rxfp2	3,88	4932438A13Rik	1,20	Zfp738	3,93
Tbkbp1	3,87	Amer2	3,88	Krtap3-3	1,19	Poli	3,92
Eps8l2	3,86	B020031M17Rik	3,88	Setd6	1,19	Cypt14	3,92
Il6	3,85	B430306N03Rik	3,88	Dnajb9	1,19	Olfr1173	3,91
Ifih1	3,84	Serpina3b	3,87	Cenpo	1,19	Mmp25	3,91
Hnrnpd	3,83	Egf	3,87	Il22ra2	1,19	Lats2	3,89
Rbfox3	3,83	Ptrf	3,87	Rasip1	1,18	Syce2	3,89
Vwa5b1	3,82	Sestd1	3,87	Hacd2	1,18	Hspa12b	3,89
Lamc3	3,82	Creb3	3,87	Hcar1	1,18	Steap1	3,88
Adck4	3,81	Vmn2r75	3,86	Sycp3	1,18	Anxa1	3,88
Cd19	3,79	Plxna2	3,86	Adgrf2	1,17	Adam17	3,88
Tbpl1	3,79	Col1a2	3,86	Ctsl	1,17	Ttc9b	3,87
Coro1b	3,77	Cylc1	3,85	Olfr1321	1,17	4931429L15Rik	3,87
Clip4	3,77	Rasgef1c	3,85	Olig1	1,16	Zfp362	3,87
Sox13	3,75	Gm4214	3,83	Dhtkd1	1,16	Tmem64	3,86
Stmn3	3,74	Hlx	3,83	Cops8	1,16	Nr1h4	3,86
Lor	3,72	Mrap	3,82	H2-Ob	1,16	Nell1	3,85
Rnf26	3,71	Slc25a16	3,82	Dnase2b	1,16	Nkd1	3,85
1700024P04Rik	3,70	Drg1	3,82	Sptssb	1,16	Ndrg3	3,85
Spats2	3,70	C4b	3,82	Zfand5	1,16	Nxpe4	3,84
Lemd3	3,70	Atp13a5	3,82	Olfr987	1,15	Slc12a6	3,84
Ferd3l	3,70	Msrb1	3,81	Shc4	1,15	Nudt4	3,83
Hbb-bh1	3,70	Mybph	3,81	Spata4	1,15	Ccr6	3,83
Smok2b	3,69	B4galt2	3,80	Sept9	1,15	9430007A20Rik	3,83
Cldn7	3,69	Mageh1	3,80	Rnf181	1,15	4930578C19Rik	3,83
Fgl1	3,69	Uhmk1	3,80	Taz	1,15	Xiap	3,83
Vmn2r93	3,68	Sfr1	3,80	Chrng	1,15	Ccdc171	3,82
Pga5	3,67	Gbas	3,80	Edc3	1,15	Mtmr10	3,82
Pramel6	3,67	5530400C23Rik	3,80	Tfcp2	1,14	Slc35f2	3,82

Erlin1	3,67	Ap2a1	3,79	Zfp146	1,14	Irs4	3,82
Wdr45b	3,67	Pax6	3,79	Fibin	1,14	Rbm4	3,81
Comtd1	3,65	Calhm2	3,79	Rxfp3	1,14	Htr7	3,81
Enah	3,65	Adcy4	3,78	Cebpzos	1,14	Oard1	3,81
Wt1	3,65	Marveld3	3,78	Dnajc4	1,13	Cdyl2	3,80
Comt	3,65	Olfr1396	3,78	Rbpms2	1,13	Ppig	3,80
Cdkn2a	3,64	Ankrd54	3,77	Gstm3	1,12	Etaa1	3,79
Prdx5	3,63	Slc6a9	3,77	Sox13	1,12	Ptpra	3,79
Rnft1	3,62	Acpp	3,76	Usp10	1,12	Acp2	3,79
Pyhin1	3,59	Cxx1c	3,76	Lamtor1	1,12	Iltifb	3,79
1700020L24Rik	3,57	Ism1	3,76	Elof1	1,12	Htr1d	3,78
Ak3	3,55	Kncn	3,76	Fn3krp	1,12	Pgm2	3,78
Defb4	3,54	Serpinb1b	3,75	Hccs	1,12	Olfr731	3,78
Gm5294	3,54	Kcnk3	3,75	Rptn	1,12	Cltc	3,78
Olfr705	3,53	Tas2r131	3,74	Tnnt1	1,12	Tcp10c	3,77
Pink1	3,53	Fbxo46	3,74	Zfy1	1,12	Card10	3,77
Pramef12	3,52	Crisp1	3,74	Zc3h12b	1,12	Olfr1419	3,77
Krt18	3,51	Alkbh7	3,74	Cdh26	1,12	Stoml3	3,77
Cplx1	3,51	Fndc4	3,73	Dnajc7	1,11	Cep295	3,77
Gab1	3,51	Krt40	3,72	Scara3	1,11	Gareml	3,76
Dgkg	3,50	Meox1	3,72	Fam92a	1,11	Srgap2	3,76
Tmem129	3,49	Olfr818	3,72	H2-Q6	1,11	Olfr825	3,75
Gchfr	3,49	Shisa2	3,72	Vmn1r179	1,11	Fndc4	3,75
Col6a1	3,49	Abcd2	3,70	Lrrc73	1,11	Zc3h12c	3,75
Kcnc4	3,48	Klhdc3	3,70	Cflar	1,10	A1cf	3,75
Tex22	3,48	Rad9b	3,70	4930444G20Rik	1,10	Tmem39a	3,75
Aqp11	3,48	Phf12	3,69	Ribc2	1,10	Tff1	3,74
Iqck	3,47	Gm9513	3,69	Cd274	1,10	Ywhae	3,74
Prl2c1	3,46	Lypd6b	3,69	Gm4763	1,10	Krtap3-2	3,74
Mrps23	3,46	Tob2	3,69	Olfr619	1,10	Fam13a	3,74
Cep83	3,45	Kif5c	3,69	Ss18	1,10	Fkrp	3,74
Mtnr1a	3,42	Nrl	3,69	Olfr1396	1,10	Osgep	3,73
Manea	3,40	Cdo1	3,68	Tekt3	1,09	Nek3	3,72
Neurod2	3,40	Lrrc20	3,68	Acrbp	1,09	Aadac	3,72
Olfr799	3,39	Syt6	3,68	Cthrc1	1,09	Ubc	3,71
Vmn1r45	3,39	Auh	3,67	Lkaaear1	1,09	Apeh	3,71
Nf1	3,39	Slc52a3	3,67	Olfr1164	1,08	Sfxn2	3,71
Sqrdl	3,37	Hspbp1	3,67	Vamp1	1,08	Dync1i1	3,70
Ptk2b	3,37	Rab26	3,67	Zfp319	1,08	Sec61g	3,70
Ptp4a1	3,36	Ppp1r14d	3,67	Zfp697	1,08	Tlk1	3,70
Lamp5	3,35	Gps2	3,67	Thumpd3	1,08	P2rx4	3,70
Bahd1	3,34	Ell	3,66	Srp72	1,08	Olfr1457	3,69
Fem1a	3,34	Rbm15b	3,66	Sprr4	1,07	Cyb5a	3,69
Tnf	3,32	Lca5l	3,66	Tmco2	1,07	Aste1	3,69
Samd1	3,31	Tmem240	3,65	Med16	1,07	Adam26a	3,68
Hectd3	3,31	Cxcl2	3,65	Plvap	1,07	Ivl	3,68
Gnai1	3,31	Gpatch2l	3,65	Olfr235	1,07	Col26a1	3,68
Fam72a	3,29	Olfr433	3,65	G3bp1	1,07	Slitrk5	3,68
Cpne5	3,29	Prex1	3,64	Ttll4	1,07	P2ry10	3,67
Ovch2	3,27	Tap1	3,64	Drp2	1,07	Unc93b1	3,67
Ces1a	3,27	Tmem132b	3,63	Tmem100	1,07	Slfn4	3,67
Heatr5a	3,27	Lpcat3	3,63	BRDN0000737634	1,07	Col20a1	3,67
Id3	3,25	Gfi1b	3,63	Ccdc59	1,07	Slc39a5	3,67
Akap9	3,24	Sec22a	3,63	Trim36	1,07	Mcam	3,67
Ripply2	3,24	Cntnap5b	3,63	Olfr1221	1,06	Krtap2-4	3,66
4930578C19Rik	3,21	Anks4b	3,63	Smo	1,06	Olfr508	3,66
Rhox2g	3,21	Pdia3	3,63	Socs1	1,06	Ubd	3,66
Gadd45g	3,20	Det1	3,62	Rtp3	1,06	D11Wsu47e	3,66
Fyb	3,19	Vmn1r83	3,62	Dpy19l2	1,05	Ssh2	3,65
Apob	3,19	Csrp2	3,62	Spef1	1,05	Strn3	3,65
Plac8l1	3,18	Saysd1	3,62	Gm10100	1,05	Otud7b	3,65
Tm6sf1	3,18	Ttc12	3,62	Arl6	1,05	Pdxk	3,65
Chka	3,18	Tns1	3,61	Dlg2	1,05	Gm14374	3,64
Tmem132d	3,17	Rrad	3,61	Rbbp6	1,05	Fgfr3	3,64
Mettl9	3,16	Egfl8	3,60	Nsmce2	1,05	Ovch2	3,64
Otud1	3,13	Serpinb5	3,59	Gm12429	1,04	Apoc2	3,64
Slc17a5	3,12	4932438H23Rik	3,59	Ctcfl	1,04	2010107G23Rik	3,64
Fam107b	3,11	Gzma	3,59	Tyrobp	1,04	Hnrnpab	3,64
Zbp1	3,10	Gfral	3,59	Adam18	1,04	Cd320	3,63
Vamp1	3,05	Tfap4	3,59	4933405L10Rik	1,04	Rnf149	3,63
Defb45	3,04	Scgb2b2	3,58	G3bp2	1,03	Ndn	3,63
Rad51b	3,04	Cyb5r4	3,58	Zswim6	1,03	Slco6c1	3,63
Pcp4	3,04	Zfp654	3,57	Polm	1,03	Alkbh4	3,63
Gramd2	3,03	Esr2	3,57	Btbd16	1,03	Qsox2	3,63
Rbm11	2,99	Lonrf1	3,57	Hspbp1	1,03	Mettl20	3,63
Hdac1	2,97	Tmem37	3,57	Tfap2b	1,03	Bhlhe22	3,62
Kif6	2,96	Slc46a2	3,57	Rbm34	1,03	Naa60	3,62
Ankrd9	2,93	Pmfbp1	3,57	Mbnl2	1,02	Cep72	3,62
Pdlim2	2,90	Pip5kl1	3,56	Rnf19a	1,02	Yipf2	3,61
Zfp641	2,89	Ubtd2	3,56	Fbn2	1,02	Ankrd60	3,61
Rhox2h	2,89	Eid2b	3,55	Gdf6	1,02	Lemd1	3,61
Doc2b	2,86	Klhl10	3,55	Hif1an	1,02	Cers4	3,61

Hlf	2,84	Kdm4a	3,55	Zfp57	1,02	Defb48	3,60
Cox7a2	2,83	Spaca3	3,55	Sos1	1,01	Zdhhc22	3,60
Scgb2b19	2,82	Cln5	3,54	Ncald	1,01	Rax	3,60
Ubxn8	2,81	Vmn1r117	3,53	E330017A01Rik	1,01	Tfe3	3,60
Carm1	2,79	Eln	3,53	1190005I06Rik	1,01	Olfr195	3,59
Gm12169	2,78	Nyap2	3,53	Ldlrad1	1,01	Rbfox1	3,59
Tomm40	2,77	Syna	3,53	Ly6g6d	1,00	Abhd17a	3,59
Hmg20b	2,77	Hipk4	3,53	Herc1	1,00	Cic	3,59
Necab3	2,76	Hoxc8	3,52	Sema4f	1,00	Ergic2	3,59
Cntln	2,76	Cdk16	3,52	1700123O20Rik	1,00	Tdpoz4	3,59
Drd3	2,75	Gnb1l	3,51	Astn2	1,00	Cyp2a12	3,59
Taar9	2,71	Fasl	3,51	Ltbp4	1,00	Susd5	3,59
Dcaf10	2,70	Mtfmt	3,50	Ppp1r3c	1,00	Cul9	3,59
Pomgnt1	2,63	Vmn2r35	3,50	Mgme1	1,00	Rabep2	3,59
Olfr193	2,60	Krt82	3,49	Gm13279	1,00	Phyhipl	3,59
Gm6890	2,57	Rbms2	3,49	Gpr82	1,00	4933427D06Rik	3,59
Tmem194	2,53	A530099J19Rik	3,49	Adck3	1,00	Prdm1	3,58
Hmgn2	2,50	Mpdu1	3,48	Aimp1	1,00	Wdfy3	3,58
Ung	2,49	Ghrhr	3,46	Sh2b3	0,99	Tecr	3,58
1700113H08Rik	2,48	Gpr180	3,45	Tmem225	0,99	Csnk2a2	3,58
Adcy3	2,47	Calr	3,45	Fah	0,99	Cryzl1	3,57
Tfg	2,44	Trip10	3,45	Rgp1	0,99	Nrg2	3,57
BC029214	2,41	Rarb	3,44	Mbnl1	0,99	Fam102a	3,57
Dnajb13	2,41	Snca	3,44	Hmmr	0,99	Mcpt2	3,56
Olfr172	2,40	1700092M07Rik	3,43	Slc30a7	0,99	Drd5	3,56
Smpd5	2,39	Hrc	3,43	Gm1045	0,99	Pm20d1	3,56
Slc18b1	2,39	Acat2	3,42	Lgals2	0,99	Zfp442	3,55
Rap1gap2	2,36	Dlc1	3,42	Klk1b11	0,99	Olfr772	3,55
BRDN0000738036	2,36	Slc34a3	3,41	Emc6	0,99	Oaf	3,55
Nrap	2,33	1700080E11Rik	3,41	Srd5a3	0,99	Nkx6-2	3,55
Abhd16a	2,30	Tmem88b	3,41	Eid2b	0,98	Rab39b	3,54
Fah	2,29	4933411K16Rik	3,41	Nim1k	0,98	Nedd9	3,54
Gpr165	2,28	Gm156	3,41	D5Ertd579e	0,98	Pkd2	3,53
Cav2	2,28	Abr	3,41	Tspyl1	0,98	Lyplal1	3,53
Cyp24a1	2,25	Gm8677	3,41	Krtap4-2	0,98	Gm7257	3,52
Wasf3	2,23	Rasgef1b	3,41	Tbc1d2	0,98	Taar6	3,52
1700016C15Rik	2,20	Grid2ip	3,40	Cyp2j5	0,98	Gm6121	3,51
Actl9	2,15	Dennd4b	3,40	Mrgpra2a	0,98	Zfyve19	3,51
Timp3	2,15	Spink11	3,40	Ism2	0,98	Efhd1	3,51
Med14	2,12	Orc2	3,40	Zfp458	0,97	1700019N19Rik	3,51
Hsd3b3	2,12	Nhlrc3	3,40	4932443I19Rik	0,97	Cutal	3,51
Trat1	2,11	Atoh1	3,40	Cyb5b	0,97	Arl2bp	3,51
Trim38	2,09	Olfr901	3,39	Slc1a7	0,97	Nudt7	3,50
Timm8a2	2,09	Cadps2	3,39	Klf13	0,97	Pgm1	3,50
Steap2	2,08	Serhl	3,39	Cdh2	0,97	Gabra1	3,50
Galnt3	2,06	Scrib	3,39	Pklr	0,97	Gm5878	3,50
Zgpat	2,06	Nop14	3,39	Vipr2	0,97	Phf19	3,49
Orc2	2,05	Arhgef5	3,39	Fxn	0,97	Sostdc1	3,49
4930428D18Rik	2,04	Ston2	3,39	Sf3b2	0,96	Gorasp1	3,49
Olfr108	2,03	Bcl6b	3,39	Cmtm5	0,96	Taf5	3,49
Etaa1	2,03	Chst13	3,38	Olfr693	0,96	Tomm40l	3,48
Chchd7	2,02	4930513O06Rik	3,38	Rcan2	0,96	2510039O18Rik	3,48
Fkbp8	2,01	Afap1l1	3,38	Epb4.1l1	0,96	Gm5464	3,47
Slc45a4	2,01	Hs1bp3	3,38	Prkd3	0,96	Dapl1	3,47
Cab39l	2,01	Adap1	3,38	Kcnk16	0,96	Xpa	3,46
Ttc27	2,01	Slc17a5	3,37	Fam131a	0,96	Rell1	3,46
Vsig1	2,00	Mdp1	3,37	Ogdh	0,95	Krt84	3,46
Unc13d	1,99	Uts2	3,37	Stap2	0,95	Cdc42ep5	3,46
Dhx30	1,98	Zfp619	3,36	Wfdc21	0,95	Lman2l	3,45
Rab8b	1,97	2310061N02Rik	3,36	Eif5b	0,95	Apba1	3,45
Tmcc1	1,97	Myo18a	3,35	Mnat1	0,95	Gstm5	3,45
Rasa3	1,95	Skil	3,35	Slc45a1	0,95	Mrgpra1	3,44
Cnn1	1,95	Olah	3,35	Cbs	0,95	Lingo3	3,44
Tnfrsf21	1,95	Nusap1	3,35	Fam71e2	0,94	Cyp2a5	3,44
Lpp	1,94	E2f5	3,35	Lrrc74a	0,94	Dpep2	3,44
Lpar3	1,93	Usp13	3,34	Man2b1	0,94	Vmac	3,44
Insl6	1,93	Bckdha	3,33	Sbk3	0,94	Lrrc48	3,44
Gm5795	1,92	Socs7	3,33	Erbb2	0,94	Fam43a	3,43
Vmn2r34	1,92	Nptx1	3,33	Aldh1a2	0,94	Olfr536	3,43
Ssr2	1,92	Oosp3	3,32	Zcchc13	0,94	Cabp1	3,43
Cblb	1,92	Aste1	3,32	Tgif2lx1	0,94	Klf15	3,43
Plod1	1,91	Cstf2	3,32	Ssr4	0,94	Rspry1	3,43
BRDN0000738356	1,90	Epm2a	3,32	Coro1a	0,93	Calcb	3,42
Sh3kbp1	1,89	Car15	3,32	Pitx2	0,93	Oprm1	3,41
5730508B09Rik	1,89	Olfm3	3,32	3830403N18Rik	0,93	Ccdc166	3,41
Olfr446	1,89	Echs1	3,32	Olfr1240	0,93	Hao2	3,41
Krtap4-1	1,87	Lemd3	3,32	Prr16	0,93	Bcl7a	3,41
Olfr1356	1,87	Olfr25	3,31	Efemp1	0,93	Fkbp1b	3,40
Zfp60	1,87	Gfra1	3,31	Lce3a	0,93	Olfr419	3,40
BRDN0000738242	1,86	Armc5	3,31	Zfp407	0,92	Bex2	3,40
Krtap4-2	1,86	Pdgfrb	3,31	1700092M07Rik	0,92	Coq9	3,40
Olfr113	1,86	Fnbp4	3,30	Cox6b2	0,92	Ccdc64b	3,40

Aipl1	1,85	Gm13125	3,30	Tceanc2	0,92	Smco2	3,40
Six3	1,85	Lrpprc	3,30	Vmn1r195	0,92	Crcp	3,39
Pdc	1,84	Ccdc47	3,30	Slc6a8	0,92	Chpf2	3,39
Hrh2	1,84	2210016L21Rik	3,29	Col9a2	0,92	Aimp1	3,39
Fst	1,84	Zfand6	3,29	Usp4	0,92	Btaf1	3,38
Nptx1	1,84	Gp1ba	3,29	Olfr1417	0,92	Ccnj	3,38
Gramd3	1,83	Efna1	3,29	Diablo	0,92	Tmcc1	3,38
Cygb	1,83	Vsig4	3,28	Zcchc14	0,92	Marveld3	3,38
Olfr427	1,83	Lig3	3,28	Zfp784	0,92	Cd79b	3,38
Olfr1180	1,83	Esrra	3,28	1700019B03Rik	0,91	Slamf9	3,38
Olfr741	1,83	Zfp61	3,28	Spcs3	0,91	Ppfibp2	3,38
Ppap2c	1,83	Mrpl51	3,27	Tdrd9	0,91	Evi2b	3,36
Hecw1	1,82	Vmn2r82	3,26	Rexo4	0,91	Apbb2	3,36
Creld1	1,82	Celsr2	3,26	Klf7	0,90	Ece2	3,36
Vmn1r139	1,82	Fbxw24	3,26	Mobp	0,90	Ppl	3,35
Ubxn11	1,81	Olfr676	3,25	Ttc28	0,90	Ppargc1b	3,35
Olfr469	1,81	Nhlrc1	3,25	Zfand3	0,90	Sp2	3,35
Dusp18	1,81	Tmem231	3,25	Slc27a3	0,90	Luzp2	3,35
Cd3d	1,80	Olfr120	3,25	Opn4	0,90	Scgb1b19	3,35
Col23a1	1,80	Olfr46	3,25	Rufy4	0,90	Tlr13	3,34
Nudt2	1,80	Olfr554	3,25	Gpatch1	0,90	Grina	3,34
Eif3a	1,80	Ak2	3,25	Slc6a2	0,90	Apba3	3,34
Sprr2d	1,80	Clec12b	3,24	Morf4l2	0,90	Decr2	3,34
Cnr1	1,80	Nt5c3	3,24	Tspan31	0,90	Ggt1	3,34
Fabp12	1,80	Gm4745	3,24	Olfr1364	0,90	Pmp22	3,33
Vmn2r21	1,80	Zfp112	3,24	Snx30	0,90	Pcsk9	3,33
Ep300	1,79	Rabep2	3,24	Nlrp9c	0,90	Tmem54	3,33
Pip5kl1	1,79	Olfr961	3,24	Slc22a28	0,90	Syt1	3,33
Znrd1as	1,79	Slc7a15	3,23	Cpa5	0,90	Ntsr2	3,33
Vmn2r26	1,79	Pnma3	3,23	9430007A20Rik	0,89	N4bp3	3,33
Irf9	1,79	Rwdd1	3,23	Fam193a	0,89	Gfi1	3,33
Olfr494	1,79	Coro1a	3,23	Nckipsd	0,89	Plec	3,33
Cys1	1,78	Agpat5	3,22	Sesn3	0,89	Tsc22d4	3,33
Zfp84	1,78	Clic6	3,22	Zfand6	0,89	Gm11565	3,33
Cd44	1,78	Fbll1	3,22	Trappc10	0,89	Gm13128	3,33
Wdr20rt	1,78	1700067P10Rik	3,22	Irgm1	0,89	Olfr1320	3,32
Lrrc59	1,78	Magea1	3,21	BRDN0000738349	0,89	P2ry2	3,32
Prss12	1,78	Jag2	3,21	Spink10	0,89	Ibtk	3,32
Phf19	1,78	Lama4	3,21	Snapin	0,89	Arid4b	3,32
Stxbp5l	1,78	Pdp1	3,21	Prmt8	0,89	Ngfrap1	3,32
Gm5132	1,77	Zfp119a	3,21	Utp15	0,89	Vmn2r90	3,31
Ift27	1,77	Mdga1	3,20	Psmd11	0,89	Fam166a	3,31
Gm8898	1,76	Ginm1	3,20	C1rb	0,89	Mlf1	3,31
Cd79b	1,76	Slc4a11	3,19	1700013D24Rik	0,88	Dnah6	3,31
Vmn1r22	1,76	Slc35d3	3,19	Rp1	0,88	Ibsp	3,31
Tmem63b	1,76	Olfr273	3,19	Tinf2	0,88	1700066B19Rik	3,30
Peak1	1,75	Adgrg7	3,19	Hsd17b14	0,88	Rhox4a	3,30
Cdkn3	1,75	Krtap11-1	3,19	Vps52	0,88	Lasp1	3,30
Dlat	1,75	Tpst1	3,19	Anxa6	0,88	Chst13	3,30
Bcat2	1,75	Defa22	3,19	Serpinb2	0,88	Fxr1	3,29
Obox1	1,75	Usp6nl	3,19	Araf	0,88	Fbln2	3,29
Nrg4	1,74	Ctsd	3,18	Ankrd61	0,88	Ndnf	3,29
Tgm5	1,74	Vmn1r32	3,18	Pabpc6	0,88	Atf5	3,28
Tfip11	1,74	Ccdc126	3,18	Gpr151	0,88	Zxdc	3,28
Tdh	1,74	Zfand3	3,18	Snx32	0,88	Gm6696	3,27
Klra9	1,74	Rhox9	3,18	Ppp2r1b	0,88	Nol7	3,27
Acta2	1,74	Frs3	3,17	Hhat	0,88	Olfml2a	3,26
Nup54	1,73	Il11	3,17	She	0,88	Dmp1	3,26
Zfp322a	1,73	Mettl21a	3,17	Csrp2bp	0,87	Rab27b	3,26
Celf6	1,73	Foxp2	3,17	Asl	0,87	Bbip1	3,26
Lmo4	1,73	Parpbp	3,17	Lcn9	0,87	Car4	3,26
Sec61a2	1,73	Gpx5	3,17	Tbc1d21	0,87	E130311K13Rik	3,26
Prph	1,73	Irs4	3,16	Baiap2l1	0,87	Tulp1	3,26
Smap1	1,73	Myl1	3,16	Zpr1	0,87	2310050C09Rik	3,25
Rfx3	1,73	Tsnaxip1	3,16	Olfr1045	0,87	Ppap2b	3,25
Vmn2r17	1,72	Trpm5	3,16	Arsa	0,87	Zfp7	3,24
Cactin	1,72	Nccrp1	3,15	Srebf1	0,87	Add2	3,24
Slc4a10	1,72	Tnfsf15	3,15	Senp1	0,87	Engase	3,24
Oplah	1,72	Bricd5	3,15	Tmem150b	0,87	Slamf8	3,23
Gpr62	1,72	Zfp446	3,15	Vmn2r1	0,87	Mfap4	3,23
Phactr3	1,71	Tgm1	3,15	Smyd5	0,87	Epha3	3,23
Tshz3	1,71	Bcl2a1b	3,15	Cdca7l	0,87	Nhlrc4	3,23
Cox15	1,71	Anks3	3,15	Rfx5	0,86	Hba-a1	3,23
Zfp574	1,71	Cd53	3,14	Vmn1r27	0,86	Clec4a4	3,23
Tas2r105	1,71	Ssr3	3,14	Dapk1	0,86	Ptgdr	3,23
Setd7	1,71	Kcne3	3,14	Atg5	0,86	Fndc3c1	3,23
Sema4a	1,71	Gpr55	3,14	Scgb1b2	0,86	Nr2f6	3,23
Slc13a3	1,71	Slc14a2	3,14	4930538K18Rik	0,86	Klf6	3,22
Myo10	1,71	Tfcp2l1	3,14	Gadd45a	0,86	Aqp11	3,22
Dbh	1,70	Igtp	3,13	Eid1	0,86	Pou2f1	3,22
Dnajc15	1,70	Trpc3	3,13	Dbr1	0,86	Aplnr	3,22
Gpr31b	1,70	Epb4.1l4b	3,13	Cd7	0,86	Clmp	3,22

Zmym4	1,70	Inpp5k	3,13	Tex38	0,86	Lrrc8d	3,21
Vmn2r111	1,69	Bfar	3,12	Zscan4b	0,85	Lipg	3,21
Mrpl52	1,69	Bloc1s4	3,12	Sox8	0,85	Rprm	3,20
D430041D05Rik	1,69	Dag1	3,12	Olfr658	0,85	Dffa	3,20
Tm4sf1	1,69	Abcc12	3,12	Akr1c6	0,85	Trpm4	3,20
Dpp7	1,69	Barx2	3,12	Btg3	0,85	1810046K07Rik	3,19
Cyp27b1	1,68	Olfr139	3,11	1700066M21Rik	0,85	Wfdc16	3,19
Fem1c	1,68	Mtfr1	3,11	Gm15085	0,85	Olfr281	3,19
Olfr450	1,68	Neu3	3,11	Anxa3	0,85	Klf7	3,18
Vmn1r198	1,68	1700024P16Rik	3,11	Mdm1	0,85	Rassf10	3,18
D030056L22Rik	1,68	Plch1	3,11	Sod3	0,85	Gpr39	3,18
Mlph	1,68	Fbxo38	3,10	Cx3cl1	0,85	Cnn1	3,18
Cideb	1,68	Dhx32	3,10	Asic3	0,85	Ulbp1	3,18
Mtr	1,68	Ctso	3,10	Slc5a4a	0,85	Ash1l	3,17
Ifna2	1,68	Spryd3	3,10	Cplx1	0,85	Eml6	3,17
Ccdc155	1,68	Rtn1	3,09	Triml1	0,85	Nhlrc1	3,16
Polk	1,68	Apob	3,08	Sh2d3c	0,85	Dzip1l	3,16
Gm10406	1,67	Pcbp2	3,08	4933402N03Rik	0,85	Tet1	3,16
Rab15	1,67	Gm13290	3,08	Prdm9	0,85	Olfr555	3,16
Recql5	1,67	Gm7157	3,08	Actl9	0,84	Myo18a	3,15
Cetn1	1,67	Atp6v1g3	3,08	Pdcd10	0,84	Foxc1	3,15
Stk26	1,67	Olfr1290	3,08	Zfp808	0,84	Guf1	3,15
Qsox2	1,67	Atp6v1c1	3,08	Cenpv	0,84	Dmrtc1b	3,15
Gsn	1,67	Ubl4b	3,08	BC048679	0,84	Sec22a	3,14
Fxn	1,67	Kif5a	3,07	Pvalb	0,84	Mog	3,14
Wfdc13	1,67	Gyk	3,06	Mink1	0,84	Glcci1	3,14
Fcnb	1,67	Gm128	3,06	Klk1b8	0,84	H1fx	3,14
Ankrd22	1,67	Olfr344	3,06	Ccdc88a	0,84	Lrrc28	3,13
Olfr63	1,67	Plagl1	3,06	Sec63	0,84	Pkd1l2	3,13
Trib1	1,67	Spag11b	3,06	Cxcl10	0,84	Tfeb	3,13
Scn2a1	1,66	Tm4sf20	3,05	Rnf113a1	0,84	Usp27x	3,12
Fibin	1,66	Lman2	3,05	Morc2b	0,84	Commd2	3,12
Lonp1	1,66	Taldo1	3,05	Foxf2	0,84	Adgrg2	3,11
Sdhc	1,66	Ikbke	3,05	Slc52a3	0,84	Pla2g5	3,11
Zbtb48	1,66	Olfr1065	3,04	Ldah	0,83	Padi2	3,11
Olfr1158	1,66	Epha3	3,04	Fam45a	0,83	Fsbp	3,10
Rhag	1,66	Fam83e	3,04	Vmn1r56	0,83	Rbm44	3,10
Pnpla6	1,66	Ldlrad4	3,04	Spag11a	0,83	Fat2	3,10
Nxt2	1,66	Il20rb	3,04	Fam114a2	0,83	Hes5	3,10
Atp13a2	1,65	Des	3,04	Gclc	0,83	G3bp2	3,10
Trip11	1,65	Nlrp4c	3,04	Lalba	0,83	Ttc16	3,10
Ly6i	1,65	Slx4ip	3,04	Clec16a	0,83	Rhobtb3	3,09
Poli	1,65	Olfr187	3,04	Atg2b	0,83	Lrrc51	3,09
Awat2	1,65	Luzp2	3,04	Haus8	0,83	Ing3	3,09
Elavl2	1,65	Rasgrp2	3,04	BRDN0000737772	0,83	Adra2a	3,09
Mrps17	1,65	Rassf4	3,04	Ces3b	0,83	Olfr62	3,08
Chrna5	1,65	Crtac1	3,03	Gm15114	0,83	Rbm11	3,08
Txndc5	1,64	Mlycd	3,03	Scgb1b7	0,82	Opa3	3,08
Ssh3	1,64	Fbxo47	3,03	Meis2	0,82	4833439L19Rik	3,08
Paip1	1,64	Cand2	3,02	Olfr819	0,82	E130308A19Rik	3,08
Ago4	1,64	Lamc1	3,01	Vmn1r10	0,82	Izumo1	3,07
Trp53	1,64	Erlec1	3,00	Angptl1	0,82	Spdef	3,07
Gja8	1,64	Ell2	3,00	Asphd2	0,82	Rusc2	3,07
Gm6904	1,64	Sh3bp5	3,00	Esx1	0,82	Fam174a	3,07
Olfr1283	1,63	Defb14	3,00	Tsga8	0,82	1700006A11Rik	3,07
Scgb2b12	1,63	Tamm41	3,00	Cdk15	0,82	Ggt6	3,07
Zyg11b	1,63	Itgb1bp1	3,00	Bdnf	0,82	Ebag9	3,07
Prr19	1,63	Slc9b2	2,99	Nudt4	0,82	Cep170	3,06
Myoz3	1,63	Gpr85	2,99	Timp4	0,81	C7	3,05
Ndufs5	1,63	B230219D22Rik	2,99	Slc12a5	0,81	Plcz1	3,05
Mt3	1,63	Otop3	2,99	Svs4	0,81	Pth2r	3,05
Fam209	1,63	Olfr382	2,99	Dkk2	0,81	Alk	3,05
Dsn1	1,63	Nfatc4	2,98	Gm13023	0,81	Mettl24	3,05
Adam21	1,62	Uhrf2	2,98	Cog4	0,81	Dab2ip	3,04
Zfx	1,62	Parp1	2,98	Lims1	0,81	Xpo7	3,04
Gm10229	1,62	Dscc1	2,98	Olfr1496	0,81	Il6	3,04
Itgb6	1,62	Sntb2	2,97	Ccdc85c	0,81	Nell2	3,04
Zcchc14	1,62	March2	2,97	Cdk2ap1	0,81	Pitx3	3,03
Dyx1c1	1,62	Rbm11	2,97	Fut7	0,81	Serpina3m	3,03
Scnm1	1,62	Ssh3	2,97	Teddm1a	0,81	Tnni1	3,03
Dnajc1	1,61	St6galnac3	2,96	Treml4	0,81	Gbe1	3,02
Jakmip2	1,61	Tram2	2,96	Myh13	0,81	Myt1	3,02
Utrn	1,61	Trp63	2,96	Zyg11a	0,81	Sppl2c	3,01
Gpr27	1,61	E4f1	2,96	Chek2	0,81	Krt7	3,01
Ncoa3	1,61	Fcrlb	2,96	Olfr520	0,81	Sim2	3,01
Fbxo45	1,61	Gm525	2,96	Fbxo22	0,80	Gabrb1	3,01
Naa15	1,61	Lgsn	2,96	Mamld1	0,80	Olfr827	3,01
Olfr341	1,61	Osbpl1a	2,96	Ngef	0,80	Olfr967	3,01
Scarb2	1,61	4921509C19Rik	2,95	Etv5	0,80	Lrch3	3,00
Cdk5rap2	1,60	Ust	2,95	Osbp	0,80	Trpc6	3,00
Tceal3	1,60	Cer1	2,95	Zfp69	0,80	Park7	3,00
Cyp2c54	1,60	Arl8b	2,95	4933421I07Rik	0,80	Tor2a	3,00

Ccnb3	1,60	Ptger1	2,95	Timm10	0,80	Dedd2	2,99
Ribc2	1,60	Arhgap6	2,95	Scn11a	0,80	1810037I17Rik	2,99
Rpl41	1,60	Trim50	2,95	Lipt1	0,80	Hpx	2,99
Krtap16-1	1,60	Cdkn1b	2,95	Tomm5	0,80	Tmem8	2,99
Uqcrb	1,60	Tmx4	2,94	Olfr342	0,79	Npepps	2,98
Cldn23	1,60	Olfr298	2,94	Pard6a	0,79	Gpr158	2,98
Olfr480	1,60	Erich4	2,94	Shc2	0,79	Olfr1495	2,98
Kcns3	1,60	Mcpt4	2,94	Sez6l	0,79	Capn10	2,98
Gpr65	1,60	Dock1	2,94	BRDN0000738365	0,79	Acer1	2,98
Cd3g	1,59	Syne3	2,94	Cyp2c70	0,79	Nutm1	2,98
Ppp1r3b	1,59	Spata2	2,92	Usp20	0,79	Wasf1	2,98
B4gat1	1,59	Serpina3m	2,92	Plekhb1	0,79	Pcdh1	2,97
Olfr139	1,59	Ppef2	2,91	Vmn1r235	0,79	Ubqln1	2,97
Pramel7	1,59	Smim18	2,91	Cnksr1	0,79	Trim43c	2,97
Cyp4a30b	1,59	Isyna1	2,91	Wars	0,79	Dlgap3	2,97
Zfp229	1,59	Pcdhb7	2,90	Olfr134	0,79	Syt3	2,96
Olfr926	1,59	Ptk2b	2,90	Mgrn1	0,79	Tbcel	2,96
Tipin	1,58	Myo10	2,90	E430025E21Rik	0,78	Ap5b1	2,96
Defb50	1,58	Zfp13	2,90	Scn8a	0,78	Rchy1	2,96
Shroom1	1,58	Krtap1-3	2,90	Ifna14	0,78	Ppp2r1b	2,96
Slc10a5	1,58	Fam71b	2,89	Olfr917	0,78	Ovol2	2,95
Zfp449	1,58	Mical1	2,89	Zfp606	0,78	Smpd2	2,95
Zfp488	1,58	Efcab11	2,89	Olfr272	0,78	Gm20826	2,95
Anxa4	1,58	Zfp319	2,89	Tmem50b	0,78	BC037034	2,94
Aph1a	1,58	Atp10a	2,89	Tex2	0,78	2610528J11Rik	2,94
Atxn7l2	1,58	Syk	2,89	Limk2	0,78	C2cd2l	2,94
Rbpms2	1,58	Fjx1	2,89	Plp1	0,78	Brinp2	2,94
Gm9994	1,58	Tpr	2,87	Cxcr6	0,78	Zeb1	2,94
Igsf8	1,58	Pdia6	2,87	C1ra	0,78	Ap3s1	2,93
Got2	1,57	Tcp11l2	2,87	Chd4	0,78	Acsl3	2,93
Ndor1	1,57	Slc2a8	2,86	Vmn1r120	0,78	Vmn2r115	2,93
Sult5a1	1,57	Vmn1r75	2,86	Atp5j2	0,78	Il9r	2,93
Mina	1,57	Lmtk2	2,86	Trak1	0,78	Efcab5	2,92
Olfr247	1,57	2310067B10Rik	2,85	Olfr1263	0,78	Fam109a	2,92
Vps52	1,56	Fcamr	2,84	Pank1	0,78	Creb3l3	2,92
Serinc3	1,56	Zfp316	2,83	Acacb	0,77	Trim69	2,91
Capn13	1,56	Cyth3	2,83	Trip6	0,77	Olfr420	2,91
Git1	1,56	Wdr86	2,83	Loxl3	0,77	Cks1brt	2,91
Irf2bp2	1,56	Krtap19-5	2,83	Vmn1r203	0,77	AF529169	2,91
Neil1	1,56	LOC100862015	2,83	Fbxw22	0,77	Fhit	2,91
Stk35	1,56	Olfr692	2,82	Pon1	0,77	Il10ra	2,90
Olfr204	1,56	Bik	2,82	Olfr175-ps1	0,77	4932411N23Rik	2,90
Zbtb5	1,56	Ppfia1	2,82	Prkce	0,77	Zfp62	2,90
1700001P01Rik	1,56	Gng8	2,82	Plekhg2	0,77	Ulk1	2,89
Serpinb3d	1,56	Prr18	2,82	Slc35a1	0,77	Barx2	2,89
Pramef6	1,56	Gpr1	2,81	Olfr1032	0,77	Abcc3	2,89
Hapln4	1,56	9030617O03Rik	2,81	Adrbk1	0,77	R3hcc1	2,88
Ccdc85a	1,56	Phyhip	2,81	Exoc6	0,77	Ly6g6f	2,88
Kdr	1,56	Spaca5	2,80	Csnk1d	0,77	Slc35f3	2,87
Stpg2	1,56	Usp14	2,80	Ipmk	0,77	Gstt4	2,87
Prpf6	1,56	Vgll3	2,80	Defb5	0,77	Esp38	2,87
Gm10100	1,56	Dpp7	2,79	Serpina3b	0,77	Prb1	2,86
Mum1l1	1,56	Plcl2	2,79	Irf1	0,77	Hsdl1	2,86
Atg3	1,55	Aqp8	2,79	Mfsd2a	0,77	Sprr2a2	2,86
Gpc4	1,55	Dnah2	2,79	Olfr1298	0,77	Cldn4	2,86
Gm11992	1,55	C1qtnf7	2,78	Apoh	0,76	Rnf219	2,85
Ak1	1,55	Gja3	2,78	Obsl1	0,76	Trmu	2,85
Cdh7	1,55	Kirrel3	2,78	Olfr1217	0,76	Jam3	2,85
Esd	1,55	Prss55	2,78	Ctbs	0,76	Rev1	2,84
Tomm40l	1,55	Ypel1	2,78	Hspa2	0,76	Nmu	2,84
Hspa1a	1,55	Lect2	2,78	Ndufs4	0,76	Scin	2,84
Pvrl4	1,55	Clec7a	2,78	Slc39a4	0,76	Zfp112	2,84
Smyd5	1,55	Nek9	2,78	Snph	0,76	Jakmip1	2,84
Mrpl51	1,55	Cab39	2,77	Fzd7	0,76	Olfr307	2,84
Olfr1085	1,55	Tnrc6a	2,77	Defb34	0,76	2310057N15Rik	2,84
Vmn2r91	1,55	Pigp	2,77	Vmn1r101	0,76	Cdca4	2,83
Vmn1r206	1,55	Smim6	2,77	Hpse2	0,76	Nipal2	2,83
Noto	1,55	Impa1	2,77	Pip5k1c	0,76	Itpkb	2,83
Itfg2	1,54	Tmem56	2,77	Olfr639	0,76	Sv2c	2,83
Krtap9-3	1,54	Xlr5a	2,77	Cryab	0,76	Dusp21	2,83
Hmg20a	1,54	Kcnj14	2,77	Zan	0,75	Asap1	2,83
Flot2	1,54	Tslp	2,76	Pip4k2b	0,75	Mospd2	2,83
Abhd16b	1,54	Chuk	2,76	Mafa	0,75	N4bp2	2,83
Myh10	1,54	Ap2b1	2,76	Pramef17	0,75	Sucnr1	2,83
Smagp	1,54	Atp2a3	2,76	Olfr727	0,75	Krtap5-4	2,83
Dnase1l2	1,54	Olfr142	2,75	Bglap2	0,75	Nipal1	2,82
Chac1	1,54	Paqr6	2,75	Arfrp1	0,75	Olfr798	2,81
Ermap	1,53	Herpud1	2,73	6030498E09Rik	0,75	Ssr1	2,81
3110082I17Rik	1,53	Rnls	2,73	Vmn2r52	0,75	Eri3	2,80
Pspc1	1,53	Apoc2	2,73	Olfr1416	0,75	Trat1	2,79
Smad2	1,53	Vwa2	2,73	Ckap2	0,75	Olfr298	2,79
Morc2a	1,53	Mpp5	2,72	Fbxl2	0,75	Mrc1	2,78

Ms4a4c	1,53
Cotl1	1,53
Fam193a	1,53
Slc38a2	1,53
Nde1	1,53
Msn	1,53
Phkg2	1,53
Il17f	1,53
Eng	1,53
Plec	1,53
Prss40	1,53
Calca	1,53
Map3k8	1,52
Mdga2	1,52
Tomt	1,52
Rpl7a	1,52
1700007G11Rik	1,52
Rhoc	1,52
Vmn1r202	1,52
Arid1b	1,52
Pth2r	1,52
Nsmf	1,52
Oscar	1,52
Rtn4rl1	1,52
Zfp185	1,52
Phpt1	1,52
Leprotl1	1,52
Gm5591	1,52
Ylpm1	1,51
Nupr1l	1,51
Ucp2	1,51
Gm1564	1,51
Sf1	1,51
Agpat9	1,51
Reg3d	1,51
Zfp428	1,51
Lhx8	1,51
Gykl1	1,51
Tmem26	1,51
Inhbb	1,51
Fbxl5	1,51
Chp2	1,51
Stoml1	1,51
Zscan12	1,50
Clec1b	1,50
Cst9	1,50
Npas3	1,50
BRDN0000737643	1,50
Tcf20	1,50
Cxx1c	1,50
Gnb3	1,50
Foxp1	1,50
Prrg4	1,50
Mbd3l2	1,50
AI118078	1,50
Cwh43	1,50
Pianp	1,50
Lrrc58	1,50
Vps37b	1,50
Agfg2	1,50
Tm9sf2	1,50
Faim	1,49
Ltf	1,49
Xlr3c	1,49
Olfr1279	1,49
Psmd9	1,49
BC017158	1,49
Olfr1129	1,49
Myl12a	1,49
Huwe1	1,49
Cd163l1	1,49
Pdia2	1,49
Tspan6	1,49
Mzf1	1,49
Treml2	1,49
Tmem28	1,49
Cd300lg	1,49
Exosc4	1,49
Ppp1r42	1,49
Actl6b	1,48
Gm765	1,48
Rimklb	1,48

Flnc	2,71
Gm15085	2,71
Akap11	2,71
Gna12	2,71
Cep170b	2,71
6820408C15Rik	2,71
Efnb1	2,71
Olfr434	2,70
Tctex1d4	2,70
Adcy6	2,69
Got1	2,68
Tmem256	2,68
Dnajb12	2,68
Tom1l2	2,68
Dab2ip	2,67
Mettl9	2,67
Scube2	2,67
Scube3	2,67
Jade1	2,67
Olfr1179	2,67
Fcf1	2,66
Cyb5r3	2,66
Dchs1	2,66
Cops4	2,65
Txndc15	2,65
Phc1	2,65
Thnsl2	2,64
Taar7d	2,63
Tor1a	2,63
4930595M18Rik	2,63
Ppp6r3	2,63
Gif	2,62
She	2,62
Cdcp2	2,62
Colgalt1	2,62
Hsd17b12	2,62
Kcnq5	2,62
Sp1	2,62
Rbpms	2,61
Armcx1	2,60
Ttc30a1	2,60
Pim1	2,60
Spred3	2,59
Clec4a3	2,58
Clcn5	2,57
Aldoc	2,57
Mtfr2	2,57
Cyc1	2,57
Nrip1	2,56
Naa38	2,56
Olfr1500	2,56
Sdr39u1	2,55
Snx29	2,54
Rassf5	2,54
Hsd3b3	2,54
Hist1h4n	2,54
Nup35	2,54
Hoxd8	2,54
Gdf11	2,54
Lipa	2,53
Cnksr2	2,52
Mesp2	2,52
Lrrc2	2,52
Cxx1a	2,52
Ccr2	2,51
Efna3	2,51
Pstpip1	2,51
Scgb1b20	2,49
Cdh24	2,49
Kcna3	2,49
Parm1	2,49
Ppp1r3e	2,49
Tbx18	2,49
H1foo	2,48
Slc22a27	2,48
Gde1	2,48
Gprin3	2,48
Pask	2,48
Adam29	2,47
Samhd1	2,46
Anxa2	2,46
Tmem42	2,45

Tlcd1	0,75
Txlng	0,74
Asb7	0,74
Tnfrsf21	0,74
Slco6d1	0,74
Slc37a1	0,74
Serpina3n	0,74
Olfr414	0,74
Olfr1320	0,74
Minpp1	0,74
Copz2	0,74
Pcdha1	0,73
Olfr1408	0,73
Cryzl1	0,73
Sipa1l3	0,73
Socs6	0,73
Crbn	0,73
Olfr1392	0,73
Wdr95	0,73
Rhot2	0,73
Tmem161a	0,73
Zfpl1	0,73
Plxnb3	0,73
Scnn1b	0,73
Slmo2	0,73
Gm10352	0,73
Elf5	0,73
Vmn1r129	0,73
Rit2	0,73
Adam3	0,73
Gpi1	0,73
Rhbdl2	0,73
Tnfrsf10b	0,72
Cables1	0,72
Naa25	0,72
BRDN0000737610	0,72
Olfr982	0,72
Fam35a	0,72
Stim1	0,72
Cnpy2	0,72
Etfb	0,72
Slc17a5	0,72
Ofd1	0,72
Gen1	0,72
Tra2b	0,72
Olfr576	0,72
Zfp189	0,72
Vmn1r115	0,72
Npvf	0,72
Lyrm4	0,72
Nlrp9b	0,72
Nkap	0,72
Olfr792	0,72
Onecut1	0,72
Nfe2l3	0,72
Cdk12	0,72
Zfp445	0,72
Cldn13	0,71
Arhgap27	0,71
Csrp1	0,71
Ddx50	0,71
Adamts14	0,71
Olfr114	0,71
Trem1	0,71
Alg14	0,71
Mug1	0,71
Olfr410	0,71
Vhl	0,71
Bmi1	0,71
Mageb18	0,71
Cers6	0,71
Dhh	0,71
Ms4a4d	0,71
Mark2	0,71
Clcn4-2	0,71
Olfr1015	0,71
Tmem252	0,70
Rft1	0,70
Slc39a14	0,70
Krtap21-1	0,70
Gsto2	0,70
Zranb2	0,70

Bmf	2,78
Eif4enif1	2,77
H6pd	2,77
Sytl2	2,77
Slc13a3	2,77
Tmem176b	2,77
Mto1	2,76
Bbs4	2,76
Lrrc8e	2,76
Ndufaf5	2,76
Olfr812	2,76
Vmn1r113	2,75
Ndufv2	2,75
Rasa3	2,75
Cldn14	2,75
Ccdc126	2,75
Dbn1	2,75
Adora3	2,75
Teddm2	2,75
Cecr6	2,75
Rdh5	2,75
Atp8b4	2,74
Pqlc3	2,74
Bmp5	2,74
Dcbld1	2,74
Plbd2	2,74
Tsx	2,73
Zik1	2,72
Fbxl2	2,72
Tox4	2,72
Ctps2	2,72
Olfr1281	2,72
Zkscan16	2,72
Nobox	2,71
Pot1b	2,71
Rbak	2,70
Usp21	2,70
Prkar2b	2,70
Olfr535	2,70
Adam24	2,70
Wdr81	2,70
Tmsb4x	2,70
Tdrd3	2,70
Rarres1	2,69
Aicda	2,69
Fam43b	2,69
Zfp474	2,69
Padi6	2,68
Tes	2,68
Nkx2-3	2,68
Mpp7	2,68
Dagla	2,67
Prl	2,67
Gpr75	2,67
Def6	2,67
Extl1	2,67
Sbds	2,67
Atp5g1	2,67
Il27ra	2,66
Bsx	2,66
Nln	2,65
Nlrp1b	2,65
Lce1a2	2,64
Olfr1382	2,64
Pafah1b3	2,64
Apoc3	2,63
Gltscr1l	2,63
Dlx2	2,63
Snx8	2,62
Shox2	2,62
Tmem256	2,62
Nsdhl	2,61
Ogfod3	2,61
Spag11b	2,61
Amotl2	2,61
Tmem233	2,60
Gzma	2,60
Oas1g	2,60
Sesn1	2,59
Gm5741	2,59
4931406P16Rik	2,59
Olfr768	2,58

Slc25a5	1,48	Gm13152	2,45	Sohlh2	0,70	Agap1	2,58
Clk3	1,48	Vax1	2,44	Ephx4	0,70	Acot7	2,58
Galnt14	1,48	Tbl3	2,44	Try4	0,70	Prr23a	2,58
Gstm2	1,48	Capns1	2,44	Enoph1	0,70	Cpa1	2,57
Efcab6	1,48	Gata6	2,43	Actl7b	0,70	Gm766	2,57
Hmgcr	1,48	Olfr969	2,43	Mrpl28	0,70	Skor2	2,57
Gabpb2	1,48	Gfra3	2,42	Sema5a	0,70	D430019H16Rik	2,56
BRDN0000738276	1,48	S100a8	2,42	Hist1h1e	0,70	Itgb5	2,55
Cib1	1,48	Trim56	2,42	Dlgap2	0,70	4930447C04Rik	2,55
Hsd17b2	1,48	Ephb1	2,41	Pramel1	0,69	Olfr397	2,55
Atp8b2	1,48	Txn2	2,41	Babam1	0,69	Olfr576	2,54
Polr2l	1,48	Lgals8	2,41	Lpo	0,69	Olfr1412	2,54
2310007B03Rik	1,48	Zfp616	2,41	Zswim4	0,69	Txndc16	2,54
Tlcd2	1,48	2310009B15Rik	2,41	Olfr493	0,69	Ubqln2	2,53
C7	1,48	Calb2	2,39	Camk2d	0,69	Cacng1	2,53
Mapre3	1,47	Micall1	2,38	Myrip	0,69	3110043O21Rik	2,53
H2-M10.2	1,47	Spop	2,37	Vmn1r137	0,69	4930502E18Rik	2,52
Ndufa5	1,47	Vmn2r2	2,36	Pcolce2	0,69	Grm4	2,52
1700015E13Rik	1,47	Gm10665	2,35	Hspa13	0,69	Dusp7	2,52
Slc5a5	1,47	Vmn1r210	2,35	Nek3	0,69	Aoc3	2,52
Trcg1	1,47	Zeb1	2,34	Cdo1	0,69	Higd2a	2,52
Nr2c2ap	1,47	Olfr589	2,34	Fstl5	0,69	Krt71	2,52
Glcci1	1,47	Hdc	2,33	Fbxo38	0,69	Susd3	2,52
Ttll6	1,47	Lipf	2,33	Szt2	0,69	Plk5	2,51
BRDN0000738358	1,47	Znrf3	2,33	Zfp275	0,69	Abcc6	2,51
Kcnd3	1,47	Npffr2	2,32	Swt1	0,69	Bche	2,51
Mstn	1,47	Gm14458	2,32	Lhpp	0,69	Dcaf12	2,51
Nfx1	1,47	Nrtn	2,32	Arhgap22	0,69	Kiss1r	2,50
Fam117b	1,47	Xaf1	2,32	BRDN0000737541	0,69	Qtrt1	2,50
Ssna1	1,46	Oxct2b	2,32	Vmn1r167	0,69	Alox5ap	2,50
Ass1	1,46	Zdhhc24	2,32	Ifna6	0,69	Qsox1	2,49
Zmym3	1,46	Gzmm	2,31	1700011L22Rik	0,69	Cdkn2b	2,49
Nkx1-2	1,46	Fam188b	2,31	Srp68	0,68	Zcchc2	2,48
Rap2b	1,46	March9	2,31	Nrtn	0,68	Hgh1	2,48
Tmem89	1,46	Sash1	2,30	Igfn1	0,68	Ppp1r14a	2,48
Elovl1	1,46	Olfr1382	2,29	Gm4847	0,68	Plet1	2,48
Hook2	1,46	Pla1a	2,28	Pcmtd2	0,68	Tarsl2	2,48
B230219D22Rik	1,46	Shf	2,28	Slc5a3	0,68	Tsen54	2,47
Dcstamp	1,46	Islr	2,28	4933409G03Rik	0,68	Timp4	2,47
Tmem200b	1,46	Zfp277	2,27	Nudt7	0,68	Syt12	2,47
Rpap1	1,46	Sh3pxd2b	2,27	1110012L19Rik	0,68	Nfil3	2,47
Pts	1,46	Arntl	2,26	Olfr124	0,68	Ptpre	2,47
Gar1	1,46	Limd2	2,25	Fbln1	0,68	Matn4	2,47
Lag3	1,46	3110035E14Rik	2,24	Hsf4	0,68	Hddc2	2,46
Lrrc72	1,45	Dynlt1b	2,24	Olfr453	0,68	Tmem52	2,45
Camsap2	1,45	Gm5114	2,24	N4bp1	0,68	Rora	2,45
Sp5	1,45	Mpl	2,23	P4hb	0,68	Iqcf3	2,45
Vps37c	1,45	Clca4a	2,22	Ubr7	0,68	Tigd2	2,45
Cbx4	1,45	Olfr1346	2,22	Grhl2	0,68	Hmx1	2,44
1110007C09Rik	1,45	Morc2b	2,21	Mroh1	0,68	Olfr1086	2,44
Dapk1	1,45	Nalcn	2,20	Tmem140	0,68	Map1a	2,42
Dfnb59	1,45	Yme1l1	2,20	BRDN0000738086	0,68	Chuk	2,41
Vtn	1,45	Usp50	2,19	Cpb1	0,67	Gpr119	2,40
Cmtm8	1,45	Zbtb22	2,19	Wnk2	0,67	Chrnb4	2,40
Btrc	1,45	Spryd4	2,15	Bag4	0,67	Tuba3a	2,40
Arhgef2	1,45	Zbtb16	2,09	Dnajc11	0,67	Cped1	2,39
Atoh8	1,45	Ly6c2	2,00	Ammecr1l	0,67	2610524H06Rik	2,39
Efcab12	1,45	Ndufaf7	1,90	Prl3d1	0,67	Uchl1	2,38
Ccdc42	1,45	Cst11	1,90	Fpr-rs3	0,67	Cav3	2,38
Bhlhb9	1,45	Pnpla7	1,90	Rem1	0,67	Sccpdh	2,38
Baiap2l2	1,44	Cables2	1,86	Kank2	0,67	Slc41a2	2,38
Jagn1	1,44	Gpr176	1,83	Uhmk1	0,67	Zswim2	2,37
Olfr868	1,44	Svopl	1,83	Slc17a1	0,67	AY074887	2,37
3632451O06Rik	1,44	Olfr1002	1,79	Ticam1	0,67	1700019D03Rik	2,37
Nmur2	1,44	Olfr1241	1,78	Grip1	0,67	Lpcat4	2,36
Atad2b	1,44	Tmem128	1,76	Ufsp1	0,67	Porcn	2,35
Tmed6	1,44	2310022B05Rik	1,75	Ly6g6e	0,67	Hipk1	2,35
Prkcsh	1,44	Kif24	1,73	Dtymk	0,67	Nupr1l	2,33
Olfr1209	1,44	C1rb	1,73	Psmb3	0,67	Shisa2	2,30
Sv2c	1,44	Fstl4	1,71	Olfr963	0,67	Hlcs	2,30
Ccser1	1,44	Rad51	1,71	Olfr39	0,67	Fut10	2,29
Gpx7	1,44	Foxred1	1,70	Actn3	0,67	Olfr418	2,28
4932411N23Rik	1,44	Card6	1,69	Dsn1	0,67	Awat2	2,27
Cfap74	1,44	Blk	1,69	Palm2	0,66	Olfr1242	2,26
Mycs	1,43	Ptpn9	1,69	Plgrkt	0,66	Gm11744	2,26
Hsph1	1,43	Wdr34	1,69	Dus2	0,66	Olfr1346	2,25
Mios	1,43	Man2a1	1,69	Rbm12b2	0,66	Clvs2	2,25
Stx11	1,43	Ago3	1,69	Olr1	0,66	Scn9a	2,23
Pon1	1,43	Ndst1	1,69	Pglyrp3	0,66	Dnd1	2,23
E330014E10Rik	1,43	Stab2	1,68	Rpp25	0,66	Tmem167b	2,22
Prr13	1,43	Zfp790	1,67	Olfr474	0,66	Unc45b	2,21
Igsf23	1,43	Mmp9	1,67	Il20ra	0,66	Tmem127	2,20

Klk13	1,43	Acads	1,67	Rab3gap2	0,66	Slc17a3	2,20
Mgp	1,43	Slc35a1	1,66	Gp1ba	0,66	Ubxn10	2,19
Gstz1	1,43	Nek8	1,66	Akr1c18	0,66	Slc25a29	2,17
2010107E04Rik	1,43	Cd300lh	1,66	Scgb1b3	0,66	Ly6h	2,17
Asgr1	1,43	Tada2a	1,66	Ptpn11	0,66	Gm9	2,16
Cldn2	1,43	Calml3	1,66	Atg2a	0,66	Sfrp2	2,16
Oxct2b	1,43	Ugt2b5	1,65	Gypa	0,66	Rmdn3	2,15
Fam134b	1,43	Krt33a	1,65	Amhr2	0,66	Upk1b	2,14
Tmem151a	1,43	Smek2	1,65	Myl12a	0,66	B4galt2	2,13
Slc9a1	1,43	Slc35f2	1,64	Gm362	0,66	Nr2c2	2,13
Lipm	1,43	Ddx56	1,64	Prorsd1	0,65	Rab26	2,12
Mien1	1,43	Enthd2	1,64	Reg3b	0,65	BC051665	2,11
Rhox11	1,42	Kif2b	1,63	Gpr25	0,65	Ndufa8	2,10
Plekhf2	1,42	Krtap27-1	1,63	Ptpn7	0,65	Tnfrsf9	2,08
Serpina9	1,42	Alb	1,62	Gm13084	0,65	AI467606	2,07
Vmn1r37	1,42	Artn	1,61	Zfp934	0,65	Fam177a	2,07
Plxnd1	1,42	Slc10a3	1,61	Tlx3	0,65	Olfr824	2,07
Kctd12	1,42	Gdf7	1,61	Ilf3	0,65	Stk24	2,01
Wtap	1,42	Cd274	1,60	Gm3750	0,65	4933402N22Rik	2,01
Dll3	1,42	Arrdc1	1,60	Adap2	0,65	Eif2ak2	2,01
Nsl1	1,42	Baiap2	1,60	Cox20	0,65	Olfr821	1,99
Rnaset2b	1,42	Nsun6	1,59	Pdhx	0,65	Tsga10	1,94
Kank1	1,42	Ccr6	1,59	Sgol2b	0,65	Pafah1b2	1,91
Cox6a2	1,42	Lpxn	1,59	Yes1	0,65	Rwdd2a	1,84
Echdc3	1,42	Gtf2a1l	1,59	Parp1	0,65	Olfr1022	1,78
Impdh1	1,42	Ppp4r4	1,59	Vmn2r5	0,65	Itgb2l	1,77
Adcy4	1,42	Acot13	1,58	Prss12	0,65	Slc25a13	1,73
Fbxw18	1,42	Olfr726	1,58	Gm4303	0,65	Grik5	1,72
Wdr53	1,42	C330018D20Rik	1,58	Vmn1r21	0,65	Meaf6	1,71
Tgfbr3l	1,42	Tktl1	1,58	Mettl14	0,65	Ywhah	1,71
Slc30a7	1,42	8030462N17Rik	1,57	Pik3r1	0,65	Clhc1	1,70
Gdf7	1,42	Klhl15	1,56	Krtap19-9b	0,65	Fam20c	1,70
Fxyd6	1,42	Pvrl3	1,56	Gramd1b	0,65	Slc30a8	1,68
Tmem184b	1,42	Zbtb38	1,56	BRDN0000737559	0,65	Rsl1	1,67
St6galnac4	1,42	Jrk	1,56	Dhx36	0,65	Gm1110	1,66
Gmpr2	1,42	Pbx3	1,56	Baat	0,65	Gm3500	1,66
Nlrp9c	1,42	Fbxw21	1,56	Ldb2	0,65	Mtdh	1,65
Rpe65	1,41	Asic4	1,55	Tmem74	0,65	Tnfaip1	1,64
Olfr958	1,41	Rps17	1,55	Slc5a11	0,64	Cts3	1,64
Asb12	1,41	Vmn2r33	1,55	Tmem199	0,64	Fmo5	1,62
Gnb4	1,41	Wbp11	1,55	Kirrel3	0,64	Zbed6	1,61
B4galt3	1,41	Sema6b	1,55	Mfsd6	0,64	Chek1	1,61
Aunip	1,41	Zswim3	1,55	Krt77	0,64	Rai2	1,60
Npepl1	1,41	Olfr1240	1,54	Tmem150c	0,64	Bank1	1,60
Pou2f2	1,41	Cyp3a57	1,54	Tmc2	0,64	Slc11a1	1,59
Stk32a	1,41	Icam2	1,53	Slc46a2	0,64	Gm1123	1,59
Prss41	1,41	Olfr305	1,53	Olfr460	0,64	Nfe2l1	1,58
Reg2	1,41	BRDN0000737930	1,53	Creb1	0,64	Sox8	1,58
Tspyl1	1,41	Ddx26b	1,53	A630095N17Rik	0,64	Morc2b	1,58
Hdgfl1	1,41	Ttc38	1,53	2410127L17Rik	0,64	Slc6a9	1,58
Dck	1,41	Cald1	1,52	Sub1	0,64	A530099J19Rik	1,57
Glrx3	1,41	Trim37	1,52	Itfg3	0,64	Ctcfl	1,57
Olfr366	1,41	Rexo4	1,52	Kif3c	0,64	Gas2l2	1,57
Flii	1,41	Bco1	1,52	Mcf2	0,64	Reg4	1,57
Gm13272	1,40	Dcp1a	1,52	Serpinf2	0,64	Zfp61	1,57
Wibg	1,40	Olfr338	1,51	Ube2b	0,64	Mrpl33	1,57
Ccdc81	1,40	Gm14351	1,51	Gm904	0,64	Zfp750	1,56
Ankrd10	1,40	Snx7	1,51	Svep1	0,64	Dhrs11	1,56
Trim62	1,40	Epha8	1,51	Irgm2	0,64	Poldip3	1,55
Rfpl3s	1,40	Tubgcp2	1,51	Cfap69	0,64	Amy1	1,55
Bmp5	1,40	Dnaja1	1,51	Brinp3	0,64	Meox2	1,55
Kansl1l	1,40	P4ha2	1,51	St8sia1	0,64	Pabpn1l	1,55
Prss56	1,40	Tmem107	1,51	Ghdc	0,64	Fam118a	1,54
Ptgfr	1,40	Ap5z1	1,51	Trim60	0,63	Dsn1	1,54
Vmn1r178	1,40	Zcchc9	1,50	Igfbp1	0,63	Pcyox1l	1,54
Snn	1,40	D430041D05Rik	1,50	Olfr1251	0,63	Amer2	1,54
Ins2	1,40	Rnf17	1,50	Dhx38	0,63	Sult2a1	1,53
1700003E16Rik	1,40	Ly6c1	1,50	Tgs1	0,63	AU019823	1,53
Nxpe5	1,40	Tgfb1i1	1,50	Glycam1	0,63	Igfl3	1,53
Bank1	1,40	Gbp10	1,49	Cpa3	0,63	Sprr2h	1,52
Serpina1b	1,40	Erlin2	1,49	Slc25a45	0,63	BRDN0000737621	1,52
Zkscan14	1,40	Ces4a	1,49	Ccdc33	0,63	Npy	1,52
Zfp868	1,39	Ttc25	1,49	Clpb	0,63	Elovl3	1,52
Tecrl	1,39	Tmem191c	1,49	Mterf1b	0,63	Ncstn	1,51
Prl7a2	1,39	4930506M07Rik	1,49	Cpsf4l	0,63	Htra2	1,51
Glce	1,39	Gstm3	1,49	Ttll9	0,63	Slc25a38	1,50
Catsper4	1,39	Ube2s	1,49	Gtf2f2	0,63	Olfm1	1,49
Cntn2	1,39	Olfr137	1,49	Gm6432	0,63	Olfr543	1,49
Fbxo30	1,39	Six6	1,49	Mrpl9	0,63	Pbrm1	1,49
Rbbp8	1,39	T	1,48	Ints10	0,63	Vav3	1,49
Ldlrad4	1,39	2300009A05Rik	1,48	Adcy8	0,63	Pax6	1,49
Prnd	1,39	Upb1	1,47	Zc3h7a	0,63	Ltf	1,49

Olfr171	1,39	Zbed6	1,47	Slc39a3	0,63	Gsk3a	1,49
Tmem9	1,39	Vmn1r218	1,47	Btbd7	0,63	Nts	1,49
Itprip	1,39	Mphosph8	1,47	Pdk1	0,63	Pcdhac2	1,49
Idh1	1,39	Olfr1155	1,47	Rnaseh2b	0,63	Epx	1,49
Olfr665	1,39	Snrpn	1,46	Synj2bp	0,63	Bcl7c	1,48
Fam53c	1,39	Olfr910	1,46	Klc2	0,63	Madcam1	1,48
Olfr677	1,39	Alx4	1,46	Arf6	0,63	Shb	1,48
Maml1	1,39	Ckap4	1,46	Cnot11	0,63	Flcn	1,48
Jazf1	1,39	Tgm3	1,46	Stap1	0,62	Mettl16	1,48
Nlgn1	1,39	Scnn1b	1,45	Dlgap5	0,62	S100a16	1,48
Stard4	1,39	Zfp960	1,45	Dmkn	0,62	Pgp	1,48
1700018C11Rik	1,39	Phgdh	1,45	Rnpepl1	0,62	Atp6ap1	1,48
Med16	1,39	Hist2h2bb	1,45	Arl13b	0,62	March9	1,48
4931428F04Rik	1,39	Acacb	1,45	Psmb11	0,62	Olfr1475	1,47
Slc46a1	1,38	Melk	1,45	Akr1b10	0,62	Smim7	1,47
Pdxdc1	1,38	Erv3	1,45	Hoxa4	0,62	Snrpb2	1,47
Lyrm5	1,38	4930558K02Rik	1,45	Nfia	0,62	Mblac1	1,47
Ube2k	1,38	Pxdn	1,44	Enpep	0,62	Olfr1321	1,47
Sntn	1,38	Stard7	1,44	Cyb561a3	0,62	Dscr3	1,47
2610015P09Rik	1,38	P4ha3	1,44	Trim13	0,62	Olfr553	1,47
Islr2	1,38	Fgf12	1,44	Pcdhb13	0,62	Nupr1	1,46
Mpp7	1,38	Metrn	1,44	Ptdss1	0,62	Olfr726	1,46
Syn1	1,38	Itfg3	1,44	Pick1	0,62	Olfr143	1,46
R3hcc1l	1,38	Vav3	1,44	Enpp2	0,62	Tigit	1,46
Lalba	1,38	Dpysl2	1,44	Xpc	0,62	Foxf2	1,46
Dmxl1	1,38	Cacnb1	1,43	Mon2	0,62	Ido1	1,46
BRDN0000738157	1,38	Foxb1	1,43	1700066B19Rik	0,62	Bcar3	1,46
Acaa1a	1,38	Hes5	1,43	Klri1	0,62	Fam65b	1,45
Ppid	1,38	Hoxd1	1,43	Gm6592	0,62	Tollip	1,45
Mrgprx1	1,38	Btbd8	1,43	Tmem92	0,62	Fam71e2	1,45
Mapre1	1,38	Plekhh1	1,43	Rad54l2	0,62	Serpina11	1,45
Cyp4f13	1,38	Clk3	1,43	Nfic	0,62	Dzank1	1,45
Inhbc	1,38	Micu1	1,43	Rhox3h	0,61	Mcu	1,45
Chpf	1,38	Fbxw11	1,43	Chad	0,61	Tex28	1,45
Clip1	1,38	Wdr37	1,42	Cfhr1	0,61	Scaf11	1,44
Actbl2	1,38	Tlr12	1,42	Olfr553	0,61	Avpr1b	1,44
Abhd3	1,38	Kras	1,42	Ooep	0,61	Serpina12	1,44
Ptrf	1,38	1700088E04Rik	1,42	Aurka	0,61	Ncoa7	1,44
Dhx38	1,37	Eci2	1,42	Hic1	0,61	Rgs11	1,44
Lrrn4	1,37	Slc25a34	1,42	Gopc	0,61	Gm2933	1,44
Skint8	1,37	Bcl2l11	1,42	Tas2r122	0,61	Cuedc2	1,44
Olfr1495	1,37	Cnih3	1,42	Csrp3	0,61	Zfp507	1,43
Tmem150b	1,37	Gm13178	1,42	Slc25a13	0,61	Ucn	1,43
Adgra2	1,37	Gpr139	1,41	Trpc5	0,61	Scn8a	1,43
Fbxo24	1,37	Olfr172	1,41	Lhx4	0,61	Pde5a	1,43
Klhl25	1,37	Ifi205	1,41	Cldn16	0,61	Gnb5	1,43
Gfap	1,37	Mthfd2l	1,41	Gja1	0,61	Ccne2	1,43
Cldn1	1,37	F13b	1,41	Ly86	0,61	Fstl4	1,43
Olfr1307	1,37	Mzt1	1,41	Rbfox1	0,61	Ambp	1,43
Arid2	1,37	Tas2r123	1,40	Rhog	0,61	Trcg1	1,43
Serpinb6e	1,37	Olfr629	1,40	Rasd2	0,61	Nkiras2	1,42
Foxa1	1,37	Adamts12	1,40	Ints6	0,61	Tex43	1,42
Tenm1	1,36	Olfr1495	1,40	Ndn	0,61	Epb4.1l1	1,42
Syt8	1,36	Fhod3	1,40	Spon1	0,61	Setd1b	1,42
Ms4a6c	1,36	Sept14	1,39	Kbtbd4	0,61	Aqp3	1,41
Snx4	1,36	Fbxo4	1,39	Kti12	0,61	Sec14l2	1,41
Olfr437	1,36	Ifitm1	1,39	Pcdha9	0,60	Foxi3	1,41
Olfr1197	1,36	Xbp1	1,39	Vmn1r52	0,60	Zfp11	1,41
Rxra	1,36	Pcdhb10	1,39	Mrpl50	0,60	Fyb	1,41
Olfr1062	1,36	Mmp14	1,39	Pih1d2	0,60	Ptar1	1,41
Cdr1	1,36	Olfr1202	1,39	Abcf3	0,60	Psg27	1,41
Pde11a	1,36	Gxylt2	1,39	Kctd12	0,60	BRDN0000737858	1,40
Figla	1,36	Slc5a8	1,39	Acot2	0,60	D2Wsu81e	1,40
Slc12a5	1,36	Prrg3	1,39	Gbgt1	0,60	Asf1a	1,40
Slc36a2	1,36	Myd88	1,39	Calb1	0,60	Map4k4	1,40
Syt11	1,36	Lmbrd1	1,39	Rad51b	0,60	Elk4	1,40
Cyp39a1	1,36	BC031181	1,38	Lrrtm2	0,60	Pelp1	1,39
Umps	1,36	Gm7030	1,38	4430402I18Rik	0,60	Aox2	1,39
Olfr1472	1,36	Fam65b	1,38	Mpp5	0,60	Tas2r143	1,39
Apex1	1,36	Stt3a	1,38	Unc93b1	0,60	Polr2a	1,39
Gm19345	1,36	Sval3	1,38	Il17d	0,60	Nr4a1	1,39
Olfr768	1,36	Pprc1	1,38	Sftpc	0,60	Gm6812	1,38
Gm11564	1,35	Foxc2	1,38	Nccrp1	0,60	Vwa7	1,38
Slc29a4	1,35	Cdnf	1,38	R3hdm4	0,60	Kcng2	1,38
Epb4.1l1	1,35	Ptgfr	1,38	Lhfp	0,60	Pbx2	1,38
Nuak2	1,35	Slc38a4	1,38	Rasal3	0,60	BRDN0000737466	1,38
Slc9b2	1,35	2210407C18Rik	1,38	Pex11b	0,60	C1qtnf1	1,38
1500015O10Rik	1,35	Gm14511	1,37	Vpreb3	0,60	Lkaaear1	1,38
Nlrp4b	1,35	Creld1	1,37	Bank1	0,60	Rgs7bp	1,37
Il21	1,35	Vmn1r3	1,37	Arhgap18	0,59	Esp3	1,37
Rrad	1,35	Cyp4f39	1,37	Immp1l	0,59	Mpz	1,37
Pkn2	1,35	Dusp16	1,37	Usp19	0,59	Ndufc1	1,37

Arntl2	1,35	Pcdhb5	1,37	Sfrp2	0,59	Sec23a	1,37
Gm7030	1,35	Olfr1006	1,37	Slc35a3	0,59	Id4	1,37
Col9a1	1,35	Slc35a2	1,37	Olfr518	0,59	Dpy30	1,37
Slit2	1,35	Ndufa9	1,37	Fgl1	0,59	Slc25a15	1,37
Mettl21b	1,35	Dlk1	1,37	Matn2	0,59	Ntn1	1,37
Cyp27a1	1,35	Ccdc141	1,36	Ankrd40	0,59	A4galt	1,37
Gm6040	1,35	Greb1l	1,36	Tmprss13	0,59	Zscan4c	1,37
Ephb6	1,35	Tpd52l1	1,36	Eqtn	0,59	Gm6710	1,36
BRDN0000737803	1,35	Mgea5	1,36	Nbeal2	0,59	Cdk16	1,36
Map2k6	1,35	Olfr734	1,36	Parp16	0,59	Bod1	1,36
Srgap1	1,35	Smpd5	1,36	Olfr138	0,59	Rcbtb1	1,36
Haus2	1,35	BC117090	1,36	Nab1	0,59	Gpd1	1,36
Fhl5	1,35	Psmg1	1,36	Akap12	0,59	Klf14	1,36
Vmn1r87	1,34	Eif4enif1	1,36	Gng2	0,59	Bola1	1,36
Fgfr1	1,34	Nae1	1,35	Parp2	0,59	Them6	1,35
Chmp4c	1,34	Olfr1220	1,35	Opa1	0,59	Macrod1	1,35
Gnpda2	1,34	L1td1	1,35	D630039A03Rik	0,59	Vdac1	1,35
St13	1,34	4930524N10Rik	1,35	Mas1	0,59	1700008P02Rik	1,35
Vps13c	1,34	Otub1	1,35	Cmtm1	0,59	Gm5114	1,35
2210016L21Rik	1,34	Krtap16-1	1,35	Slc35b4	0,59	Sart1	1,35
Pld1	1,34	Clps	1,35	Ndst3	0,59	Serping1	1,35
Olfr1228	1,34	Bard1	1,35	St8sia3	0,59	Dnajc4	1,35
Pcdhb7	1,34	Plcxd3	1,35	Olfr1136	0,59	Cnot1	1,35
Atxn7l3b	1,34	Gm15292	1,35	BRDN0000738142	0,59	Nags	1,35
Car5b	1,34	Tln1	1,35	Arrdc2	0,58	Msantd1	1,35
Suclg1	1,34	Olfr1263	1,35	Rbm15	0,58	6430548M08Rik	1,35
Smarcc1	1,34	Klrb1c	1,35	Fcrlb	0,58	Cmtm2b	1,35
Mtif2	1,34	Prpf4	1,34	Kif16b	0,58	Dcx	1,35
Pou2f3	1,34	Ccdc8	1,34	Spink2	0,58	Slc37a2	1,35
Olfr1276	1,34	Egfl7	1,34	Sepn1	0,58	Upk3bl	1,35
Rnf157	1,34	Zfp202	1,34	Exog	0,58	Olfr1462	1,35
Apln	1,34	Aqr	1,34	Reep4	0,58	Map1lc3a	1,35
Nav2	1,34	Itprip	1,34	Ctage5	0,58	Uhmk1	1,35
Gdf15	1,34	Vmn2r77	1,34	Rnpep	0,58	Lmf2	1,34
Tspan31	1,34	Cenpm	1,34	Gabrr2	0,58	Stil	1,34
Efna2	1,34	Pum1	1,34	2810007J24Rik	0,58	Crxos	1,34
Cdk5r2	1,34	Rs1	1,34	Spdl1	0,58	Gtf2b	1,34
Gm13128	1,33	Bok	1,33	Tgfbr3l	0,58	Agbl4	1,34
Med8	1,33	Olfr1320	1,33	Zmat5	0,58	Pdzk1ip1	1,34
Srpk3	1,33	Thoc6	1,33	Dut	0,58	Pisd	1,34
Gm5878	1,33	Grk4	1,33	Klhl32	0,58	Rhbdd2	1,34
Erf	1,33	Tnfsf13	1,33	Scfd2	0,58	Prl8a1	1,33
Tmem233	1,33	Gldn	1,33	C86695	0,57	Lmnb1	1,33
Clec2i	1,33	3830406C13Rik	1,33	Dnm1l	0,57	Ccnh	1,33
Sema3b	1,33	Zbtb6	1,33	Pitpnm2	0,57	Mup1	1,33
Cypt14	1,33	Zfp442	1,33	Impg1	0,57	Tuft1	1,33
Dnajc24	1,33	Klhdc8b	1,33	Pde5a	0,57	Ppp1r42	1,33
Itsn2	1,33	1600012H06Rik	1,33	Ccdc55	0,57	Ikzf3	1,33
Zfp334	1,33	Bhlhe22	1,33	Olfr126	0,57	Anapc16	1,33
Smgc	1,33	Paip2b	1,32	Cyth2	0,57	Defa25	1,33
Rnf138	1,33	Cd200r1	1,32	Agbl5	0,57	0610009B22Rik	1,32
Sptlc2	1,33	Olfr849	1,32	Pdgfb	0,57	Hmgcl	1,32
Tmeff1	1,33	Cox4i2	1,32	Rel	0,57	Carm1	1,32
LOC100502896	1,33	Plcl1	1,32	Slc2a8	0,57	Erich2	1,32
Vmn1r39	1,33	Parvg	1,32	Gm12886	0,57	Arf3	1,32
Vmn1r94	1,33	Ncstn	1,32	Ptgdr2	0,57	Proc	1,32
Pds5a	1,33	Ccdc177	1,32	Phkb	0,57	Cnbd2	1,32
Pigv	1,33	Serpine3	1,32	1700019L03Rik	0,57	Pfkfb3	1,32
Arhgap21	1,33	Jph3	1,32	Rasa3	0,57	Siah2	1,32
Vmn1r210	1,32	Ttyh3	1,32	Tarm1	0,57	Sars2	1,32
Terf2	1,32	Cdc42ep2	1,32	Olfr11	0,57	Gm7903	1,32
Cnga4	1,32	Dao	1,32	Cdyl2	0,57	Ermn	1,31
Gopc	1,32	Steap4	1,32	Rgs11	0,57	Aatf	1,31
Cbfa2t3	1,32	Ndst4	1,32	Cga	0,57	H2-Ab1	1,31
Arhgap44	1,32	Serpinb9b	1,32	Tmem9b	0,57	Sntb2	1,31
Csnk1g2	1,32	Acvr2a	1,32	Tyk2	0,57	C1galt1	1,31
Nrxn3	1,32	Ptpn6	1,32	Cd200r2	0,57	Sppl2a	1,31
Aqp1	1,32	Slc6a1	1,32	Kiz	0,57	Ppp1r14b	1,31
Pnrc1	1,32	Ddx4	1,32	Cd37	0,57	Chd9	1,31
Lce6a	1,32	Porcn	1,32	Stk10	0,57	Ugt1a1	1,31
Prmt8	1,32	Mettl25	1,31	C1rl	0,57	Cd52	1,31
Larp6	1,32	Tmem8b	1,31	Arhgap1	0,57	Fopnl	1,30
Odf4	1,32	Exd2	1,31	Cdcp1	0,57	Luzp4	1,30
Cabyr	1,32	Smap2	1,31	Nprl3	0,57	Rnf5	1,30
Macrod2	1,32	Rfng	1,31	Numa1	0,57	Lair1	1,30
Ddah1	1,32	Idi2	1,31	Zfp882	0,56	Bloc1s6	1,30
Socs5	1,32	BC053393	1,31	Atp6v0a4	0,56	Fpr2	1,30
Pccb	1,32	Peli2	1,31	Itpk1	0,56	Slc22a16	1,30
Prss8	1,32	Zfp831	1,31	Adsl	0,56	Zfp383	1,30
Mamdc2	1,32	Ccser2	1,31	Rab33a	0,56	Krtap22-2	1,30
Rnf113a2	1,31	Sh3bgrl3	1,31	Coil	0,56	Rmi2	1,30
Zfp90	1,31	Cd19	1,31	Sgms2	0,56	BRDN0000737927	1,30

Snx33	1,31
Parp6	1,31
Ctsg	1,31
Olfr1290	1,31
Chad	1,31
Mtmr7	1,31
Fam221a	1,31
Otop3	1,31
Fpgs	1,31
Piezo1	1,31
Serpina3j	1,31
Ctsl	1,31
Kdm6b	1,31
Ranbp3	1,31
Vmn1r20	1,31
Hspb2	1,31
Sp6	1,31
Sec62	1,31
Idi2	1,31
Nr2e3	1,31
Psenen	1,31
Clcnka	1,31
Ctf2	1,31
Trem1	1,31
Utp6	1,30
Phtf1	1,30
Khdrbs2	1,30
Fabp9	1,30
Adck2	1,30
Ppp3r2	1,30
Pdzd2	1,30
Gm21693	1,30
Kdelr3	1,30
Cadm4	1,30
BRDN0000737488	1,30
Oxtr	1,30
Vmn1r138	1,30
Adgrv1	1,30
Ubqln2	1,30
Ufm1	1,30
Tmx3	1,30
Gm9047	1,30
Mrpl24	1,30
Rpp30	1,30
Nsmce2	1,30
4930579G24Rik	1,30
Cntn6	1,30
Mlip	1,30
Fndc3b	1,30
Olfr1444	1,30
Spdef	1,30
Ccdc28a	1,30
Cutal	1,30
Pgm2l1	1,30
Mepe	1,30
Ykt6	1,30
Prkx	1,29
Phactr1	1,29
Ctla2a	1,29
Ly6g6c	1,29
Hspa4	1,29
1700067K01Rik	1,29
Samd5	1,29
Ccdc71	1,29
Olfr921	1,29
Six2	1,29
Smpdl3a	1,29
Spns2	1,29
BRDN0000737727	1,29
Sorcs1	1,29
Cmtm5	1,29
Elovl2	1,29
Fam181a	1,29
Itpripl2	1,29
Brsk1	1,29
5430402E10Rik	1,28
Gm4187	1,28
Gm17365	1,28
Myo1c	1,28
Drp2	1,28
Ap4e1	1,28
Fam109b	1,28
Lekr1	1,31
Kidins220	1,31
Gnl2	1,31
Gm14346	1,31
H2-T3	1,31
Zkscan8	1,31
Ttll1	1,30
Olfr121	1,30
Zfp354b	1,30
Bdkrb2	1,30
Olfr380	1,30
Akip1	1,30
Dpp3	1,30
Cemip	1,30
Rab8a	1,30
Tssk5	1,30
Sgol2b	1,30
Kctd21	1,30
Exd1	1,30
Epyc	1,30
Paip1	1,30
Klhl25	1,30
Cldn4	1,30
Gpr179	1,30
Olfr449	1,30
Kcnma1	1,30
Flna	1,30
Ccdc102a	1,30
Zfp781	1,30
Ubxn8	1,30
Gramd1c	1,29
Vmn2r121	1,29
Klf15	1,29
Gm13271	1,29
Adad2	1,29
Bnc2	1,29
Appl1	1,29
Itch	1,29
Crlf3	1,29
Prss41	1,29
Dapk2	1,29
1110032A03Rik	1,29
Zfp41	1,29
Ovch2	1,29
Hsd17b7	1,29
Exoc8	1,28
Nanos3	1,28
Dct	1,28
Apc2	1,28
Klk6	1,28
Tank	1,28
Vmn2r70	1,28
Fat4	1,28
Ucma	1,28
Lace1	1,28
Gabrg1	1,28
Olfr480	1,28
Tcp11l1	1,28
Ebf4	1,28
Unc119	1,28
Cdh7	1,28
Gpank1	1,28
Tpm2	1,28
Glud1	1,28
5730455P16Rik	1,28
Ttr	1,27
Mal2	1,27
Tacr1	1,27
Bahd1	1,27
Arpc1a	1,27
Mmp24	1,27
Gulp1	1,27
Hmmr	1,27
Tex10	1,27
Mphosph9	1,27
Adam26a	1,27
Dock6	1,27
Guf1	1,27
Dync1li2	1,27
Qrich1	1,27
Dscr3	1,27
Dhrs7c	1,27
Gp49a	0,56
Txn2	0,56
Prss27	0,56
Lepr	0,56
Chchd10	0,56
Nr0b2	0,56
Pan2	0,56
Inpp5f	0,56
Map3k6	0,56
Pomgnt2	0,56
Tbc1d20	0,56
Tcl1b3	0,56
Hist1h4f	0,56
Prkch	0,56
Olfr972	0,56
Apoa2	0,56
Acr	0,55
Prps2	0,55
Shb	0,55
BRDN0000737820	0,55
Krtdap	0,55
Kif1c	0,55
Adamtsl3	0,55
Gatsl3	0,55
Snx31	0,55
Josd2	0,55
Olfr8	0,55
Git1	0,55
Eef1a2	0,55
Ash1l	0,55
Npr1	0,55
Mapk8ip3	0,55
Rap2a	0,55
Slc25a43	0,55
Rnaseh2a	0,55
Rhbdf1	0,55
Trim31	0,55
Siah3	0,55
Prkar2a	0,54
2200002D01Rik	0,54
Sva	0,54
Olfr1006	0,54
Spink14	0,54
B930041F14Rik	0,54
Tssk4	0,54
Olfr606	0,54
1700026L06Rik	0,54
Zmym1	0,54
Rhox7a	0,54
Slc39a13	0,54
Ptpro	0,54
Il1f9	0,54
Dpcr1	0,54
Cntnap5a	0,54
Olfr1445	0,54
Cul7	0,54
Slc24a1	0,53
Olfr738	0,53
4931417E11Rik	0,53
Rab32	0,53
Mtpn	0,53
Mapk10	0,53
Arf1	0,53
Fam122a	0,53
Rassf9	0,53
Enthd2	0,53
Kansl2	0,53
Phf13	0,53
Pglyrp1	0,53
Wdr35	0,53
Lsm14b	0,53
Cidea	0,53
Pcbp4	0,53
Dcdc2a	0,53
Camk1d	0,53
Sox5	0,53
Kctd4	0,53
Pten	0,53
Commd2	0,53
Pitpnc1	0,53
Cenpa	0,53
Klhdc9	0,52
Zfp872	1,30
Gm14305	1,29
Pank3	1,29
Il17ra	1,29
Vps37c	1,29
Lsm14a	1,29
Pif1	1,29
Cyp3a44	1,29
Fbxl21	1,29
Glra4	1,29
Txndc5	1,29
Mageb18	1,29
Slc6a2	1,29
Fam92b	1,29
Fxn	1,29
Yipf5	1,29
Tada3	1,29
Adam30	1,28
Esp31	1,28
Cdk5	1,28
1700029J07Rik	1,28
Slc16a9	1,28
Cmtm3	1,28
Arap3	1,28
Efr3a	1,28
Grk6	1,28
Fmo4	1,28
Slc39a8	1,28
Insm2	1,28
2010107G12Rik	1,28
Slc2a13	1,28
Med29	1,28
Wrap53	1,28
Cirh1a	1,28
Vmn2r75	1,28
Olfr1465	1,28
Gchfr	1,28
Liph	1,28
Golga7	1,28
Cml1	1,28
1700010B08Rik	1,28
Gpr137c	1,28
Map4k5	1,28
Sh3rf3	1,27
Slco2a1	1,27
BRDN0000737511	1,27
Isx	1,27
Nme3	1,27
Rnf215	1,27
Fundc2	1,27
Trim35	1,27
Ankzf1	1,27
Cfap69	1,27
Rab33a	1,27
Cnga1	1,27
Chrng	1,27
Ero1l	1,27
Sdpr	1,27
Pced1a	1,27
Dcaf12l2	1,27
Supv3l1	1,27
1700007B14Rik	1,26
Abca7	1,26
Asap2	1,26
Hook1	1,26
Setd4	1,26
Zfp53	1,26
Megf9	1,26
Klra17	1,26
Prmt7	1,26
Fyttd1	1,26
Noxa1	1,26
Scrt1	1,26
Gpihbp1	1,26
Spsb1	1,26
Meig1	1,26
Olfr90	1,26
Prss56	1,26
Gnat3	1,25
Zfp472	1,25
Teddm3	1,25
1700012B09Rik	1,25

Slc27a6	1,28
Xndc1	1,28
Copz2	1,28
Mettl24	1,28
Olfr704	1,28
Fam19a1	1,28
Ndfip2	1,28
Ugt1a2	1,28
Zfp648	1,28
2900011O08Rik	1,28
Kdm3b	1,28
Spata31d1d	1,28
Fam180a	1,28
Nae1	1,28
Lman1	1,28
Txlna	1,27
Smek2	1,27
Imp4	1,27
Gng5	1,27
Il23r	1,27
Neurod1	1,27
Spaca5	1,27
Abhd10	1,27
Olfr693	1,27
Dytn	1,27
Lrrn4cl	1,27
Hoxb13	1,27
Tmem70	1,27
Hoxa6	1,27
Tbc1d25	1,27
Olfr1506	1,27
Fbxo6	1,27
Pllp	1,27
Akr1c12	1,27
Rem1	1,27
Bhlhe22	1,27
Usp42	1,27
Cpeb2	1,27
Gm4846	1,26
Slc14a2	1,26
Emc9	1,26
Mgarp	1,26
Vmn1r38	1,26
Mrm1	1,26
Fam174b	1,26
Rell1	1,26
Sirpa	1,26
Klhl35	1,26
Scn10a	1,26
Susd3	1,26
Ugt1a9	1,26
Fam69b	1,26
Csad	1,26
Vip	1,26
Dmtf1	1,26
Mybpc3	1,26
Olfr1288	1,26
Kif13a	1,26
Gm6406	1,26
Cdc14b	1,26
Frat2	1,26
BRDN0000738207	1,26
AU021092	1,26
Atat1	1,26
Atp6v1e2	1,26
Dhx8	1,25
C4bp	1,25
Bex4	1,25
4930595M18Rik	1,25
Olfr935	1,25
Syce1	1,25
Slc25a53	1,25
Fgf21	1,25
Ccdc120	1,25
Eln	1,25
Lrrc42	1,25
Tdp1	1,25
Pak3	1,25
Napb	1,25
Pex16	1,25
Adam26a	1,25
Sos2	1,25
Vmn1r173	1,27
Ccdc69	1,27
Zfp28	1,27
3830403N18Rik	1,27
Tril	1,27
Chmp5	1,26
Armcx2	1,26
Gch1	1,26
Tbc1d7	1,26
Mettl7a2	1,26
Olfr1049	1,26
Sult2a7	1,26
Ccdc94	1,26
Oaf	1,26
Tnfrsf13b	1,26
Emx2	1,26
Ttyh2	1,26
Med28	1,26
Olfr346	1,26
Cyp2d9	1,26
Depdc1a	1,26
Wbscr17	1,26
Crtam	1,26
Slc25a12	1,26
Lyl1	1,26
Asxl1	1,26
Mxd3	1,26
Gcfc2	1,26
4930415O20Rik	1,26
Reck	1,26
Zfp655	1,26
Cdsn	1,25
Olfr596	1,25
Ints5	1,25
Psmd4	1,25
Bpi	1,25
Sde2	1,25
D17Wsu92e	1,25
Rita1	1,25
Arhgef26	1,25
7530416G11Rik	1,25
Sdr9c7	1,25
Tex101	1,25
Tff3	1,25
Olfr479	1,25
Tcl1b5	1,25
Pias4	1,25
E330034G19Rik	1,25
AK010878	1,24
Gm13279	1,24
E330021D16Rik	1,24
Enox1	1,24
Gpr87	1,24
Uros	1,24
Kif1c	1,24
Nkiras2	1,24
H2-M1	1,24
Aldob	1,24
Trat1	1,24
Wdfy2	1,24
Tmem154	1,24
Trim75	1,24
Nova2	1,24
Dcaf8	1,24
Fnbp1	1,24
Zfp518a	1,24
4930474N05Rik	1,24
Satb1	1,24
Wdr64	1,24
2310079G19Rik	1,24
Tbc1d8b	1,23
Strap	1,23
Ripply2	1,23
2310007L24Rik	1,23
Tia1	1,23
Adam12	1,23
Dmxl2	1,23
Mroh4	1,23
Ext1	1,23
Erf	1,23
Micu2	1,23
Specc1l	1,23
Sept1	0,52
Lztr1	0,52
Abca7	0,52
Myo19	0,52
Prss54	0,52
Il6st	0,52
Fam20c	0,52
Olfr736	0,52
Olfr617	0,52
Zfp619	0,52
Srsf6	0,52
Poll	0,52
1110065P20Rik	0,52
Adra1d	0,51
Hmgcs2	0,51
Dnaaf3	0,51
Med9	0,51
Nhlrc2	0,51
4931429I11Rik	0,51
Agbl3	0,51
Myl9	0,51
Ifit3	0,50
BRDN0000737472	0,50
Pnpla1	0,50
Vmn1r220	0,49
Fer	0,49
Fdps	0,49
D3Ertd751e	0,49
Pln	0,49
Cxxc4	0,49
Thumpd1	0,48
Gm5617	0,48
Cers4	0,00
Plekhg1	0,00
Hspbap1	0,00
Cers3	0,00
Plekhg4	0,00
1700006E09Rik	0,00
Nsa2	0,00
Mug2	0,00
Zfp846	0,00
Nampt	0,00
Man2a2	0,00
Bpifb3	0,00
Bpifb2	0,00
Bpifb1	0,00
Rfx1	0,00
Man2a1	0,00
Bpifb5	0,00
Bpifb4	0,00
4931406P16Rik	0,00
Gm9573	0,00
Rhbg	0,00
Shank3	0,00
Shank1	0,00
Syt8	0,00
Syt9	0,00
Prl	0,00
Syt1	0,00
Syt2	0,00
Mier3	0,00
Syt4	0,00
Syt5	0,00
Syt6	0,00
Atxn7l2	0,00
Sept6	0,00
Prr3	0,00
Cnppd1	0,00
Pbld1	0,00
Aqp12	0,00
Atp13a2	0,00
BRDN0000738254	0,00
BRDN0000737773	0,00
BRDN0000738369	0,00
Pmvk	0,00
Fkbp1a	0,00
BRDN0000738350	0,00
Abcb1a	0,00
Baiap3	0,00
Otop2	0,00
Dag1	0,00
Myadm	0,00
Grm8	1,25
Cpq	1,25
Tas2r114	1,25
Dcun1d3	1,25
Ube2dnl1	1,25
Foxk1	1,25
Mettl9	1,25
Erich5	1,25
Surf6	1,25
Cfhr1	1,25
Ppia	1,25
Defb11	1,25
Ttc39a	1,25
Olfr1036	1,25
Tapbpl	1,25
Ppp1r37	1,25
Uba7	1,25
Utrn	1,25
C4bp	1,25
Bicc1	1,25
Arhgap33	1,25
Kank3	1,25
Fam71f1	1,24
Muc15	1,24
Prrg4	1,24
Hsd11b2	1,24
Sgol2b	1,24
Mbtps1	1,24
Shisa4	1,24
Olfr1496	1,24
Adck1	1,24
Arl15	1,24
Olfr690	1,24
Oas3	1,24
Adcy5	1,24
Nrgn	1,24
Serbp1	1,24
Ghr	1,24
Plekhn1	1,24
Vmn2r21	1,24
Arhgdib	1,24
Cdr1	1,24
Gsdma3	1,24
Ppt2	1,23
Eif2s3x	1,23
BRDN0000738241	1,23
D3Ertd254e	1,23
Tia1	1,23
Ankrd23	1,23
C2cd4a	1,23
Rala	1,23
Kctd3	1,23
Cpeb2	1,23
Psme4	1,23
Taf3	1,23
Olfr1335	1,23
Parp2	1,23
Cul7	1,23
Pof1b	1,23
Defb46	1,23
Gm3376	1,23
Neurl4	1,23
Galk2	1,23
Fam161a	1,23
Ndufaf1	1,23
Unc50	1,23
Grin2c	1,23
Ska2	1,22
Gabra2	1,22
Slc17a9	1,22
Vmn1r23	1,22
St3gal3	1,22
Tctex1d2	1,22
Hes6	1,22
Copb1	1,22
Dnttip1	1,22
Erc2	1,22
Epha4	1,22
Ctla4	1,22
Rem2	1,22
Nmrk2	1,22
Olfr1134	1,22

Tasp1	1,25
Glra4	1,25
Supt7l	1,25
Utp14a	1,25
Cyp2d9	1,24
Gata1	1,24
Olfr132	1,24
Vmn1r33	1,24
Sh3rf3	1,24
Tmem41a	1,24
Ubr3	1,24
Dgkq	1,24
Tmem14c	1,24
Luzp1	1,24
Rad51ap1	1,24
Rora	1,24
C1qtnf7	1,24
Arpc1a	1,24
Olfr470	1,24
Uhrf1bp1l	1,24
Pdcd1	1,24
Jmjd4	1,24
Rbm7	1,24
Gm15293	1,24
Hsd11b1	1,24
Stfa2l1	1,24
Cacna2d2	1,24
Gm6034	1,24
Olfr1469	1,24
Fam122a	1,24
Chid1	1,23
Fam185a	1,23
Lnpep	1,23
Dnah5	1,23
3110021N24Rik	1,23
Ranbp6	1,23
Nkx2-5	1,23
Fbxo34	1,23
Fuk	1,23
Cubn	1,23
BRDN0000737813	1,23
Ephx1	1,23
Prp2	1,23
Prkar1b	1,23
Zbtb3	1,23
4833439L19Rik	1,23
Slco1a6	1,23
Fam45a	1,23
Dstyk	1,23
Aard	1,23
Slitrk1	1,22
Hyls1	1,22
Tmem8b	1,22
Astl	1,22
Iapp	1,22
Actc1	1,22
Adgrf5	1,22
Pde10a	1,22
Agmo	1,22
Stom	1,22
Otud7b	1,22
Slc38a5	1,22
Bmpr1a	1,22
4933433C11Rik	1,22
Dnase2b	1,22
Slc41a1	1,22
Agpat3	1,22
Atxn7l3	1,22
AI467606	1,22
Sst	1,22
Prl5a1	1,22
Sh2d7	1,22
Prc1	1,22
Rccd1	1,22
Panx2	1,22
Olfr1328	1,22
Nipal3	1,21
Wbp2nl	1,21
Sprr2e	1,21
Pla2g2c	1,21
Gstm4	1,21
BRDN0000737718	1,21
Bcat2	1,23
Fbxo31	1,23
C2cd2l	1,23
Josd2	1,23
Fxyd1	1,23
A430089I19Rik	1,23
Fgf8	1,23
Prmt8	1,23
Olfr341	1,23
Slc9a1	1,23
Gid4	1,22
Sulf2	1,22
Rnf135	1,22
Myog	1,22
Gm17252	1,22
Rasl10a	1,22
Igsf5	1,22
Garnl3	1,22
Vmn1r103	1,22
Fsd2	1,22
Ptpn18	1,22
Catip	1,22
Cwc27	1,22
Myh1	1,22
Olfr834	1,22
Uggt1	1,22
Olfr1463	1,22
Ackr4	1,22
Syt1	1,22
Abi1	1,22
Adgrb1	1,22
Olfr160	1,22
Ahrr	1,22
Asb7	1,22
Gstt4	1,22
Fxyd2	1,21
Strn	1,21
Vmn1r53	1,21
Tas2r118	1,21
Cyp27b1	1,21
Cox6b1	1,21
Ccdc14	1,21
Slc25a4	1,21
Plk3	1,21
Syt4	1,21
Tdrkh	1,21
Wfdc17	1,21
Ctsm	1,21
Olfr917	1,21
Atp13a3	1,21
Ect2l	1,21
Polr2b	1,21
Rhoa	1,21
Cdca3	1,21
Sbspon	1,21
Hnf1b	1,21
Inpp5a	1,21
BRDN0000737566	1,21
Timm9	1,21
Olfr1408	1,20
Olfr1223	1,20
Spast	1,20
Akr1c13	1,20
AW146154	1,20
C8g	1,20
Stambpl1	1,20
Lypd6	1,20
Galnt2	1,20
Fgd6	1,20
Carns1	1,20
Gm6537	1,20
Slc10a4	1,20
Olfr1443	1,20
Sprr4	1,20
Tmem194b	1,20
Car6	1,20
Slc39a9	1,20
Rock1	1,20
Aktip	1,20
Cep70	1,20
Slc11a1	1,20
Nrip3	1,20
Fbxl14	0,00
Krt10	0,00
Polr2k	0,00
Erich5	0,00
Naf1	0,00
Cebpb	0,00
Cebpa	0,00
Slc25a14	0,00
Acvr1	0,00
Cebpe	0,00
Erich3	0,00
BRDN0000738351	0,00
Usp17lc	0,00
Slc25a12	0,00
Lyrm5	0,00
Car2	0,00
Olfr1290	0,00
Slc25a11	0,00
Car3	0,00
Lrrc46	0,00
Cebpz	0,00
Cpq	0,00
Slc27a6	0,00
Arl1	0,00
Bpifb6	0,00
Zfp712	0,00
Zfp710	0,00
Hfe2	0,00
Ang5	0,00
Rbm8a	0,00
Zfp715	0,00
Mllt4	0,00
Edrf1	0,00
Mllt6	0,00
Zfp719	0,00
Mllt1	0,00
Mllt3	0,00
Rgs10	0,00
Pramef6	0,00
Krt6b	0,00
Rtf1	0,00
Krt6a	0,00
4930415L06Rik	0,00
Pramef8	0,00
Rasl10a	0,00
Gm4861	0,00
Ang6	0,00
Tmem151b	0,00
Snrpd2	0,00
Lvrn	0,00
Tgfbi	0,00
Smok3a	0,00
Smok3b	0,00
Uprt	0,00
Zswim5	0,00
Zswim7	0,00
Nupl1	0,00
Nupl2	0,00
Zswim3	0,00
Clcnka	0,00
Ndrg1	0,00
Ndrg2	0,00
Clcnkb	0,00
Ttc30a2	0,00
Npat	0,00
Cst9	0,00
Ttc30a1	0,00
Olfr601	0,00
BRDN0000738180	0,00
2010315B03Rik	0,00
Pcdha8	0,00
Maz	0,00
Hnf1b	0,00
Gga3	0,00
Gga2	0,00
Eef2kmt	0,00
Zscan10	0,00
Sorbs1	0,00
Plcd4	0,00
Cdc20b	0,00
Sorbs2	0,00
Tbx2	0,00
Mtmr4	1,22
Olfr376	1,22
Tbc1d9b	1,22
Unc5cl	1,22
Fa2h	1,22
Zfp715	1,22
Ybx2	1,22
Mcidas	1,22
Eci1	1,21
Mocs2	1,21
Sln	1,21
BRDN0000737986	1,21
Ncr1	1,21
Pyurf	1,21
3632451O06Rik	1,21
Cyp2a22	1,21
Fam45a	1,21
Plekhg2	1,21
Mrm1	1,21
Slfn3	1,21
Fam135a	1,21
Tlr6	1,21
Col6a4	1,21
Cdca2	1,21
Tnfsf18	1,21
4930467E23Rik	1,21
Gm5771	1,21
Pars2	1,21
Rhox1	1,21
Pdzd3	1,21
Ints3	1,21
Slc9a2	1,21
Cpxm2	1,21
Tmed1	1,21
Abhd6	1,21
Zfp260	1,21
Olfr372	1,21
Hamp	1,21
Abcc1	1,21
Ube2dnl2	1,21
Slc24a2	1,21
Angptl4	1,20
Vmn1r6	1,20
Clic3	1,20
Ly6k	1,20
Abcb1a	1,20
Calb1	1,20
Usp32	1,20
Cenpc1	1,20
Clip1	1,20
B3glct	1,20
Brpf3	1,20
Cyp7b1	1,20
2300009A05Rik	1,20
Lipc	1,20
Megf10	1,20
Kif3c	1,20
Adamts16	1,20
Spats2l	1,20
Pigv	1,20
Sfpq	1,20
Gpr174	1,20
Ube2v2	1,20
BRDN0000737648	1,19
Kidins220	1,19
Glycam1	1,19
Gm362	1,19
Rita1	1,19
Adamts10	1,19
Spr	1,19
Ptprn2	1,19
BRDN0000738105	1,19
Krtap24-1	1,19
Sntg1	1,19
Gm13283	1,19
Tmem212	1,19
Arhgef26	1,19
Map3k2	1,19
Gm4567	1,19
Krt23	1,19
Olfr1474	1,19
Myh9	1,19

Fam46c	1,21
Kptn	1,21
2310057J18Rik	1,21
Trim11	1,21
Actn1	1,21
Zfp317	1,21
Slc1a6	1,21
Oas1e	1,21
Col28a1	1,21
Pddc1	1,21
Ctbp2	1,21
Olfr1259	1,21
Tmem39b	1,21
Malsu1	1,21
Acp2	1,21
Zdhhc23	1,21
Zc3h14	1,21
Ndrg2	1,21
Olfr738	1,21
Scn7a	1,21
Lsr	1,21
Chrna10	1,21
Spata25	1,21
Bzrap1	1,21
Prrg2	1,21
Gsx1	1,21
Crbn	1,21
Akr1b7	1,21
Vasp	1,20
Psen1	1,20
Zfp420	1,20
Olfr1162	1,20
BC021614	1,20
Pramef25	1,20
Slc36a3	1,20
Gpcpd1	1,20
Tmem30c	1,20
Grb14	1,20
Olfr676	1,20
Cd2bp2	1,20
Trp53inp1	1,20
BRDN0000737637	1,20
Olfr1443	1,20
Klhdc10	1,20
Grk5	1,20
Rbfox1	1,20
Crip1	1,20
Klf16	1,20
Zfp248	1,20
Adam29	1,20
Uba5	1,20
Ugt2b1	1,20
Olfr448	1,20
Lhfpl2	1,19
Acadsb	1,19
Spidr	1,19
Pank2	1,19
Il13	1,19
Tspan13	1,19
Stra8	1,19
Gltpd2	1,19
Ncoa1	1,19
Ms4a7	1,19
Prl2a1	1,19
Pla2g12a	1,19
Olfr410	1,19
Adamts18	1,19
Gadd45b	1,19
Slc35g3	1,19
Gm9573	1,19
Alppl2	1,19
Psme3	1,19
Phactr4	1,19
Mtcp1	1,19
Rhobtb1	1,19
Ang4	1,19
Fam53b	1,19
Dnajb2	1,19
Gm4894	1,19
Gm17727	1,19
Rptn	1,19
Dcpp2	1,19
Gm21671	1,20
BRDN0000738223	1,20
Fam71e1	1,20
Lpgat1	1,19
Nlrp9b	1,19
Olfr541	1,19
2610020H08Rik	1,19
Map3k14	1,19
Hfm1	1,19
Abi3	1,19
Gldc	1,19
Fgf21	1,19
Tppp	1,19
Rassf3	1,19
Cyp2c54	1,19
Nlgn2	1,19
2610028H24Rik	1,19
Ffar4	1,19
Ap3b2	1,19
Hdac7	1,19
Commd10	1,19
Zp3r	1,19
Sytl1	1,19
Rsl1	1,19
Zhx2	1,19
Catsper2	1,19
Rif1	1,19
Mtmr11	1,19
Usp5	1,19
Tceal1	1,19
2010012O05Rik	1,19
1700016K19Rik	1,18
Cspp1	1,18
Nipal4	1,18
Vip	1,18
Tfdp1	1,18
Ptpn7	1,18
Ctsw	1,18
Apoc4	1,18
Chga	1,18
Mamdc4	1,18
Zfp11	1,18
Txndc8	1,18
Il1f6	1,18
Dusp1	1,18
Reg3g	1,18
Oas2	1,18
Pou2f2	1,18
Mill2	1,18
Zfp750	1,18
Dlgap4	1,18
Mtx2	1,18
Amotl2	1,18
Npffr1	1,18
L3mbtl3	1,18
Pmm1	1,18
Nkx2-4	1,18
Ggh	1,18
Clec4g	1,18
Lactb2	1,17
Sema6c	1,17
Olfr1129	1,17
Acp2	1,17
1700020A23Rik	1,17
Mvb12a	1,17
BRDN0000738185	1,17
Kbtbd3	1,17
Dnah8	1,17
Fam69b	1,17
Mgat4e	1,17
Pdzd8	1,17
Tmem65	1,17
Gm17689	1,17
Tns3	1,17
Gm4858	1,17
Rnf169	1,17
Ms4a6c	1,17
Pzp	1,17
Acsm1	1,17
Sept8	1,17
Tkfc	1,17
Rab3b	1,17
Mrps2	0,00
Lingo4	0,00
Lingo3	0,00
Lingo2	0,00
Tbx3	0,00
Tshz2	0,00
Murc	0,00
Pifo	0,00
BRDN0000738085	0,00
Inhba	0,00
Inhbb	0,00
Inhbc	0,00
Inhbe	0,00
Mier1	0,00
Iqsec3	0,00
Tbx6	0,00
Pgk1	0,00
Mier2	0,00
Tamm41	0,00
Il17f	0,00
Syt3	0,00
Il17a	0,00
Il17b	0,00
Speer4d	0,00
Speer4e	0,00
Speer4f	0,00
Gp1bb	0,00
Speer4b	0,00
Speer4c	0,00
Fam151b	0,00
Cma2	0,00
Cma1	0,00
Fam151a	0,00
Runx1t1	0,00
Fam104a	0,00
AI314180	0,00
Clrn2	0,00
Clrn3	0,00
Itm2b	0,00
Clrn1	0,00
Kcnc4	0,00
Porcn	0,00
Tex9	0,00
Kcnc1	0,00
Kcnc2	0,00
Kcnc3	0,00
Ralgps2	0,00
Ralgps1	0,00
B3gntl1	0,00
Gpx3	0,00
Mkl2	0,00
Bmx	0,00
4931429L15Rik	0,00
Miip	0,00
Gpx2	0,00
Lta4h	0,00
Lmna	0,00
Sult2b1	0,00
Olfr452	0,00
Slc30a10	0,00
Sorcs2	0,00
Cmah	0,00
Arhgef26	0,00
Vwde	0,00
Ubfd1	0,00
Scg2	0,00
Fkbp15	0,00
Mcl1	0,00
Gcm1	0,00
Pttg1	0,00
Gcm2	0,00
Gm527	0,00
Trim34b	0,00
Gm525	0,00
Tssk6	0,00
Cyp2r1	0,00
Tssk2	0,00
Tssk3	0,00
Tssk1	0,00
Gria2	0,00
Gria3	0,00
Gpr182	0,00
Iqcf5	1,19
Crtam	1,19
Adam11	1,19
Snx13	1,19
Gm14393	1,19
Kansl2	1,19
Pdzd11	1,19
Cox8a	1,19
Pde6d	1,19
Slitrk3	1,19
Crygn	1,19
Ccdc124	1,19
Nlrp12	1,19
Acot5	1,19
Grin1	1,19
Ube2n	1,19
Gm10100	1,18
Tbc1d31	1,18
Impad1	1,18
Olfr620	1,18
Rorb	1,18
Gm13212	1,18
Izumo1r	1,18
Dctpp1	1,18
Ccdc50	1,18
Pfdn4	1,18
Ube2ql1	1,18
Susd4	1,18
Fgf18	1,18
Fmn1	1,18
Ophn1	1,18
BRDN0000737818	1,18
Stk3	1,18
Ppil4	1,18
Gm7157	1,18
Cnot10	1,18
Olfr734	1,18
Rbm12b2	1,18
Arhgef25	1,18
Rbm20	1,17
Zc3h8	1,17
Tcaf2	1,17
Phkg2	1,17
Atf3	1,17
Chmp5	1,17
Cyp11b2	1,17
Slc22a27	1,17
Pak2	1,17
Olfr93	1,17
Tbxa2r	1,17
Cldn17	1,17
Olfr26	1,17
Gm2913	1,17
Olfr33	1,17
Amhr2	1,17
G6pdx	1,17
Acox3	1,17
Gdf2	1,17
Cst11	1,17
Vps72	1,17
Lpar4	1,17
Olfr803	1,17
Ano9	1,17
Tcf7l2	1,17
Zan	1,17
Pygl	1,17
C87414	1,17
Zfp759	1,17
Zkscan1	1,17
Grap2	1,17
Fscn2	1,17
Tmf1	1,17
Dcbld2	1,17
Gm20823	1,17
Cryba4	1,17
Zyg11b	1,17
Taf15	1,17
Pcca	1,16
3110009E18Rik	1,16
Olfr740	1,16
Itm2b	1,16
Zfp960	1,16

Ube2z	1,19
Esp23	1,19
Cacnb4	1,19
Fiz1	1,18
Tmem69	1,18
Dmrtc2	1,18
Klrg1	1,18
Cnp	1,18
Kdf1	1,18
Gckr	1,18
Ryr1	1,18
Mthfsl	1,18
Pcdhb3	1,18
Slc4a4	1,18
Dxo	1,18
Tmprss13	1,18
Zc3hav1	1,18
Mrps7	1,18
Kdm3a	1,18
Zfp386	1,18
Tomm22	1,18
Adamtsl2	1,18
Lmod1	1,18
Rchy1	1,18
Tpm4	1,18
Ppard	1,18
Zfp53	1,18
Arid4a	1,18
Rims4	1,18
Hsf5	1,18
Frem2	1,18
Tbx3	1,18
Tmem207	1,18
Prps1	1,18
Mrps33	1,18
Sp3	1,18
Trpv2	1,17
Eno3	1,17
Olfr24	1,17
BRDN0000738116	1,17
Olfr512	1,17
BRDN0000737630	1,17
Pnoc	1,17
Kalrn	1,17
Txnrd3	1,17
Ap3s1	1,17
Olfr807	1,17
Garnl3	1,17
Mpp1	1,17
Ppap2b	1,17
Olfr420	1,17
Ctsh	1,17
Afap1l2	1,17
Sox7	1,17
Flt3	1,17
Olfr1406	1,17
Mtif3	1,17
Pydc4	1,17
Actn4	1,17
Commd9	1,17
Dlg1	1,17
Ccdc64b	1,17
Slfn9	1,17
C77370	1,17
C1qa	1,17
Ggact	1,17
Als2cl	1,17
Madd	1,17
Nemf	1,16
Wdr92	1,16
Adgrb1	1,16
Rprd1b	1,16
Vmn1r86	1,16
Tas2r131	1,16
Nup37	1,16
Ftmt	1,16
Tsen34	1,16
Ngb	1,16
Hoxc6	1,16
BRDN0000737975	1,16
Fxyd1	1,16
Hoxc12	1,16
H2-Eb1	1,17
Tmem190	1,17
Ano9	1,17
Snx32	1,17
Fam101b	1,17
BC048609	1,17
Tlr8	1,17
Asf1a	1,17
Efcab9	1,16
Sstr3	1,16
Vldlr	1,16
Optn	1,16
Ttc9c	1,16
Zc3h6	1,16
Polr3k	1,16
A230046K03Rik	1,16
Cfap221	1,16
Hist2h2ac	1,16
Cyp4f37	1,16
Nudcd1	1,16
Stxbp5l	1,16
Wscd1	1,16
Ddr1	1,16
Ccar2	1,16
Trim14	1,16
Thsd7b	1,16
Skint7	1,16
Ttc39a	1,16
Tpd52l2	1,16
Itgb2l	1,16
Dxo	1,16
Magea2	1,16
Slc12a2	1,16
Hdhd1a	1,16
Dusp10	1,16
Kcnmb2	1,16
Rasl11a	1,16
Syt17	1,16
Ankrd35	1,16
Zfp719	1,16
Nos3	1,16
Sval2	1,16
Grhl2	1,15
1700012A03Rik	1,15
Ms4a8a	1,15
Apbb2	1,15
Pou1f1	1,15
Pygm	1,15
Chrnb4	1,15
Xk	1,15
Slc10a6	1,15
Ttc37	1,15
Adamts8	1,15
Tob1	1,15
Pja2	1,15
Fam63b	1,15
Olfr1252	1,15
Prom1	1,15
Itgam	1,15
Olfr8	1,15
Padi2	1,15
Plce1	1,15
Nmrk2	1,15
Serpinb11	1,15
Stfa1	1,15
Ndc1	1,15
Olfr1247	1,15
1700101E01Rik	1,15
Ebpl	1,15
Myh11	1,15
Zfp101	1,15
Olfr1131	1,15
Rftn2	1,15
Prdx5	1,15
Olfr1089	1,15
Gtf3c1	1,15
Gm13871	1,15
Atxn7l2	1,15
Pcsk2	1,15
Commd3	1,15
Slc38a10	1,15
Ptchd4	1,15
Gpr183	0,00
Klra4	0,00
Gria4	0,00
Aqp11	0,00
Krt71	0,00
Defa26	0,00
Sass6	0,00
Tmem114	0
L1cam	0
Slc27a5	0
Slc27a4	0
4933412E24Rik	0
Cep19	0
Slc27a1	0
Slc27a2	0
Dydc2	0
Dydc1	0
Hmgxb3	0
Hmgxb4	0
Lrrk1	0
Olfr1176	0
Olfr971	0
Cdhr1	0
Cdhr2	0
Cdhr3	0
Them5	0
Zbtb48	0
BRDN0000737406	0
Slco6c1	0
Dbn1	0
1700024P16Rik	0
Dazl	0
Trim39	0
Trim38	0
Tcof1	0
Ush2a	0
Pxt1	0
Trim33	0
Trim32	0
Trim37	0
Trim35	0
Gm13288	0
Foxo3	0
Olfr978	0
Cyp39a1	0
Dlat	0
Nbr1	0
BC049762	0
Cirbp	0
Vmn1r149	0
Ifi27l2b	0
Ifi27l2a	0
Thap11	0
Prmt10	0
Btaf1	0
Wsb1	0
Fam65a	0
Tmem89	0
Dbnl	0
Olfr523	0
Olfr522	0
Olfr521	0
Mst1r	0
Olfr525	0
Olfr524	0
Fam114a1	0
Cmas	0
Aldoa	0
Aldoc	0
Aldob	0
Vmn1r138	0
Cox6c	0
Clca2	0
Cdk5	0
Pip4k2a	0
Slc24a4	0
Slc24a5	0
Slc24a2	0
Slc24a3	0
BRDN0000737843	0
Gm21671	0
Nsfl1c	0
Asic5	1,16
Tmem59	1,16
Zfp58	1,16
Glt28d2	1,16
Tmem221	1,16
Ss18l1	1,16
Cpne9	1,16
Dnajb5	1,16
4930503L19Rik	1,16
Zmym6	1,16
Mblac2	1,16
Dysf	1,16
Slc39a12	1,16
Myot	1,16
Tcl1b1	1,16
Zfp511	1,16
Inf2	1,16
Ptpn9	1,16
Miip	1,16
Gpr63	1,16
Fbxo31	1,16
Sergef	1,16
Fam185a	1,16
Tectb	1,16
Ercc5	1,16
Zfyve21	1,16
Vsig8	1,16
Pip	1,16
Pnck	1,15
Olfr444	1,15
Txnrd1	1,15
Igdcc3	1,15
Amdhd2	1,15
Tstd3	1,15
Prickle2	1,15
Acr	1,15
Pygm	1,15
Tmem125	1,15
Mphosph9	1,15
Olfr1087	1,15
Krt14	1,15
Actg2	1,15
Mrgprb2	1,15
Thop1	1,15
Fat4	1,15
Bnipl	1,15
Hectd2	1,15
Tex30	1,15
Pitx2	1,15
Krit1	1,15
Bre	1,15
Cabin1	1,15
Masp1	1,15
Cmas	1,15
AF366264	1,15
Edc3	1,15
Wnt2	1,15
Matn2	1,15
Tfap2e	1,15
Zfp456	1,15
Tcp10a	1,15
Tmem158	1,15
Tbk1	1,15
BRDN0000738156	1,14
Fam117b	1,14
Fn3krp	1,14
Kifc3	1,14
Meis3	1,14
Ndrg2	1,14
Ccnl2	1,14
Kirrel	1,14
B3gnt6	1,14
Tas2r120	1,14
Mybbp1a	1,14
Klk1b5	1,14
Ptgfrn	1,14
Ugt3a2	1,14
Tsnaxip1	1,14
Fezf1	1,14
Sipa1l1	1,14
Ddx52	1,14
Tg	1,14

Arhgap29	1,16
Ccdc43	1,16
Prss1	1,16
Zfp874a	1,16
R3hdml	1,16
Slc25a34	1,16
Ccdc70	1,16
Phlda1	1,16
Ccdc50	1,16
Nuf2	1,16
Gm5549	1,16
Scgb2b7	1,15
Trim34b	1,15
Vps45	1,15
Vmn1r180	1,15
Olfr8	1,15
Pax7	1,15
Rnf165	1,15
Mtmr9	1,15
St8sia4	1,15
Kif26b	1,15
Pdp2	1,15
Zmynd15	1,15
Nos1	1,15
Pcdhb5	1,15
Thbs3	1,15
Jade1	1,15
Ubxn2b	1,15
Dusp23	1,15
Olfr1317	1,15
Lonrf3	1,15
Mef2a	1,15
Fry	1,15
BRDN0000737734	1,15
Hspd1	1,15
Gna11	1,15
Parp11	1,15
Sema4b	1,15
Tgfbr2	1,15
Ppp1r3c	1,15
Pitpnb	1,15
Abcf3	1,15
Tlx3	1,15
Spata24	1,15
B3gnt4	1,15
Pvr	1,15
BRDN0000737537	1,15
Cyp2d12	1,15
Zcwpw1	1,15
Ckb	1,15
AI837181	1,15
Tm4sf4	1,15
Gucy2c	1,15
Ttll2	1,15
Map2	1,15
Nr0b1	1,15
Tmem252	1,15
Tpd52	1,14
Eif4g3	1,14
Chst5	1,14
Slc16a2	1,14
Olfr177	1,14
Vmn2r46	1,14
Atg2a	1,14
Lce1k	1,14
Vmn2r36	1,14
Gucy1a2	1,14
Rgs18	1,14
BRDN0000737480	1,14
Tspan10	1,14
Kcnj5	1,14
Mkl2	1,14
Npy5r	1,14
Olfr114	1,14
Il5	1,14
Ralgds	1,14
Gngt2	1,14
Vmn1r222	1,14
Lyl1	1,14
Ager	1,14
Atp6ap2	1,14
Ifrd2	1,14
Tnp1	1,14
Cul9	1,14
Actrt3	1,14
Sec16a	1,14
Slc40a1	1,14
Lrrc16b	1,14
Gas2l2	1,14
Spata31d1a	1,14
Olfr441	1,14
Gzf1	1,14
Ascl2	1,14
Gm13078	1,14
Zfp787	1,14
Npnt	1,14
Atf6	1,14
Col6a5	1,14
Zfp472	1,14
Lcn9	1,14
Akr1c14	1,14
Rasal2	1,14
Synj2bp	1,14
Trpm8	1,14
Bcas2	1,14
Lrp2	1,14
Akap10	1,14
Cyp4a12b	1,14
Sp140	1,14
Fibin	1,14
1700018C11Rik	1,14
Ica1	1,14
Lct	1,14
Olfr815	1,14
Nps	1,14
Pitpnc1	1,14
Fam208a	1,14
Olfr521	1,14
Pard6a	1,14
Fah	1,14
Ccr9	1,13
Ufl1	1,13
Musk	1,13
BRDN0000737428	1,13
Olfr417	1,13
Ctse	1,13
BRDN0000737852	1,13
Ash2l	1,13
Aunip	1,13
Bmp2	1,13
Plekha5	1,13
Olfr725	1,13
Plxdc1	1,13
Nek7	1,13
Hist1h3g	1,13
A230065H16Rik	1,13
Slit3	1,13
Olfr745	1,13
Nmral1	1,13
Fam160a1	1,13
Prss48	1,13
Mettl10	1,13
Gabra6	1,13
Ankrd6	1,13
Chst14	1,13
Vwa5a	1,13
Dusp7	1,13
Gm2012	1,13
BRDN0000738053	1,13
Nol11	1,13
Eif5a2	1,13
Sgpl1	1,13
Gnpda1	1,13
Ndufa8	1,13
1700020D05Rik	1,12
Gigyf1	1,12
Slc26a3	1,12
Prdm10	1,12
Bclaf1	1,12
Zfp692	1,12
Tbc1d21	1,12
Park2	1,12
Tcf23	1,12
Prdx3	1,12
Prss55	0
Lman1l	0
Eif2b4	0
Uggt1	0
Usp8	0
Sel1l3	0
Eif2b5	0
Gm15023	0
Sel1l2	0
Tppp	0
Usp1	0
Fnd3c2	0
Arid4b	0
Olfr679	0
Usp7	0
Olfr1110	0
Olfr1111	0
Card9	0
Olfr1113	0
Olfr1115	0
Olfr1116	0
Il1rap	0
Nr1d1	0
Nr1d2	0
Calr4	0
Tprkb	0
Card6	0
Znf512b	0
BC017643	0
Rpl38	0
Rpl39	0
Rpl36	0
Ocstamp	0
Rpl34	0
Rpl35	0
Rpl32	0
Car8	0
Rpl30	0
Rpl31	0
Anp32e	0
Vsx1	0
Olfr672	0
Fam13b	0
Fam13c	0
Fam13a	0
Bcas2	0
Bcas3	0
Bcas1	0
Hacd1	0
Exosc10	0
Gm8369	0
Arl2	0
Mthfr	0
Mthfs	0
Rad50	0
Ddr2	0
AA415398	0
Prss56	0
Sfmbt2	0
Zzef1	0
Rb1	0
Hist3h2ba	0
Brf2	0
Cebpg	0
Brf1	0
Fuca1	0
Fuca2	0
Cracr2a	0
Mlycd	0
Krt79	0
Hist4h4	0
F830016B08Rik	0
Ldb3	0
Efhb	0
Tnfrsf12a	0
Ado	0
Vmn1r54	0
1700013G24Rik	0
Farsb	0
Farsa	0
Tubg2	0
Uap1l1	0
Pvrl2	1,14
Sec24d	1,14
Vmn1r196	1,14
Dnajb2	1,14
Ssh1	1,14
Kcnc1	1,14
Lgals9	1,14
Naga	1,14
Ccdc32	1,14
Krtap19-5	1,14
Fbln5	1,13
Arl9	1,13
BRDN0000738001	1,13
2900026A02Rik	1,13
Mast2	1,13
Fundc1	1,13
Scgb2b12	1,13
Hnrnpul1	1,13
Psme3	1,13
Tctex1d4	1,13
Emc8	1,13
Cdh24	1,13
Gm14496	1,13
Ints2	1,13
Tex261	1,13
Lfng	1,13
Ago1	1,13
Hrasls	1,13
Rest	1,13
Adcy7	1,13
Il21r	1,13
Sult2a5	1,13
Zfp354a	1,13
Rpl7	1,13
Vmn2r32	1,13
Spata3	1,13
Egln3	1,13
Gm136	1,13
Brdt	1,13
Kap	1,13
Lrtm2	1,13
AI317395	1,13
Pcgf3	1,13
Olfr1044	1,13
Mpp1	1,13
Ly6i	1,13
Sla	1,13
Slc5a5	1,13
Olfr164	1,13
Nppa	1,12
Ier2	1,12
Ces4a	1,12
Zfp523	1,12
Tmem132c	1,12
Olfr67	1,12
Chrna5	1,12
Gpr179	1,12
Abca4	1,12
Kcnd3	1,12
Gm10024	1,12
Bhlha15	1,12
6430531B16Rik	1,12
Wfikkn2	1,12
4930562C15Rik	1,12
Tmco3	1,12
Tmem18	1,12
Cnih2	1,12
Lrrc6	1,12
Gbp11	1,12
Cables2	1,12
Pou2af1	1,12
Cyp2u1	1,12
Creb5	1,12
St6galnac5	1,12
Fam83f	1,12
Trim39	1,12
4933425L06Rik	1,12
Dopey2	1,12
0610007P14Rik	1,12
Ikbke	1,12
Hoxd9	1,12
Foxb1	1,12

B3gnt8	1,14
Stfa3	1,14
B3gnt3	1,14
Insl3	1,13
Wnt10b	1,13
Eras	1,13
Gldc	1,13
Ces1d	1,13
Dll4	1,13
Ntrk3	1,13
Ambp	1,13
Vmn1r6	1,13
Tsr3	1,13
4933402D24Rik	1,13
Hexim1	1,13
Zfp712	1,13
Ssx2ip	1,13
Tsc22d4	1,13
Plekhn1	1,13
Evi5l	1,13
Bard1	1,13
Kcnj9	1,13
Tle1	1,13
Aifm2	1,13
2810417H13Rik	1,13
Whamm	1,13
Tmem204	1,13
Cyp2c40	1,13
Tex13a	1,13
Nbn	1,13
Ammecr1	1,13
Sec1	1,13
Ctsr	1,13
BRDN0000737677	1,13
Clec9a	1,13
Gstp1	1,13
Poteg	1,13
U2surp	1,13
Psmd14	1,13
Sec63	1,13
Pdzd9	1,13
Vmn2r33	1,12
Gprin2	1,12
Gnrh1	1,12
Smurf2	1,12
Bri3bp	1,12
Vwa8	1,12
Rab43	1,12
Ttll5	1,12
Spcs3	1,12
Slc27a5	1,12
Gpbar1	1,12
Hdac7	1,12
Rtkn2	1,12
Gucd1	1,12
Zfp65	1,12
Rsrc1	1,12
BRDN0000738025	1,12
Kctd19	1,12
Olfr45	1,12
BRDN0000737981	1,12
Ear6	1,12
Kcnj1	1,12
Tekt2	1,12
Col4a6	1,12
Ppfia2	1,12
Mapk1ip1l	1,12
Vps54	1,12
Pld3	1,12
BRDN0000738327	1,12
Gm7694	1,12
Igtp	1,11
Skint7	1,11
Zfp865	1,11
Nkx3-2	1,11
Ranbp9	1,11
Slc25a43	1,11
Sun2	1,11
Gucy2d	1,11
BRDN0000737744	1,11
Slc34a2	1,11
Cnpy1	1,11
Sipa1l1	1,12
Tdrd9	1,12
Gnptg	1,12
Add1	1,12
1810011O10Rik	1,12
Ehmt1	1,12
Fbxo7	1,12
Ch25h	1,12
4930432K21Rik	1,12
Rimkla	1,12
P2rx4	1,12
Tmem119	1,12
BRDN0000737959	1,12
Susd5	1,12
4930453N24Rik	1,12
Osbpl6	1,12
Cuta	1,12
Leap2	1,12
Hacl1	1,12
Degs1	1,12
Clpb	1,12
Eif2ak2	1,12
Sart1	1,12
Nr1h4	1,12
Snhg11	1,12
Slc28a2	1,12
Pex6	1,11
Hs3st1	1,11
Cand1	1,11
Dennd6a	1,11
Cmtm1	1,11
Arhgap10	1,11
Tspan17	1,11
Otx2	1,11
Mcam	1,11
Rap1gds1	1,11
Nudt1	1,11
Dagla	1,11
Pde1a	1,11
Lce1a2	1,11
Mrps24	1,11
S100a13	1,11
Fhod1	1,11
Zfp341	1,11
Zfp87	1,11
Lcn2	1,11
Cmpk1	1,11
Scgb1b30	1,11
Ubxn1	1,11
CK137956	1,11
Etv4	1,11
Hmox2	1,11
Ifne	1,11
Ms4a5	1,11
Olfr1031	1,11
Olfr1201	1,11
Cracr2b	1,11
Mrpl27	1,11
Zfp600	1,11
Rab1b	1,11
BRDN0000737506	1,11
Glra1	1,11
Maats1	1,11
Bsx	1,11
Hvcn1	1,11
Atp6v0a4	1,10
Pcdhb18	1,10
Slc25a11	1,10
Phf11d	1,10
9430007A20Rik	1,10
Olfr963	1,10
Dnajc13	1,10
Sdr16c6	1,10
Blmh	1,10
Crnn	1,10
Coq9	1,10
Vmn1r196	1,10
Cfhr2	1,10
Fam118a	1,10
Kmo	1,10
Pnlip	1,10
Adcy5	1,10
Mettl1	0
Cyp2s1	0
Purg	0
Lin54	0
1810022K09Rik	0
Ncor1	0
Lysmd3	0
Rps6kl1	0
Ccdc90b	0
Ttc23l	0
Olfr948	0
Pebp1	0
Olfr945	0
Gkn1	0
Gkn2	0
Gkn3	0
Magohb	0
Abhd17b	0
Olfr943	0
Gng11	0
Hiat1	0
Rbmxl2	0
Mettl6	0
Pdss2	0
Pcolce	0
Lypd2	0
Flnb	0
Flnc	0
Gltpd2	0
Cep164	0
4930524N10Rik	0
Ankrd54	0
Cep162	0
Adm	0
Lypd1	0
Fgr	0
Irf5	0
Apaf1	0
Lrriq1	0
Lrriq3	0
Lrriq4	0
Ptx4	0
Fgb	0
Ythdc1	0
Vmn2r84	0
Vmn2r85	0
Vmn2r86	0
Tgm3	0
Hltf	0
Cnnm4	0
Vmn2r82	0
Vmn2r83	0
Pydc3	0
Vmn2r88	0
Vmn2r89	0
Mageb3	0
Tspan15	0
Mageb1	0
Acsm1	0
Qpct	0
Mageb4	0
Mageb5	0
Ptcra	0
Egf	0
Slc4a11	0
Slc4a10	0
2300002M23Rik	0
Hdgfl1	0
Zfp524	0
Mal	0
Klc1	0
4930519G04Rik	0
Rnf217	0
BRDN0000737798	0
Pou5f1	0
Chat	0
Pou5f2	0
Gfpt2	0
BRDN0000738306	0
Rnf138rt1	0
BC026585	0
Wfikkn1	0
Gm1979	1,12
Stab1	1,12
Zfp947	1,12
Utp11l	1,12
Olfr52	1,12
P3h4	1,12
Gpr107	1,12
Hrg	1,12
Ptp4a3	1,12
Ube2s	1,11
Fgg	1,11
Ackr1	1,11
Adnp2	1,11
Hddc3	1,11
Wfdc10	1,11
Agfg2	1,11
Bpifa1	1,11
Olfr1314	1,11
Trim67	1,11
Actr3b	1,11
Sugp2	1,11
Mtfmt	1,11
BRDN0000737596	1,11
Unc13d	1,11
Olfr1141	1,11
Ptpn7	1,11
Gna13	1,11
Pgam5	1,11
Neb	1,11
Ctr9	1,11
Rtbdn	1,11
Prx	1,11
Tmbim1	1,11
Kif9	1,11
Eno2	1,11
Klb	1,11
Kdm8	1,11
Tmx3	1,11
Plcl1	1,11
Olfr1404	1,11
Olfr1280	1,11
Xlr3c	1,11
Rarg	1,11
Kcnd2	1,10
Kncn	1,10
Rpf2	1,10
Ceacam5	1,10
Prpsap2	1,10
Grap	1,10
Try10	1,10
Chchd1	1,10
Nxt2	1,10
Scn1b	1,10
Mlana	1,10
Rbms3	1,10
Prss35	1,10
1700023F06Rik	1,10
Dhx34	1,10
Hmox2	1,10
Ecscr	1,10
Ip6k2	1,10
Slu7	1,10
Prkag2	1,10
Aars2	1,10
Rps6kb2	1,10
Slc22a20	1,10
Cd248	1,10
Fbxl13	1,10
Bcar1	1,10
Gabrg3	1,10
Rnaseh2a	1,10
Anxa11	1,10
Acadvl	1,10
Vmn1r222	1,10
Olfr893	1,10
9130023H24Rik	1,10
Tbc1d13	1,10
Serf1	1,10
Txndc15	1,10
Amelx	1,10
Bmpr1b	1,10
Olfr186	1,10

Krtap15	1,11	Pparg	1,10	Raly	0	Scgb2b7	1,10
Ube2e3	1,11	Atxn3	1,10	Vmn2r96	0	Grm1	1,10
BRDN0000738347	1,11	Krt72	1,10	Tmtc4	0	Zfp280d	1,10
Pdzk1ip1	1,11	Tbc1d2	1,10	Vmn2r95	0	Pgap3	1,09
Btnl1	1,11	Serpinb7	1,10	Siglecf	0	Ms4a4d	1,09
Umodl1	1,11	Hsf1	1,10	Cd33	0	Zfp367	1,09
Slc41a3	1,11	Krt77	1,10	Pold1	0	Ash2l	1,09
Rps6ka3	1,11	Mien1	1,10	Pold2	0	Adamtsl2	1,09
2510039O18Rik	1,11	Fzd10	1,10	Zfp711	0	Olfr669	1,09
Ogfrl1	1,11	Col23a1	1,10	Pold4	0	Ctdsp1	1,09
Fam168a	1,11	Serinc5	1,10	Pcdha10	0	Mppe1	1,09
Olfr695	1,11	Bdp1	1,10	Vmn2r93	0	Anapc1	1,09
Rbm44	1,11	Sptssb	1,10	Pcdha12	0	Nxf7	1,09
Ankrd61	1,11	Gm597	1,10	Apol9b	0	Olfr322	1,09
Mvb12a	1,11	Alk	1,10	Apol9a	0	Cd300lh	1,09
Zfp444	1,11	Igsf23	1,10	Krtap20-2	0	Rnase2a	1,09
Il9r	1,11	Sec14l4	1,10	Serpinb9c	0	Lpar3	1,09
A530099J19Rik	1,10	Olfr571	1,10	Hdac2	0	Ecm1	1,09
Aqp4	1,10	Hoxc5	1,10	Serpinb9e	0	Gpr55	1,09
Drc7	1,10	Gucy1b3	1,10	Serpinb9d	0	4933411K16Rik	1,09
AA467197	1,10	Sp7	1,10	Serpinb9g	0	Mrpl54	1,09
Rbp3	1,10	Nfatc2	1,09	Serpinb9f	0	Arl11	1,09
Ppp5c	1,10	B3galt1	1,09	Hdac9	0	Calr3	1,09
Aspm	1,10	Olfr301	1,09	Tshb	0	Itsn2	1,09
Hecw2	1,10	Cnr1	1,09	Foxr2	0	Nsmf	1,09
Ndufs3	1,10	Psmc3	1,09	Cel	0	Col8a2	1,09
Nr2c2	1,10	Bcl3	1,09	Pdss1	0	Zfp790	1,09
Lca5l	1,10	Ttc28	1,09	Cyp2d40	0	Olfr688	1,09
Cfap20	1,10	Elf4	1,09	Gstcd	0	Tmem29	1,09
Pcdhb14	1,10	Ccdc85a	1,09	Gzf1	0	Gm973	1,09
Prosc	1,10	Micall2	1,09	Ntf3	0	Gm13083	1,09
Ankrd35	1,10	Map2k3	1,09	Wdr45	0	Lurap1	1,09
Cnst	1,10	Gltscr1l	1,09	Tnr	0	Serpinb1a	1,09
Hs3st3a1	1,10	Notch2	1,09	Gmpr	0	Lrfn3	1,09
Mppe1	1,10	Cpxcr1	1,09	Gmps	0	Actl7a	1,09
Pacrgl	1,10	Wipf2	1,09	Epas1	0	Klhdc3	1,09
Saysd1	1,10	Olfr1389	1,09	Tmtc2	0	1190003K10Rik	1,09
Bid	1,10	Rragd	1,09	2610318N02Rik	0	Olfr1221	1,09
Cend1	1,10	Mbd3l1	1,09	Dpy19l1	0	Nmb	1,09
Exoc3l	1,10	Olfr1317	1,09	Fli1	0	Olfr1490	1,09
Rdh7	1,10	Nin	1,09	BRDN0000738323	0	Dock5	1,09
Fkbp11	1,10	Myh4	1,09	3830417A13Rik	0	Mtf2	1,09
Pcdh12	1,10	Olfr372	1,09	1700093K21Rik	0	Tmem40	1,09
Cbln4	1,10	Aspa	1,09	Cyp26b1	0	Bphl	1,09
Setd6	1,10	Arl13b	1,09	Upf3b	0	Cd300lg	1,09
Hdac9	1,10	Car5a	1,09	Upf3a	0	Ceacam12	1,09
Lbr	1,10	Zbtb20	1,09	Cog5	0	Nhsl2	1,09
Kif1a	1,10	Tgfbr1	1,09	Gm13139	0	Naa50	1,09
Ormdl2	1,10	Skint10	1,09	Agmat	0	Olfr1491	1,09
Clec2g	1,10	Aldh2	1,09	Strc	0	Pcdh19	1,09
Serpine1	1,10	Ilkap	1,09	Cyp4a32	0	Psg21	1,09
Nelfcd	1,10	Dbx1	1,09	Cyp4a31	0	Abtb1	1,09
0610010K14Rik	1,10	Wdr27	1,09	Vmn2r98	0	Fam110b	1,09
Rbp1	1,10	Trip13	1,09	P2ry6	0	4930548H24Rik	1,09
Izumo1r	1,10	Rassf2	1,09	P2ry4	0	Chrm4	1,09
4930435E12Rik	1,10	Enam	1,09	BRDN0000737995	0	Metrn	1,08
Xlr5a	1,10	Ppt2	1,09	BRDN0000737994	0	Rbsn	1,08
Olfr657	1,10	Xrn1	1,09	Tmtc1	0	Fbxl22	1,08
Hcn1	1,10	H2-T22	1,08	Pvr	0	Ndufb7	1,08
Phf21a	1,09	Gfi1	1,08	Nr1h3	0	Bhlha9	1,08
Tspan5	1,09	Wnt3a	1,08	BRDN0000737990	0	Trim24	1,08
Micu3	1,09	Lrig3	1,08	BRDN0000737993	0	Clec2g	1,08
Stard8	1,09	Bbc3	1,08	BRDN0000737992	0	Smpd1	1,08
Dapk3	1,09	Trmt2a	1,08	Zfp521	0	Osbpl2	1,08
Cdk2ap2	1,09	Cyp4a32	1,08	Ropn1	0	Gata2	1,08
Fam212a	1,09	Ndufb10	1,08	Zfp523	0	Olfr1391	1,08
Erbb3	1,09	Dusp11	1,08	Pydc4	0	Sh3bgrl3	1,08
Dennd1c	1,09	Prrg2	1,08	Clcn7	0	Cebpa	1,08
Mfsd3	1,09	Smg9	1,08	Clcn6	0	Srms	1,08
BRDN0000737690	1,09	4930415F15Rik	1,08	Clcn5	0	Zfp110	1,08
Zfp273	1,09	Atp8a2	1,08	Zfp526	0	Lin52	1,08
4930506M07Rik	1,09	Lrrc73	1,08	Hfm1	0	Defb18	1,08
Kcnc3	1,09	Olfr1402	1,08	Epb4.1l2	0	Scarb2	1,08
Vmn1r29	1,09	Cldn8	1,08	Spam1	0	Elavl3	1,08
P2ry1	1,09	Ndufs1	1,08	Ikzf5	0	Pus10	1,08
Tac2	1,09	Olfr1313	1,08	Ikzf4	0	Cdkn1b	1,08
Aim2	1,09	Zfyve26	1,08	BRDN0000738061	0	Clec4b2	1,08
Myh1	1,09	Grap	1,08	Ufsp2	0	Bbc3	1,08
Dopey2	1,09	Lrtm2	1,08	Ikzf1	0	Cela3b	1,08
Dpf1	1,09	Glra4	1,08	Gm732	0	Slc4a11	1,08
Baiap3	1,09	Fgf14	1,08	Ikzf3	0	Msx1	1,08
Actrt1	1,09	Hsd11b1	1,08	Ikzf2	0	Eps15	1,08

Prdx6b	1,09
Olfr109	1,09
Chek2	1,09
Arfgap1	1,09
Ccr6	1,09
4732456N10Rik	1,09
Slc22a2	1,09
Dcdc2c	1,09
Aga	1,09
St3gal1	1,09
Khnyn	1,09
Nkx2-4	1,08
Eif4e2	1,08
Cog6	1,08
Sap30l	1,08
Creb3	1,08
Olfr1512	1,08
Lrrc14	1,08
Olfm3	1,08
Rffl	1,08
Ccdc59	1,08
Fzd5	1,08
Tcp10c	1,08
Tox4	1,08
Zfp81	1,08
Hrct1	1,08
Olfr1297	1,08
BC048403	1,08
1110034G24Rik	1,08
Mau2	1,08
Golph3l	1,08
Lad1	1,08
Pdgfra	1,08
Lbhd1	1,08
Gsta2	1,08
Fbp1	1,08
Usp47	1,08
Olfr353	1,08
C1qtnf5	1,08
Emc10	1,08
Pde6a	1,08
Gpx5	1,08
Olfr572	1,08
Nek5	1,08
Fbxo28	1,08
Ak2	1,08
Lrrc16a	1,08
Gm8653	1,08
Olfr1441	1,08
Tardbp	1,07
Olfr794	1,07
Psmb9	1,07
Uts2r	1,07
Mocs2	1,07
Hdac8	1,07
Tubb4a	1,07
Prpf39	1,07
Bsn	1,07
BRDN0000738141	1,07
2310050C09Rik	1,07
Zfr	1,07
Inpp5d	1,07
Immp1l	1,07
Gast	1,07
2310033P09Rik	1,07
Hmgcl	1,07
Inhbe	1,07
Kcnq5	1,07
Krtap10-4	1,07
Olfr18	1,07
Ube2j2	1,07
Kifc3	1,07
Pgk2	1,07
Rgs20	1,07
Pdcd4	1,07
Lcat	1,07
Kbtbd7	1,07
Gmps	1,07
Gpr158	1,07
Map7d1	1,07
Ap1g2	1,07
BRDN0000738252	1,07
Pear1	1,08
Dclk1	1,08
Gid8	1,08
Akr1a1	1,08
Tph2	1,08
F830045P16Rik	1,08
Rell1	1,08
Rnf19a	1,08
Eapp	1,08
Hes6	1,08
Zfp534	1,08
BRDN0000737435	1,08
Gimap5	1,08
Mroh8	1,08
Klhl28	1,08
Hgh1	1,08
Chst11	1,08
Tsga13	1,08
Gorasp1	1,08
Prkar2b	1,08
Atp1a1	1,08
Xirp1	1,07
Capza2	1,07
Vmn1r78	1,07
Mast2	1,07
Setd5	1,07
Krt73	1,07
Gtf2h1	1,07
Gas7	1,07
Zfp54	1,07
Rnf130	1,07
Pla2g10	1,07
Teddm1a	1,07
Neurod6	1,07
1700001C19Rik	1,07
Aqp1	1,07
Ubtf	1,07
Vmn2r19	1,07
Slc38a11	1,07
Ccdc32	1,07
Cenpv	1,07
Thrap3	1,07
Csf1r	1,07
Plcd3	1,07
Zbtb10	1,07
Olfr410	1,07
Topaz1	1,07
Klhl42	1,07
Tnrc6c	1,07
Commd7	1,07
Cmtm8	1,07
Cdh8	1,07
Spta1	1,07
1110032F04Rik	1,07
Etaa1	1,07
Plcd4	1,07
Serpina12	1,07
Nos1ap	1,07
Gm16390	1,07
Iqcd	1,07
Drd5	1,07
Tmem40	1,07
Slc30a5	1,07
Car13	1,07
4933412E24Rik	1,07
Gpsm1	1,07
Wt1	1,07
Ak4	1,06
BC055111	1,06
Zfp943	1,06
Rpap2	1,06
Ccdc54	1,06
Nthl1	1,06
Ccnl1	1,06
Ythdc2	1,06
Serpine1	1,06
Ndufb11	1,06
Sowahb	1,06
4921504E06Rik	1,06
Srd5a2	1,06
Tex12	1,06
Ccdc117	1,06
H2afv	0
Dmrta1	0
Magee1	0
Gm11127	0
Mbtd1	0
H2afx	0
H2afy	0
H2afz	0
Krtap6-2	0
4930412D23Rik	0
Scn1b	0
Krtap6-1	0
Scn1a	0
BRDN0000738062	0
Igsf9b	0
Htr5b	0
Cdr1	0
Pigyl	0
Ciz1	0
Retsat	0
Cnn3	0
Cnn2	0
Hadhb	0
Hadha	0
Sept10	0
Fgf18	0
BRDN0000738063	0
Mgmt	0
Fgf13	0
Fgf12	0
Fgf11	0
Fgf10	0
Gm4064	0
Fgf16	0
Fgf15	0
Med20	0
Slc12a2	0
Ikbip	0
Nosip	0
Gm14744	0
Lmcd1	0
Abcb10	0
BRDN0000738064	0
Snrpd3	0
Muc15	0
Muc13	0
Angel2	0
Angel1	0
Btg2	0
Prss46	0
Olfr311	0
Prss44	0
Alox12e	0
Olfr314	0
Snrpd1	0
Prss40	0
Olfr317	0
Olfr318	0
Olfr319	0
Vmn2r124	0
Prss48	0
Tmco4	0
Olfr564	0
Nutm2	0
Phc2	0
Sec16a	0
Sec16b	0
E030002O03Rik	0
Gtf2h5	0
Peg3	0
Ccdc91	0
Uox	0
Zwint	0
1700001O22Rik	0
BRDN0000738066	0
Golt1b	0
Golt1a	0
Scgb2b15	0
Chil4	0
Cystm1	0
Med25	0
Csnk2a2	0
Atp13a5	1,08
Stk32c	1,08
Prl2a1	1,08
Slco1a1	1,08
1700015E13Rik	1,08
Smg1	1,08
Olfr1148	1,08
Tmem231	1,08
Samt2	1,07
Syt9	1,07
Igsf6	1,07
Olfr910	1,07
Prss39	1,07
Cyp2c66	1,07
Acin1	1,07
Vmn2r5	1,07
Dcdc2a	1,07
Slc9a3	1,07
Uap1l1	1,07
Prss53	1,07
Hoxa7	1,07
Trmt6	1,07
Il11	1,07
A630033H20Rik	1,07
Fam168b	1,07
Zfp804b	1,07
Neurog2	1,07
Fam71d	1,07
Nme2	1,07
Slc15a5	1,07
Mettl6	1,07
Rnf128	1,07
Aass	1,07
Myoz2	1,07
Rpl35	1,07
Chst7	1,07
Rgs20	1,07
Trib1	1,07
Gdap1l1	1,07
Pip4k2b	1,07
1700019L03Rik	1,07
Dlst	1,07
Tas2r119	1,07
Defb9	1,07
Ptprz1	1,07
Il23a	1,07
Pmaip1	1,07
8430419L09Rik	1,07
Mmd2	1,07
Dstn	1,07
Gatsl3	1,07
Lhpp	1,06
BRDN0000737538	1,06
Kif19a	1,06
Micu2	1,06
Mocos	1,06
Csnk1a1	1,06
Slpi	1,06
Mettl21a	1,06
Cmbl	1,06
Ccdc23	1,06
Snx27	1,06
Gadl1	1,06
St8sia6	1,06
Tmem115	1,06
Fam179a	1,06
Pacsin1	1,06
Trex1	1,06
4930590J08Rik	1,06
Rasgrf1	1,06
Vmn1r215	1,06
Olfr1002	1,06
Vmn1r32	1,06
Abra	1,06
0610010F05Rik	1,06
Tbc1d22b	1,06
Olfr116	1,06
Ndp	1,06
Nckap1l	1,06
Olfr121	1,06
Fam149b	1,06
Pomgnt1	1,06

Olig1	1,07	Pacs1	1,06	Csnk2a1	0	Olfr724	1,06
Bcl2l2	1,07	G6pc	1,06	Prl7c1	0	Clcnka	1,06
Grxcr1	1,07	E130309D14Rik	1,06	Sgol2a	0	Olfr1277	1,06
Klhl40	1,06	Zfp740	1,06	Dkk4	0	Nans	1,06
Fam26d	1,06	Paqr3	1,06	Pif1	0	Pkdrej	1,06
Slc25a48	1,06	Bcor	1,06	Ier3ip1	0	BRDN0000738088	1,06
Kmo	1,06	Heca	1,06	Dkk1	0	Yeats2	1,06
Ablim2	1,06	BRDN0000737487	1,06	Spint1	0	Hmgcs2	1,06
Chd4	1,06	Ear10	1,06	Terf2ip	0	S100a14	1,06
Bmper	1,06	Sass6	1,06	Rnf8	0	Fpr1	1,06
BRDN0000737397	1,06	4930523C07Rik	1,06	Rnf6	0	4933402P03Rik	1,06
Kdm1b	1,06	Zfhx3	1,06	Rnf7	0	Kpna6	1,06
Mrpl34	1,06	Olfr826	1,06	Med24	0	Pltp	1,05
C130050O18Rik	1,06	Ctdspl2	1,06	Rnf5	0	Rab5b	1,05
Diras1	1,06	Fbxl4	1,06	Chmp7	0	Tmem69	1,05
Synpr	1,06	Antxrl	1,06	Gje1	0	Mllt1	1,05
BRDN0000738038	1,06	Rhox2c	1,06	Zswim1	0	Lonrf1	1,05
Pgm2	1,06	Rad54l	1,06	Recql5	0	2700049A03Rik	1,05
BRDN0000737893	1,06	Zfp820	1,06	Recql4	0	Kcnk3	1,05
Arfgap3	1,06	BRDN0000738200	1,06	Sclt1	0	Zc2hc1a	1,05
Rdm1	1,06	Kdm6b	1,06	H2-D1	0	D130040H23Rik	1,05
Tmem194b	1,06	Mmp15	1,06	Gcnt1	0	Vmn1r137	1,05
Hist1h3d	1,06	Lipg	1,06	Olfr127	0	Melk	1,05
Mad2l1bp	1,06	Tbc1d23	1,06	Gcnt3	0	Pi4k2b	1,05
Serpinb5	1,06	Ankrd26	1,06	Paqr8	0	Mgl2	1,05
Zcchc2	1,06	Reps2	1,06	Ggt1	0	Pcnp	1,05
Wbp4	1,06	Tfap2d	1,06	Ubac1	0	Gstk1	1,05
Acot13	1,06	Apba2	1,06	Naif1	0	Igf1	1,05
Pdxp	1,06	Hspbap1	1,06	Mark4	0	Mrvi1	1,05
Art4	1,06	Kcna1	1,06	Als2cr11	0	Nfe2l2	1,05
Obox5	1,06	Itpripl1	1,06	Als2cr12	0	Tmem87a	1,05
Slamf8	1,06	Rab39b	1,06	Mark1	0	Hmbox1	1,05
Tmem248	1,06	Mettl21b	1,06	Mark3	0	Olfr716	1,05
Sox11	1,06	Klrb1f	1,06	Erp27	0	Astn2	1,05
Slc18a1	1,06	BRDN0000737883	1,06	Slc12a9	0	Mrgprf	1,05
Abcc5	1,06	Vmn2r61	1,06	Strip1	0	Fam102b	1,05
Pgm3	1,06	Dmrta2	1,05	Strip2	0	Trim47	1,05
Fabp3	1,06	Ssfa2	1,05	Ndrg3	0	Olfr747	1,05
Tinagl1	1,06	6030458C11Rik	1,05	Nmd3	0	Patl2	1,05
Eya3	1,06	Tppp3	1,05	Extl1	0	Etv4	1,05
Smim18	1,06	Try4	1,05	Extl2	0	Lrrc71	1,05
Zkscan3	1,05	Mex3b	1,05	Tnp1	0	Rictor	1,05
D8Ertd82e	1,05	Nanp	1,05	Myo10	0	Igll1	1,05
Ing2	1,05	BRDN0000737609	1,05	Mak16	0	Tmem110	1,05
Lnp	1,05	Olfr525	1,05	Ankrd10	0	Bin2	1,05
Olfr699	1,05	Akt1s1	1,05	F13b	0	Uqcrh	1,05
Gpd1l	1,05	Kcnj1	1,05	Tmem163	0	Tab2	1,05
Klhl32	1,05	Tmem55a	1,05	9330182O14Rik	0	Amz2	1,05
Pank1	1,05	Tagln	1,05	Tmem165	0	Aktip	1,05
Zfp91	1,05	Olfr1287	1,05	Tmem164	0	Glrp1	1,05
Ppp2r3c	1,05	Sltm	1,05	Tmem167	0	Trim32	1,05
Clock	1,05	Olfr612	1,05	Tmem169	0	Ckm	1,05
Olfr1157	1,05	Dleu7	1,05	Tmem168	0	Ndc1	1,05
Prm3	1,05	Klk1b24	1,05	Pspc1	0	Mrgprb5	1,05
Amy1	1,05	Rbms3	1,05	Gm14347	0	Plscr4	1,05
Rab39b	1,05	Lrcol1	1,05	Tbc1d8	0	Nlrp4b	1,05
Esp34	1,05	Olfr1097	1,05	Tbc1d9	0	4930412D23Rik	1,04
Casd1	1,05	Slfn2	1,05	Myg1	0	Figf	1,04
Itpr1	1,05	Fam57b	1,05	Sprr2e	0	Trim12a	1,04
Sval3	1,05	Hpca	1,05	Tbc1d1	0	Alad	1,04
Olfr12	1,05	Mid1ip1	1,05	Tvp23a	0	Ctso	1,04
Cmip	1,05	Gsdmc4	1,05	Tbc1d4	0	Cyp17a1	1,04
Akr1cl	1,05	Exph5	1,05	Tbc1d5	0	Ralb	1,04
Akap11	1,05	Bsdc1	1,05	Tbc1d7	0	Aspm	1,04
Dpysl5	1,05	Acaa2	1,05	Paqr7	0	Rdh14	1,04
Zfp777	1,05	Ugt3a1	1,05	BRDN0000737944	0	Tdrd1	1,04
Wdr90	1,05	Higd1c	1,05	Myo5b	0	Sdsl	1,04
Rbl2	1,05	Soat2	1,05	Spata45	0	Cd80	1,04
Hist1h2ba	1,05	Fcrl1	1,05	Arl5c	0	Olfr325	1,04
1110065P20Rik	1,05	Olfr1501	1,05	Ddx28	0	Secisbp2	1,04
Scara5	1,05	Dnmt3l	1,05	Itgax	0	Pcdhga9	1,04
Ubxn4	1,05	Rhox4b	1,05	Gm10104	0	Polr2d	1,04
Ulbp1	1,05	Cd302	1,05	Hnrnpdl	0	Vmn2r117	1,04
Degs2	1,05	Abca1	1,05	Sdccag8	0	Celsr2	1,04
Ubap2	1,05	Ak8	1,05	Ryr1	0	Map3k14	1,04
Cnnm4	1,05	Slc13a2	1,05	Gm20822	0	Dpf1	1,04
Olfr135	1,05	Dcaf4	1,05	0610009B22Rik	0	Nphs2	1,04
Lats1	1,05	Carkd	1,05	BRDN0000737888	0	4930474N05Rik	1,04
Lhpp	1,05	Dmrta1	1,05	Sdccag3	0	Stac	1,04
Lmbr1l	1,05	Slc25a38	1,05	Itgav	0	Gsg1	1,04
Emx2	1,05	Kbtbd4	1,05	Xpnpep1	0	Nabp1	1,04
Dnaic2	1,05	Slc36a3	1,05	BC031181	0	Irs1	1,04

Apom	1,05	Dapp1	1,05	Sae1	0	Ddx50	1,04
Btn2a2	1,05	Chil3	1,04	Ank3	0	Olfr1411	1,04
Phyhipl	1,05	Crisp3	1,04	Itgam	0	Dapk2	1,04
Mbl2	1,05	3830417A13Rik	1,04	BRDN0000738017	0	Vmn1r213	1,04
Vps72	1,04	Snrpb2	1,04	Itfg2	0	Sppl3	1,04
Pdgfc	1,04	Gypa	1,04	Itgae	0	Tm4sf5	1,04
Foxred1	1,04	1700010B08Rik	1,04	Ate1	0	Frmpd4	1,04
Smarcd2	1,04	Slc30a9	1,04	Gtf2e1	0	Tmprss12	1,04
Aldh1a2	1,04	Tssk4	1,04	Eif4e3	0	Tm7sf3	1,04
Pde1b	1,04	Saraf	1,04	Wls	0	Ephb6	1,04
Whrn	1,04	Sned1	1,04	Faxc	0	Tcfl5	1,04
Tmem120b	1,04	Slc39a1	1,04	Akirin2	0	Ppp4r1	1,04
Ifrd1	1,04	Alpk2	1,04	Akirin1	0	Pkp4	1,04
Hk1	1,04	Spag6	1,04	Rab3il1	0	Rltpr	1,04
Mmel1	1,04	Tmem263	1,04	Dennd4c	0	Esrra	1,04
Mical1	1,04	Mid1	1,04	Ap3m1	0	Olfr1361	1,04
Fam46a	1,04	Lhx8	1,04	Crhbp	0	H2-DMb1	1,04
Med9	1,04	Kctd15	1,04	Cd36	0	Serpinb3d	1,04
Slc1a1	1,04	Epb4.1l4a	1,04	Cul5	0	BRDN0000737428	1,04
Alg10b	1,04	Asgr1	1,04	Limd2	0	Depdc5	1,04
Ahrr	1,04	Rpa2	1,04	Limd1	0	Pah	1,04
Prom1	1,04	Slc39a14	1,04	Sgk2	0	Vmn1r18	1,04
Got1l1	1,04	Pms1	1,04	Itga8	0	Thsd1	1,04
Krt8	1,04	Rgs1	1,04	Itga9	0	D16Ertd472e	1,04
Stxbp6	1,04	Peg3	1,04	Olfr1329	0	Vmn1r128	1,04
Kcnk12	1,04	Patl1	1,04	Npsr1	0	Apoa5	1,04
Olfr1014	1,04	Olfr811	1,04	Orm2	0	Gjc3	1,04
Zfp821	1,04	Rgl3	1,04	Fancd2os	0	Mfap3l	1,04
Spink14	1,04	4930555G01Rik	1,04	Klhl42	0	Upp2	1,04
Zeb1	1,03	Rab33b	1,04	Olfr1323	0	Lax1	1,04
Lrrn3	1,03	Tjap1	1,04	Olfr1322	0	Acp6	1,03
Phc2	1,03	BRDN0000737512	1,04	Ptger1	0	Dnah9	1,03
Fxyd7	1,03	Cacng8	1,04	Itga6	0	Alox8	1,03
Pms1	1,03	Olfr1270	1,04	Cul1	0	Tnfsf14	1,03
Grm1	1,03	Dguok	1,04	Erp29	0	1700112E06Rik	1,03
Trpc7	1,03	BRDN0000738131	1,04	Zfp865	0	Etohi1	1,03
Neurod4	1,03	Il21r	1,04	Zfp866	0	Gad2	1,03
Epha7	1,03	Ppp1r12c	1,04	Zfp867	0	Maml3	1,03
Cep120	1,03	Setd1b	1,04	Cul2	0	Zswim4	1,03
Slc29a3	1,03	5730409E04Rik	1,04	Cyp1a1	0	Cass4	1,03
Filip1l	1,03	Dgat2	1,04	Cdk13	0	Btn1a1	1,03
Slc28a3	1,03	Crebl2	1,04	Flii	0	Foxa2	1,03
Trappc5	1,03	Gvin1	1,04	Noxred1	0	Pla2g4e	1,03
2010106E10Rik	1,03	Drosha	1,04	Kcnip4	0	Gpr15	1,03
Prss35	1,03	Ndufa5	1,04	Zfp868	0	Dusp6	1,03
Ctsd	1,03	Znrd1as	1,04	Zfp869	0	Tprkb	1,03
Zcchc17	1,03	Tpbg	1,04	Kcnip1	0	Smap2	1,03
Zcchc16	1,03	Olfr447	1,03	Zfp738	0	Otud4	1,03
Fli1	1,03	Ikzf5	1,03	Tpcn1	0	Olfr1469	1,03
Gabra2	1,03	Depdc7	1,03	Tpcn2	0	Rpf1	1,03
Ltk	1,03	Rpusd2	1,03	Taar4	0	Crygf	1,03
Bclaf1	1,03	Tep1	1,03	Zfp735	0	Gbas	1,03
Ihh	1,03	Suco	1,03	Cela2a	0	Epha8	1,03
Sema4d	1,03	4931423N10Rik	1,03	BRDN0000738320	0	Mrps12	1,03
Cenpq	1,03	Slc12a5	1,03	Prl8a2	0	Aimp2	1,03
Mob3c	1,03	Hgd	1,03	Stk19	0	H1fnt	1,03
Nell1	1,03	Hspa2	1,03	Schip1	0	Fam132b	1,03
Cxcl3	1,03	Myf6	1,03	Cdk14	0	Cd101	1,03
Scn8a	1,03	Olfr483	1,03	Taar1	0	Tle2	1,03
Prg3	1,03	Hddc2	1,03	Sorbs3	0	Lhx1	1,03
Slc25a28	1,03	Gm4297	1,03	Mup6	0	Ctsw	1,03
Robo4	1,03	Lrrc48	1,03	Olfr889	0	Vmn1r181	1,03
Mup20	1,03	Gabra3	1,03	Mup4	0	Rad51b	1,03
Tcap	1,03	Taco1	1,03	Snrpb2	0	Slc13a2	1,03
Twf1	1,03	Ptgr2	1,03	Fam208a	0	Ramp2	1,03
Syngr1	1,03	Clns1a	1,03	4930539E08Rik	0	Smim13	1,03
Aspn	1,03	Mtch1	1,03	Ltbp1	0	Ralgps1	1,03
Lrp2bp	1,03	Wwp2	1,03	Fam208b	0	Bsn	1,03
Tspan7	1,03	Slc24a3	1,03	Svs6	0	Olfr1383	1,03
Zfp111	1,03	Rnf146	1,03	Ptger3	0	Ceacam14	1,03
2410137M14Rik	1,03	Ndufaf5	1,03	Taar2	0	Tra2a	1,03
Mme	1,02	Olfr281	1,03	Olfr884	0	Dclk1	1,03
Gpr89	1,02	Olfr1446	1,03	Olfr885	0	3110082J24Rik	1,03
Eef1g	1,02	Htt	1,03	Mup8	0	Foxb2	1,03
Ccdc85b	1,02	Uba3	1,03	Mup9	0	Ccr4	1,03
Cd209b	1,02	Tssk2	1,03	Prol1	0	Ccdc83	1,03
Hsd3b5	1,02	Cib4	1,03	BRDN0000737583	0	C8g	1,03
Arrdc4	1,02	Zfp185	1,03	Olfr911-ps1	0	Ankrd13b	1,03
Fignl1	1,02	Khnyn	1,03	Olfr1410	0	Folh1	1,03
Ar	1,02	Gramd1a	1,03	Calr	0	Scn5a	1,03
Olfm2	1,02	Zbtb8a	1,03	M6pr	0	Adck5	1,03
Fmo9	1,02	Htr2a	1,03	Flot1	0	Gm4175	1,03

Rgs21	1,02	Dnph1	1,03	Neurod1	0	Olfr1387	1,02
Nol3	1,02	Adk	1,03	Neurod6	0	Pfkfb4	1,02
Srfbp1	1,02	Gal3st1	1,03	Neurod4	0	Efna4	1,02
Fgf9	1,02	Traf3ip1	1,03	Aspdh	0	Reep6	1,02
Olfr1537	1,02	Olfr1213	1,03	Taar9	0	Dlgap4	1,02
Camk1	1,02	Olfr821	1,03	Zfp14	0	Ccdc88a	1,02
Cnot3	1,02	Avpr1b	1,03	Zfp12	0	Pde12	1,02
Paox	1,02	Fancd2	1,03	Scrg1	0	Olfr1446	1,02
Vdac3	1,02	Zfp879	1,03	Krt42	0	Enpp4	1,02
Olfr1084	1,02	Prdx2	1,03	Zfp11	0	Hdgf	1,02
Stx18	1,02	Abtb2	1,03	Ebf4	0	Olfr482	1,02
Rsph6a	1,02	BRDN0000737651	1,03	Ebf3	0	Unc5c	1,02
BRDN0000738215	1,02	Nos1	1,03	Ebf2	0	Olfr464	1,02
Vmn1r128	1,02	Spata4	1,03	Ebf1	0	Ldb2	1,02
Ap4s1	1,02	Golph3	1,03	Klrc2	0	4921501E09Rik	1,02
Nmral1	1,02	Donson	1,03	R3hcc1l	0	Cdr2l	1,02
Fbln1	1,02	Dok3	1,03	Ndufs2	0	Cadm3	1,02
Rab3b	1,02	Vmn1r188	1,03	BRDN0000737882	0	Atp11b	1,02
Nsun4	1,02	Nanog	1,03	Wbp2nl	0	2810021J22Rik	1,02
BC004004	1,02	Prl8a2	1,03	Gm7102	0	Vmn2r54	1,02
Pus7l	1,02	Phldb1	1,03	Mbp	0	Cnpy2	1,02
Dhx34	1,02	Ifi27l2a	1,02	Mfrp	0	Nelfb	1,02
Sp8	1,02	Trpv2	1,02	Sox1	0	Trex2	1,02
Col6a2	1,02	Zfp111	1,02	Sparc	0	Mb21d1	1,02
Pnma1	1,02	Fam71e2	1,02	Zfp787	0	Cyb5r1	1,02
3830406C13Rik	1,02	Tssc1	1,02	Cxx1a	0	Pcdh12	1,02
Tmem136	1,02	Olfr93	1,02	Gon4l	0	Metrnl	1,02
Olfr955	1,02	Osbpl11	1,02	Cxx1c	0	Kat6b	1,02
BRDN0000737616	1,02	Zdbf2	1,02	Cxx1b	0	Pgk2	1,02
Rhbdd3	1,02	Avil	1,02	2610015P09Rik	0	Scamp3	1,02
Lrrc18	1,01	Pcmtd2	1,02	Ndufs3	0	Ccdc78	1,02
Gm8994	1,01	Rasgrp3	1,02	BRDN0000737881	0	Fam216a	1,02
Dcc	1,01	Slc7a1	1,02	Cacna1i	0	Nr1d1	1,02
Olfr50	1,01	Hcn2	1,02	Tshz1	0	0610030E20Rik	1,02
Hsd3b2	1,01	Gm17019	1,02	Calhm1	0	Pih1d1	1,02
Mtpn	1,01	Lrfn1	1,02	Ctdspl2	0	Ppp1ca	1,02
Olfr473	1,01	Cecr6	1,02	Cacna1a	0	BRDN0000737952	1,02
Tnnt3	1,01	Cep95	1,02	Cacna1b	0	BRDN0000737419	1,02
Mpv17l2	1,01	Gm14305	1,02	Cacna1d	0	Wbscr27	1,02
Coq5	1,01	Psmb1	1,02	Kif2b	0	Csrnp1	1,02
Hapln1	1,01	Fblim1	1,02	Lmln	0	Egr1	1,02
Sugp1	1,01	Ndufa11	1,02	C2cd4a	0	Ppp1r1a	1,01
Tatdn3	1,01	Btd	1,02	Ms4a4b	0	Hsph1	1,01
Apol11b	1,01	Hook1	1,02	Rtel1	0	Hist1h2bb	1,01
Gltscr1l	1,01	Speer2	1,02	Fam46c	0	Cyp26c1	1,01
Trip6	1,01	Klhl29	1,02	Pltp	0	Cer1	1,01
Prr9	1,01	Setd3	1,02	Gng12	0	BC051019	1,01
Itgb1	1,01	Olfr1484	1,02	Ostf1	0	Tas2r118	1,01
H2-M10.4	1,01	Psme3	1,02	Cmc1	0	Tspan9	1,01
Lgr4	1,01	Tbc1d10b	1,02	Abcg4	0	Ppip5k1	1,01
Pacs2	1,01	Gm11128	1,02	Cacna1s	0	Wfikkn1	1,01
Vmn2r12	1,01	AA467197	1,02	BRDN0000737880	0	Tmem138	1,01
Olfr390	1,01	BRDN0000737417	1,02	Gcsh	0	Timm8a2	1,01
Gripap1	1,01	Nmnat2	1,02	Kcne2	0	F13b	1,01
Qpct	1,01	Igfbp3	1,02	Kcne3	0	Gm3259	1,01
Gm20815	1,01	Prss36	1,02	Rasal2	0	Chl1	1,01
Oog4	1,01	Vmn1r125	1,02	Abi1	0	Sft2d3	1,01
Vmn1r233	1,01	Slc16a4	1,02	Kcne4	0	Wnk2	1,01
Thumpd3	1,01	Foxn4	1,02	Cplx3	0	Kcnj1	1,01
Epcam	1,01	Mroh9	1,02	Lrwd1	0	Gm16381	1,01
Spint4	1,01	2700089E24Rik	1,02	BRDN0000738041	0	Rassf6	1,01
Ttc39b	1,01	Atg9a	1,02	Dopey2	0	Zfp385b	1,01
Vmn1r90	1,01	Sdsl	1,02	Dopey1	0	Tas2r109	1,01
Tmem50b	1,01	Chsy1	1,02	Alox5	0	Eogt	1,01
Cmtm7	1,01	Dip2a	1,02	Shroom3	0	Pnpla1	1,01
Il20	1,01	Ms4a10	1,02	Shroom2	0	Csnk1g2	1,01
Olfr456	1,01	Sgsh	1,02	Shroom1	0	4921504E06Rik	1,01
Tmem132a	1,01	Atp7a	1,02	Arhgap8	0	Olfr1182	1,01
Sftpc	1,01	Cnnm3	1,02	Xrn1	0	Serpina5	1,01
Gmpr	1,01	Stk36	1,02	Xrn2	0	Prph2	1,01
Olfr554	1,01	Ndufs3	1,02	Crnn	0	Mogat2	1,01
Tmem104	1,01	Efcab14	1,02	Arhgap4	0	Pus7l	1,01
BRDN0000738326	1,01	9230110C19Rik	1,02	Pot1b	0	Matk	1,01
Foxd4	1,01	Ubash3a	1,02	Pde11a	0	Mbd3l1	1,01
Olfr1124	1,01	Flt3l	1,02	Eefsec	0	D430041D05Rik	1,01
Dsg4	1,01	Xylt2	1,01	Gm17689	0	Vkorc1l1	1,01
Hsdl2	1,01	Jag1	1,01	Ajap1	0	Zfp959	1,01
Pvrl3	1,01	Vps13b	1,01	BRDN0000738101	0	Thsd4	1,01
She	1,01	Klrb1b	1,01	Pgap3	0	Psg19	1,01
Nkain4	1,01	Olfr487	1,01	Ap5z1	0	Myh2	1,01
Sox30	1,01	Mtpap	1,01	Mars2	0	Snapc1	1,01
Ipo4	1,01	Dhx30	1,01	Col19a1	0	Dpys	1,01

Ptgs1	1,01	Sowahc	1,01	Dynlrb2	0	BRDN0000737521	1,01
Bves	1,01	Thada	1,01	BRDN0000738147	0	S1pr3	1,01
Timm17a	1,01	Dgkd	1,01	Dynlrb1	0	Batf3	1,00
Bpifb9a	1,00	Rad52	1,01	Gm1979	0	Pacrg	1,00
Hdac6	1,00	2610002M06Rik	1,01	Klhdc4	0	Cubn	1,00
Apbb1ip	1,00	Olfr592	1,01	Rpusd4	0	Schip1	1,00
Fmn1	1,00	Gnaz	1,01	Tdrkh	0	H2-M11	1,00
Snrpc	1,00	Cd300ld	1,01	Col11a2	0	Olfr1206	1,00
Cdc5l	1,00	Slc35e1	1,01	Fam43a	0	Olfr193	1,00
Slc38a4	1,00	Drd4	1,01	BRDN0000738121	0	Mrpl20	1,00
Sult1d1	1,00	Tmem33	1,01	Pars2	0	Zbtb32	1,00
Ifnab	1,00	Cdkn2d	1,01	Klra8	0	Psg18	1,00
Ubald1	1,00	Prdm5	1,01	Vmn1r185	0	Rimkla	1,00
Cbr1	1,00	Cxx1b	1,01	Gnai2	0	Xrcc2	1,00
Sh3gl1	1,00	Lrrc23	1,01	Btbd11	0	Syt13	1,00
Manf	1,00	Tmem254c	1,01	Gdpd5	0	Itgb1	1,00
Nfam1	1,00	Qdpr	1,01	Vmn1r184	0	Olfr954	1,00
Stard3nl	1,00	Actc1	1,01	Tiparp	0	BRDN0000737768	1,00
Clec4a3	1,00	Ss18l1	1,01	Ndufs7	0	Olfr974	1,00
Rspo2	1,00	Zfp281	1,01	Dync1li2	0	Ap2b1	1,00
Cdc42ep3	1,00	B4galnt2	1,01	Neu3	0	Fbxl8	1,00
1700042G07Rik	1,00	Ankrd11	1,01	BRDN0000737836	0	Slc9b2	1,00
Snx27	1,00	Papl	1,01	BRDN0000737837	0	Olfr402	1,00
Kti12	1,00	Gpr149	1,01	BRDN0000737834	0	Osbp2	1,00
Tstd1	1,00	Angel2	1,01	BRDN0000737835	0	Ptbp2	1,00
Pvalb	1,00	Manba	1,01	BRDN0000737832	0	Olfr1046	1,00
Cma1	1,00	1810055G02Rik	1,01	BRDN0000737833	0	Txnrd3	1,00
Hsp90aa1	1,00	Ltbp4	1,01	C130026I21Rik	0	Grm2	1,00
Rassf6	1,00	Spata16	1,01	Ptprb	0	Ugt2b1	1,00
Stk33	1,00	1700001L19Rik	1,01	Utp6	0	AU018091	1,00
Olfr867	1,00	Ces5a	1,01	Ctso	0	Sema7a	1,00
Tuba4a	1,00	Mtmr12	1,01	Atic	0	Ctdspl	1,00
Impg1	1,00	Rnf152	1,01	Mtch2	0	Hmox1	1,00
Ccdc163	1,00	Klk1b27	1,01	Mtch1	0	Inafm1	1,00
Tmem214	1,00	Amd2	1,01	Lrif1	0	Olfr681	1,00
Tll1	1,00	Irx5	1,01	Hmx3	0	Rnmt	1,00
Coa7	1,00	Galnt3	1,01	Kdm4d	0	Ndufaf4	1,00
Cox18	1,00	Cby1	1,01	Gnai3	0	Pik3r4	1,00
Olfr1112	1,00	Bnip1	1,01	Ankrd27	0	Hsd17b1	1,00
Cdh20	1,00	Atg10	1,01	Ankrd26	0	Gfra4	1,00
Ankra2	1,00	1700006E09Rik	1,01	Ankrd24	0	Man2a1	1,00
Olfr55	1,00	Slc38a7	1,01	Ankrd23	0	Gpr4	1,00
Zfp810	1,00	Gns	1,01	Ankrd22	0	Hdac2	1,00
Hnf1a	1,00	Acan	1,01	Tial1	0	Olr1	1,00
Mfap3l	1,00	Ccpg1os	1,01	Glis1	0	Itpkc	1,00
Cyp2w1	1,00	Mmp16	1,01	Cnot3	0	Alms1	1,00
Rhbdd1	1,00	Zfp598	1,01	Cnot2	0	Rdh13	1,00
Hal	1,00	Fkbp10	1,01	Cnot1	0	Mypn	1,00
Ndufs1	0,99	Rbfox3	1,01	Ghsr	0	Myo10	1,00
Sh2d3c	0,99	D5Ertd577e	1,01	Cnot7	0	Olfr1368	1,00
Il17rb	0,99	Sox4	1,01	Cnot6	0	Olfr1240	1,00
Fam89b	0,99	Tcf7l2	1,01	Ankrd29	0	Csn2	1,00
Olfr251	0,99	Hip1r	1,01	Cnot4	0	Lst1	1,00
Ppp2r2c	0,99	Bmp6	1,01	Sars2	0	Pcp4l1	1,00
Idh3a	0,99	Mbtd1	1,01	Ptf1a	0	Gpr88	1,00
Papolg	0,99	Samd4	1,01	Oscar	0	Agfg1	1,00
Zmym5	0,99	Olfr263	1,01	Uvrag	0	Baat	0,99
Tead3	0,99	Eif2s3y	1,01	2810021J22Rik	0	Mpzl3	0,99
March8	0,99	Klhdc7a	1,01	Ttc3	0	Rnmtl1	0,99
Hbs1l	0,99	Olfr472	1,00	Cldn6	0	Rab18	0,99
Nox3	0,99	Etv3	1,00	Rnf103	0	Olfr212	0,99
Acox3	0,99	Zfp524	1,00	Foxi1	0	Vax2	0,99
Skint3	0,99	Map2k7	1,00	Foxi3	0	Rps6kc1	0,99
Rpap3	0,99	Chn2	1,00	Vmn1r188	0	Wdr64	0,99
Adam23	0,99	Safb	1,00	Rars2	0	Casd1	0,99
Olfr944	0,99	Vmn2r83	1,00	Lgr5	0	Akap14	0,99
Abca3	0,99	Lzic	1,00	Lgr4	0	Sphkap	0,99
Aldh3a2	0,99	Dnajb5	1,00	Lgr6	0	Dyrk1b	0,99
Cpeb4	0,99	Atrn	1,00	Ncan	0	Mapk13	0,99
Olfr1442	0,99	Hoxd10	1,00	Nynrin	0	Colq	0,99
Btnl2	0,99	Olfr1453	1,00	Ttf2	0	Mri1	0,99
Ccdc167	0,99	Gimap3	1,00	Ttf1	0	Stmn1	0,99
Ctdspl2	0,99	Olfr1116	1,00	Cxcr3	0	Amy2a3	0,99
Klra2	0,99	Vtn	1,00	Cxcr2	0	Mlh1	0,99
Tia1	0,99	Zmym2	1,00	Cxcr1	0	Ifi205	0,99
Smr2	0,99	Ccdc171	1,00	Insm1	0	Gm2863	0,99
Tdo2	0,99	Olfr354	1,00	Cxcr5	0	Dguok	0,99
C78339	0,99	Alx1	1,00	Ttyh3	0	Slc22a5	0,99
Fetub	0,99	Brd4	1,00	Grasp	0	Plod1	0,99
Farp1	0,99	Hmgb2	1,00	Cyp2j11	0	Lrrc15	0,99
Gm3763	0,99	Cpt1a	1,00	Cyp2j12	0	Emc9	0,99
Ppp1r9a	0,99	Astl	1,00	Nfix	0	Ints1	0,99

4930452B06Rik	0,99	Nono	1,00	Fbxl20	0	Fabp9	0,99
Bmp2k	0,98	Ist1	1,00	Fbxl21	0	Srebf2	0,99
Rabl6	0,98	BRDN0000738094	1,00	Fbxl22	0	Tbx22	0,99
Msl1	0,98	Hcar2	1,00	Epdr1	0	Cftr	0,99
Il17rd	0,98	Srpk1	1,00	Acad8	0	Ssc5d	0,99
Mdfic	0,98	Hgsnat	1,00	Zfp292	0	Kdm3a	0,99
Mettl17	0,98	Nlrp14	1,00	Igsf8	0	Tlcd1	0,99
Sppl3	0,98	Fiz1	1,00	Igsf9	0	Acy1	0,99
7420461P10Rik	0,98	Rnase2b	1,00	Fem1c	0	Lrch2	0,99
Ddc	0,98	Tulp4	1,00	Wdtc1	0	BRDN0000737841	0,99
Npnt	0,98	Uchl3	1,00	Igsf5	0	Pcdhga3	0,99
Prm2	0,98	Fbxw4	1,00	Igsf6	0	Rbm15b	0,99
Ltbp4	0,98	Hspg2	1,00	Tmub2	0	Boll	0,99
Arl1	0,98	Opa3	1,00	Igsf1	0	Fam129c	0,99
C330018D20Rik	0,98	Sharpin	1,00	Igsf3	0	Fam160a1	0,99
Mapk4	0,98	2410127L17Rik	1,00	Tubb2b	0	Fn3k	0,99
BRDN0000738040	0,98	Hoxc4	1,00	Srms	0	Tob1	0,99
Top1mt	0,98	Armc12	1,00	Aldh16a1	0	Rasgef1c	0,99
Nudt19	0,98	Myct1	1,00	E130309D02Rik	0	Cd300a	0,99
Tmem9b	0,98	Ccdc138	1,00	Gm21693	0	Itfg3	0,99
Fam92b	0,98	Spata25	1,00	Coprs	0	Spag6l	0,99
C87414	0,98	Rhoj	1,00	C87436	0	Neurog1	0,99
Chmp3	0,98	Brsk1	1,00	Rem2	0	Kcnh1	0,99
Papd5	0,98	Nkiras1	1,00	Pkig	0	Olfr895	0,99
Rxrb	0,98	Stard3	0,99	Pkia	0	Cyp2a4	0,99
Pmvk	0,98	Cdc25c	0,99	Pkib	0	Gm527	0,99
Stat6	0,98	Lrrc75b	0,99	Rpl15	0	Dsg2	0,99
Slc2a6	0,98	Ly96	0,99	1810026J23Rik	0	Mmp9	0,99
Nckipsd	0,98	Csn2	0,99	Rpl17	0	Ucp1	0,99
Sox14	0,98	Dido1	0,99	Olfr1132	0	Cyth2	0,99
Uckl1	0,98	Rbbp6	0,99	Rpl11	0	Ankdd1b	0,99
Zfyve19	0,98	Slco6d1	0,99	Olfr1130	0	Eras	0,98
Cks1brt	0,97	Fgf23	0,99	Rpl13	0	Nova2	0,98
BRDN0000737437	0,97	Pof1b	0,99	Rpl18	0	Olfr1116	0,98
Hist1h2ae	0,97	1700021F07Rik	0,99	Rpl19	0	Lrrc74b	0,98
Tmem64	0,97	Syn1	0,99	Olfr1138	0	Mut	0,98
Vpreb3	0,97	Rnft1	0,99	Sun1	0	2700029M09Rik	0,98
Mfn2	0,97	BRDN0000737407	0,99	Ogfod1	0	Mark1	0,98
Spata31d1a	0,97	Usp24	0,99	Angpt1	0	Cyp2d22	0,98
9530068E07Rik	0,97	Kctd16	0,99	Ogfod3	0	Kcne4	0,98
Zswim8	0,97	Calhm1	0,99	Ogfod2	0	Dnah10	0,98
Aida	0,97	Timm8b	0,99	Trnt1	0	Khdc3	0,98
Thbs1	0,97	Tmprss3	0,99	Nudt10	0	Sftpc	0,98
Asic3	0,97	Arsj	0,99	Olfr504	0	D030056L22Rik	0,98
Naa11	0,97	Atoh8	0,99	Olfr507	0	Ifi27	0,98
Tbc1d16	0,97	Prl7d1	0,99	Olfr506	0	Sfxn5	0,98
Tagap1	0,97	Eif4e1b	0,99	Lyrm7	0	Snph	0,98
Mta2	0,97	Ar	0,99	Pskh1	0	Prrx2	0,98
Atp6v0a1	0,97	Ppapdc2	0,99	Olfr503	0	Myl6	0,98
BRDN0000737862	0,97	Bag1	0,99	Lnpep	0	Braf	0,98
Gm4559	0,97	Ppp2r5c	0,99	Nudt16	0	Lrrfip2	0,98
Atp2c1	0,97	Tnip2	0,99	Lyrm9	0	BRDN0000738207	0,98
Zfp27	0,97	BC003965	0,99	Folh1	0	Fam53c	0,98
Sigirr	0,97	Plcb2	0,99	Olfr509	0	Ate1	0,98
BRDN0000737486	0,97	Sprr2h	0,99	Olfr508	0	Pcdhac1	0,98
BRDN0000737978	0,97	Galnt11	0,99	Smtn	0	Bpifa6	0,98
Gm6760	0,97	Olfr608	0,99	Mphosph9	0	Krtap20-2	0,98
H1fnt	0,97	Mettl17	0,99	Gss	0	Plcxd2	0,98
Rab13	0,97	Ccnd2	0,99	Gsr	0	Defb29	0,98
Cyp3a25	0,97	Cpz	0,99	BRDN0000737788	0	Defb1	0,98
Vill	0,97	Hpse2	0,99	Cltc	0	Slc25a51	0,98
Pus3	0,97	Tsr3	0,99	Klf12	0	Sowahd	0,98
Prl8a6	0,97	Fgfr3	0,99	Ank	0	Rab11fip1	0,98
Acbd3	0,97	Apoa4	0,99	BRDN0000738024	0	Rybp	0,98
Olfr263	0,97	Pls1	0,99	Nudt15	0	Ppih	0,98
Mfrp	0,97	BRDN0000738222	0,99	Klf11	0	Megf6	0,98
Olfr1211	0,97	Utp18	0,99	Slco2a1	0	Hmgn3	0,98
Rnf215	0,96	Gm732	0,99	Acsm4	0	Plxdc2	0,98
Basp1	0,96	Sft2d1	0,99	Izumo1	0	Slc25a53	0,98
Dpysl2	0,96	Olfr243	0,99	Izumo2	0	Itgam	0,98
Bpifb3	0,96	Gimap1	0,99	Izumo3	0	Zfp617	0,98
Gpatch8	0,96	Pilrb2	0,99	Izumo4	0	Mag	0,98
Zfp871	0,96	Gpr132	0,99	BC048502	0	Mrpl12	0,98
Aldh7a1	0,96	Inpp5f	0,99	Lrfn4	0	Lor	0,98
Gm21379	0,96	Chrng	0,99	Patl2	0	Fam134a	0,98
Uck1	0,96	Nsg2	0,99	Nudt18	0	Aldh3a2	0,98
Vmn1r78	0,96	Eif4h	0,99	Pmfbp1	0	Olfr1109	0,98
Leap2	0,96	Rnf167	0,98	Tagln	0	Defb3	0,98
Akr1b10	0,96	Rybp	0,98	Acpt	0	Cenpq	0,98
Olfr433	0,96	Sdf2	0,98	Cnrip1	0	Ccdc105	0,98
Smad4	0,96	Ulk4	0,98	BRDN0000737980	0	Hspbap1	0,98
Fh1	0,96	BRDN0000738269	0,98	Palm3	0	Gm21002	0,98

Tmod3	0,96	Tmcc2	0,98	Wnt3	0	Zfp322a	0,97
Igf2bp2	0,96	Suv39h2	0,98	Wnt2	0	Bclaf1	0,97
Tmem29	0,96	Sphk2	0,98	BC049635	0	Efnb2	0,97
Fancm	0,96	Vmn1r25	0,98	Hk1	0	Fabp1	0,97
Slurp1	0,96	Eras	0,98	Unc80	0	Zbtb9	0,97
Gem	0,96	Olfr1121	0,98	Wnt4	0	Itga3	0,97
Cd200r3	0,96	Sirt5	0,98	Mob3b	0	Atrx	0,97
Rhof	0,96	Fbxw16	0,98	Mob3c	0	G6pc3	0,97
Iqcf6	0,96	Olfr1145	0,98	Mob3a	0	Ect2l	0,97
Smdt1	0,96	Tmem52	0,98	Sult2a6	0	Actbl2	0,97
Xkrx	0,96	1700013G24Rik	0,98	Sult2a7	0	Ptpn21	0,97
Cxcl12	0,96	Trnp1	0,98	Sult2a5	0	Ap1s3	0,97
Fn3k	0,96	Olfr2	0,98	Fsd1	0	Garnl3	0,97
Sgpp2	0,96	H2-Q2	0,98	Sult2a3	0	Drd2	0,97
Cyp2c39	0,96	Arhgap32	0,98	Sult2a1	0	Dolpp1	0,97
Lhx6	0,96	Grik4	0,98	BRDN0000738165	0	Pcdha3	0,97
D15Ertd621e	0,96	Mrpl33	0,98	Ppy	0	Bora	0,97
Fam192a	0,96	Mpped2	0,98	Gbp9	0	Adrm1	0,97
Pitx2	0,96	Cypt15	0,98	Gbp8	0	Olfr531	0,97
Plcd1	0,96	Limd1	0,98	Trpd52l3	0	Cdc42ep4	0,97
Pik3ip1	0,96	Tcf12	0,98	Grhl3	0	Scn4b	0,97
Nkx6-3	0,96	Slc16a5	0,98	Gbp3	0	Tex11	0,97
Nr0b2	0,96	Ten1	0,98	Gbp2	0	Dmwd	0,97
Itga3	0,96	Olfr214	0,98	Gbp7	0	Clca2	0,97
Adh5	0,96	Med30	0,98	Gbp6	0	Sptbn2	0,97
BRDN0000737915	0,96	Hist3h2ba	0,98	Gbp5	0	Rwdd1	0,97
Gm4861	0,96	Aknad1	0,98	Gbp4	0	Lhfpl2	0,97
Vmn1r9	0,96	Olfr613	0,98	Col6a1	0	Gli1	0,97
Nrip1	0,96	Gm13769	0,98	Defa5	0	Olfr183	0,97
Slc9a5	0,96	Zbtb49	0,98	Lsg1	0	Sqrdl	0,97
Brk1	0,96	Lipi	0,98	Fes	0	Ism1	0,97
Disc1	0,96	Itgbl1	0,98	Pms1	0	Podxl2	0,97
Zic3	0,95	Gm5464	0,98	Pms2	0	Ruvbl2	0,97
Cfl2	0,95	Nars2	0,98	Clcn1	0	Ano7	0,97
Zdhhc15	0,95	Pigk	0,98	Hhatl	0	Tulp3	0,97
Olfr490	0,95	Cfap53	0,98	BRDN0000738164	0	Cep164	0,97
Htr4	0,95	Xkr4	0,98	Asgr2	0	Jade1	0,97
Cyp2c37	0,95	Bpifb9a	0,98	Tmprss9	0	Map1b	0,97
Plcd4	0,95	Olfr1221	0,98	BRDN0000738264	0	Hivep2	0,97
Gm13275	0,95	Psmb9	0,97	Klkb1	0	1700016K19Rik	0,97
Nsdhl	0,95	BRDN0000738315	0,97	Col6a5	0	Epdr1	0,97
Serpinf1	0,95	Olfr1445	0,97	Tmprss2	0	Nf1	0,97
1110004F10Rik	0,95	Pde12	0,97	Necap2	0	Hist3h2ba	0,97
Ulk4	0,95	Ndufs5	0,97	Tmprss4	0	Timm10b	0,97
Kif2a	0,95	Olfr523	0,97	Tmprss5	0	Uts2b	0,97
Pcyt2	0,95	Ndufaf1	0,97	Tmprss6	0	Plekhd1	0,97
Mgme1	0,95	Olfr103	0,97	Tmprss7	0	Rhox2c	0,97
Ang2	0,95	Ube2w	0,97	Trhr	0	Fbxw26	0,97
Olfr243	0,95	Abcd3	0,97	Olfr750	0	Tcf23	0,97
Gm5168	0,95	Cst10	0,97	Rad18	0	Olfr552	0,97
Cdkl2	0,95	Tk2	0,97	Gpr3	0	Maneal	0,97
Ccr1	0,95	Prss2	0,97	BRDN0000737758	0	Gm4312	0,97
Klk1b11	0,95	Gm10436	0,97	Col6a6	0	Mtfr1	0,97
Serpinc1	0,95	Bola2	0,97	Uqcrc2	0	Plxnc1	0,97
Olfr1339	0,95	Dhrs7b	0,97	BRDN0000738268	0	B9d2	0,97
Galnt1	0,95	Pcm1	0,97	Tmed11	0	Defb26	0,97
Dennd2c	0,95	Mpz	0,97	Uqcrc1	0	Pear1	0,97
Klk1b5	0,95	Olfr1306	0,97	Dmxl2	0	Athl1	0,97
Strc	0,95	Retnla	0,97	Bcl2l14	0	Crtc1	0,97
Kcnab1	0,95	Lhx2	0,97	Fam71f1	0	Prickle3	0,97
Iqcb1	0,95	Klf2	0,97	Dmxl1	0	Ceacam20	0,97
Ccdc54	0,95	Pitpnm2	0,97	Bcl2l11	0	Fbxo11	0,97
Ptpn3	0,95	Vmn2r97	0,97	Bcl2l10	0	Olfr1350	0,96
Zdhhc9	0,95	Serpina1a	0,97	Bcl2l13	0	Mfn1	0,96
Krt81	0,95	Timp3	0,97	Olfr1209	0	Timm21	0,96
Uchl4	0,95	Vat1l	0,97	Tmprss3	0	Olfr1284	0,96
Zfp668	0,94	Zc3h12b	0,97	Tm4sf1	0	Twistnb	0,96
Shb	0,94	Sh3glb1	0,97	Tm4sf4	0	Wrn	0,96
Gabra6	0,94	Gm4763	0,97	Tm4sf5	0	Fbn1	0,96
Itih1	0,94	Ang5	0,97	Myh1	0	Olfr807	0,96
Cluh	0,94	Olfr935	0,97	Slc30a4	0	Cyp2j8	0,96
Myf6	0,94	Zbtb45	0,97	Nfatc2ip	0	Galntl5	0,96
Krt23	0,94	Prkcz	0,97	Eed	0	Kcnj2	0,96
9430015G10Rik	0,94	Clec4a4	0,97	Epb4.1l4a	0	Tmem145	0,96
Gkap1	0,94	Epn2	0,97	Tprg	0	Rasip1	0,96
Sumf2	0,94	Myo5b	0,97	Ifrd2	0	Man1a	0,96
Cept1	0,94	Grem2	0,97	Rrh	0	Olfr272	0,96
Tex43	0,94	Sypl2	0,97	Tprn	0	Phip	0,96
Meis2	0,94	Sema3e	0,97	Gm884	0	Dppa1	0,96
Zfp454	0,94	Zfp512	0,97	Chgb	0	Pcdh10	0,96
Efna1	0,94	Olfr1161	0,97	Mcu	0	Klhl9	0,96
Thg1l	0,94	Rtn4rl1	0,97	Cckar	0	Slc47a1	0,96

BRDN0000738299	0,94	Fam102b	0,97	3110082I17Rik	0	Rad52	0,96
Dlx2	0,94	Ppargc1b	0,97	Grin3a	0	Atg16l1	0,96
Fbln2	0,94	Vipr1	0,97	Eea1	0	Olfr818	0,96
Ssxb5	0,94	Blvrb	0,97	Ins2	0	Scgn	0,96
Nfkbiz	0,94	Cenpf	0,97	Atp1a4	0	Sash3	0,96
Il2	0,94	Allc	0,97	Amigo1	0	Snx4	0,96
Hdx	0,94	Emc8	0,97	Amigo3	0	Adam6a	0,96
Olfr235	0,94	Ubl3	0,97	Amigo2	0	Smg9	0,96
Oxct2a	0,94	Krtap31-2	0,97	Xrcc2	0	Anxa4	0,96
Vmn1r143	0,94	Gm1673	0,96	Atp1a2	0	Ftl1	0,96
Elf2	0,94	Wif1	0,96	Sis	0	Fam198b	0,96
Fbxo17	0,94	Acot8	0,96	Ces2a	0	Olfr569	0,96
Nps	0,94	Ap1g2	0,96	Acvrl1	0	2310057J18Rik	0,96
Dcaf15	0,94	Mkrn3	0,96	BRDN0000737949	0	Tmtc4	0,96
Aknad1	0,94	A630076J17Rik	0,96	Atp1a1	0	BRDN0000737727	0,96
Aqp3	0,94	BRDN0000737674	0,96	Fndc3c1	0	Ncmap	0,96
BRDN0000738096	0,94	Adh4	0,96	Clybl	0	Zfp688	0,96
Il1b	0,94	Vmn1r86	0,96	Id4	0	Pde6g	0,96
B230217C12Rik	0,94	Treml1	0,96	Hoxd3	0	Hint2	0,96
Szrd1	0,94	Lcn10	0,96	Id2	0	4921507P07Rik	0,96
Nlrp2	0,94	Hace1	0,96	Pgm1	0	2310035C23Rik	0,96
Smarcad1	0,94	Trdmt1	0,96	Pgm2	0	Cyp2c69	0,96
Gpr33	0,94	Cxcl16	0,96	Pgm3	0	Slc16a4	0,96
Txndc9	0,94	Gm4980	0,96	BC005537	0	Zfp109	0,96
Fam73b	0,94	B3gnt2	0,96	Slc31a1	0	Dao	0,96
Pdcd2l	0,94	Incenp	0,96	Chaf1a	0	Rims3	0,96
Ttc5	0,94	Mageb16	0,96	Olfr1206	0	Mphosph8	0,96
Mmp14	0,94	Ctla2b	0,96	BRDN0000738160	0	Gm15386	0,96
BC024139	0,94	Olfr223	0,96	Chaf1b	0	Krt82	0,96
Tmprss4	0,94	4932429P05Rik	0,96	BRDN0000738023	0	Tsc22d1	0,96
Il25	0,94	Dfnb59	0,96	Hmg20a	0	Pcnxl3	0,96
Sema6c	0,94	Sox15	0,96	Hmg20b	0	Psmc6	0,96
Dact3	0,94	Ormdl2	0,96	Hdac8	0	Rnf138rt1	0,96
Rln3	0,94	Olfr92	0,96	Cfhr2	0	Bpifa2	0,96
Lmo7	0,94	Olfr767	0,96	Procr	0	Obox5	0,96
Npr3	0,94	Mtbp	0,96	Rrnad1	0	Dlg3	0,96
Gm4922	0,94	Snta1	0,96	Cgn	0	Olfr608	0,96
Tdrp	0,94	Taz	0,96	Wwc2	0	4930544D05Rik	0,96
Itpr2	0,93	Klhl3	0,96	Nipa1	0	Olfr202	0,96
Cd74	0,93	Ap5m1	0,96	BRDN0000737490	0	Dnase2a	0,96
Pip4k2a	0,93	Fam171b	0,96	Cacnb4	0	Vmn1r12	0,96
Entpd2	0,93	Olfr30	0,96	Asgr1	0	Styxl1	0,96
Ctdspl	0,93	Map3k11	0,96	BC030336	0	Mtmr2	0,96
Olfr1179	0,93	6030419C18Rik	0,96	Neurl3	0	Sbsn	0,96
Gimap7	0,93	Serf1	0,96	Olfr98	0	Tnfrsf25	0,95
Sprr2b	0,93	Nlrp4b	0,96	Olfr99	0	BRDN0000737764	0,95
Clpx	0,93	Csf2ra	0,96	Chtf18	0	Kprp	0,95
Rpl22	0,93	Adamts1	0,96	Csn3	0	Trip11	0,95
Fstl1	0,93	Notum	0,96	Olfr94	0	Ankrd37	0,95
Sept4	0,93	Bid	0,96	Cyp2d22	0	Aknad1	0,95
Olfr549	0,93	Cyp4a14	0,96	Olfr96	0	Clic5	0,95
Serp2	0,93	Zfpl1	0,96	Olfr97	0	Olfr1338	0,95
Hadh	0,93	Podxl	0,96	Olfr90	0	Fetub	0,95
Spata5	0,93	Mb21d2	0,96	Cyp2d26	0	Pax2	0,95
BRDN0000737980	0,93	Olfr872	0,96	Olfr92	0	Olfr541	0,95
Reep3	0,93	Fut4	0,96	Olfr93	0	Srek1	0,95
Mrvi1	0,93	Kars	0,96	Obscn	0	Pola2	0,95
G630090E17Rik	0,93	Sgk2	0,96	Cyp4a14	0	Sema5b	0,95
Vmn1r52	0,93	Oma1	0,96	Supt16	0	Nppb	0,95
Speer4f	0,93	Msi2	0,96	Itprip	0	Cdk2ap1	0,95
Dab1	0,93	Chrna6	0,96	Cyp4a10	0	Ddhd1	0,95
Lrit3	0,93	Olfr802	0,96	Ankrd35	0	Zcchc11	0,95
B630005N14Rik	0,93	Olfr1410	0,96	Camk1	0	BRDN0000738034	0,95
Gm7714	0,93	Olfr624	0,96	Trub1	0	Ncdn	0,95
Ncoa2	0,93	Rtp2	0,96	Trub2	0	Atp1a4	0,95
4931406B18Rik	0,93	Gm15091	0,96	Ppp1r1c	0	Tnfsf10	0,95
Hist2h2bb	0,93	Tns2	0,96	Pramef25	0	Nagk	0,95
BRDN0000737795	0,93	C130074G19Rik	0,96	Zfp503	0	Ltbr	0,95
Blcap	0,93	Jrkl	0,96	BRDN0000738162	0	Cdc23	0,95
Clec16a	0,93	Spire1	0,96	Ppp1r1b	0	Olfr780	0,95
Kcnh4	0,93	Snap47	0,96	Zfp507	0	Msi2	0,95
Spef1	0,93	Sval1	0,96	Ammecr1	0	Tbx5	0,95
Rlf	0,93	Parp3	0,96	Tgif2	0	Tmed3	0,95
Itgb3	0,93	Pcdhb4	0,96	Gm16432	0	Myo7b	0,95
Rhox7a	0,93	Smco4	0,96	Arpp21	0	Crb3	0,95
Tgtp2	0,93	Slc9a3r2	0,95	Sirt3	0	Arsi	0,95
Olfr76	0,93	Map6	0,95	A430089I19Rik	0	Hist2h3c1	0,95
Zfp397	0,93	Fam129c	0,95	Zfp541	0	Higd1c	0,95
Bcl10	0,93	BRDN0000738314	0,95	Sncg	0	Ifnl2	0,95
Vmn1r174	0,93	Tmem151b	0,95	Barhl2	0	2310036O22Rik	0,95
Defb36	0,93	Shroom1	0,95	Barhl1	0	Hapln3	0,95
Cr1l	0,93	Man2b1	0,95	Trim61	0	Defb23	0,95

Stt3b	0,93	Rpl36al	0,95	Gsta4	0	BC051142	0,95
Bphl	0,93	Irf2bp1	0,95	Sez6l2	0	Vmn2r95	0,95
Lrrc38	0,93	BRDN0000738179	0,95	Ftmt	0	Trib3	0,95
Svs6	0,93	Dclre1b	0,95	Gsta2	0	Prkcb	0,95
Spns3	0,93	Cramp1l	0,95	Mfn1	0	Taz	0,95
1700011L22Rik	0,92	Tcf15	0,95	Pxk	0	Rab1	0,95
Ppp1r3f	0,92	Sp4	0,95	Ly6g5b	0	Gm6890	0,95
Efcab9	0,92	Ppat	0,95	Wdfy4	0	Hgsnat	0,95
Madcam1	0,92	Sox10	0,95	E230008N13Rik	0	Ccdc121	0,95
Olfr483	0,92	BRDN0000737864	0,95	BRDN0000738071	0	Ppp1r21	0,95
Olfr293	0,92	Prrx2	0,95	Wdfy3	0	Glipr1	0,95
Plekhg6	0,92	Gabbr2	0,95	Sirt2	0	Cds1	0,95
Klhl9	0,92	Ikzf3	0,95	Acy3	0	Vmn1r178	0,95
Pdzrn3	0,92	Dennd4c	0,95	Acy1	0	Ddx43	0,95
Ccnd3	0,92	Ccdc110	0,95	Pot1a	0	BRDN0000738203	0,95
Ndufs2	0,92	Nmi	0,95	Nrap	0	Vamp3	0,95
N4bp2l1	0,92	Styk1	0,95	Harbi1	0	Vmn2r89	0,95
Rasl10a	0,92	4933403O08Rik	0,95	Bptf	0	Pcdhb10	0,95
Epg5	0,92	Opn3	0,95	Lyl1	0	Vwce	0,95
Ccnb1ip1	0,92	Olfr609	0,95	Fam149b	0	Ap3b2	0,95
Ccr2	0,92	Agxt	0,95	Atp4b	0	BC035044	0,95
Haghl	0,92	Smap1	0,95	Hs1bp3	0	Hmga1-rs1	0,95
BRDN0000737698	0,92	Parp8	0,95	Med30	0	Icosl	0,95
Hnf1b	0,92	Capza3	0,95	Med31	0	Hey2	0,95
Csn1s1	0,92	Rec8	0,95	Asun	0	Krt81	0,95
Mllt11	0,92	Vmn1r9	0,95	Ctag2	0	Mier2	0,95
Dthd1	0,92	Arap2	0,95	Spryd3	0	Fbxl18	0,95
Hfe2	0,92	Src	0,95	Spryd4	0	Lrrc42	0,95
Olfr703	0,92	Csrp3	0,95	Nf2	0	Thap4	0,95
Ube2o	0,92	Fyb	0,95	Nf1	0	Olfr1445	0,95
Flrt1	0,92	Rdh7	0,95	Spryd7	0	Urad	0,95
Limd1	0,92	Iffo1	0,95	Sirt1	0	Rragb	0,95
Ocstamp	0,92	BRDN0000737420	0,95	Adcy6	0	Ufm1	0,94
Sucnr1	0,92	Ube2z	0,95	Pon2	0	Mmp8	0,94
Psmb5	0,92	Nfatc1	0,95	Scn3a	0	Serpini2	0,94
Slc39a10	0,92	Pou4f3	0,95	Scn3b	0	Cbln3	0,94
Cryz	0,92	Ccp110	0,95	1700029I15Rik	0	Adamtsl3	0,94
Spred3	0,92	Gm14781	0,95	Isoc2b	0	Slc25a2	0,94
Pde4d	0,92	BRDN0000738283	0,95	Rhox6	0	Nthl1	0,94
Dmtn	0,92	Rpe65	0,95	Kcnk1	0	Clec2d	0,94
Myo3b	0,92	Alg12	0,95	Fras1	0	Dnase1l2	0,94
Gpt2	0,92	BRDN0000738084	0,95	Trim34a	0	Rhpn1	0,94
Fgf23	0,92	Mogs	0,95	Nbl1	0	Pcolce2	0,94
Plch1	0,92	Iffo2	0,95	Npbwr1	0	Slc25a27	0,94
Pgam5	0,92	Lyz1	0,95	Phex	0	Sox5	0,94
Dzip3	0,92	Rbm12	0,95	Sncb	0	Ly6a	0,94
Adat1	0,92	Retnlb	0,95	5031410I06Rik	0	4930567H17Rik	0,94
Asf1a	0,92	Kdr	0,95	Cul3	0	Inpp5e	0,94
Taar5	0,92	4933427D14Rik	0,95	Bnip3l	0	Aldh1a7	0,94
Serhl	0,92	Elac1	0,95	9530053A07Rik	0	Dppa3	0,94
Aicda	0,92	Alkbh4	0,95	Gm6537	0	Vmn1r89	0,94
Sbsn	0,92	Aspm	0,95	Hist2h3c1	0	Mylk4	0,94
Mapre2	0,92	Pms2	0,95	Hist2h3c2	0	Olfr922	0,94
Gak	0,92	Slc25a19	0,95	S100a11	0	Pla2g2c	0,94
Taf4a	0,92	Map2k6	0,95	Ccnyl1	0	Igfbp7	0,94
Ccl26	0,92	Vwce	0,95	Tssk5	0	Syt14	0,94
Eif3k	0,92	Nxph1	0,95	Syce1l	0	Rgs21	0,94
Wnt6	0,92	Sec11c	0,95	BRDN0000738047	0	Tatdn1	0,94
Pigu	0,92	Slc25a46	0,95	Vmn1r66	0	Proser2	0,94
Naa10	0,92	Zbtb7c	0,95	Ipcef1	0	Il11ra1	0,94
Galt	0,92	Actb	0,95	Olfr120	0	Ank2	0,94
Vsig2	0,92	Rhod	0,95	Tat	0	Dpysl4	0,94
Sugp2	0,92	L1cam	0,95	Eps15	0	Grpr	0,94
Olfr518	0,92	Ascl4	0,95	Ptpru	0	Elmsan1	0,94
Dph2	0,92	Car11	0,95	Olfr339	0	Gpatch4	0,94
Cnot11	0,91	Kcnj9	0,95	Ptprs	0	Ptprj	0,94
Mcpt4	0,91	Atp2a2	0,94	Ptprr	0	Mapk1ip1	0,94
Gm9733	0,91	Slc27a3	0,94	Ptprq	0	Try5	0,94
Stmn2	0,91	Txndc11	0,94	Pth2	0	Coro2a	0,94
Pttg1	0,91	Tead1	0,94	Olfr332	0	Rab10	0,94
BRDN0000738123	0,91	Gpkow	0,94	Fam171a2	0	Zfp41	0,94
Dock8	0,91	Rcan2	0,94	Olfr330	0	Arl6ip6	0,94
Mef2d	0,91	Olfr1152	0,94	Olfr331	0	Ppm1j	0,94
1700026D08Rik	0,91	Ppp1r3a	0,94	Cyp2j9	0	Zfp819	0,94
Car3	0,91	Ostc	0,94	Dpep1	0	Iffo1	0,94
Adgrf1	0,91	Slc34a1	0,94	Ptprg	0	Tfec	0,94
Ftcd	0,91	Icos	0,94	Ptprf	0	Pcdhb21	0,94
Zfp566	0,91	1700057G04Rik	0,94	Ptpre	0	Klhl28	0,94
Asah1	0,91	Hmga2	0,94	Polr1c	0	Tspyl5	0,94
Sval1	0,91	Plekhg5	0,94	Ptprc	0	Gfra2	0,94
Zbtb49	0,91	Tpo	0,94	Dpep2	0	Tbc1d20	0,94
Hist1h1d	0,91	Syndig1	0,94	Ptpra	0	Cwc27	0,94

Dsg1a	0,91	Shb	0,94	Mtmr10	0	Igfals	0,94
Ldlrad3	0,91	Pirb	0,94	Ptprm	0	Dync1li1	0,94
Mcl1	0,91	Cask	0,94	Ptprk	0	BRDN0000738110	0,94
Iqsec1	0,91	Ino80	0,94	Ptprj	0	Cacnb1	0,94
Taar6	0,91	Prmt2	0,94	Rasa2	0	Bbs12	0,94
Olfr599	0,91	Gipc1	0,94	Pphln1	0	Slc13a4	0,94
Sapcd2	0,91	Gpr62	0,94	Gria1	0	Nrg4	0,94
A430105I19Rik	0,91	Ccl7	0,94	Rec114	0	Snx16	0,93
Celf4	0,91	4930480E11Rik	0,94	Vps8	0	Ccr2	0,93
Sim1	0,91	Vmn2r10	0,94	Klc3	0	Tssk6	0,93
Sod2	0,91	Olfr250	0,94	Olfr196	0	Syce1	0,93
Prr14l	0,91	Yy1	0,94	Olfr194	0	Gbp4	0,93
Hist1h2ad	0,91	Cfap74	0,94	Olfr195	0	Vps18	0,93
Acadvl	0,91	Aadacl3	0,94	Olfr193	0	Vmn1r52	0,93
Olfr622	0,91	Ccl25	0,94	Olfr190	0	Prima1	0,93
Adam5	0,91	Slpi	0,94	Olfr191	0	Fjx1	0,93
Tnfrsf12a	0,91	L3mbtl1	0,94	Prl7a1	0	Dtx1	0,93
Cycs	0,91	Ticam2	0,94	Prl7a2	0	2510003E04Rik	0,93
2010111I01Rik	0,91	1700019N19Rik	0,94	Olfr198	0	Arih2	0,93
Vash1	0,91	Chtf18	0,94	Olfr199	0	Far1	0,93
Nat2	0,91	Skint5	0,94	C2cd4b	0	Slc4a4	0,93
Ccdc30	0,91	Olfr96	0,94	Thop1	0	Gdap1	0,93
Kcnv2	0,91	Leo1	0,94	Six4	0	Fkbp15	0,93
Olfr52	0,91	Rreb1	0,94	Cage1	0	Pramef12	0,93
Rgs5	0,91	Lasp1	0,94	Six6	0	Ranbp10	0,93
Spatc1l	0,91	Cenpj	0,94	4930404N11Rik	0	Lrrtm2	0,93
Drd1	0,91	Olfr1178	0,94	Six1	0	Olfr745	0,93
Olfr27	0,91	Fbxl22	0,94	Six2	0	Id2	0,93
H2-Q10	0,90	BRDN0000737698	0,94	Six3	0	Ergic1	0,93
Lrrc51	0,90	Ctsg	0,94	Nphp3	0	Fbxw2	0,93
Sestd1	0,90	1600014K23Rik	0,94	Nphp1	0	Itih2	0,93
Gpr139	0,90	Atp6v1g2	0,94	4933417A18Rik	0	Emc10	0,93
E130012A19Rik	0,90	Sdk2	0,94	H2-Eb1	0	St3gal1	0,93
Alas2	0,90	Ptch2	0,94	H2-Eb2	0	Cmpk2	0,93
Pgap2	0,90	Cdcp1	0,94	Nphp4	0	Olfr44	0,93
Kcng1	0,90	Rell2	0,94	Evpl	0	Olfr559	0,93
Plcxd1	0,90	Tbpl2	0,94	Gm11554	0	Aspa	0,93
Cdc34	0,90	Mpzl2	0,94	Ankra2	0	Rbmxl2	0,93
Egfl6	0,90	Hspa12b	0,94	Fam172a	0	Hist1h4m	0,93
Olfr615	0,90	Il1rap	0,94	Fyb	0	Haus6	0,93
BC053393	0,90	Tab1	0,94	Map3k8	0	Egfr	0,93
Taf13	0,90	Sprr3	0,94	Map3k9	0	Plekhg3	0,93
Kctd4	0,90	Tnfsf18	0,94	Lrrc18	0	St18	0,93
Nadk	0,90	Krtcap3	0,94	Mb	0	Tnfrsf12a	0,93
Naa40	0,90	Olfr1043	0,94	Map3k2	0	Gm10081	0,93
Ehmt1	0,90	Cenpw	0,94	Map3k3	0	Slc7a4	0,93
Cd164	0,90	Tmbim6	0,94	Lrrc14	0	Cyp26a1	0,93
Tnfsf9	0,90	Olfr1387	0,94	Map3k1	0	Pygb	0,93
Adamdec1	0,90	Timm50	0,94	Map3k7	0	Zfp667	0,93
Tmem239	0,90	Olfr683	0,94	Map3k4	0	Usmg5	0,93
4632428N05Rik	0,90	Dpys	0,94	Map3k5	0	Otud1	0,93
Mia2	0,90	BRDN0000737803	0,93	Abca6	0	Psmc5	0,93
Aftph	0,90	Vmn1r41	0,93	Basp1	0	Dcaf13	0,93
Ppp6r1	0,90	Ralgds	0,93	Abca5	0	Zfp101	0,93
Map3k7	0,90	Rhbg	0,93	Abca2	0	Arhgef15	0,93
Syne3	0,90	Apoc3	0,93	Abca3	0	Wfdc3	0,93
Dntt	0,90	Vmn2r8	0,93	Abca1	0	Mrgprb4	0,93
Ankrd46	0,90	Olfr20	0,93	Ltk	0	Abl2	0,93
Olfr51	0,90	BRDN0000737412	0,93	Rab5a	0	Slc38a2	0,93
Zan	0,90	Tspan11	0,93	Fam32a	0	Stfa3	0,93
Olfr860	0,90	Nudt6	0,93	Abca9	0	Gprasp2	0,93
Ugt1a1	0,90	Sdr42e1	0,93	Zbtb3	0	Fam169a	0,93
Mfsd2a	0,90	Olfr53	0,93	Zbtb2	0	Vmn1r159	0,93
Cnih3	0,90	Usp27x	0,93	Zbtb1	0	1700012P22Rik	0,93
4933413G19Rik	0,90	AV320801	0,93	Hhex	0	Trpm1	0,93
Odam	0,90	Olfr130	0,93	Pdcl2	0	Ism2	0,93
Wdr34	0,90	Nphs1	0,93	Zbtb6	0	Gnas	0,93
Klhl8	0,90	Comtd1	0,93	Zbtb5	0	Nme5	0,93
Tgfbi	0,90	Trappc6a	0,93	Rfx4	0	Olfr1186	0,93
Spr	0,90	AI118078	0,93	Zbtb9	0	1810062G17Rik	0,93
Pcdha6	0,90	Sec61a2	0,93	Rras2	0	Lurap1l	0,93
4933427D06Rik	0,90	Twist2	0,93	Timm9	0	Rxra	0,93
Paxip1	0,90	8430419L09Rik	0,93	Rfx2	0	B020004J07Rik	0,93
Tex29	0,90	Rai1	0,93	Fgf23	0	Lep	0,93
Cmtm6	0,90	Gabbr1	0,93	Apopt1	0	Fryl	0,93
Gng2	0,90	Taf3	0,93	Tmem109	0	Zfand3	0,93
Fzd10	0,90	Ptn	0,93	Tmem108	0	Acnat1	0,93
Qsox1	0,90	Ffar3	0,93	Fktn	0	Rnaseh2b	0,93
Gm438	0,90	Casq2	0,93	Tmem107	0	Zfp639	0,92
Ttc21a	0,90	Timm13	0,93	Rbks	0	Olfr918	0,92
Mtmr4	0,89	Rbm4	0,93	Bora	0	Sfmbt2	0,92
Agpat6	0,89	Cxadr	0,93	Sco2	0	Cfap44	0,92

Kif14	0,89
Rnf167	0,89
Olfr1466	0,89
Trmt61a	0,89
Fam204a	0,89
Cdc42bpb	0,89
Rab3ip	0,89
Mydgf	0,89
Xylt2	0,89
Crygd	0,89
Spcs2	0,89
Hcn2	0,89
Itih5	0,89
Gmnc	0,89
Tgm2	0,89
Fbxl13	0,89
Defb26	0,89
Pias3	0,89
Olfr1161	0,89
Rtn4r	0,89
Atp6ap1l	0,89
Foxl2	0,89
Lpcat2b	0,89
Cbll1	0,89
Olfr148	0,89
Smim9	0,89
Themis3	0,89
Lman1l	0,89
Olfr417	0,89
Slc43a3	0,89
Drd4	0,89
Dpf3	0,89
Smok3a	0,89
Amh	0,89
Cacng3	0,89
Pydc3	0,89
Aste1	0,89
Sh2b2	0,89
Arih1	0,89
Kirrel3	0,89
S100a4	0,89
Adamts14	0,89
Flt1	0,89
Gli1	0,89
Igfl3	0,89
Gm2927	0,89
Zfp879	0,89
Proser2	0,89
Apol10b	0,89
Olfr514	0,88
Teddm3	0,88
Olfr281	0,88
Anp32a	0,88
Prdm11	0,88
Abca8a	0,88
Eda2r	0,88
Klf3	0,88
Tmem121	0,88
Peg3	0,88
Slco1a5	0,88
2700094K13Rik	0,88
Zfp663	0,88
Sncb	0,88
Hs1bp3	0,88
BRDN0000737941	0,88
Mc5r	0,88
Gm7073	0,88
Hid1	0,88
Slitrk5	0,88
Kcng2	0,88
Dctn4	0,88
Ssu2	0,88
Padi6	0,88
Ankhd1	0,88
Flna	0,88
Gm8660	0,88
Adtrp	0,88
Ralgapb	0,88
Ramp3	0,88
F3	0,88
BRDN0000738165	0,88
Olfr1424	0,88
Scara3	0,93
Ugt1a5	0,93
Zfp709	0,93
Lax1	0,93
Dnajc3	0,93
Ecm1	0,93
Slc18b1	0,93
Ccdc33	0,93
Aagab	0,93
H2-M2	0,93
Upf3a	0,93
Lins	0,93
Olfr285	0,93
4931409K22Rik	0,93
Zfp37	0,93
Agmat	0,93
Fbxo34	0,93
Psd2	0,93
Shisa4	0,92
Ercc6	0,92
Lrrc63	0,92
Gm608	0,92
Xlr3c	0,92
Spin4	0,92
Gas1	0,92
Hfe	0,92
Cypt2	0,92
Ces1e	0,92
Tmem57	0,92
Pgbd1	0,92
Olfr1168	0,92
Chrna10	0,92
Bag3	0,92
Gpatch2	0,92
Nutm2	0,92
Mvb12b	0,92
Defb11	0,92
Hnrnpll	0,92
Trp53	0,92
Slc2a6	0,92
Arhgef25	0,92
Fbrsl1	0,92
Zfp354a	0,92
Whamm	0,92
Mul1	0,92
BRDN0000738106	0,92
Rfc2	0,92
Unc5d	0,92
Cyp7b1	0,92
Adcyap1	0,92
Arhgap18	0,92
4930596D02Rik	0,92
S100a10	0,92
Olfr1183	0,92
Exosc7	0,92
Usp18	0,92
4933415F23Rik	0,92
Mmp13	0,92
Ly6f	0,92
Glra2	0,92
Arf3	0,92
Mrpl35	0,92
Olfr167	0,92
Emcn	0,92
Maged2	0,92
Clhc1	0,92
Olfr147	0,92
Olfr476	0,92
Olfml1	0,92
Fbln2	0,92
Ece2	0,92
AU022252	0,92
Slc24a1	0,92
Hfe2	0,92
Ethe1	0,92
Fam92b	0,92
Olfr1273-ps	0,92
Rimbp3	0,92
Tigar	0,91
Zfp870	0,91
Fam110b	0,91
BRDN0000737947	0,91
Aspscr1	0
Ift52	0
Wfikkn2	0
Ift57	0
4930503B20Rik	0
Fyn	0
Prr5l	0
Nktr	0
Setd4	0
Gabbr1	0
Txnl4b	0
Thsd1	0
Gm20809	0
BRDN0000738252	0
Pdzrn3	0
Etv1	0
G6pdx	0
Etv3	0
Etv2	0
Etv4	0
Etv6	0
Shprh	0
Nkd1	0
Sh3bp4	0
Sh3bp5	0
Pop1	0
Creb3l1	0
Pop7	0
Pop5	0
Pop4	0
Rgs1	0
Nlrc3	0
9030624J02Rik	0
Eif4g3	0
Eif4g2	0
Prps1l3	0
Apln	0
Cdhr5	0
Cyp2b10	0
Kcnk3	0
Cyp2b13	0
Gm8994	0
Lgi1	0
Gde1	0
Tceanc	0
Ptpn23	0
Slc14a2	0
Olfr1301	0
Olfr1303	0
Olfr1302	0
Col25a1	0
Olfr1307	0
C330018D20Rik	0
Olfr1309	0
Olfr1308	0
Cyth4	0
BRDN0000737493	0
Glul	0
Zfp148	0
Akap13	0
Akap10	0
Akap11	0
Tmc4	0
Olfr133	0
Tmc6	0
Tmc7	0
Tmc1	0
Nkd2	0
Tmc8	0
Zc3h12d	0
Catip	0
Dnajc14	0
Gramd2	0
Fbxl18	0
D17H6S53E	0
Anxa11	0
Anxa10	0
Ptpn21	0
Mlf1	0
Mlf2	0
Cox4i2	0
Ntan1	0
Prss48	0,92
Gm21637	0,92
B4gat1	0,92
Fgl2	0,92
Trhr2	0,92
2900092C05Rik	0,92
4933402N03Rik	0,92
Kcnf1	0,92
Olfr887	0,92
Lxn	0,92
Tas2r139	0,92
Atp5l	0,92
Tbc1d9	0,92
Dennd6b	0,92
Tgm1	0,92
Flrt2	0,92
Sh3pxd2b	0,92
Sltm	0,92
Lmbrd2	0,92
Btbd16	0,92
Serinc3	0,92
Scand1	0,92
Tmx1	0,92
A530064D06Rik	0,92
Pilra	0,92
Cldn7	0,92
Nr0b2	0,92
Ctsm	0,92
Prg4	0,92
Yipf3	0,92
Ccny	0,92
Mal2	0,92
Rasgef1a	0,92
Ceacam10	0,92
Lppr1	0,92
Casz1	0,92
Olfr484	0,92
Mrpl46	0,92
Lhfpl3	0,92
Mcpt1	0,92
BC022687	0,92
Necap1	0,92
Oas1f	0,92
Ift22	0,92
Fabp12	0,92
Eif4e	0,92
Foxi2	0,92
Defb10	0,92
Npm2	0,92
Bfar	0,92
C1ql2	0,92
Olfr659	0,92
Olfr763	0,92
Phka2	0,91
Ankk1	0,91
BRDN0000737934	0,91
Gpr83	0,91
Abcb4	0,91
Tmem200b	0,91
Vbp1	0,91
Dld	0,91
Krtap9-5	0,91
Tmem185b	0,91
Tmod4	0,91
Pcdh17	0,91
Fgfrl1	0,91
Hs2st1	0,91
Rab3a	0,91
Gm8439	0,91
Tdrp	0,91
Zfp937	0,91
Vip	0,91
Lsm1	0,91
Nr4a2	0,91
Eed	0,91
Tmem9	0,91
Rdh16	0,91
Il1f8	0,91
Clint1	0,91
Obox2	0,91
Rbmxl1	0,91
Mbtd1	0,91

Hcst	0,88
Tm6sf2	0,88
Gpr75	0,88
0610009B22Rik	0,88
Slc5a1	0,88
Ccdc6	0,88
Fam160b2	0,88
Cdkl1	0,88
Rcn2	0,88
Spata2	0,88
Cyp26a1	0,88
Sorcs3	0,88
Hlx	0,88
Josd2	0,88
Eps8l1	0,88
Asl	0,88
Ifi205	0,88
Mdh1b	0,88
Lrcol1	0,87
Abi1	0,87
Ubap1l	0,87
Sds	0,87
Asrgl1	0,87
Slc10a3	0,87
Mospd2	0,87
Bmyc	0,87
Nudt4	0,87
A3galt2	0,87
4921530L21Rik	0,87
Mtmr11	0,87
Brat1	0,87
1300017J02Rik	0,87
Kcna3	0,87
Ccdc53	0,87
Dhx58	0,87
Pepd	0,87
Zmym2	0,87
Syf2	0,87
Fam46b	0,87
Usp45	0,87
Esco1	0,87
Gpr162	0,87
Pax3	0,87
9030617O03Rik	0,87
Icam1	0,87
Cntn5	0,87
Zfp36	0,87
Gjb4	0,87
4930544D05Rik	0,87
Papola	0,87
Btbd18	0,87
1700007B14Rik	0,87
Junb	0,87
Gtsf1	0,87
Slc2a8	0,87
Tnks2	0,87
Zfp423	0,87
Parg	0,87
Rab3gap1	0,87
Marcksl1	0,87
Tmem14a	0,87
Pamr1	0,87
Kiss1	0,87
Olfr651	0,86
BRDN0000738045	0,86
Cyp4v3	0,86
Angpt1	0,86
Dbndd2	0,86
Notch3	0,86
4930402K13Rik	0,86
Fbxw19	0,86
Rexo2	0,86
Mrpl50	0,86
Ankmy1	0,86
Zfp36l3	0,86
Gm20826	0,86
Shisa8	0,86
Chmp6	0,86
Asb14	0,86
Tssc4	0,86
Smyd2	0,86
Olfr623	0,86
Ccnb3	0,91
Cyp2c65	0,91
Qsox2	0,91
Myo16	0,91
Zfp493	0,91
Slc15a4	0,91
Lrrc52	0,91
Mdh1	0,91
Evi2b	0,91
Itsn1	0,91
Ccdc58	0,91
Trip12	0,91
Galnt10	0,91
Rbm7	0,91
Ceacam20	0,91
Rab3d	0,91
Opalin	0,91
Dusp8	0,91
Fst	0,91
Ncoa3	0,91
Ier5l	0,91
Fpr-rs6	0,91
Rapgefl1	0,91
Rtn4	0,91
Hps5	0,91
Mrgprg	0,91
Nap1l3	0,91
Gimap7	0,91
Hs6st1	0,91
Pigf	0,91
Gm4894	0,91
BRDN0000737916	0,91
Kcnab2	0,91
Gm10100	0,91
Tacr3	0,91
Ddx18	0,91
Trappc9	0,91
Cd247	0,91
Mbl2	0,91
A630023A22Rik	0,91
Xpo7	0,91
Ccdc65	0,91
Fer1l4	0,91
Gng4	0,91
Eme2	0,91
Afg3l1	0,91
Gmeb1	0,91
Gm2913	0,91
Gtpbp8	0,91
Apobec1	0,91
Psmd9	0,91
BRDN0000737594	0,91
Uvrag	0,91
Lrrc71	0,91
Ins2	0,91
Cacnb3	0,91
Gm6040	0,91
Fut9	0,91
BRDN0000737830	0,90
Cfc1	0,90
Loxl4	0,90
Stk26	0,90
Ndufaf2	0,90
Fam159a	0,90
D15Ertd621e	0,90
Pld6	0,90
Vps41	0,90
Mapre1	0,90
Nfkbil1	0,90
Tex13a	0,90
Krtap9-3	0,90
Olfr1310	0,90
Itga5	0,90
Zfp945	0,90
Prima1	0,90
Asb5	0,90
Steap1	0,90
Slc26a5	0,90
Ccdc57	0,90
Zfp36l3	0,90
Olfr61	0,90
Olfr822	0,90
Cox4i1	0
Fam206a	0
Tcn2	0
Fam47c	0
Hoga1	0
Herc2	0
Herc4	0
Herc6	0
2210018M11Rik	0
Qrich1	0
Prob1	0
Zcchc12	0
Krt28	0
Rab8a	0
Zcchc17	0
Alkbh8	0
Btn1a1	0
Krt20	0
Zcchc18	0
Krt26	0
Krt27	0
Krt24	0
Krt25	0
Gm12185	0
Aurkaip1	0
Zbed3	0
Alkbh7	0
Sort1	0
Zfyve1	0
Dmrtc1b	0
Dmrtc1a	0
Dnaja2	0
Dnaja3	0
Dnaja1	0
Olfr305	0
Dnaja4	0
Psma3	0
Iah1	0
Mrpl41	0
Wdr26	0
Wdr27	0
Wdr24	0
Wdr25	0
Zswim8	0
4930513O06Rik	0
Wdr20	0
Gm19402	0
Olfr54	0
Alkbh1	0
Psma7	0
Igf2	0
Acer3	0
Apod	0
Acer1	0
Smim7	0
Smim6	0
Smim5	0
Olfr801	0
Apon	0
Apoo	0
Smim9	0
Smim8	0
BRDN0000738087	0
Entpd5	0
Il4	0
Ssna1	0
Esf1	0
Recql	0
D330045A20Rik	0
Vat1l	0
Trpt1	0
Alppl2	0
Trim30a	0
Agbl4	0
Agbl2	0
Cped1	0
Rnf125	0
Ccser2	0
Yars2	0
Kcng2	0
Olfr807	0
Tchhl1	0
Gtf2h1	0,91
Tmem67	0,91
Gm20809	0,91
Gm12169	0,91
Zdhhc18	0,91
Gja8	0,91
Olfr1154	0,91
Gapvd1	0,91
Rcan3	0,91
Dennd1a	0,91
Olfr473	0,91
BC030500	0,91
Zfp874a	0,91
Plcd4	0,91
Vmn1r175	0,91
Clpsl2	0,91
Ptgr2	0,91
Pxmp2	0,91
Olfr111	0,91
Erap1	0,91
Rab44	0,91
Parp6	0,91
Gje1	0,91
Prrc1	0,91
Rcan1	0,91
Gpr153	0,91
Hp	0,91
Nudt12	0,91
Vat1l	0,91
Als2	0,91
Vmn1r83	0,91
4933406J08Rik	0,91
Adi1	0,91
Rufy1	0,91
Gm5549	0,91
Lpcat3	0,91
Abcc8	0,91
Pigr	0,90
Prss46	0,90
Adgrf3	0,90
Cacng4	0,90
Fam3b	0,90
Pqlc1	0,90
Git1	0,90
Mreg	0,90
Sema4g	0,90
Lhx4	0,90
Pex10	0,90
Ces2g	0,90
Nyap2	0,90
Tsen34	0,90
Zbtb46	0,90
Ccdc77	0,90
Tmem8b	0,90
Nhs	0,90
Prl3d2	0,90
Slc35d2	0,90
Gm5538	0,90
Adamdec1	0,90
Tmem62	0,90
Ccl24	0,90
Cd19	0,90
Cox7a2	0,90
Rhox4d	0,90
Serpinb1b	0,90
Olfr1113	0,90
Olfr99	0,90
Plag1	0,90
Sp110	0,90
Cacna1a	0,90
Marveld2	0,90
Rgs9bp	0,90
Cntn4	0,90
Gadd45gip1	0,90
Styx	0,90
BRDN0000737449	0,90
Dpy19l3	0,90
Npy6r	0,90
Sowahb	0,90
Fpr-rs6	0,90
Card14	0,90
Krt28	0,90

Lce3e	0,86
Csf3	0,86
Clec10a	0,86
Lce1g	0,86
Fam219a	0,86
Harbi1	0,86
Cyp20a1	0,86
Rin3	0,86
Tub	0,86
Arhgdia	0,86
Soat2	0,86
Hbb-bh2	0,86
Dram2	0,86
Sec23a	0,86
Nefh	0,86
Gm19402	0,85
Capza3	0,85
Fosl2	0,85
Ascl4	0,85
Creb5	0,85
Mycl	0,85
Krt36	0,85
Map7	0,85
Bmpr2	0,85
Fam64a	0,85
Cryge	0,85
Zic2	0,85
Vamp5	0,85
Ifna1	0,85
Plek2	0,85
Il12rb1	0,85
Pogz	0,85
Mapk7	0,85
Cdadc1	0,85
Whsc1	0,85
4930453N24Rik	0,85
Tex11	0,85
BRDN0000737768	0,85
Mex3b	0,85
Purg	0,85
Rhox6	0,85
Dcaf12	0,85
Uba7	0,85
Fam214a	0,85
Vsig4	0,85
Prlhr	0,85
Olfr1020	0,85
Prkg1	0,85
Hspb7	0,85
Ldhal6b	0,85
Gng3	0,85
Mdk	0,85
Ppp1r26	0,85
Mboat4	0,85
Pilrb2	0,85
Fam3a	0,85
Tcl1	0,85
Akap7	0,85
Olfr761	0,85
Slc25a10	0,84
Mlkl	0,84
Unc13a	0,84
Cox7b2	0,84
Kcnip2	0,84
Lbx2	0,84
Mfsd7a	0,84
Adamts3	0,84
BRDN0000737462	0,84
BRDN0000738256	0,84
Pfn2	0,84
Ptprz1	0,84
C1galt1	0,84
Unc119b	0,84
Hist2h4	0,84
Prkcz	0,84
Dnajc18	0,84
Galnt7	0,84
Stx1a	0,84
Tbx10	0,84
Chd9	0,84
Gm7849	0,84
Trim65	0,84
BC049635	0,90
Hivep1	0,90
Aftph	0,90
Cd2	0,90
P2ry10	0,90
Olfr871	0,90
Nipal1	0,90
Nrg2	0,90
Serpinb1a	0,90
Ubald1	0,90
Bcar3	0,90
Gm15284	0,90
Iqub	0,90
Klhdc1	0,90
Bloc1s6	0,90
Apcdd1	0,90
Calcoco1	0,90
Aldh8a1	0,90
Tex29	0,90
Ankrd13c	0,90
Dydc2	0,90
Trim34b	0,90
Fam196b	0,90
Itga3	0,90
Apobec4	0,90
Olfr1511	0,90
Nosip	0,90
Tnfrsf14	0,90
Pih1d1	0,90
Ccdc68	0,90
Olfr1436	0,90
Tapt1	0,90
Lrrc26	0,90
1700019D03Rik	0,90
Zfand1	0,90
Zfp941	0,90
Actrt1	0,90
Lrrc47	0,90
Gabre	0,90
Rhox7a	0,90
Hist1h2bl	0,89
Ak7	0,89
Sqstm1	0,89
Phka1	0,89
I830077J02Rik	0,89
Pigb	0,89
Hoxc11	0,89
Magt1	0,89
Zfpm2	0,89
Anxa1	0,89
Dgcr2	0,89
Cdk2ap2	0,89
Ppp4r1	0,89
Gabpb2	0,89
Cyp2c55	0,89
Gsdma	0,89
Ptpre	0,89
Defb25	0,89
Emilin1	0,89
Ankrd34b	0,89
Vmn1r185	0,89
Adrbk1	0,89
6430550D23Rik	0,89
Dus4l	0,89
Cxcl14	0,89
Gpr25	0,89
Gm17660	0,89
Palld	0,89
Vmn1r158	0,89
Sh3kbp1	0,89
Pik3ip1	0,89
Ccnk	0,89
C1ql3	0,89
Depdc1b	0,89
B3galnt2	0,89
Efna5	0,89
Fam76b	0,89
Luzp4	0,89
Mtfp1	0,89
Irf7	0,89
Zw10	0,89
5730508B09Rik	0,89
Ldlr	0
Bcl7b	0
Zfp35	0
Stard8	0
Zfp37	0
Zfp30	0
Bcl7a	0
Atp6v0d2	0
Srp19	0
Stard7	0
Stard4	0
Foxo6	0
BRDN0000737671	0
Antxr1	0
Gtsf1	0
Antxr2	0
Ambp	0
Foxo4	0
Pdik1l	0
Snap29	0
Tspan11	0
Mcph1	0
Trmt2a	0
Cldn12	0
Cdk5rap2	0
Cdk5rap3	0
Pbx4	0
Ccm2	0
Pbx2	0
Fam126a	0
Fam126b	0
Pbx1	0
Olfr1389	0
Acaa1b	0
Acaa1a	0
Dock9	0
Dock3	0
Dock1	0
Zkscan3	0
Dock4	0
Dock5	0
BRDN0000737818	0
BRDN0000737819	0
Uba2	0
Lpcat2	0
Lpcat3	0
Lpcat1	0
BRDN0000737810	0
Milr1	0
BRDN0000737812	0
BRDN0000737813	0
BRDN0000737814	0
BRDN0000737815	0
BRDN0000737816	0
Zbtb45	0
Asb10	0
Tfr2	0
Asb13	0
Asb14	0
Asb15	0
Asb16	0
Dld	0
Asb18	0
Zkscan6	0
Fa2h	0
Uba3	0
Tlr11	0
Tlr12	0
Zfp119a	0
Acot10	0
4921501E09Rik	0
Morn3	0
Rnf24	0
Dpf1	0
Rnf26	0
Rnf20	0
Plcxd3	0
Hsd17b12	0
Zfp317	0
Ifnlr1	0
Hsd17b11	0
Foxk2	0
Tle1	0,90
Cplx1	0,90
Pa2g4	0,90
Tmem17	0,90
Slfn1	0,90
Tspan10	0,90
Ankrd13a	0,90
Apbb1ip	0,90
1700020A23Rik	0,90
Ace2	0,90
Olfr568	0,90
Lactbl1	0,90
Me3	0,90
Olfr1197	0,90
Mfap2	0,90
Lyrm5	0,90
Hint1	0,90
Vmn1r39	0,90
Olfr1359	0,90
Rassf7	0,90
Ddi2	0,90
Asph	0,90
Trmt11	0,90
Krt6a	0,90
Tpsb2	0,90
Hist1h2bj	0,90
Ccdc43	0,90
S100a11	0,90
Sh3rf1	0,90
Mtap7d3	0,90
Zfp697	0,90
Bdkrb2	0,90
Cyth4	0,90
Spata22	0,89
Cmpk1	0,89
Svs5	0,89
Osbpl11	0,89
Gm15080	0,89
Fibcd1	0,89
Prl7c1	0,89
Rhox7a	0,89
Ip6k1	0,89
Fbxo6	0,89
A530016L24Rik	0,89
Olfr9	0,89
Zfp1	0,89
Pcx	0,89
Flot1	0,89
Lsmem1	0,89
Rptor	0,89
Olfr483	0,89
Slc35g3	0,89
Tep1	0,89
Anxa7	0,89
Naa10	0,89
Arid5a	0,89
Defb7	0,89
Irf2bp2	0,89
Olfr1080	0,89
Tpmt	0,89
Olfr727	0,89
Tssk2	0,89
Lysmd3	0,89
Crym	0,89
Fgf4	0,89
Rsf1	0,89
Lsm8	0,89
Usp26	0,89
BRDN0000737806	0,89
Tnfsf11	0,89
Flt4	0,89
Siah1b	0,89
Gpc2	0,89
BRDN0000737502	0,89
Acsl5	0,89
Nat9	0,89
Ucp3	0,89
Fbxw10	0,89
Lrrc14	0,89
Ube2i	0,89
Ptk2	0,89
Cxcr5	0,89

Edn3	0,84
Vmn1r183	0,84
Slc2a9	0,84
Ubxn10	0,84
C2cd5	0,84
Lipk	0,84
Pygo1	0,84
Lmln	0,84
Pdha2	0,84
C1qtnf3	0,84
Galnt6	0,84
Stac2	0,84
Krtap4-16	0,84
1700013H16Rik	0,84
Nucb1	0,84
Defb8	0,84
Ist1	0,84
Zfp946	0,84
C1qb	0,84
Peli3	0,84
Fam25c	0,84
Arl8b	0,84
Tceal1	0,84
Smg7	0,84
Adarb2	0,84
Trip13	0,84
Fam160a1	0,84
Pcyt1b	0,84
Neurl4	0,84
Nr2f6	0,84
Fev	0,84
Fbxw24	0,83
Eftud1	0,83
Chd3	0,83
Dusp8	0,83
Vmn1r64	0,83
AI593442	0,83
Olfr301	0,83
Cdc42	0,83
Btnl6	0,83
Zfp14	0,83
Atp6v0a4	0,83
Agbl5	0,83
Sec11c	0,83
Mycn	0,83
Gpatch2	0,83
1190005I06Rik	0,83
Atp11b	0,83
Magea4	0,83
Celf1	0,83
Fam184b	0,83
Agpat4	0,83
Klhl11	0,83
Trex1	0,83
BRDN0000737841	0,83
Lrrc9	0,83
Gnrhr	0,83
1700088E04Rik	0,83
E2f6	0,83
Dguok	0,83
Lyplal1	0,83
Thap4	0,83
Ptgfrn	0,83
Rnf207	0,83
Aqp8	0,83
Pvrl2	0,83
Gjc3	0,83
Olfr568	0,83
Anapc16	0,82
Dscam	0,82
Slco5a1	0,82
Capn9	0,82
Ak8	0,82
E330017A01Rik	0,82
Tekt3	0,82
Dcp1b	0,82
Tmem254a	0,82
Tmem106c	0,82
Ms4a6d	0,82
Itgb2	0,82
Tlr13	0,82
Krtap16-3	0,82
Cyp11b1	0,89
Pld1	0,89
Sftpd	0,89
Olfr809	0,89
Nkx2-9	0,89
Runx1	0,89
Ppara	0,89
Mkx	0,89
Emd	0,89
Itpk1	0,89
Nlrp1b	0,89
Pklr	0,89
Dsp	0,89
BRDN0000738183	0,89
Cog3	0,89
Vmn2r14	0,89
Thoc5	0,89
Ldb3	0,89
Anxa3	0,89
Etv1	0,89
Izumo4	0,89
Trim45	0,89
BRDN0000737442	0,89
Cyp2d11	0,89
Olfr618	0,89
Olfr1307	0,89
Hist1h3d	0,89
Toe1	0,89
Wfdc8	0,89
Clnk	0,89
Slc38a1	0,89
Prl2c3	0,89
Ppp1r3c	0,89
Slc6a14	0,89
Egr1	0,89
Nmt1	0,89
Ms4a15	0,89
Slc25a44	0,89
Il17f	0,89
Prr30	0,89
Phactr3	0,89
Cd160	0,89
Sprr2a1	0,88
Il4i1	0,88
Prdx6b	0,88
Fam206a	0,88
Kcnq1	0,88
Mettl4	0,88
Serpinb3b	0,88
Slc6a5	0,88
Lrrc59	0,88
Reep2	0,88
Ubp1	0,88
Map3k15	0,88
1700008F21Rik	0,88
Vmn1r71	0,88
Trim80	0,88
Pdzd2	0,88
Plbd2	0,88
Gucd1	0,88
Cldn20	0,88
Gnas	0,88
Tnip1	0,88
Smok3a	0,88
Vstm2b	0,88
Cdk19	0,88
Sft2d3	0,88
Efna2	0,88
Ivl	0,88
Neb	0,88
Nln	0,88
Naa16	0,88
Zfp248	0,88
Tk1	0,88
Gm5108	0,88
Hist1h1d	0,88
Rab11fip4	0,88
Celf1	0,88
Olfr1109	0,88
Cd300lg	0,88
Vmn1r211	0,88
Cdc20	0,88
Dppa3	0
Phlda1	0
Gm6696	0
Zfp318	0
Acot11	0
Prr14l	0
Phlda3	0
Slc35a5	0
Rnf128	0
Rnf126	0
BRDN0000737675	0
Phlda2	0
Rnf123	0
Rnf122	0
Rnf121	0
Il2ra	0
Il2rb	0
Marveld3	0
Il2rg	0
Cngb1	0
Olfr527	0
Cngb3	0
Mrps26	0
Tbc1d30	0
Katna1	0
Tfrc	0
Tnfrsf11a	0
Tnfrsf11b	0
Slc25a29	0
Cyp2c68	0
Abhd16a	0
Pptc7	0
Mc5r	0
BRDN0000738257	0
Kctd3	0
1700029H14Rik	0
Kctd1	0
Kctd7	0
Kctd5	0
Dhx57	0
Kctd9	0
Zfp276	0
Sdk2	0
Gm4922	0
Zfp273	0
Olfr295	0
Olfr297	0
Olfr291	0
Olfr290	0
Olfr293	0
Cyct	0
Nup214	0
Minos1	0
Nup210	0
Olfr298	0
Pip4k2c	0
Ssxb8	0
Zdhhc15	0
Caln1	0
Cyp2c67	0
Synpo	0
Ntng1	0
Msmp	0
Olfr1154	0
Olfr1155	0
Olfr1156	0
Olfr1157	0
BRDN0000738169	0
Olfr1151	0
Olfr1152	0
Olfr1153	0
Slc23a1	0
Slc9a3r2	0
Slc23a3	0
Zdhhc16	0
Trdmt1	0
Msmb	0
Pik3c2g	0
Glb1l	0
Aox3	0
Synpr	0
Tnni3	0
Tmem254a	0,89
Hspa4	0,89
Lrrc3	0,89
Sept11	0,89
Tgfbr3l	0,89
Tnfrsf13c	0,89
Eif1b	0,89
Dlx6	0,89
Pcdhb11	0,89
Shcbp1	0,89
4930595M18Rik	0,89
Ttc7b	0,89
Vmn2r121	0,89
Arhgap1	0,89
Fkbp7	0,89
Dlx3	0,89
Igfbp6	0,89
Rorc	0,89
Slc5a6	0,89
Slc1a2	0,89
Rin1	0,88
Gxylt2	0,88
Gfm1	0,88
Cops7a	0,88
Tubb2a	0,88
Tnfrsf22	0,88
Cbx3	0,88
Vwa1	0,88
Dhx32	0,88
Ier3	0,88
Ago3	0,88
Ccl8	0,88
1700055N04Rik	0,88
Tmem88b	0,88
Klhl41	0,88
Gm609	0,88
Lrrc16b	0,88
Gm4301	0,88
Akt2	0,88
Nupl2	0,88
Aldh3b2	0,88
Itga6	0,88
Arhgap39	0,88
Tmem209	0,88
BRDN0000738159	0,88
Slain1	0,88
Rasl10b	0,88
2810006K23Rik	0,88
Peg3	0,88
Etl4	0,88
Pik3ap1	0,88
4930557A04Rik	0,88
Ksr1	0,88
Atp2a3	0,88
G3bp1	0,88
Rtn1	0,88
Ptprg	0,88
Ror2	0,88
Tmem45a	0,88
Zfp420	0,88
Brd7	0,88
Olfr1276	0,88
Mvp	0,88
4933403O08Rik	0,88
Galnt7	0,88
4933413G19Rik	0,88
Clec3a	0,88
Ugt2a2	0,88
Aco2	0,88
Sytl4	0,88
Ncald	0,88
Fbxo30	0,88
Cd244	0,88
Mtap	0,88
Ctss	0,88
4930506M07Rik	0,88
Ddx10	0,88
Rbm7	0,88
Uqcrfs1	0,88
6330408A02Rik	0,88
Fam221a	0,88
Kcnh6	0,88

Gm9758	0,82	Opn1mw	0,88	Armt1	0	Clec4g	0,88
Tpsg1	0,82	Pgp	0,88	Piwil2	0	Acbd5	0,88
1700001F09Rik	0,82	Gm5416	0,88	Theg	0	Olfr1286	0,88
Drd2	0,82	Olfr1325	0,88	Timm10b	0	BRDN0000738004	0,88
Cltb	0,82	Fam221a	0,88	Kcnj5	0	Khdc1b	0,88
Krtap20-2	0,82	Hoxc10	0,88	Kcnb2	0	Gm5382	0,88
G3bp2	0,82	Dnm3	0,88	Soat1	0	Olfr1477	0,88
Cap2	0,82	Edn3	0,88	Ppp2r2c	0	Rnf14	0,88
Dmrta2	0,82	Gjc1	0,88	Dusp19	0	Olfr1329	0,88
Phospho2	0,82	BRDN0000738202	0,88	Dusp18	0	Otud5	0,88
Grin2d	0,82	Pcdh15	0,88	Aox4	0	Cd3g	0,88
Gm10352	0,82	Bscl2	0,88	Dusp15	0	Pdx1	0,88
Lgr6	0,82	Lhfpl1	0,88	Dusp14	0	Axin2	0,88
Krtap19-1	0,82	Ankrd39	0,88	Dusp16	0	Aunip	0,88
Gm15056	0,82	BRDN0000738005	0,88	Dusp11	0	Dnmt1	0,88
Apeh	0,82	Atp12a	0,88	Dusp10	0	Gatad2a	0,88
Rlbp1	0,82	Lrrc10b	0,88	Dusp12	0	Fbxo24	0,88
Snapc1	0,82	Dnajc27	0,88	Myh10	0	Alas2	0,87
Trmt6	0,82	Lrp4	0,88	Myh11	0	Lmf1	0,87
Fchsd1	0,82	Dnah11	0,88	Sgpl1	0	Gulp1	0,87
Tsx	0,82	Olfr275	0,88	Myh14	0	Mak16	0,87
Chfr	0,82	Pstpip2	0,88	Myh15	0	Trim14	0,87
Rab31	0,82	Itga7	0,88	Mamstr	0	Alpk3	0,87
Aldh3b2	0,82	Olfr1164	0,88	Irs4	0	Kcnc2	0,87
Arhgef25	0,82	Mum1l1	0,88	Optc	0	Plscr2	0,87
E030002O03Rik	0,82	Pex11g	0,88	Krit1	0	Ptch1	0,87
Sfi1	0,82	Vps39	0,88	BC003331	0	Gpr152	0,87
Rspo4	0,82	Pde7a	0,88	Pitx3	0	Agpat2	0,87
Tjp3	0,82	Gbp4	0,88	Tmem59l	0	Tfap2b	0,87
2310067B10Rik	0,81	Pla2g5	0,88	Aste1	0	Rhox13	0,87
Zbtb9	0,81	Phyh	0,88	Dynlt1c	0	Mdh1	0,87
BRDN0000737626	0,81	Atp11a	0,88	Dynlt1b	0	Prf1	0,87
Pdzd7	0,81	Gm20865	0,88	Nufip2	0	Cpne1	0,87
Pkmyt1	0,81	E230019M04Rik	0,88	Panx2	0	Cckar	0,87
Olfr1448	0,81	Zfp808	0,88	Dynlt1f	0	Tjp1	0,87
Olfr688	0,81	Odf2	0,87	Olfr1437	0	Atp6v0e	0,87
Rac2	0,81	Ece1	0,87	Gps1	0	Snapc3	0,87
Slc25a46	0,81	Ces2e	0,87	Gps2	0	Slco1a5	0,87
Kcng3	0,81	4930430F08Rik	0,87	Pitx1	0	Kiz	0,87
Cep19	0,81	Igflr1	0,87	Slc9a6	0	Psg23	0,87
Olfr357	0,81	Scgb1b2	0,87	Pfkfb2	0	Sult1e1	0,87
BRDN0000738294	0,81	Polr3c	0,87	Pfkfb4	0	Tmem234	0,87
Prrg1	0,81	Zbtb7a	0,87	Rtp2	0	Olfr996	0,87
Wdr38	0,81	Gm15093	0,87	Gsdma3	0	Hmcn1	0,87
Gm16532	0,81	Pnmal2	0,87	Fam110b	0	BRDN0000737465	0,87
Ccdc186	0,81	Abcf3	0,87	Camta2	0	Guca2a	0,87
Vmn1r213	0,81	Mboat2	0,87	Rundc3b	0	Olfr906	0,87
Snx20	0,81	Fam187b	0,87	Prkrip1	0	Zfp956	0,87
Nkx2-3	0,81	4930550L24Rik	0,87	Camta1	0	Mtf1	0,87
Lactb	0,81	Nsg1	0,87	Gadl1	0	Plekhm3	0,87
Pdk2	0,81	Cckar	0,87	Mob1a	0	Opn5	0,87
Msmp	0,81	Vmn1r79	0,87	Mob1b	0	Tars2	0,87
Sufu	0,81	1700055N04Rik	0,87	BRDN0000737497	0	Cideb	0,87
Xpo5	0,81	Kcnt1	0,87	Slc20a2	0	Usp40	0,87
Dgki	0,81	Cinp	0,87	Slc20a1	0	Upp1	0,87
BRDN0000738134	0,81	Klk13	0,87	Apcdd1	0	Cdpf1	0,87
Hoxd3	0,81	Trmt1l	0,87	Dpf3	0	Zbtb22	0,87
Leo1	0,81	Taar7f	0,87	Alb	0	Bloc1s3	0,87
Nbas	0,81	Vmn2r101	0,87	Fstl4	0	Ubac2	0,87
Tpi1	0,81	Il10rb	0,87	Tfcp2l1	0	A430005L14Rik	0,87
Ezh1	0,81	Abo	0,87	Mss51	0	Paqr7	0,87
Txlnb	0,81	Panx1	0,87	BC051628	0	Syngr3	0,87
Zfp953	0,81	Nkx3-1	0,87	Tm9sf4	0	Fam89a	0,87
Rgr	0,81	Wfdc16	0,87	Mboat1	0	Ahctf1	0,87
Olfr1143	0,81	Gpr68	0,87	Scnm1	0	Eif3f	0,87
Slc1a7	0,81	Dzip3	0,87	Tm9sf1	0	Fpr-rs3	0,87
Lyve1	0,81	Fam129b	0,87	Dscaml1	0	Kctd20	0,87
Ntan1	0,81	Tmem176b	0,87	Tm9sf3	0	Hoxc8	0,87
Cacna2d3	0,81	Fscb	0,87	Mboat4	0	Nsa2	0,87
Olfr617	0,81	Proz	0,87	BRDN0000737737	0	Car8	0,87
Mrps2	0,81	Mrpl50	0,87	Cadm4	0	Ugt1a2	0,87
Cbfa2t2	0,80	Gjb4	0,87	Zik1	0	Vmn1r80	0,87
Zfp112	0,80	Mtap	0,87	Cadm2	0	Ncaph2	0,87
4930430F08Rik	0,80	Aspn	0,87	Cadm3	0	Rasl11a	0,87
Plscr2	0,80	Prl3d3	0,87	Srcin1	0	Mill2	0,87
Efna5	0,80	Cox5a	0,87	1700109H08Rik	0	F2rl1	0,87
Dok6	0,80	Prdm14	0,87	Eif3j2	0	2410004B18Rik	0,87
Notch2	0,80	Ppp1r1b	0,87	Eif3j1	0	Agxt	0,87
Ccl2	0,80	Mc4r	0,87	Ecd	0	Aaed1	0,87
Traf6	0,80	Olfr893	0,87	Tfam	0	Igf2bp3	0,87
Vmn2r11	0,80	Plscr3	0,87	Rp9	0	Nyap1	0,87
Aup1	0,80	Olfr728	0,87	Cep120	0	Prnp	0,87

Eif4a2	0,80
C1qtnf9	0,80
Clcn4-2	0,80
Olfr351	0,80
Slc25a47	0,80
Itgb3bp	0,80
F13a1	0,80
Olfr212	0,80
Ogfod1	0,80
Zfand2a	0,80
Hist2h2be	0,80
Nono	0,80
Prdx6	0,80
Olfr1427	0,80
Ropn1l	0,80
Vsig8	0,80
Defb2	0,80
Ace2	0,80
Rnf149	0,80
Tas2r115	0,80
Plp2	0,80
Poglut1	0,80
Foxe3	0,80
Pcdhb17	0,80
Defb15	0,80
Cpne7	0,80
Trap1	0,80
Ppan	0,80
Fam65b	0,79
Gins3	0,79
Ddx3x	0,79
Lvrn	0,79
Fundc2	0,79
Trappc11	0,79
Rasl11a	0,79
Olfr1066	0,79
Fam178b	0,79
Tspyl5	0,79
Traf3ip3	0,79
Ncapd2	0,79
Kcnu1	0,79
Smim5	0,79
Nptxr	0,79
Serinc2	0,79
Ghdc	0,79
Car10	0,79
Olfr1110	0,79
Hs3st3b1	0,79
Fam187b	0,79
Oxld1	0,79
Inafm1	0,79
Heca	0,79
Cdc42bpg	0,79
Gareml	0,79
Krt28	0,79
Frmpd1	0,79
Reck	0,79
Nap1l5	0,79
Rbks	0,79
Cd27	0,79
1700019A02Rik	0,79
Fam198a	0,79
Pelo	0,79
Hadha	0,79
Gm4884	0,79
Nab1	0,79
Meikin	0,79
6330403A02Rik	0,79
Mllt3	0,79
Creb3l1	0,79
2810403A07Rik	0,79
Fam92a	0,79
Fam168b	0,78
Hipk2	0,78
Tsga13	0,78
Vmn1r205	0,78
Ccsap	0,78
Far1	0,78
1700123O20Rik	0,78
Mup4	0,78
Dpcr1	0,78
BRDN0000738115	0,78
Psg22	0,87
1110059E24Rik	0,87
Mettl24	0,87
Rerg	0,87
Coq5	0,87
Zfp639	0,87
Cldnd2	0,87
Figla	0,87
Pxmp4	0,87
Atp11c	0,87
Olfr1047	0,87
BRDN0000737884	0,87
Gm21498	0,87
Ndufb2	0,87
Muc15	0,87
Tbck	0,87
Sh3bp4	0,87
Nwd1	0,87
Pigv	0,87
Rbfox1	0,87
Vmn2r74	0,87
Olfr45	0,87
Sntg1	0,87
Pde5a	0,86
Hes1	0,86
Ggt1	0,86
Rnf145	0,86
Kif3a	0,86
Tmem52b	0,86
Nceh1	0,86
Olfr522	0,86
Tuba1b	0,86
Olfr77	0,86
Sfrp2	0,86
Aldh3a2	0,86
2610015P09Rik	0,86
Trim10	0,86
Ebag9	0,86
Snx30	0,86
Nme1	0,86
Ptprz1	0,86
Slc45a1	0,86
Glt6d1	0,86
Gtpbp6	0,86
Gnai2	0,86
Crim1	0,86
Zbtb26	0,86
Hsd17b13	0,86
Ddx43	0,86
Clic3	0,86
Fmo4	0,86
Olfr397	0,86
Ccbl1	0,86
BRDN0000737807	0,86
Olfr781	0,86
Wnt7a	0,86
Ndufa4l2	0,86
Tigd4	0,86
Prkacb	0,86
Rnf208	0,86
Olfr1107	0,86
Tmem241	0,86
Spert	0,86
Neto2	0,86
Tbcb	0,86
Cdk5rap2	0,86
Yipf5	0,86
Olr1	0,86
Olfr1349	0,86
Baiap2l2	0,86
Tdp1	0,86
Ido1	0,86
Ampd3	0,86
Fra10ac1	0,86
Gm14548	0,86
Pon3	0,86
Nde1	0,86
Pak4	0,85
Ism2	0,85
Pcdhb9	0,85
Foxo4	0,85
BRDN0000737413	0,85
Olfr1382	0
Spin2c	0
Spin2d	0
Mmp8	0
Lyzl4	0
Fer1l4	0
Lyzl6	0
Lyzl1	0
Depdc1a	0
4930564B18Rik	0
Prelid1	0
Ccdc57	0
BRDN0000738123	0
Atad3a	0
Plk1	0
Cdc42ep2	0
Cdc42ep1	0
Plk2	0
Plk5	0
Kansl1l	0
Cdc42ep5	0
BRDN0000737986	0
Akt1s1	0
Mrps17	0
Ank1	0
Sptbn4	0
Sptbn2	0
Sptbn1	0
Kera	0
Nemf	0
Tiam2	0
Ocel1	0
Rrad	0
Leap2	0
Mat1a	0
Efhc2	0
Rsad2	0
Rras	0
Krtap19-3	0
Krtap19-1	0
Rpe	0
Neu2	0
Krtap19-4	0
Krtap19-5	0
Eif4ebp1	0
Bend5	0
C130050O18Rik	0
Sh2d1b1	0
BRDN0000738009	0
Smurf2	0
Sh2d1b2	0
Pdlim4	0
Pdlim5	0
Pdlim7	0
Pdlim1	0
Scn4b	0
Pdlim3	0
Tktl1	0
Tktl2	0
Tnni1	0
Bzrap1	0
Ppfibp1	0
Ppfibp2	0
BRDN0000737733	0
Hsf2bp	0
Gpaa1	0
Fopnl	0
BRDN0000738098	0
Ski	0
Ocrl	0
Elf4	0
Pcsk9	0
Rmdn2	0
Pnrc1	0
Pnrc2	0
Paics	0
Pcsk2	0
Mmgt1	0
Mmgt2	0
Krtap11-1	0
Pcsk6	0
Pcsk7	0
Mrc2	0,87
Slc25a5	0,87
Vmn2r57	0,87
Apoa4	0,87
Olfr479	0,87
Rftn2	0,87
Tex19.1	0,87
Ccdc7	0,87
Cntnap5a	0,87
Sall2	0,87
Pgam2	0,86
Cd3d	0,86
Bpgm	0,86
Hao1	0,86
Dpp4	0,86
Slc38a4	0,86
Vmn1r20	0,86
Slc25a35	0,86
Cdh9	0,86
Krt31	0,86
Gstm2	0,86
Fbn2	0,86
Grb14	0,86
Phykpl	0,86
Msx3	0,86
Ap5m1	0,86
Gpr137b	0,86
Sfrp5	0,86
Sipa1l3	0,86
Mypop	0,86
Plekhb1	0,86
Polk	0,86
Cdk14	0,86
Mcur1	0,86
Rnf113a1	0,86
Actr8	0,86
BRDN0000738225	0,86
Nup37	0,86
Brinp1	0,86
Duoxa2	0,86
Bdkrb1	0,86
Sly	0,86
Gtpbp6	0,86
Adrbk2	0,86
Gm16451	0,86
Ccdc125	0,86
Baz2b	0,86
Cngb1	0,86
Fgfr1	0,86
Cks1b	0,86
Syt7	0,86
Prm2	0,86
Crisp1	0,86
Creb3l2	0,86
Il17rd	0,86
Als2cr12	0,86
Sparcl1	0,86
Fam187a	0,86
Recql	0,86
Arl6ip1	0,86
Gla	0,86
Pou3f1	0,86
Neil1	0,86
Fam101b	0,86
Zfp709	0,86
Lyzl4	0,86
Srsf9	0,86
Gpcpd1	0,86
BRDN0000738107	0,86
Ppp1r35	0,86
Lancl1	0,86
Gm8882	0,86
Atp12a	0,86
Zfp703	0,86
Rftn1	0,86
Smim12	0,86
Spata31d1a	0,85
Mgst3	0,85
Zap70	0,85
Cylc1	0,85
Olfr1213	0,85
Fbxo33	0,85

Spink11	0,78
Olfr1386	0,78
Spaca4	0,78
Sfxn2	0,78
Diap3	0,78
Frrs1l	0,78
Acp6	0,78
Meig1	0,78
Olfr1340	0,78
Camk1g	0,78
Krtap19-3	0,78
Tyw3	0,78
Zfp202	0,78
Smco4	0,78
Psg25	0,78
Olfr963	0,78
E230008N13Rik	0,78
Cpt2	0,78
Slc6a8	0,78
Habp4	0,78
Col15a1	0,78
Sorl1	0,78
Ttyh3	0,78
Nalcn	0,78
Jakmip1	0,78
Siah3	0,78
Fut7	0,78
Samd7	0,78
Adam11	0,78
Prrt3	0,78
1700106J16Rik	0,78
Cwf19l1	0,77
Cd48	0,77
Pcsk1	0,77
Dgkh	0,77
Plod3	0,77
Mgat3	0,77
Ap5z1	0,77
Ccdc117	0,77
Ces2c	0,77
Gpr18	0,77
8430419L09Rik	0,77
Eri2	0,77
Pcdhgb1	0,77
Myl12b	0,77
Snapc5	0,77
Akap2	0,77
Tnfsfm13	0,77
Olfr110	0,77
Rcc2	0,77
Gpx4	0,77
Hipk1	0,77
Gpr84	0,77
Cttnbp2nl	0,77
Snx18	0,77
Pcdhgc5	0,77
Vmn1r101	0,77
BC089597	0,77
Arhgap27	0,77
Etv6	0,77
Bad	0,77
Styxl1	0,77
Il11ra1	0,77
Klf14	0,77
Acnat2	0,77
Tpmt	0,77
Zfp52	0,77
Mamdc4	0,77
Muc1	0,77
Olfr1026	0,77
Tcaim	0,77
Gm21541	0,77
Pa2g4	0,77
Armc10	0,77
Vmn1r170	0,77
Lcp1	0,77
Cat	0,77
Gfod2	0,77
Cryzl1	0,77
Osgepl1	0,77
Mcc	0,77
Treh	0,77

BC100451	0,85
Vmn1r29	0,85
Otp	0,85
Dtx3	0,85
Rnf125	0,85
Gli2	0,85
Phyhd1	0,85
Gm3238	0,85
Art2b	0,85
Sh3bp5l	0,85
Furin	0,85
Zfp456	0,85
Plch2	0,85
Vmn1r90	0,85
Ggct	0,85
9130023H24Rik	0,85
Babam1	0,85
Scml2	0,85
Gm10778	0,85
Sp9	0,85
Akap13	0,85
Pcsk4	0,85
Ctag2	0,85
Ccdc64b	0,85
Gm4303	0,85
Gm21002	0,85
Krtap14	0,85
Tas2r122	0,85
Slc52a2	0,85
Pipox	0,85
Fam3b	0,85
Mmel1	0,85
Adprh	0,85
Tspan1	0,85
Ociad2	0,85
Atp4b	0,85
Olfr43	0,85
Brd7	0,85
Lgi4	0,85
Amn1	0,85
Lysmd3	0,85
Prr5l	0,85
Apeh	0,85
Traf1	0,85
Trpa1	0,85
Asic5	0,85
Kcnk1	0,85
Apom	0,85
Fam83c	0,85
Pigc	0,85
Trem2	0,85
Olfr66	0,85
Pdcl3	0,85
Cd52	0,85
Dscaml1	0,85
Slc8b1	0,85
Masp1	0,85
Flt1	0,85
Ndrg1	0,85
Chst15	0,85
Nufip2	0,85
Polr3e	0,85
H2-M10.1	0,85
Fam13a	0,85
Sec22c	0,85
Akr1b8	0,85
Fam227a	0,85
Med18	0,85
Fcrls	0,85
Ndnf	0,85
Itpr3	0,85
Taok1	0,85
Espl1	0,85
Cast	0,85
Krtap4-2	0,85
Alox12b	0,85
Olfr1451	0,85
Efcab5	0,85
Prcc	0,85
Chchd6	0,85
Ankdd1b	0,85
Qpctl	0,85

Trp53tg5	0
Card14	0
D5Ertd577e	0
Scoc	0
Sptlc2	0
Sptlc3	0
Adig	0
Usp2	0
Cox17	0
Tardbp	0
C330021F23Rik	0
Gm16451	0
Usp3	0
Gm13177	0
Zzz3	0
1700084C01Rik	0
Gm13178	0
Usp5	0
Zfp13	0
Rab22a	0
Henmt1	0
Nup43	0
Mterf1a	0
9330182L06Rik	0
Lmod1	0
Cdk2ap2	0
Lmod2	0
Adi1	0
Pcsk1n	0
Tstd3	0
BRDN0000737983	0
Arsi	0
Pou2af1	0
Bcl2	0
Nolc1	0
Pzp	0
Rps15a	0
Pdk3	0
Pdk2	0
Triml2	0
Pdk4	0
Dsg2	0
Dsg3	0
Rprd2	0
Oplah	0
Dsg4	0
Adamts2	0
Grpel1	0
Grpel2	0
Pom121l2	0
6030458C11Rik	0
Fgd4	0
Il12rb1	0
Cyc1	0
Il12rb2	0
Meis3	0
Meis1	0
Fgd6	0
1700088E04Rik	0
Fcgrt	0
Prkrir	0
Samsn1	0
Ces1a	0
Ces1c	0
Scn5a	0
Ces1e	0
Ces1d	0
Hcst	0
Ces1f	0
Hif3a	0
Pecr	0
Fgd3	0
Vmn2r38	0
Tmem11	0
Calr3	0
Ckb	0
Ptgr1	0
Ptgr2	0
Golga1	0
Rwdd4a	0
Golga3	0
Golga4	0

Enah	0,85
Plekha5	0,85
Obscn	0,85
Arf5	0,85
Frs3	0,85
Olfr1507	0,85
4931431F19Rik	0,85
Atic	0,85
Rccd1	0,85
Hoxb6	0,85
Rab29	0,85
5430421N21Rik	0,85
BRDN0000738128	0,85
Tnik	0,85
Gdpd4	0,85
Bmper	0,85
Elfn2	0,85
Spaca3	0,85
Vmn1r120	0,85
Scube3	0,85
Abhd8	0,85
Clcn1	0,85
Fam81a	0,85
Cdc25c	0,85
Gm5941	0,85
Scgb2b26	0,85
Cyp2c40	0,85
Clec2h	0,85
Dnajc27	0,85
Numa1	0,85
Prosc	0,85
Scrn2	0,85
Tmpo	0,85
Col9a1	0,85
Eno3	0,85
Frmpd1	0,85
D630033O11Rik	0,85
Slc39a6	0,85
Ndufb3	0,85
Lsp1	0,85
Dgkd	0,85
Ghrl	0,85
Cnp	0,85
Sox4	0,85
Ar	0,85
Ccdc172	0,85
Ccdc134	0,85
Zscan21	0,85
Cactin	0,85
Ccer1	0,85
Itih5	0,85
Cx3cl1	0,85
Ttc39b	0,85
Rps6	0,85
Ackr2	0,85
Vmn2r26	0,85
Rps23	0,84
Rere	0,84
1700025G04Rik	0,84
Brwd1	0,84
Tspan6	0,84
Lgr4	0,84
Snap25	0,84
Magohb	0,84
Vmn1r19	0,84
Gatad1	0,84
Tnfrsf13b	0,84
Apof	0,84
Col23a1	0,84
Rab2a	0,84
Zdhhc7	0,84
Ncoa1	0,84
Retsat	0,84
Krt34	0,84
Gspt2	0,84
A630073D07Rik	0,84
Sass6	0,84
Pdgfb	0,84
Klk1b1	0,84
Aurkc	0,84
4933416C03Rik	0,84
Kcnip1	0,84

Arfgef2	0,77
Rufy2	0,77
Rnf125	0,77
Zfp773	0,77
Dclre1b	0,77
Nup35	0,76
4931408C20Rik	0,76
Mrps26	0,76
Man2b2	0,76
Pgrmc2	0,76
Zbed3	0,76
BC048671	0,76
Lpar1	0,76
Cltc	0,76
Cope	0,76
Slc25a27	0,76
Slfn2	0,76
Alms1	0,76
Grem2	0,76
Tfam	0,76
Olfr285	0,76
Gja3	0,76
Rbl1	0,76
Nek8	0,76
Lcmt2	0,76
Bcl2l14	0,76
Krtap17-1	0,76
Rrbp1	0,76
Kcne4	0,76
Arhgap33	0,76
2410012M07Rik	0,76
Clca4a	0,76
Chst7	0,76
Hcar1	0,76
Senp1	0,76
Kiss1r	0,76
Olfr10	0,76
Rhd	0,76
Lancl1	0,76
Cox6a1	0,76
Ano4	0,76
Ugp2	0,75
Tspan33	0,75
Spon1	0,75
Vmn1r125	0,75
Zfp46	0,75
Gm13152	0,75
Zscan10	0,75
BRDN0000737857	0,75
Commd2	0,75
Derl3	0,75
Scgb1a1	0,75
Pcdh20	0,75
Myo18b	0,75
Dhh	0,75
Hmbs	0,75
Igfbp7	0,75
Nepn	0,75
Gm7861	0,75
4933402P03Rik	0,75
Pbp2	0,75
Pnpla3	0,75
Zdhhc19	0,75
Olfr730	0,75
Ngfrap1	0,75
Cldn3	0,75
Gjd3	0,75
Slc32a1	0,75
Pafah2	0,75
Gid8	0,75
Gif	0,75
Nxpe4	0,75
Zfp738	0,75
Gm13084	0,75
Fcrl6	0,75
Car11	0,75
Rtn4	0,75
Glul	0,75
Foxo4	0,75
Atg16l2	0,75
Ankef1	0,75
Olfr691	0,75

Gm14743	0,85
Parp11	0,85
Olfr1426	0,85
Sun1	0,84
Gdf15	0,84
Rapgef3	0,84
Srd5a3	0,84
BRDN0000737919	0,84
Rgl1	0,84
BRDN0000738113	0,84
Kif3c	0,84
Adamtsl1	0,84
Nfya	0,84
Scn5a	0,84
Klhl13	0,84
Zfp513	0,84
Cyp4a31	0,84
Mtg2	0,84
Adora1	0,84
Nepn	0,84
Dnttip2	0,84
Cdip1	0,84
Tmed3	0,84
Vmn1r167	0,84
Cdk5rap1	0,84
Arsb	0,84
Rfxap	0,84
Rep15	0,84
Egr3	0,84
Olfr895	0,84
Gemin6	0,84
Gm20767	0,84
Fam213b	0,84
Scgn	0,84
Olfr1000	0,84
Plekhg3	0,84
Rasa4	0,84
Plvap	0,84
Efcab1	0,84
Limk2	0,84
Ccdc88b	0,84
Trove2	0,84
Pbld1	0,84
Nutm1	0,84
Rad23a	0,84
Olfr283	0,84
Ropn1l	0,84
Angpt2	0,84
Olfr514	0,84
Zfp866	0,84
Aup1	0,84
Fez1	0,84
Hypm	0,84
Cxcr6	0,84
Clic5	0,84
Sgsm2	0,84
Vmn2r11	0,84
Cerk	0,84
Rpl29	0,84
Pif1	0,84
H2-M10.4	0,84
BRDN0000737652	0,84
Rnf207	0,84
Ntmt1	0,84
Chia1	0,84
Safb2	0,84
Sh2d1b1	0,84
Tmem211	0,84
Olfr923	0,83
Olfr493	0,83
Msr1	0,83
Map9	0,83
Tmco1	0,83
Amz2	0,83
Tchh	0,83
Polm	0,83
Kcnk16	0,83
Acsl3	0,83
2700029M09Rik	0,83
Rab2a	0,83
Sumf2	0,83
Zfp663	0,83

Golga5	0
Gmfg	0
Cklf	0
Lmnb2	0
1700067K01Rik	0
Lmnb1	0
Nacc2	0
Pdzk1ip1	0
Mrpl43	0
Adgrf4	0
Sgk1	0
BRDN0000738183	0
Ring1	0
Nme7	0
Hsp90aa1	0
BRDN0000738006	0
Ndp	0
Vps51	0
Cisd2	0
Cisd3	0
Aaas	0
Cisd1	0
Fam49a	0
Mctp1	0
Tbk1	0
Fam49b	0
Rbfa	0
1700019D03Rik	0
Rbp2	0
Med10	0
Med11	0
Med12	0
Med13	0
Med14	0
Med15	0
Med17	0
Med18	0
Med19	0
Crxos	0
Egln2	0
Srfbp1	0
Nme8	0
Mamdc4	0
Rbp7	0
Mamdc2	0
Nyx	0
Osbpl1a	0
Phkg2	0
Samt2	0
Bop1	0
Ece2	0
Ece1	0
Ces2f	0
Vps13d	0
Lrrc3b	0
Soga3	0
Fam83e	0
Vps13c	0
Vps13b	0
Rptor	0
Alpk3	0
Alpk2	0
Gja4	0
Gja5	0
C2cd2l	0
Gja8	0
Cttnbp2	0
Nr5a1	0
Kif18a	0
Kif18b	0
Samt3	0
C330007P06Rik	0
Sgms1	0
Rhox12	0
Rhox13	0
Rhox10	0
Olfr156	0
Pik3ip1	0
Lrrc38	0
Twf1	0
Tcea3	0
Gm9733	0

Arhgap12	0,84
Olfr25	0,84
Sgol2a	0,84
Olfr356	0,84
Tnfaip2	0,84
Kcna6	0,84
Eif4b	0,84
Stpg2	0,84
Pnkd	0,84
Atp1a2	0,84
Cnga4	0,84
Pmf1	0,84
Srrm4	0,84
Prickle1	0,84
S100a8	0,84
Poc1b	0,84
Flt3l	0,84
Rnf169	0,84
Tmc7	0,84
Cdh3	0,84
Neurod1	0,84
Cldn23	0,84
Rnf11	0,84
Krt33a	0,84
Exd2	0,84
Daglb	0,84
Inhba	0,84
E030030I06Rik	0,84
Bet1l	0,84
Chic2	0,84
Trim42	0,84
Pemt	0,84
Sspn	0,84
Rnf123	0,84
Sox21	0,84
1110051M20Rik	0,84
Ugt2b34	0,84
Amn1	0,84
Tmem218	0,84
Ccdc71	0,84
Ifna2	0,84
Ank3	0,83
Apobr	0,83
Pigx	0,83
Morf4l2	0,83
Mrps23	0,83
Ppp6r1	0,83
Vwa8	0,83
BRDN0000737547	0,83
Zc3h7b	0,83
Psd	0,83
Olfr1126	0,83
Chst4	0,83
Map3k6	0,83
Emc3	0,83
Cst3	0,83
Arhgef5	0,83
Epha10	0,83
Slc2a7	0,83
Zfp827	0,83
Atg4b	0,83
Sidt2	0,83
Ifna16	0,83
Cep85l	0,83
Capn6	0,83
Pcmt1	0,83
Mthfr	0,83
Exosc1	0,83
Dtx3	0,83
Slc22a28	0,83
Mtr	0,83
Dck	0,83
Gm5168	0,83
Tmprss9	0,83
Sntg2	0,83
Olfr1275	0,83
C1qc	0,83
Pramel5	0,83
Vps26a	0,83
Nfatc2	0,83
Plcb3	0,83
Mmp14	0,83

Sgpp1	0,75	Ybey	0,83	Fam174b	0	Sdhaf2	0,83
Mylk	0,75	Olfr813	0,83	Fam174a	0	Psd2	0,83
Ell3	0,75	Abca16	0,83	Vsx2	0	Trappc9	0,83
H2afy2	0,75	Olfr304	0,83	Chrdl1	0	Vcan	0,83
Ndufs7	0,74	Satl1	0,83	Pkmyt1	0	Zfp940	0,83
Rpgrip1	0,74	Olfr315	0,83	Chrdl2	0	Wnt10a	0,83
Gnal	0,74	Il9r	0,83	BRDN0000738082	0	Sidt1	0,83
Tacc3	0,74	Trim2	0,83	BRDN0000737796	0	Slc14a2	0,83
Ephx2	0,74	BRDN0000738126	0,83	BRDN0000738080	0	Olfr470	0,83
Pdyn	0,74	Shroom3	0,83	Dcstamp	0	Pcdhga10	0,83
Olfr1507	0,74	Pqbp1	0,83	Zfp592	0	Dnah11	0,83
Zfp711	0,74	Sort1	0,83	BRDN0000737791	0	Nup88	0,83
Trpc1	0,74	Prickle3	0,83	BRDN0000737790	0	Slc26a2	0,83
Fndc5	0,74	Vmn2r12	0,83	Cntfr	0	Psrc1	0,83
Olfr1271	0,74	Islr2	0,83	Abcc5	0	Nfatc3	0,83
Pan2	0,74	Lamc2	0,83	BRDN0000738088	0	Olfr1000	0,83
Cdr2	0,74	Lama1	0,83	Abhd14b	0	Lims1	0,83
Agtrap	0,74	Serpina7	0,83	Syndig1	0	Wdfy2	0,83
Gpr4	0,74	Gm5662	0,83	Abcc1	0	Slc6a20b	0,83
Tmem260	0,74	Zfp955b	0,83	Abcc2	0	Tex264	0,83
Evl	0,74	Card11	0,83	Fam210a	0	Suv420h1	0,83
Il18bp	0,74	Apoa2	0,83	Tmem125	0	Syne3	0,83
Camk2a	0,74	Slc2a4	0,83	Cog6	0	Tcte3	0,83
Tmem116	0,74	Tas2r106	0,83	Tmem127	0	Hip1r	0,83
Arhgap17	0,74	Slc46a3	0,83	Desi2	0	Mab21l2	0,83
Cpm	0,74	Col8a2	0,83	Trank1	0	Fignl2	0,82
Exoc1	0,74	Spata45	0,83	Tmem123	0	Ylpm1	0,82
Lrrc48	0,74	Enox2	0,83	Desi1	0	Trim16	0,82
Ccnc	0,74	Clgn	0,83	Prdm16	0	BRDN0000737487	0,82
Yaf2	0,74	Abcb10	0,83	Tacr3	0	Lpgat1	0,82
Snca	0,74	Ccdc124	0,83	Wipi1	0	Adgrg1	0,82
E330034G19Rik	0,73	Dopey1	0,83	Tmem129	0	Slc13a5	0,82
Lpin2	0,73	Fgb	0,83	Tmem128	0	7530416G11Rik	0,82
Mroh5	0,73	BRDN0000738351	0,83	Tacr2	0	Mospd1	0,82
Ism2	0,73	Csf3r	0,83	BRDN0000737988	0	Cr2	0,82
H2-Oa	0,73	Ambp	0,83	Prpf38a	0	Gm765	0,82
Tob1	0,73	Kptn	0,83	Nepn	0	Atg4d	0,82
Poc1b	0,73	Atl2	0,83	Kdr	0	Scn2a1	0,82
Vangl2	0,73	Dpcr1	0,83	Gm3776	0	Gp2	0,82
Olfr11	0,73	Olfr481	0,83	Olfr714	0	Smpd3	0,82
Ttc21b	0,73	Plekhf2	0,83	Nudcd3	0	Hps3	0,82
Aqp6	0,73	Shprh	0,83	Olfr716	0	Vmn1r79	0,82
Reep4	0,73	Slc22a29	0,83	Nudcd1	0	Glb1l	0,82
Nfib	0,73	Sorl1	0,83	Olfr710	0	Pcdhga8	0,82
Tpm2	0,73	Fam109a	0,83	Gm2030	0	Ebf4	0,82
Cops7b	0,73	Stom	0,83	BRDN0000738228	0	Heatr3	0,82
Olfr77	0,73	Itm2c	0,83	Olfr713	0	Chfr	0,82
Natd1	0,73	Olfr58	0,83	BRDN0000738226	0	Foxo1	0,82
Ip6k2	0,73	Bud13	0,83	BRDN0000738227	0	Akt1	0,82
Atp8b3	0,73	BRDN0000737901	0,83	BRDN0000738224	0	Dnaaf3	0,82
Hgd	0,73	Pglyrp3	0,83	Ift74	0	Adar	0,82
Rbm41	0,73	Asic3	0,83	BRDN0000738222	0	BRDN0000738250	0,82
Ckm	0,73	Ppp1r37	0,83	BRDN0000738223	0	Rac3	0,82
Arl4a	0,73	Tldc1	0,83	BRDN0000738220	0	Vmn2r82	0,82
Gm11757	0,73	Arr3	0,83	Preb	0	BRDN0000738294	0,82
Crebrf	0,73	Cd28	0,83	Igfbp2	0	Olfr70	0,82
Eid1	0,73	Idua	0,83	Igfbp3	0	Gimap8	0,82
Poldip2	0,73	Man1b1	0,83	Igfbp6	0	Scgb1b7	0,82
Wnt11	0,73	Ugt8a	0,83	Celf1	0	Zkscan3	0,82
Cd177	0,73	Tgm2	0,82	Igfbp4	0	Cmtm5	0,82
Mcidas	0,73	Dtx2	0,82	Igfbp5	0	Hp1bp3	0,82
Epgn	0,73	Oxct2a	0,82	Pkhd1	0	Tbx15	0,82
BRDN0000738364	0,73	Rbm26	0,82	Rnasek	0	Ifngr2	0,82
Pxdn	0,72	Aqp6	0,82	Prep	0	Wls	0,82
A830010M20Rik	0,72	Actbl2	0,82	Myf5	0	2200002J24Rik	0,82
Qdpr	0,72	Olfr1167	0,82	2610002J02Rik	0	Smim8	0,82
Timm17b	0,72	Vwf	0,82	Slc25a22	0	Fam199x	0,82
Elovl6	0,72	Vstm5	0,82	L2hgdh	0	Aldh7a1	0,82
Lgi3	0,72	Fbxl13	0,82	Tars2	0	H2-M10.4	0,82
Tjp1	0,72	Itpr2	0,82	Myf6	0	Lhcgr	0,82
Fam167a	0,72	Nmnat1	0,82	Gm10142	0	Ebf1	0,82
Tnnt1	0,72	Ppp1r9b	0,82	Fam72a	0	Olfr124	0,82
Ces3b	0,72	Btg1-ps1	0,82	Gm10147	0	BRDN0000737570	0,82
Abca14	0,72	Hnf1a	0,82	BRDN0000737635	0	Diablo	0,82
Gnb1l	0,72	BC051665	0,82	Higd1c	0	Stt3b	0,82
Rnf208	0,72	Olfr741	0,82	Higd1b	0	Nadk	0,82
Vti1b	0,72	Foxn2	0,82	BC089491	0	Gdf9	0,82
Pithd1	0,72	Arhgef7	0,82	Slc25a21	0	Myo19	0,82
Npm3	0,72	Tmem255a	0,82	Akap5	0	Vmn1r220	0,82
Ackr3	0,72	Cmc1	0,82	Tpp1	0	H2-M2	0,82
Cdh11	0,72	Ptk6	0,82	Tnks1bp1	0	Rlf	0,82
Sftpa1	0,72	Mocs2	0,82	Tsc1	0	Myd88	0,82

2200002J24Rik	0,72	Sptbn4	0,82	Memo1	0	Fam53b	0,82
Mettl25	0,72	Ndufb6	0,82	Brix1	0	Zscan22	0,82
Zc3h3	0,72	Scn8a	0,82	BRDN0000737595	0	Shmt2	0,82
Dnajc19	0,72	Scmh1	0,82	0610040J01Rik	0	Pcdhb2	0,82
Olfr78	0,72	Pcdhgb7	0,82	Gm11437	0	1700030J22Rik	0,82
Hyal4	0,72	Camk1g	0,82	Rnase9	0	Dip2c	0,82
Hao1	0,72	Gm15293	0,82	Ap4m1	0	Pdzph1	0,82
Soga1	0,72	Elmod3	0,82	Hvcn1	0	Chpf	0,82
Atp13a1	0,72	Cyp51	0,82	Ssx2ip	0	Lect1	0,82
Pbx1	0,72	Acin1	0,82	C1d	0	Erg	0,82
Zmat2	0,72	Olfr889	0,82	Frrs1l	0	B3galt1	0,82
Ibtk	0,72	Afp	0,82	Nop10	0	Hs3st2	0,82
1700012B07Rik	0,72	Zfp160	0,82	Usp31	0	Olfr487	0,82
March1	0,72	Bpifa6	0,82	Nop14	0	Olfr613	0,82
Pawr	0,72	Mrgprh	0,82	Nop16	0	Unc13b	0,82
Mnda	0,72	Cwc25	0,82	Sult1e1	0	Ssr2	0,82
Bmf	0,72	Dynlt3	0,82	Atg9a	0	Nmi	0,81
Sowaha	0,72	Angptl1	0,82	Atg9b	0	Muc5b	0,81
Gpsm2	0,72	Hip1	0,82	Efcab12	0	Abr	0,81
Hccs	0,72	Olfr959	0,82	Efcab14	0	Lgals7	0,81
Gpr26	0,72	Anxa11	0,82	Blnk	0	Rras2	0,81
Syngr2	0,72	Dlat	0,82	Ociad2	0	Tspan32	0,81
Gm5460	0,72	Zfp580	0,82	Olfr1366	0	Slc51b	0,81
Olfr220	0,72	Pigh	0,82	BRDN0000737528	0	1200014J11Rik	0,81
Gramd1a	0,72	Sergef	0,82	Olfr1362	0	Rbks	0,81
Padi2	0,72	BRDN0000737868	0,82	Olfr1361	0	5031410I06Rik	0,81
Nfrkb	0,72	Ogfod3	0,82	Olfr1360	0	Olfr1287	0,81
Zfp30	0,71	Zfp568	0,82	Ccdc88b	0	Dcun1d2	0,81
BRDN0000737464	0,71	Vmn1r87	0,82	Actr10	0	Aff3	0,81
L3mbtl1	0,71	Ctsa	0,82	BRDN0000737411	0	Tubb2b	0,81
Fgf17	0,71	Kcnab3	0,82	Xirp1	0	Btk	0,81
3110035E14Rik	0,71	Lefty2	0,82	Plekhm1	0	Gtf2a2	0,81
2200002D01Rik	0,71	Vsig2	0,82	Plekhm2	0	Tbc1d19	0,81
D2hgdh	0,71	Tchhl1	0,82	P3h4	0	Olfr891	0,81
Scnn1g	0,71	Dact2	0,82	Cacfd1	0	Unkl	0,81
2010002M12Rik	0,71	Pyurf	0,82	Eya3	0	Olfr469	0,81
Fam73a	0,71	Npb	0,82	Fam120c	0	Rbm48	0,81
Pgf	0,71	Tvp23b	0,82	Cdc45	0	2610507B11Rik	0,81
BRDN0000738006	0,71	Zc3hav1l	0,82	Cdk5r1	0	Lipf	0,81
Lrrc74a	0,71	Tmem173	0,82	Eya2	0	AI987944	0,81
Sbpl	0,71	Tyrobp	0,82	Bod1	0	Bace1	0,81
Eml6	0,71	Cd44	0,82	Cdc40	0	Tdpoz3	0,81
Arhgap6	0,71	Sh3gl1	0,82	Cdc42	0	Parp10	0,81
Olfr304	0,71	Ahsg	0,82	Tas2r123	0	Apobec4	0,81
Wfdc10	0,71	Insl6	0,82	Tas2r121	0	Ptx3	0,81
Sergef	0,71	Kcnj8	0,82	Tas2r120	0	Mdm4	0,81
Slc26a3	0,71	Rnf180	0,82	Tma7	0	S100g	0,81
Olfr555	0,71	Prss30	0,82	Tas2r125	0	Dpy19l4	0,81
Klhl13	0,71	Wnt8a	0,82	Tas2r124	0	Zfp39	0,81
Ccdc57	0,71	Cyp2c38	0,82	Tas2r129	0	Nek7	0,81
Cryba2	0,71	Ano4	0,82	Adap1	0	Notch2	0,81
Nqo2	0,71	Rln3	0,82	Psap	0	Sec61b	0,81
Tceal8	0,71	Ube2v2	0,82	Olfr538	0	9930012K11Rik	0,81
Alcam	0,71	Hnmt	0,82	2010003K11Rik	0	Shmt1	0,81
Gm10228	0,71	1700040L02Rik	0,81	Mrpl48	0	Pcdhb9	0,81
Grin2a	0,71	Sftpa1	0,81	BRDN0000738175	0	Nyx	0,81
Magi2	0,71	Gskip	0,81	Mb21d1	0	Vmn2r63	0,81
Kctd14	0,71	Ankmy2	0,81	Mb21d2	0	Trpc7	0,81
C8b	0,71	Scarb2	0,81	Blvrb	0	Nbas	0,81
Emx1	0,71	Fbxo18	0,81	Olfr845	0	Il17re	0,81
Myh7b	0,71	Clec4n	0,81	Olfr846	0	Otoa	0,81
4931406C07Rik	0,71	Aoc1	0,81	Taf8	0	Mettl7a2	0,81
Acbd4	0,71	Synpo2	0,81	BRDN0000737596	0	Gatm	0,81
Krt6b	0,71	Mtm1	0,81	Olfr843	0	Scai	0,81
Tnr	0,71	Clec2h	0,81	BRDN0000738251	0	Pogk	0,81
Tcea3	0,71	Tex14	0,81	BRDN0000737631	0	Ppp1r3a	0,81
Hsf2	0,71	Nox3	0,81	Map2k2	0	Fezf2	0,81
Cr2	0,71	Sv2c	0,81	Fam204a	0	Kctd7	0,81
Mylk3	0,71	Plaur	0,81	Setx	0	Lrp12	0,81
Itih3	0,71	Ccdc71	0,81	Rhpn2	0	Msh2	0,81
Elof1	0,71	Vmn1r177	0,81	Map2k1	0	Aptx	0,81
Eddm3b	0,71	Abcc9	0,81	Mlh1	0	Casp3	0,81
Taf8	0,71	Poldip2	0,81	Ltn1	0	Vmn1r231	0,81
Chrac1	0,71	Il15	0,81	Gtf2a1	0	Socs2	0,81
Pilra	0,71	Slc16a2	0,81	Gtf2a2	0	Mrpl30	0,81
Olfr44	0,71	Igbp1b	0,81	Isg20	0	Maf	0,81
Olfr1156	0,71	Omp	0,81	Fam64a	0	Smc5	0,81
Cnbp	0,70	Trib3	0,81	Gipc2	0	Dhrs9	0,81
Armc8	0,70	Dgkg	0,81	Gipc3	0	Krt20	0,81
Rab22a	0,70	Vmn2r106	0,81	Otub1	0	Kcnb2	0,81
Pmepa1	0,70	Cdk15	0,81	4930596D02Rik	0	Nkx2-9	0,81
Gsdma2	0,70	Zfp868	0,81	Otub2	0	Gtf3c3	0,81

Fbxl7	0,70	Rfx3	0,81	Elavl4	0	Pigf	0,81
Sell	0,70	Tspan12	0,81	Nlrp6	0	Bcl6	0,81
Ern2	0,70	Vmn2r114	0,81	Elavl1	0	Shroom3	0,81
Wdr26	0,70	Gdf6	0,81	Elavl2	0	Acadl	0,81
Olfr1257	0,70	Rhov	0,81	Elavl3	0	BRDN0000738215	0,81
Mfap3	0,70	Dap	0,81	Gm2913	0	Tubb4b	0,81
Rgs14	0,70	Calr4	0,81	Map2k4	0	Ccdc8	0,81
Slc4a11	0,70	Ppp2r3a	0,81	Cyb5a	0	Asb16	0,81
Wipf2	0,70	Hoxb13	0,81	Tigit	0	Olfr77	0,81
Lipc	0,70	Cnot6l	0,81	Zdhhc2	0	Iqcj	0,81
Hpx	0,70	Olfr399	0,81	Upf2	0	Vmn2r77	0,81
Snapc3	0,70	Skap1	0,81	Mdh2	0	Appl2	0,81
BRDN0000737759	0,70	Pbrm1	0,81	Mdh1	0	BRDN0000737584	0,81
Jup	0,70	Hmga1	0,81	Amn1	0	Gm11711	0,81
Olfr229	0,70	Ppif	0,81	Pacsin1	0	Fam149a	0,81
Ripply3	0,70	Hn1l	0,81	Pacsin3	0	Rcn2	0,81
Ascc3	0,70	Zfp334	0,81	Pacsin2	0	Kcna2	0,81
Mrps21	0,70	Tmem60	0,81	Wdr64	0	Gm906	0,81
Cacng8	0,70	Asxl2	0,81	BRDN0000738144	0	Esp34	0,80
Isca1	0,70	Clcn1	0,81	Dnajc9	0	Hist1h4b	0,80
Nek6	0,70	Olfr742	0,81	Kcnk18	0	Lgals3	0,80
Olfr1042	0,70	Art4	0,81	Olfr656	0	Glrb	0,80
Zswim3	0,70	Creg2	0,81	Dnajc5	0	4933434E20Rik	0,80
Neurl1b	0,70	Dazap1	0,81	Dnajc6	0	Cnn3	0,80
Tbc1d10a	0,70	1500015O10Rik	0,81	Kcnk12	0	Gsta2	0,80
Eif4ebp2	0,70	Rpa3	0,81	Kcnk10	0	Lgr5	0,80
Aldh5a1	0,70	Vat1	0,81	Dnajc3	0	Vmn1r176	0,80
Krtap3-1	0,70	Gja5	0,81	Fbrsl1	0	Kctd4	0,80
Vmn1r16	0,70	Dctn1	0,81	Gpn2	0	BRDN0000738183	0,80
Khk	0,70	Fbxl15	0,81	Gpn1	0	Tspan8	0,80
Hps4	0,70	Olfr930	0,81	Timm8a1	0	Sgpp1	0,80
Skor1	0,70	Dkk1	0,81	Myc	0	Piezo2	0,80
Dis3l2	0,70	Sdc2	0,81	Myb	0	Cabp4	0,80
Mxd1	0,70	Hist1h1e	0,81	Asprv1	0	Kirrel2	0,80
Sh3glb1	0,70	Syn3	0,81	Fam53a	0	Casc4	0,80
Plac9a	0,70	Cox6c	0,81	Twist2	0	Cemip	0,80
Rsbn1l	0,69	Mrps9	0,81	Bhmt	0	Kcnh5	0,80
Myof	0,69	Sult2a3	0,81	2200002J24Rik	0	1700030F18Rik	0,80
2010107G12Rik	0,69	Dnajb6	0,81	Pccb	0	Tyr	0,80
Gbp7	0,69	Olfr1023	0,81	Rogdi	0	4931428F04Rik	0,80
Rprml	0,69	Atad5	0,81	Masp1	0	Cd2ap	0,80
BRDN0000737478	0,69	Pon1	0,81	Olfr1126	0	Olfr137	0,80
Hmgn3	0,69	Colgalt2	0,81	Fam19a3	0	Slc45a4	0,80
Vmn2r105	0,69	Fchsd2	0,81	Tlx2	0	Ccl27a	0,80
Tacc1	0,69	Igsf21	0,81	Nsg1	0	Ccdc142	0,80
Csnk1a1	0,69	Pmel	0,81	Mmp9	0	U2af1l4	0,80
Ndufa13	0,69	Rnaseh1	0,81	Naaladl1	0	2010003K11Rik	0,80
Vmn1r197	0,69	Olfr202	0,81	Fam124b	0	Hist1h4i	0,80
Safb	0,69	Ccng2	0,81	Olfr539	0	Mbnl2	0,80
Gcnt7	0,69	Adamts19	0,81	Fam124a	0	Tmem150a	0,80
Sertad3	0,69	Klf12	0,81	Daam1	0	A630076J17Rik	0,80
Mmp24	0,69	Trh	0,81	Daam2	0	4930415L06Rik	0,80
Krt27	0,69	Bmpr2	0,81	Rnaseh1	0	Fbxo39	0,80
Cox4i2	0,69	Nek2	0,81	Mocos	0	Spaca5	0,80
Tmem230	0,69	Fzd9	0,81	Rpl3l	0	Tceal5	0,80
Igsf21	0,69	Prrt4	0,81	Prkacb	0	Tns3	0,80
Zbtb7c	0,69	Mfsd4	0,81	Zfp52	0	Abca15	0,80
Aldh1a1	0,69	Mterf4	0,80	Zfp53	0	Coro1c	0,80
Zfp40	0,69	BRDN0000737432	0,80	Zfp51	0	Krtap4-7	0,80
Zfp346	0,69	Htatsf1	0,80	Ubr4	0	Tprg	0,80
Rhoq	0,69	Mst1r	0,80	Tstd1	0	9830107B12Rik	0,80
Amigo1	0,69	0610009O20Rik	0,80	Zfp58	0	Sumo3	0,80
Fam83b	0,69	Arid4a	0,80	Osgep	0	Ccdc144b	0,80
Olfr1353	0,69	Slc25a47	0,80	Cyyr1	0	Agpat4	0,80
Stradb	0,69	Alg6	0,80	Nmb	0	Eif4ebp2	0,80
Rnf144b	0,69	Ngf	0,80	Grb10	0	Nap1l3	0,80
Nlk	0,69	Fam20a	0,80	Dus1l	0	Slc35b3	0,80
Tlx2	0,69	Usp38	0,80	Grik3	0	Sorcs2	0,80
Snrk	0,69	Fgf13	0,80	Ntmt1	0	Xpc	0,80
2300002M23Rik	0,69	Gprasp1	0,80	Baiap2l2	0	Plxdc1	0,80
Erp27	0,69	Enpp5	0,80	Rap1gds1	0	Mars2	0,80
Fbxl6	0,69	Cyp2c50	0,80	Il1f6	0	5430402E10Rik	0,80
Podn	0,69	Smco3	0,80	Gtf2b	0	4931429I11Rik	0,80
Ndn	0,69	Pnrc2	0,80	Ttbk2	0	Smarcc1	0,80
Pla2g2f	0,69	Prrt3	0,80	BRDN0000738032	0	Itfg1	0,80
Ermn	0,69	Wfs1	0,80	Plp2	0	Myc	0,80
Enox2	0,69	Lsr	0,80	Gtf2i	0	Gucy1b3	0,80
Krt2	0,68	Hspb9	0,80	Nbas	0	Bnc1	0,80
Sult4a1	0,68	Tbpl1	0,80	Zyx	0	Tnip2	0,80
2410004P03Rik	0,68	Olfr1008	0,80	Luc7l2	0	Sowahc	0,80
Renbp	0,68	Cdyl2	0,80	Jakmip2	0	Sbf1	0,80
Olfr221	0,68	Reg4	0,80	Jakmip1	0	Apc	0,80

Abca13	0,68	Kdm4d	0,80	Ttbk1	0	Olfr1331	0,80
Rab38	0,68	Abhd10	0,80	Olfr446	0	Vps8	0,80
Akip1	0,68	Gpm6b	0,80	Spint3	0	Rhox10	0,79
Inpp5a	0,68	D3Ertd751e	0,80	Scaf11	0	Limd2	0,79
Tmx1	0,68	Acsbg2	0,80	Nxpe2	0	Crispld1	0,79
Prok1	0,68	Olfr153	0,80	Ncor2	0	Blcap	0,79
Gria4	0,68	Zfp420	0,80	BRDN0000737872	0	Pmvk	0,79
Zbtb16	0,68	Lta4h	0,80	BRDN0000737873	0	Lhx8	0,79
Zfp608	0,68	Cyp2d10	0,80	BRDN0000737870	0	Ephx4	0,79
Cntnap3	0,68	Nrbp2	0,80	Nxpe3	0	BRDN0000738072	0,79
Gldn	0,68	Vps35	0,80	BRDN0000737876	0	Pdia2	0,79
Brox	0,68	Usp53	0,80	Egfl8	0	Elp4	0,79
Ppig	0,68	Ptchd3	0,80	BRDN0000737874	0	Kif2b	0,79
Dagla	0,68	Ybx3	0,80	Plcd1	0	Hus1b	0,79
Gabrr3	0,68	Ppig	0,80	Cd209b	0	Ets2	0,79
Cuedc2	0,68	Mpp7	0,80	BC022687	0	Ampd2	0,79
Slain1	0,68	Slc38a5	0,80	BRDN0000737878	0	Snrnp48	0,79
Tgm3	0,68	Slc6a8	0,80	Cd209a	0	Perm1	0,79
Gm3558	0,68	Atp1b3	0,80	Ncmap	0	Scube1	0,79
Nptx2	0,68	Vmn2r20	0,80	Cd209g	0	Hyal4	0,79
Col10a1	0,68	B9d1	0,80	Sgpp2	0	Tmem150c	0,79
Nhlrc1	0,68	Crispld1	0,80	Cd209e	0	Fnip1	0,79
Taf7l	0,68	Selt	0,80	BRDN0000737656	0	Uggt2	0,79
Uhrf1bp1	0,68	Sptbn1	0,80	Nfu1	0	2610002J02Rik	0,79
Spag11a	0,68	D330045A20Rik	0,80	Ppfia3	0	Tmem238	0,79
Adra1d	0,68	Trpc2	0,80	Sec13	0	Serpinb3a	0,79
Olfr169	0,68	Ldlrad3	0,80	Insig1	0	Olfr456	0,79
Cd109	0,68	Ankrd36	0,80	Lyrm2	0	Olfr749	0,79
Ramp2	0,68	Cnn2	0,80	Krr1	0	Cox7a1	0,79
Zfp947	0,67	Cypt12	0,80	Ereg	0	Prdm6	0,79
Otud3	0,67	Ttll11	0,80	Lce6a	0	Rgs13	0,79
Nfyb	0,67	Ccdc167	0,80	Ttc29	0	Crat	0,79
Tnnc2	0,67	Rnf41	0,80	Zfp213	0	Cyp2j11	0,79
Chic2	0,67	Ttc5	0,80	Gtf2f1	0	BRDN0000737420	0,79
Mark3	0,67	Taf7	0,80	Ankrd60	0	Grhl1	0,79
Msmb	0,67	Armc3	0,80	Sgpp1	0	Gucd1	0,79
Arhgef28	0,67	Scrn2	0,80	Gm13298	0	Gm15056	0,79
Nol4l	0,67	Pigl	0,80	BRDN0000737657	0	Nphp1	0,79
S100a16	0,67	Rnf121	0,80	Zfp330	0	Ubald2	0,79
Tjp2	0,67	Edn1	0,80	Gpr135	0	Ctxn2	0,79
Trp63	0,67	BRDN0000738187	0,80	Jmy	0	Hlx	0,79
H2bfm	0,67	Rnd3	0,80	Zfp334	0	9330159F19Rik	0,79
Arih2	0,67	Hnrnpa0	0,80	Zfp335	0	Cxxc4	0,79
Man2a1	0,67	Cx3cl1	0,80	Vmn1r124	0	Pomp	0,79
Slc51b	0,67	Defb5	0,80	Dytn	0	Mpo	0,79
Rhov	0,67	Invs	0,80	Foxe1	0	Rdh10	0,79
Ppm1f	0,67	Ano2	0,80	BRDN0000738151	0	Rexo1	0,79
Unc5c	0,67	BRDN0000738091	0,80	Crispld2	0	Rmdn1	0,79
Fam20a	0,67	Phlda1	0,80	Rnf149	0	Teddm1b	0,79
Gm5416	0,67	Creb3l3	0,80	Rnf148	0	Glyat	0,79
Ttc30a2	0,67	Vwa1	0,80	BRDN0000737997	0	Khsrp	0,79
Plau	0,67	Vmn1r57	0,80	Vmn1r126	0	Clec4a1	0,79
Azin2	0,67	Rab19	0,80	Prima1	0	Cacng6	0,79
Rassf2	0,67	Gsn	0,80	Stbd1	0	Syce3	0,79
Sp4	0,67	Opn1sw	0,80	Suz12	0	Amigo1	0,79
Inpp1	0,67	Srebf2	0,80	Gmip	0	Sh3yl1	0,79
Corin	0,67	Slc22a20	0,80	Rnf141	0	Foxd2	0,79
Invs	0,67	Hrasls5	0,80	Rnf145	0	Cystm1	0,79
Slc26a10	0,67	4933416C03Rik	0,80	Aicda	0	Lrp5	0,79
Tmed3	0,66	Cytl1	0,79	Rnf146	0	Layn	0,79
Mtx1	0,66	Chrna5	0,79	Rpp38	0	Tpst1	0,79
Zfp945	0,66	Wdr46	0,79	Slco3a1	0	Gng8	0,79
Pcdha12	0,66	Scgb3a2	0,79	Mlst8	0	Lmo3	0,79
Gm17660	0,66	Serpinf1	0,79	H2-DMa	0	Ttc4	0,79
Cyp2c69	0,66	Olfr775	0,79	Chmp4b	0	Vmn1r10	0,79
Gm10354	0,66	Zbtb7b	0,79	Vmn1r122	0	Fgf10	0,79
Slc22a22	0,66	Eif4e2	0,79	1810041L15Rik	0	Cnksr2	0,79
Gm15315	0,66	Pdcd2	0,79	BRDN0000737536	0	Fes	0,79
Ndufs8	0,66	Adgrf5	0,79	Ptchd4	0	Klri2	0,79
Appbp2	0,66	Tceb3	0,79	Ptchd3	0	Olfr1229	0,79
Tmprss3	0,66	Rab22a	0,79	Ptchd2	0	Olfr1505	0,79
Lrrc15	0,66	Dntt	0,79	Ptchd1	0	Gm4944	0,79
Dpyd	0,66	Prokr2	0,79	Tfpi	0	4933428G20Rik	0,79
Zfp747	0,66	Prkd2	0,79	Tbc1d12	0	Abhd2	0,79
Kcng4	0,66	Tcstv3	0,79	Tbc1d13	0	Zmynd10	0,79
Clec12a	0,66	Syngr1	0,79	BRDN0000737655	0	Relt	0,79
Olfr145	0,66	Cplx2	0,79	Tbc1d16	0	Csnka2ip	0,79
Olfr1097	0,66	Arrdc2	0,79	Tbc1d17	0	Olfr225	0,79
Morn5	0,66	Myg1	0,79	Tbc1d14	0	Shroom2	0,79
Opn1mw	0,66	Tox	0,79	Tbc1d15	0	Efemp2	0,79
Slc35f3	0,66	Olfr1026	0,79	Fam102b	0	BRDN0000737530	0,79
Ppm1g	0,66	BC061237	0,79	Rbmxl1	0	BRDN0000738030	0,79

Gm2696	0,66	Vmn1r38	0,79	Dlg3	0	BC003331	0,79
Spg11	0,66	Ms4a6d	0,79	Dlg5	0	Tbc1d14	0,79
Rpl9	0,66	Pth2r	0,79	Dlg4	0	Npffr1	0,79
Gimap9	0,66	Klk9	0,79	Supt7l	0	Zfp592	0,79
Trem3	0,66	Zmynd19	0,79	Gpr139	0	St13	0,79
Slc25a21	0,66	Svs3a	0,79	Olfr806	0	Iqcf6	0,79
Neurl1a	0,66	Coa5	0,79	Ppme1	0	Ccdc28a	0,79
Dcbld1	0,66	Wdr33	0,79	BRDN0000737535	0	Tril	0,79
Rapsn	0,66	Lamtor1	0,79	Gm4340	0	Csf2rb2	0,79
Frmd7	0,66	Rgs4	0,79	Olfr279	0	Shf	0,79
Zfp438	0,66	Chrm5	0,79	Ssu2	0	Defb13	0,79
Perm1	0,66	Spink13	0,79	Olfr275	0	Vmn1r216	0,79
Cyp3a11	0,66	Lrrcc1	0,79	Nlrp9a	0	Olfr850	0,79
Cdyl	0,66	Fbxw20	0,79	Odf3	0	Ech1	0,78
Btbd2	0,66	Cbx4	0,79	Olfr1173	0	Mib2	0,78
Gdf5	0,66	Sele	0,79	Odf1	0	Cdh19	0,78
Olfr447	0,66	9530002B09Rik	0,79	Fastkd5	0	Atp2b2	0,78
Cmah	0,66	BRDN0000737528	0,79	Fastkd2	0	Gstm3	0,78
Piezo2	0,66	Olfr26	0,79	Fastkd3	0	Olfr401	0,78
Nccrp1	0,66	Mmp25	0,79	Odf4	0	Fbxo21	0,78
2010109A12Rik	0,66	Mamstr	0,79	Cdx1	0	Olfr867	0,78
Adgre4	0,66	Nmu	0,79	Olfr1178	0	Pdcd1	0,78
Gm8693	0,66	Tcl1	0,79	Olfr1179	0	Mical1	0,78
Dlg5	0,66	Rab3a	0,79	6330403A02Rik	0	Adgra2	0,78
Il17rc	0,66	Tmprss12	0,79	BRDN0000737534	0	Epc2	0,78
Hist1h1c	0,66	Nox1	0,79	Zfp217	0	Gpr157	0,78
Scai	0,66	Zfp931	0,79	Skint11	0	Sgk1	0,78
Mtfr1	0,66	Lmbr1	0,79	Skint10	0	Vmn1r51	0,78
Jkamp	0,66	Ifitm2	0,79	BRDN0000737653	0	Qdpr	0,78
Esrp1	0,66	Barhl1	0,79	Senp2	0	Prss52	0,78
Foxj2	0,66	Bzw1	0,79	Mmaa	0	Cox7b	0,78
4933408B17Rik	0,65	Tgm6	0,79	BRDN0000738316	0	Rtkn	0,78
Tcte3	0,65	Fbxo45	0,79	Ube2cbp	0	Pskh1	0,78
Olfr401	0,65	Pomt1	0,79	Mmab	0	2210408I21Rik	0,78
Zcchc13	0,65	Saa3	0,79	Draxin	0	Tcl1b3	0,78
Podxl2	0,65	Zbtb33	0,79	BRDN0000738317	0	Ugt2b37	0,78
Clcn3	0,65	Golga1	0,79	Rgs7bp	0	Pnoc	0,78
Krtap6-2	0,65	Sipa1l2	0,79	Stab1	0	Raf1	0,78
Olfr224	0,65	Dnajb13	0,79	BRDN0000738314	0	Cdk5rap1	0,78
Pak7	0,65	Zfp85	0,79	Stab2	0	Crybb1	0,78
Rspo3	0,65	Rad18	0,79	Slc45a4	0	Atp5sl	0,78
Olfr360	0,65	Gmcl1	0,79	BRDN0000738315	0	Ercc6l	0,78
Ckmt1	0,65	Cnga4	0,79	Slc45a3	0	Lce1l	0,78
Myl2	0,65	Gucy1a2	0,79	Slc45a2	0	Gm6592	0,78
Rps6ka4	0,65	Inhbb	0,79	BRDN0000737494	0	Tff3	0,78
Snx11	0,65	Afap1	0,79	BRDN0000737495	0	Vmn1r116	0,78
Olfr571	0,65	1190005I06Rik	0,79	BRDN0000737496	0	Gbx1	0,78
Olfr1513	0,65	Ror1	0,79	Mast3	0	Pcbp2	0,78
Zfp804a	0,65	Cyp2b13	0,79	Trmt10a	0	Gimap9	0,78
Clptm1	0,65	Tmed1	0,79	BRDN0000737491	0	Ercc8	0,78
Doc2g	0,65	Dyrk4	0,79	BRDN0000737492	0	Praf2	0,78
Atp2a2	0,65	Abcc8	0,79	Olfr661	0	Crip3	0,78
Atg4d	0,65	Mrps2	0,79	Arhgap33	0	Dpt	0,78
Slco6c1	0,65	Dock7	0,79	Plek2	0	Ankib1	0,78
Gfm2	0,65	Olfr557	0,79	BRDN0000737498	0	Tmem151b	0,78
Il17re	0,65	Map6d1	0,79	BRDN0000737499	0	Map7d1	0,78
Otx2	0,65	Prss1	0,79	Musk	0	Rapgef3	0,78
Adamts16	0,65	Dpcd	0,79	Zranb1	0	Emcn	0,78
Ano10	0,65	Slc22a17	0,79	Vldlr	0	Zfp691	0,78
Pdzd4	0,65	1600014C10Rik	0,79	Zranb3	0	Hbq1b	0,78
Ciz1	0,65	Zfp882	0,79	2810006K23Rik	0	Fam78b	0,78
Olfr66	0,65	Atp1a2	0,79	BRDN0000737848	0	Lce3a	0,78
Pigk	0,65	Espnl	0,79	Ntrk1	0	Tmem200c	0,78
Micall2	0,65	Hectd2	0,79	BRDN0000737825	0	Cyp4f18	0,78
Crat	0,64	Jmjd6	0,79	BRDN0000737651	0	Asb15	0,78
Fpr1	0,64	Bend7	0,79	Esyt2	0	Gabrr3	0,78
Tmprss11f	0,64	Slc35g3	0,79	2610008E11Rik	0	Mycbp	0,78
Gm684	0,64	Trpv5	0,79	Ntrk3	0	Commd1	0,78
Ciao1	0,64	Rpl22l1	0,79	Efr3a	0	Cmc2	0,78
Ppp1r14a	0,64	Mob3b	0,79	Crnkl1	0	Rag1	0,78
Fancf	0,64	Ccdc7	0,79	Efr3b	0	Svs2	0,78
Rdh10	0,64	Il31ra	0,79	Fnbp1	0	Apol7e	0,78
Clvs2	0,64	Pcnx	0,79	Plekhs1	0	Clspn	0,78
Pnpla8	0,64	Tfap2c	0,78	Txk	0	Hnrnpu	0,78
Ptbp3	0,64	Tead2	0,78	Postn	0	Unc119	0,78
Tmem160	0,64	Mrgprb1	0,78	Zfpm2	0	Fam19a1	0,78
Kcnj11	0,64	Mcfd2	0,78	Zfpm1	0	Ift46	0,78
Map3k4	0,64	Prg4	0,78	Csmd3	0	Kcne3	0,78
Tas2r134	0,64	Cdc42bpg	0,78	Pou4f2	0	Usf2	0,78
Taok3	0,64	BRDN0000738041	0,78	Csmd1	0	Tsacc	0,78
Olfr960	0,64	Farsb	0,78	Lad1	0	Slc4a9	0,78
Scgb2b2	0,64	Mrpl54	0,78	Morf4l1	0	Htra4	0,78

Esyt1	0,64	Pddc1	0,78	Swi5	0	Kbtbd4	0,78
Elk3	0,64	Mapk14	0,78	BRDN0000738149	0	Dhcr24	0,78
Fcrlb	0,64	Atoh7	0,78	Gsdmc2	0	Ptgdr2	0,78
Htr2a	0,64	Ttc4	0,78	Gsdmc3	0	Fam84a	0,78
Ube2a	0,64	Lox	0,78	Tmem260	0	Rprd1a	0,78
Entpd7	0,64	Armt1	0,78	Rbbp9	0	Grk1	0,78
Specc1	0,64	Otos	0,78	Hpcal1	0	Hsd17b6	0,78
Ccdc146	0,64	BRDN0000738045	0,78	Gper1	0	Sac3d1	0,78
Med13l	0,64	Lck	0,78	Hpcal4	0	Zfp648	0,78
Stau1	0,64	Zfp597	0,78	Spatc1l	0	Adssl1	0,78
Olfr804	0,64	Fam117a	0,78	Enho	0	Bcor	0,78
Galk1	0,64	Kif2a	0,78	Mybbp1a	0	Prkrip1	0,78
Gm5622	0,64	Grik5	0,78	Ciapin1	0	Zfp521	0,78
Tbx6	0,64	Rbm15	0,78	Rbbp5	0	1810009A15Rik	0,78
Amigo2	0,63	Tex43	0,78	Hsd3b5	0	Cir1	0,78
Ssc5d	0,63	Pdk3	0,78	Hsd3b6	0	Dmbt1	0,78
Gsk3b	0,63	Abcd4	0,78	Hsd3b7	0	Mettl23	0,78
Trim26	0,63	Olfr876	0,78	4930452B06Rik	0	Ywhag	0,78
Per1	0,63	Arl5b	0,78	Hsd3b3	0	Gimap4	0,78
Tmem42	0,63	F2rl1	0,78	Ifna13	0	Ppp1r11	0,78
Hira	0,63	Spry2	0,78	Ifna12	0	BC094916	0,77
Dock10	0,63	Vim	0,78	Ifna11	0	Pus3	0,77
Mettl22	0,63	Drap1	0,78	Ifna16	0	S100a1	0,77
Cinp	0,63	Trmt10a	0,78	Ifna15	0	Nufip1	0,77
BRDN0000737817	0,63	Prkch	0,78	Iffo1	0	Oc90	0,77
Olfr643	0,63	Rbm48	0,78	1700020D05Rik	0	Hyal2	0,77
Slc19a3	0,63	Cyp4a29	0,78	Iffo2	0	Rassf5	0,77
Eogt	0,63	Olfr1195	0,78	Tob1	0	AI429214	0,77
Stat5a	0,63	Npc1l1	0,78	Tob2	0	Tgfbr2	0,77
Krtap6-5	0,63	Aifm3	0,78	Fen1	0	Pik3r6	0,77
Tada1	0,63	Tshb	0,78	Kif21a	0	Asb8	0,77
Shmt1	0,63	Xkr6	0,78	Asb17	0	Csad	0,77
Rxfp4	0,63	E2f3	0,78	Iqcf6	0	Notch4	0,77
4930548H24Rik	0,63	Tgfbr3	0,78	Brinp2	0	Ndfip2	0,77
Acot8	0,63	Gm9992	0,78	Mrpl4	0	Gm12794	0,77
Olfr812	0,63	Olfr730	0,78	Neo1	0	Fermt3	0,77
Adrb2	0,63	Cst7	0,78	Mrpl2	0	Fgd1	0,77
Gm128	0,63	Noxred1	0,78	Mrpl3	0	Olfr291	0,77
Nobox	0,63	Olfr288	0,78	BRDN0000737612	0	Cyp2c65	0,77
Timd2	0,63	Foxn1	0,78	Mrpl1	0	Nlrp10	0,77
Map3k19	0,63	Atxn2l	0,78	Ffar2	0	Bend7	0,77
Fkbp7	0,63	F8	0,78	Ffar3	0	Ass1	0,77
Dse	0,63	Chp2	0,78	Mtap7d3	0	Dus4l	0,77
Glyat	0,63	Foxd1	0,78	BRDN0000737524	0	Pfdn2	0,77
Dennd4c	0,63	Mib2	0,78	Ffar4	0	Olfr353	0,77
Fcer1g	0,63	6330409D20Rik	0,78	Esp16	0	Hspbp1	0,77
Asf1b	0,62	Ids	0,78	Lmtk2	0	Iqcd	0,77
Ces2e	0,62	Ndufa3	0,78	Esp15	0	Olfr463	0,77
1700007K13Rik	0,62	Ndufb4	0,78	Galns	0	Gpr141	0,77
Hoxa9	0,62	Apln	0,78	Esp18	0	Serpinb13	0,77
4933427G17Rik	0,62	Igbp1	0,78	BRDN0000737774	0	Tor4a	0,77
Marveld1	0,62	Aurkb	0,78	Xiap	0	Tcf7l1	0,77
Igsf9	0,62	Vps72	0,78	Olfr1181	0	Ifna12	0,77
4931409K22Rik	0,62	Fbxo48	0,78	Gnb2l1	0	Olfr1263	0,77
BC021785	0,62	Ywhab	0,78	Rnf41	0	Cndp2	0,77
Scaf4	0,62	Gm20809	0,78	2700097O09Rik	0	Hist1h4n	0,77
Pot1b	0,62	Cipc	0,78	Sav1	0	Snx32	0,77
Elavl1	0,62	Rrp9	0,78	Depdc5	0	Ak3	0,77
Kdm7a	0,62	Grb10	0,78	M1ap	0	Apbb1	0,77
Fam57b	0,62	9530053A07Rik	0,78	Vmn1r148	0	Polr3gl	0,77
Tmem216	0,62	Rxrg	0,78	Arl3	0	Irf2bpl	0,77
Olfr54	0,62	Nudt14	0,78	Cyb5d1	0	Efhc1	0,77
Gen1	0,62	Ly6a	0,78	Fgg	0	Snx33	0,77
Gemin8	0,62	Olfr1228	0,78	Notum	0	Fgb	0,77
Zyg11a	0,62	Fgf3	0,78	Vars	0	Tex24	0,77
Cyb561a3	0,62	Fam179a	0,78	Rabac1	0	Stk32b	0,77
Gm4871	0,62	Olfr1086	0,78	Tlk2	0	Dlk1	0,77
Ccdc154	0,62	Slc37a4	0,78	Msrb1	0	Lce1i	0,77
Cst8	0,62	Olfr945	0,78	Msrb3	0	Plscr1	0,77
Ech1	0,62	Zc3hav1	0,77	Msrb2	0	Tomm40	0,77
Olfr654	0,62	Aldh3b1	0,77	Kpna4	0	Psmb11	0,77
Ano2	0,62	Cx3cr1	0,77	BC055324	0	Tbpl1	0,77
Fam193b	0,62	Nt5c1b	0,77	Ggps1	0	Sp9	0,77
Tlr7	0,62	Kcna4	0,77	Donson	0	C2cd4b	0,77
Dvl1	0,62	Lgals3	0,77	Zfp385a	0	Slc25a17	0,77
Plxnb1	0,62	Slc29a3	0,77	Chka	0	Il12a	0,77
Cep55	0,62	Trim26	0,77	Chkb	0	Sardh	0,77
1700009N14Rik	0,62	Lhcgr	0,77	Zfp385b	0	Ptger1	0,77
Tslp	0,62	Fam134a	0,77	Cnnm1	0	Zfp668	0,77
1500012F01Rik	0,62	Pnma1	0,77	1810011H11Rik	0	Tifab	0,77
Prlh	0,62	Sgol2a	0,77	Hlcs	0	Olfr551	0,77
Lrig3	0,62	Col12a1	0,77	Tgm1	0	Gm14440	0,77

Pip5k1a	0,62
Zbtb39	0,62
Rps5	0,62
Slamf6	0,62
Gm5634	0,61
Ltbp1	0,61
Ak7	0,61
Il13ra2	0,61
Tmem181a	0,61
Xirp2	0,61
Arhgef26	0,61
Ppm1l	0,61
Iigp1	0,61
Hoxd10	0,61
BRDN0000738059	0,61
Hils1	0,61
Arpc1b	0,61
Rgag1	0,61
Th	0,61
Pex11a	0,61
Lingo4	0,61
Ptprh	0,61
Chst1	0,61
1700047I17Rik2	0,61
Impad1	0,61
Inpp5f	0,61
9230110C19Rik	0,61
Gm5741	0,61
Olfr508	0,61
Ccdc159	0,61
Olfr633	0,61
Rassf9	0,61
Akirin1	0,61
BRDN0000738120	0,61
Tmem67	0,61
Gm11562	0,61
Wwtr1	0,61
Tspyl3	0,61
Fam216b	0,61
Olfr1163	0,61
Tm7sf3	0,61
Krt7	0,61
Gyltl1b	0,61
Sucla2	0,61
Prkci	0,60
Kif22	0,60
Apoo	0,60
Aoc1	0,60
Ubfd1	0,60
4933417A18Rik	0,60
Cml3	0,60
Lrif1	0,60
Tmem131	0,60
Ninl	0,60
Ceacam2	0,60
Hook3	0,60
Zfp606	0,60
Afg3l2	0,60
Phldb3	0,60
Nrbf2	0,60
Cstf2	0,60
Dnase1l3	0,60
BRDN0000738087	0,60
Map3k6	0,60
Ndst1	0,60
Tmem123	0,60
Tspan2	0,60
Rbms2	0,60
Bpifa1	0,60
AI413582	0,60
Tcstv3	0,60
Olfr1182	0,60
Kctd20	0,60
Hsf4	0,60
Matn2	0,60
Agfg1	0,60
Pdlim7	0,59
Kdelr2	0,59
Cd300ld	0,59
Ifne	0,59
AF529169	0,59
Ccdc64	0,59
Gm10147	0,77
Ccr3	0,77
Gm5615	0,77
Trp53inp2	0,77
Nrbf2	0,77
Klhl34	0,77
Mfsd2b	0,77
Scx	0,77
Mxra8	0,77
Slc9a3	0,77
Gm13128	0,77
Olfr1111	0,77
Tmc4	0,77
Bola3	0,77
Lrfn3	0,77
Evx1	0,77
Ptgir	0,77
BRDN0000738250	0,77
Nos2	0,77
Exosc10	0,77
Slc7a13	0,77
Trim55	0,77
Prss40	0,77
Rbfox2	0,77
Fkbp15	0,77
Slc9a5	0,77
Dhcr24	0,77
Macrod2	0,77
Dnajb8	0,77
Mpc2	0,77
Poc1a	0,77
Frs2	0,77
Clpp	0,77
Atf5	0,77
Olfr115	0,77
Tecrl	0,77
Cecr2	0,77
Olfr1181	0,77
Tshz1	0,77
Amigo3	0,77
Sox7	0,77
B930041F14Rik	0,77
Efr3a	0,77
Fzd3	0,77
Gstz1	0,77
Gstk1	0,77
Ager	0,77
Bpifb3	0,77
Foxd4	0,77
Dhx8	0,77
Vmn1r209	0,77
Elp2	0,77
Pllp	0,77
Ddb1	0,77
Cd320	0,77
Sept2	0,77
Iqcj	0,77
Ammecr1	0,77
Mfap1a	0,77
Gin1	0,77
Olfr9	0,77
Tmc8	0,77
Arhgap8	0,77
Thbs2	0,77
Hnrnpc	0,77
Zfp560	0,76
Olfr698	0,76
Nmb	0,76
Fabp1	0,76
Cubn	0,76
Chad	0,76
Rgma	0,76
Rnf34	0,76
Setx	0,76
Hmx3	0,76
4930412D23Rik	0,76
Rhox8	0,76
Fxyd3	0,76
Lum	0,76
Ccdc86	0,76
Rap1a	0,76
Ttc21b	0,76
Zdhhc14	0
Dst	0
Cnnm3	0
Aftph	0
Ano4	0
Diexf	0
Vmn2r87	0
Vmn2r80	0
Sidt2	0
Ap2b1	0
Mcm3ap	0
Pdha2	0
Fam69a	0
Msl3l2	0
BRDN0000737658	0
Tgm6	0
Zdhhc17	0
Oxa1l	0
Zfp157	0
Skap1	0
Fam179a	0
2610507B11Rik	0
Pgr15l	0
Ferd3l	0
BRDN0000738292	0
Ttr	0
Snx17	0
Capg	0
BRDN0000737659	0
Ebi3	0
Dynll1	0
Ccl19	0
Tfap4	0
Prkab1	0
Gm13154	0
Gm13157	0
Klhl13	0
Gm13152	0
Gpr75	0
Bre	0
Otud7b	0
Ankub1	0
Wwox	0
Rhebl1	0
Reps2	0
Ints3	0
Ints2	0
Ints1	0
Ints7	0
Ints5	0
Ints4	0
Ints9	0
Ints8	0
Adat1	0
Cap1	0
Cap2	0
Adat2	0
Rnf185	0
Fgfr1op	0
Papd7	0
Ubxn2a	0
Papd5	0
Atp2b1	0
BRDN0000737846	0
Atp2b3	0
Atp2b2	0
Usp47	0
Kcnj6	0
Il18	0
Nanos2	0
Dhdh	0
Vmo1	0
Il19	0
Mtss1l	0
Auh	0
Csrnp1	0
Mre11a	0
Ptafr	0
Csrnp2	0
BRDN0000737845	0
Adgrv1	0
Usp42	0
Zfp932	0,77
Ntng1	0,77
Vmn2r109	0,77
Osbpl5	0,77
Slc39a13	0,77
Adprh	0,77
Olfr275	0,77
Elovl7	0,77
Smim15	0,77
Nasp	0,77
Lpp	0,77
Cttn	0,77
Lamtor4	0,77
Repin1	0,77
Capn15	0,77
Il21	0,77
Olfr1124	0,77
Apool	0,77
Slc16a8	0,77
Olfr427	0,77
Olfr3	0,77
BRDN0000737947	0,77
Olfr1219	0,77
Ralgapb	0,76
Pin1rt1	0,76
Btg1	0,76
Trmt10b	0,76
Esr1	0,76
Klhl15	0,76
Fmo9	0,76
Hopx	0,76
Ccdc58	0,76
Csmd3	0,76
1700003F12Rik	0,76
Smarcd2	0,76
Olfr346	0,76
Arv1	0,76
Spg20	0,76
Gm11758	0,76
Noxo1	0,76
Trim60	0,76
Pde4a	0,76
Sirt4	0,76
Msra	0,76
Ccdc84	0,76
Plxna3	0,76
Zbtb38	0,76
Gpr146	0,76
Wrap73	0,76
Ndufs3	0,76
Mlycd	0,76
BRDN0000737932	0,76
Rab9	0,76
Tmem135	0,76
Defb30	0,76
Olfr294	0,76
Rsrp1	0,76
Lipo1	0,76
Gga1	0,76
Wfdc15a	0,76
Gjb5	0,76
Obsl1	0,76
Olfr1484	0,76
Ptpru	0,76
Kdm4d	0,76
Prl8a6	0,76
Pyhin1	0,76
Gm5797	0,76
Fam131c	0,76
Slc18b1	0,76
Spire2	0,76
1700001O22Rik	0,76
Kif20b	0,76
Emb	0,76
Elovl2	0,76
St6galnac2	0,76
B230118H07Rik	0,76
Itgb2	0,76
Trim13	0,76
Sh2d1a	0,76
A130010J15Rik	0,76
Eaf2	0,76

BC003965	0,59
Ivl	0,59
Bcl7b	0,59
Pcdha5	0,59
Car14	0,59
Epc1	0,59
Lce1l	0,59
Ipo5	0,59
Hspb8	0,59
Slc30a3	0,59
Cyba	0,59
Six5	0,59
Clrn3	0,59
Prkdc	0,59
Ltb	0,59
Pcnxl2	0,59
Dppa3	0,59
Pou2f1	0,59
Apol8	0,59
Cdc25a	0,59
Mmp12	0,59
Cyp2j12	0,59
Clec5a	0,59
Olfr521	0,59
Marc1	0,59
Olfr15	0,59
4930415F15Rik	0,59
Nicn1	0,59
Wdr78	0,59
Gja5	0,59
Tmem138	0,59
Nmb	0,58
Hsdl1	0,58
Proca1	0,58
Lypla2	0,58
Rfx1	0,58
Pou6f1	0,58
Extl1	0,58
Pttg1ip	0,58
Mapk1	0,58
Reps1	0,58
Pitx1	0,58
Elane	0,58
Mark4	0,58
Plcd3	0,58
Efna3	0,58
Rab6b	0,58
Maneal	0,58
Cxcl16	0,58
Cyp19a1	0,58
Trpt1	0,58
Rere	0,58
Dcaf8	0,58
Raver1	0,58
Gm12886	0,58
Micu1	0,58
Traf4	0,58
Prmt1	0,58
Gm11938	0,58
Wipf3	0,58
Brms1	0,58
Thsd1	0,58
BRDN0000738103	0,58
Gpx8	0,58
Hspa1b	0,58
Cdhr5	0,58
Pcdhb20	0,58
Slc6a14	0,58
Gjd2	0,57
Gpr119	0,57
AY761184	0,57
Spata7	0,57
Psca	0,57
Ivd	0,57
Rsph3b	0,57
Nkx2-1	0,57
Klhl24	0,57
Il3ra	0,57
Atp9b	0,57
1190007I07Rik	0,57
Mroh1	0,57
Syncrip	0,57
Trip6	0,76
Gad2	0,76
Cltc	0,76
Arl3	0,76
BC005624	0,76
Slc4a1ap	0,76
Elovl6	0,76
Gm21943	0,76
Abca9	0,76
Rab13	0,76
Agbl4	0,76
Pilrb1	0,76
Susd2	0,76
Aatf	0,76
Gmpr	0,76
Helz2	0,76
Lima1	0,76
Camp	0,76
Hbq1a	0,76
Mmp19	0,76
Smyd3	0,76
Ppp1r10	0,76
Cd40	0,76
Gpat2	0,76
Camsap1	0,76
Cdk12	0,76
Syt15	0,76
Sfmbt1	0,76
2010315B03Rik	0,76
Lrp10	0,76
Cfap44	0,76
Krba1	0,76
Ier3ip1	0,76
Chrnb1	0,76
Alkbh2	0,76
Ubd	0,76
1700019O17Rik	0,76
Olfml2b	0,76
Xkrx	0,76
Gm6583	0,76
Klra3	0,76
Olfr1230	0,76
Chmp1a	0,76
Vmn2r86	0,76
Atrx	0,76
Pex2	0,76
Cyhr1	0,76
Lrrc46	0,76
Smpdl3b	0,76
Cd40lg	0,76
Mansc1	0,76
Aldoart2	0,76
Lce1f	0,76
Itih3	0,76
Brf1	0,76
Ccser1	0,76
Strc	0,76
Olfr378	0,76
Olfr497	0,76
Impa2	0,76
Itih5	0,76
Muc1	0,76
Adgrf2	0,76
Lamtor4	0,76
Mybpc3	0,76
Mtrf1l	0,76
Gphb5	0,76
Gab2	0,76
Adipor2	0,76
H2-D1	0,76
Rtkn2	0,76
Vmn1r220	0,76
Ap2s1	0,76
BRDN0000737831	0,76
Rab5b	0,76
Olfr1229	0,76
Serpina3n	0,76
Cdc34	0,75
Slc30a10	0,75
Rfx1	0,75
Gm11710	0,75
Dennd5b	0,75
Prl6a1	0
Slco4a1	0
Jkamp	0
Hif1a	0
Csn1s2b	0
Try5	0
BRDN0000737844	0
Rasef	0
Il11	0
4930595M18Rik	0
Phyh	0
Gins2	0
Gins3	0
Gins1	0
Gins4	0
Ggta1	0
Apex2	0
Il18r1	0
Coq5	0
Ggt6	0
Ggt7	0
E130012A19Rik	0
Gm684	0
Gm136	0
Prss28	0
Prss29	0
Dcaf5	0
Pdzk1	0
Ccdc65	0
Mdk	0
Ogdhl	0
Ccdc61	0
Ccdc60	0
Ccdc63	0
Ccdc62	0
Ccdc69	0
Ccdc68	0
Gstt2	0
Pex16	0
Cct2	0
Cend1	0
Coq2	0
Dact1	0
Dact3	0
Dact2	0
Cntd1	0
Cyp7b1	0
Ebag9	0
Galk1	0
Galk2	0
Ambra1	0
Nudt16l1	0
Gstt1	0
Gm4787	0
Mc3r	0
Greb1l	0
Gm4871	0
Rbmx2	0
Grk5	0
Adprh	0
Olfr894	0
Il17re	0
Adprm	0
BRDN0000738125	0
Cryz	0
Mrps7	0
Mrps6	0
Mrps5	0
C1qc	0
Hilpda	0
Gjc1	0
Amtn	0
Crym	0
Map4	0
Vmn1r178	0
Il3ra	0
Aasdh	0
Prom2	0
Olfr484	0
Gm5800	0
Olfr486	0
Olfr487	0
Prl2c3	0,76
Bend6	0,76
Mtnr1b	0,76
Nt5c1b	0,76
Cul4a	0,76
Rfx2	0,76
Magt1	0,76
Arfip2	0,76
Rnase11	0,76
Leprotl1	0,76
Olfr603	0,76
Alx1	0,76
Kctd12	0,76
Dnajc25	0,76
Pla2g12b	0,76
Dnajc21	0,76
Cxcr6	0,76
Dusp10	0,76
Olfr994	0,76
Nt5c3	0,76
BC030867	0,76
Olfr677	0,76
Myo1d	0,76
Skint10	0,75
Hat1	0,75
Arhgef11	0,75
Alx4	0,75
Ifnb1	0,75
Slc39a2	0,75
Wipf2	0,75
Fam24a	0,75
Ptp4a1	0,75
Aldoart2	0,75
Tnni2	0,75
BC049715	0,75
Pou3f2	0,75
Olfr1061	0,75
Lpin1	0,75
Mpl	0,75
Zfp462	0,75
Krtap4-2	0,75
Zbtb24	0,75
Alb	0,75
Nrl	0,75
Gm572	0,75
Paxbp1	0,75
Methig1	0,75
Gapdhs	0,75
BRDN0000737775	0,75
Psmd11	0,75
Cachd1	0,75
Cd24a	0,75
Col5a3	0,75
Brd4	0,75
Cib4	0,75
Plac8	0,75
Medag	0,75
Abcc12	0,75
Olfr750	0,75
Olfr1370	0,75
Il20ra	0,75
Gm5634	0,75
Adamtsl1	0,75
Fbxo9	0,75
Sobp	0,75
Slc22a19	0,75
Hprt	0,75
Olfr114	0,75
Hnrnpul2	0,75
Eif4e3	0,75
Tfdp2	0,75
Morc4	0,75
Klrc2	0,75
Wdr91	0,75
Sec11a	0,75
Ccdc40	0,75
4930444G20Rik	0,75
Olfr290	0,75
Hirip3	0,75
Dhrs7b	0,75
Nkx6-1	0,75
Gm13285	0,75

Trp73	0,57
Fam110a	0,57
Prpsap1	0,57
Fcgr4	0,57
Adcyap1r1	0,57
Dnajc5g	0,57
Lcn11	0,57
Pla2g1b	0,57
Zfp426	0,57
Prr5l	0,57
Sirt5	0,57
Il6st	0,57
Afm	0,57
Fyn	0,56
Phgr1	0,56
Morf4l2	0,56
Olfr1	0,56
Dhrs3	0,56
Psapl1	0,56
Eno2	0,56
Paqr5	0,56
Gm6026	0,56
Zfp324	0,56
Hoxb5	0,56
Olfr582	0,56
6330408A02Rik	0,56
Enpep	0,56
BRDN0000738104	0,56
Ntng1	0,56
Cers5	0,56
BRDN0000738290	0,56
Gabra4	0,56
BRDN0000738340	0,56
C1ql4	0,56
BRDN0000737429	0,56
Tstd3	0,56
Nit2	0,56
Krtap19-2	0,56
Gabpb1	0,56
Gnptg	0,56
Gm15080	0,56
Cnksr1	0,56
Mrps18c	0,56
Mettl7a2	0,56
Sall2	0,56
Gnl1	0,56
Scaf11	0,56
Lce3a	0,56
Vmn2r99	0,56
Slc23a2	0,56
Endog	0,56
Zfp609	0,56
Vac14	0,56
Olfr659	0,56
Olfr558	0,56
Eif1b	0,55
Cenpj	0,55
Prr30	0,55
Tmem158	0,55
Upk2	0,55
Abhd14a	0,55
Tox	0,55
Olfr1411	0,55
Syt7	0,55
Taf10	0,55
Hoxa2	0,55
Chd1l	0,55
Cln6	0,55
Ctdsp1	0,55
1700006E09Rik	0,55
Lpar2	0,55
Fbf1	0,55
Oprd1	0,55
Cdv3	0,55
Vti1a	0,55
Exoc7	0,55
Klhdc4	0,55
Zfp661	0,55
Lmna	0,55
Dgcr8	0,55
Mpst	0,55
Olfr876	0,55
Map10	0,75
Edaradd	0,75
Gzmc	0,75
Mcf2l	0,75
Cep57l1	0,75
Igf2r	0,75
Fancg	0,75
Vill	0,75
Lilra6	0,75
Sh2d7	0,75
Olfr524	0,75
Olfr972	0,75
Gm12794	0,75
Zfp473	0,75
Sdhd	0,75
Mab21l3	0,75
Gramd2	0,75
Necab1	0,75
Ppp1r42	0,75
Klf14	0,75
Stpg1	0,75
Vmn1r5	0,75
Slc22a2	0,75
Trim62	0,75
Uba7	0,75
Fry	0,75
Col11a1	0,75
Chst7	0,75
Mertk	0,75
Bcap29	0,75
Hs6st2	0,75
4930550C14Rik	0,75
Cngb1	0,75
Pbp2	0,75
Ppwd1	0,75
Clmn	0,75
Rlim	0,75
BC048679	0,75
Scp2	0,75
Gcat	0,75
Myf5	0,75
Fam178b	0,75
Irx3	0,75
Wnt3	0,75
Amy2a3	0,75
BRDN0000737381	0,75
Zfp292	0,75
Olfr610	0,75
Dnajb14	0,75
Uck1	0,75
Atg101	0,75
P2rx5	0,75
Olfr222	0,75
Pla2g2c	0,75
Khsrp	0,75
Gas6	0,75
Arhgef33	0,75
Uba6	0,75
Pde6b	0,75
Ppp2r2d	0,75
Adss	0,75
Olfr1418	0,75
Colec11	0,75
Olfr855	0,75
Lrrc8d	0,75
Hyal6	0,75
Cabp4	0,75
Fam73a	0,75
Liph	0,75
Sel1l2	0,75
Morc1	0,75
Speer4f	0,75
Sec14l3	0,75
Rce1	0,75
Cklf	0,75
Plb1	0,75
Adra2a	0,75
4930427A07Rik	0,75
Ankrd55	0,75
Fanca	0,75
Msh2	0,75
Mta2	0,75
Olfr480	0
Olfr481	0
Olfr482	0
Olfr483	0
Eif3l	0
Tulp3	0
Tulp1	0
Olfr488	0
Tulp4	0
Lpp	0
Slc12a4	0
Pvrl3	0
Slc12a6	0
Jagn1	0
Rln1	0
Slc12a3	0
Kdm6b	0
Spint4	0
Cwc27	0
Abce1	0
Cwc22	0
D130043K22Rik	0
Hhip	0
Scn7a	0
Slc16a9	0
Ces3a	0
Nalcn	0
Mylk4	0
Mylk2	0
Cry1	0
Cry2	0
Rhox3e	0
Rhox3f	0
Rhox3g	0
Rhox3a	0
Vcpip1	0
Fndc8	0
Atp6v1c2	0
Atp6v1c1	0
Nus1	0
Slc16a8	0
Bex6	0
Pcgf3	0
Bex4	0
Pcgf5	0
4933427D06Rik	0
Bex1	0
Pcgf6	0
BRDN0000738201	0
Olfr734	0
Spata21	0
Olfr732	0
Olfr733	0
Spata24	0
Olfr731	0
Vmn1r217	0
Gosr2	0
Vmn1r215	0
Vmn1r214	0
Vmn1r213	0
Vmn1r212	0
Vmn1r211	0
Vmn1r210	0
Nanp	0
Nans	0
Prcp	0
Vmn1r219	0
Vmn1r218	0
Bend6	0
Bend7	0
Bend4	0
Gm7325	0
Bend3	0
Olfm2	0
Sfi1	0
Sprr2b	0
Lrrc52	0
Dars2	0
Lrrc56	0
Lrrc57	0
Lrrc55	0
Lrrc58	0
Bpifb3	0,75
Panx1	0,75
Ttc3	0,75
Zmynd8	0,75
Mfge8	0,75
Cd44	0,75
Tpm3	0,75
Elovl4	0,75
Pomt2	0,75
Olfr279	0,75
Rap1gap2	0,75
B230217C12Rik	0,75
Hmgcll1	0,75
Itpr2	0,75
Ppp2r2c	0,75
H2afv	0,75
Saa2	0,75
Snrnp200	0,75
Ap4s1	0,75
Ptgfr	0,75
Lrrc20	0,75
Nos3	0,74
Copz2	0,74
F2r	0,74
Vsig2	0,74
Slc16a5	0,74
Bmp4	0,74
Acsf3	0,74
Armc12	0,74
Mpzl1	0,74
Tspan31	0,74
Mdfi	0,74
Tceanc2	0,74
Gm5414	0,74
Cga	0,74
Rhox2b	0,74
1700034E13Rik	0,74
Ccni	0,74
Antxr2	0,74
Nagpa	0,74
Neurod2	0,74
Olfr1487	0,74
Elovl6	0,74
Gm12253	0,74
Fbxo28	0,74
Cdkal1	0,74
Ccdc137	0,74
Gpr25	0,74
Mmel1	0,74
BRDN0000738355	0,74
Dlx1	0,74
Spryd3	0,74
Tab3	0,74
Slc17a1	0,74
Dmrtc1a	0,74
BRDN0000737498	0,74
Aqp1	0,74
Olfr513	0,74
Anxa9	0,74
Arpp21	0,74
A330050F15Rik	0,74
Plekhj1	0,74
Chst10	0,74
Prkcz	0,74
D5Ertd579e	0,74
Plekhh2	0,74
Sdc2	0,74
Ski	0,74
Cdx2	0,74
Surf1	0,74
Slc6a8	0,74
Rdh11	0,74
Tnfaip8l3	0,74
Krtap5-1	0,74
Sema4d	0,74
Lman1l	0,74
Obp1a	0,74
Atoh8	0,74
Nubpl	0,74
Olfr155	0,74
Slc25a43	0,74
Mfsd10	0,74

Agtr1a	0,55	Ube3c	0,75	Lrrc59	0	Aplf	0,74
Hp	0,55	Gm8439	0,75	Sult1c1	0	Fbxw15	0,74
Asb7	0,54	Cartpt	0,75	Nod2	0	Fam195b	0,74
Tm2d2	0,54	Hist1h3c	0,75	Slc11a2	0	Crtc3	0,74
Tfb2m	0,54	Lim2	0,75	Ubb	0	Gria1	0,74
Tnpo2	0,54	Olfr938	0,74	Slc11a1	0	Avl9	0,74
Igll1	0,54	Dnase2a	0,74	Snupn	0	Pnp2	0,74
Cacng6	0,54	Prm3	0,74	Gm14420	0	Lce1b	0,74
Abca7	0,54	Cpeb3	0,74	BRDN0000737929	0	Lyrm1	0,74
Vmn2r4	0,54	BRDN0000738020	0,74	Olfr1349	0	Olfr685	0,74
BRDN0000738308	0,54	Lrrn4	0,74	Olfr1348	0	Clec14a	0,74
Vmn1r113	0,54	Vmn1r81	0,74	Spata2l	0	Pygo1	0,74
Foxe1	0,54	Ppp1r14b	0,74	Zc3h12a	0	Pde6c	0,74
C1qtnf2	0,54	Strn3	0,74	Gdi2	0	Olfr328	0,74
2310047M10Rik	0,54	BRDN0000737533	0,74	Hsd17b1	0	Dtna	0,73
Snai3	0,54	Kctd9	0,74	Olfr1431	0	Olfr17	0,73
Olfr697	0,54	Olfr293	0,74	Mrto4	0	Zfp868	0,73
Tfpi	0,54	Pam	0,74	1700030J22Rik	0	Cdc25a	0,73
Dusp28	0,54	Llgl2	0,74	Thoc1	0	Nostrin	0,73
Urod	0,54	Polr2g	0,74	Hsd17b6	0	Gatb	0,73
BRDN0000738266	0,54	Arhgef15	0,74	Olfr1342	0	Necab1	0,73
Zfp385a	0,54	Nkx6-1	0,74	Col8a2	0	Dynll1	0,73
1110012L19Rik	0,54	Cntn4	0,74	Col8a1	0	Kcnn3	0,73
Hoxd9	0,54	Cblc	0,74	Cyp4x1	0	Sdc4	0,73
Gtf2ird2	0,54	Mark3	0,74	Polq	0	Timmdc1	0,73
Gpr85	0,54	Zfp286	0,74	Hbb-y	0	Npas3	0,73
Tmprss11c	0,54	Fam118b	0,74	H2-Ab1	0	Pqbp1	0,73
Olfr685	0,53	Pds5b	0,74	Gpnmb	0	Nlrp14	0,73
Pax1	0,53	Tbc1d4	0,74	Fam132a	0	Tesk1	0,73
Krt17	0,53	Ifitm3	0,74	Polg	0	Map4k1	0,73
Fbxl4	0,53	Cox11	0,74	Pole	0	Olfr1026	0,73
Spop	0,53	Psme1	0,74	Pold3	0	Wfdc9	0,73
Vmn1r23	0,53	Zdhhc23	0,74	Polb	0	Shisa6	0,73
Syn2	0,53	Gm94	0,74	Plekho2	0	Flywch1	0,73
Accs	0,53	Rbmx2	0,74	Poln	0	Gm13271	0,73
Mb21d2	0,53	Ppapdc1a	0,74	Polk	0	1700029F12Rik	0,73
Scin	0,53	Vmn1r216	0,74	Kcnq4	0	Bag1	0,73
BRDN0000738167	0,53	Rassf7	0,74	Poli	0	Fam171b	0,73
Rfpl4	0,53	Fut8	0,74	Polh	0	Ankrd9	0,73
Map3k7cl	0,53	Otud6b	0,74	Dram1	0	Rangrf	0,73
4933425L06Rik	0,53	BRDN0000737415	0,74	Mesdc1	0	Smpdl3a	0,73
V1rd19	0,53	Gimap9	0,74	Mesdc2	0	Ahrr	0,73
Atrn	0,53	Adgre4	0,74	Dram2	0	Sox6	0,73
Ugt1a7c	0,53	Pgr	0,74	Olfr862	0	Wfdc12	0,73
Adamtsl3	0,53	Dedd2	0,74	Olfr860	0	Tgfbrap1	0,73
Arhgap30	0,53	Apoa1bp	0,74	Ubl4	0	Abcg8	0,73
Capn15	0,53	Olfr993	0,74	Rtn4	0	Gldc	0,73
Olfr1167	0,53	Qrich2	0,74	Tmem240	0	Ocm	0,73
Kctd10	0,53	Vash2	0,74	Cer1	0	Cx3cr1	0,73
Slc27a1	0,53	Bdnf	0,74	3110052M02Rik	0	Tbr1	0,73
Prnp	0,53	Vmn2r4	0,74	Olfr868	0	Fam71f2	0,73
BRDN0000737665	0,52	Stk32c	0,74	Olfr869	0	Pgs1	0,73
Nynrin	0,52	4933417A18Rik	0,74	Olfr1282	0	Vmn1r177	0,73
Islr	0,52	Cr1l	0,74	Rwdd2b	0	Tmem63c	0,73
Clec4n	0,52	Uap1l1	0,74	Aqp5	0	Fhad1	0,73
Tcp11l1	0,52	S100b	0,74	Ugt2b36	0	Pias2	0,73
G6b	0,52	Gpr141	0,74	Fcho1	0	Cwh43	0,73
Vmn1r12	0,52	Cdk20	0,74	Acaca	0	Sh3pxd2a	0,73
Vmn1r75	0,52	BC048507	0,74	Fam122b	0	Ddit4l	0,73
Phex	0,52	Trim46	0,74	Dupd1	0	Ppef2	0,73
Gm3417	0,52	Spata31d1d	0,74	Pa2g4	0	Mfsd4	0,73
Arl6ip4	0,52	Gpr173	0,74	Cdc6	0	Olfr316	0,73
C130060K24Rik	0,52	Anks1b	0,74	Cdc7	0	Msl1	0,73
Aqp12	0,52	Dio2	0,74	Olfr70	0	4933427G17Rik	0,73
Olfr877	0,52	D3Ertd254e	0,74	Olfr1344	0	Spire1	0,73
Fam173a	0,52	Unk	0,74	Vrk3	0	Trp53inp1	0,73
Sec22b	0,52	Abhd8	0,74	Aqp1	0	Nfatc2ip	0,73
1200014J11Rik	0,52	Nt5e	0,74	Ralb	0	Arvcf	0,73
Srpk1	0,52	Arhgap42	0,74	Prr12	0	Brd8	0,73
Mfhas1	0,51	Cnot11	0,74	Prpf8	0	Bnip2	0,73
Vrk3	0,51	Tomm40	0,74	Prr11	0	Ly6e	0,73
I830077J02Rik	0,51	Gm5113	0,74	Prr14	0	Olfr1102	0,73
Pltp	0,51	Glb1	0,74	Aurkc	0	Cdk6	0,73
Slc16a11	0,51	Pnmt	0,74	Fam122c	0	Foxred2	0,73
Cln3	0,51	Gpr21	0,74	Prpf3	0	Pptc7	0,73
Fads1	0,51	Hoxc9	0,74	Prr19	0	Stfa1	0,73
1700013G24Rik	0,51	Mrps34	0,74	Ckap4	0	Mknk2	0,73
C130074G19Rik	0,51	Prl2a1	0,74	Prpf4	0	Olfr115	0,73
Boc	0,51	Slc29a1	0,74	Prpf6	0	Wdr34	0,73
Gm11559	0,51	Rgs16	0,74	BC051019	0	Cenpb	0,73
Crot	0,51	Sh2d2a	0,74	Shisa9	0	Tcn2	0,73
Agap1	0,51	Apod	0,74	Shc1	0	Nkain4	0,73

Foxg1	0,51	Fam124b	0,74	Isy1	0	Gng7	0,73
Tmem55b	0,51	Magi3	0,74	Ifi27	0	Thumpd1	0,73
Kcnc2	0,51	Actg2	0,74	Hdac3	0	Nfe2	0,73
Mccc1	0,51	Dnajc30	0,74	Dkkl1	0	Vmn1r104	0,73
BRDN0000737697	0,51	Fut1	0,74	Cstl1	0	Vmn2r4	0,73
Plac9b	0,51	Frk	0,74	Ugt1a7c	0	Champ1	0,73
Akr7a5	0,51	Pthlh	0,74	Pkp3	0	Mettl4	0,73
Bpifc	0,50	Esp16	0,74	Pkp2	0	Arhgef6	0,73
Frmd4a	0,50	Rapsn	0,74	Pkp1	0	Krtap31-2	0,73
Coq6	0,50	Olfr707	0,74	Hdac5	0	Aamdc	0,73
Sec16b	0,50	Pign	0,74	Myo7a	0	Ccdc88c	0,73
Hexdc	0,50	Tmem50b	0,74	Aqp9	0	Gpbp1l1	0,73
Olfr348	0,50	Pced1a	0,74	Ninl	0	Gpr142	0,73
Fxyd3	0,50	Xpo5	0,74	Nsmf	0	Slc10a1	0,73
D630003M21Rik	0,50	Gja4	0,74	Hdac4	0	Mecp2	0,73
Slco6d1	0,50	C4bp	0,74	Lrrc30	0	Aif1	0,73
BRDN0000738125	0,50	Fgf4	0,74	Wt1	0	Gm6408	0,73
Mef2b	0,50	Phrf1	0,73	Itch	0	Krt16	0,73
Actl11	0,50	Tnni3k	0,73	Hdac6	0	Rrp1	0,73
Krtap9-5	0,50	BRDN0000737449	0,73	Zg16	0	Pknox2	0,73
Ocln	0,50	Fam50b	0,73	Vmn1r221	0	Vmn1r54	0,73
Ccl20	0,50	Olfr1359	0,73	Tas2r109	0	Ube2z	0,73
Defb12	0,50	Tmbim4	0,73	Tas2r108	0	Snx15	0,73
Tor1aip2	0,50	Vmn2r3	0,73	Mical2	0	Sarm1	0,73
2900092C05Rik	0,50	Prss46	0,73	Itln1	0	Mxd1	0,73
Prickle3	0,50	Rwdd2b	0,73	Dio1	0	Dpcd	0,73
Ahcy	0,50	Dram2	0,73	Dio2	0	Spata2l	0,73
Cdk5rap3	0,49	Zfp593	0,73	Dio3	0	Gcc2	0,72
Tmem45b	0,49	Pkhd1	0,73	Cd200r1	0	E330017A01Rik	0,72
Bbx	0,49	Fcer1a	0,73	Tas2r102	0	Asic3	0,72
Dusp26	0,49	Rgsl1	0,73	Tas2r105	0	Sept14	0,72
Map2k2	0,49	Abhd17a	0,73	Tas2r104	0	a	0,72
BRDN0000737714	0,49	Cd68	0,73	Tas2r106	0	Gm5111	0,72
Lpcat1	0,49	Figf	0,73	Abhd16b	0	Vmn2r97	0,72
Plxdc1	0,49	Bdh1	0,73	Scaper	0	Wipf1	0,72
Dtx3	0,49	Fkbp7	0,73	1700030K09Rik	0	Gm5800	0,72
Ube2l6	0,49	Tdrd3	0,73	BRDN0000737727	0	Traf7	0,72
Has3	0,49	Ftl1	0,73	Frk	0	Olfr394	0,72
4930524B15Rik	0,48	Alpk3	0,73	Acox3	0	Gjc2	0,72
Vmn1r5	0,48	Ganc	0,73	Acox1	0	Plat	0,72
Rassf5	0,48	Capsl	0,73	Ergic2	0	Mcts2	0,72
Mlst8	0,48	Myo15	0,73	Ergic3	0	D6Ertd527e	0,72
Psmg1	0,48	Emc9	0,73	Oprm1	0	Vps4b	0,72
Fuca1	0,48	Clec4a1	0,73	Ergic1	0	Elmo3	0,72
Spns1	0,47	Mchr1	0,73	Derl1	0	Ddn	0,72
Isyna1	0,47	Hcrt	0,73	Derl2	0	Sorcs1	0,72
Tspyl4	0,47	Stac2	0,73	Derl3	0	Zak	0,72
Insm1	0,47	Gm10352	0,73	Pnmal2	0	AU022252	0,72
Tmem92	0,47	Zbtb24	0,73	Trp53i11	0	Prpsap1	0,72
Hrasls	0,47	Gm13103	0,73	Pnmal1	0	Cyp4x1	0,72
Mbnl1	0,47	Trim60	0,73	Apcs	0	P2ry4	0,72
Xpnpep2	0,46	Igfbp4	0,73	Fry	0	Mfsd5	0,72
Spaca1	0,46	Olfr555	0,73	Notch3	0	Gfral	0,72
BRDN0000738144	0,44	Elk1	0,73	Olfr1448	0	Olfr1413	0,72
Zzz3	0,00	Ube2ql1	0,73	BRDN0000737723	0	U2af1	0,72
Zzef1	0,00	Slc2a9	0,73	Clic6	0	Thap3	0,72
Zyx	0,00	Gm17296	0,73	Clic4	0	Chchd7	0,72
Zxdc	0,00	2810006K23Rik	0,73	Sdhaf3	0	Grp	0,72
Zxdb	0,00	Smtnl2	0,73	Sdhaf4	0	Fam129b	0,72
Zwint	0,00	Hspb7	0,73	Cyp3a41b	0	Gm590	0,72
Zwilch	0,00	Cxxc5	0,73	Cyp3a41a	0	Trdn	0,72
Zw10	0,00	Ogfod1	0,73	Clic1	0	BRDN0000737735	0,72
Zufsp	0,00	Camsap2	0,73	BRDN0000737721	0	Ppp1r12c	0,72
Zswim7	0,00	Nfkbib	0,73	Esp38	0	Olfr123	0,72
Zswim6	0,00	Bace2	0,73	Gfi1b	0	Mthfd2l	0,72
Zswim5	0,00	Elp4	0,73	Gabarap	0	Ivns1abp	0,72
Zswim4	0,00	Rapgef1	0,73	Zfp78	0	Mocs1	0,72
Zswim2	0,00	Pik3r5	0,73	Xkr6	0	Ranbp6	0,72
Zswim1	0,00	Nxph3	0,73	Zscan29	0	Bmp10	0,72
Zscan5b	0,00	Olfr971	0,73	Gm2933	0	Mybph	0,72
Zscan4f	0,00	Pik3r2	0,73	Ceacam2	0	Dnajc16	0,72
Zscan4d	0,00	Phf13	0,73	Aplp1	0	Zfp354b	0,72
Zscan4c	0,00	Socs5	0,73	Btbd17	0	Dll1	0,72
Zscan4b	0,00	Scamp1	0,73	Zscan20	0	Cyp2c54	0,72
Zscan29	0,00	Tm4sf1	0,73	Zscan21	0	BRDN0000737902	0,72
Zscan25	0,00	Ldhd	0,73	Zscan22	0	Gm20852	0,72
Zscan21	0,00	Dgki	0,73	Lmf2	0	Nudt19	0,72
Zscan20	0,00	Slc18a3	0,73	Lmf1	0	Phf11d	0,72
Zscan2	0,00	Hopx	0,73	Atoh8	0	St3gal4	0,72
Zscan18	0,00	Pdlim7	0,73	Tepp	0	Zfp955b	0,72
Zrsr2	0,00	Cd8b1	0,73	BRDN0000738158	0	Cd200	0,72
Zrsr1	0,00	Klk4	0,73	Ano8	0	1500012F01Rik	0,72

Zranb3	0,00
Zranb2	0,00
Zranb1	0,00
Zpr1	0,00
Zpld1	0,00
Zpbp2	0,00
Zpbp	0,00
Zp3r	0,00
Zp3	0,00
Zp2	0,00
Zp1	0,00
Znrf4	0,00
Znrf3	0,00
Znrf2	0,00
Znrf1	0,00
Znrd1	0,00
Znhit6	0,00
Znhit3	0,00
Znhit2	0,00
Znfx1	0,00
Znf512b	0,00
Zmynd8	0,00
Zmynd19	0,00
Zmynd12	0,00
Zmynd11	0,00
Zmynd10	0,00
Zmym6	0,00
Zmym1	0,00
Zmpste24	0,00
Zmiz2	0,00
Zmiz1	0,00
Zmat5	0,00
Zmat4	0,00
Zmat3	0,00
Zmat1	0,00
Zkscan8	0,00
Zkscan7	0,00
Zkscan6	0,00
Zkscan5	0,00
Zkscan4	0,00
Zkscan2	0,00
Zkscan17	0,00
Zkscan16	0,00
Zkscan1	0,00
Zim1	0,00
Zik1	0,00
Zic5	0,00
Zic4	0,00
Zic1	0,00
Zhx3	0,00
Zhx2	0,00
Zgrf1	0,00
Zglp1	0,00
Zg16	0,00
Zfyve28	0,00
Zfyve27	0,00
Zfyve26	0,00
Zfyve21	0,00
Zfyve16	0,00
Zfyve1	0,00
Zfy2	0,00
Zfy1	0,00
Zfr2	0,00
Zfpm2	0,00
Zfpm1	0,00
Zfpl1	0,00
Zfp97	0,00
Zfp963	0,00
Zfp961	0,00
Zfp960	0,00
Zfp959	0,00
Zfp957	0,00
Zfp956	0,00
Zfp955b	0,00
Zfp955a	0,00
Zfp954	0,00
Zfp952	0,00
Zfp951	0,00
Zfp949	0,00
Zfp948	0,00
Zfp944	0,00
Zfp943	0,00
Lipt2	0,73
Tnnt2	0,73
Slc4a3	0,73
Cdkn2c	0,73
Morf4l1	0,73
H2-M11	0,73
Tbx2	0,73
Pvrl1	0,73
Ugt2b35	0,73
Ppdpf	0,73
Plscr4	0,73
Pbdc1	0,73
Coro1b	0,73
BRDN0000737681	0,73
Pdyn	0,73
Pmf1	0,73
Zfp184	0,73
Mtrf1	0,73
Rarres1	0,73
Sgcg	0,73
Aadat	0,73
Pard6g	0,73
Krt9	0,73
Prkci	0,73
Usp17ld	0,73
Zmym3	0,73
Olfr808	0,73
Mptx1	0,73
Fam160a2	0,73
Hoxa6	0,73
G6b	0,73
Pdzk1	0,73
Nefh	0,73
BRDN0000737496	0,73
Mob1a	0,73
Rtbdn	0,73
Atp6v0e	0,72
Ppa1	0,72
BRDN0000737734	0,72
A4gnt	0,72
Snx9	0,72
BRDN0000737854	0,72
Taar7b	0,72
Crtc2	0,72
Kif13a	0,72
Rasa2	0,72
Prnp	0,72
Zfp58	0,72
Npsr1	0,72
Pip4k2c	0,72
Gdap2	0,72
Gm20823	0,72
Ccdc12	0,72
Scn7a	0,72
Hltf	0,72
Grin2d	0,72
Dlx4	0,72
Krt24	0,72
Gpt2	0,72
Psg26	0,72
Pcdh20	0,72
Alpl	0,72
Mrps31	0,72
A830018L16Rik	0,72
Olfr1354	0,72
Gm5797	0,72
Ubox5	0,72
Tcf21	0,72
Vmn2r80	0,72
Foxa3	0,72
Isl2	0,72
Lmo1	0,72
Birc6	0,72
Cd81	0,72
Gpbar1	0,72
Abca15	0,72
Nf1	0,72
Drd3	0,72
Tgif2lx2	0,72
Olfr13	0,72
Dbp	0,72
Ces1c	0,72
Smim11	0
Tmf1	0
Slc34a1	0
Acoxl	0
Tshr	0
Dus3l	0
1700037C18Rik	0
Tssc4	0
Alyref	0
1700010I14Rik	0
Tssc1	0
Zfyve19	0
BRDN0000737858	0
BRDN0000737859	0
Zfp687	0
Gm16390	0
Tmco1	0
Tmco3	0
BRDN0000737850	0
Tmco5	0
Tmco6	0
BRDN0000737853	0
Srebf2	0
Hrasls5	0
Zmat3	0
Zmat4	0
Mpped1	0
Ube2v1	0
Ube2v2	0
BC030500	0
Chrna1	0
Mpped2	0
Rab24	0
Ankrd45	0
Ankrd44	0
Aida	0
Pom121	0
Ctf1	0
Ppara	0
Ankrd49	0
Vwc2	0
Slc34a3	0
Krt2	0
Mef2a	0
Btbd9	0
Btbd8	0
Krt5	0
Mef2d	0
Arid4a	0
Btbd6	0
Btbd1	0
Btbd3	0
Btbd2	0
Cluh	0
Rnf167	0
Itgb2	0
Rnf165	0
Zfp352	0
Rnf169	0
Rnf168	0
2210408I21Rik	0
Rabgap1l	0
Noc4l	0
Eomes	0
Eaf1	0
Eaf2	0
N4bp2l2	0
Gm4187	0
BRDN0000737611	0
Vamp8	0
Pdzrn4	0
Rad51d	0
Rad51c	0
Dnpep	0
Mc1r	0
1700057G04Rik	0
Isx	0
Ddx39b	0
Sval1	0
Scgb2b24	0
Phgr1	0
Erc2	0
Anpep	0,72
Tmem87b	0,72
Zfp93	0,72
Tank	0,72
Ly6g6c	0,72
Fsd1	0,72
Scnn1g	0,72
Lrrc1	0,72
Olfr1415	0,72
Kdm5c	0,72
Syn3	0,72
Zfp13	0,72
Tmtc3	0,72
Btg1-ps2	0,72
1810011O10Rik	0,72
Tas1r1	0,72
Olfr683	0,72
4932418E24Rik	0,72
Arhgef28	0,72
Zfp429	0,72
Zbtb44	0,72
Nrd1	0,72
Olfr1459	0,72
Abcf2	0,72
Pam	0,72
Olfr1209	0,72
AY761184	0,72
Dcaf17	0,72
Olfr800	0,72
Prr16	0,72
H2-Q2	0,72
Adgrf2	0,72
Slc9a6	0,72
Olfml2b	0,72
Klhl14	0,72
Ankrd34c	0,72
Xrcc4	0,72
Chad	0,72
Celf5	0,72
Amer3	0,72
6330403A02Rik	0,72
Cdo1	0,72
Grtp1	0,72
Pibf1	0,71
4930513O06Rik	0,71
Tex9	0,71
Capn12	0,71
Il15	0,71
Ets1	0,71
Gm14431	0,71
BRDN0000737964	0,71
Hoxb3	0,71
Zfp524	0,71
Vmn2r13	0,71
Lefty1	0,71
Lmx1b	0,71
S100a9	0,71
Psmb4	0,71
Krtap6-5	0,71
Uhrf1bp1	0,71
Gnb2l1	0,71
Klk8	0,71
Fam104a	0,71
Tspan2	0,71
Olfr1388	0,71
Fbxo25	0,71
Runx1	0,71
Il17rb	0,71
BRDN0000738186	0,71
Wnt8b	0,71
Rheb	0,71
Foxp3	0,71
Ankrd63	0,71
Chchd4	0,71
Hspb8	0,71
Ddx58	0,71
Lsm12	0,71
Bcl2	0,71
Dos	0,71
Fchsd2	0,71
Vmn1r90	0,71
Ror1	0,71

Zfp942	0,00	Ptdss1	0,72	Sult1d1	0	Ceacam13	0,71
Zfp941	0,00	Gnpda2	0,72	Gzmf	0	Tenm3	0,71
Zfp940	0,00	Slc31a1	0,72	Ston2	0	Txlna	0,71
Zfp94	0,00	Arl10	0,72	Grin2c	0	Aup1	0,71
Zfp938	0,00	1700102P08Rik	0,72	Chmp2a	0	Olfr924	0,71
Zfp937	0,00	Ccnjl	0,72	Dhx16	0	Sox14	0,71
Zfp935	0,00	Crip2	0,72	Dhx15	0	Crb1	0,71
Zfp934	0,00	Ccdc97	0,72	Zfp239	0	Ankrd52	0,71
Zfp933	0,00	Hyal2	0,72	Ankzf1	0	A630023A22Rik	0,71
Zfp932	0,00	Bcl9l	0,72	Mthfd2	0	Asphd1	0,71
Zfp931	0,00	Pigx	0,72	Mthfd1	0	Pclo	0,71
Zfp93	0,00	Lipe	0,72	Stpg2	0	Slc35e4	0,71
Zfp92	0,00	Lzts3	0,72	Fam159b	0	Gm614	0,71
Zfp9	0,00	BC030500	0,72	Fam159a	0	Larp1	0,71
Zfp882	0,00	Cryba4	0,72	Katnal1	0	Ptpn6	0,71
Zfp874b	0,00	Chdh	0,72	Olfr259	0	Kl	0,71
Zfp873	0,00	Hoxc6	0,72	BRDN0000737617	0	BRDN0000737755	0,71
Zfp872	0,00	Ppp1r18	0,72	Wdr20rt	0	Mettl2	0,71
Zfp870	0,00	Kank4	0,72	Dbf4	0	Gpld1	0,71
Zfp87	0,00	Ndufa13	0,72	Qrich2	0	Gpr45	0,71
Zfp869	0,00	Prkcq	0,72	Tmeff1	0	Tmem106c	0,71
Zfp867	0,00	Nudc	0,71	Olfr251	0	Angptl7	0,71
Zfp866	0,00	Jak3	0,71	Olfr250	0	Foxn2	0,71
Zfp850	0,00	Eaf2	0,71	Stip1	0	4933411G11Rik	0,71
Zfp85	0,00	Vmn1r234	0,71	Osbp2	0	Kel	0,71
Zfp846	0,00	Arl16	0,71	Pmel	0	Olfr804	0,71
Zfp839	0,00	Trmt11	0,71	Strn	0	Rbp2	0,71
Zfp831	0,00	Il24	0,71	Tpbpb	0	Rapgef5	0,71
Zfp830	0,00	Cbr4	0,71	Alg8	0	Tmem261	0,71
Zfp827	0,00	Mrgprb8	0,71	Alg9	0	Arpc5l	0,71
Zfp820	0,00	Olfr697	0,71	Hal	0	Vmn1r44	0,71
Zfp82	0,00	Mmp3	0,71	Tgm4	0	Greb1	0,71
Zfp819	0,00	Mfsd7b	0,71	Fam180a	0	Evx1	0,71
Zfp809	0,00	Ulk3	0,71	Alg2	0	Olfr248	0,71
Zfp808	0,00	Fam89b	0,71	Alg3	0	Olfr1510	0,71
Zfp804b	0,00	Ptch1	0,71	Fmr1nb	0	Phf10	0,71
Zfp800	0,00	Pcdhgb6	0,71	Alg1	0	Plekhf1	0,71
Zfp799	0,00	Gpr37	0,71	Alg6	0	Zmym1	0,71
Zfp791	0,00	Ceacam11	0,71	Ntn1	0	E030002O03Rik	0,71
Zfp790	0,00	Wfdc9	0,71	BRDN0000737614	0	Otogl	0,71
Zfp786	0,00	Rom1	0,71	Rnaset2a	0	Ccdc24	0,71
Zfp784	0,00	Bmi1	0,71	1700012P22Rik	0	BRDN0000738306	0,71
Zfp781	0,00	Pole3	0,71	Wif1	0	Hist1h4c	0,71
Zfp780b	0,00	Cxcr2	0,71	Prim1	0	Veph1	0,71
Zfp78	0,00	Olfr432	0,71	Olfr583	0	Jsrp1	0,71
Zfp775	0,00	Onecut2	0,71	Prim2	0	Olfr54	0,71
Zfp772	0,00	Rasgrp4	0,71	Acap3	0	BRDN0000737452	0,71
Zfp771	0,00	Gbp9	0,71	Mcf2l	0	Cd28	0,71
Zfp770	0,00	Ctdsp2	0,71	Adrbk2	0	Tmem205	0,71
Zfp768	0,00	Scgb1b3	0,71	Gyg	0	Dclk2	0,71
Zfp764	0,00	Kdelr3	0,71	Reck	0	Mex3a	0,70
Zfp763	0,00	Rpl39l	0,71	Dsg1c	0	Olfr743	0,70
Zfp760	0,00	Drd1	0,71	Hephl1	0	Ttc30b	0,70
Zfp759	0,00	BRDN0000738186	0,71	Bbs10	0	Glt8d1	0,70
Zfp758	0,00	Gm5901	0,71	BRDN0000738081	0	Itga7	0,70
Zfp750	0,00	Grn	0,71	Ahctf1	0	Olfr1014	0,70
Zfp748	0,00	Ulk2	0,71	Gmppa	0	Ffar2	0,70
Zfp740	0,00	Lyrm5	0,71	Pfn2	0	Rtn4r	0,70
Zfp735	0,00	Klk8	0,71	Irf2bpl	0	Dpp8	0,70
Zfp729b	0,00	Kctd19	0,71	Kcnn4	0	Tor1b	0,70
Zfp72	0,00	Olfr287	0,71	Fam132b	0	Mc5r	0,70
Zfp719	0,00	Ndufb3	0,71	1200014J11Rik	0	Fam49a	0,70
Zfp715	0,00	Tlx1	0,71	Slc30a9	0	Susd1	0,70
Zfp710	0,00	Tnp2	0,71	Slc30a8	0	Fxr2	0,70
Zfp709	0,00	Vmn1r157	0,71	Tysnd1	0	Tmem51	0,70
Zfp706	0,00	Epc1	0,71	Rabl6	0	Atp1b2	0,70
Zfp704	0,00	4930578C19Rik	0,71	Pfn4	0	Slc41a3	0,70
Zfp703	0,00	Tmem206	0,71	Slc30a6	0	Tbc1d2b	0,70
Zfp7	0,00	Lypd3	0,71	Rabl2	0	Pld3	0,70
Zfp697	0,00	Stx17	0,71	Slc30a3	0	Chrna9	0,70
Zfp692	0,00	Cybb	0,71	Slc30a2	0	Usp2	0,70
Zfp691	0,00	Dpy19l4	0,71	Utf1	0	Dek	0,70
Zfp69	0,00	Olfr314	0,71	S100pbp	0	Ces2h	0,70
Zfp689	0,00	Dmgdh	0,71	Pacrgl	0	0610009O20Rik	0,70
Zfp688	0,00	Arhgap19	0,71	Edil3	0	Ifnab	0,70
Zfp687	0,00	Selenbp1	0,71	Gjb5	0	Fnbp1	0,70
Zfp68	0,00	4933421I07Rik	0,71	Kdf1	0	Stk17b	0,70
Zfp677	0,00	Blm	0,71	Vmn1r135	0	BRDN0000737432	0,70
Zfp672	0,00	Gm12169	0,71	Brpf3	0	Zkscan14	0,70
Zfp667	0,00	Nupr1	0,71	Slc16a14	0	Eva1a	0,70
Zfp664	0,00	Atox1	0,71	Brpf1	0	Nos2	0,70
Zfp658	0,00	Zcchc24	0,71	BRDN0000737996	0	Ccdc109b	0,70

Zfp655	0,00
Zfp654	0,00
Zfp652	0,00
Zfp651	0,00
Zfp647	0,00
Zfp646	0,00
Zfp644	0,00
Zfp639	0,00
Zfp637	0,00
Zfp629	0,00
Zfp628	0,00
Zfp623	0,00
Zfp62	0,00
Zfp619	0,00
Zfp618	0,00
Zfp617	0,00
Zfp616	0,00
Zfp612	0,00
Zfp61	0,00
Zfp607	0,00
Zfp605	0,00
Zfp600	0,00
Zfp599	0,00
Zfp598	0,00
Zfp597	0,00
Zfp595	0,00
Zfp593	0,00
Zfp592	0,00
Zfp59	0,00
Zfp583	0,00
Zfp580	0,00
Zfp58	0,00
Zfp579	0,00
Zfp575	0,00
Zfp57	0,00
Zfp568	0,00
Zfp563	0,00
Zfp560	0,00
Zfp558	0,00
Zfp553	0,00
Zfp551	0,00
Zfp541	0,00
Zfp54	0,00
Zfp536	0,00
Zfp534	0,00
Zfp526	0,00
Zfp524	0,00
Zfp523	0,00
Zfp521	0,00
Zfp518b	0,00
Zfp518a	0,00
Zfp516	0
Zfp513	0
Zfp512	0
Zfp511	0
Zfp51	0
Zfp507	0
Zfp503	0
Zfp493	0
Zfp474	0
Zfp473	0
Zfp472	0
Zfp467	0
Zfp462	0
Zfp459	0
Zfp458	0
Zfp457	0
Zfp456	0
Zfp455	0
Zfp451	0
Zfp446	0
Zfp445	0
Zfp442	0
Zfp429	0
Zfp422	0
Zfp42	0
Zfp418	0
Zfp414	0
Zfp410	0
Zfp41	0
Zfp408	0
Zfp407	0
Aldh1a2	0,71
Fam107b	0,71
Gm13889	0,71
Tlx2	0,71
Fbxo36	0,71
Rps27a	0,71
Vmn1r115	0,71
Itsn2	0,71
Spred1	0,71
Serpinb9d	0,71
AI429214	0,71
Ttc33	0,71
9130019O22Rik	0,71
Plaa	0,71
Dhrs3	0,71
Gjd4	0,71
Ctnnal1	0,71
Bend6	0,71
Exoc4	0,71
3110007F17Rik	0,71
Matn2	0,71
Tas2r119	0,71
Lgals4	0,71
Vmn1r229	0,71
Msantd1	0,71
Rab9	0,71
Mier2	0,71
Zfp82	0,71
Tmprss11f	0,71
Col26a1	0,71
Lipc	0,71
Tmco6	0,71
Olfr519	0,71
Kynu	0,71
Slco1a5	0,71
Stk25	0,71
Gm13298	0,71
Dnah9	0,71
Agtpbp1	0,71
Olfr512	0,71
Zfp672	0,71
Hmgn2	0,71
Olig1	0,71
Tesk2	0,71
Cd3e	0,71
Cd48	0,71
Fam132b	0,70
4933425L06Rik	0,70
Hdac2	0,70
Tbc1d13	0,70
Plod3	0,70
Zdhhc16	0,70
Vmn2r58	0,70
Zic1	0,70
Tmem179	0,70
1700042G07Rik	0,70
Sla2	0,70
BRDN0000737385	0,70
Ago4	0,70
Slc35c2	0,70
Fgf5	0,70
Afg3l2	0,70
Fen1	0,70
Gm12429	0,70
Ubxn6	0,70
Leprotl1	0,70
Ntn4	0,70
Arhgef28	0,70
Gm15315	0,70
Tbx10	0,70
Krt13	0,70
Prss37	0,70
App	0,70
6030498E09Rik	0,70
Ccdc36	0,70
Zfp846	0,70
Rtn2	0,70
Jmjd8	0,70
Lcorl	0,70
Inppl1	0,70
P3h2	0,70
Rfesd	0,70
Slc16a11	0
Slc16a10	0
Slc16a13	0
Slc16a12	0
Crls1	0
BRDN0000737991	0
Vmn1r9	0
Acsl5	0
Lyst	0
Urb1	0
Erbb3	0
Cnst	0
Helq	0
Mcts1	0
Spast	0
Helz	0
Irf2bp2	0
Hipk2	0
Vmn1r90	0
Irf2bp1	0
Pcnt	0
Vmn1r94	0
Hipk4	0
Hnrnpl	0
B9d2	0
B9d1	0
Jchain	0
BRDN0000738271	0
Rgag4	0
Lsm8	0
Fos	0
Lsm4	0
Lsm5	0
Eef1d	0
Lsm7	0
Jph1	0
Olfr171	0
Lsm2	0
Lsm3	0
Fam103a1	0
Gm1553	0
Phyhipl	0
Guk1	0
Zmynd10	0
Zmynd11	0
Aim1l	0
Mon1a	0
Mon1b	0
Zmynd19	0
Tpx2	0
Neto2	0
Inmt	0
Grb7	0
BRDN0000738030	0
BRDN0000737716	0
Ppp1r26	0
Ppp1r27	0
Traf6	0
Hnrnpf	0
Rasgrf1	0
4933436I01Rik	0
Dlec1	0
Zcrb1	0
Esp31	0
Esp34	0
Dph2	0
Esp36	0
Slc36a2	0
Hist2h2bb	0
Dph3	0
Hist2h2be	0
Ankar	0
Traf3ip2	0
Traf3ip1	0
BRDN0000738109	0
Vprbp	0
Sac3d1	0
Lpxn	0
Calcrl	0
Lrba	0
H2-DMb1	0
Spop	0
Mef2d	0,70
Ldlrad2	0,70
Smr2	0,70
BRDN0000738015	0,70
Lrpprc	0,70
Syf2	0,70
Cbr3	0,70
Ubap1	0,70
Olfr1200	0,70
Acot2	0,70
Olfr27	0,70
Cyp4a12a	0,70
9430016H08Rik	0,70
Olfr1253	0,70
Dynll2	0,70
Nusap1	0,70
Ccdc102a	0,70
Tmem201	0,70
Slc22a30	0,70
Pcdhb20	0,70
Leo1	0,70
Tm6sf2	0,70
Spem1	0,70
Txnl4a	0,70
Vmn1r172	0,70
Xaf1	0,70
BRDN0000737909	0,70
Egf	0,70
Olfr169	0,70
H2afz	0,70
Naa20	0,70
Gm21951	0,70
Ormdl3	0,70
Olfr102	0,70
Grm5	0,70
Aoc1	0,70
Mdp1	0,70
Ttll12	0,70
Kat7	0,70
Gsc	0,70
Ttbk1	0,70
Krtap9-1	0,70
Adora2b	0,70
Pde1a	0,70
C1d	0,70
Ms4a3	0,70
Slc35b2	0,70
Fbll1	0,70
Wdr86	0,70
Lama3	0,70
Calu	0,70
Hsf4	0,69
Gm4981	0,69
Myof	0,69
Gpr1	0,69
Slc35a2	0,69
Fgf23	0,69
Pard6g	0,69
Yars2	0,69
Sgsm3	0,69
BRDN0000738295	0,69
Fam69a	0,69
Mtl5	0,69
Cpn1	0,69
Ncam2	0,69
Vmn2r124	0,69
Ttpa	0,69
Morc2a	0,69
Zic4	0,69
Ntn5	0,69
Olfr1230	0,69
Bok	0,69
Ctsl	0,69
Gm10352	0,69
Galp	0,69
Cep250	0,69
Emp2	0,69
Rnh1	0,69
Gas2l1	0,69
Met	0,69
Crygs	0,69
1700037C18Rik	0,69

Zfp398	0
Zfp395	0
Zfp39	0
Zfp385c	0
Zfp385b	0
Zfp384	0
Zfp383	0
Zfp382	0
Zfp37	0
Zfp36l2	0
Zfp36l1	0
Zfp369	0
Zfp367	0
Zfp366	0
Zfp365	0
Zfp362	0
Zfp358	0
Zfp354c	0
Zfp354b	0
Zfp354a	0
Zfp352	0
Zfp35	0
Zfp345	0
Zfp341	0
Zfp335	0
Zfp330	0
Zfp329	0
Zfp326	0
Zfp319	0
Zfp318	0
Zfp316	0
Zfp300	0
Zfp3	0
Zfp296	0
Zfp292	0
Zfp287	0
Zfp286	0
Zfp282	0
Zfp281	0
Zfp280d	0
Zfp280c	0
Zfp280b	0
Zfp28	0
Zfp277	0
Zfp276	0
Zfp275	0
Zfp266	0
Zfp263	0
Zfp260	0
Zfp26	0
Zfp251	0
Zfp239	0
Zfp235	0
Zfp219	0
Zfp217	0
Zfp213	0
Zfp212	0
Zfp207	0
Zfp2	0
Zfp191	0
Zfp189	0
Zfp184	0
Zfp182	0
Zfp180	0
Zfp174	0
Zfp169	0
Zfp160	0
Zfp157	0
Zfp148	0
Zfp146	0
Zfp143	0
Zfp131	0
Zfp13	0
Zfp128	0
Zfp120	0
Zfp12	0
Zfp119b	0
Zfp119a	0
Zfp114	0
Zfp113	0
Zfp110	0
Zfp11	0
Olfr1404	0,70
Nlrp9c	0,70
Stra6	0,70
Olfr601	0,70
Hhat	0,70
4933402P03Rik	0,70
Gsg1l	0,70
Il1f9	0,70
Apof	0,70
Gramd1b	0,70
Hspa5	0,70
Tbx22	0,70
Ccdc185	0,70
Coro2b	0,70
Gp2	0,70
Hist1h2bj	0,70
Pdc	0,70
Lancl1	0,70
Cpxm2	0,70
Sapcd1	0,70
Tmem30b	0,70
Cd72	0,70
Rtn4ip1	0,70
Nova1	0,70
Kcnh2	0,70
Reg1	0,70
Pnpla6	0,70
Olfr675	0,70
Xpnpep2	0,70
Slc23a1	0,70
Slc46a1	0,70
Vps51	0,70
Tes	0,70
1700020N15Rik	0,70
Cmas	0,70
Trim12a	0,70
Akt1	0,70
Phf21b	0,70
Hykk	0,70
Rsu1	0,70
Pfkfb4	0,70
Pa2g4	0,70
Arhgef37	0,70
Lgals9	0,70
Lmx1b	0,70
Arid3b	0,70
Doc2b	0,70
Ubfd1	0,70
Cyp27a1	0,70
Atp9b	0,70
Ogfr	0,70
Fgg	0,69
Esr1	0,69
Man1c1	0,69
Cyp2d12	0,69
Zfp59	0,69
Wdtc1	0,69
Ccl2	0,69
Elf1	0,69
Rftn1	0,69
BRDN0000738169	0,69
BRDN0000737489	0,69
Dhdh	0,69
Gsc2	0,69
Rem1	0,69
Sufu	0,69
Mansc4	0,69
Smarcd1	0,69
Sox3	0,69
Agbl3	0,69
Il1a	0,69
Fbxl2	0,69
Tgfb3	0,69
Slc43a1	0,69
Edn2	0,69
Rmnd5a	0,69
Plcz1	0,69
Nbeal1	0,69
Kctd11	0,69
Tacstd2	0,69
3110057O12Rik	0,69
Ttc8	0,69
Tlr13	0
Adm2	0
Tbrg1	0
Drc7	0
Palmd	0
Aym1	0
Olfr1211	0
Bbs9	0
Wasf3	0
Fam196b	0
Fam196a	0
Dnajc28	0
Dnajc27	0
Tbrg4	0
Dnajc25	0
Dnajc24	0
Dnajc22	0
Dnajc21	0
Agr3	0
Apobec3	0
Hspb11	0
Apobec1	0
Plcxd1	0
Mtmr9	0
Apobec4	0
Scgb2b3	0
Mtmr4	0
Mtmr3	0
Mtmr2	0
Mtmr1	0
Isyna1	0
Olfr461	0
Cars	0
Man1a	0
Traip	0
Churc1	0
Abcb11	0
2410015M20Rik	0
Btc	0
Nck2	0
Sntn	0
Trabd2b	0
Carf	0
Tnnc1	0
Vmn1r230	0
Olfr177	0
Apba1	0
Helz2	0
Apba3	0
Apba2	0
Traf7	0
Rpap1	0
4931406B18Rik	0
Gdf10	0
Gpr50	0
Gpr55	0
2310002L09Rik	0
Agtr2	0
Myrf	0
Gm13088	0
Vmn1r233	0
Gstk1	0
Otop1	0
Abcb1b	0
Otop3	0
Car9	0
Scarf1	0
Rufy3	0
Acss3	0
Acss2	0
Acss1	0
Car1	0
Car6	0
Car7	0
Car4	0
Dsc1	0
Crtc1	0
Cldn23	0
Crtc3	0
Cyba	0
D17Wsu92e	0
Cldn22	0
Mesdc2	0,69
Ccdc183	0,69
Pyroxd2	0,69
Twist2	0,69
Myg1	0,69
Rpe65	0,69
Nexn	0,69
Aadat	0,69
Bfsp2	0,69
Polm	0,69
Dmpk	0,69
Unc13c	0,69
Olfr2	0,69
Ddo	0,69
Fbxo4	0,69
BRDN0000738081	0,69
Brpf1	0,69
Scgb2b24	0,69
Lyz1	0,69
Cep170b	0,69
Unc5a	0,69
Ticrr	0,69
Ldhb	0,69
Trpt1	0,69
Ltb4r2	0,69
Gipc3	0,69
Tcf15	0,69
Npr3	0,69
C130079G13Rik	0,69
Apc2	0,69
Rhox12	0,69
Ggt5	0,69
Ralgps2	0,69
Foxd1	0,69
Gm8298	0,69
Fam83e	0,69
Slco1a6	0,69
Awat1	0,69
1700067P10Rik	0,69
Pigyl	0,69
Tyw1	0,69
Ptch2	0,69
Isg20	0,69
Igfbp4	0,69
Lrrc2	0,69
Parl	0,69
Zmiz2	0,69
Erlin2	0,69
Cdan1	0,69
Ifitm1	0,69
Hnrnph2	0,69
Olfr285	0,69
BRDN0000738312	0,69
Tnni3	0,69
Cdh4	0,69
Srp19	0,69
Tnfrsf1b	0,69
H2-Aa	0,69
Limch1	0,69
Samt4	0,69
Anxa6	0,69
Tbc1d24	0,69
Prm3	0,69
Them4	0,69
BRDN0000737443	0,68
Pax9	0,68
Slc5a4b	0,68
Cyld	0,68
Ube2w	0,68
Smr3a	0,68
Car5a	0,68
Tmem25	0,68
Cyp2c50	0,68
Cdk11b	0,68
Galr1	0,68
Fbxo16	0,68
Epha7	0,68
Lmod1	0,68
Rbm41	0,68
Paqr5	0,68
Pcgf6	0,68
Tmsb15b2	0,68

Zfp109	0	BC017158	0,69	Ces5a	0	Dpysl3	0,68
Zfp106	0	Arhgap40	0,69	Mlip	0	Prok2	0,68
Zfp105	0	Satb2	0,69	Pgk2	0	Fstl3	0,68
Zfp101	0	Qsox1	0,69	Hint1	0	Cd8b1	0,68
Zfp1	0	Nfkbiz	0,69	Hint2	0	Olfr556	0,68
Zfml	0	Enpp4	0,69	Misp	0	Fam188b	0,68
Zfhx4	0	Bloc1s5	0,69	Hsf1	0	Cdh18	0,68
Zfhx3	0	Htr5a	0,69	Smtnl2	0	Fbxw24	0,68
Zfhx2	0	Jak2	0,69	Gm2a	0	Fanci	0,68
Zfc3h1	0	Exosc2	0,69	Hsf5	0	Ttc38	0,68
Zfat	0	Hoga1	0,69	Accsl	0	Mterf1b	0,68
Zfand6	0	Dnaja4	0,69	Rps6kb1	0	Rsad2	0,68
Zfand5	0	Pclo	0,69	Sgsh	0	Rdh8	0,68
Zfand3	0	C5ar2	0,69	8030462N17Rik	0	Afmid	0,68
Zfand2b	0	Ccdc142	0,69	Prlh	0	Olfr1199	0,68
Zfand1	0	Foxi2	0,69	Foxd1	0	Fam151b	0,68
Zer1	0	Mfsd6l	0,69	Naalad2	0	Mettl11b	0,68
Zeb2	0	Olfr393	0,69	Ctnnd2	0	Fam171a2	0,68
Zdhhc8	0	Kif5b	0,69	Drap1	0	Wnt16	0,68
Zdhhc7	0	Dmrt3	0,69	Trim80	0	Tppp	0,68
Zdhhc6	0	Rnf39	0,69	Cldn24	0	Rab38	0,68
Zdhhc5	0	Olfr23	0,69	Gramd3	0	Olfr282	0,68
Zdhhc4	0	Wdr53	0,69	Tmem88	0	Jph4	0,68
Zdhhc3	0	Snx15	0,69	Mbl2	0	Zfp37	0,68
Zdhhc25	0	Gm8660	0,69	Tmem81	0	Map1s	0,68
Zdhhc24	0	Bhlha9	0,69	Tmem80	0	Kif16b	0,68
Zdhhc22	0	Gtf2ird2	0,69	Tmem82	0	Olfr243	0,68
Zdhhc21	0	Grasp	0,69	Tec	0	Rp1l1	0,68
Zdhhc20	0	Olfr1054	0,69	Ppip5k1	0	Abcb10	0,68
Zdhhc2	0	Caly	0,69	H2afj	0	Slc34a2	0,68
Zdhhc18	0	Ccne1	0,69	Agtr1b	0	Cdc42se1	0,68
Zdhhc17	0	Olfr1437	0,69	Agtr1a	0	Nanos3	0,68
Zdhhc16	0	Pradc1	0,69	Sh3tc1	0	Atp5j2	0,68
Zdhhc14	0	BRDN0000738261	0,69	Eras	0	BC089597	0,68
Zdhhc13	0	Ermp1	0,69	Lypd6	0	Ncan	0,68
Zdhhc12	0	Pcyt1b	0,69	Gm17727	0	AI607873	0,68
Zdhhc11	0	Irx4	0,69	Lypd4	0	Ccdc63	0,68
Zdhhc1	0	Adam15	0,69	Lypd5	0	Fthl17	0,68
Zdbf2	0	Haus6	0,69	4932411E22Rik	0	Pcyox1	0,68
Zcrb1	0	Oit1	0,69	Lypd3	0	Vmn1r107	0,68
Zcchc9	0	Gpr34	0,69	Dntt	0	Hax1	0,68
Zcchc8	0	Tagln3	0,69	Lysmd4	0	Smarcd3	0,68
Zcchc7	0	Gck	0,69	Ttl	0	Olfr1189	0,68
Zcchc6	0	Cited1	0,69	Akr1d1	0	Pdss1	0,68
Zcchc5	0	Uggt2	0,68	Kiss1	0	Trappc3	0,68
Zcchc4	0	Snx20	0,68	Lypd8	0	Trappc6b	0,68
Zcchc3	0	Ambra1	0,68	Hypm	0	Csf2ra	0,68
Zcchc24	0	Evpl	0,68	Ddit4	0	Olfr998	0,68
Zcchc18	0	Dydc1	0,68	Aamp	0	Pprc1	0,68
Zcchc12	0	Smim24	0,68	Hypk	0	Chrnd	0,68
Zcchc11	0	Dock11	0,68	BC061237	0	Slc37a1	0,68
Zcchc10	0	Olfr738	0,68	Nxn	0	Sbk3	0,68
Zc4h2	0	Cntf	0,68	Sbsn	0	Defb43	0,68
Zc3hav1l	0	Arhgap28	0,68	Ccz1	0	S1pr2	0,68
Zc3h8	0	Gja6	0,68	Kars	0	Cplx3	0,68
Zc3h7b	0	Prss50	0,68	Dpy19l3	0	Fam228b	0,68
Zc3h7a	0	1700034O15Rik	0,68	Pnldc1	0	Kif14	0,68
Zc3h6	0	Renbp	0,68	Aipl1	0	Slc7a7	0,68
Zc3h4	0	Galntl5	0,68	3110035E14Rik	0	Car1	0,68
Zc3h18	0	Abcc2	0,68	Ccdc47	0	Olfr1239	0,68
Zc3h15	0	Pou3f4	0,68	Cald1	0	Afm	0,68
Zc3h13	0	Vmn1r160	0,68	Krtap6-5	0	Magea5	0,68
Zc3h12d	0	Fam134b	0,68	Ccdc43	0	Olfr521	0,68
Zc3h12c	0	Ephx4	0,68	Ccdc42	0	Cramp1l	0,68
Zc3h12b	0	Olfr507	0,68	Nrarp	0	Dbf4	0,67
Zc3h12a	0	Myh2	0,68	Epb4.1l5	0	Terf2	0,67
Zc3h11a	0	Papln	0,68	Olfr575	0	Trpm2	0,67
Zc3h10	0	Vmn2r59	0,68	Pggt1b	0	Ccdc61	0,67
Zc2hc1c	0	Gm8369	0,68	D7Ertd443e	0	Olfr159	0,67
Zc2hc1b	0	Tns4	0,68	Actl6a	0	Src	0,67
Zc2hc1a	0	Olfr203	0,68	Actl6b	0	Hsd17b12	0,67
Zbtb8os	0	Dcn	0,68	Tmem8c	0	Ehd1	0,67
Zbtb8b	0	Ercc6l2	0,68	Tmem8b	0	Fgf6	0,67
Zbtb7b	0	Gdf5	0,68	D130040H23Rik	0	Oscp1	0,67
Zbtb7a	0	Samd5	0,68	Pde8a	0	Scube2	0,67
Zbtb6	0	Nphs2	0,68	Gm21119	0	Slc2a9	0,67
Zbtb46	0	Pdk1	0,68	Gm4794	0	Tex13a	0,67
Zbtb45	0	Dhrs4	0,68	2510002D24Rik	0	Uhrf1bp1l	0,67
Zbtb44	0	Fabp2	0,68	Reps1	0	Swi5	0,67
Zbtb43	0	Klrc3	0,68	Vma21	0	Ssbp4	0,67
Zbtb42	0	Fn3krp	0,68	Gm4791	0	Dusp4	0,67
Zbtb41	0	Supt4a	0,68	Flna	0	Nek9	0,67

Zbtb40	0	Lamp5	0,68	Psen2	0	Zfp85	0,67
Zbtb4	0	Park7	0,68	Psen1	0	Vdac3	0,67
Zbtb38	0	Zic4	0,68	Nr2e3	0	Mapkapk2	0,67
Zbtb37	0	Scnn1a	0,68	Cks1brt	0	1700012A03Rik	0,67
Zbtb34	0	Zfp488	0,68	Fcnb	0	Slc35d1	0,67
Zbtb33	0	Krt42	0,68	Fcna	0	Olfr1256	0,67
Zbtb32	0	Zscan25	0,68	Mylip	0	Mapt	0,67
Zbtb26	0	Olfr1391	0,68	Gpatch2l	0	Phtf1	0,67
Zbtb25	0	Nell1	0,68	Kcnh2	0	Drd3	0,67
Zbtb24	0	Kbtbd12	0,68	Nrbp2	0	Olfr145	0,67
Zbtb22	0	1700129C05Rik	0,68	Xpnpep2	0	Tbx6	0,67
Zbtb21	0	Cdh2	0,68	Sgk3	0	Ccdc85c	0,67
Zbtb20	0	Olfr835	0,68	Orm1	0	Gm8898	0,67
Zbtb2	0	Olfr1022	0,68	Cbwd1	0	Ifi27l2b	0,67
Zbtb18	0	Plekhh3	0,68	Tmem258	0	BRDN0000737738	0,67
Zbtb17	0	Usp10	0,68	Arl2bp	0	Tspan1	0,67
Zbtb14	0	BRDN0000738306	0,68	Tmem256	0	Gapt	0,67
Zbtb12	0	Klk1b8	0,68	Diras2	0	Pla2g12a	0,67
Zbtb11	0	Slc25a13	0,68	Tmem253	0	Trip12	0,67
Zbtb10	0	Cfap20	0,68	Tmem251	0	Mmp17	0,67
Zbtb1	0	Il25	0,68	BRDN0000737861	0	Ints4	0,67
Zbed6	0	Zswim8	0,68	Rexo1	0	Cyp1a2	0,67
Zbed5	0	Klrc2	0,68	Rxfp1	0	Klk1b11	0,67
Zbed4	0	Rax	0,68	Gm5771	0	1700007K09Rik	0,67
Zbbx	0	Rgs17	0,68	Rexo2	0	Dgat1	0,67
Zar1l	0	Tmprss11a	0,68	Numb	0	Irgq	0,67
Zar1	0	Sp6	0,68	2610002M06Rik	0	Haus1	0,67
Zap70	0	Zfp105	0,68	Slc52a2	0	Srr	0,67
Zak	0	Phgr1	0,68	BRDN0000738046	0	Cma1	0,67
Zadh2	0	Rab12	0,68	Abcg1	0	Sh3gl2	0,67
Yy2	0	Vmn1r180	0,68	Abcg3	0	Slco2b1	0,67
Yy1	0	Serpina1f	0,68	Csl	0	Cpt1a	0,67
Ywhaz	0	Olfr986	0,68	BRDN0000738043	0	A630095E13Rik	0,67
Ywhaq	0	Olfr1045	0,68	BRDN0000738040	0	Sgsh	0,67
Ywhah	0	Gm5741	0,68	Adh6a	0	Rnase13	0,67
Ywhag	0	Yif1b	0,68	Abcg8	0	Nrk	0,67
Ywhae	0	Tcte2	0,68	Sox30	0	Ipo13	0,67
Ywhab	0	Add3	0,68	Ifnab	0	Coro2b	0,67
Ythdf3	0	Trpm2	0,68	BRDN0000738049	0	Grsf1	0,67
Ythdf2	0	Cyp2a12	0,68	Kdm4c	0	Zpbp2	0,67
Ythdf1	0	Nsun4	0,68	Kdm4b	0	BRDN0000738130	0,67
Ythdc2	0	Pcdhgb5	0,68	Kdm4a	0	Spred3	0,67
Ythdc1	0	Tgfb2	0,68	4930474N05Rik	0	Olfr1128	0,67
Yrdc	0	Snx4	0,68	Vmn1r183	0	Cygb	0,67
Ypel5	0	Ost4	0,68	Rpusd1	0	Cst10	0,67
Ypel4	0	Tgif2	0,68	Vmn1r181	0	Manbal	0,67
Ypel3	0	Zfp366	0,68	Vmn1r180	0	Lrif1	0,67
Ypel2	0	Gm14137	0,68	Dnal4	0	Fuz	0,67
Ypel1	0	Nobox	0,68	Fam178b	0	Psmd10	0,67
Yod1	0	Gnat1	0,67	4930578I06Rik	0	Vasp	0,67
Yme1l1	0	Gm14151	0,67	Dnal1	0	Mup21	0,67
Yipf7	0	Mgll	0,67	Otud6b	0	Rrh	0,67
Yipf6	0	Jam2	0,67	Otud6a	0	Il9	0,67
Yipf5	0	Wbp1	0,67	Tmub1	0	Plau	0,67
Yipf4	0	Brsk2	0,67	BRDN0000738197	0	Spaca7	0,67
Yipf3	0	Ctla4	0,67	C1s2	0	Pramef8	0,67
Yipf2	0	Notch1	0,67	Znrd1as	0	Lifr	0,67
Yipf1	0	Klf8	0,67	Col2a1	0	Unc79	0,67
Yif1b	0	Tex24	0,67	BRDN0000737863	0	Nfkbid	0,67
Yif1a	0	Tas2r103	0,67	BRDN0000737920	0	Slc8a3	0,67
Yes1	0	Tmco5	0,67	Olfr1394	0	Pdzk1	0,67
Yeats4	0	Mgarp	0,67	Olfr955	0	Tfg	0,67
Yeats2	0	Col9a1	0,67	Scn9a	0	Scgb1b20	0,67
Ydjc	0	Gata5	0,67	Tmem41a	0	Armc5	0,67
Ybx3	0	Gm4070	0,67	Fitm2	0	Tspan5	0,67
Ybx2	0	Cep68	0,67	Olfr1467	0	Atp1b3	0,67
Ybx1	0	Bbs2	0,67	Lman1	0	Prkag3	0,67
Ybey	0	Mup6	0,67	Lman2	0	U2af2	0,67
Yars2	0	1110017D15Rik	0,67	Tmtc3	0	Itprip	0,67
Yars	0	Txndc9	0,67	Fgf14	0	Etnk2	0,67
Yap1	0	Ceacam19	0,67	Zfp804a	0	2310067B10Rik	0,67
Yae1d1	0	Tmsb10	0,67	Col4a1	0	Tacr1	0,67
Xylt1	0	Gm1527	0,67	Olfr1461	0	Vmn1r200	0,67
Xylb	0	Gm4871	0,67	BRDN0000738262	0	Pigg	0,67
Xxylt1	0	Gsr	0,67	Arfip1	0	Ddx26b	0,67
Xrra1	0	9530077C05Rik	0,67	Arfip2	0	Slc25a30	0,67
Xrn2	0	Naa10	0,67	BRDN0000738261	0	Nckap5	0,67
Xrn1	0	Mtcp1	0,67	Necap1	0	Chac1	0,67
Xrcc6bp1	0	Scyl2	0,67	Vmn1r238	0	Gle1	0,67
Xrcc6	0	Olfr666	0,67	1700016H13Rik	0	Ms4a15	0,67
Xrcc5	0	Edc4	0,67	Gnas	0	Akap2	0,67
Xrcc4	0	Ccr10	0,67	Vmn1r234	0	Prr22	0,67

Xrcc2	0	Tmem141	0,67	Vmn1r237	0	Ranbp2	0,67
Xrcc1	0	Brip1	0,67	Vmn1r236	0	Hapln4	0,67
Xpr1	0	Mrpl47	0,67	Vmn1r231	0	Wscd1	0,67
Xpot	0	BC052040	0,67	Dcst1	0	Ifna5	0,67
Xpo7	0	Dym	0,67	Gnaz	0	BRDN0000737609	0,67
Xpo6	0	Bambi	0,67	Vmn1r232	0	Myh4	0,66
Xpo4	0	Rnf183	0,67	Napg	0	Dlc1	0,66
Xpo1	0	Zscan4b	0,67	Slco1b2	0	Hyou1	0,66
Xpnpep1	0	Myo1d	0,67	Napb	0	Cldn2	0,66
Xpc	0	Lurap1	0,67	Napa	0	Ifna9	0,66
Xntrpc	0	Plekha8	0,67	Saysd1	0	Dctn5	0,66
Xlr5b	0	Slc2a5	0,67	Gnal	0	Lmo7	0,66
Xlr4b	0	Gcsh	0,67	Snx18	0	Vezf1	0,66
Xlr4a	0	Cd79b	0,67	BRDN0000737544	0	Blvra	0,66
Xlr3b	0	Cdc37l1	0,67	1700025F22Rik	0	Arl6	0,66
Xlr3a	0	Tmem117	0,67	Os9	0	Clca3a2	0,66
Xlr	0	Mgam	0,67	Vmac	0	Nox1	0,66
Xkr9	0	Pdcd10	0,67	Gm6484	0	Eif1ax	0,66
Xkr8	0	Serpinb9e	0,67	Tubb2a	0	C8a	0,66
Xkr7	0	Cd3g	0,67	Bmf	0	Prrc2b	0,66
Xkr6	0	BRDN0000737477	0,67	AI593442	0	Rbm43	0,66
Xkr4	0	Klb	0,67	Olfr469	0	Nkx2-5	0,66
Xk	0	Abhd11	0,67	Pon3	0	Rexo2	0,66
Xirp1	0	Tmem159	0,67	9030617O03Rik	0	AF251705	0,66
Xiap	0	Gpr4	0,67	Nsmce1	0	Satb1	0,66
Xdh	0	Gipc3	0,67	Angpt2	0	Nefh	0,66
Xcr1	0	Galk1	0,67	S100a10	0	Serpina3n	0,66
Xcl1	0	Pet117	0,67	S100a13	0	Rprml	0,66
Xbp1	0	Olfr688	0,67	Utp18	0	BRDN0000738260	0,66
Xaf1	0	Olfr1474	0,67	Dmbx1	0	Uts2r	0,66
Xab2	0	Otop1	0,67	Lrrc71	0	Lpin3	0,66
Wwp2	0	Llph	0,67	Fam21	0	Ssxb5	0,66
Wwp1	0	Fgd4	0,67	Hells	0	Hexdc	0,66
Wwox	0	Tnfsf11	0,67	Rrp1	0	Olfml3	0,66
Wwc2	0	Ngrn	0,67	Faah	0	Olfr224	0,66
Wwc1	0	Sema3c	0,67	Snurf	0	Hexa	0,66
Wtip	0	Gaa	0,67	Hyou1	0	Eif3h	0,66
Wscd1	0	Bst1	0,67	Plod2	0	Mc3r	0,66
Wsb2	0	Olfr508	0,67	Plod3	0	Sprr2d	0,66
Wsb1	0	Zfp68	0,67	Kcnh8	0	Hibch	0,66
Wrnip1	0	Ephb4	0,67	Nek4	0	Caskin2	0,66
Wrn	0	Lsm14a	0,67	Skp1a	0	Dap3	0,66
Wrb	0	Bcat1	0,67	Olfr1411	0	Sspo	0,66
Wrap73	0	Nsun7	0,67	Ushbp1	0	Wdr4	0,66
Wrap53	0	Ldb1	0,67	Olfr1413	0	Dnpep	0,66
Wnt9b	0	Rbm28	0,67	Olfr1412	0	Gm16501	0,66
Wnt9a	0	Upk3b	0,67	Olfr1415	0	Slc52a3	0,66
Wnt8a	0	Nmd3	0,67	Olfr1414	0	Myl6b	0,66
Wnt7b	0	Arhgap5	0,67	Olfr1419	0	Tpp1	0,66
Wnt7a	0	Slc25a20	0,66	Olfr1418	0	Aco1	0,66
Wnt5b	0	Zbtb43	0,66	Gabbr2	0	Tubgcp6	0,66
Wnt5a	0	Rgs21	0,66	Smad6	0	Oosp1	0,66
Wnt4	0	Yy2	0,66	Olfml2a	0	BRDN0000737407	0,66
Wnt3a	0	Fam179b	0,66	Pir	0	Mtmr14	0,66
Wnt3	0	Rspo3	0,66	Ugp2	0	Loh12cr1	0,66
Wnt2b	0	Slc35a5	0,66	Cpped1	0	Gnai2	0,66
Wnt2	0	Olfr1440	0,66	Myo9b	0	Angptl1	0,66
Wnt16	0	Klf13	0,66	Cep126	0	Prss2	0,66
Wnt10a	0	Mboat1	0,66	Ugt8a	0	Kif13b	0,66
Wnt1	0	Dyx1c1	0,66	Smyd4	0	Dicer1	0,66
Wnk4	0	Hgf	0,66	Mydgf	0	Fosl2	0,66
Wnk3	0	3110082J24Rik	0,66	Fam107b	0	Olfr1355	0,66
Wnk2	0	Med13l	0,66	Osm	0	Iba57	0,66
Wnk1	0	Taar8b	0,66	Smyd1	0	Ftcd	0,66
Wls	0	Kmt2b	0,66	Smyd2	0	Hdac5	0,66
Wiz	0	Klk1b21	0,66	Smyd3	0	Cdt1	0,66
Wisp3	0	Pex7	0,66	Dock10	0	Spats1	0,66
Wisp2	0	Trmt10c	0,66	Dock11	0	BRDN0000737957	0,66
Wisp1	0	Lcor	0,66	Lrrc72	0	Scyl2	0,66
Wipi1	0	Rbmxl1	0,66	Entpd7	0	Echdc2	0,66
Wipf1	0	Pogk	0,66	Aox1	0	Clec4d	0,66
Wif1	0	BC051019	0,66	Aox2	0	Slc36a3	0,66
Whsc1l1	0	Chm	0,66	Entpd4	0	Nrn1	0,66
Wfs1	0	Eif3e	0,66	Entpd3	0	Olfr1250	0,66
Wfikkn2	0	Ptprk	0,66	Olfr805	0	Slc35g2	0,66
Wfikkn1	0	Iglon5	0,66	Gm4884	0	Lce1m	0,66
Wfdc9	0	Apcs	0,66	Snap23	0	Unc5b	0,66
Wfdc8	0	Tbc1d14	0,66	Olfr808	0	Spred1	0,66
Wfdc6b	0	Cep76	0,66	Olfr809	0	Psg17	0,66
Wfdc6a	0	Zfp7	0,66	Avpr2	0	Isl2	0,66
Wfdc5	0	Rbbp7	0,66	Rtl1	0	Grin3b	0,66
Wfdc3	0	Atg2a	0,66	Agxt	0	Angel2	0,66

Wfdc21	0	Sdc1	0,66	Fam60a	0	Adcy4	0,66
Wfdc2	0	Il11ra1	0,66	Sh3bp5l	0	Myo1c	0,66
Wfdc18	0	Pomk	0,66	Entpd8	0	4933436I01Rik	0,66
Wfdc17	0	Cep250	0,66	Zbtb40	0	Orm3	0,66
Wfdc16	0	Dsel	0,66	Zbtb41	0	Ipo9	0,66
Wfdc15b	0	Tubd1	0,66	Zbtb42	0	Paqr6	0,66
Wfdc15a	0	Gm4952	0,66	Zbtb43	0	Hist2h3c2	0,66
Wfdc12	0	Fam192a	0,66	Zbtb44	0	Mtif2	0,66
Wfdc11	0	Ccl19	0,66	Gm6034	0	Prss1	0,66
Wfdc1	0	Tmc5	0,66	Zbtb46	0	Cyp46a1	0,66
Wee2	0	Pax1	0,66	Mob2	0	Nup85	0,66
Wee1	0	Il17ra	0,66	Zbtb49	0	Tgds	0,66
Wdtc1	0	Pcdha10	0,66	Map1lc3b	0	Cers5	0,66
Wdsub1	0	BRDN0000738355	0,66	Dync2h1	0	Myl7	0,66
Wdr95	0	Ptchd1	0,66	Mob4	0	Rfwd3	0,66
Wdr93	0	Rragc	0,66	Cyp2c50	0	B3gnt9	0,66
Wdr91	0	Ppfia2	0,66	Gapvd1	0	Cxcl14	0,66
Wdr89	0	Clvs1	0,66	Ngfrap1	0	Caly	0,66
Wdr86	0	Rhox3h	0,66	Olfr1347	0	Vmn1r28	0,66
Wdr83	0	Ocln	0,66	Prdx6	0	Tiam2	0,66
Wdr82	0	Slc22a14	0,66	Prdx5	0	Mtmr1	0,66
Wdr81	0	Fxyd5	0,66	Prdx4	0	Pithd1	0,66
Wdr77	0	Pcp2	0,66	Prdx3	0	Lhfpl1	0,66
Wdr75	0	Gng12	0,66	Prdx2	0	Zkscan7	0,66
Wdr74	0	Hivep3	0,66	Prdx1	0	Pbld1	0,65
Wdr73	0	BRDN0000738180	0,66	Astn1	0	Xrcc3	0,65
Wdr72	0	Pgm5	0,66	Tgoln1	0	Idh2	0,65
Wdr70	0	Gm10024	0,66	Mgat5b	0	Usp44	0,65
Wdr7	0	Usp1	0,66	Tmem246	0	Smad7	0,65
Wdr64	0	Aspg	0,66	Fth1	0	Net1	0,65
Wdr63	0	Pcid2	0,66	Panx1	0	Gata1	0,65
Wdr62	0	Srp54a	0,66	Prr32	0	Dnajb13	0,65
Wdr61	0	BRDN0000737964	0,66	Panx3	0	Trp53i13	0,65
Wdr60	0	Fahd2a	0,66	Clca3a2	0	Wfdc2	0,65
Wdr6	0	Wwp1	0,66	Prr36	0	Ccnf	0,65
Wdr5b	0	Calcoco2	0,66	Dpf2	0	Ccl17	0,65
Wdr59	0	Magee2	0,66	Olfr310	0	Galnt10	0,65
Wdr54	0	Tc2n	0,66	Olfr1346	0	Ccdc181	0,65
Wdr5	0	Rmnd1	0,66	Olfr462	0	Nceh1	0,65
Wdr48	0	Hrh2	0,66	Mplkip	0	Slc27a5	0,65
Wdr47	0	Rb1cc1	0,66	Olfr763	0	Abracl	0,65
Wdr46	0	Asb1	0,66	Stfa2l1	0	Phox2a	0,65
Wdr45	0	Gm11437	0,66	Phyhd1	0	Dbt	0,65
Wdr43	0	Olfr1170	0,66	Ppp2r2a	0	Rnf34	0,65
Wdr41	0	Piwil2	0,66	Spag11b	0	Il3	0,65
Wdr4	0	Rhox2d	0,66	Olfr312	0	Olfr1232	0,65
Wdr37	0	5430427O19Rik	0,66	Pqbp1	0	Abca14	0,65
Wdr36	0	Gtpbp2	0,66	P4ha2	0	Galm	0,65
Wdr35	0	Plat	0,65	P4ha3	0	Myrip	0,65
Wdr33	0	Chd4	0,65	Nkiras2	0	Ggnbp2	0,65
Wdr31	0	Rheb	0,65	P4ha1	0	Ocstamp	0,65
Wdr3	0	Olfr1298	0,65	Mup21	0	C87977	0,65
Wdr27	0	Wdr90	0,65	Mup20	0	1600012H06Rik	0,65
Wdr25	0	Krtap13	0,65	Abi3bp	0	Klf8	0,65
Wdr24	0	Gm2a	0,65	Ppp2r2b	0	BRDN0000738246	0,65
Wdr20	0	Epb4.1l5	0,65	Olfr315	0	Ms4a4b	0,65
Wdr19	0	Cdh3	0,65	Wdr41	0	Olfr378	0,65
Wdr18	0	Rnase2a	0,65	Wdr43	0	Ppard	0,65
Wdr13	0	Tas2r136	0,65	Olfr316	0	St7	0,65
Wdr12	0	Nradd	0,65	Wdr46	0	Ahcyl2	0,65
Wdr11	0	Adgrg1	0,65	Wdr47	0	Pglyrp1	0,65
Wdr1	0	Gabpa	0,65	Wdr48	0	Adm	0,65
Wdpcp	0	Igfn1	0,65	Prss41	0	Tsc22d3	0,65
Wdfy4	0	Itga1	0,65	Sergef	0	Duox1	0,65
Wdfy3	0	Zbtb40	0,65	Wipf2	0	Armcx2	0,65
Wdfy2	0	Pex11a	0,65	Prodh	0	Smurf2	0,65
Wdfy1	0	Tanc2	0,65	Pramel7	0	Nat10	0,65
Wbscr28	0	Olfr414	0,65	Pramel5	0	Sema3f	0,65
Wbscr27	0	Fem1a	0,65	Narfl	0	Dpp7	0,65
Wbscr22	0	Tomm40l	0,65	Pramel3	0	Tmem189	0,65
Wbscr17	0	Btbd6	0,65	Use1	0	1700019B03Rik	0,65
Wbscr16	0	Apol9b	0,65	Vps53	0	Pinx1	0,65
Wbp5	0	Olfr735	0,65	Gm960	0	Tmc4	0,65
Wbp2	0	Igdcc3	0,65	Psma4	0	BRDN0000738169	0,65
Wbp1l	0	Gckr	0,65	Olfr1340	0	Pdf	0,65
Wbp1	0	Plxna1	0,65	Vps54	0	Serpina1d	0,65
Wasl	0	Gpa33	0,65	Egln3	0	Wdr41	0,65
Wash1	0	Ntpcr	0,65	Slc41a1	0	Pask	0,65
Wasf2	0	Tmem26	0,65	Egln1	0	Mybl2	0,65
Wasf1	0	Rprd1a	0,65	Lnx2	0	Bcl11a	0,65
Was	0	Olfr1137	0,65	Gxylt2	0	Nov	0,65
Wars2	0	Tas2r143	0,65	Lnx1	0	Eps8l2	0,65

Wars	0	Pnoc	0,65	Wnt10a	0	Dfnb59	0,65
Wap	0	Gstm5	0,65	Wnt10b	0	Sbp	0,65
Wac	0	Olfr1062	0,65	Olfr48	0	Hoxc12	0,65
Vwf	0	Abcb4	0,65	Abhd14a	0	Grik3	0,65
Vwde	0	Cst12	0,65	Psg16	0	Commd9	0,65
Vwce	0	4921530L21Rik	0,65	BRDN0000737987	0	Jmjd6	0,65
Vwc2l	0	Znrf2	0,65	Tmem241	0	Mgat4c	0,65
Vwc2	0	Fkbp2	0,65	Slc41a2	0	Adgrb2	0,65
Vwa9	0	Kcnmb3	0,65	Cyp2c54	0	Tomm34	0,65
Vwa7	0	Nes	0,65	Apeh	0	Tll1	0,65
Vwa5b2	0	Vmn2r54	0,65	Tarsl2	0	Gm3604	0,65
Vwa5a	0	Pla2g4b	0,65	Olfr45	0	Bpifc	0,65
Vwa3a	0	Wnt5b	0,65	Lrrc19	0	BRDN0000738113	0,65
Vwa2	0	Ptgr1	0,65	Eno2	0	Nucb2	0,65
Vwa1	0	Ubtfl1	0,65	Stam	0	Olfr1082	0,65
Vtcn1	0	Rnf2	0,65	Fam120b	0	Vmn1r100	0,65
Vta1	0	Cldn14	0,65	Olfr43	0	Prkcsh	0,65
Vsx2	0	Cd79a	0,65	Fam120a	0	Ankrd35	0,65
Vsx1	0	5430435G22Rik	0,65	Mastl	0	Ano2	0,65
Vstm5	0	Il17a	0,65	Pla1a	0	Mmp3	0,65
Vstm4	0	Spg11	0,65	Stard3nl	0	Mdc1	0,65
Vstm2l	0	Myb	0,65	Sf3b5	0	Ppp1r12a	0,65
Vstm2b	0	Gm13040	0,65	Cfap74	0	Trpv4	0,65
Vstm2a	0	1110065P20Rik	0,65	Eepd1	0	Dynap	0,65
Vsnl1	0	Aqp7	0,65	Olfr834	0	Ctsb	0,65
Vsig10	0	Rnf114	0,65	Rpf2	0	Phldb2	0,65
Vrtn	0	Atp13a1	0,65	4921539E11Rik	0	Akap11	0,65
Vrk2	0	Try10	0,65	2310022A10Rik	0	1700042B14Rik	0,65
Vrk1	0	Pla2g16	0,65	Hira	0	Zfp609	0,65
Vps9d1	0	Slc22a4	0,65	BRDN0000737941	0	Gpr20	0,65
Vps8	0	Saa2	0,65	Nagpa	0	Ms4a18	0,64
Vps53	0	LOC100038947	0,65	Olfr836	0	Irx6	0,64
Vps51	0	Rmdn3	0,65	Ttc9	0	Ephb1	0,64
Vps4b	0	Tlr11	0,65	Phc3	0	Trib2	0,64
Vps4a	0	Tmem35	0,65	Cep85l	0	Zfp329	0,64
Vps41	0	Krtap24-1	0,65	Sf3b1	0	Mmp21	0,64
Vps39	0	Kcnh4	0,65	Park7	0	Vti1b	0,64
Vps37d	0	Ttc36	0,65	Klra10	0	Zfp946	0,64
Vps37a	0	Npvf	0,65	Grik4	0	Dcaf12l1	0,64
Vps36	0	Crh	0,65	Grik5	0	Zmym3	0,64
Vps35	0	Agr2	0,65	Park2	0	Tubb6	0,64
Vps33b	0	AA986860	0,65	Nbea	0	Dlgap1	0,64
Vps33a	0	1110059G10Rik	0,65	Fam213b	0	Aldh1a3	0,64
Vps29	0	Khk	0,65	Nxpe4	0	Fcgr4	0,64
Vps26b	0	BC051628	0,65	Nxpe5	0	Tmem52b	0,64
Vps26a	0	BRDN0000737452	0,65	Tnfrsf1b	0	Cox11	0,64
Vps25	0	Rfpl4	0,65	Lrrc17	0	Rab13	0,64
Vps18	0	Trim15	0,65	Tmem161b	0	Ipcef1	0,64
Vps16	0	Swsap1	0,65	Stx5a	0	Astl	0,64
Vps13d	0	Mndal	0,65	Hspb6	0	Ankrd26	0,64
Vps13b	0	Cdc42ep1	0,65	Lhx5	0	Oog3	0,64
Vps13a	0	Parvb	0,65	Aplnr	0	Vmn2r69	0,64
Vps11	0	Abhd17c	0,65	Exoc3l	0	Hace1	0,64
Vpreb2	0	Aifm1	0,65	Gm2016	0	Calm4	0,64
Vpreb1	0	Olfr1162	0,65	E130308A19Rik	0	Svs3b	0,64
Vprbp	0	Trp53i11	0,65	Anapc16	0	Krt2	0,64
Vopp1	0	Ccdc181	0,65	Anapc11	0	Psmb2	0,64
Vnn3	0	Dsg2	0,65	Anapc10	0	Hist1h2bm	0,64
Vnn1	0	Cyb561a3	0,64	Ret	0	Senp3	0,64
Vmp1	0	Pdha2	0,64	Foxn1	0	Adora1	0,64
Vmo1	0	Plscr1	0,64	Mast1	0	Cysltr2	0,64
Vmn2r98	0	Tars2	0,64	Mast2	0	Cd247	0,64
Vmn2r97	0	Klhl14	0,64	Setd1b	0	Tm4sf4	0,64
Vmn2r96	0	Sertm1	0,64	Mast4	0	Gm21379	0,64
Vmn2r95	0	Eddm3b	0,64	Gbe1	0	Sept8	0,64
Vmn2r92	0	Entpd8	0,64	Lhx9	0	Lce1d	0,64
Vmn2r90	0	Cyp4f15	0,64	Cnpy1	0	3010026O09Rik	0,64
Vmn2r9	0	Spock1	0,64	Cd59b	0	Mcoln1	0,64
Vmn2r89	0	Gm14501	0,64	S100a8	0	Klhdc9	0,64
Vmn2r88	0	Rgs9	0,64	Mgst2	0	Arsk	0,64
Vmn2r87	0	Mdfic	0,64	Ggcx	0	Sox2	0,64
Vmn2r86	0	Rprd2	0,64	Gm10408	0	Snapin	0,64
Vmn2r85	0	N4bp1	0,64	Spdyb	0	Ube3a	0,64
Vmn2r84	0	Cacng3	0,64	Psip1	0	Ear14	0,64
Vmn2r83	0	Cd2ap	0,64	Cand2	0	Nuggc	0,64
Vmn2r82	0	BRDN0000737583	0,64	Cand1	0	Lman1	0,64
Vmn2r80	0	Bod1	0,64	Cyp3a44	0	Nos1	0,64
Vmn2r8	0	4930428D18Rik	0,64	Krt33b	0	Epb4.1l4b	0,64
Vmn2r79	0	Oasl1	0,64	Krt33a	0	Pcsk4	0,64
Vmn2r78	0	Egfem1	0,64	Rnf44	0	Olfr157	0,64
Vmn2r77	0	Mrm1	0,64	Gm10406	0	4930596D02Rik	0,64
Vmn2r76	0	Ear14	0,64	Trrap	0	Slc25a45	0,64

Vmn2r75	0	Bnc1	0,64	Rnf40	0	Ccdc158	0,64
Vmn2r74	0	Adam3	0,64	Foxa1	0	Ttf1	0,64
Vmn2r73	0	Kctd2	0,64	Kpna6	0	Sptbn1	0,64
Vmn2r72	0	Fermt1	0,64	Foxa3	0	Rnaset2a	0,64
Vmn2r71	0	Olfr1261	0,64	Cyb5d2	0	Tnp1	0,64
Vmn2r70	0	Tmem214	0,64	Kpna3	0	Cyp2j12	0,64
Vmn2r7	0	Lrrc28	0,64	Kpna2	0	Tgfa	0,64
Vmn2r69	0	Ldah	0,64	Kpna1	0	Pgap1	0,64
Vmn2r68	0	Tm7sf3	0,64	Phldb1	0	Xrcc6bp1	0,64
Vmn2r67	0	Gna13	0,64	Spink13	0	Vmn1r58	0,64
Vmn2r66	0	Atg3	0,64	Spink11	0	Cypt4	0,64
Vmn2r65	0	Nxpe2	0,64	S100a6	0	Tbx18	0,64
Vmn2r63	0	Acot10	0,64	Usp49	0	Cldn18	0,64
Vmn2r62	0	Smad7	0,64	Ttll8	0	Rae1	0,64
Vmn2r61	0	Mptx2	0,64	Usp45	0	Gm10229	0,64
Vmn2r60	0	Ccdc53	0,64	Usp44	0	Tcl1b5	0,64
Vmn2r6	0	Olfr1322	0,64	Cnot10	0	Tlr12	0,64
Vmn2r59	0	BRDN0000737835	0,64	Usp40	0	Aipl1	0,64
Vmn2r58	0	Olfr67	0,64	Usp43	0	Uimc1	0,64
Vmn2r57	0	D630039A03Rik	0,64	Sbspon	0	Vmn2r20	0,64
Vmn2r56	0	Kbtbd7	0,64	Ska1	0	Polr3f	0,64
Vmn2r55	0	Prss3	0,64	Ska3	0	BRDN0000737649	0,64
Vmn2r54	0	Cycs	0,64	Ska2	0	Krt222	0,64
Vmn2r53	0	Fcgrt	0,64	Coq9	0	Adam39	0,64
Vmn2r52	0	Olfm1	0,64	Timm21	0	Lypd4	0,64
Vmn2r51	0	BRDN0000738079	0,64	Amacr	0	Ftsj2	0,64
Vmn2r5	0	Isca1	0,64	Gdpgp1	0	Gpx6	0,64
Vmn2r49	0	Slc4a5	0,64	Coq6	0	Tmem117	0,64
Vmn2r48	0	Iqgap1	0,64	Coq3	0	Pcdh18	0,64
Vmn2r47	0	Fcgr3	0,64	Ilf2	0	Cdc37	0,64
Vmn2r45	0	Hecw1	0,64	Smurf1	0	9030617O03Rik	0,64
Vmn2r44	0	Galk2	0,64	Cyp51	0	Olfr247	0,64
Vmn2r42	0	Pabpc6	0,64	1700019G17Rik	0	Acbd3	0,64
Vmn2r41	0	Pdcd5	0,64	Nhlh1	0	Oxtr	0,64
Vmn2r40	0	Prkd1	0,64	Zpld1	0	Ica1	0,64
Vmn2r39	0	Lrig1	0,64	Dapk2	0	Cort	0,64
Vmn2r38	0	Lin7a	0,64	Pgam1	0	Ttf2	0,64
Vmn2r37	0	Krt7	0,64	Scp2	0	Gm5127	0,64
Vmn2r35	0	Olfr419	0,64	Gbx1	0	Pla2g1b	0,64
Vmn2r32	0	Ppp1ca	0,64	Spata31d1a	0	Acvr1c	0,64
Vmn2r31	0	Kmt2d	0,64	Spata31d1c	0	Ephb3	0,64
Vmn2r30	0	Defb33	0,64	Dlk1	0	Rab28	0,64
Vmn2r3	0	Farp2	0,64	Gngt2	0	Meikin	0,64
Vmn2r29	0	Lcat	0,64	Gngt1	0	Pcdhga5	0,64
Vmn2r28	0	Psmb11	0,64	Dlk2	0	Gm8677	0,64
Vmn2r27	0	Eml1	0,64	Cartpt	0	Raver2	0,64
Vmn2r25	0	Trmt1	0,64	Zfp212	0	Tgfbr1	0,64
Vmn2r24	0	Lrrc51	0,64	Dhx32	0	Myf6	0,64
Vmn2r23	0	Sparc	0,64	Dhx33	0	Snx25	0,64
Vmn2r22	0	Tspan10	0,64	Dhx34	0	Gm6377	0,64
Vmn2r20	0	Olfr33	0,64	Dhx35	0	Ube2a	0,64
Vmn2r2	0	Cyp2j8	0,64	Dhx37	0	Tspan12	0,63
Vmn2r19	0	Neurod2	0,64	Olfr231	0	Fyn	0,63
Vmn2r18	0	Srrm1	0,64	Iqcd	0	Pfkl	0,63
Vmn2r16	0	Pik3ap1	0,64	Sdad1	0	Rab40b	0,63
Vmn2r15	0	Irf9	0,64	Gm10670	0	Mafk	0,63
Vmn2r14	0	Krt10	0,64	Cops2	0	BRDN0000738338	0,63
Vmn2r13	0	Slc1a5	0,64	Cops3	0	Olfr695	0,63
Vmn2r124	0	Tmbim1	0,64	Olfr239	0	Sec31a	0,63
Vmn2r121	0	Etfdh	0,64	Cops6	0	Mtpn	0,63
Vmn2r120	0	Plekhf1	0,64	Cops4	0	Pcdhga7	0,63
Vmn2r118	0	Tmem229b	0,64	Cops5	0	4930579F01Rik	0,63
Vmn2r117	0	Zfp189	0,64	Lrrc10	0	1700102P08Rik	0,63
Vmn2r116	0	Cldn12	0,64	Hspb3	0	Mapkbp1	0,63
Vmn2r115	0	Psmc3ip	0,64	AI661453	0	Nup62	0,63
Vmn2r114	0	Zfp810	0,64	Olfr1061	0	Nek1	0,63
Vmn2r113	0	BRDN0000737612	0,64	Gm4301	0	Tbc1d4	0,63
Vmn2r112	0	Acot11	0,64	Fbxl15	0	Cdkl4	0,63
Vmn2r110	0	Agfg2	0,64	Gm4302	0	Tnfaip8l2	0,63
Vmn2r109	0	4930468A15Rik	0,64	Gm4305	0	Tead1	0,63
Vmn2r108	0	C87414	0,64	Senp5	0	Epm2aip1	0,63
Vmn2r107	0	Asap2	0,64	Gm4307	0	Tcte2	0,63
Vmn2r106	0	Scn9a	0,64	Dhcr24	0	Syt10	0,63
Vmn2r104	0	Grid1	0,64	1700024G13Rik	0	Slc5a8	0,63
Vmn2r103	0	Pgr15l	0,64	Ccnb2	0	Ctdnep1	0,63
Vmn2r102	0	Il1rl2	0,64	Zfp280c	0	Calm2	0,63
Vmn2r101	0	Olfr566	0,64	Zfp280d	0	Hspa1b	0,63
Vmn2r100	0	4933405L10Rik	0,64	BC048671	0	Ypel4	0,63
Vmn2r10	0	Dnlz	0,64	Cyp2c55	0	Btbd2	0,63
Vmn2r1	0	Fancl	0,64	C4bp	0	Samhd1	0,63
Vmn1r95	0	Cfap58	0,64	Blm	0	Dpyd	0,63
Vmn1r91	0	Fkbp3	0,64	Smim1	0	Cyp4a12b	0,63

Vmn1r89	0	Cers3	0,64	Nov	0	Olfr617	0,63
Vmn1r88	0	Get4	0,64	Olfr1066	0	Drc7	0,63
Vmn1r85	0	Kdf1	0,64	Blk	0	Odf3b	0,63
Vmn1r84	0	Maml3	0,64	Dscam	0	Cd4	0,63
Vmn1r83	0	Mcph1	0,64	Kcna10	0	Jade3	0,63
Vmn1r82	0	Kcnd1	0,64	Hck	0	Gzme	0,63
Vmn1r81	0	Nostrin	0,64	Numbl	0	Kcnk5	0,63
Vmn1r80	0	H2afj	0,64	Prg3	0	Arl10	0,63
Vmn1r79	0	Sprr2i	0,63	Cobll1	0	Ncapg	0,63
Vmn1r77	0	1110004E09Rik	0,63	Brca1	0	Znrf1	0,63
Vmn1r76	0	Ttc21a	0,63	Cux2	0	Hmx2	0,63
Vmn1r74	0	Tmie	0,63	Brca2	0	Ssc4d	0,63
Vmn1r73	0	Sftpc	0,63	Olfr589	0	Olfr666	0,63
Vmn1r72	0	Madd	0,63	Fcamr	0	St14	0,63
Vmn1r71	0	Grip2	0,63	Lama3	0	Creld2	0,63
Vmn1r70	0	Tex30	0,63	Olfr585	0	Ly96	0,63
Vmn1r7	0	Gm20867	0,63	Olfr584	0	Nle1	0,63
Vmn1r69	0	Htr1d	0,63	Tmeff2	0	Gls2	0,63
Vmn1r68	0	Armcx6	0,63	Olfr586	0	Hoxa2	0,63
Vmn1r67	0	Men1	0,63	Acap1	0	Vwa5a	0,63
Vmn1r66	0	Ahsa2	0,63	Acap2	0	1700001C19Rik	0,63
Vmn1r65	0	Thbs4	0,63	Olfr582	0	Man2b1	0,63
Vmn1r63	0	Vmn1r63	0,63	Baz1a	0	Ak1	0,63
Vmn1r62	0	Noxo1	0,63	Pfn1	0	Rpl29	0,63
Vmn1r61	0	Tmem47	0,63	U2af1l4	0	Rogdi	0,63
Vmn1r60	0	Ndufb7	0,63	Pfn3	0	Tyrp1	0,63
Vmn1r59	0	Ccl26	0,63	Ep400	0	Kctd13	0,63
Vmn1r58	0	Olfr716	0,63	Slc7a15	0	Bag4	0,63
Vmn1r57	0	Vmn1r15	0,63	4931431F19Rik	0	Bcl6b	0,63
Vmn1r56	0	Cbll1	0,63	Urb2	0	Fgfbp1	0,63
Vmn1r55	0	Tspo	0,63	Serpinh1	0	Ascl3	0,63
Vmn1r54	0	Kbtbd8	0,63	1700017B05Rik	0	Fam209	0,63
Vmn1r53	0	Prpf39	0,63	Bco1	0	Zmat4	0,63
Vmn1r51	0	Selp	0,63	Ubash3a	0	Iqcb1	0,63
Vmn1r50	0	Mylk3	0,63	Ubash3b	0	Mme	0,63
Vmn1r49	0	Slco1a6	0,63	Rdh12	0	Gm20867	0,63
Vmn1r48	0	Esd	0,63	1700018C11Rik	0	Tlcd2	0,63
Vmn1r47	0	Rbfa	0,63	Ldha	0	Retnlg	0,63
Vmn1r46	0	Rassf10	0,63	Rdh13	0	Gm10220	0,63
Vmn1r44	0	2900092C05Rik	0,63	Mrps23	0	Pou4f2	0,63
Vmn1r43	0	Fam229a	0,63	Frem3	0	B3gnt8	0,63
Vmn1r42	0	Efcab3	0,63	Frem2	0	Srpk3	0,63
Vmn1r41	0	Cep85	0,63	Frem1	0	Sp7	0,63
Vmn1r40	0	Adm	0,63	Stox1	0	Inca1	0,63
Vmn1r4	0	Il13ra1	0,63	Stox2	0	Hnrnpd	0,63
Vmn1r36	0	Gm9376	0,63	Slc7a13	0	Trim8	0,63
Vmn1r35	0	Clec14a	0,63	Snx19	0	Vgll1	0,63
Vmn1r34	0	Pcdha7	0,63	Lipa	0	Selk	0,63
Vmn1r32	0	Olfr259	0,63	Lipc	0	Kat5	0,63
Vmn1r31	0	Mad2l1bp	0,63	4930544G11Rik	0	Prdm11	0,63
Vmn1r30	0	Wdr7	0,63	Lipe	0	Egfl6	0,63
Vmn1r3	0	Rgs9bp	0,63	Lipg	0	Clybl	0,63
Vmn1r28	0	Gins1	0,63	Dtd1	0	Rac2	0,63
Vmn1r27	0	BRDN0000737403	0,63	Lipi	0	Shbg	0,63
Vmn1r25	0	E430018J23Rik	0,63	Hc	0	Kif3b	0,63
Vmn1r24	0	Nfat5	0,63	Lipk	0	Axl	0,63
Vmn1r238	0	Lrrc8b	0,63	Cldn20	0	Wfdc6a	0,63
Vmn1r237	0	Gm14345	0,63	Lipm	0	Ldhal6b	0,63
Vmn1r236	0	Ctbp1	0,63	Zfp426	0	Gss	0,63
Vmn1r235	0	Sv2a	0,63	Man1b1	0	Klre1	0,63
Vmn1r234	0	Prorsd1	0,63	Lipn	0	Zfp934	0,63
Vmn1r232	0	Bcas1	0,63	Ttk	0	Esp6Esp5	0,63
Vmn1r231	0	Fzd6	0,63	Mgst3	0	Fancb	0,63
Vmn1r230	0	Nexn	0,63	Tmod3	0	Tmem79	0,63
Vmn1r229	0	H2bfm	0,63	Samd7	0	Krt25	0,63
Vmn1r228	0	Tbc1d8	0,63	Vmn1r78	0	Gsdma2	0,63
Vmn1r226	0	Olfr1507	0,63	Vmn1r79	0	Gprc6a	0,63
Vmn1r225	0	Mga	0,63	Nnat	0	Iscu	0,63
Vmn1r224	0	Rimbp2	0,63	Vmn1r74	0	Pebp1	0,63
Vmn1r223	0	Zkscan5	0,63	Vmn1r75	0	Olfr699	0,63
Vmn1r221	0	Try5	0,63	Vmn1r76	0	Pcbp3	0,63
Vmn1r220	0	Casd1	0,63	Vmn1r77	0	Pom121	0,63
Vmn1r219	0	Grb14	0,63	Vmn1r70	0	Sh2b2	0,63
Vmn1r218	0	Ralb	0,63	Vmn1r71	0	Grid2	0,63
Vmn1r217	0	Cthrc1	0,63	Sucnr1	0	Sar1b	0,63
Vmn1r216	0	St3gal5	0,63	Vmn1r73	0	Ube2c	0,63
Vmn1r215	0	Clec9a	0,63	Rln3	0	Olfr1500	0,63
Vmn1r212	0	Pyroxd2	0,63	Diras1	0	Ccl3	0,63
Vmn1r211	0	Hlcs	0,63	Zfp422	0	Pusl1	0,63
Vmn1r21	0	Serpina1b	0,63	Clspn	0	Lnp	0,63
Vmn1r209	0	Oscp1	0,63	Zfp961	0	Cacybp	0,63
Vmn1r204	0	Jam3	0,63	Zfp960	0	Oacyl	0,63

Vmn1r203	0	Slc4a10	0,63	Zfp963	0	Pigw	0,63
Vmn1r201	0	Jup	0,63	Oca2	0	BRDN0000738347	0,63
Vmn1r200	0	Tulp1	0,63	Grhpr	0	4932429P05Rik	0,63
Vmn1r2	0	Gm3604	0,63	Dkk3	0	Vcam1	0,63
Vmn1r199	0	Ccl27b	0,63	Cul4a	0	BRDN0000737624	0,62
Vmn1r196	0	Zfp938	0,63	Galr2	0	Calcoco2	0,62
Vmn1r195	0	Trib2	0,63	Galr1	0	Echs1	0,62
Vmn1r194	0	Kif3b	0,63	Cul4b	0	Tgm2	0,62
Vmn1r193	0	Gatsl3	0,63	Qrfp	0	Nck2	0,62
Vmn1r192	0	Guk1	0,63	0610007P14Rik	0	Ctsa	0,62
Vmn1r191	0	Acap3	0,63	Gm13547	0	Tm4sf1	0,62
Vmn1r19	0	Syn2	0,63	BRDN0000737546	0	Cd163l1	0,62
Vmn1r188	0	Arhgef16	0,63	Cryba2	0	Dtx2	0,62
Vmn1r185	0	Ankrd13d	0,63	Cryba1	0	4921517D22Rik	0,62
Vmn1r184	0	Slc44a3	0,63	BRDN0000737545	0	Muc1	0,62
Vmn1r181	0	Nudt7	0,63	BRDN0000737542	0	Serpina7	0,62
Vmn1r18	0	Krt76	0,63	BRDN0000737543	0	1700123K08Rik	0,62
Vmn1r179	0	Otor	0,63	BRDN0000737540	0	Tnnt1	0,62
Vmn1r177	0	Krt35	0,63	Nek6	0	Urgcp	0,62
Vmn1r176	0	Rps6kl1	0,63	Nek7	0	Baiap3	0,62
Vmn1r175	0	Tfec	0,63	Aldh1a3	0	Ap3d1	0,62
Vmn1r173	0	Pdia2	0,63	Nek5	0	Gm11596	0,62
Vmn1r172	0	Map3k2	0,63	Nek2	0	Foxj1	0,62
Vmn1r171	0	Erich2	0,63	Nek1	0	Amdhd1	0,62
Vmn1r17	0	Nup54	0,63	Hist1h2aa	0	Alg9	0,62
Vmn1r169	0	Traf7	0,63	B3gnt3	0	Zfp69	0,62
Vmn1r168	0	Idh3g	0,63	Hba-x	0	Spata6	0,62
Vmn1r167	0	2810403A07Rik	0,63	Ndfip1	0	Fstl1	0,62
Vmn1r163	0	Cdkl2	0,62	Nek8	0	Nsl1	0,62
Vmn1r160	0	Sult1c2	0,62	Nek9	0	Slc9a9	0,62
Vmn1r159	0	Gm6710	0,62	Ccdc18	0	Fsd2	0,62
Vmn1r158	0	1700030K09Rik	0,62	4921509C19Rik	0	Ogdh	0,62
Vmn1r157	0	Nfatc3	0,62	Hist1h2ab	0	Plp1	0,62
Vmn1r151	0	Exo1	0,62	Idua	0	Notch1	0,62
Vmn1r15	0	Ckb	0,62	Tnfsf13	0	Ccdc163	0,62
Vmn1r149	0	Olfr780	0,62	Tnfsf12	0	D230025D16Rik	0,62
Vmn1r148	0	Scgb1b24	0,62	Tnfsf11	0	Cyp2d10	0,62
Vmn1r142	0	Cyp2d22	0,62	Psg17	0	Ret	0,62
Vmn1r14	0	Map3k7cl	0,62	Cep170b	0	Ppp2cb	0,62
Vmn1r137	0	Ehmt2	0,62	Tnfsf15	0	2010002M12Rik	0,62
Vmn1r135	0	Cxcr4	0,62	Tnfsf14	0	Txn2	0,62
Vmn1r132	0	Mapk8	0,62	Olfr3	0	Gm1564	0,62
Vmn1r131	0	Eno4	0,62	Tnfsf18	0	Fyco1	0,62
Vmn1r13	0	Tsfm	0,62	Psg18	0	Pgr15l	0,62
Vmn1r129	0	Ccdc85b	0,62	Psg19	0	Nde1	0,62
Vmn1r126	0	Pqlc1	0,62	B3gnt9	0	Olfr1204	0,62
Vmn1r124	0	E230008N13Rik	0,62	Setd1a	0	Atp5b	0,62
Vmn1r123	0	Ntn1	0,62	5730507C01Rik	0	Wnt7a	0,62
Vmn1r122	0	Ppp1r15a	0,62	Gm7534	0	Olfr652	0,62
Vmn1r121	0	Spata5	0,62	Gm2012	0	Hnrnph1	0,62
Vmn1r120	0	Srp72	0,62	BRDN0000737438	0	Ddx20	0,62
Vmn1r119	0	Pdzd4	0,62	Aim1	0	Inpp5k	0,62
Vmn1r117	0	Sun3	0,62	Rab26	0	Hnrnpf	0,62
Vmn1r116	0	Tdrp	0,62	Arhgef10l	0	Ric8b	0,62
Vmn1r115	0	Arfgap1	0,62	Gcsam	0	Idnk	0,62
Vmn1r114	0	Mospd2	0,62	Slc30a1	0	Ccdc129	0,62
Vmn1r112	0	Cnksr3	0,62	Gbx2	0	Maats1	0,62
Vmn1r11	0	Pxk	0,62	4930415O20Rik	0	Olfr56	0,62
Vmn1r107	0	Zfp36	0,62	Ccdc134	0	Mov10l1	0,62
Vmn1r104	0	3110043O21Rik	0,62	Timp2	0	Usp11	0,62
Vmn1r103	0	Prlhr	0,62	Timp1	0	Rnf40	0,62
Vmn1r100	0	Gm7849	0,62	Senp8	0	Klk1b27	0,62
Vmn1r10	0	Rps6kc1	0,62	Ccdc64	0	Slc26a9	0,62
Vmn1r1	0	Tbx15	0,62	Senp6	0	Mrpl3	0,62
Vmac	0	Aif1l	0,62	Olfr1062	0	Gtf2ird2	0,62
Vma21	0	Cep78	0,62	Stambp	0	4930486L24Rik	0,62
Vldlr	0	Gm6792	0,62	Senp3	0	Pirt	0,62
Vkorc1l1	0	Olfr1314	0,62	BC100530	0	Rgn	0,62
Vkorc1	0	Ccnl2	0,62	Chmp6	0	H2-T3	0,62
Vit	0	Ak5	0,62	Ddr1	0	Slc39a4	0,62
Vipr2	0	Bag5	0,62	Chmp5	0	Dhodh	0,62
Vipr1	0	Olfr862	0,62	BRDN0000737439	0	1700080E11Rik	0,62
Vipas39	0	Lrfn2	0,62	Hnrnph3	0	Lima1	0,62
Vimp	0	Nit2	0,62	Cyp4a30b	0	Prss54	0,62
Vim	0	Otud7b	0,62	B3gnt4	0	Crispld2	0,62
Vil1	0	Camk2n2	0,62	B3gnt5	0	Maged1	0,62
Vhl	0	Lyrm4	0,62	Prdm14	0	Henmt1	0,62
Vgll4	0	Gusb	0,62	B3gnt7	0	Vmn2r9	0,62
Vgll3	0	Papss1	0,62	Ostm1	0	Pcsk7	0,62
Vgll2	0	Epc2	0,62	Il6ra	0	Olfr609	0,62
Vgll1	0	Zdhhc4	0,62	B3gnt2	0	Olfr1156	0,62
Vgf	0	Pak1ip1	0,62	Il13ra2	0	Mib1	0,62

Vezf1	0	Arf5	0,62	Olfr970	0	Kif1b	0,62
Veph1	0	Tcaf1	0,62	4930449I24Rik	0	Nkiras1	0,62
Vegfc	0	Ankrd27	0,62	B3gnt8	0	Ifna4	0,62
Vegfb	0	Tctex1d1	0,62	Gcnt7	0	Ang5	0,62
Vegfa	0	Emc3	0,62	Lce1e	0	Gm16390	0,62
Vdr	0	Gpr63	0,62	Rpl10a	0	Olfr317	0,62
Vdac1	0	Rps3	0,62	Calhm2	0	Ncf1	0,62
Vcpkmt	0	Sbno2	0,62	Rpl10l	0	2310061I04Rik	0,62
Vcpip1	0	Cyp11a1	0,62	Mogat2	0	Dnaja1	0,62
Vcp	0	Ms4a1	0,62	Lgsn	0	Hif1an	0,62
Vcl	0	Bpifb4	0,62	Fam170b	0	Olfr510	0,62
Vcan	0	Phf11b	0,62	Vmn2r26	0	Rab36	0,62
Vcam1	0	Soga3	0,62	Vmn2r27	0	2300002M23Rik	0,62
Vbp1	0	Tbp	0,62	Vmn2r24	0	Lage3	0,62
Vax2	0	Tubgcp6	0,62	Vmn2r25	0	Nqo1	0,62
Vax1	0	BRDN0000737755	0,62	Vmn2r22	0	Gpr139	0,62
Vav3	0	Tgif1	0,62	Slx4	0	Slc4a1ap	0,62
Vav1	0	Pcdhb3	0,62	Vmn2r20	0	Fam114a1	0,62
Vat1l	0	Ercc6l	0,62	Pnkp	0	Msln	0,62
Vat1	0	Atp6v0d1	0,62	Selenbp1	0	2310039H08Rik	0,62
Vash2	0	Lmln	0,62	Selenbp2	0	Cpa2	0,62
Vars2	0	Cideb	0,62	Csf1	0	Fads6	0,62
Vars	0	Slc24a2	0,62	Csf2	0	Coq10a	0,62
Vapb	0	Selplg	0,62	Vmn2r29	0	AI182371	0,62
Vapa	0	Dcaf12l1	0,62	Appl1	0	Rmdn2	0,62
Vangl1	0	Zbtb8b	0,62	Appl2	0	Slc23a3	0,62
Vamp8	0	Olfr502	0,62	Pnkd	0	Sgtb	0,62
Vamp3	0	Pla2g12b	0,62	Aim2	0	Atp13a4	0,62
Vamp2	0	Klhdc10	0,62	Prnp	0	Arhgap25	0,62
V1ra8	0	Wdr63	0,62	Slfn5	0	Abcc9	0,61
Uxt	0	BC021891	0,62	Pqlc3	0	Wfdc13	0,61
Uxs1	0	Il17d	0,62	Pqlc2	0	D330045A20Rik	0,61
Uvssa	0	Tmem180	0,61	Pqlc1	0	Pcdha12	0,61
Uvrag	0	G2e3	0,61	Dclre1a	0	Glis1	0,61
Uty	0	Sept11	0,61	Sycn	0	Ogfod2	0,61
Uts2b	0	Grin2c	0,61	Amph	0	Ift27	0,61
Uts2	0	Edem3	0,61	Baz1b	0	Dcaf6	0,61
Utp23	0	Tmprss6	0,61	Zfp583	0	6430550D23Rik	0,61
Utp20	0	4930519G04Rik	0,61	Zfp580	0	Olfr594	0,61
Utp18	0	Fam210b	0,61	Crtam	0	Ctnnd2	0,61
Utp15	0	Pla2r1	0,61	BC021785	0	Rab40c	0,61
Utp14b	0	Corin	0,61	Lce1k	0	Slc35c1	0,61
Utp11l	0	Lpcat4	0,61	Gpr39	0	BRDN0000737761	0,61
Utf1	0	Thap3	0,61	Mypn	0	Camp	0,61
Ust	0	Lat2	0,61	AV320801	0	Nrp1	0,61
Uspl1	0	Chit1	0,61	Olfr1008	0	Tnfsfm13	0,61
Usp9y	0	H2-DMb2	0,61	Gpr33	0	Trp53i11	0,61
Usp9x	0	Micu3	0,61	Cd2ap	0	A630001G21Rik	0,61
Usp8	0	Tmem251	0,61	Gpr34	0	4930524B15Rik	0,61
Usp7	0	F7	0,61	Gpr37	0	Naaa	0,61
Usp6nl	0	Ildr2	0,61	Vmn2r23	0	Acsbg1	0,61
Usp54	0	Krtap19-2	0,61	Stx8	0	Gm8994	0,61
Usp51	0	Dffb	0,61	Pof1b	0	Cux1	0,61
Usp50	0	Nfx1	0,61	Trak2	0	Gtf2a1	0,61
Usp5	0	Pphln1	0,61	Stx2	0	Klhl10	0,61
Usp49	0	Twsg1	0,61	Col27a1	0	Ppm1d	0,61
Usp46	0	Pkd1l2	0,61	Stx7	0	Zfp90	0,61
Usp44	0	Pigw	0,61	Stx6	0	1700092M07Rik	0,61
Usp43	0	BRDN0000737996	0,61	9230110F15Rik	0	Toporsl	0,61
Usp40	0	Fam169a	0,61	Tsacc	0	Rpp25l	0,61
Usp4	0	Gm8909	0,61	1700023F06Rik	0	Olfr639	0,61
Usp39	0	Usp9y	0,61	Sh3bgr	0	Prpf6	0,61
Usp38	0	Utrn	0,61	Pram1	0	Cilp	0,61
Usp37	0	Gale	0,61	Gp9	0	Ildr1	0,61
Usp36	0	Vta1	0,61	1700001L19Rik	0	Tlr1	0,61
Usp35	0	Kiss1	0,61	Gm4944	0	2700060E02Rik	0,61
Usp34	0	Serpina5	0,61	Fzd10	0	Olfr1453	0,61
Usp33	0	Slamf6	0,61	Ctnnb1	0	Phex	0,61
Usp32	0	Acss3	0,61	Olfr832	0	Ccdc42	0,61
Usp31	0	Zdhhc18	0,61	Ndufa5	0	Olfr1166	0,61
Usp30	0	BRDN0000737953	0,61	Fam105a	0	Srprb	0,61
Usp3	0	Tal2	0,61	Vmn2r28	0	Otop1	0,61
Usp29	0	Peli3	0,61	Edc4	0	Dkk4	0,61
Usp28	0	Olfr1331	0,61	Heca	0	Nradd	0,61
Usp27x	0	Kank3	0,61	Avpi1	0	Nubp1	0,61
Usp26	0	Mvp	0,61	Deptor	0	Rgs10	0,61
Usp25	0	Pkn2	0,61	Csf3	0	Fam73a	0,61
Usp24	0	Zfp318	0,61	Nek10	0	Olfr849	0,61
Usp22	0	Olfr1423	0,61	Asap1	0	Bub1	0,61
Usp21	0	Cdk2ap1	0,61	D430041D05Rik	0	Sp5	0,61
Usp20	0	Rinl	0,61	Asap3	0	Odc1	0,61
Usp2	0	Hmx1	0,61	Kat8	0	Stx8	0,61

Usp19	0	Acot1	0,61	BRDN0000738329	0	Tnfaip6	0,61
Usp18	0	Rap2c	0,61	Tmem121	0	Syne2	0,61
Usp17ld	0	Pfkl	0,61	Nuf2	0	Arl5a	0,61
Usp17lc	0	Bsg	0,61	Lmntd2	0	Cpsf6	0,61
Usp17lb	0	Eif3j1	0,61	Kat5	0	2210016F16Rik	0,61
Usp17la	0	C1galt1	0,61	Hyls1	0	Btla	0,61
Usp16	0	Hacd3	0,61	Kat7	0	Htr2c	0,61
Usp15	0	Fam24a	0,61	Pfas	0	Slx4	0,61
Usp14	0	Cts7	0,61	BRDN0000737570	0	BRDN0000737881	0,61
Usp13	0	Fgf16	0,61	Ly96	0	Tac2	0,61
Usp12	0	Golga3	0,61	Luc7l	0	Cntfr	0,61
Usp11	0	Apoa1	0,61	Gsg1l	0	Rpusd2	0,61
Usp10	0	Esyt1	0,61	Mfsd12	0	Fam189a2	0,61
Usp1	0	Syt8	0,61	BRDN0000738300	0	Doc2a	0,61
Uso1	0	Olfr473	0,61	Wnt9b	0	C1ql3	0,61
Usmg5	0	P2ry4	0,61	Wnt9a	0	Gpr12	0,61
Ushbp1	0	Pcdha9	0,61	Ndufa9	0	Pira1	0,61
Ush2a	0	Smpd3	0,61	Coro1b	0	Cyb5b	0,61
Ush1g	0	Ints10	0,61	Rab38	0	Erbb2	0,61
Ush1c	0	BRDN0000737799	0,61	Arl4c	0	Sdccag8	0,61
Usf2	0	Cuedc2	0,61	Krt86	0	Vmn1r2	0,61
Use1	0	Naga	0,61	Arhgap31	0	Bmp15	0,61
Usb1	0	Rnaseh2a	0,61	Dclre1b	0	Asah2	0,61
Uros	0	Gtf2h5	0,61	Ccdc23	0	Ngrn	0,61
Uroc1	0	Mb	0,61	Ccdc22	0	Casp8ap2	0,61
Urm1	0	P2ry12	0,61	Sag	0	Podn	0,61
Uri1	0	Fam98c	0,61	Ccdc24	0	Oasl2	0,61
Urgcp	0	Osr2	0,61	Ccdc27	0	Hmga2	0,61
Urb2	0	Bcdin3d	0,61	Dclre1c	0	Sgce	0,61
Urb1	0	1700006A11Rik	0,61	Apol7a	0	Sft2d2	0,61
Urah	0	Dhrs9	0,61	Apol7c	0	Ankrd13d	0,61
Urad	0	Nfic	0,61	Apol7b	0	Wnt9a	0,61
Uqcrq	0	Ctbs	0,61	Apol7e	0	Vps54	0,61
Uqcrh	0	Rhox4g	0,61	Vegfa	0	Morn1	0,61
Uqcrfs1	0	Manf	0,61	Crtap	0	Kif26a	0,61
Uqcrc2	0	Sos1	0,61	Fcf1	0	Tmem80	0,61
Uqcrc1	0	Amelx	0,61	Arhgap35	0	Ctrc	0,61
Uqcr11	0	Sec14l1	0,60	Timmdc1	0	Gm15293	0,61
Uqcr10	0	1810043H04Rik	0,60	Extl3	0	Olfr993	0,61
Uqcc3	0	Gbf1	0,60	Lrch2	0	Serpini1	0,61
Uqcc2	0	Kif19a	0,60	BRDN0000737927	0	BRDN0000737593	0,61
Uqcc1	0	Gm3376	0,60	Uxs1	0	Fscn3	0,61
Uprt	0	Mapk12	0,60	Asxl1	0	Whamm	0,61
Upp2	0	Dlx6	0,60	Ism1	0	Fcamr	0,61
Upp1	0	Jkamp	0,60	Asxl2	0	Dscc1	0,61
Upk3bl	0	B4galt4	0,60	Fibp	0	Hhatl	0,60
Upk3b	0	Fbxl16	0,60	Pxmp2	0	9430015G10Rik	0,60
Upk3a	0	Cpa6	0,60	Lrrc51	0	Cep128	0,60
Upk1b	0	Dbn1	0,60	Letmd1	0	Tbc1d16	0,60
Upk1a	0	Olfr605	0,60	Pycard	0	Stx4a	0,60
Upf3b	0	Prss57	0,60	Gabrd	0	Sh3bgr	0,60
Upf3a	0	Siglech	0,60	Tia1	0	2610528A11Rik	0,60
Upf2	0	Btaf1	0,60	Lrsam1	0	Hadhb	0,60
Upf1	0	Cyp2c44	0,60	Armc12	0	Chrna2	0,60
Upb1	0	2610034B18Rik	0,60	Pcdha2	0	Vmn2r67	0,60
Uox	0	Strada	0,60	Ptges3l	0	Pls1	0,60
Unkl	0	4930548H24Rik	0,60	Kcnmb3	0	Stmnd1	0,60
Unk	0	Tprg	0,60	Gm13083	0	Foxq1	0,60
Uncx	0	Crct1	0,60	Pcdha7	0	Setbp1	0,60
Unc93b1	0	BRDN0000738341	0,60	Pcdha4	0	Trim46	0,60
Unc93a	0	Swi5	0,60	Ccsap	0	Olfr885	0,60
Unc80	0	Klra10	0,60	Arf3	0	Krtap4-16	0,60
Unc79	0	Atxn7	0,60	Cep97	0	1700109H08Rik	0,60
Unc5d	0	Pln	0,60	BRDN0000737693	0	Sncaip	0,60
Unc5cl	0	Gml	0,60	Scrt2	0	Cldn13	0,60
Unc5b	0	Jagn1	0,60	Gm5795	0	Cbx2	0,60
Unc5a	0	Rhox4f	0,60	Cacna2d3	0	Krt77	0,60
Unc50	0	Fcho2	0,60	Gm5797	0	Tcf3	0,60
Unc45b	0	Siah3	0,60	Gm5796	0	Ccl26	0,60
Unc45a	0	Prr3	0,60	Cacna2d4	0	Slc22a12	0,60
Unc13c	0	Atp8b2	0,60	BRDN0000737735	0	Tamm41	0,60
Unc13b	0	Mt2	0,60	Rpn1	0	Soat2	0,60
Unc119	0	Zfp386	0,60	Rpn2	0	Dpf2	0,60
Umod	0	Mum1	0,60	BRDN0000737736	0	BC053393	0,60
Ulk3	0	Fbxo9	0,60	BRDN0000737731	0	Sh3d19	0,60
Ulk2	0	Arrb2	0,60	BRDN0000738065	0	BRDN0000738077	0,60
Ulk1	0	Ssxb5	0,60	Olfr684	0	Nudt8	0,60
Uimc1	0	Arap3	0,60	BRDN0000738067	0	Zfp276	0,60
Uhrf2	0	Atp1b4	0,60	3110001I22Rik	0	Suox	0,60
Uhrf1	0	Fam47c	0,60	Lhx1	0	Stk40	0,60
Uhmk1	0	Iqgap2	0,60	Dguok	0	Slc22a7	0,60
Ugt8a	0	Ncoa4	0,60	BRDN0000737739	0	Als2cl	0,60

Ugt3a2	0	Vmn1r77	0,60	Mill2	0	Gm5615	0,60
Ugt3a1	0	2310002L09Rik	0,60	BC021891	0	Nlgn2	0,60
Ugt2b5	0	Acad8	0,60	Tmem182	0	Lin7b	0,60
Ugt2b38	0	Atad2	0,60	Tmem180	0	S1pr4	0,60
Ugt2b37	0	Olfr111	0,60	Tmem186	0	Olfr20	0,60
Ugt2b36	0	Kazald1	0,60	I830077J02Rik	0	Rasal2	0,60
Ugt2b35	0	Prdm8	0,60	Pdcd7	0	Zdhhc23	0,60
Ugt2b34	0	Slmap	0,60	Pdcd4	0	Oxct2b	0,60
Ugt2a3	0	Hepacam	0,60	Kdm2b	0	Tbc1d21	0,60
Ugt2a2	0	Nfe2l3	0,60	Pdcd2	0	Myo1e	0,60
Ugt1a6b	0	Prkaa2	0,60	Map3k7cl	0	Olfr592	0,60
Ugt1a6a	0	Gab1	0,60	Adprhl1	0	Gimap6	0,60
Ugt1a5	0	Dcstamp	0,60	Ndufv3	0	Bmp7	0,60
Ugt1a10	0	Olfr348	0,60	Ndufv2	0	Lhx2	0,60
Uggt2	0	Asb15	0,60	Cys1	0	Pou5f1	0,60
Uggt1	0	Zfp367	0,60	Dnase1l1	0	Arap2	0,60
Ugdh	0	Notch3	0,60	Dnase1l2	0	4930428D18Rik	0,60
Ugcg	0	Dlst	0,60	Dnase1l3	0	Zfp810	0,60
Ufsp2	0	Olfr1225	0,60	Col15a1	0	Cisd1	0,60
Ufsp1	0	Fmnl1	0,60	Atp6v1g3	0	Fgf12	0,60
Ufl1	0	Grp	0,60	Atp6v1g2	0	Mpp4	0,60
Ufd1l	0	Fam120a	0,60	Atp6v1g1	0	Pla2g2d	0,60
Ufc1	0	Ern1	0,60	Txnrd2	0	Hmgcr	0,60
Uevld	0	Tmem170b	0,60	Txnrd3	0	Ciz1	0,60
Ucp3	0	Ptcra	0,60	Txnrd1	0	Sod1	0,60
Ucp1	0	Tbata	0,60	Dnase2a	0	BRDN0000738163	0,60
Ucn3	0	Prl8a6	0,60	Proz	0	Cst9	0,60
Ucn2	0	BRDN0000737382	0,60	Acvr1c	0	Fcf1	0,60
Ucn	0	Pramef17	0,60	Acvr1b	0	Pura	0,60
Ucma	0	D5Ertd579e	0,60	BRDN0000737579	0	Hkdc1	0,60
Uck2	0	3110079O15Rik	0,60	Zfp346	0	Rbm28	0,60
Uchl5	0	Ttll8	0,60	Acot12	0	Cwc15	0,60
Uchl3	0	Gpr160	0,60	Ino80	0	Ogdhl	0,60
Uchl1	0	Rassf1	0,60	Nars	0	Mrpl35	0,60
Ubxn7	0	Olfr870	0,59	Actrt1	0	Pcdha6	0,60
Ubxn6	0	Slc9a4	0,59	Hace1	0	1700019O17Rik	0,60
Ubxn2a	0	Cryab	0,59	Oxct1	0	Apoc4	0,60
Ubxn1	0	Acsm3	0,59	Dync1h1	0	Pcbp4	0,60
Ubtfl1	0	Olfr18	0,59	Ndufa4l2	0	Robo4	0,60
Ubtf	0	Pbx1	0,59	Narf	0	Tmem184b	0,60
Ubtd2	0	Olfr685	0,59	Proc	0	BRDN0000737876	0,60
Ubtd1	0	Stx12	0,59	Dpep3	0	Kremen1	0,60
Ubr7	0	Vnn3	0,59	Ext2	0	Olfr957	0,60
Ubr5	0	Iqsec3	0,59	Prnd	0	Gm7714	0,60
Ubr4	0	Six4	0,59	Ttll7	0	Magea4	0,60
Ubr2	0	Nab2	0,59	Ttll6	0	Rabggta	0,60
Ubr1	0	Olfr577	0,59	Ttll1	0	Eif2b5	0,60
Ubqlnl	0	Olfr1288	0,59	Lonrf1	0	Gm10697	0,60
Ubqln4	0	BRDN0000738288	0,59	Ttll3	0	Kcnj9	0,60
Ubqln3	0	Chmp7	0,59	Ttll2	0	Olfr371	0,60
Ubqln1	0	Slc45a4	0,59	4933408B17Rik	0	Kmo	0,60
Ubp1	0	Endod1	0,59	Upk3bl	0	Egr2	0,60
Ubox5	0	Tigd3	0,59	Lemd2	0	Gimap3	0,60
Ubn2	0	Olfr1378	0,59	D2hgdh	0	Znrf4	0,60
Ubn1	0	Pnpla2	0,59	Clnk	0	Klhl31	0,60
Ubl7	0	Il4	0,59	Adam34	0	Gsdmc2	0,60
Ubl5	0	Ceacam2	0,59	Ifitm1	0	Alpl	0,60
Ubl4b	0	Ppp2r2a	0,59	Kcnv2	0	Ptk2b	0,60
Ubl4	0	Tbx5	0,59	Adamtsl5	0	Mybl1	0,60
Ubl3	0	Lce1l	0,59	Abtb1	0	Efs	0,60
Ubiad1	0	Ppm1f	0,59	Fyco1	0	Atad2b	0,60
Ube4b	0	Rarres2	0,59	Sar1a	0	Cbfb	0,60
Ube4a	0	Tspan6	0,59	Sar1b	0	BRDN0000738133	0,60
Ube3c	0	Olfr518	0,59	Nkx2-5	0	Pank2	0,60
Ube3b	0	Tacr2	0,59	Pik3r5	0	Pcdhga11	0,60
Ube3a	0	Dnali1	0,59	Gpa33	0	Arsg	0,60
Ube2w	0	Ypel4	0,59	Adam33	0	Camk2a	0,60
Ube2v2	0	Gria2	0,59	Dagla	0	Fam107b	0,60
Ube2u	0	Vax2	0,59	Olfr1388	0	Hap1	0,60
Ube2t	0	Pcdhgb4	0,59	Daglb	0	Prdm16	0,59
Ube2s	0	Sorbs3	0,59	Casq1	0	Atxn1l	0,59
Ube2r2	0	Gstm6	0,59	Adam30	0	Rsph3b	0,59
Ube2ql1	0	Fgf18	0,59	Ccdc158	0	Ifrd2	0,59
Ube2q2	0	BRDN0000737833	0,59	Casq2	0	Cd38	0,59
Ube2q1	0	Serpinb9c	0,59	Olfr1381	0	Gm5592	0,59
Ube2n	0	Ass1	0,59	Olfr1380	0	Dydc2	0,59
Ube2m	0	BRDN0000738099	0,59	Olfr1383	0	Fbf1	0,59
Ube2l3	0	Cd164	0,59	BC089597	0	Cib1	0,59
Ube2j1	0	Id3	0,59	Olfr1385	0	Cyp1a1	0,59
Ube2i	0	Ccdc89	0,59	Olfr1384	0	Aff1	0,59
Ube2h	0	Mab21l2	0,59	Olfr1387	0	Iqsec3	0,59
Ube2g2	0	Sertad2	0,59	Olfr1386	0	Ncbp2	0,59

Ube2g1	0
Ube2f	0
Ube2e2	0
Ube2e1	0
Ube2dnl2	0
Ube2dnl1	0
Ube2d2b	0
Ube2d2a	0
Ube2d1	0
Ube2cbp	0
Ube2c	0
Ube2b	0
Ubd	0
Ubc	0
Ubb	0
Ubash3b	0
Ubash3a	0
Ubap2l	0
Ubap1	0
Ubald2	0
Ubac2	0
Ubac1	0
Uba6	0
Uba52	0
Uba3	0
Uba2	0
Uba1y	0
Uba1	0
Uap1l1	0
Uaca	0
U2af2	0
U2af1l4	0
U2af1	0
Tyw5	0
Tyw1	0
Tysnd1	0
Tyrp1	0
Tyrobp	0
Tyro3	0
Tyr	0
Tyms	0
Tyk2	0
Txnrd2	0
Txnrd1	0
Txnl4b	0
Txnl4a	0
Txnl1	0
Txnip	0
Txndc8	0
Txndc2	0
Txndc17	0
Txndc16	0
Txndc15	0
Txndc12	0
Txndc11	0
Txn2	0
Txn1	0
Txlng	0
Txk	0
Twsg1	0
Twistnb	0
Twist2	0
Twist1	0
Twf2	0
Tvp23b	0
Tvp23a	0
Tut1	0
Tusc5	0
Tusc2	0
Tusc1	0
Tulp4	0
Tulp3	0
Tulp1	0
Tuft1	0
Tufm	0
Tubgcp6	0
Tubgcp5	0
Tubgcp4	0
Tubgcp3	0
Tubgcp2	0
Tubg2	0
Tubg1	0
Olfr112	0,59
Scgb2b19	0,59
Nsf	0,59
Naa15	0,59
Alms1	0,59
Clec10a	0,59
Tigd2	0,59
Brs3	0,59
Lcn5	0,59
Cela1	0,59
Gm15140	0,59
Fgf7	0,59
Ankhd1	0,59
Slc17a7	0,59
Als2	0,59
Ms4a4b	0,59
Sirt1	0,59
Arl8a	0,59
Olfr714	0,59
Sco2	0,59
Aldh7a1	0,59
Skint3	0,59
Fut7	0,59
Reep4	0,59
Sprr2k	0,59
Glmn	0,59
Msh6	0,59
Spib	0,59
Lpcat1	0,59
Tsc1	0,59
Olfr452	0,59
Got1l1	0,59
Klk11	0,59
Map7d1	0,59
Snai2	0,59
Gpc6	0,59
BRDN0000737478	0,59
Cwf19l1	0,59
Car2	0,59
Dalrd3	0,59
Plekhg1	0,59
1700021K19Rik	0,59
Cldn1	0,59
Vmn2r66	0,59
Scgb3a1	0,59
Sh2d1a	0,59
E030030I06Rik	0,59
Ndufs7	0,59
Rttn	0,59
Stard3nl	0,59
BRDN0000738285	0,59
Lpo	0,59
Ednrb	0,58
Marveld2	0,58
Magoh	0,58
Ptgs2	0,58
Tsr2	0,58
Tdh	0,58
Ficd	0,58
Irf5	0,58
Os9	0,58
Pdia5	0,58
Dph6	0,58
Lce1c	0,58
Fam149b	0,58
Tmem115	0,58
Sfrp1	0,58
D1Ertd622e	0,58
Insig2	0,58
Prmt7	0,58
Smad2	0,58
1810030O07Rik	0,58
Prelid2	0,58
Vmn1r68	0,58
Cldn2	0,58
Phf7	0,58
Fgfr4	0,58
Zc3h12d	0,58
Serbp1	0,58
Olfr934	0,58
Npy1r	0,58
Vti1b	0,58
Olfr1477	0
Gm5108	0
Olfr1475	0
Aadac	0
Abtb2	0
Olfr1472	0
Olfr1471	0
BRDN0000737907	0
Nthl1	0
Sugct	0
Mnx1	0
Xlr	0
BRDN0000738103	0
Jrkl	0
Manba	0
Cdc27	0
Cdc26	0
Cdc23	0
Lin28b	0
Lin28a	0
Cdc20	0
Olfr772	0
Cln6	0
Cln5	0
Olfr771	0
Far1	0
Olfr777	0
Olfr774	0
Far2	0
BRDN0000738244	0
BRDN0000738245	0
Rtfdc1	0
BRDN0000738247	0
Mapre1	0
Nol10	0
Mapre3	0
BRDN0000738243	0
Morn5	0
Pou1f1	0
Olfr828	0
Olfr829	0
Morn1	0
Serpina1f	0
Morn2	0
Olfr822	0
Siglece	0
Siglecg	0
Olfr826	0
Olfr824	0
Olfr825	0
Ccdc64b	0
Sbk2	0
Bag1	0
Ttll11	0
Ttll10	0
Ttll13	0
Speg	0
Zbtb22	0
Prss16	0
Timm50	0
Zbtb21	0
Zbtb26	0
Kdm8	0
Zbtb24	0
Zbtb25	0
Mgat4e	0
Ucp3	0
Pnlip	0
BRDN0000737650	0
Adtrp	0
Psd2	0
Chodl	0
Otud5	0
Otud4	0
Eif1ax	0
Otud1	0
Adamts12	0
Eif1ad	0
Insl5	0
Sap30bp	0
Nhlrc4	0
Saxo1	0
Timp3	0,59
Trappc13	0,59
Srrm3	0,59
Tmem86a	0,59
Gm1604b	0,59
Olfr103	0,59
Tgoln1	0,59
Crabp2	0,59
Zfp282	0,59
Mapre2	0,59
Dock10	0,59
Tenm2	0,59
Hrsp12	0,59
Grip1	0,59
Mettl7a3	0,59
Ccdc85b	0,59
2610301B20Rik	0,59
Olfr920	0,59
Lepr	0,59
Slc2a8	0,59
Adipoq	0,59
Lamtor3	0,59
Runx3	0,59
Pdzd7	0,59
Obox1	0,59
Vmn1r91	0,59
Plin2	0,59
Syn1	0,59
Olfr601	0,59
Fabp4	0,59
Mettl21c	0,59
Piezo1	0,59
Dnah8	0,59
Tcap	0,59
Hes1	0,59
Elf5	0,59
Olfr1298	0,59
Olfr1348	0,59
Olfr799	0,59
Adcy2	0,59
Ddias	0,59
Rnf214	0,59
Rps3	0,59
Asb13	0,59
Pak3	0,59
Ptprb	0,59
Gm3417	0,59
Dnajc30	0,59
Ybey	0,59
Gfra1	0,59
Rnf146	0,59
Pms2	0,59
Vmn2r11	0,59
Acrbp	0,59
Qser1	0,59
Dgkh	0,59
1700034O15Rik	0,59
Rgs5	0,59
C1qtnf6	0,59
Olfr313	0,59
1810030O07Rik	0,59
Ago2	0,59
Clip2	0,59
Olfr309	0,59
BRDN0000737787	0,59
Myb	0,59
H2-T22	0,59
Olfr527	0,59
Olfr598	0,59
Gng13	0,59
Got1l1	0,59
Stoml1	0,59
Clic6	0,59
Olfr503	0,59
Hist1h4k	0,59
Rtn4	0,59
Agt	0,59
Gm14322	0,59
Thumpd3	0,59
Thbs3	0,59
Myoz3	0,59
Pml	0,59

Tube1	0
Tubd1	0
Tubb6	0
Tubb5	0
Tubb4b	0
Tubb2b	0
Tubb2a	0
Tubb1	0
Tubal3	0
Tuba8	0
Tuba3b	0
Tuba3a	0
Tuba1c	0
Tuba1b	0
Tuba1a	0
Ttyh2	0
Ttr	0
Ttpal	0
Ttpa	0
Ttll9	0
Ttll8	0
Ttll7	0
Ttll4	0
Ttll3	0
Ttll13	0
Ttll12	0
Ttll11	0
Ttll10	0
Ttll1	0
Ttl	0
Ttk	0
Tti2	0
Ttf2	0
Ttf1	0
Ttc9c	0
Ttc9b	0
Ttc9	0
Ttc8	0
Ttc7b	0
Ttc4	0
Ttc39d	0
Ttc39c	0
Ttc39a	0
Ttc38	0
Ttc37	0
Ttc36	0
Ttc34	0
Ttc33	0
Ttc32	0
Ttc30b	0
Ttc30a1	0
Ttc3	0
Ttc29	0
Ttc28	0
Ttc26	0
Ttc25	0
Ttc24	0
Ttc23l	0
Ttc23	0
Ttc22	0
Ttc19	0
Ttc17	0
Ttc16	0
Ttc14	0
Ttc12	0
Ttc1	0
Ttbk2	0
Ttbk1	0
Tstd2	0
Tsta3	0
Tst	0
Tssk6	0
Tssk5	0
Tssk4	0
Tssk3	0
Tssk2	0
Tssk1	0
Tssc1	0
Tsr2	0
Tsr1	0
Tspyl2	0
Tspo2	0
Ssh1	0,58
4930564B18Rik	0,58
Dcpp2	0,58
Rasgef1a	0,58
Abhd2	0,58
Cry2	0,58
Taar7a	0,58
Thoc1	0,58
Tsen2	0,58
Pate4	0,58
Olfr1061	0,58
4632428N05Rik	0,58
BRDN0000737447	0,58
Nppc	0,58
Spaca4	0,58
Npas2	0,58
1700013F07Rik	0,58
Trim29	0,58
Rpl27	0,58
Elf5	0,58
Stmn4	0,58
Tmem210	0,58
Akr1c19	0,58
Rfwd2	0,58
Ube4a	0,58
Hdac10	0,58
Strip2	0,58
Shoc2	0,58
Stt3b	0,58
Magea4	0,58
BRDN0000737621	0,58
BRDN0000737437	0,58
Col6a4	0,58
Mylk4	0,58
Ermn	0,58
Mfsd7c	0,58
Syt3	0,58
Timmdc1	0,58
2210010C04Rik	0,58
Cep44	0,58
Pcdha12	0,58
Glt8d2	0,58
Lcn6	0,58
Atp2b1	0,58
Ret	0,58
BRDN0000738069	0,58
Pou4f2	0,58
Zfp410	0,58
Foxg1	0,58
Hectd1	0,58
Rbm34	0,58
Lrrtm2	0,58
Tmem242	0,58
Arhgdib	0,58
Oas1g	0,58
Sycp2	0,58
Adgre1	0,58
Slc35f6	0,58
Baat	0,58
Mei4	0,58
Mpp3	0,58
Tmem9b	0,58
Teddm2	0,58
Stard4	0,57
Hsp90ab1	0,57
Mex3c	0,57
Cyp1a1	0,57
Slc41a3	0,57
Arsk	0,57
Plin4	0,57
Olfr992	0,57
BRDN0000737785	0,57
Kdelr2	0,57
Eci1	0,57
Ly6h	0,57
Fgfr1	0,57
Slc17a1	0,57
Sit1	0,57
Iyd	0,57
Arxes1	0,57
Slc25a43	0,57
Fbf1	0,57
Mgat4a	0
Lrrc15	0
Msr1	0
Amica1	0
1700001P01Rik	0
Mdn1	0
Cckbr	0
Fam76a	0
Insl6	0
Ccdc130	0
Mgat4c	0
Ccdc132	0
BRDN0000738318	0
BRDN0000738195	0
Mgat4b	0
Ccdc138	0
Gemin8	0
Edar	0
Gsap	0
Gm5592	0
Tut1	0
Cdon	0
E330021D16Rik	0
Insl3	0
Slc1a4	0
Ormdl2	0
Slc1a6	0
Siglec1	0
Slc1a2	0
Tspo2	0
Ptprz1	0
BRDN0000737625	0
Vps35	0
Vps36	0
Vps39	0
Id3	0
Mat2b	0
Fkbpl	0
Celf3	0
Tas2r140	0
Tas2r143	0
Unc79	0
Cml5	0
Adam26a	0
Adam26b	0
Vwa3a	0
Olfr447	0
Ryr2	0
1700034O15Rik	0
Gprin3	0
Gprin1	0
BRDN0000737795	0
Arid2	0
Gm20826	0
Pde4dip	0
Vegfc	0
Lysmd1	0
Gm21498	0
Anapc1	0
1700017D01Rik	0
Maea	0
Anapc2	0
Anapc5	0
Anapc4	0
Anapc7	0
C1qtnf9	0
1700016C15Rik	0
Adamts16	0
Rufy1	0
Fam188a	0
Rufy2	0
C1qtnf6	0
2700081O15Rik	0
C1qtnf5	0
Ror1	0
Zic5	0
Gm11563	0
Spg11	0
Gast	0
Eef2	0
Serpinb6d	0
Zfp113	0
Bhmt2	0,59
Cdk17	0,59
Lynx1	0,59
Mxd4	0,59
Rasl11b	0,59
Serpinb6c	0,59
Mctp1	0,59
Olah	0,58
Cybb	0,58
1700011E24Rik	0,58
Olfr59	0,58
Dffb	0,58
Lzts3	0,58
Cspp1	0,58
Ctdsp2	0,58
Trappc3l	0,58
Ccbl1	0,58
Csl	0,58
Ehd3	0,58
Pou2f2	0,58
Fzd1	0,58
Mcm4	0,58
Arl6ip5	0,58
Dock6	0,58
Gm5728	0,58
BRDN0000737565	0,58
Slc19a1	0,58
Arrdc4	0,58
BRDN0000737826	0,58
Setd1a	0,58
Fam212a	0,58
Olfr493	0,58
Ltk	0,58
Ptrf	0,58
Trim63	0,58
BRDN0000737921	0,58
Zfp385a	0,58
Esam	0,58
Zglp1	0,58
Dpp3	0,58
Slc35e2	0,58
Fgf7	0,58
Ndufa9	0,58
Acad11	0,58
Fam50b	0,58
Clnk	0,58
2810417H13Rik	0,58
Taar7d	0,58
Fam155a	0,58
Spon1	0,58
Hnrnpr	0,58
Thtpa	0,58
Rd3l	0,58
Pla2g3	0,58
Bcorl1	0,58
Hoxd3	0,58
Sbno2	0,58
Olfr199	0,58
Zfp664	0,58
Ccdc62	0,58
Slc30a7	0,58
Gm3701	0,58
Mov10	0,58
L3mbtl2	0,58
Akr1b10	0,58
Trappc10	0,58
5830411N06Rik	0,58
Slc1a1	0,58
Trpm8	0,58
Klhl6	0,58
Serpina9	0,58
Erich4	0,58
Cthrc1	0,58
4932411E22Rik	0,58
F2rl2	0,58
Jakmip2	0,58
Fcgr3	0,58
Tpst2	0,58
Rims4	0,58
Hpca	0,58
Olfr729	0,58
Colgalt2	0,58

Tspo	0
Tspan9	0
Tspan8	0
Tspan4	0
Tspan32	0
Tspan3	0
Tspan18	0
Tspan17	0
Tspan15	0
Tspan14	0
Tspan12	0
Tspan11	0
Tspan1	0
Tsnaxip1	0
Tsnax	0
Tsn	0
Tsku	0
Tsks	0
Tshz2	0
Tshz1	0
Tshr	0
Tshb	0
Tsga8	0
Tsga10	0
Tsg101	0
Tsfm	0
Tsen54	0
Tsen2	0
Tsen15	0
Tsc22d3	0
Tsc22d2	0
Tsc22d1	0
Tsc2	0
Tsc1	0
Tsacc	0
Try5	0
Try4	0
Try10	0
Trub2	0
Trub1	0
Trrap	0
Trpv6	0
Trpv5	0
Trpv4	0
Trpv3	0
Trpv1	0
Trps1	0
Trpm8	0
Trpm7	0
Trpm6	0
Trpm5	0
Trpm4	0
Trpm3	0
Trpm2	0
Trpm1	0
Trpd52l3	0
Trpc6	0
Trpc5	0
Trpc4ap	0
Trpc4	0
Trpc3	0
Trpc2	0
Trpa1	0
Trp53tg5	0
Trp53rk	0
Trp53inp2	0
Trp53i13	0
Trp53i11	0
Trp53bp2	0
Trp53bp1	0
Trove2	0
Troap	0
Tro	0
Trnt1	0
Trnp1	0
Trnau1ap	0
Trmu	0
Trmt5	0
Trmt44	0
Trmt2b	0
Trmt2a	0
Trmt1l	0
Zrsr2	0,57
Lrrc16a	0,57
Trappc2l	0,57
C1qtnf9	0,57
Dusp5	0,57
Asprv1	0,57
Mlph	0,57
Adgrl2	0,57
BC089491	0,57
Tmed9	0,57
Ap5b1	0,57
Ugt1a2	0,57
Stk32b	0,57
Olfr828	0,57
Olfr1020	0,57
Ctgf	0,57
Prr15	0,57
Acoxl	0,57
Olfr59	0,57
Zfp397	0,57
Myh3	0,57
Krt25	0,57
Raet1e	0,57
D230025D16Rik	0,57
Trim12c	0,57
Olfr65	0,57
Ccdc42	0,57
Opn5	0,57
Akap5	0,57
Kcnj12	0,57
Ppp1r3g	0,57
Srsf12	0,57
G0s2	0,57
Ctsq	0,57
Slc12a4	0,57
Gm12185	0,57
Emc1	0,57
Ydjc	0,57
Vac14	0,57
Hsdl2	0,57
Cacna2d2	0,57
Lix1	0,57
Uncx	0,57
Fxr1	0,57
Vmn1r59	0,57
Gm614	0,57
Cstf3	0,57
Epdr1	0,57
Celf4	0,57
Slc35e2	0,57
Sptb	0,57
Uty	0,57
Accsl	0,57
Cdc25a	0,57
Hrg	0,57
1700001J03Rik	0,57
Gpr65	0,57
Snx21	0,57
Lin52	0,57
Trim72	0,57
Gm4307	0,57
Mtcl1	0,57
Megf6	0,57
Pcgf6	0,57
Slc34a2	0,57
Ulk1	0,57
Pdlim3	0,57
Lyz2	0,57
Apba1	0,57
Epsti1	0,57
BRDN0000738295	0,57
Pinlyp	0,57
Eps8l1	0,57
Tmtc2	0,57
Fthl17	0,57
E130012A19Rik	0,57
Dync2h1	0,57
Ptchd2	0,57
Gys2	0,57
Sult4a1	0,56
Gsdmd	0,56
Cdh13	0,56
Adamts19	0
Slc2a9	0
Slc2a7	0
Slc2a6	0
Slc2a5	0
Slc2a4	0
Slc2a3	0
Slc2a2	0
Slc2a1	0
Rarres1	0
Rab17	0
2810474O19Rik	0
Ccnl2	0
Daxx	0
Grpr	0
Nusap1	0
Gm13276	0
Map7	0
Gm13271	0
Fv1	0
Olfr478	0
BRDN0000737890	0
BRDN0000737891	0
BRDN0000737892	0
Plcb3	0
Plcb4	0
Padi1	0
BRDN0000737792	0
Hamp2	0
Supt5	0
Amy1	0
Hras	0
Spdef	0
Aak1	0
Fam189a2	0
Scrib	0
Gas8	0
Hist1h2ah	0
Sgsm3	0
Sgsm2	0
Sgsm1	0
Acot9	0
Slc15a2	0
Slc15a3	0
4933427D14Rik	0
Ctxn3	0
Gas7	0
Gas6	0
Olfr476	0
Fez2	0
Lamp5	0
Phf11a	0
Pld6	0
Pyurf	0
Gtf2e2	0
Olfr470	0
Dmrtb1	0
Eif4e2	0
Ddo	0
Ddn	0
Zfp398	0
2610301B20Rik	0
Ddc	0
Zfp397	0
Zfp395	0
Chmp1a	0
Foxc1	0
Mrpl36	0
Adgrg7	0
Mrpl34	0
Mrpl35	0
Mrpl32	0
Mrpl33	0
Gm4141	0
Adgrg6	0
Rhox3c	0
Gabrq	0
Gabrp	0
Mrpl38	0
Nek11	0
Cplx2	0
Ppil1	0
Atn1	0,58
F3	0,58
Olfr790	0,58
Trpc1	0,58
Mtx2	0,58
Bcl2l2	0,58
9430038I01Rik	0,58
Usp18	0,58
B4galnt1	0,58
Retn	0,58
Grid2ip	0,58
Celf3	0,58
Tex12	0,58
Ccng1	0,58
Galnt1	0,58
Il1f6	0,58
Akip1	0,57
Rnaseh2c	0,57
Epgn	0,57
Tcf24	0,57
Ccdc120	0,57
Ppp1r32	0,57
Olfr1311	0,57
Gypa	0,57
Tbc1d17	0,57
Serp2	0,57
Pitpnc1	0,57
Pigl	0,57
Slc28a1	0,57
Fkbp2	0,57
Mettl7a1	0,57
Chid1	0,57
Foxc2	0,57
Rtn4rl2	0,57
Col14a1	0,57
Trf	0,57
Olfr171	0,57
Birc2	0,57
Clec16a	0,57
Zbtb4	0,57
Fuk	0,57
Exoc5	0,57
Pirb	0,57
BRDN0000737789	0,57
Lonrf3	0,57
Myl12a	0,57
Stam2	0,57
Mfsd2b	0,57
St3gal6	0,57
Vmn1r197	0,57
Olfr1489	0,57
Myl3	0,57
Aif1l	0,57
Cript	0,57
Eppin	0,57
Umps	0,57
Plvap	0,57
Stx1a	0,57
Ogn	0,57
Rad51	0,57
Pnp	0,57
Timp2	0,57
Gramd1a	0,57
Sfmbt1	0,57
Fgd5	0,57
Cc2d1b	0,57
Gm6537	0,57
Rbp7	0,57
Slc12a8	0,57
Hnf1b	0,57
Vmn1r30	0,57
Igsf11	0,57
Adcy8	0,57
Lce6a	0,57
Pdia4	0,57
Nr2f1	0,57
Usp6nl	0,57
Dbi	0,57
Gm17727	0,57
Olfr538	0,57
Gm5108	0,57
Gpr156	0,57

Trmt13	0	Gabrb3	0,56	Slc31a2	0	Ugt3a1	0,57
Trmt12	0	Htr1f	0,56	Oas2	0	Heatr5b	0,57
Trmt112	0	Ogdhl	0,56	Ppil4	0	Lclat1	0,57
Trmt11	0	Olfr888	0,56	Asap2	0	Cys1	0,57
Trmt10c	0	Psmb8	0,56	Ppil6	0	Emilin1	0,57
Trmt10b	0	Alg2	0,56	Khdc1a	0	Scamp2	0,57
Trmt10a	0	Cd207	0,56	Gabre	0	Tpgs1	0,57
Trmt1	0	Zbtb41	0,56	Dmrt1	0	B3gat3	0,57
Trit1	0	St7l	0,56	Camk2n1	0	Eomes	0,57
Triqk	0	Mtfr1l	0,56	BRDN0000738200	0	Eif2s3y	0,57
Trip4	0	Ptbp2	0,56	Cenpb	0	Shroom4	0,57
Trip12	0	1700106J16Rik	0,56	Fam92b	0	Iah1	0,57
Trip10	0	Olfr1154	0,56	Nup88	0	Ly6f	0,57
Triobp	0	Sec31a	0,56	Coro2a	0	Ankrd44	0,57
Trio	0	Ccdc114	0,56	Gm5635	0	Cops3	0,57
Triml2	0	Bcl9	0,56	E030018B13Rik	0	Hsd3b3	0,57
Triml1	0	Atp5g2	0,56	Clasp2	0	BRDN0000737903	0,57
Trim9	0	Gfm2	0,56	Afap1l2	0	Prkcdbp	0,57
Trim80	0	Tnfaip8	0,56	Mtm1	0	Slc4a3	0,57
Trim8	0	Klra5	0,56	Nacc1	0	Pydc3	0,57
Trim75	0	Olfr570	0,56	Olfr5	0	Sgk3	0,57
Trim72	0	Dos	0,56	Nup85	0	Apol9b	0,57
Trim71	0	Braf	0,56	Olfr957	0	Mpc2	0,57
Trim68	0	Gm13285	0,56	A630073D07Rik	0	BRDN0000737849	0,56
Trim67	0	Zfp623	0,56	Arhgef40	0	Tomm6	0,56
Trim66	0	Mc3r	0,56	Ap3m2	0	Ppap2a	0,56
Trim63	0	G6pdx	0,56	Mrgpra3	0	Olfr453	0,56
Trim61	0	Exoc6b	0,56	Nova2	0	Zbtb17	0,56
Trim60	0	Gm10639	0,56	Tmem120a	0	Miox	0,56
Trim6	0	Btnl6	0,56	Dcpp1	0	Zfp719	0,56
Trim59	0	N4bp2l1	0,56	Hmgn5	0	Olfr632	0,56
Trim58	0	Sept12	0,56	Cmip	0	Vmn2r51	0,56
Trim56	0	Isca2	0,56	Hmgn3	0	Usp22	0,56
Trim55	0	Tinf2	0,56	Olfr218	0	BRDN0000737534	0,56
Trim54	0	Npw	0,56	AI429214	0	Camk1d	0,56
Trim52	0	Onecut3	0,56	Olfr215	0	Tbc1d12	0,56
Trim50	0	Olfr456	0,56	Olfr214	0	Letm1	0,56
Trim47	0	Rab24	0,56	Gm17455	0	Ndufaf2	0,56
Trim46	0	G3bp1	0,56	Olfr211	0	Cecr2	0,56
Trim45	0	Vars2	0,56	Olfr213	0	Fcho2	0,56
Trim44	0	Clmp	0,56	Olfr212	0	Hspb9	0,56
Trim43c	0	Cdk8	0,56	Nfs1	0	Xpnpep2	0,56
Trim43b	0	Lbx2	0,56	Glyat	0	Gm6034	0,56
Trim43a	0	Fam188a	0,56	Serpinb6b	0	Ankef1	0,56
Trim42	0	Olfr1465	0,56	Stc1	0	BRDN0000738104	0,56
Trim41	0	Zscan21	0,56	Stc2	0	Tbl2	0,56
Trim40	0	Slc13a3	0,56	Cdkn2b	0	Foxe3	0,56
Trim39	0	Lpar1	0,56	Srsf4	0	Ptprs	0,56
Trim37	0	Aqp5	0,56	Dstyk	0	Olfr112	0,56
Trim36	0	Abhd13	0,56	Ccnd3	0	Phf6	0,56
Trim35	0	Prss21	0,56	BRDN0000737459	0	Kif26b	0,56
Trim34a	0	Dlg3	0,56	Srsf7	0	Slc44a3	0,56
Trim33	0	Tmem260	0,56	Specc1	0	Krt8	0,56
Trim32	0	Myof	0,56	Cep55	0	Mmaa	0,56
Trim31	0	Tmem178	0,56	Gm20767	0	Mamld1	0,56
Trim30d	0	Dbndd2	0,56	1190002N15Rik	0	Prop1	0,56
Trim30a	0	C9	0,56	4933433C11Rik	0	Impg2	0,56
Trim3	0	Cdk18	0,56	1700018F24Rik	0	Pdss2	0,56
Trim29	0	Usp17lc	0,56	BRDN0000738304	0	Slc35b4	0,56
Trim28	0	Ptges3	0,56	Cd74	0	Zfp14	0,56
Trim27	0	Il23a	0,56	Barx2	0	Tbc1d10c	0,56
Trim25	0	Zfp637	0,56	Gstm5	0	Vmn2r108	0,56
Trim24	0	Sumo3	0,56	4930504O13Rik	0	Tnks2	0,56
Trim21	0	Sdhaf1	0,56	BRDN0000738307	0	Wdr7	0,56
Trim2	0	Olfr368	0,56	Hist1h2af	0	Suco	0,56
Trim17	0	Sgms2	0,56	Hspb9	0	Pcyt1b	0,56
Trim16	0	Pcdhga4	0,56	Dcxr	0	Sgsm1	0,56
Trim15	0	Sec14l5	0,56	Epyc	0	Uck1	0,56
Trim14	0	Vsx1	0,56	Brcc3	0	Vmn2r1	0,56
Trim13	0	Triml1	0,56	Iqca	0	Capza3	0,56
Trim12c	0	Mpc1	0,56	1700011H14Rik	0	Elavl4	0,56
Trim12a	0	Defb10	0,56	Barx1	0	Dnali1	0,56
Trim10	0	Erlin1	0,56	BRDN0000738308	0	Aqp7	0,56
Tril	0	Ctnna3	0,56	Moxd1	0	Plcb1	0,56
Trib3	0	Il23r	0,56	Iqcg	0	BRDN0000737955	0,56
Trib2	0	Ppp1r14a	0,56	Nudt21	0	Olfr1416	0,56
Triap1	0	Atg2b	0,56	Moxd2	0	Olfr63	0,56
Trhr2	0	Spesp1	0,56	Elp6	0	Olfr684	0,56
Trhr	0	Olfr1196	0,56	Gem	0	Gm20854	0,56
Trhde	0	Pitx3	0,56	Cyp2g1	0	Sult4a1	0,56
Trh	0	Cyp2a4	0,56	Hps3	0	Itgbl1	0,56
Trf	0	Reep6	0,56	Fam162b	0	Galnt4	0,56

Trex2	0	Fbl	0,56	8030423J24Rik	0	Spaca6	0,56
Treml4	0	Chchd1	0,56	Tex15	0	Calcr	0,56
Treml1	0	Thsd1	0,56	Tex14	0	Immp1l	0,56
Trem2	0	Zfp110	0,56	Cetn4	0	Ednra	0,56
Trdn	0	Lmtk3	0,56	Tex11	0	Rnd3	0,56
Trdmt1	0	Mfsd3	0,56	Cetn2	0	4430402I18Rik	0,56
Trappc9	0	Dck	0,56	Cetn1	0	Ccdc69	0,56
Trappc8	0	Slc25a27	0,56	Tex12	0	Fam76a	0,56
Trappc6b	0	Olfr693	0,56	Wibg	0	BRDN0000737512	0,56
Trappc6a	0	Nqo2	0,56	Olfr1328	0	Runx2	0,56
Trappc4	0	Klhl24	0,56	BRDN0000737388	0	Rhox11	0,56
Trappc3l	0	Tmem238	0,55	P2ry1	0	Eif2s2	0,56
Trappc3	0	Adat1	0,55	BRDN0000737384	0	Cd86	0,56
Trappc2l	0	Obsl1	0,55	BRDN0000737385	0	Ccdc116	0,56
Trappc2	0	Tff1	0,55	BRDN0000737386	0	Sntn	0,56
Trappc13	0	Rab11fip5	0,55	BRDN0000737387	0	Ttc19	0,56
Trappc12	0	Hsd17b1	0,55	BRDN0000737380	0	Robo3	0,56
Trappc10	0	Id4	0,55	BRDN0000737381	0	Trim62	0,56
Trappc1	0	Naif1	0,55	BRDN0000737382	0	Hsd17b14	0,56
Trap1a	0	Tgfbr2	0,55	Nop58	0	Ifi47	0,56
Trank1	0	Arsa	0,55	Pex3	0	Tbx3	0,56
Tram2	0	Gabra1	0,55	Vmn1r55	0	Zfp839	0,56
Tram1	0	Ang	0,55	Pex5	0	S100a13	0,56
Trak2	0	Fam151b	0,55	Vmn1r53	0	Osm	0,56
Trak1	0	Zfp609	0,55	Vmn1r50	0	Fbxo36	0,56
Traip	0	Sgta	0,55	Pex6	0	Htr4	0,56
Trafd1	0	Nol4l	0,55	Gm7168	0	Fam222a	0,56
Traf7	0	Slc5a1	0,55	Gsdma	0	Olfr1448	0,56
Traf3ip2	0	Chrna4	0,55	Reg4	0	Olfr810	0,56
Traf3ip1	0	Best3	0,55	Gsdmc	0	Psmg3	0,56
Traf3	0	9230104L09Rik	0,55	Reg2	0	Tatdn2	0,56
Traf2	0	Upk3a	0,55	Vmn1r58	0	Fubp1	0,56
Traf1	0	Kcns2	0,55	Vmn1r59	0	Cd300lf	0,56
Trabd2b	0	Prdm16	0,55	3110007F17Rik	0	Rspo4	0,56
Trabd	0	2310057N15Rik	0,55	Ubl4b	0	Gale	0,56
Tra2b	0	Zswim6	0,55	Sh2d2a	0	Tmem176a	0,56
Tra2a	0	Cnfn	0,55	Plb1	0	Clk2	0,56
Tpx2	0	Acvr1c	0,55	Rp1l1	0	Ctsr	0,56
Tpte	0	Ets1	0,55	Itga1	0	Nt5dc2	0,56
Tpt1	0	Cep85l	0,55	Mrpl27	0	Tmed6	0,56
Tpst2	0	Pabpc2	0,55	Cd320	0	Triml1	0,56
Tpst1	0	Pard6b	0,55	Acsf2	0	Zfp418	0,56
Tpsb2	0	March1	0,55	Dtnbp1	0	Whsc1l1	0,56
Tprn	0	Slc30a4	0,55	Hadh	0	Gjd4	0,56
Tprkb	0	Pcdhgc5	0,55	Nop56	0	Cblb	0,55
Tprgl	0	Tmem29	0,55	Ctps2	0	Cln5	0,55
Tprg	0	Ido2	0,55	Rpsa	0	Kpna1	0,55
Tpra1	0	Cd24a	0,55	Chrac1	0	Mmp10	0,55
Tpr	0	Zim1	0,55	D630023F18Rik	0	Itpripl1	0,55
Tppp3	0	Dsc2	0,55	BRDN0000737923	0	Capn8	0,55
Tppp2	0	1700026D08Rik	0,55	Itga5	0	Kctd11	0,55
Tppp	0	Spata1	0,55	Slc5a12	0	4930558K02Rik	0,55
Tpp1	0	Taar3	0,55	Arrdc5	0	Pdpn	0,55
Tpo	0	Lrrd1	0,55	Slc5a10	0	Aven	0,55
Tpm3	0	2610301B20Rik	0,55	Arrdc1	0	Gp6	0,55
Tpm1	0	Cbl	0,55	Arrdc3	0	Igflr1	0,55
Tpk1	0	Ubr5	0,55	BRDN0000737560	0	Ercc1	0,55
Tph2	0	Nwd2	0,55	BRDN0000737561	0	Slc26a3	0,55
Tph1	0	Hectd3	0,55	BRDN0000737562	0	Naa25	0,55
Tpgs2	0	Kcnip3	0,55	Itga7	0	Tpbg	0,55
Tpgs1	0	Tjp2	0,55	Plag1	0	Ablim3	0,55
Tpd52l2	0	Mcts2	0,55	BRDN0000737565	0	BRDN0000737824	0,55
Tpd52l1	0	Phex	0,55	BRDN0000737566	0	Drd4	0,55
Tpcn2	0	Htr2c	0,55	D10Jhu81e	0	Il4i1	0,55
Tpcn1	0	Exoc3l	0,55	Gm10024	0	Rasl10a	0,55
Tpbpb	0	Icosl	0,55	Yod1	0	Cyp2d9	0,55
Tpbpa	0	Csnk2a2	0,55	Zfp112	0	Olfr1454	0,55
Tpbg	0	Fat1	0,55	Pck1	0	Col7a1	0,55
Tox3	0	Grhl1	0,55	Kcnv1	0	BRDN0000738224	0,55
Tor4a	0	Myl12a	0,55	Stra8	0	Frem2	0,55
Tor3a	0	BRDN0000737763	0,55	P2ry14	0	Tigar	0,55
Tor2a	0	E130309D02Rik	0,55	Stra6	0	Rabgef1	0,55
Tor1b	0	Gpr146	0,55	P2ry10	0	Suclg2	0,55
Tor1aip1	0	Npr2	0,55	Rbm7	0	Olfr1008	0,55
Tor1a	0	Slc5a5	0,55	P2ry12	0	Ndufa10	0,55
Toporsl	0	Mfsd5	0,55	P2ry13	0	Uts2	0,55
Topors	0	Fam122b	0,55	Sord	0	Atpaf1	0,55
Topbp1	0	Hnrnpa2b1	0,55	Rhbdd2	0	Extl3	0,55
Topaz1	0	Adra2c	0,55	Rhbdd3	0	Adam22	0,55
Top3b	0	Slc10a1	0,55	4732456N10Rik	0	Anks3	0,55
Top3a	0	Bop1	0,55	Rsbn1	0	Umod	0,55
Top2b	0	Cdk5r1	0,55	Cpn2	0	Pigu	0,55

Top2a	0	Hist1h2ap	0,55	Cpn1	0	Hdgfl1	0,55
Top1	0	Lrrc57	0,55	Dnph1	0	Zcchc3	0,55
Tonsl	0	Ceacam14	0,55	Sarnp	0	Gm4858	0,55
Tomm70a	0	Slc12a8	0,55	Tgfbr3	0	Plcd3	0,55
Tomm7	0	Zswim2	0,55	Slc38a5	0	Dll4	0,55
Tomm6	0	Amh	0,55	Tgfbr1	0	BRDN0000737760	0,55
Tomm5	0	4930557A04Rik	0,55	Phka2	0	2900011O08Rik	0,55
Tomm34	0	Fh1	0,55	Fkbp2	0	Gm13051	0,55
Tomm20l	0	H2-M10.5	0,55	Sell	0	Gm4836	0,55
Tomm20	0	Gpx2	0,55	Slc38a4	0	Olfr1057	0,55
Tom1l2	0	Defb34	0,55	Ctf2	0	Rnls	0,55
Tom1	0	Adgb	0,55	Pde10a	0	Tmed9	0,55
Tollip	0	Mpp4	0,55	Slc38a7	0	Adam34	0,55
Toe1	0	1700018B08Rik	0,55	Thbs3	0	H2-Q10	0,55
Tob2	0	Rnf113a1	0,55	Rps7	0	Wfdc5	0,55
Tns4	0	Tas2r120	0,55	Rps6	0	Olfr1471	0,55
Tns3	0	Olfr498	0,55	Rps5	0	Cblc	0,55
Tns2	0	Olfr1182	0,55	Slc34a2	0	Crkl	0,55
Tns1	0	Lrp11	0,55	Cyp2c37	0	Acad9	0,55
Tnrc6c	0	Gm6086	0,55	Rps2	0	Arpc4	0,55
Tnrc6a	0	Slc44a5	0,55	Tet1	0	Gpr87	0,55
Tnrc18	0	Sh2d4a	0,55	Gm14851	0	BRDN0000738261	0,55
Tnpo3	0	Lhx6	0,55	Alg11	0	Blvrb	0,55
Tnpo1	0	Lig4	0,55	Cyp2c39	0	Pcdh20	0,55
Tnp2	0	Gm906	0,55	Cyp2c38	0	Gm4952	0,55
Tnp1	0	Gm20826	0,55	Golga7	0	Olfml1	0,55
Tnnt2	0	Nudt10	0,55	Rps9	0	Patl1	0,55
Tnni3k	0	Thumpd1	0,55	Nck1	0	Arl8a	0,55
Tnni3	0	Gramd3	0,55	Drg2	0	Ccr1	0,55
Tnni2	0	BRDN0000737834	0,55	Drg1	0	Rabac1	0,55
Tnni1	0	Spr	0,54	Sdf4	0	1110025L11Rik	0,55
Tnnc1	0	Klrg2	0,54	Selm	0	Uqcc3	0,55
Tnn	0	Neurog3	0,54	Sdf2	0	Gcnt7	0,55
Tnmd	0	Tagap	0,54	Olfr1048	0	Cdadc1	0,55
Tnks1bp1	0	Armcx4	0,54	Olfr1049	0	Gdf15	0,55
Tnks	0	Tbxa2r	0,54	Olfr1046	0	Tmem203	0,55
Tnk1	0	BC089597	0,54	Olfr1047	0	Slc2a12	0,55
Tnip3	0	Cyb5r1	0,54	Olfr1044	0	Olfr502	0,55
Tnip2	0	Mrgprf	0,54	Olfr1042	0	Klk1b3	0,55
Tnip1	0	Ccl6	0,54	Olfr1043	0	Cd276	0,55
Tnik	0	Gm21693	0,54	Evc2	0	Ager	0,55
Tnfsf8	0	Sugp2	0,54	Kcnip2	0	Ralgds	0,54
Tnfsf4	0	Gucy2g	0,54	Zfp943	0	Amica1	0,54
Tnfsf18	0	Spt1	0,54	Hcar2	0	Csnk1e	0,54
Tnfsf15	0	Cmtm3	0,54	Zfp941	0	Gabpb1	0,54
Tnfsf14	0	Mdm4	0,54	Zfp940	0	Ercc3	0,54
Tnfsf13	0	G3bp2	0,54	Zfp947	0	Olfr1313	0,54
Tnfsf12	0	Tor1b	0,54	Zfp946	0	Slc2a1	0,54
Tnfsf11	0	Apold1	0,54	Zfp945	0	Trp53inp2	0,54
Tnfsf10	0	Prm2	0,54	Zfp944	0	1700025F22Rik	0,54
Tnfrsf9	0	Pcsk1n	0,54	Zfp949	0	Ndufb6	0,54
Tnfrsf8	0	Cdc42ep5	0,54	Zfp948	0	Pdk2	0,54
Tnfrsf4	0	Bbs5	0,54	Hcn3	0	Iqub	0,54
Tnfrsf26	0	Xndc1	0,54	Capzb	0	Mettl22	0,54
Tnfrsf25	0	Olfr803	0,54	Lrrc7	0	Ssh3	0,54
Tnfrsf23	0	Ufc1	0,54	Zfp365	0	Rgs2	0,54
Tnfrsf22	0	Tcl1b3	0,54	BRDN0000737464	0	Ddx41	0,54
Tnfrsf1b	0	Stx2	0,54	Fam131c	0	Apbb3	0,54
Tnfrsf1a	0	L3hypdh	0,54	Cenpw	0	Fam213b	0,54
Tnfrsf19	0	Csrp1	0,54	Slc38a8	0	Ppfia1	0,54
Tnfrsf18	0	Il18bp	0,54	Nfkbid	0	Clca4a	0,54
Tnfrsf17	0	Caps2	0,54	BRDN0000737451	0	Vmn1r148	0,54
Tnfrsf14	0	Slmo1	0,54	Ch25h	0	Mccc2	0,54
Tnfrsf13c	0	Nup85	0,54	4930447A16Rik	0	Tpm1	0,54
Tnfrsf13b	0	Olfr684	0,54	Gm8765	0	Ceacam2	0,54
Tnfrsf11b	0	Ccdc109b	0,54	Rhbdl1	0	2410131K14Rik	0,54
Tnfrsf11a	0	BC051142	0,54	Vmn2r40	0	Krtap12-1	0,54
Tnfrsf10b	0	Naip6	0,54	Vmn2r41	0	Srsf2	0,54
Tnfaip8l3	0	Gpr135	0,54	Vmn2r42	0	Xirp2	0,54
Tnfaip8l2	0	Necab2	0,54	Vmn2r44	0	Ripply3	0,54
Tnfaip8l1	0	Ccnb2	0,54	Vmn2r45	0	Esco2	0,54
Tnfaip8	0	Crybb3	0,54	Vmn2r46	0	Cmtm1	0,54
Tnfaip6	0	Rpp14	0,54	Vmn2r47	0	Rpl37a	0,54
Tnfaip3	0	BRDN0000737758	0,54	Vmn2r48	0	Gpbar1	0,54
Tnfaip2	0	Hist1h3a	0,54	Vmn2r49	0	Tcf21	0,54
Tnfaip1	0	Acap2	0,54	Mapk1ip1	0	Msx2	0,54
Tmx4	0	4922502D21Rik	0,54	Arl8a	0	Epc1	0,54
Tmx2	0	Commd8	0,54	Grb14	0	Gm732	0,54
Tmub2	0	Hoxb4	0,54	Cacnb1	0	Hist1h2ag	0,54
Tmub1	0	Hist2h3c2	0,54	Hsp90ab1	0	Ccl27b	0,54
Tmtc4	0	Olfr310	0,54	3110021N24Rik	0	Olfr975	0,54
Tmtc3	0	Ccdc149	0,54	Megf8	0	Pgrmc1	0,54

Tmtc2	0	Hs3st2	0,54	0610030E20Rik	0	Taar8b	0,54
Tmtc1	0	Eqtn	0,54	Pcdhb22	0	Agtr1b	0,54
Tmsb4x	0	Ccdc63	0,54	Arhgdib	0	Dcaf8	0,54
Tmsb15b2	0	Sptlc1	0,54	Pcdhb20	0	Neu1	0,54
Tmsb15b1	0	Fndc9	0,54	Pcdhb21	0	Smyd3	0,54
Tmsb15a	0	Akr1cl	0,54	Megf6	0	Dr1	0,54
Tmsb10	0	Fundc1	0,54	Slc22a18	0	Fcrlb	0,54
Tmprss9	0	Map2k5	0,54	Gpr15	0	Zfp787	0,54
Tmprss7	0	Brpf1	0,54	Gsto1	0	Fancd2	0,54
Tmprss6	0	Zfp426	0,54	Cenpu	0	Fads3	0,54
Tmprss5	0	Sephs2	0,54	Gpr12	0	Lcor	0,54
Tmprss2	0	Cdca2	0,54	Pabpc1l	0	C8b	0,54
Tmprss15	0	Pdcd7	0,54	Yif1a	0	Rgcc	0,54
Tmprss11g	0	Zfp326	0,54	BRDN0000738102	0	Akr1b8	0,54
Tmprss11e	0	Map1a	0,54	Gpr19	0	Pex5l	0,54
Tmprss11d	0	Map4k4	0,54	Gpr18	0	Dact3	0,54
Tmprss11b	0	2010107G12Rik	0,54	Tram1	0	Hsd17b2	0,54
Tmprss11a	0	Rdh9	0,54	Ppbp	0	Comp	0,54
Tmppe	0	Slc27a1	0,54	Tmem38a	0	Phldb3	0,54
Tmpo	0	Slfnl1	0,54	Gm12253	0	Prpf40b	0,54
Tmod4	0	Vmn1r58	0,54	Sec11a	0	Cpped1	0,54
Tmod2	0	Krtap8-1	0,54	Svs1	0	Guca2b	0,54
Tmod1	0	Trp53i13	0,54	Srgap2	0	Ramp3	0,54
Tmigd1	0	H2-Ke6	0,53	Cav2	0	Sept1	0,54
Tmie	0	Tas1r1	0,53	Tor2a	0	Calhm2	0,54
Tmf1	0	Wasf1	0,53	Pbk	0	Mpzl2	0,54
Tmem98	0	Styxl1	0,53	Nkg7	0	Sorl1	0,54
Tmem97	0	Fam64a	0,53	Rad23b	0	Rs1	0,54
Tmem95	0	Cbr2	0,53	Shcbp1	0	Rerg	0,54
Tmem91	0	Suv420h1	0,53	Rad23a	0	Gm2016	0,54
Tmem8c	0	Lrrc17	0,53	Tac2	0	Gsto2	0,54
Tmem88b	0	Hmgcs2	0,53	Tac4	0	4921530L21Rik	0,54
Tmem88	0	Map3k6	0,53	Olfr1054	0	Wnt3a	0,54
Tmem87b	0	Snrnp27	0,53	Rhobtb2	0	Abhd14b	0,54
Tmem87a	0	Accs	0,53	Rhobtb3	0	Qtrtd1	0,54
Tmem86b	0	Pla2g4d	0,53	Apobr	0	Psat1	0,54
Tmem86a	0	Mmachc	0,53	P2rx3	0	Azin1	0,54
Tmem82	0	Kif27	0,53	1700042B14Rik	0	Vmn1r219	0,53
Tmem81	0	Pcdhga11	0,53	Ido2	0	Bcas1	0,53
Tmem80	0	Vamp5	0,53	Tbck	0	Car13	0,53
Tmem8	0	Olfr777	0,53	Degs1	0	Rps16	0,53
Tmem79	0	Nr1h3	0,53	Mmrn2	0	Adh4	0,53
Tmem74b	0	1700113H08Rik	0,53	Mmrn1	0	Ankrd55	0,53
Tmem74	0	Arhgdia	0,53	Tbcd	0	Myo7a	0,53
Tmem72	0	Cnp	0,53	Tbcc	0	Pld2	0,53
Tmem71	0	Olfr867	0,53	Tbcb	0	Mvb12a	0,53
Tmem68	0	Josd1	0,53	Tbca	0	9130019O22Rik	0,53
Tmem65	0	Tmem170	0,53	Rltpr	0	Gm608	0,53
Tmem63c	0	Tmem71	0,53	Trim9	0	Mup4	0,53
Tmem63a	0	Cyp24a1	0,53	Trim8	0	Acaa2	0,53
Tmem62	0	Gm10104	0,53	Sugt1	0	Basp1	0,53
Tmem60	0	Amt	0,53	Tmem44	0	Krtap11-1	0,53
Tmem59l	0	Cd86	0,53	Trim3	0	BRDN0000737522	0,53
Tmem59	0	Nup153	0,53	Usp51	0	Dbndd1	0,53
Tmem57	0	Ugt1a7c	0,53	Stxbp5l	0	Gdpgp1	0,53
Tmem56	0	Bhlhb9	0,53	Tmem43	0	Zfp945	0,53
Tmem55a	0	Tshr	0,53	Trim6	0	Trappc11	0,53
Tmem54	0	Tcl1b1	0,53	Ddx43	0	4931406B18Rik	0,53
Tmem53	0	Noto	0,53	Mn1	0	Gm7361	0,53
Tmem52b	0	Pitpnm1	0,53	Rgn	0	BRDN0000738045	0,53
Tmem52	0	Celf2	0,53	BRDN0000737456	0	Zbtb45	0,53
Tmem51	0	Vmn1r170	0,53	Tmem38b	0	Extl2	0,53
Tmem50a	0	Tiprl	0,53	Col5a2	0	Fcho1	0,53
Tmem5	0	Por	0,53	Ttc14	0	Papln	0,53
Tmem47	0	Zfp12	0,53	Ttc17	0	Scyl3	0,53
Tmem45a	0	Olfr740	0,53	Ttc16	0	Mex3c	0,53
Tmem44	0	Fam109b	0,53	Ttc12	0	Ceacam1	0,53
Tmem43	0	Chrna7	0,53	Lmbrd2	0	Dennd1b	0,53
Tmem41b	0	Chd8	0,53	Lmbrd1	0	Slc20a1	0,53
Tmem40	0	St6galnac6	0,53	BRDN0000737842	0	Sult2a7	0,53
Tmem39a	0	Ccdc105	0,53	Rab40c	0	Zcchc24	0,53
Tmem38b	0	Mfap4	0,53	Rab40b	0	Upk3a	0,53
Tmem38a	0	Fam199x	0,53	BRDN0000737719	0	Tysnd1	0,53
Tmem37	0	Gpr84	0,53	Idh3a	0	Clstn2	0,53
Tmem35	0	Plxnc1	0,53	Olfr888	0	Nlrp4c	0,53
Tmem33	0	BRDN0000737386	0,53	Idh3b	0	Ackr3	0,53
Tmem30b	0	Nkx1-1	0,53	Glra2	0	Lyrm2	0,53
Tmem30a	0	Ino80d	0,53	Idh3g	0	Slc24a4	0,53
Tmem27	0	Phkg2	0,53	Gm156	0	Rap1gap	0,53
Tmem263	0	Olfr148	0,53	BRDN0000737457	0	Iqsec2	0,53
Tmem261	0	Olfr466	0,53	Clec4a4	0	Cacul1	0,53
Tmem259	0	Cox6a1	0,53	Clec4a3	0	Arl4c	0,53

Tmem258	0	Adam26b	0,53	Clec4a2	0	Cxx1a	0,53
Tmem256	0	Slc35d2	0,53	Clec4a1	0	Ccdc74a	0,53
Tmem255b	0	Dedd	0,53	Krt83	0	Olfr1323	0,53
Tmem255a	0	Plekho2	0,53	BRDN0000737558	0	Olfr1270	0,53
Tmem254c	0	Zfp563	0,53	Sipa1	0	1810055G02Rik	0,53
Tmem254b	0	Nhlh2	0,53	Mup2	0	Scrn3	0,53
Tmem253	0	Tnfrsf8	0,53	4930467E23Rik	0	Sgpp2	0,53
Tmem251	0	Hsd17b14	0,53	Wnt7a	0	Eva1c	0,53
Tmem25	0	Hesx1	0,53	Ssbp3	0	Otub2	0,53
Tmem247	0	Gzmk	0,53	Lamp1	0	Adss	0,53
Tmem246	0	Paxbp1	0,53	Trpc4ap	0	Trmt44	0,53
Tmem243	0	Cldn3	0,53	Osbpl11	0	Dph5	0,53
Tmem242	0	Vmn2r23	0,53	Tsta3	0	Mesp2	0,53
Tmem241	0	Wtip	0,53	Ssbp2	0	Serpinc1	0,53
Tmem240	0	Dnajc7	0,53	Tcl1	0	Cyp27a1	0,53
Tmem238	0	Rhd	0,53	Htr7	0	Gm1553	0,53
Tmem237	0	Npm3	0,53	Gadd45b	0	1300017J02Rik	0,53
Tmem236	0	Klf16	0,53	Mup1	0	Brip1	0,52
Tmem235	0	Olfr1323	0,53	Mnt	0	Wdr47	0,52
Tmem234	0	Actr3	0,53	Fam221a	0	Tmem98	0,52
Tmem232	0	Krtap6-5	0,53	Fam221b	0	Defa26	0,52
Tmem231	0	Vmn2r30	0,52	Gars	0	Npm3	0,52
Tmem229b	0	Hspa14	0,52	Lsm12	0	Gm10439	0,52
Tmem229a	0	Selo	0,52	Prkcz	0	Nat14	0,52
Tmem225	0	BRDN0000738174	0,52	Scx	0	Skor1	0,52
Tmem222	0	Zfp42	0,52	Olfr881	0	Hectd3	0,52
Tmem221	0	Vamp8	0,52	Sct	0	1810043H04Rik	0,52
Tmem220	0	Wisp3	0,52	Tfe3	0	BRDN0000737864	0,52
Tmem219	0	Ssr1	0,52	Olfr1511	0	Nog	0,52
Tmem218	0	Tac4	0,52	Slc25a40	0	H2afy	0,52
Tmem217	0	Vmn1r198	0,52	Slc25a41	0	Dgkg	0,52
Tmem215	0	Olfr960	0,52	Slc25a42	0	Ppargc1a	0,52
Tmem213	0	Myrf	0,52	Slc25a44	0	Nek5	0,52
Tmem212	0	Pnpla5	0,52	Slc25a46	0	Nmral1	0,52
Tmem211	0	Iqcg	0,52	Slc25a47	0	Sytl5	0,52
Tmem210	0	Birc2	0,52	Slc25a48	0	Tbkbp1	0,52
Tmem209	0	AF251705	0,52	Svs2	0	Ercc2	0,52
Tmem208	0	Tnfsf12	0,52	Olfr1513	0	Sfn	0,52
Tmem206	0	Spint2	0,52	Ecm2	0	Fermt1	0,52
Tmem205	0	1700093K21Rik	0,52	Ecm1	0	Pip4k2a	0,52
Tmem203	0	Olfr597	0,52	Kcnt1	0	C5ar1	0,52
Tmem202	0	Gm11554	0,52	Tmem212	0	Scn3a	0,52
Tmem201	0	Fam71d	0,52	Tmem211	0	Olfr722	0,52
Tmem200c	0	Ythdf3	0,52	Tmem210	0	Mapk9	0,52
Tmem200a	0	4930544D05Rik	0,52	Tmem217	0	Pcdhgb5	0,52
Tmem2	0	Isl1	0,52	Tmem216	0	Nhsl1	0,52
Tmem199	0	Pgam1	0,52	Tmem215	0	Kcng4	0,52
Tmem198	0	Gm8898	0,52	Tmem214	0	Ferd3l	0,52
Tmem196	0	Pus1	0,52	8430408G22Rik	0	Iyd	0,52
Tmem192	0	Lynx1	0,52	Tmem219	0	Zc3hav1	0,52
Tmem191c	0	1700018F24Rik	0,52	Olfr887	0	Pik3r5	0,52
Tmem190	0	Gm14374	0,52	1700030F18Rik	0	Acap3	0,52
Tmem19	0	Polg	0,52	BRDN0000737475	0	Arhgef37	0,52
Tmem189	0	Sp2	0,52	Sh3rf2	0	Zbed3	0,52
Tmem186	0	Ifitm5	0,52	Sh3rf3	0	Lgalsl	0,52
Tmem185b	0	Kcnn1	0,52	Hbegf	0	Setd5	0,52
Tmem184c	0	Itpripl2	0,52	Olfr866	0	Tmc8	0,52
Tmem184a	0	Zfp174	0,52	C9	0	Cyb561	0,52
Tmem183a	0	Fam13c	0,52	Fam24a	0	1700047I17Rik2	0,52
Tmem182	0	Apopt1	0,52	Rtbdn	0	Nudcd2	0,52
Tmem180	0	Nlrp6	0,52	Cytl1	0	BRDN0000737846	0,52
Tmem18	0	6430571L13Rik	0,52	Tecrl	0	Rap1gds1	0,52
Tmem179	0	Hoxd3	0,52	Srrm3	0	Shank3	0,52
Tmem178b	0	Kcns1	0,52	Lrrc74b	0	Cxcl3	0,52
Tmem178	0	Rhobtb1	0,52	Asxl3	0	Dym	0,52
Tmem177	0	Car9	0,52	Iqcf3	0	1700018F24Rik	0,52
Tmem176b	0	Mocs1	0,52	Knstrn	0	Ankle1	0,52
Tmem176a	0	Olfr1009	0,52	Vmn1r142	0	Olfr741	0,52
Tmem175	0	Magi2	0,52	Ppm1e	0	Doc2b	0,52
Tmem174	0	Nhlh1	0,52	Ppm1d	0	Zfp846	0,52
Tmem173	0	S100a1	0,52	Dnah6	0	Phox2b	0,52
Tmem171	0	BRDN0000737517	0,52	Ppm1b	0	Hdac10	0,52
Tmem170b	0	Crip1	0,52	Rpl8	0	Dbx2	0,52
Tmem170	0	Snrk	0,52	Rpl9	0	Rab11fip4	0,52
Tmem17	0	Bap1	0,52	Rpl6	0	Ankrd6	0,52
Tmem169	0	Tfrc	0,52	Rpl7	0	Nipa2	0,52
Tmem168	0	Gm11992	0,52	Rpl4	0	Dnase1l1	0,52
Tmem167b	0	Tas1r3	0,52	Ppm1l	0	Phf21a	0,52
Tmem167	0	Mycbpap	0,52	Ppm1k	0	Pepd	0,52
Tmem165	0	Ldlrad2	0,52	Rpl3	0	Chit1	0,52
Tmem164	0	Kcnj5	0,52	Ppm1h	0	Tfap2c	0,52
Tmem163	0	Kcne1	0,52	Pid1	0	Spns2	0,52

Tmem161b	0	Chrnb2	0,52	C77080	0	Jup	0,52
Tmem161a	0	Tmem222	0,52	Gns	0	Olig1	0,52
Tmem159	0	Kif26b	0,52	Glp1r	0	Prlhr	0,52
Tmem154	0	Ces3a	0,51	Lcor	0	Tmem191c	0,52
Tmem151b	0	Cnnm2	0,51	Mecr	0	Olfr399	0,52
Tmem150c	0	Sfrp5	0,51	4930550C14Rik	0	2510002D24Rik	0,52
Tmem150a	0	Bhlhe41	0,51	Olfr689	0	Vmn1r33	0,52
Tmem147	0	Camkk2	0,51	Hs3st5	0	Olfr1217	0,52
Tmem145	0	Ppp6c	0,51	Hs3st4	0	4933427D14Rik	0,52
Tmem144	0	Apol10b	0,51	Aldh4a1	0	Gm11567	0,52
Tmem143	0	Olfr1505	0,51	Hs3st2	0	Calr4	0,51
Tmem141	0	Teddm1b	0,51	Hs3st1	0	Speer2	0,51
Tmem140	0	Zcchc14	0,51	Dll1	0	D1Pas1	0,51
Tmem139	0	BRDN0000737522	0,51	Zhx2	0	Tecrl	0,51
Tmem135	0	Ttc9	0,51	Pgam2	0	Elk1	0,51
Tmem132c	0	Emid1	0,51	Zhx1	0	Pnldc1	0,51
Tmem132b	0	Ilf2	0,51	Atp6v1e1	0	Ptpn3	0,51
Tmem130	0	Marco	0,51	Atp6v1e2	0	Pde4b	0,51
Tmem128	0	Faxc	0,51	Cntn2	0	7420426K07Rik	0,51
Tmem127	0	Rab33a	0,51	Cntn3	0	Lcn6	0,51
Tmem126b	0	Fkbpl	0,51	Chadl	0	Ambra1	0,51
Tmem126a	0	Ephb3	0,51	Cntn1	0	Pdia6	0,51
Tmem125	0	Wdfy1	0,51	Cntn6	0	Tnfsf12	0,51
Tmem120a	0	Stk39	0,51	Ccdc32	0	Anln	0,51
Tmem119	0	6330416G13Rik	0,51	Pld3	0	Exoc6b	0,51
Tmem117	0	Elovl5	0,51	BRDN0000738294	0	H1foo	0,51
Tmem115	0	Gm590	0,51	Mcur1	0	Dock4	0,51
Tmem114	0	Pm20d1	0,51	Nat8l	0	Slc25a11	0,51
Tmem110	0	Olfr545	0,51	4930447C04Rik	0	Atp6v1c2	0,51
Tmem11	0	Cyp2b23	0,51	Ncoa1	0	Ston1	0,51
Tmem109	0	Glrx5	0,51	Neurod2	0	Gk5	0,51
Tmem108	0	Klk1b11	0,51	Trappc3l	0	Optc	0,51
Tmem107	0	Syne2	0,51	Dqx1	0	Insl3	0,51
Tmem106b	0	Ascl3	0,51	Col26a1	0	Il10	0,51
Tmem106a	0	Glyatl3	0,51	D8Ertd738e	0	Tmbim6	0,51
Tmem102	0	Ska1	0,51	Pag1	0	Pcdha4	0,51
Tmem101	0	Cpeb4	0,51	Tle1	0	Npas4	0,51
Tmem100	0	Gm6406	0,51	Lect2	0	Gls	0,51
Tmeff2	0	Zfp276	0,51	Arhgap30	0	Ubl7	0,51
Tmed9	0	Adamts16	0,51	Tle6	0	Pip5k1a	0,51
Tmed8	0	Papolg	0,51	Ncr1	0	Fam173a	0,51
Tmed7	0	Hoxb7	0,51	Utp11l	0	Spata31d1d	0,51
Tmed5	0	Kiz	0,51	Tacstd2	0	Zfp407	0,51
Tmed4	0	Chic1	0,51	Srrm4	0	Chtf18	0,51
Tmed2	0	Gpr39	0,51	Rbpjl	0	Eif4e2	0,51
Tmed11	0	Lce1j	0,51	Espl1	0	Bcap29	0,51
Tmed10	0	Snx12	0,51	Rex2	0	Sun2	0,51
Tmed1	0	Fdxr	0,51	BC068281	0	Vrk3	0,51
Tmco6	0	Col5a2	0,51	Fancg	0	Ipmk	0,51
Tmco5b	0	Ascc1	0,51	Gm5634	0	Dbp	0,51
Tmco5	0	Qser1	0,51	Irf4	0	Tmem241	0,51
Tmco4	0	Gas2l3	0,51	Slc15a4	0	Zbtb16	0,51
Tmco3	0	C77370	0,51	Defb47	0	Smtnl2	0,51
Tmco2	0	Pcdhb6	0,51	Defb46	0	Rsph4a	0,51
Tmco1	0	BRDN0000737445	0,51	Defb45	0	Nphp3	0,51
Tmcc3	0	Mgat5b	0,51	Lpcat2b	0	Rab3il1	0,51
Tmcc2	0	Mrgprb4	0,51	Defb43	0	Laptm4a	0,51
Tmc8	0	Fam193a	0,51	Gm4461	0	Fabp2	0,51
Tmc7	0	Socs6	0,51	Cfap45	0	Arhgap28	0,51
Tmc6	0	BRDN0000737552	0,51	Defb40	0	Tax1bp1	0,51
Tmc5	0	Ghrl	0,51	BRDN0000737717	0	Klra6	0,51
Tmc4	0	Cracr2a	0,51	Olfr123	0	Zfp764	0,51
Tmc2	0	Slc22a8	0,50	BRDN0000738000	0	Vps39	0,51
Tmc1	0	Atxn7l3b	0,50	BRDN0000737714	0	Bzw1	0,51
Tmbim7	0	Olfr520	0,50	Fam178a	0	Zbtb49	0,51
Tmbim6	0	Ntng1	0,50	BRDN0000738007	0	Tnnc2	0,50
Tmbim4	0	March4	0,50	BRDN0000737711	0	Traf4	0,50
Tmbim1	0	Rad51b	0,50	Defb48	0	Sub1	0,50
Tma7	0	Gk2	0,50	Arid3a	0	Lhfp	0,50
Tma16	0	Kdelr1	0,50	Nat9	0	Hs3st3b1	0,50
Tm9sf4	0	BRDN0000737874	0,50	Fhod3	0	Rab12	0,50
Tm9sf3	0	Cda	0,50	Olfr1459	0	Peli2	0,50
Tm9sf1	0	Dnajc18	0,50	Olfr653	0	Thrsp	0,50
Tm7sf2	0	Uxs1	0,50	Sncaip	0	Inpp5a	0,50
Tm4sf5	0	Agbl5	0,50	Plxna2	0	Msantd2	0,50
Tm4sf20	0	Ces1d	0,50	Nat3	0	Lrrc4	0,50
Tm4sf19	0	Dhtkd1	0,50	Krt40	0	Btg3	0,50
Tm2d3	0	Nt5c2	0,50	Olfr1457	0	C130074G19Rik	0,50
Tm2d1	0	Nlrp3	0,50	Olfr652	0	Kdelr1	0,50
Tlx1	0	Aox2	0,50	Olfr1451	0	Smo	0,50
Tlr9	0	3110001I22Rik	0,50	Olfr1450	0	Desi2	0,50
Tlr8	0	Sae1	0,50	Olfr1453	0	Mboat2	0,50

Tlr6	0	Zfp428	0,50	Sulf2	0	Myo1g	0,50
Tlr4	0	Ocm	0,50	Hacd3	0	Prl6a1	0,50
Tlr3	0	Gria4	0,50	Sulf1	0	BRDN0000738054	0,50
Tlr2	0	Pcsk6	0,50	Aldh7a1	0	Cckbr	0,50
Tlr12	0	Cldn22	0,50	Pomt1	0	Siah3	0,50
Tlr11	0	Olfr68	0,50	Pcdha6	0	Kcnb1	0,50
Tlr1	0	Sec22b	0,50	Pomt2	0	Mccc1	0,50
Tln2	0	Fth1	0,50	Krtap8-1	0	Krtap27-1	0,50
Tln1	0	N4bp2l2	0,50	Mxd1	0	Olfr979	0,50
Tll2	0	Ankrd61	0,50	Moap1	0	Adck2	0,50
Tlk2	0	Lsm3	0,50	Sdr39u1	0	Gm21498	0,50
Tlk1	0	Srsf6	0,50	Irg1	0	Zfand6	0,50
Tle6	0	Cant1	0,50	Tram2	0	Npc2	0,50
Tle4	0	Gpr153	0,50	Grm7	0	Gm1587	0,50
Tle3	0	Nat6	0,50	Plxna1	0	Amotl1	0,50
Tle2	0	Foxs1	0,50	Olfr659	0	Nf2	0,50
Tldc2	0	Zfp408	0,50	Pcdha5	0	Tas2r137	0,50
Tldc1	0	Parp4	0,50	Ezr	0	BRDN0000737788	0,50
Tlcd1	0	Gm648	0,50	1110008F13Rik	0	Tmem173	0,50
Tktl2	0	Zfp703	0,49	Grm1	0	Apcs	0,50
Tktl1	0	Matn1	0,49	Atrnl1	0	Nr5a2	0,49
Tkt	0	Ubqln1	0,49	Setd8	0	Tfpi	0,49
Tkfc	0	Akap17b	0,49	Fat1	0	Zfp874b	0,49
Tk2	0	Tcf19	0,49	Olfr798	0	Me1	0,49
Tk1	0	Pde6g	0,49	Fat4	0	BRDN0000737531	0,49
Tjap1	0	Larp1	0,49	Olfr794	0	Avpi1	0,49
Tirap	0	Apbb3	0,49	Olfr796	0	BRDN0000737725	0,49
Tiprl	0	Vstm2a	0,49	Olfr128	0	Capn5	0,49
Tiparp	0	Car3	0,49	Olfr790	0	Myl9	0,49
Tinf2	0	Lhx5	0,49	4930506M07Rik	0	Tktl2	0,49
Tinag	0	Olfr713	0,49	Zscan25	0	Ccnjl	0,49
Timp4	0	Asb9	0,49	Irf9	0	8030411F24Rik	0,49
Timp2	0	Dlg5	0,49	Wrb	0	Map3k9	0,49
Timp1	0	Ubap2	0,49	Gpatch3	0	Psme1	0,49
Timmdc1	0	Gm16532	0,49	Wrn	0	Zfp566	0,49
Timm9	0	Slc25a18	0,49	Pik3ap1	0	C030039L03Rik	0,49
Timm8b	0	Amer3	0,49	Arhgef28	0	Fscn1	0,49
Timm8a1	0	Lrriq4	0,49	Tspan2	0	Vmn1r237	0,49
Timm50	0	Lancl3	0,49	Tspan3	0	Pxdc1	0,49
Timm44	0	Tra2a	0,49	Tspan1	0	Gsc2	0,49
Timm23	0	Gmip	0,49	Tspan6	0	Fbxo27	0,49
Timm22	0	Ankrd50	0,49	Tspan7	0	Sec1	0,49
Timm21	0	Rapgef4	0,49	Cln8	0	Otof	0,49
Timm13	0	Anxa7	0,49	Tspan5	0	Ddx39	0,49
Timm10b	0	Carhsp1	0,49	Tspan8	0	Slc22a2	0,49
Timm10	0	Gstm7	0,49	Arf4	0	Galnt12	0,49
Timeless	0	Olfr322	0,49	Pm20d1	0	Irg1	0,49
Timd4	0	Gm20822	0,49	1700001J03Rik	0	Gnb1l	0,49
Tigit	0	Vpreb3	0,49	Pm20d2	0	Gm2799	0,49
Tigd5	0	Hecw2	0,49	Plekhg3	0	Coprs	0,49
Tigd4	0	Irgc1	0,49	Stxbp2	0	Tbpl2	0,48
Tigd3	0	Pcbp4	0,48	Tnmd	0	Klhdc4	0,48
Tigd2	0	2810021J22Rik	0,48	Zfhx4	0	Il5	0,48
Tigar	0	BRDN0000737940	0,48	Zfhx3	0	Irx5	0,48
Tifab	0	Mtnr1b	0,48	Tmie	0	Sftpa1	0,48
Tifa	0	Tbx3	0,48	R3hdml	0	Fermt2	0,48
Tie1	0	Ciz1	0,48	Gm11128	0	Ldlrad3	0,48
Ticrr	0	Dlec1	0,48	Bcl9	0	Foxd4	0,48
Ticam2	0	BRDN0000737767	0,48	1700106J16Rik	0	Dennd5b	0,48
Ticam1	0	Hes2	0,48	Prm3	0	Barhl2	0,48
Tiam2	0	Ctsb	0,48	Prm2	0	Hdac11	0,48
Tiam1	0	St6galnac1	0,48	Hmha1	0	Olfr95	0,48
Tial1	0	Fam136a	0,48	Hes1	0	Msi1	0,48
Thyn1	0	Kdelc2	0,48	Gm14440	0	Usp49	0,48
Thy1	0	Dok2	0,48	Bcl6	0	Pou3f3	0,48
Thumpd2	0	Olfr1461	0,48	Gm14446	0	BRDN0000738146	0,47
Thumpd1	0	Smarcad1	0,48	Hes5	0	Spink6	0,47
Thtpa	0	Letm2	0,48	Hes6	0	Tnks1bp1	0,47
Thsd7b	0	Mplkip	0,48	Hes7	0	Slamf1	0,47
Thsd7a	0	Hapln2	0,48	Avpr1b	0	Mbd1	0,47
Thsd4	0	D7Ertd443e	0,48	Avpr1a	0	Setd7	0,47
Thrsp	0	Ctrc	0,48	Stxbp5	0	Mboat4	0,47
Thrb	0	Trim6	0,48	Ccdc116	0	Olfr374	0,47
Thrap3	0	Olfr1494	0,47	Ccdc117	0	Churc1	0,47
Thra	0	Bpifc	0,47	Ccdc114	0	Lrmp	0,47
Thpo	0	Olfr1053	0,47	Qpctl	0	Crip1	0,46
Thop1	0	Pxdc1	0,47	Ccdc112	0	Spta1	0,46
Thoc7	0	Pcdhga5	0,47	Ccdc113	0	BRDN0000738232	0,46
Thoc6	0	Zscan4d	0,47	Ccdc110	0	Zzz3	0,00
Thoc5	0	Dcaf11	0,47	Wdr86	0	Zzef1	0,00
Thoc3	0	Grcc10	0,47	Lamc1	0	Zyx	0,00
Thoc2	0	Fzd1	0,47	Pcp4l1	0	Zyg11a	0,00

Thoc1	0	Stfa3	0,46	Gm9376	0	Zxdb	0,00
Thnsl2	0	Nup188	0,46	Wdr82	0	Zwint	0,00
Thnsl1	0	Sirt7	0,46	Wdr83	0	Zwilch	0,00
Themis2	0	Eva1a	0,46	Ndufb10	0	Zw10	0,00
Them7	0	Map3k3	0,46	4930579G24Rik	0	Zufsp	0,00
Them6	0	9130008F23Rik	0,45	BRDN0000737432	0	Zswim8	0,00
Them5	0	Dennd1b	0,45	Smu1	0	Zswim7	0,00
Them4	0	Zzz3	0,00	Wdr89	0	Zswim6	0,00
Theg	0	Zzef1	0,00	Nucks1	0	Zswim5	0,00
Thbs4	0	Zyx	0,00	Mlph	0	Zswim1	0,00
Thbs2	0	Zyg11b	0,00	Msmo1	0	Zscan5b	0,00
Thbd	0	Zyg11a	0,00	Ost4	0	Zscan4f	0,00
Thap7	0	Zxdc	0,00	Cacna2d1	0	Zscan4d	0,00
Thap3	0	Zxdb	0,00	Stk38l	0	Zscan4b	0,00
Thap2	0	Zwint	0,00	Hdhd3	0	Zscan29	0,00
Thap11	0	Zwilch	0,00	Hdhd2	0	Zscan25	0,00
Thap1	0	Zufsp	0,00	Vps16	0	Zscan20	0,00
Thada	0	Zswim7	0,00	Itih2	0	Zscan2	0,00
Tha1	0	Zswim5	0,00	R3hdm1	0	Zscan12	0,00
Tgtp1	0	Zswim4	0,00	Vps11	0	Zscan10	0,00
Tgs1	0	Zswim1	0,00	Nid2	0	Zrsr2	0,00
Tgoln1	0	Zscan5b	0,00	Nid1	0	Zrsr1	0,00
Tgm6	0	Zscan4f	0,00	4930550L24Rik	0	Zranb3	0,00
Tgm4	0	Zscan4c	0,00	Sf3a1	0	Zranb2	0,00
Tgm1	0	Zscan29	0,00	Sf3a2	0	Zranb1	0,00
Tgif2lx2	0	Zscan22	0,00	Sf3a3	0	Zpr1	0,00
Tgif2lx1	0	Zscan20	0,00	BRDN0000738283	0	Zpld1	0,00
Tgif2	0	Zscan2	0,00	Kif5a	0	Zpbp	0,00
Tgif1	0	Zscan18	0,00	Pitpna	0	Zp3r	0,00
Tgfbrap1	0	Zscan12	0,00	BRDN0000737847	0	Zp3	0,00
Tgfbr3	0	Zscan10	0,00	Plekhg6	0	Zp2	0,00
Tgfbr1	0	Zrsr1	0,00	Pitpnb	0	Zp1	0,00
Tgfb3	0	Zranb3	0,00	BRDN0000738060	0	Znrf3	0,00
Tgfb2	0	Zranb2	0,00	Sprtn	0	Znrf2	0,00
Tgfb1i1	0	Zranb1	0,00	Aagab	0	Znrd1as	0,00
Tgfb1	0	Zpr1	0,00	Ly6g6c	0	Znrd1	0,00
Tgfa	0	Zpld1	0,00	Ly6g6f	0	Znhit6	0,00
Tgds	0	Zpbp2	0,00	Sstr1	0	Znhit3	0,00
Tg	0	Zpbp	0,00	Sstr3	0	Znhit2	0,00
Tfrc	0	Zp3	0,00	Sstr2	0	Znfx1	0,00
Tfr2	0	Zp2	0,00	Sstr5	0	Znf512b	0,00
Tfpt	0	Zp1	0,00	Sstr4	0	Zmynd19	0,00
Tfpi2	0	Znrf4	0,00	Prame	0	Zmynd15	0,00
Tff3	0	Znrf1	0,00	9130011E15Rik	0	Zmynd12	0,00
Tff2	0	Znrd1	0,00	Stxbp6	0	Zmynd11	0,00
Tff1	0	Znhit6	0,00	BRDN0000737730	0	Zmym5	0,00
Tfec	0	Znhit3	0,00	Stx1a	0	Zmym4	0,00
Tfeb	0	Znhit2	0,00	Emilin1	0	Zmym2	0,00
Tfdp2	0	Znfx1	0,00	BRDN0000737597	0	Zmpste24	0,00
Tfdp1	0	Znf512b	0,00	Nr5a2	0	Zmiz1	0,00
Tfcp2l1	0	Zmynd8	0,00	C1ql2	0	Zmat5	0,00
Tfcp2	0	Zmynd15	0,00	Prpf38b	0	Zmat3	0,00
Tfb1m	0	Zmynd12	0,00	Krt80	0	Zmat2	0,00
Tfap4	0	Zmynd11	0,00	Ercc1	0	Zmat1	0,00
Tfap2e	0	Zmynd10	0,00	Ercc2	0	Zkscan8	0,00
Tfap2d	0	Zmym6	0,00	Ercc4	0	Zkscan6	0,00
Tfap2c	0	Zmym5	0,00	Ercc5	0	Zkscan4	0,00
Tfap2b	0	Zmym4	0,00	Ilkap	0	Zkscan2	0,00
Tfap2a	0	Zmym1	0,00	Srpk3	0	Zkscan17	0,00
Tex9	0	Zmpste24	0,00	Scpep1	0	Zim1	0,00
Tex40	0	Zmiz1	0,00	BRDN0000738068	0	Zic5	0,00
Tex38	0	Zmat5	0,00	Ifnl2	0	Zic3	0,00
Tex37	0	Zmat4	0,00	Foxh1	0	Zic2	0,00
Tex36	0	Zmat3	0,00	Exd2	0	Zhx3	0,00
Tex33	0	Zmat2	0,00	Exd1	0	Zhx2	0,00
Tex30	0	Zmat1	0,00	BRDN0000738069	0	Zhx1	0,00
Tex28	0	Zkscan7	0,00	Ifi47	0	Zgrf1	0,00
Tex264	0	Zkscan6	0,00	Ppp1r16a	0	Zgpat	0,00
Tex261	0	Zkscan4	0,00	Ppp1r16b	0	Zg16	0,00
Tex26	0	Zkscan3	0,00	Ifi44	0	Zfyve28	0,00
Tex24	0	Zkscan2	0,00	Nlrx1	0	Zfyve27	0,00
Tex21	0	Zkscan17	0,00	Secisbp2	0	Zfyve26	0,00
Tex2	0	Zkscan16	0,00	P4htm	0	Zfyve16	0,00
Tex19.2	0	Zkscan14	0,00	Tbccd1	0	Zfyve1	0,00
Tex19.1	0	Zkscan1	0,00	Maneal	0	Zfy2	0,00
Tex16	0	Zik1	0,00	Myadml2	0	Zfy1	0,00
Tex15	0	Zic5	0,00	Gm13251	0	Zfx	0,00
Tex14	0	Zic3	0,00	Slc1a1	0	Zfr2	0,00
Tex13	0	Zic2	0,00	Rpap2	0	Zfr	0,00
Tex12	0	Zhx3	0,00	Rpap3	0	Zfpm2	0,00
Tex101	0	Zhx1	0,00	Hexim2	0	Zfpm1	0,00
Tex10	0	Zgrf1	0	BRDN0000737738	0	Zfpl1	0,00

Tet2	0	Zgpat	0	Rab39b	0	Zfp97	0,00
Tet1	0	Zglp1	0	Mill1	0	Zfp963	0,00
Tespa1	0	Zg16	0	Zfp628	0	Zfp961	0,00
Tesk2	0	Zfyve28	0	Zfp629	0	Zfp957	0,00
Tesk1	0	Zfyve27	0	Plekhg5	0	Zfp955a	0,00
Tesc	0	Zfyve21	0	Nsmaf	0	Zfp954	0,00
Tes	0	Zfyve19	0	Tmed8	0	Zfp953	0,00
Tert	0	Zfyve16	0	Zfp623	0	Zfp952	0,00
Terf2ip	0	Zfyve1	0	Ms4a8a	0	Zfp951	0,00
Terf1	0	Zfy2	0	Sult5a1	0	Zfp949	0,00
Tepp	0	Zfy1	0	Gnb5	0	Zfp948	0,00
Tep1	0	Zfx	0	Cp	0	Zfp944	0,00
Tenm3	0	Zfr2	0	Mbtps1	0	Zfp943	0,00
Tenm2	0	Zfr	0	Lrit2	0	Zfp942	0,00
Ten1	0	Zfpm1	0	Lrit3	0	Zfp941	0,00
Telo2	0	Zfp97	0	Lrit1	0	Zfp94	0,00
Tekt5	0	Zfp963	0	Plcl2	0	Zfp938	0,00
Tekt4	0	Zfp961	0	BC016579	0	Zfp935	0,00
Tekt1	0	Zfp959	0	Plcl1	0	Zfp933	0,00
Tek	0	Zfp957	0	Stx11	0	Zfp931	0,00
Tefm	0	Zfp956	0	Msantd2	0	Zfp92	0,00
Tef	0	Zfp955a	0	Amfr	0	Zfp91	0,00
Teddm2	0	Zfp954	0	Stx12	0	Zfp9	0,00
Teddm1b	0	Zfp953	0	Keg1	0	Zfp882	0,00
Teddm1a	0	Zfp952	0	Sall3	0	Zfp879	0,00
Tectb	0	Zfp951	0	Zscan4c	0	Zfp873	0,00
Tecta	0	Zfp949	0	Tcp11l1	0	Zfp871	0,00
Tecr	0	Zfp948	0	Zscan4f	0	Zfp870	0,00
Tecpr2	0	Zfp947	0	Armcx5	0	Zfp87	0,00
Tecpr1	0	Zfp946	0	Zscan4d	0	Zfp869	0,00
Tec	0	Zfp944	0	Tcaf3	0	Zfp867	0,00
Tead4	0	Zfp942	0	1700013H16Rik	0	Zfp866	0,00
Tead1	0	Zfp940	0	Tcaf1	0	Zfp865	0,00
Tdrkh	0	Zfp94	0	Olfr1193	0	Zfp850	0,00
Tdrd9	0	Zfp937	0	Gsdmd	0	Zfp84	0,00
Tdrd7	0	Zfp935	0	Slc1a3	0	Zfp831	0,00
Tdrd6	0	Zfp934	0	Krtap5-5	0	Zfp830	0,00
Tdrd5	0	Zfp933	0	Alk	0	Zfp821	0,00
Tdrd3	0	Zfp932	0	Armcx3	0	Zfp820	0,00
Tdrd12	0	Zfp93	0	Olfr173	0	Zfp82	0,00
Tdrd1	0	Zfp92	0	Armcx2	0	Zfp81	0,00
Tdpoz5	0	Zfp91	0	Tyms	0	Zfp809	0,00
Tdpoz4	0	Zfp90	0	Tppp3	0	Zfp808	0,00
Tdpoz3	0	Zfp9	0	Tppp2	0	Zfp804a	0,00
Tdpoz2	0	Zfp874b	0	Armcx1	0	Zfp800	0,00
Tdpoz1	0	Zfp874a	0	Lyrm1	0	Zfp799	0,00
Tdp2	0	Zfp873	0	Pmp2	0	Zfp791	0,00
Tdgf1	0	Zfp872	0	Olfr1197	0	Zfp786	0,00
Tdg	0	Zfp871	0	Vmn1r191	0	Zfp784	0,00
Tctn3	0	Zfp869	0	Tmem189	0	Zfp781	0,00
Tctn1	0	Zfp867	0	Mrpl10	0	Zfp780b	0,00
Tctex1d4	0	Zfp865	0	Mrpl11	0	Zfp78	0,00
Tctex1d2	0	Zfp850	0	Mrpl12	0	Zfp777	0,00
Tctex1d1	0	Zfp84	0	Mrpl14	0	Zfp775	0,00
Tcte2	0	Zfp839	0	Mybph	0	Zfp773	0,00
Tcte1	0	Zfp830	0	Mrpl16	0	Zfp772	0,00
Tcstv1	0	Zfp827	0	Spint5	0	Zfp771	0,00
Tcp11l2	0	Zfp821	0	Mrpl18	0	Zfp770	0,00
Tcp11	0	Zfp819	0	Mrpl19	0	Zfp768	0,00
Tcp10b	0	Zfp81	0	Krtap5-3	0	Zfp763	0,00
Tcp10a	0	Zfp809	0	Ier3	0	Zfp760	0,00
Tcp1	0	Zfp804b	0	Rab11fip5	0	Zfp758	0,00
Tcof1	0	Zfp804a	0	Aaed1	0	Zfp748	0,00
Tcn2	0	Zfp800	0	Dtnb	0	Zfp747	0,00
Tcl1b5	0	Zfp799	0	Stac	0	Zfp746	0,00
Tcl1b4	0	Zfp791	0	Dtna	0	Zfp740	0,00
Tcl1b3	0	Zfp786	0	Pdxp	0	Zfp735	0,00
Tcl1b2	0	Zfp784	0	Ache	0	Zfp729b	0,00
Tcl1b1	0	Zfp780b	0	Gabpa	0	Zfp72	0,00
Tcirg1	0	Zfp78	0	Pdcd1	0	Zfp712	0,00
Tchp	0	Zfp777	0	Glt28d2	0	Zfp711	0,00
Tchhl1	0	Zfp775	0	Prrg4	0	Zfp710	0,00
Tchh	0	Zfp773	0	Gpr85	0	Zfp706	0,00
Tcfl5	0	Zfp772	0	Prrg1	0	Zfp704	0,00
Tcf7l2	0	Zfp771	0	Prrg2	0	Zfp692	0,00
Tcf7l1	0	Zfp770	0	Prpf19	0	Zfp689	0,00
Tcf7	0	Zfp768	0	Prpf18	0	Zfp687	0,00
Tcf4	0	Zfp764	0	Poldip3	0	Zfp68	0,00
Tcf3	0	Zfp763	0	Atg4d	0	Zfp677	0,00
Tcf25	0	Zfp760	0	Cacna1e	0	Zfp672	0,00
Tcf24	0	Zfp759	0	Gstp2	0	Zfp663	0,00
Tcf23	0	Zfp758	0	Sdr42e1	0	Zfp661	0,00

Tcf21	0	Zfp748	0	Arhgef25	0	Zfp658	0,00
Tcf19	0	Zfp747	0	Hcrt	0	Zfp655	0,00
Tcf15	0	Zfp746	0	H2-M10.1	0	Zfp654	0,00
Tcf12	0	Zfp738	0	H2-M10.3	0	Zfp652	0,00
Tcerg1l	0	Zfp735	0	BC052040	0	Zfp651	0,00
Tcerg1	0	Zfp729b	0	H2-M10.5	0	Zfp65	0,00
Tceb3	0	Zfp72	0	H2-M10.4	0	Zfp647	0,00
Tceb2	0	Zfp715	0	Adss	0	Zfp646	0,00
Tceb1	0	Zfp712	0	Dbx2	0	Zfp644	0,00
Tceanc2	0	Zfp711	0	Ccser1	0	Zfp641	0,00
Tceanc	0	Zfp710	0	Dbx1	0	Zfp637	0,00
Tceal7	0	Zfp706	0	Rdm1	0	Zfp629	0,00
Tceal6	0	Zfp704	0	Cyp4a29	0	Zfp628	0,00
Tceal5	0	Zfp697	0	Poldip2	0	Zfp623	0,00
Tcea2	0	Zfp691	0	Eva1a	0	Zfp619	0,00
Tcea1	0	Zfp69	0	Eva1b	0	Zfp618	0,00
Tcam1	0	Zfp689	0	Eva1c	0	Zfp616	0,00
Tcaf3	0	Zfp688	0	Map1lc3a	0	Zfp612	0,00
Tcaf2	0	Zfp687	0	Cdpf1	0	Zfp608	0,00
Tcaf1	0	Zfp677	0	Stag2	0	Zfp607	0,00
Tc2n	0	Zfp668	0	Htatsf1	0	Zfp606	0,00
Tbxas1	0	Zfp667	0	Olfr593	0	Zfp605	0,00
Tbxa2r	0	Zfp664	0	Impad1	0	Zfp600	0,00
Tbx5	0	Zfp661	0	Gpr87	0	Zfp60	0,00
Tbx4	0	Zfp658	0	Ptprn2	0	Zfp599	0,00
Tbx22	0	Zfp652	0	Stag1	0	Zfp598	0,00
Tbx21	0	Zfp651	0	Cyfip1	0	Zfp597	0,00
Tbx20	0	Zfp65	0	H3f3b	0	Zfp595	0,00
Tbx2	0	Zfp648	0	Clec12a	0	Zfp593	0,00
Tbx19	0	Zfp647	0	Snx2	0	Zfp59	0,00
Tbx18	0	Zfp646	0	Olfr596	0	Zfp583	0,00
Tbx15	0	Zfp644	0	Hspb2	0	Zfp579	0,00
Tbx1	0	Zfp641	0	Cdca5	0	Zfp575	0,00
Tbrg4	0	Zfp629	0	Hgs	0	Zfp574	0,00
Tbrg1	0	Zfp628	0	Abo	0	Zfp57	0,00
Tbr1	0	Zfp62	0	Acat1	0	Zfp568	0,00
Tbpl2	0	Zfp618	0	Abr	0	Zfp563	0,00
Tbp	0	Zfp617	0	Acat3	0	Zfp558	0,00
Tbl3	0	Zfp612	0	Ica1	0	Zfp551	0,00
Tbl2	0	Zfp608	0	Gnb3	0	Zfp541	0,00
Tbl1xr1	0	Zfp607	0	Mitf	0	Zfp54	0,00
Tbl1x	0	Zfp606	0	Hgf	0	Zfp536	0,00
Tbk1	0	Zfp605	0	Tctex1d2	0	Zfp534	0,00
Tbck	0	Zfp60	0	Kctd21	0	Zfp526	0,00
Tbcel	0	Zfp599	0	Tctex1d4	0	Zfp52	0,00
Tbcd	0	Zfp595	0	Cd52	0	Zfp518b	0,00
Tbccd1	0	Zfp592	0	Cd53	0	Zfp518a	0,00
Tbcc	0	Zfp583	0	Cmc2	0	Zfp516	0,00
Tbcb	0	Zfp579	0	Ctnnal1	0	Zfp513	0,00
Tbca	0	Zfp575	0	Snx5	0	Zfp512	0,00
Tbc1d9b	0	Zfp574	0	Cd55	0	Zfp51	0,00
Tbc1d9	0	Zfp57	0	Clec4g	0	Zfp503	0,00
Tbc1d8b	0	Zfp566	0	Cd8b1	0	Zfp493	0,00
Tbc1d8	0	Zfp558	0	Clec4e	0	Zfp488	0,00
Tbc1d7	0	Zfp551	0	Clec4d	0	Zfp473	0,00
Tbc1d5	0	Zfp541	0	Amdhd2	0	Zfp467	0,00
Tbc1d4	0	Zfp536	0	Smdt1	0	Zfp46	0,00
Tbc1d32	0	Zfp53	0	Smcr8	0	Zfp459	0,00
Tbc1d31	0	Zfp526	0	Nudt9	0	Zfp458	0,00
Tbc1d30	0	Zfp523	0	Lactbl1	0	Zfp457	0,00
Tbc1d2b	0	Zfp521	0	Gnb2	0	Zfp455	0,00
Tbc1d24	0	Zfp52	0	Rfc3	0	Zfp454	0,00
Tbc1d23	0	Zfp518b	0	Mto1	0	Zfp451	0,00
Tbc1d22b	0	Zfp516	0	Gm10697	0	Zfp449	0,00
Tbc1d22a	0	Zfp511	0	Ggn	0	Zfp446	0,00
Tbc1d21	0	Zfp51	0	Ggh	0	Zfp445	0,00
Tbc1d20	0	Zfp507	0	Krt84	0	Zfp444	0,00
Tbc1d2	0	Zfp503	0	Gmnc	0	Zfp438	0,00
Tbc1d19	0	Zfp474	0	Mmp14	0	Zfp428	0,00
Tbc1d17	0	Zfp467	0	Mmp15	0	Zfp426	0,00
Tbc1d15	0	Zfp462	0	Mmp16	0	Zfp423	0,00
Tbc1d14	0	Zfp46	0	Mmp10	0	Zfp422	0,00
Tbc1d13	0	Zfp459	0	Mmp11	0	Zfp42	0,00
Tbc1d12	0	Zfp458	0	Mmp12	0	Zfp414	0,00
Tbc1d10c	0	Zfp457	0	Tpo	0	Zfp410	0,00
Tbc1d10b	0	Zfp455	0	Tex30	0	Zfp408	0,00
Tbc1d1	0	Zfp454	0	Tex37	0	Zfp40	0,00
Tbata	0	Zfp451	0	Mmp19	0	Zfp398	0,00
Taz	0	Zfp449	0	Cyp2e1	0	Zfp397	0,00
Tax1bp3	0	Zfp445	0	Pcdhb8	0	Zfp395	0,00
Tax1bp1	0	Zfp444	0	Fdxr	0	Zfp386	0,00
Tatdn2	0	Zfp438	0	Klrb1f	0	Zfp385c	0,00

Tatdn1	0	Zfp429	0	Kcne1	0	Zfp384	0,00
Tat	0	Zfp423	0	Rfc2	0	Zfp382	0,00
Tas2r144	0	Zfp422	0	Tpr	0	Zfp36l3	0,00
Tas2r143	0	Zfp418	0	Klrb1b	0	Zfp36l2	0,00
Tas2r140	0	Zfp414	0	Klrb1c	0	Zfp36l1	0,00
Tas2r139	0	Zfp407	0	Arglu1	0	Zfp369	0,00
Tas2r138	0	Zfp398	0	Cmtr2	0	Zfp366	0,00
Tas2r137	0	Zfp39	0	Cmtr1	0	Zfp365	0,00
Tas2r136	0	Zfp385c	0	Gpsm2	0	Zfp36	0,00
Tas2r135	0	Zfp385b	0	Gpsm3	0	Zfp358	0,00
Tas2r130	0	Zfp384	0	Gpsm1	0	Zfp354c	0,00
Tas2r129	0	Zfp383	0	Vmn1r30	0	Zfp352	0,00
Tas2r126	0	Zfp382	0	Vmn1r31	0	Zfp35	0,00
Tas2r125	0	Zfp36l2	0	Vmn1r32	0	Zfp346	0,00
Tas2r124	0	Zfp36l1	0	Vmn1r33	0	Zfp345	0,00
Tas2r123	0	Zfp369	0	Ermn	0	Zfp341	0,00
Tas2r122	0	Zfp365	0	Vmn1r35	0	Zfp335	0,00
Tas2r121	0	Zfp362	0	Vmn1r36	0	Zfp334	0,00
Tas2r120	0	Zfp358	0	Vmn1r37	0	Zfp330	0,00
Tas2r119	0	Zfp354c	0	Vmn1r38	0	Zfp326	0,00
Tas2r118	0	Zfp352	0	Kcnrg	0	Zfp324	0,00
Tas2r117	0	Zfp35	0	Ptx3	0	Zfp318	0,00
Tas2r116	0	Zfp346	0	Ppef2	0	Zfp317	0,00
Tas2r114	0	Zfp345	0	Tph2	0	Zfp316	0,00
Tas2r113	0	Zfp335	0	Ppef1	0	Zfp300	0,00
Tas2r110	0	Zfp330	0	Cstf1	0	Zfp30	0,00
Tas2r109	0	Zfp329	0	BRDN0000737909	0	Zfp3	0,00
Tas2r108	0	Zfp324	0	Cypt12	0	Zfp296	0,00
Tas2r107	0	Zfp322a	0	Mtor	0	Zfp292	0,00
Tas2r106	0	Zfp317	0	Slc29a2	0	Zfp287	0,00
Tas2r104	0	Zfp300	0	Slc29a1	0	Zfp286	0,00
Tas2r103	0	Zfp30	0	Galnt13	0	Zfp281	0,00
Tas2r102	0	Zfp3	0	Sez6	0	Zfp280c	0,00
Tas1r3	0	Zfp296	0	Cd5l	0	Zfp280b	0,00
Tas1r2	0	Zfp287	0	Trdn	0	Zfp28	0,00
Tas1r1	0	Zfp282	0	Olfr635	0	Zfp277	0,00
Tarsl2	0	Zfp280d	0	Klk1b16	0	Zfp275	0,00
Tars2	0	Zfp280c	0	Nt5c1a	0	Zfp273	0,00
Tars	0	Zfp280b	0	Olfr631	0	Zfp27	0,00
Tarm1	0	Zfp275	0	Nup107	0	Zfp266	0,00
Tarbp2	0	Zfp273	0	Cdkn2aip	0	Zfp263	0,00
Tapt1	0	Zfp27	0	Vmn1r199	0	Zfp26	0,00
Tapbpl	0	Zfp266	0	Olfr638	0	Zfp251	0,00
Tapbp	0	Zfp263	0	BRDN0000737502	0	Zfp248	0,00
Tap2	0	Zfp260	0	BRDN0000737503	0	Zfp239	0,00
Tap1	0	Zfp26	0	BRDN0000737500	0	Zfp235	0,00
Taok1	0	Zfp251	0	BC147527	0	Zfp229	0,00
Tank	0	Zfp239	0	BRDN0000737506	0	Zfp219	0,00
Tango6	0	Zfp235	0	BRDN0000737507	0	Zfp217	0,00
Tango2	0	Zfp229	0	Adpgk	0	Zfp213	0,00
Tanc2	0	Zfp219	0	BRDN0000737505	0	Zfp212	0,00
Tanc1	0	Zfp217	0	BRDN0000737508	0	Zfp207	0,00
Tamm41	0	Zfp213	0	BRDN0000737509	0	Zfp202	0,00
Taldo1	0	Zfp212	0	Top2b	0	Zfp2	0,00
Tal2	0	Zfp207	0	Uqcc3	0	Zfp191	0,00
Tal1	0	Zfp2	0	Top2a	0	Zfp189	0,00
Tagln3	0	Zfp191	0	Ppp1r42	0	Zfp185	0,00
Tagln2	0	Zfp182	0	Gm5294	0	Zfp184	0,00
Tagln	0	Zfp180	0	Rspo3	0	Zfp182	0,00
Tagap	0	Zfp169	0	Ugt2b5	0	Zfp180	0,00
Taf9b	0	Zfp157	0	Dppa5a	0	Zfp174	0,00
Taf7	0	Zfp148	0	Ppargc1a	0	Zfp169	0,00
Taf6l	0	Zfp146	0	Ppargc1b	0	Zfp160	0,00
Taf6	0	Zfp143	0	Gm8898	0	Zfp157	0,00
Taf5l	0	Zfp14	0	Rhbdf2	0	Zfp148	0,00
Taf5	0	Zfp131	0	2010005H15Rik	0	Zfp146	0,00
Taf4b	0	Zfp128	0	Poteg	0	Zfp143	0,00
Taf3	0	Zfp120	0	Tmem178b	0	Zfp131	0,00
Taf2	0	Zfp119b	0	Hsh2d	0	Zfp128	0,00
Taf1d	0	Zfp114	0	4930433I11Rik	0	Zfp120	0,00
Taf1c	0	Zfp113	0	Scimp	0	Zfp12	0,00
Taf1b	0	Zfp109	0	Arhgap9	0	Zfp119b	0,00
Taf15	0	Zfp106	0	Gm14781	0	Zfp119a	0,00
Taf12	0	Zfp1	0	Uroc1	0	Zfp114	0,00
Taf11	0	Zfml	0	Nos2	0	Zfp113	0,00
Tada3	0	Zfhx4	0	Foxp2	0	Zfp111	0,00
Tada2b	0	Zfhx2	0	Shroom4	0	Zfp106	0,00
Tada2a	0	Zfc3h1	0	Suv420h2	0	Zfp105	0,00
Tacstd2	0	Zfat	0	Tspan4	0	Zfml	0,00
Tacr3	0	Zfand5	0	1700029F12Rik	0	Zfhx4	0,00
Tacr2	0	Zfand2b	0	2700029M09Rik	0	Zfhx3	0,00
Tacr1	0	Zfand2a	0	Rfk	0	Zfhx2	0,00

Taco1	0	Zer1	0	Vangl1	0	Zfc3h1	0,00
Tacc2	0	Zeb2	0	Vangl2	0	Zfat	0,00
Tac4	0	Zdhhc9	0	BRDN0000737726	0	Zfand5	0,00
Tac1	0	Zdhhc8	0	Ing1	0	Zfand2b	0,00
Tab3	0	Zdhhc7	0	Ing2	0	Zfand2a	0,00
Tab2	0	Zdhhc6	0	Ing3	0	Zfand1	0,00
Tab1	0	Zdhhc5	0	Olfr1024	0	Zer1	0,00
Taar8c	0	Zdhhc3	0	Snrnp200	0	Zeb2	0,00
Taar8b	0	Zdhhc25	0	Olfr1026	0	Zdhhc9	0,00
Taar8a	0	Zdhhc22	0	Olfr1306	0	Zdhhc8	0,00
Taar7f	0	Zdhhc21	0	Olfr749	0	Zdhhc6	0,00
Taar7e	0	Zdhhc20	0	Il18rap	0	Zdhhc5	0,00
Taar7d	0	Zdhhc2	0	Olfr1022	0	Zdhhc3	0,00
Taar7b	0	Zdhhc19	0	Olfr1023	0	Zdhhc25	0,00
Taar7a	0	Zdhhc17	0	Olfr1028	0	Zdhhc24	0,00
Taar4	0	Zdhhc15	0	Olfr1029	0	Zdhhc21	0,00
Taar3	0	Zdhhc14	0	Olfr1226	0	Zdhhc20	0,00
Taar2	0	Zdhhc13	0	Cog7	0	Zdhhc2	0,00
Taar1	0	Zdhhc12	0	Efs	0	Zdhhc19	0,00
T	0	Zdhhc11	0	C3ar1	0	Zdhhc17	0,00
Syvn1	0	Zdhhc1	0	Clec2d	0	Zdhhc16	0,00
Sytl5	0	Zcwpw1	0	Dnah2	0	Zdhhc15	0,00
Sytl4	0	Zcrb1	0	Med29	0	Zdhhc14	0,00
Sytl3	0	Zcchc8	0	Olfr1225	0	Zdhhc13	0,00
Sytl2	0	Zcchc7	0	Fgf17	0	Zdhhc12	0,00
Syt6	0	Zcchc6	0	Nobox	0	Zdhhc11	0,00
Syt5	0	Zcchc5	0	Sostdc1	0	Zdhhc1	0,00
Syt4	0	Zcchc4	0	Rassf8	0	Zdbf2	0,00
Syt3	0	Zcchc3	0	Kalrn	0	Zcwpw1	0,00
Syt2	0	Zcchc2	0	Snrk	0	Zcrb1	0,00
Syt17	0	Zcchc18	0	Dmp1	0	Zcchc9	0,00
Syt16	0	Zcchc17	0	Mael	0	Zcchc8	0,00
Syt15	0	Zcchc16	0	Uba6	0	Zcchc7	0,00
Syt14	0	Zcchc13	0	Uba7	0	Zcchc6	0,00
Syt13	0	Zcchc12	0	Uba5	0	Zcchc5	0,00
Syt12	0	Zcchc11	0	Gm4907	0	Zcchc4	0,00
Syt10	0	Zcchc10	0	Olfr1220	0	Zcchc18	0,00
Syt1	0	Zc4h2	0	Uba1	0	Zcchc17	0,00
Sys1	0	Zc3h8	0	BRDN0000737478	0	Zcchc16	0,00
Sypl2	0	Zc3h7b	0	Pwp2	0	Zcchc14	0,00
Sypl	0	Zc3h7a	0	Cyp11b1	0	Zcchc13	0,00
Syp	0	Zc3h4	0	Ascl5	0	Zcchc12	0,00
Synrg	0	Zc3h3	0	Lig3	0	Zcchc10	0,00
Synpo2l	0	Zc3h18	0	Pmf1	0	Zc4h2	0,00
Synpo2	0	Zc3h15	0	Nos1	0	Zc3hav1l	0,00
Synpo	0	Zc3h14	0	Vmn2r68	0	Zc3h7a	0,00
Synm	0	Zc3h13	0	Vmn2r69	0	Zc3h6	0,00
Synj2bp	0	Zc3h12c	0	Creg1	0	Zc3h4	0,00
Synj2	0	Zc3h12a	0	Stk31	0	Zc3h3	0,00
Synj1	0	Zc3h11a	0	Vmn2r62	0	Zc3h18	0,00
Syngr4	0	Zc3h10	0	Sin3b	0	Zc3h15	0,00
Syngr3	0	Zc2hc1c	0	Vmn2r60	0	Zc3h14	0,00
Syngap1	0	Zc2hc1b	0	Vmn2r61	0	Zc3h13	0,00
Syne4	0	Zc2hc1a	0	Vmn2r66	0	Zc3h12d	0,00
Syne2	0	Zbtb9	0	Scgb3a2	0	Zc3h12b	0,00
Syne1	0	Zbtb8os	0	Vmn2r65	0	Zc3h12a	0,00
Syndig1l	0	Zbtb5	0	Med21	0	Zc3h11a	0,00
Syndig1	0	Zbtb48	0	Tnip1	0	Zc3h10	0,00
Sync	0	Zbtb44	0	Uckl1	0	Zc2hc1c	0,00
Synb	0	Zbtb42	0	Mmp23	0	Zc2hc1b	0,00
Syna	0	Zbtb4	0	Tnfrsf4	0	Zbtb8os	0,00
Syn3	0	Zbtb39	0	Clint1	0	Zbtb8b	0,00
Sympk	0	Zbtb37	0	Pnoc	0	Zbtb8a	0,00
Syk	0	Zbtb34	0	Mrps25	0	Zbtb7c	0,00
Syde1	0	Zbtb32	0	Sin3a	0	Zbtb7b	0,00
Sycp3	0	Zbtb3	0	Rassf3	0	Zbtb7a	0,00
Sycp2	0	Zbtb25	0	Sap25	0	Zbtb6	0,00
Sycp1	0	Zbtb21	0	Zfp36l3	0	Zbtb5	0,00
Sycn	0	Zbtb2	0	Ttll5	0	Zbtb48	0,00
Syce3	0	Zbtb18	0	Med22	0	Zbtb43	0,00
Syce2	0	Zbtb17	0	BRDN0000737955	0	Zbtb42	0,00
Syce1l	0	Zbtb14	0	Hat1	0	Zbtb41	0,00
Sybu	0	Zbtb12	0	Sltm	0	Zbtb40	0,00
Syap1	0	Zbtb11	0	4930544D05Rik	0	Zbtb39	0,00
Swt1	0	Zbtb1	0	Gstm2	0	Zbtb37	0,00
Swsap1	0	Zbp1	0	Gstm4	0	Zbtb34	0,00
Swi5	0	Zbed5	0	Strbp	0	Zbtb33	0,00
Swap70	0	Zbed4	0	Gstm6	0	Zbtb3	0,00
Svs5	0	Zbed3	0	Oasl1	0	Zbtb26	0,00
Svs4	0	Zbbx	0	Ddb2	0	Zbtb25	0,00
Svs3b	0	Zar1l	0	BRDN0000737476	0	Zbtb21	0,00
Svs3a	0	Zar1	0	Rassf7	0	Zbtb20	0,00

Svs2	0	Zap70	0	Dmpk	0	Zbtb2	0,00
Svs1	0	Zan	0	Stk39	0	Zbtb18	0,00
Svopl	0	Zak	0	Pdf	0	Zbtb14	0,00
Svop	0	Zadh2	0	Pdc	0	Zbtb12	0,00
Svip	0	Ywhaz	0	Dck	0	Zbtb11	0,00
Svil	0	Ywhaq	0	3632451O06Rik	0	Zbtb10	0,00
Svep1	0	Ywhah	0	Dsc2	0	Zbtb1	0,00
Sval2	0	Ywhag	0	Pno1	0	Zbp1	0,00
Sva	0	Ywhae	0	Rad21l	0	Zbed5	0,00
Sv2b	0	Ythdf2	0	Shmt2	0	Zbed4	0,00
Sv2a	0	Ythdf1	0	Abhd17a	0	Zbbx	0,00
Suz12	0	Ythdc1	0	Snrnp35	0	Zar1l	0,00
Suv420h2	0	Yrdc	0	Lamp3	0	Zar1	0,00
Suv420h1	0	Ypel5	0	Lamp2	0	Zadh2	0,00
Suv39h2	0	Ypel3	0	Was	0	Yy2	0,00
Suv39h1	0	Ypel2	0	Abi3	0	Yy1	0,00
Susd6	0	Yod1	0	Kcnj2	0	Ywhaz	0,00
Susd5	0	Ylpm1	0	Olfr800	0	Ywhaq	0,00
Susd4	0	Ykt6	0	Myt1	0	Ywhab	0,00
Susd2	0	Yipf7	0	Tmem67	0	Ythdf3	0,00
Surf6	0	Yipf6	0	Tmem65	0	Ythdf2	0,00
Surf4	0	Yipf4	0	Tmem64	0	Ythdf1	0,00
Surf1	0	Yipf3	0	Id1	0	Ythdc2	0,00
Supv3l1	0	Yipf2	0	Tmem62	0	Ythdc1	0,00
Supt6	0	Yipf1	0	Sh3bgrl3	0	Yrdc	0,00
Supt5	0	Yif1a	0	Sh3bgrl2	0	Ypel5	0,00
Supt4a	0	Yes1	0	Cd276	0	Ypel3	0,00
Supt3	0	Yeats4	0	Fignl2	0	Ypel2	0,00
Supt20	0	Yeats2	0	Olfr243	0	Ypel1	0,00
Supt16	0	Ybx1	0	Tmem69	0	Yod1	0,00
Suox	0	Yars2	0	Foxred2	0	Yme1l1	0,00
Sun3	0	Yars	0	Foxred1	0	Ykt6	0,00
Sun1	0	Yap1	0	Plac9b	0	Yipf7	0,00
Sumo3	0	Yaf2	0	Serpinb12	0	Yipf6	0,00
Sumo2	0	Yae1d1	0	Slc25a16	0	Yipf4	0,00
Sumo1	0	Xylt1	0	Stxbp3a	0	Yipf1	0,00
Sumf1	0	Xylb	0	4930430F08Rik	0	Yif1b	0,00
Sult6b1	0	Xxylt1	0	Acmsd	0	Yif1a	0,00
Sult3a1	0	Xrra1	0	Bag3	0	Yes1	0,00
Sult2b1	0	Xrn2	0	Nrk	0	Yeats4	0,00
Sult2a7	0	Xrcc6bp1	0	Cpox	0	Ydjc	0,00
Sult2a6	0	Xrcc6	0	H1f0	0	Ybx3	0,00
Sult2a5	0	Xrcc5	0	Bcam	0	Ybx1	0,00
Sult2a3	0	Xrcc4	0	Nrm	0	Yars	0,00
Sult1e1	0	Xrcc3	0	Nrl	0	Yap1	0,00
Sult1c2	0	Xrcc2	0	Litaf	0	Yaf2	0,00
Sult1c1	0	Xrcc1	0	Pros1	0	Yae1d1	0,00
Sult1b1	0	Xpr1	0	Olfr572	0	Xylt2	0,00
Sulf2	0	Xpot	0	Ttc33	0	Xylt1	0,00
Sulf1	0	Xpo6	0	Ttc32	0	Xylb	0,00
Sugt1	0	Xpo4	0	Ttc37	0	Xxylt1	0,00
Sugct	0	Xpo1	0	Ttc36	0	Xrra1	0,00
Suds3	0	Xpnpep1	0	Ttc34	0	Xrn2	0,00
Suco	0	Xpc	0	4933403O08Rik	0	Xrn1	0,00
Suclg2	0	Xpa	0	Plxnd1	0	Xrcc6	0,00
Sub1	0	Xntrpc	0	St6galnac5	0	Xrcc5	0,00
Styx	0	Xlr4b	0	Ttc38	0	Xrcc1	0,00
Styk1	0	Xlr4a	0	St6galnac3	0	Xpr1	0,00
Stxbp5	0	Xlr3b	0	St6galnac2	0	Xpot	0,00
Stxbp4	0	Xlr3a	0	St6galnac1	0	Xpo6	0,00
Stxbp3a	0	Xlr	0	Cox7b2	0	Xpo5	0,00
Stxbp2	0	Xkr9	0	Rara	0	Xpo4	0,00
Stxbp1	0	Xkr8	0	Olfml2b	0	Xpo1	0,00
Stx8	0	Xkr7	0	Gm609	0	Xpnpep1	0,00
Stx7	0	Xkr5	0	Aoah	0	Xntrpc	0,00
Stx6	0	Xirp2	0	Brd4	0	Xndc1	0,00
Stx5a	0	Xiap	0	Fam43b	0	Xlr5b	0,00
Stx4a	0	Xdh	0	Ttc30b	0	Xlr5a	0,00
Stx3	0	Xcr1	0	Ufm1	0	Xlr4b	0,00
Stx2	0	Xcl1	0	Btrc	0	Xlr4a	0,00
Stx1b	0	Xab2	0	2810408M09Rik	0	Xlr3b	0,00
Stx19	0	Wwtr1	0	Pde2a	0	Xlr3a	0,00
Stx17	0	Wwox	0	Wap	0	Xlr	0,00
Stx16	0	Wwc2	0	Rars	0	Xkrx	0,00
Stx12	0	Wwc1	0	Rarg	0	Xkr9	0,00
Stub1	0	Wtap	0	5430421N21Rik	0	Xkr8	0,00
Stt3a	0	Wsb2	0	Fam195b	0	Xkr7	0,00
Strn4	0	Wsb1	0	Wnt5b	0	Xkr6	0,00
Strn	0	Wrnip1	0	Mlx	0	Xkr5	0,00
Strip2	0	Wrn	0	Tada1	0	Xkr4	0,00
Strip1	0	Wrb	0	Gpatch4	0	Xk	0,00
Strbp	0	Wrap73	0	Tada3	0	Xirp1	0,00

Strap	0	Wrap53	0	Col11a1	0	Xdh	0,00
Strada	0	Wnt9b	0	Tlcd2	0	Xcr1	0,00
Stra6	0	Wnt9a	0	Arhgap39	0	Xcl1	0,00
Stra13	0	Wnt8b	0	Ptms	0	Xbp1	0,00
Stpg1	0	Wnt7b	0	Ccdc102a	0	Xab2	0,00
Stox2	0	Wnt6	0	Ugt3a2	0	Wwtr1	0,00
Stox1	0	Wnt5a	0	Gpatch2	0	Wwp2	0,00
Ston2	0	Wnt4	0	Psme4	0	Wwp1	0,00
Ston1	0	Wnt2b	0	Ugt3a1	0	Wwc2	0,00
Stoml3	0	Wnt2	0	Psme2	0	Wwc1	0,00
Stoml2	0	Wnt16	0	Psme3	0	Wtip	0,00
Stmnd1	0	Wnt11	0	Ndufb11	0	Wtap	0,00
Stmn4	0	Wnt10b	0	1700056E22Rik	0	Wt1	0,00
Stmn1	0	Wnt10a	0	Synpo2	0	Wsb2	0,00
Stkld1	0	Wnt1	0	Bag6	0	Wsb1	0,00
Stk40	0	Wnk4	0	Ino80d	0	Wrnip1	0,00
Stk4	0	Wnk3	0	Vmn1r46	0	Wrb	0,00
Stk39	0	Wnk2	0	Ino80b	0	Wnt9b	0,00
Stk38l	0	Wnk1	0	Ino80c	0	Wnt8a	0,00
Stk38	0	Wls	0	Prosc	0	Wnt7b	0,00
Stk36	0	Wiz	0	Crat	0	Wnt6	0,00
Stk32c	0	Wisp2	0	Pcdh9	0	Wnt5b	0,00
Stk32b	0	Wisp1	0	Rspo1	0	Wnt5a	0,00
Stk31	0	Wipi1	0	Tectb	0	Wnt4	0,00
Stk3	0	Wipf3	0	Rspo4	0	Wnt3	0,00
Stk25	0	Wipf1	0	Gnpda2	0	Wnt2b	0,00
Stk24	0	Wibg	0	Pcdh1	0	Wnt11	0,00
Stk19	0	Whsc1l1	0	Obox5	0	Wnt10b	0,00
Stk17b	0	Whsc1	0	Gnpda1	0	Wnt1	0,00
Stk16	0	Whrn	0	Obox3	0	Wnk4	0,00
Stk11ip	0	Wfikkn2	0	Obox2	0	Wnk3	0,00
Stk11	0	Wfikkn1	0	Obox1	0	Wnk1	0,00
Stk10	0	Wfdc6b	0	Pcdh7	0	Wiz	0,00
Stip1	0	Wfdc6a	0	Epha3	0	Wisp3	0,00
Stim2	0	Wfdc5	0	H2-M10.2	0	Wisp2	0,00
Stim1	0	Wfdc3	0	Rbm4	0	Wisp1	0,00
Stil	0	Wfdc21	0	Tbcel	0	Wipi1	0,00
Stfa2	0	Wfdc2	0	Tmem239	0	Wipf3	0,00
Stfa1	0	Wfdc18	0	Tmem238	0	Wif1	0,00
Steap4	0	Wfdc15b	0	Tmem235	0	Wibg	0,00
Steap3	0	Wfdc15a	0	Tmem234	0	Whsc1	0,00
Steap1	0	Wfdc13	0	Tmem236	0	Whrn	0,00
Stc2	0	Wfdc12	0	Tmem231	0	Wfs1	0,00
Stc1	0	Wfdc11	0	Tmem230	0	Wfdc8	0,00
Stbd1	0	Wfdc10	0	Atp5g3	0	Wfdc6b	0,00
Stau2	0	Wfdc1	0	Tmem232	0	Wfdc21	0,00
Stat5b	0	Wee2	0	Scfd1	0	Wfdc18	0,00
Stat4	0	Wee1	0	Zfp454	0	Wfdc17	0,00
Stat3	0	Wdsub1	0	Zfp457	0	Wfdc15b	0,00
Stard7	0	Wdr95	0	Zfp451	0	Wfdc11	0,00
Stard6	0	Wdr93	0	Dbndd2	0	Wfdc1	0,00
Stard5	0	Wdr92	0	Cntln	0	Wee2	0,00
Stard3	0	Wdr91	0	Trmt1l	0	Wee1	0,00
Stard13	0	Wdr89	0	BRDN0000738153	0	Wdtc1	0,00
Stard10	0	Wdr83	0	Zfp459	0	Wdsub1	0,00
Star	0	Wdr82	0	BC037034	0	Wdr95	0,00
Stap2	0	Wdr81	0	Ggt5	0	Wdr93	0,00
Stap1	0	Wdr78	0	Fam195a	0	Wdr92	0,00
Stambpl1	0	Wdr77	0	Akr1b8	0	Wdr90	0,00
Stambp	0	Wdr75	0	Akr1b3	0	Wdr89	0,00
Stam2	0	Wdr74	0	Lxn	0	Wdr83	0,00
Stam	0	Wdr73	0	Akr1b7	0	Wdr82	0,00
Stag3	0	Wdr72	0	Dync1li1	0	Wdr78	0,00
Stag2	0	Wdr70	0	Thumpd2	0	Wdr77	0,00
Stag1	0	Wdr62	0	Maml2	0	Wdr75	0,00
Stac	0	Wdr61	0	Gm10256	0	Wdr74	0,00
Stab2	0	Wdr60	0	Mypop	0	Wdr73	0,00
Stab1	0	Wdr6	0	Maml1	0	Wdr72	0,00
St8sia6	0	Wdr5b	0	Large	0	Wdr70	0,00
St8sia5	0	Wdr59	0	1110025L11Rik	0	Wdr63	0,00
St8sia3	0	Wdr54	0	Dnah9	0	Wdr62	0,00
St8sia2	0	Wdr5	0	BRDN0000737839	0	Wdr61	0,00
St8sia1	0	Wdr48	0	Olfr745	0	Wdr60	0,00
St7l	0	Wdr47	0	Gm20594	0	Wdr6	0,00
St7	0	Wdr45b	0	Bmpr1a	0	Wdr5b	0,00
St6galnac6	0	Wdr45	0	Bmpr1b	0	Wdr59	0,00
St6galnac5	0	Wdr43	0	Hes2	0	Wdr54	0,00
St6galnac3	0	Wdr41	0	Rcbtb1	0	Wdr53	0,00
St6galnac2	0	Wdr4	0	Gm13871	0	Wdr5	0,00
St6galnac1	0	Wdr38	0	Qk	0	Wdr48	0,00
St6gal1	0	Wdr36	0	Hdx	0	Wdr46	0,00
St5	0	Wdr35	0	5430435G22Rik	0	Wdr45b	0,00

St3gal6	0	Wdr31	0	Esr1	0	Wdr45	0,00
St3gal5	0	Wdr3	0	BRDN0000737466	0	Wdr43	0,00
St3gal4	0	Wdr26	0	Bace2	0	Wdr38	0,00
St3gal3	0	Wdr25	0	Slc25a20	0	Wdr37	0,00
St3gal2	0	Wdr24	0	Uchl1	0	Wdr36	0,00
St18	0	Wdr20rt	0	Serp2	0	Wdr35	0,00
St14	0	Wdr20	0	Serp1	0	Wdr33	0,00
Ssxb9	0	Wdr19	0	Olfr1474	0	Wdr31	0,00
Ssxb8	0	Wdr18	0	Mpl	0	Wdr3	0,00
Ssxb3	0	Wdr13	0	Cml1	0	Wdr27	0,00
Ssxb2	0	Wdr12	0	Cml2	0	Wdr26	0,00
Ssxb10	0	Wdr11	0	Tapt1	0	Wdr25	0,00
Ssxb1	0	Wdr1	0	Ppp2r5d	0	Wdr24	0,00
Ssx9	0	Wdpcp	0	Ppp2r5e	0	Wdr20rt	0,00
Ssu72	0	Wdfy4	0	Rergl	0	Wdr20	0,00
Ssty1	0	Wdfy3	0	Ppp2r5a	0	Wdr19	0,00
Sstr5	0	Wbscr28	0	Ppp2r5b	0	Wdr18	0,00
Sstr3	0	Wbscr27	0	Ppp2r5c	0	Wdr13	0,00
Sstr2	0	Wbscr22	0	Camkmt	0	Wdr12	0,00
Sstr1	0	Wbscr16	0	Nfkb1	0	Wdr11	0,00
Sssca1	0	Wbp5	0	Psors1c2	0	Wdr1	0,00
Ssrp1	0	Wbp4	0	BRDN0000737585	0	Wdpcp	0,00
Ssr4	0	Wbp2nl	0	Klhdc8a	0	Wdfy4	0,00
Ssr3	0	Wbp2	0	Edem3	0	Wdfy1	0,00
Ssr1	0	Wbp1l	0	Wars2	0	Wbscr28	0,00
Sspo	0	Wasl	0	Zfp296	0	Wbscr22	0,00
Sspn	0	Wash1	0	Exoc2	0	Wbscr17	0,00
Ssmem1	0	Wasf3	0	Dr1	0	Wbscr16	0,00
Ssh2	0	Wasf2	0	Wfdc12	0	Wbp5	0,00
Ssh1	0	Was	0	Bpifa1	0	Wbp4	0,00
Ssfa2	0	Wars2	0	Omp	0	Wbp2nl	0,00
Ssc4d	0	Wars	0	Tmem184a	0	Wbp2	0,00
Ssbp4	0	Wap	0	Tmem184b	0	Wbp1l	0,00
Ssbp3	0	Wac	0	Tmem184c	0	Wbp11	0,00
Ssbp2	0	Vwde	0	Tfdp2	0	Wbp1	0,00
Ssbp1	0	Vwc2l	0	Tfdp1	0	Wasl	0,00
Ss18l1	0	Vwc2	0	Scin	0	Wash1	0,00
Ss18	0	Vwa9	0	Zfp474	0	Wasf3	0,00
Sry	0	Vwa8	0	Fam81a	0	Wasf2	0,00
Srxn1	0	Vwa7	0	Arntl2	0	Was	0,00
Srsf9	0	Vwa5b2	0	Wfdc17	0	Wars2	0,00
Srsf7	0	Vwa5b1	0	Tfec	0	Wars	0,00
Srsf6	0	Vwa3a	0	Tfeb	0	Wap	0,00
Srsf5	0	Vti1a	0	Olfr802	0	Wac	0,00
Srsf4	0	Vtcn1	0	Sumo3	0	Vwf	0,00
Srsf3	0	Vsx2	0	Sumo2	0	Vwde	0,00
Srsf2	0	Vstm4	0	Pds5a	0	Vwc2l	0,00
Srsf12	0	Vstm2l	0	Pds5b	0	Vwc2	0,00
Srsf11	0	Vsnl1	0	Vmn1r169	0	Vwa9	0,00
Srsf10	0	Vsig8	0	Defb20	0	Vwa5b2	0,00
Srsf1	0	Vsig10	0	Defb23	0	Vwa5b1	0,00
Srrt	0	Vsig1	0	Defb22	0	Vwa3a	0,00
Srrm4	0	Vrtn	0	Defb25	0	Vwa2	0,00
Srrm3	0	Vrk3	0	1700028K03Rik	0	Vtn	0,00
Srrm1	0	Vrk2	0	BRDN0000738022	0	Vti1a	0,00
Srrd	0	Vrk1	0	Defb26	0	Vtcn1	0,00
Srr	0	Vps8	0	Defb29	0	Vta1	0,00
Srpx2	0	Vps54	0	Vmn1r160	0	Vsx2	0,00
Srpx	0	Vps53	0	Rb1cc1	0	Vsx1	0,00
Srprb	0	Vps52	0	BRDN0000738028	0	Vstm5	0,00
Srpr	0	Vps4b	0	Olfr401	0	Vstm4	0,00
Srpk2	0	Vps4a	0	Olfr402	0	Vstm2l	0,00
Srp9	0	Vps45	0	Olfr403	0	Vstm2b	0,00
Srp72	0	Vps37d	0	Atpaf2	0	Vstm2a	0,00
Srp68	0	Vps37c	0	Podn	0	Vsnl1	0,00
Srp54b	0	Vps37b	0	Atpaf1	0	Vsig4	0,00
Srp54a	0	Vps37a	0	Gm3139	0	Vsig10	0,00
Srp19	0	Vps36	0	Smndc1	0	Vsig1	0,00
Srms	0	Vps33b	0	9130204L05Rik	0	Vrtn	0,00
Srm	0	Vps33a	0	Col22a1	0	Vrk2	0,00
Sri	0	Vps29	0	BC048403	0	Vrk1	0,00
Srgn	0	Vps26b	0	Espnl	0	Vps9d1	0,00
Srgap3	0	Vps26a	0	BRDN0000738353	0	Vps53	0,00
Srgap2	0	Vps25	0	F2r	0	Vps52	0,00
Srf	0	Vps18	0	Trappc11	0	Vps51	0,00
Srek1	0	Vps16	0	Trappc13	0	Vps4a	0,00
Srebf2	0	Vps13d	0	Trappc12	0	Vps45	0,00
Srebf1	0	Vps13c	0	Tsnaxip1	0	Vps41	0,00
Srd5a3	0	Vps13a	0	Gatad1	0	Vps37d	0,00
Srd5a2	0	Vps11	0	Ms4a13	0	Vps37b	0,00
Srd5a1	0	Vpreb2	0	Tubb6	0	Vps37a	0,00
Srcin1	0	Vpreb1	0	Vipas39	0	Vps36	0,00

Srcap	0	Vprbp	0	Tubb5	0	Vps35	0,00
Src	0	Vnn1	0	BC027231	0	Vps33b	0,00
Srbd1	0	Vmp1	0	Tubb3	0	Vps33a	0,00
Sqstm1	0	Vmo1	0	Tubb1	0	Vps29	0,00
Sqle	0	Vmn2r99	0	Tbx10	0	Vps26b	0,00
Spz1	0	Vmn2r98	0	BRDN0000738281	0	Vps25	0,00
Spty2d1	0	Vmn2r96	0	BRDN0000738282	0	Vps16	0,00
Sptssb	0	Vmn2r95	0	Cnot8	0	Vps13d	0,00
Sptssa	0	Vmn2r93	0	BRDN0000738284	0	Vps13c	0,00
Sptlc3	0	Vmn2r92	0	Tcerg1l	0	Vps13b	0,00
Sptlc1	0	Vmn2r91	0	Pet100	0	Vps13a	0,00
Sptbn4	0	Vmn2r90	0	BRDN0000738287	0	Vps11	0,00
Sptbn2	0	Vmn2r9	0	Tbx18	0	Vpreb3	0,00
Sptbn1	0	Vmn2r89	0	BRDN0000738289	0	Vpreb2	0,00
Sptb	0	Vmn2r88	0	9330159F19Rik	0	Vpreb1	0,00
Sptan1	0	Vmn2r87	0	Comtd1	0	Vprbp	0,00
Spta1	0	Vmn2r85	0	Pdlim2	0	Vopp1	0,00
Spt1	0	Vmn2r84	0	Kcnip3	0	Vnn3	0,00
Spsb4	0	Vmn2r79	0	Tcerg1	0	Vnn1	0,00
Spsb2	0	Vmn2r78	0	Syt7	0	Vmp1	0,00
Spsb1	0	Vmn2r76	0	Nr2c2ap	0	Vmo1	0,00
Spryd7	0	Vmn2r73	0	Kcne1l	0	Vmn2r99	0,00
Spryd4	0	Vmn2r72	0	Trmt13	0	Vmn2r98	0,00
Spryd3	0	Vmn2r71	0	Fam134a	0	Vmn2r96	0,00
Spry4	0	Vmn2r7	0	Ghrhr	0	Vmn2r93	0,00
Spry2	0	Vmn2r68	0	Slc9a4	0	Vmn2r92	0,00
Sprtn	0	Vmn2r67	0	Irak2	0	Vmn2r91	0,00
Sprr4	0	Vmn2r65	0	Irak3	0	Vmn2r88	0,00
Sprr3	0	Vmn2r63	0	Ccdc28b	0	Vmn2r87	0,00
Sprr2k	0	Vmn2r62	0	Irak1	0	Vmn2r86	0,00
Sprr2i	0	Vmn2r60	0	Irak4	0	Vmn2r85	0,00
Sprr2h	0	Vmn2r6	0	Sohlh1	0	Vmn2r83	0,00
Sprr2f	0	Vmn2r57	0	Fam160b2	0	Vmn2r8	0,00
Sprr2a2	0	Vmn2r56	0	Nifk	0	Vmn2r79	0,00
Sprr2a1	0	Vmn2r55	0	Ngdn	0	Vmn2r78	0,00
Sprr1b	0	Vmn2r53	0	Olfr773	0	Vmn2r76	0,00
Sprr1a	0	Vmn2r52	0	Ap4s1	0	Vmn2r74	0,00
Sprn	0	Vmn2r51	0	Chst2	0	Vmn2r73	0,00
Spred2	0	Vmn2r5	0	Olfr770	0	Vmn2r72	0,00
Spred1	0	Vmn2r49	0	Lig4	0	Vmn2r71	0,00
Sppl2c	0	Vmn2r48	0	Prrc2c	0	Vmn2r70	0,00
Sppl2b	0	Vmn2r47	0	Gng4	0	Vmn2r7	0,00
Sppl2a	0	Vmn2r46	0	Gng5	0	Vmn2r68	0,00
Spp2	0	Vmn2r45	0	Lig1	0	Vmn2r66	0,00
Spp1	0	Vmn2r44	0	Dclk1	0	Vmn2r65	0,00
Spopl	0	Vmn2r42	0	9230104L09Rik	0	Vmn2r62	0,00
Spon2	0	Vmn2r41	0	Cln3	0	Vmn2r61	0,00
Spock3	0	Vmn2r40	0	Ankrd28	0	Vmn2r60	0,00
Spock2	0	Vmn2r39	0	Gng8	0	Vmn2r6	0,00
Spock1	0	Vmn2r38	0	BRDN0000738249	0	Vmn2r59	0,00
Spo11	0	Vmn2r37	0	Ccdc178	0	Vmn2r58	0,00
Spn	0	Vmn2r36	0	Tspyl5	0	Vmn2r56	0,00
Spire2	0	Vmn2r34	0	Pknox2	0	Vmn2r53	0,00
Spire1	0	Vmn2r32	0	Vps26a	0	Vmn2r52	0,00
Spint5	0	Vmn2r31	0	Tspyl2	0	Vmn2r49	0,00
Spint3	0	Vmn2r29	0	Vps26b	0	Vmn2r48	0,00
Spint2	0	Vmn2r28	0	Ccdc170	0	Vmn2r47	0,00
Spint1	0	Vmn2r27	0	Ccdc171	0	Vmn2r46	0,00
Spinkl	0	Vmn2r26	0	Ccdc172	0	Vmn2r45	0,00
Spink8	0	Vmn2r25	0	Kdelc2	0	Vmn2r44	0,00
Spink7	0	Vmn2r24	0	Ccdc174	0	Vmn2r42	0,00
Spink6	0	Vmn2r22	0	Ccdc175	0	Vmn2r41	0,00
Spink5	0	Vmn2r21	0	Ccdc176	0	Vmn2r40	0,00
Spink4	0	Vmn2r18	0	Tmem183a	0	Vmn2r39	0,00
Spink3	0	Vmn2r17	0	Rhox4f	0	Vmn2r37	0,00
Spink2	0	Vmn2r16	0	Gpd1	0	Vmn2r36	0,00
Spink13	0	Vmn2r15	0	Gpd2	0	Vmn2r35	0,00
Spink12	0	Vmn2r13	0	Prss51	0	Vmn2r34	0,00
Spink10	0	Vmn2r124	0	BRDN0000737942	0	Vmn2r33	0,00
Spin4	0	Vmn2r120	0	Olfr328	0	Vmn2r31	0,00
Spin2d	0	Vmn2r118	0	Gm7903	0	Vmn2r30	0,00
Spin2c	0	Vmn2r117	0	Vrk1	0	Vmn2r3	0,00
Spin1	0	Vmn2r116	0	Klrd1	0	Vmn2r29	0,00
Spice1	0	Vmn2r115	0	Plbd1	0	Vmn2r28	0,00
Spic	0	Vmn2r113	0	Nol11	0	Vmn2r27	0,00
Spib	0	Vmn2r112	0	Dip2c	0	Vmn2r25	0,00
Spi1	0	Vmn2r111	0	Olfr38	0	Vmn2r24	0,00
Sphkap	0	Vmn2r110	0	BRDN0000738241	0	Vmn2r23	0,00
Sphk2	0	Vmn2r109	0	Cars2	0	Vmn2r22	0,00
Sphk1	0	Vmn2r108	0	Cdk1	0	Vmn2r2	0,00
Spg7	0	Vmn2r107	0	Cdk2	0	Vmn2r19	0,00
Spg21	0	Vmn2r105	0	Cdk4	0	Vmn2r18	0,00

Spg20	0	Vmn2r104	0	Sacm1l	0	Vmn2r17	0,00
Spesp1	0	Vmn2r103	0	Cdk6	0	Vmn2r16	0,00
Spert	0	Vmn2r102	0	Cdk7	0	Vmn2r15	0,00
Spen	0	Vmn2r100	0	Cdk8	0	Vmn2r14	0,00
Spem1	0	Vmn2r1	0	Cdk9	0	Vmn2r120	0,00
Speg	0	Vmn1r95	0	Lrrc4	0	Vmn2r12	0,00
Speer4e	0	Vmn1r94	0	Pea15a	0	Vmn2r118	0,00
Speer4d	0	Vmn1r91	0	Clasrp	0	Vmn2r116	0,00
Speer4c	0	Vmn1r89	0	Srek1	0	Vmn2r114	0,00
Speer4b	0	Vmn1r88	0	Tlr3	0	Vmn2r113	0,00
Speer3	0	Vmn1r85	0	Morn4	0	Vmn2r112	0,00
Speer2	0	Vmn1r84	0	Cst10	0	Vmn2r111	0,00
Specc1l	0	Vmn1r82	0	Cst11	0	Vmn2r110	0,00
Spdyb	0	Vmn1r80	0	Cst12	0	Vmn2r107	0,00
Spdya	0	Vmn1r76	0	Cst13	0	Vmn2r106	0,00
Spdl1	0	Vmn1r74	0	Tcf7	0	Vmn2r105	0,00
Spc24	0	Vmn1r73	0	2310057N15Rik	0	Vmn2r104	0,00
Spats2l	0	Vmn1r72	0	Aup1	0	Vmn2r103	0,00
Spats1	0	Vmn1r70	0	Olfr33	0	Vmn2r102	0,00
Spatc1	0	Vmn1r7	0	Siglech	0	Vmn2r101	0,00
Spata9	0	Vmn1r69	0	Cdc34	0	Vmn2r100	0,00
Spata6	0	Vmn1r67	0	Psmc2	0	Vmn2r10	0,00
Spata45	0	Vmn1r66	0	Bet1l	0	Vmn1r95	0,00
Spata4	0	Vmn1r65	0	Rtn1	0	Vmn1r94	0,00
Spata33	0	Vmn1r64	0	Fut11	0	Vmn1r9	0,00
Spata32	0	Vmn1r62	0	Mxd4	0	Vmn1r88	0,00
Spata31d1c	0	Vmn1r61	0	Ppp3r2	0	Vmn1r87	0,00
Spata31d1b	0	Vmn1r60	0	Ppp3r1	0	Vmn1r86	0,00
Spata31	0	Vmn1r6	0	Suv39h1	0	Vmn1r85	0,00
Spata3	0	Vmn1r56	0	Pde4d	0	Vmn1r84	0,00
Spata2l	0	Vmn1r55	0	Suv39h2	0	Vmn1r82	0,00
Spata22	0	Vmn1r54	0	Saal1	0	Vmn1r81	0,00
Spata21	0	Vmn1r52	0	Hcn1	0	Vmn1r78	0,00
Spata20	0	Vmn1r51	0	Psmc6	0	Vmn1r77	0,00
Spata18	0	Vmn1r50	0	Olfr823	0	Vmn1r76	0,00
Spata17	0	Vmn1r49	0	Sppl2a	0	Vmn1r75	0,00
Spata16	0	Vmn1r48	0	Sppl2c	0	Vmn1r74	0,00
Spata13	0	Vmn1r47	0	Papolg	0	Vmn1r73	0,00
Spata1	0	Vmn1r46	0	Ppp1r14b	0	Vmn1r72	0,00
Spast	0	Vmn1r45	0	Fam184a	0	Vmn1r71	0,00
Sparcl1	0	Vmn1r44	0	Fam184b	0	Vmn1r70	0,00
Sparc	0	Vmn1r43	0	Kcp	0	Vmn1r7	0,00
Spam1	0	Vmn1r42	0	Vasp	0	Vmn1r69	0,00
Spag9	0	Vmn1r40	0	S100a1	0	Vmn1r68	0,00
Spag8	0	Vmn1r4	0	Olfr821	0	Vmn1r67	0,00
Spag7	0	Vmn1r39	0	Smc6	0	Vmn1r66	0,00
Spag6l	0	Vmn1r37	0	Slc16a5	0	Vmn1r65	0,00
Spag6	0	Vmn1r36	0	Arih2	0	Vmn1r64	0,00
Spag5	0	Vmn1r35	0	Slc16a4	0	Vmn1r63	0,00
Spag4	0	Vmn1r34	0	Zfp608	0	Vmn1r62	0,00
Spag17	0	Vmn1r33	0	Zfp609	0	Vmn1r61	0,00
Spag16	0	Vmn1r31	0	Olfr804	0	Vmn1r60	0,00
Spag11b	0	Vmn1r30	0	Slc16a7	0	Vmn1r59	0,00
Spag1	0	Vmn1r28	0	Ndnl2	0	Vmn1r57	0,00
Spaca7	0	Vmn1r27	0	Zfp600	0	Vmn1r56	0,00
Spaca6	0	Vmn1r26	0	Slc16a6	0	Vmn1r55	0,00
Spaca3	0	Vmn1r24	0	Zfp605	0	Vmn1r53	0,00
Spa17	0	Vmn1r238	0	Zfp607	0	Vmn1r50	0,00
Sp9	0	Vmn1r237	0	Foxi2	0	Vmn1r5	0,00
Sp7	0	Vmn1r236	0	Tmem160	0	Vmn1r49	0,00
Sp2	0	Vmn1r235	0	Tyw1	0	Vmn1r48	0,00
Sp140	0	Vmn1r233	0	Xrra1	0	Vmn1r47	0,00
Sp110	0	Vmn1r232	0	Ppapdc1b	0	Vmn1r46	0
Sp100	0	Vmn1r231	0	Ppapdc1a	0	Vmn1r45	0
Sp1	0	Vmn1r230	0	Bub1	0	Vmn1r43	0
Sox9	0	Vmn1r23	0	Prkar1a	0	Vmn1r42	0
Sox8	0	Vmn1r228	0	Prkar1b	0	Vmn1r41	0
Sox6	0	Vmn1r226	0	Camk2n2	0	Vmn1r40	0
Sox5	0	Vmn1r225	0	Cyp2c40	0	Vmn1r4	0
Sox4	0	Vmn1r224	0	Wwp1	0	Vmn1r38	0
Sox3	0	Vmn1r223	0	Rnase4	0	Vmn1r37	0
Sox21	0	Vmn1r221	0	Ttll12	0	Vmn1r36	0
Sox2	0	Vmn1r22	0	Hcn2	0	Vmn1r35	0
Sox18	0	Vmn1r219	0	Ogfrl1	0	Vmn1r34	0
Sox17	0	Vmn1r217	0	4931423N10Rik	0	Vmn1r31	0
Sox15	0	Vmn1r215	0	Usp29	0	Vmn1r3	0
Sox12	0	Vmn1r214	0	Usp28	0	Vmn1r29	0
Sox10	0	Vmn1r213	0	Usp26	0	Vmn1r26	0
Sox1	0	Vmn1r212	0	Usp25	0	Vmn1r25	0
Sowahd	0	Vmn1r21	0	Zbtb20	0	Vmn1r24	0
Sowahc	0	Vmn1r208	0	Usp21	0	Vmn1r238	0
Sowahb	0	Vmn1r206	0	4931428F04Rik	0	Vmn1r236	0

Sostdc1	0
Sost	0
Sos1	0
Sort1	0
Sord	0
Sorcs2	0
Sorbs3	0
Sorbs2	0
Sorbs1	0
Sohlh2	0
Sohlh1	0
Soga3	0
Sod3	0
Sod1	0
Socs7	0
Socs6	0
Socs4	0
Socs3	0
Socs2	0
Socs1	0
Sobp	0
Soat1	0
Snx9	0
Snx8	0
Snx7	0
Snx6	0
Snx5	0
Snx32	0
Snx31	0
Snx3	0
Snx29	0
Snx25	0
Snx24	0
Snx22	0
Snx21	0
Snx2	0
Snx19	0
Snx17	0
Snx16	0
Snx15	0
Snx13	0
Snx12	0
Snx10	0
Snx1	0
Snw1	0
Snurf	0
Snupn	0
Sntg2	0
Sntg1	0
Sntb2	0
Sntb1	0
Snta1	0
Snrpn	0
Snrpg	0
Snrpf	0
Snrpe	0
Snrpd3	0
Snrpd2	0
Snrpd1	0
Snrpb2	0
Snrpb	0
Snrpa1	0
Snrpa	0
Snrnp70	0
Snrnp48	0
Snrnp40	0
Snrnp35	0
Snrnp27	0
Snrnp25	0
Snrnp200	0
Snph	0
Snip1	0
Snhg11	0
Snf8	0
Sned1	0
Snd1	0
Sncg	0
Sncaip	0
Snapin	0
Snapc4	0
Snapc2	0
Snap91	0
Vmn1r205	0
Vmn1r204	0
Vmn1r203	0
Vmn1r202	0
Vmn1r201	0
Vmn1r200	0
Vmn1r20	0
Vmn1r2	0
Vmn1r199	0
Vmn1r197	0
Vmn1r195	0
Vmn1r194	0
Vmn1r193	0
Vmn1r192	0
Vmn1r191	0
Vmn1r19	0
Vmn1r184	0
Vmn1r183	0
Vmn1r181	0
Vmn1r18	0
Vmn1r179	0
Vmn1r178	0
Vmn1r176	0
Vmn1r175	0
Vmn1r174	0
Vmn1r172	0
Vmn1r171	0
Vmn1r17	0
Vmn1r169	0
Vmn1r168	0
Vmn1r163	0
Vmn1r16	0
Vmn1r159	0
Vmn1r151	0
Vmn1r149	0
Vmn1r148	0
Vmn1r143	0
Vmn1r142	0
Vmn1r14	0
Vmn1r139	0
Vmn1r138	0
Vmn1r137	0
Vmn1r135	0
Vmn1r132	0
Vmn1r131	0
Vmn1r13	0
Vmn1r129	0
Vmn1r128	0
Vmn1r126	0
Vmn1r124	0
Vmn1r123	0
Vmn1r122	0
Vmn1r121	0
Vmn1r120	0
Vmn1r12	0
Vmn1r119	0
Vmn1r116	0
Vmn1r114	0
Vmn1r113	0
Vmn1r112	0
Vmn1r11	0
Vmn1r107	0
Vmn1r104	0
Vmn1r101	0
Vmn1r100	0
Vmn1r10	0
Vmn1r1	0
Vmac	0
Vma21	0
Vkorc1l1	0
Vkorc1	0
Vit	0
Vipr2	0
Vipas39	0
Vimp	0
Vil1	0
Vhl	0
Vgll4	0
Vgll2	0
Vgll1	0
Vgf	0
Vezf1	0
Ccndbp1	0
4930432K21Rik	0
Them6	0
Ugt2b1	0
BRDN0000738044	0
Ddt	0
Dstn	0
Naprt	0
Hils1	0
Gm1993	0
Ctns	0
Atxn2l	0
Parm1	0
Olfr1532-ps1	0
Prpf39	0
Lmtk3	0
9830147E19Rik	0
Avl9	0
Hcn4	0
Prpf31	0
Topaz1	0
Alas1	0
Alas2	0
4930568D16Rik	0
Htr3b	0
Ednra	0
Brwd1	0
V1rd19	0
Scn2b	0
Hmgb3	0
Mina	0
Hmgb1	0
Ssc5d	0
Fam84b	0
Tmem254a	0
Tmem254b	0
Ramp2	0
Tigar	0
Slc5a1	0
Slc5a2	0
Syt11	0
Adamts15	0
Slc5a6	0
Syt15	0
Slc5a8	0
Adamts18	0
Rita1	0
Zcchc4	0
1700006A11Rik	0
Clec10a	0
Renbp	0
Ttyh2	0
Nrcam	0
Otud3	0
Caskin1	0
Skap2	0
Ptgfr	0
Thpo	0
Sprr2i	0
Sprr2h	0
Sprr2k	0
Sp110	0
Sprr2d	0
5430427O19Rik	0
Slc19a2	0
Ubald1	0
Ubald2	0
Slc19a1	0
Chic1	0
Clec2h	0
Gm10665	0
Man2c1	0
Clec2l	0
Styk1	0
Gm10662	0
Calcoco2	0
2310057M21Rik	0
Clec2e	0
Ephb2	0
Sval3	0
Tmem55a	0
Tmem55b	0
Vmn1r235	0
Vmn1r234	0
Vmn1r233	0
Vmn1r232	0
Vmn1r230	0
Vmn1r229	0
Vmn1r228	0
Vmn1r226	0
Vmn1r225	0
Vmn1r224	0
Vmn1r223	0
Vmn1r221	0
Vmn1r22	0
Vmn1r218	0
Vmn1r217	0
Vmn1r214	0
Vmn1r212	0
Vmn1r211	0
Vmn1r210	0
Vmn1r21	0
Vmn1r209	0
Vmn1r208	0
Vmn1r206	0
Vmn1r205	0
Vmn1r204	0
Vmn1r203	0
Vmn1r202	0
Vmn1r201	0
Vmn1r199	0
Vmn1r198	0
Vmn1r195	0
Vmn1r194	0
Vmn1r193	0
Vmn1r192	0
Vmn1r191	0
Vmn1r188	0
Vmn1r185	0
Vmn1r184	0
Vmn1r183	0
Vmn1r180	0
Vmn1r179	0
Vmn1r174	0
Vmn1r173	0
Vmn1r171	0
Vmn1r170	0
Vmn1r17	0
Vmn1r169	0
Vmn1r168	0
Vmn1r167	0
Vmn1r163	0
Vmn1r160	0
Vmn1r16	0
Vmn1r158	0
Vmn1r157	0
Vmn1r151	0
Vmn1r15	0
Vmn1r149	0
Vmn1r143	0
Vmn1r142	0
Vmn1r14	0
Vmn1r139	0
Vmn1r138	0
Vmn1r135	0
Vmn1r132	0
Vmn1r131	0
Vmn1r13	0
Vmn1r129	0
Vmn1r126	0
Vmn1r125	0
Vmn1r124	0
Vmn1r123	0
Vmn1r122	0
Vmn1r121	0
Vmn1r119	0
Vmn1r117	0
Vmn1r115	0
Vmn1r114	0
Vmn1r112	0
Vmn1r11	0
Vmn1r103	0
Vmn1r101	0
Vmn1r1	0

Snap47	0	Veph1	0	Gal	0	Vma21	0
Snap29	0	Vegfc	0	Gak	0	Vldlr	0
Snap25	0	Vegfb	0	Selt	0	Vkorc1	0
Snap23	0	Vegfa	0	Cited2	0	Vit	0
Snai2	0	Vdr	0	Scgb1b20	0	Vipr2	0
Snai1	0	Vdac3	0	Rusc1	0	Vipr1	0
Smyd4	0	Vdac1	0	Scgb1b27	0	Vipas39	0
Smyd3	0	Vcpkmt	0	Gaa	0	Vimp	0
Smyd1	0	Vcpip1	0	Mta3	0	Vim	0
Smurf1	0	Vcp	0	BRDN0000738045	0	Vill	0
Smug1	0	Vcl	0	Trh	0	Vil1	0
Smu1	0	Vcan	0	Nceh1	0	Vhl	0
Smtnl2	0	Vcam1	0	Ehd4	0	Vgll4	0
Smtnl1	0	Vbp1	0	Ehd3	0	Vgll3	0
Smtn	0	Vav1	0	Zfp952	0	Vgll2	0
Sms	0	Vasp	0	Tro	0	Vgf	0
Smr3a	0	Vasn	0	Fam166a	0	Vegfc	0
Smpx	0	Vash1	0	Fam166b	0	Vegfb	0
Smpdl3b	0	Vars	0	Ppwd1	0	Vegfa	0
Smpd4	0	Vapb	0	Zc2hc1b	0	Vdr	0
Smpd3	0	Vapa	0	Zc2hc1c	0	Vcpkmt	0
Smpd2	0	Vangl2	0	Zc2hc1a	0	Vcpip1	0
Smpd1	0	Vangl1	0	Slc35a4	0	Vcp	0
Smox	0	Vamp3	0	Trpa1	0	Vcl	0
Smok3b	0	Vamp2	0	BRDN0000737690	0	Vax1	0
Smok2a	0	Vamp1	0	BRDN0000737691	0	Vav1	0
Smoc2	0	V1rd19	0	BRDN0000737696	0	Vat1	0
Smo	0	V1ra8	0	Prkd1	0	Vasn	0
Smndc1	0	Uxt	0	Abl1	0	Vash2	0
Smn1	0	Uvssa	0	Atxn7	0	Vash1	0
Smlr1	0	Uts2r	0	BRDN0000737698	0	Vars2	0
Smim8	0	Uts2b	0	Abl2	0	Vars	0
Smim7	0	Utp6	0	Atxn2	0	Vapb	0
Smim6	0	Utp23	0	Atxn3	0	Vapa	0
Smim3	0	Utp20	0	Atxn1	0	Vangl2	0
Smim24	0	Utp15	0	Slc6a3	0	Vangl1	0
Smim23	0	Utp14b	0	Slc6a1	0	Vamp8	0
Smim22	0	Utp14a	0	Plscr5	0	Vamp5	0
Smim20	0	Utp11l	0	Slc6a7	0	Vamp2	0
Smim19	0	Utf1	0	Slc6a6	0	Vamp1	0
Smim15	0	Uspl1	0	Slc6a5	0	Vac14	0
Smim14	0	Usp9x	0	Slc6a4	0	V1rd19	0
Smim13	0	Usp8	0	Vmn1r12	0	V1ra8	0
Smim12	0	Usp7	0	Vmn1r13	0	Uxt	0
Smim11	0	Usp54	0	Vmn1r16	0	Uxs1	0
Smim1	0	Usp51	0	Vmn1r17	0	Uvssa	0
Smg9	0	Usp49	0	Zfp959	0	Uvrag	0
Smg8	0	Usp47	0	Olfr181	0	Uty	0
Smg6	0	Usp46	0	Ppm1g	0	Utp6	0
Smg5	0	Usp45	0	Fthl17	0	Utp23	0
Smg1	0	Usp44	0	Olfr180	0	Utp20	0
Smek1	0	Usp43	0	Dclk2	0	Utp18	0
Smcr8	0	Usp42	0	Rpgrip1l	0	Utp15	0
Smco3	0	Usp40	0	Gphn	0	Utp14b	0
Smco2	0	Usp4	0	Mtap	0	Utp14a	0
Smco1	0	Usp39	0	Cd3e	0	Utf1	0
Smc6	0	Usp37	0	Pfpl	0	Ust	0
Smc5	0	Usp36	0	Col17a1	0	Uspl1	0
Smc4	0	Usp35	0	Bcap31	0	Usp9y	0
Smc3	0	Usp34	0	Olfr610	0	Usp9x	0
Smc2	0	Usp33	0	Olfr613	0	Usp8	0
Smc1b	0	Usp32	0	Eml1	0	Usp7	0
Smc1a	0	Usp31	0	Eef1g	0	Usp54	0
Smarce1	0	Usp30	0	Fam76b	0	Usp53	0
Smarcd3	0	Usp3	0	Plac8	0	Usp51	0
Smarcd1	0	Usp29	0	Nup160	0	Usp50	0
Smarcc2	0	Usp28	0	Clip4	0	Usp5	0
Smarcb1	0	Usp26	0	BRDN0000737520	0	Usp47	0
Smarcal1	0	Usp25	0	BRDN0000737521	0	Usp45	0
Smarca5	0	Usp22	0	BRDN0000738343	0	Usp43	0
Smarca4	0	Usp21	0	1700001C02Rik	0	Usp42	0
Smarca2	0	Usp20	0	Bivm	0	Usp4	0
Smarca1	0	Usp19	0	Elac2	0	Usp39	0
Smap2	0	Usp17lb	0	Ccdc137	0	Usp38	0
Smad9	0	Usp17la	0	Ubxn8	0	Usp37	0
Smad7	0	Usp16	0	Olfr187	0	Usp36	0
Smad6	0	Usp15	0	Ankhd1	0	Usp35	0
Smad5	0	Usp12	0	Ubxn4	0	Usp34	0
Smad3	0	Uso1	0	Lrtm1	0	Usp33	0
Smad1	0	Usmg5	0	Ubxn6	0	Usp31	0
Sly	0	Ushbp1	0	Olfr186	0	Usp30	0
Slxl1	0	Ush2a	0	Gtpbp8	0	Usp3	0

Slx4ip	0	Ush1g	0	Ubxn1	0	Usp29	0
Slx4	0	Ush1c	0	Olfr609	0	Usp28	0
Slx1b	0	Usf2	0	Xkr5	0	Usp25	0
Slx	0	Use1	0	Xkr4	0	Usp24	0
Slu7	0	Usb1	0	Ttpal	0	Usp20	0
Sltm	0	Urod	0	Pibf1	0	Usp19	0
Slpi	0	Uroc1	0	Filip1l	0	Usp17ld	0
Sln	0	Urm1	0	H2-Ke6	0	Usp17lc	0
Slmo2	0	Uri1	0	Fstl3	0	Usp17lb	0
Slmo1	0	Urgcp	0	Xkr9	0	Usp17la	0
Slmap	0	Urb2	0	Cpb2	0	Usp16	0
Slk	0	Urb1	0	Lep	0	Usp15	0
Slitrk6	0	Urah	0	Dis3	0	Usp14	0
Slitrk4	0	Urad	0	Rint1	0	Usp13	0
Slitrk3	0	Uqcrq	0	Nin	0	Usp12	0
Slitrk2	0	Uqcrh	0	Rprm	0	Usp10	0
Slit3	0	Uqcrfs1	0	Grcc10	0	Usp1	0
Slit1	0	Uqcrc2	0	Atp5o	0	Uso1	0
Slirp	0	Uqcrc1	0	Zfp185	0	Ushbp1	0
Slfnl1	0	Uqcrb	0	Zfp184	0	Ush2a	0
Slfn8	0	Uqcr11	0	4931414P19Rik	0	Ush1g	0
Slfn5	0	Uqcr10	0	Setdb2	0	Ush1c	0
Slfn4	0	Uqcc3	0	4930523C07Rik	0	Use1	0
Slfn3	0	Uqcc2	0	Grap	0	Usb1	0
Slfn14	0	Uqcc1	0	Lctl	0	Uros	0
Slfn1	0	Uprt	0	Hax1	0	Urod	0
Slco4c1	0	Upp2	0	Rdx	0	Uroc1	0
Slco4a1	0	Upp1	0	Gkap1	0	Urm1	0
Slco3a1	0	Upk3bl	0	Pirb	0	Uri1	0
Slco2b1	0	Upk2	0	Vmn2r100	0	Urb2	0
Slco2a1	0	Upk1b	0	Banf1	0	Urb1	0
Slco1c1	0	Upk1a	0	Vmn2r102	0	Urah	0
Slco1b2	0	Upf3b	0	Olfr1009	0	Uqcrq	0
Slco1a4	0	Upf2	0	Vmn2r104	0	Uqcrc2	0
Slco1a1	0	Upf1	0	Vmn2r105	0	Uqcrc1	0
Slc9c1	0	Uox	0	Vmn2r106	0	Uqcrb	0
Slc9b1	0	Unkl	0	Vmn2r107	0	Uqcr11	0
Slc9a9	0	Ung	0	Olfr1002	0	Uqcr10	0
Slc9a7	0	Unc93b1	0	Vmn2r109	0	Uqcc2	0
Slc9a6	0	Unc93a	0	Olfr1000	0	Uqcc1	0
Slc9a4	0	Unc80	0	Cyp4v3	0	Uprt	0
Slc9a3r2	0	Unc79	0	Olfr784	0	Upk3b	0
Slc9a3r1	0	Unc5cl	0	Gusb	0	Upk2	0
Slc9a3	0	Unc5c	0	Olfr933	0	Upk1a	0
Slc9a2	0	Unc5b	0	Rnase11	0	Upf3b	0
Slc8b1	0	Unc5a	0	Fscb	0	Upf3a	0
Slc8a3	0	Unc50	0	Rnase13	0	Upf2	0
Slc8a2	0	Unc45b	0	Rnase12	0	Upf1	0
Slc8a1	0	Unc45a	0	Bhlhe40	0	Upb1	0
Slc7a9	0	Unc13d	0	Krtap9-1	0	Uox	0
Slc7a8	0	Unc13c	0	Krtap9-3	0	Unk	0
Slc7a7	0	Unc13b	0	Xkrx	0	Ung	0
Slc7a6os	0	Unc13a	0	Olfr550	0	Uncx	0
Slc7a6	0	Unc119b	0	Gjd2	0	Unc93a	0
Slc7a5	0	Umps	0	Megf9	0	Unc80	0
Slc7a4	0	Umodl1	0	Tmsb15a	0	Unc5d	0
Slc7a3	0	Umod	0	Psca	0	Unc45a	0
Slc7a2	0	Ulbp1	0	Rtn4rl1	0	Unc13a	0
Slc7a15	0	Uimc1	0	2210016F16Rik	0	Unc119b	0
Slc7a14	0	Uhrf1bp1l	0	Rtn4rl2	0	Umodl1	0
Slc7a13	0	Uhrf1bp1	0	Gm8720	0	Ulk4	0
Slc7a11	0	Uhrf1	0	Tnfaip8l3	0	Ulk3	0
Slc7a10	0	Ugt3a2	0	Tnfaip8l2	0	Ulk2	0
Slc7a1	0	Ugt2b38	0	Tnfaip8l1	0	Uhrf2	0
Slc6a9	0	Ugt2b37	0	Cyp4a12a	0	Uhrf1	0
Slc6a7	0	Ugt2b36	0	Fbxw8	0	Ugt8a	0
Slc6a6	0	Ugt2b34	0	Myoz2	0	Ugt2b38	0
Slc6a5	0	Ugt2b1	0	Ddost	0	Ugt2b36	0
Slc6a4	0	Ugt1a9	0	Klhl38	0	Ugt2b35	0
Slc6a3	0	Ugt1a6b	0	Slc1a5	0	Ugt2a3	0
Slc6a20b	0	Ugt1a6a	0	Ankib1	0	Ugt1a9	0
Slc6a20a	0	Ugt1a10	0	Lpar3	0	Ugt1a7c	0
Slc6a2	0	Ugt1a1	0	Fbxw2	0	Ugt1a6b	0
Slc6a19	0	Ugp2	0	Lpar1	0	Ugt1a6a	0
Slc6a18	0	Ugdh	0	Lpar6	0	Ugt1a5	0
Slc6a17	0	Ugcg	0	Fbxw5	0	Ugt1a10	0
Slc6a15	0	Ufsp2	0	Lpar4	0	Ugp2	0
Slc6a13	0	Ufsp1	0	Lpar5	0	Uggt1	0
Slc6a12	0	Ufm1	0	Hus1b	0	Ugcg	0
Slc6a11	0	Ufd1l	0	Myoz1	0	Ufsp2	0
Slc6a1	0	Uevld	0	Pard6g	0	Ufsp1	0
Slc5a9	0	Ucp3	0	Naa40	0	Ufl1	0

Slc5a8	0
Slc5a7	0
Slc5a6	0
Slc5a4b	0
Slc5a4a	0
Slc5a3	0
Slc5a2	0
Slc5a12	0
Slc5a11	0
Slc5a10	0
Slc52a3	0
Slc52a2	0
Slc51a	0
Slc50a1	0
Slc4a9	0
Slc4a8	0
Slc4a7	0
Slc4a5	0
Slc4a3	0
Slc4a2	0
Slc4a1ap	0
Slc4a1	0
Slc48a1	0
Slc47a2	0
Slc47a1	0
Slc46a3	0
Slc46a2	0
Slc45a3	0
Slc45a2	0
Slc45a1	0
Slc44a5	0
Slc44a4	0
Slc44a3	0
Slc44a2	0
Slc44a1	0
Slc43a2	0
Slc43a1	0
Slc41a2	0
Slc40a1	0
Slc3a2	0
Slc3a1	0
Slc39a9	0
Slc39a8	0
Slc39a7	0
Slc39a6	0
Slc39a5	0
Slc39a4	0
Slc39a3	0
Slc39a2	0
Slc39a14	0
Slc39a13	0
Slc39a12	0
Slc39a11	0
Slc39a1	0
Slc38a9	0
Slc38a8	0
Slc38a7	0
Slc38a6	0
Slc38a3	0
Slc38a11	0
Slc38a10	0
Slc38a1	0
Slc37a4	0
Slc37a3	0
Slc37a2	0
Slc37a1	0
Slc36a4	0
Slc36a1	0
Slc35g2	0
Slc35g1	0
Slc35f5	0
Slc35f4	0
Slc35f2	0
Slc35f1	0
Slc35e4	0
Slc35e3	0
Slc35e2	0
Slc35e1	0
Slc35d3	0
Slc35d2	0
Slc35d1	0
Slc35c2	0
Ucp2	0
Ucp1	0
Ucn3	0
Ucn2	0
Ucn	0
Uckl1	0
Uck2	0
Uchl5	0
Uchl4	0
Uchl1	0
Ubxn7	0
Ubxn4	0
Ubxn2b	0
Ubxn2a	0
Ubxn11	0
Ubxn10	0
Ubtd1	0
Ubr7	0
Ubr4	0
Ubr3	0
Ubr2	0
Ubr1	0
Ubqlnl	0
Ubqln4	0
Ubqln3	0
Ubqln2	0
Ubn2	0
Ubn1	0
Ublcp1	0
Ubl7	0
Ubl5	0
Ubl4	0
Ubiad1	0
Ube4b	0
Ube3b	0
Ube3a	0
Ube2v1	0
Ube2u	0
Ube2r2	0
Ube2q2	0
Ube2q1	0
Ube2o	0
Ube2n	0
Ube2m	0
Ube2l6	0
Ube2l3	0
Ube2k	0
Ube2j2	0
Ube2j1	0
Ube2i	0
Ube2h	0
Ube2g2	0
Ube2g1	0
Ube2f	0
Ube2e3	0
Ube2e2	0
Ube2e1	0
Ube2dnl2	0
Ube2dnl1	0
Ube2d2b	0
Ube2d2a	0
Ube2d1	0
Ube2cbp	0
Ube2c	0
Ube2b	0
Ube2a	0
Ubc	0
Ubb	0
Ubash3b	0
Ubap2l	0
Ubap1l	0
Ubap1	0
Ubald2	0
Ubac2	0
Ubac1	0
Uba52	0
Uba5	0
Uba2	0
Uba1y	0
Uba1	0
Uaca	0
U2surp	0
Ntn4	0
Ntn5	0
Ccrl2	0
Col4a6	0
Col4a5	0
Nfkbib	0
Col4a3	0
Col4a2	0
Fbxl8	0
Fam84a	0
Prrc2a	0
Smim23	0
Smim20	0
Vwc2l	0
Synrg	0
Smim24	0
Arl4a	0
Slc18b1	0
H2-DMb2	0
Gldn	0
Gldc	0
U2surp	0
B3gat3	0
Gm1564	0
Nlrc5	0
St7l	0
Hp1bp3	0
Ahcy	0
Tlr7	0
Hdac7	0
Rpl14	0
Olfr1137	0
Tubb4b	0
Tubb4a	0
Afmid	0
Olfr1134	0
Ap3b2	0
5830411N06Rik	0
Cmpk2	0
Olfr1135	0
Pf4	0
Tomm22	0
Irx5	0
Rpl10	0
Ap1b1	0
Prl3d3	0
Prl3d2	0
Olfr1133	0
Aoc3	0
Aoc2	0
Rpl12	0
Rab33b	0
Azgp1	0
Olfr1131	0
Mageb16	0
Map3k11	0
Map3k12	0
Map3k13	0
Map3k14	0
Galnt9	0
Fam155a	0
Galnt4	0
Ell3	0
Galnt6	0
Galnt7	0
Nrp1	0
Nrp2	0
Hydin	0
Plxnb1	0
Ncbp1	0
Ncbp2	0
Tas2r144	0
Nars2	0
Slc9a3r1	0
Pcdhb6	0
Npb	0
Shisa4	0
Shisa5	0
Shisa6	0
Shisa7	0
Npy	0
Shisa2	0
Ufd1l	0
Ufc1	0
Uevld	0
Ucp2	0
Ucn3	0
Ucn2	0
Ucma	0
Uckl1	0
Uck2	0
Uchl5	0
Uchl4	0
Uchl3	0
Ubxn8	0
Ubxn7	0
Ubxn6	0
Ubxn4	0
Ubxn2b	0
Ubxn2a	0
Ubxn11	0
Ubxn1	0
Ubtfl1	0
Ubtf	0
Ubtd2	0
Ubtd1	0
Ubr5	0
Ubr4	0
Ubr3	0
Ubr2	0
Ubr1	0
Ubqlnl	0
Ubqln4	0
Ubqln3	0
Ubp1	0
Ubox5	0
Ubn2	0
Ubn1	0
Ublcp1	0
Ubl5	0
Ubl4b	0
Ubl4	0
Ubl3	0
Ubiad1	0
Ubfd1	0
Ube4b	0
Ube4a	0
Ube3c	0
Ube3b	0
Ube2v1	0
Ube2u	0
Ube2t	0
Ube2r2	0
Ube2q2	0
Ube2q1	0
Ube2o	0
Ube2m	0
Ube2l6	0
Ube2l3	0
Ube2k	0
Ube2j2	0
Ube2j1	0
Ube2h	0
Ube2g2	0
Ube2g1	0
Ube2f	0
Ube2e3	0
Ube2e2	0
Ube2e1	0
Ube2d2b	0
Ube2d2a	0
Ube2d1	0
Ube2cbp	0
Ube2b	0
Ubb	0
Ubash3b	0
Ubash3a	0
Ubap2l	0
Ubap2	0
Ubap1l	0
Ubald1	0
Ubac1	0
Uba6	0
Uba52	0

Slc35c1	0	U2af2	0	Ido1	0	Uba5	0
Slc35b4	0	U2af1l4	0	Npw	0	Uba3	0
Slc35b3	0	U2af1	0	Grin3b	0	Uba2	0
Slc35b2	0	Tyw5	0	Hpdl	0	Uba1y	0
Slc35b1	0	Tyw3	0	Nps	0	Uba1	0
Slc35a5	0	Tyw1	0	Kif3a	0	Uaca	0
Slc35a4	0	Tysnd1	0	AY358078	0	U2surp	0
Slc35a3	0	Tyrp1	0	Kazn	0	Tyw5	0
Slc35a2	0	Tyro3	0	Popdc3	0	Tyw3	0
Slc35a1	0	Tyr	0	Rsph4a	0	Tyrobp	0
Slc34a3	0	Tyms	0	Azin1	0	Tyro3	0
Slc34a1	0	Tyk2	0	Gucy1b3	0	Tyms	0
Slc33a1	0	Txnrd3	0	Ufc1	0	Tyk2	0
Slc31a2	0	Txnrd2	0	Krtap31-1	0	Txnrd2	0
Slc31a1	0	Txnrd1	0	Psmg4	0	Txnl4b	0
Slc30a9	0	Txnl4b	0	Psmg1	0	Txnl1	0
Slc30a8	0	Txnl4a	0	Psmg2	0	Txnip	0
Slc30a6	0	Txnl1	0	Gnrh1	0	Txndc9	0
Slc30a5	0	Txnip	0	Trpv5	0	Txndc8	0
Slc30a4	0	Txndc5	0	Trpv4	0	Txndc2	0
Slc30a2	0	Txndc2	0	Trpv6	0	Txndc17	0
Slc30a10	0	Txndc17	0	Trpv1	0	Txndc12	0
Slc2a7	0	Txndc16	0	BRDN0000737395	0	Txndc11	0
Slc2a5	0	Txndc12	0	Malt1	0	Txn1	0
Slc2a4	0	Txn1	0	Trpv2	0	Txlng	0
Slc2a3	0	Txlng	0	Thsd7b	0	Txlnb	0
Slc2a2	0	Txlnb	0	Arhgap5	0	Txk	0
Slc2a13	0	Txlna	0	Magoh	0	Twsg1	0
Slc2a12	0	Txk	0	BC049352	0	Twist1	0
Slc2a10	0	Twistnb	0	Xpo4	0	Twf2	0
Slc2a1	0	Twist1	0	4931406C07Rik	0	Twf1	0
Slc29a2	0	Twf2	0	Xpo6	0	Tvp23b	0
Slc29a1	0	Tvp23a	0	Hsd17b4	0	Tvp23a	0
Slc28a2	0	Tut1	0	Krtap31-2	0	Tut1	0
Slc28a1	0	Tusc2	0	Dohh	0	Tusc5	0
Slc27a4	0	Tusc1	0	4933405O20Rik	0	Tusc2	0
Slc27a3	0	Tulp3	0	Hist2h2aa1	0	Tusc1	0
Slc27a2	0	Tuft1	0	Ube2g2	0	Tulp4	0
Slc26a9	0	Tufm	0	Kcnh5	0	Tufm	0
Slc26a8	0	Tubgcp5	0	Kcnh4	0	Tubgcp5	0
Slc26a7	0	Tubgcp4	0	Kcnh7	0	Tubgcp4	0
Slc26a5	0	Tubgcp3	0	Prdm15	0	Tubgcp3	0
Slc26a4	0	Tubg1	0	Kcnh1	0	Tubgcp2	0
Slc26a2	0	Tube1	0	Prdm13	0	Tubg2	0
Slc26a11	0	Tubb6	0	Prdm10	0	Tubg1	0
Slc26a1	0	Tubb5	0	Prdm11	0	Tube1	0
Slc25a54	0	Tubb4b	0	Cpne3	0	Tubd1	0
Slc25a51	0	Tubb4a	0	Rpa1	0	Tubb5	0
Slc25a45	0	Tubb3	0	Cpne1	0	Tubb4a	0
Slc25a44	0	Tubb2b	0	9130401M01Rik	0	Tubb3	0
Slc25a42	0	Tubb2a	0	Cpne7	0	Tubb1	0
Slc25a41	0	Tubb1	0	Cpne6	0	Tuba8	0
Slc25a40	0	Tubal3	0	Cpne5	0	Tuba4a	0
Slc25a4	0	Tuba8	0	Cpne4	0	Tuba3b	0
Slc25a38	0	Tuba4a	0	Olfr1325	0	Tuba1c	0
Slc25a37	0	Tuba3b	0	Olfr728	0	Tuba1b	0
Slc25a36	0	Tuba3a	0	Gm16501	0	Tuba1a	0
Slc25a35	0	Tuba1c	0	Cpeb3	0	Tub	0
Slc25a33	0	Tuba1a	0	Tmem191c	0	Ttyh3	0
Slc25a32	0	Tub	0	Lin52	0	Ttyh2	0
Slc25a31	0	Ttpal	0	Fancc	0	Ttr	0
Slc25a30	0	Ttpa	0	BRDN0000738096	0	Ttpal	0
Slc25a29	0	Ttll9	0	BC061212	0	Ttll9	0
Slc25a26	0	Ttll7	0	Iscu	0	Ttll8	0
Slc25a25	0	Ttll6	0	Flt3l	0	Ttll7	0
Slc25a24	0	Ttll5	0	Arhgap28	0	Ttll6	0
Slc25a23	0	Ttll4	0	Fnta	0	Ttll5	0
Slc25a22	0	Ttll3	0	Fntb	0	Ttll4	0
Slc25a20	0	Ttll2	0	Gm3701	0	Ttll3	0
Slc25a2	0	Ttll13	0	Mettl8	0	Ttll2	0
Slc25a19	0	Ttll12	0	Fam134c	0	Ttll13	0
Slc25a18	0	Ttll10	0	Gpha2	0	Ttll11	0
Slc25a17	0	Ttl	0	Zfp473	0	Ttll10	0
Slc25a15	0	Ttk	0	Zfp472	0	Ttll1	0
Slc25a14	0	Tti2	0	Olfr1324	0	Ttl	0
Slc25a13	0	Ttf2	0	Cmtm2a	0	Ttk	0
Slc25a12	0	Ttf1	0	Cmtm2b	0	Tti2	0
Slc25a11	0	Ttc9b	0	Rbm33	0	Ttc9c	0
Slc25a1	0	Ttc7b	0	Cyth1	0	Ttc9	0
Slc24a5	0	Ttc7	0	Sec61g	0	Ttc8	0
Slc24a4	0	Ttc39d	0	Cyth3	0	Ttc7	0
Slc24a3	0	Ttc39c	0	Rbm38	0	Ttc5	0

Slc24a2	0	Ttc39b	0	Sept3	0	Ttc39d	0
Slc24a1	0	Ttc34	0	Sept2	0	Ttc39c	0
Slc23a3	0	Ttc32	0	Akap17b	0	Ttc37	0
Slc23a1	0	Ttc30b	0	Pira1	0	Ttc36	0
Slc22a8	0	Ttc30a2	0	Tet2	0	Ttc34	0
Slc22a7	0	Ttc3	0	Lrrc42	0	Ttc33	0
Slc22a6	0	Ttc29	0	Kmt2b	0	Ttc32	0
Slc22a5	0	Ttc27	0	Sgcg	0	Ttc30a2	0
Slc22a4	0	Ttc26	0	Tacc1	0	Ttc30a1	0
Slc22a30	0	Ttc24	0	Sgce	0	Ttc29	0
Slc22a3	0	Ttc23l	0	Sgcd	0	Ttc28	0
Slc22a29	0	Ttc23	0	Foxo1	0	Ttc27	0
Slc22a28	0	Ttc22	0	Kmt2a	0	Ttc26	0
Slc22a27	0	Ttc19	0	Tecr	0	Ttc25	0
Slc22a26	0	Ttc17	0	Apbb1	0	Ttc24	0
Slc22a23	0	Ttc16	0	4930415F15Rik	0	Ttc23l	0
Slc22a21	0	Ttc14	0	Dctpp1	0	Ttc23	0
Slc22a20	0	Ttc1	0	Sgcz	0	Ttc22	0
Slc22a19	0	Ttbk2	0	Col1a1	0	Ttc21b	0
Slc22a18	0	Ttbk1	0	Pcnxl3	0	Ttc21a	0
Slc22a17	0	Tsx	0	Tmem174	0	Ttc17	0
Slc22a16	0	Tstd3	0	Proser3	0	Ttc14	0
Slc22a15	0	Tstd2	0	Mzf1	0	Ttc12	0
Slc22a14	0	Tstd1	0	Ahcyl2	0	Ttc1	0
Slc22a13	0	Tsta3	0	Cyp4b1	0	Ttbk2	0
Slc22a12	0	Tst	0	Tmem173	0	Tstd2	0
Slc22a1	0	Tssk6	0	Eci3	0	Tstd1	0
Slc20a2	0	Tssk1	0	Eci2	0	Tsta3	0
Slc20a1	0	Tssc4	0	Eci1	0	Tst	0
Slc1a5	0	Tsr1	0	Ceacam19	0	Tssk5	0
Slc1a4	0	Tspyl5	0	Ceacam18	0	Tssk4	0
Slc1a3	0	Tspyl4	0	Ipo8	0	Tssk3	0
Slc1a2	0	Tspyl3	0	Ipo9	0	Tssk1	0
Slc19a2	0	Tspyl2	0	Oprk1	0	Tssc4	0
Slc19a1	0	Tspyl1	0	Ipo7	0	Tssc1	0
Slc18a3	0	Tspo2	0	Ipo4	0	Tsr3	0
Slc18a2	0	Tspan9	0	Ipo5	0	Tsr2	0
Slc17a9	0	Tspan8	0	Ceacam11	0	Tsr1	0
Slc17a8	0	Tspan7	0	Fbxo10	0	Tspyl4	0
Slc17a7	0	Tspan5	0	Ceacam13	0	Tspyl3	0
Slc17a6	0	Tspan4	0	Ceacam12	0	Tspyl2	0
Slc17a4	0	Tspan33	0	Slc12a1	0	Tspyl1	0
Slc17a3	0	Tspan32	0	Fbxo11	0	Tspo2	0
Slc17a2	0	Tspan31	0	Fam212b	0	Tspo	0
Slc17a1	0	Tspan3	0	Yrdc	0	Tspan7	0
Slc16a9	0	Tspan2	0	B4galnt4	0	Tspan4	0
Slc16a8	0	Tspan15	0	Gm6408	0	Tspan33	0
Slc16a7	0	Tspan14	0	Scgn	0	Tspan3	0
Slc16a6	0	Tspan13	0	9530002B09Rik	0	Tspan18	0
Slc16a5	0	Tsnax	0	Nln	0	Tspan17	0
Slc16a4	0	Tsn	0	Gm3404	0	Tspan14	0
Slc16a3	0	Tsku	0	Gm6406	0	Tspan13	0
Slc16a14	0	Tsks	0	Emilin2	0	Tspan11	0
Slc16a13	0	Tshz3	0	Olfr354	0	Tsnax	0
Slc16a12	0	Tshz2	0	Olfr427	0	Tsn	0
Slc16a10	0	Tsga8	0	Gm17677	0	Tslp	0
Slc16a1	0	Tsga10	0	C2cd5	0	Tsku	0
Slc15a5	0	Tsg101	0	Olfr420	0	Tsks	0
Slc15a4	0	Tsen54	0	Prmt2	0	Tshz3	0
Slc15a3	0	Tsen34	0	Prmt3	0	Tshz2	0
Slc15a2	0	Tsen15	0	Prmt1	0	Tshz1	0
Slc15a1	0	Tsc22d4	0	Hfe	0	Tshr	0
Slc14a1	0	Tsc22d3	0	Prmt7	0	Tshb	0
Slc13a5	0	Tsc22d2	0	Olfr429	0	Tsga8	0
Slc13a2	0	Tsc22d1	0	Mrgprb3	0	Tsga13	0
Slc13a1	0	Tsc2	0	Mrgprb2	0	Tsg101	0
Slc12a9	0	Tsacc	0	Mrgprb1	0	Tsfm	0
Slc12a8	0	Trub2	0	BRDN0000737750	0	Tsen2	0
Slc12a7	0	Trub1	0	BRDN0000737757	0	Tsen15	0
Slc12a6	0	Trrap	0	BRDN0000737756	0	Tsc22d2	0
Slc12a4	0	Trpv6	0	Mrgprb5	0	Tsc2	0
Slc12a3	0	Trpv4	0	Spink8	0	Tsc1	0
Slc12a2	0	Trpv3	0	Spink7	0	Try4	0
Slc12a1	0	Trpv1	0	Spink6	0	Trub2	0
Slc11a2	0	Trpt1	0	Spink5	0	Trub1	0
Slc11a1	0	Trps1	0	Spink4	0	Trrap	0
Slc10a7	0	Trpm7	0	Spink3	0	Trpv6	0
Slc10a6	0	Trpm6	0	Vmn1r100	0	Trpv5	0
Slc10a4	0	Trpm4	0	Dfna5	0	Trpv3	0
Slc10a2	0	Trpm3	0	Ostn	0	Trpv2	0
Slc10a1	0	Trpm1	0	Slc28a2	0	Trpv1	0
Slbp	0	Trpd52l3	0	Adora1	0	Trps1	0

Slamf9	0	Trpc7	0	Larp1b	0	Trpm7	0
Slamf7	0	Trpc6	0	Scg5	0	Trpm6	0
Slamf1	0	Trpc5	0	Ppapdc2	0	Trpm5	0
Slain2	0	Trpc4ap	0	Rnft2	0	Trpm3	0
Sla2	0	Trpc4	0	Rnft1	0	Trpd52l3	0
Sla	0	Trpc1	0	Ric1	0	Trpc5	0
Skp2	0	Trp73	0	Ctif	0	Trpc4ap	0
Skp1a	0	Trp53tg5	0	2610305D13Rik	0	Trpc4	0
Skor2	0	Trp53rk	0	BRDN0000737914	0	Trpc3	0
Skiv2l2	0	Trp53inp1	0	Hus1	0	Trpc2	0
Skint6	0	Trp53bp2	0	Tubgcp2	0	Trpa1	0
Skint5	0	Trp53bp1	0	Tubgcp3	0	Trp73	0
Skint4	0	Troap	0	Tubgcp4	0	Trp63	0
Skint2	0	Tro	0	Tubgcp5	0	Trp53tg5	0
Skint11	0	Trnt1	0	Tubgcp6	0	Trp53rk	0
Skint10	0	Trnau1ap	0	Crhr1	0	Trp53bp2	0
Skint1	0	Trmu	0	Stim2	0	Trp53bp1	0
Skil	0	Trmt61a	0	Spinkl	0	Trp53	0
Ski	0	Trmt6	0	Gm5726	0	Trove2	0
Skap2	0	Trmt5	0	Adam29	0	Troap	0
Skap1	0	Trmt44	0	Rffl	0	Tro	0
Ska3	0	Trmt2b	0	Gsc	0	Trnt1	0
Ska2	0	Trmt13	0	Adam28	0	Trnp1	0
Ska1	0	Trmt12	0	Soat2	0	Trnau1ap	0
Six6	0	Trmt112	0	C1qtnf2	0	Trmt61a	0
Six4	0	Trmt10b	0	BC048507	0	Trmt5	0
Six1	0	Trit1	0	Mapk8ip2	0	Trmt2b	0
Siva1	0	Triqk	0	BRDN0000738013	0	Trmt2a	0
Sit1	0	Trip4	0	C1qtnf3	0	Trmt1l	0
Sis	0	Trip11	0	Pak4	0	Trmt13	0
Sirt7	0	Triobp	0	Pak6	0	Trmt12	0
Sirt6	0	Trio	0	1600002H07Rik	0	Trmt112	0
Sirt4	0	Triml2	0	Ssmem1	0	Trmt10c	0
Sirt3	0	Trim9	0	Pak1	0	Trmt10a	0
Sirt2	0	Trim8	0	Pak3	0	Trmt1	0
Sirt1	0	Trim71	0	Olfr398	0	Trit1	0
Sirpb1b	0	Trim69	0	C1qtnf1	0	Triqk	0
Sirpb1a	0	Trim68	0	Nxph1	0	Trip6	0
Sipa1l3	0	Trim67	0	Slc35f4	0	Trip4	0
Sipa1l2	0	Trim66	0	Nedd9	0	Trip13	0
Sipa1l1	0	Trim65	0	9230112D13Rik	0	Trip10	0
Sipa1	0	Trim63	0	BRDN0000737647	0	Triobp	0
Sin3b	0	Trim61	0	Pth2r	0	Trio	0
Sin3a	0	Trim59	0	Yipf1	0	Triml2	0
Sim2	0	Trim58	0	Slc35f6	0	Trim9	0
Sil1	0	Trim54	0	C1qtnf4	0	Trim80	0
Sike1	0	Trim52	0	Mtmr14	0	Trim75	0
Sik3	0	Trim47	0	BRDN0000737641	0	Trim72	0
Sik2	0	Trim43c	0	BRDN0000737504	0	Trim71	0
Sigmar1	0	Trim43b	0	D830030K20Rik	0	Trim68	0
Siglech	0	Trim43a	0	Peo1	0	Trim66	0
Siglecg	0	Trim42	0	Dnajc16	0	Trim65	0
Siglecf	0	Trim41	0	Pmp22	0	Trim61	0
Siglece	0	Trim40	0	Cd300a	0	Trim6	0
Siglec15	0	Trim39	0	Ruvbl1	0	Trim59	0
Siglec1	0	Trim38	0	Tti2	0	Trim58	0
Sidt2	0	Trim36	0	Pla2r1	0	Trim56	0
Sidt1	0	Trim35	0	Yipf5	0	Trim55	0
Siah2	0	Trim34a	0	BRDN0000738157	0	Trim54	0
Siah1b	0	Trim33	0	Gnb1	0	Trim52	0
Siah1a	0	Trim32	0	Yipf4	0	Trim50	0
Siae	0	Trim31	0	Olfr1499	0	Trim45	0
Shroom4	0	Trim30d	0	Dnajc15	0	Trim44	0
Shroom3	0	Trim30a	0	Erv3	0	Trim43b	0
Shroom2	0	Trim3	0	Smarce1	0	Trim43a	0
Shprh	0	Trim28	0	H3f3a	0	Trim41	0
Shpk	0	Trim27	0	Olfr642	0	Trim40	0
Shox2	0	Trim25	0	Eif4e	0	Trim38	0
Shoc2	0	Trim21	0	Olfr1490	0	Trim37	0
Shmt2	0	Trim17	0	Tns1	0	Trim36	0
Shkbp1	0	Trim16	0	Gsn	0	Trim34b	0
Shisa9	0	Trim13	0	Olfr1495	0	Trim34a	0
Shisa7	0	Trim11	0	Olfr392	0	Trim33	0
Shisa6	0	Trib1	0	Olfr1497	0	Trim31	0
Shisa5	0	Triap1	0	BRDN0000738286	0	Trim30d	0
Shisa4	0	Trhr2	0	Olfr394	0	Trim30a	0
Shisa3	0	Trhr	0	Cabp5	0	Trim3	0
Shisa2	0	Trhde	0	Cabp4	0	Trim29	0
Shh	0	Trf	0	Cabp2	0	Trim28	0
Shfm1	0	Trex2	0	Cabp1	0	Trim27	0
Shf	0	Trex1	0	BRDN0000737648	0	Trim26	0
Shd	0	Treml4	0	Krtap4-6	0	Trim25	0

Shcbp1l	0	Treml2	0	Lysmd2	0	Trim21	0
Shcbp1	0	Trem3	0	Fam98a	0	Trim2	0
Shc4	0	Trem1	0	Ak4	0	Trim17	0
Shc2	0	Treh	0	Inafm1	0	Trim15	0
Shc1	0	Trdn	0	Klrb1	0	Trim12c	0
Shbg	0	Trcg1	0	Gfi1	0	Trim11	0
Sharpin	0	Trappc8	0	Smarcb1	0	Trim10	0
Shank3	0	Trappc6b	0	Mog	0	Triap1	0
Shank1	0	Trappc5	0	Cftr	0	Trhr	0
Sh3yl1	0	Trappc4	0	Anln	0	Trhde	0
Sh3tc2	0	Trappc3l	0	Lrrc10b	0	Trh	0
Sh3tc1	0	Trappc3	0	Olfr541	0	Treml4	0
Sh3rf2	0	Trappc2	0	Hk2	0	Treml2	0
Sh3rf1	0	Trappc13	0	Stard5	0	Treml1	0
Sh3pxd2b	0	Trappc12	0	Bche	0	Trem3	0
Sh3pxd2a	0	Trappc11	0	Tapbpl	0	Trem2	0
Sh3glb2	0	Trappc10	0	Hk3	0	Trem1	0
Sh3gl2	0	Trappc1	0	Dnajc19	0	Treh	0
Sh3d21	0	Trap1a	0	Wnt1	0	Trdmt1	0
Sh3d19	0	Trap1	0	4933402E13Rik	0	Trappc8	0
Sh3bp5l	0	Trank1	0	Naa11	0	Trappc6a	0
Sh3bp5	0	Tram1	0	Fam186b	0	Trappc5	0
Sh3bp4	0	Trak2	0	Il22	0	Trappc4	0
Sh3bp2	0	Trak1	0	LOC100038947	0	Trappc2l	0
Sh3bgrl3	0	Traip	0	BRDN0000738105	0	Trappc2	0
Sh3bgrl2	0	Trafd1	0	Wnt6	0	Trappc12	0
Sh3bgrl	0	Traf6	0	Jph2	0	Trappc1	0
Sh3bgr	0	Traf4	0	Jph3	0	Trap1a	0
Sh2d5	0	Traf3ip3	0	Icam5	0	Trap1	0
Sh2d4b	0	Traf3ip2	0	R3hcc1	0	Trank1	0
Sh2d4a	0	Traf3	0	Icam2	0	Tram2	0
Sh2d2a	0	Traf2	0	Icam1	0	Tram1	0
Sh2d1b2	0	Tradd	0	Tcea1	0	Trak2	0
Sh2d1b1	0	Trabd2b	0	Foxl2	0	Trak1	0
Sh2d1a	0	Trabd	0	Syde1	0	Traip	0
Sh2b3	0	Tra2b	0	Nxph4	0	Trafd1	0
Sh2b1	0	Tpx2	0	Syngap1	0	Traf6	0
Sgtb	0	Tpte	0	Amz1	0	Traf3ip3	0
Sgta	0	Tpt1	0	Amz2	0	Traf3ip2	0
Sgsm3	0	Tpst2	0	Krtap13	0	Traf3ip1	0
Sgsm2	0	Tpsg1	0	Tpm4	0	Traf3	0
Sgsm1	0	Tpsb2	0	Coq10b	0	Traf2	0
Sgsh	0	Tprn	0	Tpm2	0	Traf1	0
Sgpl1	0	Tprkb	0	Tpm1	0	Tradd	0
Sgol2b	0	Tprgl	0	Coq10a	0	Trabd2b	0
Sgol2a	0	Tpra1	0	Ccdc152	0	Trabd	0
Sgol1	0	Tppp2	0	Spic	0	Tra2b	0
Sgms2	0	Tpp1	0	Ccdc150	0	Tpx2	0
Sgms1	0	Tpmt	0	Ccdc151	0	Tpte	0
Sgk3	0	Tpm4	0	Ccdc157	0	Tpt1	0
Sgk2	0	Tpm3	0	Ccdc154	0	Tpsg1	0
Sgk1	0	Tpm1	0	Ccdc155	0	Tprn	0
Sgip1	0	Tpk1	0	Mmp7	0	Tprgl	0
Sgcz	0	Tpi1	0	Brs3	0	Tpra1	0
Sgcg	0	Tph1	0	Ccdc159	0	Tpr	0
Sgce	0	Tpgs2	0	Mmp2	0	Tppp3	0
Sgcd	0	Tpgs1	0	Mmp3	0	Tppp2	0
Sgcb	0	Tpd52	0	Slc26a5	0	Tpo	0
Sgca	0	Tpcn2	0	Zfp663	0	Tpm4	0
Sfxn5	0	Tpcn1	0	2610028H24Rik	0	Tpm2	0
Sfxn4	0	Tpbpb	0	Zfp661	0	Tpk1	0
Sfxn3	0	Tpbpa	0	Jmjd6	0	Tpi1	0
Sfxn1	0	Tox4	0	Zfp667	0	Tph2	0
Sftpd	0	Tox3	0	Zfp664	0	Tph1	0
Sfta2	0	Tor4a	0	Gm13212	0	Tpgs2	0
Sft2d3	0	Tor3a	0	Rarres2	0	Tpd52l2	0
Sft2d2	0	Tor2a	0	Zfp668	0	Tpd52l1	0
Sft2d1	0	Tor1aip2	0	Slc26a7	0	Tpd52	0
Sfswap	0	Tor1aip1	0	C030039L03Rik	0	Tpcn2	0
Sfrp5	0	Toporsl	0	Pou2f3	0	Tpcn1	0
Sfrp4	0	Topors	0	Plch2	0	Tpbpb	0
Sfrp2	0	Topbp1	0	Slc26a8	0	Tox3	0
Sfrp1	0	Top3b	0	Secisbp2l	0	Tox	0
Sfr1	0	Top3a	0	Plch1	0	Tor3a	0
Sfpq	0	Top2b	0	Susd2	0	Tor1aip2	0
Sfn	0	Top2a	0	Susd3	0	Tor1aip1	0
Sfmbt2	0	Top1mt	0	Slc26a9	0	Tor1a	0
Sfmbt1	0	Top1	0	Skp2	0	Topors	0
Sf3b6	0	Tonsl	0	Cherp	0	Topbp1	0
Sf3b5	0	Tomt	0	Zbtb8os	0	Topaz1	0
Sf3b4	0	Tomm70a	0	BRDN0000737574	0	Top3b	0
Sf3b3	0	Tomm7	0	Capza3	0	Top3a	0

Sf3b2	0	Tomm6	0	Tyrp1	0	Top2b	0
Sf3b1	0	Tomm5	0	Capza1	0	Top2a	0
Sf3a3	0	Tomm34	0	Spi1	0	Top1mt	0
Sf3a2	0	Tomm22	0	Olfr998	0	Top1	0
Sf3a1	0	Tomm20l	0	Rasgrp3	0	Tonsl	0
Sez6l2	0	Tomm20	0	Olfr996	0	Tomt	0
Sez6l	0	Tom1	0	Serinc4	0	Tomm70a	0
Sez6	0	Tollip	0	Olfr994	0	Tomm7	0
Setx	0	Tnrc18	0	Tpmt	0	Tomm5	0
Setmar	0	Tnpo3	0	Serinc1	0	Tomm22	0
Setdb2	0	Tnpo2	0	Olfr993	0	Tomm20l	0
Setdb1	0	Tnpo1	0	Serinc3	0	Tomm20	0
Setd8	0	Tnnt3	0	AA792892	0	Tom1l2	0
Setd5	0	Tnnt1	0	2300003K06Rik	0	Tom1	0
Setd4	0	Tnni3	0	Ctsc	0	Toe1	0
Setd3	0	Tnni2	0	Radil	0	Tob2	0
Setd2	0	Tnni1	0	Snx33	0	Tns4	0
Setd1b	0	Tnnc2	0	Dpm3	0	Tns2	0
Setd1a	0	Tnnc1	0	Cdh11	0	Tns1	0
Setbp1	0	Tnn	0	Cdh10	0	Tnrc6c	0
Set	0	Tnmd	0	Cdh13	0	Tnrc6a	0
Sesn3	0	Tnks2	0	Cdh12	0	Tnrc18	0
Sesn2	0	Tnks1bp1	0	Cdh15	0	Tnr	0
Sesn1	0	Tnks	0	Tgif2lx2	0	Tnpo3	0
Sertm1	0	Tnk1	0	Cdh16	0	Tnpo2	0
Sertad4	0	Tnip3	0	Gm5382	0	Tnpo1	0
Sertad2	0	Tnik	0	Cdh18	0	Tnp2	0
Sertad1	0	Tnfsfm13	0	Pou2f1	0	Tnnt3	0
Serpini2	0	Tnfsf9	0	Chm	0	Tnnt2	0
Serpini1	0	Tnfsf8	0	Mrpl54	0	Tnni3k	0
Serpinh1	0	Tnfsf4	0	Olfr64	0	Tnnc1	0
Serping1	0	Tnfsf14	0	Olfr67	0	Tnn	0
Serpinf2	0	Tnfsf10	0	Mrpl57	0	Tnmd	0
Serpine3	0	Tnfrsf9	0	Olfr60	0	Tnks	0
Serpine2	0	Tnfrsf4	0	Olfr63	0	Tnk1	0
Serpind1	0	Tnfrsf26	0	Olfr62	0	Tnip3	0
Serpinb9g	0	Tnfrsf25	0	BRDN0000738313	0	Tnip1	0
Serpinb9f	0	Tnfrsf23	0	Acly	0	Tnfsf9	0
Serpinb9e	0	Tnfrsf22	0	Olfr69	0	Tnfsf8	0
Serpinb9d	0	Tnfrsf21	0	Olfr68	0	Tnfsf4	0
Serpinb9c	0	Tnfrsf1b	0	Grhl1	0	Tnfsf15	0
Serpinb9b	0	Tnfrsf1a	0	Pld4	0	Tnfsf13	0
Serpinb9	0	Tnfrsf19	0	Npl	0	Tnfrsf8	0
Serpinb8	0	Tnfrsf18	0	Pcdh8	0	Tnfrsf4	0
Serpinb7	0	Tnfrsf17	0	Gm13277	0	Tnfrsf26	0
Serpinb6d	0	Tnfrsf13c	0	Sh2d4a	0	Tnfrsf23	0
Serpinb6c	0	Tnfrsf12a	0	Tmem147	0	Tnfrsf21	0
Serpinb6b	0	Tnfrsf11b	0	Pld2	0	Tnfrsf1a	0
Serpinb6a	0	Tnfrsf11a	0	Sh2d4b	0	Tnfrsf19	0
Serpinb3c	0	Tnfrsf10b	0	Pou2f2	0	Tnfrsf18	0
Serpinb3b	0	Tnfaip8l3	0	Sod1	0	Tnfrsf17	0
Serpinb3a	0	Tnfaip8l2	0	Ctnna1	0	Tnfrsf14	0
Serpinb2	0	Tnfaip8l1	0	Nfyc	0	Tnfrsf11b	0
Serpinb1c	0	Tnfaip6	0	Nfyb	0	Tnfrsf11a	0
Serpinb1b	0	Tnfaip3	0	Nfya	0	Tnfrsf10b	0
Serpinb1a	0	Tnfaip2	0	Dnd1	0	Tnfaip8l1	0
Serpinb13	0	Tnfaip1	0	4930427A07Rik	0	Tnfaip8	0
Serpinb12	0	Tnf	0	Gm13272	0	Tnfaip3	0
Serpinb11	0	Tmx3	0	Riok3	0	Tnf	0
Serpina7	0	Tmx2	0	D030056L22Rik	0	Tmx4	0
Serpina6	0	Tmx1	0	Kirrel2	0	Tmx2	0
Serpina5	0	Tmub2	0	Tmem51	0	Tmub2	0
Serpina3n	0	Tmub1	0	Gzmd	0	Tmub1	0
Serpina3m	0	Tmtc4	0	Gzme	0	Tmtc2	0
Serpina3k	0	Tmtc1	0	Scamp4	0	Tmtc1	0
Serpina3i	0	Tmsb4x	0	Gzmg	0	Tmsb15b1	0
Serpina3f	0	Tmsb15b2	0	Scamp2	0	Tmsb15a	0
Serpina3c	0	Tmsb15b1	0	Gzma	0	Tmsb10	0
Serpina3b	0	Tmsb15a	0	Gzmb	0	Tmprss7	0
Serpina3a	0	Tmprss9	0	Plcb1	0	Tmprss6	0
Serpina1f	0	Tmprss7	0	Ids	0	Tmprss5	0
Serpina1e	0	Tmprss5	0	Il1f10	0	Tmprss4	0
Serpina1d	0	Tmprss4	0	Unc45a	0	Tmprss3	0
Serpina1c	0	Tmprss2	0	Plcb2	0	Tmprss2	0
Serpina1a	0	Tmprss15	0	Unc45b	0	Tmprss15	0
Serpina12	0	Tmprss13	0	Polr2e	0	Tmprss13	0
Serpina11	0	Tmprss11g	0	Polr2d	0	Tmprss11g	0
Serpina10	0	Tmprss11e	0	Polr2g	0	Tmprss11f	0
Serp1	0	Tmprss11d	0	Polr2f	0	Tmprss11e	0
Serinc5	0	Tmprss11c	0	Polr2a	0	Tmprss11d	0
Serinc4	0	Tmprss11b	0	Mansc1	0	Tmprss11c	0
Serinc1	0	Tmppe	0	Polr2c	0	Tmprss11b	0

Serf2	0
Serf1	0
Serbp1	0
Serac1	0
Sepw1	0
Sept9	0
Sept8	0
Sept7	0
Sept6	0
Sept5	0
Sept3	0
Sept2	0
Sept14	0
Sept12	0
Sept11	0
Sept10	0
Sept1	0
Sepsecs	0
Sepp1	0
Sepn1	0
Sephs2	0
Sephs1	0
Sep15	0
Senp8	0
Senp6	0
Senp5	0
Senp3	0
Senp2	0
Sema7a	0
Sema6d	0
Sema6b	0
Sema6a	0
Sema5b	0
Sema5a	0
Sema4g	0
Sema4f	0
Sema3g	0
Sema3f	0
Sema3e	0
Sema3d	0
Sema3c	0
Selt	0
Selplg	0
Selp	0
Selo	0
Selm	0
Selk	0
Selenbp2	0
Selenbp1	0
Sele	0
Sel1l3	0
Sel1l2	0
Sel1l	0
Seh1l	0
Sectm1b	0
Sectm1a	0
Secisbp2l	0
Secisbp2	0
Sec61g	0
Sec61b	0
Sec61a1	0
Sec31b	0
Sec31a	0
Sec24d	0
Sec24c	0
Sec24b	0
Sec24a	0
Sec23ip	0
Sec23b	0
Sec22c	0
Sec22a	0
Sec16a	0
Sec14l5	0
Sec14l4	0
Sec14l3	0
Sec14l2	0
Sec14l1	0
Sec13	0
Sec11a	0
Sebox	0
Sdsl	0
Sdr9c7	0
Tmpo	0
Tmod4	0
Tmod3	0
Tmod2	0
Tmod1	0
Tmigd1	0
Tmf1	0
Tmem98	0
Tmem97	0
Tmem95	0
Tmem92	0
Tmem91	0
Tmem9	0
Tmem8c	0
Tmem89	0
Tmem88	0
Tmem87b	0
Tmem87a	0
Tmem86b	0
Tmem86a	0
Tmem82	0
Tmem81	0
Tmem80	0
Tmem8	0
Tmem79	0
Tmem74b	0
Tmem74	0
Tmem72	0
Tmem70	0
Tmem69	0
Tmem68	0
Tmem67	0
Tmem64	0
Tmem63c	0
Tmem63b	0
Tmem63a	0
Tmem62	0
Tmem59l	0
Tmem59	0
Tmem55b	0
Tmem54	0
Tmem53	0
Tmem51	0
Tmem50a	0
Tmem5	0
Tmem45b	0
Tmem45a2	0
Tmem45a	0
Tmem44	0
Tmem43	0
Tmem41b	0
Tmem39b	0
Tmem39a	0
Tmem38b	0
Tmem38a	0
Tmem30c	0
Tmem30a	0
Tmem28	0
Tmem27	0
Tmem261	0
Tmem259	0
Tmem258	0
Tmem255b	0
Tmem254b	0
Tmem254a	0
Tmem253	0
Tmem252	0
Tmem25	0
Tmem248	0
Tmem247	0
Tmem246	0
Tmem243	0
Tmem239	0
Tmem237	0
Tmem236	0
Tmem235	0
Tmem234	0
Tmem233	0
Tmem232	0
Tmem230	0
Tmem229a	0
Tmem225	0
Polr2b	0
Htr1d	0
Polr2l	0
Htr1f	0
Polr2i	0
Polr2h	0
Htr1b	0
Polr2j	0
Olfr549	0
Fev	0
Bsg	0
Ptrf	0
BRDN0000737897	0
Slc48a1	0
BRDN0000737898	0
Ust	0
Atp11c	0
Skil	0
Atp11a	0
BRDN0000737899	0
Fibcd1	0
Hoxb13	0
Hibch	0
Defa2	0
Hmbs	0
Afm	0
Supt6	0
Slc28a1	0
Gsc2	0
Rdh11	0
Adgre5	0
AU019823	0
Thrb	0
Greb1	0
Thra	0
Olfr698	0
Cd14	0
Rps10	0
Ube2r2	0
Abra	0
Krt16	0
Afp	0
Htra3	0
A530016L24Rik	0
D16Ertd472e	0
Olfr2	0
Fbxo44	0
Fbxo47	0
Olfr1	0
Vasn	0
Fbxo40	0
Fbxo43	0
Fbxo42	0
Lix1	0
Skor2	0
Olfr9	0
Eri2	0
Fbxo48	0
Ndc1	0
Eri1	0
Gm5127	0
Trpc7	0
Ccni	0
Baiap2	0
Ccdc136	0
Mup3	0
Xab2	0
Gm5128	0
Gm20852	0
BRDN0000738189	0
BRDN0000738188	0
Chd1l	0
Rasl10b	0
BRDN0000737587	0
BRDN0000738182	0
BRDN0000738181	0
Svopl	0
BRDN0000738187	0
BRDN0000738186	0
BRDN0000738185	0
BRDN0000738184	0
Per3	0
Tmprss11a	0
Tmppe	0
Tmod3	0
Tmod2	0
Tmod1	0
Tmigd1	0
Tmie	0
Tmem9b	0
Tmem97	0
Tmem95	0
Tmem92	0
Tmem91	0
Tmem8c	0
Tmem89	0
Tmem88	0
Tmem86b	0
Tmem82	0
Tmem81	0
Tmem74b	0
Tmem74	0
Tmem72	0
Tmem71	0
Tmem70	0
Tmem68	0
Tmem65	0
Tmem63b	0
Tmem63a	0
Tmem60	0
Tmem59l	0
Tmem57	0
Tmem56	0
Tmem55b	0
Tmem55a	0
Tmem53	0
Tmem50b	0
Tmem50a	0
Tmem5	0
Tmem47	0
Tmem45b	0
Tmem45a2	0
Tmem44	0
Tmem43	0
Tmem42	0
Tmem41b	0
Tmem41a	0
Tmem39b	0
Tmem38b	0
Tmem38a	0
Tmem37	0
Tmem35	0
Tmem33	0
Tmem30c	0
Tmem30b	0
Tmem30a	0
Tmem28	0
Tmem27	0
Tmem263	0
Tmem260	0
Tmem26	0
Tmem259	0
Tmem258	0
Tmem255b	0
Tmem255a	0
Tmem254c	0
Tmem254b	0
Tmem253	0
Tmem252	0
Tmem251	0
Tmem248	0
Tmem247	0
Tmem246	0
Tmem243	0
Tmem240	0
Tmem239	0
Tmem237	0
Tmem236	0
Tmem235	0
Tmem232	0
Tmem230	0
Tmem229b	0
Tmem229a	0
Tmem225	0

Sdr42e1	0	Tmem220	0	Per2	0	Tmem222	0
Sdr39u1	0	Tmem219	0	Per1	0	Tmem220	0
Sdr16c6	0	Tmem218	0	Tbx1	0	Tmem219	0
Sdr16c5	0	Tmem217	0	Cnbd2	0	Tmem217	0
Sdpr	0	Tmem216	0	Tbx4	0	Tmem216	0
Sdk2	0	Tmem213	0	Tbx5	0	Tmem215	0
Sdk1	0	Tmem212	0	Nos3	0	Tmem214	0
Sdhd	0	Tmem209	0	Ptges	0	Tmem213	0
Sdhb	0	Tmem208	0	Slc35c2	0	Tmem211	0
Sdhaf4	0	Tmem207	0	Enthd1	0	Tmem210	0
Sdhaf3	0	Tmem205	0	Slc35c1	0	Tmem208	0
Sdhaf2	0	Tmem203	0	Dbi	0	Tmem207	0
Sdhaf1	0	Tmem202	0	Zdbf2	0	Tmem206	0
Sdha	0	Tmem201	0	Slc15a1	0	Tmem204	0
Sdf4	0	Tmem200c	0	Tha1	0	Tmem202	0
Sdf2l1	0	Tmem200b	0	Ss18l1	0	Tmem200a	0
Sdf2	0	Tmem200a	0	Htr2a	0	Tmem2	0
Sde2	0	Tmem2	0	Gdf3	0	Tmem199	0
Sdccag8	0	Tmem199	0	C1galt1	0	Tmem198	0
Sdccag3	0	Tmem198	0	Mid1ip1	0	Tmem196	0
Sdcbp2	0	Tmem196	0	Wsb2	0	Tmem194b	0
Sdcbp	0	Tmem194	0	Gdf2	0	Tmem194	0
Sdc4	0	Tmem192	0	Olfr350	0	Tmem192	0
Sdc3	0	Tmem19	0	Dbp	0	Tmem190	0
Sdc2	0	Tmem189	0	Mafg	0	Tmem19	0
Sdc1	0	Tmem186	0	Tesk1	0	Tmem186	0
Sdad1	0	Tmem185b	0	Tesk2	0	Tmem184c	0
Scyl3	0	Tmem184c	0	BRDN0000738367	0	Tmem184a	0
Scyl2	0	Tmem184b	0	Edaradd	0	Tmem183a	0
Scyl1	0	Tmem184a	0	Olfr678	0	Tmem182	0
Scx	0	Tmem183a	0	BRDN0000738363	0	Tmem181a	0
Scube3	0	Tmem182	0	BRDN0000738362	0	Tmem180	0
Scube2	0	Tmem181a	0	BRDN0000738361	0	Tmem179	0
Sctr	0	Tmem18	0	BRDN0000738360	0	Tmem178b	0
Sct	0	Tmem178b	0	Vsig10	0	Tmem178	0
Scrt2	0	Tmem177	0	Cenpc1	0	Tmem177	0
Scrt1	0	Tmem176a	0	Olfr671	0	Tmem175	0
Scrn3	0	Tmem175	0	Olfr670	0	Tmem174	0
Scrn2	0	Tmem174	0	Olfr677	0	Tmem171	0
Scrn1	0	Tmem171	0	Olfr676	0	Tmem170b	0
Scrib	0	Tmem17	0	Olfr675	0	Tmem170	0
Scrg1	0	Tmem169	0	Olfr1313	0	Tmem169	0
Scpep1	0	Tmem168	0	Purb	0	Tmem168	0
Scp2d1	0	Tmem167b	0	Pura	0	Tmem167	0
Scp2	0	Tmem167	0	Ldb1	0	Tmem165	0
Scoc	0	Tmem165	0	Olfr1310	0	Tmem164	0
Sco2	0	Tmem164	0	Gm9125	0	Tmem163	0
Sco1	0	Tmem163	0	Ctse	0	Tmem161b	0
Scnn1b	0	Tmem161b	0	Lonp2	0	Tmem161a	0
Scnn1a	0	Tmem161a	0	Epc2	0	Tmem160	0
Scn5a	0	Tmem160	0	Lonp1	0	Tmem159	0
Scn4b	0	Tmem158	0	Pwp1	0	Tmem154	0
Scn4a	0	Tmem151a	0	Olfr975	0	Tmem151a	0
Scn3b	0	Tmem150c	0	Tbc1d22b	0	Tmem150b	0
Scn3a	0	Tmem150b	0	Tbc1d22a	0	Tmem14c	0
Scn2b	0	Tmem150a	0	Insr	0	Tmem14a	0
Scn1b	0	Tmem14c	0	Pmch	0	Tmem147	0
Scn1a	0	Tmem14a	0	Kmo	0	Tmem144	0
Scn11a	0	Tmem147	0	Cyp2a5	0	Tmem143	0
Scml4	0	Tmem145	0	Cyp2a4	0	Tmem141	0
Scml2	0	Tmem144	0	Xpr1	0	Tmem140	0
Scmh1	0	Tmem143	0	Lzic	0	Tmem139	0
Scly	0	Tmem140	0	Abca8b	0	Tmem136	0
Sclt1	0	Tmem139	0	Ntpcr	0	Tmem132d	0
Scimp	0	Tmem138	0	Arsg	0	Tmem132b	0
Schip1	0	Tmem136	0	Apol10a	0	Tmem132a	0
Scgn	0	Tmem135	0	Arsb	0	Tmem130	0
Scgb3a2	0	Tmem132d	0	Ifng	0	Tmem129	0
Scgb3a1	0	Tmem132c	0	Gm14743	0	Tmem128	0
Scgb2b3	0	Tmem132a	0	Olfr202	0	Tmem126b	0
Scgb2b27	0	Tmem131	0	Arsj	0	Tmem126a	0
Scgb2b26	0	Tmem130	0	Arsk	0	Tmem123	0
Scgb2b24	0	Tmem129	0	Rftn2	0	Tmem121	0
Scgb2b20	0	Tmem127	0	Rftn1	0	Tmem120b	0
Scgb2b17	0	Tmem126a	0	Fryl	0	Tmem120a	0
Scgb2b15	0	Tmem125	0	Vmn2r120	0	Tmem119	0
Scgb1c1	0	Tmem123	0	Vmn2r121	0	Tmem116	0
Scgb1b7	0	Tmem121	0	Atp5k	0	Tmem114	0
Scgb1b3	0	Tmem120b	0	Parp11	0	Tmem11	0
Scgb1b29	0	Tmem120a	0	Lcmt2	0	Tmem109	0
Scgb1b27	0	Tmem116	0	Ksr2	0	Tmem108	0
Scgb1b24	0	Tmem114	0	Lox	0	Tmem107	0

Scgb1b20	0	Tmem110	0	Paqr9	0	Tmem106b	0
Scgb1b2	0	Tmem11	0	Ccdc14	0	Tmem106a	0
Scgb1b19	0	Tmem109	0	Ythdc2	0	Tmem104	0
Scg5	0	Tmem108	0	Gmcl1l	0	Tmem102	0
Scg3	0	Tmem106c	0	Ivns1abp	0	Tmem101	0
Scg2	0	Tmem106b	0	Paqr3	0	Tmem100	0
Scfd2	0	Tmem106a	0	Paqr5	0	Tmeff2	0
Scfd1	0	Tmem104	0	Paqr4	0	Tmeff1	0
Scel	0	Tmem102	0	Igfbpl1	0	Tmed8	0
Scd4	0	Tmem101	0	Paqr6	0	Tmed7	0
Scd3	0	Tmem100	0	Kng2	0	Tmed5	0
Scd2	0	Tmeff2	0	Smco4	0	Tmed4	0
Scd1	0	Tmeff1	0	Ccdc17	0	Tmed2	0
Sccpdh	0	Tmed8	0	Dennd4a	0	Tmed11	0
Scarf2	0	Tmed7	0	Dennd4b	0	Tmed10	0
Scarf1	0	Tmed6	0	Birc6	0	Tmco6	0
Scarb1	0	Tmed5	0	Ankk1	0	Tmco5b	0
Scara3	0	Tmed4	0	Tmed10	0	Tmco5	0
Scaper	0	Tmed2	0	Tpt1	0	Tmco4	0
Scap	0	Tmed11	0	Btk	0	Tmco2	0
Scand1	0	Tmed10	0	Taar5	0	Tmco1	0
Scamp5	0	Tmco5b	0	Caly	0	Tmcc3	0
Scamp4	0	Tmco4	0	Gm8298	0	Tmcc2	0
Scamp3	0	Tmco3	0	Pnck	0	Tmc6	0
Scamp2	0	Tmco2	0	Taar3	0	Tmc5	0
Scamp1	0	Tmcc3	0	Gm11992	0	Tmc2	0
Scaf8	0	Tmcc1	0	Mpp7	0	Tmc1	0
Scaf1	0	Tmc7	0	Stk11	0	Tmbim7	0
Sc5d	0	Tmc6	0	Stk16	0	Tmbim4	0
Sbspon	0	Tmc1	0	Calu	0	Tma7	0
Sbp	0	Tmbim7	0	Pappa	0	Tma16	0
Sbno2	0	Tma7	0	Hdac11	0	Tm9sf4	0
Sbno1	0	Tma16	0	Cbfb	0	Tm9sf3	0
Sbk3	0	Tm9sf4	0	Olfr345	0	Tm9sf2	0
Sbk2	0	Tm9sf3	0	Rasal1	0	Tm9sf1	0
Sbk1	0	Tm9sf2	0	Pced1a	0	Tm7sf2	0
Sbf1	0	Tm9sf1	0	Lsm11	0	Tm6sf1	0
Sbds	0	Tm7sf2	0	Exoc7	0	Tm4sf20	0
Saxo2	0	Tm6sf2	0	Rpl36a	0	Tm4sf19	0
Saxo1	0	Tm6sf1	0	Syne4	0	Tm2d3	0
Sav1	0	Tm4sf5	0	Mbip	0	Tm2d2	0
Satb2	0	Tm4sf19	0	Aprt	0	Tm2d1	0
Satb1	0	Tm2d3	0	Phb	0	Tlx3	0
Sat2	0	Tm2d2	0	Mustn1	0	Tlx2	0
Sat1	0	Tm2d1	0	St3gal4	0	Tlx1	0
Sass6	0	Tlx3	0	Dmtn	0	Tlr8	0
Sash3	0	Tlr9	0	Ccdc3	0	Tlr7	0
Sash1	0	Tlr7	0	Atp5h	0	Tlr4	0
Sart3	0	Tlr6	0	Ccdc6	0	Tlr3	0
Sart1	0	Tlr4	0	Ccdc7	0	Tlr2	0
Sars2	0	Tlr3	0	Itga3	0	Tlr11	0
Sars	0	Tlr2	0	Zxdb	0	Tln2	0
Sarnp	0	Tlr13	0	Ccdc8	0	Tln1	0
Sarm1	0	Tlr1	0	Ccdc9	0	Tll2	0
Sardh	0	Tln2	0	Guca1a	0	Tlk2	0
Saraf	0	Tll2	0	Guca1b	0	Tle6	0
Sar1b	0	Tll1	0	Slpi	0	Tle4	0
Sar1a	0	Tlk2	0	Krt14	0	Tle3	0
Sapcd1	0	Tlk1	0	Nhs	0	Tldc2	0
Sap30bp	0	Tle6	0	Ninj1	0	Tldc1	0
Sap30	0	Tle4	0	Hyal3	0	Tktl1	0
Sap25	0	Tle3	0	Hyal2	0	Tkt	0
Sap18	0	Tle2	0	Acsm3	0	Tkfc	0
Sap130	0	Tle1	0	Acsm2	0	Tk2	0
Samt4	0	Tldc2	0	Acsm5	0	Tk1	0
Samt3	0	Tlcd2	0	Hyal6	0	Tjp3	0
Samt2	0	Tlcd1	0	Hyal5	0	Tjp2	0
Samsn1	0	Tktl2	0	Hyal4	0	Tjap1	0
Samm50	0	Tkt	0	Trim46	0	Tirap	0
Samhd1	0	Tjp3	0	Cyld	0	Tiprl	0
Samd9l	0	Tjp1	0	Adhfe1	0	Tipin	0
Samd4	0	Tirap	0	Trim47	0	Tiparp	0
Samd3	0	Tipin	0	Kir3dl1	0	Tinf2	0
Samd15	0	Tiparp	0	Kir3dl2	0	Tinagl1	0
Samd14	0	Tinagl1	0	Shkbp1	0	Tinag	0
Samd12	0	Tinag	0	Myh2	0	Timp1	0
Samd11	0	Timp4	0	Myh3	0	Timm9	0
Samd10	0	Timp2	0	Gypc	0	Timm8b	0
Sall3	0	Timm8a2	0	Atp8a2	0	Timm8a1	0
Sall1	0	Timm8a1	0	Myh7	0	Timm50	0
Sag	0	Timm44	0	Myh4	0	Timm44	0
Safb2	0	Timm23	0	Prss57	0	Timm23	0

Sae1	0
Sacm1l	0
Sac3d1	0
Saal1	0
Saa4	0
Saa3	0
Saa2	0
Saa1	0
S1pr5	0
S1pr4	0
S1pr3	0
S1pr2	0
S1pr1	0
S100z	0
S100pbp	0
S100g	0
S100b	0
S100a9	0
S100a8	0
S100a7a	0
S100a6	0
S100a5	0
S100a3	0
S100a2	0
S100a14	0
S100a13	0
S100a11	0
S100a10	0
S100a1	0
Ryr2	0
Ryk	0
Rybp	0
Rxrg	0
Rxfp3	0
Rxfp2	0
Rxfp1	0
Rwdd4a	0
Rwdd3	0
Rwdd2b	0
Rwdd2a	0
Rwdd1	0
Ruvbl2	0
Ruvbl1	0
Rusc2	0
Rusc1	0
Runx3	0
Runx2	0
Runx1t1	0
Runx1	0
Rundc3b	0
Rundc3a	0
Rundc1	0
Rufy4	0
Rufy3	0
Rufy1	0
Rttn	0
Rtp4	0
Rtp3	0
Rtp2	0
Rtp1	0
Rtn4rl2	0
Rtn4ip1	0
Rtn3	0
Rtn2	0
Rtn1	0
Rtl1	0
Rtkn	0
Rtfdc1	0
Rtf1	0
Rtel1	0
Rtcb	0
Rtca	0
Rtbdn	0
Rsu1	0
Rsrp1	0
Rspry1	0
Rspo1	0
Rsph9	0
Rsph4a	0
Rsph3a	0
Rsph14	0
Rsph1	0

Timm22	0
Timm21	0
Timm17b	0
Timm17a	0
Timm10b	0
Timm10	0
Timeless	0
Timd4	0
Timd2	0
Tigit	0
Tigd5	0
Tifab	0
Tifa	0
Tie1	0
Ticrr	0
Ticam1	0
Tiam2	0
Tiam1	0
Tial1	0
Thyn1	0
Thy1	0
Thumpd3	0
Thumpd2	0
Thtpa	0
Thsd7a	0
Thsd4	0
Thrsp	0
Thrb	0
Thra	0
Thpo	0
Thop1	0
Thoc7	0
Thoc3	0
Thoc2	0
Thnsl1	0
Thg1l	0
Themis3	0
Themis2	0
Them7	0
Them6	0
Them5	0
Them4	0
Theg	0
Thbs3	0
Thbs1	0
Thbd	0
Thap7	0
Thap4	0
Thap2	0
Thap11	0
Thap1	0
Tha1	0
Th	0
Tgtp2	0
Tgtp1	0
Tgs1	0
Tgoln1	0
Tgm5	0
Tgm4	0
Tgif2lx1	0
Tgfbrap1	0
Tgfbr3l	0
Tgfbi	0
Tgfb1	0
Tgfa	0
Tgds	0
Tg	0
Tfr2	0
Tfpt	0
Tfpi2	0
Tfpi	0
Tfip11	0
Tfg	0
Tff2	0
Tfeb	0
Tfe3	0
Tfdp2	0
Tfcp2	0
Tfb2m	0
Tfb1m	0
Tfap2e	0
Tfap2b	0

Trim42	0
Myh8	0
Myh9	0
Trp53bp1	0
Trp53bp2	0
Gm14124	0
BC024978	0
Prl3b1	0
Ucn	0
Snrnp70	0
4930549C01Rik	0
4930468A15Rik	0
Rhox1	0
Tmem29	0
Tmem28	0
Kif1a	0
Kif1b	0
Rhox8	0
Rhox9	0
Ube2ql1	0
Tmem27	0
Tmem26	0
Mitd1	0
Prdm4	0
Prdm5	0
Prdm6	0
Prdm1	0
Prdm2	0
Epb4.1l4b	0
Mrpl39	0
Prdm8	0
Uba1y	0
Rangrf	0
Tmpo	0
Champ1	0
Rab5b	0
Unc119b	0
Atp5l	0
Krtap22-2	0
Rgs4	0
Rgs5	0
Tm6sf2	0
Rgs7	0
Glipr1l1	0
Epb4.1l3	0
Rgs2	0
Glipr1l2	0
2700062C07Rik	0
Krtap10-4	0
Nvl	0
Rgs8	0
Rgs9	0
2810459M11Rik	0
Camp	0
Cplx4	0
Gnptg	0
Aass	0
Prickle2	0
Fam47e	0
Psma2	0
Pde6c	0
Psma1	0
Kif11	0
Kif12	0
Psma5	0
Olfr703	0
Krt13	0
Psma8	0
BRDN0000737732	0
Gata4	0
Lrguk	0
Gm15127	0
Gata5	0
Msto1	0
Tmigd1	0
Gm5460	0
Rpl18a	0
9530077C05Rik	0
Gm5464	0
BRDN0000738372	0
BRDN0000737679	0
Ttc7b	0

Timm22	0
Timm17b	0
Timm17a	0
Timm13	0
Timm10	0
Timeless	0
Timd4	0
Timd2	0
Tigd5	0
Tigd3	0
Tifa	0
Tie1	0
Ticam2	0
Ticam1	0
Tiam1	0
Tial1	0
Thyn1	0
Thy1	0
Thumpd2	0
Thsd7b	0
Thsd7a	0
Thrb	0
Thrap3	0
Thra	0
Thpo	0
Thoc7	0
Thoc6	0
Thoc5	0
Thoc3	0
Thoc2	0
Thoc1	0
Thnsl2	0
Thnsl1	0
Thg1l	0
Themis3	0
Themis2	0
Them7	0
Them5	0
Theg	0
Thbs4	0
Thbs2	0
Thbs1	0
Thbd	0
Thap7	0
Thap2	0
Thap11	0
Thap1	0
Thada	0
Tha1	0
Th	0
Tgtp2	0
Tgtp1	0
Tgs1	0
Tgm6	0
Tgm5	0
Tgm4	0
Tgm3	0
Tgif2lx2	0
Tgif2lx1	0
Tgif2	0
Tgif1	0
Tgfbr3	0
Tgfbi	0
Tgfb3	0
Tgfb2	0
Tgfb1i1	0
Tgfb1	0
Tfrc	0
Tfr2	0
Tfpi2	0
Tfip11	0
Tff2	0
Tfdp1	0
Tfcp2l1	0
Tfcp2	0
Tfb2m	0
Tfb1m	0
Tfap4	0
Tfap2d	0
Tfap2a	0
Tfam	0
Tex40	0

Rslcan18	0
Rsl24d1	0
Rsl1d1	0
Rsl1	0
Rsg1	0
Rsf1	0
Rsc1a1	0
Rsbn1	0
Rsad2	0
Rsad1	0
Rs1	0
Rrs1	0
Rrp9	0
Rrp8	0
Rrp7a	0
Rrp36	0
Rrp1b	0
Rrp15	0
Rrp12	0
Rrp1	0
Rrnad1	0
Rrn3	0
Rrm2b	0
Rrm2	0
Rrm1	0
Rrh	0
Rreb1	0
Rras2	0
Rras	0
Rragd	0
Rragc	0
Rragb	0
Rraga	0
Rqcd1	0
Rpusd4	0
Rpusd3	0
Rpusd2	0
Rpusd1	0
Rptor	0
Rpsa	0
Rps9	0
Rps8	0
Rps7	0
Rps6kc1	0
Rps6kb2	0
Rps6kb1	0
Rps6ka6	0
Rps6ka5	0
Rps6ka2	0
Rps6ka1	0
Rps6	0
Rps4x	0
Rps3a1	0
Rps3	0
Rps29	0
Rps28	0
Rps27rt	0
Rps27l	0
Rps27a	0
Rps27	0
Rps26	0
Rps25	0
Rps24	0
Rps23	0
Rps21	0
Rps20	0
Rps2	0
Rps19bp1	0
Rps19	0
Rps18	0
Rps17	0
Rps16	0
Rps15a	0
Rps15	0
Rps14	0
Rps13	0
Rps12	0
Rps11	0
Rps10	0
Rprm	0
Rprd2	0
Rprd1a	0

Tfap2a	0
Tfam	0
Tex9	0
Tex40	0
Tex38	0
Tex37	0
Tex36	0
Tex33	0
Tex28	0
Tex264	0
Tex261	0
Tex26	0
Tex22	0
Tex21	0
Tex2	0
Tex19.2	0
Tex19.1	0
Tex16	0
Tex15	0
Tex13	0
Tex11	0
Tet2	0
Tet1	0
Tespa1	0
Tesk1	0
Tescl	0
Tesc	0
Tert	0
Terf2ip	0
Terf2	0
Terf1	0
Tepp	0
Tenm3	0
Tenm2	0
Tenm1	0
Telo2	0
Tekt5	0
Tekt4	0
Tekt3	0
Tekt2	0
Tekt1	0
Tek	0
Tefm	0
Tef	0
Teddm3	0
Tectb	0
Tecta	0
Tecr	0
Tecpr2	0
Tecpr1	0
Tead4	0
Tead3	0
Tdrd7	0
Tdrd6	0
Tdrd5	0
Tdrd12	0
Tdrd1	0
Tdpoz5	0
Tdpoz4	0
Tdpoz3	0
Tdpoz2	0
Tdpoz1	0
Tdp2	0
Tdo2	0
Tdgf1	0
Tdg	0
Tctn3	0
Tctn1	0
Tctex1d2	0
Tcte3	0
Tcte1	0
Tcstv1	0
Tcp11	0
Tcp10c	0
Tcp10b	0
Tcp1	0
Tcof1	0
Tcn2	0
Tcl1b4	0
Tcl1b2	0
Tcirg1	0
Tchp	0

Olfr702	0
Chga	0
Fam227b	0
Wnt16	0
G6pd2	0
Fam227a	0
Cndp1	0
Wnt11	0
Srsf12	0
Atp5c1	0
C1ql1	0
Kcnj1	0
C1ql3	0
Zdhhc11	0
Rhod	0
Zdhhc13	0
Zdhhc12	0
Kcnj9	0
Kcnj8	0
Zdhhc19	0
Zdhhc18	0
BRDN0000737465	0
Lgals9	0
Lgals8	0
Fosl1	0
Crem	0
Gm5934	0
Lgals3	0
Lgals1	0
Gnb1l	0
Lgals7	0
Erbb4	0
Lgals4	0
Bnipl	0
Zfp418	0
Gm614	0
Ins1	0
Zfp410	0
G2e3	0
Klk1b27	0
Zfp414	0
Olfr44	0
Rbm12	0
Olfr700	0
Rbm10	0
Rbm17	0
BRDN0000737463	0
Rbm14	0
Rbm19	0
Rbm18	0
Ppp2r1a	0
Zfx	0
Casp1	0
Trp53i13	0
Arnt	0
Gm10921	0
Zfr	0
Tcp11l2	0
Gm10922	0
Nadk2	0
Cep89	0
N6amt1	0
N6amt2	0
Dcx	0
Irx4	0
Gfpt1	0
Cep83	0
Sgip1	0
Cep85	0
Adgrg5	0
Twf2	0
Epc1	0
Tmsb15b1	0
Agmo	0
Cd164l2	0
Ccdc50	0
Lmo2	0
Ube2e1	0
Ube2e3	0
Ube2e2	0
Pex11a	0
Slc25a23	0

Tex38	0
Tex37	0
Tex36	0
Tex33	0
Tex29	0
Tex26	0
Tex22	0
Tex21	0
Tex2	0
Tex19.2	0
Tex16	0
Tex15	0
Tex14	0
Tex13	0
Tex101	0
Tex10	0
Tet2	0
Tespa1	0
Tesk2	0
Tescl	0
Tesc	0
Tert	0
Terf2ip	0
Terf1	0
Tepp	0
Tenm1	0
Ten1	0
Telo2	0
Tekt5	0
Tekt4	0
Tekt3	0
Tekt2	0
Tekt1	0
Tefm	0
Tef	0
Teddm1a	0
Tecta	0
Tecpr2	0
Tecpr1	0
Tec	0
Tead4	0
Tead3	0
Tead2	0
Tdrkh	0
Tdrd9	0
Tdrd7	0
Tdrd6	0
Tdrd5	0
Tdrd12	0
Tdpoz2	0
Tdpoz1	0
Tdp2	0
Tdp1	0
Tdo2	0
Tdh	0
Tdgf1	0
Tdg	0
Tctn3	0
Tctn1	0
Tctex1d1	0
Tcte1	0
Tcstv3	0
Tcstv1	0
Tcp11l2	0
Tcp11l1	0
Tcp11	0
Tcp10b	0
Tcp1	0
Tcof1	0
Tcl1b4	0
Tcl1b2	0
Tcl1	0
Tcirg1	0
Tchp	0
Tchhl1	0
Tchh	0
Tcf7	0
Tcf4	0
Tcf25	0
Tcf20	0
Tcf19	0
Tcf12	0

Rpp40	0	Tcfl5	0	Ptk2b	0	Tcerg1l	0
Rpp38	0	Tcf7l1	0	Slc25a26	0	Tcerg1	0
Rpp25l	0	Tcf7	0	Slc25a27	0	Tceb3	0
Rpp25	0	Tcf4	0	Slc25a24	0	Tceb2	0
Rpp21	0	Tcf3	0	Slc25a25	0	Tceb1	0
Rpp14	0	Tcf25	0	Sprr2a1	0	Tceanc	0
Rpn2	0	Tcf24	0	Eya4	0	Tceal8	0
Rpn1	0	Tcf20	0	Slc25a28	0	Tceal7	0
Rplp2	0	Tcerg1l	0	Sprr2a2	0	Tceal6	0
Rplp1	0	Tcerg1	0	Pcyt1a	0	Tceal3	0
Rplp0	0	Tceb2	0	Bnip1	0	Tceal1	0
Rpl8	0	Tceb1	0	Bnip2	0	Tcea3	0
Rpl7l1	0	Tceanc2	0	Bnip3	0	Tcea2	0
Rpl7	0	Tceanc	0	Pcsk1	0	Tcea1	0
Rpl6	0	Tceal8	0	Dhx8	0	Tcam1	0
Rpl5	0	Tceal7	0	Gm6460	0	Tcaim	0
Rpl4	0	Tceal6	0	Gm2897	0	Tcaf3	0
Rpl3l	0	Tceal5	0	Oasl2	0	Tcaf1	0
Rpl39l	0	Tceal3	0	Colec12	0	Tc2n	0
Rpl39	0	Tcea3	0	Taar8b	0	Tbxas1	0
Rpl38	0	Tcea2	0	Ckmt2	0	Tbx4	0
Rpl37a	0	Tcea1	0	Tmem261	0	Tbx21	0
Rpl37	0	Tcam1	0	Tas2r126	0	Tbx20	0
Rpl36al	0	Tcaim	0	Cbr4	0	Tbx2	0
Rpl36a	0	Tcaf3	0	Prkdc	0	Tbx19	0
Rpl36	0	Tcaf2	0	Zbtb38	0	Tbx10	0
Rpl35a	0	Tbxas1	0	Zfp28	0	Tbx1	0
Rpl35	0	Tbx6	0	Zbtb8b	0	Tbrg4	0
Rpl34	0	Tbx4	0	4930503E14Rik	0	Tbrg1	0
Rpl31	0	Tbx21	0	Adgrg3	0	Tbp	0
Rpl30	0	Tbx20	0	Prtn3	0	Tbl3	0
Rpl3	0	Tbx19	0	Etfa	0	Tbl1xr1	0
Rpl29	0	Tbx1	0	Olfr441	0	Tbl1x	0
Rpl28	0	Tbrg4	0	Olfr444	0	Tbck	0
Rpl27a	0	Tbrg1	0	Las1l	0	Tbcd	0
Rpl27	0	Tbr1	0	Olfr643	0	Tbccd1	0
Rpl26	0	Tbl2	0	Olfr448	0	Tbcc	0
Rpl24	0	Tbl1xr1	0	Olfr449	0	Tbcb	0
Rpl23a	0	Tbl1x	0	Olfr640	0	Tbca	0
Rpl23	0	Tbkbp1	0	Xdh	0	Tbc1d8b	0
Rpl22l1	0	Tbk1	0	Vmn1r125	0	Tbc1d8	0
Rpl21	0	Tbcel	0	Poglut1	0	Tbc1d7	0
Rpl19	0	Tbcd	0	Gpr137	0	Tbc1d5	0
Rpl18a	0	Tbccd1	0	Olfr137	0	Tbc1d32	0
Rpl18	0	Tbcc	0	Vmn1r121	0	Tbc1d30	0
Rpl17	0	Tbca	0	Vmn1r123	0	Tbc1d25	0
Rpl15	0	Tbc1d9b	0	Gpr132	0	Tbc1d23	0
Rpl14	0	Tbc1d9	0	Hibadh	0	Tbc1d22a	0
Rpl13	0	Tbc1d5	0	Olfr130	0	Tbc1d2	0
Rpl12	0	Tbc1d32	0	Vmn1r128	0	Tbc1d15	0
Rpl11	0	Tbc1d31	0	Olfr131	0	Tbc1d10b	0
Rpl10l	0	Tbc1d30	0	Colec10	0	Tbc1d10a	0
Rpl10a	0	Tbc1d2b	0	9130409I23Rik	0	Tbc1d1	0
Rpl10	0	Tbc1d25	0	Olfr644	0	Tbata	0
Rpia	0	Tbc1d24	0	Zfp27	0	Tax1bp3	0
Rph3al	0	Tbc1d22b	0	Capn15	0	Tatdn3	0
Rph3a	0	Tbc1d22a	0	Olfr645	0	Tat	0
Rpgrip1l	0	Tbc1d20	0	Capn13	0	Tasp1	0
Rpgr	0	Tbc1d19	0	Capn12	0	Tas2r144	0
Rpf2	0	Tbc1d17	0	Capn11	0	Tas2r140	0
Rpf1	0	Tbc1d16	0	Capn10	0	Tas2r138	0
Rpe	0	Tbc1d15	0	Frat2	0	Tas2r136	0
Rpap2	0	Tbc1d12	0	Dcpp3	0	Tas2r135	0
Rpain	0	Tbc1d10c	0	Atf4	0	Tas2r134	0
Rpa3	0	Tbc1d10a	0	Prrx1	0	Tas2r131	0
Rpa2	0	Tbc1d1	0	Prrx2	0	Tas2r130	0
Rpa1	0	Tax1bp3	0	Prkd2	0	Tas2r129	0
Rp9	0	Tax1bp1	0	Mtfmt	0	Tas2r126	0
Rp2h	0	Tatdn3	0	Bet1	0	Tas2r125	0
Rp1l1	0	Tatdn2	0	BRDN0000738337	0	Tas2r124	0
Rp1	0	Tatdn1	0	Tlk1	0	Tas2r123	0
Ros1	0	Tat	0	Vsnl1	0	Tas2r122	0
Rorc	0	Tasp1	0	Cbr1	0	Tas2r121	0
Rorb	0	Tas2r144	0	Smarca4	0	Tas2r117	0
Ror2	0	Tas2r140	0	Cggbp1	0	Tas2r116	0
Ror1	0	Tas2r139	0	BRDN0000738331	0	Tas2r113	0
Ropn1	0	Tas2r138	0	Etf1	0	Tas2r110	0
Romo1	0	Tas2r137	0	Ercc6l2	0	Tas2r108	0
Rom1	0	Tas2r135	0	Nanos1	0	Tas2r107	0
Rogdi	0	Tas2r134	0	Nacad	0	Tas2r106	0
Rock2	0	Tas2r130	0	Cmklr1	0	Tas2r105	0
Rock1	0	Tas2r129	0	Gcdh	0	Tas2r104	0

Robo3	0
Robo1	0
Rnps1	0
Rnpepl1	0
Rnpep	0
Rnpc3	0
Rnmtl1	0
Rnmt	0
Rnls	0
Rnh1	0
Rnft2	0
Rnf8	0
Rnf7	0
Rnf6	0
Rnf5	0
Rnf44	0
Rnf41	0
Rnf40	0
Rnf4	0
Rnf39	0
Rnf38	0
Rnf34	0
Rnf32	0
Rnf31	0
Rnf24	0
Rnf222	0
Rnf220	0
Rnf219	0
Rnf217	0
Rnf216	0
Rnf214	0
Rnf20	0
Rnf2	0
Rnf19b	0
Rnf19a	0
Rnf185	0
Rnf183	0
Rnf182	0
Rnf181	0
Rnf180	0
Rnf17	0
Rnf169	0
Rnf168	0
Rnf166	0
Rnf152	0
Rnf151	0
Rnf150	0
Rnf148	0
Rnf146	0
Rnf145	0
Rnf144a	0
Rnf141	0
Rnf14	0
Rnf139	0
Rnf138rt1	0
Rnf135	0
Rnf130	0
Rnf128	0
Rnf126	0
Rnf123	0
Rnf122	0
Rnf121	0
Rnf115	0
Rnf114	0
Rnf113a1	0
Rnf112	0
Rnf111	0
Rnf11	0
Rnf103	0
Rnf10	0
Rnd3	0
Rnd2	0
Rnd1	0
Rnaset2a	0
Rnasel	0
Rnasek	0
Rnaseh2c	0
Rnaseh2b	0
Rnaseh2a	0
Rnaseh1	0
Rnase9	0
Rnase6	0

Tas2r126	0
Tas2r125	0
Tas2r124	0
Tas2r121	0
Tas2r117	0
Tas2r116	0
Tas2r115	0
Tas2r114	0
Tas2r110	0
Tas2r109	0
Tas2r108	0
Tas2r107	0
Tas2r105	0
Tas2r104	0
Tas2r102	0
Tas1r2	0
Tarsl2	0
Tars	0
Tarm1	0
Tardbp	0
Tarbp2	0
Tapbpl	0
Taok3	0
Tango6	0
Tango2	0
Tanc1	0
Tal1	0
Tagln2	0
Tagap1	0
Taf9b	0
Taf8	0
Taf7l	0
Taf6l	0
Taf6	0
Taf5l	0
Taf5	0
Taf4b	0
Taf4a	0
Taf2	0
Taf1d	0
Taf1c	0
Taf1b	0
Taf15	0
Taf13	0
Taf12	0
Taf11	0
Taf10	0
Tada3	0
Tada2b	0
Tada1	0
Tacc3	0
Tacc2	0
Tacc1	0
Tac2	0
Tac1	0
Tab3	0
Tab2	0
Taar9	0
Taar8c	0
Taar8a	0
Taar7e	0
Taar6	0
Taar4	0
Taar2	0
Taar1	0
Szt2	0
Szrd1	0
Syvn1	0
Sytl5	0
Sytl4	0
Sytl3	0
Sytl2	0
Syt9	0
Syt7	0
Syt5	0
Syt16	0
Syt14	0
Syt13	0
Syt12	0
Syt11	0
Syt10	0
Sys1	0

Hpx	0
Bace1	0
Npas4	0
Npas2	0
Npas1	0
Olfr347	0
Hpn	0
Casp8	0
Vwa5b2	0
Vwa5b1	0
Il17rd	0
Il17rc	0
Il17rb	0
Il17ra	0
Hpd	0
Phox2b	0
Dnah7b	0
Nr6a1	0
Tagap	0
Dnah7a	0
1700080E11Rik	0
Gm14351	0
Olfr502	0
Rmnd5a	0
Olfm3	0
Pdilt	0
Olfm1	0
Nanos3	0
Casp9	0
Rmnd5b	0
Olfm4	0
Thoc3	0
Thoc2	0
Zc3h12c	0
Thoc7	0
Thoc6	0
Thoc5	0
B3galt4	0
B3galt5	0
B3galt6	0
Nr4a1	0
Raet1e	0
Raet1d	0
B3galt2	0
Wnk3	0
Wnk1	0
Pyhin1	0
Wnk4	0
Uso1	0
Ncstn	0
Hdac1	0
Tmem63a	0
Tmem63c	0
Tmem63b	0
Sntb2	0
Erbb2ip	0
Sntb1	0
Tfap2a	0
Scel	0
Tfap2c	0
Tfap2e	0
Tfap2d	0
Rhox11	0
Tpd52	0
Osbpl5	0
Chrne	0
Rer1	0
Wwc1	0
Pcbp3	0
Faim2	0
Faim3	0
Ncapg2	0
BRDN0000737701	0
2700094K13Rik	0
Gm8882	0
Dmap1	0
Fam160b1	0
Bcl6b	0
Eps8l3	0
Dph1	0
Olfr1219	0
Olfr1218	0

Tas2r103	0
Tas2r102	0
Tas1r3	0
Tas1r2	0
Tars	0
Tarm1	0
Tardbp	0
Tarbp2	0
Tapt1	0
Tapbp	0
Tap2	0
Tap1	0
Taok3	0
Taok1	0
Tango6	0
Tango2	0
Tanc2	0
Tanc1	0
Taldo1	0
Tal2	0
Tal1	0
Tagln3	0
Tagln2	0
Tagln	0
Tagap1	0
Tagap	0
Taf9b	0
Taf8	0
Taf7l	0
Taf7	0
Taf6l	0
Taf6	0
Taf5l	0
Taf4b	0
Taf4a	0
Taf2	0
Taf1d	0
Taf1c	0
Taf1b	0
Taf13	0
Taf12	0
Taf11	0
Taf10	0
Tada2b	0
Tada2a	0
Tada1	0
Tacstd2	0
Tacr3	0
Tacr2	0
Taco1	0
Tacc3	0
Tacc2	0
Tacc1	0
Tac4	0
Tac1	0
Tab1	0
Taar9	0
Taar8c	0
Taar8a	0
Taar7e	0
Taar7b	0
Taar7a	0
Taar5	0
Taar4	0
Taar3	0
Taar1	0
T	0
Szt2	0
Szrd1	0
Syvn1	0
Sytl3	0
Sytl1	0
Syt8	0
Syt6	0
Syt5	0
Syt4	0
Syt2	0
Syt17	0
Syt16	0
Syt15	0
Syt11	0
Sys1	0

Rnase4	0	Sypl	0	Dph5	0	Sypl2	0
Rnase2b	0	Syp	0	Dph6	0	Sypl	0
Rnase2a	0	Synrg	0	Dph7	0	Syp	0
Rnase13	0	Synpr	0	Olfr1213	0	Synrg	0
Rnase12	0	Synpo2l	0	Olfr1212	0	Synpr	0
Rnase11	0	Synpo	0	Cwc15	0	Synpo2l	0
Rnase10	0	Synm	0	BRDN0000737707	0	Synpo2	0
Rmnd5b	0	Synj2	0	Olfr1216	0	Synj2bp	0
Rmnd5a	0	Synj1	0	Olfr1215	0	Synj2	0
Rmnd1	0	Syngr4	0	Rph3a	0	Synj1	0
Rmi2	0	Syngr3	0	Defb50	0	Syngr4	0
Rmi1	0	Syngr2	0	Rab36	0	Syngr2	0
Rmdn3	0	Syngap1	0	BRDN0000737709	0	Syngr1	0
Rmdn2	0	Syne1	0	Msx1	0	Syngap1	0
Rmdn1	0	Syndig1l	0	Msx2	0	Syne4	0
Rltpr	0	Syncrip	0	Msx3	0	Syne1	0
Rln1	0	Sync	0	Abcc12	0	Syndig1	0
Rlim	0	Synb	0	Abcc10	0	Syncrip	0
Rita1	0	Sympk	0	Ccnd2	0	Sync	0
Rit2	0	Syf2	0	Zfp648	0	Syna	0
Rit1	0	Syde2	0	Csn2	0	Syn2	0
Ripply1	0	Syde1	0	Lbp	0	Sympk	0
Ripk4	0	Sycp3	0	Zfp644	0	Syk	0
Ripk3	0	Sycp1	0	Sh3tc2	0	Syde2	0
Ripk2	0	Sycn	0	Zfp646	0	Syde1	0
Ripk1	0	Syce3	0	Zfp647	0	Sycp3	0
Riok3	0	Syce2	0	Caml	0	Sycp2	0
Riok1	0	Syce1l	0	Pum2	0	Sycn	0
Rint1	0	Syce1	0	Pum1	0	Syce1l	0
Rinl	0	Sybu	0	Nr2e1	0	Sybu	0
Ring1	0	Syap1	0	Mertk	0	Syap1	0
Rin2	0	Swt1	0	BC048609	0	Swt1	0
Rin1	0	Swap70	0	Gm11541	0	Swsap1	0
Rims3	0	Svs6	0	Tnks2	0	Swap70	0
Rims2	0	Svs5	0	Npepps	0	Svs6	0
Rimkla	0	Svs4	0	Mdfi	0	Svs4	0
Rimbp3	0	Svs3b	0	Snx16	0	Svs3a	0
Rimbp2	0	Svs2	0	Snx15	0	Svs1	0
Rilpl2	0	Svs1	0	Snx14	0	Svopl	0
Rilpl1	0	Svop	0	Snx13	0	Svop	0
Rilp	0	Svip	0	Snx12	0	Svip	0
Riiad1	0	Svil	0	Snx11	0	Svil	0
Rif1	0	Svep1	0	Snx10	0	Svep1	0
Rictor	0	Sva	0	Zfp804b	0	Sval3	0
Ric8b	0	Sv2b	0	4921507P07Rik	0	Sval2	0
Ric8	0	Suz12	0	Agrp	0	Sval1	0
Ric3	0	Suv420h2	0	Olfr91	0	Sva	0
Ric1	0	Suv39h1	0	Agrn	0	Sv2b	0
Rhpn2	0	Susd4	0	Tmppe	0	Sv2a	0
Rhpn1	0	Susd3	0	Sftpa1	0	Suz12	0
Rhox9	0	Susd1	0	Scaf4	0	Suv420h2	0
Rhox8	0	Surf6	0	Artn	0	Suv39h2	0
Rhox4g	0	Surf4	0	Malsu1	0	Suv39h1	0
Rhox4f	0	Surf1	0	Rora	0	Susd2	0
Rhox4e	0	Supv3l1	0	Zfp955b	0	Surf4	0
Rhox4d	0	Supt7l	0	1700019A02Rik	0	Supt7l	0
Rhox4c	0	Supt6	0	Zfp955a	0	Supt6	0
Rhox4b	0	Supt5	0	Grip2	0	Supt5	0
Rhox4a	0	Supt3	0	Otogl	0	Supt4a	0
Rhox3h	0	Supt20	0	Dlx3	0	Supt3	0
Rhox3g	0	Supt16	0	Carm1	0	Supt20	0
Rhox3f	0	Suox	0	Dspp	0	Supt16	0
Rhox3e	0	Sun2	0	Olfr49	0	Sun3	0
Rhox3c	0	Sumo2	0	Mars	0	Sun1	0
Rhox3a	0	Sumo1	0	Olfr47	0	Sumo2	0
Rhox2f	0	Sumf1	0	Olfr46	0	Sumo1	0
Rhox2d	0	Sult6b1	0	Ear6	0	Sumf2	0
Rhox2c	0	Sult5a1	0	Grrp1	0	Sumf1	0
Rhox2b	0	Sult3a1	0	Ndc80	0	Sult6b1	0
Rhox13	0	Sult2b1	0	Ear1	0	Sult5a1	0
Rhox12	0	Sult2a6	0	Ear2	0	Sult3a1	0
Rhox10	0	Sult2a5	0	Ric8b	0	Sult2b1	0
Rhox1	0	Sult2a1	0	Lhx2	0	Sult2a6	0
Rhou	0	Sult1e1	0	Lhx3	0	Sult2a3	0
Rhot2	0	Sult1d1	0	Lrr1	0	Sult1d1	0
Rhot1	0	Sult1c1	0	Lhx6	0	Sult1c2	0
Rhoj	0	Sult1b1	0	Tnfrsf1a	0	Sult1c1	0
Rhoh	0	Sulf1	0	Lhx8	0	Sult1b1	0
Rhog	0	Sugt1	0	Zfp729b	0	Sulf2	0
Rhod	0	Sugp1	0	Sept7	0	Sulf1	0
Rhobtb3	0	Sugct	0	Mib1	0	Sugt1	0
Rhobtb2	0	Suds3	0	Mib2	0	Sugp1	0

Rhob	0	Sucnr1	0	Ppp1r3d	0	Sugct	0
Rhoa	0	Suclg2	0	Nhlh2	0	Sufu	0
Rho	0	Suclg1	0	1110037F02Rik	0	Suds3	0
Rhebl1	0	Sucla2	0	Art4	0	Suclg1	0
Rheb	0	Sub1	0	Zfat	0	Sucla2	0
Rhcg	0	Styx	0	Gm13769	0	Styk1	0
Rhbg	0	Stxbp6	0	Olfr172	0	Stxbp6	0
Rhbdl3	0	Stxbp5	0	Pole4	0	Stxbp5l	0
Rhbdl2	0	Stxbp4	0	Pabpc1	0	Stxbp5	0
Rhbdl1	0	Stxbp3a	0	Pabpc2	0	Stxbp4	0
Rhbdf2	0	Stxbp2	0	Il31	0	Stxbp3a	0
Rhbdf1	0	Stxbp1	0	Gstz1	0	Stxbp2	0
Rhbdd2	0	Stx8	0	Pabpc5	0	Stxbp1	0
Rgsl1	0	Stx7	0	C530008M17Rik	0	Stx5a	0
Rgs9bp	0	Stx6	0	Samd4	0	Stx3	0
Rgs9	0	Stx5a	0	Samd5	0	Stx2	0
Rgs8	0	Stx4a	0	Dtd2	0	Stx1b	0
Rgs7bp	0	Stx3	0	Stk3	0	Stx19	0
Rgs7	0	Stx1b	0	Stk4	0	Stx18	0
Rgs4	0	Stx1a	0	Samd1	0	Stx17	0
Rgs22	0	Stx18	0	Tnrc6a	0	Stx16	0
Rgs2	0	Stx16	0	Cpsf6	0	Stx12	0
Rgs19	0	Stx11	0	Cd70	0	Stx11	0
Rgs17	0	Stub1	0	Sema6d	0	Stub1	0
Rgs16	0	Strn4	0	Gmppb	0	Stt3a	0
Rgs13	0	Strip1	0	Sema6b	0	Strn4	0
Rgs12	0	Strbp	0	Sema6a	0	Strn	0
Rgs11	0	Stradb	0	Gm13119	0	Strip2	0
Rgs10	0	Stra8	0	Cpsf4	0	Strip1	0
Rgs1	0	Stra13	0	Cd72	0	Strc	0
Rgp1	0	Stpg2	0	Otulin	0	Strbp	0
Rgn	0	Stox2	0	Ggnbp1	0	Strap	0
Rgmb	0	Stox1	0	Has1	0	Stradb	0
Rgma	0	Ston1	0	Tnfrsf17	0	Strada	0
Rgl3	0	Stoml3	0	Tle3	0	Stra8	0
Rgl2	0	Stoml2	0	Pde4a	0	Stra6	0
Rgl1	0	Stoml1	0	Prap1	0	Stra13	0
Rgcc	0	Stmnd1	0	Mfsd3	0	Stpg1	0
Rgag4	0	Stmn3	0	Tnfrsf18	0	Stox2	0
Rfxank	0	Stmn2	0	Tnfrsf19	0	Stox1	0
Rfx8	0	Stkld1	0	Dcps	0	Ston2	0
Rfx7	0	Stk40	0	Olfr1253	0	Stoml2	0
Rfx6	0	Stk4	0	Gm436	0	Stom	0
Rfx5	0	Stk38l	0	Gml	0	Stmn4	0
Rfx4	0	Stk38	0	Gm438	0	Stmn3	0
Rfx2	0	Stk35	0	Pepd	0	Stmn2	0
Rfwd3	0	Stk33	0	Tkfc	0	Stkld1	0
Rfwd2	0	Stk32a	0	Serpinb9	0	Stk4	0
Rftn2	0	Stk31	0	Serpinb8	0	Stk39	0
Rftn1	0	Stk3	0	Clec14a	0	Stk38l	0
Rft1	0	Stk24	0	Rae1	0	Stk38	0
Rfpl4b	0	Stk19	0	Ada	0	Stk36	0
Rfng	0	Stk17b	0	Armc10	0	Stk35	0
Rfk	0	Stk16	0	Gm7257	0	Stk33	0
Rfesd	0	Stk11ip	0	Cnnm2	0	Stk32a	0
Rfc5	0	Stk11	0	Serpinb5	0	Stk31	0
Rfc4	0	Stk10	0	Adk	0	Stk26	0
Rfc3	0	Stip1	0	Serpinb7	0	Stk25	0
Rfc2	0	Stim2	0	Lrpprc	0	Stk19	0
Rfc1	0	Stim1	0	Adprhl2	0	Stk16	0
Rexo4	0	Stil	0	Gm3415	0	Stk11ip	0
Rexo1	0	Stfa2l1	0	Gm3417	0	Stk11	0
Rex2	0	Stfa2	0	Gapdhs	0	Stk10	0
Rev3l	0	Steap3	0	Actrt2	0	Stip1	0
Rev1	0	Steap2	0	Actrt3	0	Stim2	0
Retsat	0	Stc2	0	Gpbp1	0	Stim1	0
Retnlg	0	Stc1	0	Catsperd	0	Stfa2l1	0
Retnlb	0	Stbd1	0	Nptx2	0	Stfa2	0
Retnla	0	Stau2	0	Nptx1	0	Steap4	0
Retn	0	Stau1	0	4930557A04Rik	0	Steap3	0
Ret	0	Stat6	0	BRDN0000737906	0	Steap2	0
Rest	0	Stat5b	0	Onecut2	0	Stc2	0
Resp18	0	Stat5a	0	BRDN0000737904	0	Stc1	0
Rergl	0	Stat4	0	BRDN0000737905	0	Stbd1	0
Rerg	0	Stat3	0	BRDN0000737902	0	Stau2	0
Rer1	0	Stat1	0	BRDN0000737903	0	Stau1	0
Reps2	0	Stard8	0	BRDN0000737900	0	Stat6	0
Repin1	0	Stard6	0	BRDN0000737901	0	Stat5b	0
Rep15	0	Stard5	0	Bag2	0	Stat5a	0
Ren1	0	Stard13	0	Dcp2	0	Stat4	0
Rem2	0	Star	0	Olfr389	0	Stat3	0
Relt	0	Stap2	0	Obp2a	0	Stat1	0

Reln	0	Stap1	0	BRDN0000737652	0	Stard8	0
Rell2	0	Stambp	0	Gpr146	0	Stard7	0
Relb	0	Stam2	0	Bag5	0	Stard6	0
Rela	0	Stam	0	G6b	0	Stard5	0
Rel	0	Stag3	0	Olfr382	0	Stard4	0
Reg4	0	Stag2	0	Olfr381	0	Stard3nl	0
Reg3g	0	Stag1	0	Olfr380	0	Stard3	0
Reg3b	0	Stac3	0	Lrch3	0	Stard13	0
Reg3a	0	Stac	0	BRDN0000738168	0	Stard10	0
Reg1	0	Stab1	0	Olfr385	0	Star	0
Reep6	0	St8sia6	0	Olfr384	0	Stap2	0
Reep5	0	St8sia5	0	Rasl2-9	0	Stap1	0
Reep2	0	St8sia4	0	Gm9	0	Stambpl1	0
Reep1	0	St8sia3	0	Tnf	0	Stambp	0
Recql4	0	St8sia2	0	Nr1i3	0	Stam	0
Recql	0	St8sia1	0	Nudt22	0	Stag3	0
Rec8	0	St7	0	Slc35e1	0	Stag2	0
Rec114	0	St6galnac5	0	Slc35e2	0	Stag1	0
Rdh9	0	St6galnac4	0	Slc35e3	0	Stac3	0
Rdh8	0	St6galnac2	0	Sap130	0	Stac2	0
Rdh5	0	St6gal1	0	Fam111a	0	Stab2	0
Rdh19	0	St5	0	Ccnl1	0	St8sia5	0
Rdh16	0	St3gal6	0	Ggact	0	St8sia4	0
Rdh14	0	St3gal4	0	Lyg1	0	St8sia3	0
Rdh13	0	St3gal3	0	Lyg2	0	St8sia2	0
Rdh12	0	St3gal2	0	Rab3gap1	0	St8sia1	0
Rdh11	0	St3gal1	0	Pdcl	0	St7l	0
Rdh1	0	St18	0	Tmem8	0	St6galnac6	0
Rd3l	0	St14	0	Tmem9	0	St6galnac4	0
Rcvrn	0	St13	0	Gm21541	0	St6galnac3	0
Rcsd1	0	Ssxb9	0	Rabep2	0	St6galnac1	0
Rcor1	0	Ssxb8	0	Adgrg2	0	St6gal1	0
Rcn3	0	Ssxb3	0	Adgrg1	0	St5	0
Rcn1	0	Ssxb2	0	Rabep1	0	St3gal5	0
Rcl1	0	Ssxb10	0	Tmem2	0	St3gal2	0
Rce1	0	Ssxb1	0	Tmem5	0	Ssxb9	0
Rcc1	0	Ssx9	0	Rps26	0	Ssxb8	0
Rcbtb1	0	Ssx2ip	0	Rps27	0	Ssxb3	0
Rcan3	0	Ssu72	0	Rps24	0	Ssxb2	0
Rcan2	0	Ssu2	0	Rps25	0	Ssxb10	0
Rcan1	0	Ssty1	0	BRDN0000738305	0	Ssxb1	0
Rc3h2	0	Sstr5	0	Rps23	0	Ssx9	0
Rc3h1	0	Sstr4	0	Rps20	0	Ssx2ip	0
Rbx1	0	Sstr2	0	Rps21	0	Ssu72	0
Rbsn	0	Sstr1	0	BRDN0000738309	0	Ssu2	0
Rbpms	0	Sst	0	Mis18bp1	0	Ssty1	0
Rbpjl	0	Sssca1	0	Rps28	0	Sstr5	0
Rbpj	0	Ssrp1	0	Rps29	0	Sstr4	0
Rbp7	0	Ssr4	0	4933406M09Rik	0	Sstr2	0
Rbp4	0	Ssr2	0	Esd	0	Sstr1	0
Rbp2	0	Sspo	0	Cdc42bpg	0	Sst	0
Rbmxl2	0	Sspn	0	Kcnf1	0	Sssca1	0
Rbmxl1	0	Ssna1	0	BRDN0000738124	0	Ssrp1	0
Rbmx2	0	Ssmem1	0	AW209491	0	Ssr4	0
Rbmx	0	Ssh2	0	Lcp2	0	Ssr3	0
Rbms3	0	Ssc5d	0	Lcp1	0	Ssmem1	0
Rbms1	0	Ssc4d	0	Gsta3	0	Ssfa2	0
Rbm8a	0	Ssbp4	0	Rpp25l	0	Ssbp3	0
Rbm6	0	Ssbp3	0	Fam162a	0	Ssbp2	0
Rbm5	0	Ssbp2	0	Fam69c	0	Ssbp1	0
Rbm4b	0	Ssbp1	0	Serpina3a	0	Ssb	0
Rbm48	0	Ssb	0	4930548H24Rik	0	Ss18	0
Rbm47	0	Ss18	0	Plekha8	0	Sry	0
Rbm45	0	Sry	0	Nfkbia	0	Srxn1	0
Rbm43	0	Srxn1	0	Nwd1	0	Srsf7	0
Rbm42	0	Srsf9	0	Chuk	0	Srsf6	0
Rbm4	0	Srsf7	0	Mmp25	0	Srsf5	0
Rbm39	0	Srsf5	0	Nwd2	0	Srsf3	0
Rbm38	0	Srsf4	0	Arhgdig	0	Srsf12	0
Rbm34	0	Srsf3	0	Golgb1	0	Srsf11	0
Rbm33	0	Srsf2	0	Padi2	0	Srsf10	0
Rbm3	0	Srsf11	0	Yif1b	0	Srsf1	0
Rbm28	0	Srsf10	0	Smim13	0	Srrt	0
Rbm27	0	Srsf1	0	Arhgdia	0	Srrm1	0
Rbm26	0	Srrt	0	Jdp2	0	Srrd	0
Rbm25	0	Srrm4	0	Gpr142	0	Srpx2	0
Rbm24	0	Srrm3	0	Padi4	0	Srpx	0
Rbm22	0	Srrd	0	Hhipl1	0	Srpr	0
Rbm20	0	Srr	0	Wscd1	0	Srpk2	0
Rbm19	0	Srpx2	0	Hhipl2	0	Srpk1	0
Rbm18	0	Srpx	0	Gm20604	0	Srp9	0
Rbm17	0	Srprb	0	5430401F13Rik	0	Srp72	0

Rbm15b	0	Srpr	0	Pate4	0	Srp68	0
Rbm15	0	Srpk3	0	Cetn3	0	Srp54b	0
Rbm14	0	Srpk2	0	Hrh1	0	Srp54a	0
Rbm12b2	0	Srp9	0	Ap2s1	0	Srm	0
Rbm12b1	0	Srp68	0	Olfr1273-ps	0	Sri	0
Rbm12	0	Srp54b	0	Clca3b	0	Srgn	0
Rbm10	0	Srp19	0	Fam131b	0	Srgap3	0
Rbfox2	0	Srms	0	Olfr1510	0	Srgap1	0
Rbfa	0	Srm	0	Tex13	0	Srfbp1	0
Rbck1	0	Sri	0	Olfr1512	0	Srf	0
Rbbp9	0	Srgn	0	Oxtr	0	Srebf1	0
Rbbp8nl	0	Srgap3	0	Tff3	0	Srd5a3	0
Rbbp7	0	Srgap2	0	Fxyd6	0	Srd5a1	0
Rbbp6	0	Srgap1	0	Ccnd1	0	Srcin1	0
Rbbp5	0	Srfbp1	0	Rnmt	0	Srcap	0
Rbbp4	0	Srf	0	Bud13	0	Srbd1	0
Rbak	0	Srek1	0	A830080D01Rik	0	Sqstm1	0
Rb1cc1	0	Srebf1	0	Ybey	0	Sqle	0
Rb1	0	Srd5a1	0	Ppapdc3	0	Spz1	0
Rax	0	Srcin1	0	Lsm14a	0	Spty2d1	0
Raver2	0	Srcap	0	Cdc42bpb	0	Sptssb	0
Rassf8	0	Srbd1	0	Olfr655	0	Sptssa	0
Rassf7	0	Sqrdl	0	Fancf	0	Sptlc3	0
Rassf4	0	Sqle	0	Olfr125	0	Sptlc2	0
Rassf3	0	Spz1	0	Fancb	0	Sptlc1	0
Rassf10	0	Spty2d1	0	Fanca	0	Sptbn4	0
Rassf1	0	Sptssa	0	Grm5	0	Sptb	0
Rasl2-9	0	Sptlc3	0	Fancm	0	Sptan1	0
Rasl12	0	Sptlc2	0	Fancl	0	Spt1	0
Rasl11b	0	Sptbn2	0	Dennd6b	0	Spsb4	0
Rasl10b	0	Sptan1	0	Fanci	0	Spsb2	0
Rasip1	0	Spsb4	0	Dennd6a	0	Spryd7	0
Rasgrp4	0	Spsb2	0	Qrsl1	0	Spryd4	0
Rasgrp3	0	Spsb1	0	Atp8a1	0	Spry4	0
Rasgrp2	0	Spryd7	0	4933406J08Rik	0	Spry2	0
Rasgrp1	0	Spry4	0	Micall1	0	Sprtn	0
Rasgrf1	0	Sprtn	0	Maob	0	Sprr4	0
Rasgef1c	0	Sprr2f	0	Maoa	0	Sprr3	0
Rasgef1b	0	Sprr2e	0	Atp5sl	0	Sprr2k	0
Rasgef1a	0	Sprr2d	0	1700003E16Rik	0	Sprr2i	0
Rasef	0	Sprr2b	0	Slc35e4	0	Sprr2f	0
Rasd2	0	Sprr2a2	0	Olfr1463	0	Sprr2e	0
Rasd1	0	Sprr1b	0	Dbh	0	Sprr2b	0
Rasal2	0	Sprr1a	0	Atp4a	0	Sprr2a1	0
Rasal1	0	Sprn	0	H2-M9	0	Sprr1b	0
Rasa4	0	Spred2	0	Med1	0	Sprr1a	0
Rasa2	0	Sppl3	0	Sirpb1a	0	Sprn	0
Rasa1	0	Sppl2c	0	Fam149a	0	Spred2	0
Rars2	0	Sppl2b	0	Mmp21	0	Sppl2b	0
Rars	0	Sppl2a	0	H2-M1	0	Spp2	0
Rarres2	0	Spp2	0	H2-M3	0	Spp1	0
Rarres1	0	Spp1	0	H2-M2	0	Spopl	0
Rarg	0	Spopl	0	H2-M5	0	Spop	0
Rarb	0	Spon2	0	Atg4c	0	Spon2	0
Rara	0	Spon1	0	Atg4b	0	Spock3	0
Raph1	0	Spock3	0	Mynn	0	Spock2	0
Rapgefl1	0	Spock2	0	Olfr1368	0	Spock1	0
Rapgef6	0	Spo11	0	Cox6b1	0	Spo11	0
Rapgef5	0	Spns3	0	Tmcc1	0	Spns3	0
Rapgef4	0	Spns2	0	Cd109	0	Spns1	0
Rapgef3	0	Spns1	0	Rtn4r	0	Spn	0
Rapgef1	0	Spn	0	Snx3	0	Spint5	0
Rap2c	0	Spire2	0	Gm15093	0	Spint4	0
Rap2a	0	Spint5	0	Snx1	0	Spint3	0
Rap1gds1	0	Spint4	0	Gm15091	0	Spint2	0
Rap1gap	0	Spint3	0	Snx7	0	Spint1	0
Rap1b	0	Spint1	0	Gm15097	0	Spinkl	0
Rap1a	0	Spinkl	0	A630076J17Rik	0	Spink8	0
Rangrf	0	Spink8	0	Vmn1r57	0	Spink7	0
Rangap1	0	Spink7	0	Snx9	0	Spink5	0
Ranbp2	0	Spink6	0	Inca1	0	Spink4	0
Ranbp17	0	Spink5	0	Osgepl1	0	Spink3	0
Ranbp10	0	Spink4	0	Cks1b	0	Spink2	0
Ranbp1	0	Spink3	0	Pex2	0	Spink14	0
Ran	0	Spink2	0	Krt4	0	Spink13	0
Ramp1	0	Spink14	0	Fxyd2	0	Spink12	0
Ralyl	0	Spink12	0	Ifnz	0	Spink11	0
Raly	0	Spink10	0	Syne1	0	Spink10	0
Ralgps2	0	Spin2d	0	Fam170a	0	Spin4	0
Ralgps1	0	Spin2c	0	Syne2	0	Spin2d	0
Ralgapa2	0	Spin1	0	Wtip	0	Spin2c	0
Ralgapa1	0	Spidr	0	Pex7	0	Spin1	0

Ralbp1	0
Ralb	0
Rala	0
Rai2	0
Rai14	0
Rai1	0
Rag2	0
Rag1	0
Raf1	0
Raet1e	0
Raet1d	0
Rae1	0
Radil	0
Rad9b	0
Rad9a	0
Rad54l2	0
Rad54l	0
Rad54b	0
Rad52	0
Rad51d	0
Rad51c	0
Rad51ap2	0
Rad51	0
Rad50	0
Rad23b	0
Rad23a	0
Rad21l	0
Rad21	0
Rad18	0
Rad17	0
Rad1	0
Racgap1	0
Rac3	0
Rac1	0
Rabl3	0
Rabif	0
Rabggtb	0
Rabggta	0
Rabgef1	0
Rabgap1l	0
Rabgap1	0
Rabep2	0
Rabep1	0
Rabac1	0
Rab9b	0
Rab9	0
Rab8a	0
Rab7	0
Rab6a	0
Rab5b	0
Rab5a	0
Rab4b	0
Rab4a	0
Rab44	0
Rab42	0
Rab40c	0
Rab40b	0
Rab3il1	0
Rab3gap2	0
Rab3d	0
Rab3c	0
Rab3a	0
Rab39	0
Rab37	0
Rab36	0
Rab35	0
Rab34	0
Rab33b	0
Rab33a	0
Rab32	0
Rab30	0
Rab2b	0
Rab2a	0
Rab29	0
Rab28	0
Rab27b	0
Rab27a	0
Rab26	0
Rab25	0
Rab24	0
Rab23	0
Rab21	0
Spice1	0
Spic	0
Spi1	0
Sphkap	0
Sphk1	0
Spg7	0
Spg21	0
Spg20	0
Spen	0
Spem1	0
Speg	0
Spef1	0
Speer4e	0
Speer4d	0
Speer4c	0
Speer4b	0
Speer3	0
Specc1	0
Spdyb	0
Spdya	0
Spdl1	0
Spdef	0
Spcs3	0
Spcs2	0
Spc24	0
Spats2l	0
Spats2	0
Spats1	0
Spatc1l	0
Spata9	0
Spata7	0
Spata6	0
Spata33	0
Spata32	0
Spata31d1c	0
Spata31d1b	0
Spata31	0
Spata3	0
Spata2l	0
Spata24	0
Spata22	0
Spata21	0
Spata20	0
Spata17	0
Spata13	0
Sparcl1	0
Spam1	0
Spag9	0
Spag8	0
Spag7	0
Spag6l	0
Spag5	0
Spag4	0
Spag17	0
Spag16	0
Spag11a	0
Spag1	0
Spaca7	0
Spaca6	0
Spaca1	0
Spa17	0
Sp8	0
Sp5	0
Sp3	0
Sp110	0
Sp100	0
Sox9	0
Sox8	0
Sox6	0
Sox5	0
Sox30	0
Sox21	0
Sox2	0
Sox18	0
Sox17	0
Sox14	0
Sox13	0
Sox12	0
Sox11	0
Sox1	0
Sowahd	0
Sowaha	0
Ift80	0
Ift81	0
Slfn14	0
Ybx1	0
Vmn1r51	0
Ift88	0
Dapl1	0
Tnip2	0
Tnip3	0
Stk33	0
Stk35	0
Stk36	0
Stk38	0
Ell2	0
Depdc1b	0
Ddb1	0
Gm14305	0
Shmt1	0
1700008O03Rik	0
Pim2	0
Pim3	0
Wbscr17	0
Pim1	0
3110009E18Rik	0
Gm15107	0
Pla2g16	0
Gpr149	0
Abca13	0
Epha10	0
Pla2g10	0
Npff	0
Reg1	0
Rbbp8nl	0
Bcl2l1	0
Azi2	0
Bcl2l2	0
Isoc1	0
Nts	0
Gm11565	0
Fxyd1	0
Sec24d	0
BRDN0000738117	0
Sec24a	0
Sec24c	0
Sec24b	0
Fgfr4	0
Myd88	0
Fgfr2	0
Fgfr3	0
Fgfr1	0
Mvp	0
Ccdc89	0
Stkld1	0
Ccdc83	0
Ccdc82	0
Ccdc81	0
Ccdc80	0
Ccdc87	0
Ccdc86	0
Ccdc84	0
Tango6	0
Osmr	0
Mvd	0
Tango2	0
Tgfb1i1	0
Psmc1	0
Pde4b	0
Psmc3	0
Psmc4	0
Psmc5	0
Dolk	0
Nr2c2	0
Rpl37	0
BRDN0000737389	0
En1	0
Zfp781	0
Aqr	0
Hsp90b1	0
Zfp786	0
Trmt5	0
Rapgefl1	0
Gdf11	0
Spidr	0
Spice1	0
Spic	0
Spib	0
Spi1	0
Sphk2	0
Sphk1	0
Spg7	0
Spg21	0
Spg11	0
Spesp1	0
Spert	0
Spen	0
Speg	0
Spef1	0
Speer4f	0
Speer4e	0
Speer4d	0
Speer4c	0
Speer4b	0
Speer3	0
Specc1l	0
Specc1	0
Spdyb	0
Spdya	0
Spdl1	0
Spcs3	0
Spcs2	0
Spc24	0
Spats2	0
Spatc1l	0
Spatc1	0
Spata9	0
Spata7	0
Spata5	0
Spata45	0
Spata4	0
Spata33	0
Spata32	0
Spata31d1c	0
Spata31d1b	0
Spata31	0
Spata25	0
Spata24	0
Spata21	0
Spata20	0
Spata2	0
Spata18	0
Spata17	0
Spata16	0
Spata13	0
Spata1	0
Spast	0
Sparc	0
Spam1	0
Spag9	0
Spag8	0
Spag7	0
Spag6	0
Spag5	0
Spag4	0
Spag17	0
Spag16	0
Spag11a	0
Spag1	0
Spaca4	0
Spaca1	0
Spa17	0
Sp8	0
Sp6	0
Sp4	0
Sp3	0
Sp140	0
Sp100	0
Sp1	0
Sox9	0
Sox7	0
Sox30	0
Sox3	0
Sox18	0
Sox17	0
Sox15	0

Rab20	0
Rab1b	0
Rab19	0
Rab18	0
Rab17	0
Rab12	0
Rab11fip5	0
Rab11fip4	0
Rab11fip3	0
Rab11fip1	0
Rab11b	0
Rab11a	0
Rab10	0
Rab1	0
R3hdm4	0
R3hdm1	0
R3hcc1	0
Qtrtd1	0
Qtrt1	0
Qser1	0
Qrsl1	0
Qrich2	0
Qrich1	0
Qrfpr	0
Qrfp	0
Qprt	0
Qpctl	0
Qk	0
Qars	0
Pzp	0
Pyurf	0
Pyroxd2	0
Pyroxd1	0
Pygo2	0
Pygm	0
Pygl	0
Pygb	0
Pycrl	0
Pycr2	0
Pycr1	0
Pycard	0
Pxylp1	0
Pxt1	0
Pxn	0
Pxmp4	0
Pxmp2	0
Pxk	0
Pxdc1	0
Pwwp2b	0
Pwwp2a	0
Pwp2	0
Pwp1	0
Pvrl1	0
Pusl1	0
Pus7	0
Pus10	0
Pus1	0
Purb	0
Pura	0
Pum2	0
Pum1	0
Puf60	0
Ptx4	0
Ptx3	0
Ptrhd1	0
Ptrh2	0
Ptrh1	0
Ptpru	0
Ptprt	0
Ptprs	0
Ptprr	0
Ptprq	0
Ptpro	0
Ptprn2	0
Ptprm	0
Ptprk	0
Ptprj	0
Ptprg	0
Ptprf	0
Ptpre	0
Ptprcap	0
Ptprc	0
Sostdc1	0
Sost	0
Sos2	0
Sord	0
Sorcs3	0
Sorcs2	0
Sorcs1	0
Sorbs2	0
Sorbs1	0
Sohlh2	0
Sohlh1	0
Soga1	0
Sod3	0
Sod2	0
Sod1	0
Socs4	0
Socs3	0
Socs2	0
Socs1	0
Sobp	0
Soat1	0
Snx8	0
Snx6	0
Snx5	0
Snx33	0
Snx31	0
Snx3	0
Snx27	0
Snx25	0
Snx24	0
Snx22	0
Snx2	0
Snx19	0
Snx18	0
Snx16	0
Snx14	0
Snx13	0
Snx11	0
Snx10	0
Snx1	0
Snw1	0
Snurf	0
Snupn	0
Sntn	0
Sntg2	0
Sntb1	0
Snrpg	0
Snrpf	0
Snrpe	0
Snrpd3	0
Snrpd2	0
Snrpd1	0
Snrpc	0
Snrpb	0
Snrpa1	0
Snrpa	0
Snrnp70	0
Snrnp48	0
Snrnp40	0
Snrnp35	0
Snrnp25	0
Snrnp200	0
Snph	0
Snn	0
Snip1	0
Snf8	0
Snd1	0
Sncg	0
Sncb	0
Sncaip	0
Snapin	0
Snapc5	0
Snapc4	0
Snapc3	0
Snapc2	0
Snapc1	0
Snap91	0
Snap29	0
Snap25	0
Snap23	0
Snai3	0
Snai1	0
Teddm2	0
Teddm3	0
BRDN0000738070	0
Canx	0
Ttc9b	0
Arl9	0
Kcmf1	0
Iars2	0
Efnb1	0
Got1l1	0
9130023H24Rik	0
Thap1	0
Mrps35	0
Mrps34	0
Atp5a1	0
Mrps36	0
Mrps31	0
Mrps30	0
Mrps33	0
Rfpl3s	0
Zfp84	0
Zfp87	0
Efhd2	0
Zfp81	0
Gm13057	0
Mtnr1b	0
Cript	0
Zfp438	0
B020004C17Rik	0
Gchfr	0
Sepp1	0
Sel1l	0
Actg1	0
Actg2	0
Chrnb1	0
Chrnb3	0
Chrnb4	0
Plekha2	0
Fto	0
Olfr1442	0
Scamp5	0
Olfr767	0
Gnb4	0
Nlrc4	0
Ago4	0
Lars2	0
Ago1	0
Ago3	0
Ago2	0
Rgs16	0
Crispld1	0
Vbp1	0
Ppp2r3a	0
Fxyd4	0
C1qbp	0
Lca5l	0
Arhgap26	0
Arhgap25	0
Arhgap24	0
Sirt7	0
Arhgap29	0
Crip1	0
Msantd1	0
Crip3	0
Msantd3	0
Arrdc4	0
BRDN0000737826	0
2810417H13Rik	0
Car5a	0
Car5b	0
BRDN0000737569	0
Dhfr	0
Hspb1	0
Efhd1	0
Adam25	0
Adam24	0
Adam21	0
Adam20	0
Adam23	0
Adam22	0
Gipr	0
Ifit2	0
Sox13	0
Sox12	0
Sox11	0
Sox10	0
Sox1	0
Sowaha	0
Sost	0
Sos2	0
Sos1	0
Sort1	0
Sord	0
Sorcs3	0
Sorbs3	0
Sorbs2	0
Sorbs1	0
Sohlh2	0
Sohlh1	0
Soga3	0
Soga1	0
Sod3	0
Sod2	0
Socs7	0
Socs6	0
Socs5	0
Socs4	0
Socs3	0
Socs1	0
Soat1	0
Snx9	0
Snx7	0
Snx6	0
Snx5	0
Snx31	0
Snx30	0
Snx3	0
Snx29	0
Snx24	0
Snx22	0
Snx21	0
Snx20	0
Snx2	0
Snx19	0
Snx18	0
Snx17	0
Snx14	0
Snx12	0
Snx11	0
Snx10	0
Snx1	0
Snw1	0
Snurf	0
Snupn	0
Sntb1	0
Snta1	0
Snrpn	0
Snrpg	0
Snrpf	0
Snrpe	0
Snrpd3	0
Snrpd2	0
Snrpd1	0
Snrpc	0
Snrpb	0
Snrpa1	0
Snrpa	0
Snrnp70	0
Snrnp40	0
Snrnp35	0
Snrnp27	0
Snrnp25	0
Snrk	0
Snn	0
Snip1	0
Snf8	0
Sned1	0
Snd1	0
Sncg	0
Sncb	0
Snca	0
Snapc5	0
Snapc4	0
Snapc2	0

Ptprb	0	Smyd5	0	Ifit1	0	Snap91	0
Ptpra	0	Smyd4	0	Gm1587	0	Snap47	0
Ptpn9	0	Smyd2	0	Errfi1	0	Snap29	0
Ptpn7	0	Smyd1	0	Inip	0	Snap23	0
Ptpn6	0	Smurf2	0	Mthfsd	0	Snai3	0
Ptpn5	0	Smurf1	0	Snd1	0	Snai2	0
Ptpn4	0	Smug1	0	Synb	0	Snai1	0
Ptpn23	0	Smu1	0	Proca1	0	Smyd5	0
Ptpn22	0	Smtnl1	0	Mthfsl	0	Smyd4	0
Ptpn21	0	Smtn	0	Vkorc1l1	0	Smyd2	0
Ptpn20	0	Sms	0	Hspb8	0	Smyd1	0
Ptpn2	0	Smr3a	0	Sfmbt1	0	Smurf1	0
Ptpn18	0	Smr2	0	Fam25c	0	Smug1	0
Ptpn14	0	Smpx	0	Lsmem1	0	Smu1	0
Ptpn13	0	Smpdl3a	0	Lrrc75a	0	Smtnl1	0
Ptpn12	0	Smpd4	0	Gm21950	0	Smtn	0
Ptpn11	0	Smpd2	0	Gm21951	0	Sms	0
Ptpn1	0	Smpd1	0	Ddx23	0	Smpx	0
Ptpdc1	0	Smox	0	Ddx21	0	Smpdl3b	0
Ptp4a3	0	Smok3b	0	Ddx20	0	Smpd5	0
Ptp4a2	0	Smok2b	0	Ddx27	0	Smpd4	0
Ptov1	0	Smok2a	0	Ddx25	0	Smox	0
Ptn	0	Smoc2	0	Ddx24	0	Smok3b	0
Ptms	0	Smo	0	Paox	0	Smok3a	0
Ptma	0	Smndc1	0	Olfr463	0	Smok2b	0
Ptk7	0	Smn1	0	Dirc2	0	Smok2a	0
Ptk6	0	Smlr1	0	Olfr466	0	Smoc2	0
Ptk2	0	Smim9	0	Olfr467	0	Smndc1	0
Pthlh	0	Smim8	0	Olfr464	0	Smn1	0
Pth2	0	Smim5	0	Pdcd11	0	Smlr1	0
Pth1r	0	Smim3	0	Gpr119	0	Smim9	0
Pth	0	Smim23	0	Mxra8	0	Smim6	0
Ptgs2	0	Smim22	0	Dyrk1b	0	Smim5	0
Ptgr2	0	Smim20	0	Olfr570	0	Smim3	0
Ptgr1	0	Smim19	0	Syn3	0	Smim24	0
Ptgis	0	Smim15	0	1110017D15Rik	0	Smim23	0
Ptgir	0	Smim14	0	Lrrn4	0	Smim22	0
Ptges3l	0	Smim13	0	2310050C09Rik	0	Smim20	0
Ptges3	0	Smim12	0	Iqub	0	Smim19	0
Ptges2	0	Smim11	0	Lrrn2	0	Smim18	0
Ptges	0	Smim1	0	Iws1	0	Smim14	0
Ptger4	0	Smgc	0	Adrb1	0	Smim11	0
Ptger3	0	Smg8	0	Adrb2	0	Smim1	0
Ptger2	0	Smg7	0	Adrb3	0	Smgc	0
Ptger1	0	Smg6	0	Il12a	0	Smg8	0
Ptgdr2	0	Smg5	0	Nell1	0	Smg7	0
Ptgdr	0	Smg1	0	Copb1	0	Smg6	0
Ptf1a	0	Smek1	0	Tubd1	0	Smg5	0
Pten	0	Smdt1	0	Copb2	0	Smek2	0
Ptdss2	0	Smcr8	0	Ccbl1	0	Smek1	0
Ptdss1	0	Smco2	0	Nell2	0	Smdt1	0
Ptcra	0	Smco1	0	BRDN0000738111	0	Smcr8	0
Ptchd4	0	Smc6	0	Ccbl2	0	Smco4	0
Ptchd3	0	Smc5	0	a	0	Smco3	0
Ptchd2	0	Smc4	0	Dcaf12l2	0	Smco1	0
Ptchd1	0	Smc3	0	Dcaf12l1	0	Smc6	0
Ptch2	0	Smc2	0	Apbb1ip	0	Smc4	0
Ptch1	0	Smc1b	0	B4galnt1	0	Smc3	0
Ptcd3	0	Smc1a	0	B4galnt2	0	Smc2	0
Ptcd2	0	Smarce1	0	B4galnt3	0	Smc1b	0
Ptcd1	0	Smarcd3	0	Lrrd1	0	Smc1a	0
Ptbp2	0	Smarcd2	0	Birc5	0	Smarce1	0
Ptbp1	0	Smarcc2	0	Birc3	0	Smarcd1	0
Ptar1	0	Smarcc1	0	Birc2	0	Smarcc2	0
Ptafr	0	Smarcb1	0	Lsm10	0	Smarcb1	0
Pstpip2	0	Smarcal1	0	Eef1b2	0	Smarcal1	0
Pstpip1	0	Smarca5	0	Ctu2	0	Smarcad1	0
Pstk	0	Smarca4	0	Gnptab	0	Smarca5	0
Psrc1	0	Smarca2	0	Lurap1l	0	Smarca4	0
Pspn	0	Smarca1	0	Ctu1	0	Smarca2	0
Psph	0	Smagp	0	Tex13a	0	Smarca1	0
Psors1c2	0	Smad9	0	Pkd2l2	0	Smap1	0
Psmg4	0	Smad6	0	Pkd2l1	0	Smagp	0
Psmg3	0	Smad5	0	BRDN0000738347	0	Smad9	0
Psmg2	0	Smad4	0	Zfp747	0	Smad6	0
Psme4	0	Smad3	0	Trim44	0	Smad5	0
Psme2	0	Smad1	0	Trim45	0	Smad4	0
Psme1	0	Sly	0	Rap1gap2	0	Smad3	0
Psmd8	0	Slxl1	0	Syn1	0	Smad2	0
Psmd7	0	Slx4	0	Trim40	0	Smad1	0
Psmd6	0	Slx1b	0	Trim41	0	Slxl1	0
Psmd5	0	Slx	0	Cab39l	0	Slx4ip	0

Psmd4	0	Slurp1	0	Nfat5	0	Slx1b	0
Psmd3	0	Slu7	0	Rerg	0	Slx	0
Psmd2	0	Sln	0	Vstm2l	0	Slurp1	0
Psmd13	0	Slmo2	0	Gm9047	0	Slmo2	0
Psmd12	0	Slk	0	Vstm2a	0	Slmo1	0
Psmd11	0	Slitrk6	0	Vstm2b	0	Slmap	0
Psmd10	0	Slitrk5	0	Tpsb2	0	Slk	0
Psmd1	0	Slitrk4	0	Hsd17b3	0	Slitrk6	0
Psmc6	0	Slitrk3	0	Acaa2	0	Slitrk4	0
Psmc5	0	Slitrk2	0	Tacc3	0	Slitrk2	0
Psmc4	0	Slitrk1	0	Trps1	0	Slitrk1	0
Psmc3ip	0	Slit2	0	Rnf144b	0	Slit3	0
Psmc3	0	Slit1	0	Gm5938	0	Slit2	0
Psmc2	0	Slirp	0	Hrk	0	Slit1	0
Psmc1	0	Slfn9	0	Rnf144a	0	Slirp	0
Psmb8	0	Slfn8	0	Gm5935	0	Slfnl1	0
Psmb7	0	Slfn5	0	Tuba4a	0	Slfn9	0
Psmb6	0	Slfn4	0	Nfkb2	0	Slfn8	0
Psmb4	0	Slfn3	0	Gm5936	0	Slfn5	0
Psmb3	0	Slfn14	0	BRDN0000738217	0	Slfn2	0
Psmb2	0	Slfn1	0	BRDN0000737460	0	Slfn14	0
Psmb11	0	Slco6c1	0	Hrc	0	Slco6d1	0
Psmb10	0	Slco5a1	0	BRDN0000737462	0	Slco5a1	0
Psmb1	0	Slco4c1	0	BRDN0000737979	0	Slco4c1	0
Psma8	0	Slco4a1	0	Ctsq	0	Slco4a1	0
Psma7	0	Slco3a1	0	Sun2	0	Slco3a1	0
Psma6	0	Slco2b1	0	Sun3	0	Slco1c1	0
Psma5	0	Slco2a1	0	Rgl3	0	Slco1b2	0
Psma4	0	Slco1c1	0	Rgl2	0	Slco1a4	0
Psma3	0	Slco1b2	0	Kdm5a	0	Slc9c1	0
Psma2	0	Slco1a4	0	Zbtb8a	0	Slc9b1	0
Psma1	0	Slco1a1	0	AY761185	0	Slc9a7	0
Pskh1	0	Slc9c1	0	AY761184	0	Slc9a5	0
Psip1	0	Slc9b1	0	Taar7b	0	Slc9a4	0
Psg29	0	Slc9a9	0	Taar7a	0	Slc9a3r2	0
Psg28	0	Slc9a7	0	Inadl	0	Slc9a3r1	0
Psg27	0	Slc9a6	0	Mtx3	0	Slc9a1	0
Psg26	0	Slc9a3r1	0	Mtx2	0	Slc8b1	0
Psg23	0	Slc9a2	0	Taar7e	0	Slc8a2	0
Psg22	0	Slc8a3	0	Taar7d	0	Slc8a1	0
Psg21	0	Slc8a2	0	Atf5	0	Slc7a9	0
Psg19	0	Slc8a1	0	Smarca1	0	Slc7a8	0
Psg18	0	Slc7a9	0	Smarca2	0	Slc7a6os	0
Psg17	0	Slc7a8	0	Atf6	0	Slc7a6	0
Psg16	0	Slc7a7	0	Atf1	0	Slc7a5	0
Psen2	0	Slc7a6os	0	Smarca5	0	Slc7a3	0
Psd4	0	Slc7a6	0	Atf2	0	Slc7a2	0
Psd3	0	Slc7a5	0	BRDN0000738203	0	Slc7a15	0
Psd2	0	Slc7a4	0	Tox3	0	Slc7a14	0
Psd	0	Slc7a2	0	Rhbdd1	0	Slc7a13	0
Psat1	0	Slc7a14	0	Olfr338	0	Slc7a11	0
Psap	0	Slc7a11	0	Pde6g	0	Slc7a10	0
Prx	0	Slc7a10	0	BRDN0000738253	0	Slc7a1	0
Prune2	0	Slc6a7	0	Ptprt	0	Slc6a7	0
Prune	0	Slc6a6	0	Rtp1	0	Slc6a6	0
Prtn3	0	Slc6a4	0	Cmtm3	0	Slc6a5	0
Prtg	0	Slc6a3	0	Il15ra	0	Slc6a4	0
Prss58	0	Slc6a20b	0	Cmtm7	0	Slc6a3	0
Prss57	0	Slc6a20a	0	Cmtm4	0	Slc6a20a	0
Prss55	0	Slc6a2	0	Olfr1189	0	Slc6a19	0
Prss54	0	Slc6a19	0	Olfr1188	0	Slc6a18	0
Prss53	0	Slc6a18	0	Tnnt3	0	Slc6a17	0
Prss52	0	Slc6a17	0	Lias	0	Slc6a15	0
Prss51	0	Slc6a15	0	1600014C23Rik	0	Slc6a14	0
Prss50	0	Slc6a13	0	Olfr1183	0	Slc6a13	0
Prss48	0	Slc6a12	0	Olfr1182	0	Slc6a12	0
Prss46	0	Slc6a11	0	Gfm2	0	Slc6a11	0
Prss45	0	Slc5a9	0	Olfr1180	0	Slc6a1	0
Prss44	0	Slc5a7	0	Olfr1186	0	Slc5a9	0
Prss43	0	Slc5a6	0	Olfr1184	0	Slc5a7	0
Prss42	0	Slc5a4b	0	Grik2	0	Slc5a4a	0
Prss39	0	Slc5a4a	0	1700001C19Rik	0	Slc5a3	0
Prss38	0	Slc5a3	0	Cpsf7	0	Slc5a2	0
Prss37	0	Slc5a2	0	Stt3b	0	Slc5a12	0
Prss36	0	Slc5a12	0	Stt3a	0	Slc5a11	0
Prss34	0	Slc5a11	0	Mul1	0	Slc5a10	0
Prss33	0	Slc5a10	0	Cpsf3	0	Slc5a1	0
Prss32	0	Slc51b	0	Cpsf2	0	Slc52a2	0
Prss30	0	Slc51a	0	Cpsf1	0	Slc51a	0
Prss3	0	Slc50a1	0	Sbds	0	Slc50a1	0
Prss29	0	Slc4a9	0	Ndrg4	0	Slc4a8	0
Prss28	0	Slc4a8	0	Trappc2l	0	Slc4a7	0

Prss27	0	Slc4a7	0	BRDN0000738301	0	Slc4a5	0
Prss23	0	Slc4a4	0	Aspg	0	Slc4a2	0
Prss22	0	Slc4a2	0	Aspa	0	Slc4a10	0
Prss2	0	Slc4a1	0	Eng	0	Slc4a1	0
Prss16	0	Slc48a1	0	Aspm	0	Slc48a1	0
Prrx2	0	Slc47a2	0	Aspn	0	Slc47a2	0
Prrx1	0	Slc47a1	0	Asph	0	Slc46a3	0
Prrt4	0	Slc45a3	0	Chml	0	Slc46a2	0
Prrt2	0	Slc45a2	0	B4galt4	0	Slc46a1	0
Prrt1	0	Slc44a4	0	Rrn3	0	Slc45a3	0
Prrg3	0	Slc44a2	0	Tpi1	0	Slc45a1	0
Prrc2c	0	Slc44a1	0	Clu	0	Slc44a5	0
Prrc2b	0	Slc43a3	0	Akap1	0	Slc44a4	0
Prrc2a	0	Slc43a2	0	Olfr1231	0	Slc44a2	0
Prrc1	0	Slc41a2	0	Olfr1230	0	Slc44a1	0
Prr7	0	Slc41a1	0	Olfr1233	0	Slc43a3	0
Prr5	0	Slc3a2	0	Olfr1232	0	Slc43a2	0
Prr36	0	Slc3a1	0	Becn1	0	Slc43a1	0
Prr3	0	Slc39a8	0	4930402F06Rik	0	Slc41a1	0
Prr27	0	Slc39a7	0	Siah2	0	Slc40a1	0
Prr23a	0	Slc39a6	0	Pate2	0	Slc3a2	0
Prr22	0	Slc39a5	0	Olfr1239	0	Slc3a1	0
Prr18	0	Slc39a4	0	Olfr1238	0	Slc39a9	0
Prr16	0	Slc39a3	0	Adamts13	0	Slc39a7	0
Prr15l	0	Slc39a2	0	Akap3	0	Slc39a3	0
Prr15	0	Slc39a13	0	Fam135a	0	Slc39a14	0
Prr14	0	Slc39a12	0	Tcf15	0	Slc39a11	0
Prr12	0	Slc39a11	0	Fam135b	0	Slc39a10	0
Prr11	0	Slc39a10	0	Tcf12	0	Slc39a1	0
Prpsap2	0	Slc38a9	0	Hdc	0	Slc38a9	0
Prps2	0	Slc38a6	0	Fndc3a	0	Slc38a8	0
Prps1l3	0	Slc38a3	0	Fndc3b	0	Slc38a7	0
Prps1l1	0	Slc38a2	0	Syne3	0	Slc38a6	0
Prph2	0	Slc37a3	0	Tcf19	0	Slc38a5	0
Prpf8	0	Slc37a2	0	Ybx3	0	Slc38a3	0
Prpf4b	0	Slc37a1	0	Ybx2	0	Slc38a11	0
Prpf40b	0	Slc36a4	0	Gm11562	0	Slc38a10	0
Prpf40a	0	Slc36a2	0	Phpt1	0	Slc38a1	0
Prpf4	0	Slc36a1	0	Ovgp1	0	Slc37a4	0
Prpf38b	0	Slc35g2	0	Gm11567	0	Slc37a3	0
Prpf38a	0	Slc35g1	0	Gm11564	0	Slc36a4	0
Prpf31	0	Slc35f5	0	Nono	0	Slc36a2	0
Prpf3	0	Slc35f4	0	Gm11568	0	Slc36a1	0
Prpf19	0	Slc35f3	0	Gm11569	0	Slc35g1	0
Prpf18	0	Slc35f1	0	Rpusd2	0	Slc35f6	0
Proz	0	Slc35e4	0	Dazap2	0	Slc35f5	0
Prox2	0	Slc35e3	0	Trmt6	0	Slc35f4	0
Prox1	0	Slc35d1	0	Rasa4	0	Slc35f1	0
Proser3	0	Slc35c1	0	Gatsl2	0	Slc35e3	0
Pros1	0	Slc35b4	0	Eif2s3y	0	Slc35e1	0
Prorsd1	0	Slc35b3	0	Eif2s3x	0	Slc35d3	0
Prop1	0	Slc35b2	0	Lrp6	0	Slc35c2	0
Prom2	0	Slc35b1	0	BRDN0000738204	0	Slc35b1	0
Prol1	0	Slc35a4	0	BRDN0000738302	0	Slc35a5	0
Prokr2	0	Slc35a3	0	BRDN0000738285	0	Slc35a4	0
Prokr1	0	Slc33a1	0	Sox18	0	Slc35a3	0
Prok2	0	Slc32a1	0	Hspb7	0	Slc35a1	0
Prodh2	0	Slc31a2	0	Rgs14	0	Slc34a3	0
Prodh	0	Slc30a8	0	Fxyd5	0	Slc34a1	0
Proc	0	Slc30a7	0	Rgs12	0	Slc33a1	0
Prob1	0	Slc30a6	0	Rgs13	0	Slc32a1	0
Prn	0	Slc30a3	0	Sirt5	0	Slc31a2	0
Prmt7	0	Slc30a2	0	Sox11	0	Slc31a1	0
Prmt6	0	Slc30a1	0	Sox12	0	Slc30a9	0
Prmt5	0	Slc2a7	0	Sox14	0	Slc30a6	0
Prmt3	0	Slc2a3	0	Sox15	0	Slc30a5	0
Prmt2	0	Slc2a2	0	Rgs18	0	Slc30a4	0
Prmt10	0	Slc2a13	0	Rasa1	0	Slc30a3	0
Prlr	0	Slc2a12	0	Zbtb10	0	Slc30a2	0
Prl8a9	0	Slc2a10	0	Rps3	0	Slc30a10	0
Prl8a8	0	Slc2a1	0	Rnf151	0	Slc30a1	0
Prl8a2	0	Slc29a4	0	Ptprh	0	Slc2a6	0
Prl8a1	0	Slc29a2	0	Ebna1bp2	0	Slc2a5	0
Prl7d1	0	Slc28a3	0	Anxa5	0	Slc2a4	0
Prl7c1	0	Slc28a1	0	Taf1b	0	Slc2a3	0
Prl7b1	0	Slc27a6	0	Stil	0	Slc2a2	0
Prl7a1	0	Slc27a5	0	Dbt	0	Slc2a10	0
Prl6a1	0	Slc27a4	0	Upk3b	0	Slc29a4	0
Prl4a1	0	Slc27a2	0	Upk3a	0	Slc29a3	0
Prl3d3	0	Slc26a9	0	2310014L17Rik	0	Slc29a2	0
Prl3d2	0	Slc26a8	0	Myzap	0	Slc29a1	0
Prl3d1	0	Slc26a7	0	Pdpn	0	Slc28a3	0

Prl3c1	0	Slc26a4	0	Hn1l	0	Slc28a2	0
Prl3b1	0	Slc26a2	0	Pdpr	0	Slc27a6	0
Prl3a1	0	Slc26a11	0	Gm15284	0	Slc27a4	0
Prl2c5	0	Slc26a10	0	Mapt	0	Slc27a3	0
Prl2c3	0	Slc26a1	0	Gm14850	0	Slc27a2	0
Prl2c2	0	Slc25a54	0	Olfr20	0	Slc27a1	0
Prl2b1	0	Slc25a53	0	Olfr23	0	Slc26a8	0
Prl	0	Slc25a51	0	Olfr25	0	Slc26a7	0
Prkrir	0	Slc25a5	0	Olfr24	0	Slc26a5	0
Prkrip1	0	Slc25a48	0	Olfr27	0	Slc26a4	0
Prkra	0	Slc25a45	0	Olfr26	0	Slc26a11	0
Prkg2	0	Slc25a42	0	Lrp2	0	Slc26a10	0
Prkd3	0	Slc25a41	0	Htr5a	0	Slc26a1	0
Prkd2	0	Slc25a40	0	Pde6a	0	Slc25a54	0
Prkd1	0	Slc25a37	0	Alg13	0	Slc25a48	0
Prkcq	0	Slc25a36	0	Rfwd2	0	Slc25a47	0
Prkch	0	Slc25a35	0	Rfwd3	0	Slc25a44	0
Prkcg	0	Slc25a33	0	Lrp4	0	Slc25a42	0
Prkce	0	Slc25a32	0	Lrp5	0	Slc25a41	0
Prkcdbp	0	Slc25a31	0	Lrp2bp	0	Slc25a40	0
Prkcd	0	Slc25a30	0	Lbhd1	0	Slc25a4	0
Prkcb	0	Slc25a29	0	4930402H24Rik	0	Slc25a37	0
Prkca	0	Slc25a28	0	Tfpt	0	Slc25a36	0
Prkar2b	0	Slc25a26	0	1700019N19Rik	0	Slc25a34	0
Prkar2a	0	Slc25a25	0	Mvk	0	Slc25a33	0
Prkar1a	0	Slc25a24	0	Prl5a1	0	Slc25a32	0
Prkag3	0	Slc25a23	0	BRDN0000738288	0	Slc25a31	0
Prkag2	0	Slc25a22	0	Rps8	0	Slc25a28	0
Prkag1	0	Slc25a21	0	Krtap3-1	0	Slc25a26	0
Prkacb	0	Slc25a2	0	Smarcd2	0	Slc25a25	0
Prkaca	0	Slc25a17	0	Acp6	0	Slc25a24	0
Prkab2	0	Slc25a15	0	Add3	0	Slc25a23	0
Prkab1	0	Slc25a14	0	Add2	0	Slc25a22	0
Prkaa2	0	Slc25a10	0	Acp2	0	Slc25a21	0
Prkaa1	0	Slc25a1	0	Wwtr1	0	Slc25a20	0
Prima1	0	Slc24a5	0	Arid3c	0	Slc25a19	0
Prim2	0	Slc24a4	0	Map2	0	Slc25a18	0
Prim1	0	Slc23a3	0	Taf12	0	Slc25a16	0
Prickle2	0	Slc23a2	0	Itm2a	0	Slc25a14	0
Prickle1	0	Slc22a7	0	Taf10	0	Slc25a12	0
Prh1	0	Slc22a6	0	Map6	0	Slc25a10	0
Prg4	0	Slc22a5	0	Taf15	0	Slc25a1	0
Prg2	0	Slc22a30	0	Gpcpd1	0	Slc24a5	0
Prf1	0	Slc22a3	0	Sema4a	0	Slc24a3	0
Prex2	0	Slc22a28	0	Sema4b	0	Slc24a1	0
Prex1	0	Slc22a26	0	Map9	0	Slc23a2	0
Prepl	0	Slc22a23	0	Cops7b	0	Slc23a1	0
Prep	0	Slc22a22	0	Cops7a	0	Slc22a8	0
Prelp	0	Slc22a21	0	Klhl25	0	Slc22a6	0
Prelid2	0	Slc22a19	0	Klhl24	0	Slc22a4	0
Prelid1	0	Slc22a18	0	Klhl26	0	Slc22a3	0
Preb	0	Slc22a16	0	Klhl21	0	Slc22a29	0
Prdx4	0	Slc22a15	0	Klhl20	0	Slc22a26	0
Prdx3	0	Slc22a13	0	Klhl23	0	Slc22a23	0
Prdx2	0	Slc22a12	0	Raph1	0	Slc22a22	0
Prdx1	0	Slc22a1	0	Tgds	0	Slc22a21	0
Prdm9	0	Slc20a2	0	Pgpep1l	0	Slc22a18	0
Prdm8	0	Slc20a1	0	Il21r	0	Slc22a17	0
Prdm6	0	Slc1a7	0	Klhl29	0	Slc22a15	0
Prdm5	0	Slc1a6	0	2810408A11Rik	0	Slc22a14	0
Prdm4	0	Slc1a4	0	BRDN0000738205	0	Slc22a13	0
Prdm2	0	Slc1a3	0	BRDN0000737946	0	Slc22a1	0
Prdm16	0	Slc1a2	0	1700042G07Rik	0	Slc20a2	0
Prdm15	0	Slc1a1	0	Gm5346	0	Slc1a7	0
Prdm14	0	Slc19a3	0	Mesp1	0	Slc1a6	0
Prdm13	0	Slc19a2	0	Mesp2	0	Slc1a5	0
Prdm12	0	Slc19a1	0	Pde6d	0	Slc1a4	0
Prdm10	0	Slc18a2	0	Ift140	0	Slc1a3	0
Prdm1	0	Slc18a1	0	Fmo2	0	Slc19a3	0
Prcp	0	Slc17a9	0	Trip4	0	Slc19a2	0
Prcc	0	Slc17a8	0	Fmo1	0	Slc18a3	0
Prb1	0	Slc17a6	0	Fmo6	0	Slc18a2	0
Prap1	0	Slc17a4	0	Tbc1d25	0	Slc18a1	0
Pramel5	0	Slc17a3	0	Fmo4	0	Slc17a8	0
Pramel3	0	Slc17a2	0	Fmo5	0	Slc17a7	0
Pramel1	0	Slc16a9	0	Ackr4	0	Slc17a6	0
Pramef8	0	Slc16a8	0	Gsg2	0	Slc17a5	0
Pramef17	0	Slc16a7	0	Gsg1	0	Slc17a4	0
Prame	0	Slc16a6	0	Fmo9	0	Slc17a2	0
Pram1	0	Slc16a3	0	Spred1	0	Slc16a7	0
Praf2	0	Slc16a14	0	Ackr2	0	Slc16a6	0
Pradc1	0	Slc16a13	0	Ackr3	0	Slc16a3	0

Pqlc3	0
Pqlc2	0
Pqlc1	0
Pqbp1	0
Ppy	0
Ppwd1	0
Pptc7	0
Ppt2	0
Ppt1	0
Pprc1	0
Ppp6r3	0
Ppp6c	0
Ppp4r4	0
Ppp4r2	0
Ppp4r1	0
Ppp4c	0
Ppp3r1	0
Ppp3cb	0
Ppp3ca	0
Ppp2r5e	0
Ppp2r5d	0
Ppp2r5c	0
Ppp2r5b	0
Ppp2r4	0
Ppp2r3a	0
Ppp2r2d	0
Ppp2r2b	0
Ppp2r2a	0
Ppp2r1b	0
Ppp2r1a	0
Ppp2cb	0
Ppp2ca	0
Ppp1r9b	0
Ppp1r8	0
Ppp1r7	0
Ppp1r3g	0
Ppp1r3e	0
Ppp1r3d	0
Ppp1r3a	0
Ppp1r37	0
Ppp1r36	0
Ppp1r35	0
Ppp1r32	0
Ppp1r27	0
Ppp1r21	0
Ppp1r2	0
Ppp1r1c	0
Ppp1r1b	0
Ppp1r1a	0
Ppp1r18	0
Ppp1r17	0
Ppp1r16b	0
Ppp1r16a	0
Ppp1r15b	0
Ppp1r15a	0
Ppp1r14d	0
Ppp1r14c	0
Ppp1r14b	0
Ppp1r13l	0
Ppp1r13b	0
Ppp1r12c	0
Ppp1r12b	0
Ppp1r12a	0
Ppp1r11	0
Ppp1r10	0
Ppp1cc	0
Ppp1cb	0
Ppp1ca	0
Ppox	0
Ppme1	0
Ppm1n	0
Ppm1m	0
Ppm1k	0
Ppm1j	0
Ppm1h	0
Ppm1e	0
Ppm1d	0
Ppm1b	0
Ppm1a	0
Ppl	0
Ppip5k1	0
Ppil6	0
Slc16a12	0
Slc16a11	0
Slc16a10	0
Slc16a1	0
Slc15a5	0
Slc15a3	0
Slc15a2	0
Slc15a1	0
Slc14a1	0
Slc13a5	0
Slc13a4	0
Slc13a1	0
Slc12a9	0
Slc12a7	0
Slc12a3	0
Slc12a1	0
Slc11a2	0
Slc10a7	0
Slc10a5	0
Slc10a2	0
Slamf9	0
Slamf8	0
Slamf7	0
Slamf1	0
Slain2	0
Slain1	0
Sla	0
Skp2	0
Skp1a	0
Skor2	0
Skor1	0
Skiv2l2	0
Skiv2l	0
Skint8	0
Skint6	0
Skint4	0
Skint2	0
Skint11	0
Skint1	0
Ski	0
Skap2	0
Ska3	0
Ska2	0
Six5	0
Six3	0
Six2	0
Six1	0
Siva1	0
Sis	0
Sirt6	0
Sirt4	0
Sirt3	0
Sirt2	0
Sirpb1b	0
Sirpb1a	0
Sirpa	0
Sipa1l3	0
Sipa1	0
Sin3b	0
Sin3a	0
Sim2	0
Sim1	0
Sil1	0
Sike1	0
Sik3	0
Sik2	0
Sigmar1	0
Siglecg	0
Siglecf	0
Siglece	0
Siglec15	0
Siglec1	0
Sigirr	0
Sidt2	0
Sidt1	0
Siah2	0
Siah1b	0
Siah1a	0
Siae	0
Shroom4	0
Shpk	0
Shox2	0
Rag2	0
Rag1	0
Olfr1040	0
Anxa4	0
Sertad4	0
Clhc1	0
Strap	0
Pin1rt1	0
Serpind1	0
Acp1	0
Adamtsl2	0
Xaf1	0
Adamtsl1	0
Fam168b	0
Add1	0
Adamtsl4	0
Zfp942	0
Frzb	0
Rplp2	0
Rplp1	0
Rplp0	0
Cadps	0
Mllt10	0
Mtg1	0
BRDN0000737928	0
Mtus1	0
Gm5168	0
Gm5169	0
Acsf3	0
Fads1	0
Gm14459	0
Fads3	0
Fads2	0
BRDN0000737924	0
BRDN0000737925	0
BRDN0000737926	0
Fads6	0
Slc35g2	0
BRDN0000737672	0
Slc35g1	0
BRDN0000737674	0
Mef2b	0
Vmn1r34	0
Lat2	0
BRDN0000737678	0
Ccnb3	0
6430550D23Rik	0
Ptgis	0
Ptgir	0
Zfp493	0
C2cd4c	0
Thap7	0
Cish	0
Eps8	0
5430419D17Rik	0
BRDN0000738263	0
Fam117a	0
Gltscr1l	0
Cd79b	0
Cd79a	0
5031414D18Rik	0
Taf13	0
Nhp2l1	0
Ecel1	0
Pik3r6	0
Vac14	0
Mecp2	0
4932438H23Rik	0
Pdp2	0
Adgra1	0
Echdc2	0
Adgra3	0
Chrna7	0
Pde6h	0
Fars2	0
BRDN0000738260	0
Fsbp	0
Chsy1	0
Leng1	0
Zw10	0
Stau1	0
Awat1	0
Slc16a2	0
Slc16a14	0
Slc16a13	0
Slc16a12	0
Slc16a11	0
Slc16a10	0
Slc16a1	0
Slc15a4	0
Slc15a3	0
Slc15a2	0
Slc15a1	0
Slc14a1	0
Slc13a1	0
Slc12a9	0
Slc12a7	0
Slc12a5	0
Slc12a4	0
Slc12a3	0
Slc12a2	0
Slc12a1	0
Slc11a2	0
Slc10a7	0
Slc10a6	0
Slc10a5	0
Slc10a4	0
Slc10a3	0
Slc10a2	0
Slbp	0
Slamf7	0
Slamf6	0
Slain2	0
Sla2	0
Skp2	0
Skp1a	0
Skiv2l2	0
Skiv2l	0
Skint8	0
Skint7	0
Skint6	0
Skint5	0
Skint4	0
Skint3	0
Skint2	0
Skint11	0
Skint1	0
Skil	0
Skap2	0
Skap1	0
Ska3	0
Ska1	0
Six6	0
Six5	0
Six4	0
Six3	0
Six2	0
Six1	0
Siva1	0
Sit1	0
Sirt7	0
Sirt6	0
Sirt5	0
Sirt3	0
Sirt2	0
Sirt1	0
Sirpb1b	0
Sirpb1a	0
Sirpa	0
Sipa1l2	0
Sipa1	0
Sin3b	0
Sin3a	0
Sim1	0
Sil1	0
Sike1	0
Sik3	0
Sik2	0
Sigmar1	0
Siglech	0
Siglecg	0
Siglecf	0
Siglece	0
Siglec15	0

Ppil4	0	Shmt2	0	Awat2	0	Siglec1	0
Ppil3	0	Shmt1	0	Pik3r2	0	Sigirr	0
Ppil2	0	Shkbp1	0	Rnase2a	0	Siah1a	0
Ppil1	0	Shisa9	0	Ticrr	0	Siae	0
Ppih	0	Shisa8	0	Ccnb1	0	Shroom1	0
Ppif	0	Shisa7	0	Gm10081	0	Shprh	0
Ppie	0	Shisa6	0	Rrs1	0	Shpk	0
Ppic	0	Shisa5	0	Gm20747	0	Shoc2	0
Ppib	0	Shisa3	0	Ceacam16	0	Shkbp1	0
Ppia	0	Shh	0	Zfp91	0	Shisa9	0
Pphln1	0	Shfm1	0	Ccdc103	0	Shisa8	0
Ppfibp2	0	Shd	0	Echdc1	0	Shisa7	0
Ppfibp1	0	Shcbp1l	0	Gm10778	0	Shisa5	0
Ppfia4	0	Shcbp1	0	Oxr1	0	Shisa3	0
Ppfia3	0	Shc4	0	Ier5l	0	Shh	0
Ppfia1	0	Shc2	0	Zbtb34	0	Shfm1	0
Ppef2	0	Shc1	0	Iqgap1	0	She	0
Ppef1	0	Shbg	0	A530064D06Rik	0	Shd	0
Ppdpf	0	Shank3	0	Iqgap3	0	Shcbp1l	0
Ppcs	0	Shank1	0	Gm6588	0	Shc4	0
Ppcdc	0	Sh3yl1	0	Lct	0	Shc2	0
Ppbp	0	Sh3tc2	0	Srp54a	0	Shc1	0
Ppat	0	Sh3tc1	0	Zfp128	0	Sharpin	0
Ppargc1b	0	Sh3rf3	0	Pdxdc1	0	Shank1	0
Ppargc1a	0	Sh3rf2	0	2010300C02Rik	0	Sh3tc2	0
Pparg	0	Sh3rf1	0	Tceb2	0	Sh3tc1	0
Ppara	0	Sh3pxd2a	0	Tceb1	0	Sh3rf2	0
Ppapdc3	0	Sh3glb2	0	Zfp120	0	Sh3kbp1	0
Ppapdc2	0	Sh3gl2	0	Unc5cl	0	Sh3glb2	0
Ppapdc1b	0	Sh3d21	0	Chrna9	0	Sh3glb1	0
Ppapdc1a	0	Sh3d19	0	BRDN0000738266	0	Sh3gl1	0
Ppap2a	0	Sh3bp2	0	Whrn	0	Sh3d21	0
Ppa2	0	Sh3bgrl2	0	Matr3	0	Sh3bp5l	0
Ppa1	0	Sh3bgrl	0	BRDN0000738148	0	Sh3bp5	0
Pp2d1	0	Sh3bgr	0	Tcp10b	0	Sh3bp4	0
Pou5f2	0	Sh2d5	0	BRDN0000738146	0	Sh3bp2	0
Pou5f1	0	Sh2d4b	0	BRDN0000738145	0	Sh3bgrl2	0
Pou4f3	0	Sh2d3c	0	Sema4g	0	Sh3bgrl	0
Pou4f2	0	Sh2d1b2	0	BRDN0000738143	0	Sh2d7	0
Pou4f1	0	Sh2b3	0	BRDN0000738141	0	Sh2d5	0
Pou3f4	0	Sh2b2	0	BRDN0000738140	0	Sh2d4b	0
Pou3f3	0	Sh2b1	0	Timd2	0	Sh2d4a	0
Pou3f2	0	Sgtb	0	Sfrp5	0	Sh2d3c	0
Pou3f1	0	Sgsm3	0	Timd4	0	Sh2d2a	0
Pou2af1	0	Sgsm1	0	Bpi	0	Sh2d1b2	0
Pou1f1	0	Sgpp2	0	Idi1	0	Sh2d1b1	0
Pot1a	0	Sgpp1	0	Idi2	0	Sh2b3	0
Postn	0	Sgol1	0	Olfr1537	0	Sh2b1	0
Porcn	0	Sgms1	0	Ucma	0	Sgta	0
Por	0	Sgk3	0	Olfr1535	0	Sgsm2	0
Popdc3	0	Sgk1	0	BRDN0000738267	0	Sgpl1	0
Pop7	0	Sgip1	0	Sox9	0	Sgol1	0
Pop5	0	Sgcz	0	1190007I07Rik	0	Sgms2	0
Pop4	0	Sgce	0	Trnp1	0	Sgms1	0
Pop1	0	Sgcd	0	Sox3	0	Sgk2	0
Pon3	0	Sgcb	0	Cables2	0	Sgip1	0
Pon2	0	Sgca	0	Myocd	0	Sgcz	0
Pomt2	0	Sfxn5	0	Sox7	0	Sgcg	0
Pomt1	0	Sfxn4	0	Sox4	0	Sgcd	0
Pomp	0	Sfxn3	0	Cd2bp2	0	Sgcb	0
Pomk	0	Sfxn2	0	Klhl22	0	Sgca	0
Pomgnt2	0	Sfxn1	0	Bcl11a	0	Sfxn4	0
Pomc	0	Sfta2	0	Dand5	0	Sfxn3	0
Pom121l2	0	Sft2d2	0	Cps1	0	Sfxn1	0
Pom121	0	Sfswap	0	Mettl23	0	Sftpd	0
Polrmt	0	Sfrp4	0	Mettl22	0	Sfta2	0
Polr3k	0	Sfpq	0	Olfr103	0	Sft2d1	0
Polr3h	0	Sfn	0	Mettl20	0	Sfswap	0
Polr3gl	0	Sfmbt2	0	Ntsr2	0	Sfrp4	0
Polr3g	0	Sfi1	0	Olfr107	0	Sfrp1	0
Polr3f	0	Sf3b6	0	Mettl24	0	Sfr1	0
Polr3e	0	Sf3b5	0	BRDN0000737582	0	Sfi1	0
Polr3d	0	Sf3b4	0	Olfr108	0	Sf3b6	0
Polr3c	0	Sf3b3	0	BRDN0000737580	0	Sf3b5	0
Polr3b	0	Sf3b2	0	BRDN0000737581	0	Sf3b4	0
Polr3a	0	Sf3b1	0	BRDN0000738327	0	Sf3b3	0
Polr2m	0	Sf3a3	0	BRDN0000738326	0	Sf3b2	0
Polr2k	0	Sf3a2	0	BRDN0000738325	0	Sf3b1	0
Polr2j	0	Sf3a1	0	1110059E24Rik	0	Sf3a3	0
Polr2i	0	Sf1	0	Csdc2	0	Sf3a2	0
Polr2h	0	Sez6l2	0	Hinfp	0	Sf3a1	0
Polr2g	0	Sez6l	0	Olfr1128	0	Sf1	0

Polr2f	0	Sez6	0	Alg12	0	Sez6l2	0
Polr2e	0	Setmar	0	Phrf1	0	Sez6l	0
Polr2d	0	Setdb2	0	Gm4981	0	Sez6	0
Polr2c	0	Setdb1	0	Gm4980	0	Setx	0
Polr2b	0	Setd8	0	BRDN0000738265	0	Setmar	0
Polr2a	0	Setd7	0	Calm4	0	Setdb2	0
Polr1e	0	Setd6	0	Klhl28	0	Setdb1	0
Polr1d	0	Setd4	0	Mus81	0	Setd8	0
Polr1c	0	Setd2	0	Padi3	0	Setd3	0
Polr1b	0	Setd1a	0	Anks1b	0	Setd2	0
Polr1a	0	Setbp1	0	Krtap4-13	0	Set	0
Polq	0	Set	0	4930503L19Rik	0	Sestd1	0
Poln	0	Sesn3	0	Iigp1	0	Sesn3	0
Polm	0	Sesn2	0	Krtap4-16	0	Sesn2	0
Poll	0	Sesn1	0	Parg	0	Sertm1	0
Polh	0	Sertad4	0	Krtap26-1	0	Sertad4	0
Polg2	0	Sertad3	0	Rsu1	0	Sertad3	0
Polg	0	Sertad1	0	Prss22	0	Sertad2	0
Pole4	0	Serpini2	0	Lyve1	0	Sertad1	0
Pole3	0	Serpini1	0	Capn8	0	Serpinh1	0
Pole2	0	Serpinh1	0	Parn	0	Serpinf2	0
Pole	0	Serping1	0	Parl	0	Serpinf1	0
Poldip3	0	Serpinf2	0	Il21	0	Serpine3	0
Pold4	0	Serpine2	0	Il20	0	Serpine2	0
Pold3	0	Serpind1	0	Hspa12a	0	Serpine1	0
Pold2	0	Serpinc1	0	Il25	0	Serpind1	0
Pold1	0	Serpinb9g	0	Il24	0	Serpinb9g	0
Polb	0	Serpinb9f	0	Il27	0	Serpinb9f	0
Pola2	0	Serpinb9	0	Olfr1120	0	Serpinb9e	0
Pola1	0	Serpinb8	0	Srp54b	0	Serpinb9d	0
Pogk	0	Serpinb6e	0	Olfr1123	0	Serpinb9c	0
Pofut2	0	Serpinb6d	0	Prepl	0	Serpinb9b	0
Pofut1	0	Serpinb6c	0	Pnma3	0	Serpinb9	0
Pof1b	0	Serpinb6b	0	Pgs1	0	Serpinb8	0
Podxl	0	Serpinb6a	0	H2-Oa	0	Serpinb7	0
Podnl1	0	Serpinb3d	0	Cpxm1	0	Serpinb6e	0
Poc5	0	Serpinb3c	0	Ltb	0	Serpinb6d	0
Poc1a	0	Serpinb3a	0	Cpxm2	0	Serpinb6b	0
Pnrc2	0	Serpinb2	0	Kntc1	0	Serpinb6a	0
Pnpt1	0	Serpinb1c	0	Ophn1	0	Serpinb5	0
Pnpo	0	Serpinb13	0	Myl7	0	Serpinb3c	0
Pnpla7	0	Serpinb12	0	Myl4	0	Serpinb3b	0
Pnpla5	0	Serpina9	0	Plekhb2	0	Serpinb2	0
Pnpla2	0	Serpina6	0	Myl2	0	Serpinb1c	0
Pnpla1	0	Serpina3j	0	Myl3	0	Serpinb12	0
Pnp2	0	Serpina3i	0	Pdx1	0	Serpinb11	0
Pnp	0	Serpina3f	0	Myl1	0	Serpina6	0
Pno1	0	Serpina3c	0	Gal3st1	0	Serpina3k	0
Pnn	0	Serpina3a	0	Dnaaf1	0	Serpina3j	0
Pnmt	0	Serpina1e	0	Gal3st3	0	Serpina3i	0
Pnmal2	0	Serpina1d	0	Csgalnact2	0	Serpina3f	0
Pnmal1	0	Serpina1c	0	Dnaaf5	0	Serpina3c	0
Pnma5	0	Serpina11	0	Fxyd3	0	Serpina3b	0
Pnma3	0	Serpina10	0	Tomm40	0	Serpina3a	0
Pnma2	0	Serp2	0	Dexi	0	Serpina1f	0
Pnliprp2	0	Serp1	0	Arih1	0	Serpina1e	0
Pnliprp1	0	Serinc4	0	Pipox	0	Serpina1c	0
Pnlip	0	Serinc3	0	Lta	0	Serpina1b	0
Pnldc1	0	Serinc2	0	Fam53b	0	Serpina1a	0
Pnkp	0	Serinc1	0	Cd40	0	Serpina10	0
Pnkd	0	Serf2	0	Itm2c	0	Serp1	0
Pnck	0	Serac1	0	Fam53c	0	Serinc5	0
Pms2	0	Sepw1	0	Tlr2	0	Serinc4	0
Pmpcb	0	Sept9	0	Arl8b	0	Serinc2	0
Pmpca	0	Sept7	0	Tlr1	0	Serinc1	0
Pmp22	0	Sept6	0	Tlr6	0	Serhl	0
Pmp2	0	Sept5	0	St13	0	Serf2	0
Pmm2	0	Sept4	0	Tlr4	0	Serac1	0
Pmm1	0	Sept3	0	Ctnnbip1	0	Sepw1	0
Pml	0	Sept10	0	Klk4	0	Sept9	0
Pmfbp1	0	Sept1	0	Tlr8	0	Sept7	0
Pmf1	0	Sepsecs	0	Tlr9	0	Sept6	0
Pmel	0	Sepp1	0	Hars	0	Sept5	0
Pmch	0	Sepn1	0	Bicd2	0	Sept4	0
Pmaip1	0	Sephs1	0	St18	0	Sept3	0
Pm20d2	0	Sep15	0	Ltf	0	Sept2	0
Pm20d1	0	Senp8	0	Adamts5	0	Sept12	0
Plxnc1	0	Senp6	0	Adamts4	0	Sept10	0
Plxnb3	0	Senp5	0	Ube2l6	0	Sepsecs	0
Plxnb2	0	Senp3	0	Adamts6	0	Sepp1	0
Plxna4	0	Senp2	0	Adamts1	0	Sepn1	0
Plxna3	0	Senp1	0	Adamts3	0	Sephs2	0

Plxna2	0
Plxna1	0
Plxdc2	0
Plvap	0
Plscr5	0
Plscr4	0
Plscr3	0
Plscr1	0
Pls3	0
Pls1	0
Plrg1	0
Plp1	0
Plod2	0
Pln	0
Plk5	0
Plk3	0
Plk2	0
Plk1	0
Plin5	0
Plin4	0
Plin3	0
Plin2	0
Plin1	0
Plgrkt	0
Plg	0
Plet1	0
Plekhs1	0
Plekho2	0
Plekho1	0
Plekhm3	0
Plekhm2	0
Plekhm1	0
Plekhj1	0
Plekhh3	0
Plekhh2	0
Plekhh1	0
Plekhg5	0
Plekhg4	0
Plekhg3	0
Plekhg2	0
Plekhg1	0
Plekhf1	0
Plekhd1	0
Plekhb2	0
Plekhb1	0
Plekha8	0
Plekha5	0
Plekha4	0
Plekha3	0
Plekha2	0
Plek	0
Pld6	0
Pld5	0
Pld4	0
Pld2	0
Plcz1	0
Plcxd3	0
Plcxd2	0
Plcl2	0
Plcl1	0
Plch2	0
Plce1	0
Plcb4	0
Plcb3	0
Plcb2	0
Plcb1	0
Plbd2	0
Plbd1	0
Plb1	0
Plaur	0
Plat	0
Plagl2	0
Plagl1	0
Plag1	0
Plac8	0
Plac1	0
Plaa	0
Pla2r1	0
Pla2g7	0
Pla2g6	0
Pla2g5	0
Pla2g4f	0
Sema7a	0
Sema6d	0
Sema6a	0
Sema5b	0
Sema5a	0
Sema4g	0
Sema4f	0
Sema4d	0
Sema4b	0
Sema4a	0
Sema3g	0
Sema3f	0
Sema3d	0
Sema3b	0
Sema3a	0
Selm	0
Sell	0
Selk	0
Selenbp2	0
Sel1l3	0
Sel1l	0
Seh1l	0
Sectm1b	0
Sectm1a	0
Secisbp2l	0
Secisbp2	0
Sec63	0
Sec62	0
Sec61g	0
Sec61b	0
Sec61a1	0
Sec31b	0
Sec24d	0
Sec24c	0
Sec24b	0
Sec24a	0
Sec23ip	0
Sec23b	0
Sec23a	0
Sec16b	0
Sec14l2	0
Sec13	0
Sec11a	0
Sec1	0
Sebox	0
Sds	0
Sdr16c5	0
Sdpr	0
Sdk1	0
Sdhc	0
Sdhb	0
Sdhaf4	0
Sdhaf3	0
Sdha	0
Sdf4	0
Sdf2l1	0
Sdccag8	0
Sdccag3	0
Sdcbp2	0
Sdcbp	0
Sdc4	0
Sdc3	0
Sdad1	0
Scyl3	0
Scyl1	0
Scube1	0
Sctr	0
Sct	0
Scrt2	0
Scrt1	0
Scrn3	0
Scrn1	0
Scrg1	0
Scpep1	0
Scp2d1	0
Scoc	0
Sco1	0
Scnn1g	0
Scnm1	0
Scn4b	0
Scn4a	0
Scn3b	0
Prok2	0
Mfi2	0
Whsc1	0
Hnf4g	0
Klk7	0
Hnf4a	0
Adamts8	0
Hist1h2bf	0
Hist1h2ba	0
Hist1h2bb	0
Slc9b1	0
Hist1h2bm	0
Hist1h2bn	0
Slc9b2	0
Hist1h2bh	0
Perm1	0
Hist1h2bj	0
Hist1h2bk	0
Mmel1	0
Ar	0
Rab1b	0
Ldhal6b	0
Gucy1a3	0
A430078G23Rik	0
Dnase1	0
Gucy1a2	0
Rnf4	0
Noxa1	0
1700123K08Rik	0
Adad1	0
A2m	0
Adad2	0
BRDN0000737862	0
Mtr	0
Hmox1	0
Ndufaf2	0
Tk1	0
Ackr1	0
Stk32c	0
Pla2g2c	0
Pla2g2e	0
Spred2	0
Obp1a	0
Pla2g2f	0
Rfx8	0
Hmox2	0
Pdcl3	0
Lrrc75b	0
Naca	0
Cuta	0
Cutc	0
S1pr3	0
S1pr2	0
S1pr1	0
Zbtb4	0
S1pr5	0
S1pr4	0
Rab19	0
BRDN0000737985	0
Ptpn9	0
Defa-rs1	0
Rhot1	0
Rpf1	0
Got2	0
Rab10	0
Ptpn1	0
Rab12	0
Rfx6	0
Tbx20	0
Ptpn4	0
BRDN0000737590	0
Gpr17	0
Rfx7	0
Pink1	0
Cuedc1	0
Cuedc2	0
Slc22a30	0
Orc4	0
Orc5	0
Orc2	0
Orc3	0
Rassf6	0
Sephs1	0
Sep15	0
Senp8	0
Senp6	0
Senp5	0
Senp2	0
Senp1	0
Sema6d	0
Sema6c	0
Sema6b	0
Sema6a	0
Sema5a	0
Sema4f	0
Sema4b	0
Sema4a	0
Sema3g	0
Sema3e	0
Sema3d	0
Sema3c	0
Sema3b	0
Sema3a	0
Selt	0
Selplg	0
Selp	0
Selo	0
Selm	0
Sell	0
Selenbp2	0
Selenbp1	0
Sel1l3	0
Sel1l2	0
Sel1l	0
Seh1l	0
Sectm1b	0
Sectm1a	0
Secisbp2l	0
Sec63	0
Sec62	0
Sec61a2	0
Sec61a1	0
Sec31b	0
Sec24c	0
Sec24b	0
Sec24a	0
Sec23ip	0
Sec23b	0
Sec22c	0
Sec22b	0
Sec16b	0
Sec16a	0
Sec14l5	0
Sec14l4	0
Sec14l3	0
Sec14l1	0
Sec13	0
Sec11c	0
Sebox	0
Sds	0
Sdr9c7	0
Sdr42e1	0
Sdr39u1	0
Sdr16c6	0
Sdr16c5	0
Sdk2	0
Sdk1	0
Sdhd	0
Sdhc	0
Sdhb	0
Sdhaf4	0
Sdhaf3	0
Sdhaf1	0
Sdha	0
Sdf4	0
Sdf2l1	0
Sdf2	0
Sde2	0
Sdccag3	0
Sdcbp2	0
Sdcbp	0
Sdc3	0
Sdc1	0
Sdad1	0

Pla2g4e	0	Scn2b	0	Orc1	0	Scyl1	0
Pla2g4d	0	Scn2a1	0	Ankdd1b	0	Scx	0
Pla2g4c	0	Scn1b	0	Gm2696	0	Sctr	0
Pla2g4b	0	Scn1a	0	Mfsd6l	0	Sct	0
Pla2g3	0	Scn11a	0	Hecw1	0	Scrt2	0
Pla2g2e	0	Scn10a	0	Hecw2	0	Scrn1	0
Pla2g2d	0	Scml4	0	Nploc4	0	Scrib	0
Pla2g16	0	Scly	0	Rfx3	0	Scrg1	0
Pla2g15	0	Sclt1	0	Acta2	0	Scpep1	0
Pla2g12b	0	Scin	0	Irx1	0	Scp2d1	0
Pla2g10	0	Scimp	0	Acta1	0	Scp2	0
Pla1a	0	Schip1	0	Usp38	0	Sco2	0
Pkp4	0	Scgb2b7	0	Ash2l	0	Sco1	0
Pkp3	0	Scgb2b3	0	2510009E07Rik	0	Scnn1b	0
Pkp2	0	Scgb2b27	0	Penk	0	Scnn1a	0
Pkp1	0	Scgb2b26	0	1500009L16Rik	0	Scnm1	0
Pknox2	0	Scgb2b24	0	Gm15140	0	Scn7a	0
Pknox1	0	Scgb2b20	0	Clip3	0	Scn4a	0
Pkn3	0	Scgb2b17	0	Gm1673	0	Scn3b	0
Pkn1	0	Scgb2b15	0	2010107G23Rik	0	Scn2b	0
Pkm	0	Scgb2b12	0	Olfr516	0	Scn1a	0
Pklr	0	Scgb1c1	0	Creb5	0	Scn11a	0
Pkig	0	Scgb1b7	0	Mpv17l2	0	Scn10a	0
Pkib	0	Scgb1b29	0	Ndufb9	0	Scml4	0
Pkia	0	Scgb1b27	0	Ndufb8	0	Scml2	0
Pkhd1	0	Scgb1b19	0	Ndufb7	0	Scmh1	0
Pkdrej	0	Scgb1a1	0	Ndufb6	0	Scly	0
Pkdcc	0	Scg5	0	Ndufb5	0	Sclt1	0
Pkd2l2	0	Scg3	0	Ndufb4	0	Scimp	0
Pkd2l1	0	Scg2	0	Ndufb3	0	Scgb3a2	0
Pkd2	0	Scfd2	0	Ndufb2	0	Scgb3a1	0
Pkd1l3	0	Scfd1	0	Smarcal1	0	Scgb2b3	0
Pkd1l2	0	Scel	0	Dcun1d1	0	Scgb2b27	0
Pkd1	0	Scd4	0	Dcun1d2	0	Scgb2b20	0
Pja2	0	Scd3	0	Dcun1d4	0	Scgb2b2	0
Pja1	0	Scd2	0	Dcun1d5	0	Scgb2b19	0
Piwil2	0	Scd1	0	Tmem102	0	Scgb2b17	0
Piwil1	0	Sccpdh	0	Ddx3x	0	Scgb2b15	0
Pitx3	0	Scarf2	0	Frmd6	0	Scgb1c1	0
Pitrm1	0	Scarf1	0	Sil1	0	Scgb1b30	0
Pitpnm3	0	Scarb1	0	Olfr519	0	Scgb1b3	0
Pitpnm2	0	Scara5	0	Vtcn1	0	Scgb1b29	0
Pitpnm1	0	Scaper	0	Shpk	0	Scgb1b27	0
Pitpnc1	0	Scap	0	1700129C05Rik	0	Scgb1b24	0
Pitpna	0	Scand1	0	Tas2r103	0	Scgb1b2	0
Pisd	0	Scamp5	0	Fam168a	0	Scgb1a1	0
Pirt	0	Scamp4	0	BRDN0000737841	0	Scg5	0
Pirb	0	Scamp3	0	Vezf1	0	Scg3	0
Pira1	0	Scamp2	0	BRDN0000738027	0	Scg2	0
Pir	0	Scai	0	H2-M11	0	Scfd2	0
Pipox	0	Scaf8	0	Mrps16	0	Scfd1	0
Pip5k1c	0	Scaf4	0	Mrps15	0	Scel	0
Pip4k2c	0	Scaf11	0	Nxt1	0	Scd4	0
Pip4k2b	0	Scaf1	0	Kcnn3	0	Scd3	0
Pip	0	Sc5d	0	Mrps12	0	Scd2	0
Pinx1	0	Sbsn	0	Mrps11	0	Scd1	0
Pin4	0	Sbpl	0	Mrps10	0	Scarf2	0
Pin1rt1	0	Sbp	0	Mei1	0	Scarf1	0
Pin1	0	Sbno1	0	Ap5m1	0	Scarb1	0
Pim3	0	Sbk3	0	Tmem104	0	Scara5	0
Pim2	0	Sbk2	0	Sycp2	0	Scara3	0
Pim1	0	Sbk1	0	Olfr895	0	Scaper	0
Pilrb1	0	Sbf1	0	Gm21975	0	Scap	0
Pik3r6	0	Sbds	0	Kcnab1	0	Scamp5	0
Pik3r5	0	Saxo2	0	Casp7	0	Scamp4	0
Pik3r4	0	Saxo1	0	Mtg2	0	Scamp1	0
Pik3r3	0	Sav1	0	Ctnna3	0	Scaf8	0
Pik3r2	0	Sat2	0	Gckr	0	Scaf4	0
Pik3r1	0	Sat1	0	Ap5s1	0	Scaf1	0
Pik3cd	0	Sash3	0	Rhox4d	0	Sc5d	0
Pik3cb	0	Sart3	0	Gm572	0	Sbspon	0
Pik3ca	0	Sars2	0	Ppp1r12a	0	Sbpl	0
Pik3c3	0	Sars	0	Camsap3	0	Sbno1	0
Pik3c2g	0	Sarnp	0	Mllt11	0	Sbk2	0
Pik3c2b	0	Sarm1	0	D8Ertd82e	0	Sbk1	0
Pik3c2a	0	Sardh	0	Cfap97	0	Saysd1	0
Pik3ap1	0	Sar1b	0	Msl1	0	Saxo2	0
Pih1d3	0	Sar1a	0	Ddx49	0	Saxo1	0
Pih1d2	0	Sapcd2	0	Plscr1	0	Sav1	0
Pih1d1	0	Sap30l	0	Ctsk	0	Satl1	0
Pigz	0	Sap30bp	0	2410131K14Rik	0	Satb2	0
Pigyl	0	Sap30	0	Ucn3	0	Sat2	0

Pigx	0
Pigw	0
Pigt	0
Pigs	0
Pigr	0
Pigq	0
Pigp	0
Pign	0
Pigm	0
Pigl	0
Pigh	0
Pigg	0
Pigf	0
Pigc	0
Pigb	0
Piga	0
Pifo	0
Pif1	0
Pidd1	0
Pid1	0
Pick1	0
Picalm	0
Pibf1	0
Pias4	0
Pias2	0
Pias1	0
Pi4kb	0
Pi4ka	0
Pi4k2b	0
Pi4k2a	0
Pi16	0
Pi15	0
Phykpl	0
Phyhip	0
Phyhd1	0
Phyh	0
Phtf2	0
Phrf1	0
Phox2b	0
Phox2a	0
Phospho1	0
Phlpp2	0
Phlpp1	0
Phldb2	0
Phldb1	0
Phlda3	0
Phlda2	0
Phkg1	0
Phkb	0
Phka2	0
Phka1	0
Phip	0
Phgdh	0
Phf8	0
Phf7	0
Phf6	0
Phf5a	0
Phf3	0
Phf23	0
Phf21b	0
Phf20l1	0
Phf20	0
Phf2	0
Phf13	0
Phf12	0
Phf11d	0
Phf11c	0
Phf11a	0
Phf10	0
Phf1	0
Phc3	0
Phc1	0
Phb2	0
Phb	0
Phax	0
Phactr2	0
Pgs1	0
Pgrmc1	0
Pgr15l	0
Pgr	0
Pgpep1l	0
Pgpep1	0
Sap25	0
Sap18	0
Sap130	0
Samt4	0
Samt3	0
Samt2	0
Samsn1	0
Samm50	0
Samd9l	0
Samd7	0
Samd3	0
Samd15	0
Samd14	0
Samd12	0
Samd11	0
Samd10	0
Samd1	0
Sall3	0
Sall2	0
Sall1	0
Sacm1l	0
Sac3d1	0
Saal1	0
Saa4	0
Saa1	0
S1pr5	0
S1pr4	0
S1pr3	0
S1pr1	0
S100z	0
S100pbp	0
S100g	0
S100a9	0
S100a7a	0
S100a6	0
S100a5	0
S100a4	0
S100a3	0
S100a2	0
S100a16	0
S100a14	0
S100a11	0
Ryr2	0
Ryr1	0
Ryk	0
Rxrb	0
Rxra	0
Rxfp4	0
Rxfp3	0
Rxfp1	0
Rwdd4a	0
Rwdd3	0
Rwdd2a	0
Ruvbl2	0
Ruvbl1	0
Rusc1	0
Runx3	0
Runx1t1	0
Rundc3a	0
Rundc1	0
Rufy4	0
Rufy3	0
Rufy2	0
Rufy1	0
Rtp4	0
Rtp3	0
Rtp1	0
Rtn4rl2	0
Rtn4r	0
Rtn3	0
Rtl1	0
Rtkn	0
Rtfdc1	0
Rtf1	0
Rtel1	0
Rtcb	0
Rtca	0
Rsrp1	0
Rsrc1	0
Rspry1	0
Rspo4	0
Rspo1	0
Grid2	0
Ddx41	0
Ctsa	0
Ctsb	0
Ddx42	0
Ctsd	0
Adam12	0
Ddx47	0
Ddx46	0
Gpr179	0
Ero1lb	0
Ctsz	0
Khk	0
Amd2	0
Evi2a	0
Rab15	0
Ctsr	0
Cav1	0
Gpr174	0
Gpr176	0
Kcnab3	0
Bbox1	0
Clpsl2	0
Atad5	0
Prrt4	0
Micall2	0
Col14a1	0
Hoxd13	0
Hoxd10	0
Atad2	0
Unc119	0
Srgn	0
Tst	0
Mapk1	0
Mapk6	0
Mapk7	0
Mapk4	0
BRDN0000737921	0
Gabra5	0
Gabra4	0
Eif5a2	0
Mapk9	0
Urgcp	0
BRDN0000737922	0
Gabra3	0
Gabra2	0
Hoxc9	0
Hoxc8	0
Mrvi1	0
Safb	0
Spata31d1b	0
Kctd8	0
Ptges3	0
Ptges2	0
Hoxc5	0
Kat2b	0
Kat2a	0
Hoxc6	0
Cts8	0
Pcdhgb2	0
Pcdhgb1	0
Pcdhgb7	0
Pcdhgb6	0
Pcdhgb5	0
Pcdhgb4	0
Fbln2	0
Trim62	0
Trim63	0
Fbln7	0
Trim65	0
Fbln5	0
Trim67	0
Socs5	0
BRDN0000737670	0
Socs7	0
BRDN0000738020	0
Socs3	0
Dhrs9	0
Dhrs7	0
Dhrs4	0
Eif2d	0
Dhrs2	0
Sat1	0
Sash1	0
Sart3	0
Sars	0
Sarnp	0
Saraf	0
Sar1a	0
Sapcd2	0
Sapcd1	0
Sap30l	0
Sap30bp	0
Sap30	0
Sap25	0
Sap18	0
Sap130	0
Samt3	0
Samsn1	0
Samm50	0
Samd9l	0
Samd7	0
Samd5	0
Samd4	0
Samd3	0
Samd15	0
Samd14	0
Samd12	0
Samd11	0
Samd10	0
Samd1	0
Sall3	0
Sall1	0
Sag	0
Safb2	0
Safb	0
Sae1	0
Sacm1l	0
Saal1	0
Saa4	0
Saa3	0
Saa1	0
S1pr5	0
S1pr1	0
S100z	0
S100pbp	0
S100b	0
S100a7a	0
S100a6	0
S100a5	0
S100a4	0
S100a3	0
S100a2	0
S100a10	0
Ryr2	0
Ryr1	0
Ryk	0
Rxrg	0
Rxrb	0
Rxfp4	0
Rxfp3	0
Rxfp2	0
Rxfp1	0
Rwdd4a	0
Rwdd3	0
Rwdd2b	0
Ruvbl1	0
Rusc1	0
Runx1t1	0
Rundc3b	0
Rundc3a	0
Rundc1	0
Rufy4	0
Rufy3	0
Rufy2	0
Rttn	0
Rtp4	0
Rtp3	0
Rtp2	0
Rtp1	0
Rtn4rl1	0
Rtn4ip1	0
Rtn3	0
Rtn2	0

Pgp	0
Pgm1	0
Pglyrp4	0
Pglyrp3	0
Pglyrp2	0
Pglyrp1	0
Pgls	0
Pgk1	0
Pggt1b	0
Pgd	0
Pgc	0
Pgbd5	0
Pgbd1	0
Pgap3	0
Pgap1	0
Pgam2	0
Pgam1	0
Pfpl	0
Pfn4	0
Pfn3	0
Pfn1	0
Pfkp	0
Pfkm	0
Pfkl	0
Pfkfb4	0
Pfkfb3	0
Pfkfb2	0
Pfdn6	0
Pfdn5	0
Pfdn4	0
Pfdn2	0
Pfdn1	0
Pfas	0
Pf4	0
Pex7	0
Pex6	0
Pex5l	0
Pex5	0
Pex3	0
Pex2	0
Pex19	0
Pex14	0
Pex13	0
Pex12	0
Pex11g	0
Pex11b	0
Pex10	0
Pex1	0
Pet2	0
Pet117	0
Pet100	0
Pes1	0
Perp	0
Per3	0
Per2	0
Peo1	0
Penk	0
Pemt	0
Pelp1	0
Peli2	0
Peli1	0
Peg12	0
Peg10	0
Pef1	0
Pecr	0
Pebp4	0
Pebp1	0
Pear1	0
Pea15a	0
Pdzrn4	0
Pdzph1	0
Pdzk1	0
Pdzd8	0
Pdzd3	0
Pdzd11	0
Pdxk	0
Pdx1	0
Pdss2	0
Pdss1	0
Pds5b	0
Pdrg1	0
Pdpr	0
Rsph9	0
Rsph6a	0
Rsph4a	0
Rsph3b	0
Rsph3a	0
Rsph14	0
Rsph1	0
Rslcan18	0
Rsl24d1	0
Rsl1d1	0
Rsg1	0
Rsf1	0
Rsbn1l	0
Rsbn1	0
Rsad2	0
Rsad1	0
Rrs1	0
Rrp8	0
Rrp7a	0
Rrp36	0
Rrp1b	0
Rrp15	0
Rrp12	0
Rrp1	0
Rrnad1	0
Rrn3	0
Rrm2b	0
Rrm2	0
Rrm1	0
Rrh	0
Rrbp1	0
Rras2	0
Rras	0
Rragb	0
Rraga	0
Rqcd1	0
Rpusd4	0
Rpusd3	0
Rpusd1	0
Rptor	0
Rptn	0
Rpsa	0
Rps9	0
Rps8	0
Rps7	0
Rps6kb2	0
Rps6kb1	0
Rps6ka6	0
Rps6ka5	0
Rps6ka4	0
Rps6ka3	0
Rps6ka2	0
Rps6ka1	0
Rps6	0
Rps5	0
Rps4x	0
Rps3a1	0
Rps29	0
Rps28	0
Rps27rt	0
Rps27l	0
Rps27	0
Rps26	0
Rps25	0
Rps24	0
Rps23	0
Rps21	0
Rps20	0
Rps2	0
Rps19bp1	0
Rps19	0
Rps18	0
Rps16	0
Rps15a	0
Rps15	0
Rps14	0
Rps13	0
Rps12	0
Rps11	0
Rps10	0
Rprml	0
Rprm	0
Retn	0
Eif2a	0
Dhrs1	0
Spaca5	0
BRDN0000737445	0
BRDN0000737673	0
Neil1	0
Ppp1cb	0
Neil3	0
Tnn	0
1700020L24Rik	0
Hey2	0
Pdzph1	0
BRDN0000737449	0
Hey1	0
Smarcc2	0
Smarcc1	0
BRDN0000737676	0
Cyp2a12	0
Rhox4c	0
Gna14	0
Gna15	0
Gk5	0
Gna13	0
Cdsn	0
Gna11	0
Clic5	0
Cysltr2	0
Hgh1	0
Carkd	0
Cysltr1	0
Heyl	0
Xpot	0
Cdkn1c	0
Cdkn1b	0
Cdkn1a	0
Trem2	0
Trmt10b	0
Tmem132a	0
Chpt1	0
Gm4567	0
Nfkbil1	0
Txnip	0
AI607873	0
Scai	0
Incenp	0
Oxld1	0
Tef	0
Lix1l	0
Cds1	0
Cds2	0
Macf1	0
Aldoart2	0
Nt5c2	0
Cacna1g	0
BRDN0000738190	0
Rbsn	0
Sv2a	0
Scap	0
Sv2b	0
Klc4	0
Olfr992	0
Nucb2	0
Tomm7	0
Sbno1	0
Eln	0
Fmod	0
Ell	0
Hspa14	0
Esco2	0
Esco1	0
Tor1aip1	0
Smek1	0
Nol8	0
Smek2	0
Tpk1	0
Nol7	0
Nol6	0
Fam117b	0
Chrna5	0
Fnbp1l	0
Efnb2	0
Rtl1	0
Rtkn2	0
Rtfdc1	0
Rtf1	0
Rtel1	0
Rtcb	0
Rtca	0
Rsu1	0
Rsrc1	0
Rspo3	0
Rspo2	0
Rspo1	0
Rsph9	0
Rsph6a	0
Rsph3a	0
Rsph1	0
Rslcan18	0
Rsl24d1	0
Rsl1d1	0
Rsg1	0
Rsc1a1	0
Rsbn1l	0
Rsbn1	0
Rsad1	0
Rrs1	0
Rrp9	0
Rrp8	0
Rrp7a	0
Rrp36	0
Rrp1b	0
Rrp15	0
Rrp12	0
Rrnad1	0
Rrn3	0
Rrm2b	0
Rrm2	0
Rrm1	0
Rreb1	0
Rrbp1	0
Rras	0
Rragd	0
Rragc	0
Rraga	0
Rrad	0
Rqcd1	0
Rpusd4	0
Rpusd3	0
Rpusd1	0
Rptn	0
Rpsa	0
Rps9	0
Rps8	0
Rps7	0
Rps6kl1	0
Rps6kb1	0
Rps6ka6	0
Rps6ka5	0
Rps6ka4	0
Rps6ka3	0
Rps6ka2	0
Rps6ka1	0
Rps5	0
Rps4x	0
Rps3a1	0
Rps29	0
Rps28	0
Rps27rt	0
Rps27l	0
Rps27a	0
Rps27	0
Rps26	0
Rps25	0
Rps24	0
Rps21	0
Rps20	0
Rps2	0
Rps19bp1	0
Rps19	0
Rps18	0
Rps17	0
Rps15a	0
Rps15	0

Pdpn	0
Pdpk1	0
Pdp1	0
Pdlim5	0
Pdlim4	0
Pdlim3	0
Pdlim1	0
Pdk4	0
Pdk3	0
Pdk1	0
Pdilt	0
Pdik1l	0
Pdia6	0
Pdia5	0
Pdia4	0
Pdia3	0
Pdhx	0
Pdhb	0
Pdha1	0
Pdgfrl	0
Pdgfrb	0
Pdgfd	0
Pdgfb	0
Pdgfa	0
Pdf	0
Pde9a	0
Pde8a	0
Pde7b	0
Pde7a	0
Pde6h	0
Pde6g	0
Pde6d	0
Pde6c	0
Pde6b	0
Pde5a	0
Pde4dip	0
Pde4b	0
Pde4a	0
Pde3b	0
Pde3a	0
Pde2a	0
Pde1c	0
Pde1a	0
Pde12	0
Pdcl3	0
Pdcl2	0
Pdcl	0
Pdcd7	0
Pdcd6ip	0
Pdcd6	0
Pdcd5	0
Pdcd2	0
Pdcd1lg2	0
Pdcd11	0
Pdcd10	0
Pdap1	0
Pcyt1a	0
Pcyox1l	0
Pcyox1	0
Pcx	0
Pctp	0
Pcsk9	0
Pcsk7	0
Pcsk6	0
Pcsk5	0
Pcsk4	0
Pcsk2	0
Pcsk1n	0
Pcp4l1	0
Pcp2	0
Pcolce2	0
Pcolce	0
Pcnxl4	0
Pcnxl3	0
Pcnx	0
Pcnt	0
Pcnp	0
Pcna	0
Pcmtd2	0
Pcmtd1	0
Pcmt1	0
Pcm1	0
Rprd1b	0
Rpp40	0
Rpp38	0
Rpp30	0
Rpp25l	0
Rpp25	0
Rpp21	0
Rpn2	0
Rpn1	0
Rplp2	0
Rplp1	0
Rplp0	0
Rpl9	0
Rpl8	0
Rpl7l1	0
Rpl7a	0
Rpl7	0
Rpl6	0
Rpl5	0
Rpl41	0
Rpl4	0
Rpl3l	0
Rpl39	0
Rpl38	0
Rpl37a	0
Rpl37	0
Rpl36a	0
Rpl36	0
Rpl35a	0
Rpl35	0
Rpl34	0
Rpl32	0
Rpl31	0
Rpl30	0
Rpl3	0
Rpl28	0
Rpl27a	0
Rpl26	0
Rpl24	0
Rpl23a	0
Rpl23	0
Rpl22	0
Rpl21	0
Rpl19	0
Rpl18a	0
Rpl18	0
Rpl17	0
Rpl15	0
Rpl14	0
Rpl13	0
Rpl12	0
Rpl11	0
Rpl10l	0
Rpl10a	0
Rpl10	0
Rpia	0
Rph3al	0
Rph3a	0
Rpgrip1l	0
Rpgrip1	0
Rpgr	0
Rpf2	0
Rpf1	0
Rpe	0
Rpap3	0
Rpap1	0
Rpain	0
Rpa1	0
Rp9	0
Rp2h	0
Rp1l1	0
Rp1	0
Rorc	0
Rorb	0
Rora	0
Ror2	0
Ropn1	0
Romo1	0
Rogdi	0
Rock2	0
Robo4	0
Robo3	0
Olfr1256	0
Olfr1255	0
Fpr-rs4	0
Il1rl1	0
Il1rl2	0
Olfr1250	0
Bri3	0
Pkd1l3	0
Mst1	0
Olfr1259	0
Olfr1258	0
Ms4a6c	0
Ms4a6b	0
Ms4a6d	0
Chrna6	0
BRDN0000738280	0
Cluap1	0
Ppp1r12c	0
Sh3bp2	0
Pomgnt1	0
Serbp1	0
Adam1b	0
Gdpd3	0
Hspa1a	0
Gdpd1	0
Mcoln1	0
Mcoln3	0
Mogat1	0
Olfr934	0
Olfr935	0
Olfr936	0
Olfr937	0
Olfr930	0
Hspa1l	0
Npnt	0
Ypel3	0
Rspry1	0
Crabp1	0
Crabp2	0
Txndc8	0
Gm1110	0
Ccl28	0
Mstn	0
Htt	0
Cd163l1	0
Smad2	0
BRDN0000737453	0
Ccl20	0
Ccl22	0
Ccl25	0
Ccl24	0
Tmem151a	0
Ick	0
Rchy1	0
Taf7l	0
Cnp	0
Mpv17l	0
Zfp777	0
Gm2863	0
Avil	0
Cilp2	0
Upk1a	0
Rsf1	0
Aff2	0
BRDN0000737887	0
Brap	0
Aff1	0
Th	0
Ctnnbl1	0
Fcrl1	0
Mppe1	0
Mavs	0
Fcrl5	0
Fcrl6	0
Syna	0
Map4k1	0
Map4k3	0
Trim2	0
Isg20l2	0
Mis18a	0
BRDN0000737883	0
Wash1	0
Rps14	0
Rps13	0
Rps12	0
Rps11	0
Rps10	0
Rprd2	0
Rprd1b	0
Rpp40	0
Rpp38	0
Rpp30	0
Rpp25	0
Rpp21	0
Rpp14	0
Rpn2	0
Rpn1	0
Rplp2	0
Rplp1	0
Rplp0	0
Rpl9	0
Rpl8	0
Rpl7l1	0
Rpl7a	0
Rpl6	0
Rpl5	0
Rpl41	0
Rpl4	0
Rpl39l	0
Rpl39	0
Rpl38	0
Rpl37	0
Rpl36al	0
Rpl36a	0
Rpl36	0
Rpl35a	0
Rpl34	0
Rpl32	0
Rpl31	0
Rpl30	0
Rpl3	0
Rpl28	0
Rpl27a	0
Rpl27	0
Rpl26	0
Rpl24	0
Rpl23a	0
Rpl23	0
Rpl22l1	0
Rpl22	0
Rpl21	0
Rpl19	0
Rpl18a	0
Rpl18	0
Rpl17	0
Rpl15	0
Rpl14	0
Rpl13	0
Rpl12	0
Rpl11	0
Rpl10l	0
Rpl10a	0
Rpl10	0
Rpia	0
Rph3al	0
Rph3a	0
Rpgrip1l	0
Rpgrip1	0
Rpgr	0
Rpe	0
Rpap3	0
Rpap2	0
Rpap1	0
Rpain	0
Rpa3	0
Rpa2	0
Rpa1	0
Rp9	0
Rp2h	0
Rp1	0
Ros1	0
Ropn1l	0
Ropn1	0
Romo1	0

Pclo	0	Robo1	0	Tmem40	0	Rom1	0
Pck2	0	Rnps1	0	Prps1l1	0	Rock2	0
Pck1	0	Rnpepl1	0	Xcl1	0	Rock1	0
Pcif1	0	Rnpep	0	Adgra2	0	Robo1	0
Pcid2	0	Rnpc3	0	Fam217a	0	Rnps1	0
Pcgf6	0	Rnmtl1	0	Gm813	0	Rnpepl1	0
Pcgf5	0	Rnmt	0	Fam217b	0	Rnpep	0
Pcgf2	0	Rnh1	0	BRDN0000738364	0	Rnpc3	0
Pcf11	0	Rnft2	0	Lcn11	0	Rnft2	0
Pced1b	0	Rnf8	0	Tmem42	0	Rnft1	0
Pced1a	0	Rnf7	0	Lcn10	0	Rnf8	0
Pcdhgc3	0	Rnf6	0	Dtx4	0	Rnf7	0
Pcdhgb7	0	Rnf5	0	Rasgef1b	0	Rnf6	0
Pcdhgb6	0	Rnf44	0	Rasgef1a	0	Rnf44	0
Pcdhgb5	0	Rnf40	0	Top1mt	0	Rnf41	0
Pcdhgb4	0	Rnf4	0	Dis3l	0	Rnf4	0
Pcdhgb2	0	Rnf38	0	Nipal3	0	Rnf39	0
Pcdhga9	0	Rnf32	0	Dtx2	0	Rnf38	0
Pcdhga8	0	Rnf31	0	Dtx3	0	Rnf32	0
Pcdhga7	0	Rnf26	0	Ccdc71l	0	Rnf31	0
Pcdhga6	0	Rnf24	0	Fcrla	0	Rnf26	0
Pcdhga5	0	Rnf222	0	Hdlbp	0	Rnf24	0
Pcdhga4	0	Rnf220	0	Eif5	0	Rnf222	0
Pcdhga3	0	Rnf219	0	Eif6	0	Rnf220	0
Pcdhga2	0	Rnf217	0	Wdr60	0	Rnf217	0
Pcdhga12	0	Rnf216	0	Il23r	0	Rnf216	0
Pcdhga11	0	Rnf215	0	Fcrls	0	Rnf208	0
Pcdhga10	0	Rnf214	0	Unc13d	0	Rnf207	0
Pcdhb9	0	Rnf20	0	Coch	0	Rnf20	0
Pcdhb8	0	Rnf19b	0	Pola2	0	Rnf2	0
Pcdhb6	0	Rnf186	0	Pola1	0	Rnf19b	0
Pcdhb4	0	Rnf185	0	Cyb5r4	0	Rnf19a	0
Pcdhb22	0	Rnf182	0	Cyb5r3	0	Rnf186	0
Pcdhb21	0	Rnf181	0	Cyb5r2	0	Rnf185	0
Pcdhb2	0	Rnf170	0	Cyb5r1	0	Rnf183	0
Pcdhb19	0	Rnf168	0	Sh3d21	0	Rnf182	0
Pcdhb18	0	Rnf166	0	Mycbp	0	Rnf181	0
Pcdhb16	0	Rnf165	0	Prss1	0	Rnf180	0
Pcdhb15	0	Rnf157	0	Rapgef5	0	Rnf170	0
Pcdhb13	0	Rnf151	0	Rapgef6	0	Rnf17	0
Pcdhb12	0	Rnf150	0	Mtss1	0	Rnf168	0
Pcdhb11	0	Rnf149	0	Dmc1	0	Rnf167	0
Pcdhb10	0	Rnf148	0	Rapgef3	0	Rnf166	0
Pcdhb1	0	Rnf144b	0	Hcrtr1	0	Rnf165	0
Pcdhac2	0	Rnf144a	0	Prss8	0	Rnf157	0
Pcdhac1	0	Rnf141	0	Vta1	0	Rnf152	0
Pcdha9	0	Rnf14	0	Btf3l4	0	Rnf151	0
Pcdha8	0	Rnf139	0	Fbl	0	Rnf150	0
Pcdha7	0	Rnf138rt1	0	Lars	0	Rnf148	0
Pcdha4	0	Rnf138	0	Rbm6	0	Rnf145	0
Pcdha3	0	Rnf128	0	Cnih3	0	Rnf144b	0
Pcdha2	0	Rnf126	0	Cnih2	0	Rnf144a	0
Pcdha10	0	Rnf123	0	Rbm3	0	Rnf141	0
Pcdha1	0	Rnf122	0	Cnih4	0	Rnf139	0
Pcdh9	0	Rnf115	0	Gip	0	Rnf138	0
Pcdh8	0	Rnf113a2	0	Sele	0	Rnf135	0
Pcdh7	0	Rnf112	0	Adck4	0	Rnf130	0
Pcdh19	0	Rnf111	0	Adck5	0	Rnf126	0
Pcdh18	0	Rnf11	0	Adck2	0	Rnf125	0
Pcdh17	0	Rnf103	0	Adck1	0	Rnf122	0
Pcdh15	0	Rnf10	0	Pelo	0	Rnf121	0
Pcdh11x	0	Rnd2	0	Serpinf1	0	Rnf115	0
Pcdh10	0	Rnd1	0	Cbfa2t3	0	Rnf114	0
Pcdh1	0	Rnaset2b	0	Fbxo25	0	Rnf113a2	0
Pcca	0	Rnaset2a	0	Fbxo24	0	Rnf112	0
Pcbp4	0	Rnasel	0	Nde1	0	Rnf111	0
Pcbp3	0	Rnasek	0	Rpl36al	0	Rnf10	0
Pcbp2	0	Rnaseh2c	0	Scyl1	0	Rnd2	0
Pcbd1	0	Rnaseh2b	0	Selp	0	Rnd1	0
Pbxip1	0	Rnase9	0	Asf1a	0	Rnaset2b	0
Pbx4	0	Rnase6	0	Asf1b	0	Rnasel	0
Pbx3	0	Rnase4	0	Sp8	0	Rnasek	0
Pbx2	0	Rnase13	0	Sp9	0	Rnaseh1	0
Pbsn	0	Rnase12	0	Zfp574	0	Rnase9	0
Pbrm1	0	Rnase11	0	Zfp575	0	Rnase6	0
Pbld1	0	Rnase10	0	Hoxd11	0	Rnase4	0
Pbk	0	Rmnd5b	0	Gm5142	0	Rnase2b	0
Pbdc1	0	Rmi2	0	Col5a3	0	Rnase12	0
Paxbp1	0	Rmi1	0	Sp1	0	Rnase10	0
Pax9	0	Rmdn2	0	Sp2	0	Rmnd5b	0
Pax8	0	Rmdn1	0	Nog	0	Rmnd5a	0
Pax6	0	Rltpr	0	Gm5148	0	Rmnd1	0

Pax5	0
Pax4	0
Pax2	0
Patl2	0
Patl1	0
Pate4	0
Pate2	0
Pask	0
Parvg	0
Parvb	0
Parva	0
Pars2	0
Parpbp	0
Parp9	0
Parp8	0
Parp4	0
Parp3	0
Parp2	0
Parp16	0
Parp14	0
Parp12	0
Parp10	0
Parp1	0
Parn	0
Parm1	0
Parl	0
Park7	0
Park2	0
Pard6g	0
Pard6b	0
Pard6a	0
Pard3b	0
Pard3	0
Paqr9	0
Paqr8	0
Paqr7	0
Paqr6	0
Paqr4	0
Paqr3	0
Papss2	0
Papss1	0
Pappa2	0
Pappa	0
Papolb	0
Papl	0
Papd7	0
Papd4	0
Panx3	0
Panx1	0
Pank3	0
Pan3	0
Pam16	0
Pam	0
Palmd	0
Palm3	0
Palm2	0
Palm	0
Palld	0
Pald1	0
Palb2	0
Pak6	0
Pak4	0
Pak2	0
Pak1ip1	0
Pak1	0
Paip2b	0
Paip2	0
Paics	0
Pagr1a	0
Pag1	0
Pafah1b3	0
Pafah1b2	0
Pafah1b1	0
Paf1	0
Padi4	0
Padi3	0
Padi1	0
Pacsin3	0
Pacsin2	0
Pacsin1	0
Pacs1	0
Pacrg	0
Rln1	0
Rlf	0
Rlbp1	0
Rit2	0
Rit1	0
Ripply3	0
Ripply1	0
Ripk4	0
Ripk3	0
Ripk2	0
Ripk1	0
Riok3	0
Riok1	0
Rint1	0
Ring1	0
Rin3	0
Rin2	0
Rin1	0
Rims4	0
Rims3	0
Rims2	0
Rimklb	0
Rilpl2	0
Rilpl1	0
Rilp	0
Riiad1	0
Rictor	0
Ric8b	0
Ric3	0
Ric1	0
Ribc2	0
Rhpn2	0
Rhpn1	0
Rhox6	0
Rhox4e	0
Rhox4d	0
Rhox4c	0
Rhox4a	0
Rhox3g	0
Rhox3f	0
Rhox3e	0
Rhox3c	0
Rhox3a	0
Rhox2h	0
Rhox2g	0
Rhox2f	0
Rhox2b	0
Rhox13	0
Rhox12	0
Rhox11	0
Rhox10	0
Rhox1	0
Rhou	0
Rhot2	0
Rhot1	0
Rhoq	0
Rhog	0
Rhof	0
Rhoc	0
Rhobtb3	0
Rhobtb2	0
Rhob	0
Rho	0
Rhebl1	0
Rhcg	0
Rhbdl3	0
Rhbdl2	0
Rhbdl1	0
Rhbdf2	0
Rhbdf1	0
Rhbdd3	0
Rhbdd2	0
Rhbdd1	0
Rhag	0
Rgs8	0
Rgs7bp	0
Rgs7	0
Rgs5	0
Rgs22	0
Rgs20	0
Rgs2	0
Rgs19	0
4932429P05Rik	0
Prpf40a	0
Sp7	0
7420461P10Rik	0
BRDN0000737943	0
BRDN0000737940	0
Col5a1	0
Rnf219	0
BRDN0000737947	0
Slamf9	0
BRDN0000737945	0
Rnf215	0
Rnf214	0
BRDN0000737948	0
Rnf216	0
Pxmp4	0
Olfr592	0
Slamf1	0
Prss52	0
9430038I01Rik	0
Mterf2	0
Mag	0
Tbr1	0
Mterf4	0
Rbmx	0
Gm12789	0
Mrs2	0
Trpm8	0
Kdm3b	0
Trpm4	0
Olfr1305	0
Trpm6	0
Trpm7	0
N4bp2	0
Trpm1	0
Trpm2	0
Trpm3	0
Naaa	0
Dgkh	0
Ttc19	0
Spr	0
Hlx	0
Gse1	0
2610034B18Rik	0
Creb3l3	0
Spn	0
Olfr1391	0
Gm3696	0
D430042O09Rik	0
Oxnad1	0
Lacc1	0
Asnsd1	0
Ifnar2	0
Zfp106	0
Zfp105	0
Ifnar1	0
Zfp101	0
4930522H14Rik	0
Gprc5d	0
Gprc5a	0
Zfp109	0
Gprc5b	0
Cdc37	0
Nyap1	0
Tmem185b	0
Rlf	0
Akap14	0
Abt1	0
BRDN0000738128	0
BRDN0000737619	0
BRDN0000738209	0
Mycbp2	0
Arhgap36	0
Olfr348	0
H2-Aa	0
BRDN0000737613	0
Olfr344	0
Echdc3	0
Olfr341	0
Olfr340	0
Olfr1082	0
Dennd2c	0
Rmi1	0
Rln3	0
Rln1	0
Rlim	0
Rlbp1	0
Rit2	0
Rit1	0
Ripply2	0
Ripply1	0
Ripk4	0
Ripk3	0
Ripk2	0
Ripk1	0
Riok3	0
Riok1	0
Rint1	0
Rinl	0
Ring1	0
Rin3	0
Rin2	0
Rims2	0
Rimklb	0
Rimbp3	0
Rimbp2	0
Rilpl2	0
Rilpl1	0
Rilp	0
Rif1	0
Ric8	0
Ric3	0
Ric1	0
Ribc2	0
Rhpn2	0
Rhox9	0
Rhox8	0
Rhox6	0
Rhox4g	0
Rhox4f	0
Rhox4e	0
Rhox4c	0
Rhox4b	0
Rhox3h	0
Rhox3g	0
Rhox3f	0
Rhox3e	0
Rhox3c	0
Rhox3a	0
Rhox2h	0
Rhox2g	0
Rhox2f	0
Rhox2d	0
Rhov	0
Rhou	0
Rhot2	0
Rhot1	0
Rhoq	0
Rhoj	0
Rhoh	0
Rhog	0
Rhof	0
Rhod	0
Rhoc	0
Rhobtb2	0
Rhobtb1	0
Rhob	0
Rhoa	0
Rho	0
Rhebl1	0
Rhd	0
Rhcg	0
Rhbg	0
Rhbdl3	0
Rhbdl2	0
Rhbdl1	0
Rhbdf2	0
Rhbdf1	0
Rhbdd3	0
Rhbdd1	0
Rhag	0
Rgsl1	0
Rgs9	0
Rgs8	0

Pabpn1l	0	Rgs18	0	Olfr1080	0	Rgs7	0
Pabpn1	0	Rgs14	0	Dennd2a	0	Rgs4	0
Pabpc6	0	Rgs13	0	Olfr1086	0	Rgs22	0
Pabpc5	0	Rgs12	0	Olfr1087	0	Rgs19	0
Pabpc4l	0	Rgs11	0	Olfr1084	0	Rgs18	0
Pabpc2	0	Rgs10	0	Olfr1085	0	Rgs17	0
Pabpc1l	0	Rgr	0	Olfr1089	0	Rgs16	0
Pabpc1	0	Rgp1	0	AU018091	0	Rgs14	0
P4htm	0	Rgn	0	Uqcrb	0	Rgs12	0
P4hb	0	Rgmb	0	AI118078	0	Rgs1	0
P4ha3	0	Rgl2	0	Ldhd	0	Rgr	0
P4ha2	0	Rgcc	0	Sept14	0	Rgp1	0
P4ha1	0	Rgag4	0	Elf3	0	Rgmb	0
P3h4	0	Rgag1	0	Pmm1	0	Rgma	0
P3h3	0	Rfxank	0	Xrcc6bp1	0	Rgl3	0
P3h1	0	Rfx8	0	Sec61a1	0	Rgl2	0
P2ry6	0	Rfx7	0	Mettl11b	0	Rgl1	0
P2ry4	0	Rfx6	0	Sept12	0	Rgag4	0
P2ry2	0	Rfx5	0	Prl2b1	0	Rgag1	0
P2ry14	0	Rfx4	0	Dpy19l4	0	Rfxap	0
P2ry13	0	Rfx2	0	BRDN0000737751	0	Rfxank	0
P2ry12	0	Rfwd3	0	Defb19	0	Rfx8	0
P2ry10	0	Rft1	0	Ahr	0	Rfx7	0
P2rx6	0	Rfpl4b	0	Ptger4	0	Rfx6	0
P2rx5	0	Rfpl3s	0	Olfr169	0	Rfx5	0
P2rx4	0	Rfk	0	Olfr168	0	Rfx4	0
P2rx3	0	Rffl	0	Rbms1	0	Rfx3	0
P2rx2	0	Rfc5	0	Rbms3	0	Rfx1	0
P2rx1	0	Rfc4	0	Rbms2	0	Rfwd2	0
Oxt	0	Rfc3	0	Jag2	0	Rft1	0
Oxsr1	0	Rfc1	0	Olfr690	0	Rfpl4b	0
Oxsm	0	Rexo2	0	Jag1	0	Rfpl4	0
Oxr1	0	Rexo1	0	Olfr695	0	Rfpl3s	0
Oxnad1	0	Rex2	0	Aldh1l1	0	Rfng	0
Oxgr1	0	Rev3l	0	Olfr165	0	Rfk	0
Oxct1	0	Rev1	0	Olfr164	0	Rffl	0
Oxa1l	0	Retsat	0	Chst11	0	Rfesd	0
Ovol2	0	Retnlg	0	Chst10	0	Rfc5	0
Ovol1	0	Retn	0	Akr1c14	0	Rfc4	0
Ovgp1	0	Rest	0	Chst12	0	Rfc3	0
Ovca2	0	Resp18	0	Akr1c12	0	Rfc2	0
Otx1	0	Rergl	0	Chst14	0	Rfc1	0
Otulin	0	Rere	0	Bspry	0	Rexo4	0
Otud7a	0	Rer1	0	Bach2	0	Rex2	0
Otud6b	0	Reps1	0	Bach1	0	Rev3l	0
Otud6a	0	Repin1	0	Myl10	0	Retnlb	0
Otud5	0	Ren1	0	Akr1c19	0	Retnla	0
Otud4	0	Rem2	0	Bccip	0	Resp18	0
Otub2	0	Relt	0	Inpp4a	0	Rergl	0
Otub1	0	Reln	0	Foxb1	0	Rer1	0
Ott	0	Relb	0	Srd5a2	0	Reps2	0
Otp	0	Rela	0	Srd5a1	0	Reps1	0
Otos	0	Rel	0	Mcrs1	0	Rep15	0
Otor	0	Reg3d	0	Uggt2	0	Renbp	0
Otop2	0	Reg3b	0	Gm11237	0	Ren1	0
Otop1	0	Reg3a	0	BRDN0000738368	0	Rem1	0
Otol1	0	Reg2	0	Papl	0	Reln	0
Otogl	0	Reep5	0	Rfxank	0	Rell2	0
Otog	0	Reep3	0	Fam89b	0	Relb	0
Otof	0	Reep1	0	Hap1	0	Rela	0
Otoa	0	Recql5	0	Marf1	0	Rel	0
Otc	0	Recql4	0	Bnc2	0	Reg3g	0
Ostn	0	Recql	0	Mknk2	0	Reg3d	0
Ostm1	0	Rec114	0	Pnp	0	Reg3b	0
Ostf1	0	Rdx	0	Iqgap2	0	Reg3a	0
Ostc	0	Rdm1	0	Mknk1	0	Reg2	0
Ost4	0	Rdh8	0	Imp3	0	Reg1	0
Osr2	0	Rdh5	0	Pnn	0	Reep5	0
Osr1	0	Rdh19	0	Zkscan14	0	Reep4	0
Osmr	0	Rdh16	0	Zkscan17	0	Reep3	0
Osm	0	Rdh13	0	Wnt7b	0	Reep2	0
Osgin2	0	Rdh11	0	Bcl2l12	0	Reep1	0
Osgin1	0	Rdh10	0	Tcfl5	0	Recql5	0
Osgep	0	Rdh1	0	Nsl1	0	Recql4	0
Oser1	0	Rd3l	0	Klk1b1	0	Reck	0
Oscp1	0	Rcvrn	0	Lrrc8a	0	Rec8	0
Osbpl9	0	Rcsd1	0	Zfp810	0	Rec114	0
Osbpl8	0	Rcor1	0	Lrrc8c	0	Rdx	0
Osbpl6	0	Rcn3	0	Mrpl24	0	Rdm1	0
Osbpl5	0	Rcn2	0	Rpe65	0	Rdh9	0
Osbpl2	0	Rcn1	0	Lgmn	0	Rdh7	0
Osbpl1a	0	Rcl1	0	4933428G20Rik	0	Rdh19	0

Osbpl11	0	Rchy1	0	Zfp819	0	Rdh12	0
Osbp2	0	Rccd1	0	Rit1	0	Rdh1	0
Osbp	0	Rcc2	0	2010107E04Rik	0	Rcvrn	0
Os9	0	Rcc1	0	Mrfap1	0	Rcsd1	0
Ormdl3	0	Rcbtb1	0	Trim55	0	Rcor1	0
Ormdl1	0	Rcan3	0	Slc51b	0	Rcn3	0
Orm3	0	Rcan1	0	Cyp27b1	0	Rcn1	0
Orm2	0	Rc3h2	0	Krtap24-1	0	Rcl1	0
Orm1	0	Rc3h1	0	BRDN0000737444	0	Rce1	0
Orc6	0	Rbx1	0	Galp	0	Rcc2	0
Orc5	0	Rbsn	0	1810009J06Rik	0	Rcc1	0
Orc4	0	Rbpms2	0	Cxcl16	0	Rcan2	0
Orc3	0	Rbpjl	0	A730017C20Rik	0	Rc3h2	0
Orc1	0	Rbpj	0	Klk1b4	0	Rc3h1	0
Oraov1	0	Rbp7	0	Gcnt4	0	Rbx1	0
Orai3	0	Rbp4	0	Mybl2	0	Rbpms2	0
Orai2	0	Rbp3	0	Scarb1	0	Rbpms	0
Orai1	0	Rbp2	0	Defb15	0	Rbpjl	0
Optn	0	Rbp1	0	H1fnt	0	Rbpj	0
Optc	0	Rbmxl2	0	Bpifa3	0	Rbp4	0
Oprm1	0	Rbmx	0	Nr2f1	0	Rbp3	0
Oprl1	0	Rbms1	0	Bpifa6	0	Rbp1	0
Oprk1	0	Rbm8a	0	1110001J03Rik	0	Rbmx2	0
Opn4	0	Rbm6	0	Fbxo2	0	Rbmx	0
Opn3	0	Rbm5	0	Fbxo3	0	Rbms2	0
Opn1sw	0	Rbm4b	0	Fbxo4	0	Rbms1	0
Ophn1	0	Rbm47	0	Fbxo5	0	Rbm8a	0
Opalin	0	Rbm45	0	Fbxo6	0	Rbm6	0
Opa3	0	Rbm44	0	Rad9b	0	Rbm5	0
Opa1	0	Rbm43	0	Mroh5	0	Rbm4b	0
Oosp3	0	Rbm42	0	Fbxo9	0	Rbm47	0
Oosp2	0	Rbm41	0	Mroh7	0	Rbm45	0
Oosp1	0	Rbm39	0	Mroh6	0	Rbm42	0
Oog3	0	Rbm38	0	Vsig4	0	Rbm39	0
Oog2	0	Rbm33	0	BRDN0000737908	0	Rbm38	0
Ooep	0	Rbm3	0	Rmnd1	0	Rbm34	0
Onecut3	0	Rbm27	0	Capns1	0	Rbm33	0
Onecut2	0	Rbm25	0	Lrrc23	0	Rbm3	0
Onecut1	0	Rbm24	0	Vrtn	0	Rbm27	0
Omt2b	0	Rbm22	0	Capns2	0	Rbm26	0
Omt2a	0	Rbm20	0	Tatdn1	0	Rbm25	0
Omp	0	Rbm19	0	Mr1	0	Rbm24	0
Omg	0	Rbm18	0	Rrp7a	0	Rbm22	0
Omd	0	Rbm17	0	Terf2	0	Rbm19	0
Oma1	0	Rbm14	0	Pla2g7	0	Rbm18	0
Olr1	0	Rbm12b2	0	Nfam1	0	Rbm17	0
Olig3	0	Rbm12b1	0	Pla2g5	0	Rbm15	0
Olig2	0	Rbm10	0	Pla2g3	0	Rbm14	0
Olfr998	0	Rbl2	0	Vsig1	0	Rbm12b1	0
Olfr996	0	Rbl1	0	Gm8660	0	Rbm12	0
Olfr995	0	Rbks	0	Aff3	0	Rbm10	0
Olfr994	0	Rbck1	0	Fhod1	0	Rbl2	0
Olfr993	0	Rbbp9	0	Slc6a9	0	Rbl1	0
Olfr992	0	Rbbp8nl	0	Rbpms	0	Rbfox3	0
Olfr99	0	Rbbp8	0	8030411F24Rik	0	Rbfox2	0
Olfr988	0	Rbbp5	0	Gm15056	0	Rbfa	0
Olfr987	0	Rbbp4	0	Rab3d	0	Rbck1	0
Olfr986	0	Rbak	0	Rab3c	0	Rbbp9	0
Olfr983	0	Rb1	0	Rab3b	0	Rbbp8nl	0
Olfr982	0	Raver2	0	Rab3a	0	Rbbp8	0
Olfr981	0	Raver1	0	Nae1	0	Rbbp7	0
Olfr980	0	Rassf9	0	Defb10	0	Rbbp6	0
Olfr98	0	Rassf8	0	BRDN0000738202	0	Rbbp5	0
Olfr979	0	Rassf6	0	Nr2f2	0	Rbbp4	0
Olfr978	0	Rasl2-9	0	Imp4	0	Rb1cc1	0
Olfr976	0	Rasl12	0	Cbln4	0	Rb1	0
Olfr975	0	Rasl11b	0	Qtrt1	0	Raver1	0
Olfr974	0	Rasl10b	0	Cbln2	0	Rassf9	0
Olfr972	0	Rasip1	0	Cbln3	0	Rassf8	0
Olfr971	0	Rasgrp1	0	Cbln1	0	Rassf4	0
Olfr970	0	Rasgrf1	0	Rab11a	0	Rassf3	0
Olfr97	0	Rasef	0	Oxct2a	0	Rassf2	0
Olfr969	0	Rasd2	0	Osbpl8	0	Rassf1	0
Olfr967	0	Rasd1	0	Rab11b	0	Rasl2-9	0
Olfr965	0	Rasal3	0	Zfp748	0	Rasl12	0
Olfr961	0	Rasal1	0	Galm	0	Rasgrp4	0
Olfr96	0	Rasa3	0	Pla2g4c	0	Rasgrp3	0
Olfr959	0	Rasa1	0	Slc47a2	0	Rasgrp2	0
Olfr957	0	Rars2	0	Slc47a1	0	Rasgrp1	0
Olfr954	0	Rars	0	Zfp746	0	Rasef	0
Olfr952	0	Rara	0	Osbpl6	0	Rasd2	0
Olfr951	0	Raph1	0	Gm11938	0	Rasd1	0

Olfr95	0	Rapgef6	0	Pla2g4e	0	Rasal3	0
Olfr948	0	Rapgef5	0	Defb11	0	Rasal1	0
Olfr945	0	Rap2b	0	Cblb	0	Rasa4	0
Olfr943	0	Rap2a	0	Rab35	0	Rasa2	0
Olfr94	0	Rap1gap2	0	Rab34	0	Rasa1	0
Olfr938	0	Rap1gap	0	Rab31	0	Rars2	0
Olfr937	0	Rap1b	0	Rab30	0	Rars	0
Olfr936	0	Rangrf	0	Yipf6	0	Rarres2	0
Olfr934	0	Rangap1	0	Gm906	0	Rarb	0
Olfr933	0	Ranbp9	0	Mpst	0	Rara	0
Olfr930	0	Ranbp6	0	Rab39	0	Rapsn	0
Olfr93	0	Ranbp3	0	Scgb1b30	0	Raph1	0
Olfr924	0	Ranbp2	0	Gm15315	0	Rapgefl1	0
Olfr923	0	Ranbp17	0	Spock1	0	Rapgef6	0
Olfr922	0	Ranbp1	0	Spock2	0	Rapgef4	0
Olfr920	0	Ran	0	Spock3	0	Rapgef1	0
Olfr919	0	Ramp3	0	Sapcd1	0	Rap2c	0
Olfr918	0	Ramp2	0	Hapln4	0	Rap2b	0
Olfr917	0	Ramp1	0	Sapcd2	0	Rap2a	0
Olfr916	0	Ralyl	0	Tspo	0	Rap1b	0
Olfr914	0	Ralgps2	0	Defb12	0	Rap1a	0
Olfr913	0	Ralgps1	0	Hapln3	0	Rangap1	0
Olfr912	0	Ralgapb	0	Hapln2	0	Ranbp9	0
Olfr911-ps1	0	Ralgapa2	0	Mthfd2l	0	Ranbp3	0
Olfr910	0	Ralgapa1	0	Wnt3a	0	Ranbp17	0
Olfr91	0	Ralbp1	0	Tnfaip8	0	Ranbp1	0
Olfr907	0	Rala	0	Dbpht2	0	Ran	0
Olfr906	0	Rai2	0	Slc22a19	0	Ramp1	0
Olfr905	0	Rai14	0	Slc22a16	0	Ralyl	0
Olfr904	0	Rag2	0	Slc22a17	0	Raly	0
Olfr902	0	Rag1	0	Slc22a14	0	Ralgapa2	0
Olfr901	0	Raf1	0	Slc22a15	0	Ralgapa1	0
Olfr90	0	Raet1d	0	Tnfaip1	0	Ralbp1	0
Olfr9	0	Rae1	0	Slc22a13	0	Rai14	0
Olfr898	0	Radil	0	Mfhas1	0	Rai1	0
Olfr895	0	Rad9a	0	Tnfaip2	0	Rag2	0
Olfr894	0	Rad54l2	0	Sox2	0	Raet1e	0
Olfr893	0	Rad54b	0	Ndor1	0	Raet1d	0
Olfr891	0	Rad51d	0	BRDN0000737779	0	Radil	0
Olfr890	0	Rad51c	0	Gm5901	0	Rad9b	0
Olfr889	0	Rad51ap2	0	1700021K19Rik	0	Rad9a	0
Olfr888	0	Rad51ap1	0	BRDN0000737452	0	Rad54l2	0
Olfr887	0	Rad50	0	Filip1	0	Rad54l	0
Olfr885	0	Rad23b	0	C1qb	0	Rad54b	0
Olfr884	0	Rad21l	0	Elac1	0	Rad51d	0
Olfr883	0	Rad21	0	Mtrf1l	0	Rad51c	0
Olfr881	0	Rad17	0	Adam6a	0	Rad51ap2	0
Olfr878	0	Rad1	0	Actc1	0	Rad51ap1	0
Olfr875	0	Racgap1	0	Adam6b	0	Rad50	0
Olfr874	0	Rac3	0	Heatr5b	0	Rad23b	0
Olfr873	0	Rac2	0	Herc3	0	Rad23a	0
Olfr872	0	Rac1	0	Pdzd4	0	Rad21l	0
Olfr871	0	Rabl6	0	Adra2b	0	Rad21	0
Olfr870	0	Rabl3	0	Car10	0	Rad18	0
Olfr869	0	Rabl2	0	Car11	0	Rad17	0
Olfr866	0	Rabif	0	Car12	0	Rad1	0
Olfr862	0	Rabggtb	0	Car13	0	Racgap1	0
Olfr859	0	Rabggta	0	Car14	0	Rac1	0
Olfr857	0	Rabgap1l	0	Car15	0	Rabl6	0
Olfr855	0	Rabgap1	0	Pex11g	0	Rabl3	0
Olfr854	0	Rabep1	0	Cep68	0	Rabl2	0
Olfr853	0	Rabac1	0	Arv1	0	Rabif	0
Olfr851	0	Rab9b	0	Tcl1b5	0	Rabggtb	0
Olfr850	0	Rab8b	0	Hrnr	0	Rabgap1l	0
Olfr849	0	Rab7	0	Gnl2	0	Rabgap1	0
Olfr847	0	Rab6b	0	Aldh3a2	0	Rabep1	0
Olfr846	0	Rab6a	0	Aldh3a1	0	Rab9b	0
Olfr845	0	Rab5a	0	Tnfsf9	0	Rab8b	0
Olfr843	0	Rab4b	0	Gnl1	0	Rab8a	0
Olfr836	0	Rab4a	0	Cttn	0	Rab7	0
Olfr835	0	Rab44	0	BRDN0000737633	0	Rab6b	0
Olfr834	0	Rab43	0	AI846148	0	Rab6a	0
Olfr832	0	Rab42	0	Dot1l	0	Rab5a	0
Olfr830	0	Rab40c	0	Fbxw17	0	Rab4b	0
Olfr829	0	Rab40b	0	Arhgef10	0	Rab4a	0
Olfr828	0	Rab3ip	0	Arhgef11	0	Rab43	0
Olfr827	0	Rab3il1	0	Arhgef12	0	Rab42	0
Olfr826	0	Rab3gap2	0	Eddm3b	0	Rab3ip	0
Olfr825	0	Rab3gap1	0	Arhgef15	0	Rab3gap2	0
Olfr824	0	Rab3c	0	Arhgef16	0	Rab3gap1	0
Olfr823	0	Rab39	0	Arhgef17	0	Rab3d	0
Olfr822	0	Rab38	0	Arhgef18	0	Rab3c	0

Olfr821	0	Rab37	0	Enpp4	0	Rab3b	0
Olfr820	0	Rab36	0	Psapl1	0	Rab39	0
Olfr818	0	Rab35	0	Enpp3	0	Rab37	0
Olfr816	0	Rab34	0	Tollip	0	Rab35	0
Olfr815	0	Rab32	0	Enpp1	0	Rab34	0
Olfr814	0	Rab31	0	Kcnt2	0	Rab33b	0
Olfr813	0	Rab30	0	Ankrd42	0	Rab32	0
Olfr811	0	Rab2b	0	Pja1	0	Rab31	0
Olfr810	0	Rab29	0	Pja2	0	Rab30	0
Olfr809	0	Rab28	0	Zbtb7b	0	Rab2b	0
Olfr808	0	Rab27b	0	Elfn1	0	Rab27a	0
Olfr806	0	Rab27a	0	Elfn2	0	Rab25	0
Olfr805	0	Rab25	0	Zc3h13	0	Rab24	0
Olfr803	0	Rab23	0	Rab8b	0	Rab23	0
Olfr802	0	Rab21	0	Atp6v1f	0	Rab22a	0
Olfr801	0	Rab20	0	Atp6v1a	0	Rab21	0
Olfr800	0	Rab18	0	AU040320	0	Rab20	0
Olfr798	0	Rab17	0	Dusp5	0	Rab1b	0
Olfr796	0	Rab15	0	Sirpb1b	0	Rab19	0
Olfr792	0	Rab11fip1	0	Atp6v1h	0	Rab17	0
Olfr791	0	Rab11b	0	Prr5	0	Rab11fip5	0
Olfr790	0	Rab11a	0	Nat2	0	Rab11fip3	0
Olfr788	0	Rab10	0	Zcchc10	0	Rab11b	0
Olfr787	0	R3hdml	0	Gm17365	0	Rab11a	0
Olfr786	0	R3hdm4	0	Immt	0	R3hdml	0
Olfr784	0	R3hdm1	0	Zbtb32	0	R3hdm4	0
Olfr782	0	R3hcc1l	0	Zcchc16	0	R3hdm1	0
Olfr781	0	R3hcc1	0	Gpr153	0	R3hcc1l	0
Olfr780	0	Qtrtd1	0	Olfr101	0	Qrsl1	0
Olfr777	0	Qtrt1	0	Gpr150	0	Qrich2	0
Olfr776	0	Qrsl1	0	Gpr157	0	Qrich1	0
Olfr775	0	Qrfpr	0	Gpr156	0	Qrfpr	0
Olfr774	0	Qrfp	0	Gpr155	0	Qrfp	0
Olfr773	0	Qprt	0	Xlr5a	0	Qprt	0
Olfr772	0	Qpct	0	Xlr5b	0	Qpctl	0
Olfr771	0	Qk	0	Gpr158	0	Qpct	0
Olfr770	0	Qars	0	Try10	0	Qk	0
Olfr769	0	Pyroxd1	0	Mxra7	0	Qars	0
Olfr767	0	Pyhin1	0	Olfr102	0	Pzp	0
Olfr765	0	Pygo2	0	Krt23	0	Pyroxd1	0
Olfr763	0	Pygo1	0	BRDN0000737486	0	Pygo2	0
Olfr750	0	Pygl	0	Aldh3b2	0	Pydc4	0
Olfr749	0	Pygb	0	BRDN0000738340	0	Pycrl	0
Olfr748	0	Pydc4	0	Zfp740	0	Pycr2	0
Olfr747	0	Pydc3	0	Pcid2	0	Pycr1	0
Olfr746	0	Pycrl	0	Uspl1	0	Pycard	0
Olfr745	0	Pycr2	0	Mettl25	0	Pxylp1	0
Olfr744	0	Pycr1	0	BRDN0000738358	0	Pxt1	0
Olfr743	0	Pycard	0	Defa25	0	Pxn	0
Olfr742	0	Pxylp1	0	Klk1b24	0	Pxmp4	0
Olfr740	0	Pxt1	0	Hoxa7	0	Pxk	0
Olfr74	0	Pxn	0	Hoxa6	0	Pxdn	0
Olfr739	0	Pxmp2	0	Hoxa5	0	Pwwp2b	0
Olfr736	0	Pwwp2b	0	Hoxa3	0	Pwwp2a	0
Olfr735	0	Pwwp2a	0	Hoxa2	0	Pwp2	0
Olfr734	0	Pwp2	0	Hoxa1	0	Pwp1	0
Olfr733	0	Pwp1	0	Nmnat3	0	Pvrl4	0
Olfr732	0	Pvrl4	0	Reep1	0	Pvrl3	0
Olfr731	0	Pvr	0	Pdpk1	0	Pvrl1	0
Olfr73	0	Pvalb	0	Reep3	0	Pvr	0
Olfr729	0	Pusl1	0	Reep2	0	Pvalb	0
Olfr728	0	Pus7l	0	Reep5	0	Pus7	0
Olfr727	0	Pus7	0	Hoxa9	0	Pus1	0
Olfr726	0	Pus3	0	Reep6	0	Purg	0
Olfr725	0	Pus10	0	BC048562	0	Purb	0
Olfr724	0	Purg	0	Ptpn5	0	Pum2	0
Olfr723	0	Purb	0	Nmnat1	0	Pum1	0
Olfr722	0	Pura	0	Nanog	0	Puf60	0
Olfr720	0	Pum2	0	Crkl	0	Ptx4	0
Olfr716	0	Puf60	0	Etl4	0	Pttg1ip	0
Olfr715	0	Ptx4	0	BRDN0000737586	0	Pttg1	0
Olfr714	0	Ptx3	0	Sh3rf1	0	Pts	0
Olfr713	0	Pttg1ip	0	Zim1	0	Ptrhd1	0
Olfr711	0	Pttg1	0	Acot7	0	Ptrh2	0
Olfr710	0	Pts	0	Clp1	0	Ptrh1	0
Olfr71	0	Ptrhd1	0	BRDN0000737584	0	Ptprt	0
Olfr707	0	Ptrh2	0	Pmm2	0	Ptprr	0
Olfr706	0	Ptrh1	0	Olfr203	0	Ptprq	0
Olfr702	0	Ptprt	0	BC094916	0	Ptprm	0
Olfr701	0	Ptprs	0	Rfng	0	Ptprk	0
Olfr700	0	Ptprr	0	Ift122	0	Ptprh	0
Olfr70	0	Ptprq	0	BC049715	0	Ptprf	0

Olfr698	0
Olfr694	0
Olfr692	0
Olfr690	0
Olfr69	0
Olfr689	0
Olfr686	0
Olfr684	0
Olfr683	0
Olfr681	0
Olfr68	0
Olfr679	0
Olfr678	0
Olfr675	0
Olfr672	0
Olfr671	0
Olfr670	0
Olfr67	0
Olfr669	0
Olfr668	0
Olfr667	0
Olfr666	0
Olfr661	0
Olfr658	0
Olfr656	0
Olfr655	0
Olfr652	0
Olfr65	0
Olfr649	0
Olfr648	0
Olfr646	0
Olfr645	0
Olfr644	0
Olfr642	0
Olfr641	0
Olfr640	0
Olfr64	0
Olfr639	0
Olfr638	0
Olfr635	0
Olfr632	0
Olfr631	0
Olfr630	0
Olfr629	0
Olfr628	0
Olfr624	0
Olfr620	0
Olfr62	0
Olfr618	0
Olfr616	0
Olfr613	0
Olfr612	0
Olfr611	0
Olfr610	0
Olfr61	0
Olfr609	0
Olfr608	0
Olfr606	0
Olfr605	0
Olfr603	0
Olfr601	0
Olfr600	0
Olfr60	0
Olfr6	0
Olfr598	0
Olfr597	0
Olfr596	0
Olfr594	0
Olfr593	0
Olfr592	0
Olfr591	0
Olfr59	0
Olfr589	0
Olfr586	0
Olfr585	0
Olfr584	0
Olfr583	0
Olfr58	0
Olfr578	0
Olfr577	0
Olfr576	0
Olfr575	0
Ptpro	0
Ptprn2	0
Ptprm	0
Ptprj	0
Ptprh	0
Ptprg	0
Ptprf	0
Ptprcap	0
Ptprc	0
Ptprb	0
Ptpra	0
Ptpn5	0
Ptpn4	0
Ptpn3	0
Ptpn23	0
Ptpn22	0
Ptpn21	0
Ptpn20	0
Ptpn2	0
Ptpn14	0
Ptpn13	0
Ptpn12	0
Ptpn11	0
Ptpn1	0
Ptpdc1	0
Ptp4a3	0
Ptp4a2	0
Ptp4a1	0
Ptov1	0
Ptms	0
Ptma	0
Ptk7	0
Ptk2	0
Pth2	0
Pth1r	0
Pth	0
Ptgs1	0
Ptgis	0
Ptgfrn	0
Ptges3l	0
Ptges2	0
Ptges	0
Ptger4	0
Ptger3	0
Ptger2	0
Ptgdr2	0
Ptgdr	0
Ptf1a	0
Pten	0
Ptdss2	0
Ptcd3	0
Ptcd2	0
Ptcd1	0
Ptbp3	0
Ptbp1	0
Ptar1	0
Ptafr	0
Pstk	0
Psrc1	0
Pspn	0
Psph	0
Pspc1	0
Psors1c2	0
Psmg4	0
Psmg3	0
Psmg2	0
Psme4	0
Psme2	0
Psmd8	0
Psmd7	0
Psmd6	0
Psmd5	0
Psmd3	0
Psmd2	0
Psmd14	0
Psmd13	0
Psmd12	0
Psmd11	0
Psmd10	0
Psmd1	0
Psmc6	0
Psmc5	0
Tatdn2	0
Olfr574	0
Pdgfa	0
Olfr577	0
Pdgfd	0
Olfr571	0
Tctn1	0
Pspn	0
Ccdc126	0
Ap2a2	0
Olfr578	0
Vtn	0
BRDN0000737421	0
Adra1a	0
BRDN0000737423	0
BRDN0000737422	0
BRDN0000737425	0
BRDN0000737424	0
BRDN0000737427	0
BRDN0000737426	0
BRDN0000737429	0
BRDN0000737428	0
Olfr205	0
Gfap	0
Npc1l1	0
Zfp97	0
Bin2	0
BRDN0000738328	0
Gpr180	0
Lim2	0
Sucla2	0
Ltb4r1	0
Fbxw26	0
Gm10324	0
Fam229a	0
Crisp4	0
Crisp1	0
Clstn1	0
Crisp3	0
Glb1	0
Kremen2	0
Kremen1	0
Klhdc1	0
Pacs1	0
Pacs2	0
Tril	0
Prr13	0
Arrb2	0
Fam179b	0
Ovch2	0
Ahi1	0
Adamts20	0
Mea1	0
Ccl27b	0
Ccl27a	0
Zufsp	0
Nip7	0
Raver1	0
Exoc6b	0
BRDN0000738359	0
Hnmt	0
4930578C19Rik	0
Dnah17	0
Dnah10	0
Dnah11	0
Ctc1	0
Sfta2	0
Tcte3	0
Tcte2	0
Tcte1	0
Sybu	0
Agap1	0
Agap2	0
Ubtd1	0
Prlhr	0
Tbc1d8b	0
Zfp92	0
Zwilch	0
Prl2c3	0
Htatip2	0
Vmn1r143	0
Gm6890	0
Ptprcap	0
Ptprc	0
Ptpn5	0
Ptpn4	0
Ptpn23	0
Ptpn22	0
Ptpn20	0
Ptpn2	0
Ptpn18	0
Ptpn14	0
Ptpn13	0
Ptpn12	0
Ptpn11	0
Ptpn1	0
Ptpdc1	0
Ptp4a2	0
Ptov1	0
Ptn	0
Ptms	0
Ptma	0
Ptk7	0
Ptk6	0
Pthlh	0
Pth2	0
Pth1r	0
Pth	0
Ptgs2	0
Ptgs1	0
Ptgr1	0
Ptgis	0
Ptgir	0
Ptges3l	0
Ptges3	0
Ptges2	0
Ptges	0
Ptger4	0
Ptger3	0
Ptger2	0
Ptf1a	0
Pten	0
Ptdss2	0
Ptdss1	0
Ptcra	0
Ptchd4	0
Ptchd3	0
Ptchd2	0
Ptchd1	0
Ptcd3	0
Ptcd2	0
Ptcd1	0
Ptbp3	0
Ptbp1	0
Ptafr	0
Pstpip2	0
Pstpip1	0
Pstk	0
Pspn	0
Psph	0
Pspc1	0
Psors1c2	0
Psmg4	0
Psmg2	0
Psmg1	0
Psme2	0
Psmd9	0
Psmd8	0
Psmd7	0
Psmd6	0
Psmd5	0
Psmd4	0
Psmd3	0
Psmd2	0
Psmd14	0
Psmd13	0
Psmd12	0
Psmd1	0
Psmc4	0
Psmc3ip	0
Psmc3	0
Psmc2	0
Psmc1	0
Psmb9	0

Olfr574	0	Psmc4	0	Astl	0	Psmb7	0
Olfr570	0	Psmc2	0	Sirpa	0	Psmb6	0
Olfr57	0	Psmc1	0	Ppm1f	0	Psmb5	0
Olfr569	0	Psmb7	0	Gm14511	0	Psmb3	0
Olfr566	0	Psmb6	0	Gm11744	0	Psmb10	0
Olfr564	0	Psmb5	0	Mab21l3	0	Psmb1	0
Olfr561	0	Psmb4	0	Brk1	0	Psma8	0
Olfr56	0	Psmb3	0	Olfr1279	0	Psma7	0
Olfr559	0	Psmb2	0	Olfr1278	0	Psma6	0
Olfr557	0	Psmb10	0	Zfp93	0	Psma5	0
Olfr556	0	Psma8	0	Taar6	0	Psma4	0
Olfr553	0	Psma7	0	Ascc3	0	Psma3	0
Olfr552	0	Psma6	0	Dusp4	0	Psma2	0
Olfr551	0	Psma5	0	Ascc1	0	Psma1	0
Olfr550	0	Psma4	0	Dusp6	0	Psip1	0
Olfr547	0	Psma3	0	Olfr1271	0	Psg29	0
Olfr545	0	Psma2	0	Olfr1270	0	Psg28	0
Olfr544	0	Psma1	0	Dusp3	0	Psg26	0
Olfr543	0	Pskh1	0	Dusp2	0	Psg25	0
Olfr541	0	Psip1	0	Paip2b	0	Psg22	0
Olfr539	0	Psg29	0	Spp2	0	Psg16	0
Olfr538	0	Psg28	0	Natd1	0	Psenen	0
Olfr536	0	Psg27	0	Mfge8	0	Psen2	0
Olfr535	0	Psg25	0	Rab42	0	Psen1	0
Olfr533	0	Psg23	0	Ccbe1	0	Psd4	0
Olfr532	0	Psg21	0	Dnah5	0	Psd3	0
Olfr531	0	Psg19	0	Ubl7	0	Psca	0
Olfr530	0	Psg18	0	Vimp	0	Psapl1	0
Olfr53	0	Psg17	0	Ccne2	0	Psap	0
Olfr527	0	Psg16	0	Gli1	0	Prune2	0
Olfr525	0	Psenen	0	Zfp90	0	Prune	0
Olfr524	0	Psen2	0	Gbp11	0	Prtn3	0
Olfr523	0	Psen1	0	Ppm1n	0	Prtg	0
Olfr522	0	Psd4	0	Ms4a4c	0	Prss8	0
Olfr520	0	Psd3	0	Tcl1b1	0	Prss58	0
Olfr519	0	Psd	0	Ppm1m	0	Prss57	0
Olfr517	0	Psca	0	Tcl1b4	0	Prss55	0
Olfr516	0	Psat1	0	Olfr916	0	Prss51	0
Olfr513	0	Psapl1	0	Olfr914	0	Prss50	0
Olfr510	0	Psap	0	Rpl5	0	Prss45	0
Olfr509	0	Prx	0	Olfr912	0	Prss44	0
Olfr507	0	Prune2	0	Olfr913	0	Prss42	0
Olfr506	0	Prune	0	Olfr910	0	Prss41	0
Olfr504	0	Prtn3	0	BRDN0000738029	0	Prss40	0
Olfr503	0	Prtg	0	Olfr918	0	Prss38	0
Olfr5	0	Prss8	0	Olfr919	0	Prss37	0
Olfr497	0	Prss58	0	Fezf1	0	Prss36	0
Olfr495	0	Prss56	0	BRDN0000738322	0	Prss34	0
Olfr493	0	Prss54	0	Agpat5	0	Prss33	0
Olfr491	0	Prss53	0	BRDN0000737754	0	Prss32	0
Olfr49	0	Prss52	0	Ogg1	0	Prss30	0
Olfr488	0	Prss51	0	Spaca1	0	Prss3	0
Olfr487	0	Prss45	0	Dapp1	0	Prss29	0
Olfr486	0	Prss44	0	Igfbp7	0	Prss28	0
Olfr484	0	Prss43	0	Qrfpr	0	Prss27	0
Olfr482	0	Prss42	0	Slc5a5	0	Prss23	0
Olfr481	0	Prss38	0	Stom	0	Prss22	0
Olfr48	0	Prss35	0	Dlgap1	0	Prss21	0
Olfr479	0	Prss34	0	Gpalpp1	0	Prss16	0
Olfr478	0	Prss33	0	BRDN0000738321	0	Prss12	0
Olfr477	0	Prss32	0	Dlgap4	0	Prrx1	0
Olfr476	0	Prss29	0	Msh4	0	Prrt4	0
Olfr474	0	Prss28	0	E230025N22Rik	0	Prrt3	0
Olfr472	0	Prss27	0	Apof	0	Prrt2	0
Olfr47	0	Prss23	0	BRDN0000738207	0	Prrt1	0
Olfr467	0	Prss22	0	Smox	0	Prrg3	0
Olfr466	0	Prss16	0	Itfg1	0	Prrg2	0
Olfr464	0	Prss12	0	Coa5	0	Prrg1	0
Olfr463	0	Prrx1	0	Cdt1	0	Prrc2c	0
Olfr462	0	Prrt2	0	Coa7	0	Prrc2a	0
Olfr461	0	Prrt1	0	Coa6	0	Prr9	0
Olfr460	0	Prrg4	0	Sap30l	0	Prr7	0
Olfr46	0	Prrg1	0	Coa3	0	Prr5l	0
Olfr459	0	Prrc2c	0	Ltc4s	0	Prr5	0
Olfr458	0	Prrc2b	0	Bmp10	0	Prr36	0
Olfr457	0	Prrc2a	0	Pak1ip1	0	Prr32	0
Olfr455	0	Prrc1	0	Bmp15	0	Prr30	0
Olfr453	0	Prr9	0	Snca	0	Prr3	0
Olfr452	0	Prr7	0	Ubr3	0	Prr27	0
Olfr449	0	Prr5	0	Ubr2	0	Prr19	0
Olfr444	0	Prr36	0	Tmprss11b	0	Prr18	0
Olfr441	0	Prr32	0	Tmprss11c	0	Prr15l	0

Olfr435	0	Prr27	0	Matk	0	Prr15	0
Olfr434	0	Prr23a	0	Tmprss11f	0	Prr14l	0
Olfr432	0	Prr22	0	Mid1	0	Prr14	0
Olfr430	0	Prr19	0	Spaca4	0	Prr13	0
Olfr43	0	Prr16	0	Plg	0	Prr12	0
Olfr429	0	Prr15l	0	Spaca6	0	Prr11	0
Olfr424	0	Prr14l	0	Spaca7	0	Prps2	0
Olfr419	0	Prr14	0	Ngb	0	Prps1l3	0
Olfr418	0	Prr13	0	Pdgfrb	0	Prps1l1	0
Olfr414	0	Prr12	0	Spaca3	0	Prps1	0
Olfr412	0	Prr11	0	Rad21	0	Prph	0
Olfr411	0	Prpsap2	0	Psd	0	Prpf8	0
Olfr406	0	Prpsap1	0	Fis1	0	Prpf4b	0
Olfr403	0	Prps2	0	Myeov2	0	Prpf40a	0
Olfr402	0	Prps1l3	0	Fut2	0	Prpf4	0
Olfr399	0	Prps1l1	0	Fut1	0	Prpf39	0
Olfr398	0	Prps1	0	Dhodh	0	Prpf38b	0
Olfr397	0	Prph2	0	Fhl1	0	Prpf38a	0
Olfr395	0	Prph	0	Msh6	0	Prpf31	0
Olfr394	0	Prpf8	0	Eif2s1	0	Prpf3	0
Olfr393	0	Prpf6	0	Fam219b	0	Prpf19	0
Olfr392	0	Prpf4b	0	Fam219a	0	Prpf18	0
Olfr39	0	Prpf40b	0	Eif2s2	0	Prp2	0
Olfr385	0	Prpf40a	0	Fam57b	0	Proz	0
Olfr384	0	Prpf38b	0	Ivd	0	Prox2	0
Olfr382	0	Prpf38a	0	Apom	0	Prox1	0
Olfr381	0	Prpf31	0	Tas1r2	0	Proser3	0
Olfr380	0	Prpf3	0	Hs3st6	0	Pros1	0
Olfr38	0	Prpf19	0	Tnk1	0	Prorsd1	0
Olfr378	0	Prpf18	0	Serpina9	0	Prom2	0
Olfr376	0	Prp2	0	Sned1	0	Prom1	0
Olfr374	0	Prox2	0	Yars	0	Prol1	0
Olfr373	0	Prox1	0	Tinag	0	Prokr2	0
Olfr372	0	Proser3	0	Cant1	0	Prokr1	0
Olfr371	0	Proser2	0	Scnn1g	0	Prok1	0
Olfr370	0	Prosc	0	Ak8	0	Prodh2	0
Olfr368	0	Pros1	0	Sdf2l1	0	Prodh	0
Olfr365	0	Prop1	0	Pllp	0	Procr	0
Olfr362	0	Prom2	0	Tgfbrap1	0	Proca1	0
Olfr361	0	Prol1	0	Foxq1	0	Prob1	0
Olfr358	0	Prokr1	0	Uxt	0	Prnd	0
Olfr356	0	Prok2	0	9130008F23Rik	0	Prn	0
Olfr355	0	Prok1	0	Slc25a2	0	Prmt8	0
Olfr354	0	Prodh2	0	Slc25a1	0	Prmt6	0
Olfr352	0	Prodh	0	Polg2	0	Prmt5	0
Olfr350	0	Procr	0	Sccpdh	0	Prmt3	0
Olfr347	0	Proca1	0	Slc25a5	0	Prmt2	0
Olfr346	0	Prob1	0	Enam	0	Prmt10	0
Olfr345	0	Prnd	0	Cacng8	0	Prmt1	0
Olfr344	0	Prn	0	A430005L14Rik	0	Prlr	0
Olfr340	0	Prmt6	0	Trmt44	0	Prlh	0
Olfr339	0	Prmt5	0	Lamc2	0	Prl8a9	0
Olfr338	0	Prmt3	0	Lamc3	0	Prl8a8	0
Olfr332	0	Prmt10	0	Cacng2	0	Prl8a2	0
Olfr331	0	Prmt1	0	Cacng3	0	Prl7b1	0
Olfr330	0	Prlr	0	Cacng4	0	Prl7a2	0
Olfr33	0	Prlh	0	Cacng5	0	Prl7a1	0
Olfr328	0	Prl8a9	0	Cacng6	0	Prl5a1	0
Olfr325	0	Prl8a8	0	Cacng7	0	Prl4a1	0
Olfr324	0	Prl8a1	0	Colgalt2	0	Prl3d3	0
Olfr323	0	Prl7c1	0	Colgalt1	0	Prl3d1	0
Olfr322	0	Prl7b1	0	Rac2	0	Prl3c1	0
Olfr320	0	Prl7a2	0	Rac3	0	Prl3b1	0
Olfr32	0	Prl7a1	0	Rac1	0	Prl3a1	0
Olfr319	0	Prl6a1	0	Tescl	0	Prl2c5	0
Olfr318	0	Prl5a1	0	Chia1	0	Prl2c2	0
Olfr317	0	Prl4a1	0	BRDN0000738324	0	Prl2c1	0
Olfr316	0	Prl3d2	0	Cd93	0	Prl2b1	0
Olfr315	0	Prl3d1	0	Angptl7	0	Prkx	0
Olfr314	0	Prl3c1	0	Angptl6	0	Prkrir	0
Olfr313	0	Prl3b1	0	Angptl4	0	Prkra	0
Olfr312	0	Prl3a1	0	Angptl3	0	Prkg2	0
Olfr311	0	Prl2c5	0	Angptl2	0	Prkg1	0
Olfr310	0	Prl2c2	0	Fam71f2	0	Prkdc	0
Olfr31	0	Prl2c1	0	Vmn1r116	0	Prkd3	0
Olfr309	0	Prl2b1	0	1110004F10Rik	0	Prkd2	0
Olfr308	0	Prl	0	Clcc1	0	Prkd1	0
Olfr307	0	Prkx	0	Tdh	0	Prkcq	0
Olfr305	0	Prkrir	0	BRDN0000737968	0	Prkci	0
Olfr303	0	Prkrip1	0	Nms	0	Prkch	0
Olfr30	0	Prkra	0	Zfp558	0	Prkcg	0
Olfr3	0	Prkg2	0	BRDN0000737965	0	Prkce	0

Olfr299	0	Prkg1	0	BRDN0000737966	0	Prkcd	0
Olfr298	0	Prkdc	0	BRDN0000737967	0	Prkca	0
Olfr297	0	Prkd3	0	Tdg	0	Prkar2a	0
Olfr295	0	Prkcsh	0	BRDN0000737961	0	Prkar1b	0
Olfr292	0	Prkcg	0	BRDN0000737962	0	Prkar1a	0
Olfr291	0	Prkce	0	1600012H06Rik	0	Prkag1	0
Olfr290	0	Prkcdbp	0	Gm765	0	Prkacb	0
Olfr288	0	Prkcd	0	Gm766	0	Prkaca	0
Olfr287	0	Prkcb	0	Eid2	0	Prkab2	0
Olfr284	0	Prkca	0	BC053393	0	Prkab1	0
Olfr283	0	Prkar2a	0	Mzt2	0	Prkaa2	0
Olfr282	0	Prkar1b	0	Pycr1	0	Prkaa1	0
Olfr279	0	Prkar1a	0	1700080O16Rik	0	Primpol	0
Olfr275	0	Prkag3	0	Nmi	0	Prim2	0
Olfr273	0	Prkag2	0	Ly6f	0	Prim1	0
Olfr272	0	Prkag1	0	Ly6e	0	Prh1	0
Olfr270	0	Prkaca	0	Ly6d	0	Prg3	0
Olfr267	0	Prkab2	0	Srf	0	Prg2	0
Olfr266	0	Prkab1	0	Ly6a	0	Prex2	0
Olfr262	0	Prkaa1	0	Nfe2l2	0	Prex1	0
Olfr26	0	Primpol	0	Nfe2l1	0	Prepl	0
Olfr259	0	Prim2	0	Gk2	0	Prep	0
Olfr250	0	Prim1	0	Ly6k	0	Prelp	0
Olfr25	0	Prickle2	0	Lactb2	0	Prelid2	0
Olfr248	0	Prickle1	0	Ly6i	0	Prelid1	0
Olfr239	0	Prh1	0	Srm	0	Preb	0
Olfr237-ps1	0	Prg3	0	Srr	0	Prdx6b	0
Olfr231	0	Prg2	0	Gm13306	0	Prdx6	0
Olfr23	0	Prf1	0	Gnpat	0	Prdx5	0
Olfr228	0	Prex2	0	6820408C15Rik	0	Prdx4	0
Olfr225	0	Prepl	0	Opn5	0	Prdx3	0
Olfr223	0	Prep	0	Opn3	0	Prdx2	0
Olfr222	0	Prelp	0	BRDN0000737664	0	Prdx1	0
Olfr218	0	Prelid1	0	Rasgrp2	0	Prdm9	0
Olfr215	0	Preb	0	Iqcf4	0	Prdm8	0
Olfr214	0	Prdx6	0	Iqcf5	0	Prdm4	0
Olfr213	0	Prdx4	0	Adcyap1	0	Prdm2	0
Olfr211	0	Prdx1	0	Rasgrp4	0	Prdm15	0
Olfr209	0	Prdm9	0	Pycrl	0	Prdm14	0
Olfr206	0	Prdm6	0	Spire1	0	Prdm13	0
Olfr205	0	Prdm4	0	Spire2	0	Prdm12	0
Olfr203	0	Prdm2	0	Pcgf2	0	Prdm10	0
Olfr202	0	Prdm15	0	Cryl1	0	Prcp	0
Olfr201	0	Prdm13	0	Gch1	0	Prcc	0
Olfr20	0	Prdm12	0	Kcng1	0	Prc1	0
Olfr2	0	Prdm11	0	Gpr137c	0	Prap1	0
Olfr199	0	Prdm1	0	Gpr137b	0	Pramel7	0
Olfr198	0	Prcp	0	Miox	0	Pramel6	0
Olfr196	0	Prc1	0	Kcng3	0	Pramel3	0
Olfr195	0	Prb1	0	BRDN0000737778	0	Pramel1	0
Olfr194	0	Prap1	0	Kcng4	0	Pramef6	0
Olfr191	0	Pramel7	0	AF529169	0	Pramef25	0
Olfr190	0	Pramel6	0	Emc10	0	Pramef17	0
Olfr19	0	Pramel5	0	Lor	0	Prame	0
Olfr187	0	Pramel3	0	Zfp160	0	Pram1	0
Olfr186	0	Pramel1	0	Kif13b	0	Pradc1	0
Olfr183	0	Pramef8	0	Naa60	0	Pqlc2	0
Olfr181	0	Pramef6	0	Sdha	0	Ppy	0
Olfr180	0	Pramef25	0	Sdhc	0	Ppwd1	0
Olfr178	0	Pramef12	0	Sdhb	0	Ppt1	0
Olfr173	0	Prame	0	Sdhd	0	Ppp6r3	0
Olfr170	0	Pram1	0	Tctex1d1	0	Ppp6r2	0
Olfr17	0	Praf2	0	Gata6	0	Ppp6c	0
Olfr168	0	Pqlc3	0	Epm2a	0	Ppp5c	0
Olfr167	0	Pqlc2	0	Gal3st2	0	Ppp4r4	0
Olfr166	0	Ppy	0	BRDN0000737533	0	Ppp4r2	0
Olfr165	0	Pptc7	0	Pcdhga7	0	Ppp4c	0
Olfr164	0	Ppt1	0	Fbxl6	0	Ppp3r2	0
Olfr161	0	Ppp6r2	0	Zfp36	0	Ppp3r1	0
Olfr160	0	Ppp6r1	0	Ropn1l	0	Ppp3cb	0
Olfr16	0	Ppp5c	0	Gm8300	0	Ppp3ca	0
Olfr159	0	Ppp4r2	0	Olfr368	0	Ppp2r5e	0
Olfr157	0	Ppp4c	0	BRDN0000737636	0	Ppp2r5d	0
Olfr156	0	Ppp3r2	0	BRDN0000738100	0	Ppp2r5c	0
Olfr155	0	Ppp3r1	0	BRDN0000738107	0	Ppp2r5b	0
Olfr154	0	Ppp3cb	0	BRDN0000738106	0	Ppp2r5a	0
Olfr1535	0	Ppp3ca	0	BRDN0000737632	0	Ppp2r4	0
Olfr1532-ps1	0	Ppp2r5e	0	Tle2	0	Ppp2r3c	0
Olfr153	0	Ppp2r5d	0	Rps4x	0	Ppp2r3a	0
Olfr152	0	Ppp2r5b	0	Olfr360	0	Ppp2r2d	0
Olfr1511	0	Ppp2r5a	0	Dusp9	0	Ppp2r2b	0
Olfr1510	0	Ppp2r4	0	BRDN0000738108	0	Ppp2r2a	0

Olfr1509	0	Ppp2r3c	0	BRDN0000737638	0	Ppp2r1a	0
Olfr1505	0	Ppp2r2c	0	BRDN0000737639	0	Ppp2ca	0
Olfr1504	0	Ppp2r2b	0	Qprt	0	Ppp1r9b	0
Olfr1502	0	Ppp2r1b	0	Olfr366	0	Ppp1r9a	0
Olfr1501	0	Ppp2r1a	0	Sypl2	0	Ppp1r8	0
Olfr1500	0	Ppp2cb	0	Sfrp4	0	Ppp1r7	0
Olfr150	0	Ppp2ca	0	Etaa1	0	Ppp1r3g	0
Olfr1499	0	Ppp1r9a	0	BRDN0000738344	0	Ppp1r3f	0
Olfr1497	0	Ppp1r8	0	Oacyl	0	Ppp1r3e	0
Olfr1496	0	Ppp1r7	0	Nrxn3	0	Ppp1r3d	0
Olfr1494	0	Ppp1r3f	0	Nrxn2	0	Ppp1r3c	0
Olfr1491	0	Ppp1r3d	0	Nrxn1	0	Ppp1r3b	0
Olfr1490	0	Ppp1r3b	0	Anks1	0	Ppp1r36	0
Olfr149	0	Ppp1r36	0	Anks3	0	Ppp1r27	0
Olfr1489	0	Ppp1r35	0	Anks6	0	Ppp1r26	0
Olfr1487	0	Ppp1r32	0	Stard3	0	Ppp1r2	0
Olfr1484	0	Ppp1r27	0	Dusp8	0	Ppp1r1c	0
Olfr1480	0	Ppp1r26	0	Ndufaf4	0	Ppp1r1b	0
Olfr1477	0	Ppp1r21	0	Tle4	0	Ppp1r18	0
Olfr1475	0	Ppp1r2	0	Tmem86a	0	Ppp1r17	0
Olfr1474	0	Ppp1r1c	0	Tmem86b	0	Ppp1r16b	0
Olfr1471	0	Ppp1r1a	0	Olfr145	0	Ppp1r16a	0
Olfr147	0	Ppp1r17	0	Rusc2	0	Ppp1r15b	0
Olfr1467	0	Ppp1r16b	0	Olfr147	0	Ppp1r15a	0
Olfr1465	0	Ppp1r16a	0	Olfr146	0	Ppp1r14d	0
Olfr1463	0	Ppp1r15b	0	Olfr141	0	Ppp1r14c	0
Olfr1462	0	Ppp1r14c	0	Olfr140	0	Ppp1r13l	0
Olfr1461	0	Ppp1r13l	0	Olfr143	0	Ppp1r13b	0
Olfr146	0	Ppp1r13b	0	Olfr142	0	Ppp1r12b	0
Olfr1459	0	Ppp1r12b	0	Grk6	0	Ppp1r10	0
Olfr1457	0	Ppp1r12a	0	BRDN0000738240	0	Ppp1cb	0
Olfr1454	0	Ppp1r11	0	Grk4	0	Ppox	0
Olfr1453	0	Ppp1cc	0	Fuom	0	Ppme1	0
Olfr1451	0	Ppp1cb	0	Olfr149	0	Ppm1n	0
Olfr1450	0	Ppox	0	Olfr148	0	Ppm1m	0
Olfr1449	0	Ppme1	0	Zfp39	0	Ppm1l	0
Olfr1447	0	Ppm1n	0	BRDN0000737398	0	Ppm1k	0
Olfr1446	0	Ppm1m	0	Atp9b	0	Ppm1h	0
Olfr1445	0	Ppm1l	0	Atp9a	0	Ppm1g	0
Olfr1440	0	Ppm1k	0	Gorasp2	0	Ppm1f	0
Olfr1437	0	Ppm1j	0	Dctn2	0	Ppm1e	0
Olfr1436	0	Ppm1h	0	Dctn3	0	Ppm1b	0
Olfr1434	0	Ppm1g	0	Liph	0	Ppm1a	0
Olfr1431	0	Ppm1e	0	Dctn1	0	Ppil6	0
Olfr143	0	Ppm1d	0	Dctn6	0	Ppil3	0
Olfr1428	0	Ppm1b	0	Tada2b	0	Ppil2	0
Olfr1426	0	Ppm1a	0	Dctn4	0	Ppil1	0
Olfr1425	0	Ppl	0	Dctn5	0	Ppif	0
Olfr1423	0	Ppip5k1	0	Znrf4	0	Ppie	0
Olfr1420	0	Ppil6	0	Cpne9	0	Ppid	0
Olfr142	0	Ppil4	0	Znrf1	0	Ppic	0
Olfr1419	0	Ppil3	0	Znrf3	0	Ppib	0
Olfr1418	0	Ppil2	0	Znrf2	0	Pphln1	0
Olfr1417	0	Ppil1	0	Rgs22	0	Ppfibp1	0
Olfr1416	0	Ppih	0	Pbsn	0	Ppfia4	0
Olfr1415	0	Ppie	0	Slc38a11	0	Ppfia3	0
Olfr1414	0	Ppid	0	Slc38a10	0	Ppfia2	0
Olfr1413	0	Ppic	0	Apol8	0	Ppef1	0
Olfr1412	0	Ppib	0	Apol6	0	Ppdpf	0
Olfr1410	0	Ppia	0	Abcg5	0	Ppcs	0
Olfr141	0	Ppfibp2	0	A630033H20Rik	0	Ppcdc	0
Olfr1408	0	Ppfibp1	0	BRDN0000737759	0	Ppbp	0
Olfr1404	0	Ppfia4	0	Cpne8	0	Ppat	0
Olfr1402	0	Ppfia3	0	Ube2g1	0	Pparg	0
Olfr140	0	Ppef1	0	BRDN0000737390	0	Ppara	0
Olfr1396	0	Ppcs	0	BRDN0000738242	0	Ppapdc3	0
Olfr1395	0	Ppcdc	0	Map7d2	0	Ppapdc2	0
Olfr1393	0	Ppbp	0	Galnt1	0	Ppapdc1b	0
Olfr1392	0	Ppargc1a	0	BRDN0000737397	0	Ppapdc1a	0
Olfr1391	0	Ppard	0	Gm5891	0	Ppap2c	0
Olfr1390	0	Ppapdc3	0	Glrb	0	Ppan	0
Olfr1389	0	Ppapdc1b	0	5730508B09Rik	0	Ppa2	0
Olfr1388	0	Ppap2c	0	Dmtf1	0	Ppa1	0
Olfr1387	0	Ppap2b	0	C8a	0	Pp2d1	0
Olfr1385	0	Ppap2a	0	Slco4c1	0	Pou6f1	0
Olfr1384	0	Ppan	0	BC028528	0	Pou5f2	0
Olfr1383	0	Ppa2	0	Pddc1	0	Pou4f3	0
Olfr1382	0	Pp2d1	0	Glrx	0	Pou4f1	0
Olfr1381	0	Pou6f1	0	Atxn10	0	Pou3f4	0
Olfr1380	0	Pou5f2	0	S100g	0	Pou2f3	0
Olfr138	0	Pou5f1	0	S100b	0	Pou1f1	0
Olfr1378	0	Pou4f1	0	Rnmtl1	0	Poteg	0

Olfr1377	0	Pou3f3	0	Duoxa2	0	Pot1a	0
Olfr1373	0	Pou3f2	0	Plekhf1	0	Postn	0
Olfr1371	0	Pou3f1	0	Zfp839	0	Por	0
Olfr1370	0	Pou2f3	0	Plekhf2	0	Popdc3	0
Olfr137	0	Pou2f1	0	Trim10	0	Pop7	0
Olfr1368	0	Pou2af1	0	Zfp831	0	Pop5	0
Olfr1367	0	Poteg	0	Zfp830	0	Pop4	0
Olfr1366	0	Pot1b	0	Fermt2	0	Pop1	0
Olfr1364	0	Pot1a	0	Fermt3	0	Pon3	0
Olfr1362	0	Postn	0	Trmt2b	0	Pon2	0
Olfr1361	0	Popdc3	0	Fermt1	0	Pon1	0
Olfr1360	0	Pop7	0	Gm19345	0	Pomt1	0
Olfr136	0	Pop5	0	Sectm1b	0	Pomk	0
Olfr1359	0	Pop4	0	Aunip	0	Pomgnt2	0
Olfr1357	0	Pop1	0	Ermard	0	Pomc	0
Olfr1355	0	Pon2	0	BRDN0000738256	0	Pom121l2	0
Olfr1354	0	Pomt2	0	Gm20877	0	Polrmt	0
Olfr1352	0	Pomp	0	Atxn1l	0	Polr3k	0
Olfr1351	0	Pomgnt2	0	Cd1d1	0	Polr3h	0
Olfr1350	0	Pomgnt1	0	Stk17b	0	Polr3g	0
Olfr1349	0	Pomc	0	Sectm1a	0	Polr3e	0
Olfr1348	0	Pom121l2	0	Sys1	0	Polr3d	0
Olfr1347	0	Pom121	0	Dusp7	0	Polr3c	0
Olfr1346	0	Polrmt	0	Taar8c	0	Polr3b	0
Olfr1344	0	Polr3h	0	Ets2	0	Polr3a	0
Olfr1342	0	Polr3gl	0	Dyrk2	0	Polr2m	0
Olfr1341	0	Polr3g	0	Ets1	0	Polr2l	0
Olfr134	0	Polr3f	0	Taar8a	0	Polr2k	0
Olfr1338	0	Polr3d	0	BRDN0000738342	0	Polr2j	0
Olfr1337	0	Polr3b	0	Vmn1r29	0	Polr2i	0
Olfr1336	0	Polr3a	0	Tor3a	0	Polr2h	0
Olfr1335	0	Polr2m	0	Sall2	0	Polr2g	0
Olfr1333	0	Polr2l	0	Sec22c	0	Polr2f	0
Olfr1331	0	Polr2k	0	Sec22a	0	Polr2e	0
Olfr1330	0	Polr2j	0	Ptpn18	0	Polr2c	0
Olfr133	0	Polr2i	0	Shf	0	Polr2b	0
Olfr1329	0	Polr2h	0	Ptpn13	0	Polr1e	0
Olfr1325	0	Polr2f	0	Ptpn12	0	Polr1d	0
Olfr1324	0	Polr2e	0	Ptpn14	0	Polr1c	0
Olfr1323	0	Polr2d	0	Gm11487	0	Polr1b	0
Olfr1322	0	Polr2c	0	Ube2d2a	0	Polr1a	0
Olfr1321	0	Polr2a	0	Hist1h2bp	0	Polq	0
Olfr1320	0	Polr1e	0	Cdip1	0	Poln	0
Olfr1318	0	Polr1d	0	Ube2d2b	0	Poll	0
Olfr1316	0	Polr1c	0	Zfand2b	0	Polh	0
Olfr1314	0	Polr1b	0	Swap70	0	Polg2	0
Olfr1313	0	Polr1a	0	Cfap221	0	Polg	0
Olfr1312	0	Polq	0	Zfand2a	0	Pole4	0
Olfr1311	0	Poln	0	Tasp1	0	Pole3	0
Olfr1310	0	Poll	0	Piga	0	Pole2	0
Olfr131	0	Polk	0	Pigb	0	Pole	0
Olfr1309	0	Poli	0	Pigc	0	Poldip2	0
Olfr1308	0	Polh	0	Itgb5	0	Pold4	0
Olfr1306	0	Polg2	0	Pigf	0	Pold3	0
Olfr1305	0	Pole4	0	Pigg	0	Pold2	0
Olfr1303	0	Pole2	0	Tsr1	0	Pold1	0
Olfr1302	0	Pole	0	Itgb8	0	Polb	0
Olfr1301	0	Poldip3	0	Tsr3	0	Pola1	0
Olfr130	0	Pold4	0	Tsr2	0	Pogz	0
Olfr13	0	Pold3	0	Pigl	0	Poglut1	0
Olfr1299	0	Pold2	0	Pigm	0	Pofut2	0
Olfr1298	0	Pold1	0	Pign	0	Pofut1	0
Olfr1295	0	Polb	0	Mpi	0	Podxl	0
Olfr1294	0	Pola2	0	Pigp	0	Podnl1	0
Olfr1289	0	Pola1	0	Pigq	0	Poc5	0
Olfr1287	0	Pogz	0	Pigr	0	Poc1a	0
Olfr1286	0	Poglut1	0	Pigs	0	Pnrc2	0
Olfr1284	0	Pofut2	0	Caprin2	0	Pnrc1	0
Olfr1282	0	Pofut1	0	Pigu	0	Pnpt1	0
Olfr1281	0	Podxl2	0	Pigv	0	Pnpo	0
Olfr1280	0	Podnl1	0	Mt3	0	Pnpla8	0
Olfr128	0	Podn	0	Optn	0	Pnpla7	0
Olfr1278	0	Poc5	0	Pnma2	0	Pnpla6	0
Olfr1277	0	Poc1b	0	Mpz	0	Pnpla5	0
Olfr1275	0	Pnrc1	0	Defb42	0	Pnpla3	0
Olfr1273-ps	0	Pnpt1	0	Mt1	0	Pnpla2	0
Olfr1270	0	Pnpo	0	Zfp763	0	Pno1	0
Olfr127	0	Pnpla8	0	Gnl3l	0	Pnn	0
Olfr1269	0	Pnpla3	0	Zfp760	0	Pnmt	0
Olfr1265	0	Pnpla1	0	Wac	0	Pnmal2	0
Olfr1264	0	Pnp2	0	BRDN0000738352	0	Pnmal1	0
Olfr1263	0	Pnp	0	BRDN0000737718	0	Pnma5	0

Olfr1262	0	Pno1	0	Dock8	0	Pnma3	0
Olfr1261	0	Pnn	0	Zfp768	0	Pnma2	0
Olfr1260	0	Pnmal1	0	Tmx4	0	Pnma1	0
Olfr126	0	Pnma5	0	Tmx1	0	Pnliprp2	0
Olfr1258	0	Pnma2	0	Tmx3	0	Pnliprp1	0
Olfr1256	0	Pnliprp2	0	BRDN0000738003	0	Pnlip	0
Olfr1255	0	Pnliprp1	0	Cyp4a12b	0	Pnkp	0
Olfr1254	0	Pnldc1	0	Myo15	0	Pms1	0
Olfr1253	0	Pnkp	0	Cdipt	0	Pmpcb	0
Olfr1252	0	Pnkd	0	Ddias	0	Pmpca	0
Olfr1251	0	Pnck	0	Arl13a	0	Pmp2	0
Olfr1250	0	Pmvk	0	BRDN0000738001	0	Pmm2	0
Olfr125	0	Pmpcb	0	Nags	0	Pmm1	0
Olfr1249	0	Pmpca	0	Pinlyp	0	Pmfbp1	0
Olfr1248	0	Pmp22	0	Btf3	0	Pmepa1	0
Olfr1247	0	Pmp2	0	Ppp2ca	0	Pmel	0
Olfr1246	0	Pmm2	0	C87977	0	Pmch	0
Olfr1245	0	Pml	0	Lrrc39	0	Pm20d2	0
Olfr1243	0	Pmepa1	0	Ppp2cb	0	Plxnd1	0
Olfr1242	0	Pmch	0	Kitl	0	Plxnb3	0
Olfr1241	0	Pmaip1	0	Gm4858	0	Plxnb2	0
Olfr1240	0	Pm20d2	0	BRDN0000737712	0	Plxnb1	0
Olfr124	0	Plxnd1	0	Rnf157	0	Plxna4	0
Olfr1239	0	Plxnb3	0	Uchl5	0	Plxna2	0
Olfr1238	0	Plxnb2	0	BRDN0000738004	0	Plxna1	0
Olfr1234	0	Plxnb1	0	Naga	0	Plscr5	0
Olfr1233	0	Plxna4	0	Defb36	0	Plscr3	0
Olfr1232	0	Plxna3	0	BRDN0000737710	0	Pls3	0
Olfr1231	0	Plxdc2	0	Nagk	0	Plrg1	0
Olfr1230	0	Plscr5	0	Hepacam	0	Plp2	0
Olfr123	0	Plscr2	0	Krt75	0	Plod3	0
Olfr1229	0	Pls3	0	Krt76	0	Plod2	0
Olfr1226	0	Plrg1	0	Llgl2	0	Pln	0
Olfr1225	0	Plp2	0	Mboat7	0	Pllp	0
Olfr1222	0	Plp1	0	Krt73	0	Plk3	0
Olfr1221	0	Plod2	0	Krt72	0	Plk2	0
Olfr1220	0	Plod1	0	Huwe1	0	Plk1	0
Olfr122	0	Plk5	0	Pcdhga10	0	Plin5	0
Olfr1219	0	Plk2	0	Krt78	0	Plin4	0
Olfr1218	0	Plk1	0	Pcdhga12	0	Plin3	0
Olfr1217	0	Plin5	0	Spag6l	0	Plin1	0
Olfr1216	0	Plin3	0	Hist1h2bl	0	Plgrkt	0
Olfr1215	0	Plin2	0	Nat8	0	Plg	0
Olfr1214	0	Plin1	0	Treml2	0	Plekhs1	0
Olfr1213	0	Plgrkt	0	Ugt2b37	0	Plekho2	0
Olfr1212	0	Plg	0	Neurog3	0	Plekho1	0
Olfr121	0	Plet1	0	Neurog2	0	Plekhm2	0
Olfr1208	0	Plekhs1	0	Neurog1	0	Plekhm1	0
Olfr1206	0	Plekho1	0	D1Pas1	0	Plekhh3	0
Olfr1205	0	Plekhn1	0	Cdc42ep4	0	Plekhh1	0
Olfr1204	0	Plekhm3	0	Hoxd12	0	Plekhg6	0
Olfr1202	0	Plekhm2	0	Olfr924	0	Plekhg5	0
Olfr1201	0	Plekhm1	0	Rccd1	0	Plekhg4	0
Olfr1200	0	Plekhj1	0	Nup133	0	Plekhg1	0
Olfr120	0	Plekhh2	0	Nr0b1	0	Plekhf2	0
Olfr1198	0	Plekhg6	0	Ptar1	0	Plekhb2	0
Olfr1196	0	Plekhg4	0	Xylt2	0	Plekha8	0
Olfr1195	0	Plekhg2	0	Cct8l1	0	Plekha4	0
Olfr1193	0	Plekhd1	0	Xylt1	0	Plekha3	0
Olfr119	0	Plekhb2	0	Pglyrp2	0	Plekha2	0
Olfr1189	0	Plekhb1	0	Gpr31b	0	Plek2	0
Olfr1188	0	Plekha4	0	Cbll1	0	Plek	0
Olfr1186	0	Plekha3	0	Pglyrp4	0	Pld6	0
Olfr1184	0	Plekha2	0	Kdelr2	0	Pld5	0
Olfr1183	0	Plek2	0	Kdelr3	0	Pld4	0
Olfr1181	0	Plek	0	Kdelr1	0	Pld1	0
Olfr118	0	Plec	0	BRDN0000738225	0	Plcxd3	0
Olfr1178	0	Pld5	0	Nat1	0	Plcxd1	0
Olfr1176	0	Pld4	0	Itga2	0	Plcl2	0
Olfr1173	0	Pld3	0	Rab11fip3	0	Plch2	0
Olfr1170	0	Pld2	0	Gm5538	0	Plch1	0
Olfr117	0	Plcxd2	0	Rab11fip1	0	Plce1	0
Olfr1168	0	Plcxd1	0	Dnmt1	0	Plcd1	0
Olfr1166	0	Plcd1	0	Lrig3	0	Plcb2	0
Olfr1164	0	Plcb4	0	Arhgap40	0	Plbd1	0
Olfr1160	0	Plcb3	0	Lrig1	0	Plb1	0
Olfr116	0	Plcb1	0	Rab11fip4	0	Plaur	0
Olfr1155	0	Plbd1	0	Morc2a	0	Plagl2	0
Olfr1154	0	Plau	0	Nat6	0	Plagl1	0
Olfr1152	0	Plagl2	0	Ppp2r4	0	Plac9b	0
Olfr1151	0	Plag1	0	Thyn1	0	Plac9a	0
Olfr115	0	Plac9b	0	Krtap4-9	0	Plac8l1	0

Olfr1148	0	Plac9a	0	Krtap4-8	0	Plac1	0
Olfr1145	0	Plac8l1	0	Slitrk4	0	Plaa	0
Olfr1141	0	Plac8	0	Slitrk5	0	Pla2r1	0
Olfr1140	0	Plac1	0	Slitrk2	0	Pla2g7	0
Olfr1138	0	Pla2g7	0	Arhgef33	0	Pla2g6	0
Olfr1137	0	Pla2g6	0	Slitrk1	0	Pla2g4f	0
Olfr1136	0	Pla2g4f	0	Krtap4-1	0	Pla2g4d	0
Olfr1135	0	Pla2g4e	0	Fam96a	0	Pla2g4c	0
Olfr1134	0	Pla2g4c	0	Ager	0	Pla2g4b	0
Olfr1133	0	Pla2g3	0	Fam96b	0	Pla2g2f	0
Olfr1132	0	Pla2g2f	0	Lect1	0	Pla2g2e	0
Olfr1131	0	Pla2g2e	0	Ccpg1	0	Pla2g16	0
Olfr1130	0	Pla2g2d	0	Pvrl1	0	Pla2g15	0
Olfr1126	0	Pla2g1b	0	Acot6	0	Pla2g10	0
Olfr1123	0	Pla2g15	0	Ppp3cb	0	Pla1a	0
Olfr1122	0	Pla2g12a	0	Fcgr4	0	Pkp3	0
Olfr1121	0	Pkp4	0	Fcgr3	0	Pkp2	0
Olfr1120	0	Pkp3	0	Hspa9	0	Pkp1	0
Olfr112	0	Pkp2	0	Fcgr1	0	Pknox1	0
Olfr1116	0	Pkp1	0	Hspa8	0	Pkn3	0
Olfr1115	0	Pknox2	0	Ckmt1	0	Pkn2	0
Olfr1113	0	Pknox1	0	Cfap58	0	Pkn1	0
Olfr1111	0	Pkn3	0	Krt7	0	Pkmyt1	0
Olfr111	0	Pkn1	0	Pxn	0	Pkm	0
Olfr1109	0	Pkmyt1	0	Cfap57	0	Pklr	0
Olfr1107	0	Pkm	0	4930430A15Rik	0	Pkig	0
Olfr1106	0	Pkig	0	Asb12	0	Pkib	0
Olfr1105	0	Pkib	0	Cfap53	0	Pkia	0
Olfr1104	0	Pkia	0	Ncln	0	Pkhd1	0
Olfr1102	0	Pkdrej	0	Mettl3	0	Pkdcc	0
Olfr1101	0	Pkdcc	0	St8sia2	0	Pkd2l2	0
Olfr1100	0	Pkd2l2	0	Mrgprx1	0	Pkd2l1	0
Olfr1099	0	Pkd2l1	0	Plau	0	Pkd1l3	0
Olfr1098	0	Pkd2	0	Mrgprx2	0	Pkd1	0
Olfr1095	0	Pkd1l3	0	Ptbp3	0	Pja2	0
Olfr1094	0	Pkd1	0	Ush1c	0	Pja1	0
Olfr1093	0	Pja1	0	Ptbp1	0	Piwil2	0
Olfr1090	0	Piwil1	0	Ush1g	0	Piwil1	0
Olfr1089	0	Pitx2	0	Chchd3	0	Pitx1	0
Olfr1087	0	Pitx1	0	Chchd1	0	Pitrm1	0
Olfr1086	0	Pitrm1	0	Chchd6	0	Pitpnm3	0
Olfr1082	0	Pitpnm3	0	Chchd7	0	Pitpnm2	0
Olfr1080	0	Pitpnb	0	Chchd4	0	Pitpnm1	0
Olfr1079	0	Pitpna	0	Chchd5	0	Pitpnb	0
Olfr1076	0	Pithd1	0	Epha7	0	Pitpna	0
Olfr107	0	Pisd	0	Lmo1	0	Pir	0
Olfr1065	0	Pirt	0	Lekr1	0	Pipox	0
Olfr1061	0	Pira1	0	Lmo3	0	Pip5kl1	0
Olfr1058	0	Pir	0	Lmo4	0	Pip5k1c	0
Olfr1057	0	Pip5k1c	0	Epha2	0	Pip4k2c	0
Olfr1056	0	Pip5k1a	0	Epha1	0	Pinlyp	0
Olfr1054	0	Pip4k2b	0	Lmo7	0	Pink1	0
Olfr1053	0	Pip4k2a	0	Rgl1	0	Pin4	0
Olfr1052	0	Pip	0	Bpifb9b	0	Pin1	0
Olfr1051	0	Pinx1	0	Bpifb9a	0	Pim3	0
Olfr1049	0	Pink1	0	Fam83h	0	Pim2	0
Olfr1048	0	Pin4	0	Gna12	0	Pim1	0
Olfr1047	0	Pin1rt1	0	Acot4	0	Pilrb2	0
Olfr1046	0	Pin1	0	Cby1	0	Pilrb1	0
Olfr1045	0	Pim3	0	Tmem45a	0	Pik3r3	0
Olfr1044	0	Pim2	0	Tmem45b	0	Pik3r2	0
Olfr1043	0	Pilra	0	Pbxip1	0	Pik3r1	0
Olfr1040	0	Pik3r6	0	Hykk	0	Pik3ip1	0
Olfr1039	0	Pik3r4	0	Gclm	0	Pik3cg	0
Olfr1037	0	Pik3r3	0	Ak3	0	Pik3cd	0
Olfr1036	0	Pik3r1	0	Nrbp1	0	Pik3cb	0
Olfr1034	0	Pik3cg	0	Rp2h	0	Pik3ca	0
Olfr1032	0	Pik3cd	0	Dnajb14	0	Pik3c3	0
Olfr1031	0	Pik3cb	0	Aldh2	0	Pik3c2g	0
Olfr1030	0	Pik3ca	0	Gdi1	0	Pik3c2b	0
Olfr103	0	Pik3c3	0	Fam83c	0	Pik3c2a	0
Olfr1029	0	Pik3c2g	0	Grap2	0	Pih1d3	0
Olfr1028	0	Pik3c2b	0	Fam150a	0	Pih1d2	0
Olfr1024	0	Pik3c2a	0	Gm13275	0	Pigz	0
Olfr1023	0	Pih1d3	0	Fam150b	0	Pigt	0
Olfr1022	0	Pih1d2	0	Batf2	0	Pigs	0
Olfr102	0	Pigz	0	Batf3	0	Pigq	0
Olfr1019	0	Pigyl	0	Fam173a	0	Pigp	0
Olfr1018	0	Pigu	0	Fam83a	0	Pign	0
Olfr1016	0	Pigt	0	Zfp119b	0	Pigm	0
Olfr1015	0	Pigs	0	Parva	0	Pigk	0
Olfr1013	0	Pigr	0	Trim28	0	Pigh	0

Olfr1012	0	Pigq	0	1700020N15Rik	0	Pigc	0
Olfr101	0	Pigm	0	Ankrd63	0	Pigb	0
Olfr1009	0	Pigg	0	Trim24	0	Piga	0
Olfr1008	0	Piga	0	St8sia6	0	Pifo	0
Olfr1006	0	Pifo	0	Trim26	0	Pidd1	0
Olfr1002	0	Piezo2	0	St8sia4	0	Pid1	0
Olfr1000	0	Piezo1	0	Tanc1	0	Pick1	0
Olfml3	0	Pidd1	0	Trim21	0	Picalm	0
Olfml2b	0	Pid1	0	Mfsd2b	0	Pias4	0
Olfml2a	0	Pick1	0	Ctdsp1	0	Pias3	0
Olfml1	0	Picalm	0	Fabp6	0	Pias1	0
Olfm4	0	Pibf1	0	Fabp7	0	Pianp	0
Olfm1	0	Pias3	0	Itpkc	0	Pi4kb	0
Olah	0	Pias2	0	Fabp5	0	Pi4ka	0
Ola1	0	Pias1	0	Olfr132	0	Pi4k2a	0
Oit3	0	Pianp	0	Fabp3	0	Pi16	0
Oit1	0	Pi4kb	0	Foxl1	0	Pi15	0
Oip5	0	Pi4ka	0	Sart3	0	Phyhip	0
Ogt	0	Pi4k2b	0	Sart1	0	Phyhd1	0
Ogn	0	Pi4k2a	0	Hip1r	0	Phyh	0
Ogg1	0	Pi16	0	Dazap1	0	Phtf2	0
Ogfr	0	Pi15	0	Cdc42bpa	0	Phrf1	0
Ogfod3	0	Phykpl	0	Ttc5	0	Phpt1	0
Ogfod2	0	Phyhipl	0	Ttc4	0	Phospho2	0
Ogdhl	0	Phtf2	0	Ttc7	0	Phospho1	0
Ogdh	0	Phtf1	0	LOC100502896	0	Phlpp2	0
Ofcc1	0	Phpt1	0	Ttc1	0	Phlpp1	0
Odf3l2	0	Phox2b	0	Rpl7l1	0	Phldb1	0
Odf3l1	0	Phox2a	0	Olfr558	0	Phlda3	0
Odf3b	0	Phospho2	0	Emd	0	Phlda2	0
Odf3	0	Phospho1	0	Olfr556	0	Phlda1	0
Odf2l	0	Phlpp2	0	Olfr557	0	Phkg1	0
Odf2	0	Phlpp1	0	BRDN0000737401	0	Phkb	0
Odf1	0	Phldb3	0	Olfr555	0	Phka1	0
Odc1	0	Phldb2	0	Olfr552	0	Phgr1	0
Ocrl	0	Phlda3	0	4930558K02Rik	0	Phgdh	0
Ocm	0	Phlda2	0	BRDN0000737405	0	Phf8	0
Ociad2	0	Phkg1	0	Olfr551	0	Phf7	0
Ociad1	0	Phkb	0	Dolpp1	0	Phf5a	0
Ocel1	0	Phka2	0	Olfr17	0	Phf3	0
Oca2	0	Phip	0	Myo1a	0	Phf23	0
Oc90	0	Phf8	0	Vgll2	0	Phf21b	0
Obsl1	0	Phf6	0	Vgll3	0	Phf20l1	0
Obscn	0	Phf5a	0	Gatad2a	0	Phf20	0
Obp2b	0	Phf3	0	Thnsl2	0	Phf2	0
Obp2a	0	Phf23	0	Vgll4	0	Phf13	0
Obp1a	0	Phf21a	0	Vash2	0	Phf12	0
Obox6	0	Phf20l1	0	Lcmt1	0	Phf11c	0
Obox3	0	Phf20	0	Fat3	0	Phf11b	0
Obox2	0	Phf19	0	Nup50	0	Phf11a	0
Obfc1	0	Phf11c	0	F8a	0	Phf1	0
Oat	0	Phf11a	0	AI413582	0	Phc3	0
Oasl2	0	Phf10	0	Raver2	0	Phc2	0
Oas3	0	Phf1	0	Nup54	0	Phb2	0
Oas2	0	Phc3	0	Olfr513	0	Phb	0
Oas1h	0	Phc2	0	Emb	0	Phax	0
Oas1g	0	Phb2	0	Atl3	0	Phactr4	0
Oas1f	0	Phb	0	Atl2	0	Phactr3	0
Oas1d	0	Phax	0	Atl1	0	Phactr2	0
Oas1c	0	Phactr4	0	Ldlrap1	0	Phactr1	0
Oas1a	0	Phactr2	0	Pxdc1	0	Pgrmc2	0
Oard1	0	Phactr1	0	Ascl2	0	Pgr	0
Oaf	0	Pgs1	0	4921536K21Rik	0	Pgpep1l	0
Oacyl	0	Pgrmc2	0	Zfp316	0	Pgpep1	0
Nyx	0	Pgrmc1	0	Hoxc12	0	Pgm5	0
Nyap2	0	Pgpep1l	0	Olfr53	0	Pgm3	0
Nyap1	0	Pgpep1	0	Hsd17b13	0	Pgm2l1	0
Nxt1	0	Pgm3	0	Acot1	0	Pglyrp4	0
Nxph4	0	Pgm2l1	0	Hsd17b10	0	Pglyrp3	0
Nxph3	0	Pgm2	0	Rsg1	0	Pglyrp2	0
Nxph2	0	Pgm1	0	Ulk1	0	Pgls	0
Nxph1	0	Pglyrp4	0	Kel	0	Pgk1	0
Nxpe3	0	Pglyrp2	0	Anxa2	0	Pggt1b	0
Nxpe2	0	Pglyrp1	0	BRDN0000737409	0	Pgf	0
Nxnl2	0	Pgls	0	Setd3	0	Pgd	0
Nxnl1	0	Pgk2	0	Mrgpra2b	0	Pgc	0
Nxn	0	Pgk1	0	Dscc1	0	Pgbd5	0
Nxf7	0	Pggt1b	0	Ruvbl2	0	Pgbd1	0
Nxf3	0	Pgf	0	Setd2	0	Pgap2	0
Nxf2	0	Pgd	0	Rpl29	0	Pgam1	0
Nxf1	0	Pgc	0	Rpl28	0	Pga5	0
Nwd2	0	Pgbd5	0	Farp1	0	Pfpl	0

Nwd1	0	Pgap3	0	Setd5	0	Pfn4	0
Nvl	0	Pgap2	0	Rpl27	0	Pfn3	0
Nutm2	0	Pgap1	0	Rpl26	0	Pfn2	0
Nutm1	0	Pgam5	0	Jrk	0	Pfn1	0
Nutf2	0	Pgam2	0	Rpl23	0	Pfkp	0
Nusap1	0	Pga5	0	Rpl22	0	Pfkm	0
Nus1	0	Pfpl	0	Nap1l2	0	Pfkfb2	0
Nupr1	0	Pfn4	0	Setd7	0	Pfdn6	0
Nupl2	0	Pfn3	0	Vstm5	0	Pfdn5	0
Nupl1	0	Pfn2	0	Vstm4	0	Pfdn1	0
Nup98	0	Pfn1	0	Oosp2	0	Pfas	0
Nup93	0	Pfkp	0	Oosp3	0	Pf4	0
Nup88	0	Pfkm	0	Bcar1	0	Pex7	0
Nup85	0	Pfkfb3	0	Bcar3	0	Pex6	0
Nup62	0	Pfkfb2	0	Pla2g2d	0	Pex5	0
Nup50	0	Pfdn6	0	Sorl1	0	Pex3	0
Nup43	0	Pfdn5	0	Wdr90	0	Pex2	0
Nup214	0	Pfdn4	0	Strn4	0	Pex19	0
Nup210l	0	Pfdn2	0	Sdsl	0	Pex16	0
Nup210	0	Pfdn1	0	Strn3	0	Pex14	0
Nup205	0	Pfas	0	Atp13a1	0	Pex13	0
Nup188	0	Pf4	0	Tom1	0	Pex12	0
Nup160	0	Pex5l	0	Atp13a3	0	Pex11g	0
Nup155	0	Pex5	0	Gm15293	0	Pex11b	0
Nup153	0	Pex3	0	Atp13a5	0	Pex11a	0
Nup133	0	Pex19	0	Atp13a4	0	Pex1	0
Nup107	0	Pex16	0	Ehf	0	Pet2	0
Numbl	0	Pex14	0	Ascl1	0	Pet117	0
Numb	0	Pex13	0	Olfr1297	0	Pet100	0
Numa1	0	Pex12	0	Olfr1295	0	Pes1	0
Nuggc	0	Pex11b	0	Olfr1294	0	Perp	0
Nufip2	0	Pex10	0	Ranbp10	0	Per3	0
Nufip1	0	Pex1	0	Olfr1299	0	Per1	0
Nudt9	0	Pet2	0	Ranbp17	0	Peo1	0
Nudt8	0	Pet100	0	Scube2	0	Penk	0
Nudt7	0	Pes1	0	Scube3	0	Pelo	0
Nudt6	0	Perp	0	Scube1	0	Peli3	0
Nudt5	0	Perm1	0	Pkd2	0	Peli1	0
Nudt3	0	Per3	0	Pkd1	0	Peg12	0
Nudt22	0	Per2	0	Crtc2	0	Peg10	0
Nudt21	0	Per1	0	Gm15299	0	Pef1	0
Nudt18	0	Pepd	0	Gpx1	0	Pecr	0
Nudt17	0	Peo1	0	Ctrb1	0	Pebp4	0
Nudt16l1	0	Penk	0	Gpx5	0	Peak1	0
Nudt16	0	Pemt	0	Gpx4	0	Pea15a	0
Nudt15	0	Pelp1	0	Gpx7	0	Pdzrn4	0
Nudt14	0	Pelo	0	Gpx6	0	Pdzrn3	0
Nudt13	0	Peli1	0	Gpx8	0	Pdzd9	0
Nudt12	0	Peg12	0	Unc50	0	Pdzd8	0
Nudt11	0	Peg10	0	Flt1	0	Pdzd4	0
Nudt10	0	Pef1	0	Dlc1	0	Pdzd2	0
Nudt1	0	Pecr	0	Them7	0	Pdyn	0
Nudcd3	0	Pebp4	0	Ctdspl	0	Pdxp	0
Nudcd2	0	Pebp1	0	Them4	0	Pdxdc1	0
Nudcd1	0	Peak1	0	Olfr974	0	Pds5b	0
Nudc	0	Pea15a	0	Ifi204	0	Pds5a	0
Nucks1	0	Pdzrn3	0	Olfr976	0	Pdrg1	0
Nucb2	0	Pdzph1	0	Spg7	0	Pdpr	0
Nubpl	0	Pdzk1ip1	0	Ube3b	0	Pdpk1	0
Nubp2	0	Pdzd9	0	Olfr979	0	Pdp2	0
Nubp1	0	Pdzd7	0	Ube3a	0	Pdlim7	0
Nuak1	0	Pdzd3	0	Tomt	0	Pdlim5	0
Ntsr2	0	Pdzd11	0	Hmga1-rs1	0	Pdlim4	0
Ntsr1	0	Pdxp	0	Uqcrq	0	Pdlim3	0
Nts	0	Pdxk	0	Hspd1	0	Pdlim2	0
Ntrk2	0	Pdxdc1	0	Spa17	0	Pdlim1	0
Ntrk1	0	Pdx1	0	Uqcrh	0	Pdk4	0
Ntpcr	0	Pdss2	0	Ahrr	0	Pdk3	0
Ntn5	0	Pdss1	0	Mbl1	0	Pdilt	0
Ntn4	0	Pds5a	0	Rmi1	0	Pdik1l	0
Ntn3	0	Pdrg1	0	Rmi2	0	Pdia5	0
Ntn1	0	Pdpr	0	Mageh1	0	Pdia3	0
Ntmt1	0	Pdpn	0	Col10a1	0	Pdhx	0
Nthl1	0	Pdpk1	0	Mettl9	0	Pdhb	0
Ntf5	0	Pdp2	0	Ifi205	0	Pdha2	0
Ntf3	0	Pdlim5	0	Cdk20	0	Pdha1	0
Nt5m	0	Pdlim4	0	Cep170	0	Pdgfrl	0
Nt5e	0	Pdlim2	0	Unc5b	0	Pdgfrb	0
Nt5dc3	0	Pdlim1	0	Cfap20	0	Pdgfra	0
Nt5dc2	0	Pdk4	0	Mettl2	0	Pdgfd	0
Nt5dc1	0	Pdk2	0	Unc5a	0	Pdgfc	0
Nt5c3	0	Pdilt	0	Mettl4	0	Pdgfa	0

Nt5c2	0	Pdik1l	0	Mettl5	0	Pde9a	0
Nt5c1b	0	Pdia4	0	Unc5d	0	Pde8a	0
Nt5c1a	0	Pdhx	0	Phf3	0	Pde7b	0
Nt5c	0	Pdhb	0	Fastk	0	Pde7a	0
Nsun7	0	Pdha1	0	Socs2	0	Pde6h	0
Nsun6	0	Pdgfrl	0	3010026O09Rik	0	Pde6b	0
Nsun5	0	Pdgfra	0	Hsdl2	0	Pde6a	0
Nsun3	0	Pdgfd	0	Esyt1	0	Pde4dip	0
Nsun2	0	Pdgfc	0	Ccar1	0	Pde4d	0
Nsmce4a	0	Pdgfb	0	Ccar2	0	Pde3b	0
Nsmce1	0	Pdgfa	0	Phf1	0	Pde3a	0
Nsmaf	0	Pdf	0	Olfr791	0	Pde2a	0
Nsg2	0	Pde9a	0	Vmn2r97	0	Pde1c	0
Nsg1	0	Pde8a	0	Rab27a	0	Pde1b	0
Nsfl1c	0	Pde7b	0	Rab27b	0	Pde11a	0
Nsf	0	Pde6h	0	Cog8	0	Pde10a	0
Nsd1	0	Pde6d	0	Aven	0	Pddc1	0
Nsa2	0	Pde6c	0	Vmn2r92	0	Pdcl3	0
Nrxn2	0	Pde6a	0	Vmn2r91	0	Pdcl2	0
Nrxn1	0	Pde4dip	0	Ptrhd1	0	Pdcl	0
Nrtn	0	Pde4d	0	Cog3	0	Pdcd7	0
Nrsn2	0	Pde4b	0	Cog2	0	Pdcd6ip	0
Nrsn1	0	Pde4a	0	Cog1	0	Pdcd6	0
Nrp2	0	Pde3b	0	Szrd1	0	Pdcd5	0
Nrp1	0	Pde3a	0	2700089E24Rik	0	Pdcd4	0
Nrn1l	0	Pde2a	0	Glcci1	0	Pdcd2l	0
Nrn1	0	Pde1c	0	Vmn2r99	0	Pdcd2	0
Nrm	0	Pde1b	0	Sc5d	0	Pdcd1lg2	0
Nrl	0	Pde11a	0	Deaf1	0	Pdcd11	0
Nrk	0	Pde10a	0	Magee2	0	Pdcd10	0
Nrip3	0	Pdcl2	0	Actbl2	0	Pdc	0
Nrip2	0	Pdcl	0	Rybp	0	Pdap1	0
Nrgn	0	Pdcd6ip	0	Fam189a1	0	Pcyt2	0
Nrg3	0	Pdcd6	0	Yap1	0	Pcyt1a	0
Nrg2	0	Pdcd4	0	Tspan9	0	Pctp	0
Nrg1	0	Pdcd2l	0	Rab18	0	Pcsk6	0
Nrf1	0	Pdcd1lg2	0	Gm15800	0	Pcsk5	0
Nrep	0	Pdcd11	0	Igf1	0	Pcsk2	0
Nrde2	0	Pdcd1	0	Esyt3	0	Pcsk1n	0
Nrd1	0	Pdap1	0	Spo11	0	Pcsk1	0
Nrcam	0	Pcyt2	0	Ppcs	0	Pcp4	0
Nrbp2	0	Pcyt1a	0	Gm853	0	Pcp2	0
Nrbp1	0	Pcyox1l	0	Cyp2d11	0	Pcolce	0
Nras	0	Pcyox1	0	Cd6	0	Pcnxl4	0
Nrarp	0	Pcx	0	Lgalsl	0	Pcnxl2	0
Nradd	0	Pctp	0	Defa-rs7	0	Pcnx	0
Nr6a1	0	Pcsk9	0	Ubap2	0	Pcnt	0
Nr5a2	0	Pcsk7	0	Ubap1	0	Pcna	0
Nr5a1	0	Pcsk5	0	Ppap2c	0	Pcmtd2	0
Nr4a3	0	Pcsk1	0	Ppap2b	0	Pcmtd1	0
Nr4a2	0	Pcp4l1	0	Ppap2a	0	Pcm1	0
Nr4a1	0	Pcp4	0	BRDN0000738138	0	Pck2	0
Nr3c2	0	Pcolce2	0	Tpd52l1	0	Pck1	0
Nr3c1	0	Pcolce	0	Leng9	0	Pcif1	0
Nr2f2	0	Pcnxl4	0	Klhl41	0	Pcid2	0
Nr2f1	0	Pcnxl3	0	Klhl40	0	Pcgf5	0
Nr2e1	0	Pcnxl2	0	Pole3	0	Pcgf2	0
Nr2c1	0	Pcnt	0	Pole2	0	Pcf11	0
Nr1i3	0	Pcnp	0	Eps8l1	0	Pced1b	0
Nr1i2	0	Pcna	0	Rab13	0	Pcdhgc5	0
Nr1h5	0	Pcmtd1	0	Sos2	0	Pcdhgc3	0
Nr1h4	0	Pcmt1	0	Lama1	0	Pcdhgb7	0
Nr1h3	0	Pck2	0	Lama2	0	Pcdhgb6	0
Nr1h2	0	Pck1	0	Got1	0	Pcdhgb4	0
Nr1d2	0	Pcif1	0	Lama4	0	Pcdhgb2	0
Nr1d1	0	Pcgf5	0	Olfr1057	0	Pcdhgb1	0
Nqo1	0	Pcgf3	0	Sike1	0	Pcdhga6	0
Npy6r	0	Pcgf2	0	Ibsp	0	Pcdhga4	0
Npy4r	0	Pcf11	0	Skiv2l2	0	Pcdhga2	0
Npy2r	0	Pced1b	0	Gng10	0	Pcdhga12	0
Npy1r	0	Pcdhgc3	0	Tbc1d32	0	Pcdhb8	0
Npy	0	Pcdhgb2	0	Foxs1	0	Pcdhb7	0
Npw	0	Pcdhgb1	0	Ptpn6	0	Pcdhb6	0
Npvf	0	Pcdhga9	0	Atrip	0	Pcdhb5	0
Nptn	0	Pcdhga8	0	BRDN0000737469	0	Pcdhb4	0
Npsr1	0	Pcdhga7	0	Utrn	0	Pcdhb3	0
Nprl3	0	Pcdhga6	0	Arhgap11a	0	Pcdhb22	0
Nprl2	0	Pcdhga3	0	Prph2	0	Pcdhb19	0
Npr2	0	Pcdhga2	0	Clptm1l	0	Pcdhb18	0
Npr1	0	Pcdhga12	0	Adat3	0	Pcdhb17	0
Nppc	0	Pcdhga10	0	Wdpcp	0	Pcdhb16	0
Nppb	0	Pcdhb8	0	Gm13287	0	Pcdhb15	0

Nppa	0
Npm2	0
Npm1	0
Nploc4	0
Npl	0
Nphs2	0
Nphs1	0
Nphp4	0
Nphp3	0
Nphp1	0
Npffr2	0
Npffr1	0
Npff	0
Npepps	0
Npcd	0
Npc2	0
Npc1l1	0
Npc1	0
Npbwr1	0
Npb	0
Npat	0
Npas4	0
Npas2	0
Npas1	0
Noxred1	0
Noxo1	0
Noxa1	0
Nox4	0
Nox1	0
Nova2	0
Nova1	0
Nov	0
Notum	0
Notch4	0
Notch1	0
Nostrin	0
Nosip	0
Nos3	0
Nos2	0
Nos1ap	0
Nop9	0
Nop58	0
Nop56	0
Nop2	0
Nop16	0
Nop14	0
Nop10	0
Nomo1	0
Nom1	0
Nolc1	0
Nol8	0
Nol7	0
Nol6	0
Nol12	0
Nol11	0
Nol10	0
Nog	0
Nodal	0
Nod2	0
Nod1	0
Noc4l	0
Noc3l	0
Noc2l	0
Nob1	0
Noa1	0
Nnmt	0
Nnat	0
Nmur1	0
Nmu	0
Nmt2	0
Nmt1	0
Nms	0
Nmrk2	0
Nmrk1	0
Nmnat3	0
Nmnat2	0
Nmnat1	0
Nmi	0
Nme8	0
Nme7	0
Nme6	0
Nme5	0
Pcdhb22	0
Pcdhb21	0
Pcdhb20	0
Pcdhb2	0
Pcdhb19	0
Pcdhb16	0
Pcdhb15	0
Pcdhb14	0
Pcdhb13	0
Pcdhb12	0
Pcdhb11	0
Pcdhb1	0
Pcdhac2	0
Pcdhac1	0
Pcdha8	0
Pcdha6	0
Pcdha5	0
Pcdha4	0
Pcdha3	0
Pcdha2	0
Pcdha1	0
Pcdh8	0
Pcdh7	0
Pcdh19	0
Pcdh18	0
Pcdh17	0
Pcdh12	0
Pcdh11x	0
Pcdh10	0
Pcdh1	0
Pccb	0
Pcca	0
Pcbp3	0
Pcbd1	0
Pbxip1	0
Pbx4	0
Pbx2	0
Pbsn	0
Pbk	0
Paxip1	0
Pax9	0
Pax8	0
Pax7	0
Pax5	0
Pax4	0
Pax3	0
Pax2	0
Pawr	0
Patl2	0
Pate2	0
Parva	0
Pars2	0
Parp9	0
Parp6	0
Parp2	0
Parp16	0
Parp14	0
Parp12	0
Parp10	0
Parn	0
Parl	0
Parg	0
Pard3b	0
Pard3	0
Paqr9	0
Paqr8	0
Paqr7	0
Paqr5	0
Paqr4	0
Papss2	0
Pappa2	0
Pappa	0
Papolb	0
Papola	0
Papd7	0
Papd5	0
Papd4	0
Paox	0
Panx3	0
Panx2	0
Pank3	0
Pank2	0
Mien1	0
B3glct	0
Cxcl1	0
Gm13043	0
Cxcl3	0
Cxcl2	0
Cxcl5	0
Pianp	0
Tbxa2r	0
Cxcl9	0
Olfr303	0
St5	0
Golim4	0
St7	0
Ccrn4l	0
Cyp26a1	0
B630005N14Rik	0
P2rx1	0
Mipol1	0
P2rx2	0
P2rx5	0
P2rx4	0
P2rx7	0
P2rx6	0
Mphosph8	0
Myo18a	0
Fsd2	0
Klf17	0
Klf16	0
Klf15	0
Klf14	0
BRDN0000737981	0
Tfg	0
Itgb1bp2	0
Zfp536	0
Zfp534	0
Mphosph6	0
Lsm1	0
Orc6	0
Ncl	0
L1td1	0
Limch1	0
Ngfr	0
1700011E24Rik	0
Map6d1	0
D730048I06Rik	0
Erich4	0
Kctd2	0
Phb2	0
Als2	0
Hps5	0
Hps4	0
Hps6	0
Hps1	0
Wdfy2	0
Wdfy1	0
Manea	0
Lamtor4	0
Lamtor2	0
Lamtor3	0
Hes3	0
Dhx58	0
Pabpn1l	0
Fam133b	0
Serpinb6e	0
Adipor2	0
Serpinb6a	0
Cbl	0
Serpinb6c	0
Arhgef39	0
Bcl3	0
Fam199x	0
Flrt1	0
4930428D18Rik	0
Sult3a1	0
4933402J07Rik	0
Fgf22	0
Pla2g12a	0
Fgf20	0
Fgf21	0
Gm15319	0
Lif	0
Pcdhb14	0
Pcdhb13	0
Pcdhb12	0
Pcdhb1	0
Pcdha9	0
Pcdha8	0
Pcdha7	0
Pcdha5	0
Pcdha2	0
Pcdha10	0
Pcdha1	0
Pcdh9	0
Pcdh8	0
Pcdh7	0
Pcdh15	0
Pcdh11x	0
Pccb	0
Pcbd1	0
Pbxip1	0
Pbx4	0
Pbx3	0
Pbx1	0
Pbsn	0
Pbp2	0
Pbk	0
Pbdc1	0
Paxip1	0
Pax8	0
Pax7	0
Pax5	0
Pax4	0
Pax3	0
Pax1	0
Pawr	0
Pate4	0
Pate2	0
Parvg	0
Parvb	0
Parva	0
Parpbp	0
Parp9	0
Parp8	0
Parp4	0
Parp3	0
Parp16	0
Parp14	0
Parp12	0
Parp11	0
Parp1	0
Parn	0
Parm1	0
Park2	0
Parg	0
Pard6b	0
Pard6a	0
Pard3b	0
Pard3	0
Paqr9	0
Paqr8	0
Paqr4	0
Paqr3	0
Papss2	0
Papss1	0
Pappa2	0
Pappa	0
Papolg	0
Papola	0
Papl	0
Papd7	0
Papd5	0
Paox	0
Panx3	0
Panx2	0
Pank1	0
Pan3	0
Pan2	0
Pamr1	0
Pam16	0
Palmd	0
Palm3	0
Palm2	0
Palm	0

Nme4	0	Pank1	0	BRDN0000738172	0	Palld	0
Nme3	0	Pan3	0	Uck1	0	Pald1	0
Nme2	0	Pan2	0	Uck2	0	Palb2	0
Nme1	0	Pamr1	0	Ift172	0	Pak7	0
Nmd3	0	Pam16	0	Apex1	0	Pak6	0
Nmbr	0	Palmd	0	BRDN0000738042	0	Pak4	0
Nlrx1	0	Palm3	0	Muc20	0	Pak1ip1	0
Nlrp9b	0	Palm2	0	Tmem194b	0	Pak1	0
Nlrp9a	0	Palm	0	Tmem176a	0	Paip2b	0
Nlrp6	0	Pald1	0	Polr3gl	0	Paip2	0
Nlrp4f	0	Palb2	0	Tmem176b	0	Paip1	0
Nlrp4e	0	Pak7	0	Aldh1b1	0	Paics	0
Nlrp4c	0	Pak6	0	Syf2	0	Pagr1a	0
Nlrp4a	0	Pak3	0	Fank1	0	Pag1	0
Nlrp3	0	Pak2	0	Zfp358	0	Pafah2	0
Nlrp1b	0	Pak1	0	Pus7l	0	Pafah1b1	0
Nlrp1a	0	Paip2	0	Eri3	0	Paf1	0
Nlrp14	0	Paics	0	Olfr301	0	Padi4	0
Nlrp12	0	Pah	0	Lilrb4	0	Padi3	0
Nlrp10	0	Pagr1a	0	Zbed4	0	Padi1	0
Nlrc5	0	Pag1	0	Zbed5	0	Pacsin3	0
Nlrc4	0	Pafah2	0	Zbed6	0	Pacsin2	0
Nlrc3	0	Pafah1b3	0	Olfr304	0	Pacs2	0
Nln	0	Pafah1b2	0	Susd1	0	Pacs1	0
Nlgn2	0	Pafah1b1	0	Olfr309	0	Pacrgl	0
Nle1	0	Paf1	0	Olfr308	0	Pabpn1	0
Nkx6-2	0	Padi6	0	Rgs9bp	0	Pabpc6	0
Nkx6-1	0	Padi4	0	0610010K14Rik	0	Pabpc5	0
Nkx3-1	0	Padi3	0	Mgl2	0	Pabpc4l	0
Nkx2-9	0	Padi1	0	Kap	0	Pabpc2	0
Nkx2-6	0	Pacsin3	0	Rho	0	Pabpc1l	0
Nkx2-2	0	Pacsin1	0	BRDN0000738037	0	Pabpc1	0
Nkx1-1	0	Pacs2	0	Ilvbl	0	P4htm	0
Nktr	0	Pacrgl	0	Mif4gd	0	P4hb	0
Nkrf	0	Pacrg	0	Alpl	0	P4ha3	0
Nkpd1	0	Pabpn1l	0	Ccdc115	0	P4ha2	0
Nkiras2	0	Pabpn1	0	Klhl11	0	P4ha1	0
Nkiras1	0	Pabpc5	0	Atg101	0	P3h3	0
Nkg7	0	Pabpc4l	0	Fam78a	0	P3h2	0
Nkd2	0	Pabpc1l	0	Treh	0	P3h1	0
Nkd1	0	Pabpc1	0	Olfr292	0	P2ry6	0
Nkapl	0	P4htm	0	Lefty2	0	P2ry14	0
Nkap	0	P4hb	0	Lefty1	0	P2ry13	0
Nkain3	0	P4ha1	0	Hist2h4	0	P2ry12	0
Nkain2	0	P3h4	0	Slc7a6os	0	P2ry1	0
Nit1	0	P3h3	0	Cstf3	0	P2rx7	0
Nisch	0	P3h1	0	Ap2a1	0	P2rx6	0
Nipsnap3b	0	P2ry6	0	Klhl14	0	P2rx5	0
Nipsnap3a	0	P2ry2	0	Cstf2	0	P2rx3	0
Nipsnap1	0	P2ry14	0	Klf10	0	P2rx2	0
Nipbl	0	P2ry13	0	Kbtbd7	0	P2rx1	0
Nipal4	0	P2ry1	0	Kbtbd3	0	Oxt	0
Nipal2	0	P2rx7	0	Kbtbd2	0	Oxsr1	0
Nipal1	0	P2rx6	0	Gzmm	0	Oxsm	0
Nipa2	0	P2rx3	0	Glg1	0	Oxr1	0
Nipa1	0	P2rx2	0	Olfr299	0	Oxnad1	0
Nip7	0	P2rx1	0	Mgll	0	Oxld1	0
Ninj2	0	Oxtr	0	Smtnl1	0	Oxgr1	0
Ninj1	0	Oxt	0	Kbtbd8	0	Oxct2a	0
Nin	0	Oxsr1	0	Sh3d19	0	Oxct1	0
Nim1k	0	Oxsm	0	Mettl7a2	0	Oxa1l	0
Nifk	0	Oxr1	0	Mettl7a3	0	Ovol1	0
Nid2	0	Oxnad1	0	Mettl7a1	0	Ovgp1	0
Nid1	0	Oxld1	0	Neu4	0	Ovca2	0
Nhsl2	0	Oxgr1	0	Lrrc9	0	Otx2	0
Nhsl1	0	Oxct1	0	Rce1	0	Otx1	0
Nhp2l1	0	Oxa1l	0	Atp7a	0	Otulin	0
Nhp2	0	Ovol2	0	Neu1	0	Otud7a	0
Nhlrc4	0	Ovol1	0	Rsad1	0	Otud6b	0
Nhlrc3	0	Ovgp1	0	Atp7b	0	Otud6a	0
Nhlrc2	0	Ovca2	0	Tceb3	0	Otud3	0
Nhlh2	0	Otx1	0	Znrd1	0	Otub1	0
Nhlh1	0	Otulin	0	BRDN0000737868	0	Ott	0
Ngrn	0	Otud7a	0	Slc8b1	0	Otp	0
Ngp	0	Otud6a	0	Nav3	0	Otos	0
Ngly1	0	Otud5	0	En2	0	Otor	0
Ngfr	0	Otud4	0	A930009A15Rik	0	Otop3	0
Ngf	0	Otud3	0	Gpd1l	0	Otop2	0
Ngef	0	Otud1	0	Sh3yl1	0	Otol1	0
Ngdn	0	Otub2	0	Ip6k3	0	Otog	0
Nfyc	0	Ott	0	Ip6k2	0	Otc	0
Nfya	0	Otop2	0	Ip6k1	0	Ostn	0

Nfxl1	0	Otol1	0	Nme4	0	Ostm1	0
Nfu1	0	Otogl	0	Nme5	0	Ostf1	0
Nfs1	0	Otog	0	Nme6	0	Ostc	0
Nfkbil1	0	Otof	0	Bms1	0	Ost4	0
Nfkbid	0	Otoa	0	Nme1	0	Osr2	0
Nfkbib	0	Otc	0	E230019M04Rik	0	Osr1	0
Nfkbia	0	Ostn	0	Nme3	0	Osmr	0
Nfkb2	0	Ostm1	0	Cstb	0	Osgin2	0
Nfkb1	0	Ostf1	0	Hsd11b2	0	Osgin1	0
Nfix	0	Osr1	0	Arpc5l	0	Osgepl1	0
Nfil3	0	Osmr	0	Eapp	0	Oser1	0
Nfic	0	Osm	0	C1qtnf7	0	Oscar	0
Nfia	0	Osgin2	0	Tmem154	0	Osbpl9	0
Nfe2l3	0	Osgin1	0	Sfpq	0	Osbpl8	0
Nfe2l2	0	Osgepl1	0	BRDN0000737893	0	Osbpl6	0
Nfe2l1	0	Osgep	0	Ubqln2	0	Osbpl1a	0
Nfe2	0	Oser1	0	Top3b	0	Osbp	0
Nfatc4	0	Oscar	0	Tmem158	0	Os9	0
Nfatc3	0	Osbpl9	0	Tmem159	0	Ormdl2	0
Nfatc2ip	0	Osbpl8	0	Tacr1	0	Ormdl1	0
Nfatc2	0	Osbpl5	0	Scaf1	0	Orm2	0
Nfatc1	0	Osbpl2	0	Cptp	0	Orm1	0
Nfat5	0	Osbp2	0	2410002F23Rik	0	Orc6	0
Nfasc	0	Osbp	0	Pgrmc1	0	Orc5	0
Nf2	0	Ormdl3	0	Oxt	0	Orc4	0
Nexn	0	Ormdl1	0	Zfp850	0	Orc3	0
Neurog3	0	Orm3	0	Poc5	0	Orc2	0
Neurog2	0	Orm2	0	Tmem45a2	0	Orc1	0
Neurog1	0	Orm1	0	Plekhh3	0	Oraov1	0
Neurod6	0	Orc6	0	Plekhh2	0	Orai3	0
Neurl3	0	Orc5	0	Plekhh1	0	Orai2	0
Neurl2	0	Orc4	0	Hid1	0	Orai1	0
Neu4	0	Orc3	0	Sost	0	Optn	0
Neu3	0	Orc1	0	P3h3	0	Oprl1	0
Neu2	0	Oraov1	0	P3h2	0	Oprk1	0
Neu1	0	Orai3	0	P3h1	0	Oprd1	0
Neto2	0	Orai2	0	Lrrcc1	0	Opn4	0
Neto1	0	Orai1	0	Blvra	0	Opn3	0
Net1	0	Optc	0	Slc46a3	0	Opn1mw	0
Nes	0	Oprm1	0	Carns1	0	Oplah	0
Neo1	0	Oprl1	0	Slc46a1	0	Opalin	0
Nenf	0	Oprk1	0	Zdhhc1	0	Opa1	0
Nell2	0	Oprd1	0	Gm5878	0	Oosp3	0
Nelfe	0	Opn4	0	Zdhhc3	0	Oosp2	0
Nelfb	0	Oplah	0	Ddit3	0	Oog4	0
Nelfa	0	Ophn1	0	Zdhhc5	0	Oog2	0
Nek9	0	Opa1	0	Zdhhc4	0	Ooep	0
Nek7	0	Oosp2	0	Zdhhc7	0	Onecut3	0
Nek4	0	Oosp1	0	Zdhhc6	0	Onecut2	0
Nek3	0	Oog4	0	Zdhhc9	0	Onecut1	0
Nek2	0	Oog3	0	Zdhhc8	0	Omt2b	0
Nek11	0	Oog2	0	Gm20854	0	Omt2a	0
Nek10	0	Ooep	0	Vps18	0	Omp	0
Nek1	0	Onecut1	0	Il11ra1	0	Omg	0
Neil3	0	Omt2b	0	Tlx1	0	Omd	0
Neil2	0	Omt2a	0	Scn10a	0	Oma1	0
Negr1	0	Omg	0	Cst8	0	Olig3	0
Nefm	0	Omd	0	Slc23a2	0	Olig2	0
Nefl	0	Olig3	0	AI182371	0	Olfr995	0
Nedd9	0	Olig2	0	Cst3	0	Olfr992	0
Nedd8	0	Olfr998	0	Cst6	0	Olfr988	0
Nedd4l	0	Olfr996	0	Cst7	0	Olfr987	0
Nedd4	0	Olfr995	0	Dera	0	Olfr986	0
Nedd1	0	Olfr994	0	Ubap1l	0	Olfr983	0
Necap2	0	Olfr99	0	Fubp1	0	Olfr982	0
Necap1	0	Olfr988	0	Usmg5	0	Olfr981	0
Necab2	0	Olfr987	0	Pdcd5	0	Olfr980	0
Necab1	0	Olfr983	0	Zfhx2	0	Olfr98	0
Nebl	0	Olfr982	0	Bbx	0	Olfr978	0
Neb	0	Olfr981	0	Grm2	0	Olfr976	0
Ndufv3	0	Olfr98	0	Dlx1	0	Olfr972	0
Ndufv2	0	Olfr979	0	Vmn2r70	0	Olfr971	0
Ndufs6	0	Olfr978	0	BRDN0000738370	0	Olfr970	0
Ndufs4	0	Olfr976	0	BRDN0000737780	0	Olfr97	0
Ndufc2	0	Olfr975	0	Olfr1093	0	Olfr969	0
Ndufc1	0	Olfr974	0	Daf2	0	Olfr968	0
Ndufb9	0	Olfr970	0	Gm14496	0	Olfr965	0
Ndufb8	0	Olfr97	0	Opn1mw	0	Olfr963	0
Ndufb7	0	Olfr968	0	Gzmk	0	Olfr961	0
Ndufb6	0	Olfr967	0	Ap4b1	0	Olfr960	0
Ndufb5	0	Olfr965	0	Dleu7	0	Olfr96	0
Ndufb4	0	Olfr958	0	BRDN0000737782	0	Olfr959	0

Ndufb3	0	Olfr957	0	Agr2	0	Olfr958	0
Ndufb2	0	Olfr955	0	Olfr1338	0	Olfr955	0
Ndufb11	0	Olfr954	0	Olfr1339	0	Olfr952	0
Ndufb10	0	Olfr951	0	BRDN0000738057	0	Olfr951	0
Ndufaf7	0	Olfr95	0	Lrrfip2	0	Olfr948	0
Ndufaf6	0	Olfr948	0	Olfr1330	0	Olfr945	0
Ndufaf5	0	Olfr944	0	Olfr1331	0	Olfr944	0
Ndufaf4	0	Olfr943	0	Noxo1	0	Olfr943	0
Ndufaf2	0	Olfr94	0	Olfr1333	0	Olfr94	0
Ndufaf1	0	Olfr937	0	Olfr1335	0	Olfr938	0
Ndufab1	0	Olfr936	0	Olfr1336	0	Olfr937	0
Ndufa9	0	Olfr933	0	Olfr1337	0	Olfr936	0
Ndufa8	0	Olfr926	0	Sod2	0	Olfr935	0
Ndufa7	0	Olfr924	0	Sptan1	0	Olfr934	0
Ndufa6	0	Olfr922	0	Gc	0	Olfr933	0
Ndufa4l2	0	Olfr921	0	Casd1	0	Olfr930	0
Ndufa4	0	Olfr920	0	Hivep1	0	Olfr926	0
Ndufa3	0	Olfr919	0	Hivep2	0	Olfr923	0
Ndufa2	0	Olfr918	0	Hivep3	0	Olfr921	0
Ndufa11	0	Olfr916	0	Kiss1r	0	Olfr92	0
Ndufa10	0	Olfr914	0	Zfp703	0	Olfr919	0
Ndufa1	0	Olfr913	0	BRDN0000737402	0	Olfr917	0
Ndst4	0	Olfr912	0	Zfp704	0	Olfr916	0
Ndst3	0	Olfr911-ps1	0	Zfp706	0	Olfr914	0
Ndst2	0	Olfr91	0	Zfp709	0	Olfr913	0
Ndrg4	0	Olfr907	0	Abhd15	0	Olfr912	0
Ndrg3	0	Olfr906	0	Sufu	0	Olfr911-ps1	0
Ndrg1	0	Olfr905	0	Abhd10	0	Olfr91	0
Ndp	0	Olfr904	0	Abhd11	0	Olfr907	0
Ndnl2	0	Olfr902	0	Abhd12	0	Olfr905	0
Ndnf	0	Olfr90	0	Abhd13	0	Olfr904	0
Ndfip1	0	Olfr898	0	Ephx3	0	Olfr902	0
Ndel1	0	Olfr894	0	Fhad1	0	Olfr901	0
Ndc80	0	Olfr891	0	Bbc3	0	Olfr898	0
Ndc1	0	Olfr890	0	Trove2	0	Olfr894	0
Ncstn	0	Olfr887	0	Fbxo34	0	Olfr890	0
Ncs1	0	Olfr885	0	Cct8	0	Olfr889	0
Ncr1	0	Olfr884	0	Olfr898	0	Olfr888	0
Ncor2	0	Olfr883	0	2310079G19Rik	0	Olfr884	0
Ncor1	0	Olfr881	0	Olfr893	0	Olfr883	0
Ncoa7	0	Olfr878	0	Cct3	0	Olfr881	0
Ncoa6	0	Olfr877	0	Olfr891	0	Olfr878	0
Ncoa5	0	Olfr875	0	Olfr890	0	Olfr877	0
Ncoa4	0	Olfr874	0	Cct7	0	Olfr876	0
Ncmap	0	Olfr873	0	Cct4	0	Olfr875	0
Ncln	0	Olfr869	0	Cct5	0	Olfr874	0
Ncl	0	Olfr868	0	Serping1	0	Olfr873	0
Nckap5l	0	Olfr866	0	Depdc7	0	Olfr872	0
Nckap5	0	Olfr860	0	Prom1	0	Olfr871	0
Nckap1l	0	Olfr859	0	Pdia6	0	Olfr870	0
Nckap1	0	Olfr857	0	Chtf8	0	Olfr869	0
Nck2	0	Olfr854	0	Efhc1	0	Olfr868	0
Nck1	0	Olfr853	0	Pdia5	0	Olfr866	0
Ncf4	0	Olfr851	0	Crebzf	0	Olfr862	0
Ncf2	0	Olfr850	0	Cmbl	0	Olfr860	0
Ncf1	0	Olfr847	0	Pdia4	0	Olfr859	0
Nceh1	0	Olfr846	0	Brsk2	0	Olfr857	0
Ncdn	0	Olfr845	0	Brsk1	0	Olfr855	0
Ncbp2	0	Olfr843	0	Spag16	0	Olfr854	0
Ncbp1	0	Olfr836	0	Selo	0	Olfr853	0
Ncaph2	0	Olfr832	0	Disc1	0	Olfr851	0
Ncaph	0	Olfr830	0	Ppp1r8	0	Olfr847	0
Ncapg2	0	Olfr829	0	Fam129a	0	Olfr846	0
Ncapg	0	Olfr827	0	Vwce	0	Olfr845	0
Ncapd3	0	Olfr825	0	Fam129c	0	Olfr843	0
Ncan	0	Olfr824	0	Fam129b	0	Olfr836	0
Ncam2	0	Olfr823	0	Letm1	0	Olfr835	0
Ncam1	0	Olfr820	0	Tm4sf19	0	Olfr834	0
Ncald	0	Olfr819	0	Pik3c3	0	Olfr832	0
Nbr1	0	Olfr816	0	Lmbr1	0	Olfr830	0
Nbl1	0	Olfr814	0	Grxcr1	0	Olfr829	0
Nbeal2	0	Olfr812	0	D630003M21Rik	0	Olfr828	0
Nbeal1	0	Olfr810	0	Notch4	0	Olfr826	0
Nbea	0	Olfr807	0	Kif6	0	Olfr823	0
Nav3	0	Olfr806	0	Notch1	0	Olfr822	0
Nav1	0	Olfr805	0	Notch2	0	Olfr820	0
Nat9	0	Olfr804	0	Mest	0	Olfr819	0
Nat8l	0	Olfr801	0	Rps19bp1	0	Olfr816	0
Nat8	0	Olfr800	0	Skiv2l	0	Olfr815	0
Nat6	0	Olfr799	0	Bphl	0	Olfr814	0
Nat14	0	Olfr798	0	Grxcr2	0	Olfr813	0
Nat10	0	Olfr796	0	Pip5kl1	0	Olfr811	0

Nat1	0	Olfr794	0	Vamp3	0	Olfr809	0
Nasp	0	Olfr792	0	Vamp2	0	Olfr808	0
Nars2	0	Olfr791	0	Vamp5	0	Olfr806	0
Nars	0	Olfr790	0	Syncrip	0	Olfr805	0
Narfl	0	Olfr788	0	Amer1	0	Olfr802	0
Narf	0	Olfr787	0	Amer2	0	Olfr801	0
Napsa	0	Olfr786	0	Amer3	0	Olfr8	0
Naprt	0	Olfr784	0	Cela3b	0	Olfr796	0
Napg	0	Olfr782	0	Cerk	0	Olfr792	0
Napepld	0	Olfr78	0	Mtif2	0	Olfr791	0
Napa	0	Olfr776	0	Itpr2	0	Olfr788	0
Nap1l4	0	Olfr774	0	Gimap8	0	Olfr787	0
Nap1l3	0	Olfr773	0	Gimap9	0	Olfr786	0
Nap1l2	0	Olfr772	0	Spz1	0	Olfr784	0
Nap1l1	0	Olfr771	0	Gm3763	0	Olfr782	0
Nans	0	Olfr770	0	Gimap4	0	Olfr781	0
Nanp	0	Olfr769	0	Gimap5	0	Olfr78	0
Nanos3	0	Olfr768	0	Gimap6	0	Olfr777	0
Nanos2	0	Olfr765	0	Gimap7	0	Olfr776	0
Nanos1	0	Olfr763	0	Gimap1	0	Olfr775	0
Nanog	0	Olfr761	0	Gimap3	0	Olfr774	0
Nampt	0	Olfr76	0	Kcnd1	0	Olfr773	0
Naip6	0	Olfr750	0	Dcakd	0	Olfr771	0
Naip5	0	Olfr749	0	Kcnd3	0	Olfr770	0
Naip2	0	Olfr748	0	Kcnd2	0	Olfr769	0
Naip1	0	Olfr747	0	Sec14l3	0	Olfr767	0
Naif1	0	Olfr746	0	Sec14l2	0	Olfr765	0
Nags	0	Olfr744	0	Sec14l1	0	Olfr761	0
Nagpa	0	Olfr743	0	Net1	0	Olfr76	0
Naglu	0	Olfr739	0	Sec14l5	0	Olfr748	0
Naga	0	Olfr736	0	Sec14l4	0	Olfr746	0
Naf1	0	Olfr733	0	BRDN0000737488	0	Olfr744	0
Nadk2	0	Olfr732	0	A630001G21Rik	0	Olfr742	0
Nacc2	0	Olfr731	0	Olfr707	0	Olfr74	0
Nacc1	0	Olfr73	0	Myl12b	0	Olfr739	0
Nacad	0	Olfr729	0	Olfr706	0	Olfr738	0
Naca	0	Olfr727	0	Supt20	0	Olfr736	0
Nabp2	0	Olfr724	0	Syt13	0	Olfr735	0
Nab2	0	Olfr723	0	E4f1	0	Olfr733	0
Naaladl1	0	Olfr722	0	March2	0	Olfr732	0
Naaa	0	Olfr720	0	Etnppl	0	Olfr730	0
Naa60	0	Olfr715	0	Mcm7	0	Olfr73	0
Naa50	0	Olfr711	0	Mcm6	0	Olfr728	0
Naa38	0	Olfr710	0	Mcm5	0	Olfr725	0
Naa35	0	Olfr71	0	Rpl7a	0	Olfr723	0
Naa30	0	Olfr706	0	Mcm3	0	Olfr720	0
Naa25	0	Olfr705	0	Mcm2	0	Olfr715	0
Naa20	0	Olfr703	0	1110034G24Rik	0	Olfr714	0
Naa16	0	Olfr702	0	Fadd	0	Olfr713	0
N6amt2	0	Olfr701	0	Il22ra1	0	Olfr711	0
N6amt1	0	Olfr700	0	Cox7b	0	Olfr710	0
N4bp3	0	Olfr70	0	Cox7c	0	Olfr71	0
N4bp2l2	0	Olfr699	0	Mcm9	0	Olfr707	0
N4bp2	0	Olfr695	0	Mcm8	0	Olfr706	0
N4bp1	0	Olfr694	0	Nxt2	0	Olfr705	0
Mzt2	0	Olfr691	0	Cecr2	0	Olfr703	0
Mzt1	0	Olfr690	0	Cecr5	0	Olfr702	0
Mzb1	0	Olfr69	0	Cecr6	0	Olfr701	0
Myzap	0	Olfr689	0	BRDN0000737936	0	Olfr700	0
Myt1l	0	Olfr686	0	Nlrp4b	0	Olfr698	0
Myt1	0	Olfr681	0	Nlrp4c	0	Olfr697	0
Myrip	0	Olfr679	0	Nlrp4a	0	Olfr694	0
Myrfl	0	Olfr678	0	Clgn	0	Olfr693	0
Myrf	0	Olfr677	0	Nlrp4e	0	Olfr692	0
Mypop	0	Olfr672	0	Acvr2b	0	Olfr691	0
Mypn	0	Olfr671	0	Spry2	0	Olfr69	0
Myoz2	0	Olfr670	0	Mrps14	0	Olfr689	0
Myoz1	0	Olfr669	0	Zfp874a	0	Olfr68	0
Myot	0	Olfr668	0	Zfp874b	0	Olfr679	0
Myom3	0	Olfr667	0	Spry4	0	Olfr678	0
Myom2	0	Olfr665	0	Tmem30a	0	Olfr676	0
Myom1	0	Olfr661	0	Tmem30c	0	Olfr675	0
Myog	0	Olfr659	0	Tmem30b	0	Olfr672	0
Myocd	0	Olfr658	0	Lman2l	0	Olfr671	0
Myoc	0	Olfr657	0	Pnmt	0	Olfr670	0
Myo9b	0	Olfr656	0	Kcnn2	0	Olfr668	0
Myo9a	0	Olfr655	0	Ercc8	0	Olfr667	0
Myo7b	0	Olfr654	0	Dalrd3	0	Olfr665	0
Myo7a	0	Olfr653	0	Hdgfrp3	0	Olfr661	0
Myo5c	0	Olfr652	0	Rnf208	0	Olfr66	0
Myo5b	0	Olfr651	0	BRDN0000737608	0	Olfr658	0
Myo1h	0	Olfr649	0	Gabrg3	0	Olfr657	0

Myo1g	0	Olfr648	0	Gabrg2	0	Olfr656	0
Myo1f	0	Olfr646	0	Gabrg1	0	Olfr655	0
Myo1e	0	Olfr645	0	BRDN0000737930	0	Olfr654	0
Myo1d	0	Olfr644	0	Spin4	0	Olfr653	0
Myo1b	0	Olfr643	0	0610010F05Rik	0	Olfr651	0
Myo1a	0	Olfr642	0	Ssx9	0	Olfr65	0
Myo19	0	Olfr641	0	Fam107a	0	Olfr649	0
Myo18a	0	Olfr640	0	Stx1b	0	Olfr648	0
Myo16	0	Olfr64	0	Rnf207	0	Olfr646	0
Myo15	0	Olfr639	0	BRDN0000737761	0	Olfr645	0
Mynn	0	Olfr638	0	Dlx6	0	Olfr644	0
Mylpf	0	Olfr635	0	4933411K16Rik	0	Olfr643	0
Mylk4	0	Olfr633	0	Mepce	0	Olfr642	0
Mylk2	0	Olfr632	0	Pax4	0	Olfr641	0
Mylip	0	Olfr631	0	Olfr307	0	Olfr640	0
Myl9	0	Olfr630	0	Crot	0	Olfr64	0
Myl7	0	Olfr63	0	Mei4	0	Olfr638	0
Myl6b	0	Olfr628	0	Klra17	0	Olfr635	0
Myl6	0	Olfr623	0	Fam222a	0	Olfr633	0
Myl4	0	Olfr622	0	Rnf114	0	Olfr631	0
Myl3	0	Olfr620	0	Rnf115	0	Olfr630	0
Myl10	0	Olfr62	0	Rnf112	0	Olfr629	0
Myl1	0	Olfr619	0	BRDN0000737939	0	Olfr628	0
Myh9	0	Olfr617	0	Mdga2	0	Olfr624	0
Myh8	0	Olfr616	0	Cfap70	0	Olfr623	0
Myh7	0	Olfr615	0	Foxn4	0	Olfr622	0
Myh6	0	Olfr611	0	Pax1	0	Olfr618	0
Myh4	0	Olfr606	0	Upk2	0	Olfr616	0
Myh3	0	Olfr603	0	Krt82	0	Olfr615	0
Myh2	0	Olfr600	0	Foxn2	0	Olfr612	0
Myh15	0	Olfr60	0	Foxn3	0	Olfr611	0
Myh14	0	Olfr6	0	Vps29	0	Olfr610	0
Myh13	0	Olfr599	0	Gfer	0	Olfr61	0
Myh11	0	Olfr598	0	Ercc3	0	Olfr606	0
Myg1	0	Olfr593	0	Chordc1	0	Olfr605	0
Myf5	0	Olfr591	0	Olfr530	0	Olfr600	0
Myeov2	0	Olfr586	0	Olfr531	0	Olfr60	0
Myef2	0	Olfr585	0	Olfr532	0	Olfr6	0
Myd88	0	Olfr584	0	Olfr533	0	Olfr599	0
Myct1	0	Olfr583	0	Olfr535	0	Olfr597	0
Mycbpap	0	Olfr582	0	Olfr536	0	Olfr596	0
Mycbp2	0	Olfr578	0	Paxip1	0	Olfr593	0
Mycbp	0	Olfr576	0	Dynlt1a	0	Olfr591	0
Myc	0	Olfr575	0	Fbxl13	0	Olfr589	0
Mybphl	0	Olfr574	0	Fbxl12	0	Olfr586	0
Mybph	0	Olfr572	0	Rap2b	0	Olfr585	0
Mybpc2	0	Olfr57	0	Ercc6	0	Olfr584	0
Mybpc1	0	Olfr569	0	Fbxl17	0	Olfr582	0
Mybl2	0	Olfr568	0	Fbxl16	0	Olfr58	0
Mybl1	0	Olfr564	0	Fbxl19	0	Olfr578	0
Mybbp1a	0	Olfr561	0	Reg3g	0	Olfr577	0
Myb	0	Olfr56	0	Reg3d	0	Olfr575	0
Myadml2	0	Olfr559	0	Letm2	0	Olfr574	0
Myadm	0	Olfr558	0	D11Wsu47e	0	Olfr572	0
Mxra8	0	Olfr556	0	Reg3a	0	Olfr571	0
Mxra7	0	Olfr553	0	Zfp286	0	Olfr570	0
Mxi1	0	Olfr552	0	Zfp287	0	Olfr57	0
Mxd4	0	Olfr551	0	Zfp281	0	Olfr566	0
Mxd3	0	Olfr550	0	Zfp282	0	Olfr564	0
Mvk	0	Olfr55	0	Pax9	0	Olfr561	0
Mvd	0	Olfr549	0	Gm9513	0	Olfr558	0
Mvb12b	0	Olfr547	0	Katnb1	0	Olfr557	0
Mutyh	0	Olfr544	0	Nup35	0	Olfr554	0
Mut	0	Olfr543	0	Nup37	0	Olfr550	0
Mustn1	0	Olfr54	0	Ndfip2	0	Olfr55	0
Musk	0	Olfr539	0	Znhit3	0	Olfr549	0
Mus81	0	Olfr538	0	Igsf23	0	Olfr547	0
Murc	0	Olfr536	0	Igsf21	0	Olfr545	0
Mup9	0	Olfr533	0	Gorab	0	Olfr544	0
Mup8	0	Olfr532	0	Nufip1	0	Olfr539	0
Mup6	0	Olfr531	0	BRDN0000737937	0	Olfr533	0
Mup5	0	Olfr530	0	Slfnl1	0	Olfr532	0
Mup3	0	Olfr527	0	Lce1f	0	Olfr530	0
Mup21	0	Olfr52	0	Nit2	0	Olfr53	0
Mup2	0	Olfr517	0	Nit1	0	Olfr525	0
Mup19	0	Olfr516	0	Ptk2	0	Olfr524	0
Mup16	0	Olfr513	0	Lce1b	0	Olfr523	0
Mup14	0	Olfr510	0	Lce1c	0	Olfr522	0
Mup10	0	Olfr51	0	Ptk7	0	Olfr520	0
Mup1	0	Olfr509	0	Ptk6	0	Olfr519	0
Mum1	0	Olfr506	0	Glod5	0	Olfr518	0
Mul1	0	Olfr504	0	Glod4	0	Olfr517	0

Mug2	0	Olfr503	0	Lce1l	0	Olfr516	0
Mug1	0	Olfr50	0	Lce1m	0	Olfr514	0
Muc5b	0	Olfr5	0	Lce1j	0	Olfr512	0
Muc5ac	0	Olfr495	0	1700029P11Rik	0	Olfr51	0
Muc4	0	Olfr494	0	Lce1h	0	Olfr509	0
Muc20	0	Olfr491	0	Lce1i	0	Olfr507	0
Muc15	0	Olfr490	0	Stx18	0	Olfr504	0
Muc13	0	Olfr49	0	Olfr1102	0	Olfr50	0
Mtx3	0	Olfr488	0	Pygb	0	Olfr5	0
Mtx2	0	Olfr486	0	Olfr1100	0	Olfr498	0
Mtus1	0	Olfr484	0	Olfr1107	0	Olfr497	0
Mttp	0	Olfr482	0	Olfr1106	0	Olfr495	0
Mtss1l	0	Olfr48	0	Olfr1105	0	Olfr494	0
Mtss1	0	Olfr478	0	Olfr1104	0	Olfr491	0
Mtrr	0	Olfr477	0	5730455P16Rik	0	Olfr490	0
Mtrf1l	0	Olfr474	0	Olfr1109	0	Olfr49	0
Mtrf1	0	Olfr47	0	Pygl	0	Olfr488	0
Mtpap	0	Olfr469	0	Odc1	0	Olfr486	0
Mtor	0	Olfr467	0	Gp2	0	Olfr481	0
Mto1	0	Olfr464	0	Susd4	0	Olfr480	0
Mtnr1b	0	Olfr463	0	Gbas	0	Olfr48	0
Mtmr6	0	Olfr462	0	Zfml	0	Olfr478	0
Mtmr3	0	Olfr461	0	Dhx40	0	Olfr477	0
Mtmr2	0	Olfr460	0	Oxct2b	0	Olfr476	0
Mtmr14	0	Olfr459	0	Mybpc3	0	Olfr474	0
Mtmr12	0	Olfr458	0	Mybpc2	0	Olfr472	0
Mtmr10	0	Olfr457	0	Mybpc1	0	Olfr47	0
Mtmr1	0	Olfr455	0	Gramd4	0	Olfr467	0
Mtm1	0	Olfr453	0	Irx2	0	Olfr466	0
Mtl5	0	Olfr450	0	Pfkfb3	0	Olfr462	0
Mthfsd	0	Olfr448	0	BRDN0000738366	0	Olfr461	0
Mthfs	0	Olfr446	0	Gabra1	0	Olfr460	0
Mthfr	0	Olfr444	0	Pdap1	0	Olfr46	0
Mthfd2l	0	Olfr44	0	1700101E01Rik	0	Olfr459	0
Mthfd2	0	Olfr437	0	Lonrf3	0	Olfr458	0
Mthfd1l	0	Olfr435	0	Lonrf2	0	Olfr457	0
Mthfd1	0	Olfr430	0	Ext1	0	Olfr455	0
Mtg2	0	Olfr429	0	Dgat1	0	Olfr452	0
Mtg1	0	Olfr427	0	Dgat2	0	Olfr450	0
Mtfr2	0	Olfr424	0	Madd	0	Olfr45	0
Mtfr1l	0	Olfr420	0	Bahd1	0	Olfr449	0
Mtfp1	0	Olfr418	0	Spen	0	Olfr448	0
Mtfmt	0	Olfr412	0	BRDN0000738150	0	Olfr447	0
Mtf2	0	Olfr411	0	Mmd2	0	Olfr446	0
Mtf1	0	Olfr406	0	Ucp2	0	Olfr441	0
Mterf4	0	Olfr403	0	Sbk1	0	Olfr437	0
Mterf3	0	Olfr402	0	Mdp1	0	Olfr435	0
Mterf2	0	Olfr401	0	Adcy1	0	Olfr434	0
Mterf1b	0	Olfr398	0	Gsdma2	0	Olfr433	0
Mterf1a	0	Olfr394	0	1700018B08Rik	0	Olfr432	0
Mtdh	0	Olfr392	0	Il20rb	0	Olfr430	0
Mtcl1	0	Olfr390	0	Mat2a	0	Olfr43	0
Mtch2	0	Olfr39	0	Ormdl3	0	Olfr429	0
Mtch1	0	Olfr389	0	Ormdl1	0	Olfr424	0
Mtbp	0	Olfr385	0	Gemin2	0	Olfr417	0
Mtap7d3	0	Olfr384	0	Adcy3	0	Olfr414	0
Mtap	0	Olfr381	0	Gemin6	0	Olfr412	0
Mta3	0	Olfr38	0	Gemin7	0	Olfr411	0
Mt2	0	Olfr376	0	Gemin4	0	Olfr410	0
Mt1	0	Olfr374	0	Gemin5	0	Olfr406	0
Msx3	0	Olfr373	0	Grin2b	0	Olfr403	0
Msx2	0	Olfr371	0	Sppl3	0	Olfr398	0
Msx1	0	Olfr370	0	2310034C09Rik	0	Olfr395	0
Msto1	0	Olfr366	0	Grin2a	0	Olfr393	0
Mst1r	0	Olfr365	0	Pbdc1	0	Olfr392	0
Mst1	0	Olfr362	0	Grin2d	0	Olfr390	0
Mss51	0	Olfr361	0	Spsb2	0	Olfr389	0
Msrb3	0	Olfr360	0	Spsb1	0	Olfr385	0
Msrb2	0	Olfr358	0	Sptssa	0	Olfr384	0
Msrb1	0	Olfr357	0	Ctxn1	0	Olfr382	0
Msra	0	Olfr356	0	Cmss1	0	Olfr381	0
Msr1	0	Olfr355	0	Ctsh	0	Olfr380	0
Msmo1	0	Olfr353	0	Olfr958	0	Olfr38	0
Mslnl	0	Olfr352	0	Olfr959	0	Olfr373	0
Msln	0	Olfr351	0	Phf10	0	Olfr370	0
Msl3l2	0	Olfr350	0	Ccno	0	Olfr368	0
Msi1	0	Olfr347	0	Phf12	0	Olfr366	0
Msh6	0	Olfr345	0	Olfr952	0	Olfr365	0
Msh5	0	Olfr342	0	Cpd	0	Olfr362	0
Msh4	0	Olfr339	0	Olfr951	0	Olfr361	0
Msh3	0	Olfr332	0	Tmem120b	0	Olfr360	0
Msh2	0	Olfr331	0	Phf19	0	Olfr358	0

Msgn1	0	Olfr330	0	Olfr954	0	Olfr357	0
Msc	0	Olfr328	0	Msra	0	Olfr355	0
Msantd3	0	Olfr325	0	Mospd3	0	Olfr354	0
Msantd2	0	Olfr324	0	Mospd2	0	Olfr352	0
Msantd1	0	Olfr323	0	Mospd1	0	Olfr351	0
Ms4a8a	0	Olfr320	0	4933425L06Rik	0	Olfr350	0
Ms4a6b	0	Olfr32	0	Setbp1	0	Olfr348	0
Ms4a5	0	Olfr319	0	Grid1	0	Olfr347	0
Ms4a4d	0	Olfr318	0	Csrp2	0	Olfr345	0
Ms4a4b	0	Olfr317	0	Cdkn2a	0	Olfr344	0
Ms4a3	0	Olfr316	0	Prss42	0	Olfr342	0
Ms4a2	0	Olfr313	0	BRDN0000738133	0	Olfr341	0
Ms4a18	0	Olfr312	0	Defb8	0	Olfr340	0
Ms4a15	0	Olfr311	0	Defb9	0	Olfr339	0
Ms4a13	0	Olfr31	0	Krtcap3	0	Olfr338	0
Ms4a10	0	Olfr309	0	Defb2	0	Olfr332	0
Ms4a1	0	Olfr308	0	Defb3	0	Olfr331	0
Mrto4	0	Olfr307	0	Defb1	0	Olfr330	0
Mrs2	0	Olfr303	0	Defb6	0	Olfr324	0
Mrrf	0	Olfr3	0	Defb7	0	Olfr323	0
Mrps9	0	Olfr299	0	Defb4	0	Olfr320	0
Mrps6	0	Olfr297	0	Fkbp4	0	Olfr32	0
Mrps5	0	Olfr295	0	Fkbp5	0	Olfr319	0
Mrps36	0	Olfr294	0	Fkbp6	0	Olfr318	0
Mrps35	0	Olfr292	0	Fkbp7	0	Olfr315	0
Mrps34	0	Olfr291	0	Lurap1	0	Olfr314	0
Mrps31	0	Olfr290	0	Acadsb	0	Olfr312	0
Mrps30	0	Olfr284	0	Fkbp3	0	Olfr311	0
Mrps28	0	Olfr279	0	Nrde2	0	Olfr310	0
Mrps27	0	Olfr272	0	Prss43	0	Olfr31	0
Mrps25	0	Olfr270	0	Fkbp8	0	Olfr308	0
Mrps24	0	Olfr27	0	Fkbp9	0	Olfr305	0
Mrps22	0	Olfr267	0	Kank3	0	Olfr304	0
Mrps18b	0	Olfr266	0	Ssc4d	0	Olfr303	0
Mrps18a	0	Olfr262	0	Tmem41b	0	Olfr301	0
Mrps16	0	Olfr251	0	Podxl2	0	Olfr30	0
Mrps15	0	Olfr248	0	Wasf1	0	Olfr299	0
Mrps14	0	Olfr247	0	Wasf2	0	Olfr297	0
Mrps12	0	Olfr24	0	Msgn1	0	Olfr295	0
Mrps11	0	Olfr239	0	BRDN0000738154	0	Olfr293	0
Mrps10	0	Olfr237-ps1	0	Kank1	0	Olfr292	0
Mrpl9	0	Olfr235	0	Rsc1a1	0	Olfr288	0
Mrpl57	0	Olfr231	0	Srgap3	0	Olfr287	0
Mrpl55	0	Olfr229	0	Kidins220	0	Olfr284	0
Mrpl54	0	Olfr228	0	Psat1	0	Olfr283	0
Mrpl53	0	Olfr225	0	BC100451	0	Olfr273	0
Mrpl49	0	Olfr224	0	Ctsf	0	Olfr270	0
Mrpl48	0	Olfr221	0	Pbrm1	0	Olfr266	0
Mrpl47	0	Olfr220	0	Taf9b	0	Olfr263	0
Mrpl46	0	Olfr218	0	Pdhb	0	Olfr262	0
Mrpl45	0	Olfr215	0	Ctsg	0	Olfr259	0
Mrpl44	0	Olfr213	0	Gm11758	0	Olfr251	0
Mrpl43	0	Olfr212	0	Gm17296	0	Olfr250	0
Mrpl42	0	Olfr211	0	Nudt19	0	Olfr24	0
Mrpl41	0	Olfr209	0	Myog	0	Olfr239	0
Mrpl40	0	Olfr206	0	Lancl3	0	Olfr237-ps1	0
Mrpl4	0	Olfr205	0	Galnt5	0	Olfr235	0
Mrpl39	0	Olfr204	0	Atp8b2	0	Olfr231	0
Mrpl38	0	Olfr201	0	BRDN0000738034	0	Olfr23	0
Mrpl37	0	Olfr199	0	Xcr1	0	Olfr229	0
Mrpl36	0	Olfr198	0	Unc13b	0	Olfr228	0
Mrpl35	0	Olfr196	0	Mid2	0	Olfr223	0
Mrpl33	0	Olfr195	0	Prss23	0	Olfr222	0
Mrpl32	0	Olfr194	0	Nipbl	0	Olfr221	0
Mrpl30	0	Olfr193	0	Prkcsh	0	Olfr220	0
Mrpl3	0	Olfr191	0	Cdkn2d	0	Olfr218	0
Mrpl28	0	Olfr19	0	Hao1	0	Olfr215	0
Mrpl27	0	Olfr186	0	Hao2	0	Olfr214	0
Mrpl23	0	Olfr183	0	C7	0	Olfr213	0
Mrpl22	0	Olfr181	0	Rtca	0	Olfr211	0
Mrpl20	0	Olfr180	0	Kcnk13	0	Olfr209	0
Mrpl2	0	Olfr178	0	Cchcr1	0	Olfr206	0
Mrpl19	0	Olfr177	0	BRDN0000738156	0	Olfr205	0
Mrpl18	0	Olfr175-ps1	0	Mcam	0	Olfr204	0
Mrpl17	0	Olfr173	0	Trappc5	0	Olfr203	0
Mrpl16	0	Olfr171	0	Unc13c	0	Olfr201	0
Mrpl15	0	Olfr170	0	Ciart	0	Olfr198	0
Mrpl14	0	Olfr17	0	Trappc4	0	Olfr196	0
Mrpl13	0	Olfr169	0	Gpr171	0	Olfr194	0
Mrpl12	0	Olfr168	0	Ap1s1	0	Olfr191	0
Mrpl11	0	Olfr166	0	Ap1s2	0	Olfr190	0
Mrpl10	0	Olfr165	0	Ap1s3	0	Olfr19	0

Mrpl1	0
Mroh9	0
Mroh7	0
Mroh6	0
Mroh4	0
Mroh2b	0
Mroh2a	0
Mri1	0
Mrgprx2	0
Mrgprh	0
Mrgprg	0
Mrgprf	0
Mrgpre	0
Mrgprd	0
Mrgprb8	0
Mrgprb5	0
Mrgprb4	0
Mrgprb3	0
Mrgprb2	0
Mrgprb1	0
Mrgpra4	0
Mrgpra3	0
Mrgpra2b	0
Mrgpra2a	0
Mrgpra1	0
Mrgbp	0
Mrfap1	0
Mreg	0
Mre11a	0
Mrc2	0
Mrc1	0
Mras	0
Mrap2	0
Mrap	0
Mr1	0
Mpzl3	0
Mpzl2	0
Mpzl1	0
Mpz	0
Mpv17l	0
Mpv17	0
Mptx2	0
Mptx1	0
Mprip	0
Mpped2	0
Mpped1	0
Mpp6	0
Mpp5	0
Mpp4	0
Mpp3	0
Mpp2	0
Mpo	0
Mpnd	0
Mplkip	0
Mpl	0
Mpi	0
Mphosph9	0
Mphosph8	0
Mphosph6	0
Mphosph10	0
Mpg	0
Mpeg1	0
Mpdu1	0
Mpc2	0
Mpc1	0
Moxd2	0
Moxd1	0
Mov10l1	0
Mov10	0
Mospd3	0
Mospd1	0
Morn4	0
Morn3	0
Morn2	0
Morn1	0
Morc4	0
Morc3	0
Morc2b	0
Morc1	0
Mon2	0
Mon1b	0
Mon1a	0
Olfr164	0
Olfr16	0
Olfr159	0
Olfr157	0
Olfr156	0
Olfr155	0
Olfr154	0
Olfr1537	0
Olfr1535	0
Olfr1532-ps1	0
Olfr152	0
Olfr1513	0
Olfr1512	0
Olfr1510	0
Olfr1506	0
Olfr1504	0
Olfr150	0
Olfr15	0
Olfr1499	0
Olfr1497	0
Olfr1496	0
Olfr1491	0
Olfr1490	0
Olfr149	0
Olfr1489	0
Olfr1487	0
Olfr1480	0
Olfr1477	0
Olfr1475	0
Olfr1472	0
Olfr1471	0
Olfr1469	0
Olfr1467	0
Olfr1466	0
Olfr1462	0
Olfr146	0
Olfr1459	0
Olfr1457	0
Olfr1454	0
Olfr1450	0
Olfr145	0
Olfr1449	0
Olfr1447	0
Olfr1444	0
Olfr1442	0
Olfr1441	0
Olfr1434	0
Olfr1431	0
Olfr143	0
Olfr1428	0
Olfr1427	0
Olfr1425	0
Olfr1424	0
Olfr1420	0
Olfr1419	0
Olfr1417	0
Olfr1416	0
Olfr1415	0
Olfr1414	0
Olfr1413	0
Olfr1411	0
Olfr141	0
Olfr1406	0
Olfr140	0
Olfr1395	0
Olfr1394	0
Olfr1393	0
Olfr1392	0
Olfr1390	0
Olfr1388	0
Olfr1386	0
Olfr1385	0
Olfr1384	0
Olfr1383	0
Olfr1381	0
Olfr1380	0
Olfr138	0
Olfr1377	0
Olfr1373	0
Olfr1371	0
Olfr1370	0
Olfr1368	0
Dmgdh	0
Uty	0
Gpr173	0
Capn7	0
Evi2b	0
Hepacam2	0
Slc7a9	0
Htr1a	0
Ftl1	0
Tmc5	0
Fgfbp3	0
Fgfbp1	0
Slc7a7	0
Cs	0
Btg1-ps1	0
Grm3	0
Adgrb3	0
Slc7a4	0
Capn2	0
Krtap5-4	0
Nop9	0
9230110C19Rik	0
Krtap5-1	0
Krtap5-2	0
Unc13a	0
Lbx2	0
Dzip1l	0
Tm9sf2	0
Cenpm	0
Kctd13	0
Cdca2	0
Cdca3	0
Zfp516	0
Gm13128	0
Cdca7	0
Cdca4	0
Zfp513	0
Gm13124	0
Gm13125	0
Cdca8	0
Anapc13	0
Rrbp1	0
Cyp26c1	0
BRDN0000738159	0
BRDN0000738038	0
Krba1	0
Pemt	0
Tcf4	0
Twist1	0
Fzr1	0
Wnt5a	0
AU022252	0
Cd4	0
Cd5	0
Svop	0
Capn9	0
Cd2	0
Epha8	0
Sall1	0
Cadm1	0
Cd9	0
Chrna2	0
Med28	0
Mrm1	0
Stx17	0
Cep128	0
Rassf1	0
Atad1	0
Med23	0
Rassf2	0
Nras	0
Rassf4	0
Med27	0
Med26	0
Stx19	0
Olfr1377	0
Dnm1	0
Prrt3	0
Dnm3	0
Dnm2	0
Prrt2	0
Cyb561d2	0
Olfr187	0
Olfr181	0
Olfr180	0
Olfr18	0
Olfr178	0
Olfr177	0
Olfr175-ps1	0
Olfr173	0
Olfr172	0
Olfr170	0
Olfr168	0
Olfr167	0
Olfr166	0
Olfr165	0
Olfr161	0
Olfr160	0
Olfr16	0
Olfr156	0
Olfr154	0
Olfr1537	0
Olfr1535	0
Olfr1532-ps1	0
Olfr153	0
Olfr152	0
Olfr1513	0
Olfr1512	0
Olfr1511	0
Olfr1509	0
Olfr1506	0
Olfr1504	0
Olfr1501	0
Olfr150	0
Olfr15	0
Olfr1499	0
Olfr1497	0
Olfr1494	0
Olfr149	0
Olfr1480	0
Olfr148	0
Olfr1472	0
Olfr147	0
Olfr1467	0
Olfr1466	0
Olfr1463	0
Olfr1461	0
Olfr146	0
Olfr1451	0
Olfr1450	0
Olfr1449	0
Olfr1447	0
Olfr1444	0
Olfr1443	0
Olfr1442	0
Olfr1441	0
Olfr1440	0
Olfr1437	0
Olfr1436	0
Olfr1434	0
Olfr1431	0
Olfr1428	0
Olfr1427	0
Olfr1426	0
Olfr1425	0
Olfr1424	0
Olfr1423	0
Olfr1420	0
Olfr142	0
Olfr1418	0
Olfr1417	0
Olfr1414	0
Olfr1410	0
Olfr141	0
Olfr1408	0
Olfr1406	0
Olfr1402	0
Olfr140	0
Olfr1395	0
Olfr1394	0
Olfr1393	0
Olfr1392	0
Olfr1390	0
Olfr139	0

Mok	0	Olfr1367	0	Gba2	0	Olfr1389	0
Mogs	0	Olfr1366	0	Spag17	0	Olfr1386	0
Mogat2	0	Olfr1364	0	Sphk2	0	Olfr1385	0
Mogat1	0	Olfr1362	0	Sphk1	0	Olfr1384	0
Mog	0	Olfr1361	0	Tsku	0	Olfr1381	0
Mocs3	0	Olfr1360	0	Cda	0	Olfr1380	0
Mocs1	0	Olfr136	0	Gm4070	0	Olfr138	0
Mocos	0	Olfr1357	0	BRDN0000738054	0	Olfr1378	0
Mobp	0	Olfr1356	0	Tuba3b	0	Olfr1377	0
Mob4	0	Olfr1355	0	Iqcb1	0	Olfr1373	0
Mob3b	0	Olfr1353	0	Bfsp2	0	Olfr1371	0
Mob3a	0	Olfr1352	0	Grtp1	0	Olfr1367	0
Mob2	0	Olfr1351	0	T	0	Olfr1366	0
Mob1b	0	Olfr1350	0	Bfsp1	0	Olfr1364	0
Mob1a	0	Olfr135	0	Dvl3	0	Olfr1362	0
Moap1	0	Olfr1348	0	Rev3l	0	Olfr1360	0
Mnx1	0	Olfr1347	0	Pde6b	0	Olfr136	0
Mnt	0	Olfr1344	0	Spesp1	0	Olfr1357	0
Mns1	0	Olfr1342	0	Leprot	0	Olfr1356	0
Mndal	0	Olfr1341	0	Uchl4	0	Olfr1354	0
Mnd1	0	Olfr1340	0	Klhdc10	0	Olfr1353	0
Mnat1	0	Olfr134	0	Uchl3	0	Olfr1352	0
Mn1	0	Olfr1339	0	Wfdc11	0	Olfr1351	0
Mms22l	0	Olfr1338	0	Wfdc10	0	Olfr135	0
Mms19	0	Olfr1337	0	Wfdc13	0	Olfr1349	0
Mmrn2	0	Olfr1336	0	BRDN0000738008	0	Olfr1347	0
Mmrn1	0	Olfr1335	0	Toporsl	0	Olfr1344	0
Mmp8	0	Olfr1333	0	Fam171b	0	Olfr1342	0
Mmp7	0	Olfr1330	0	Tcaf2	0	Olfr1341	0
Mmp3	0	Olfr133	0	Lrrc36	0	Olfr1340	0
Mmp27	0	Olfr1329	0	Wfdc18	0	Olfr134	0
Mmp25	0	Olfr1328	0	Meaf6	0	Olfr1339	0
Mmp23	0	Olfr1324	0	Vopp1	0	Olfr1337	0
Mmp21	0	Olfr1321	0	Aplp2	0	Olfr1336	0
Mmp20	0	Olfr132	0	Dpp10	0	Olfr1333	0
Mmp2	0	Olfr1318	0	2610020H08Rik	0	Olfr1330	0
Mmp1b	0	Olfr1316	0	Coro2b	0	Olfr133	0
Mmp1a	0	Olfr1312	0	Tmbim7	0	Olfr1328	0
Mmp19	0	Olfr1311	0	Tmbim6	0	Olfr1325	0
Mmp17	0	Olfr131	0	Tmbim4	0	Olfr1324	0
Mmp16	0	Olfr1309	0	Mapk8	0	Olfr1322	0
Mmp15	0	Olfr1308	0	Tmbim1	0	Olfr132	0
Mmp13	0	Olfr1305	0	Rhox4e	0	Olfr1318	0
Mmp11	0	Olfr1303	0	Rhox4g	0	Olfr1317	0
Mmp10	0	Olfr1302	0	Gabra6	0	Olfr1316	0
Mmgt2	0	Olfr1301	0	Rhox4a	0	Olfr1312	0
Mmgt1	0	Olfr1299	0	Prss50	0	Olfr1310	0
Mmd2	0	Olfr1297	0	Prss53	0	Olfr131	0
Mmd	0	Olfr1295	0	Rhox4b	0	Olfr1309	0
Mmadhc	0	Olfr1294	0	Olfr325	0	Olfr1308	0
Mmachc	0	Olfr1289	0	Olfr324	0	Olfr1306	0
Mmab	0	Olfr1286	0	D230025D16Rik	0	Olfr1305	0
Mmaa	0	Olfr1284	0	Olfr320	0	Olfr1303	0
Mlycd	0	Olfr1283	0	Olfr323	0	Olfr1302	0
Mlxipl	0	Olfr1282	0	Olfr322	0	Olfr130	0
Mlxip	0	Olfr1281	0	Commd10	0	Olfr13	0
Mlx	0	Olfr1280	0	Slc17a4	0	Olfr1299	0
Mllt6	0	Olfr128	0	Tvp23b	0	Olfr1297	0
Mllt4	0	Olfr1279	0	Gm128	0	Olfr1295	0
Mllt10	0	Olfr1278	0	Ddit4l	0	Olfr1294	0
Mllt1	0	Olfr1277	0	Ckap5	0	Olfr1290	0
Mlh1	0	Olfr1276	0	Pef1	0	Olfr1289	0
Mlf2	0	Olfr1275	0	Ndufab1	0	Olfr1288	0
Mlf1	0	Olfr1271	0	Nudt8	0	Olfr1283	0
Mlec	0	Olfr127	0	BRDN0000738015	0	Olfr1282	0
Mlc1	0	Olfr1269	0	1700015F17Rik	0	Olfr128	0
Mlana	0	Olfr1265	0	Fam71a	0	Olfr1279	0
Mkx	0	Olfr1264	0	Pp2d1	0	Olfr1278	0
Mks1	0	Olfr1262	0	Gcn1l1	0	Olfr1273-ps	0
Mkrn3	0	Olfr1260	0	Scand1	0	Olfr1271	0
Mkrn2os	0	Olfr126	0	Ist1	0	Olfr127	0
Mkrn1	0	Olfr1259	0	Caskin2	0	Olfr1269	0
Mknk2	0	Olfr1258	0	Ces4a	0	Olfr1265	0
Mknk1	0	Olfr1257	0	Crim1	0	Olfr1264	0
Mkln1	0	Olfr1256	0	Prl7b1	0	Olfr1262	0
Mkl1	0	Olfr1255	0	Hrct1	0	Olfr1261	0
Mkks	0	Olfr1254	0	Zmpste24	0	Olfr1260	0
Mki67	0	Olfr1253	0	Rcc1	0	Olfr126	0
Mixl1	0	Olfr1251	0	Rcc2	0	Olfr1259	0
Mitf	0	Olfr1250	0	Sssca1	0	Olfr1258	0
Mitd1	0	Olfr125	0	Tirap	0	Olfr1257	0
Misp	0	Olfr1249	0	Atp5s	0	Olfr1255	0

Mis18bp1	0	Olfr1248	0	Eml3	0	Olfr1254	0
Mis18a	0	Olfr1246	0	Olfr183	0	Olfr1252	0
Mis12	0	Olfr1245	0	4930590J08Rik	0	Olfr1251	0
Mipol1	0	Olfr1243	0	Eml6	0	Olfr125	0
Mipep	0	Olfr1242	0	Eml4	0	Olfr1249	0
Mip	0	Olfr124	0	Eml5	0	Olfr1248	0
Miox	0	Olfr1239	0	Afg3l1	0	Olfr1247	0
Minpp1	0	Olfr1238	0	Atp5j	0	Olfr1246	0
Minos1	0	Olfr1234	0	Arc	0	Olfr1245	0
Mink1	0	Olfr1233	0	Nphs2	0	Olfr1243	0
Milr1	0	Olfr1232	0	Tmem247	0	Olfr1241	0
Mill1	0	Olfr1231	0	Nphs1	0	Olfr1238	0
Miip	0	Olfr123	0	Atp5b	0	Olfr1234	0
Mif4gd	0	Olfr1226	0	Pus10	0	Olfr1233	0
Mif	0	Olfr1222	0	Gjd4	0	Olfr1231	0
Mier3	0	Olfr122	0	Gjd3	0	Olfr1228	0
Mier2	0	Olfr1219	0	Nudt2	0	Olfr1226	0
Mier1	0	Olfr1218	0	Atp5e	0	Olfr1225	0
Mief2	0	Olfr1217	0	Atp5d	0	Olfr1223	0
Mief1	0	Olfr1216	0	0610010B08Rik	0	Olfr1222	0
Mid2	0	Olfr1215	0	Nup62cl	0	Olfr1220	0
Mid1ip1	0	Olfr1214	0	Rhobtb1	0	Olfr122	0
Mid1	0	Olfr1212	0	Nme2	0	Olfr1218	0
Micu2	0	Olfr1211	0	Cept1	0	Olfr1216	0
Micall1	0	Olfr1209	0	Txlnb	0	Olfr1215	0
Micalcl	0	Olfr1208	0	Txlna	0	Olfr1214	0
Mical3	0	Olfr1206	0	Svil	0	Olfr1212	0
Mical2	0	Olfr1205	0	Iyd	0	Olfr1211	0
Mib2	0	Olfr1204	0	BRDN0000737760	0	Olfr1208	0
Mib1	0	Olfr1200	0	AW551984	0	Olfr1205	0
Mia	0	Olfr12	0	Jak3	0	Olfr1202	0
Mgst3	0	Olfr1199	0	Slc4a4	0	Olfr1201	0
Mgst2	0	Olfr1198	0	Doc2a	0	Olfr120	0
Mgst1	0	Olfr1197	0	Trim68	0	Olfr12	0
Mgrn1	0	Olfr1193	0	Doc2b	0	Olfr1198	0
Mgmt	0	Olfr119	0	4933413G19Rik	0	Olfr1196	0
Mgll	0	Olfr1189	0	Doc2g	0	Olfr1195	0
Mgl2	0	Olfr1188	0	Trim69	0	Olfr1193	0
Mgea5	0	Olfr1186	0	Cpeb4	0	Olfr119	0
Mgat5b	0	Olfr1184	0	Cpeb1	0	Olfr1188	0
Mgat5	0	Olfr1180	0	Tjp3	0	Olfr1184	0
Mgat4e	0	Olfr118	0	Rpa3	0	Olfr1183	0
Mgat4d	0	Olfr1176	0	Cpeb2	0	Olfr1181	0
Mgat4c	0	Olfr1173	0	Dnali1	0	Olfr1180	0
Mgat4b	0	Olfr117	0	BRDN0000738139	0	Olfr118	0
Mgat4a	0	Olfr1166	0	Tmem178	0	Olfr1179	0
Mgat2	0	Olfr1163	0	Tmem179	0	Olfr1178	0
Mgat1	0	Olfr1160	0	Tmem177	0	Olfr1176	0
Mgam	0	Olfr1158	0	Proser2	0	Olfr1170	0
Mga	0	Olfr1157	0	Tmem175	0	Olfr117	0
Mfsd9	0	Olfr1156	0	Plk3	0	Olfr1168	0
Mfsd8	0	Olfr1153	0	Tmem170	0	Olfr1167	0
Mfsd7c	0	Olfr1151	0	Tmem171	0	Olfr1164	0
Mfsd7b	0	Olfr1148	0	C2cd2	0	Olfr1163	0
Mfsd6l	0	Olfr1143	0	Ift27	0	Olfr1162	0
Mfsd6	0	Olfr1141	0	Ift20	0	Olfr1161	0
Mfsd5	0	Olfr1140	0	Hand2	0	Olfr1160	0
Mfsd4	0	Olfr114	0	Ift22	0	Olfr1158	0
Mfsd2b	0	Olfr1138	0	Rap2c	0	Olfr1157	0
Mfsd12	0	Olfr1135	0	Plekhj1	0	Olfr1155	0
Mfsd11	0	Olfr1134	0	Nisch	0	Olfr1153	0
Mfsd10	0	Olfr1132	0	BRDN0000737668	0	Olfr1152	0
Mfsd1	0	Olfr1130	0	Sirt6	0	Olfr1151	0
Mfng	0	Olfr113	0	BC049730	0	Olfr1145	0
Mfn1	0	Olfr1128	0	Pdxk	0	Olfr1143	0
Mfi2	0	Olfr1126	0	BRDN0000737666	0	Olfr1140	0
Mfge8	0	Olfr1124	0	Elf1	0	Olfr1138	0
Mfap5	0	Olfr1123	0	Star	0	Olfr1137	0
Mfap4	0	Olfr1122	0	Yipf3	0	Olfr1136	0
Mfap2	0	Olfr1120	0	Elf2	0	Olfr1135	0
Mfap1b	0	Olfr1115	0	Yipf7	0	Olfr1133	0
Mfap1a	0	Olfr1113	0	2010012O05Rik	0	Olfr1132	0
Mex3d	0	Olfr1112	0	Aptx	0	Olfr1131	0
Mex3c	0	Olfr1110	0	Hprt	0	Olfr1130	0
Mex3a	0	Olfr1106	0	Hist1h1t	0	Olfr113	0
Mettl8	0	Olfr1105	0	Gm20831	0	Olfr1129	0
Mettl7b	0	Olfr1104	0	Ppt2	0	Olfr1123	0
Mettl7a3	0	Olfr1102	0	Mrpl17	0	Olfr1122	0
Mettl7a1	0	Olfr1101	0	Syde2	0	Olfr1121	0
Mettl6	0	Olfr1100	0	Ces1g	0	Olfr1120	0
Mettl5	0	Olfr110	0	Sqle	0	Olfr1115	0
Mettl4	0	Olfr11	0	Slc26a1	0	Olfr1112	0

Mettl3	0
Mettl23	0
Mettl21e	0
Mettl21c	0
Mettl21a	0
Mettl2	0
Mettl18	0
Mettl16	0
Mettl15	0
Mettl14	0
Mettl13	0
Mettl11b	0
Mettl10	0
Mettl1	0
Metrnl	0
Metrn	0
Methig1	0
Metap2	0
Metap1d	0
Metap1	0
Met	0
Mest	0
Mesp2	0
Mesp1	0
Mesdc2	0
Mesdc1	0
Mertk	0
Mepce	0
Mep1b	0
Meox2	0
Meox1	0
Men1	0
Memo1	0
Meis3	0
Meis1	0
Meiob	0
Mei4	0
Mei1	0
Megf9	0
Megf8	0
Megf6	0
Megf10	0
Mefv	0
Medag	0
Med7	0
Med4	0
Med31	0
Med30	0
Med29	0
Med28	0
Med27	0
Med26	0
Med25	0
Med24	0
Med23	0
Med22	0
Med21	0
Med20	0
Med19	0
Med18	0
Med17	0
Med15	0
Med13	0
Med12	0
Med11	0
Med10	0
Med1	0
Mecr	0
Mecp2	0
Meaf6	0
Mea1	0
Me3	0
Me2	0
Me1	0
Mdp1	0
Mdn1	0
Mdm4	0
Mdm2	0
Mdm1	0
Mdh2	0
Mdh1	0
Mdga1	0
Olfr1099	0
Olfr1098	0
Olfr1095	0
Olfr1094	0
Olfr1093	0
Olfr1090	0
Olfr109	0
Olfr1087	0
Olfr1085	0
Olfr1084	0
Olfr1082	0
Olfr1080	0
Olfr108	0
Olfr1079	0
Olfr1076	0
Olfr107	0
Olfr1066	0
Olfr1058	0
Olfr1057	0
Olfr1056	0
Olfr1052	0
Olfr1051	0
Olfr1048	0
Olfr1044	0
Olfr1042	0
Olfr1040	0
Olfr1039	0
Olfr1037	0
Olfr1036	0
Olfr1034	0
Olfr1032	0
Olfr1030	0
Olfr1029	0
Olfr1028	0
Olfr1024	0
Olfr102	0
Olfr1019	0
Olfr1018	0
Olfr1016	0
Olfr1015	0
Olfr1014	0
Olfr1013	0
Olfr1012	0
Olfr101	0
Olfr10	0
Olfr1	0
Olfml3	0
Olfml2a	0
Olfm4	0
Olfm2	0
Ola1	0
Oit3	0
Oip5	0
Ogt	0
Ogn	0
Ogg1	0
Ogfrl1	0
Ogfod2	0
Ogdh	0
Ofd1	0
Ofcc1	0
Odf4	0
Odf3l2	0
Odf3l1	0
Odf3b	0
Odf3	0
Odf2l	0
Odf1	0
Odc1	0
Odam	0
Ocstamp	0
Ocrl	0
Ociad1	0
Ocel1	0
Oca2	0
Oc90	0
Obscn	0
Obp2b	0
Obp2a	0
Obp1a	0
Obox6	0
Obox5	0
Slc26a2	0
Slc26a3	0
Slc26a4	0
Cts6	0
Krtap14	0
Krtap15	0
Hist1h1d	0
Cts7	0
Hist1h1a	0
Hist1h1b	0
Hist1h1c	0
Gm6792	0
L3hypdh	0
Rab9b	0
Lime1	0
Tecpr1	0
Gm5916	0
LOC100862015	0
Tmem25	0
Ftsj2	0
Ftsj3	0
BRDN0000737644	0
Ftsj1	0
Chac2	0
Olfr95	0
Prf1	0
Trnau1ap	0
Dad1	0
Skor1	0
Gdf9	0
Olfr854	0
Ptma	0
Olfr1318	0
Olfr1316	0
Olfr1317	0
Olfr1314	0
Zglp1	0
Olfr1312	0
Cbr3	0
Cbr2	0
Zfp873	0
Zfp871	0
Zfp870	0
Vcam1	0
Abca8a	0
Ksr1	0
Zfp879	0
Nradd	0
Agpat2	0
St3gal3	0
St3gal2	0
St3gal1	0
Exoc1	0
St3gal6	0
Exoc4	0
Exoc5	0
Exoc8	0
Rtkn2	0
Dhrs3	0
Loxhd1	0
Ccr1	0
Ccr2	0
Ccr3	0
Rrm2b	0
Ccr5	0
Ccr6	0
Glt1d1	0
Ccr8	0
Ccr9	0
Uqcrfs1	0
Nfxl1	0
1700023E05Rik	0
Prickle1	0
Gm6086	0
Prickle3	0
Mrps22	0
E130311K13Rik	0
Rilp	0
Vcan	0
Snrpa1	0
1700008I05Rik	0
Isg15	0
Olfr1111	0
Olfr1110	0
Olfr1107	0
Olfr1105	0
Olfr1104	0
Olfr1101	0
Olfr1100	0
Olfr110	0
Olfr11	0
Olfr1099	0
Olfr1098	0
Olfr1097	0
Olfr1095	0
Olfr1094	0
Olfr1093	0
Olfr1090	0
Olfr1089	0
Olfr1085	0
Olfr1084	0
Olfr108	0
Olfr1079	0
Olfr1076	0
Olfr107	0
Olfr1066	0
Olfr1065	0
Olfr1062	0
Olfr1058	0
Olfr1056	0
Olfr1054	0
Olfr1053	0
Olfr1052	0
Olfr1051	0
Olfr1049	0
Olfr1048	0
Olfr1047	0
Olfr1045	0
Olfr1043	0
Olfr1042	0
Olfr1040	0
Olfr1039	0
Olfr1037	0
Olfr1034	0
Olfr1032	0
Olfr1031	0
Olfr1030	0
Olfr1029	0
Olfr1028	0
Olfr1024	0
Olfr1023	0
Olfr1020	0
Olfr1019	0
Olfr1018	0
Olfr1016	0
Olfr1015	0
Olfr1013	0
Olfr1012	0
Olfr101	0
Olfr1009	0
Olfr1006	0
Olfr10	0
Olfr1	0
Olfm4	0
Olfm3	0
Olfm2	0
Ola1	0
Oit3	0
Oit1	0
Oip5	0
Ogt	0
Ogg1	0
Ogfrl1	0
Ogfr	0
Ogfod1	0
Ofd1	0
Ofcc1	0
Odf4	0
Odf3l2	0
Odf3l1	0
Odf3	0
Odf2l	0
Odf2	0
Odf1	0

Mdfi	0	Obox3	0	Agpat9	0	Odam	0
Mdc1	0	Obox2	0	Dsc3	0	Ocrl	0
Mcur1	0	Obox1	0	BRDN0000738297	0	Ocln	0
Mcu	0	Obfc1	0	BRDN0000737443	0	Ociad2	0
Mcts2	0	Oat	0	Rictor	0	Ociad1	0
Mcts1	0	Oasl2	0	1700008F21Rik	0	Ocel1	0
Mctp2	0	Oas3	0	Rnf152	0	Oca2	0
Mctp1	0	Oas1h	0	Ppie	0	Obp2b	0
Mcrs1	0	Oas1f	0	BRDN0000737442	0	Obp2a	0
Mcpt9	0	Oas1e	0	Krt39	0	Obox6	0
Mcpt8	0	Oas1d	0	Slc37a2	0	Obox3	0
Mcpt2	0	Oas1c	0	Slc41a3	0	Obfc1	0
Mcpt1	0	Oas1a	0	BRDN0000737441	0	Oat	0
Mcph1	0	Oard1	0	Slc37a3	0	Oasl1	0
Mcoln3	0	Oacyl	0	Metap1	0	Oas2	0
Mcoln1	0	Nyx	0	Metap2	0	Oas1h	0
Mcmdc2	0	Nynrin	0	Krt35	0	Oas1e	0
Mcmbp	0	Nyap1	0	Krt34	0	Oas1d	0
Mcm9	0	Nxt2	0	Krt36	0	Oas1c	0
Mcm8	0	Nxt1	0	1700012B09Rik	0	Oas1a	0
Mcm7	0	Nxph4	0	Lcn6	0	Nynrin	0
Mcm6	0	Nxph2	0	Gprc5c	0	Nxt1	0
Mcm5	0	Nxpe5	0	Snrpc	0	Nxph4	0
Mcm4	0	Nxpe4	0	Phox2a	0	Nxph3	0
Mcm3ap	0	Nxpe3	0	Ceacam20	0	Nxph2	0
Mcm3	0	Nxnl2	0	Smr3a	0	Nxph1	0
Mcm2	0	Nxnl1	0	Fpr-rs6	0	Nxpe5	0
Mcm10	0	Nxn	0	Gm5615	0	Nxpe3	0
Mchr1	0	Nxf7	0	Taf6	0	Nxpe2	0
Mcfd2	0	Nxf3	0	Nlgn2	0	Nxnl2	0
Mcf2l	0	Nxf2	0	Slc37a4	0	Nxnl1	0
Mcf2	0	Nxf1	0	Nlgn1	0	Nxn	0
Mcemp1	0	Nvl	0	Lcn2	0	Nxf3	0
Mcee	0	Nutf2	0	Gm6880	0	Nxf2	0
Mccc2	0	Nus1	0	3110079O15Rik	0	Nxf1	0
Mcat	0	Nupr1l	0	Wdr34	0	Nwd2	0
Mcam	0	Nupl2	0	Wdr37	0	Nwd1	0
Mc4r	0	Nupl1	0	Wdr36	0	Nvl	0
Mc3r	0	Nup98	0	Wdr31	0	Nutm2	0
Mc2r	0	Nup93	0	Pinx1	0	Nutf2	0
Mc1r	0	Nup88	0	Wdr33	0	Nus1	0
Mbtps1	0	Nup62cl	0	BRDN0000737448	0	Nupl1	0
Mbtd1	0	Nup62	0	Mrps18b	0	Nup98	0
Mbp	0	Nup50	0	Wdr38	0	Nup93	0
Mboat7	0	Nup43	0	Olfr237-ps1	0	Nup62cl	0
Mboat2	0	Nup37	0	Vwa8	0	Nup54	0
Mboat1	0	Nup214	0	Vwa9	0	Nup50	0
Mbnl2	0	Nup210l	0	Vwa7	0	Nup43	0
Mblac2	0	Nup210	0	Vwa2	0	Nup35	0
Mblac1	0	Nup205	0	Vwa1	0	Nup214	0
Mbl1	0	Nup160	0	Ralgds	0	Nup210l	0
Mbip	0	Nup155	0	Hunk	0	Nup210	0
Mbd6	0	Nup133	0	Tmem167b	0	Nup205	0
Mbd5	0	Nup107	0	Ptch2	0	Nup188	0
Mbd4	0	Numbl	0	Bcorl1	0	Nup160	0
Mbd3l1	0	Numb	0	Txndc9	0	Nup155	0
Mbd3	0	Numa1	0	Ptch1	0	Nup153	0
Mbd2	0	Nuggc	0	Yme1l1	0	Nup133	0
Mbd1	0	Nufip1	0	Cryba4	0	Nup107	0
Mb21d1	0	Nuf2	0	Gstp1	0	Numbl	0
Mb	0	Nudt8	0	Arl6ip1	0	Numb	0
Maz	0	Nudt5	0	Fam69b	0	Nufip2	0
Max	0	Nudt4	0	Hrh4	0	Nuf2	0
Mavs	0	Nudt3	0	Tmem203	0	Nudt9	0
Matr3	0	Nudt22	0	Hrh2	0	Nudt6	0
Matn4	0	Nudt21	0	Hrh3	0	Nudt5	0
Matn3	0	Nudt2	0	Padi6	0	Nudt3	0
Matn1	0	Nudt18	0	Sirt4	0	Nudt22	0
Matk	0	Nudt17	0	Hba-a1	0	Nudt21	0
Mat2b	0	Nudt16l1	0	4932418E24Rik	0	Nudt2	0
Mat2a	0	Nudt16	0	Wiz	0	Nudt18	0
Mat1a	0	Nudt15	0	Sharpin	0	Nudt17	0
Mastl	0	Nudt13	0	Cd101	0	Nudt16l1	0
Mast4	0	Nudt12	0	Cmpk1	0	Nudt16	0
Mast3	0	Nudt11	0	Xpa	0	Nudt15	0
Mast2	0	Nudcd3	0	Tmem207	0	Nudt14	0
Mast1	0	Nudcd2	0	Gm14920	0	Nudt13	0
Masp2	0	Nucks1	0	Zrsr2	0	Nudt11	0
Masp1	0	Nucb2	0	Atp6v0d1	0	Nudt10	0
Mas1	0	Nucb1	0	BRDN0000738115	0	Nudt1	0
Marveld3	0	Nubpl	0	BRDN0000737467	0	Nudcd3	0
Marveld2	0	Nubp2	0	Ten1	0	Nudcd1	0

Mars2	0
Mars	0
Mark2	0
Mark1	0
Marf1	0
Marco	0
Marcks	0
March9	0
March7	0
March6	0
March5	0
March4	0
March2	0
March11	0
Marc2	0
Mapt	0
Mapkbp1	0
Mapkapk3	0
Mapkapk2	0
Mapkap1	0
Mapk9	0
Mapk8ip3	0
Mapk8ip2	0
Mapk8ip1	0
Mapk8	0
Mapk6	0
Mapk3	0
Mapk15	0
Mapk14	0
Mapk13	0
Mapk12	0
Mapk11	0
Mapk10	0
Map9	0
Map7d2	0
Map6d1	0
Map6	0
Map4k5	0
Map4k4	0
Map4k3	0
Map4k2	0
Map4k1	0
Map4	0
Map3k9	0
Map3k5	0
Map3k3	0
Map3k2	0
Map3k15	0
Map3k14	0
Map3k13	0
Map3k12	0
Map3k11	0
Map3k1	0
Map2k7	0
Map2k5	0
Map2k4	0
Map2k3	0
Map2k1	0
Map1s	0
Map1lc3b	0
Map1lc3a	0
Map1b	0
Map1a	0
Map10	0
Maob	0
Maoa	0
Mansc4	0
Mansc1	0
Manbal	0
Manba	0
Man2c1	0
Man2b1	0
Man2a2	0
Man1c1	0
Man1b1	0
Man1a2	0
Man1a	0
Mamstr	0
Mamld1	0
Maml3	0
Maml2	0
Malt1	0
Nubp1	0
Nuak2	0
Nuak1	0
Ntsr2	0
Ntsr1	0
Nts	0
Ntrk3	0
Ntrk2	0
Ntrk1	0
Ntn5	0
Ntn3	0
Ntf5	0
Ntf3	0
Ntan1	0
Nt5m	0
Nt5dc3	0
Nt5dc2	0
Nt5dc1	0
Nt5c3b	0
Nt5c1a	0
Nt5c	0
Nsun5	0
Nsun3	0
Nsun2	0
Nsmf	0
Nsmce4a	0
Nsmce2	0
Nsmce1	0
Nsmaf	0
Nsl1	0
Nsfl1c	0
Nsdhl	0
Nsd1	0
Nsa2	0
Nrxn3	0
Nrxn2	0
Nrxn1	0
Nrsn2	0
Nrsn1	0
Nrp2	0
Nrp1	0
Nrn1l	0
Nrn1	0
Nrm	0
Nrk	0
Nrip2	0
Nrgn	0
Nrg4	0
Nrg3	0
Nrg1	0
Nrf1	0
Nrep	0
Nrde2	0
Nrd1	0
Nrcam	0
Nrbp1	0
Nras	0
Nrarp	0
Nrap	0
Nr6a1	0
Nr5a2	0
Nr5a1	0
Nr4a3	0
Nr4a2	0
Nr4a1	0
Nr3c2	0
Nr3c1	0
Nr2f6	0
Nr2f2	0
Nr2f1	0
Nr2e3	0
Nr2e1	0
Nr2c2ap	0
Nr2c2	0
Nr2c1	0
Nr1i3	0
Nr1i2	0
Nr1h5	0
Nr1h2	0
Nr1d2	0
Nr1d1	0
Nr0b2	0
Mc4r	0
Mblac2	0
Cnot6l	0
Supt4a	0
Mblac1	0
BRDN0000737598	0
Npy5r	0
AW549877	0
Tbpl1	0
BRDN0000737829	0
BRDN0000737828	0
Cldn9	0
Ina	0
Smarcad1	0
BRDN0000737824	0
BRDN0000737827	0
H2-M10.6	0
BRDN0000737821	0
BRDN0000737823	0
BRDN0000737822	0
Uts2	0
BRDN0000737800	0
Trap1a	0
Faf1	0
Uncx	0
Pycr2	0
Faf2	0
Lrrn3	0
Pcdhb9	0
Lrrn1	0
Tiam1	0
Ankrd36	0
Ankrd37	0
Ankrd32	0
Ankrd33	0
Fam101b	0
Fam101a	0
Gm10471	0
Gtf3a	0
BRDN0000737383	0
BRDN0000737592	0
Zfp9	0
Slc35b3	0
Eme1	0
Zfp7	0
Zfp1	0
Zfp2	0
Zfp3	0
Rnf130	0
Hgsnat	0
Rnf135	0
Hexdc	0
Pcdhb2	0
Rnf138	0
Rnf139	0
Olfr111	0
Itgb1bp1	0
Aplf	0
Dbil5	0
Pck2	0
Rpp40	0
Grp	0
Odam	0
Relt	0
Tbc1d23	0
Mrap	0
Gp5	0
Tbc1d24	0
Grn	0
Relb	0
Rela	0
Pcdhb4	0
Mcts2	0
Cntf	0
Hrg	0
5830473C10Rik	0
Poc1b	0
Zfp266	0
Zfp263	0
Zfp260	0
Gp6	0
Gm2799	0
Nudc	0
Nucks1	0
Nucb1	0
Nubp2	0
Nuak2	0
Nuak1	0
Ntsr1	0
Ntrk3	0
Ntrk2	0
Ntrk1	0
Ntpcr	0
Ntn4	0
Ntn3	0
Ntmt1	0
Ntf5	0
Ntf3	0
Ntan1	0
Nt5m	0
Nt5e	0
Nt5dc3	0
Nt5dc1	0
Nt5c3b	0
Nt5c2	0
Nt5c1a	0
Nt5c	0
Nsun7	0
Nsun6	0
Nsun5	0
Nsun4	0
Nsun3	0
Nsun2	0
Nsmce4a	0
Nsmce2	0
Nsmce1	0
Nsmaf	0
Nsg2	0
Nsg1	0
Nsfl1c	0
Nsf	0
Nsd1	0
Nrxn3	0
Nrxn2	0
Nrxn1	0
Nrtn	0
Nrsn2	0
Nrsn1	0
Nrp2	0
Nrn1l	0
Nrm	0
Nrip3	0
Nrip2	0
Nrip1	0
Nrg3	0
Nrg1	0
Nrf1	0
Nrep	0
Nrde2	0
Nrcam	0
Nrbp2	0
Nrbp1	0
Nrbf2	0
Nras	0
Nrap	0
Nr6a1	0
Nr5a1	0
Nr4a3	0
Nr3c2	0
Nr3c1	0
Nr2f2	0
Nr2e3	0
Nr2e1	0
Nr2c2ap	0
Nr1i3	0
Nr1i2	0
Nr1h5	0
Nr1h3	0
Nr1h2	0
Nr1d2	0
Nqo2	0
Npy5r	0
Npy4r	0
Npy2r	0

Mall	0
Mal2	0
Mal	0
Mak16	0
Mak	0
Magt1	0
Magohb	0
Magoh	0
Magix	0
Magi3	0
Magi1	0
Magel2	0
Mageh1	0
Magee2	0
Magee1	0
Maged2	0
Maged1	0
Mageb5	0
Mageb4	0
Mageb3	0
Mageb18	0
Mageb16	0
Mageb1	0
Magea8	0
Magea6	0
Magea5	0
Magea3	0
Magea2	0
Magea10	0
Magea1	0
Mag	0
Mafk	0
Mafg	0
Mafb	0
Mafa	0
Maf1	0
Maf	0
Mael	0
Maea	0
Mad2l1	0
Mad1l1	0
Macrod1	0
Macf1	0
Macc1	0
Mab21l3	0
Mab21l2	0
Mab21l1	0
Maats1	0
M6pr	0
M1ap	0
Lzts3	0
Lzts2	0
Lzts1	0
Lztr1	0
Lztfl1	0
Lzic	0
Lyzl6	0
Lyzl4	0
Lyzl1	0
Lyz2	0
Lyz1	0
Lyst	0
Lysmd4	0
Lysmd3	0
Lysmd1	0
Lyrm9	0
Lyrm7	0
Lyrm4	0
Lyrm2	0
Lyrm1	0
Lypla1	0
Lypd8	0
Lypd6b	0
Lypd6	0
Lypd5	0
Lypd4	0
Lypd3	0
Lypd2	0
Lypd1	0
Lynx1	0
Lyn	0
Lyg2	0
Nr0b1	0
Nqo1	0
Npy6r	0
Npy5r	0
Npy4r	0
Npy2r	0
Npy	0
Nptxr	0
Nptx2	0
Nptn	0
Nprl3	0
Nprl2	0
Npr3	0
Npr1	0
Nppb	0
Nppa	0
Npm2	0
Npm1	0
Nploc4	0
Npl	0
Nphp4	0
Nphp3	0
Nphp1	0
Npff	0
Npepps	0
Npepl1	0
Npcd	0
Npc2	0
Npc1	0
Npbwr1	0
Npat	0
Npas4	0
Npas3	0
Npas1	0
Noxa1	0
Nox4	0
Nov	0
Notch4	0
Nop9	0
Nop58	0
Nop56	0
Nop2	0
Nop16	0
Nop10	0
Nomo1	0
Nom1	0
Nolc1	0
Nol8	0
Nol7	0
Nol6	0
Nol3	0
Nol12	0
Nol10	0
Nog	0
Nodal	0
Nod2	0
Nod1	0
Noc4l	0
Noc3l	0
Noc2l	0
Nob1	0
Noa1	0
Nnmt	0
Nnat	0
Nmur2	0
Nmur1	0
Nmt2	0
Nms	0
Nmrk1	0
Nmnat3	0
Nme8	0
Nme7	0
Nme6	0
Nme5	0
Nme4	0
Nme3	0
Nme2	0
Nmbr	0
Nlrx1	0
Nlrp9a	0
Nlrp4f	0
Nlrp4e	0
Afap1	0
C1s1	0
1110008P14Rik	0
Aip	0
BRDN0000737895	0
Olfr1129	0
Tbc1d2b	0
4933402N22Rik	0
Calm5	0
Calm2	0
Calm3	0
Calm1	0
Olfr1121	0
Lce3e	0
Lce3f	0
Olfr1122	0
Lce3b	0
Lag3	0
Klk1	0
BRDN0000738163	0
BRDN0000737482	0
Gm6710	0
Fam83f	0
Klk6	0
Mtrr	0
Klk8	0
Klk9	0
Apold1	0
BRDN0000737399	0
Rmdn3	0
Frg1	0
Wtap	0
Map2k6	0
Cox5a	0
Cox5b	0
Fscn3	0
Rmdn1	0
BRDN0000737700	0
1700034J05Rik	0
Olfr512	0
Slx4ip	0
Olfr510	0
Grk1	0
Frmd8	0
Olfr517	0
Olfr514	0
Vmn1r170	0
BRDN0000737838	0
Zfp692	0
Frmd7	0
Prr15l	0
Card11	0
Frmd3	0
Mxi1	0
Card10	0
Zfp553	0
Vmn1r177	0
Crtac1	0
Gm6583	0
BRDN0000737480	0
Fam83d	0
BRDN0000737531	0
Pcsk4	0
Ccp110	0
Zfp691	0
Tomm40l	0
AF067063	0
Fbll1	0
Clec12b	0
Pafah2	0
Txndc2	0
Trhde	0
Gpt2	0
Cyfip2	0
BRDN0000737487	0
Tmem237	0
Chfr	0
Atad2b	0
E2f7	0
Eda2r	0
E2f5	0
E2f4	0
Npy1r	0
Npw	0
Npvf	0
Nptxr	0
Nptx2	0
Nptx1	0
Nptn	0
Npsr1	0
Nps	0
Nprl3	0
Nprl2	0
Npr2	0
Npr1	0
Nppc	0
Npnt	0
Npm1	0
Nploc4	0
Npl	0
Nphs1	0
Nphp4	0
Npffr2	0
Npff	0
Npepl1	0
Npcd	0
Npc1l1	0
Npc1	0
Npbwr1	0
Npb	0
Npat	0
Npas2	0
Npas1	0
Noxred1	0
Nox4	0
Nox3	0
Nova1	0
Notum	0
Noto	0
Notch3	0
Nosip	0
Nos1ap	0
Nop9	0
Nop58	0
Nop56	0
Nop2	0
Nop16	0
Nop14	0
Nop10	0
Nono	0
Nomo1	0
Nom1	0
Nolc1	0
Nol8	0
Nol6	0
Nol4l	0
Nol3	0
Nol12	0
Nol11	0
Nol10	0
Nodal	0
Nod2	0
Nod1	0
Noc4l	0
Noc3l	0
Noc2l	0
Nob1	0
Noa1	0
Nnmt	0
Nnat	0
Nmur2	0
Nmur1	0
Nmt1	0
Nms	0
Nmrk1	0
Nmnat3	0
Nmnat2	0
Nmnat1	0
Nme8	0
Nme7	0
Nme6	0
Nme4	0
Nme1	0
Nmd3	0

Lyg1	0
Lyar	0
Ly96	0
Ly9	0
Ly75	0
Ly6k	0
Ly6h	0
Ly6g6f	0
Ly6g6e	0
Ly6g6d	0
Ly6g5c	0
Ly6g5b	0
Ly6f	0
Ly6e	0
Ly6d	0
Ly6c2	0
Ly6c1	0
Ly6a	0
Lxn	0
Luzp4	0
Luzp2	0
Lurap1l	0
Lurap1	0
Lum	0
Luc7l2	0
Luc7l	0
Ltv1	0
Ltn1	0
Ltc4s	0
Ltbr	0
Ltb4r2	0
Ltb4r1	0
Lta4h	0
Lta	0
Lst1	0
Lsp1	0
Lsmem1	0
Lsm8	0
Lsm7	0
Lsm6	0
Lsm5	0
Lsm4	0
Lsm3	0
Lsm2	0
Lsm14b	0
Lsm14a	0
Lsm12	0
Lsm11	0
Lsm10	0
Lsm1	0
Lsg1	0
Lrwd1	0
Lrtm2	0
Lrtm1	0
Lrsam1	0
Lrrtm4	0
Lrrtm3	0
Lrrtm2	0
Lrrtm1	0
Lrrn2	0
Lrrn1	0
Lrrk2	0
Lrriq4	0
Lrriq3	0
Lrriq1	0
Lrrfip2	0
Lrrfip1	0
Lrrd1	0
Lrrcc1	0
Lrrc8e	0
Lrrc8d	0
Lrrc8c	0
Lrrc8b	0
Lrrc8a	0
Lrrc75b	0
Lrrc75a	0
Lrrc74b	0
Lrrc73	0
Lrrc71	0
Lrrc7	0
Lrrc69	0
Lrrc66	0
Nlrp4a	0
Nlrp2	0
Nlrp1a	0
Nlrp12	0
Nlrp10	0
Nlrc5	0
Nlrc4	0
Nlrc3	0
Nlk	0
Nlgn1	0
Nle1	0
Nkx6-3	0
Nkx6-2	0
Nkx3-2	0
Nkx2-6	0
Nkx2-5	0
Nkx2-3	0
Nkx2-2	0
Nkx2-1	0
Nkx1-2	0
Nktr	0
Nkrf	0
Nkpd1	0
Nkg7	0
Nkd2	0
Nkd1	0
Nkapl	0
Nkap	0
Nkain4	0
Nkain3	0
Nkain2	0
Nit1	0
Nisch	0
Nipsnap3b	0
Nipsnap3a	0
Nipsnap1	0
Nipbl	0
Nipal3	0
Nipal2	0
Nipa2	0
Nipa1	0
Nip7	0
Ninl	0
Ninj2	0
Ninj1	0
Nim1k	0
Nifk	0
Nid2	0
Nid1	0
Nicn1	0
Nhsl2	0
Nhsl1	0
Nhs	0
Nhp2l1	0
Nhp2	0
Nhlrc4	0
Nhlrc2	0
Ngp	0
Ngly1	0
Ngfrap1	0
Ngfr	0
Ngef	0
Ngdn	0
Ngb	0
Nfyc	0
Nfyb	0
Nfxl1	0
Nfu1	0
Nfs1	0
Nfrkb	0
Nfkbid	0
Nfkbia	0
Nfkb2	0
Nfkb1	0
Nfix	0
Nfil3	0
Nfib	0
Nfia	0
Nfe2l2	0
Nfe2l1	0
Nfe2	0
Nfatc2ip	0
E2f3	0
Tmem126a	0
Tmem126b	0
Jarid2	0
Asz1	0
Csf1r	0
1700012L04Rik	0
Riiad1	0
E2f8	0
Unc93a	0
BRDN0000738018	0
Prok1	0
Braf	0
Bola1	0
Bola3	0
Bola2	0
Dyx1c1	0
Itgbl1	0
Batf	0
Slc25a54	0
Garem	0
Katnbl1	0
Guf1	0
Rabgap1	0
Cep131	0
Acat2	0
Cep135	0
Palld	0
Nod1	0
Fam192a	0
Abat	0
Arrb1	0
Gmeb2	0
Hlf	0
Gmeb1	0
Rs1	0
Scgb2b20	0
Mmp1a	0
Aph1c	0
Aph1b	0
Aph1a	0
Cemip	0
Ctla2a	0
Mmp1b	0
Spata22	0
BRDN0000738002	0
Pgls	0
Vmn1r171	0
Brinp1	0
Magea8	0
Pnpo	0
BRDN0000738248	0
Magea3	0
Magea2	0
Magea1	0
Magea6	0
Magea5	0
Magea4	0
Ctla2b	0
1700007G11Rik	0
Tmem233	0
BRDN0000737530	0
Ppp1r14d	0
Krcc1	0
Pi4k2b	0
Pi4k2a	0
Scgb2b26	0
9430020K01Rik	0
Pnliprp1	0
Spata20	0
Nkpd1	0
Clec4n	0
Pnp2	0
Ihh	0
Gtpbp4	0
Gtpbp6	0
Gtpbp1	0
2410004B18Rik	0
Mdga1	0
D3Ertd254e	0
Klhdc2	0
Sv2c	0
Nmbr	0
Nlrx1	0
Nlrp9c	0
Nlrp9b	0
Nlrp9a	0
Nlrp6	0
Nlrp4f	0
Nlrp4e	0
Nlrp3	0
Nlrp2	0
Nlrp1a	0
Nlrc5	0
Nlrc4	0
Nlrc3	0
Nlk	0
Nlgn1	0
Nkx6-3	0
Nkx3-2	0
Nkx3-1	0
Nkx2-6	0
Nkx2-4	0
Nkx2-2	0
Nkx2-1	0
Nkx1-2	0
Nkx1-1	0
Nktr	0
Nkrf	0
Nkpd1	0
Nkg7	0
Nkd2	0
Nkapl	0
Nkap	0
Nkain3	0
Nkain2	0
Nit2	0
Nit1	0
Nisch	0
Nipsnap3b	0
Nipsnap3a	0
Nipsnap1	0
Nipbl	0
Nipal4	0
Nipal3	0
Nipa1	0
Nip7	0
Ninl	0
Ninj2	0
Ninj1	0
Nin	0
Nim1k	0
Nifk	0
Nid2	0
Nid1	0
Nicn1	0
Nhp2l1	0
Nhp2	0
Nhlrc3	0
Nhlrc2	0
Nhlh2	0
Nhlh1	0
Ngp	0
Ngly1	0
Ngfr	0
Ngf	0
Ngef	0
Ngdn	0
Ngb	0
Nfyc	0
Nfyb	0
Nfya	0
Nfxl1	0
Nfx1	0
Nfu1	0
Nfs1	0
Nfrkb	0
Nfkbiz	0
Nfkbil1	0
Nfkbib	0
Nfkbia	0
Nfkb2	0
Nfkb1	0
Nfix	0

Lrrc63	0	Nfasc	0	BRDN0000737708	0	Nfic	0
Lrrc61	0	Nfam1	0	Xbp1	0	Nfib	0
Lrrc6	0	Nf2	0	Gm8267	0	Nfia	0
Lrrc57	0	Neurog2	0	Trio	0	Nfe2l3	0
Lrrc56	0	Neurog1	0	Eda	0	Nfatc4	0
Lrrc55	0	Neurod4	0	Tpst2	0	Nfatc1	0
Lrrc52	0	Neurod1	0	Hamp	0	Nfat5	0
Lrrc4c	0	Neurl4	0	Tpst1	0	Nfasc	0
Lrrc4b	0	Neurl3	0	Gcgr	0	Nfam1	0
Lrrc49	0	Neurl2	0	Pcmt1	0	Neurog3	0
Lrrc47	0	Neurl1b	0	Gli2	0	Neurod6	0
Lrrc46	0	Neurl1a	0	Kctd12b	0	Neurod4	0
Lrrc45	0	Neu4	0	Clec4f	0	Neurl3	0
Lrrc43	0	Neu2	0	Fgd5	0	Neurl2	0
Lrrc41	0	Neu1	0	Cyp2d34	0	Neurl1b	0
Lrrc40	0	Neto1	0	Ahnak	0	Neurl1a	0
Lrrc4	0	Net1	0	Ubxn11	0	Neu4	0
Lrrc3b	0	Neo1	0	Hscb	0	Neu3	0
Lrrc39	0	Nenf	0	Tomm6	0	Neu2	0
Lrrc36	0	Nemf	0	Sec11c	0	Neto2	0
Lrrc34	0	Nell2	0	BRDN0000738173	0	Neto1	0
Lrrc32	0	Nelfe	0	Gm13103	0	Nes	0
Lrrc30	0	Nelfcd	0	Ktn1	0	Nepn	0
Lrrc3	0	Nelfb	0	Sbno2	0	Neo1	0
Lrrc29	0	Nelfa	0	Pramef12	0	Nenf	0
Lrrc28	0	Nek6	0	B3gnt6	0	Nemf	0
Lrrc27	0	Nek5	0	Gng13	0	Nelfe	0
Lrrc26	0	Nek4	0	Arpp19	0	Nelfcd	0
Lrrc25	0	Nek3	0	Ngp	0	Nelfa	0
Lrrc24	0	Nek11	0	Sdr16c6	0	Nek8	0
Lrrc23	0	Nek10	0	Ccer1	0	Nek6	0
Lrrc20	0	Nek1	0	Nlrp4f	0	Nek4	0
Lrrc2	0	Neil3	0	Hsbp1l1	0	Nek2	0
Lrrc19	0	Neil2	0	Mga	0	Nek11	0
Lrrc17	0	Neil1	0	Il1rapl1	0	Nek10	0
Lrrc16b	0	Negr1	0	BRDN0000738011	0	Neil3	0
Lrrc14b	0	Nefm	0	Spem1	0	Neil2	0
Lrrc10b	0	Nefl	0	Tbp	0	Negr1	0
Lrrc10	0	Nedd9	0	Sdr16c5	0	Nefl	0
Lrrc1	0	Nedd8	0	Mylk3	0	Nedd8	0
Lrr1	0	Nedd4l	0	2310035C23Rik	0	Nedd4l	0
Lrpprc	0	Nedd4	0	Atp6v1b1	0	Nedd4	0
Lrpap1	0	Nedd1	0	Mgp	0	Nedd1	0
Lrp6	0	Necap2	0	Lmod3	0	Necap2	0
Lrp5	0	Necap1	0	Svip	0	Necab3	0
Lrp4	0	Necab3	0	Serpinb9b	0	Necab2	0
Lrp2	0	Nebl	0	Emp3	0	Nebl	0
Lrp1b	0	Ndufv3	0	BRDN0000738171	0	Ndufv3	0
Lrp12	0	Ndufv2	0	Cdk18	0	Ndufs8	0
Lrp11	0	Ndufs8	0	Dpt	0	Ndufs7	0
Lrp10	0	Ndufs6	0	Hgfac	0	Ndufs6	0
Lrp1	0	Ndufs4	0	Fshb	0	Ndufs5	0
Lrmp	0	Ndufs2	0	Mrgbp	0	Ndufs4	0
Lrit2	0	Ndufc2	0	Shd	0	Ndufs2	0
Lrit1	0	Ndufc1	0	Fam228a	0	Ndufs1	0
Lrig2	0	Ndufb9	0	Fshr	0	Ndufc2	0
Lrig1	0	Ndufb8	0	Shh	0	Ndufb9	0
Lrguk	0	Ndufb5	0	Zfp809	0	Ndufb8	0
Lrg1	0	Ndufaf6	0	Smc1a	0	Ndufb5	0
Lrfn4	0	Ndufaf4	0	Ptgs2	0	Ndufb4	0
Lrfn3	0	Ndufab1	0	Ptgs1	0	Ndufb2	0
Lrfn2	0	Ndufa7	0	Smc1b	0	Ndufb11	0
Lrfn1	0	Ndufa6	0	Adnp	0	Ndufb10	0
Lrch4	0	Ndufa2	0	Cfh	0	Ndufaf7	0
Lrch3	0	Ndufa10	0	Cfi	0	Ndufaf6	0
Lrch2	0	Ndufa1	0	Trabd	0	Ndufab1	0
Lrch1	0	Ndst3	0	Cfd	0	Ndufa7	0
Lrba	0	Ndst2	0	Tsen54	0	Ndufa6	0
Lrat	0	Ndrg4	0	Slmap	0	Ndufa5	0
Lpxn	0	Ndrg3	0	Rai1	0	Ndufa4l2	0
Lppr4	0	Ndrg2	0	Obfc1	0	Ndufa4	0
Lppr3	0	Ndp	0	Actl11	0	Ndufa3	0
Lppr1	0	Ndor1	0	Actl10	0	Ndufa2	0
Lpo	0	Ndnl2	0	Apoa1bp	0	Ndufa13	0
Lpl	0	Ndfip2	0	BRDN0000737471	0	Ndufa11	0
Lpin3	0	Ndfip1	0	Cela1	0	Ndufa1	0
Lpgat1	0	Ndel1	0	Cfp	0	Ndst4	0
Lpcat4	0	Ndc80	0	BRDN0000738012	0	Ndst3	0
Lpcat3	0	Ncs1	0	7530416G11Rik	0	Ndst2	0
Lpcat2	0	Ncr1	0	1700047I17Rik2	0	Ndst1	0
Lpar6	0	Ncor2	0	Rimbp2	0	Ndrg4	0
Lpar5	0	Ncor1	0	Rimbp3	0	Ndrg1	0

Lpar4	0	Ncoa6	0	Gm5728	0	Ndor1	0
Loxl4	0	Ncoa5	0	Fam118b	0	Ndnl2	0
Loxl3	0	Ncoa2	0	Fam173b	0	Ndfip1	0
Loxl2	0	Ncoa1	0	Pdzd7	0	Ndel1	0
Loxl1	0	Ncmap	0	Kif21b	0	Ndc80	0
Lox	0	Ncln	0	Mmp17	0	Ncs1	0
Lonrf2	0	Ncl	0	Pdzd3	0	Ncor2	0
Lonrf1	0	Nckipsd	0	Pdzd2	0	Ncor1	0
Lonp2	0	Nckap5l	0	Ston1	0	Ncoa6	0
Loh12cr1	0	Nckap5	0	Samd12	0	Ncoa5	0
LOC100862015	0	Nckap1l	0	Ddx11	0	Ncoa4	0
LOC100861615	0	Nckap1	0	Lum	0	Ncoa3	0
LOC100048884	0	Nck2	0	Pdzd9	0	Ncoa2	0
LOC100038947	0	Nck1	0	Pdzd8	0	Ncln	0
Lnx2	0	Ncf4	0	Zfp41	0	Ncl	0
Lnx1	0	Ncf2	0	G0s2	0	Nckipsd	0
Lmx1b	0	Ncf1	0	Trp73	0	Nckap5l	0
Lmx1a	0	Ncdn	0	Zgpat	0	Nckap1	0
Lmtk3	0	Ncbp2	0	Trhr2	0	Nck1	0
Lmtk2	0	Ncbp1	0	Ssfa2	0	Ncf4	0
Lmod3	0	Ncaph2	0	Bak1	0	Ncf2	0
Lmod2	0	Ncaph	0	Nlrp2	0	Nccrp1	0
Lmo3	0	Ncapg2	0	Slco5a1	0	Ncbp1	0
Lmo2	0	Ncapg	0	Alg10b	0	Ncaph	0
Lmo1	0	Ncapd3	0	Nol3	0	Ncapg2	0
Lmntd2	0	Ncapd2	0	Phf8	0	Ncapd3	0
Lmnb2	0	Ncan	0	Cacul1	0	Ncapd2	0
Lmnb1	0	Ncam2	0	Phf6	0	Ncam1	0
Lmf2	0	Ncam1	0	Phf7	0	Nbr1	0
Lmf1	0	Ncald	0	BRDN0000738014	0	Nbn	0
Lmcd1	0	Nbr1	0	Phf2	0	Nbl1	0
Lmbrd2	0	Nbn	0	Hsdl1	0	Nbeal2	0
Lmbrd1	0	Nbl1	0	Fgd2	0	Nbeal1	0
Lmbr1	0	Nbeal2	0	Krt12	0	Nbea	0
Lman2l	0	Nbea	0	Tnpo2	0	Nav3	0
Lman2	0	Nbas	0	Tnpo3	0	Nav2	0
Llph	0	Nav3	0	Tnpo1	0	Nav1	0
Llgl2	0	Nav2	0	Efnb3	0	Natd1	0
Llgl1	0	Nav1	0	Trim12a	0	Nat8l	0
Lkaaear1	0	Natd1	0	Gtf2a1l	0	Nat8	0
Lix1l	0	Nat9	0	Trim50	0	Nat6	0
Lix1	0	Nat8l	0	Tex36	0	Nat3	0
Litaf	0	Nat8	0	Aknad1	0	Nat2	0
Lipt2	0	Nat3	0	Olfr1254	0	Nat1	0
Lipt1	0	Nat2	0	Trim43b	0	Nars2	0
Lipo1	0	Nat14	0	Sema4d	0	Nars	0
Lipn	0	Nat10	0	Olfr1158	0	Narfl	0
Lipi	0	Nat1	0	Trim43a	0	Narf	0
Liph	0	Nasp	0	Scn4a	0	Napsa	0
Lipg	0	Nars	0	Fblim1	0	Naprt	0
Lipf	0	Narfl	0	Cyp7a1	0	Napg	0
Lipe	0	Narf	0	Cdkn2aipnl	0	Napepld	0
Lipa	0	Napsa	0	Olfr1252	0	Napb	0
Lins	0	Naprt	0	Grem2	0	Napa	0
Lingo3	0	Napg	0	Klb	0	Nap1l5	0
Lingo2	0	Napepld	0	Grem1	0	Nap1l4	0
Lin9	0	Napa	0	Fhit	0	Nap1l2	0
Lin7c	0	Nap1l5	0	BRDN0000737998	0	Nap1l1	0
Lin7b	0	Nap1l4	0	Pla2g12b	0	Nanp	0
Lin7a	0	Nap1l2	0	App	0	Nanos2	0
Lin54	0	Nap1l1	0	Lsm6	0	Nanos1	0
Lin52	0	Nans	0	BRDN0000738016	0	Nanog	0
Lin37	0	Nanos2	0	Apc	0	Nampt	0
Lin28b	0	Nanos1	0	Krt18	0	Nalcn	0
Lin28a	0	Nampt	0	Klrb1a	0	Naip6	0
Lims2	0	Naip5	0	Foxa2	0	Naip5	0
Lims1	0	Naip2	0	Adora2a	0	Naip2	0
Limk2	0	Naip1	0	Adora2b	0	Naip1	0
Lime1	0	Nags	0	Mis12	0	Naif1	0
Limd2	0	Nagpa	0	BRDN0000737549	0	Naglu	0
Limch1	0	Naglu	0	Tsc2	0	Naf1	0
Lima1	0	Nagk	0	Hoxa13	0	Nae1	0
Lim2	0	Naf1	0	Hoxa11	0	Nadk2	0
Lilrb4	0	Nadk2	0	Hoxa10	0	Nacc2	0
Lilra6	0	Nadk	0	Hyal1	0	Nacc1	0
Lig4	0	Nacc2	0	BRDN0000737789	0	Nacad	0
Lig3	0	Nacc1	0	Abcb7	0	Naca	0
Lig1	0	Nacad	0	Abcb6	0	Nabp2	0
Lif	0	Naca	0	Abcb5	0	Nab2	0
Lias	0	Nabp2	0	Abcb4	0	Nab1	0
Lhx9	0	Nabp1	0	Dhx30	0	Naaladl1	0
Lhx5	0	Nab1	0	BRDN0000737781	0	Naalad2	0

Lhx4	0	Naaladl1	0	Abcb9	0	Naa40	0
Lhx3	0	Naalad2	0	BRDN0000737783	0	Naa38	0
Lhx2	0	Naaa	0	Fosl2	0	Naa35	0
Lhx1	0	Naa60	0	BRDN0000737785	0	Naa30	0
Lhfpl5	0	Naa50	0	BRDN0000737786	0	Naa16	0
Lhfpl4	0	Naa40	0	BRDN0000737787	0	Naa15	0
Lhfpl3	0	Naa35	0	BRDN0000737623	0	Naa11	0
Lhfpl1	0	Naa30	0	Ephx2	0	N6amt2	0
Lhfp	0	Naa25	0	Slbp	0	N6amt1	0
Lhcgr	0	Naa20	0	BC035044	0	N4bp2l2	0
Lhb	0	Naa11	0	Arpc3	0	N4bp2l1	0
Lgsn	0	N6amt2	0	Arpc2	0	N4bp1	0
Lgr5	0	N6amt1	0	Arpc1a	0	Mzt2	0
Lgmn	0	N4bp3	0	Otc	0	Mzt1	0
Lgi4	0	N4bp2	0	Arpc5	0	Mzf1	0
Lgi1	0	Mzt2	0	Arpc4	0	Mzb1	0
Lgalsl	0	Mzf1	0	Tmem119	0	Myzap	0
Lgals9	0	Mzb1	0	2310047M10Rik	0	Myt1l	0
Lgals8	0	Myzap	0	Ahsg	0	Myrfl	0
Lgals7	0	Myt1l	0	Otp	0	Myrf	0
Lgals4	0	Myt1	0	Itpr3	0	Myoz1	0
Lgals3bp	0	Myrip	0	Itpr1	0	Myom3	0
Lgals3	0	Myrfl	0	Ott	0	Myom2	0
Lgals2	0	Mypop	0	Tmem115	0	Myog	0
Lgals12	0	Mypn	0	Tmem116	0	Myod1	0
Lgals1	0	Myoz3	0	Tmem117	0	Myocd	0
Lfng	0	Myoz2	0	Spata13	0	Myoc	0
Letmd1	0	Myoz1	0	Ift43	0	Myo9b	0
Letm2	0	Myot	0	Olfr705	0	Myo9a	0
Letm1	0	Myom3	0	Olfr704	0	Myo5c	0
Leprot	0	Myom2	0	Spata17	0	Myo5b	0
Lepr	0	Myom1	0	Spata16	0	Myo3b	0
Lep	0	Myod1	0	Olfr701	0	Myo1h	0
Leng9	0	Myocd	0	Rundc1	0	Myo1f	0
Leng1	0	Myoc	0	Sft2d3	0	Myo1b	0
Lenep	0	Myo9b	0	Sft2d2	0	Myo1a	0
Lemd2	0	Myo9a	0	Sft2d1	0	Myo18b	0
Lemd1	0	Myo7b	0	Spata18	0	Myo16	0
Lelp1	0	Myo7a	0	Zc3h4	0	Myo15	0
Lekr1	0	Myo5c	0	Pax5	0	Mynn	0
Lefty2	0	Myo3b	0	N4bp3	0	Mylpf	0
Lefty1	0	Myo1h	0	Pax7	0	Mylk3	0
Lef1	0	Myo1g	0	Pax6	0	Mylk2	0
Lect2	0	Myo1f	0	Gm20816	0	Mylk	0
Lect1	0	Myo1e	0	Txndc15	0	Mylip	0
Ldoc1l	0	Myo1c	0	Pax3	0	Myl4	0
Ldlrap1	0	Myo1b	0	Gm20815	0	Myl2	0
Ldlrad2	0	Myo1a	0	Mphosph10	0	Myl12b	0
Ldlrad1	0	Myo19	0	Mrpl42	0	Myl10	0
Ldlr	0	Myo18b	0	Nr2c1	0	Myl1	0
Ldhd	0	Mynn	0	Flywch2	0	Myh8	0
Ldhc	0	Mylpf	0	Pax8	0	Myh7b	0
Ldhb	0	Mylk2	0	Flywch1	0	Myh7	0
Ldha	0	Mylk	0	Fcer1a	0	Myh6	0
Ldb3	0	Myl9	0	U2af1	0	Myh3	0
Ldb2	0	Myl7	0	Rpgr	0	Myh15	0
Ldb1	0	Myl6b	0	U2af2	0	Myh14	0
Ldah	0	Myl6	0	Stoml3	0	Myh13	0
Lctl	0	Myl4	0	Stoml2	0	Myh11	0
Lct	0	Myl3	0	Stoml1	0	Myh10	0
Lcp2	0	Myl2	0	Kif19a	0	Myh1	0
Lcorl	0	Myl12b	0	Hist1h3b	0	Myf5	0
Lcor	0	Myl10	0	Hist1h3c	0	Myeov2	0
Lcn9	0	Myh9	0	Hist1h3a	0	Myef2	0
Lcn8	0	Myh8	0	Hist1h3f	0	Mydgf	0
Lcn6	0	Myh7b	0	Hist1h3g	0	Myct1	0
Lcn5	0	Myh7	0	Hist1h3d	0	Mycs	0
Lcn4	0	Myh6	0	Hist1h3e	0	Mycn	0
Lcn3	0	Myh15	0	Telo2	0	Mycl	0
Lcn2	0	Myh14	0	Hist1h3h	0	Mycbpap	0
Lcn12	0	Myh13	0	Hist1h3i	0	Mycbp2	0
Lcn10	0	Myh10	0	Lemd3	0	Mybphl	0
Lcmt1	0	Myeov2	0	Sarm1	0	Mybpc3	0
Lclat1	0	Myef2	0	Lemd1	0	Mybpc2	0
Lck	0	Mydgf	0	Trappc9	0	Mybpc1	0
Lce3f	0	Mycs	0	Trappc8	0	Myadml2	0
Lce3d	0	Mycn	0	Pabpn1	0	Myadm	0
Lce3c	0	Mycl	0	Triqk	0	Mxra8	0
Lce3b	0	Mycbp2	0	Phtf2	0	Mxra7	0
Lce1m	0	Mycbp	0	Trappc1	0	Mxi1	0
Lce1j	0	Myc	0	Rtcb	0	Mxd3	0
Lce1i	0	Mybphl	0	Trappc3	0	Mvk	0

Lce1h	0	Mybpc2	0	Trappc2	0	Mvd	0
Lce1f	0	Mybpc1	0	Vcl	0	Mvb12b	0
Lce1e	0	Mybl2	0	Thrsp	0	Mutyh	0
Lce1d	0	Mybl1	0	Bloc1s1	0	Mustn1	0
Lce1c	0	Mybbp1a	0	Vcp	0	Musk	0
Lce1b	0	Myadml2	0	Bglap	0	Mus81	0
Lce1a2	0	Myadm	0	Capn5	0	Murc	0
Lce1a1	0	Mxra7	0	Mum1l1	0	Mup9	0
Lca5	0	Mxi1	0	Capn3	0	Mup8	0
Lbx1	0	Mxd4	0	Mchr1	0	Mup6	0
Lbp	0	Mxd1	0	Capn1	0	Mup5	0
Lbh	0	Mvk	0	Dab2	0	Mup3	0
Layn	0	Mvd	0	Dab1	0	Mup20	0
Lax1	0	Mutyh	0	AU022751	0	Mup2	0
Lats2	0	Mut	0	Ankrd33b	0	Mup19	0
Lat2	0	Mustn1	0	Cyp2b23	0	Mup16	0
Lasp1	0	Mus81	0	1700008P02Rik	0	Mup14	0
Las1l	0	Murc	0	BRDN0000738019	0	Mup10	0
Lars2	0	Mup9	0	Ces1b	0	Mum1l1	0
Lars	0	Mup8	0	Olfr1370	0	Mum1	0
Larp7	0	Mup5	0	Olfr1371	0	Mul1	0
Larp4b	0	Mup4	0	Gbp2b	0	Mug2	0
Larp4	0	Mup3	0	Srxn1	0	Muc5ac	0
Larp1b	0	Mup21	0	Cym	0	Muc4	0
Larp1	0	Mup20	0	Olfr1378	0	Muc20	0
Large	0	Mup2	0	Defb30	0	Muc13	0
Laptm4b	0	Mup19	0	Comt	0	Mtx3	0
Laptm4a	0	Mup16	0	Lgi4	0	Mtx1	0
Lap3	0	Mup14	0	Fam71d	0	Mtus1	0
Lao1	0	Mup10	0	Itga11	0	Mttp	0
Lancl3	0	Mup1	0	Fam71b	0	Mtss1l	0
Lancl2	0	Mug2	0	Golm1	0	Mtss1	0
Lamtor4	0	Mug1	0	Timm22	0	Mtrr	0
Lamtor3	0	Muc5b	0	Eif4e1b	0	Mtrf1l	0
Lamtor2	0	Muc5ac	0	Nsd1	0	Mtrf1	0
Lamtor1	0	Muc4	0	Gm4477	0	Mtpap	0
Lamp3	0	Muc20	0	Plxdc2	0	Mtor	0
Lamp2	0	Muc13	0	Plxdc1	0	Mtnr1a	0
Lamp1	0	Mtx3	0	3110043O21Rik	0	Mtmr9	0
Lamc2	0	Mtx1	0	Tas2r130	0	Mtmr7	0
Lamc1	0	Mtus1	0	Gm14632	0	Mtmr6	0
Lamb3	0	Mttp	0	Tas2r134	0	Mtmr3	0
Lamb2	0	Mtss1l	0	Tas2r135	0	Mtmr12	0
Lama5	0	Mtss1	0	Tas2r136	0	Mtmr11	0
Lama4	0	Mtrr	0	Tas2r137	0	Mtm1	0
Lama3	0	Mtr	0	Tas2r138	0	Mtif3	0
Lama2	0	Mtpn	0	Tas2r139	0	Mthfsl	0
Lama1	0	Mtor	0	Olfr418	0	Mthfsd	0
Lair1	0	Mto1	0	Ebpl	0	Mthfs	0
Lage3	0	Mtnr1a	0	9430069I07Rik	0	Mthfd2	0
Lactbl1	0	Mtmr9	0	BRDN0000737762	0	Mthfd1l	0
Lactb2	0	Mtmr7	0	Gm4836	0	Mthfd1	0
Lace1	0	Mtmr6	0	Olfr859	0	Mtg2	0
Lacc1	0	Mtmr4	0	BRDN0000737763	0	Mtg1	0
l7Rn6	0	Mtmr3	0	Olfr857	0	Mtfr2	0
L3mbtl4	0	Mtmr2	0	Olfr855	0	Mtfr1l	0
L3mbtl3	0	Mtmr14	0	Chac1	0	Mtfp1	0
L3mbtl2	0	Mtmr10	0	Olfr853	0	Mterf4	0
L3hypdh	0	Mtmr1	0	Bckdk	0	Mterf3	0
L2hgdh	0	Mtl5	0	Olfr851	0	Mterf2	0
L1td1	0	Mtif3	0	Olfr850	0	Mterf1a	0
L1cam	0	Mtif2	0	Timm17b	0	Mtcp1	0
Kynu	0	Mthfsl	0	Pramel6	0	Mtcl1	0
Ky	0	Mthfsd	0	Timm17a	0	Mtch2	0
Ktn1	0	Mthfs	0	Olfr776	0	Mtch1	0
Ksr2	0	Mthfr	0	Slc6a20b	0	Mtbp	0
Ksr1	0	Mthfd2	0	Slc6a20a	0	Mta3	0
Krtdap	0	Mthfd1l	0	Hspa1b	0	Mta2	0
Krtcap3	0	Mthfd1	0	Glrx5	0	Mt3	0
Krtcap2	0	Mtg1	0	Glrx2	0	Mt2	0
Krtap9-1	0	Mtf2	0	Glrx3	0	Mt1	0
Krtap8-1	0	Mtf1	0	Ear14	0	Msto1	0
Krtap7-1	0	Mterf3	0	Arfgef1	0	Mstn	0
Krtap6-1	0	Mterf2	0	Arfgef2	0	Mst1r	0
Krtap5-5	0	Mterf1b	0	Arfgef3	0	Mst1	0
Krtap5-4	0	Mterf1a	0	Olfr938	0	Mss51	0
Krtap5-3	0	Mtdh	0	BRDN0000737606	0	Msrb3	0
Krtap5-2	0	Mtch2	0	BRDN0000737765	0	Msrb2	0
Krtap5-1	0	Mtap7d3	0	Gid4	0	Msrb1	0
Krtap4-9	0	Mta3	0	Zrsr1	0	Msr1	0
Krtap4-8	0	Mt3	0	Gpank1	0	Msn	0
Krtap4-7	0	Mt1	0	Oas3	0	Msmp	0

Krtap4-6	0	Msx3	0	Peli1	0	Msmb	0
Krtap4-13	0	Msx2	0	Gdpd4	0	Mslnl	0
Krtap3-3	0	Msx1	0	Adipor1	0	Msl3l2	0
Krtap31-2	0	Msto1	0	Ube3c	0	Msh6	0
Krtap31-1	0	Mstn	0	Prl4a1	0	Msh5	0
Krtap27-1	0	Mst1	0	Sympk	0	Msh4	0
Krtap26-1	0	Mss51	0	Gid8	0	Msh3	0
Krtap24-1	0	Msrb3	0	Kcnmb1	0	Msgn1	0
Krtap2-4	0	Msrb2	0	Tesc	0	Msc	0
Krtap22-2	0	Msra	0	Mapkap1	0	Msantd3	0
Krtap21-1	0	Msn	0	Gatm	0	Ms4a8a	0
Krtap19-9b	0	Msmp	0	Bid	0	Ms4a7	0
Krtap19-5	0	Msmo1	0	Acpp	0	Ms4a6d	0
Krtap19-4	0	Msmb	0	Olfr633	0	Ms4a6c	0
Krtap1-5	0	Mslnl	0	Aanat	0	Ms4a6b	0
Krtap1-4	0	Msln	0	Aatk	0	Ms4a5	0
Krtap14	0	Msl3l2	0	Gatb	0	Ms4a4c	0
Krtap13-1	0	Msl1	0	Gatc	0	Ms4a2	0
Krtap1-3	0	Msi1	0	BRDN0000737807	0	Ms4a13	0
Krtap13	0	Msh5	0	Prss58	0	Ms4a10	0
Krtap12-1	0	Msh4	0	Dlgap3	0	Ms4a1	0
Krtap11-1	0	Msh3	0	Jam2	0	Mrto4	0
Krt9	0	Msgn1	0	Nrd1	0	Mrs2	0
Krt86	0	Msc	0	Jam3	0	Mrrf	0
Krt84	0	Msantd3	0	Lpcat4	0	Mrps9	0
Krt83	0	Msantd2	0	Golga2	0	Mrps7	0
Krt82	0	Ms4a7	0	Wdr13	0	Mrps6	0
Krt80	0	Ms4a6b	0	Wdr12	0	Mrps5	0
Krt79	0	Ms4a4d	0	Wdr11	0	Mrps36	0
Krt78	0	Ms4a4c	0	Eftud1	0	Mrps35	0
Krt77	0	Ms4a2	0	Lair1	0	Mrps34	0
Krt76	0	Ms4a18	0	1700011A15Rik	0	Mrps33	0
Krt75	0	Ms4a13	0	Gm933	0	Mrps31	0
Krt73	0	Mrvi1	0	Plce1	0	Mrps30	0
Krt72	0	Mrto4	0	Wdr19	0	Mrps28	0
Krt71	0	Mrs2	0	Wdr18	0	Mrps27	0
Krt6a	0	Mrrf	0	Ano6	0	Mrps26	0
Krt5	0	Mrps7	0	BC030867	0	Mrps25	0
Krt42	0	Mrps6	0	Rad17	0	Mrps24	0
Krt40	0	Mrps5	0	Bub3	0	Mrps22	0
Krt4	0	Mrps36	0	Dpyd	0	Mrps21	0
Krt39	0	Mrps35	0	Serf1	0	Mrps2	0
Krt35	0	Mrps33	0	Chrnd	0	Mrps18c	0
Krt34	0	Mrps30	0	Serf2	0	Mrps18b	0
Krt33b	0	Mrps28	0	Ano1	0	Mrps18a	0
Krt33a	0	Mrps27	0	Dpys	0	Mrps17	0
Krt32	0	Mrps26	0	Itgb2l	0	Mrps16	0
Krt31	0	Mrps25	0	Hsfy2	0	Mrps15	0
Krt26	0	Mrps23	0	Mpdu1	0	Mrps14	0
Krt25	0	Mrps22	0	Ablim3	0	Mrps11	0
Krt24	0	Mrps21	0	Ablim2	0	Mrps10	0
Krt222	0	Mrps18c	0	Gdap1l1	0	Mrpl9	0
Krt20	0	Mrps18b	0	Sardh	0	Mrpl57	0
Krt19	0	Mrps18a	0	BRDN0000737809	0	Mrpl55	0
Krt16	0	Mrps17	0	Cers5	0	Mrpl53	0
Krt15	0	Mrps16	0	Atxn7l3b	0	Mrpl52	0
Krt14	0	Mrps15	0	Atp6v1d	0	Mrpl51	0
Krt13	0	Mrps14	0	Cdc25c	0	Mrpl50	0
Krt12	0	Mrps12	0	Cdc25b	0	Mrpl49	0
Krt10	0	Mrps11	0	Cdc25a	0	Mrpl48	0
Krt1	0	Mrps10	0	Ear10	0	Mrpl47	0
Krr1	0	Mrpl9	0	Fam207a	0	Mrpl45	0
Krit1	0	Mrpl57	0	Krt17	0	Mrpl44	0
Kri1	0	Mrpl55	0	Zfp40	0	Mrpl43	0
Kremen2	0	Mrpl53	0	Krt15	0	Mrpl42	0
Kremen1	0	Mrpl52	0	Gskip	0	Mrpl41	0
Krcc1	0	Mrpl49	0	Mtif3	0	Mrpl40	0
Krba1	0	Mrpl48	0	A630023A22Rik	0	Mrpl4	0
Kras	0	Mrpl46	0	Zfp46	0	Mrpl39	0
Kprp	0	Mrpl45	0	Srpk2	0	Mrpl38	0
Kpnb1	0	Mrpl44	0	Bfar	0	Mrpl37	0
Kpna6	0	Mrpl43	0	Krt19	0	Mrpl36	0
Kpna4	0	Mrpl42	0	AA986860	0	Mrpl34	0
Kpna3	0	Mrpl41	0	Slc10a3	0	Mrpl32	0
Kpna2	0	Mrpl40	0	Lgi3	0	Mrpl28	0
Kpna1	0	Mrpl4	0	Oma1	0	Mrpl27	0
Kntc1	0	Mrpl39	0	Igflr1	0	Mrpl24	0
Knstrn	0	Mrpl38	0	Slc10a2	0	Mrpl23	0
Knop1	0	Mrpl37	0	Cd160	0	Mrpl22	0
Kng2	0	Mrpl36	0	Cd163	0	Mrpl2	0
Kng1	0	Mrpl34	0	Ulbp1	0	Mrpl19	0
Kndc1	0	Mrpl32	0	Cd164	0	Mrpl18	0

Kncn	0
Kmt2e	0
Kmt2d	0
Kmt2c	0
Kmt2b	0
Kmt2a	0
Klrk1	0
Klri2	0
Klri1	0
Klrg2	0
Klre1	0
Klrd1	0
Klrc3	0
Klrc2	0
Klrc1	0
Klrb1f	0
Klrb1c	0
Klrb1b	0
Klrb1a	0
Klrb1	0
Klra8	0
Klra7	0
Klra6	0
Klra5	0
Klra3	0
Klra17	0
Klra10	0
Klra1	0
Klkb1	0
Klk9	0
Klk8	0
Klk7	0
Klk6	0
Klk5	0
Klk4	0
Klk1b9	0
Klk1b8	0
Klk1b4	0
Klk1b3	0
Klk1b27	0
Klk1b26	0
Klk1b24	0
Klk1b22	0
Klk1b21	0
Klk1b16	0
Klk1b1	0
Klk15	0
Klk14	0
Klk12	0
Klk11	0
Klk10	0
Klk1	0
Klhl7	0
Klhl6	0
Klhl5	0
Klhl42	0
Klhl41	0
Klhl38	0
Klhl36	0
Klhl34	0
Klhl33	0
Klhl31	0
Klhl30	0
Klhl3	0
Klhl29	0
Klhl28	0
Klhl26	0
Klhl23	0
Klhl22	0
Klhl21	0
Klhl20	0
Klhl18	0
Klhl17	0
Klhl15	0
Klhl14	0
Klhl10	0
Klhl1	0
Klhdc9	0
Klhdc8b	0
Klhdc8a	0
Klhdc7b	0
Klhdc7a	0
Mrpl30	0
Mrpl3	0
Mrpl28	0
Mrpl24	0
Mrpl23	0
Mrpl22	0
Mrpl20	0
Mrpl2	0
Mrpl19	0
Mrpl18	0
Mrpl17	0
Mrpl16	0
Mrpl15	0
Mrpl14	0
Mrpl13	0
Mrpl12	0
Mrpl11	0
Mrpl10	0
Mrpl1	0
Mroh7	0
Mroh5	0
Mroh2b	0
Mroh2a	0
Mroh1	0
Mri1	0
Mrgprx2	0
Mrgprx1	0
Mrgpre	0
Mrgprd	0
Mrgprb5	0
Mrgprb2	0
Mrgpra4	0
Mrgpra3	0
Mrgpra2b	0
Mrgpra2a	0
Mrgpra1	0
Mrgbp	0
Mrfap1	0
Mreg	0
Mre11a	0
Mrc2	0
Mrc1	0
Mras	0
Mrap2	0
Mr1	0
Mpzl3	0
Mpzl1	0
Mpv17l2	0
Mpv17l	0
Mpv17	0
Mpst	0
Mprip	0
Mpped1	0
Mppe1	0
Mpp6	0
Mpp2	0
Mpp1	0
Mpo	0
Mpnd	0
Mpi	0
Mphosph6	0
Mphosph10	0
Mpg	0
Mpeg1	0
Moxd2	0
Moxd1	0
Mov10l1	0
Mov10	0
Mospd3	0
Mospd1	0
Morn5	0
Morn4	0
Morn3	0
Morn2	0
Morn1	0
Morf4l2	0
Morc4	0
Morc3	0
Morc2a	0
Mon2	0
Mon1b	0
Mon1a	0
Srp9	0
BRDN0000737645	0
Aco1	0
Rnf182	0
Acsbg1	0
Acsbg2	0
Grin1	0
Srpr	0
Gtpbp10	0
4921524J17Rik	0
Zfyve21	0
Zfyve27	0
Zfyve26	0
Mixl1	0
Uqcc1	0
Ccdc74a	0
Zfyve28	0
Golph3l	0
C87499	0
Eftud2	0
BRDN0000737806	0
BRDN0000737805	0
BRDN0000737804	0
BRDN0000737803	0
BRDN0000737802	0
BRDN0000737801	0
Nedd1	0
Il31ra	0
Tdrd12	0
BRDN0000737784	0
Uqcc2	0
Srpk1	0
Csnk2b	0
H2bfm	0
A1bg	0
Nedd8	0
Ncf2	0
Slc10a1	0
Tuba8	0
Slc10a7	0
Slc10a6	0
Slc10a5	0
Slc10a4	0
Slc44a3	0
Grina	0
Nedd4	0
Egflam	0
Pml	0
Hbq1b	0
Rnf38	0
Rnf39	0
Ankrd16	0
Ankrd17	0
Rnf34	0
Hdgf	0
Rnf32	0
Tmem213	0
Ankrd11	0
Ywhaz	0
Foxj1	0
Foxj2	0
Foxj3	0
Zfp300	0
Zfr2	0
C130079G13Rik	0
Epor	0
4921524L21Rik	0
Col9a3	0
Mcemp1	0
Col9a1	0
Spatc1	0
Ywhah	0
BRDN0000737808	0
Ywhab	0
Bcl9l	0
BRDN0000737578	0
Ccl26	0
Cwf19l1	0
Ywhag	0
Cwf19l2	0
Meiob	0
Dsel	0
Mrpl17	0
Mrpl16	0
Mrpl15	0
Mrpl14	0
Mrpl13	0
Mrpl11	0
Mrpl10	0
Mrpl1	0
Mroh9	0
Mroh8	0
Mroh7	0
Mroh6	0
Mroh5	0
Mroh4	0
Mroh2b	0
Mroh2a	0
Mroh1	0
Mrgprx2	0
Mrgprx1	0
Mrgprh	0
Mrgprg	0
Mrgpre	0
Mrgprd	0
Mrgprb8	0
Mrgprb3	0
Mrgprb1	0
Mrgpra4	0
Mrgpra3	0
Mrgpra2b	0
Mrgpra2a	0
Mrgbp	0
Mrfap1	0
Mre11a	0
Mras	0
Mrap2	0
Mrap	0
Mr1	0
Mpv17l2	0
Mpv17l	0
Mpv17	0
Mptx2	0
Mptx1	0
Mpst	0
Mpped2	0
Mpped1	0
Mpp6	0
Mpp5	0
Mpp2	0
Mpnd	0
Mplkip	0
Mpi	0
Mphosph6	0
Mphosph10	0
Mpg	0
Mpeg1	0
Mpdu1	0
Mpc1	0
Moxd2	0
Moxd1	0
Mospd3	0
Morn5	0
Morn4	0
Morn3	0
Morn2	0
Morf4l1	0
Morc3	0
Morc1	0
Mon2	0
Mon1b	0
Mon1a	0
Mok	0
Mogs	0
Mogat1	0
Mocs3	0
Mobp	0
Mob4	0
Mob3c	0
Mob3b	0
Mob3a	0
Mob2	0
Mob1b	0
Mob1a	0

Klhdc3	0	Mok	0	Tmem106b	0	Moap1	0
Klhdc2	0	Mogat2	0	Gigyf1	0	Mnx1	0
Klhdc1	0	Mogat1	0	Gigyf2	0	Mnt	0
Klf9	0	Mog	0	Tmem106a	0	Mns1	0
Klf8	0	Mocs3	0	Rpp21	0	Mndal	0
Klf7	0	Mocos	0	BC055111	0	Mnda	0
Klf6	0	Mobp	0	Gzmc	0	Mnd1	0
Klf5	0	Mob4	0	Bod1l	0	Mnat1	0
Klf4	0	Mob3c	0	Cdyl	0	Mn1	0
Klf2	0	Mob3a	0	Ngrn	0	Mms22l	0
Klf17	0	Mob2	0	Myo9a	0	Mms19	0
Klf15	0	Mob1b	0	Grid2ip	0	Mmrn2	0
Klf13	0	Moap1	0	Mrpl47	0	Mmrn1	0
Klf12	0	Mnx1	0	Vmn1r60	0	Mmp7	0
Klf11	0	Mnt	0	Pi15	0	Mmp27	0
Klf10	0	Mns1	0	Zfp248	0	Mmp24	0
Klf1	0	Mnda	0	Itpka	0	Mmp23	0
Klc4	0	Mnd1	0	Gpkow	0	Mmp20	0
Klc3	0	Mnat1	0	Gap43	0	Mmp2	0
Klc2	0	Mn1	0	Hmbox1	0	Mmp1b	0
Klc1	0	Mms22l	0	BRDN0000737640	0	Mmp1a	0
Klb	0	Mms19	0	Ankrd12	0	Mmp19	0
Kl	0	Mmrn2	0	Golph3	0	Mmp16	0
Kiz	0	Mmrn1	0	Arhgap21	0	Mmp15	0
Kitl	0	Mmp8	0	Olfr282	0	Mmp13	0
Kit	0	Mmp7	0	Olfr283	0	Mmp12	0
Kirrel2	0	Mmp27	0	4933427G17Rik	0	Mmp11	0
Kirrel	0	Mmp23	0	Olfr281	0	Mmgt2	0
Kir3dl2	0	Mmp21	0	Olfr287	0	Mmgt1	0
Kir3dl1	0	Mmp20	0	Olfr284	0	Mmd	0
Kin	0	Mmp2	0	Cyp4f39	0	Mmadhc	0
Kifc5b	0	Mmp1b	0	Olfr288	0	Mmachc	0
Kifc2	0	Mmp1a	0	Rnf31	0	Mmab	0
Kifc1	0	Mmp17	0	Nup205	0	Mlxipl	0
Kif9	0	Mmp12	0	Esrrg	0	Mlxip	0
Kif7	0	Mmp11	0	Gm3376	0	Mlx	0
Kif5c	0	Mmp10	0	Dmd	0	Mlst8	0
Kif5b	0	Mmgt2	0	BRDN0000737643	0	Mlph	0
Kif5a	0	Mmgt1	0	Olfr1148	0	Mllt6	0
Kif4	0	Mme	0	Trpv3	0	Mllt4	0
Kif3c	0	Mmd2	0	Olfr1145	0	Mllt3	0
Kif3b	0	Mmd	0	Olfr1143	0	Mllt11	0
Kif3a	0	Mmadhc	0	Sh3glb1	0	Mllt10	0
Kif2c	0	Mmab	0	Olfr1141	0	Mlkl	0
Kif2b	0	Mmaa	0	Olfr1140	0	Mlip	0
Kif27	0	Mlxipl	0	4933430I17Rik	0	Mlf2	0
Kif26a	0	Mlxip	0	Zic4	0	Mlec	0
Kif24	0	Mlx	0	Mttp	0	Mlc1	0
Kif23	0	Mlst8	0	Upk1b	0	Mkx	0
Kif21b	0	Mllt6	0	Olfr1420	0	Mks1	0
Kif21a	0	Mllt4	0	Mbd3l1	0	Mkrn3	0
Kif20b	0	Mllt3	0	Mbd3l2	0	Mkrn2os	0
Kif20a	0	Mllt11	0	Rpl41	0	Mkrn1	0
Kif1c	0	Mllt10	0	BRDN0000737642	0	Mknk1	0
Kif1b	0	Mllt1	0	Mff	0	Mkln1	0
Kif19a	0	Mlkl	0	Rhov	0	Mkl2	0
Kif18b	0	Mlip	0	Rlbp1	0	Mkl1	0
Kif18a	0	Mlh1	0	Rabgef1	0	Mkks	0
Kif17	0	Mlf2	0	Bcat2	0	Mki67	0
Kif16b	0	Mlf1	0	Bcat1	0	Mixl1	0
Kif15	0	Mlec	0	Dusp28	0	Mitf	0
Kif13b	0	Mlc1	0	Cib3	0	Mitd1	0
Kif12	0	Mlana	0	1700034E13Rik	0	Misp	0
Kif11	0	Mks1	0	Lenep	0	Mis18bp1	0
Kidins220	0	Mkrn2os	0	3300002I08Rik	0	Mis18a	0
Khsrp	0	Mkrn1	0	Exph5	0	Mis12	0
Khdrbs3	0	Mknk2	0	Cib4	0	Mipol1	0
Khdrbs1	0	Mknk1	0	Dusp21	0	Mipep	0
Khdc3	0	Mkln1	0	Dusp22	0	Mip	0
Khdc1c	0	Mkl2	0	Dusp23	0	Mios	0
Khdc1b	0	Mkl1	0	Cep104	0	Minpp1	0
Khdc1a	0	Mkks	0	Dusp26	0	Minos1	0
Kera	0	Mki67	0	Dusp27	0	Mink1	0
Kel	0	Mixl1	0	Ywhaq	0	Mina	0
Keg1	0	Mitf	0	Myod1	0	Milr1	0
Keap1	0	Mitd1	0	Zbtb12	0	Mill1	0
Kdsr	0	Misp	0	Trex2	0	Mif4gd	0
Kdm8	0	Mis18bp1	0	Trex1	0	Mif	0
Kdm5d	0	Mis18a	0	Zbtb11	0	Mier3	0
Kdm5c	0	Mis12	0	Aldh8a1	0	Mier1	0
Kdm5b	0	Mipol1	0	Peli2	0	Mien1	0
Kdm5a	0	Mipep	0	4933434E20Rik	0	Mief2	0

Kdm4d	0	Mip	0	Tg	0	Mief1	0
Kdm4c	0	Miox	0	Phf5a	0	Mid2	0
Kdm4b	0	Mios	0	2010109I03Rik	0	Mid1ip1	0
Kdm4a	0	Minpp1	0	Rai14	0	Mid1	0
Kdm2b	0	Minos1	0	Tmem170b	0	Micu3	0
Kdm2a	0	Mink1	0	BRDN0000738005	0	Micu1	0
Kdm1a	0	Mina	0	Gpr1	0	Micall2	0
Kdelr1	0	Milr1	0	Gpr6	0	Micall1	0
Kdelc2	0	Mill1	0	Olfr775	0	Micalcl	0
Kdelc1	0	Miip	0	Gpr4	0	Mical3	0
Kctd9	0	Mif4gd	0	Cmya5	0	Mical2	0
Kctd8	0	Mif	0	Prodh2	0	Mia2	0
Kctd7	0	Mier3	0	Khdrbs1	0	Mia	0
Kctd5	0	Mier1	0	Rfpl4b	0	Mgst2	0
Kctd3	0	Mief2	0	Khdrbs3	0	Mgst1	0
Kctd21	0	Mief1	0	Khdrbs2	0	Mgrn1	0
Kctd2	0	Mid2	0	Ren1	0	Mgp	0
Kctd18	0	Micalcl	0	D15Ertd621e	0	Mgmt	0
Kctd16	0	Mical3	0	Gm12942	0	Mgme1	0
Kctd15	0	Mical2	0	Mtfr1l	0	Mgll	0
Kctd13	0	Mib1	0	Csf3r	0	Mgea5	0
Kctd12b	0	Mia2	0	Chdh	0	Mgat5b	0
Kctd11	0	Mia	0	Plin4	0	Mgat5	0
Kctd1	0	Mgst3	0	Plin5	0	Mgat4e	0
Kcp	0	Mgst2	0	1700028P14Rik	0	Mgat4d	0
Kcnv1	0	Mgst1	0	Plin3	0	Mgat4b	0
Kcnt2	0	Mgrn1	0	Mctp2	0	Mgat4a	0
Kcnt1	0	Mgp	0	Zfp937	0	Mgat3	0
Kcns2	0	Mgmt	0	Zfp935	0	Mgat2	0
Kcns1	0	Mgme1	0	Zfp932	0	Mgat1	0
Kcnrg	0	Mgl2	0	Zfp933	0	Mgarp	0
Kcnq4	0	Mgat5	0	Zfp931	0	Mgam	0
Kcnq3	0	Mgat4d	0	Tfb2m	0	Mfsd9	0
Kcnq2	0	Mgat4c	0	Colec11	0	Mfsd8	0
Kcnq1	0	Mgat4b	0	Zfp938	0	Mfsd7c	0
Kcnn4	0	Mgat4a	0	Safb2	0	Mfsd7b	0
Kcnn3	0	Mgat3	0	Gcc2	0	Mfsd7a	0
Kcnn2	0	Mgat2	0	Gm9112	0	Mfsd6l	0
Kcnn1	0	Mgat1	0	Map4k2	0	Mfsd6	0
Kcnmb4	0	Mfsd9	0	Cep112	0	Mfsd3	0
Kcnmb3	0	Mfsd8	0	Map4k5	0	Mfsd2a	0
Kcnmb2	0	Mfsd7a	0	Nup188	0	Mfsd12	0
Kcnmb1	0	Mfsd6	0	Rnf150	0	Mfsd11	0
Kcnma1	0	Mfsd2a	0	Cuzd1	0	Mfsd1	0
Kcnk9	0	Mfsd12	0	Map4k4	0	Mfrp	0
Kcnk7	0	Mfsd11	0	Chd1	0	Mfng	0
Kcnk6	0	Mfsd10	0	Spidr	0	Mfn2	0
Kcnk5	0	Mfsd1	0	Zfp143	0	Mfi2	0
Kcnk4	0	Mfrp	0	Chd2	0	Mfhas1	0
Kcnk3	0	Mfng	0	Spats2l	0	Mff	0
Kcnk2	0	Mfn2	0	Chd6	0	Mfap5	0
Kcnk18	0	Mfn1	0	Chd9	0	Mfap3	0
Kcnk16	0	Mfi2	0	Chd8	0	Mfap1b	0
Kcnk13	0	Mfhas1	0	BRDN0000738134	0	Mfap1a	0
Kcnk10	0	Mfge8	0	Ralyl	0	Mex3d	0
Kcnk1	0	Mff	0	Ankmy1	0	Mex3b	0
Kcnj8	0	Mfap5	0	Nob1	0	Mettl8	0
Kcnj6	0	Mfap3l	0	Ppp6r2	0	Mettl7b	0
Kcnj2	0	Mfap3	0	Ssxb10	0	Mettl5	0
Kcnj16	0	Mfap2	0	Ppp6r1	0	Mettl3	0
Kcnj15	0	Mfap1b	0	BRDN0000738330	0	Mettl25	0
Kcnj14	0	Mex3d	0	BRDN0000737649	0	Mettl21e	0
Kcnj13	0	Mex3a	0	Ywhae	0	Mettl21b	0
Kcnj12	0	Mettl8	0	BRDN0000737511	0	Mettl18	0
Kcnj10	0	Mettl7b	0	Ntsr1	0	Mettl17	0
Kcnip4	0	Mettl7a3	0	Ipo11	0	Mettl15	0
Kcnip3	0	Mettl7a1	0	Ipo13	0	Mettl14	0
Kcnip1	0	Mettl6	0	Areg	0	Mettl13	0
Kcnh8	0	Mettl5	0	Olfr1426	0	Mettl10	0
Kcnh7	0	Mettl3	0	2900026A02Rik	0	Mettl1	0
Kcnh6	0	Mettl23	0	Ing4	0	Metap2	0
Kcnh5	0	Mettl21e	0	Erp44	0	Metap1d	0
Kcnh3	0	Mettl21c	0	Tmem229a	0	Metap1	0
Kcnh2	0	Mettl20	0	Tmem229b	0	Mest	0
Kcnh1	0	Mettl2	0	Ing5	0	Mesp1	0
Kcnf1	0	Mettl18	0	Fyttd1	0	Mesdc1	0
Kcne3	0	Mettl16	0	Fh1	0	Mertk	0
Kcne2	0	Mettl15	0	Tmem106c	0	Mepe	0
Kcne1l	0	Mettl14	0	Flcn	0	Mepce	0
Kcne1	0	Mettl13	0	BRDN0000738131	0	Mep1b	0
Kcnd1	0	Mettl11b	0	BRDN0000737725	0	Meox1	0
Kcnc1	0	Mettl1	0	Tmem218	0	Men1	0

Kcnb2	0
Kcnb1	0
Kcnab3	0
Kcnab2	0
Kcna7	0
Kcna6	0
Kcna5	0
Kcna4	0
Kcna2	0
Kcna10	0
Kcna1	0
Kcmf1	0
Kbtbd8	0
Kbtbd4	0
Kbtbd3	0
Kbtbd2	0
Kbtbd13	0
Kbtbd12	0
Kbtbd11	0
Kazn	0
Kazald1	0
Katnbl1	0
Katnb1	0
Katnal1	0
Katna1	0
Kat8	0
Kat7	0
Kat6b	0
Kat6a	0
Kat5	0
Kat2b	0
Kat2a	0
Kars	0
Kap	0
Kansl3	0
Kansl2	0
Kank4	0
Kank3	0
Kank2	0
Jun	0
Jtb	0
Jsrp1	0
Jrkl	0
Jrk	0
Jph4	0
Jph3	0
Jph2	0
Jph1	0
Josd1	0
Jmy	0
Jmjd8	0
Jmjd7	0
Jmjd6	0
Jmjd1c	0
Jdp2	0
Jchain	0
Jarid2	0
Jam3	0
Jam2	0
Jak3	0
Jag2	0
Jag1	0
Jade3	0
Jade2	0
Izumo4	0
Izumo3	0
Izumo2	0
Izumo1	0
Iyd	0
Iws1	0
Ivns1abp	0
Itsn1	0
Itpripl1	0
Itpr3	0
Itpkb	0
Itpka	0
Itpk1	0
Itpa	0
Itm2c	0
Itm2b	0
Itm2a	0
Itln1	0
Metrnl	0
Methig1	0
Metap2	0
Metap1d	0
Metap1	0
Met	0
Mest	0
Mesp1	0
Mesdc2	0
Mesdc1	0
Mepe	0
Mepce	0
Mep1b	0
Meox2	0
Memo1	0
Meis3	0
Meis2	0
Meis1	0
Meiob	0
Meikin	0
Meig1	0
Mei1	0
Megf9	0
Megf8	0
Megf10	0
Mefv	0
Mef2d	0
Mef2b	0
Mef2a	0
Medag	0
Med9	0
Med8	0
Med7	0
Med4	0
Med31	0
Med29	0
Med27	0
Med26	0
Med25	0
Med24	0
Med23	0
Med22	0
Med21	0
Med20	0
Med19	0
Med17	0
Med16	0
Med15	0
Med14	0
Med13	0
Med12	0
Med11	0
Med10	0
Med1	0
Mecr	0
Mecp2	0
Meaf6	0
Mea1	0
Me3	0
Me2	0
Me1	0
Mdn1	0
Mdm2	0
Mdm1	0
Mdk	0
Mdh2	0
Mdh1b	0
Mdga2	0
Mdc1	0
Mcur1	0
Mcu	0
Mcts1	0
Mctp2	0
Mctp1	0
Mcrs1	0
Mcpt9	0
Mcpt8	0
Mcpt2	0
Mcpt1	0
Mcoln3	0
Mcoln1	0
Mcmdc2	0
Agfg2	0
Mlec	0
Rgs19	0
Tmem88b	0
Rbm4b	0
Foxc2	0
Gm815	0
Xlr3b	0
Zic3	0
Hbb-bh2	0
Chit1	0
Engase	0
BRDN0000738136	0
Thrap3	0
Gm10639	0
Tecpr2	0
Praf2	0
Clcn3	0
C78339	0
Olfr1020	0
BRDN0000738137	0
Cyp2d12	0
Cyp2d10	0
Esp3	0
Dicer1	0
Gcc1	0
Sspn	0
BC030307	0
Aarsd1	0
Plac8l1	0
Clcn2	0
Ebp	0
Col23a1	0
Zic1	0
Cxcl13	0
Cxcl12	0
Cxcl15	0
Cxcl14	0
BRDN0000737720	0
Ssxb5	0
Tgfa	0
Ssxb1	0
Ssxb3	0
Gm13051	0
Racgap1	0
Bhlhe22	0
B3galt1	0
Neb	0
Bysl	0
Osr2	0
Bhlhe23	0
Xlr3a	0
Cnpy4	0
Ifltd1	0
BRDN0000737978	0
Cdc42se1	0
Nes	0
Dtx1	0
Smg5	0
Smg7	0
Smg6	0
Smg1	0
Il23a	0
Adh5	0
Adh4	0
Pi16	0
Ankfy1	0
Smg9	0
Smg8	0
Met	0
Lao1	0
Nrep	0
Edem2	0
Ddx18	0
Slc28a3	0
Atp2c2	0
Atp2c1	0
Slmo1	0
Alpk1	0
BRDN0000738303	0
Kynu	0
Npy4r	0
Memo1	0
Meis2	0
Meiob	0
Mei4	0
Mei1	0
Megf8	0
Mefv	0
Mef2b	0
Mef2a	0
Med9	0
Med8	0
Med7	0
Med4	0
Med31	0
Med30	0
Med28	0
Med27	0
Med26	0
Med25	0
Med24	0
Med23	0
Med22	0
Med21	0
Med20	0
Med19	0
Med18	0
Med17	0
Med16	0
Med15	0
Med14	0
Med13l	0
Med13	0
Med12	0
Med11	0
Med10	0
Med1	0
Mecr	0
Mea1	0
Me2	0
Mdn1	0
Mdm2	0
Mdm1	0
Mdk	0
Mdh2	0
Mdh1b	0
Mdga2	0
Mdga1	0
Mdfic	0
Mcts1	0
Mctp2	0
Mcrs1	0
Mcpt9	0
Mcpt8	0
Mcpt4	0
Mcph1	0
Mcoln3	0
Mcmdc2	0
Mcmbp	0
Mcm9	0
Mcm8	0
Mcm7	0
Mcm6	0
Mcm5	0
Mcm3ap	0
Mcm3	0
Mcm2	0
Mcm10	0
Mcl1	0
Mchr1	0
Mcfd2	0
Mcf2l	0
Mcf2	0
Mcemp1	0
Mcee	0
Mcc	0
Mcat	0
Mc4r	0
Mc2r	0
Mc1r	0
Mbp	0
Mboat7	0
Mboat1	0

Itk	0	Mcmbp	0	Fndc9	0	Mbnl1	0
Itih4	0	Mcm9	0	Fcer2a	0	Mbl2	0
Itih2	0	Mcm8	0	Fndc4	0	Mbl1	0
Itgbl1	0	Mcm7	0	Fndc5	0	Mbip	0
Itgb8	0	Mcm6	0	Gpihbp1	0	Mbd6	0
Itgb7	0	Mcm5	0	Fndc7	0	Mbd5	0
Itgb5	0	Mcm4	0	Fndc1	0	Mbd4	0
Itgb2l	0	Mcm3ap	0	2810428I15Rik	0	Mbd3l2	0
Itgb1bp1	0	Mcm3	0	Gja3	0	Mbd3	0
Itgax	0	Mcm2	0	Gm4214	0	Mbd2	0
Itgav	0	Mcm10	0	Shoc2	0	Mb21d2	0
Itgam	0	Mcl1	0	Msln	0	Mb	0
Itgal	0	Mcidas	0	Mrpl44	0	Maz	0
Itgae	0	Mcf2	0	Dpysl3	0	Max	0
Itga9	0	Mcemp1	0	Olfr1454	0	Mavs	0
Itga8	0	Mcee	0	Fer1l5	0	Mau2	0
Itga7	0	Mccc2	0	Xxylt1	0	Matr3	0
Itga6	0	Mccc1	0	Gja6	0	Matn3	0
Itga5	0	Mcc	0	Egr2	0	Matn1	0
Itga2b	0	Mcat	0	Egr3	0	Mat2b	0
Itga2	0	Mc5r	0	Egr1	0	Mat2a	0
Itga11	0	Mc2r	0	Slc7a14	0	Mat1a	0
Itga1	0	Mc1r	0	Tradd	0	Mastl	0
Itfg3	0	Mbtps1	0	Uhrf1bp1	0	Mast4	0
Itfg1	0	Mbp	0	Me1	0	Mast3	0
Itch	0	Mboat7	0	Abhd4	0	Mast1	0
Isy1	0	Mboat4	0	Smgc	0	Masp2	0
Isx	0	Mbnl2	0	Me2	0	Mas1	0
Ispd	0	Mbnl1	0	Col24a1	0	Marveld1	0
Isoc2b	0	Mblac2	0	Fam193b	0	Mars	0
Isoc2a	0	Mblac1	0	Tnfrsf13b	0	Mark4	0
Isoc1	0	Mbl1	0	Wdr7	0	Mark3	0
Ism1	0	Mbip	0	BRDN0000737588	0	Mark2	0
Isl2	0	Mbd6	0	Wdr6	0	Marf1	0
Isl1	0	Mbd5	0	BRDN0000737615	0	Marco	0
Isg20l2	0	Mbd4	0	Fam175a	0	Marcksl1	0
Isg20	0	Mbd3l2	0	Kcnk5	0	Marcks	0
Isg15	0	Mbd3	0	Ccdc169	0	March8	0
Iscu	0	Mbd2	0	Gm4297	0	March7	0
Isca2	0	Mbd1	0	Dvl1	0	March6	0
Irx6	0	Mb21d1	0	Bsph2	0	March5	0
Irx5	0	Maz	0	Wdr3	0	March4	0
Irx4	0	Max	0	Bsph1	0	March2	0
Irx3	0	Mavs	0	Tnfrsf13c	0	March11	0
Irx2	0	Mau2	0	Aasdhppt	0	March10	0
Irx1	0	Matr3	0	Ccdc77	0	March1	0
Irs4	0	Matn4	0	BRDN0000737589	0	Marc2	0
Irs3	0	Matn3	0	Rasd1	0	Marc1	0
Irs2	0	Matk	0	Ccdc71	0	Mapre3	0
Irs1	0	Mat2b	0	Kcnk6	0	Mapre1	0
Irgq	0	Mat2a	0	Slc32a1	0	Mapkapk3	0
Irgm2	0	Mat1a	0	Sgtb	0	Mapkap1	0
Irgm1	0	Mastl	0	BRDN0000737973	0	Mapk8ip3	0
Irgc1	0	Mast4	0	Ccdc78	0	Mapk8ip2	0
Irg1	0	Mast3	0	Ccdc79	0	Mapk8ip1	0
Irf7	0	Mast1	0	Trpc2	0	Mapk8	0
Irf6	0	Masp2	0	Trpc3	0	Mapk7	0
Irf5	0	Mas1	0	Trpc1	0	Mapk6	0
Irf4	0	Marveld1	0	Trpc6	0	Mapk4	0
Irf3	0	Mars2	0	Podnl1	0	Mapk3	0
Irf2bpl	0	Mars	0	1700113H08Rik	0	Mapk1ip1l	0
Irf2bp1	0	Mark4	0	Ccdc125	0	Mapk15	0
Irf2	0	Mark2	0	Vmn1r18	0	Mapk14	0
Irf1	0	Mark1	0	Gm3558	0	Mapk12	0
Ireb2	0	Marf1	0	Tspan18	0	Mapk11	0
Irak4	0	Marcksl1	0	Nfatc1	0	Mapk10	0
Irak3	0	March8	0	Nfatc2	0	Mapk1	0
Irak2	0	March7	0	Nfatc3	0	Map9	0
Irak1bp1	0	March6	0	Nfatc4	0	Map7d2	0
Irak1	0	March5	0	B3galnt2	0	Map7	0
Iqub	0	March11	0	B3galnt1	0	Map6d1	0
Iqsec3	0	March10	0	Larp1	0	Map6	0
Iqsec2	0	Marc2	0	Tspan10	0	Map4k3	0
Iqgap3	0	Marc1	0	Zfp579	0	Map4k2	0
Iqgap2	0	Mapt	0	Tspan12	0	Map4	0
Iqgap1	0	Mapre3	0	Olfr285	0	Map3k8	0
Iqcj	0	Mapre2	0	Celsr3	0	Map3k7cl	0
Iqch	0	Mapkbp1	0	Celsr2	0	Map3k7	0
Iqcg	0	Mapkapk3	0	Celsr1	0	Map3k5	0
Iqcf5	0	Mapkapk2	0	Tspan17	0	Map3k4	0
Iqcf4	0	Mapkap1	0	Sowaha	0	Map3k3	0
Iqcf3	0	Mapk9	0	Sowahb	0	Map3k19	0

Iqcf1	0
Iqce	0
Iqcc	0
Iqca	0
Ippk	0
Ipp	0
Ipo9	0
Ipo8	0
Ipo7	0
Ipo13	0
Ipo11	0
Ipmk	0
Ipcef1	0
Ip6k3	0
Ip6k1	0
Ints9	0
Ints8	0
Ints7	0
Ints6	0
Ints5	0
Ints4	0
Ints3	0
Ints2	0
Ints10	0
Ints1	0
Insrr	0
Insr	0
Insm2	0
Insl5	0
Insig2	0
Insig1	0
Ins1	0
Inppl1	0
Inpp5k	0
Inpp5j	0
Inpp5e	0
Inpp5b	0
Inpp4a	0
Ino80d	0
Ino80c	0
Ino80b	0
Ino80	0
Inmt	0
Inip	0
Inhba	0
Inha	0
Ing5	0
Ing4	0
Ing3	0
Ing1	0
Inf2	0
Incenp	0
Inca1	0
Inadl	0
Ina	0
Impg2	0
Impdh2	0
Impact	0
Impa2	0
Impa1	0
Imp3	0
Immt	0
Ilvbl	0
Iltifb	0
Ilkap	0
Ilk	0
Ilf3	0
Ilf2	0
Ildr2	0
Ildr1	0
Il9	0
Il7r	0
Il7	0
Il6ra	0
Il5ra	0
Il4ra	0
Il4i1	0
Il4	0
Il34	0
Il33	0
Il31ra	0
Il31	0
Mapk8ip3	0
Mapk8ip2	0
Mapk8ip1	0
Mapk7	0
Mapk6	0
Mapk4	0
Mapk3	0
Mapk1ip1l	0
Mapk15	0
Mapk13	0
Mapk11	0
Mapk10	0
Mapk1	0
Map7d2	0
Map4k5	0
Map4k3	0
Map4k2	0
Map4k1	0
Map4	0
Map3k9	0
Map3k8	0
Map3k7	0
Map3k5	0
Map3k4	0
Map3k19	0
Map3k13	0
Map3k12	0
Map3k1	0
Map2k4	0
Map2k2	0
Map2k1	0
Map2	0
Map1s	0
Map1lc3b	0
Map1lc3a	0
Map1b	0
Maob	0
Maoa	0
Maneal	0
Manea	0
Manbal	0
Man2c1	0
Man2b2	0
Man2a2	0
Man1a2	0
Mamld1	0
Maml2	0
Maml1	0
Mamdc2	0
Malt1	0
Malsu1	0
Mall	0
Mal	0
Mak16	0
Mak	0
Magohb	0
Magix	0
Magi1	0
Magel2	0
Magee1	0
Maged1	0
Mageb5	0
Mageb4	0
Mageb3	0
Mageb18	0
Mageb1	0
Magea8	0
Magea6	0
Magea5	0
Magea3	0
Magea10	0
Mag	0
Mafk	0
Mafg	0
Mafb	0
Mafa	0
Maf1	0
Maf	0
Mael	0
Maea	0
Madcam1	0
Mad2l1	0
Sowahc	0
Sowahd	0
Insrr	0
BRDN0000738048	0
Snx6	0
Tmsb15b2	0
Helb	0
BRDN0000737408	0
Folr1	0
Folr2	0
Olfr1390	0
Ankrd7	0
Epgn	0
Ankrd2	0
Ripply2	0
BRDN0000737971	0
Flad1	0
Ankrd9	0
Omt2a	0
Nat10	0
Marcks	0
Ndufa10	0
Ndufa11	0
BRDN0000738196	0
Ndufa13	0
Wee2	0
Wee1	0
Gm4906	0
Olfr497	0
Slc7a11	0
Olfr494	0
Olfr491	0
Olfr490	0
Xylb	0
Hsd11b1	0
BRDN0000737729	0
Resp18	0
BRDN0000738091	0
Gpatch11	0
BRDN0000738093	0
Oog4	0
Oog3	0
Oog2	0
BRDN0000738097	0
Cyp11b2	0
Det1	0
Esrra	0
Abcd3	0
Abcd2	0
Abcd4	0
Clstn2	0
Clstn3	0
Rapgef4	0
Tmem130	0
Tmem131	0
Pomp	0
Slirp	0
Dpp9	0
Tmem138	0
Tmem139	0
Prss3	0
Mocs3	0
Ndufs1	0
Ndufaf7	0
Ndufaf6	0
Ndufs5	0
Ndufs6	0
Pomc	0
Ndufs8	0
Psenen	0
Atp6v1b2	0
B3gat2	0
Lrrc32	0
Meox2	0
Pomk	0
Lyplal1	0
Olfr720	0
Olfr723	0
Olfr722	0
Olfr725	0
Olfr724	0
Olfr726	0
Map3k15	0
Map3k13	0
Map3k12	0
Map3k11	0
Map3k1	0
Map2k7	0
Map2k6	0
Map2k5	0
Map2k4	0
Map2k3	0
Map2k2	0
Map2k1	0
Map2	0
Map1lc3b	0
Map10	0
Maob	0
Maoa	0
Mansc4	0
Mansc1	0
Manf	0
Manea	0
Manba	0
Man2c1	0
Man2b2	0
Man2a2	0
Man1c1	0
Man1b1	0
Man1a2	0
Mamstr	0
Maml2	0
Maml1	0
Mamdc4	0
Mamdc2	0
Malt1	0
Malsu1	0
Mall	0
Mal	0
Mak	0
Magoh	0
Magix	0
Magi3	0
Magi2	0
Magi1	0
Magel2	0
Mageh1	0
Magee2	0
Magee1	0
Maged2	0
Mageb5	0
Mageb4	0
Mageb3	0
Mageb16	0
Mageb1	0
Magea8	0
Magea3	0
Magea2	0
Magea10	0
Magea1	0
Mafg	0
Mafb	0
Mafa	0
Maf1	0
Mael	0
Maea	0
Madd	0
Mad2l1bp	0
Mad2l1	0
Mad1l1	0
Macrod2	0
Macf1	0
Macc1	0
Mab21l3	0
Mab21l1	0
M6pr	0
M1ap	0
Lzts2	0
Lzts1	0
Lztr1	0
Lztfl1	0
Lzic	0
Lyzl6	0
Lyzl1	0

Il3	0
Il2rg	0
Il2rb	0
Il2ra	0
Il27ra	0
Il27	0
Il24	0
Il23a	0
Il22ra2	0
Il22ra1	0
Il22	0
Il21r	0
Il20rb	0
Il20ra	0
Il1rn	0
Il1rl2	0
Il1rl1	0
Il1rapl1	0
Il1rap	0
Il1r2	0
Il1r1	0
Il1f9	0
Il1f8	0
Il1f6	0
Il1f5	0
Il1f10	0
Il1a	0
Il19	0
Il18rap	0
Il18r1	0
Il18	0
Il17ra	0
Il17d	0
Il17b	0
Il17a	0
Il16	0
Il15ra	0
Il15	0
Il13ra1	0
Il12rb2	0
Il12a	0
Il11	0
Il10rb	0
Il10ra	0
Il10	0
Ikzf5	0
Ikzf4	0
Ikzf3	0
Ikzf2	0
Ikzf1	0
Ikbkg	0
Ikbke	0
Ikbkb	0
Ikbkap	0
Ikbip	0
Ik	0
Igsf9b	0
Igsf6	0
Igsf5	0
Igsf3	0
Igsf11	0
Igsf10	0
Igsf1	0
Iglon5	0
Ighmbp2	0
Igfn1	0
Igflr1	0
Igfbpl1	0
Igfbp6	0
Igfbp5	0
Igfbp4	0
Igfbp3	0
Igfbp2	0
Igfbp1	0
Igfals	0
Igf2r	0
Igf2bp3	0
Igf2bp1	0
Igf2	0
Igf1r	0
Igf1	0
Igdcc4	0
Mad1l1	0
Macrod1	0
Macf1	0
Macc1	0
Mab21l1	0
M6pr	0
M1ap	0
Lzts2	0
Lzts1	0
Lztr1	0
Lztfl1	0
Lyzl6	0
Lyzl4	0
Lyzl1	0
Lyve1	0
Lyst	0
Lysmd4	0
Lysmd2	0
Lysmd1	0
Lyrm9	0
Lyrm7	0
Lyrm2	0
Lyrm1	0
Lyplal1	0
Lypla2	0
Lypla1	0
Lypd8	0
Lypd5	0
Lypd4	0
Lypd2	0
Lypd1	0
Lyn	0
Lyg2	0
Lyg1	0
Lyar	0
Ly9	0
Ly86	0
Ly75	0
Ly6k	0
Ly6i	0
Ly6g6f	0
Ly6g6e	0
Ly6g6d	0
Ly6g6c	0
Ly6g5c	0
Ly6g5b	0
Ly6e	0
Ly6d	0
Lxn	0
Lvrn	0
Luzp1	0
Lurap1l	0
Luc7l2	0
Luc7l	0
Ltv1	0
Ltn1	0
Ltk	0
Ltf	0
Ltc4s	0
Ltbr	0
Ltbp1	0
Ltb4r2	0
Ltb4r1	0
Ltb	0
Lta	0
Lst1	0
Lsp1	0
Lsmem1	0
Lsm8	0
Lsm7	0
Lsm6	0
Lsm5	0
Lsm4	0
Lsm2	0
Lsm14b	0
Lsm12	0
Lsm11	0
Lsm10	0
Lsm1	0
Lsg1	0
Lrwd1	0
Lrtm1	0
Olfr729	0
Lrrc34	0
BRDN0000738237	0
BRDN0000738236	0
Spata31	0
Rabl3	0
Spata33	0
Spata32	0
Corin	0
Vmn1r205	0
Vmn1r206	0
Naa20	0
Xrcc5	0
Vmn1r201	0
Vmn1r202	0
Gm5868	0
Vmn1r208	0
Vmn1r209	0
Gm7714	0
BRDN0000737975	0
Creg2	0
Yipf2	0
BRDN0000738083	0
Ncf4	0
Usb1	0
Atp1b2	0
Atp1b3	0
Atp1b1	0
Tmem60	0
Lrrc26	0
Lrrc25	0
Lrrc24	0
Arfgap2	0
Clptm1	0
1700112E06Rik	0
Vmn2r63	0
Mvb12b	0
Abcc8	0
Mvb12a	0
Lrrc29	0
Lrrc28	0
Cnih1	0
Ercc6l	0
Mad2l1bp	0
BRDN0000738084	0
Ppm1j	0
Gas2l2	0
Cep350	0
Lima1	0
Gm14434	0
Rbm5	0
Gm14431	0
Scgb3a1	0
Olfr1428	0
Ugt1a6b	0
Ugt1a6a	0
Olfr1359	0
Ang2	0
Ccnc	0
Olfr1352	0
Prb1	0
Olfr1350	0
Olfr1423	0
Ccnh	0
Olfr1425	0
Ccnj	0
Olfr1355	0
BRDN0000738089	0
Nrsn1	0
Hnrnpa0	0
Selk	0
Rin1	0
Siva1	0
Rin3	0
Perp	0
F830045P16Rik	0
Fam73a	0
Zap70	0
Mlkl	0
Cep57	0
Wbp2	0
Wbp1	0
Lyz2	0
Lyve1	0
Lyst	0
Lysmd4	0
Lysmd2	0
Lysmd1	0
Lyrm9	0
Lyrm7	0
Lyrm4	0
Lypla2	0
Lypla1	0
Lypd8	0
Lypd6b	0
Lypd6	0
Lypd5	0
Lypd3	0
Lypd2	0
Lypd1	0
Lyn	0
Lyl1	0
Lyg2	0
Lyg1	0
Lyar	0
Ly86	0
Ly75	0
Ly6g6e	0
Ly6g6d	0
Ly6g5c	0
Ly6g5b	0
Ly6d	0
Ly6c2	0
Ly6c1	0
Lvrn	0
Luzp1	0
Lum	0
Luc7l2	0
Luc7l	0
Ltv1	0
Ltn1	0
Ltc4s	0
Ltbp4	0
Ltbp1	0
Ltb4r1	0
Ltb	0
Lta4h	0
Lta	0
Lsr	0
Lsm7	0
Lsm6	0
Lsm5	0
Lsm4	0
Lsm3	0
Lsm2	0
Lsm14b	0
Lsm11	0
Lsm10	0
Lsg1	0
Lrwd1	0
Lrtm1	0
Lrsam1	0
Lrrtm4	0
Lrrtm3	0
Lrrtm1	0
Lrrn4cl	0
Lrrn4	0
Lrrn3	0
Lrrn2	0
Lrrn1	0
Lrrk2	0
Lrrk1	0
Lrriq3	0
Lrriq1	0
Lrrfip1	0
Lrrd1	0
Lrrcc1	0
Lrrc9	0
Lrrc8c	0
Lrrc8b	0
Lrrc8a	0
Lrrc75b	0
Lrrc75a	0
Lrrc74a	0

Igdcc3	0
Igbp1b	0
Igbp1	0
Ift88	0
Ift81	0
Ift74	0
Ift57	0
Ift52	0
Ift46	0
Ift43	0
Ift22	0
Ift20	0
Ift172	0
Ift140	0
Ift122	0
Ifnz	0
Ifnlr1	0
Ifnl3	0
Ifnl2	0
Ifnk	0
Ifng	0
Ifnb1	0
Ifnar2	0
Ifnar1	0
Ifna9	0
Ifna7	0
Ifna6	0
Ifna5	0
Ifna4	0
Ifna16	0
Ifna15	0
Ifna14	0
Ifna13	0
Ifna12	0
Ifna11	0
Ifltd1	0
Ifitm6	0
Ifitm5	0
Ifitm3	0
Ifitm2	0
Ifitm1	0
Ifit3	0
Ifit2	0
Ifit1	0
Ifi47	0
Ifi44	0
Ifi35	0
Ifi30	0
Ifi27l2b	0
Ifi27l2a	0
Ifi27	0
Ifi204	0
Ifi203	0
Ifi202b	0
Iffo2	0
Iffo1	0
Ier5l	0
Ier5	0
Ier3ip1	0
Ier3	0
Ier2	0
Idua	0
Ids	0
Ido2	0
Ido1	0
Idnk	0
Idi1	0
Idh3g	0
Idh3b	0
Idh2	0
Id4	0
Id2	0
Id1	0
Icosl	0
Icos	0
Icmt	0
Ick	0
Ice2	0
Ice1	0
Icam5	0
Icam4	0
Icam2	0
Lrsam1	0
Lrrtm4	0
Lrrtm3	0
Lrrtm1	0
Lrrn4cl	0
Lrrn3	0
Lrrn2	0
Lrrn1	0
Lrrk2	0
Lrrk1	0
Lrriq3	0
Lrriq1	0
Lrrfip2	0
Lrrfip1	0
Lrrc9	0
Lrrc8e	0
Lrrc8c	0
Lrrc8a	0
Lrrc75a	0
Lrrc74b	0
Lrrc74a	0
Lrrc72	0
Lrrc7	0
Lrrc69	0
Lrrc66	0
Lrrc61	0
Lrrc6	0
Lrrc58	0
Lrrc56	0
Lrrc55	0
Lrrc4c	0
Lrrc4b	0
Lrrc49	0
Lrrc45	0
Lrrc43	0
Lrrc42	0
Lrrc41	0
Lrrc40	0
Lrrc4	0
Lrrc3b	0
Lrrc39	0
Lrrc38	0
Lrrc36	0
Lrrc34	0
Lrrc32	0
Lrrc30	0
Lrrc3	0
Lrrc29	0
Lrrc27	0
Lrrc25	0
Lrrc24	0
Lrrc19	0
Lrrc18	0
Lrrc15	0
Lrrc14b	0
Lrrc14	0
Lrrc10	0
Lrrc1	0
Lrr1	0
Lrpap1	0
Lrp6	0
Lrp5	0
Lrp2bp	0
Lrp1b	0
Lrp12	0
Lrp1	0
Lrmp	0
Lrit3	0
Lrit2	0
Lrit1	0
Lrig2	0
Lrif1	0
Lrg1	0
Lrfn4	0
Lrch4	0
Lrch3	0
Lrch2	0
Lrch1	0
Lrba	0
Lrat	0
Lppr4	0
Lppr3	0
Abcc3	0
Plekhn1	0
Wbp5	0
Wbp4	0
Gm8653	0
Tas2r114	0
Tas2r115	0
Asah1	0
Tas2r110	0
1500012F01Rik	0
C1galt1c1	0
Tas2r118	0
Cndp2	0
Olfr878	0
Gm5114	0
Rgcc	0
Olfr871	0
Olfr870	0
Olfr873	0
Olfr872	0
Olfr875	0
Olfr874	0
Olfr876	0
Cdc5l	0
Romo1	0
Abcc9	0
Gif	0
Gm6040	0
Higd2a	0
BRDN0000737407	0
Nppa	0
Rest	0
BC004004	0
Rinl	0
Alox5ap	0
Hist2h3b	0
Hnrnpab	0
Fbxo27	0
Gcnt2	0
Impact	0
Ptrh2	0
Ptrh1	0
Tmem74b	0
Olfr1311	0
Gulp1	0
Cyp8b1	0
Zcchc24	0
Taco1	0
Slc22a4	0
Slc22a5	0
Slc22a6	0
Slc22a7	0
Synj1	0
Slc22a1	0
Slc22a2	0
Slc22a3	0
Gm15386	0
Mkks	0
Dscr3	0
Slc22a8	0
2310039H08Rik	0
Pnpla8	0
Cib2	0
Dnajb8	0
Dnajb3	0
Dnajb2	0
Serpine3	0
Dnajb7	0
Dnajb6	0
Cdr2l	0
Dnajb4	0
Edf1	0
Wdr78	0
Ticam2	0
Cib1	0
Gm7849	0
Gstm1	0
Xrcc1	0
Wdr70	0
Xrcc3	0
Wdr72	0
Wdr75	0
Lrrc73	0
Lrrc72	0
Lrrc7	0
Lrrc69	0
Lrrc66	0
Lrrc63	0
Lrrc61	0
Lrrc59	0
Lrrc58	0
Lrrc57	0
Lrrc56	0
Lrrc55	0
Lrrc52	0
Lrrc4c	0
Lrrc4b	0
Lrrc49	0
Lrrc47	0
Lrrc46	0
Lrrc45	0
Lrrc43	0
Lrrc41	0
Lrrc40	0
Lrrc3b	0
Lrrc39	0
Lrrc38	0
Lrrc36	0
Lrrc34	0
Lrrc32	0
Lrrc30	0
Lrrc29	0
Lrrc27	0
Lrrc26	0
Lrrc25	0
Lrrc24	0
Lrrc19	0
Lrrc18	0
Lrrc17	0
Lrrc16a	0
Lrrc14b	0
Lrrc10b	0
Lrrc10	0
Lrr1	0
Lrpap1	0
Lrp6	0
Lrp4	0
Lrp2bp	0
Lrp2	0
Lrp1b	0
Lrp11	0
Lrp10	0
Lrp1	0
Lrit3	0
Lrit2	0
Lrit1	0
Lrig3	0
Lrig2	0
Lrig1	0
Lrguk	0
Lrg1	0
Lrfn4	0
Lrfn2	0
Lrfn1	0
Lrcol1	0
Lrch4	0
Lrch1	0
Lrba	0
Lrat	0
Lpxn	0
Lppr4	0
Lppr3	0
Lpo	0
Lpl	0
Lpin2	0
Lpcat2b	0
Lpcat2	0
Lpcat1	0
Lpar6	0
Lpar5	0
Lpar2	0
Lpar1	0
Loxl4	0
Loxl3	0

Ica1l	0
Ica1	0
Ibsp	0
Iba57	0
Iars2	0
Iars	0
Iah1	0
I830012O16Rik	0
Hypm	0
Hypk	0
Hyou1	0
Hykk	0
Hydin	0
Hyal6	0
Hyal5	0
Hyal3	0
Hyal2	0
Hyal1	0
Hvcn1	0
Hus1b	0
Hus1	0
Hunk	0
Htt	0
Htra4	0
Htra3	0
Htra2	0
Htra1	0
Htr7	0
Htr6	0
Htr5b	0
Htr5a	0
Htr3b	0
Htr3a	0
Htr2c	0
Htr1f	0
Htr1d	0
Htr1b	0
Htr1a	0
Htatsf1	0
Htatip2	0
Hspg2	0
Hspe1	0
Hspbp1	0
Hspbap1	0
Hspb9	0
Hspb6	0
Hspb3	0
Hspb11	0
Hspb1	0
Hspa9	0
Hspa8	0
Hspa5	0
Hspa4l	0
Hspa2	0
Hspa1l	0
Hspa14	0
Hspa13	0
Hspa12b	0
Hspa12a	0
Hsp90b1	0
Hsp90ab1	0
Hsh2d	0
Hsfy2	0
Hsf2bp	0
Hsf1	0
Hsd3b7	0
Hsd3b6	0
Hsd17b7	0
Hsd17b6	0
Hsd17b4	0
Hsd17b3	0
Hsd17b14	0
Hsd17b13	0
Hsd17b12	0
Hsd17b11	0
Hsd17b10	0
Hsd17b1	0
Hsd11b2	0
Hscb	0
Hsbp1l1	0
Hsbp1	0
Hs6st3	0
Lppr1	0
Lpp	0
Lpl	0
Lpin3	0
Lpin2	0
Lpin1	0
Lpcat2b	0
Lpcat2	0
Lpar6	0
Lpar5	0
Lpar4	0
Lpar3	0
Lpar2	0
Loxl3	0
Loxl2	0
Loxl1	0
Loxhd1	0
Lor	0
Lonrf3	0
Lonrf2	0
Lonp2	0
Lonp1	0
Loh12cr1	0
LOC100861615	0
LOC100502896	0
LOC100048884	0
Lnx1	0
Lnpep	0
Lnp	0
Lmx1a	0
Lmod3	0
Lmod2	0
Lmod1	0
Lmo7	0
Lmo4	0
Lmo3	0
Lmo2	0
Lmntd2	0
Lmnb2	0
Lmnb1	0
Lmna	0
Lmf2	0
Lmf1	0
Lmcd1	0
Lmbr1l	0
Lman2l	0
Lman1l	0
Lman1	0
Llgl1	0
Lkaaear1	0
Lix1l	0
Litaf	0
Lipt1	0
Lipo1	0
Lipn	0
Lipm	0
Lipk	0
Lingo4	0
Lingo3	0
Lingo2	0
Lin9	0
Lin7c	0
Lin7b	0
Lin54	0
Lin37	0
Lin28b	0
Lin28a	0
Lims2	0
Lims1	0
Lime1	0
Limch1	0
Lilrb4	0
Lilra5	0
Lig1	0
Lifr	0
Lif	0
Lias	0
Lhx9	0
Lhx4	0
Lhx3	0
Lhx1	0
Lhpp	0
Xrcc4	0
Wdr77	0
Xrcc6	0
Abcc4	0
Fam65c	0
Fam65b	0
Mgarp	0
Camsap2	0
Arl14epl	0
Camsap1	0
Ptcd2	0
Ptcd3	0
Apob	0
Ptcd1	0
Hs6st1	0
Hs6st2	0
Hs6st3	0
Mfsd7c	0
Kif5b	0
Gstm7	0
Aldh3b1	0
Slc16a1	0
Rpl23a	0
Tdrd3	0
Tdrd1	0
Tdrd7	0
Tdrd6	0
Tdrd5	0
Vmn1r163	0
Ctdp1	0
Alox12	0
Zfp68	0
Kif5c	0
BC021614	0
Diap1	0
Acad11	0
Abcc6	0
Zfp62	0
Zfp61	0
Zfp60	0
Nudcd2	0
Ahdc1	0
Sp3	0
Olfr715	0
Sp4	0
Mycbpap	0
Sp5	0
Ctgf	0
Sp6	0
Rnf111	0
Hbp1	0
Pusl1	0
BRDN0000738206	0
Izumo1r	0
Tor4a	0
Gfod2	0
Gfod1	0
BRDN0000737603	0
Gm16381	0
BRDN0000738229	0
Ppm1a	0
BRDN0000737860	0
Usp9x	0
Usp9y	0
BRDN0000737865	0
BRDN0000737864	0
BRDN0000737867	0
BRDN0000737866	0
BRDN0000737869	0
Mfap3l	0
Cybb	0
Mccc2	0
Mccc1	0
1700029J07Rik	0
Rnf14	0
Rnf17	0
Rnf10	0
Rnf11	0
BRDN0000737525	0
Srrt	0
Gm10439	0
Slamf8	0
Loxl2	0
Loxl1	0
Loxhd1	0
Lox	0
Lonrf2	0
Lonp2	0
Lonp1	0
LOC100862015	0
LOC100861615	0
LOC100502896	0
LOC100048884	0
LOC100038947	0
Lnx2	0
Lnpep	0
Lmx1a	0
Lmtk3	0
Lmtk2	0
Lmod3	0
Lmod2	0
Lmo4	0
Lmo2	0
Lmo1	0
Lmntd2	0
Lmnb2	0
Lmna	0
Lmln	0
Lmcd1	0
Lmbrd1	0
Lmbr1l	0
Lmbr1	0
Lman2	0
Llph	0
Llgl2	0
Llgl1	0
Lix1l	0
Lix1	0
Litaf	0
Lipt2	0
Lipt1	0
Lipn	0
Lipm	0
Lipk	0
Lipi	0
Lipe	0
Lipa	0
Lins	0
Lingo4	0
Lingo2	0
Lin9	0
Lin7c	0
Lin7a	0
Lin54	0
Lin37	0
Lin28b	0
Lin28a	0
Lims2	0
Lime1	0
Limd1	0
Lim2	0
Lilrb4	0
Lilra6	0
Lilra5	0
Lig4	0
Lig3	0
Lig1	0
Lif	0
Lias	0
Lhx9	0
Lhx6	0
Lhx5	0
Lhx3	0
Lhfpl5	0
Lhfpl4	0
Lhb	0
Lgsn	0
Lgr6	0
Lgmn	0
Lgi4	0
Lgi3	0
Lgi1	0
Lgals8	0
Lgals4	0

Hs6st2	0	Lhfpl5	0	Pear1	0	Lgals3bp	0
Hs6st1	0	Lhfpl4	0	Bst1	0	Lgals2	0
Hs3st6	0	Lhfpl3	0	Nckap5l	0	Lgals12	0
Hs3st5	0	Lhfpl2	0	Pilrb2	0	Lgals1	0
Hs3st4	0	Lhfp	0	Pilrb1	0	Letmd1	0
Hs3st2	0	Lhb	0	Slamf6	0	Letm2	0
Hs3st1	0	Lgr6	0	Srrd	0	Leprot	0
Hs2st1	0	Lgr5	0	Cyp3a16	0	Leng9	0
Hrsp12	0	Lgr4	0	BRDN0000738221	0	Leng1	0
Hrnr	0	Lgmn	0	Mcm4	0	Lenep	0
Hrk	0	Lgi3	0	Cyp3a11	0	Lemd3	0
Hrh4	0	Lgi1	0	Cyp3a13	0	Lemd2	0
Hrh3	0	Lgalsl	0	2210010C04Rik	0	Lelp1	0
Hrh1	0	Lgals7	0	Hn1	0	Lekr1	0
Hrg	0	Lgals3bp	0	Ankrd13d	0	Lefty2	0
Hrc	0	Lgals2	0	Clca1	0	Lef1	0
Hrasls5	0	Lgals12	0	Ankrd13b	0	Lect2	0
Hras	0	Lgals1	0	Ublcp1	0	Leap2	0
Hr	0	Lfng	0	Zfp329	0	Ldoc1l	0
Hpse2	0	Letmd1	0	Dfnb59	0	Ldlrap1	0
Hpse	0	Letm1	0	Zfp326	0	Ldlrad4	0
Hps6	0	Leprot	0	Foxd4	0	Ldlrad1	0
Hps5	0	Lepr	0	Zfp324	0	Ldlr	0
Hps3	0	Lep	0	Foxd2	0	Ldhc	0
Hps1	0	Leng9	0	Foxd3	0	Ldha	0
Hprt	0	Leng1	0	Gm21319	0	Ldb3	0
Hpn	0	Lenep	0	Habp4	0	Ldb1	0
Hpgds	0	Lemd2	0	BRDN0000737768	0	Ldah	0
Hpgd	0	Lemd1	0	Spata9	0	Lctl	0
Hpdl	0	Lelp1	0	Bpifc	0	Lcp2	0
Hpd	0	Lefty1	0	Slc39a6	0	Lcp1	0
Hpcal4	0	Lef1	0	Ddrgk1	0	Lcorl	0
Hpcal1	0	Lect1	0	Spata3	0	Lcn9	0
Hpca	0	Ldoc1l	0	Spata2	0	Lcn8	0
Hp1bp3	0	Ldlrap1	0	Spata1	0	Lcn5	0
Hoxd8	0	Ldlrad1	0	Spata7	0	Lcn4	0
Hoxd4	0	Ldlr	0	Spata6	0	Lcn3	0
Hoxd13	0	Ldhc	0	Spata5	0	Lcn2	0
Hoxd12	0	Ldhb	0	Arap3	0	Lcn12	0
Hoxd11	0	Ldhal6b	0	BRDN0000737527	0	Lcn11	0
Hoxd1	0	Ldha	0	Bhlha15	0	Lcn10	0
Hoxc9	0	Ldb2	0	Rab3ip	0	Lcmt2	0
Hoxc8	0	Lctl	0	Oc90	0	Lcmt1	0
Hoxc5	0	Lcp2	0	BRDN0000737769	0	Lck	0
Hoxc4	0	Lcp1	0	Esrp2	0	Lce3f	0
Hoxc13	0	Lcn8	0	Esrp1	0	Lce3e	0
Hoxc11	0	Lcn4	0	Vim	0	Lce3d	0
Hoxc10	0	Lcn3	0	Mzt1	0	Lce3c	0
Hoxb9	0	Lcn12	0	Arap2	0	Lce3b	0
Hoxb8	0	Lcn11	0	Myo7b	0	Lce1k	0
Hoxb7	0	Lcmt2	0	BRDN0000737607	0	Lce1j	0
Hoxb6	0	Lcmt1	0	Sesn1	0	Lce1h	0
Hoxb4	0	Lclat1	0	Sesn2	0	Lce1g	0
Hoxb3	0	Lce6a	0	Cd3eap	0	Lce1f	0
Hoxb2	0	Lce3f	0	Tgtp1	0	Lce1e	0
Hoxb1	0	Lce3e	0	Tgtp2	0	Lce1c	0
Hoxa7	0	Lce3d	0	A330050F15Rik	0	Lce1a1	0
Hoxa5	0	Lce3c	0	Rnasel	0	Lcat	0
Hoxa4	0	Lce3b	0	Cox18	0	Lca5l	0
Hoxa3	0	Lce3a	0	Cox19	0	Lca5	0
Hoxa13	0	Lce1m	0	Ssr2	0	Lbx2	0
Hoxa11	0	Lce1k	0	Ssr3	0	Lbx1	0
Hoxa10	0	Lce1i	0	Focad	0	Lbr	0
Hoxa1	0	Lce1h	0	Mterf3	0	Lbp	0
Hormad2	0	Lce1g	0	Cox10	0	Lbhd1	0
Hormad1	0	Lce1e	0	Cox11	0	Lbh	0
Hopx	0	Lce1d	0	Olfr266	0	Lats1	0
Hook1	0	Lce1b	0	Olfr267	0	Lat2	0
Homez	0	Lce1a1	0	Arap1	0	Las1l	0
Homer3	0	Lca5	0	Cox15	0	Lars2	0
Homer2	0	Lbx1	0	Tyr	0	Lars	0
Homer1	0	Lbr	0	Icmt	0	Larp7	0
Hoga1	0	Lbp	0	Olfr1167	0	Larp6	0
Hnrnpul2	0	Lbhd1	0	Olfr1166	0	Larp4b	0
Hnrnpul1	0	Lbh	0	Olfr1161	0	Larp4	0
Hnrnpu	0	Layn	0	Olfr1160	0	Larp1b	0
Hnrnpr	0	Lats2	0	Olfr1163	0	Large	0
Hnrnpm	0	Lats1	0	Olfr1162	0	Laptm4b	0
Hnrnpll	0	Las1l	0	Sprr2f	0	Lap3	0
Hnrnpl	0	Lars2	0	Snta1	0	Lao1	0
Hnrnpk	0	Lars	0	Olfr1168	0	Lancl3	0
Hnrnph3	0	Larp7	0	Etfdh	0	Lancl2	0

Hnrnph2	0	Larp6	0	Pfkp	0	Lamtor2	0
Hnrnph1	0	Larp4b	0	Pfkm	0	Lamtor1	0
Hnrnpf	0	Larp4	0	Pfkl	0	Lamp5	0
Hnrnpdl	0	Larp1b	0	Cdadc1	0	Lamp3	0
Hnrnpc	0	Large	0	Dos	0	Lamp2	0
Hnrnpab	0	Laptm4b	0	BRDN0000738095	0	Lamp1	0
Hnrnpa3	0	Laptm4a	0	Fam177a	0	Lamc3	0
Hnrnpa2b1	0	Lap3	0	BRDN0000737522	0	Lamc2	0
Hnrnpa1	0	Lao1	0	C230052I12Rik	0	Lamc1	0
Hnrnpa0	0	Lamtor3	0	Stac2	0	Lamb3	0
Hnmt	0	Lamtor2	0	Stac3	0	Lamb2	0
Hnf4g	0	Lamp3	0	Dysf	0	Lama5	0
Hnf4a	0	Lamp2	0	Osgin2	0	Lama4	0
Hn1l	0	Lamp1	0	Acan	0	Lama2	0
Hn1	0	Lamc3	0	1700001F09Rik	0	Lama1	0
Hmx3	0	Lamb3	0	Hnrnpm	0	Lalba	0
Hmx2	0	Lamb2	0	Nomo1	0	Lag3	0
Hmx1	0	Lama5	0	BRDN0000737483	0	Lad1	0
Hmox2	0	Lama3	0	Cnga1	0	Lactb2	0
Hmox1	0	Lama2	0	Cnga2	0	Lactb	0
Hmmr	0	Lalba	0	Cnga3	0	Lace1	0
Hmha1	0	Lair1	0	Cnga4	0	Lacc1	0
Hmgxb4	0	Lage3	0	Fech	0	l7Rn6	0
Hmgxb3	0	Lag3	0	BRDN0000737485	0	L3mbtl3	0
Hmgn5	0	Lad1	0	BRDN0000737484	0	L3hypdh	0
Hmgn1	0	Lactbl1	0	Snai2	0	L2hgdh	0
Hmgcs2	0	Lactb	0	Snai3	0	L1td1	0
Hmgcs1	0	Lacc1	0	BRDN0000737489	0	L1cam	0
Hmgcll1	0	l7Rn6	0	Snai1	0	Kynu	0
Hmgb4	0	L3mbtl4	0	BRDN0000737523	0	Ky	0
Hmgb3	0	L3mbtl2	0	Actn2	0	Ktn1	0
Hmgb2	0	L2hgdh	0	Higd1a	0	Kti12	0
Hmgb1	0	Ky	0	Msh2	0	Ksr2	0
Hmga2	0	Ktn1	0	Msh3	0	Krtdap	0
Hmga1-rs1	0	Kti12	0	Vps37c	0	Krtcap3	0
Hmga1	0	Ksr2	0	Vps37b	0	Krtcap2	0
Hmcn1	0	Ksr1	0	Vps37a	0	Krtap9-3	0
Hmces	0	Krtdap	0	Trpm5	0	Krtap8-1	0
Hmbox1	0	Krtap9-5	0	BRDN0000738167	0	Krtap7-1	0
Hltf	0	Krtap9-1	0	Mapkapk3	0	Krtap6-2	0
Hlcs	0	Krtap7-1	0	Mapkapk2	0	Krtap6-1	0
Hkdc1	0	Krtap6-2	0	Kifc5b	0	Krtap5-5	0
Hk3	0	Krtap6-1	0	Ppp1r3g	0	Krtap5-3	0
Hk2	0	Krtap5-5	0	Cldn11	0	Krtap5-2	0
Hjurp	0	Krtap5-4	0	Ppp1r3b	0	Krtap4-9	0
Hivep3	0	Krtap5-3	0	Ppp1r3a	0	Krtap4-8	0
Hivep2	0	Krtap5-2	0	Anxa9	0	Krtap4-6	0
Hivep1	0	Krtap5-1	0	Anxa8	0	Krtap4-13	0
Hist4h4	0	Krtap4-9	0	Cldn18	0	Krtap4-1	0
Hist3h2ba	0	Krtap4-8	0	Nenf	0	Krtap3-3	0
Hist3h2a	0	Krtap4-7	0	Pla2g4d	0	Krtap31-1	0
Hist2h3c2	0	Krtap4-6	0	Vmn1r85	0	Krtap3-1	0
Hist2h3c1	0	Krtap4-16	0	Vmn1r84	0	Krtap26-1	0
Hist2h3b	0	Krtap4-13	0	Rad51ap2	0	Krtap21-1	0
Hist2h2ac	0	Krtap4-1	0	Slc5a4b	0	Krtap19-9b	0
Hist2h2ab	0	Krtap3-3	0	Vmn1r81	0	Krtap19-4	0
Hist2h2aa2	0	Krtap3-2	0	Vmn1r80	0	Krtap19-3	0
Hist2h2aa1	0	Krtap31-1	0	Vmn1r83	0	Krtap19-2	0
Hist1h4n	0	Krtap3-1	0	Vmn1r82	0	Krtap19-1	0
Hist1h4m	0	Krtap26-1	0	Gramd1c	0	Krtap17-1	0
Hist1h4j	0	Krtap2-4	0	Bap1	0	Krtap16-3	0
Hist1h4i	0	Krtap22-2	0	Slx1b	0	Krtap16-1	0
Hist1h4h	0	Krtap21-1	0	Vmn1r88	0	Krtap1-5	0
Hist1h4f	0	Krtap20-2	0	Trp63	0	Krtap15	0
Hist1h4d	0	Krtap19-9b	0	Riok1	0	Krtap1-4	0
Hist1h4c	0	Krtap19-4	0	BRDN0000737677	0	Krtap14	0
Hist1h4b	0	Krtap19-3	0	Bbip1	0	Krtap13-1	0
Hist1h4a	0	Krtap19-1	0	Hspa12b	0	Krtap1-3	0
Hist1h3i	0	Krtap17-1	0	Brox	0	Krtap13	0
Hist1h3h	0	Krtap16-3	0	Mrps18a	0	Krtap10-4	0
Hist1h3g	0	Krtap1-5	0	Bax	0	Krt9	0
Hist1h3f	0	Krtap15	0	Mrps18c	0	Krt86	0
Hist1h3e	0	Krtap1-4	0	Crybb3	0	Krt83	0
Hist1h3c	0	Krtap13-1	0	Bad	0	Krt80	0
Hist1h3b	0	Krtap12-1	0	Crybb1	0	Krt79	0
Hist1h3a	0	Krtap10-4	0	Cyp46a1	0	Krt78	0
Hist1h2bp	0	Krt86	0	Btn2a2	0	Krt76	0
Hist1h2bn	0	Krt84	0	Nipal4	0	Krt75	0
Hist1h2bm	0	Krt83	0	Tmem68	0	Krt73	0
Hist1h2bl	0	Krt81	0	Abhd12b	0	Krt72	0
Hist1h2bk	0	Krt80	0	Toe1	0	Krt6b	0
Hist1h2bj	0	Krt8	0	Nipsnap1	0	Krt5	0

Hist1h2bh	0	Krt79	0	Stk11ip	0	Krt42	0
Hist1h2bf	0	Krt78	0	Eogt	0	Krt40	0
Hist1h2bb	0	Krt75	0	Ciita	0	Krt4	0
Hist1h2ap	0	Krt71	0	Cacna2d2	0	Krt39	0
Hist1h2ao	0	Krt6b	0	Ppp1r37	0	Krt36	0
Hist1h2an	0	Krt6a	0	Ppp1r36	0	Krt35	0
Hist1h2ak	0	Krt5	0	Ppp1r35	0	Krt33b	0
Hist1h2ai	0	Krt4	0	Cyb561	0	Krt32	0
Hist1h2ah	0	Krt39	0	Ppp1r32	0	Krt27	0
Hist1h2ag	0	Krt36	0	Plagl2	0	Krt26	0
Hist1h2af	0	Krt34	0	Zfp639	0	Krt24	0
Hist1h2ac	0	Krt33b	0	BRDN0000737741	0	Krt19	0
Hist1h2ab	0	Krt32	0	Plagl1	0	Krt18	0
Hist1h2aa	0	Krt31	0	Athl1	0	Krt17	0
Hist1h1t	0	Krt28	0	Ap4e1	0	Krt15	0
Hist1h1e	0	Krt27	0	Fn1	0	Krt13	0
Hist1h1b	0	Krt26	0	A830010M20Rik	0	Krt12	0
Hist1h1a	0	Krt23	0	Naip5	0	Krt10	0
Hirip3	0	Krt222	0	Mrap2	0	Krt1	0
Hipk4	0	Krt20	0	Esp24	0	Krr1	0
Hipk3	0	Krt2	0	Esp23	0	Kri1	0
Hip1r	0	Krt19	0	Pyroxd2	0	Kremen2	0
Hip1	0	Krt18	0	Pyroxd1	0	Krcc1	0
Hint3	0	Krt17	0	Naip2	0	Krba1	0
Hint2	0	Krt16	0	Rc3h1	0	Kras	0
Hint1	0	Krt15	0	Slain2	0	Kptn	0
Hinfp	0	Krt14	0	Slain1	0	Kpnb1	0
Hilpda	0	Krt12	0	Rc3h2	0	Kpna4	0
Higd2a	0	Krt1	0	Eno4	0	Kpna3	0
Higd1c	0	Krr1	0	Psg28	0	Kpna2	0
Higd1b	0	Krit1	0	Gpbar1	0	Kntc1	0
Higd1a	0	Kri1	0	Psg25	0	Knstrn	0
Hif3a	0	Kremen2	0	Psg27	0	Knop1	0
Hif1an	0	Kremen1	0	Psg26	0	Kng2	0
Hif1a	0	Krcc1	0	Psg21	0	Kng1	0
Hic2	0	Kprp	0	Psg23	0	Kndc1	0
Hic1	0	Kpnb1	0	Psg22	0	Kmt2e	0
Hibch	0	Kpna6	0	Ankle1	0	Kmt2d	0
Hibadh	0	Kpna4	0	Dxo	0	Kmt2c	0
Hiatl1	0	Kpna3	0	Slc16a2	0	Kmt2b	0
Hiat1	0	Kpna2	0	Omg	0	Kmt2a	0
Hhla1	0	Kpna1	0	Glmp	0	Klrk1	0
Hhipl2	0	Kntc1	0	Omd	0	Klri1	0
Hhipl1	0	Knstrn	0	Syngr1	0	Klrg2	0
Hhip	0	Knop1	0	Plcz1	0	Klrg1	0
Hhex	0	Kng2	0	Syngr3	0	Klrd1	0
Hhatl	0	Kng1	0	Rnase6	0	Klrc3	0
Hhat	0	Kndc1	0	Syngr4	0	Klrc1	0
Hgsnat	0	Kmt2e	0	Mbd5	0	Klrb1f	0
Hgs	0	Kmt2c	0	Mbd4	0	Klrb1c	0
Hgh1	0	Kmt2a	0	Mbd6	0	Klrb1b	0
Hgfac	0	Klrk1	0	Mbd1	0	Klrb1a	0
Hgf	0	Klri2	0	Pabpc4l	0	Klrb1	0
Hfm1	0	Klri1	0	Mbd3	0	Klra9	0
Hfe	0	Klrg1	0	Mbd2	0	Klra8	0
Heyl	0	Klre1	0	Glmn	0	Klra7	0
Hey2	0	Klrd1	0	Pafah1b3	0	Klra5	0
Hey1	0	Klrc1	0	BRDN0000737767	0	Klra4	0
Hexim2	0	Klrb1a	0	Il27ra	0	Klra3	0
Hexb	0	Klrb1	0	BC005561	0	Klra2	0
Hexa	0	Klra9	0	Arhgef9	0	Klra10	0
Hesx1	0	Klra7	0	Arhgef6	0	Klra1	0
Hes7	0	Klra6	0	Arhgef7	0	Klkb1	0
Hes6	0	Klra4	0	Arhgef5	0	Klk9	0
Hes5	0	Klra2	0	Arhgef2	0	Klk7	0
Hes3	0	Klra17	0	Arhgef3	0	Klk6	0
Hes2	0	Klra1	0	Zc3h18	0	Klk5	0
Hes1	0	Klkb1	0	Arhgef1	0	Klk4	0
Herpud2	0	Klk7	0	BRDN0000737854	0	Klk1b9	0
Herpud1	0	Klk5	0	Samd3	0	Klk1b8	0
Herc6	0	Klk1b9	0	Msi2	0	Klk1b4	0
Herc4	0	Klk1b5	0	Mief1	0	Klk1b26	0
Herc3	0	Klk1b4	0	Mief2	0	Klk1b24	0
Herc2	0	Klk1b3	0	Ung	0	Klk1b22	0
Herc1	0	Klk1b26	0	Polrmt	0	Klk1b21	0
Hephl1	0	Klk1b22	0	BRDN0000737764	0	Klk1b16	0
Heph	0	Klk1b16	0	Tas1r3	0	Klk15	0
Hepacam2	0	Klk1b1	0	Unk	0	Klk14	0
Hepacam	0	Klk15	0	Snap25	0	Klk13	0
Henmt1	0	Klk14	0	Pstpip1	0	Klk12	0
Hemt1	0	Klk12	0	Dido1	0	Klk11	0
Hemk1	0	Klk10	0	Cct6b	0	Klk10	0

Hemgn	0	Klk1	0	Epb4.1	0	Klk1	0
Helz2	0	Klhl9	0	Trmu	0	Klhl8	0
Helz	0	Klhl8	0	Crebl2	0	Klhl7	0
Helt	0	Klhl7	0	BRDN0000737855	0	Klhl5	0
Helq	0	Klhl6	0	Haao	0	Klhl42	0
Hells	0	Klhl5	0	Vmn2r19	0	Klhl40	0
Hectd2	0	Klhl41	0	4833427G06Rik	0	Klhl38	0
Hectd1	0	Klhl40	0	Vmn2r17	0	Klhl36	0
Hebp2	0	Klhl38	0	Vmn2r16	0	Klhl35	0
Hebp1	0	Klhl36	0	Vmn2r15	0	Klhl34	0
Heatr9	0	Klhl35	0	Vmn2r14	0	Klhl33	0
Heatr6	0	Klhl33	0	Vmn2r13	0	Klhl32	0
Heatr5b	0	Klhl32	0	Vmn2r12	0	Klhl30	0
Heatr3	0	Klhl31	0	Vmn2r11	0	Klhl3	0
Heatr1	0	Klhl30	0	Vmn2r10	0	Klhl29	0
Hdlbp	0	Klhl26	0	Bcan	0	Klhl26	0
Hdhd3	0	Klhl23	0	Rec8	0	Klhl25	0
Hdhd2	0	Klhl22	0	Mcm10	0	Klhl23	0
Hdhd1a	0	Klhl21	0	Mkln1	0	Klhl22	0
Hdgfrp3	0	Klhl20	0	Wfdc16	0	Klhl21	0
Hdgf	0	Klhl18	0	Paip2	0	Klhl20	0
Hddc3	0	Klhl17	0	Efcab11	0	Klhl18	0
Hddc2	0	Klhl11	0	Gpr65	0	Klhl13	0
Hdc	0	Klhl1	0	Gpr62	0	Klhl11	0
Hdac5	0	Klhdc9	0	Gpr63	0	Klhl1	0
Hdac4	0	Klhdc8a	0	Efcab10	0	Klhdc8b	0
Hdac3	0	Klhdc7b	0	Fan1	0	Klhdc8a	0
Hdac2	0	Klhdc4	0	Tyw5	0	Klhdc7b	0
Hdac11	0	Klhdc2	0	Bdh2	0	Klhdc7a	0
Hdac10	0	Klf9	0	2010106E10Rik	0	Klhdc2	0
Hcrtr1	0	Klf7	0	Gpr68	0	Klhdc10	0
Hcrt	0	Klf6	0	Tyw3	0	Klhdc1	0
Hcn4	0	Klf5	0	Samt4	0	Klf9	0
Hcn3	0	Klf4	0	Traf1	0	Klf5	0
Hcls1	0	Klf3	0	Traf2	0	Klf4	0
Hck	0	Klf17	0	Traf3	0	Klf3	0
Hcfc2	0	Klf11	0	Traf4	0	Klf2	0
Hcfc1r1	0	Klf10	0	Atp2a1	0	Klf17	0
Hcfc1	0	Klf1	0	Atp2a2	0	Klf16	0
Hcar2	0	Klc4	0	Atp2a3	0	Klf13	0
Hc	0	Klc3	0	Asic5	0	Klf12	0
Hbq1b	0	Klc2	0	Asic4	0	Klf11	0
Hbq1a	0	Klc1	0	Pnpt1	0	Klf10	0
Hbp1	0	Kl	0	Acin1	0	Klf1	0
Hbegf	0	Kitl	0	Asic1	0	Klc4	0
Hbb-y	0	Kit	0	Klhdc7b	0	Klc3	0
Hba-x	0	Kiss1r	0	Pde9a	0	Klc2	0
Hba-a2	0	Kirrel2	0	Sfxn5	0	Klc1	0
Hba-a1	0	Kirrel	0	Nkx3-2	0	Kitl	0
Hax1	0	Kir3dl2	0	Nkx3-1	0	Kit	0
Havcr2	0	Kir3dl1	0	Rbak	0	Kiss1	0
Havcr1	0	Kin	0	Sfxn2	0	Kirrel3	0
Haus8	0	Kifc5b	0	Cyhr1	0	Kir3dl2	0
Haus7	0	Kifc3	0	Klhdc7a	0	Kir3dl1	0
Haus6	0	Kifc2	0	Acp5	0	Kin	0
Haus5	0	Kifc1	0	Gm6902	0	Kifc5b	0
Haus4	0	Kif9	0	Olfr1367	0	Kifc2	0
Haus3	0	Kif7	0	Shfm1	0	Kifc1	0
Haus1	0	Kif6	0	Slx	0	Kif7	0
Hat1	0	Kif4	0	Sly	0	Kif6	0
Has2	0	Kif2c	0	Rps6kb2	0	Kif5c	0
Has1	0	Kif26a	0	1700010B08Rik	0	Kif5b	0
Hars	0	Kif23	0	Sfxn1	0	Kif5a	0
Hapln3	0	Kif22	0	Chd3	0	Kif4	0
Hapln2	0	Kif21b	0	Npy6r	0	Kif3a	0
Hap1	0	Kif21a	0	4833439L19Rik	0	Kif2c	0
Hao2	0	Kif20b	0	Mutyh	0	Kif2a	0
Hand2	0	Kif20a	0	Spt1	0	Kif23	0
Hand1	0	Kif1a	0	Sln	0	Kif22	0
Hamp2	0	Kif18b	0	Ociad1	0	Kif21b	0
Hamp	0	Kif18a	0	Prkaca	0	Kif21a	0
Hagh	0	Kif17	0	Mkl1	0	Kif20a	0
Hadhb	0	Kif16b	0	Nr3c2	0	Kif1c	0
Hacl1	0	Kif15	0	Slk	0	Kif1a	0
Hace1	0	Kif14	0	Ddah2	0	Kif18b	0
Hacd4	0	Kif13b	0	Chd5	0	Kif18a	0
Hacd3	0	Kif12	0	Sla	0	Kif17	0
Hacd2	0	Kif11	0	Zc3h7b	0	Kif15	0
Hacd1	0	Khdrbs3	0	Xk	0	Kif13a	0
Haao	0	Khdrbs2	0	Tmem98	0	Kif12	0
H6pd	0	Khdrbs1	0	1700067P10Rik	0	Kif11	0
H60c	0	Khdc3	0	Ccdc160	0	Khnyn	0

H60b	0
H3f3b	0
H3f3a	0
H2-T3	0
H2-T24	0
H2-T22	0
H2-Q7	0
H2-Q6	0
H2-Q4	0
H2-Q2	0
H2-Ob	0
H2-M9	0
H2-M5	0
H2-M3	0
H2-M2	0
H2-M11	0
H2-M10.6	0
H2-M10.5	0
H2-M10.3	0
H2-M10.1	0
H2-M1	0
H2-Ke6	0
H2-Eb2	0
H2-Eb1	0
H2-DMb2	0
H2-DMb1	0
H2-DMa	0
H2-D1	0
H2afz	0
H2afy	0
H2afx	0
H2afv	0
H2afj	0
H2afb3	0
H2afb2	0
H2afb1	0
H2-Ab1	0
H2-Aa	0
H1fx	0
H1foo	0
H1f0	0
H13	0
Gzmm	0
Gzmk	0
Gzmg	0
Gzmf	0
Gzme	0
Gzmd	0
Gzmc	0
Gzmb	0
Gzma	0
Gzf1	0
Gys2	0
Gys1	0
Gypc	0
Gypa	0
Gyk	0
Gyg	0
Gxylt2	0
Gvin1	0
Gusb	0
Gulp1	0
Gulo	0
Guk1	0
Guf1	0
Gucy2g	0
Gucy2f	0
Gucy2e	0
Gucy1b3	0
Gucy1b2	0
Gucy1a3	0
Guca2b	0
Guca2a	0
Guca1b	0
Guca1a	0
Gtsf1l	0
Gtse1	0
Gtpbp8	0
Gtpbp6	0
Gtpbp4	0
Gtpbp3	0
Gtpbp10	0
Khdc1c	0
Khdc1b	0
Khdc1a	0
Kera	0
Kel	0
Keg1	0
Keap1	0
Kdsr	0
Kdm8	0
Kdm7a	0
Kdm5d	0
Kdm5c	0
Kdm5b	0
Kdm5a	0
Kdm4c	0
Kdm4b	0
Kdm3b	0
Kdm3a	0
Kdm2b	0
Kdm2a	0
Kdm1b	0
Kdm1a	0
Kdelc1	0
Kctd8	0
Kctd7	0
Kctd5	0
Kctd4	0
Kctd3	0
Kctd20	0
Kctd18	0
Kctd14	0
Kctd13	0
Kctd12b	0
Kctd12	0
Kctd10	0
Kctd1	0
Kcp	0
Kcnv2	0
Kcnv1	0
Kcnu1	0
Kcnt2	0
Kcns3	0
Kcnrg	0
Kcnq4	0
Kcnq3	0
Kcnq2	0
Kcnn4	0
Kcnn3	0
Kcnn2	0
Kcnmb4	0
Kcnmb1	0
Kcnk9	0
Kcnk7	0
Kcnk6	0
Kcnk5	0
Kcnk4	0
Kcnk2	0
Kcnk18	0
Kcnk13	0
Kcnk12	0
Kcnk10	0
Kcnj6	0
Kcnj2	0
Kcnj16	0
Kcnj15	0
Kcnj13	0
Kcnj11	0
Kcnj10	0
Kcnip4	0
Kcnip2	0
Kcnip1	0
Kcnh8	0
Kcnh7	0
Kcnh6	0
Kcnh5	0
Kcnh3	0
Kcnh1	0
Kcng4	0
Kcng3	0
Kcng2	0
Kcng1	0
Kcnf1	0
Chd7	0
BRDN0000737851	0
BRDN0000738270	0
Msi1	0
Tmem91	0
Tmem97	0
Tmem95	0
BRDN0000737745	0
Coq4	0
Med13l	0
Scmh1	0
Nrg1	0
Nrg3	0
Nrg2	0
Nrg4	0
Aadacl3	0
Aadacl2	0
Psrc1	0
Ccdc88c	0
Knop1	0
Lipt2	0
Lclat1	0
Fpr2	0
Fpr3	0
Akr1e1	0
Prss33	0
Prss32	0
Mefv	0
Rarb	0
Upf1	0
Prss36	0
Prss35	0
Kdm7a	0
Gm694	0
Actr6	0
Vmn2r3	0
Prss38	0
Actr3	0
Actr2	0
Lax1	0
Fus	0
Galnt16	0
Ccdc51	0
Ces2b	0
Galnt15	0
Galnt12	0
Galnt10	0
Ces2g	0
Ces2h	0
Ankmy2	0
Kit	0
Rhox2c	0
Rhox2b	0
Plekhm3	0
Rhox2f	0
Nelfcd	0
Rhox2d	0
AI317395	0
Prelp	0
Rhox2h	0
Ppp6r3	0
Pttg1ip	0
Hmgn2	0
AF366264	0
Prl7d1	0
Atm	0
Hoxb8	0
2010107G12Rik	0
S100a3	0
Kcnn1	0
Pacrg	0
Runx2	0
Fbn1	0
Hmgn1	0
Gm3500	0
Trim71	0
Usp13	0
Gjb1	0
Nt5dc2	0
Gjb3	0
Gjb2	0
4921517D22Rik	0
Khk	0
Khdrbs3	0
Khdrbs2	0
Khdrbs1	0
Khdc1c	0
Khdc1a	0
Kera	0
Keg1	0
Keap1	0
Kdsr	0
Kdr	0
Kdm7a	0
Kdm6b	0
Kdm5d	0
Kdm5b	0
Kdm5a	0
Kdm4c	0
Kdm4b	0
Kdm4a	0
Kdm3b	0
Kdm2b	0
Kdm2a	0
Kdm1b	0
Kdm1a	0
Kdf1	0
Kdelr3	0
Kdelr2	0
Kdelc2	0
Kdelc1	0
Kctd9	0
Kctd8	0
Kctd5	0
Kctd21	0
Kctd2	0
Kctd19	0
Kctd18	0
Kctd16	0
Kctd15	0
Kctd14	0
Kctd12b	0
Kctd10	0
Kctd1	0
Kcp	0
Kcnv2	0
Kcnv1	0
Kcnu1	0
Kcnt2	0
Kcnt1	0
Kcns3	0
Kcns2	0
Kcns1	0
Kcnrg	0
Kcnq5	0
Kcnq4	0
Kcnq3	0
Kcnq2	0
Kcnn4	0
Kcnn2	0
Kcnn1	0
Kcnmb4	0
Kcnmb3	0
Kcnmb2	0
Kcnmb1	0
Kcnma1	0
Kcnk9	0
Kcnk7	0
Kcnk6	0
Kcnk4	0
Kcnk2	0
Kcnk18	0
Kcnk16	0
Kcnk13	0
Kcnk12	0
Kcnk10	0
Kcnk1	0
Kcnj8	0
Kcnj6	0
Kcnj5	0
Kcnj16	0
Kcnj15	0
Kcnj14	0
Kcnj13	0

Gtpbp1	0	Kcne4	0	Gjb4	0	Kcnj12	0
Gtf3c6	0	Kcne2	0	Nt5dc3	0	Kcnj11	0
Gtf3c5	0	Kcne1l	0	Ralgapb	0	Kcnj10	0
Gtf3c4	0	Kcnd3	0	Myrfl	0	Kcnip4	0
Gtf3c3	0	Kcnd2	0	H1foo	0	Kcnip3	0
Gtf3c1	0	Kcnc4	0	Hoxb3	0	Kcnip2	0
Gtf3a	0	Kcnc3	0	Itk	0	Kcnh8	0
Gtf2ird1	0	Kcnc2	0	Hoxb1	0	Kcnh7	0
Gtf2i	0	Kcnc1	0	Abcf2	0	Kcnh4	0
Gtf2h5	0	Kcnb2	0	Abcf1	0	Kcnh3	0
Gtf2h4	0	Kcnb1	0	Col7a1	0	Kcnh2	0
Gtf2h3	0	Kcnab1	0	Hoxb6	0	Kcng1	0
Gtf2h2	0	Kcna7	0	BRDN0000737856	0	Kcne2	0
Gtf2h1	0	Kcna6	0	Alms1	0	Kcne1l	0
Gtf2f2	0	Kcna5	0	Vmn1r194	0	Kcnd1	0
Gtf2f1	0	Kcna2	0	Slc6a19	0	Kcnc4	0
Gtf2e2	0	Kcna10	0	Ifnl3	0	Kcnc3	0
Gtf2e1	0	Kcmf1	0	Magi3	0	Kcnab3	0
Gtf2b	0	Kbtbd2	0	Magi2	0	Kcnab2	0
Gtf2a2	0	Kbtbd13	0	Vmn1r192	0	Kcnab1	0
Gtf2a1l	0	Kbtbd11	0	Vmn1r193	0	Kcna7	0
Gtf2a1	0	Kazn	0	Slc6a13	0	Kcna5	0
Gtdc1	0	Katnbl1	0	Slc6a12	0	Kcna4	0
Gsx2	0	Katnb1	0	Tdp1	0	Kcna3	0
Gstt4	0	Katnal1	0	Tdp2	0	Kcna10	0
Gstt3	0	Katna1	0	Chek1	0	Kcna1	0
Gstt2	0	Kat8	0	BRDN0000738129	0	Kcmf1	0
Gstt1	0	Kat7	0	Lrrc4c	0	Kbtbd8	0
Gstp2	0	Kat6b	0	Lrrc4b	0	Kbtbd7	0
Gsto2	0	Kat6a	0	Ppfia2	0	Kbtbd3	0
Gsto1	0	Kat5	0	Ugt1a10	0	Kbtbd2	0
Gstm7	0	Kat2b	0	Ppfia1	0	Kbtbd13	0
Gstm6	0	Kap	0	BRDN0000737618	0	Kbtbd12	0
Gstm5	0	Kansl3	0	1700122O11Rik	0	Kbtbd11	0
Gstm1	0	Kansl2	0	Fuk	0	Kazn	0
Gstk1	0	Kansl1l	0	Tspan32	0	Kazald1	0
Gstcd	0	Kank2	0	Tspan33	0	Katnbl1	0
Gsta4	0	Kank1	0	Pam16	0	Katnb1	0
Gsta3	0	Kalrn	0	Runx1	0	Katnal1	0
Gss	0	Junb	0	Alad	0	Katna1	0
Gsr	0	Jun	0	Ephx1	0	Kat8	0
Gspt2	0	Jtb	0	Ccdc85a	0	Kat6a	0
Gspt1	0	Jsrp1	0	Zfy2	0	Kat2b	0
Gskip	0	Jph4	0	BRDN0000738216	0	Kat2a	0
Gsk3a	0	Jph2	0	Vmn1r228	0	Kars	0
Gsg2	0	Jph1	0	Olfr748	0	Kansl3	0
Gsg1l	0	Jmy	0	BRDN0000738213	0	Kansl1l	0
Gsg1	0	Jmjd7	0	BRDN0000738212	0	Kank4	0
Gse1	0	Jmjd4	0	BRDN0000738211	0	Kank1	0
Gsdmd	0	Jmjd1c	0	BRDN0000738210	0	Kalrn	0
Gsdmc4	0	Jdp2	0	Vmn1r222	0	Junb	0
Gsdmc3	0	Jchain	0	Gabrr1	0	Jun	0
Gsdmc2	0	Jazf1	0	Gabrr3	0	Jtb	0
Gsdmc	0	Jarid2	0	Vmn1r226	0	Jrkl	0
Gsdma3	0	Jakmip2	0	Olfr746	0	Jrk	0
Gsc2	0	Jakmip1	0	Vmn1r224	0	Jph3	0
Gsc	0	Jak1	0	Vmn1r225	0	Jph2	0
Gsap	0	Jade3	0	Slco1a4	0	Jph1	0
Grxcr2	0	Jade2	0	Slco1a5	0	Josd2	0
Grwd1	0	Izumo3	0	Slco1a6	0	Josd1	0
Grtp1	0	Izumo2	0	Set	0	Jmy	0
Grsf1	0	Izumo1r	0	Slco1a1	0	Jmjd8	0
Grrp1	0	Izumo1	0	Olfr346	0	Jmjd7	0
Grpr	0	Iws1	0	Olfr1424	0	Jmjd4	0
Grpel2	0	Ivns1abp	0	AW146154	0	Jmjd1c	0
Grpel1	0	Ivd	0	BRDN0000738122	0	Jkamp	0
Grp	0	Itpr1	0	Blcap	0	Jdp2	0
Grn	0	Itpkc	0	BRDN0000737616	0	Jchain	0
Grm8	0	Itpkb	0	Otx1	0	Jazf1	0
Grm7	0	Itpka	0	St8sia5	0	Jarid2	0
Grm6	0	Itpa	0	Otx2	0	Jam2	0
Grm5	0	Itm2b	0	Lrrc41	0	Jak3	0
Grm4	0	Itm2a	0	Lrrc40	0	Jak2	0
Grm3	0	Itln1	0	Lrrc43	0	Jak1	0
Grm2	0	Itk	0	Lrrc66	0	Jagn1	0
Grk6	0	Itih4	0	Lrrc45	0	Jag2	0
Grk4	0	Itih2	0	BRDN0000738127	0	Jag1	0
Grk1	0	Itih1	0	Utp20	0	Jade2	0
Grip2	0	Itgb8	0	Hs2st1	0	Izumo4	0
Grip1	0	Itgb7	0	Lrrc49	0	Izumo3	0
Grina	0	Itgb6	0	Lrrc48	0	Izumo2	0
Grin3b	0	Itgb5	0	BRDN0000738126	0	Iws1	0

Grin3a	0	Itgb3bp	0	Olfr849	0	Ivd	0
Grin2c	0	Itgb3	0	Sult1b1	0	Itsn1	0
Grin2b	0	Itgb2	0	Gm648	0	Itpripl2	0
Grin1	0	Itgb1	0	H1fx	0	Itpr3	0
Grik5	0	Itgax	0	Zbtb7a	0	Itpr1	0
Grik4	0	Itgav	0	Lpin3	0	Itpka	0
Grik3	0	Itgal	0	Zbtb7c	0	Itpk1	0
Grik2	0	Itgae	0	Olfr566	0	Itpa	0
Grifin	0	Itga9	0	Rfesd	0	Itm2c	0
Grid2ip	0	Itga8	0	Vgf	0	Itm2a	0
Grid2	0	Itga6	0	Btla	0	Itln1	0
Grid1	0	Itga2b	0	Lcn5	0	Itk	0
Gria3	0	Itga2	0	Themis3	0	Itih4	0
Gria2	0	Itga11	0	Themis2	0	Itih3	0
Gria1	0	Itfg2	0	Cbx8	0	Itih1	0
Grhpr	0	Itfg1	0	Olfr1406	0	Itgb8	0
Grhl3	0	Isy1	0	Cse1l	0	Itgb7	0
Grhl2	0	Isx	0	Cbx5	0	Itgb6	0
Grhl1	0	Ispd	0	Cbx4	0	Itgb3bp	0
Grem1	0	Isoc2b	0	Cbx3	0	Itgb3	0
Greb1l	0	Isoc2a	0	Cbx2	0	Itgb1bp2	0
Greb1	0	Isoc1	0	Cbx1	0	Itgb1bp1	0
Grcc10	0	Isg20l2	0	Furin	0	Itgax	0
Grb7	0	Isg20	0	Olfr847	0	Itgav	0
Grb2	0	Isg15	0	Olfr561	0	Itgal	0
Grb10	0	Iscu	0	Cdc73	0	Itgae	0
Grasp	0	Irx6	0	Sdcbp2	0	Itga9	0
Grap2	0	Irx2	0	Gm6121	0	Itga8	0
Grap	0	Irx1	0	Zc3hav1l	0	Itga5	0
Gramd4	0	Irs3	0	Tars	0	Itga2b	0
Gramd1c	0	Irs2	0	Gcfc2	0	Itga2	0
Gramd1b	0	Irs1	0	Slc43a3	0	Itga11	0
Gpx6	0	Irgq	0	Slc43a2	0	Itga1	0
Gpx3	0	Irgm2	0	Slc43a1	0	Itfg2	0
Gpx2	0	Irgm1	0	Gas1	0	Itch	0
Gpx1	0	Irg1	0	Olfr812	0	Isyna1	0
Gpsm3	0	Irf6	0	Ifna9	0	Isy1	0
Gpsm1	0	Irf4	0	Olfr810	0	Ist1	0
Gps2	0	Irf3	0	Ccl6	0	Ispd	0
Gps1	0	Irf2bpl	0	Olfr816	0	Isoc2b	0
Gprin3	0	Irf2bp2	0	Olfr815	0	Isoc2a	0
Gprin1	0	Irf2	0	Klrk1	0	Isoc1	0
Gprc6a	0	Irf1	0	Ifna2	0	Islr2	0
Gprc5d	0	Ireb2	0	Ccl8	0	Islr	0
Gprc5c	0	Irak4	0	Olfr818	0	Isl1	0
Gprc5b	0	Irak3	0	Ifna7	0	Isg20l2	0
Gprc5a	0	Irak2	0	Ifna5	0	Isg15	0
Gprasp2	0	Irak1bp1	0	Ifna4	0	Isca2	0
Gprasp1	0	Irak1	0	Gm6026	0	Isca1	0
Gpr87	0	Iqsec2	0	Sigirr	0	Irx4	0
Gpr83	0	Iqsec1	0	Ttpa	0	Irx3	0
Gpr82	0	Iqgap3	0	BRDN0000737436	0	Irx2	0
Gpr68	0	Iqck	0	Pcyox1l	0	Irx1	0
Gpr63	0	Iqch	0	Skint2	0	Irs3	0
Gpr6	0	Iqcf6	0	Abca4	0	Irs2	0
Gpr55	0	Iqcf5	0	Rsph6a	0	Irgm2	0
Gpr50	0	Iqcf4	0	Utp14a	0	Irgm1	0
Gpr45	0	Iqcf3	0	Utp14b	0	Irgc1	0
Gpr39	0	Iqcf1	0	Ubqln3	0	Irf9	0
Gpr37l1	0	Iqce	0	Prr27	0	Irf8	0
Gpr37	0	Iqcc	0	Eif2b3	0	Irf7	0
Gpr35	0	Iqcb1	0	Ap3d1	0	Irf6	0
Gpr34	0	Iqca	0	Prr22	0	Irf5	0
Gpr3	0	Ippk	0	6030468B19Rik	0	Irf4	0
Gpr25	0	Ipp	0	BRDN0000737437	0	Irf3	0
Gpr21	0	Ipo9	0	Tecta	0	Irf2bp1	0
Gpr20	0	Ipo8	0	Ubqln1	0	Irf2	0
Gpr19	0	Ipo7	0	Rspo2	0	Irf1	0
Gpr183	0	Ipo5	0	Tsen15	0	Ireb2	0
Gpr182	0	Ipo4	0	Ifi30	0	Irak4	0
Gpr180	0	Ipo13	0	Cryaa	0	Irak3	0
Gpr179	0	Ipo11	0	Zcchc11	0	Irak2	0
Gpr176	0	Ipmk	0	Sprn	0	Irak1bp1	0
Gpr174	0	Ipcef1	0	Rnaset2b	0	Irak1	0
Gpr173	0	Ip6k3	0	Ubqln4	0	Iqsec1	0
Gpr171	0	Ip6k2	0	Gm7861	0	Iqgap3	0
Gpr17	0	Ip6k1	0	Crebrf	0	Iqgap2	0
Gpr160	0	Ints9	0	Mdm2	0	Iqgap1	0
Gpr157	0	Ints8	0	Mdm4	0	Iqck	0
Gpr156	0	Ints7	0	Ror2	0	Iqch	0
Gpr155	0	Ints6	0	Arel1	0	Iqcg	0
Gpr153	0	Ints4	0	Cdan1	0	Iqcf4	0

Gpr152	0
Gpr151	0
Gpr150	0
Gpr15	0
Gpr149	0
Gpr146	0
Gpr143	0
Gpr142	0
Gpr141	0
Gpr137c	0
Gpr137b	0
Gpr137	0
Gpr135	0
Gpr132	0
Gpr12	0
Gpr108	0
Gpr107	0
Gpr101	0
Gpr1	0
Gpnmb	0
Gpn2	0
Gpn1	0
Gpm6b	0
Gpm6a	0
Gpld1	0
Gpkow	0
Gpihbp1	0
Gpi1	0
Gphn	0
Gphb5	0
Gpha2	0
Gper1	0
Gpd2	0
Gpd1	0
Gpc6	0
Gpc5	0
Gpc3	0
Gpc2	0
Gpc1	0
Gpbp1l1	0
Gpbp1	0
Gpatch4	0
Gpatch3	0
Gpatch2l	0
Gpatch11	0
Gpatch1	0
Gpat2	0
Gpank1	0
Gpaa1	0
Gpa33	0
Gp9	0
Gp6	0
Gp5	0
Gp49a	0
Gp2	0
Gp1bb	0
Gp1ba	0
Got1	0
Gosr2	0
Gosr1	0
Gorasp2	0
Gorasp1	0
Gorab	0
Gon4l	0
Golt1b	0
Golt1a	0
Golph3	0
Golm1	0
Golim4	0
Golgb1	0
Golga7b	0
Golga7	0
Golga5	0
Golga4	0
Golga3	0
Golga2	0
Golga1	0
Gns	0
Gnptab	0
Gnpnat1	0
Gnpda1	0
Gnpat	0
Ints3	0
Ints2	0
Ints1	0
Insrr	0
Insr	0
Insm2	0
Insl5	0
Insl3	0
Insig1	0
Ins1	0
Inpp5j	0
Inpp5e	0
Inpp5d	0
Inpp5b	0
Inpp4a	0
Inpp1	0
Ino80c	0
Ino80b	0
Inmt	0
Inip	0
Inhbe	0
Inhbc	0
Inhba	0
Inha	0
Ing5	0
Ing4	0
Ing3	0
Ing2	0
Ing1	0
Inf2	0
Inca1	0
Inafm1	0
Inadl	0
Ina	0
Impg2	0
Impg1	0
Impdh2	0
Impdh1	0
Impact	0
Imp4	0
Imp3	0
Immt	0
Immp1l	0
Ilvbl	0
Iltifb	0
Ilk	0
Ilf3	0
Ildr1	0
Il9	0
Il7r	0
Il7	0
Il6st	0
Il6ra	0
Il6	0
Il5ra	0
Il5	0
Il4ra	0
Il3ra	0
Il34	0
Il33	0
Il31	0
Il3	0
Il2rg	0
Il2rb	0
Il2ra	0
Il27ra	0
Il27	0
Il22ra2	0
Il22ra1	0
Il22	0
Il21	0
Il20ra	0
Il20	0
Il2	0
Il1rn	0
Il1rl1	0
Il1rapl1	0
Il1r2	0
Il1r1	0
Il1f8	0
Il1f5	0
Il1f10	0
Slc44a1	0
Slc44a2	0
Wdr54	0
Slc44a4	0
Slc44a5	0
Laptm4b	0
Pik3ca	0
Pik3cb	0
Pik3cd	0
Atraid	0
Wdr59	0
Pik3cg	0
Nkain2	0
Sec61a2	0
1700102P08Rik	0
Obox6	0
Gm973	0
Ldhc	0
Suclg1	0
Fam63a	0
9430015G10Rik	0
Nxf1	0
Nxf2	0
Nxf3	0
Mapkbp1	0
H2afb3	0
H2afb2	0
H2afb1	0
Shbg	0
A930018P22Rik	0
Micalcl	0
Atxn7l3	0
Skint6	0
Atxn7l1	0
Ssty1	0
Ncaph2	0
Bscl2	0
Gdf7	0
Ttc9c	0
Uevld	0
Cfap126	0
Ciao1	0
Zscan18	0
Tox4	0
Gm2927	0
Wdr5b	0
Scrn3	0
Scrn2	0
Scrn1	0
Zscan12	0
Mpc2	0
Pls1	0
Pls3	0
Pard3	0
Camk2g	0
Tefm	0
Cyp19a1	0
Mc2r	0
BRDN0000737669	0
Kif26a	0
Gm13285	0
Sdr9c7	0
A530032D15Rik	0
Xlr4a	0
Gm13283	0
Skint8	0
Lrg1	0
Tsc22d2	0
Olfr699	0
BRDN0000737849	0
Copa	0
AK010878	0
Nlrp3	0
Cope	0
Atp5g1	0
Gm5591	0
BRDN0000737840	0
Eif2b2	0
Gm5595	0
Eif2b1	0
Pcf11	0
Cd302	0
Iqcf1	0
Iqce	0
Iqcc	0
Iqca	0
Ippk	0
Ipp	0
Ipo8	0
Ipo7	0
Ipo5	0
Ipo4	0
Ipo11	0
Ip6k3	0
Invs	0
Ints9	0
Ints8	0
Ints7	0
Ints6	0
Ints5	0
Ints10	0
Insrr	0
Insr	0
Insm1	0
Insl6	0
Insl5	0
Insig2	0
Insig1	0
Ins2	0
Ins1	0
Inppl1	0
Inpp5j	0
Inpp5f	0
Inpp5d	0
Inpp5b	0
Inpp4a	0
Inpp1	0
Ino80d	0
Ino80c	0
Ino80b	0
Ino80	0
Inmt	0
Inip	0
Inhbe	0
Inhbc	0
Inhbb	0
Inha	0
Ing5	0
Ing4	0
Ing2	0
Ing1	0
Incenp	0
Inadl	0
Ina	0
Impg1	0
Impdh2	0
Impdh1	0
Impact	0
Impa2	0
Impa1	0
Imp4	0
Imp3	0
Immt	0
Ilvbl	0
Ilkap	0
Ilk	0
Ilf3	0
Ildr2	0
Il7r	0
Il7	0
Il6st	0
Il6ra	0
Il5ra	0
Il4ra	0
Il4	0
Il34	0
Il33	0
Il31ra	0
Il31	0
Il2rg	0
Il2rb	0
Il2ra	0
Il27	0
Il25	0

Gnmt	0	Il1b	0	Anp32b	0	Il24	0
Gnl3l	0	Il19	0	Rad52	0	Il23r	0
Gnl3	0	Il18rap	0	Rad51	0	Il22ra2	0
Gnl2	0	Il18r1	0	Anp32a	0	Il22ra1	0
Gngt1	0	Il18	0	Atp5g2	0	Il22	0
Gng8	0	Il17re	0	Rnf19b	0	Il20rb	0
Gng7	0	Il17rd	0	Mfap1b	0	Il20	0
Gng4	0	Il17rc	0	Mfap1a	0	Il2	0
Gng13	0	Il17rb	0	Ankrd52	0	Il1rn	0
Gng12	0	Il16	0	Zfp455	0	Il1rl2	0
Gng11	0	Il15ra	0	Nkiras1	0	Il1rl1	0
Gng10	0	Il13ra2	0	Pebp4	0	Il1rapl1	0
Gne	0	Il13	0	Gdap2	0	Il1rap	0
Gnb5	0	Il12rb2	0	Gdap1	0	Il1r2	0
Gnb2l1	0	Il12rb1	0	Ppil2	0	Il1r1	0
Gnb2	0	Il12a	0	BRDN0000737667	0	Il1f9	0
Gnb1	0	Il10ra	0	Gm10413	0	Il1f5	0
Gnaz	0	Il10	0	Olfr691	0	Il1f10	0
Gnat3	0	Ikzf4	0	Zfp456	0	Il1b	0
Gnat2	0	Ikzf2	0	Foxf1	0	Il1a	0
Gnat1	0	Ikzf1	0	Mpc1	0	Il19	0
Gnas	0	Ikbkg	0	Mcee	0	Il18rap	0
Gnaq	0	Ikbkb	0	Sorcs1	0	Il18r1	0
Gnao1	0	Ikbkap	0	Olfr161	0	Il18bp	0
Gnai3	0	Ikbip	0	Camk2b	0	Il18	0
Gnai2	0	Ik	0	Ptpn2	0	Il17rc	0
Gna15	0	Iigp1	0	Mpeg1	0	Il17f	0
Gna14	0	Ihh	0	Olfr160	0	Il17d	0
Gna13	0	Igsf9b	0	Myh6	0	Il17b	0
Gna12	0	Igsf9	0	Ppil3	0	Il17a	0
Gmppb	0	Igsf8	0	Cirh1a	0	Il16	0
Gmppa	0	Igsf6	0	Zfp341	0	Il15ra	0
Gmnn	0	Igsf3	0	Gm21379	0	Il13ra2	0
Gml	0	Igsf11	0	Kpnb1	0	Il13ra1	0
Gmip	0	Igsf10	0	Zfp345	0	Il13	0
Gmfg	0	Igsf1	0	Olfr166	0	Il12rb2	0
Gmfb	0	Igll1	0	Smlr1	0	Il12rb1	0
Gmeb2	0	Ighmbp2	0	Gapt	0	Il10rb	0
Gmeb1	0	Igfbpl1	0	Olfr697	0	Ikzf5	0
Gmds	0	Igfbp7	0	Bst2	0	Ikzf4	0
Gmcl1l	0	Igfbp6	0	Gm597	0	Ikzf2	0
Gmcl1	0	Igfbp5	0	Gm4177	0	Ikzf1	0
Gm9992	0	Igfbp2	0	Gm595	0	Ikbkg	0
Gm996	0	Igfbp1	0	Gm4175	0	Ikbkb	0
Gm973	0	Igfals	0	Alox12b	0	Ikbkap	0
Gm960	0	Igf2bp3	0	Gm590	0	Ikbip	0
Gm9513	0	Igf2bp2	0	Scgb2b17	0	Ik	0
Gm94	0	Igf2bp1	0	Phka1	0	Iigp1	0
Gm9376	0	Igf2	0	Scgb2b12	0	Ihh	0
Gm933	0	Igf1r	0	Luzp4	0	Igtp	0
Gm9268	0	Igdcc4	0	Nmral1	0	Igsf9b	0
Gm9125	0	Ift88	0	Smagp	0	Igsf9	0
Gm9112	0	Ift81	0	Zbbx	0	Igsf8	0
Gm906	0	Ift80	0	Scgb2b19	0	Igsf5	0
Gm904	0	Ift74	0	Nubp2	0	Igsf3	0
Gm9	0	Ift57	0	Nubp1	0	Igsf23	0
Gm8923	0	Ift52	0	Dhx29	0	Igsf21	0
Gm8882	0	Ift46	0	Myo5c	0	Igsf10	0
Gm884	0	Ift43	0	Chst15	0	Igsf1	0
Gm8765	0	Ift27	0	Btnl2	0	Iglon5	0
Gm8720	0	Ift22	0	Zfp207	0	Ighmbp2	0
Gm8677	0	Ift20	0	Gga1	0	Igfn1	0
Gm853	0	Ift172	0	Zfp202	0	Igfbpl1	0
Gm8439	0	Ift140	0	Ntf5	0	Igfbp5	0
Gm8369	0	Ift122	0	Htr4	0	Igfbp3	0
Gm8300	0	Ifrd2	0	Olfr247	0	Igfbp1	0
Gm8298	0	Ifrd1	0	Icos	0	Igf2r	0
Gm8267	0	Ifnz	0	Cdk19	0	Igf2bp2	0
Gm826	0	Ifnlr1	0	Defa20	0	Igf2bp1	0
Gm815	0	Ifnl3	0	Prl8a9	0	Igf2	0
Gm813	0	Ifnl2	0	Prl8a8	0	Igdcc4	0
Gm7978	0	Ifnk	0	Prl8a6	0	Igbp1b	0
Gm7903	0	Ifngr2	0	Cdk10	0	Igbp1	0
Gm773	0	Ifngr1	0	Cdk16	0	Ift88	0
Gm766	0	Ifng	0	Cdk17	0	Ift81	0
Gm7534	0	Ifnb1	0	Olfr248	0	Ift80	0
Gm7361	0	Ifnar2	0	Tcirg1	0	Ift74	0
Gm7325	0	Ifnar1	0	Olfr1112	0	Ift57	0
Gm732	0	Ifnab	0	Syp	0	Ift52	0
Gm7257	0	Ifna9	0	Otol1	0	Ift43	0
Gm7168	0	Ifna7	0	Scnn1a	0	Ift20	0
Gm7157	0	Ifna6	0	Gm4312	0	Ift172	0

Gm7102	0	Ifna5	0	Fbf1	0	Ift140	0
Gm694	0	Ifna4	0	Ccna2	0	Ift122	0
Gm6902	0	Ifna2	0	Vax2	0	Ifrd1	0
Gm6880	0	Ifna16	0	Vax1	0	Ifnz	0
Gm6812	0	Ifna15	0	Ccna1	0	Ifnlr1	0
Gm6792	0	Ifna14	0	Metap1d	0	Ifnl3	0
Gm6710	0	Ifna13	0	Cab39	0	Ifnk	0
Gm6696	0	Ifna12	0	Prpf4b	0	Ifngr1	0
Gm6592	0	Ifna11	0	Nubpl	0	Ifng	0
Gm6588	0	Ifna1	0	Gm3336	0	Ifne	0
Gm6583	0	Ifltd1	0	Cldn8	0	Ifnar2	0
Gm6537	0	Ifitm6	0	Igbp1b	0	Ifnar1	0
Gm6484	0	Ifit3	0	Adgrl4	0	Ifna7	0
Gm648	0	Ifit2	0	Kprp	0	Ifna6	0
Gm6460	0	Ifit1	0	Adgrl2	0	Ifna15	0
Gm6432	0	Ifih1	0	Arcn1	0	Ifna14	0
Gm6408	0	Ifi47	0	Adgrl1	0	Ifna13	0
Gm6377	0	Ifi44	0	Cldn1	0	Ifna11	0
Gm6164	0	Ifi35	0	Cldn2	0	Ifna1	0
Gm614	0	Ifi30	0	Cldn3	0	Ifltd1	0
Gm6121	0	Ifi27l2b	0	Cldn4	0	Ifitm6	0
Gm609	0	Ifi27	0	Cldn5	0	Ifitm5	0
Gm6086	0	Ifi204	0	Hist3h2a	0	Ifitm3	0
Gm608	0	Ifi203	0	Cldn7	0	Ifitm2	0
Gm597	0	Ifi202b	0	Cnr1	0	Ifit3	0
Gm595	0	Ier5	0	Sspo	0	Ifit2	0
Gm5941	0	Ier3	0	Olfr591	0	Ifit1	0
Gm5938	0	Ier2	0	Casp8ap2	0	Ifih1	0
Gm5936	0	Idnk	0	Umod	0	Ifi44	0
Gm5935	0	Idi1	0	Cldnd1	0	Ifi35	0
Gm5934	0	Idh3b	0	Psmc3ip	0	Ifi30	0
Gm5916	0	Idh3a	0	Cldnd2	0	Ifi27l2a	0
Gm5901	0	Idh2	0	Nudt12	0	Ifi204	0
Gm590	0	Idh1	0	Nudt13	0	Ifi203	0
Gm5891	0	Id2	0	Rcor1	0	Ifi202b	0
Gm5885	0	Id1	0	Nudt11	0	Iffo2	0
Gm5800	0	Icmt	0	Clta	0	Ier5l	0
Gm5797	0	Ick	0	Nudt17	0	Ier5	0
Gm5796	0	Ice2	0	Nudt14	0	Ier3ip1	0
Gm5771	0	Ice1	0	Cltb	0	Idua	0
Gm5728	0	Icam5	0	Serpini1	0	Ids	0
Gm5726	0	Icam4	0	Serpini2	0	Ido2	0
Gm572	0	Icam1	0	Prkra	0	Idi2	0
Gm5662	0	Ica1l	0	Accs	0	Idi1	0
Gm5640	0	Ibsp	0	Cd300e	0	Idh3g	0
Gm5635	0	Iba57	0	Vmn1r229	0	Idh3b	0
Gm5617	0	Iars2	0	Tchp	0	Idh3a	0
Gm5615	0	Iars	0	Cpt1b	0	Idh1	0
Gm561	0	Iah1	0	Pkn1	0	Id3	0
Gm5595	0	I830012O16Rik	0	Cldn10	0	Id1	0
Gm5592	0	Hypk	0	Naglu	0	Icos	0
Gm5538	0	Hyou1	0	Pkn2	0	Icmt	0
Gm5483	0	Hyls1	0	Gulo	0	Ick	0
Gm5464	0	Hydin	0	Ppp1r14a	0	Ice2	0
Gm5458	0	Hyal5	0	Dnajc2	0	Ice1	0
Gm5415	0	Hyal4	0	Vdr	0	Icam5	0
Gm5414	0	Hyal3	0	BRDN0000737660	0	Icam4	0
Gm5382	0	Hyal1	0	Ginm1	0	Icam2	0
Gm5346	0	Huwe1	0	Ifi202b	0	Ica1l	0
Gm527	0	Hus1b	0	Maml3	0	Iars2	0
Gm525	0	Hus1	0	Ppp1r1a	0	Iars	0
Gm5169	0	Hunk	0	Rps6ka1	0	Iapp	0
Gm5150	0	Htra3	0	Rps6ka2	0	I830077J02Rik	0
Gm5148	0	Htra2	0	Rps6ka3	0	I830012O16Rik	0
Gm5142	0	Htra1	0	Rps6ka4	0	Hypm	0
Gm5134	0	Htr7	0	Rps6ka5	0	Hypk	0
Gm5128	0	Htr6	0	Rps6ka6	0	Hyls1	0
Gm5127	0	Htr5b	0	Uqcr10	0	Hykk	0
Gm5114	0	Htr4	0	Uqcr11	0	Hydin	0
Gm5113	0	Htr3b	0	Tnfsf10	0	Hyal6	0
Gm5111	0	Htr3a	0	Tchh	0	Hyal5	0
Gm5108	0	Htr1b	0	Vmn1r69	0	Hyal3	0
Gm5082	0	Htr1a	0	Vmn1r68	0	Hyal1	0
Gm4981	0	Htatip2	0	Stambpl1	0	Hvcn1	0
Gm4980	0	Hsph1	0	Vmn1r65	0	Huwe1	0
Gm4952	0	Hspe1	0	Vmn1r64	0	Hus1	0
Gm4951	0	Hspd1	0	Vmn1r63	0	Hunk	0
Gm4944	0	Hspb8	0	Vmn1r62	0	Htt	0
Gm4937	0	Hspb6	0	Vmn1r61	0	Htra3	0
Gm4907	0	Hspb3	0	Fam171a1	0	Htra1	0
Gm4906	0	Hspb2	0	1110038F14Rik	0	Htr6	0
Gm4858	0	Hspb11	0	Ubqlnl	0	Htr5b	0

Gm4847	0	Hspb1	0	Vrk2	0	Htr5a	0
Gm4841	0	Hspa9	0	Morc3	0	Htr3b	0
Gm4836	0	Hspa8	0	Slc3a2	0	Htr3a	0
Gm4794	0	Hspa4l	0	Olfr1214	0	Htr2a	0
Gm4791	0	Hspa4	0	Slc3a1	0	Htr1f	0
Gm4788	0	Hspa1l	0	Ceacam1	0	Htr1b	0
Gm4787	0	Hspa1b	0	Ceacam3	0	Htr1a	0
Gm4763	0	Hspa1a	0	Bloc1s4	0	Htatsf1	0
Gm4745	0	Hspa13	0	Ceacam5	0	Htatip2	0
Gm4724	0	Hspa12a	0	Plin1	0	Hspg2	0
Gm4567	0	Hsp90b1	0	Glo1	0	Hspe1	0
Gm4498	0	Hsp90aa1	0	Ceacam9	0	Hspd1	0
Gm4477	0	Hsh2d	0	Bnc1	0	Hspb7	0
Gm4461	0	Hsfy2	0	Boc	0	Hspb6	0
Gm44	0	Hsf5	0	Entpd6	0	Hspb3	0
Gm436	0	Hsf4	0	Il4i1	0	Hspb2	0
Gm4340	0	Hsf2bp	0	Bloc1s2	0	Hspb11	0
Gm4312	0	Hsf2	0	Wfdc15a	0	Hspb1	0
Gm4307	0	Hsdl1	0	Wfdc15b	0	Hspa9	0
Gm4305	0	Hsd3b7	0	Prr9	0	Hspa8	0
Gm4303	0	Hsd3b6	0	Slc14a1	0	Hspa5	0
Gm4302	0	Hsd3b5	0	Treml1	0	Hspa4l	0
Gm4301	0	Hsd3b2	0	Gm14085	0	Hspa2	0
Gm4297	0	Hsd17b6	0	Ppp1r11	0	Hspa1l	0
Gm428	0	Hsd17b4	0	Ppp1r10	0	Hspa1a	0
Gm4214	0	Hsd17b3	0	Scn2a1	0	Hspa14	0
Gm4201	0	Hsd17b2	0	Ppp1r17	0	Hspa13	0
Gm4177	0	Hsd17b11	0	Cdr2	0	Hspa12a	0
Gm4175	0	Hsd17b10	0	Alkbh6	0	Hsp90ab1	0
Gm4141	0	Hsd11b2	0	Ppp1r18	0	Hsp90aa1	0
Gm4133	0	Hscb	0	Alkbh4	0	Hsh2d	0
Gm4070	0	Hsbp1l1	0	Alkbh5	0	Hsfy2	0
Gm4064	0	Hsbp1	0	Alkbh2	0	Hsf5	0
Gm382	0	Hs6st3	0	Alkbh3	0	Hsf2bp	0
Gm3776	0	Hs3st6	0	Cfc1	0	Hsf2	0
Gm3750	0	Hs3st5	0	Rpl39l	0	Hsf1	0
Gm3701	0	Hs3st4	0	Tmem47	0	Hsdl2	0
Gm3696	0	Hs3st3b1	0	Olfr820	0	Hsd3b6	0
Gm3646	0	Hs3st3a1	0	Zkscan1	0	Hsd3b5	0
Gm364	0	Hs2st1	0	Pik3c2b	0	Hsd17b7	0
Gm362	0	Hrsp12	0	Pik3c2a	0	Hsd17b4	0
Gm3500	0	Hrnr	0	Zkscan2	0	Hsd17b3	0
Gm3488	0	Hrk	0	Zkscan5	0	Hsd17b13	0
Gm3415	0	Hrh4	0	Zkscan4	0	Hsd17b11	0
Gm3404	0	Hrh3	0	Zkscan7	0	Hsd17b10	0
Gm3376	0	Hrh1	0	Tor1b	0	Hsd11b1	0
Gm3336	0	Hrct1	0	Zkscan8	0	Hscb	0
Gm3286	0	Hrasls	0	Mrgprh	0	Hsbp1l1	0
Gm3259	0	Hras	0	Btnl9	0	Hsbp1	0
Gm3238	0	Hr	0	Vmn2r90	0	Hs6st3	0
Gm3139	0	Hpx	0	Pcdhac1	0	Hs6st2	0
Gm2a	0	Hpse	0	Cyp2c69	0	Hs6st1	0
Gm2933	0	Hps6	0	Snf8	0	Hs3st6	0
Gm2913	0	Hps4	0	Comp	0	Hs3st5	0
Gm2897	0	Hps3	0	Amd1	0	Hs3st4	0
Gm2863	0	Hps1	0	Ssxb2	0	Hs3st3a1	0
Gm2825	0	Hprt	0	Mrgprd	0	Hs3st1	0
Gm2799	0	Hpn	0	Mrgpre	0	Hs1bp3	0
Gm2663	0	Hpgds	0	Mrgprg	0	Hrnr	0
Gm266	0	Hpgd	0	Aloxe3	0	Hrk	0
Gm21975	0	Hpdl	0	Gadd45gip1	0	Hrh4	0
Gm21951	0	Hpd	0	Vmn1r104	0	Hrh3	0
Gm21950	0	Hpcal4	0	Vmn1r223	0	Hrh2	0
Gm21943	0	Hpcal1	0	1110032F04Rik	0	Hrh1	0
Gm21671	0	Hp1bp3	0	Amy2a5	0	Hrct1	0
Gm21637	0	Hp	0	Glce	0	Hrc	0
Gm21498	0	Hoxd4	0	Clvs2	0	Hrasls5	0
Gm21319	0	Hoxd13	0	Fstl1	0	Hras	0
Gm21119	0	Hoxd12	0	Eef2k	0	Hr	0
Gm21002	0	Hoxd11	0	Loxl1	0	Hpse2	0
Gm20917	0	Hoxc13	0	Loxl2	0	Hpse	0
Gm20877	0	Hoxc12	0	Loxl4	0	Hps6	0
Gm20867	0	Hoxb9	0	Pagr1a	0	Hps5	0
Gm20865	0	Hoxb8	0	Hagh	0	Hps4	0
Gm20854	0	Hoxb6	0	Ssxb9	0	Hps1	0
Gm20852	0	Hoxb5	0	Dnajc30	0	Hpn	0
Gm20831	0	Hoxb3	0	2900011O08Rik	0	Hpgds	0
Gm20823	0	Hoxb2	0	Osr1	0	Hpgd	0
Gm20822	0	Hoxb1	0	BRDN0000737501	0	Hpdl	0
Gm20816	0	Hoxa9	0	Ccnt1	0	Hpd	0
Gm20809	0	Hoxa7	0	Puf60	0	Hpcal4	0
Gm20767	0	Hoxa5	0	Tnni2	0	Hpcal1	0

Gm20747	0	Hoxa4	0	Olfr167	0	Hoxd8	0
Gm20738	0	Hoxa3	0	Eif4ebp3	0	Hoxd4	0
Gm20736	0	Hoxa2	0	Eif4ebp2	0	Hoxd13	0
Gm20604	0	Hoxa13	0	Man2b2	0	Hoxd12	0
Gm20594	0	Hoxa11	0	Paxbp1	0	Hoxd11	0
Gm2030	0	Hoxa10	0	Pdyn	0	Hoxd10	0
Gm2016	0	Hoxa1	0	Mboat2	0	Hoxc9	0
Gm2012	0	Hormad2	0	Gm4951	0	Hoxc6	0
Gm1993	0	Hormad1	0	Gm4952	0	Hoxc5	0
Gm1979	0	Hook3	0	Zkscan16	0	Hoxc4	0
Gm19668	0	Hook2	0	Olfr827	0	Hoxc13	0
Gm17689	0	Homez	0	Slc13a1	0	Hoxc11	0
Gm17677	0	Homer3	0	Slc13a2	0	Hoxc10	0
Gm17455	0	Homer2	0	Slc13a3	0	Hoxb9	0
Gm17359	0	Homer1	0	Slc13a4	0	Hoxb8	0
Gm17296	0	Hnrnpul2	0	Slc13a5	0	Hoxb7	0
Gm17252	0	Hnrnpul1	0	Akp3	0	Hoxb5	0
Gm17019	0	Hnrnpu	0	Vmn2r39	0	Hoxb4	0
Gm1673	0	Hnrnpr	0	Pcdhb12	0	Hoxb2	0
Gm1661	0	Hnrnpm	0	Pcdhb11	0	Hoxb13	0
Gm166	0	Hnrnpl	0	Pcdhb10	0	Hoxb1	0
Gm16513	0	Hnrnpk	0	Pcdhb17	0	Hoxa9	0
Gm16501	0	Hnrnph3	0	Pcdhb16	0	Hoxa6	0
Gm16451	0	Hnrnph2	0	Pcdhb15	0	Hoxa5	0
Gm16432	0	Hnrnph1	0	Pcdhb14	0	Hoxa4	0
Gm16390	0	Hnrnpf	0	Vmn2r31	0	Hoxa3	0
Gm16381	0	Hnrnpdl	0	Vmn2r30	0	Hoxa13	0
Gm1604b	0	Hnrnpd	0	Vmn2r33	0	Hoxa11	0
Gm1587	0	Hnrnpab	0	Vmn2r32	0	Hoxa10	0
Gm15800	0	Hnrnpa3	0	Vmn2r35	0	Hoxa1	0
Gm156	0	Hnrnpa1	0	Vmn2r34	0	Hormad2	0
Gm1553	0	Hnf4g	0	Yaf2	0	Hormad1	0
Gm15386	0	Hnf4a	0	Vmn2r36	0	Hook3	0
Gm15319	0	Hn1	0	Eif3h	0	Hook2	0
Gm15308	0	Hmx2	0	Ero1l	0	Homez	0
Gm15299	0	Hmox1	0	Urm1	0	Homer3	0
Gm15292	0	Hmha1	0	Zfp598	0	Homer2	0
Gm15284	0	Hmgxb4	0	Zfp599	0	Homer1	0
Gm1527	0	Hmgxb3	0	BRDN0000737984	0	Hoga1	0
Gm15140	0	Hmgn5	0	Zfp595	0	Hnrnpm	0
Gm15127	0	Hmgn3	0	Zfp597	0	Hnrnpll	0
Gm15114	0	Hmgn1	0	Prkaa2	0	Hnrnpl	0
Gm15107	0	Hmgcs1	0	Prkaa1	0	Hnrnpk	0
Gm15097	0	Hmgcr	0	Zfp593	0	Hnrnph3	0
Gm15093	0	Hmgcll1	0	Grwd1	0	Hnrnpdl	0
Gm15091	0	Hmgcl	0	BRDN0000737591	0	Hnrnpc	0
Gm15085	0	Hmgb4	0	E330034G19Rik	0	Hnrnpa3	0
Gm15023	0	Hmgb3	0	Gpr45	0	Hnrnpa2b1	0
Gm14920	0	Hmgb1	0	Hemt1	0	Hnrnpa1	0
Gm14851	0	Hmga1-rs1	0	Icosl	0	Hnrnpa0	0
Gm14850	0	Hmg20b	0	Pofut2	0	Hnmt	0
Gm14781	0	Hmg20a	0	Dennd3	0	Hnf4g	0
Gm14744	0	Hmcn1	0	Pofut1	0	Hnf4a	0
Gm14743	0	Hmces	0	Taf7	0	Hnf1a	0
Gm14632	0	Hmbs	0	Taf5	0	Hn1l	0
Gm14548	0	Hmbox1	0	Snw1	0	Hn1	0
Gm14511	0	Hlf	0	Taf3	0	Hmx3	0
Gm14501	0	Hkdc1	0	Taf2	0	Hmmr	0
Gm14496	0	Hk3	0	Olfr1019	0	Hmha1	0
Gm14479	0	Hk2	0	Scara5	0	Hmgxb4	0
Gm14474	0	Hk1	0	Snn	0	Hmgxb3	0
Gm14459	0	Hjurp	0	Max	0	Hmgn5	0
Gm14458	0	Hist4h4	0	Smc3	0	Hmgn2	0
Gm14446	0	Hist3h2a	0	Smc2	0	Hmgn1	0
Gm14440	0	Hist2h4	0	Smc5	0	Hmgcs1	0
Gm14434	0	Hist2h3c1	0	Smc4	0	Hmgb4	0
Gm14431	0	Hist2h3b	0	Slc33a1	0	Hmgb3	0
Gm14420	0	Hist2h2be	0	Map2k3	0	Hmgb2	0
Gm14393	0	Hist2h2ab	0	Actl7a	0	Hmgb1	0
Gm14374	0	Hist2h2aa2	0	Gtsf1l	0	Hmga1	0
Gm14351	0	Hist2h2aa1	0	Map2k7	0	Hmg20a	0
Gm14347	0	Hist1h4m	0	Pxdn	0	Hmces	0
Gm14346	0	Hist1h4k	0	Maf	0	Hmbs	0
Gm14345	0	Hist1h4j	0	Nmnat2	0	Hltf	0
Gm14325	0	Hist1h4i	0	Mak	0	Hlf	0
Gm14322	0	Hist1h4h	0	Gm11757	0	Hk3	0
Gm14308	0	Hist1h4f	0	L3mbtl4	0	Hk2	0
Gm14305	0	Hist1h4d	0	Eif3d	0	Hk1	0
Gm14151	0	Hist1h4c	0	Cml3	0	Hjurp	0
Gm14137	0	Hist1h4b	0	Adh1	0	Hivep3	0
Gm14124	0	Hist1h3i	0	Cask	0	Hivep1	0
Gm14085	0	Hist1h3h	0	Cast	0	Hist4h4	0

Gm13889	0	Hist1h3f	0	Zhx3	0	Hist3h2a	0
Gm13871	0	Hist1h3e	0	Insig2	0	Hist2h4	0
Gm13769	0	Hist1h3b	0	Gm14501	0	Hist2h3b	0
Gm136	0	Hist1h2bp	0	Casr	0	Hist2h2be	0
Gm13547	0	Hist1h2bn	0	Adh7	0	Hist2h2bb	0
Gm13306	0	Hist1h2bm	0	Npffr1	0	Hist2h2ac	0
Gm13298	0	Hist1h2bk	0	Foxe3	0	Hist2h2ab	0
Gm13290	0	Hist1h2bh	0	Styx	0	Hist2h2aa2	0
Gm13288	0	Hist1h2bf	0	Ltv1	0	Hist2h2aa1	0
Gm13287	0	Hist1h2bb	0	Scgb2b7	0	Hist1h4j	0
Gm13285	0	Hist1h2ba	0	Scgb2b2	0	Hist1h4h	0
Gm13283	0	Hist1h2ao	0	Csn1s1	0	Hist1h4f	0
Gm13279	0	Hist1h2an	0	Olfr1269	0	Hist1h4d	0
Gm13278	0	Hist1h2ak	0	Actr1b	0	Hist1h4a	0
Gm13277	0	Hist1h2ai	0	Actr1a	0	Hist1h3i	0
Gm13276	0	Hist1h2ah	0	Ppdpf	0	Hist1h3h	0
Gm13251	0	Hist1h2ag	0	Prox1	0	Hist1h3g	0
Gm13247	0	Hist1h2af	0	Prox2	0	Hist1h3f	0
Gm13242	0	Hist1h2ae	0	Smug1	0	Hist1h3e	0
Gm13212	0	Hist1h2ad	0	Mrpl23	0	Hist1h3d	0
Gm13178	0	Hist1h2ab	0	Frmpd3	0	Hist1h3c	0
Gm13177	0	Hist1h2aa	0	Atp8b5	0	Hist1h3b	0
Gm13157	0	Hist1h1t	0	BRDN0000738215	0	Hist1h3a	0
Gm13154	0	Hist1h1c	0	Olfml1	0	Hist1h2bp	0
Gm13139	0	Hist1h1b	0	Islr	0	Hist1h2bn	0
Gm13125	0	Hist1h1a	0	Olfml3	0	Hist1h2bl	0
Gm13124	0	Hirip3	0	Rep15	0	Hist1h2bk	0
Gm13119	0	Hira	0	Tinagl1	0	Hist1h2bh	0
Gm13103	0	Hipk3	0	Pkdcc	0	Hist1h2bf	0
Gm13088	0	Hipk2	0	Gm3286	0	Hist1h2ba	0
Gm13083	0	Hipk1	0	Tbl2	0	Hist1h2ap	0
Gm13078	0	Hint3	0	Tbl3	0	Hist1h2ao	0
Gm13057	0	Hint2	0	Kdm5d	0	Hist1h2an	0
Gm13051	0	Hint1	0	Disp2	0	Hist1h2ak	0
Gm13040	0	Hinfp	0	Plekhd1	0	Hist1h2ai	0
Gm13023	0	Hils1	0	1810055G02Rik	0	Hist1h2ah	0
Gm12942	0	Hilpda	0	Kdm5c	0	Hist1h2af	0
Gm12888	0	Higd2a	0	Otud7a	0	Hist1h2ae	0
Gm12887	0	Higd1b	0	Ccdc38	0	Hist1h2ad	0
Gm12794	0	Higd1a	0	Ccdc39	0	Hist1h2ac	0
Gm12789	0	Hif3a	0	Atp6ap1l	0	Hist1h2ab	0
Gm12695	0	Hif1an	0	Olfr944	0	Hist1h1t	0
Gm12429	0	Hif1a	0	Ccdc30	0	Hist1h1e	0
Gm12253	0	Hid1	0	4930567H17Rik	0	Hist1h1d	0
Gm12185	0	Hic2	0	Ccdc36	0	Hist1h1c	0
Gm11937	0	Hic1	0	Ccdc37	0	Hist1h1b	0
Gm11744	0	Hibch	0	Ccdc34	0	Hist1h1a	0
Gm11711	0	Hibadh	0	2210407C18Rik	0	Hira	0
Gm11710	0	Hiatl1	0	Gtf3c3	0	Hipk4	0
Gm11596	0	Hiat1	0	Nefm	0	Hipk3	0
Gm11595	0	Hhla1	0	Gtf3c1	0	Hipk2	0
Gm11569	0	Hhipl2	0	1600015I10Rik	0	Hip1	0
Gm11568	0	Hhipl1	0	Gtf3c6	0	Hint3	0
Gm11567	0	Hhip	0	Gtf3c5	0	Hinfp	0
Gm11565	0	Hhex	0	Gtf3c4	0	Hils1	0
Gm11563	0	Hhatl	0	Fam209	0	Hilpda	0
Gm11554	0	Hgs	0	Olfr1265	0	Higd1b	0
Gm11541	0	Hgfac	0	Topors	0	Higd1a	0
Gm11487	0	Heyl	0	Gm364	0	Hif3a	0
Gm11437	0	Hey2	0	Zfp54	0	Hif1a	0
Gm11237	0	Hey1	0	BRDN0000737680	0	Hid1	0
Gm1123	0	Hexim2	0	Isl1	0	Hic2	0
Gm11128	0	Hexim1	0	Isl2	0	Hic1	0
Gm11127	0	Hexdc	0	Edem1	0	Hibadh	0
Gm1110	0	Hexb	0	Endog	0	Hiatl1	0
Gm10922	0	Hexa	0	Gm4788	0	Hiat1	0
Gm10921	0	Hes7	0	Fam228b	0	Hhla1	0
Gm10778	0	Herpud2	0	Syce1	0	Hhipl2	0
Gm10767	0	Herc6	0	Syce3	0	Hhipl1	0
Gm10697	0	Herc4	0	Syce2	0	Hhip	0
Gm10696	0	Herc3	0	Eps8l2	0	Hhex	0
Gm10670	0	Herc2	0	Med7	0	Hhat	0
Gm10665	0	Herc1	0	Med4	0	Hgs	0
Gm10662	0	Hephl1	0	Ift46	0	Hgfac	0
Gm10639	0	Heph	0	2410004P03Rik	0	Hgf	0
Gm10471	0	Hepacam2	0	Med8	0	Hgd	0
Gm1045	0	Henmt1	0	Endov	0	Hfm1	0
Gm10439	0	Hemt1	0	Endou	0	Hfe2	0
Gm10436	0	Hemk1	0	Erlin1	0	Hfe	0
Gm10413	0	Hemgn	0	Cxadr	0	Heyl	0
Gm10408	0	Helz	0	Tmem248	0	Hey1	0
Gm10377	0	Helt	0	Erlin2	0	Hexim2	0

Gm10375	0
Gm10334	0
Gm10324	0
Gm10267	0
Gm10256	0
Gm10220	0
Gm10147	0
Gm10142	0
Gm10104	0
Gm10094	0
Gm10081	0
Gm10057	0
Gm10024	0
Glyr1	0
Glyctk	0
Glycam1	0
Glyatl3	0
Glud1	0
Gltscr2	0
Gltscr1	0
Gltp	0
Glt8d2	0
Glt8d1	0
Glt6d1	0
Glt28d2	0
Glt1d1	0
Gls2	0
Gls	0
Glrx5	0
Glrx2	0
Glrx	0
Glrp1	0
Glrb	0
Glra3	0
Glra2	0
Glra1	0
Glp2r	0
Glp1r	0
Glod5	0
Glod4	0
Glo1	0
Glmp	0
Glmn	0
Glis2	0
Glis1	0
Glipr2	0
Glipr1l2	0
Glipr1l1	0
Glipr1	0
Gli2	0
Glg1	0
Gle1	0
Glb1l	0
Glb1	0
Gla	0
Gkn3	0
Gkn2	0
Gkn1	0
Gk5	0
Gk2	0
Gje1	0
Gjd4	0
Gjc2	0
Gjc1	0
Gjb6	0
Gjb5	0
Gjb3	0
Gjb2	0
Gja6	0
Gja4	0
Gja10	0
Gja1	0
Gipr	0
Gipc3	0
Gipc2	0
Gipc1	0
Gip	0
Gins4	0
Gins2	0
Gins1	0
Ginm1	0
Gin1	0
Helq	0
Hells	0
Helb	0
Hebp2	0
Hebp1	0
Heatr9	0
Heatr6	0
Heatr5b	0
Heatr5a	0
Heatr3	0
Heatr1	0
Hdx	0
Hdlbp	0
Hdhd3	0
Hdhd2	0
Hdgfrp3	0
Hdgfl1	0
Hdgf	0
Hddc3	0
Hdac9	0
Hdac8	0
Hdac6	0
Hdac5	0
Hdac4	0
Hdac3	0
Hdac11	0
Hdac1	0
Hcst	0
Hcrtr1	0
Hcn4	0
Hcn3	0
Hcls1	0
Hck	0
Hcfc2	0
Hcfc1r1	0
Hcfc1	0
Hccs	0
Hcar1	0
Hc	0
Hbs1l	0
Hbq1b	0
Hbp1	0
Hbegf	0
Hbb-y	0
Hbb-bh2	0
Hbb-bh1	0
Hba-x	0
Hba-a2	0
Hba-a1	0
Hax1	0
Havcr2	0
Havcr1	0
Haus8	0
Haus7	0
Haus5	0
Haus4	0
Haus3	0
Haus2	0
Haus1	0
Hat1	0
Has3	0
Has2	0
Has1	0
Hars	0
Harbi1	0
Hapln4	0
Hapln3	0
Hapln1	0
Hap1	0
Hao2	0
Hao1	0
Hand2	0
Hand1	0
Hamp2	0
Hamp	0
Hal	0
Haghl	0
Hagh	0
Hadhb	0
Hadha	0
Hadh	0
Hacd4	0
BRDN0000737683	0
Arg1	0
Arg2	0
Cpt2	0
Kcnq2	0
Mreg	0
Tmem242	0
Tmem243	0
Bpifa2	0
Hapln1	0
Endod1	0
Olfr1016	0
Npcd	0
Dip2a	0
Lrrc47	0
Wdr81	0
BRDN0000738055	0
Il1rn	0
BRDN0000737724	0
Efna4	0
BRDN0000737722	0
BRDN0000738050	0
BRDN0000738053	0
BRDN0000738052	0
Ttc8	0
Nsg2	0
BRDN0000738059	0
BRDN0000738058	0
BRDN0000737728	0
Trim30d	0
Pparg	0
Ppard	0
Fasn	0
Tifa	0
Fasl	0
Dennd2d	0
Wipf3	0
5530400C23Rik	0
Wipf1	0
Bpifa5	0
Galnt11	0
Yeats2	0
Fam3a	0
Fam3b	0
Fam3c	0
BRDN0000738166	0
Hoxc11	0
Hoxc10	0
Hoxc13	0
Bbs12	0
BRDN0000737982	0
Ube2l3	0
Mroh9	0
Spty2d1	0
Rbm15b	0
Mroh8	0
BRDN0000737684	0
BRDN0000737593	0
BRDN0000738273	0
1700055N04Rik	0
BRDN0000738275	0
BRDN0000738274	0
BRDN0000738277	0
BRDN0000738276	0
BRDN0000738279	0
BRDN0000738278	0
Slc36a4	0
Slc36a3	0
Slc36a1	0
Cd177	0
Fbxo8	0
Hmcn1	0
Rpia	0
Bmper	0
Ficd	0
Mroh4	0
Tdrp	0
Il1r2	0
Il1r1	0
Slco1c1	0
Srprb	0
1810062G17Rik	0
Hexim1	0
Hexb	0
Hesx1	0
Hes7	0
Hes3	0
Hes2	0
Herpud2	0
Herpud1	0
Herc6	0
Herc4	0
Herc3	0
Herc2	0
Herc1	0
Heph	0
Hepacam2	0
Hepacam	0
Hemt1	0
Hemk1	0
Hemgn	0
Helz2	0
Helz	0
Helt	0
Helq	0
Hells	0
Helb	0
Hecw2	0
Hecw1	0
Hectd1	0
Heca	0
Hebp2	0
Hebp1	0
Heatr9	0
Heatr6	0
Heatr5a	0
Heatr1	0
Hdx	0
Hdlbp	0
Hdhd3	0
Hdhd2	0
Hdhd1a	0
Hdgfrp3	0
Hdc	0
Hdac9	0
Hdac8	0
Hdac7	0
Hdac6	0
Hdac4	0
Hdac3	0
Hdac1	0
Hcst	0
Hcrtr1	0
Hcrt	0
Hcn4	0
Hcn3	0
Hcn2	0
Hcn1	0
Hcls1	0
Hck	0
Hcfc2	0
Hcfc1r1	0
Hcfc1	0
Hccs	0
Hcar2	0
Hcar1	0
Hc	0
Hbs1l	0
Hbq1a	0
Hbp1	0
Hbb-y	0
Hbb-bh2	0
Hbb-bh1	0
Hba-x	0
Hba-a2	0
Havcr2	0
Havcr1	0
Haus8	0
Haus7	0
Haus5	0
Haus4	0
Haus3	0
Haus2	0
Has3	0

Gimap8	0	Hacd2	0	Ubiad1	0	Has2	0
Gimap6	0	Hacd1	0	Dync1i2	0	Has1	0
Gimap5	0	Habp4	0	Akr7a5	0	Hars	0
Gimap4	0	Haao	0	Olfr1012	0	Harbi1	0
Gimap3	0	H6pd	0	Ppp4r4	0	Hapln2	0
Gimap1	0	H60c	0	Lrrc69	0	Hapln1	0
Gigyf2	0	H60b	0	Patl1	0	Hand2	0
Gigyf1	0	H3f3b	0	Lsr	0	Hand1	0
Gid4	0	H3f3a	0	Lrrc63	0	Hamp2	0
Ghsr	0	H2-T24	0	Lrrc61	0	Hal	0
Ghrl	0	H2-T23	0	Leprotl1	0	Haghl	0
Ghrhr	0	H2-Q7	0	8430419L09Rik	0	Hagh	0
Ghrh	0	H2-Q6	0	2300005B03Rik	0	Hadha	0
Ghr	0	H2-Q4	0	Kptn	0	Hadh	0
Ghitm	0	H2-Q10	0	Gm8439	0	Hacl1	0
Gh	0	H2-Q1	0	E2f6	0	Hacd4	0
Ggta1	0	H2-Ob	0	Ccnjl	0	Hacd3	0
Ggt7	0	H2-Oa	0	Pisd	0	Hacd2	0
Ggt6	0	H2-M9	0	Lipo1	0	Hacd1	0
Ggt5	0	H2-M5	0	Rxfp2	0	Habp4	0
Ggt1	0	H2-M3	0	Rxfp4	0	Haao	0
Ggps1	0	H2-M10.6	0	Gm8923	0	H60c	0
Ggnbp2	0	H2-M10.3	0	Olfr1465	0	H60b	0
Ggnbp1	0	H2-M10.2	0	Olfr1466	0	H3f3b	0
Ggn	0	H2-Eb2	0	Olfr1395	0	H3f3a	0
Ggh	0	H2-DMb1	0	Olfr1393	0	H2-T24	0
Ggcx	0	H2-DMa	0	Olfr1462	0	H2-T23	0
Ggct	0	H2afz	0	Rnpc3	0	H2-Q6	0
Gga3	0	H2afy2	0	Ms4a10	0	H2-Q4	0
Gga2	0	H2afx	0	Olfr1469	0	H2-Q1	0
Gga1	0	H2afv	0	Rnh1	0	H2-Ob	0
Gfy	0	H2afb3	0	Fam50b	0	H2-Oa	0
Gfral	0	H2afb2	0	Defb21	0	H2-M9	0
Gfra4	0	H2afb1	0	Tert	0	H2-M5	0
Gfra3	0	H2-Ab1	0	Cdc16	0	H2-M3	0
Gfra2	0	H2-Aa	0	Tbc1d10b	0	H2-M10.6	0
Gfra1	0	H1fx	0	BRDN0000738025	0	H2-M10.5	0
Gfpt2	0	H1fnt	0	Nuak1	0	H2-M10.3	0
Gfpt1	0	H1f0	0	Nuak2	0	H2-M10.2	0
Gfod1	0	H13	0	Tmem259	0	H2-M10.1	0
Gfm1	0	Gzmg	0	Dffa	0	H2-M1	0
Gfi1b	0	Gzmf	0	Dffb	0	H2-Ke6	0
Gfi1	0	Gzme	0	Olfr765	0	H2-Eb2	0
Gfer	0	Gzmd	0	Klk12	0	H2-Eb1	0
Get4	0	Gzmb	0	Zcwpw1	0	H2-DMb2	0
Gemin7	0	Gys1	0	BRDN0000737877	0	H2-DMa	0
Gemin6	0	Gypc	0	Olfr761	0	H2-D1	0
Gemin5	0	Gyltl1b	0	Bcor	0	H2bfm	0
Gemin4	0	Gykl1	0	Nptxr	0	H2afy2	0
Gemin2	0	Gyg	0	Col4a4	0	H2afx	0
Gdpgp1	0	Gulo	0	Olfr769	0	H2afj	0
Gdpd5	0	Gucy2f	0	Olfr768	0	H2afb3	0
Gdpd4	0	Gucy2e	0	BRDN0000738021	0	H2afb2	0
Gdpd3	0	Gucy2d	0	A230065H16Rik	0	H2afb1	0
Gdpd1	0	Gucy2c	0	Nasp	0	H1f0	0
Gdnf	0	Gucy1b2	0	BRDN0000738161	0	H13	0
Gdi2	0	Gucy1a3	0	Gm4894	0	Gzmm	0
Gdi1	0	Guca2b	0	Olfr835	0	Gzmk	0
Gdf9	0	Guca2a	0	Sf3b4	0	Gzmg	0
Gdf6	0	Guca1b	0	Sf3b6	0	Gzmf	0
Gdf3	0	Guca1a	0	Olfr830	0	Gzmd	0
Gdf2	0	Gtsf1l	0	Sf3b3	0	Gzmc	0
Gdf11	0	Gtsf1	0	Syvn1	0	Gzmb	0
Gdf10	0	Gtse1	0	Dach1	0	Gzf1	0
Gde1	0	Gtpbp4	0	S100a9	0	Gys2	0
Gdap2	0	Gtpbp3	0	Gdnf	0	Gys1	0
Gdap1l1	0	Gtpbp10	0	BRDN0000737770	0	Gypc	0
Gdap1	0	Gtpbp1	0	S100a5	0	Gyltl1b	0
Gda	0	Gtf3c6	0	S100a4	0	Gykl1	0
Gcsh	0	Gtf3c5	0	Phldb2	0	Gyg	0
Gcsam	0	Gtf3c4	0	Phldb3	0	Gvin1	0
Gcnt4	0	Gtf3c3	0	Timm23	0	Gusb	0
Gcnt3	0	Gtf3a	0	Olfr406	0	Gulo	0
Gcnt2	0	Gtf2ird1	0	4932411N23Rik	0	Guk1	0
Gcnt1	0	Gtf2i	0	S100a2	0	Gucy2g	0
Gcn1l1	0	Gtf2h4	0	Ap3b1	0	Gucy2f	0
Gcm2	0	Gtf2h3	0	Pla2g6	0	Gucy2e	0
Gcm1	0	Gtf2h2	0	F3	0	Gucy2d	0
Gclm	0	Gtf2f2	0	F7	0	Gucy2c	0
Gclc	0	Gtf2f1	0	F8	0	Gucy1b2	0
Gck	0	Gtf2e2	0	2310007B03Rik	0	Gucy1a3	0
Gch1	0	Gtf2e1	0	Doxl2	0	Gucy1a2	0

Gcgr	0	Gtf2b	0	Armc3	0	Guca1b	0
Gcfc2	0	Gtf2a2	0	Rdh14	0	Guca1a	0
Gcdh	0	Gtf2a1	0	Rdh16	0	Gtsf1l	0
Gcc2	0	Gtdc1	0	Terf1	0	Gtsf1	0
Gcc1	0	Gsx2	0	Rdh10	0	Gtse1	0
Gcat	0	Gsx1	0	BRDN0000737777	0	Gtpbp8	0
Gca	0	Gstt3	0	Bco2	0	Gtpbp4	0
Gc	0	Gstt2	0	Hiatl1	0	Gtpbp3	0
Gbx2	0	Gstt1	0	Klk1b3	0	Gtpbp2	0
Gbx1	0	Gstp2	0	Gm14479	0	Gtpbp10	0
Gbp9	0	Gstp1	0	BRDN0000737776	0	Gtpbp1	0
Gbp8	0	Gsto2	0	Phtf1	0	Gtf3c6	0
Gbp6	0	Gsto1	0	Rdh19	0	Gtf3c5	0
Gbp5	0	Gstm4	0	Ccdc127	0	Gtf3c4	0
Gbp4	0	Gstm2	0	Farp2	0	Gtf3c1	0
Gbp3	0	Gstm1	0	Oraov1	0	Gtf3a	0
Gbp2b	0	Gstcd	0	Ccdc124	0	Gtf2ird1	0
Gbp2	0	Gsta4	0	Ccdc122	0	Gtf2i	0
Gbp11	0	Gsta3	0	Ccdc121	0	Gtf2h5	0
Gbp10	0	Gsta2	0	Ccdc120	0	Gtf2h4	0
Gbgt1	0	Gss	0	Cyp2c65	0	Gtf2h3	0
Gbe1	0	Gspt2	0	Ccdc129	0	Gtf2h2	0
Gbas	0	Gspt1	0	Ect2l	0	Gtf2f2	0
Gba2	0	Gsk3a	0	Slfn8	0	Gtf2f1	0
Gba	0	Gsg2	0	Slfn9	0	Gtf2e2	0
Gatsl3	0	Gsg1	0	Sh3glb2	0	Gtf2e1	0
Gatsl2	0	Gse1	0	Egr4	0	Gtf2a1l	0
Gatm	0	Gsdmc3	0	Disp1	0	Gtdc1	0
Gatc	0	Gsdmc2	0	Slfn2	0	Gsx2	0
Gatb	0	Gsdmc	0	Slfn3	0	Gsx1	0
Gatad2a	0	Gsdma3	0	Slfn1	0	Gstz1	0
Gatad1	0	Gsdma2	0	Slfn4	0	Gstt3	0
Gata6	0	Gsc	0	Stx4a	0	Gstt2	0
Gata5	0	Gsap	0	Vps45	0	Gstt1	0
Gata4	0	Grxcr2	0	Lpgat1	0	Gstp2	0
Gata3	0	Grxcr1	0	Vps41	0	Gstp1	0
Gata2	0	Grwd1	0	Odf2l	0	Gsto1	0
Gas8	0	Grtp1	0	Pcyox1	0	Gstm7	0
Gas7	0	Grsf1	0	Olfr905	0	Gstm6	0
Gas6	0	Grrp1	0	Irx6	0	Gstm4	0
Gas2l3	0	Grpr	0	Tnni3k	0	Gstm1	0
Gas2l2	0	Grpel2	0	Usf2	0	Gstcd	0
Gas2l1	0	Grpel1	0	BRDN0000737871	0	Gsta4	0
Gas2	0	Grm8	0	Gm8693	0	Gsta3	0
Gas1	0	Grm7	0	Klri2	0	Gsr	0
Gart	0	Grm6	0	2310061N02Rik	0	Gspt1	0
Gars	0	Grm5	0	BRDN0000737665	0	Gsn	0
Garem	0	Grm4	0	Pcbd1	0	Gskip	0
Gapvd1	0	Grm3	0	Micu3	0	Gsk3b	0
Gapt	0	Grm2	0	Prl2c2	0	Gsg2	0
Gapdhs	0	Grm1	0	Micu1	0	Gsg1l	0
Gapdh	0	Grk6	0	Plcd3	0	Gse1	0
Gap43	0	Grk5	0	Brat1	0	Gsdmd	0
Ganc	0	Grk1	0	BRDN0000738355	0	Gsdmc4	0
Ganab	0	Gripap1	0	Nptn	0	Gsdmc3	0
Gan	0	Grip1	0	Me3	0	Gsdmc	0
Galr2	0	Grina	0	Tusc5	0	Gsdma	0
Galr1	0	Grin3b	0	Ndst1	0	Gsap	0
Galp	0	Grin3a	0	Ndst2	0	Grxcr2	0
Galntl5	0	Grin2b	0	Ndst4	0	Grxcr1	0
Galnt9	0	Grin1	0	St6galnac6	0	Grwd1	0
Galnt5	0	Grik3	0	Rax	0	Grrp1	0
Galnt4	0	Grik2	0	Rell1	0	Grpel2	0
Galnt2	0	Grifin	0	Pcsk5	0	Grpel1	0
Galnt16	0	Grid2	0	Cabyr	0	Grn	0
Galnt15	0	Gria3	0	Nkx2-9	0	Grm7	0
Galnt13	0	Gria1	0	Gm10229	0	Grm6	0
Galnt12	0	Grhpr	0	Igfals	0	Grm3	0
Galnt11	0	Grhl3	0	Hcfc1r1	0	Grk5	0
Galnt10	0	Grem1	0	Cd209c	0	Grk4	0
Galns	0	Greb1	0	Nkx2-2	0	Gripap1	0
Galm	0	Grb7	0	Phactr1	0	Grip2	0
Galk2	0	Grb2	0	Nkx2-1	0	Grin3a	0
Gale	0	Grap2	0	Nkx2-6	0	Grin2d	0
Galc	0	Gramd4	0	Nkx2-4	0	Grin2b	0
Gal3st4	0	Gpx8	0	Pi4kb	0	Grik4	0
Gal3st3	0	Gpx7	0	Irgq	0	Grik2	0
Gal3st2	0	Gpx6	0	BRDN0000737879	0	Grifin	0
Gal3st1	0	Gpx4	0	Tusc2	0	Grid1	0
Gal	0	Gpx3	0	Pygo2	0	Gria4	0
Gadl1	0	Gpx1	0	Fbrs	0	Gria3	0
Gadd45gip1	0	Gpsm3	0	Cd209f	0	Grhpr	0

Gadd45a	0
Gad2	0
Gad1	0
Gabrr2	0
Gabrr1	0
Gabrq	0
Gabrp	0
Gabrg3	0
Gabrg2	0
Gabrg1	0
Gabre	0
Gabrd	0
Gabrb3	0
Gabrb2	0
Gabrb1	0
Gabra5	0
Gabra3	0
Gabra1	0
Gabpa	0
Gabbr2	0
Gabbr1	0
Gabarapl2	0
Gabarapl1	0
Gabarap	0
Gab3	0
Gab2	0
Gaa	0
G6pdx	0
G6pd2	0
G6pc3	0
G6pc2	0
G6pc	0
G3bp1	0
G2e3	0
G0s2	0
Fzr1	0
Fzd9	0
Fzd8	0
Fzd7	0
Fzd6	0
Fzd4	0
Fzd3	0
Fzd1	0
Fyttd1	0
Fyco1	0
Fxyd5	0
Fxyd4	0
Fxyd2	0
Fxr2	0
Fxr1	0
Fv1	0
Fuz	0
Fut9	0
Fut8	0
Fut4	0
Fut2	0
Fut11	0
Fut10	0
Fut1	0
Fus	0
Furin	0
Fuom	0
Fundc1	0
Fuca2	0
Fubp3	0
Fubp1	0
Ftsj3	0
Ftsj2	0
Ftsj1	0
Fto	0
Ftl1	0
Fthl17	0
Fth1	0
Fstl5	0
Fstl4	0
Fstl3	0
Fsip1	0
Fshr	0
Fshb	0
Fsd2	0
Fsd1l	0
Fsd1	0

Gpsm2	0
Gps1	0
Gprin2	0
Gprin1	0
Gprc6a	0
Gprc5d	0
Gprc5c	0
Gprc5b	0
Gprc5a	0
Gprasp2	0
Gpr89	0
Gpr88	0
Gpr83	0
Gpr82	0
Gpr75	0
Gpr6	0
Gpr50	0
Gpr45	0
Gpr37l1	0
Gpr35	0
Gpr33	0
Gpr31b	0
Gpr3	0
Gpr27	0
Gpr26	0
Gpr20	0
Gpr19	0
Gpr183	0
Gpr182	0
Gpr18	0
Gpr174	0
Gpr171	0
Gpr17	0
Gpr165	0
Gpr162	0
Gpr158	0
Gpr157	0
Gpr156	0
Gpr155	0
Gpr152	0
Gpr151	0
Gpr150	0
Gpr15	0
Gpr143	0
Gpr142	0
Gpr137c	0
Gpr137b	0
Gpr137	0
Gpr12	0
Gpr119	0
Gpr108	0
Gpr107	0
Gpr101	0
Gpnmb	0
Gpn2	0
Gpn1	0
Gpm6a	0
Gpld1	0
Gpihbp1	0
Gpi1	0
Gphn	0
Gpha2	0
Gper1	0
Gpd2	0
Gpd1l	0
Gpd1	0
Gpcpd1	0
Gpc5	0
Gpc4	0
Gpc3	0
Gpc2	0
Gpc1	0
Gpbp1l1	0
Gpbp1	0
Gpatch8	0
Gpatch4	0
Gpatch3	0
Gpatch11	0
Gpatch1	0
Gpalpp1	0
Gpaa1	0
Gp9	0

Rpl27a	0
BRDN0000737513	0
Psd3	0
Mgat4d	0
Stra13	0
Vps4a	0
Vps4b	0
Psd4	0
Vwa5a	0
BRDN0000737514	0
Veph1	0
Hspg2	0
Irx3	0
St6galnac4	0
Bckdha	0
Gm14374	0
BRDN0000737519	0
Lrrc16b	0
Oser1	0
Sdpr	0
Ofcc1	0
Ctcf	0
B4gat1	0
Ube2q1	0
Mtrf1	0
Ube2q2	0
Ildr2	0
Ildr1	0
Dhrs11	0
Dhrs13	0
Iqcc	0
Fam109b	0
Fam109a	0
Papola	0
Spp1	0
Iqce	0
Supv3l1	0
Iqck	0
Iqcj	0
Iqch	0
Fam175b	0
Gar1	0
Fgfrl1	0
2700049A03Rik	0
Kri1	0
Cyp3a57	0
Cyp3a59	0
Thbs2	0
Cdc14b	0
Cdc14a	0
Thbs1	0
Zfp362	0
Aldh6a1	0
Prl2c5	0
Zfp367	0
Zfp366	0
Dmrtc2	0
Hjurp	0
Zfp369	0
Zeb1	0
Fbxo7	0
Zeb2	0
Pth1r	0
Ndnf	0
Olfr473	0
Serhl	0
Fam189b	0
Usp54	0
Usp53	0
Usp50	0
Nuggc	0
Negr1	0
Clec3b	0
Aif1	0
Rapgef1	0
Ssbp1	0
Pclo	0
Gart	0
Cd180	0
Serpinb11	0
BRDN0000737594	0
Bpnt1	0

Grhl3	0
Grhl2	0
Grem2	0
Grem1	0
Greb1l	0
Grcc10	0
Grb7	0
Grb2	0
Grb10	0
Grasp	0
Gramd4	0
Gramd3	0
Gramd2	0
Gramd1c	0
Gramd1b	0
Gpx8	0
Gpx7	0
Gpx5	0
Gpx4	0
Gpx3	0
Gpx2	0
Gpx1	0
Gpt2	0
Gpsm3	0
Gpsm2	0
Gpsm1	0
Gps2	0
Gps1	0
Gprin3	0
Gprin2	0
Gprin1	0
Gprc5d	0
Gprc5c	0
Gprc5b	0
Gprc5a	0
Gprasp1	0
Gpr89	0
Gpr85	0
Gpr84	0
Gpr82	0
Gpr68	0
Gpr65	0
Gpr62	0
Gpr6	0
Gpr50	0
Gpr37l1	0
Gpr37	0
Gpr35	0
Gpr34	0
Gpr33	0
Gpr31b	0
Gpr3	0
Gpr26	0
Gpr21	0
Gpr183	0
Gpr182	0
Gpr180	0
Gpr18	0
Gpr176	0
Gpr173	0
Gpr171	0
Gpr17	0
Gpr165	0
Gpr162	0
Gpr160	0
Gpr155	0
Gpr151	0
Gpr150	0
Gpr149	0
Gpr143	0
Gpr135	0
Gpr132	0
Gpr108	0
Gpr101	0
Gpnmb	0
Gpn2	0
Gpn1	0
Gpm6b	0
Gpm6a	0
Gpkow	0
Gpi1	0
Gphn	0

Fscn3	0	Gp6	0	Fam163b	0	Gphb5	0
Fscn2	0	Gp5	0	Nup98	0	Gpha2	0
Fscn1	0	Gp49a	0	Mnd1	0	Gper1	0
Fscb	0	Gp1bb	0	BRDN0000737931	0	Gpd2	0
Fsbp	0	Got2	0	Nup93	0	Gpd1l	0
Frzb	0	Gosr2	0	Sctr	0	Gpc6	0
Fryl	0	Gosr1	0	Dll3	0	Gpc5	0
Frs3	0	Gorasp2	0	Dll4	0	Gpc4	0
Frs2	0	Gorab	0	Madcam1	0	Gpc3	0
Frrs1	0	Gopc	0	Pknox1	0	Gpc1	0
Frmpd4	0	Gon4l	0	Atat1	0	Gpbp1	0
Frmpd3	0	Golt1b	0	Olfr220	0	Gpatch8	0
Frmd8	0	Golt1a	0	Olfr221	0	Gpatch3	0
Frmd6	0	Golph3l	0	Olfr222	0	Gpatch2l	0
Frmd3	0	Golm1	0	Arid3b	0	Gpatch2	0
Frk	0	Golim4	0	Olfr224	0	Gpatch11	0
Frg1	0	Golgb1	0	Olfr225	0	Gpatch1	0
Frem3	0	Golga7b	0	Alox8	0	Gpat2	0
Frem1	0	Golga7	0	Olfr228	0	Gpank1	0
Fras1	0	Golga5	0	Olfr229	0	Gpalpp1	0
Fra10ac1	0	Golga4	0	Sult6b1	0	Gpaa1	0
Fpr-rs6	0	Golga2	0	Pcca	0	Gpa33	0
Fpr-rs3	0	Gnrhr	0	Ankrd66	0	Gp9	0
Fpr3	0	Gnrh1	0	Ppp1r3e	0	Gp5	0
Fpgt	0	Gnptab	0	Urad	0	Gp49a	0
Foxs1	0	Gnpnat1	0	4933416C03Rik	0	Gp1bb	0
Foxred2	0	Gnpat	0	Ubac2	0	Gp1ba	0
Foxr2	0	Gnmt	0	Gas2	0	Got2	0
Foxr1	0	Gnl3l	0	Rabif	0	Got1	0
Foxq1	0	Gnl3	0	Ace3	0	Gosr2	0
Foxp4	0	Gnl1	0	Ace2	0	Gosr1	0
Foxp3	0	Gngt2	0	Serpinb13	0	Gorasp2	0
Foxp2	0	Gngt1	0	Fdx1	0	Gorab	0
Foxo6	0	Gng7	0	Gm11596	0	Gopc	0
Foxo3	0	Gng5	0	Rpa2	0	Gon4l	0
Foxo1	0	Gng3	0	Urah	0	Golt1b	0
Foxn4	0	Gng2	0	Tbx15	0	Golt1a	0
Foxn3	0	Gng13	0	Tmed7	0	Golph3l	0
Foxn2	0	Gng11	0	Tmed6	0	Golph3	0
Foxn1	0	Gng10	0	Tmed5	0	Golm1	0
Foxl1	0	Gne	0	Tmed4	0	Golim4	0
Foxk2	0	Gnb5	0	Tmed3	0	Golgb1	0
Foxk1	0	Gnb4	0	Tmed2	0	Golga7b	0
Foxj3	0	Gnb3	0	Rad54b	0	Golga5	0
Foxj1	0	Gnb2l1	0	Rad54l	0	Golga4	0
Foxi3	0	Gnb2	0	Nqo2	0	Golga3	0
Foxi2	0	Gnb1	0	Tmed9	0	Golga2	0
Foxi1	0	Gnat3	0	Nqo1	0	Golga1	0
Foxh1	0	Gnat2	0	9530003J23Rik	0	Gns	0
Foxf2	0	Gnaq	0	Tbx19	0	Gnrhr	0
Foxf1	0	Gnal	0	Mnda	0	Gnrh1	0
Foxd3	0	Gnai3	0	Tifab	0	Gnptg	0
Foxd2	0	Gnai1	0	Cachd1	0	Gnptab	0
Foxd1	0	Gna15	0	Ccdc73	0	Gnpnat1	0
Foxc2	0	Gna14	0	Elmod1	0	Gnpda2	0
Foxc1	0	Gna11	0	Olfr598	0	Gnpda1	0
Foxb2	0	Gmps	0	Olfr599	0	Gnpat	0
Foxa3	0	Gmpr2	0	Ccdc70	0	Gnmt	0
Foxa2	0	Gmppb	0	Kcnab2	0	Gnl3l	0
Fosl1	0	Gmppa	0	Ssrp1	0	Gnl3	0
Fosb	0	Gmnn	0	Stag3	0	Gnl2	0
Fos	0	Gmnc	0	Sp140	0	Gnl1	0
Fopnl	0	Gmfg	0	Gm11937	0	Gngt2	0
Folr2	0	Gmfb	0	Olfr594	0	Gngt1	0
Folr1	0	Gmeb2	0	Fgl2	0	Gng5	0
Folh1	0	Gmds	0	Amdhd1	0	Gng4	0
Focad	0	Gmcl1l	0	Ipp	0	Gng3	0
Fntb	0	Gm9994	0	Osbpl9	0	Gng2	0
Fnta	0	Gm996	0	Sfswap	0	Gng12	0
Fnip2	0	Gm9758	0	Gm10696	0	Gng11	0
Fnip1	0	Gm973	0	Gmnn	0	Gng10	0
Fndc9	0	Gm960	0	Rbx1	0	Gne	0
Fndc8	0	Gm9573	0	Ehhadh	0	Gnb4	0
Fndc7	0	Gm933	0	Uhrf1	0	Gnb3	0
Fndc4	0	Gm9268	0	Uhrf2	0	Gnb2	0
Fndc3c1	0	Gm9125	0	Cfdp1	0	Gnb1	0
Fndc3a	0	Gm9112	0	Tsg101	0	Gnaz	0
Fndc1	0	Gm9047	0	Mki67	0	Gnat2	0
Fnd3c2	0	Gm904	0	Hrasls	0	Gnat1	0
Fnbp4	0	Gm9	0	Piezo2	0	Gnaq	0
Fnbp1l	0	Gm8994	0	Piezo1	0	Gnao1	0
Fnbp1	0	Gm8923	0	Sox10	0	Gnal	0

Fn3krp	0	Gm8882	0	Vps33b	0	Gnai3	0
Fn1	0	Gm884	0	Vps33a	0	Gnai1	0
Fmr1nb	0	Gm8765	0	0610009O20Rik	0	Gna15	0
Fmod	0	Gm8720	0	Pla2g4b	0	Gna14	0
Fmo6	0	Gm8693	0	Olfr803	0	Gna12	0
Fmo5	0	Gm8653	0	Mmp24	0	Gna11	0
Fmo4	0	Gm853	0	Mmp27	0	Gmps	0
Fmo2	0	Gm8300	0	Sgta	0	Gmpr2	0
Fmo1	0	Gm8298	0	Spin1	0	Gmpr	0
Fmnl1	0	Gm8267	0	Mmp20	0	Gmppb	0
Fmn2	0	Gm826	0	Mep1b	0	Gmppa	0
Flywch2	0	Gm815	0	Pald1	0	Gmnn	0
Flywch1	0	Gm813	0	Mpnd	0	Gmnc	0
Flt4	0	Gm7978	0	Gm7157	0	Gml	0
Flt3l	0	Gm7903	0	Prc1	0	Gmip	0
Flrt3	0	Gm7861	0	Nucb1	0	Gmfg	0
Flrt2	0	Gm773	0	Fez1	0	Gmfb	0
Flot1	0	Gm7714	0	Mrpl13	0	Gmeb2	0
Flnc	0	Gm7694	0	Mettl13	0	Gmeb1	0
Flnb	0	Gm766	0	Rps6kc1	0	Gmds	0
Flcn	0	Gm765	0	Pla2g4f	0	Gmcl1l	0
Flad1	0	Gm7534	0	Vmn1r49	0	Gmcl1	0
Fktn	0	Gm7361	0	Vmn1r48	0	Gm9994	0
Fkrp	0	Gm7325	0	Gm3646	0	Gm9992	0
Fkbpl	0	Gm7257	0	Cmtm8	0	Gm996	0
Fkbp9	0	Gm7168	0	Ogfr	0	Gm9758	0
Fkbp6	0	Gm7102	0	Gpatch8	0	Gm9733	0
Fkbp5	0	Gm7073	0	Vmn1r41	0	Gm960	0
Fkbp4	0	Gm694	0	Vmn1r40	0	Gm9573	0
Fkbp3	0	Gm6904	0	Vmn1r43	0	Gm9513	0
Fkbp2	0	Gm6902	0	Vmn1r42	0	Gm94	0
Fkbp1b	0	Gm6890	0	Vmn1r45	0	Gm9376	0
Fkbp1a	0	Gm6880	0	Vmn1r44	0	Gm933	0
Fkbp15	0	Gm684	0	Vmn1r47	0	Gm9268	0
Fkbp14	0	Gm6812	0	Rab37	0	Gm9125	0
Fkbp10	0	Gm6760	0	Trpc4	0	Gm9112	0
Fjx1	0	Gm6696	0	Epb4.2	0	Gm9047	0
Fitm2	0	Gm6592	0	Mri1	0	Gm904	0
Fitm1	0	Gm6588	0	Ugcg	0	Gm8923	0
Fis1	0	Gm6460	0	Hddc2	0	Gm8909	0
Fip1l1	0	Gm6432	0	Hddc3	0	Gm884	0
Filip1	0	Gm6408	0	Chn2	0	Gm8765	0
Fignl2	0	Gm6377	0	Chn1	0	Gm8720	0
Fign	0	Gm6164	0	AY074887	0	Gm8693	0
Figf	0	Gm6121	0	BC003965	0	Gm8660	0
Fig4	0	Gm609	0	BRDN0000737555	0	Gm8653	0
Ficd	0	Gm6034	0	BRDN0000737554	0	Gm853	0
Fibp	0	Gm6026	0	BRDN0000737557	0	Gm8369	0
Fibcd1	0	Gm595	0	BRDN0000737556	0	Gm8300	0
Fhod3	0	Gm5941	0	BRDN0000737551	0	Gm8267	0
Fhod1	0	Gm5938	0	BRDN0000737550	0	Gm826	0
Fhl4	0	Gm5936	0	BRDN0000737553	0	Gm815	0
Fhl2	0	Gm5935	0	BRDN0000737552	0	Gm813	0
Fhl1	0	Gm5934	0	Myo3b	0	Gm7978	0
Fhit	0	Gm5916	0	Mrpl15	0	Gm7861	0
Fhdc1	0	Gm5891	0	Clec7a	0	Gm7849	0
Fhad1	0	Gm5885	0	Grsf1	0	Gm773	0
Fgr	0	Gm5878	0	BRDN0000737440	0	Gm7694	0
Fgl2	0	Gm5868	0	Tmem50a	0	Gm7534	0
Fggy	0	Gm5800	0	Ffar1	0	Gm7325	0
Fgg	0	Gm5796	0	Tjp2	0	Gm7168	0
Fgfrl1	0	Gm5795	0	Olfr157	0	Gm7102	0
Fgfr4	0	Gm5771	0	BRDN0000737662	0	Gm7073	0
Fgfr3	0	Gm5728	0	Scly	0	Gm7030	0
Fgfr2	0	Gm5726	0	Epg5	0	Gm694	0
Fgfr1op	0	Gm572	0	Dmrta2	0	Gm6904	0
Fgfbp3	0	Gm5640	0	Olfr1257	0	Gm6902	0
Fgfbp1	0	Gm5635	0	Nedd4l	0	Gm6880	0
Fgf8	0	Gm5634	0	Impg2	0	Gm684	0
Fgf7	0	Gm5622	0	2310007L24Rik	0	Gm6792	0
Fgf6	0	Gm5617	0	Ppan	0	Gm6760	0
Fgf5	0	Gm561	0	Cyp2c44	0	Gm6588	0
Fgf4	0	Gm5595	0	Ppat	0	Gm6583	0
Fgf3	0	Gm5592	0	Gtdc1	0	Gm6484	0
Fgf22	0	Gm5591	0	Hmgcll1	0	Gm648	0
Fgf20	0	Gm5549	0	Pah	0	Gm6460	0
Fgf18	0	Gm5538	0	Tex101	0	Gm6432	0
Fgf16	0	Gm5483	0	Pam	0	Gm6406	0
Fgf15	0	Gm5460	0	Asb2	0	Gm6164	0
Fgf14	0	Gm5458	0	Olfr1079	0	Gm6086	0
Fgf13	0	Gm5415	0	Asb1	0	Gm6040	0
Fgf12	0	Gm5414	0	Asb6	0	Gm6026	0

Fgf11	0	Gm5382	0	Asb4	0	Gm597	0
Fgf10	0	Gm5346	0	Asb5	0	Gm595	0
Fgf1	0	Gm5294	0	Asb8	0	Gm5938	0
Fgd6	0	Gm527	0	Asb9	0	Gm5936	0
Fgd5	0	Gm5169	0	Metrnl	0	Gm5935	0
Fgd4	0	Gm5168	0	Olfr1076	0	Gm5934	0
Fgd3	0	Gm5150	0	Zfp954	0	Gm5916	0
Fgd2	0	Gm5148	0	Lrrtm4	0	Gm5901	0
Fgd1	0	Gm5142	0	Zfp956	0	Gm5891	0
Fgb	0	Gm5134	0	Zfp957	0	Gm5885	0
Ffar4	0	Gm5132	0	Lrrtm1	0	Gm5868	0
Ffar3	0	Gm5128	0	Zfp951	0	Gm5796	0
Ffar2	0	Gm5127	0	Lrrtm3	0	Gm5795	0
Ffar1	0	Gm5111	0	Sorcs3	0	Gm5726	0
Fezf2	0	Gm5082	0	Olfr109	0	Gm5662	0
Fezf1	0	Gm4981	0	Rpp30	0	Gm5640	0
Fez2	0	Gm4951	0	Gpr152	0	Gm5635	0
Fez1	0	Gm4944	0	Rragd	0	Gm5622	0
Fes	0	Gm4937	0	Tspan13	0	Gm5617	0
Fermt3	0	Gm4922	0	Rraga	0	Gm561	0
Fermt2	0	Gm4907	0	Ddi2	0	Gm5595	0
Fermt1	0	Gm4906	0	Ddi1	0	Gm5591	0
Fer1l5	0	Gm4884	0	Rragb	0	Gm5483	0
Fer1l4	0	Gm4861	0	Ap1m2	0	Gm5460	0
Fer	0	Gm4847	0	Tspan14	0	Gm5458	0
Fen1	0	Gm4846	0	Vmn2r67	0	Gm5416	0
Fem1b	0	Gm4841	0	Ap1m1	0	Gm5415	0
Fech	0	Gm4836	0	Ghitm	0	Gm5346	0
Fdxr	0	Gm4794	0	Foxk1	0	Gm5294	0
Fdxacb1	0	Gm4791	0	BRDN0000737568	0	Gm525	0
Fdx1l	0	Gm4788	0	Ensa	0	Gm5150	0
Fdx1	0	Gm4787	0	Ppa1	0	Gm5148	0
Fdps	0	Gm4724	0	Isca1	0	Gm5142	0
Fcrls	0	Gm4567	0	Gapdh	0	Gm5134	0
Fcrla	0	Gm4559	0	Vpreb1	0	Gm5132	0
Fcrl5	0	Gm4498	0	Vpreb2	0	Gm5128	0
Fcrl1	0	Gm4477	0	Vmn2r53	0	Gm5113	0
Fcna	0	Gm4461	0	Vmn2r51	0	Gm5082	0
Fchsd2	0	Gm44	0	Vmn2r57	0	Gm4980	0
Fcho2	0	Gm438	0	Vmn2r56	0	Gm4951	0
Fcho1	0	Gm436	0	Vmn2r55	0	Gm4937	0
Fcgrt	0	Gm4340	0	Vmn2r54	0	Gm4922	0
Fcgr3	0	Gm4312	0	1110007C09Rik	0	Gm4907	0
Fcgr2b	0	Gm4305	0	Vmn2r59	0	Gm4906	0
Fcgr1	0	Gm4302	0	Olfr1030	0	Gm4894	0
Fcgbp	0	Gm4301	0	Gm4937	0	Gm4884	0
Fcf1	0	Gm428	0	Ripk1	0	Gm4871	0
Fcer2a	0	Gm4201	0	Dnaic2	0	Gm4861	0
Fcer1a	0	Gm4187	0	Ripk3	0	Gm4847	0
Fcamr	0	Gm4177	0	Ripk4	0	Gm4846	0
Fbxw9	0	Gm4175	0	Gucy1b2	0	Gm4841	0
Fbxw8	0	Gm4141	0	Qdpr	0	Gm4794	0
Fbxw7	0	Gm4133	0	BRDN0000738339	0	Gm4791	0
Fbxw5	0	Gm4064	0	Plet1	0	Gm4788	0
Fbxw4	0	Gm382	0	Sdc3	0	Gm4787	0
Fbxw28	0	Gm3776	0	Zfp251	0	Gm4763	0
Fbxw26	0	Gm3763	0	Prkag1	0	Gm4745	0
Fbxw22	0	Gm3750	0	Prkag3	0	Gm4724	0
Fbxw21	0	Gm3701	0	Prkag2	0	Gm4559	0
Fbxw20	0	Gm3696	0	Apmap	0	Gm4498	0
Fbxw2	0	Gm3646	0	Apbb2	0	Gm4477	0
Fbxw17	0	Gm364	0	Apbb3	0	Gm4461	0
Fbxw16	0	Gm362	0	Wnt8a	0	Gm44	0
Fbxw15	0	Gm3558	0	Wnt8b	0	Gm438	0
Fbxw13	0	Gm3500	0	Cfap36	0	Gm436	0
Fbxw11	0	Gm3488	0	Gpr20	0	Gm4340	0
Fbxw10	0	Gm3417	0	Gpr21	0	Gm4307	0
Fbxo9	0	Gm3415	0	Gpr26	0	Gm4305	0
Fbxo8	0	Gm3404	0	Gpr27	0	Gm4303	0
Fbxo7	0	Gm3336	0	D6Ertd527e	0	Gm4302	0
Fbxo5	0	Gm3286	0	Nav1	0	Gm4297	0
Fbxo48	0	Gm3259	0	Rsph1	0	Gm428	0
Fbxo47	0	Gm3139	0	Olfr1351	0	Gm4214	0
Fbxo46	0	Gm2933	0	Cabin1	0	Gm4201	0
Fbxo44	0	Gm2927	0	Cyp17a1	0	Gm4187	0
Fbxo43	0	Gm2897	0	H2-Q4	0	Gm4177	0
Fbxo42	0	Gm2863	0	Maf1	0	Gm4141	0
Fbxo41	0	Gm2825	0	Rhoq	0	Gm4133	0
Fbxo40	0	Gm2799	0	Rtn4ip1	0	Gm4070	0
Fbxo4	0	Gm2696	0	Ostc	0	Gm4064	0
Fbxo39	0	Gm2663	0	Rhou	0	Gm382	0
Fbxo38	0	Gm266	0	Mok	0	Gm3776	0

Fbxo36	0
Fbxo33	0
Fbxo32	0
Fbxo31	0
Fbxo3	0
Fbxo27	0
Fbxo25	0
Fbxo22	0
Fbxo21	0
Fbxo2	0
Fbxo18	0
Fbxo16	0
Fbxo15	0
Fbxo11	0
Fbxo10	0
Fbxl8	0
Fbxl22	0
Fbxl21	0
Fbxl20	0
Fbxl2	0
Fbxl19	0
Fbxl18	0
Fbxl17	0
Fbxl16	0
Fbxl15	0
Fbxl14	0
Fbxl12	0
Fbrsl1	0
Fbrs	0
Fbp2	0
Fbn2	0
Fbn1	0
Fbln7	0
Fbln5	0
Fbll1	0
Fblim1	0
Fbl	0
Faxc	0
Fau	0
Fat4	0
Fat3	0
Fat2	0
Fat1	0
Fastkd5	0
Fastkd3	0
Fastkd2	0
Fastkd1	0
Fastk	0
Fasn	0
Fasl	0
Fas	0
Farsb	0
Farsa	0
Fars2	0
Farp2	0
Far2	0
Fap	0
Fank1	0
Fancl	0
Fanci	0
Fancg	0
Fancd2os	0
Fancd2	0
Fancc	0
Fancb	0
Fanca	0
Fan1	0
Fam98c	0
Fam98b	0
Fam98a	0
Fam96b	0
Fam96a	0
Fam89a	0
Fam84b	0
Fam84a	0
Fam83h	0
Fam83g	0
Fam83f	0
Fam83e	0
Fam83d	0
Fam83c	0
Fam83a	0
Gm21975	0
Gm21951	0
Gm21950	0
Gm21637	0
Gm21541	0
Gm21379	0
Gm21319	0
Gm21119	0
Gm20917	0
Gm20877	0
Gm20854	0
Gm20852	0
Gm20831	0
Gm20816	0
Gm20815	0
Gm20747	0
Gm20738	0
Gm20736	0
Gm20604	0
Gm20594	0
Gm2030	0
Gm2016	0
Gm1993	0
Gm1979	0
Gm19668	0
Gm19402	0
Gm19345	0
Gm17727	0
Gm17677	0
Gm17455	0
Gm17365	0
Gm17359	0
Gm1661	0
Gm166	0
Gm16513	0
Gm16501	0
Gm16451	0
Gm16432	0
Gm16381	0
Gm1604b	0
Gm1587	0
Gm15800	0
Gm1564	0
Gm1553	0
Gm15386	0
Gm15319	0
Gm15308	0
Gm15299	0
Gm15127	0
Gm15114	0
Gm15107	0
Gm15097	0
Gm15080	0
Gm15056	0
Gm15023	0
Gm14920	0
Gm14851	0
Gm14850	0
Gm14744	0
Gm14632	0
Gm14496	0
Gm14479	0
Gm14474	0
Gm14459	0
Gm14446	0
Gm14440	0
Gm14434	0
Gm14431	0
Gm14393	0
Gm14347	0
Gm14325	0
Gm14322	0
Gm14308	0
Gm14124	0
Gm14085	0
Gm136	0
Gm13547	0
Gm13306	0
Gm13288	0
Gm13287	0
Gm13283	0
Gm13278	0
Dnajc17	0
Npy2r	0
Dnajc12	0
Dnajc13	0
Dnajc10	0
Rhoc	0
Rhob	0
Rhoa	0
Adora3	0
Dnajc18	0
Gnai1	0
Dlg1	0
Tnfaip6	0
Slc22a12	0
Efemp2	0
Ankrd6	0
G6pc3	0
Nelfe	0
Arhgap6	0
Myoz3	0
Fam102a	0
Tnfaip3	0
Acer2	0
Tmem59	0
Tmem56	0
Tmem57	0
Tmem54	0
Tmem52	0
Tmem53	0
Ctnna2	0
Ankrd1	0
Papss1	0
Papss2	0
Ankrd13c	0
Bves	0
Ctxn2	0
Pitpnm3	0
Mafk	0
Cidec	0
Scml2	0
Mafb	0
Gm19668	0
Scml4	0
Tspyl4	0
Dbndd1	0
Gm166	0
Ces2c	0
Actn4	0
Parp14	0
Parp12	0
Parp10	0
Omt2b	0
Nle1	0
Ccdc15	0
Siae	0
Ola1	0
BRDN0000738135	0
Ccdc12	0
Ccdc13	0
Upb1	0
Hdac10	0
Kdm3a	0
Nfrkb	0
Mrc1	0
Mrc2	0
Lrrc27	0
Hrsp12	0
Cep152	0
Emc1	0
Ankrd34a	0
Ankrd34c	0
Ankrd34b	0
Kdelc1	0
Anpep	0
Vmn1r168	0
Gm5416	0
Gm5414	0
Olfr1356	0
Gata2	0
Gata3	0
Ethe1	0
Gata1	0
Gm3763	0
Gm3750	0
Gm3696	0
Gm3646	0
Gm364	0
Gm3558	0
Gm3488	0
Gm3415	0
Gm3404	0
Gm3336	0
Gm3286	0
Gm3238	0
Gm3139	0
Gm2a	0
Gm2927	0
Gm2897	0
Gm2825	0
Gm2696	0
Gm2663	0
Gm266	0
Gm21975	0
Gm21950	0
Gm21943	0
Gm21693	0
Gm21671	0
Gm21541	0
Gm21319	0
Gm21119	0
Gm20917	0
Gm20877	0
Gm20865	0
Gm20831	0
Gm20822	0
Gm20816	0
Gm20815	0
Gm20767	0
Gm20747	0
Gm20738	0
Gm20736	0
Gm20604	0
Gm20594	0
Gm2030	0
Gm2012	0
Gm1993	0
Gm19668	0
Gm19402	0
Gm19345	0
Gm17689	0
Gm17677	0
Gm17660	0
Gm17455	0
Gm17365	0
Gm17359	0
Gm17296	0
Gm17252	0
Gm17019	0
Gm1673	0
Gm1661	0
Gm166	0
Gm16532	0
Gm16513	0
Gm16432	0
Gm15800	0
Gm156	0
Gm15319	0
Gm15315	0
Gm15308	0
Gm15299	0
Gm15292	0
Gm15284	0
Gm1527	0
Gm15140	0
Gm15127	0
Gm15114	0
Gm15107	0
Gm15097	0
Gm15093	0
Gm15091	0
Gm15085	0
Gm15023	0
Gm14920	0
Gm14851	0

Fam81a	0	Gm13277	0	Ccdc173	0	Gm14850	0
Fam78b	0	Gm13276	0	Tmod4	0	Gm14781	0
Fam78a	0	Gm13275	0	Hbq1a	0	Gm14744	0
Fam76b	0	Gm13272	0	Olah	0	Gm14743	0
Fam76a	0	Gm13251	0	Gm382	0	Gm14632	0
Fam71f2	0	Gm13247	0	Tcf7l1	0	Gm14548	0
Fam71f1	0	Gm13242	0	Tcf7l2	0	Gm14511	0
Fam71e2	0	Gm13212	0	Esp6Esp5	0	Gm14501	0
Fam71e1	0	Gm13177	0	Tmem263	0	Gm14479	0
Fam71d	0	Gm13157	0	Kcns2	0	Gm14474	0
Fam71b	0	Gm13154	0	Kcns3	0	Gm14459	0
Fam71a	0	Gm13139	0	Socs4	0	Gm14458	0
Fam69c	0	Gm13119	0	Ccdc177	0	Gm14446	0
Fam69a	0	Gm13088	0	Lipf	0	Gm14434	0
Fam65c	0	Gm13084	0	Thbd	0	Gm14420	0
Fam65a	0	Gm13083	0	Axl	0	Gm14351	0
Fam63b	0	Gm13057	0	Vmn1r204	0	Gm14347	0
Fam63a	0	Gm13051	0	Pmpca	0	Gm14346	0
Fam60a	0	Gm13043	0	BRDN0000737969	0	Gm14345	0
Fam58b	0	Gm13023	0	Olfr273	0	Gm14325	0
Fam57a	0	Gm12942	0	Tmod2	0	Gm14308	0
Fam53a	0	Gm12888	0	Kcnma1	0	Gm14137	0
Fam50b	0	Gm12887	0	A1cf	0	Gm14124	0
Fam50a	0	Gm12886	0	Tmprss11a	0	Gm14085	0
Fam49b	0	Gm12789	0	Akr1a1	0	Gm13889	0
Fam49a	0	Gm12695	0	D1Ertd622e	0	Gm13871	0
Fam47e	0	Gm12253	0	Sdhaf1	0	Gm13769	0
Fam47c	0	Gm11938	0	Sst	0	Gm13547	0
Fam46d	0	Gm11937	0	Sla2	0	Gm13306	0
Fam43b	0	Gm11758	0	1500015O10Rik	0	Gm13298	0
Fam43a	0	Gm11757	0	Kifc2	0	Gm13290	0
Fam3c	0	Gm11744	0	Kifc3	0	Gm13288	0
Fam3b	0	Gm11711	0	Kifc1	0	Gm13287	0
Fam35a	0	Gm11596	0	Ap1g2	0	Gm13279	0
Fam32a	0	Gm11595	0	Vmn1r151	0	Gm13278	0
Fam26f	0	Gm11569	0	BRDN0000737702	0	Gm13277	0
Fam26e	0	Gm11568	0	BRDN0000737703	0	Gm13276	0
Fam24a	0	Gm11567	0	BRDN0000737704	0	Gm13275	0
Fam229a	0	Gm11565	0	BRDN0000737705	0	Gm13272	0
Fam228b	0	Gm11564	0	BRDN0000737706	0	Gm13251	0
Fam228a	0	Gm11563	0	Vmn1r157	0	Gm13247	0
Fam227b	0	Gm11562	0	Vmn1r158	0	Gm13242	0
Fam227a	0	Gm11559	0	Vmn1r159	0	Gm13178	0
Fam222a	0	Gm11541	0	Olfr1170	0	Gm13177	0
Fam221b	0	Gm11487	0	Tmem190	0	Gm13157	0
Fam219b	0	Gm11237	0	Tmem192	0	Gm13154	0
Fam217b	0	Gm1123	0	1810009A15Rik	0	Gm13152	0
Fam217a	0	Gm11127	0	Tmem194	0	Gm13139	0
Fam216a	0	Gm1110	0	Tmem196	0	Gm13125	0
Fam214b	0	Gm10922	0	Tmem198	0	Gm13124	0
Fam213b	0	Gm10921	0	BRDN0000737563	0	Gm13119	0
Fam212b	0	Gm10767	0	Tra2a	0	Gm13103	0
Fam210b	0	Gm10697	0	Prrt1	0	Gm13088	0
Fam210a	0	Gm10696	0	Mfsd9	0	Gm13084	0
Fam21	0	Gm10670	0	Mfsd8	0	Gm13078	0
Fam20c	0	Gm10662	0	Rasgrp1	0	Gm13057	0
Fam20b	0	Gm10471	0	Ap5b1	0	Gm13043	0
Fam208b	0	Gm1045	0	Mfsd1	0	Gm13040	0
Fam208a	0	Gm10439	0	Tldc1	0	Gm13023	0
Fam207a	0	Gm10413	0	Tldc2	0	Gm12942	0
Fam206a	0	Gm10408	0	Fastkd1	0	Gm12888	0
Fam19a5	0	Gm10406	0	Mfsd5	0	Gm12887	0
Fam19a4	0	Gm10377	0	Mfsd4	0	Gm12886	0
Fam19a3	0	Gm10375	0	Cdrt4	0	Gm128	0
Fam19a2	0	Gm10354	0	Rtp4	0	Gm12789	0
Fam199x	0	Gm10334	0	3110040N11Rik	0	Gm12695	0
Fam198b	0	Gm10324	0	Tjp1	0	Gm12429	0
Fam196b	0	Gm10267	0	Phkg1	0	Gm12185	0
Fam196a	0	Gm10256	0	Tceal8	0	Gm11992	0
Fam195b	0	Gm10229	0	Nt5dc1	0	Gm11938	0
Fam195a	0	Gm10228	0	BRDN0000737564	0	Gm11937	0
Fam189b	0	Gm10220	0	Gle1	0	Gm11757	0
Fam189a2	0	Gm10142	0	BRDN0000737775	0	Gm11710	0
Fam189a1	0	Gm10094	0	Trcg1	0	Gm11595	0
Fam188b	0	Gm10081	0	Stk32b	0	Gm11569	0
Fam188a	0	Gm10057	0	Stk32a	0	Gm11568	0
Fam186b	0	Glyr1	0	Cdx2	0	Gm11564	0
Fam184a	0	Glyctk	0	Pi4ka	0	Gm11563	0
Fam183b	0	Glycam1	0	Tbx21	0	Gm11562	0
Fam179b	0	Glyat	0	Denr	0	Gm11559	0
Fam179a	0	Glul	0	Tbx22	0	Gm11554	0
Fam178a	0	Gltscr2	0	Lrrc14b	0	Gm11541	0

Fam177a	0	Gltscr1	0	Ncs1	0	Gm11487	0
Fam175b	0	Gltpd2	0	Tff2	0	Gm11437	0
Fam175a	0	Gltp	0	Cdx4	0	Gm11237	0
Fam174a	0	Glt8d1	0	Gnmt	0	Gm11128	0
Fam173b	0	Glt28d2	0	Ahsa2	0	Gm11127	0
Fam172a	0	Glt1d1	0	Slit3	0	Gm10922	0
Fam171b	0	Gls2	0	Slit2	0	Gm10921	0
Fam171a2	0	Gls	0	Slit1	0	Gm10778	0
Fam171a1	0	Glrx3	0	Glt6d1	0	Gm10767	0
Fam170b	0	Glrx2	0	Sap30	0	Gm10696	0
Fam170a	0	Glrx	0	Olfr498	0	Gm10670	0
Fam169a	0	Glrp1	0	BRDN0000737481	0	Gm10665	0
Fam167b	0	Glrb	0	Plbd2	0	Gm10662	0
Fam166b	0	Glra3	0	Lamb2	0	Gm10639	0
Fam166a	0	Glp2r	0	BRDN0000738090	0	Gm10471	0
Fam163b	0	Glp1r	0	G6pc2	0	Gm1045	0
Fam162b	0	Glod5	0	Mup19	0	Gm10436	0
Fam162a	0	Glod4	0	Akip1	0	Gm10413	0
Fam161b	0	Glo1	0	Spta1	0	Gm10408	0
Fam161a	0	Glmp	0	BRDN0000738092	0	Gm10406	0
Fam160b1	0	Glis2	0	BRDN0000738079	0	Gm10377	0
Fam160a2	0	Glis1	0	BRDN0000738078	0	Gm10375	0
Fam159b	0	Glipr2	0	BRDN0000738077	0	Gm10354	0
Fam159a	0	Glipr1l1	0	BRDN0000738076	0	Gm10334	0
Fam155a	0	Glipr1	0	BRDN0000738075	0	Gm10324	0
Fam151b	0	Glg1	0	BRDN0000738074	0	Gm10267	0
Fam151a	0	Gle1	0	BRDN0000738073	0	Gm10256	0
Fam150b	0	Glce	0	BRDN0000738072	0	Gm10228	0
Fam150a	0	Glcci1	0	Coro6	0	Gm10147	0
Fam149b	0	Glb1l	0	Coro7	0	Gm10142	0
Fam149a	0	Gla	0	Tcap	0	Gm10104	0
Fam13c	0	Gkn3	0	4930562C15Rik	0	Gm10094	0
Fam13b	0	Gkn2	0	Thap2	0	Gm10057	0
Fam13a	0	Gkn1	0	Olfr1449	0	Glyr1	0
Fam136a	0	Gkap1	0	Thap4	0	Glyctk	0
Fam135b	0	Gk5	0	Casp3	0	Glyatl3	0
Fam135a	0	Gje1	0	Triobp	0	Glul	0
Fam134c	0	Gjd3	0	Sox6	0	Gltscr2	0
Fam134a	0	Gjd2	0	Ccm2l	0	Gltscr1	0
Fam133b	0	Gjc3	0	Olfr1443	0	Gltpd2	0
Fam132b	0	Gjc2	0	Olfr1440	0	Gltp	0
Fam132a	0	Gjb6	0	Olfr1441	0	Glt8d2	0
Fam131c	0	Gjb5	0	Olfr1446	0	Glt6d1	0
Fam131b	0	Gjb3	0	Olfr1447	0	Glt1d1	0
Fam131a	0	Gjb2	0	Olfr1444	0	Glrx5	0
Fam129c	0	Gjb1	0	Wrnip1	0	Glrx3	0
Fam129b	0	Gja8	0	Ube2dnl2	0	Glrx2	0
Fam129a	0	Gja10	0	BRDN0000738099	0	Glrx	0
Fam126b	0	Gja1	0	Tgfbr2	0	Glra3	0
Fam126a	0	Git1	0	Ube2dnl1	0	Glra2	0
Fam124b	0	Gipr	0	BRDN0000737567	0	Glra1	0
Fam124a	0	Gipc2	0	Esam	0	Glp2r	0
Fam122c	0	Gip	0	Gm6164	0	Glp1r	0
Fam122b	0	Gins4	0	Muc5ac	0	Glod5	0
Fam120c	0	Gins3	0	Cdnf	0	Glod4	0
Fam120b	0	Gins2	0	Scgb1a1	0	Glo1	0
Fam120a	0	Gimap8	0	Dchs1	0	Glmp	0
Fam118b	0	Gimap6	0	Map1a	0	Glmn	0
Fam118a	0	Gimap4	0	Map1b	0	Glis2	0
Fam117a	0	Gigyf2	0	2700060E02Rik	0	Glipr2	0
Fam114a2	0	Ghsr	0	Yy1	0	Glipr1l2	0
Fam114a1	0	Ghrh	0	Atox1	0	Glipr1l1	0
Fam111a	0	Ghr	0	Yy2	0	Gli2	0
Fam110c	0	Ghitm	0	Aimp2	0	Glg1	0
Fam110b	0	Ghdc	0	Nfib	0	Gldn	0
Fam109a	0	Gh	0	BRDN0000738259	0	Glce	0
Fam107a	0	Ggta1	0	Map1s	0	Glb1	0
Fam105a	0	Ggt7	0	Olfr788	0	Gkn3	0
Fam104a	0	Ggt6	0	Olfr787	0	Gkn2	0
Fam103a1	0	Ggt5	0	Olfr786	0	Gkn1	0
Fam102b	0	Ggps1	0	Elp5	0	Gkap1	0
Fam102a	0	Ggnbp2	0	Elp4	0	Gk2	0
Fam101b	0	Ggnbp1	0	Elp3	0	Gjd3	0
Fam101a	0	Ggn	0	Olfr782	0	Gjd2	0
Faim3	0	Ggcx	0	Olfr781	0	Gjc1	0
Faim2	0	Ggact	0	Olfr780	0	Gjb6	0
Fahd2a	0	Gga3	0	Akap4	0	Gjb4	0
Faf2	0	Gga2	0	Acvr2a	0	Gjb3	0
Faf1	0	Gga1	0	Akap6	0	Gjb2	0
Fads6	0	Gfy	0	Akap7	0	Gjb1	0
Fads3	0	Gfra4	0	Nsmce4a	0	Gja6	0
Fads2	0	Gfra2	0	Glrp1	0	Gja5	0

Fadd	0	Gfpt2	0	Trp53	0	Gja4	0
Fabp7	0	Gfpt1	0	Prlr	0	Gja3	0
Fabp6	0	Gfod2	0	Sephs2	0	Gja10	0
Fabp5	0	Gfod1	0	Sephs1	0	Gja1	0
Fabp4	0	Gfm1	0	Acox2	0	Gipr	0
Fabp2	0	Gfer	0	Akap9	0	Gipc2	0
Fabp1	0	Gfap	0	Zbtb39	0	Gipc1	0
Faah	0	Gen1	0	Trip10	0	Gip	0
Fa2h	0	Gemin8	0	Sec23ip	0	Gins4	0
F8a	0	Gemin7	0	Trip11	0	Gins3	0
F830045P16Rik	0	Gemin5	0	Phlpp2	0	Gins2	0
F830016B08Rik	0	Gemin4	0	Tmem136	0	Gins1	0
F8	0	Gemin2	0	Zbtb33	0	Ginm1	0
F7	0	Gem	0	Phlpp1	0	Gin1	0
F2rl3	0	Gdpgp1	0	Timm44	0	Gimap7	0
F2rl2	0	Gdpd5	0	Zbtb37	0	Gimap5	0
F2rl1	0	Gdpd4	0	Tns3	0	Gimap1	0
F2r	0	Gdpd3	0	Prkx	0	Gigyf1	0
F13b	0	Gdpd1	0	BRDN0000738094	0	Gif	0
F11r	0	Gdnf	0	1600014K23Rik	0	Gid8	0
F10	0	Gdi1	0	Cep295	0	Gid4	0
Ezr	0	Gdf9	0	Tmem135	0	Ghsr	0
Ezh2	0	Gdf3	0	Clmn	0	Ghrhr	0
Eya4	0	Gdf2	0	Cep290	0	Ghrh	0
Eya2	0	Gdf10	0	Map10	0	Ghitm	0
Extl3	0	Gdap1l1	0	Akap8l	0	Ghdc	0
Extl2	0	Gdap1	0	Clmp	0	Gh	0
Ext2	0	Gda	0	Exosc9	0	Ggta1	0
Ext1	0	Gcsam	0	Nxnl2	0	Ggt7	0
Exph5	0	Gcnt7	0	Gm14458	0	Ggps1	0
Exosc9	0	Gcnt4	0	Ndufa2	0	Ggnbp1	0
Exosc8	0	Gcnt3	0	Olfr450	0	Ggn	0
Exosc7	0	Gcnt2	0	Stxbp1	0	Ggh	0
Exosc5	0	Gcnt1	0	Gm14325	0	Ggcx	0
Exosc3	0	Gcn1l1	0	Ramp1	0	Ggct	0
Exosc2	0	Gcm2	0	Pafah1b1	0	Ggact	0
Exosc10	0	Gcm1	0	Gm14322	0	Gga3	0
Exosc1	0	Gclm	0	Pafah1b2	0	Gga2	0
Exog	0	Gclc	0	Ccdc101	0	Gfy	0
Exoc8	0	Gchfr	0	Npr2	0	Gfra3	0
Exoc6b	0	Gcgr	0	Npr3	0	Gfpt2	0
Exoc6	0	Gcdh	0	Ccdc105	0	Gfpt1	0
Exoc5	0	Gcc2	0	Dner	0	Gfod2	0
Exoc4	0	Gcc1	0	Ccdc107	0	Gfod1	0
Exoc3l4	0	Gca	0	Ccdc106	0	Gfm2	0
Exoc2	0	Gc	0	Ccdc108	0	Gfi1b	0
Exo5	0	Gbx2	0	D930015E06Rik	0	Gfer	0
Exo1	0	Gbx1	0	1110059G10Rik	0	Gfap	0
Exd2	0	Gbp8	0	BRDN0000738218	0	Get4	0
Exd1	0	Gbp7	0	Ubd	0	Gen1	0
Ewsr1	0	Gbp6	0	Klhdc3	0	Gemin8	0
Evx2	0	Gbp5	0	Mup14	0	Gemin7	0
Evx1	0	Gbp3	0	Mup16	0	Gemin6	0
Evpl	0	Gbp2	0	Mup10	0	Gemin5	0
Evi5	0	Gbp11	0	Ppp1r14c	0	Gemin4	0
Evi2b	0	Gbgt1	0	Chst13	0	Gemin2	0
Evi2a	0	Gbe1	0	Ndufaf5	0	Gem	0
Evc2	0	Gba2	0	Exosc1	0	Gdpd5	0
Evc	0	Gba	0	Tm7sf3	0	Gdpd3	0
Eva1c	0	Gatm	0	Tm7sf2	0	Gdpd1	0
Eva1b	0	Gatc	0	Vps25	0	Gdnf	0
Eva1a	0	Gatb	0	Wdr93	0	Gdi2	0
Etv5	0	Gatad2a	0	Wdr92	0	Gdi1	0
Etv4	0	Gatad1	0	Wdr91	0	Gdf7	0
Etv3	0	Gata4	0	Nkapl	0	Gdf6	0
Etv2	0	Gata3	0	Mocs1	0	Gdf5	0
Etv1	0	Gata2	0	Exosc7	0	Gdf3	0
Ets2	0	Gata1	0	Pde1c	0	Gdf11	0
Ets1	0	Gast	0	Gucd1	0	Gdf10	0
Etohi1	0	Gas8	0	Ndufaf1	0	Gde1	0
Etnppl	0	Gas2l1	0	Exosc5	0	Gdap2	0
Etnk2	0	Gas2	0	Klrg1	0	Gda	0
Etnk1	0	Gart	0	Pde1a	0	Gcsh	0
Etl4	0	Gars	0	Phyhip	0	Gcsam	0
Ethe1	0	Gareml	0	Zscan5b	0	Gcnt4	0
Etfdh	0	Garem	0	Fam98c	0	Gcnt3	0
Etfb	0	Gar1	0	Wdr45b	0	Gcnt2	0
Etfa	0	Gapvd1	0	Trmt10c	0	Gcnt1	0
Etf1	0	Gapt	0	Fam98b	0	Gcn1l1	0
Esyt3	0	Gapdhs	0	Zp1	0	Gcm2	0
Esyt2	0	Gapdh	0	Mup5	0	Gcm1	0
Esx1	0	Gap43	0	Zp3	0	Gclm	0

Esrrg	0	Ganab	0	Zp2	0	Gclc	0
Esrra	0	Gan	0	Ifih1	0	Gckr	0
Esrp2	0	Gamt	0	Olfr455	0	Gck	0
Esr2	0	Galt	0	Gm5150	0	Gch1	0
Esr1	0	Galr2	0	Actr8	0	Gcgr	0
Espnl	0	Galr1	0	Emid1	0	Gcfc2	0
Espn	0	Galp	0	Xndc1	0	Gcdh	0
Espl1	0	Galnt9	0	Epcam	0	Gcc1	0
Esp8	0	Galnt7	0	Sema3d	0	Gcat	0
Esp6Esp5	0	Galnt6	0	Dtx3l	0	Gca	0
Esp6	0	Galnt5	0	Ripply1	0	Gc	0
Esp4	0	Galnt4	0	Igdcc3	0	Gbx2	0
Esp38	0	Galnt16	0	Zfp563	0	Gbp9	0
Esp36	0	Galnt15	0	Spg21	0	Gbp8	0
Esp31	0	Galnt14	0	Spg20	0	Gbp7	0
Esp3	0	Galnt13	0	6330409D20Rik	0	Gbp6	0
Esp24	0	Galnt12	0	Igdcc4	0	Gbp5	0
Esp18	0	Galnt1	0	Cxcr4	0	Gbp3	0
Esp16	0	Galns	0	Rtkn	0	Gbp2b	0
Esp15	0	Galm	0	Meox1	0	Gbp2	0
Esp1	0	Galc	0	2010001E11Rik	0	Gbp10	0
Esm1	0	Gal3st4	0	Mgat1	0	Gbgt1	0
Esf1	0	Gal3st3	0	Mgat2	0	Gbf1	0
Esco2	0	Gal3st2	0	Mgat3	0	Gba2	0
Esam	0	Gal	0	Mgat5	0	Gba	0
Erv3	0	Gak	0	Trem3	0	Gatsl2	0
Errfi1	0	Gadl1	0	A4galt	0	Gatc	0
Erp44	0	Gadd45gip1	0	1190003K10Rik	0	Gata6	0
Erp29	0	Gadd45g	0	Nt5c3	0	Gata5	0
Ero1lb	0	Gadd45b	0	Tor1aip2	0	Gata4	0
Ero1l	0	Gadd45a	0	Atrn	0	Gata3	0
Ern1	0	Gad1	0	Krtap1-3	0	Gast	0
Ermp1	0	Gabrr3	0	Krtap1-4	0	Gas8	0
Ermard	0	Gabrr2	0	Krtap1-5	0	Gas7	0
Erlin2	0	Gabrr1	0	Pkd1l2	0	Gas6	0
Erlec1	0	Gabrq	0	BRDN0000737811	0	Gas2l3	0
Erich6	0	Gabrp	0	Robo1	0	Gas2	0
Erich5	0	Gabrg3	0	Robo3	0	Gas1	0
Erich4	0	Gabrg2	0	Robo4	0	Gart	0
Erich3	0	Gabrd	0	Zfp568	0	Gars	0
Erich2	0	Gabrb2	0	Atoh7	0	Garem	0
Erich1	0	Gabrb1	0	Runx3	0	Gar1	0
Eri3	0	Gabra5	0	Gm5082	0	Gapdh	0
Eri1	0	Gabra4	0	BRDN0000737889	0	Ganc	0
Erh	0	Gabra2	0	Aldh1l2	0	Ganab	0
Ergic3	0	Gabpb1	0	Nxph3	0	Gan	0
Ergic2	0	Gabarapl2	0	Nxph2	0	Gamt	0
Ergic1	0	Gabarapl1	0	Cdc123	0	Galt	0
Erg	0	Gabarap	0	Gm13242	0	Galr2	0
Ereg	0	Gab3	0	Txndc5	0	Galnt9	0
Ercc8	0	G6pd2	0	Ufd1l	0	Galnt6	0
Ercc6l2	0	G6pc3	0	Gm13247	0	Galnt5	0
Ercc6l	0	G6pc2	0	BRDN0000738238	0	Galnt3	0
Ercc6	0	G630090E17Rik	0	BRDN0000737951	0	Galnt2	0
Ercc5	0	Fzr1	0	Rd3l	0	Galnt16	0
Ercc4	0	Fzd8	0	4930435E12Rik	0	Galnt15	0
Ercc3	0	Fzd7	0	Scyl2	0	Galnt14	0
Ercc2	0	Fzd5	0	Scyl3	0	Galnt13	0
Ercc1	0	Fzd4	0	Actr5	0	Galnt11	0
Erc2	0	Fzd2	0	Cbfa2t2	0	Galns	0
Erbb4	0	Fyttd1	0	Tns4	0	Galk1	0
Erbb2ip	0	Fyn	0	BRDN0000737952	0	Galc	0
Erbb2	0	Fyco1	0	Mcat	0	Gal3st4	0
Erap1	0	Fxyd7	0	Ankef1	0	Gal3st3	0
Eral1	0	Fxyd4	0	Pdcd1lg2	0	Gal3st2	0
Eqtn	0	Fxr2	0	Gbf1	0	Gal3st1	0
Epyc	0	Fxn	0	Gfm1	0	Gal	0
Epx	0	Fv1	0	Fsip1	0	Gak	0
Ept1	0	Fuz	0	Dhrs7b	0	Gadd45g	0
Epsti1	0	Fut2	0	Dhrs7c	0	Gadd45b	0
Eps8l3	0	Fut11	0	BRDN0000737915	0	Gadd45a	0
Eps8	0	Fut10	0	Slc40a1	0	Gad1	0
Eps15l1	0	Fus	0	Sqstm1	0	Gabrr2	0
Eps15	0	Fuom	0	BRDN0000738231	0	Gabrr1	0
Eprs	0	Fundc2	0	Kcnu1	0	Gabrq	0
Eppin	0	Fuk	0	Ckap2l	0	Gabrp	0
Epor	0	Fuca2	0	Ly6g5c	0	Gabrg2	0
Epo	0	Fuca1	0	Ly6c1	0	Gabrg1	0
Epn3	0	Fubp3	0	BRDN0000737959	0	Gabrd	0
Epn2	0	Fubp1	0	Fut10	0	Gabrb3	0
Epn1	0	Ftsj3	0	BRDN0000738233	0	Gabrb2	0
Epm2aip1	0	Ftsj2	0	Pdcd6	0	Gabra6	0

Epm2a	0	Ftsj1	0	Fam89a	0	Gabra5	0
Ephx4	0	Fto	0	Zfp384	0	Gabra4	0
Ephx3	0	Ftmt	0	Foxb2	0	Gabra3	0
Ephb4	0	Ftcd	0	Zfp386	0	Gabpb2	0
Ephb3	0	Fstl5	0	Zfp383	0	Gabpa	0
Ephb2	0	Fstl3	0	Zfp382	0	Gabbr2	0
Ephb1	0	Fstl1	0	Galt	0	Gabbr1	0
Epha8	0	Fsip1	0	Gm4133	0	Gabarapl2	0
Epha6	0	Fshr	0	1810024B03Rik	0	Gabarapl1	0
Epha4	0	Fshb	0	Klk1b5	0	Gabarap	0
Epha3	0	Fsd1l	0	Mrpl20	0	Gab3	0
Epha2	0	Fsd1	0	Vsig8	0	Gab2	0
Epha10	0	Fscn3	0	Mrpl22	0	Gab1	0
Epha1	0	Fscn2	0	Klk1b9	0	Gaa	0
Epdr1	0	Fscn1	0	Rad9a	0	G6pd2	0
Epc2	0	Fsbp	0	Vsig2	0	G6pc2	0
Epb4.2	0	Frzb	0	Tatdn3	0	G6pc	0
Epb4.1l5	0	Fryl	0	Tmem110	0	G6b	0
Epb4.1l4b	0	Frrs1l	0	Begain	0	G630090E17Rik	0
Epb4.1l4a	0	Frrs1	0	Gale	0	G2e3	0
Epb4.1l3	0	Frmpd4	0	BRDN0000737715	0	G0s2	0
Epb4.1l2	0	Frmpd3	0	Galc	0	Fzr1	0
Epb4.1	0	Frmpd1	0	Vmn1r200	0	Fzd9	0
Epas1	0	Frmd8	0	Sec31b	0	Fzd8	0
Ep400	0	Frmd7	0	Sec31a	0	Fzd7	0
Eomes	0	Frmd6	0	Fam161a	0	Fzd6	0
Eny2	0	Frmd4a	0	Thap3	0	Fzd5	0
Entpd8	0	Frmd3	0	Fam161b	0	Fzd4	0
Entpd6	0	Frg1	0	Hcfc2	0	Fzd3	0
Entpd5	0	Frem3	0	Hcfc1	0	Fzd2	0
Entpd4	0	Frem2	0	Gm5622	0	Fzd10	0
Entpd3	0	Frem1	0	Erlec1	0	Fxyd7	0
Enthd2	0	Frat2	0	Myot	0	Fxyd6	0
Enthd1	0	Fras1	0	Mtl5	0	Fxyd5	0
Ensa	0	Fpr-rs4	0	Atp11b	0	Fxyd4	0
Enpp7	0	Fpr-rs3	0	Csnk1g2	0	Fxyd3	0
Enpp6	0	Fpr3	0	Csnk1g3	0	Fxyd2	0
Enpp5	0	Fpr2	0	Amy2a4	0	Fxyd1	0
Enpp4	0	Fpr1	0	Csnk1g1	0	Fv1	0
Enpp3	0	Fpgt	0	Amy2a2	0	Fut8	0
Enpp2	0	Fpgs	0	Amy2a3	0	Fut4	0
Enpp1	0	Foxred2	0	Crmp1	0	Fut2	0
Enox1	0	Foxr2	0	Stk40	0	Fut11	0
Enoph1	0	Foxr1	0	Tnks	0	Fut1	0
Eno4	0	Foxq1	0	Mkrn1	0	Fus	0
Enkur	0	Foxp4	0	Olfr209	0	Fuca2	0
Enho	0	Foxp3	0	Hsd17b2	0	Fuca1	0
Engase	0	Foxp1	0	BRDN0000737989	0	Fubp3	0
Endov	0	Foxo6	0	Cramp1l	0	Ftsj3	0
Endou	0	Foxo3	0	Sox17	0	Ftsj1	0
Endod1	0	Foxo1	0	Arid1b	0	Fth1	0
Enc1	0	Foxn3	0	Arid1a	0	Fstl5	0
Enam	0	Foxl2	0	Olfr201	0	Fst	0
En2	0	Foxl1	0	Olfr206	0	Fshr	0
En1	0	Foxk2	0	Mkrn3	0	Fshb	0
Emp3	0	Foxk1	0	Olfr204	0	Fsd1l	0
Emp2	0	Foxj3	0	Uros	0	Fscb	0
Emp1	0	Foxj2	0	6430531B16Rik	0	Frzb	0
Eml5	0	Foxj1	0	Atcay	0	Fry	0
Eml4	0	Foxi3	0	Urod	0	Frs2	0
Eml3	0	Foxi1	0	Atrx	0	Frrs1l	0
Eml1	0	Foxh1	0	BRDN0000737817	0	Frrs1	0
Emilin3	0	Foxf2	0	Pbx3	0	Frmpd3	0
Emilin2	0	Foxf1	0	Gltp	0	Frmd8	0
Emilin1	0	Foxe3	0	Cep44	0	Frmd7	0
Emid1	0	Foxe1	0	Fbxw11	0	Frmd6	0
Emg1	0	Foxd3	0	Prpf40b	0	Frmd4a	0
Eme2	0	Foxd2	0	1700016D06Rik	0	Frmd3	0
Eme1	0	Foxc1	0	Meig1	0	Frk	0
Emd	0	Foxb2	0	Tnrc6c	0	Frg1	0
Emcn	0	Foxa2	0	Dek	0	Frem3	0
Emc8	0	Foxa1	0	Vmn2r74	0	Frem1	0
Emc7	0	Fosl2	0	Aifm2	0	Frat2	0
Emc6	0	Fosl1	0	Aifm3	0	Fras1	0
Emc4	0	Fosb	0	Aifm1	0	Fra10ac1	0
Emc3	0	Fos	0	BC027072	0	Fpr-rs4	0
Emc2	0	Fopnl	0	Chil3	0	Fpr3	0
Emc1	0	Folr2	0	Clpx	0	Fpgt	0
Emb	0	Folr1	0	Chil1	0	Fpgs	0
Elp6	0	Folh1	0	Sppl2b	0	Foxs1	0
Elp5	0	Focad	0	Cd63	0	Foxred1	0
Elp4	0	Fntb	0	Chil6	0	Foxr2	0

Elp3	0	Fnta	0	Seh1l	0	Foxr1	0
Elp2	0	Fnip2	0	Pias3	0	Foxp4	0
Elovl7	0	Fnip1	0	Ivl	0	Foxp2	0
Elovl5	0	Fndc8	0	Clpp	0	Foxp1	0
Elovl4	0	Fndc7	0	Clps	0	Foxo6	0
Elovl3	0	Fndc5	0	Fnbp4	0	Foxo4	0
Elmsan1	0	Fndc3c1	0	Neil2	0	Foxo3	0
Elmod3	0	Fndc3b	0	Cd69	0	Foxn4	0
Elmod2	0	Fndc3a	0	Cd68	0	Foxn3	0
Elmod1	0	Fndc1	0	Papolb	0	Foxn1	0
Elmo3	0	Fnd3c2	0	Bok	0	Foxl2	0
Elmo2	0	Fnbp1l	0	Eif4enif1	0	Foxl1	0
Elmo1	0	Fn3k	0	Asrgl1	0	Foxk2	0
Ell2	0	Fn1	0	Serpinb3c	0	Foxj3	0
Ell	0	Fmr1nb	0	Ern2	0	Foxj2	0
Elk4	0	Fmod	0	Ern1	0	Foxh1	0
Elk1	0	Fmo9	0	Gm9992	0	Foxg1	0
Elfn2	0	Fmo6	0	Abcg2	0	Foxf1	0
Elfn1	0	Fmo5	0	Ryk	0	Foxe1	0
Elf5	0	Fmo2	0	Gm9994	0	Foxd3	0
Elf4	0	Fmo1	0	Cyp2d9	0	Foxa3	0
Elf3	0	Fmn2	0	Tonsl	0	Foxa1	0
Elf1	0	Fmn1	0	1700019O17Rik	0	Fosl1	0
Elavl4	0	Flywch2	0	Crp	0	Fos	0
Elavl3	0	Flywch1	0	Stfa3	0	Folr2	0
Elac2	0	Flt4	0	Stfa2	0	Folr1	0
Elac1	0	Flt3	0	Stfa1	0	Focad	0
Eif6	0	Flrt3	0	Drosha	0	Fntb	0
Eif5b	0	Flrt2	0	Fbp1	0	Fnta	0
Eif5a2	0	Flrt1	0	Fbp2	0	Fnip2	0
Eif5a	0	Flot2	0	BRDN0000737393	0	Fndc9	0
Eif5	0	Flot1	0	BRDN0000737392	0	Fndc8	0
Eif4h	0	Flnb	0	Serpinb3b	0	Fndc7	0
Eif4g2	0	Flii	0	Mtpap	0	Fndc5	0
Eif4enif1	0	Fli1	0	Adssl1	0	Fndc3b	0
Eif4ebp3	0	Flcn	0	BRDN0000737396	0	Fndc3a	0
Eif4ebp1	0	Flad1	0	Taf1c	0	Fndc1	0
Eif4e3	0	Fktn	0	BRDN0000737394	0	Fnd3c2	0
Eif4e1b	0	Fkrp	0	Vmn1r23	0	Fnbp4	0
Eif4e	0	Fkbp9	0	Vmn1r22	0	Fnbp1l	0
Eif4b	0	Fkbp8	0	Vmn1r20	0	Fn1	0
Eif4a3	0	Fkbp6	0	Vmn1r26	0	Fmr1nb	0
Eif4a1	0	Fkbp5	0	Vmn1r25	0	Fmod	0
Eif3m	0	Fkbp4	0	Vmn1r24	0	Fmo6	0
Eif3l	0	Fkbp1b	0	BRDN0000737743	0	Fmo2	0
Eif3j2	0	Fkbp1a	0	Chpf2	0	Fmo1	0
Eif3j1	0	Fkbp14	0	Vmn1r28	0	Fmnl1	0
Eif3i	0	Fkbp11	0	Lmx1a	0	Fmn2	0
Eif3h	0	Fitm2	0	Lmx1b	0	Flywch2	0
Eif3g	0	Fitm1	0	Nelfa	0	Flt3	0
Eif3f	0	Fis1	0	Colq	0	Flt1	0
Eif3e	0	Fip1l1	0	Nbn	0	Flrt3	0
Eif3d	0	Filip1l	0	Olfr867	0	Flrt1	0
Eif3c	0	Filip1	0	Myo16	0	Flot2	0
Eif3b	0	Fignl2	0	Hsd17b7	0	Flnc	0
Eif2s3y	0	Fignl1	0	Jtb	0	Flnb	0
Eif2s3x	0	Fign	0	Crx	0	Flna	0
Eif2s2	0	Fig4	0	Arfgap3	0	Flii	0
Eif2s1	0	Fibp	0	4930564D02Rik	0	Fli1	0
Eif2d	0	Fibcd1	0	Tmem19	0	Flad1	0
Eif2b5	0	Fhl5	0	2900092C05Rik	0	Fktn	0
Eif2b4	0	Fhl4	0	Ace	0	Fkbpl	0
Eif2b3	0	Fhl2	0	Acd	0	Fkbp9	0
Eif2b2	0	Fhl1	0	Mrpl37	0	Fkbp8	0
Eif2b1	0	Fhit	0	Kcnq1	0	Fkbp6	0
Eif2ak4	0	Fhdc1	0	Lrrc20	0	Fkbp5	0
Eif2ak3	0	Fhad1	0	Dmrt2	0	Fkbp4	0
Eif2ak2	0	Fgr	0	Cep192	0	Fkbp3	0
Eif2a	0	Fgl2	0	BRDN0000737577	0	Fkbp1a	0
Eif1ax	0	Fgl1	0	BRDN0000737575	0	Fkbp14	0
Eif1ad	0	Fggy	0	Dmrt3	0	Fkbp11	0
Eif1a	0	Fgfrl1	0	BRDN0000737573	0	Fkbp10	0
Eid2b	0	Fgfr2	0	BRDN0000737572	0	Fiz1	0
Eid2	0	Fgfr1op	0	BRDN0000737571	0	Fitm2	0
Ei24	0	Fgfbp3	0	Ccdc85b	0	Fitm1	0
Ehmt2	0	Fgfbp1	0	Chl1	0	Fis1	0
Ehhadh	0	Fgf9	0	Klk1b21	0	Fip1l1	0
Ehf	0	Fgf6	0	Klk1b22	0	Filip1l	0
Ehd4	0	Fgf22	0	Ier5	0	Filip1	0
Ehd3	0	Fgf20	0	Klk1b26	0	Fignl1	0
Ehd2	0	Fgf17	0	Hnrnph2	0	Fign	0
Ehd1	0	Fgf15	0	Clec5a	0	Figla	0

Ehbp1l1	0	Fgf11	0	Qser1	0	Fig4	0
Ehbp1	0	Fgf10	0	Myo1h	0	Ficd	0
Egr4	0	Fgf1	0	Myo1g	0	Fibp	0
Egr3	0	Fgd5	0	Myo1f	0	Fibin	0
Egr2	0	Fgd3	0	Myo1e	0	Fhod3	0
Egr1	0	Fgd2	0	Myo1d	0	Fhod1	0
Egln3	0	Fgd1	0	Myo1c	0	Fhl5	0
Egln2	0	Ffar2	0	Myo1b	0	Fhl4	0
Egln1	0	Ffar1	0	Taar7f	0	Fhl2	0
Egfr	0	Fezf2	0	Gda	0	Fhl1	0
Egflam	0	Fezf1	0	Brip1	0	Fhdc1	0
Egfl8	0	Fez2	0	Kcnmb2	0	Fh1	0
Egfl7	0	Fev	0	Baz2a	0	Fgr	0
Egfem1	0	Fetub	0	Baz2b	0	Fgl1	0
Egf	0	Fes	0	BRDN0000737916	0	Fggy	0
Eftud2	0	Fermt3	0	A430105I19Rik	0	Fgfr4	0
Efs	0	Fermt2	0	Pcx	0	Fgfr2	0
Efr3b	0	Ferd3l	0	Cygb	0	Fgfr1op	0
Efr3a	0	Fer1l5	0	Dnaaf2	0	Fgf9	0
Efnb3	0	Fer	0	BRDN0000737468	0	Fgf8	0
Efnb2	0	Fem1c	0	Rbbp8	0	Fgf5	0
Efnb1	0	Fem1b	0	Strada	0	Fgf3	0
Efna4	0	Fech	0	Nmt2	0	Fgf22	0
Efhd2	0	Fdxacb1	0	Nmt1	0	Fgf21	0
Efhd1	0	Fdx1l	0	C77370	0	Fgf20	0
Efhc2	0	Fdx1	0	Rpgrip1	0	Fgf17	0
Efhc1	0	Fdps	0	Cyp2c29	0	Fgf16	0
Efhb	0	Fcrla	0	BRDN0000737473	0	Fgf15	0
Efemp2	0	Fcrl6	0	Gm5483	0	Fgf14	0
Efemp1	0	Fcrl5	0	BRDN0000737713	0	Fgf13	0
Efcc1	0	Fcnb	0	Olfr1051	0	Fgf11	0
Efcab5	0	Fcna	0	Olfr1053	0	Fgf1	0
Efcab3	0	Fchsd1	0	Olfr1052	0	Fgd6	0
Efcab2	0	Fcho1	0	Rasl12	0	Fgd4	0
Efcab14	0	Fcgr4	0	Olfr1056	0	Fgd3	0
Efcab11	0	Fcgr2b	0	Olfr1058	0	Fgd2	0
Efcab10	0	Fcgr1	0	Drd4	0	Ffar4	0
Efcab1	0	Fcgbp	0	Drd5	0	Ffar3	0
Eepd1	0	Fcer2a	0	Drd2	0	Ffar1	0
Eefsec	0	Fcer1g	0	Drd3	0	Fez2	0
Eef2kmt	0	Fbxw9	0	Drd1	0	Fez1	0
Eef2k	0	Fbxw8	0	Stradb	0	Fev	0
Eef2	0	Fbxw7	0	Catsper3	0	Fer1l5	0
Eef1e1	0	Fbxw5	0	Glyr1	0	Fer1l4	0
Eef1d	0	Fbxw28	0	BRDN0000737910	0	Fer	0
Eef1b2	0	Fbxw26	0	Cacybp	0	Fen1	0
Eef1a2	0	Fbxw22	0	Zfp688	0	Fem1c	0
Eef1a1	0	Fbxw2	0	Olfr122	0	Fem1a	0
Eed	0	Fbxw19	0	Sh3gl2	0	Fech	0
Eea1	0	Fbxw18	0	Inha	0	Fdxr	0
Edrf1	0	Fbxw17	0	Samd14	0	Fdxacb1	0
Ednrb	0	Fbxw15	0	Samd15	0	Fdx1l	0
Ednra	0	Fbxw13	0	Bri3bp	0	Fdx1	0
Edn2	0	Fbxw10	0	Gm14151	0	Fdps	0
Edn1	0	Fbxo8	0	Samd10	0	Fcrls	0
Edil3	0	Fbxo6	0	Samd11	0	Fcrla	0
Edf1	0	Fbxo5	0	Rbbp7	0	Fcrl6	0
Edem3	0	Fbxo44	0	Olfr361	0	Fcrl5	0
Edem2	0	Fbxo43	0	Pdgfra	0	Fcnb	0
Edem1	0	Fbxo42	0	Ccny	0	Fcna	0
Edc4	0	Fbxo41	0	Gnaq	0	Fchsd1	0
Edc3	0	Fbxo40	0	Akt1	0	Fcgrt	0
Edaradd	0	Fbxo39	0	Akt2	0	Fcgr2b	0
Edar	0	Fbxo33	0	Akt3	0	Fcgr1	0
Eda	0	Fbxo32	0	Zfp235	0	Fcgbp	0
Ect2l	0	Fbxo30	0	1700007K09Rik	0	Fcer2a	0
Ect2	0	Fbxo3	0	Olfr121	0	Fcer1a	0
Ecsit	0	Fbxo28	0	Rbbp4	0	Fbxw9	0
Ecscr	0	Fbxo27	0	Top3a	0	Fbxw8	0
Ecm2	0	Fbxo25	0	Mrpl30	0	Fbxw7	0
Eci3	0	Fbxo24	0	Naa16	0	Fbxw5	0
Eci2	0	Fbxo22	0	Ang4	0	Fbxw4	0
Eci1	0	Fbxo21	0	BRDN0000737420	0	Fbxw28	0
Echs1	0	Fbxo2	0	Naa15	0	Fbxw22	0
Echdc2	0	Fbxo17	0	Vmn2r79	0	Fbxw21	0
Echdc1	0	Fbxo16	0	Vmn2r78	0	Fbxw20	0
Ecel1	0	Fbxo15	0	Naa10	0	Fbxw19	0
Ece2	0	Fbxo11	0	Cux1	0	Fbxw18	0
Ece1	0	Fbxo10	0	Vmn2r75	0	Fbxw17	0
Ecd	0	Fbxl8	0	Fubp3	0	Fbxw16	0
Ebpl	0	Fbxl7	0	Vmn2r77	0	Fbxw13	0
Ebp	0	Fbxl6	0	Vmn2r76	0	Fbxw11	0

Ebna1bp2	0	Fbxl5	0	Vmn2r71	0	Fbxo8	0
Ebi3	0	Fbxl21	0	Slu7	0	Fbxo7	0
Ebf4	0	Fbxl20	0	Vmn2r73	0	Fbxo5	0
Ebf3	0	Fbxl19	0	Vmn2r72	0	Fbxo48	0
Ebf2	0	Fbxl18	0	Taf1d	0	Fbxo47	0
Ebf1	0	Fbxl17	0	Apoc4	0	Fbxo46	0
Ebag9	0	Fbxl14	0	BRDN0000737912	0	Fbxo45	0
Ear2	0	Fbxl12	0	Apoc2	0	Fbxo44	0
Ear14	0	Fbrs	0	Apoc3	0	Fbxo43	0
Ear10	0	Fbp2	0	Sap18	0	Fbxo42	0
Ear1	0	Fbp1	0	Apoc1	0	Fbxo41	0
Eapp	0	Fbn2	0	Noto	0	Fbxo40	0
Eaf2	0	Fbn1	0	Mex3a	0	Fbxo38	0
Eaf1	0	Fbln7	0	Tspyl3	0	Fbxo34	0
E4f1	0	Fbln5	0	Gh	0	Fbxo32	0
E430025E21Rik	0	Fbln1	0	E2f1	0	Fbxo3	0
E430018J23Rik	0	Fau	0	Pradc1	0	Fbxo22	0
E330021D16Rik	0	Fat3	0	1600014C10Rik	0	Fbxo2	0
E2f8	0	Fat2	0	Pde3a	0	Fbxo18	0
E2f7	0	Fastkd5	0	Dsg1a	0	Fbxo17	0
E2f5	0	Fastkd2	0	Pde3b	0	Fbxo15	0
E2f4	0	Fastkd1	0	March5	0	Fbxo10	0
E2f3	0	Fastk	0	March4	0	Fbxl7	0
E2f1	0	Fasn	0	March7	0	Fbxl6	0
E230025N22Rik	0	Fas	0	March6	0	Fbxl5	0
E230019M04Rik	0	Farsa	0	March1	0	Fbxl4	0
E130309D14Rik	0	Fars2	0	AI597479	0	Fbxl20	0
E130309D02Rik	0	Farp1	0	Olfr1353	0	Fbxl19	0
E130308A19Rik	0	Far2	0	Slc18a3	0	Fbxl17	0
E030030I06Rik	0	Far1	0	Slc18a2	0	Fbxl16	0
E030018B13Rik	0	Fap	0	Slc18a1	0	Fbxl15	0
Dzip1l	0	Fank1	0	Agtrap	0	Fbxl14	0
Dzank1	0	Fancm	0	March9	0	Fbxl12	0
Dysf	0	Fanci	0	March8	0	Fbrsl1	0
Dyrk4	0	Fancf	0	Mrrf	0	Fbrs	0
Dyrk3	0	Fancd2os	0	Ctss	0	Fbp2	0
Dyrk2	0	Fancc	0	Sbp	0	Fbp1	0
Dyrk1b	0	Fancb	0	Mad2l1	0	Fbln7	0
Dyrk1a	0	Fan1	0	Tab3	0	Fbln1	0
Dynlt3	0	Fam98b	0	Tab2	0	Fblim1	0
Dynlt1f	0	Fam98a	0	Tab1	0	Fbl	0
Dynlt1c	0	Fam96b	0	Fam222b	0	Faxc	0
Dynlt1b	0	Fam96a	0	Dsg1b	0	Fau	0
Dynlt1a	0	Fam92a	0	Olfr1357	0	Fat3	0
Dynlrb2	0	Fam89a	0	Vil1	0	Fastkd5	0
Dynlrb1	0	Fam84b	0	Olfr1354	0	Fastkd3	0
Dynll2	0	Fam84a	0	Metrn	0	Fastkd2	0
Dynll1	0	Fam83h	0	Mme	0	Fastkd1	0
Dync2h1	0	Fam83g	0	Mmd	0	Fastk	0
Dync1li2	0	Fam83f	0	Olfr1427	0	Fasn	0
Dync1li1	0	Fam83d	0	Wasl	0	Fasl	0
Dync1i2	0	Fam83b	0	Adcy4	0	Fas	0
Dync1i1	0	Fam83a	0	Adcy5	0	Farsb	0
Dync1h1	0	Fam81a	0	Ppp1r7	0	Farsa	0
Dynap	0	Fam78b	0	Adcy7	0	Fars2	0
Dym	0	Fam78a	0	Cpm	0	Farp2	0
Dydc2	0	Fam76a	0	Adcy2	0	Farp1	0
Dydc1	0	Fam73b	0	Ppp1r2	0	Far2	0
Dvl3	0	Fam72a	0	Rprml	0	Fap	0
Dut	0	Fam71f2	0	Krtcap2	0	Fank1	0
Dusp7	0	Fam71f1	0	Tmem79	0	Fancm	0
Dusp6	0	Fam71a	0	Cpz	0	Fancl	0
Dusp5	0	Fam69c	0	Btbd18	0	Fancg	0
Dusp4	0	Fam69a	0	Plscr3	0	Fancf	0
Dusp3	0	Fam65c	0	Ttc25	0	Fancc	0
Dusp27	0	Fam65a	0	Tmem70	0	Fanca	0
Dusp22	0	Fam63a	0	Tmem71	0	Fan1	0
Dusp21	0	Fam60a	0	Tmem72	0	Fam98c	0
Dusp2	0	Fam58b	0	Rassf10	0	Fam98b	0
Dusp19	0	Fam57a	0	BRDN0000737461	0	Fam98a	0
Dusp16	0	Fam53c	0	Slc6a18	0	Fam96b	0
Dusp15	0	Fam53b	0	Ttc27	0	Fam96a	0
Dusp14	0	Fam53a	0	Slc7a8	0	Fam92a	0
Dusp12	0	Fam50a	0	Laptm4a	0	Fam89b	0
Dusp11	0	Fam49b	0	Slc7a6	0	Fam84b	0
Dusp10	0	Fam49a	0	Arxes2	0	Fam83h	0
Dusp1	0	Fam47e	0	Arxes1	0	Fam83g	0
Dus4l	0	Fam46d	0	Slc7a5	0	Fam83d	0
Dus3l	0	Fam46c	0	Slc7a2	0	Fam83c	0
Dus2	0	Fam46b	0	Slc7a3	0	Fam83b	0
Dus1l	0	Fam46a	0	Slc7a1	0	Fam83a	0
Dupd1	0	Fam45a	0	Foxg1	0	Fam78a	0

Duoxa2	0	Fam43b	0	Olfr1234	0	Fam76b	0
Duox2	0	Fam43a	0	Garnl3	0	Fam73b	0
Duox1	0	Fam3c	0	Hnrnpa3	0	Fam72a	0
Dtymk	0	Fam3a	0	Col16a1	0	Fam71e1	0
Dtx4	0	Fam35a	0	Ttc23	0	Fam71b	0
Dtx3l	0	Fam32a	0	Nrsn2	0	Fam71a	0
Dtx2	0	Fam26f	0	Il9r	0	Fam69c	0
Dtx1	0	Fam26e	0	Nmur2	0	Fam69b	0
Dtwd2	0	Fam26d	0	Nmur1	0	Fam65c	0
Dtwd1	0	Fam25c	0	Hnrnpa1	0	Fam65a	0
Dtnbp1	0	Fam228b	0	Slc17a8	0	Fam64a	0
Dtnb	0	Fam228a	0	Slc17a9	0	Fam63b	0
Dtna	0	Fam227b	0	Rsl24d1	0	Fam60a	0
Dtd2	0	Fam222b	0	Stub1	0	Fam58b	0
Dtd1	0	Fam222a	0	Slc17a6	0	Fam57b	0
Dstn	0	Fam221b	0	Slc17a7	0	Fam57a	0
Dst	0	Fam219b	0	Slc17a2	0	Fam53a	0
Dspp	0	Fam219a	0	Slc17a3	0	Fam50a	0
Dsp	0	Fam217b	0	Sh3pxd2b	0	Fam49b	0
Dsg3	0	Fam217a	0	Slc38a2	0	Fam47e	0
Dsg2	0	Fam216b	0	Ctsw	0	Fam47c	0
Dsg1c	0	Fam216a	0	BRDN0000737911	0	Fam46d	0
Dsg1b	0	Fam214b	0	Rin2	0	Fam46c	0
Dsel	0	Fam214a	0	Vmn1r2	0	Fam46b	0
Dscr3	0	Fam212b	0	Piwil1	0	Fam46a	0
Dscc1	0	Fam212a	0	Akr1cl	0	Fam3c	0
Dscaml1	0	Fam210a	0	Sh3pxd2a	0	Fam3a	0
Dsc3	0	Fam21	0	Psmd9	0	Fam35a	0
Dsc2	0	Fam20c	0	Psmd8	0	Fam32a	0
Dsc1	0	Fam20b	0	Npc2	0	Fam26f	0
Drosha	0	Fam209	0	Psmd5	0	Fam26e	0
Drg2	0	Fam208b	0	Psmd4	0	Fam26d	0
Drg1	0	Fam207a	0	Psmd7	0	Fam25c	0
Drd5	0	Fam204a	0	Psmd6	0	Fam229a	0
Draxin	0	Fam19a5	0	Psmd1	0	Fam228a	0
Drap1	0	Fam19a4	0	Sgol1	0	Fam227b	0
Dram1	0	Fam19a3	0	Psmd3	0	Fam227a	0
Dr1	0	Fam19a2	0	Psmd2	0	Fam222b	0
Dqx1	0	Fam19a1	0	Map3k19	0	Fam221b	0
Dpysl4	0	Fam198b	0	Agpat6	0	Fam219b	0
Dpysl3	0	Fam198a	0	Ica1l	0	Fam219a	0
Dpys	0	Fam196a	0	Agpat4	0	Fam217b	0
Dpy30	0	Fam195b	0	Agpat3	0	Fam217a	0
Dpy19l4	0	Fam195a	0	Ifrd1	0	Fam216b	0
Dpy19l3	0	Fam193b	0	Agpat1	0	Fam214b	0
Dpy19l2	0	Fam189b	0	Dct	0	Fam214a	0
Dpy19l1	0	Fam189a2	0	Cntnap5b	0	Fam212b	0
Dpt	0	Fam189a1	0	Hoxc4	0	Fam210b	0
Dppa5a	0	Fam187a	0	Nprl2	0	Fam210a	0
Dppa4	0	Fam186b	0	Slc25a53	0	Fam21	0
Dppa2	0	Fam185a	0	Lcn4	0	Fam20b	0
Dppa1	0	Fam184b	0	Slc25a51	0	Fam20a	0
Dpp9	0	Fam184a	0	Itpripl2	0	Fam208b	0
Dpp8	0	Fam183b	0	Lcn3	0	Fam208a	0
Dpp6	0	Fam181a	0	Itpripl1	0	Fam207a	0
Dpp4	0	Fam180a	0	Pkdrej	0	Fam206a	0
Dpp3	0	Fam178a	0	Prl2a1	0	Fam204a	0
Dpp10	0	Fam177a	0	Lcn8	0	Fam19a5	0
Dpm3	0	Fam175b	0	Ermp1	0	Fam19a4	0
Dpm2	0	Fam175a	0	Prr30	0	Fam19a3	0
Dpm1	0	Fam174b	0	Nipsnap3a	0	Fam19a2	0
Dph7	0	Fam174a	0	Tmem201	0	Fam198a	0
Dph6	0	Fam173b	0	Tmem202	0	Fam196b	0
Dph5	0	Fam173a	0	Nipsnap3b	0	Fam196a	0
Dph3	0	Fam172a	0	Tmem204	0	Fam195a	0
Dph1	0	Fam171a2	0	Tmem205	0	Fam193b	0
Dpf2	0	Fam171a1	0	Tmem206	0	Fam193a	0
Dpep3	0	Fam170b	0	Hba-a2	0	Fam192a	0
Dpep2	0	Fam170a	0	Tmem208	0	Fam189b	0
Dpep1	0	Fam168b	0	Tmem209	0	Fam189a1	0
Dpcd	0	Fam168a	0	Phf20l1	0	Fam188a	0
Dpagt1	0	Fam167b	0	Iltifb	0	Fam187b	0
Doxl2	0	Fam167a	0	Cactin	0	Fam186b	0
Dot1l	0	Fam166b	0	Zfp446	0	Fam184b	0
Dos	0	Fam166a	0	Zfp444	0	Fam184a	0
Dopey1	0	Fam163b	0	Zfp442	0	Fam183b	0
Donson	0	Fam162b	0	Pcdhb3	0	Fam181a	0
Dolpp1	0	Fam162a	0	Zpbp	0	Fam180a	0
Dolk	0	Fam161b	0	Mex3c	0	Fam179b	0
Dok5	0	Fam161a	0	Ddx31	0	Fam178b	0
Dok4	0	Fam160b2	0	Pcdhb7	0	Fam178a	0
Dok3	0	Fam160b1	0	Mras	0	Fam175b	0

Dok2	0	Fam159b	0	Pcdhb5	0	Fam175a	0
Dok1	0	Fam155a	0	Zfp449	0	Fam174b	0
Dohh	0	Fam151a	0	Lce1a1	0	Fam173b	0
Dock9	0	Fam150b	0	Lce1a2	0	Fam172a	0
Dock7	0	Fam150a	0	Mcfd2	0	Fam171a1	0
Dock6	0	Fam149a	0	Noa1	0	Fam170b	0
Dock5	0	Fam13b	0	Vmn1r176	0	Fam170a	0
Dock4	0	Fam135b	0	Tas2r116	0	Fam168a	0
Dock3	0	Fam135a	0	Vmn1r174	0	Fam167b	0
Dock11	0	Fam134c	0	Vmn1r175	0	Fam167a	0
Dock1	0	Fam133b	0	Vmn1r172	0	Fam166b	0
Doc2a	0	Fam132a	0	Vmn1r173	0	Fam163b	0
Dnttip2	0	Fam131c	0	Gm10220	0	Fam162b	0
Dnttip1	0	Fam131b	0	Larp4b	0	Fam162a	0
Dnph1	0	Fam131a	0	Rfc5	0	Fam161b	0
Dnpep	0	Fam129a	0	Rfc4	0	Fam160b2	0
Dnmt3l	0	Fam126b	0	Pcdh15	0	Fam160b1	0
Dnmt3b	0	Fam126a	0	Rfc1	0	Fam160a2	0
Dnmt3a	0	Fam124a	0	Ptdss2	0	Fam159b	0
Dnmt1	0	Fam122a	0	Gm10228	0	Fam159a	0
Dnm3	0	Fam120c	0	Mgst1	0	Fam151a	0
Dnm2	0	Fam120b	0	Sars	0	Fam150b	0
Dnm1l	0	Fam117b	0	Tas2r113	0	Fam150a	0
Dnm1	0	Fam114a2	0	Odf2	0	Fam13c	0
Dnlz	0	Fam114a1	0	BRDN0000738114	0	Fam13b	0
Dner	0	Fam111a	0	Pnliprp2	0	Fam136a	0
Dnd1	0	Fam110c	0	Asah2	0	Fam135b	0
Dnase2a	0	Fam110a	0	Pygm	0	Fam134c	0
Dnase1l1	0	Fam107a	0	Gm4201	0	Fam134b	0
Dnase1	0	Fam105a	0	Fign	0	Fam133b	0
Dnali1	0	Fam104a	0	BRDN0000738116	0	Fam132a	0
Dnal4	0	Fam103a1	0	Usp22	0	Fam131b	0
Dnal1	0	Fam102a	0	Klhl6	0	Fam131a	0
Dnajc9	0	Fam101a	0	Klhl7	0	Fam129a	0
Dnajc7	0	Faim3	0	Klhl5	0	Fam126b	0
Dnajc6	0	Faim2	0	Emp2	0	Fam126a	0
Dnajc5b	0	Faim	0	Klhl3	0	Fam124b	0
Dnajc5	0	Faf2	0	Emp1	0	Fam124a	0
Dnajc4	0	Faf1	0	BRDN0000738110	0	Fam122c	0
Dnajc30	0	Fads6	0	Npm2	0	Fam122b	0
Dnajc3	0	Fads3	0	Lce3d	0	Fam122a	0
Dnajc28	0	Fads2	0	Klhl8	0	Fam120c	0
Dnajc27	0	Fads1	0	Klhl9	0	Fam120b	0
Dnajc25	0	Fadd	0	Smap1	0	Fam120a	0
Dnajc22	0	Fabp9	0	Smap2	0	Fam118b	0
Dnajc21	0	Fabp7	0	BRDN0000738112	0	Fam117a	0
Dnajc2	0	Fabp6	0	Zmynd8	0	Fam114a2	0
Dnajc17	0	Fabp5	0	Paf1	0	Fam111a	0
Dnajc16	0	Fabp4	0	BRDN0000738113	0	Fam110c	0
Dnajc14	0	Fabp3	0	Uaca	0	Fam110a	0
Dnajc13	0	Fabp12	0	Atf7ip	0	Fam109b	0
Dnajc12	0	Faah	0	BRDN0000737601	0	Fam107a	0
Dnajc11	0	Fa2h	0	Uhrf1bp1l	0	Fam105a	0
Dnajc10	0	F8a	0	Sumo1	0	Fam103a1	0
Dnajb9	0	F830016B08Rik	0	Hbb-bh1	0	Fam101a	0
Dnajb8	0	F3	0	Ppa2	0	Faim3	0
Dnajb7	0	F2rl3	0	Slc35f3	0	Faim2	0
Dnajb6	0	F2rl2	0	Rlim	0	Faim	0
Dnajb5	0	F2r	0	Nlk	0	Fahd2a	0
Dnajb4	0	F13a1	0	Cox7a2l	0	Fah	0
Dnajb3	0	F11r	0	Kdm1a	0	Faf2	0
Dnajb14	0	F10	0	Kdm1b	0	Faf1	0
Dnajb12	0	Ezr	0	BRDN0000737602	0	Fads2	0
Dnajb11	0	Ezh2	0	C87414	0	Fads1	0
Dnajb1	0	Ezh1	0	BRDN0000737605	0	Fadd	0
Dnaja4	0	Eya4	0	BRDN0000738269	0	Fabp7	0
Dnaja3	0	Eya3	0	Tac1	0	Fabp6	0
Dnaja2	0	Eya2	0	Slc6a17	0	Fabp5	0
Dnaja1	0	Extl3	0	Olfr351	0	Fabp3	0
Dnaic1	0	Extl2	0	Rimkla	0	Faah	0
Dnah9	0	Extl1	0	Defb33	0	F8a	0
Dnah8	0	Ext2	0	Fig4	0	F830045P16Rik	0
Dnah7b	0	Exosc9	0	Rimklb	0	F830016B08Rik	0
Dnah7a	0	Exosc8	0	Defb37	0	F8	0
Dnah6	0	Exosc5	0	Defb35	0	F7	0
Dnah2	0	Exosc4	0	Olfr417	0	F2rl3	0
Dnah17	0	Exosc3	0	BRDN0000738010	0	F13a1	0
Dnah11	0	Exosc1	0	Defb38	0	F11r	0
Dnah10	0	Exog	0	BRDN0000737766	0	F10	0
Dnah1	0	Exoc7	0	Kif27	0	Ezr	0
Dnaaf5	0	Exoc6	0	Olfr411	0	Ezh2	0
Dnaaf3	0	Exoc5	0	Rif1	0	Ezh1	0

Dnaaf1	0	Exoc3l4	0	Ano9	0	Eya4	0
Dna2	0	Exoc2	0	Cd200r3	0	Eya3	0
Dmxl2	0	Exoc1	0	Itsn1	0	Eya2	0
Dmwd	0	Exo5	0	Ano5	0	Ext2	0
Dmrtc1b	0	Ewsr1	0	Mogs	0	Ext1	0
Dmrtc1a	0	Evx2	0	Itsn2	0	Exph5	0
Dmrtb1	0	Evl	0	Ano2	0	Exosc9	0
Dmrta1	0	Evi5l	0	Ano3	0	Exosc8	0
Dmrt3	0	Evi2a	0	Dach2	0	Exosc7	0
Dmrt2	0	Evc2	0	Pdgfc	0	Exosc5	0
Dmrt1	0	Evc	0	4930524B15Rik	0	Exosc4	0
Dmpk	0	Eva1c	0	Snhg11	0	Exosc3	0
Dmp1	0	Eva1b	0	BRDN0000737742	0	Exosc2	0
Dmkn	0	Etv6	0	Kif22	0	Exosc10	0
Dmgdh	0	Etv5	0	Nkx6-2	0	Exog	0
Dmd	0	Etv2	0	Nkx6-3	0	Exoc8	0
Dmc1	0	Ets2	0	Nkx6-1	0	Exoc7	0
Dmbx1	0	Etohi1	0	Irf3	0	Exoc6	0
Dmbt1	0	Etnppl	0	Irf2	0	Exoc4	0
Dmap1	0	Etnk2	0	Irf7	0	Exoc3l4	0
Dlx6	0	Etnk1	0	Irf6	0	Exoc3l	0
Dlx5	0	Etl4	0	Slc6a15	0	Exoc2	0
Dlx4	0	Etfb	0	Irf8	0	Exoc1	0
Dlx3	0	Etfa	0	Nodal	0	Exo5	0
Dlx1	0	Etf1	0	Gm12887	0	Exo1	0
Dlst	0	Esyt3	0	Picalm	0	Exd1	0
Dll1	0	Esyt2	0	Clk1	0	Ewsr1	0
Dlk2	0	Esx1	0	Clk2	0	Evx2	0
Dlk1	0	Esrrg	0	Clk3	0	Evpl	0
Dlgap5	0	Esrp2	0	Gm12888	0	Evl	0
Dlgap4	0	Esrp1	0	Herpud2	0	Evi5l	0
Dlgap3	0	Espn	0	Herpud1	0	Evi5	0
Dlgap2	0	Esp8	0	Lelp1	0	Evi2a	0
Dlgap1	0	Esp6Esp5	0	Zbtb17	0	Evc2	0
Dlg4	0	Esp6	0	Zbtb16	0	Evc	0
Dlg3	0	Esp4	0	BRDN0000737913	0	Eva1b	0
Dlg2	0	Esp38	0	Zbtb14	0	Etv6	0
Dleu7	0	Esp36	0	Slc6a14	0	Etv5	0
Dlec1	0	Esp34	0	Olfr1099	0	Etv3	0
Dld	0	Esp31	0	Zbtb18	0	Etv2	0
Dlc1	0	Esp3	0	Fdxacb1	0	Etv1	0
Dkkl1	0	Esp24	0	Haus3	0	Etnppl	0
Dkk4	0	Esp23	0	Phgdh	0	Etnk1	0
Dkk2	0	Esp18	0	Sde2	0	Ethe1	0
Dkk1	0	Esp15	0	Cttnbp2nl	0	Etfdh	0
Dkc1	0	Esp1	0	Agfg1	0	Etfb	0
Disp2	0	Esm1	0	Marveld1	0	Etfa	0
Disp1	0	Esf1	0	Dmbt1	0	Etf1	0
Dis3l	0	Esco2	0	Gtf2h3	0	Esyt3	0
Dis3	0	Esco1	0	Gtf2h2	0	Esyt2	0
Dirc2	0	Esam	0	Gtf2h1	0	Esyt1	0
Diras2	0	Errfi1	0	Cdc42se2	0	Esx1	0
Dip2c	0	Erp29	0	Zfp36l1	0	Esrrg	0
Dip2a	0	Erp27	0	Zfp36l2	0	Esrp2	0
Dio3	0	Ero1lb	0	Gtf2h4	0	Esrp1	0
Dio2	0	Ero1l	0	Gm14308	0	Esr2	0
Dio1	0	Ern2	0	Abhd8	0	Espnl	0
Dimt1	0	Ermard	0	Abhd2	0	Espn	0
Diexf	0	Ermap	0	Abhd3	0	Espl1	0
Dido1	0	Erich6	0	Abhd6	0	Esp8	0
Dicer1	0	Erich5	0	Eral1	0	Esp6	0
Diap2	0	Erich3	0	Abhd5	0	Esp36	0
Diap1	0	Erich1	0	D10Wsu102e	0	Esp24	0
Diablo	0	Eri3	0	Retnlb	0	Esp23	0
Dhx57	0	Eri2	0	Wdr5	0	Esp18	0
Dhx40	0	Eri1	0	Wdr4	0	Esp16	0
Dhx37	0	Erh	0	Retnlg	0	Esp15	0
Dhx36	0	Ergic3	0	Wdr1	0	Esp1	0
Dhx35	0	Ergic2	0	Ccdc163	0	Esm1	0
Dhx33	0	Ergic1	0	Atp1b4	0	Esf1	0
Dhx32	0	Erg	0	Mdc1	0	Esd	0
Dhx29	0	Ereg	0	Ccdc167	0	Esco1	0
Dhx16	0	Ercc8	0	Ccdc166	0	Erv3	0
Dhx15	0	Ercc5	0	Lce3c	0	Erp44	0
Dhtkd1	0	Ercc4	0	Fcgbp	0	Erp29	0
Dhrs9	0	Ercc3	0	Prkci	0	Erp27	0
Dhrs7c	0	Ercc2	0	Mlxip	0	Ero1lb	0
Dhrs7b	0	Ercc1	0	Crygn	0	Ern2	0
Dhrs7	0	Erc2	0	Cryga	0	Ern1	0
Dhrs4	0	Erbb4	0	Crygb	0	Ermp1	0
Dhrs2	0	Erbb3	0	Crygc	0	Ermard	0
Dhrs13	0	Erbb2ip	0	Crygd	0	Ermap	0

Dhrs11	0	Erbb2	0	Cryge	0	Erlin1	0
Dhrs1	0	Eral1	0	Crygf	0	Erlec1	0
Dhps	0	Epx	0	Il5ra	0	Erich6	0
Dhodh	0	Ept1	0	2310067B10Rik	0	Erich3	0
Dhfr	0	Eps8l3	0	Dpcd	0	Erich1	0
Dhdh	0	Eps8l2	0	Grifin	0	Eri2	0
Dhdds	0	Eps8	0	Sep15	0	Eri1	0
Dhcr7	0	Eps15l1	0	Bin1	0	Erh	0
Dhcr24	0	Eps15	0	Gm996	0	Ergic3	0
Dgkz	0	Eprs	0	Gm7978	0	Erf	0
Dgke	0	Eppin	0	Tpbg	0	Ereg	0
Dgkd	0	Epor	0	Man1c1	0	Ercc6l2	0
Dgkb	0	Epo	0	Fbxw13	0	Ercc6	0
Dgka	0	Epn3	0	Bin3	0	Ercc4	0
Dgcr2	0	Epn1	0	Ccdc40	0	Erbb4	0
Dgcr14	0	Epm2aip1	0	B230216G23Rik	0	Erbb3	0
Dgat2l6	0	Ephx3	0	Klre1	0	Erbb2ip	0
Dgat2	0	Ephx2	0	Ptgfrn	0	Eral1	0
Dgat1	0	Ephx1	0	Troap	0	Eqtn	0
Dfna5	0	Ephb6	0	Bcl2a1d	0	Epyc	0
Dffb	0	Ephb2	0	Tbxas1	0	Ept1	0
Dffa	0	Epha7	0	Lage3	0	Epsti1	0
Dexi	0	Epha6	0	Kif14	0	Eps8l3	0
Det1	0	Epha4	0	Evi5	0	Eps8l1	0
Desi2	0	Epha2	0	Scgb1c1	0	Eps8	0
Desi1	0	Epha10	0	Prl2c1	0	Eps15l1	0
Des	0	Epha1	0	Sebox	0	Eprs	0
Derl2	0	Epgn	0	Trim43c	0	Epor	0
Derl1	0	Epg5	0	9930012K11Rik	0	Epo	0
Dera	0	Epcam	0	Fbxw28	0	Epn3	0
Deptor	0	Epb4.2	0	Pmepa1	0	Epn2	0
Depdc7	0	Epb4.1l3	0	Sptlc1	0	Epn1	0
Depdc5	0	Epb4.1l2	0	Adcyap1r1	0	Epm2a	0
Depdc1b	0	Epb4.1l1	0	Nppb	0	Ephx3	0
Depdc1a	0	Epb4.1	0	Nppc	0	Ephx2	0
Denr	0	Epas1	0	Olfr683	0	Ephx1	0
Dennd6b	0	Ep400	0	Pou3f3	0	Ephb4	0
Dennd6a	0	Ep300	0	Pou3f2	0	Ephb2	0
Dennd5b	0	Eomes	0	Pou3f1	0	Epha6	0
Dennd5a	0	Eogt	0	Bcl2a1b	0	Epha2	0
Dennd4b	0	Eny2	0	Kif15	0	Epha1	0
Dennd4a	0	Entpd7	0	Pou3f4	0	Epg5	0
Dennd3	0	Entpd6	0	Olfr681	0	Epcam	0
Dennd2d	0	Entpd5	0	2310003H01Rik	0	Epb4.2	0
Dennd2a	0	Entpd4	0	Samm50	0	Epb4.1l5	0
Dennd1b	0	Entpd3	0	Tpm3	0	Epb4.1l4a	0
Dennd1a	0	Entpd2	0	Ltb4r2	0	Epb4.1l3	0
Dek	0	Enthd1	0	Vip	0	Epb4.1l2	0
Degs1	0	Ensa	0	Zfp385c	0	Epb4.1	0
Defb9	0	Enpp7	0	Vit	0	Epas1	0
Defb7	0	Enpp6	0	Micu2	0	Ep400	0
Defb6	0	Enpp3	0	Gin1	0	Ep300	0
Defb5	0	Enpp2	0	Fbxw21	0	Eny2	0
Defb48	0	Enpp1	0	Gm15308	0	Entpd8	0
Defb47	0	Enpep	0	Osbpl2	0	Entpd7	0
Defb46	0	Enoph1	0	Glra4	0	Entpd6	0
Defb43	0	Eno3	0	Fbxw20	0	Entpd5	0
Defb42	0	Eno2	0	Tsen34	0	Entpd4	0
Defb40	0	Enkur	0	Rps27rt	0	Entpd3	0
Defb39	0	Enho	0	Htr3a	0	Entpd2	0
Defb38	0	Engase	0	Bhmt2	0	Enthd2	0
Defb37	0	Eng	0	Mdh1b	0	Enthd1	0
Defb35	0	Endov	0	Brms1	0	Ensa	0
Defb34	0	Endou	0	Olfr688	0	Enpp7	0
Defb33	0	Endog	0	Htr2c	0	Enpp6	0
Defb30	0	Enc1	0	Brwd3	0	Enpp5	0
Defb3	0	Enah	0	Hcls1	0	Enpp3	0
Defb29	0	En2	0	Zfp637	0	Enpp2	0
Defb25	0	En1	0	Fbxw24	0	Enpp1	0
Defb23	0	Emx1	0	Ednrb	0	Enpep	0
Defb22	0	Emp3	0	Kif17	0	Enox2	0
Defb20	0	Emp1	0	Bdkrb2	0	Enox1	0
Defb19	0	Eml6	0	Bard1	0	Enoph1	0
Defb18	0	Eml5	0	Pdia2	0	Eno4	0
Defb14	0	Eml4	0	Emc9	0	Enkur	0
Defb13	0	Eml3	0	Atp12a	0	Enho	0
Defb11	0	Emilin3	0	Mks1	0	Eng	0
Defb10	0	Emilin2	0	Tsfm	0	Endov	0
Defb1	0	Emg1	0	Sfxn4	0	Endou	0
Defa-rs7	0	Eme1	0	Bsnd	0	Endog	0
Defa-rs1	0	Emc7	0	Gm12794	0	Endod1	0
Defa5	0	Emc6	0	Sfxn3	0	Enc1	0

Defa26	0	Emc4	0	Olfr692	0	Enam	0
Defa25	0	Emc2	0	Rrp12	0	En2	0
Defa22	0	Emc10	0	Nabp1	0	En1	0
Defa21	0	Emb	0	1700123I01Rik	0	Emx2	0
Defa20	0	Elp6	0	Sh3kbp1	0	Emx1	0
Defa2	0	Elp5	0	Rrp15	0	Emp3	0
Def8	0	Elp3	0	Rpl22l1	0	Emp1	0
Def6	0	Elovl4	0	Clns1a	0	Eml5	0
Dedd2	0	Elovl3	0	Prune	0	Eml4	0
Dedd	0	Elovl2	0	Il3	0	Eml3	0
Decr2	0	Elovl1	0	St6gal1	0	Eml1	0
Decr1	0	Elof1	0	Sms	0	Emilin3	0
Deb1	0	Elmsan1	0	Crisp2	0	Emilin2	0
Deaf1	0	Elmod2	0	Fuz	0	Emid1	0
Ddx6	0	Elmod1	0	Usp12	0	Emg1	0
Ddx59	0	Elmo3	0	Olfr170	0	Eme2	0
Ddx58	0	Elmo2	0	Usp11	0	Eme1	0
Ddx56	0	Elmo1	0	Usp16	0	Emd	0
Ddx55	0	Ell3	0	Dsp	0	Emc7	0
Ddx54	0	Elk4	0	Usp14	0	Emc6	0
Ddx52	0	Elk3	0	Usp15	0	Emc4	0
Ddx51	0	Elfn2	0	Kat6b	0	Emc2	0
Ddx50	0	Elfn1	0	Usp18	0	Emc1	0
Ddx5	0	Elf3	0	Mettl10	0	Elp6	0
Ddx49	0	Elf2	0	Bcdin3d	0	Elp5	0
Ddx47	0	Elavl4	0	Sema6c	0	Elp3	0
Ddx46	0	Elavl3	0	1810043G02Rik	0	Elp2	0
Ddx43	0	Elavl2	0	Prpsap1	0	Elovl5	0
Ddx42	0	Elavl1	0	Calb2	0	Elovl1	0
Ddx41	0	Elane	0	Prpsap2	0	Elof1	0
Ddx4	0	Elac2	0	Rrp1b	0	Eln	0
Ddx3y	0	Eif6	0	Emc7	0	Elmod3	0
Ddx39b	0	Eif5b	0	Sptb	0	Elmod2	0
Ddx39	0	Eif5a	0	Fscn2	0	Elmod1	0
Ddx31	0	Eif5	0	Ganc	0	Elmo2	0
Ddx28	0	Eif4g3	0	Olfr694	0	Elmo1	0
Ddx27	0	Eif4g2	0	BRDN0000737797	0	Ell3	0
Ddx25	0	Eif4ebp3	0	Fscn1	0	Ell2	0
Ddx24	0	Eif4ebp2	0	Kat6a	0	Ell	0
Ddx23	0	Eif4ebp1	0	Hmgb2	0	Elk3	0
Ddx21	0	Eif4e3	0	Emc4	0	Elfn1	0
Ddx20	0	Eif4e	0	Fam185a	0	Elf4	0
Ddx19b	0	Eif4b	0	BRDN0000737624	0	Elf3	0
Ddx19a	0	Eif4a3	0	Fam167b	0	Elf2	0
Ddx18	0	Eif4a2	0	Fam167a	0	Elf1	0
Ddx17	0	Eif4a1	0	Synj2	0	Elavl2	0
Ddx11	0	Eif3m	0	Napepld	0	Elavl1	0
Ddx10	0	Eif3l	0	Scgb1b19	0	Elane	0
Ddx1	0	Eif3k	0	Sparcl1	0	Elac2	0
Ddt	0	Eif3j2	0	Cilp	0	Elac1	0
Ddrgk1	0	Eif3i	0	Ibtk	0	Eif6	0
Ddr2	0	Eif3h	0	Mrgprb8	0	Eif5b	0
Ddr1	0	Eif3g	0	Ramp3	0	Eif5a2	0
Ddost	0	Eif3f	0	Gmpr2	0	Eif5a	0
Ddo	0	Eif3d	0	Hmgb4	0	Eif5	0
Ddn	0	Eif3c	0	Slc4a1ap	0	Eif4h	0
Ddit4l	0	Eif3b	0	Pxylp1	0	Eif4g3	0
Ddit4	0	Eif3a	0	Syt12	0	Eif4g2	0
Ddit3	0	Eif2s3x	0	Dse	0	Eif4ebp3	0
Ddias	0	Eif2s2	0	Adamts10	0	Eif4ebp1	0
Ddi2	0	Eif2s1	0	Syk	0	Eif4e1b	0
Ddi1	0	Eif2d	0	Hmga2	0	Eif4a3	0
Ddhd2	0	Eif2b5	0	Syt10	0	Eif4a2	0
Ddhd1	0	Eif2b4	0	Hmga1	0	Eif4a1	0
Ddb2	0	Eif2b3	0	Acnat1	0	Eif3m	0
Ddb1	0	Eif2b2	0	Tnik	0	Eif3l	0
Ddah2	0	Eif2b1	0	BC017158	0	Eif3k	0
Dda1	0	Eif2ak4	0	Syt16	0	Eif3j2	0
Dcxr	0	Eif2ak3	0	Havcr1	0	Eif3i	0
Dcx	0	Eif2a	0	Havcr2	0	Eif3g	0
Dcun1d5	0	Eif1b	0	Syt17	0	Eif3e	0
Dcun1d4	0	Eif1ax	0	Reln	0	Eif3d	0
Dcun1d3	0	Eif1ad	0	Acnat2	0	Eif3c	0
Dcun1d2	0	Eif1a	0	B020031M17Rik	0	Eif3b	0
Dcun1d1	0	Eid2	0	Syt14	0	Eif3a	0
Dctpp1	0	Eid1	0	Oard1	0	Eif2s1	0
Dctn6	0	Ei24	0	Tyro3	0	Eif2d	0
Dctn5	0	Ehhadh	0	Slc5a7	0	Eif2b4	0
Dctn3	0	Ehf	0	Frs3	0	Eif2b3	0
Dctn2	0	Ehd4	0	Frs2	0	Eif2b2	0
Dctn1	0	Ehd3	0	Ice2	0	Eif2b1	0
Dctd	0	Ehd2	0	Ice1	0	Eif2ak4	0

Dct	0	Ehd1	0	Pdrg1	0	Eif2ak3	0
Dcst1	0	Ehbp1l1	0	Slc5a9	0	Eif2a	0
Dcps	0	Ehbp1	0	Clvs1	0	Eif1ad	0
Dcpp3	0	Egr4	0	Rad51ap1	0	Eif1a	0
Dcpp1	0	Egr2	0	Sp100	0	Eid2b	0
Dcp2	0	Egln3	0	Kcns1	0	Eid2	0
Dcp1a	0	Egln2	0	Nckap1l	0	Eid1	0
Dcn	0	Egln1	0	Napsa	0	Ei24	0
Dclre1c	0	Egfr	0	Cd48	0	Ehmt2	0
Dclre1a	0	Egflam	0	BRDN0000737600	0	Ehmt1	0
Dclk3	0	Egfl6	0	Cd44	0	Ehhadh	0
Dclk2	0	Eftud2	0	Cd47	0	Ehf	0
Dclk1	0	Eftud1	0	Cd46	0	Ehd4	0
Dchs1	0	Efs	0	Rap1b	0	Ehd2	0
Dcdc2b	0	Efr3b	0	Rap1a	0	Ehbp1l1	0
Dcdc2a	0	Efnb3	0	L3mbtl1	0	Ehbp1	0
Dcbld2	0	Efnb2	0	Tex19.1	0	Egr4	0
Dcakd	0	Efhd2	0	L3mbtl3	0	Egr3	0
Dcaf7	0	Efhd1	0	Satb2	0	Egln2	0
Dcaf6	0	Efhc2	0	Satb1	0	Egln1	0
Dcaf5	0	Efhc1	0	Ocln	0	Egflam	0
Dcaf4	0	Efhb	0	BRDN0000737830	0	Egfl8	0
Dcaf17	0	Efemp2	0	Ep300	0	Egfl7	0
Dcaf13	0	Efemp1	0	Gprasp2	0	Egfem1	0
Dcaf12l2	0	Efcc1	0	Tex33	0	Eftud2	0
Dcaf12l1	0	Efcab6	0	Gprasp1	0	Eftud1	0
Dcaf11	0	Efcab2	0	Ncoa2	0	Efr3b	0
Dbx2	0	Efcab12	0	Ncoa3	0	Efnb3	0
Dbx1	0	Efcab10	0	Rgr	0	Efnb1	0
Dbt	0	Eepd1	0	Cyp2b9	0	Efna5	0
Dbr1	0	Eefsec	0	Ncoa6	0	Efna3	0
Dbpht2	0	Eef2kmt	0	Ncoa7	0	Efna2	0
Dbp	0	Eef2k	0	Ncoa4	0	Efna1	0
Dbnl	0	Eef2	0	Ncoa5	0	Efhd2	0
Dbndd1	0	Eef1g	0	Nefl	0	Efhc2	0
Dbn1	0	Eef1e1	0	Prg4	0	Efhb	0
Dbil5	0	Eef1d	0	Nefh	0	Efemp1	0
Dbi	0	Eef1b2	0	Pigt	0	Efcc1	0
Dbf4	0	Eef1a2	0	Rev1	0	Efcab9	0
Dazl	0	Eef1a1	0	Chrd	0	Efcab6	0
Dazap2	0	Eed	0	Dnajb5	0	Efcab3	0
Dazap1	0	Eea1	0	BRDN0000737831	0	Efcab2	0
Daxx	0	Edrf1	0	Ucp1	0	Efcab14	0
Dars2	0	Ednra	0	Atf6b	0	Efcab12	0
Dars	0	Edil3	0	Rassf5	0	Efcab11	0
Dapp1	0	Edf1	0	4833420G17Rik	0	Efcab10	0
Dapl1	0	Edem2	0	Bik	0	Efcab1	0
Dapk2	0	Edc3	0	Pip5k1a	0	Eepd1	0
Dap3	0	Edar	0	Ppp4r1	0	Eefsec	0
Dap	0	Eda2r	0	Ppp4r2	0	Eef2kmt	0
Dao	0	Eda	0	Dnlz	0	Eef2	0
Dand5	0	Ect2	0	Ccng2	0	Eef1g	0
Dalrd3	0	Ecsit	0	BRDN0000737958	0	Eef1e1	0
Daglb	0	Ecscr	0	Armc8	0	Eef1d	0
Dag1	0	Ecm2	0	Armc9	0	Eef1b2	0
Daf2	0	Eci3	0	Armc6	0	Eef1a2	0
Dad1	0	Echdc3	0	Armc7	0	Eef1a1	0
Dact2	0	Echdc2	0	Armc4	0	Eea1	0
Dact1	0	Echdc1	0	Armc5	0	Edrf1	0
Dach2	0	Ech1	0	Armc2	0	Ednrb	0
Dach1	0	Ecel1	0	Gm3604	0	Edn3	0
Dab2ip	0	Ecd	0	Prss2	0	Edn2	0
Dab2	0	Ebp	0	Olfr624	0	Edn1	0
Daam2	0	Ebna1bp2	0	Dctd	0	Edil3	0
Daam1	0	Ebi3	0	Olfr620	0	Edf1	0
D930020B18Rik	0	Ebf3	0	Olfr622	0	Edem3	0
D930015E06Rik	0	Ebf2	0	Olfr623	0	Edem2	0
D8Ertd738e	0	Ebf1	0	BRDN0000738356	0	Edem1	0
D830030K20Rik	0	Ear6	0	BRDN0000738357	0	Eddm3b	0
D7Ertd443e	0	Ear2	0	BRDN0000738354	0	Edc4	0
D730048I06Rik	0	Ear1	0	Bcap29	0	Edaradd	0
D6Wsu163e	0	Eaf1	0	Olfr628	0	Edar	0
D6Ertd527e	0	E430025E21Rik	0	Olfr629	0	Eda2r	0
D630045J12Rik	0	E330017A01Rik	0	Rell2	0	Eda	0
D630039A03Rik	0	E330014E10Rik	0	Lynx1	0	Ect2	0
D630033O11Rik	0	E2f8	0	Nox1	0	Ecsit	0
D630023F18Rik	0	E2f6	0	BRDN0000737510	0	Ecm2	0
D5Ertd579e	0	E2f4	0	Nox3	0	Eci3	0
D5Ertd577e	0	E2f1	0	BRDN0000737512	0	Eci2	0
D430042O09Rik	0	E230025N22Rik	0	BRDN0000737515	0	Echdc3	0
D430019H16Rik	0	E130311K13Rik	0	Nox4	0	Echdc1	0
D3Ertd751e	0	E130308A19Rik	0	BRDN0000737517	0	Ecel1	0

D3Ertd254e	0
D330045A20Rik	0
D2Wsu81e	0
D230025D16Rik	0
D1Pas1	0
D1Ertd622e	0
D19Bwg1357e	0
D17Wsu92e	0
D17H6S53E	0
D16Ertd472e	0
D130043K22Rik	0
D130040H23Rik	0
D11Wsu47e	0
D10Wsu102e	0
D10Jhu81e	0
Cyyr1	0
Cytl1	0
Cytip	0
Cyth4	0
Cyth3	0
Cyth2	0
Cyth1	0
Cystm1	0
Cysrt1	0
Cysltr2	0
Cysltr1	0
Cyr61	0
Cypt4	0
Cypt3	0
Cypt2	0
Cypt15	0
Cypt12	0
Cypt1	0
Cyp8b1	0
Cyp7b1	0
Cyp7a1	0
Cyp51	0
Cyp4x1	0
Cyp4f40	0
Cyp4f39	0
Cyp4f37	0
Cyp4f18	0
Cyp4f17	0
Cyp4f16	0
Cyp4f15	0
Cyp4f14	0
Cyp4b1	0
Cyp4a32	0
Cyp4a31	0
Cyp4a29	0
Cyp4a14	0
Cyp4a12b	0
Cyp4a12a	0
Cyp4a10	0
Cyp46a1	0
Cyp3a59	0
Cyp3a57	0
Cyp3a44	0
Cyp3a41b	0
Cyp3a41a	0
Cyp3a16	0
Cyp3a13	0
Cyp2u1	0
Cyp2t4	0
Cyp2s1	0
Cyp2r1	0
Cyp2j9	0
Cyp2j8	0
Cyp2j6	0
Cyp2j5	0
Cyp2j13	0
Cyp2j11	0
Cyp2g1	0
Cyp2f2	0
Cyp2e1	0
Cyp2d40	0
Cyp2d34	0
Cyp2d26	0
Cyp2d22	0
Cyp2d11	0
Cyp2d10	0
Cyp2c70	0
E030018B13Rik	0
E030002O03Rik	0
Dzip1l	0
Dzank1	0
Dytn	0
Dysf	0
Dyrk3	0
Dyrk2	0
Dyrk1a	0
Dynlt1f	0
Dynlt1c	0
Dynlrb2	0
Dynlrb1	0
Dynll2	0
Dynll1	0
Dync1li1	0
Dync1i2	0
Dync1i1	0
Dync1h1	0
Dynap	0
Dvl3	0
Dvl1	0
Dut	0
Dusp9	0
Dusp6	0
Dusp4	0
Dusp3	0
Dusp28	0
Dusp27	0
Dusp26	0
Dusp23	0
Dusp22	0
Dusp21	0
Dusp2	0
Dusp19	0
Dusp18	0
Dusp15	0
Dusp14	0
Dusp12	0
Dus3l	0
Dus2	0
Dus1l	0
Dupd1	0
Duoxa2	0
Duox2	0
Duox1	0
Dtymk	0
Dtx4	0
Dtx3l	0
Dtx1	0
Dtwd2	0
Dtwd1	0
Dtnbp1	0
Dtnb	0
Dthd1	0
Dtd2	0
Dtd1	0
Dstyk	0
Dstn	0
Dst	0
Dspp	0
Dsn1	0
Dsg4	0
Dsg3	0
Dsg1c	0
Dsg1b	0
Dsg1a	0
Dse	0
Dsc3	0
Dsc1	0
Drp2	0
Drd2	0
Drc7	0
Draxin	0
Dram1	0
Dr1	0
Dqx1	0
Dpysl4	0
Dpysl3	0
Dpyd	0
Dpy30	0
Dpy19l3	0
BRDN0000737516	0
Gm20917	0
BRDN0000737518	0
Krtap2-4	0
Apoa5	0
Tmco5b	0
Wdr73	0
Tbl1xr1	0
Gfy	0
Akr1c13	0
1700007K13Rik	0
Chic2	0
Snip1	0
Wrap73	0
BRDN0000738177	0
Clec3a	0
Vmn2r58	0
Tex24	0
Zfp191	0
Tex26	0
Nelfb	0
Tex21	0
Tex22	0
Cdcp2	0
Tex28	0
Tex29	0
Eppin	0
Gsk3a	0
4922502D21Rik	0
Gsk3b	0
Vars2	0
Alcam	0
Prkab2	0
Dmwd	0
Inf2	0
Hmces	0
Olfr1037	0
Olfr1036	0
Olfr1034	0
Sphkap	0
Olfr1031	0
Isca2	0
Sdc1	0
Sdc2	0
Ehbp1	0
Sdc4	0
Olfr1039	0
Slc19a3	0
Mapk14	0
Mapk15	0
Agap3	0
Mapk11	0
Mapk12	0
Mapk13	0
Wfs1	0
Vps13a	0
Magt1	0
Clec2i	0
Gabrb3	0
Camk1g	0
Zfp26	0
Slc15a5	0
4933402D24Rik	0
Mgea5	0
Hnrnpul2	0
Hnrnpul1	0
Ddx17	0
Lgals12	0
Naa38	0
Gdf5	0
Gpbp1l1	0
Naa35	0
Rnase2b	0
Naa30	0
Snx4	0
Mettl21e	0
Dnah8	0
Mettl21c	0
Mettl21b	0
Mettl21a	0
Calcoco1	0
Apoa4	0
Ece1	0
Ecd	0
Ebpl	0
Ebp	0
Ebna1bp2	0
Ebi3	0
Ebf3	0
Ebf2	0
Ear6	0
Ear2	0
Ear10	0
Ear1	0
Eapp	0
Eaf1	0
E4f1	0
E430025E21Rik	0
E430018J23Rik	0
E330034G19Rik	0
E330021D16Rik	0
E330014E10Rik	0
E2f8	0
E2f7	0
E2f6	0
E2f5	0
E2f4	0
E2f3	0
E2f1	0
E230025N22Rik	0
E230019M04Rik	0
E230008N13Rik	0
E130309D14Rik	0
E130309D02Rik	0
E130012A19Rik	0
E030018B13Rik	0
Dzip3	0
Dyx1c1	0
Dyrk4	0
Dyrk3	0
Dyrk2	0
Dyrk1a	0
Dynlt3	0
Dynlt1f	0
Dynlt1c	0
Dynlt1b	0
Dynlt1a	0
Dynlrb2	0
Dynlrb1	0
Dync2h1	0
Dync1li2	0
Dync1i2	0
Dync1h1	0
Dydc1	0
Dxo	0
Dvl3	0
Dvl1	0
Dut	0
Dusp9	0
Dusp8	0
Dusp5	0
Dusp3	0
Dusp28	0
Dusp27	0
Dusp26	0
Dusp23	0
Dusp22	0
Dusp2	0
Dusp19	0
Dusp18	0
Dusp16	0
Dusp15	0
Dusp14	0
Dusp12	0
Dusp11	0
Dusp1	0
Dus3l	0
Dus2	0
Dus1l	0
Dupd1	0
Duox2	0
Dtymk	0
Dtx4	0
Dtx3l	0

Cyp2c68	0
Cyp2c67	0
Cyp2c66	0
Cyp2c65	0
Cyp2c55	0
Cyp2c50	0
Cyp2c44	0
Cyp2c38	0
Cyp2c29	0
Cyp2b9	0
Cyp2b23	0
Cyp2b13	0
Cyp2b10	0
Cyp2a5	0
Cyp2a4	0
Cyp2a22	0
Cyp2a12	0
Cyp26c1	0
Cyp21a1	0
Cyp1b1	0
Cyp1a2	0
Cyp1a1	0
Cyp17a1	0
Cyp11b2	0
Cyp11b1	0
Cyp11a1	0
Cym	0
Cyld	0
Cylc2	0
Cylc1	0
Cyhr1	0
Cyfip2	0
Cyfip1	0
Cyct	0
Cyc1	0
Cybrd1	0
Cybb	0
Cyb5r4	0
Cyb5r3	0
Cyb5r2	0
Cyb5r1	0
Cyb5d2	0
Cyb5d1	0
Cyb5b	0
Cyb5a	0
Cyb561d1	0
Cyb561	0
Cxxc5	0
Cxxc4	0
Cxxc1	0
Cxx1b	0
Cxx1a	0
Cxcr6	0
Cxcr5	0
Cxcr4	0
Cxcr3	0
Cxcr2	0
Cxcr1	0
Cxcl9	0
Cxcl5	0
Cxcl2	0
Cxcl15	0
Cxcl14	0
Cxcl13	0
Cxcl10	0
Cxcl1	0
Cxadr	0
Cx3cr1	0
Cx3cl1	0
Cwf19l2	0
Cwc27	0
Cwc25	0
Cwc22	0
Cwc15	0
Cuzd1	0
Cux2	0
Cux1	0
Cutc	0
Cuta	0
Cul9	0
Cul5	0
Cul4b	0
Dpy19l2	0
Dpy19l1	0
Dpt	0
Dppa5a	0
Dppa4	0
Dppa3	0
Dppa2	0
Dppa1	0
Dpp9	0
Dpp8	0
Dpp6	0
Dpp10	0
Dpm3	0
Dpm2	0
Dpm1	0
Dph7	0
Dph5	0
Dph3	0
Dph2	0
Dph1	0
Dpf3	0
Dpf2	0
Dpf1	0
Dpep3	0
Dpep2	0
Dpep1	0
Dpagt1	0
Doxl2	0
Dot1l	0
Dopey2	0
Dolpp1	0
Dolk	0
Dok6	0
Dok5	0
Dok4	0
Dok1	0
Dohh	0
Dock9	0
Dock5	0
Dock4	0
Dock3	0
Dock10	0
Doc2g	0
Doc2a	0
Dnttip1	0
Dnpep	0
Dnmt3b	0
Dnmt3a	0
Dnmt1	0
Dnm2	0
Dnm1l	0
Dnm1	0
Dner	0
Dnd1	0
Dnase2b	0
Dnase1l3	0
Dnase1l2	0
Dnase1l1	0
Dnase1	0
Dnal4	0
Dnal1	0
Dnajc9	0
Dnajc8	0
Dnajc6	0
Dnajc5g	0
Dnajc5b	0
Dnajc5	0
Dnajc4	0
Dnajc28	0
Dnajc25	0
Dnajc24	0
Dnajc22	0
Dnajc21	0
Dnajc2	0
Dnajc19	0
Dnajc17	0
Dnajc16	0
Dnajc15	0
Dnajc14	0
Dnajc12	0
Dnajc11	0
Dnajc10	0
Aldh5a1	0
Trip12	0
Trip13	0
Apoa1	0
Cr2	0
Adam7	0
Adam4	0
Manbal	0
Adam2	0
Rhcg	0
Pde1b	0
Ubp1	0
Aif1l	0
Olfr262	0
Adam9	0
Serpina1b	0
Serpina1c	0
Ripply3	0
Serpina1a	0
Aatf	0
Clec2g	0
Serpina1d	0
Serpina1e	0
Sval2	0
Pdp1	0
Mkx	0
Kank4	0
Fahd2a	0
BRDN0000737537	0
Sds	0
Ncf1	0
BRDN0000738235	0
Zfp827	0
Lrcol1	0
Ap2m1	0
Snrnp25	0
Tomm34	0
Snrnp27	0
2310011J03Rik	0
BRDN0000737794	0
Tstd2	0
AB124611	0
Serpina12	0
Serpina10	0
Serpina11	0
Tmem17	0
Orm3	0
Tmem18	0
Shisa8	0
Mab21l1	0
Itpa	0
Bhlha9	0
Mab21l2	0
Gcat	0
Crk	0
Crh	0
Cited1	0
Bmp8a	0
Orai2	0
Orai3	0
Cela3a	0
Orai1	0
Bmp8b	0
Zfp322a	0
Dnajc5b	0
Kirrel	0
BRDN0000737654	0
Methig1	0
Dnajc5g	0
Ypel4	0
Pdgfrl	0
Mta2	0
Bdp1	0
Ttc24	0
Cox7a1	0
Ttc26	0
Cited4	0
Ap1ar	0
Ttc22	0
Arl5b	0
Ufl1	0
Znhit2	0
Dtwd2	0
Dtwd1	0
Dtnbp1	0
Dtnb	0
Dthd1	0
Dtd2	0
Dtd1	0
Dstyk	0
Dst	0
Dspp	0
Dsp	0
Dsg4	0
Dsg3	0
Dsg1c	0
Dsg1b	0
Dsg1a	0
Dsel	0
Dse	0
Dscaml1	0
Dscam	0
Dsc3	0
Dsc2	0
Dsc1	0
Drp2	0
Drosha	0
Drg2	0
Drg1	0
Drd1	0
Draxin	0
Drap1	0
Dram2	0
Dram1	0
Dqx1	0
Dpysl5	0
Dpysl2	0
Dpy19l2	0
Dpy19l1	0
Dppa5a	0
Dppa4	0
Dppa2	0
Dpp9	0
Dpp6	0
Dpp10	0
Dpm3	0
Dpm2	0
Dpm1	0
Dph7	0
Dph6	0
Dph3	0
Dph2	0
Dph1	0
Dpf3	0
Dpep3	0
Dpep1	0
Dpcr1	0
Dpagt1	0
Doxl2	0
Dot1l	0
Dopey1	0
Donson	0
Dolk	0
Dok6	0
Dok5	0
Dok4	0
Dok3	0
Dok2	0
Dok1	0
Dohh	0
Dock9	0
Dock8	0
Dock7	0
Dock3	0
Dock11	0
Dock1	0
Doc2g	0
Dnttip2	0
Dntt	0
Dnph1	0
Dnmt3l	0
Dnmt3b	0
Dnmt3a	0
Dnm3	0

Cul4a	0
Cul3	0
Cul2	0
Cul1	0
Cuedc1	0
Ctxn3	0
Ctxn2	0
Ctxn1	0
Ctu2	0
Ctu1	0
Cttnbp2	0
Cttn	0
Ctsz	0
Ctsw	0
Ctss	0
Ctsq	0
Ctso	0
Ctsm	0
Ctsll3	0
Ctsk	0
Ctsf	0
Ctse	0
Ctsc	0
Ctsb	0
Ctsa	0
Cts8	0
Cts7	0
Cts6	0
Cts3	0
Ctrl	0
Ctrc	0
Ctrb1	0
Ctr9	0
Ctps2	0
Ctps	0
Ctns	0
Ctnnd2	0
Ctnnbl1	0
Ctnnbip1	0
Ctnnb1	0
Ctnnal1	0
Ctnna3	0
Ctnna2	0
Ctnna1	0
Ctla4	0
Ctif	0
Cthrc1	0
Cth	0
Ctgf	0
Ctf1	0
Ctdsp2	0
Ctdp1	0
Ctdnep1	0
Ctcfl	0
Ctcf	0
Ctc1	0
Ctbs	0
Ctbp1	0
Ctage5	0
Ctag2	0
Cstl1	0
Cstf3	0
Cstf2t	0
Cstf1	0
Cstb	0
Cstad	0
Csta1	0
Cst7	0
Cst6	0
Cst3	0
Cst13	0
Cst12	0
Cst11	0
Cst10	0
Csrp3	0
Csrp2bp	0
Csrp2	0
Csrp1	0
Csrnp2	0
Csrnp1	0
Cspp1	0
Cspg5	0
Dnajc1	0
Dnajb9	0
Dnajb7	0
Dnajb4	0
Dnajb3	0
Dnajb2	0
Dnajb11	0
Dnajb1	0
Dnaja3	0
Dnaja2	0
Dnaic2	0
Dnaic1	0
Dnah7b	0
Dnah7a	0
Dnah6	0
Dnah17	0
Dnah10	0
Dnah1	0
Dnaaf5	0
Dnaaf3	0
Dnaaf2	0
Dnaaf1	0
Dna2	0
Dmxl1	0
Dmwd	0
Dmtn	0
Dmtf1	0
Dmrtc2	0
Dmrtc1b	0
Dmrtc1a	0
Dmrtb1	0
Dmrt2	0
Dmrt1	0
Dmpk	0
Dmp1	0
Dmkn	0
Dmd	0
Dmc1	0
Dmbx1	0
Dmbt1	0
Dmap1	0
Dlx5	0
Dlx3	0
Dlx2	0
Dlx1	0
Dll4	0
Dll3	0
Dll1	0
Dlk2	0
Dlgap5	0
Dlgap3	0
Dlgap2	0
Dlgap1	0
Dlg4	0
Dlg2	0
Dlg1	0
Dld	0
Dkkl1	0
Dkk4	0
Dkk3	0
Dkk2	0
Dkc1	0
Disp2	0
Disp1	0
Disc1	0
Dis3l2	0
Dis3l	0
Dis3	0
Dirc2	0
Diras2	0
Diras1	0
Dip2c	0
Dio3	0
Dio1	0
Dimt1	0
Diexf	0
Dicer1	0
Diap3	0
Diap2	0
Diap1	0
Diablo	0
Dhx58	0
Gm11559	0
Znhit6	0
Tnfsfm13	0
Cks2	0
Hacd4	0
Pcdh12	0
Olfr1275	0
Pcdh10	0
4930579F01Rik	0
Pcdh17	0
Ascc2	0
Cyr61	0
Pcdh18	0
Pcdh19	0
Npm1	0
Olfr1277	0
Glp2r	0
Ehd2	0
Kansl3	0
Olfr1276	0
Ehd1	0
Kif24	0
Gm5458	0
Alox15	0
Kif23	0
Apitd1	0
Zfp277	0
Arid5b	0
Atmin	0
Rsbn1l	0
Adipoq	0
Atp6v0a1	0
Bcl2l15	0
Nadk	0
Papd4	0
Lce1g	0
Kctd18	0
Lrp12	0
Pvrl2	0
BRDN0000737793	0
Men1	0
Mapk1ip1l	0
Diap3	0
Cwh43	0
A230046K03Rik	0
Gprin2	0
Diap2	0
Tmem222	0
Tnfrsf14	0
Tmem220	0
Tmem221	0
Nabp2	0
Olfr735	0
BRDN0000737692	0
Zfp462	0
Lce1d	0
Zfp467	0
Gng7	0
Gm16532	0
Stmnd1	0
Nsf	0
Atp2b4	0
Spns1	0
Egfl6	0
Spns3	0
Spns2	0
Clec4b1	0
Kif2a	0
Xpo5	0
Kif2c	0
Fggy	0
Pogk	0
Plxnb2	0
Gabarapl1	0
Slc35a2	0
Aire	0
Gabarapl2	0
BRDN0000737695	0
Megf10	0
Prr15	0
Saraf	0
Pogz	0
Dnm2	0
Dnm1l	0
Dnm1	0
Dnlz	0
Dner	0
Dnase2b	0
Dnase1l3	0
Dnase1	0
Dnal4	0
Dnal1	0
Dnajc9	0
Dnajc8	0
Dnajc7	0
Dnajc6	0
Dnajc5g	0
Dnajc5b	0
Dnajc5	0
Dnajc3	0
Dnajc28	0
Dnajc24	0
Dnajc22	0
Dnajc2	0
Dnajc19	0
Dnajc18	0
Dnajc17	0
Dnajc15	0
Dnajc14	0
Dnajc13	0
Dnajc12	0
Dnajc11	0
Dnajc10	0
Dnajc1	0
Dnajb9	0
Dnajb8	0
Dnajb7	0
Dnajb6	0
Dnajb4	0
Dnajb3	0
Dnajb14	0
Dnajb12	0
Dnajb11	0
Dnajb1	0
Dnaja4	0
Dnaja3	0
Dnaja2	0
Dnaic2	0
Dnaic1	0
Dnah7b	0
Dnah7a	0
Dnah5	0
Dnah2	0
Dnah17	0
Dnah1	0
Dnaaf5	0
Dnaaf2	0
Dnaaf1	0
Dna2	0
Dmxl2	0
Dmxl1	0
Dmtn	0
Dmtf1	0
Dmrtc2	0
Dmrtb1	0
Dmrta2	0
Dmrta1	0
Dmrt3	0
Dmrt2	0
Dmrt1	0
Dmkn	0
Dmgdh	0
Dmd	0
Dmc1	0
Dmbx1	0
Dmap1	0
Dlx5	0
Dlx4	0
Dll3	0
Dlk2	0
Dlgap5	0
Dlgap2	0
Dlg5	0
Dlg4	0

Cspg4	0	Dhx57	0	Khnyn	0	Dlg2	0
Csnka2ip	0	Dhx40	0	Olfr395	0	Dlg1	0
Csnk2b	0	Dhx38	0	BRDN0000737699	0	Dleu7	0
Csnk2a2	0	Dhx37	0	Ube2j1	0	Dlec1	0
Csnk2a1	0	Dhx36	0	Ube2j2	0	Dlat	0
Csnk1g3	0	Dhx34	0	Irak1bp1	0	Dkkl1	0
Csnk1g1	0	Dhx33	0	Oas1e	0	Dkk3	0
Csnk1e	0	Dhx29	0	Krtap9-5	0	Dkk2	0
Csnk1d	0	Dhx16	0	Ccdc66	0	Dkk1	0
Csn3	0	Dhx15	0	Ly9	0	Dkc1	0
Csn2	0	Dhrs7	0	Ech1	0	Disp2	0
Csmd3	0	Dhrs2	0	Mipep	0	Disp1	0
Csmd1	0	Dhrs13	0	Medag	0	Disc1	0
Csl	0	Dhrs11	0	Dpy30	0	Dis3l2	0
Csgalnact2	0	Dhrs1	0	Xpo7	0	Dis3l	0
Csgalnact1	0	Dhps	0	Olfr730	0	Dis3	0
Csf3r	0	Dhodh	0	Plin2	0	Dirc2	0
Csf2rb2	0	Dhh	0	Oprl1	0	Diras2	0
Csf2rb	0	Dhfr	0	Gm5415	0	Diras1	0
Csf2ra	0	Dhdds	0	BRDN0000737999	0	Dip2a	0
Csf2	0	Dhcr7	0	Sat1	0	Dio3	0
Csf1r	0	Dgkz	0	Sat2	0	Dio2	0
Csf1	0	Dgkq	0	Atg16l1	0	Dio1	0
Cse1l	0	Dgkh	0	Atg16l2	0	Dimt1	0
Csde1	0	Dgke	0	Plscr4	0	Diexf	0
Csdc2	0	Dgkb	0	Pgm5	0	Dido1	0
Cs	0	Dgka	0	Tcl1b2	0	Diap3	0
Crym	0	Dgcr8	0	Vmn1r19	0	Diap2	0
Cryl1	0	Dgcr14	0	Mylpf	0	Diap1	0
Crygs	0	Dgat2l6	0	1700061G19Rik	0	Dhx8	0
Crygn	0	Dgat1	0	Plscr2	0	Dhx58	0
Crygf	0	Dfna5	0	Lfng	0	Dhx57	0
Crygc	0	Dffa	0	Entpd2	0	Dhx40	0
Crygb	0	Dexi	0	Grm8	0	Dhx38	0
Cryga	0	Desi2	0	Gng3	0	Dhx37	0
Crybb3	0	Desi1	0	Ccl21b	0	Dhx36	0
Crybb1	0	Derl3	0	Sult4a1	0	Dhx35	0
Cryba4	0	Derl2	0	Gm17660	0	Dhx33	0
Cryba1	0	Derl1	0	Hesx1	0	Dhx30	0
Cryab	0	Dera	0	Pcyt2	0	Dhx29	0
Cryaa	0	Deptor	0	1700001K19Rik	0	Dhx16	0
Cry2	0	Depdc5	0	Plek	0	Dhx15	0
Cry1	0	Denr	0	BRDN0000737744	0	Dhtkd1	0
Crxos	0	Dennd6b	0	Tmem143	0	Dhrs7c	0
Crx	0	Dennd4a	0	BRDN0000737746	0	Dhrs7	0
Crtc2	0	Dennd3	0	Mtfp1	0	Dhrs3	0
Crtc1	0	Dennd2d	0	Defb18	0	Dhrs2	0
Crtap	0	Dennd2c	0	Oxsr1	0	Dhrs13	0
Crtam	0	Dennd2a	0	Ly6c2	0	Dhrs1	0
Crtac1	0	Dennd1c	0	Vmn1r114	0	Dhps	0
Crp	0	Dennd1a	0	Lyn	0	Dhh	0
Crocc	0	Dek	0	Olfr432	0	Dhfr	0
Crnn	0	Degs2	0	Olfr435	0	Dhdh	0
Crnkl1	0	Defb9	0	BRDN0000737749	0	Dhdds	0
Crmp1	0	Defb8	0	Olfr437	0	Dhcr7	0
Crls1	0	Defb7	0	Defb13	0	Dgkz	0
Crlf3	0	Defb6	0	Gm94	0	Dgkq	0
Crlf2	0	Defb50	0	Olfr1484	0	Dgki	0
Crlf1	0	Defb48	0	Vmn1r11	0	Dgke	0
Crkl	0	Defb47	0	Olfr1480	0	Dgkb	0
Crk	0	Defb46	0	Mycn	0	Dgka	0
Crispld2	0	Defb45	0	Gal3st4	0	Dgcr8	0
Crispld1	0	Defb43	0	Kif7	0	Dgcr2	0
Crisp4	0	Defb42	0	Kif4	0	Dgcr14	0
Crisp3	0	Defb40	0	Ncdn	0	Dgat2l6	0
Crisp2	0	Defb4	0	Pde7b	0	Dgat2	0
Crisp1	0	Defb39	0	Mns1	0	Dfna5	0
Cript	0	Defb38	0	Olfr1489	0	Dexi	0
Crip3	0	Defb37	0	Vmn1r14	0	Det1	0
Crip2	0	Defb36	0	Zfand1	0	Desi1	0
Crim1	0	Defb35	0	Prkg2	0	Des	0
Crhr1	0	Defb30	0	Figf	0	Derl3	0
Crhbp	0	Defb3	0	Vmn1r15	0	Derl2	0
Crh	0	Defb29	0	Timm8b	0	Derl1	0
Crem	0	Defb28	0	Tead4	0	Dera	0
Creld2	0	Defb26	0	BRDN0000738033	0	Deptor	0
Creg2	0	Defb23	0	Fam19a4	0	Depdc7	0
Creg1	0	Defb22	0	Fam19a5	0	Depdc1a	0
Crebzf	0	Defb21	0	Fam19a2	0	Denr	0
Crebl2	0	Defb20	0	Acyp2	0	Dennd6a	0
Crebbp	0	Defb2	0	BRDN0000738299	0	Dennd5a	0
Creb3l4	0	Defb19	0	BRDN0000738298	0	Dennd4c	0

Creb3l2	0	Defb18	0	Hr	0	Dennd4b	0
Creb1	0	Defb15	0	BRDN0000738296	0	Dennd4a	0
Crct1	0	Defb13	0	Hp	0	Dennd3	0
Crcp	0	Defb12	0	Uimc1	0	Dennd2d	0
Crb3	0	Defb1	0	BRDN0000738293	0	Dennd2c	0
Crb2	0	Defa-rs7	0	BRDN0000738031	0	Dennd2a	0
Crb1	0	Defa-rs1	0	BRDN0000738291	0	Dennd1c	0
Cramp1l	0	Defa5	0	BRDN0000738290	0	Degs2	0
Cradd	0	Defa26	0	Pet117	0	Degs1	0
Cracr2b	0	Defa25	0	BRDN0000737747	0	Defb8	0
Cracr2a	0	Defa21	0	Lifr	0	Defb6	0
Crabp2	0	Defa20	0	Mtnr1a	0	Defb50	0
Crabp1	0	Defa2	0	Heph	0	Defb5	0
Cpz	0	Def8	0	Olfr741	0	Defb47	0
Cpxm2	0	Def6	0	Spata25	0	Defb45	0
Cpxm1	0	Decr2	0	Gm4498	0	Defb42	0
Cpxcr1	0	Decr1	0	Amotl1	0	Defb40	0
Cpvl	0	Deb1	0	4930486L24Rik	0	Defb4	0
Cptp	0	Deaf1	0	Amotl2	0	Defb39	0
Cpt1c	0	Ddx6	0	Lmbr1l	0	Defb38	0
Cpt1b	0	Ddx59	0	BRDN0000738035	0	Defb37	0
Cpt1a	0	Ddx58	0	Olig2	0	Defb36	0
Cpsf7	0	Ddx55	0	Fam20a	0	Defb35	0
Cpsf6	0	Ddx54	0	Fam20b	0	Defb34	0
Cpsf4l	0	Ddx52	0	Muc4	0	Defb33	0
Cpsf4	0	Ddx51	0	Defb14	0	Defb28	0
Cpsf3l	0	Ddx50	0	Bambi	0	Defb25	0
Cpsf3	0	Ddx5	0	Olfr430	0	Defb22	0
Cpsf2	0	Ddx49	0	Tgm2	0	Defb21	0
Cpsf1	0	Ddx47	0	Tmem132d	0	Defb20	0
Cps1	0	Ddx46	0	Sugp2	0	Defb2	0
Cpq	0	Ddx42	0	Olfr433	0	Defb19	0
Cpped1	0	Ddx41	0	Tmem132c	0	Defb15	0
Cpox	0	Ddx3y	0	Tmem132b	0	Defb14	0
Cpne9	0	Ddx3x	0	S100a14	0	Defb12	0
Cpne8	0	Ddx39b	0	Vmn1r117	0	Defa-rs7	0
Cpne6	0	Ddx39	0	Spag8	0	Defa-rs1	0
Cpne4	0	Ddx31	0	Spag9	0	Defa5	0
Cpne3	0	Ddx28	0	Spag6	0	Defa22	0
Cpne2	0	Ddx27	0	Spag7	0	Defa21	0
Cpne1	0	Ddx25	0	Spag4	0	Defa20	0
Cpn2	0	Ddx24	0	Spag5	0	Defa2	0
Cplx4	0	Ddx23	0	Foxr1	0	Def8	0
Cplx3	0	Ddx21	0	Spag1	0	Dedd	0
Cplx2	0	Ddx20	0	Wdsub1	0	Decr1	0
Cped1	0	Ddx19b	0	Pkp4	0	Deb1	0
Cpeb3	0	Ddx19a	0	Smarcd3	0	Deaf1	0
Cpeb1	0	Ddx17	0	Vmn1r112	0	Ddx6	0
Cpe	0	Ddx11	0	Smarcd1	0	Ddx59	0
Cpd	0	Ddx10	0	Ncapg	0	Ddx56	0
Cpb2	0	Ddx1	0	Faim	0	Ddx55	0
Cpb1	0	Ddt	0	Gm608	0	Ddx54	0
Cpa6	0	Ddrgk1	0	Cd3g	0	Ddx51	0
Cpa5	0	Ddr2	0	BRDN0000737753	0	Ddx5	0
Cpa4	0	Ddost	0	Nol4l	0	Ddx49	0
Cpa3	0	Ddo	0	Prh1	0	Ddx47	0
Cpa2	0	Ddn	0	Fat2	0	Ddx46	0
Cpa1	0	Ddit4l	0	Plac1	0	Ddx42	0
Cp	0	Ddit4	0	Cabs1	0	Ddx4	0
Cox8c	0	Ddit3	0	Ehbp1l1	0	Ddx3y	0
Cox8b	0	Ddias	0	Olfr1487	0	Ddx3x	0
Cox8a	0	Ddi2	0	Fzd8	0	Ddx39b	0
Cox7c	0	Ddi1	0	Blzf1	0	Ddx31	0
Cox7b	0	Ddhd2	0	Tsks	0	Ddx28	0
Cox7a2l	0	Ddhd1	0	Rock1	0	Ddx27	0
Cox7a1	0	Ddc	0	Rock2	0	Ddx25	0
Cox6c	0	Ddb2	0	Gpc1	0	Ddx24	0
Cox6b2	0	Ddah2	0	Gpc2	0	Ddx23	0
Cox6b1	0	Ddah1	0	Olfr612	0	Ddx21	0
Cox5b	0	Dda1	0	Gpc4	0	Ddx19b	0
Cox5a	0	Dcxr	0	Gpc5	0	Ddx19a	0
Cox4i1	0	Dcx	0	Gpc6	0	Ddx18	0
Cox20	0	Dcun1d5	0	Cysrt1	0	Ddx17	0
Cox19	0	Dcun1d4	0	Olfr615	0	Ddx11	0
Cox17	0	Dcun1d3	0	BRDN0000737529	0	Ddx1	0
Cox16	0	Dcun1d2	0	Klrc1	0	Ddt	0
Cox14	0	Dcun1d1	0	Tgfb1	0	Ddrgk1	0
Cox11	0	Dctpp1	0	Klrc3	0	Ddr2	0
Cox10	0	Dctn6	0	Nsdhl	0	Ddr1	0
Cort	0	Dctn5	0	Bbs1	0	Ddost	0
Coro7	0	Dctn4	0	Mycs	0	Ddit4	0
Coro6	0	Dctn3	0	Kif9	0	Ddit3	0

Coro2b	0	Dctn2	0	Bbs5	0	Ddi1	0
Coro2a	0	Dctd	0	Bbs4	0	Ddhd2	0
Coro1c	0	Dcst1	0	Bbs7	0	Ddc	0
Coro1a	0	Dcps	0	Cd19	0	Ddb2	0
Coq9	0	Dcpp3	0	Tigd3	0	Ddb1	0
Coq4	0	Dcpp1	0	Tigd2	0	Ddah2	0
Coq3	0	Dcp2	0	Tigd5	0	Ddah1	0
Coq2	0	Dcp1b	0	Olfr618	0	Dda1	0
Coq10b	0	Dclre1c	0	Nr1h4	0	Dcxr	0
Coq10a	0	Dclre1a	0	Nr1h5	0	Dcun1d5	0
Copz1	0	Dclk3	0	Fn3k	0	Dcun1d4	0
Cops8	0	Dclk2	0	Repin1	0	Dcun1d1	0
Cops7a	0	Dcdc2c	0	BRDN0000737526	0	Dctn6	0
Cops6	0	Dcdc2b	0	Man1a2	0	Dctn4	0
Cops5	0	Dcdc2a	0	Aadat	0	Dctn3	0
Cops4	0	Dcc	0	BRDN0000738346	0	Dctn2	0
Cops3	0	Dcbld2	0	Snx8	0	Dctn1	0
Cops2	0	Dcbld1	0	BRDN0000738341	0	Dctd	0
Coprs	0	Dcakd	0	Znfx1	0	Dct	0
Copg1	0	Dcaf7	0	H6pd	0	Dcstamp	0
Copb2	0	Dcaf6	0	Camk2a	0	Dcst1	0
Copb1	0	Dcaf5	0	Acbd3	0	Dcps	0
Copa	0	Dcaf17	0	Acbd7	0	Dcpp3	0
Comp	0	Dcaf15	0	Acbd6	0	Dcpp2	0
Commd8	0	Dcaf13	0	Acbd5	0	Dcpp1	0
Commd7	0	Dcaf12l2	0	Acbd4	0	Dcp2	0
Commd6	0	Dcaf12	0	Best3	0	Dcp1b	0
Commd5	0	Dcaf10	0	Best2	0	Dcp1a	0
Commd4	0	Dbx2	0	Best1	0	Dcn	0
Commd3	0	Dbt	0	Hbs1l	0	Dclre1c	0
Commd10	0	Dbr1	0	Zmym2	0	Dclre1b	0
Commd1	0	Dbpht2	0	Zmym3	0	Dclre1a	0
Colq	0	Dbnl	0	Fam160a2	0	Dclk3	0
Colgalt2	0	Dbndd1	0	Zmym6	0	Dchs1	0
Colec12	0	Dbil5	0	Zmym4	0	Dcdc2c	0
Colec11	0	Dbi	0	Zmym5	0	Dcdc2b	0
Colec10	0	Dbh	0	Rslcan18	0	Dcakd	0
Col9a3	0	Dbf4	0	Ampd2	0	Dcaf7	0
Col9a2	0	Dazl	0	Ampd1	0	Dcaf5	0
Col8a2	0	Dazap2	0	Gm3488	0	Dcaf4	0
Col8a1	0	Daxx	0	Top1	0	Dcaf15	0
Col7a1	0	Dars2	0	Rala	0	Dcaf11	0
Col6a6	0	Dars	0	Cylc1	0	Dcaf10	0
Col6a5	0	Dapl1	0	Cylc2	0	Dbx1	0
Col6a4	0	Dapk3	0	Ccdc146	0	Dbr1	0
Col6a3	0	Dapk1	0	Ccdc141	0	Dbpht2	0
Col5a3	0	Dap3	0	Ccdc142	0	Dbnl	0
Col5a2	0	Dand5	0	Gpc3	0	Dbndd2	0
Col5a1	0	Daglb	0	Prkg1	0	Dbil5	0
Col4a5	0	Daf2	0	Nrf1	0	Dbh	0
Col4a4	0	Dad1	0	Ccdc149	0	Dazl	0
Col4a3bp	0	Dact3	0	Dpm2	0	Dazap2	0
Col4a3	0	Dact1	0	Dpm1	0	Dazap1	0
Col4a2	0	Dach2	0	6430571L13Rik	0	Daxx	0
Col4a1	0	Dach1	0	Zfp617	0	Dars2	0
Col3a1	0	Dab2	0	Zfp616	0	Dars	0
Col2a1	0	Dab1	0	Zfp612	0	Dapp1	0
Col27a1	0	Daam2	0	Twsg1	0	Dapk3	0
Col26a1	0	Daam1	0	Lypla1	0	Dapk1	0
Col25a1	0	D930020B18Rik	0	Lypla2	0	Dap	0
Col24a1	0	D930015E06Rik	0	Zfp618	0	Dand5	0
Col22a1	0	D8Ertd82e	0	Polr3f	0	Dalrd3	0
Col20a1	0	D8Ertd738e	0	Polr3g	0	Dag1	0
Col1a2	0	D830030K20Rik	0	Polr3d	0	Daf2	0
Col1a1	0	D730048I06Rik	0	Polr3e	0	Dad1	0
Col19a1	0	D6Wsu163e	0	Polr3b	0	Dact2	0
Col17a1	0	D6Ertd527e	0	Polr3c	0	Dact1	0
Col16a1	0	D630045J12Rik	0	Polr3a	0	Dach2	0
Col14a1	0	D630033O11Rik	0	Cd99l2	0	Dach1	0
Col12a1	0	D630023F18Rik	0	Polr3k	0	Dab2	0
Col11a2	0	D630003M21Rik	0	Polr3h	0	Dab1	0
Col11a1	0	D430042O09Rik	0	Gm10436	0	Daam2	0
Cog8	0	D430019H16Rik	0	Enox1	0	Daam1	0
Cog7	0	D2Wsu81e	0	Olfr980	0	D930020B18Rik	0
Cog5	0	D2hgdh	0	Olfr983	0	D930015E06Rik	0
Cog4	0	D1Pas1	0	Khdc1b	0	D8Ertd82e	0
Cog3	0	D19Bwg1357e	0	Olfr986	0	D8Ertd738e	0
Cog2	0	D17H6S53E	0	Olfr988	0	D830030K20Rik	0
Cog1	0	D16Ertd472e	0	Atp10b	0	D7Ertd443e	0
Coch	0	D130043K22Rik	0	Nr2f6	0	D730048I06Rik	0
Cobll1	0	D130040H23Rik	0	Tmem200a	0	D6Wsu163e	0
Cobl	0	D11Wsu47e	0	Tmem255b	0	D630045J12Rik	0

Coa6	0	D10Wsu102e	0	Tmem200c	0	D630039A03Rik	0
Coa5	0	D10Jhu81e	0	Tmem200b	0	D630023F18Rik	0
Coa4	0	D030056L22Rik	0	Tenm3	0	D630003M21Rik	0
Coa3	0	Cyyr1	0	Tenm2	0	D5Ertd577e	0
Cntrob	0	Cytip	0	Tenm1	0	D430042O09Rik	0
Cntnap5c	0	Cyth4	0	Mios	0	D3Ertd751e	0
Cntnap5b	0	Cyth2	0	Cc2d1a	0	D2hgdh	0
Cntnap5a	0	Cyth1	0	Tex16	0	D1Ertd622e	0
Cntnap4	0	Cystm1	0	Stard10	0	D19Bwg1357e	0
Cntnap2	0	Cysrt1	0	Cpsf3l	0	D17Wsu92e	0
Cntnap1	0	Cysltr2	0	Luzp2	0	D17H6S53E	0
Cntn4	0	Cysltr1	0	Cdkl5	0	D15Ertd621e	0
Cntn3	0	Cys1	0	Cdkl4	0	D130043K22Rik	0
Cntn1	0	Cyr61	0	Cc2d1b	0	D10Wsu102e	0
Cntfr	0	Cypt4	0	Cdkl1	0	D10Jhu81e	0
Cntf	0	Cypt3	0	Xkr7	0	Cyyr1	0
Cntd1	0	Cypt14	0	Cdkl3	0	Cytl1	0
Cnrip1	0	Cypt1	0	Cdkl2	0	Cytip	0
Cnpy4	0	Cyp8b1	0	Olfr76	0	Cyth3	0
Cnpy2	0	Cyp7a1	0	Usp39	0	Cyth1	0
Cnppd1	0	Cyp4x1	0	Tpsg1	0	Cysrt1	0
Cnot8	0	Cyp4v3	0	Adra1b	0	Cysltr1	0
Cnot7	0	Cyp4f40	0	Olfr559	0	Cyr61	0
Cnot6l	0	Cyp4f18	0	Olfr73	0	Cypt3	0
Cnot6	0	Cyp4f17	0	Glt8d1	0	Cypt2	0
Cnot4	0	Cyp4f16	0	Olfr71	0	Cypt15	0
Cnot2	0	Cyp4f14	0	Usp30	0	Cypt12	0
Cnot10	0	Cyp4f13	0	Zfp65	0	Cypt1	0
Cnot1	0	Cyp4b1	0	Usp32	0	Cyp8b1	0
Cnnm3	0	Cyp4a30b	0	Usp33	0	Cyp7a1	0
Cnnm2	0	Cyp4a12a	0	Usp34	0	Cyp51	0
Cnnm1	0	Cyp4a10	0	Usp35	0	Cyp4v3	0
Cnn3	0	Cyp3a59	0	Usp36	0	Cyp4f40	0
Cnn2	0	Cyp3a44	0	Usp37	0	Cyp4f39	0
Cnksr3	0	Cyp3a41b	0	Nkx1-2	0	Cyp4f37	0
Cnksr2	0	Cyp3a41a	0	Fbxo21	0	Cyp4f17	0
Cnih4	0	Cyp3a25	0	Gm14474	0	Cyp4f16	0
Cnih2	0	Cyp3a16	0	Oas1f	0	Cyp4f15	0
Cnih1	0	Cyp3a13	0	Fam19a1	0	Cyp4f14	0
Cngb3	0	Cyp3a11	0	Dhps	0	Cyp4f13	0
Cngb1	0	Cyp39a1	0	Timp3	0	Cyp4b1	0
Cnga3	0	Cyp2w1	0	Ppp1r15a	0	Cyp4a32	0
Cnga2	0	Cyp2u1	0	Cubn	0	Cyp4a31	0
Cnga1	0	Cyp2t4	0	Ppp1r15b	0	Cyp4a30b	0
Cnfn	0	Cyp2s1	0	Tex10	0	Cyp4a29	0
Cnep1r1	0	Cyp2r1	0	Fam187a	0	Cyp4a14	0
Cndp2	0	Cyp2j9	0	Fam187b	0	Cyp4a10	0
Cndp1	0	Cyp2j6	0	Olfr263	0	Cyp3a59	0
Cnbd2	0	Cyp2j5	0	BRDN0000738295	0	Cyp3a57	0
Cmya5	0	Cyp2j13	0	Prss21	0	Cyp3a41b	0
Cmtr2	0	Cyp2j12	0	Tsn	0	Cyp3a41a	0
Cmtr1	0	Cyp2j11	0	Afap1l1	0	Cyp3a25	0
Cmtm4	0	Cyp2g1	0	Cyp1b1	0	Cyp3a16	0
Cmtm3	0	Cyp2f2	0	Rasl11b	0	Cyp3a13	0
Cmtm2b	0	Cyp2e1	0	Rasl11a	0	Cyp3a11	0
Cmtm2a	0	Cyp2d40	0	Lace1	0	Cyp39a1	0
Cmtm1	0	Cyp2d34	0	Xkr8	0	Cyp2w1	0
Cmss1	0	Cyp2d26	0	Ssh2	0	Cyp2t4	0
Cmpk2	0	Cyp2c70	0	Ssh3	0	Cyp2s1	0
Cmpk1	0	Cyp2c69	0	Tsx	0	Cyp2r1	0
Cml5	0	Cyp2c68	0	Ssh1	0	Cyp2j9	0
Cml2	0	Cyp2c67	0	Ctbp1	0	Cyp2j6	0
Cml1	0	Cyp2c66	0	Ctbp2	0	Cyp2j5	0
Cmklr1	0	Cyp2c40	0	Hemk1	0	Cyp2j13	0
Cmc2	0	Cyp2c39	0	6330408A02Rik	0	Cyp2g1	0
Cmc1	0	Cyp2c37	0	Cinp	0	Cyp2f2	0
Cmbl	0	Cyp2c29	0	E130309D14Rik	0	Cyp2e1	0
Cmas	0	Cyp2b9	0	Nfx1	0	Cyp2d40	0
Cma2	0	Cyp2b10	0	Cts3	0	Cyp2d34	0
Clybl	0	Cyp2a5	0	Rdh8	0	Cyp2d26	0
Clvs1	0	Cyp2a22	0	Rdh9	0	Cyp2d12	0
Cluap1	0	Cyp26c1	0	Akap2	0	Cyp2d11	0
Clu	0	Cyp26b1	0	Rdh7	0	Cyp2c70	0
Clta	0	Cyp26a1	0	Dao	0	Cyp2c68	0
Clstn3	0	Cyp21a1	0	Rdh1	0	Cyp2c67	0
Clstn2	0	Cyp20a1	0	4933402P03Rik	0	Cyp2c55	0
Clstn1	0	Cyp1b1	0	A4gnt	0	Cyp2c44	0
Clspn	0	Cyp1a2	0	Dap	0	Cyp2c39	0
Clrn2	0	Cyp19a1	0	Coa4	0	Cyp2c38	0
Clrn1	0	Cyp17a1	0	Alyref2	0	Cyp2c37	0
Clptm1l	0	Cyp11b2	0	Olfr611	0	Cyp2c29	0
Clpsl2	0	Cym	0	BRDN0000737857	0	Cyp2b9	0

Clps	0	Cyld	0	S100a7a	0	Cyp2b23	0
Clpp	0	Cylc2	0	Cic	0	Cyp2b13	0
Clpb	0	Cygb	0	Kctd11	0	Cyp2b10	0
Clp1	0	Cyfip2	0	Zgrf1	0	Cyp27b1	0
Clns1a	0	Cyfip1	0	Kctd16	0	Cyp26b1	0
Clnk	0	Cyct	0	Kctd14	0	Cyp24a1	0
Cln8	0	Cybrd1	0	Hmgcl	0	Cyp21a1	0
Cln5	0	Cyba	0	Nap1l1	0	Cyp20a1	0
Clmp	0	Cyb5r2	0	Kctd19	0	Cyp1b1	0
Clmn	0	Cyb5d2	0	Plrg1	0	Cyp19a1	0
Clk2	0	Cyb5d1	0	Nap1l5	0	Cyp11b1	0
Clk1	0	Cyb5b	0	Adgrd1	0	Cyp11a1	0
Clip3	0	Cyb5a	0	Gmds	0	Cym	0
Clip2	0	Cyb561d2	0	Wbp11	0	Cylc2	0
Clint1	0	Cyb561d1	0	Hmgcr	0	Cyhr1	0
Clic6	0	Cyb561	0	Fgf8	0	Cyfip2	0
Clic5	0	Cxxc4	0	Fgf7	0	Cyfip1	0
Clic4	0	Cxxc1	0	Rnf170	0	Cyct	0
Clic3	0	Cxcr5	0	Fgf5	0	Cycs	0
Clic1	0	Cxcr3	0	Fgf4	0	Cyc1	0
Clhc1	0	Cxcr1	0	Fgf3	0	Cybrd1	0
Clgn	0	Cxcl9	0	Gipc1	0	Cyba	0
Clec7a	0	Cxcl5	0	Fgf1	0	Cyb5r3	0
Clec4g	0	Cxcl3	0	Asna1	0	Cyb5r2	0
Clec4f	0	Cxcl15	0	Smco3	0	Cyb5d2	0
Clec4e	0	Cxcl13	0	Smco2	0	Cyb5d1	0
Clec4d	0	Cxcl12	0	Smco1	0	Cyb561d2	0
Clec4b2	0	Cxcl10	0	Cd28	0	Cyb561d1	0
Clec4b1	0	Cxcl1	0	Spred3	0	Cyb561a3	0
Clec4a4	0	Cwh43	0	Serpina5	0	Cxxc5	0
Clec4a2	0	Cwf19l2	0	Serpina6	0	Cxxc1	0
Clec4a1	0	Cwc22	0	Serpina7	0	Cxx1b	0
Clec3a	0	Cwc15	0	Cd22	0	Cxcr4	0
Clec2l	0	Cuzd1	0	Ankrd13a	0	Cxcr3	0
Clec2h	0	Cux2	0	Cd27	0	Cxcr2	0
Clec2e	0	Cux1	0	Setdb1	0	Cxcr1	0
Clec2d	0	Cutc	0	Olfr434	0	Cxcl9	0
Clec1a	0	Cutal	0	Ugt1a5	0	Cxcl5	0
Clec14a	0	Cul5	0	Zfp180	0	Cxcl2	0
Clec12b	0	Cul4b	0	Satl1	0	Cxcl16	0
Clec11a	0	Cul4a	0	Ugt1a1	0	Cxcl15	0
Cldnd2	0	Cul3	0	Ugt1a2	0	Cxcl13	0
Cldnd1	0	Cul2	0	Dpysl4	0	Cxcl12	0
Cldn9	0	Cul1	0	Tmem52b	0	Cxcl1	0
Cldn8	0	Cuedc1	0	Dpysl2	0	Cxadr	0
Cldn6	0	Ctxn3	0	Apol11b	0	Cwf19l2	0
Cldn5	0	Ctxn2	0	Apol11a	0	Cwf19l1	0
Cldn4	0	Ctxn1	0	BRDN0000738194	0	Cwc25	0
Cldn24	0	Ctu2	0	Arl6ip6	0	Cwc22	0
Cldn22	0	Ctu1	0	Palb2	0	Cuzd1	0
Cldn20	0	Cttnbp2nl	0	Arl6ip4	0	Cux2	0
Cldn19	0	Cttnbp2	0	Dab2ip	0	Cutc	0
Cldn18	0	Cttn	0	BRDN0000738191	0	Cuta	0
Cldn17	0	Ctsz	0	Tfpi2	0	Cul5	0
Cldn16	0	Ctss	0	Slc35b1	0	Cul4b	0
Cldn15	0	Ctsr	0	Fchsd1	0	Cul3	0
Cldn14	0	Ctsll3	0	Fchsd2	0	Cul2	0
Cldn13	0	Ctsl	0	Slc35b2	0	Cul1	0
Cldn12	0	Ctsk	0	BRDN0000737685	0	Cuedc1	0
Cldn11	0	Ctsh	0	BRDN0000737686	0	Ctxn3	0
Cldn10	0	Ctsf	0	BRDN0000737689	0	Ctxn1	0
Clcnkb	0	Ctsc	0	BRDN0000737688	0	Ctu2	0
Clcn7	0	Cts8	0	Gosr1	0	Ctu1	0
Clcn6	0	Cts6	0	Chpf	0	Cttnbp2nl	0
Clcn5	0	Cts3	0	Actb	0	Cttnbp2	0
Clcn2	0	Ctrl	0	BRDN0000737548	0	Ctsz	0
Clcn1	0	Ctrb1	0	Taf5l	0	Ctsll3	0
Clcf1	0	Ctr9	0	Otoa	0	Ctsk	0
Clcc1	0	Ctps2	0	Olfr666	0	Ctsh	0
Clca4b	0	Ctps	0	Psmb10	0	Ctsg	0
Clca3b	0	Ctns	0	Sqrdl	0	Ctsf	0
Clca3a2	0	Ctnnd2	0	Tmprss11d	0	Ctsd	0
Clca2	0	Ctnnbl1	0	H2-T22	0	Ctsc	0
Clca1	0	Ctnnbip1	0	H2-T24	0	Cts8	0
Clasrp	0	Ctnnb1	0	Ept1	0	Cts7	0
Clasp2	0	Ctnna2	0	Tmprss11e	0	Cts6	0
Cks2	0	Ctnna1	0	BRDN0000737447	0	Ctrl	0
Cks1b	0	Ctla2a	0	Lyz2	0	Ctrb1	0
Ckmt2	0	Ctif	0	Lyz1	0	Ctps	0
Cklf	0	Cth	0	Fpgt	0	Ctns	0
Ckap5	0	Ctf2	0	Fpgs	0	Ctnnbl1	0
Ckap4	0	Ctf1	0	Peg10	0	Ctnnbip1	0

Ckap2l	0
Ckap2	0
CK137956	0
Cited4	0
Cited2	0
Cited1	0
Cish	0
Cisd3	0
Cisd2	0
Cisd1	0
Cirh1a	0
Cirbp	0
Cir1	0
Cipc	0
Cilp2	0
Cilp	0
Ciita	0
Cidec	0
Cidea	0
Cic	0
Cib4	0
Cib3	0
Cib2	0
Ciart	0
Ciapin1	0
Churc1	0
Chuk	0
Chtf8	0
Chtf18	0
Chsy3	0
Chsy1	0
Chst9	0
Chst8	0
Chst4	0
Chst3	0
Chst2	0
Chst15	0
Chst14	0
Chst12	0
Chst11	0
Chst10	0
Chrng	0
Chrne	0
Chrnd	0
Chrnb4	0
Chrnb3	0
Chrnb2	0
Chrnb1	0
Chrna9	0
Chrna7	0
Chrna6	0
Chrna4	0
Chrna3	0
Chrna2	0
Chrna1	0
Chrm5	0
Chrm4	0
Chrm3	0
Chrm2	0
Chrm1	0
Chrdl2	0
Chrdl1	0
Chrd	0
Chpt1	0
Chpf2	0
Chp1	0
Chordc1	0
Chodl	0
Chn2	0
Chmp7	0
Chmp5	0
Chmp4b	0
Chmp2b	0
Chmp2a	0
Chmp1a	0
Chml	0
Chm	0
Chl1	0
Chkb	0
Chit1	0
Chil6	0
Chil4	0
Ctdspl	0
Ctdsp1	0
Ctdp1	0
Ctdnep1	0
Ctcfl	0
Ctcf	0
Ctc1	0
Ctbp2	0
Ctage5	0
Cstl1	0
Cstf2t	0
Cstf1	0
Cstb	0
Cstad	0
Csta1	0
Cst9	0
Cst8	0
Cst6	0
Cst3	0
Cst13	0
Csrp2bp	0
Csrnp2	0
Csrnp1	0
Cspg5	0
Cspg4	0
Csnka2ip	0
Csnk2b	0
Csnk2a1	0
Csnk1g3	0
Csnk1g1	0
Csnk1e	0
Csnk1d	0
Csnk1a1	0
Csn3	0
Csn1s2b	0
Csn1s1	0
Csmd3	0
Csmd1	0
Csl	0
Csgalnact2	0
Csgalnact1	0
Csf3	0
Csf2rb2	0
Csf2rb	0
Csf2	0
Csf1	0
Cse1l	0
Csde1	0
Csdc2	0
Csad	0
Cs	0
Cryzl1	0
Crym	0
Cryl1	0
Crygs	0
Crygn	0
Crygf	0
Cryge	0
Crygd	0
Crygc	0
Crygb	0
Cryga	0
Crybb1	0
Cryba2	0
Cryba1	0
Cryaa	0
Cry1	0
Crxos	0
Crx	0
Crtc3	0
Crtc1	0
Crtap	0
Crp	0
Crot	0
Crocc	0
Crnkl1	0
Crmp1	0
Crls1	0
Crlf2	0
Crlf1	0
Crkl	0
Crk	0
Dennd5a	0
Tctn3	0
Ppp1r9a	0
Usp46	0
Tmprss11g	0
Angpt4	0
Ccnb1ip1	0
BRDN0000738378	0
BRDN0000737539	0
Agl	0
B230219D22Rik	0
Olfr608	0
Layn	0
BRDN0000738371	0
Mgam	0
Olfr605	0
Chp1	0
Olfr603	0
Aga	0
Chp2	0
Smim3	0
Gm10057	0
Vcpkmt	0
Ptprcap	0
Tsen2	0
Sprr1a	0
Sprr1b	0
Agt	0
Rab25	0
Prdx6b	0
Pgm2l1	0
Pcyt1b	0
Mtmr11	0
Myef2	0
Vmn2r101	0
Cpa2	0
Calcr	0
Cpa1	0
Cpa6	0
Vapb	0
Vapa	0
Fbxo28	0
Tex43	0
Tex40	0
Vmn2r103	0
Clec1a	0
Clec1b	0
Rab29	0
Synpo2l	0
Calca	0
Calcb	0
Gadd45g	0
Dgkz	0
Pus1	0
Pus3	0
Pus7	0
Dgkq	0
Olfr616	0
Csad	0
Vmn2r113	0
Vmn2r108	0
Vmn2r111	0
Dgki	0
Bmpr2	0
Olfr1018	0
Vmn2r115	0
Vmn2r114	0
Olfr1014	0
Vmn2r118	0
Dgkg	0
Dgkd	0
Dgke	0
Lst1	0
Gm13278	0
Ogt	0
Lama5	0
Cntnap3	0
Tomm70a	0
Timm8a2	0
Fut4	0
Pwwp2b	0
Pwwp2a	0
Ctnnb1	0
Ctnnal1	0
Ctnna3	0
Ctnna2	0
Ctnna1	0
Ctla2b	0
Ctla2a	0
Ctif	0
Cth	0
Ctgf	0
Ctf2	0
Ctf1	0
Ctdspl2	0
Ctdp1	0
Ctcf	0
Ctc1	0
Ctbs	0
Ctbp2	0
Ctbp1	0
Ctage5	0
Ctag2	0
Cstl1	0
Cstf3	0
Cstf2t	0
Cstf2	0
Cstf1	0
Cstb	0
Cstad	0
Csta1	0
Cst8	0
Cst7	0
Cst6	0
Cst13	0
Cst12	0
Csrp2bp	0
Csrp2	0
Csrp1	0
Csrnp2	0
Cspg5	0
Cspg4	0
Csnk2b	0
Csnk2a1	0
Csnk1g3	0
Csnk1g1	0
Csnk1d	0
Csn3	0
Csn1s2b	0
Csn1s1	0
Csmd1	0
Csgalnact2	0
Csgalnact1	0
Csf3r	0
Csf3	0
Csf2rb	0
Csf2	0
Csf1r	0
Csf1	0
Cse1l	0
Csde1	0
Csdc2	0
Cs	0
Cryz	0
Cryl1	0
Cryge	0
Crygd	0
Crygc	0
Crygb	0
Cryga	0
Crybb3	0
Cryba2	0
Cryba1	0
Cryab	0
Cryaa	0
Cry2	0
Cry1	0
Crx	0
Crtc2	0
Crtap	0
Crtac1	0
Crp	0
Crot	0
Crocc	0

Chil3	0
Chil1	0
Chic1	0
Chia1	0
Chgb	0
Chga	0
Cherp	0
Chek1	0
Chdh	0
Chd8	0
Chd7	0
Chd6	0
Chd5	0
Chd2	0
Chd1	0
Chchd6	0
Chchd5	0
Chchd4	0
Chchd3	0
Chchd2	0
Chchd10	0
Chchd1	0
Chat	0
Champ1	0
Chaf1b	0
Chaf1a	0
Chadl	0
Chac2	0
Ch25h	0
Cgrrf1	0
Cgref1	0
Cgn	0
Cggbp1	0
Cga	0
Cftr	0
Cfp	0
Cflar	0
Cfi	0
Cfhr2	0
Cfhr1	0
Cfh	0
Cfdp1	0
Cfd	0
Cfc1	0
Cfap97	0
Cfap70	0
Cfap69	0
Cfap58	0
Cfap57	0
Cfap53	0
Cfap52	0
Cfap45	0
Cfap44	0
Cfap36	0
Cfap221	0
Cfap126	0
Cetn4	0
Cetn3	0
Cetn2	0
Ces4a	0
Ces3a	0
Ces2h	0
Ces2g	0
Ces2f	0
Ces2b	0
Ces2a	0
Ces1g	0
Ces1f	0
Ces1e	0
Ces1c	0
Ces1b	0
Cers6	0
Cers4	0
Cers3	0
Cerkl	0
Cerk	0
Cercam	0
Cer1	0
Cep97	0
Cep95	0
Cep89	0
Cep85l	0

Crispld2	0
Crisp4	0
Crisp2	0
Cript	0
Crip3	0
Crhr1	0
Crhbp	0
Crem	0
Creld2	0
Creg1	0
Crebzf	0
Crebrf	0
Crebbp	0
Creb5	0
Creb3l4	0
Creb3l2	0
Creb3l1	0
Creb1	0
Crcp	0
Crbn	0
Crb3	0
Crb2	0
Crb1	0
Crat	0
Cradd	0
Crabp2	0
Crabp1	0
Cr2	0
Cpxm1	0
Cpvl	0
Cptp	0
Cpt2	0
Cpt1c	0
Cpt1b	0
Cpsf7	0
Cpsf6	0
Cpsf4l	0
Cpsf4	0
Cpsf3l	0
Cpsf3	0
Cpsf2	0
Cpsf1	0
Cps1	0
Cpq	0
Cpped1	0
Cpox	0
Cpne9	0
Cpne8	0
Cpne7	0
Cpne6	0
Cpne5	0
Cpne4	0
Cpne3	0
Cpne2	0
Cpne1	0
Cpn2	0
Cpn1	0
Cplx4	0
Cplx3	0
Cplx1	0
Cped1	0
Cpeb2	0
Cpeb1	0
Cpe	0
Cpd	0
Cpb2	0
Cpb1	0
Cpa5	0
Cpa4	0
Cpa3	0
Cpa2	0
Cpa1	0
Cp	0
Cox8c	0
Cox8b	0
Cox8a	0
Cox7c	0
Cox7b2	0
Cox7b	0
Cox7a2l	0
Cox7a2	0
Cox7a1	0

Gan	0
Sugp1	0
Taf11	0
Il7r	0
Dnttip1	0
Dnttip2	0
Cyp11a1	0
Evl	0
Olfr357	0
Fgf6	0
Evc	0
Bicc1	0
Steap1	0
Dynap	0
Nostrin	0
Ubn2	0
Ubn1	0
Rnase10	0
Capn6	0
Iglon5	0
Ecscr	0
Heatr5a	0
Naa50	0
Creb3	0
Adra2a	0
Adra2c	0
2010109A12Rik	0
Impa1	0
Impa2	0
Nmbr	0
Fut9	0
Bhlhe41	0
B4galt1	0
B4galt3	0
Fut8	0
B4galt5	0
Fosb	0
B4galt7	0
B4galt6	0
Rsph3b	0
Ehmt2	0
Trap1	0
Mia	0
Aars	0
Lrrc16a	0
Fam46a	0
Fam46b	0
Mif	0
Pde7a	0
Rhag	0
Fam57a	0
Vmn2r9	0
Vmn2r8	0
Vmn2r7	0
Vmn2r6	0
Vmn2r4	0
Aard	0
Vmn2r2	0
Llph	0
Aggf1	0
Apol10b	0
Zfp229	0
Olfr313	0
Tmem255a	0
Atf7ip2	0
Sfn	0
Elk3	0
Elk1	0
Elk4	0
Igfl3	0
Serac1	0
Prl3c1	0
Ncam1	0
Ncam2	0
Olfr353	0
Ubox5	0
Tank	0
Rap1gap	0
Eif2ak4	0
Tmem35	0
Aar2	0
Tmem37	0

Crnn	0
Crnkl1	0
Crmp1	0
Crls1	0
Crlf3	0
Crlf2	0
Crlf1	0
Crk	0
Crisp4	0
Crisp3	0
Crisp2	0
Crip2	0
Crim1	0
Crhr1	0
Crhbp	0
Crh	0
Crem	0
Creld1	0
Creg1	0
Crebzf	0
Crebrf	0
Crebl2	0
Crebbp	0
Creb3l4	0
Creb3l1	0
Creb3	0
Creb1	0
Crct1	0
Crbn	0
Crb2	0
Cradd	0
Cracr2b	0
Cracr2a	0
Crabp1	0
Cr1l	0
Cpz	0
Cpxm1	0
Cpxcr1	0
Cpvl	0
Cptp	0
Cpt2	0
Cpt1c	0
Cpt1b	0
Cpsf7	0
Cpsf4l	0
Cpsf4	0
Cpsf3l	0
Cpsf3	0
Cpsf2	0
Cpsf1	0
Cps1	0
Cpox	0
Cpne8	0
Cpne6	0
Cpne5	0
Cpne4	0
Cpne3	0
Cpne2	0
Cpn2	0
Cpm	0
Cplx4	0
Cplx2	0
Cpeb4	0
Cpeb3	0
Cpeb1	0
Cpe	0
Cpd	0
Cpb2	0
Cpb1	0
Cpa6	0
Cpa5	0
Cpa3	0
Cp	0
Cox8c	0
Cox8b	0
Cox7c	0
Cox7b2	0
Cox7a2l	0
Cox6c	0
Cox6b2	0
Cox6b1	0
Cox6a2	0

Cep85	0	Cox6b2	0	Tmem33	0	Cox6a1	0
Cep78	0	Cox6a2	0	Prr7	0	Cox5b	0
Cep76	0	Cox5b	0	Pcmtd1	0	Cox5a	0
Cep72	0	Cox4i1	0	Cnep1r1	0	Cox4i2	0
Cep70	0	Cox20	0	Nckap5	0	Cox4i1	0
Cep68	0	Cox19	0	Cth	0	Cox20	0
Cep57l1	0	Cox18	0	Elmo1	0	Cox19	0
Cep57	0	Cox17	0	Nckap1	0	Cox18	0
Cep44	0	Cox16	0	Elmo3	0	Cox17	0
Cep350	0	Cox15	0	Pgf	0	Cox16	0
Cep295	0	Cox14	0	Grm6	0	Cox15	0
Cep290	0	Cox10	0	Pgd	0	Cox14	0
Cep250	0	Cotl1	0	Rpl37a	0	Cox10	0
Cep192	0	Cort	0	Olfr12	0	Cotl1	0
Cep170b	0	Coro7	0	Pgc	0	Coro7	0
Cep170	0	Coro6	0	Olfr419	0	Coro6	0
Cep164	0	Coro2a	0	Sf1	0	Coro1b	0
Cep162	0	Coro1c	0	Dok2	0	Coro1a	0
Cep152	0	Coq6	0	Dok3	0	Corin	0
Cep135	0	Coq4	0	Soga1	0	Coq6	0
Cep131	0	Coq3	0	Dok1	0	Coq5	0
Cep128	0	Coq2	0	F11r	0	Coq4	0
Cep126	0	Coq10b	0	Pgp	0	Coq3	0
Cep112	0	Coq10a	0	Dok5	0	Coq2	0
Cep104	0	Copz2	0	F2rl3	0	Coq10b	0
Cenpw	0	Copz1	0	F2rl2	0	Copz1	0
Cenpv	0	Cops8	0	F2rl1	0	Cops8	0
Cenpu	0	Cops7b	0	Stx3	0	Cops7b	0
Cenpt	0	Cops7a	0	March11	0	Cops6	0
Cenpp	0	Cops6	0	March10	0	Cops5	0
Cenpo	0	Cops5	0	Olfr13	0	Cops4	0
Cenpn	0	Cops3	0	Dnmt3a	0	Cops2	0
Cenpm	0	Cops2	0	Ifnk	0	Copg1	0
Cenpk	0	Coprs	0	Rom1	0	Cope	0
Cenph	0	Copg1	0	Olfr711	0	Copb2	0
Cenpf	0	Cope	0	Gjc2	0	Copa	0
Cenpe	0	Copb2	0	Cdh17	0	Comtd1	0
Cenpc1	0	Copb1	0	Rnf2	0	Comt	0
Cenpb	0	Copa	0	Dnmt3l	0	Commd8	0
Cenpa	0	Comt	0	Gjc3	0	Commd7	0
Cemip	0	Comp	0	Melk	0	Commd6	0
Celsr3	0	Commd9	0	Scp2d1	0	Commd5	0
Celsr2	0	Commd6	0	C1qa	0	Commd4	0
Celsr1	0	Commd5	0	Rims4	0	Commd3	0
Celf5	0	Commd4	0	Plcxd2	0	Commd10	0
Celf3	0	Commd2	0	Rims2	0	Colgalt1	0
Celf2	0	Commd1	0	Rims3	0	Colec12	0
Cela3b	0	Colq	0	Igbp1	0	Colec11	0
Cela3a	0	Colec12	0	Fezf2	0	Colec10	0
Cela2a	0	Colec10	0	Tas2r117	0	Col9a3	0
Cela1	0	Col9a3	0	Arhgef19	0	Col9a2	0
Cel	0	Col9a2	0	Rangap1	0	Col8a1	0
Cecr6	0	Col8a1	0	Cd244	0	Col6a6	0
Cecr5	0	Col7a1	0	Cd247	0	Col6a5	0
Cecr2	0	Col6a6	0	Gsx2	0	Col6a3	0
Cebpzos	0	Col6a3	0	Cytip	0	Col6a2	0
Cebpz	0	Col6a2	0	Cd248	0	Col6a1	0
Cebpg	0	Col6a1	0	Cd209d	0	Col5a2	0
Cebpe	0	Col5a3	0	Nkx2-3	0	Col5a1	0
Cebpb	0	Col5a1	0	Rsph9	0	Col4a6	0
Cebpa	0	Col4a6	0	Caap1	0	Col4a5	0
Ceacam9	0	Col4a5	0	Cdh19	0	Col4a4	0
Ceacam5	0	Col4a4	0	Upp2	0	Col4a3bp	0
Ceacam3	0	Col4a3bp	0	Ddhd1	0	Col4a3	0
Ceacam20	0	Col4a3	0	Wdr74	0	Col4a2	0
Ceacam19	0	Col4a2	0	Ddhd2	0	Col4a1	0
Ceacam18	0	Col4a1	0	Zfp42	0	Col3a1	0
Ceacam16	0	Col3a1	0	Pdzd11	0	Col2a1	0
Ceacam15	0	Col2a1	0	Gm16513	0	Col28a1	0
Ceacam14	0	Col28a1	0	Retnla	0	Col27a1	0
Ceacam13	0	Col27a1	0	Zdhhc20	0	Col25a1	0
Ceacam12	0	Col25a1	0	Zdhhc21	0	Col24a1	0
Ceacam11	0	Col24a1	0	Zdhhc22	0	Col22a1	0
Ceacam10	0	Col22a1	0	Zdhhc23	0	Col1a2	0
Ceacam1	0	Col20a1	0	Zdhhc24	0	Col1a1	0
Cdyl2	0	Col1a1	0	Zdhhc25	0	Col19a1	0
Cdx4	0	Col19a1	0	Wnt2b	0	Col17a1	0
Cdx2	0	Col17a1	0	Serpina3i	0	Col16a1	0
Cdx1	0	Col16a1	0	Zfp408	0	Col15a1	0
Cdt1	0	Col15a1	0	Serpina3k	0	Col12a1	0
Cdsn	0	Col14a1	0	Cd24a	0	Col11a2	0
Cds2	0	Col11a2	0	Fbxw9	0	Col11a1	0

Cds1	0	Col10a1	0	Cox14	0	Col10a1	0
Cdrt4	0	Coil	0	Serpina3c	0	Coil	0
Cdr2l	0	Cog8	0	Serpina3f	0	Cog8	0
Cdpf1	0	Cog7	0	Rbm26	0	Cog7	0
Cdon	0	Cog6	0	Rbm27	0	Cog6	0
Cdo1	0	Cog5	0	Stat5a	0	Cog5	0
Cdnf	0	Cog4	0	Rbm25	0	Cog4	0
Cdkn2d	0	Cog2	0	Cox16	0	Cog3	0
Cdkn2c	0	Cog1	0	Rbm20	0	Cog2	0
Cdkn2b	0	Coch	0	Bcr	0	Cog1	0
Cdkn2aipnl	0	Cobll1	0	C130060K24Rik	0	Coch	0
Cdkn2aip	0	Cobl	0	Enah	0	Cobll1	0
Cdkn1c	0	Coa6	0	Rbm28	0	Cobl	0
Cdkn1b	0	Coa4	0	Krtap19-2	0	Coa7	0
Cdkn1a	0	Coa3	0	Clock	0	Coa6	0
Cdkl5	0	Cntrob	0	Wisp2	0	Coa5	0
Cdkl4	0	Cntnap5c	0	Wisp3	0	Coa4	0
Cdkl3	0	Cntnap5a	0	Tespa1	0	Coa3	0
Cdkal1	0	Cntnap4	0	Gm10267	0	Cntrob	0
Cdk9	0	Cntnap3	0	Ykt6	0	Cntnap5c	0
Cdk8	0	Cntnap2	0	Crb2	0	Cntnap5b	0
Cdk7	0	Cntnap1	0	Lpar2	0	Cntnap4	0
Cdk6	0	Cntn6	0	Krt222	0	Cntnap3	0
Cdk5rap1	0	Cntn5	0	Sh2b2	0	Cntnap2	0
Cdk5r1	0	Cntn3	0	Sh2b1	0	Cntn6	0
Cdk5	0	Cntn2	0	Cntn5	0	Cntn5	0
Cdk4	0	Cntn1	0	Rhbdl3	0	Cntn3	0
Cdk2ap1	0	Cntln	0	Slc25a4	0	Cntn2	0
Cdk20	0	Cntfr	0	Kif20b	0	Cntn1	0
Cdk2	0	Cntd1	0	Lef1	0	Cntln	0
Cdk19	0	Cnst	0	Ireb2	0	Cntf	0
Cdk18	0	Cnrip1	0	Stmn4	0	Cntd1	0
Cdk17	0	Cnpy4	0	Gspt1	0	Cnst	0
Cdk16	0	Cnpy2	0	BRDN0000738336	0	Cnrip1	0
Cdk15	0	Cnpy1	0	Stmn1	0	Cnr1	0
Cdk14	0	Cnppd1	0	Stmn3	0	Cnpy4	0
Cdk13	0	Cnot8	0	Stmn2	0	Cnpy1	0
Cdk12	0	Cnot7	0	Fbxw4	0	Cnppd1	0
Cdk11b	0	Cnot6	0	Eif2ak3	0	Cnot8	0
Cdk10	0	Cnot4	0	Eif2ak2	0	Cnot7	0
Cdk1	0	Cnot3	0	Rab2b	0	Cnot6l	0
Cdipt	0	Cnot2	0	Ube2d1	0	Cnot6	0
Cdip1	0	Cnot10	0	Camkk2	0	Cnot4	0
Cdhr3	0	Cnot1	0	Camkk1	0	Cnot3	0
Cdhr1	0	Cnnm4	0	Fkbp1b	0	Cnot2	0
Cdh9	0	Cnnm1	0	BRDN0000738104	0	Cnot11	0
Cdh8	0	Cnn3	0	Slc25a19	0	Cnnm4	0
Cdh6	0	Cnn1	0	Slc25a18	0	Cnnm3	0
Cdh5	0	Cnksr1	0	Slc25a17	0	Cnnm2	0
Cdh4	0	Cnih4	0	Erich6	0	Cnnm1	0
Cdh3	0	Cnih1	0	Slc25a15	0	Cnksr3	0
Cdh26	0	Cngb3	0	Olfr412	0	Cnksr1	0
Cdh24	0	Cnga3	0	Erich2	0	Cnih4	0
Cdh23	0	Cnga2	0	Erich1	0	Cnih3	0
Cdh22	0	Cnga1	0	Slc25a10	0	Cnih1	0
Cdh2	0	Cnep1r1	0	Btg1-ps2	0	Cngb3	0
Cdh19	0	Cndp2	0	Cspg4	0	Cnga3	0
Cdh18	0	Cndp1	0	Cspg5	0	Cnga2	0
Cdh17	0	Cnbp	0	Nudc	0	Cnfn	0
Cdh16	0	Cnbd2	0	Tgfb2	0	Cnep1r1	0
Cdh15	0	Cmya5	0	Trmt1	0	Cndp1	0
Cdh13	0	Cmtr2	0	Spint2	0	Cnbp	0
Cdh12	0	Cmtr1	0	2410137M14Rik	0	Cmya5	0
Cdh10	0	Cmtm7	0	Pitpnm1	0	Cmtr2	0
Cdh1	0	Cmtm6	0	Nol12	0	Cmtr1	0
Cdcp2	0	Cmtm5	0	4930505A04Rik	0	Cmtm8	0
Cdcp1	0	Cmtm4	0	F10	0	Cmtm7	0
Cdca8	0	Cmtm2b	0	BRDN0000737886	0	Cmtm6	0
Cdca7l	0	Cmtm2a	0	Pigw	0	Cmtm4	0
Cdca7	0	Cmss1	0	Fkbp14	0	Cmtm2a	0
Cdca5	0	Cmpk2	0	Mocs2	0	Cmss1	0
Cdca4	0	Cml5	0	Olfr457	0	Cml5	0
Cdca3	0	Cml3	0	Olfr456	0	Cml3	0
Cdca2	0	Cml2	0	Fkbp10	0	Cml2	0
Cdc73	0	Cml1	0	Fkbp11	0	Cmklr1	0
Cdc7	0	Cmklr1	0	Cadps2	0	Cmip	0
Cdc6	0	Cmip	0	Olfr459	0	Cmc1	0
Cdc45	0	Cmc2	0	Olfr458	0	Cmah	0
Cdc42se2	0	Cmbl	0	Slc12a7	0	Cma2	0
Cdc42se1	0	Cmah	0	Jade2	0	Clvs1	0
Cdc42ep5	0	Cma2	0	Vmn1r132	0	Cluh	0
Cdc42ep4	0	Cma1	0	2210404O09Rik	0	Cluap1	0

Cdc42ep2	0
Cdc42ep1	0
Cdc42bpa	0
Cdc40	0
Cdc37l1	0
Cdc37	0
Cdc27	0
Cdc26	0
Cdc25c	0
Cdc25b	0
Cdc23	0
Cdc20b	0
Cdc20	0
Cdc16	0
Cdc14a	0
Cdc123	0
Cdan1	0
Cda	0
Cd99l2	0
Cd96	0
Cd93	0
Cd9	0
Cd8b1	0
Cd8a	0
Cd86	0
Cd84	0
Cd83	0
Cd82	0
Cd81	0
Cd80	0
Cd79a	0
Cd72	0
Cd70	0
Cd7	0
Cd69	0
Cd68	0
Cd63	0
Cd6	0
Cd5l	0
Cd59b	0
Cd59a	0
Cd55	0
Cd53	0
Cd52	0
Cd5	0
Cd47	0
Cd46	0
Cd40lg	0
Cd40	0
Cd4	0
Cd3eap	0
Cd3e	0
Cd38	0
Cd37	0
Cd36	0
Cd34	0
Cd33	0
Cd320	0
Cd300lh	0
Cd300lf	0
Cd300lb	0
Cd300e	0
Cd300a	0
Cd2ap	0
Cd28	0
Cd276	0
Cd274	0
Cd24a	0
Cd248	0
Cd247	0
Cd244	0
Cd226	0
Cd22	0
Cd209g	0
Cd209f	0
Cd209e	0
Cd209d	0
Cd209c	0
Cd209a	0
Cd207	0
Cd200r4	0
Cd200r2	0
Clybl	0
Clvs2	0
Cluh	0
Cluap1	0
Clu	0
Cltb	0
Clta	0
Clstn3	0
Clstn2	0
Clstn1	0
Clspn	0
Clrn3	0
Clrn2	0
Clrn1	0
Clpx	0
Clptm1l	0
Clptm1	0
Clpsl2	0
Clp1	0
Clock	0
Cln8	0
Cln6	0
Cln3	0
Clk2	0
Clk1	0
Clip4	0
Clip3	0
Clip2	0
Clip1	0
Clint1	0
Clic4	0
Clic1	0
Clec5a	0
Clec4f	0
Clec4e	0
Clec4d	0
Clec4b2	0
Clec4b1	0
Clec4a2	0
Clec3b	0
Clec3a	0
Clec2l	0
Clec2i	0
Clec2g	0
Clec2e	0
Clec2d	0
Clec1b	0
Clec1a	0
Clec12a	0
Clec11a	0
Cldnd1	0
Cldn9	0
Cldn7	0
Cldn6	0
Cldn5	0
Cldn24	0
Cldn23	0
Cldn19	0
Cldn18	0
Cldn17	0
Cldn16	0
Cldn15	0
Cldn13	0
Cldn11	0
Cldn10	0
Clcnkb	0
Clcnka	0
Clcn7	0
Clcn6	0
Clcn4-2	0
Clcn3	0
Clcn2	0
Clcf1	0
Clcc1	0
Clca4b	0
Clca3b	0
Clca3a2	0
Clca2	0
Clca1	0
Clasrp	0
Clasp2	0
Cks2	0
Vmn1r131	0
Mtfr1	0
Mtfr2	0
Vmn1r139	0
Pvrl4	0
Ifitm3	0
Cdk5rap1	0
Bbs2	0
Camkv	0
Ifitm6	0
Ifitm5	0
Scd2	0
Scd3	0
Mybphl	0
Scd1	0
Mtcp1	0
Selplg	0
Scd4	0
Pard6b	0
Tubg1	0
Htra1	0
Copg1	0
Ddx26b	0
1700015G11Rik	0
Tbkbp1	0
Snap91	0
A430033K04Rik	0
Cwc25	0
Tarbp2	0
Zfp518a	0
Fem1b	0
Des	0
Zfp518b	0
Cntnap5c	0
Slc12a8	0
Specc1l	0
Cd96	0
Prkar2b	0
Prkcdbp	0
Dcp1b	0
Dcp1a	0
Ppp5c	0
Il4ra	0
Fem1a	0
Suco	0
Tigd4	0
Mip	0
Klk5	0
Gucy2c	0
Gucy2d	0
Gucy2e	0
Gucy2f	0
Gucy2g	0
Keap1	0
Olfr813	0
Kcnq5	0
Fam26f	0
Fam26d	0
Fam26e	0
Homez	0
Arl4d	0
Pcdhac2	0
Tnp2	0
Umps	0
Cstf2t	0
Gm14345	0
Dcpp2	0
Txnl1	0
Gm14346	0
Pgpep1	0
Slc4a5	0
Oas1h	0
Slc4a7	0
Tnfsf8	0
Slc4a1	0
Cul9	0
Slc4a3	0
Slc4a2	0
Oas1a	0
Lyar	0
Oas1c	0
Slc4a9	0
Clu	0
Cltb	0
Clta	0
Clstn3	0
Clstn1	0
Clrn3	0
Clrn2	0
Clrn1	0
Clpx	0
Clptm1l	0
Clptm1	0
Clps	0
Clpp	0
Clpb	0
Clp1	0
Clock	0
Clns1a	0
Cln8	0
Cln6	0
Cln3	0
Clmn	0
Clk3	0
Clk1	0
Clip4	0
Clip3	0
Clic4	0
Clic1	0
Clgn	0
Clec7a	0
Clec5a	0
Clec4n	0
Clec4f	0
Clec4e	0
Clec4b1	0
Clec4a2	0
Clec3b	0
Clec2l	0
Clec2i	0
Clec2e	0
Clec1b	0
Clec1a	0
Clec12b	0
Clec12a	0
Clec11a	0
Clec10a	0
Cldnd2	0
Cldnd1	0
Cldn9	0
Cldn8	0
Cldn6	0
Cldn5	0
Cldn3	0
Cldn24	0
Cldn22	0
Cldn19	0
Cldn16	0
Cldn15	0
Cldn12	0
Cldn11	0
Cldn10	0
Cldn1	0
Clcnkb	0
Clcn7	0
Clcn6	0
Clcn5	0
Clcn4-2	0
Clcn3	0
Clcn2	0
Clcf1	0
Clcc1	0
Clca4b	0
Clca3b	0
Clca1	0
Clasrp	0
Clasp2	0
Cks2	0
Ckmt2	0
Ckmt1	0
Cklf	0
Ckb	0
Ckap5	0
Ckap4	0

Cd200r1	0
Cd200	0
Cd2	0
Cd1d1	0
Cd180	0
Cd164l2	0
Cd163	0
Cd160	0
Cd151	0
Cd14	0
Cd101	0
Ccz1	0
Cct8l1	0
Cct8	0
Cct7	0
Cct6b	0
Cct5	0
Cct4	0
Cct3	0
Cct2	0
Ccser2	0
Ccs	0
Ccrn4l	0
Ccrl2	0
Ccr9	0
Ccr8	0
Ccr7	0
Ccr5	0
Ccr4	0
Ccr3	0
Ccr1l1	0
Ccr10	0
Ccpg1os	0
Ccpg1	0
Ccp110	0
Ccnyl1	0
Ccny	0
Ccnt2	0
Ccnt1	0
Ccno	0
Ccnl2	0
Ccnl1	0
Ccnk	0
Ccnjl	0
Ccnj	0
Ccni	0
Ccnh	0
Ccng2	0
Ccng1	0
Ccnf	0
Ccne2	0
Ccne1	0
Ccndbp1	0
Ccnd2	0
Ccnd1	0
Ccnb2	0
Ccnb1	0
Ccna2	0
Ccna1	0
Ccm2l	0
Ccm2	0
Ccl9	0
Ccl8	0
Ccl7	0
Ccl6	0
Ccl5	0
Ccl4	0
Ccl3	0
Ccl28	0
Ccl27b	0
Ccl27a	0
Ccl25	0
Ccl24	0
Ccl22	0
Ccl21b	0
Ccl19	0
Ccl17	0
Ccl11	0
Ccl1	0
Cckbr	0
Cckar	0
Cck	0

Cks1brt	0
Cks1b	0
Ckmt2	0
Ckmt1	0
Ckm	0
Ckap5	0
Ckap2l	0
Ckap2	0
Cited4	0
Cited2	0
Cish	0
Cisd3	0
Cisd2	0
Cisd1	0
Cirh1a	0
Cirbp	0
Cir1	0
Cilp2	0
Cilp	0
Ciita	0
Cidec	0
Cidea	0
Cic	0
Cib3	0
Cib2	0
Cib1	0
Ciart	0
Ciapin1	0
Ciao1	0
Churc1	0
Chtf8	0
Chsy3	0
Chst9	0
Chst8	0
Chst5	0
Chst3	0
Chst2	0
Chst12	0
Chst10	0
Chst1	0
Chrne	0
Chrnd	0
Chrnb3	0
Chrna9	0
Chrna3	0
Chrna2	0
Chrna1	0
Chrm4	0
Chrm3	0
Chrm2	0
Chrm1	0
Chrdl2	0
Chrdl1	0
Chrd	0
Chrac1	0
Chpt1	0
Chpf2	0
Chpf	0
Chp1	0
Chordc1	0
Chodl	0
Chn1	0
Chmp6	0
Chmp4c	0
Chmp4b	0
Chmp3	0
Chmp2b	0
Chmp2a	0
Chml	0
Chl1	0
Chkb	0
Chka	0
Chil6	0
Chil4	0
Chil1	0
Chid1	0
Chic2	0
Chgb	0
Chfr	0
Cherp	0
Chek2	0
Chek1	0

Slc4a8	0
Oas1g	0
Tnfsf4	0
Osgin1	0
Vmn2r37	0
Lcat	0
Hspa4l	0
1700015E13Rik	0
Kcnq3	0
Mt2	0
Ovca2	0
Ptp4a1	0
Ptp4a3	0
Ptp4a2	0
Igf2bp1	0
Igf2bp3	0
Igf2bp2	0
2010002M12Rik	0
Pgap1	0
Dcbld2	0
Dcbld1	0
Pgap2	0
Macc1	0
Klra9	0
Rab20	0
Klra7	0
Klra6	0
Klra5	0
Maats1	0
Klra3	0
Klra2	0
Klra1	0
Cdh9	0
Cdh8	0
Mfap5	0
Mfap4	0
Mfap3	0
Mfap2	0
Cdh1	0
Arl5a	0
Cdh3	0
Cdh5	0
Cdh4	0
Cdh7	0
Cdh6	0
Olfr654	0
Zfp551	0
Mov10	0
Nupr1	0
Tas2r119	0
Pcdhga8	0
Nt5c3b	0
Nmu	0
Mcmdc2	0
Dthd1	0
Col6a3	0
Col6a2	0
Svs3b	0
Col6a4	0
Dynll2	0
Svs3a	0
Olfr1208	0
Olfr657	0
Ghrh	0
Tbc1d19	0
Ghrl	0
Olfr1200	0
Olfr1201	0
Olfr1202	0
Olfr1204	0
Olfr1205	0
Cep57l1	0
BRDN0000737964	0
Neurl2	0
Neurl4	0
Efcc1	0
Hormad2	0
Gsx1	0
Hormad1	0
Eif5a	0
Gm7361	0
BRDN0000738208	0

Ckap2l	0
Ckap2	0
CK137956	0
Cited4	0
Cited2	0
Cited1	0
Cish	0
Cisd3	0
Cisd2	0
Cirbp	0
Cipc	0
Cinp	0
Cilp2	0
Ciita	0
Cidec	0
Cidea	0
Cib3	0
Cib2	0
Ciart	0
Ciapin1	0
Ciao1	0
Chtf8	0
Chsy3	0
Chsy1	0
Chst9	0
Chst8	0
Chst5	0
Chst3	0
Chst2	0
Chst15	0
Chst14	0
Chst12	0
Chst11	0
Chst1	0
Chrne	0
Chrnb3	0
Chrnb2	0
Chrnb1	0
Chrna7	0
Chrna6	0
Chrna4	0
Chrna3	0
Chrna10	0
Chrna1	0
Chrm5	0
Chrm3	0
Chrm2	0
Chrm1	0
Chrdl2	0
Chrdl1	0
Chrd	0
Chrac1	0
Chpt1	0
Chp2	0
Chp1	0
Chordc1	0
Chodl	0
Chn2	0
Chn1	0
Chmp7	0
Chmp6	0
Chmp4c	0
Chmp4b	0
Chmp3	0
Chmp2b	0
Chmp2a	0
Chmp1a	0
Chml	0
Chm	0
Chkb	0
Chka	0
Chil6	0
Chil4	0
Chil3	0
Chil1	0
Chic1	0
Chia1	0
Chgb	0
Chga	0
Cherp	0
Chek2	0
Chdh	0

Ccin	0
Cchcr1	0
Ccer1	0
Ccdc97	0
Ccdc96	0
Ccdc94	0
Ccdc93	0
Ccdc92	0
Ccdc91	0
Ccdc90b	0
Ccdc9	0
Ccdc89	0
Ccdc88c	0
Ccdc88b	0
Ccdc88a	0
Ccdc87	0
Ccdc86	0
Ccdc85c	0
Ccdc84	0
Ccdc83	0
Ccdc82	0
Ccdc80	0
Ccdc8	0
Ccdc79	0
Ccdc78	0
Ccdc77	0
Ccdc74a	0
Ccdc73	0
Ccdc71l	0
Ccdc7	0
Ccdc69	0
Ccdc68	0
Ccdc66	0
Ccdc65	0
Ccdc63	0
Ccdc62	0
Ccdc61	0
Ccdc60	0
Ccdc58	0
Ccdc55	0
Ccdc51	0
Ccdc47	0
Ccdc42b	0
Ccdc40	0
Ccdc39	0
Ccdc38	0
Ccdc37	0
Ccdc36	0
Ccdc34	0
Ccdc33	0
Ccdc32	0
Ccdc3	0
Ccdc28b	0
Ccdc27	0
Ccdc24	0
Ccdc23	0
Ccdc22	0
Ccdc185	0
Ccdc184	0
Ccdc183	0
Ccdc181	0
Ccdc18	0
Ccdc178	0
Ccdc177	0
Ccdc176	0
Ccdc175	0
Ccdc174	0
Ccdc173	0
Ccdc172	0
Ccdc171	0
Ccdc170	0
Ccdc17	0
Ccdc169	0
Ccdc166	0
Ccdc160	0
Ccdc158	0
Ccdc157	0
Ccdc152	0
Ccdc151	0
Ccdc150	0
Ccdc15	0
Ccdc149	0
Chd9	0
Chd7	0
Chd6	0
Chd5	0
Chd3	0
Chd2	0
Chd1l	0
Chd1	0
Chchd7	0
Chchd5	0
Chchd4	0
Chchd3	0
Chchd2	0
Chchd10	0
Chat	0
Champ1	0
Chaf1b	0
Chaf1a	0
Chadl	0
Chac2	0
Chac1	0
Cgrrf1	0
Cgref1	0
Cgn	0
Cggbp1	0
Cga	0
Cftr	0
Cfp	0
Cflar	0
Cfl2	0
Cfi	0
Cfhr1	0
Cfh	0
Cfdp1	0
Cfd	0
Cfap97	0
Cfap70	0
Cfap69	0
Cfap57	0
Cfap52	0
Cfap45	0
Cfap36	0
Cfap126	0
Cetn4	0
Cetn3	0
Cetn2	0
Cetn1	0
Ces3b	0
Ces2h	0
Ces2g	0
Ces2f	0
Ces2c	0
Ces2b	0
Ces2a	0
Ces1g	0
Ces1f	0
Ces1b	0
Ces1a	0
Cers6	0
Cers5	0
Cers4	0
Cerkl	0
Cercam	0
Cept1	0
Cep97	0
Cep89	0
Cep83	0
Cep72	0
Cep57	0
Cep55	0
Cep350	0
Cep295	0
Cep290	0
Cep192	0
Cep19	0
Cep170	0
Cep164	0
Cep162	0
Cep152	0
Cep135	0
Cep131	0
Cep128	0
Zfp672	0
Zfp677	0
Oas1d	0
BRDN0000737960	0
Polr1a	0
Polr1b	0
Txn1	0
Polr1d	0
Polr1e	0
Cacng1	0
1110032A03Rik	0
Stau2	0
BRDN0000737963	0
Olfr651	0
Tgif1	0
Bex2	0
Sox21	0
Thsd4	0
Olfr739	0
Adarb2	0
Crct1	0
BC051665	0
Adarb1	0
Creb3l2	0
Prcc	0
Snx29	0
Plec	0
Creb3l4	0
Fra10ac1	0
Snx22	0
Lrmp	0
Snx20	0
Snx21	0
Serpinb1c	0
Snx27	0
Snx24	0
Snx25	0
Fam181a	0
Vps37d	0
Sfr1	0
Fam160a1	0
Cdkn3	0
Vnn3	0
Mdfic	0
Vnn1	0
Msh5	0
Olfr50	0
Olfr51	0
Olfr52	0
Mrpl40	0
Olfr55	0
Mrpl45	0
Olfr57	0
Olfr58	0
Olfr59	0
Mrpl49	0
Ldoc1l	0
Bcl7c	0
Swsap1	0
C4b	0
Ano7	0
BRDN0000737734	0
Vmn1r216	0
B230217C12Rik	0
Gltscr1	0
Gltscr2	0
Kcna3	0
Dppa2	0
Ampd3	0
Magel2	0
Dppa1	0
Tiprl	0
Ppp1r13b	0
Dppa4	0
Marveld2	0
Sh3bgrl	0
Ppp1r13l	0
Lbx1	0
Smpdl3b	0
Smpdl3a	0
Pou4f1	0
Ctdnep1	0
Chd8	0
Chd7	0
Chd6	0
Chd5	0
Chd4	0
Chd3	0
Chd2	0
Chd1l	0
Chd1	0
Chchd6	0
Chchd5	0
Chchd3	0
Chchd2	0
Chchd10	0
Chat	0
Chaf1b	0
Chaf1a	0
Chadl	0
Chac2	0
Ch25h	0
Cgrrf1	0
Cgref1	0
Cgn	0
Cggbp1	0
Cfp	0
Cflar	0
Cfl2	0
Cfi	0
Cfhr2	0
Cfh	0
Cfdp1	0
Cfd	0
Cfc1	0
Cfap97	0
Cfap74	0
Cfap70	0
Cfap58	0
Cfap57	0
Cfap53	0
Cfap52	0
Cfap45	0
Cfap36	0
Cfap221	0
Cfap20	0
Cfap126	0
Cetn4	0
Cetn3	0
Cetn2	0
Cetn1	0
Ces5a	0
Ces3b	0
Ces3a	0
Ces2f	0
Ces2e	0
Ces2c	0
Ces2b	0
Ces2a	0
Ces1g	0
Ces1f	0
Ces1e	0
Ces1d	0
Ces1c	0
Ces1b	0
Ces1a	0
Cers6	0
Cers3	0
Cerkl	0
Cerk	0
Cercam	0
Cept1	0
Cep97	0
Cep95	0
Cep89	0
Cep85	0
Cep83	0
Cep78	0
Cep76	0
Cep70	0
Cep68	0
Cep57l1	0
Cep57	0
Cep55	0

Ccdc144b	0	Cep126	0	Src	0	Cep44	0
Ccdc142	0	Cep120	0	A3galt2	0	Cep350	0
Ccdc141	0	Cep112	0	Rbpj	0	Cep290	0
Ccdc14	0	Cep104	0	Cyp2t4	0	Cep192	0
Ccdc138	0	Cenpu	0	Fam110a	0	Cep19	0
Ccdc137	0	Cenpt	0	Fam110c	0	Cep162	0
Ccdc136	0	Cenpq	0	Fhdc1	0	Cep152	0
Ccdc134	0	Cenpp	0	Krtap13-1	0	Cep135	0
Ccdc132	0	Cenpo	0	Dusp1	0	Cep131	0
Ccdc130	0	Cenpn	0	Cipc	0	Cep126	0
Ccdc13	0	Cenpk	0	Ptgdr	0	Cep120	0
Ccdc129	0	Cenph	0	AI837181	0	Cep112	0
Ccdc127	0	Cenpe	0	4921511H03Rik	0	Cep104	0
Ccdc125	0	Cenpc1	0	Pigz	0	Cenpw	0
Ccdc124	0	Cenpb	0	Dcn	0	Cenpv	0
Ccdc122	0	Cenpa	0	Ccnf	0	Cenpu	0
Ccdc121	0	Cend1	0	Ppp1r9b	0	Cenpt	0
Ccdc12	0	Celsr3	0	Mtbp	0	Cenpp	0
Ccdc116	0	Celsr1	0	0610011F06Rik	0	Cenpo	0
Ccdc115	0	Celf6	0	Onecut3	0	Cenpn	0
Ccdc114	0	Celf5	0	Hist2h2ab	0	Cenpm	0
Ccdc113	0	Celf3	0	Dcc	0	Cenpk	0
Ccdc112	0	Cela3b	0	Hist2h2ac	0	Cenpj	0
Ccdc109b	0	Cela3a	0	Tnfrsf23	0	Cenph	0
Ccdc108	0	Cela2a	0	Tnfrsf22	0	Cenpf	0
Ccdc107	0	Cel	0	Mmachc	0	Cenpe	0
Ccdc106	0	Cecr5	0	Mtx1	0	Cenpa	0
Ccdc105	0	Cebpzos	0	Tnfrsf26	0	Cend1	0
Ccdc103	0	Cebpz	0	Tnfrsf25	0	Celsr3	0
Ccdc102a	0	Cebpg	0	Gnat1	0	Celsr1	0
Ccdc101	0	Cebpe	0	2810004N23Rik	0	Celf6	0
Ccbl2	0	Cebpb	0	Gnat3	0	Celf4	0
Ccbl1	0	Cebpa	0	Gnat2	0	Celf2	0
Ccbe1	0	Ceacam9	0	Lap3	0	Celf1	0
Ccar2	0	Ceacam5	0	Enpp5	0	Cela3a	0
Ccar1	0	Ceacam3	0	Tomm20	0	Cela2a	0
Cc2d2a	0	Ceacam18	0	Gm17252	0	Cela1	0
Cc2d1b	0	Ceacam16	0	Anxa1	0	Cel	0
Cc2d1a	0	Ceacam15	0	Noc2l	0	Cecr5	0
Cby1	0	Ceacam13	0	Fgd1	0	Cebpzos	0
Cbx8	0	Ceacam12	0	Hnf1a	0	Cebpz	0
Cbx5	0	Ceacam1	0	Ppp1r3f	0	Cebpg	0
Cbx3	0	Cdyl	0	Ckm	0	Cebpe	0
Cbx2	0	Cdx4	0	Adgrf1	0	Cebpb	0
Cbx1	0	Cdx2	0	Adgrf3	0	Ceacam9	0
Cbwd1	0	Cdx1	0	Gmfb	0	Ceacam3	0
Cbs	0	Cdv3	0	Adgrf5	0	Ceacam19	0
Cbr4	0	Cdt1	0	Gm428	0	Ceacam18	0
Cbr3	0	Cds2	0	Snapc3	0	Ceacam16	0
Cbr2	0	Cds1	0	Snapc2	0	Ceacam15	0
Cbln3	0	Cdrt4	0	Snapc1	0	Ceacam11	0
Cbln2	0	Cdr2l	0	Rbp3	0	Cdyl	0
Cbln1	0	Cdr2	0	Rbp4	0	Cdx4	0
Cblc	0	Cdr1	0	Stxbp4	0	Cdx1	0
Cbl	0	Cdpf1	0	Snapc5	0	Cdv3	0
Cbfb	0	Cdon	0	Snapc4	0	Cdsn	0
Cav3	0	Cdkn3	0	Smpx	0	Cds2	0
Cav1	0	Cdkn2b	0	Ly6h	0	Cdrt4	0
Catsperd	0	Cdkn2aipnl	0	Als2cl	0	Cdr2	0
Catsperb	0	Cdkn2aip	0	7420426K07Rik	0	Cdon	0
Catsper3	0	Cdkn2a	0	Uri1	0	Cdkn3	0
Catsper2	0	Cdkn1c	0	Fignl1	0	Cdkn2d	0
Catsper1	0	Cdkn1a	0	Bhlhb9	0	Cdkn2c	0
Catip	0	Cdkl5	0	Nt5m	0	Cdkn2aipnl	0
Casz1	0	Cdkl4	0	Anxa7	0	Cdkn2aip	0
Cast	0	Cdkl3	0	Fam210b	0	Cdkn2a	0
Cass4	0	Cdkl1	0	Serpinc1	0	Cdkn1c	0
Casr	0	Cdkal1	0	Zfp780b	0	Cdkn1a	0
Casq2	0	Cdk9	0	Celf6	0	Cdkl5	0
Casq1	0	Cdk7	0	Nt5c	0	Cdkl3	0
Casp9	0	Cdk6	0	Cldn15	0	Cdkl2	0
Casp8ap2	0	Cdk5rap3	0	Nt5e	0	Cdkl1	0
Casp8	0	Cdk5r2	0	Celf2	0	Cdk9	0
Casp7	0	Cdk5	0	Figla	0	Cdk8	0
Casp3	0	Cdk4	0	Zfp512	0	Cdk7	0
Casp14	0	Cdk2	0	Gm5134	0	Cdk5rap3	0
Casp12	0	Cdk17	0	Ubr1	0	Cdk5rap2	0
Casp1	0	Cdk14	0	Gm5640	0	Cdk5r2	0
Caskin2	0	Cdk13	0	Slc50a1	0	Cdk5r1	0
Caskin1	0	Cdk11b	0	Gm5132	0	Cdk4	0
Cask	0	Cdk10	0	Pask	0	Cdk2ap2	0
Casc5	0	Cdk1	0	Slc25a31	0	Cdk20	0

Casc4	0	Cdipt	0	Ifne	0	Cdk2	0
Casc3	0	Cdhr5	0	Xirp2	0	Cdk19	0
Casc1	0	Cdhr3	0	Kras	0	Cdk18	0
Cartpt	0	Cdhr2	0	BRDN0000738176	0	Cdk15	0
Cars2	0	Cdhr1	0	Aoc1	0	Cdk13	0
Cars	0	Cdh9	0	BRDN0000738174	0	Cdk12	0
Carns1	0	Cdh6	0	Cldn19	0	Cdk10	0
Carkd	0	Cdh5	0	Rhpn1	0	Cdk1	0
Carhsp1	0	Cdh4	0	Nebl	0	Cdipt	0
Carf	0	Cdh26	0	BRDN0000738170	0	Cdip1	0
Card9	0	Cdh23	0	D630033O11Rik	0	Cdhr5	0
Card6	0	Cdh22	0	BRDN0000738178	0	Cdhr3	0
Card14	0	Cdh20	0	BRDN0000738179	0	Cdhr2	0
Card11	0	Cdh19	0	Ezh1	0	Cdhr1	0
Card10	0	Cdh18	0	Ezh2	0	Cdh8	0
Car9	0	Cdh17	0	Clca4b	0	Cdh7	0
Car8	0	Cdh16	0	Clca4a	0	Cdh6	0
Car7	0	Cdh12	0	Slc35d3	0	Cdh5	0
Car6	0	Cdh10	0	Slc35d2	0	Cdh26	0
Car4	0	Cdh1	0	Slc35d1	0	Cdh23	0
Car2	0	Cdca8	0	Rnls	0	Cdh22	0
Car15	0	Cdca7l	0	Zmiz1	0	Cdh20	0
Car13	0	Cdca7	0	Mettl7b	0	Cdh2	0
Car12	0	Cdca5	0	Lrrk2	0	Cdh17	0
Car1	0	Cdca4	0	Sry	0	Cdh16	0
Capzb	0	Cdc73	0	Zc3h10	0	Cdh15	0
Capza2	0	Cdc7	0	Alx3	0	Cdh13	0
Capza1	0	Cdc6	0	Ppt1	0	Cdh12	0
Capsl	0	Cdc5l	0	Alx1	0	Cdh11	0
Caps2	0	Cdc45	0	Lpl	0	Cdh10	0
Caprin2	0	Cdc42se2	0	Ccl2	0	Cdh1	0
Caprin1	0	Cdc42se1	0	Vmn1r87	0	Cdcp2	0
Capns2	0	Cdc42ep4	0	Alx4	0	Cdcp1	0
Capns1	0	Cdc42ep3	0	Vmn1r86	0	Cdca8	0
Capn8	0	Cdc42bpb	0	Vdac3	0	Cdca7l	0
Capn7	0	Cdc42bpa	0	Zc3h15	0	Cdca7	0
Capn6	0	Cdc42	0	Vdac1	0	Cdca5	0
Capn5	0	Cdc40	0	Il18bp	0	Cdca3	0
Capn3	0	Cdc37	0	Ralgapa1	0	Cdc73	0
Capn2	0	Cdc27	0	Ralgapa2	0	Cdc7	0
Capn12	0	Cdc26	0	Zc3h14	0	Cdc6	0
Capn11	0	Cdc25b	0	Olfr668	0	Cdc5l	0
Capn10	0	Cdc23	0	Olfr669	0	Cdc45	0
Capn1	0	Cdc20b	0	BRDN0000738310	0	Cdc42se2	0
Capg	0	Cdc16	0	BRDN0000738311	0	Cdc42ep3	0
Cap1	0	Cdc14b	0	Rps3a1	0	Cdc42ep2	0
Canx	0	Cdc14a	0	Rcn2	0	Cdc42ep1	0
Cant1	0	Cdc123	0	Rcn1	0	Cdc42bpg	0
Cand2	0	Cdan1	0	Gba	0	Cdc42bpb	0
Cand1	0	Cdadc1	0	Rrp8	0	Cdc42bpa	0
Camta2	0	Cd99l2	0	Rrp9	0	Cdc42	0
Camsap3	0	Cd96	0	Npepl1	0	Cdc40	0
Camsap1	0	Cd93	0	BRDN0000738319	0	Cdc37l1	0
Camp	0	Cd9	0	Olfr665	0	Cdc34	0
Caml	0	Cd8a	0	Ntrk2	0	Cdc27	0
Camkv	0	Cd84	0	Olfr667	0	Cdc26	0
Camkmt	0	Cd83	0	Dna2	0	Cdc25b	0
Camkk2	0	Cd82	0	Arr3	0	Cdc20b	0
Camkk1	0	Cd80	0	Phip	0	Cdc20	0
Camk4	0	Cd74	0	Mrpl46	0	Cdc16	0
Camk2n2	0	Cd70	0	Tgfb3	0	Cdc14b	0
Camk2n1	0	Cd7	0	Krtap16-1	0	Cdc14a	0
Camk2g	0	Cd69	0	Dtwd2	0	Cdc123	0
Camk2d	0	Cd63	0	Gramd1a	0	Cda	0
Camk2b	0	Cd6	0	Dtwd1	0	Cd99l2	0
Camk1d	0	Cd5l	0	Nupr1l	0	Cd96	0
Caly	0	Cd59b	0	Olfr270	0	Cd93	0
Calu	0	Cd59a	0	Il10ra	0	Cd9	0
Calr4	0	Cd55	0	Il10rb	0	Cd8a	0
Calr3	0	Cd5	0	Vmn1r89	0	Cd84	0
Calr	0	Cd47	0	Ubr5	0	Cd83	0
Caln1	0	Cd46	0	Iqcf1	0	Cd82	0
Calml4	0	Cd4	0	Cyp2c66	0	Cd81	0
Calml3	0	Cd3eap	0	Epsti1	0	Cd79a	0
Calm5	0	Cd3d	0	Ftcd	0	Cd74	0
Calm4	0	Cd38	0	Gnrhr	0	Cd72	0
Calm3	0	Cd37	0	Fhl5	0	Cd70	0
Calm2	0	Cd36	0	Lcorl	0	Cd7	0
Calhm2	0	Cd34	0	Map3k15	0	Cd69	0
Calhm1	0	Cd33	0	Cdc37l1	0	Cd68	0
Cald1	0	Cd300lf	0	Oaf	0	Cd63	0
Calcrl	0	Cd300lb	0	Tdo2	0	Cd6	0

Calcr	0
Calcoco2	0
Calcb	0
Calb2	0
Calb1	0
Cage1	0
Cadps2	0
Cadps	0
Cadm3	0
Cadm2	0
Cadm1	0
Cad	0
Cacybp	0
Cacul1	0
Cacng7	0
Cacng5	0
Cacng4	0
Cacng2	0
Cacng1	0
Cacnb3	0
Cacnb1	0
Cacna2d4	0
Cacna2d1	0
Cacna1s	0
Cacna1i	0
Cacna1g	0
Cacna1e	0
Cacna1d	0
Cacna1c	0
Cacna1b	0
Cacna1a	0
Cachd1	0
Cacfd1	0
Cabs1	0
Cabp7	0
Cabp5	0
Cabp4	0
Cabp2	0
Cabp1	0
Cables2	0
Cables1	0
Cabin1	0
Cab39	0
Caap1	0
C9	0
C8g	0
C8a	0
C87977	0
C87499	0
C87436	0
C86695	0
C77080	0
C5ar2	0
C5ar1	0
C530008M17Rik	0
C4b	0
C3ar1	0
C330027C09Rik	0
C330021F23Rik	0
C330007P06Rik	0
C2cd4d	0
C2cd4c	0
C2cd4b	0
C2cd4a	0
C2cd2l	0
C230052I12Rik	0
C2	0
C1s2	0
C1s1	0
C1rl	0
C1rb	0
C1ra	0
C1qtnf6	0
C1qtnf4	0
C1qtnf1	0
C1ql3	0
C1ql2	0
C1ql1	0
C1qc	0
C1qbp	0
C1galt1c1	0
C1d	0
Cd300e	0
Cd300a	0
Cd2bp2	0
Cd276	0
Cd27	0
Cd248	0
Cd244	0
Cd226	0
Cd22	0
Cd209g	0
Cd209f	0
Cd209e	0
Cd209d	0
Cd209c	0
Cd209b	0
Cd209a	0
Cd200r4	0
Cd200r3	0
Cd200r2	0
Cd200	0
Cd1d1	0
Cd180	0
Cd164l2	0
Cd163l1	0
Cd163	0
Cd151	0
Cd14	0
Cd109	0
Cd101	0
Ccz1	0
Cct8l1	0
Cct8	0
Cct7	0
Cct6b	0
Cct5	0
Cct4	0
Cct3	0
Cct2	0
Ccsap	0
Ccs	0
Ccrn4l	0
Ccrl2	0
Ccr8	0
Ccr7	0
Ccr5	0
Ccr4	0
Ccr1l1	0
Ccr1	0
Ccpg1	0
Ccnyl1	0
Ccny	0
Ccnt2	0
Ccnt1	0
Ccno	0
Ccnj	0
Ccni	0
Ccnh	0
Ccng1	0
Ccnf	0
Ccne2	0
Ccndbp1	0
Ccnd3	0
Ccnd1	0
Ccnc	0
Ccnb1ip1	0
Ccnb1	0
Ccna2	0
Ccna1	0
Ccm2l	0
Ccm2	0
Ccl9	0
Ccl8	0
Ccl5	0
Ccl4	0
Ccl3	0
Ccl28	0
Ccl27a	0
Ccl24	0
Ccl22	0
Ccl21b	0
Ccl20	0
Ccl17	0
Ccl1	0
Il10	0
Il13	0
Il15	0
Il16	0
Oat	0
Ppih	0
Ppig	0
Ppif	0
Ppid	0
Ppic	0
Ppib	0
Ppia	0
Trp53inp2	0
Igf1r	0
Sytl1	0
Olfr632	0
Islr2	0
Nup153	0
1700037H04Rik	0
Grb2	0
Galnt2	0
Nup155	0
Ubc	0
Galnt3	0
Ubl5	0
Aqp7	0
Mapre2	0
Aqp2	0
Aqp3	0
Ubl3	0
Egfem1	0
BRDN0000737771	0
Gyltl1b	0
Clec4b2	0
Aqp8	0
Gm14393	0
Rcvrn	0
Enox2	0
Zadh2	0
Lhfpl4	0
Il1a	0
Il1b	0
Lhfpl1	0
Lhfpl2	0
Lhfpl3	0
Tax1bp1	0
Smim12	0
Tax1bp3	0
Tub	0
Rrp36	0
Smim15	0
Smim14	0
Olfr630	0
Smim19	0
Smim18	0
Kif13a	0
Spopl	0
Ik	0
Cldn17	0
Atp10a	0
Umodl1	0
Cox6a1	0
Dync1i1	0
Ccl5	0
Cox6a2	0
Tal1	0
Gm15080	0
Tal2	0
3830406C13Rik	0
Acsl3	0
Hnrnpr	0
Acsl1	0
Acsl6	0
Hnrnpu	0
Acsl4	0
Fam58b	0
Arl11	0
Arl10	0
Hnrnpk	0
Arl15	0
Arl14	0
Cd5l	0
Cd59b	0
Cd59a	0
Cd55	0
Cd53	0
Cd5	0
Cd48	0
Cd47	0
Cd46	0
Cd40lg	0
Cd40	0
Cd3eap	0
Cd3e	0
Cd37	0
Cd36	0
Cd34	0
Cd33	0
Cd302	0
Cd300ld	0
Cd300lb	0
Cd300e	0
Cd2bp2	0
Cd274	0
Cd27	0
Cd226	0
Cd22	0
Cd209g	0
Cd209f	0
Cd209e	0
Cd209d	0
Cd209c	0
Cd209b	0
Cd209a	0
Cd207	0
Cd200r4	0
Cd200r3	0
Cd200r2	0
Cd200r1	0
Cd2	0
Cd1d1	0
Cd180	0
Cd177	0
Cd164l2	0
Cd164	0
Cd163	0
Cd160	0
Cd151	0
Cd14	0
Cd109	0
Ccz1	0
Cct8l1	0
Cct8	0
Cct7	0
Cct6b	0
Cct5	0
Cct4	0
Cct3	0
Cct2	0
Ccser1	0
Ccsap	0
Ccs	0
Ccrn4l	0
Ccrl2	0
Ccr9	0
Ccr8	0
Ccr7	0
Ccr5	0
Ccr1l1	0
Ccpg1os	0
Ccpg1	0
Ccp110	0
Ccnyl1	0
Ccnt2	0
Ccnt1	0
Ccno	0
Ccnl1	0
Ccnk	0
Ccng2	0
Ccne1	0
Ccndbp1	0
Ccnd3	0
Ccnd2	0

C130079G13Rik	0	Ccl11	0	Tcp11	0	Ccnd1	0
C130026I21Rik	0	Ccl1	0	Arl16	0	Ccnc	0
C030039L03Rik	0	Cckbr	0	Neto1	0	Ccnb3	0
Bzw2	0	Cck	0	Hnrnpc	0	Ccnb2	0
Bzw1	0	Ccin	0	Hnrnpd	0	Ccnb1ip1	0
Bysl	0	Cchcr1	0	A830018L16Rik	0	Ccnb1	0
Bud13	0	Ccer1	0	Lims2	0	Ccna2	0
Bub3	0	Ccdc96	0	1700011I03Rik	0	Ccna1	0
Bub1b	0	Ccdc93	0	Prl3a1	0	Ccm2l	0
Bub1	0	Ccdc92	0	Trim12c	0	Ccm2	0
Btnl9	0	Ccdc91	0	Ccnk	0	Ccl9	0
Btnl4	0	Ccdc90b	0	Olfr1341	0	Ccl7	0
Btn1a1	0	Ccdc9	0	Khsrp	0	Ccl6	0
Btla	0	Ccdc88c	0	Olfr1436	0	Ccl5	0
Btk	0	Ccdc88a	0	Mylk	0	Ccl4	0
Btg3	0	Ccdc87	0	Stk26	0	Ccl28	0
Btg2	0	Ccdc85c	0	Stk25	0	Ccl25	0
Btg1-ps2	0	Ccdc84	0	Stk24	0	Ccl22	0
Btg1-ps1	0	Ccdc83	0	Gm14137	0	Ccl21b	0
Btg1	0	Ccdc82	0	Tnnc2	0	Ccl20	0
Btf3l4	0	Ccdc81	0	Olfr1434	0	Ccl2	0
Btf3	0	Ccdc79	0	Dda1	0	Ccl19	0
Btd	0	Ccdc78	0	Marcksl1	0	Ccl11	0
Btc	0	Ccdc77	0	Wbscr27	0	Ccl1	0
Btbd9	0	Ccdc74a	0	Wbscr22	0	Cck	0
Btbd8	0	Ccdc73	0	Rbm11	0	Ccin	0
Btbd7	0	Ccdc71l	0	Vmn1r5	0	Cchcr1	0
Btbd6	0	Ccdc70	0	Shisa3	0	Ccdc97	0
Btbd3	0	Ccdc66	0	Wbscr28	0	Ccdc96	0
Btbd17	0	Ccdc64	0	Cybrd1	0	Ccdc94	0
Btbd16	0	Ccdc62	0	Sgcb	0	Ccdc93	0
Btbd11	0	Ccdc61	0	Manf	0	Ccdc92	0
Btbd10	0	Ccdc60	0	Anks4b	0	Ccdc91	0
Btbd1	0	Ccdc6	0	Rsph14	0	Ccdc90b	0
Bsx	0	Ccdc59	0	Pin1	0	Ccdc9	0
Bst2	0	Ccdc55	0	Pin4	0	Ccdc89	0
Bst1	0	Ccdc51	0	Fzd1	0	Ccdc88b	0
Bspry	0	Ccdc50	0	Fzd2	0	Ccdc87	0
Bsph2	0	Ccdc43	0	Fzd3	0	Ccdc86	0
Bsph1	0	Ccdc42b	0	Fzd4	0	Ccdc85a	0
Bsnd	0	Ccdc40	0	Fzd5	0	Ccdc82	0
Bsg	0	Ccdc39	0	Fzd6	0	Ccdc81	0
Bsdc1	0	Ccdc38	0	Plxna3	0	Ccdc80	0
Bscl2	0	Ccdc37	0	Fzd9	0	Ccdc79	0
Brwd3	0	Ccdc34	0	Ninj2	0	Ccdc73	0
Brwd1	0	Ccdc30	0	Csf2rb2	0	Ccdc71l	0
Brsk2	0	Ccdc3	0	B430306N03Rik	0	Ccdc70	0
Brs3	0	Ccdc28b	0	Rpl35a	0	Ccdc68	0
Brpf3	0	Ccdc28a	0	Plxna4	0	Ccdc66	0
Brpf1	0	Ccdc27	0	Adamdec1	0	Ccdc65	0
Brms1l	0	Ccdc24	0	Cerkl	0	Ccdc64	0
Brix1	0	Ccdc23	0	Nnmt	0	Ccdc60	0
Brip1	0	Ccdc22	0	Pard3b	0	Ccdc6	0
Brinp3	0	Ccdc186	0	Usp27x	0	Ccdc59	0
Brinp2	0	Ccdc184	0	Pip	0	Ccdc57	0
Brinp1	0	Ccdc183	0	Guca2a	0	Ccdc55	0
Bricd5	0	Ccdc18	0	Guca2b	0	Ccdc54	0
Bri3	0	Ccdc178	0	Tjap1	0	Ccdc53	0
Brf2	0	Ccdc176	0	BRDN0000737576	0	Ccdc51	0
Brf1	0	Ccdc175	0	2410089E03Rik	0	Ccdc47	0
Bre	0	Ccdc174	0	Ccdc94	0	Ccdc42b	0
Brdt	0	Ccdc173	0	Ppp2r2d	0	Ccdc39	0
BRDN0000738379	0	Ccdc172	0	Ccdc96	0	Ccdc38	0
BRDN0000738378	0	Ccdc170	0	Ccdc97	0	Ccdc37	0
BRDN0000738377	0	Ccdc17	0	Krtap3-2	0	Ccdc36	0
BRDN0000738376	0	Ccdc169	0	Ccdc92	0	Ccdc34	0
BRDN0000738375	0	Ccdc166	0	Ccdc93	0	Ccdc33	0
BRDN0000738374	0	Ccdc163	0	Zfp169	0	Ccdc30	0
BRDN0000738373	0	Ccdc160	0	Pax2	0	Ccdc3	0
BRDN0000738372	0	Ccdc159	0	Stard13	0	Ccdc28b	0
BRDN0000738371	0	Ccdc158	0	Psmb9	0	Ccdc27	0
BRDN0000738370	0	Ccdc157	0	Psmb8	0	Ccdc22	0
BRDN0000738369	0	Ccdc155	0	Psmb7	0	Ccdc186	0
BRDN0000738368	0	Ccdc154	0	Psmb6	0	Ccdc185	0
BRDN0000738367	0	Ccdc153	0	Psmb5	0	Ccdc184	0
BRDN0000738366	0	Ccdc152	0	Psmb4	0	Ccdc18	0
BRDN0000738365	0	Ccdc151	0	Psmb2	0	Ccdc178	0
BRDN0000738363	0	Ccdc150	0	Psmb1	0	Ccdc177	0
BRDN0000738362	0	Ccdc15	0	Zpbp2	0	Ccdc176	0
BRDN0000738361	0	Ccdc146	0	Zfp799	0	Ccdc175	0
BRDN0000738360	0	Ccdc144b	0	Spdya	0	Ccdc174	0
BRDN0000738359	0	Ccdc137	0	Cd59a	0	Ccdc173	0

BRDN0000738357	0	Ccdc136	0	Zfp790	0	Ccdc170	0
BRDN0000738355	0	Ccdc134	0	Zfp791	0	Ccdc17	0
BRDN0000738354	0	Ccdc132	0	Ccng1	0	Ccdc169	0
BRDN0000738353	0	Ccdc130	0	Srpx	0	Ccdc167	0
BRDN0000738352	0	Ccdc13	0	Acadm	0	Ccdc160	0
BRDN0000738351	0	Ccdc129	0	Rab44	0	Ccdc159	0
BRDN0000738350	0	Ccdc127	0	Pgam5	0	Ccdc157	0
BRDN0000738349	0	Ccdc122	0	Cd226	0	Ccdc155	0
BRDN0000738348	0	Ccdc121	0	Trib1	0	Ccdc154	0
BRDN0000738346	0	Ccdc120	0	Trib2	0	Ccdc153	0
BRDN0000738345	0	Ccdc116	0	Trib3	0	Ccdc152	0
BRDN0000738344	0	Ccdc115	0	Ank2	0	Ccdc151	0
BRDN0000738343	0	Ccdc113	0	Prex2	0	Ccdc150	0
BRDN0000738342	0	Ccdc112	0	Kcnk9	0	Ccdc15	0
BRDN0000738341	0	Ccdc108	0	Mrps28	0	Ccdc149	0
BRDN0000738339	0	Ccdc107	0	Prex1	0	Ccdc146	0
BRDN0000738338	0	Ccdc106	0	Kcnk4	0	Ccdc141	0
BRDN0000738337	0	Ccdc103	0	Mrps27	0	Ccdc14	0
BRDN0000738336	0	Ccdc101	0	Mrps24	0	Ccdc138	0
BRDN0000738335	0	Ccbl2	0	Kcnk7	0	Ccdc136	0
BRDN0000738334	0	Ccar1	0	Slc7a10	0	Ccdc132	0
BRDN0000738333	0	Cc2d2a	0	Hist1h2ap	0	Ccdc130	0
BRDN0000738332	0	Cc2d1b	0	Kcnk2	0	Ccdc13	0
BRDN0000738331	0	Cc2d1a	0	Mrps21	0	Ccdc127	0
BRDN0000738330	0	Cbx8	0	Hist1h2ao	0	Ccdc122	0
BRDN0000738329	0	Cbx5	0	Hist1h2an	0	Ccdc12	0
BRDN0000738328	0	Cbx3	0	Hist1h2ai	0	Ccdc117	0
BRDN0000738325	0	Cbx2	0	Gphb5	0	Ccdc115	0
BRDN0000738324	0	Cbx1	0	Hist1h2ak	0	Ccdc114	0
BRDN0000738323	0	Cbwd1	0	Zfp423	0	Ccdc113	0
BRDN0000738322	0	Cbs	0	Hist1h2ae	0	Ccdc112	0
BRDN0000738321	0	Cbr3	0	Hist1h2ad	0	Ccdc110	0
BRDN0000738320	0	Cbr1	0	Hist1h2ag	0	Ccdc108	0
BRDN0000738319	0	Cbln4	0	Nat14	0	Ccdc107	0
BRDN0000738318	0	Cbln3	0	Zfp428	0	Ccdc106	0
BRDN0000738317	0	Cbln2	0	Zfp429	0	Ccdc103	0
BRDN0000738316	0	Cbln1	0	Hist1h2ac	0	Ccdc101	0
BRDN0000738315	0	Cblb	0	Smad9	0	Ccbl2	0
BRDN0000738314	0	Cbfb	0	Podxl	0	Ccbe1	0
BRDN0000738313	0	Cbfa2t3	0	Plekho1	0	Ccar2	0
BRDN0000738312	0	Cbfa2t2	0	Rab4b	0	Ccar1	0
BRDN0000738311	0	Cav3	0	Sepw1	0	Cc2d2a	0
BRDN0000738310	0	Cav2	0	Rab4a	0	Cc2d1a	0
BRDN0000738309	0	Cav1	0	BRDN0000738056	0	Cby1	0
BRDN0000738307	0	Catsperd	0	A230050P20Rik	0	Cbx8	0
BRDN0000738306	0	Catsperb	0	Gm1527	0	Cbx5	0
BRDN0000738305	0	Catsper4	0	Spice1	0	Cbx4	0
BRDN0000738304	0	Catsper3	0	Olfr74	0	Cbx1	0
BRDN0000738303	0	Catsper1	0	1700007B14Rik	0	Cbwd1	0
BRDN0000738302	0	Cat	0	Wfdc9	0	Cbs	0
BRDN0000738301	0	Casz1	0	Wfdc8	0	Cbr4	0
BRDN0000738300	0	Cass4	0	Frrs1	0	Cbr1	0
BRDN0000738298	0	Casr	0	Wfdc1	0	Cbln4	0
BRDN0000738297	0	Casq1	0	Smoc2	0	Cbln2	0
BRDN0000738296	0	Casp9	0	Wfdc3	0	Cbln1	0
BRDN0000738295	0	Casp8ap2	0	Wfdc2	0	Cbll1	0
BRDN0000738293	0	Casp8	0	Wfdc5	0	Cbl	0
BRDN0000738292	0	Casp7	0	Hpgd	0	Cbfa2t3	0
BRDN0000738291	0	Casp3	0	Gas2l1	0	Cbfa2t2	0
BRDN0000738289	0	Casp14	0	Glt8d2	0	Cav2	0
BRDN0000738288	0	Casp12	0	Ndufa6	0	Cav1	0
BRDN0000738287	0	Casp1	0	Ndufa7	0	Catsperd	0
BRDN0000738286	0	Caskin2	0	Ndufa4	0	Catsperb	0
BRDN0000738285	0	Caskin1	0	Smad7	0	Catsper4	0
BRDN0000738284	0	Casc5	0	Nicn1	0	Catsper3	0
BRDN0000738283	0	Casc4	0	Ndufa3	0	Catsper2	0
BRDN0000738282	0	Casc3	0	Btd	0	Catsper1	0
BRDN0000738281	0	Casc1	0	Ndufa1	0	Catip	0
BRDN0000738280	0	Cars2	0	Jun	0	Cat	0
BRDN0000738279	0	Cars	0	Psmg3	0	Cast	0
BRDN0000738278	0	Carm1	0	Ndufa8	0	Casr	0
BRDN0000738277	0	Carf	0	Smad1	0	Casq2	0
BRDN0000738275	0	Card9	0	Lrch4	0	Casq1	0
BRDN0000738274	0	Card14	0	Coro1c	0	Casp9	0
BRDN0000738273	0	Card10	0	Arhgap32	0	Casp8	0
BRDN0000738272	0	Car8	0	Cypt4	0	Casp7	0
BRDN0000738271	0	Car7	0	Lrch1	0	Casp14	0
BRDN0000738270	0	Car5b	0	Srpx2	0	Casp12	0
BRDN0000738269	0	Car4	0	Vegfb	0	Casp1	0
BRDN0000738268	0	Car14	0	Mettl17	0	Caskin1	0
BRDN0000738267	0	Car12	0	Atp6ap2	0	Cask	0
BRDN0000738265	0	Car10	0	Tpgs1	0	Casc5	0

BRDN0000738264	0	Car1	0	Cep95	0	Casc3	0
BRDN0000738263	0	Capzb	0	Tmprss12	0	Casc1	0
BRDN0000738262	0	Capza1	0	Gm4724	0	Cartpt	0
BRDN0000738261	0	Caprin2	0	Ect2	0	Cars2	0
BRDN0000738260	0	Caprin1	0	CK137956	0	Cars	0
BRDN0000738259	0	Capns2	0	Casp12	0	Carns1	0
BRDN0000738258	0	Capn9	0	Slc25a35	0	Carkd	0
BRDN0000738257	0	Capn8	0	Slc25a34	0	Carhsp1	0
BRDN0000738255	0	Capn7	0	Slc25a37	0	Carf	0
BRDN0000738254	0	Capn6	0	Slc25a36	0	Card9	0
BRDN0000738253	0	Capn5	0	Adam32	0	Card6	0
BRDN0000738251	0	Capn3	0	Slc25a30	0	Card11	0
BRDN0000738250	0	Capn2	0	Slc25a33	0	Car9	0
BRDN0000738249	0	Capn15	0	Slc25a32	0	Car7	0
BRDN0000738248	0	Capn13	0	Rpusd3	0	Car6	0
BRDN0000738247	0	Capn12	0	BRDN0000737630	0	Car5b	0
BRDN0000738246	0	Capn11	0	Gas2l3	0	Car3	0
BRDN0000738245	0	Capn10	0	Yae1d1	0	Car2	0
BRDN0000738244	0	Capn1	0	Slc25a38	0	Car15	0
BRDN0000738243	0	Capg	0	Adam39	0	Car14	0
BRDN0000738241	0	Cap2	0	Heatr6	0	Car12	0
BRDN0000738240	0	Cap1	0	Xpo1	0	Car11	0
BRDN0000738239	0	Canx	0	Naip6	0	Car10	0
BRDN0000738238	0	Camta2	0	Uvssa	0	Capzb	0
BRDN0000738237	0	Camta1	0	Heatr1	0	Capza2	0
BRDN0000738236	0	Camsap3	0	Naip1	0	Capza1	0
BRDN0000738235	0	Caml	0	Saxo2	0	Capsl	0
BRDN0000738234	0	Camkv	0	Tmprss15	0	Caps2	0
BRDN0000738233	0	Camkmt	0	Prr23a	0	Caprin2	0
BRDN0000738232	0	Camkk1	0	Heatr9	0	Caprin1	0
BRDN0000738231	0	Camk4	0	Lrrc8e	0	Capns2	0
BRDN0000738230	0	Camk2n1	0	Ispd	0	Capns1	0
BRDN0000738229	0	Camk2g	0	9530068E07Rik	0	Capn9	0
BRDN0000738228	0	Camk2d	0	Thsd7a	0	Capn7	0
BRDN0000738227	0	Camk2b	0	Rtn2	0	Capn3	0
BRDN0000738226	0	Camk2a	0	Ceacam15	0	Capn2	0
BRDN0000738225	0	Camk1d	0	Rtn3	0	Capn13	0
BRDN0000738224	0	Camk1	0	Gm21943	0	Capn11	0
BRDN0000738223	0	Calu	0	1700020A23Rik	0	Capn1	0
BRDN0000738222	0	Calr3	0	Zfp182	0	Capg	0
BRDN0000738221	0	Caln1	0	Olfr362	0	Cap2	0
BRDN0000738220	0	Calml4	0	Olfr479	0	Cap1	0
BRDN0000738219	0	Calm5	0	Def6	0	Canx	0
BRDN0000738218	0	Calm4	0	Ddx10	0	Cant1	0
BRDN0000738217	0	Calm3	0	Tipin	0	Cand2	0
BRDN0000738216	0	Calm2	0	Cr1l	0	Cand1	0
BRDN0000738214	0	Calm1	0	Olfr477	0	Camta1	0
BRDN0000738213	0	Calcrl	0	1700024P04Rik	0	Camsap3	0
BRDN0000738212	0	Calcr	0	Lrrc8d	0	Camsap2	0
BRDN0000738211	0	Calcb	0	Olfr472	0	Camsap1	0
BRDN0000738210	0	Calca	0	Gpr101	0	Caml	0
BRDN0000738209	0	Calb1	0	Eno3	0	Camkv	0
BRDN0000738208	0	Cage1	0	Ighmbp2	0	Camkmt	0
BRDN0000738206	0	Cadps	0	Arnt2	0	Camkk2	0
BRDN0000738205	0	Cadm4	0	Gpr107	0	Camkk1	0
BRDN0000738204	0	Cadm3	0	Gpr108	0	Camk4	0
BRDN0000738203	0	Cadm2	0	Psg29	0	Camk2n2	0
BRDN0000738202	0	Cadm1	0	Mrgpra1	0	Camk2n1	0
BRDN0000738201	0	Cad	0	Ippk	0	Camk2g	0
BRDN0000738200	0	Cacybp	0	Mrgpra4	0	Camk2d	0
BRDN0000738199	0	Cacul1	0	Timm13	0	Camk2b	0
BRDN0000738198	0	Cactin	0	Srsf5	0	Camk1g	0
BRDN0000738197	0	Cacng7	0	Cdkn2c	0	Camk1	0
BRDN0000738196	0	Cacng6	0	LOC100861615	0	Calr	0
BRDN0000738195	0	Cacng5	0	Srsf1	0	Caln1	0
BRDN0000738194	0	Cacng4	0	Bean1	0	Calml4	0
BRDN0000738193	0	Cacng2	0	Srsf3	0	Calml3	0
BRDN0000738192	0	Cacng1	0	Srsf2	0	Calm5	0
BRDN0000738191	0	Cacnb4	0	Rqcd1	0	Calm3	0
BRDN0000738190	0	Cacna2d4	0	Srsf9	0	Calm1	0
BRDN0000738189	0	Cacna2d3	0	Vmn2r18	0	Calhm1	0
BRDN0000738188	0	Cacna2d1	0	Sfrp1	0	Cald1	0
BRDN0000738187	0	Cacna1s	0	Gpm6a	0	Calcrl	0
BRDN0000738186	0	Cacna1i	0	BRDN0000737418	0	Calcoco1	0
BRDN0000738185	0	Cacna1g	0	Mapk3	0	Calca	0
BRDN0000738184	0	Cacna1e	0	Tube1	0	Calb2	0
BRDN0000738183	0	Cacna1d	0	BRDN0000737419	0	Cage1	0
BRDN0000738182	0	Cacna1c	0	Tll1	0	Cadps2	0
BRDN0000738181	0	Cacna1b	0	Tll2	0	Cadps	0
BRDN0000738180	0	Cacna1a	0	Slc38a6	0	Cadm4	0
BRDN0000738179	0	Cachd1	0	Slc38a1	0	Cadm2	0
BRDN0000738178	0	Cacfd1	0	Gm7073	0	Cadm1	0

BRDN0000738177	0	Cabyr	0	Slc38a3	0	Cad	0
BRDN0000738176	0	Cabs1	0	Tc2n	0	Cacng8	0
BRDN0000738175	0	Cabp7	0	Dars	0	Cacng7	0
BRDN0000738174	0	Cabp5	0	Lrrc6	0	Cacng5	0
BRDN0000738173	0	Cabp2	0	Atp6v0e	0	Cacng3	0
BRDN0000738172	0	Cabp1	0	Slc38a9	0	Cacng2	0
BRDN0000738171	0	Cables1	0	Lrrc1	0	Cacnb4	0
BRDN0000738170	0	Cabin1	0	Lrrc2	0	Cacnb3	0
BRDN0000738169	0	Cab39l	0	Lrrc3	0	Cacna2d4	0
BRDN0000738168	0	Caap1	0	Ldlrad2	0	Cacna2d3	0
BRDN0000738166	0	C8b	0	Ldlrad3	0	Cacna2d2	0
BRDN0000738164	0	C8a	0	Ldlrad4	0	Cacna2d1	0
BRDN0000738163	0	C87977	0	Prtg	0	Cacna1s	0
BRDN0000738162	0	C87499	0	Npm3	0	Cacna1i	0
BRDN0000738161	0	C87436	0	BRDN0000737415	0	Cacna1g	0
BRDN0000738160	0	C86695	0	1700021F05Rik	0	Cacna1e	0
BRDN0000738159	0	C78339	0	Rgmb	0	Cacna1c	0
BRDN0000738158	0	C77080	0	Cd300lf	0	Cacna1b	0
BRDN0000738156	0	C7	0	Olfr495	0	Cacfd1	0
BRDN0000738155	0	C5ar1	0	Rgma	0	Cabyr	0
BRDN0000738154	0	C530008M17Rik	0	Neurl1b	0	Cabs1	0
BRDN0000738153	0	C3ar1	0	Degs2	0	Cabp7	0
BRDN0000738152	0	C330027C09Rik	0	Neurl1a	0	Cabp5	0
BRDN0000738151	0	C330021F23Rik	0	Prps1	0	Cabp2	0
BRDN0000738150	0	C330007P06Rik	0	Prkca	0	Cables1	0
BRDN0000738149	0	C2cd5	0	2310057J18Rik	0	Cab39l	0
BRDN0000738148	0	C2cd4d	0	Prkcd	0	Cab39	0
BRDN0000738147	0	C2cd4c	0	Prkcg	0	Caap1	0
BRDN0000738146	0	C2cd4b	0	2010111I01Rik	0	C9	0
BRDN0000738145	0	C2cd4a	0	Htr6	0	C87499	0
BRDN0000738143	0	C2cd2	0	Ankle2	0	C87436	0
BRDN0000738142	0	C230052I12Rik	0	Tom1l2	0	C86695	0
BRDN0000738140	0	C2	0	Tmem181a	0	C78339	0
BRDN0000738139	0	C1s2	0	BRDN0000737479	0	C77370	0
BRDN0000738138	0	C1s1	0	Prkcq	0	C77080	0
BRDN0000738137	0	C1rl	0	BRDN0000737477	0	C5ar2	0
BRDN0000738136	0	C1ra	0	BRDN0000737474	0	C530008M17Rik	0
BRDN0000738135	0	C1qtnf6	0	BRDN0000737413	0	C4b	0
BRDN0000738133	0	C1qtnf5	0	BRDN0000737470	0	C330027C09Rik	0
BRDN0000738132	0	C1qtnf4	0	1700013F07Rik	0	C330021F23Rik	0
BRDN0000738131	0	C1qtnf3	0	9930111J21Rik1	0	C330018D20Rik	0
BRDN0000738130	0	C1qtnf2	0	2610524H06Rik	0	C330007P06Rik	0
BRDN0000738129	0	C1qtnf1	0	Tor1a	0	C2cd5	0
BRDN0000738128	0	C1ql4	0	Mprip	0	C2cd4d	0
BRDN0000738127	0	C1ql2	0	Chrm5	0	C2cd4c	0
BRDN0000738126	0	C1ql1	0	Chrm4	0	C2cd2	0
BRDN0000738124	0	C1qc	0	Chrm3	0	C230052I12Rik	0
BRDN0000738122	0	C1qbp	0	Chrm2	0	C2	0
BRDN0000738121	0	C1qb	0	Chrm1	0	C1s2	0
BRDN0000738119	0	C1qa	0	Ceacam10	0	C1s1	0
BRDN0000738118	0	C1galt1c1	0	Atg3	0	C1rl	0
BRDN0000738117	0	C1d	0	Myo18b	0	C1rb	0
BRDN0000738114	0	C130079G13Rik	0	Mslnl	0	C1ra	0
BRDN0000738113	0	C130060K24Rik	0	Zic2	0	C1qtnf9	0
BRDN0000738112	0	C130050O18Rik	0	Atg7	0	C1qtnf7	0
BRDN0000738111	0	C130026I21Rik	0	Cox8c	0	C1qtnf5	0
BRDN0000738110	0	C030039L03Rik	0	Copz1	0	C1qtnf4	0
BRDN0000738109	0	Bzw2	0	Syngr2	0	C1qtnf3	0
BRDN0000738108	0	Bzrap1	0	4930480E11Rik	0	C1qtnf2	0
BRDN0000738107	0	Bysl	0	Inpp1	0	C1ql4	0
BRDN0000738106	0	Bves	0	Amelx	0	C1ql1	0
BRDN0000738105	0	Bub3	0	Crcp	0	C1qbp	0
BRDN0000738102	0	Bub1b	0	Hpse	0	C1qb	0
BRDN0000738101	0	Bub1	0	Inpp5d	0	C1qa	0
BRDN0000738100	0	Btrc	0	Kcnh6	0	C1galt1c1	0
BRDN0000738099	0	Btnl9	0	Rragc	0	C130060K24Rik	0
BRDN0000738098	0	Btnl4	0	Prdm12	0	C130050O18Rik	0
BRDN0000738097	0	Btnl2	0	1700021F07Rik	0	C130026I21Rik	0
BRDN0000738095	0	Btnl1	0	Olfr1198	0	Bzw2	0
BRDN0000738094	0	Btn2a2	0	Olfr1199	0	Bzrap1	0
BRDN0000738093	0	Btn1a1	0	Lrrc8b	0	Bysl	0
BRDN0000738092	0	Btla	0	Cdv3	0	Bves	0
BRDN0000738091	0	Btk	0	Kcnh3	0	Bud13	0
BRDN0000738090	0	Btg3	0	A130010J15Rik	0	Bub3	0
BRDN0000738089	0	Btg2	0	Armcx6	0	Bub1b	0
BRDN0000738088	0	Btg1-ps2	0	Nova1	0	Btrc	0
BRDN0000738086	0	Btg1	0	Armcx4	0	Btnl9	0
BRDN0000738085	0	Btf3l4	0	Cstad	0	Btnl6	0
BRDN0000738084	0	Btf3	0	Olfr1195	0	Btnl4	0
BRDN0000738083	0	Btc	0	Olfr1196	0	Btnl2	0
BRDN0000738082	0	Btbd9	0	Hpgds	0	Btnl1	0
BRDN0000738081	0	Btbd7	0	Klf6	0	Btn2a2	0

BRDN0000738080	0
BRDN0000738079	0
BRDN0000738078	0
BRDN0000738077	0
BRDN0000738076	0
BRDN0000738075	0
BRDN0000738074	0
BRDN0000738073	0
BRDN0000738072	0
BRDN0000738071	0
BRDN0000738070	0
BRDN0000738069	0
BRDN0000738068	0
BRDN0000738067	0
BRDN0000738066	0
BRDN0000738065	0
BRDN0000738064	0
BRDN0000738063	0
BRDN0000738062	0
BRDN0000738061	0
BRDN0000738060	0
BRDN0000738058	0
BRDN0000738057	0
BRDN0000738056	0
BRDN0000738055	0
BRDN0000738054	0
BRDN0000738053	0
BRDN0000738052	0
BRDN0000738051	0
BRDN0000738050	0
BRDN0000738049	0
BRDN0000738048	0
BRDN0000738047	0
BRDN0000738046	0
BRDN0000738044	0
BRDN0000738043	0
BRDN0000738042	0
BRDN0000738041	0
BRDN0000738039	0
BRDN0000738037	0
BRDN0000738035	0
BRDN0000738034	0
BRDN0000738033	0
BRDN0000738032	0
BRDN0000738031	0
BRDN0000738030	0
BRDN0000738029	0
BRDN0000738028	0
BRDN0000738027	0
BRDN0000738026	0
BRDN0000738024	0
BRDN0000738023	0
BRDN0000738022	0
BRDN0000738021	0
BRDN0000738020	0
BRDN0000738019	0
BRDN0000738018	0
BRDN0000738017	0
BRDN0000738016	0
BRDN0000738015	0
BRDN0000738014	0
BRDN0000738013	0
BRDN0000738012	0
BRDN0000738011	0
BRDN0000738010	0
BRDN0000738009	0
BRDN0000738008	0
BRDN0000738007	0
BRDN0000738005	0
BRDN0000738004	0
BRDN0000738003	0
BRDN0000738002	0
BRDN0000738001	0
BRDN0000738000	0
BRDN0000737999	0
BRDN0000737998	0
BRDN0000737997	0
BRDN0000737996	0
BRDN0000737995	0
BRDN0000737994	0
BRDN0000737993	0
BRDN0000737992	0
Btbd3	0
Btbd2	0
Btbd18	0
Btbd17	0
Btbd16	0
Btbd11	0
Btbd10	0
Btbd1	0
Bst2	0
Bspry	0
Bsph2	0
Bsph1	0
Bsnd	0
Bsn	0
Brwd3	0
Brwd1	0
Brpf3	0
Brox	0
Brms1l	0
Brms1	0
Brk1	0
Brix1	0
Brinp3	0
Brinp2	0
Brinp1	0
Bri3bp	0
Bri3	0
Brf2	0
Bre	0
Brdt	0
BRDN0000738379	0
BRDN0000738378	0
BRDN0000738377	0
BRDN0000738376	0
BRDN0000738375	0
BRDN0000738374	0
BRDN0000738373	0
BRDN0000738372	0
BRDN0000738371	0
BRDN0000738370	0
BRDN0000738369	0
BRDN0000738368	0
BRDN0000738367	0
BRDN0000738366	0
BRDN0000738365	0
BRDN0000738364	0
BRDN0000738363	0
BRDN0000738362	0
BRDN0000738361	0
BRDN0000738360	0
BRDN0000738359	0
BRDN0000738358	0
BRDN0000738357	0
BRDN0000738356	0
BRDN0000738354	0
BRDN0000738353	0
BRDN0000738352	0
BRDN0000738350	0
BRDN0000738349	0
BRDN0000738348	0
BRDN0000738347	0
BRDN0000738346	0
BRDN0000738345	0
BRDN0000738344	0
BRDN0000738343	0
BRDN0000738342	0
BRDN0000738340	0
BRDN0000738339	0
BRDN0000738338	0
BRDN0000738337	0
BRDN0000738336	0
BRDN0000738335	0
BRDN0000738334	0
BRDN0000738333	0
BRDN0000738332	0
BRDN0000738331	0
BRDN0000738330	0
BRDN0000738329	0
BRDN0000738328	0
BRDN0000738327	0
BRDN0000738326	0
BRDN0000738325	0
Klf5	0
Klf4	0
Klf3	0
Klf2	0
Klf1	0
Cpne2	0
Homer1	0
Homer3	0
Homer2	0
Slitrk6	0
Klf9	0
Klf8	0
Arntl	0
Gm5662	0
Zc3h11a	0
Sytl4	0
Pex5l	0
Aebp2	0
Ncapd2	0
Ncapd3	0
Tph1	0
Ehmt1	0
Ang	0
Cypt15	0
Nt5c1b	0
Rrm1	0
Vipr1	0
Gm11711	0
Nup62	0
Prr18	0
Pcdhb1	0
Kif3b	0
Olfr1228	0
Olfr1229	0
Ascl3	0
Vmn2r21	0
Nlrp1b	0
Olfr1222	0
Olfr1223	0
1810030O07Rik	0
P2ry2	0
D6Wsu163e	0
Art2b	0
Rapsn	0
Twistnb	0
Ccdc181	0
Csta1	0
Ccdc183	0
Ccdc185	0
Ccdc184	0
Ccdc186	0
Plac9a	0
Zfp658	0
Zfp652	0
Zfp651	0
Thtpa	0
Haghl	0
Zfp655	0
Zfp654	0
Adgre1	0
1700012B07Rik	0
Pced1b	0
Zswim2	0
Olfr294	0
Sema7a	0
Gad1	0
Gad2	0
Gm1123	0
Gm266	0
Rhoj	0
G630090E17Rik	0
Pgrmc2	0
Taldo1	0
Ms4a18	0
3110057O12Rik	0
Elovl5	0
Nipa2	0
Tdpoz3	0
Tdpoz2	0
Tdpoz1	0
Rpain	0
Ajuba	0
Btg2	0
Btg1-ps1	0
Btf3l4	0
Btf3	0
Btd	0
Btc	0
Btbd9	0
Btbd8	0
Btbd7	0
Btbd6	0
Btbd3	0
Btbd18	0
Btbd17	0
Btbd11	0
Btbd10	0
Btbd1	0
Bst2	0
Bst1	0
Bspry	0
Bsph2	0
Bsph1	0
Bsnd	0
Bsg	0
Bsdc1	0
Bscl2	0
Brwd3	0
Brsk2	0
Brs3	0
Brox	0
Brms1l	0
Brms1	0
Brk1	0
Brix1	0
Brinp3	0
Bricd5	0
Bri3bp	0
Bri3	0
Brf2	0
Brf1	0
BRDN0000738379	0
BRDN0000738378	0
BRDN0000738377	0
BRDN0000738376	0
BRDN0000738375	0
BRDN0000738374	0
BRDN0000738373	0
BRDN0000738372	0
BRDN0000738371	0
BRDN0000738370	0
BRDN0000738369	0
BRDN0000738368	0
BRDN0000738367	0
BRDN0000738366	0
BRDN0000738365	0
BRDN0000738364	0
BRDN0000738363	0
BRDN0000738362	0
BRDN0000738361	0
BRDN0000738360	0
BRDN0000738359	0
BRDN0000738358	0
BRDN0000738357	0
BRDN0000738356	0
BRDN0000738354	0
BRDN0000738353	0
BRDN0000738352	0
BRDN0000738351	0
BRDN0000738350	0
BRDN0000738349	0
BRDN0000738348	0
BRDN0000738346	0
BRDN0000738345	0
BRDN0000738344	0
BRDN0000738343	0
BRDN0000738342	0
BRDN0000738341	0
BRDN0000738340	0
BRDN0000738339	0
BRDN0000738337	0
BRDN0000738336	0
BRDN0000738335	0
BRDN0000738334	0

BRDN0000737991	0
BRDN0000737990	0
BRDN0000737989	0
BRDN0000737988	0
BRDN0000737987	0
BRDN0000737986	0
BRDN0000737985	0
BRDN0000737984	0
BRDN0000737983	0
BRDN0000737982	0
BRDN0000737979	0
BRDN0000737977	0
BRDN0000737976	0
BRDN0000737974	0
BRDN0000737973	0
BRDN0000737972	0
BRDN0000737971	0
BRDN0000737970	0
BRDN0000737969	0
BRDN0000737968	0
BRDN0000737967	0
BRDN0000737966	0
BRDN0000737965	0
BRDN0000737964	0
BRDN0000737963	0
BRDN0000737962	0
BRDN0000737961	0
BRDN0000737960	0
BRDN0000737959	0
BRDN0000737958	0
BRDN0000737957	0
BRDN0000737956	0
BRDN0000737955	0
BRDN0000737954	0
BRDN0000737953	0
BRDN0000737952	0
BRDN0000737951	0
BRDN0000737950	0
BRDN0000737949	0
BRDN0000737948	0
BRDN0000737947	0
BRDN0000737946	0
BRDN0000737945	0
BRDN0000737944	0
BRDN0000737943	0
BRDN0000737942	0
BRDN0000737940	0
BRDN0000737939	0
BRDN0000737938	0
BRDN0000737937	0
BRDN0000737936	0
BRDN0000737935	0
BRDN0000737934	0
BRDN0000737933	0
BRDN0000737932	0
BRDN0000737931	0
BRDN0000737930	0
BRDN0000737929	0
BRDN0000737928	0
BRDN0000737927	0
BRDN0000737926	0
BRDN0000737925	0
BRDN0000737924	0
BRDN0000737923	0
BRDN0000737922	0
BRDN0000737921	0
BRDN0000737920	0
BRDN0000737919	0
BRDN0000737918	0
BRDN0000737917	0
BRDN0000737916	0
BRDN0000737914	0
BRDN0000737913	0
BRDN0000737912	0
BRDN0000737911	0
BRDN0000737910	0
BRDN0000737909	0
BRDN0000737908	0
BRDN0000737907	0
BRDN0000737906	0
BRDN0000737905	0
BRDN0000737904	0
BRDN0000738324	0
BRDN0000738323	0
BRDN0000738322	0
BRDN0000738321	0
BRDN0000738320	0
BRDN0000738319	0
BRDN0000738318	0
BRDN0000738317	0
BRDN0000738316	0
BRDN0000738313	0
BRDN0000738312	0
BRDN0000738311	0
BRDN0000738310	0
BRDN0000738309	0
BRDN0000738308	0
BRDN0000738307	0
BRDN0000738305	0
BRDN0000738304	0
BRDN0000738303	0
BRDN0000738302	0
BRDN0000738301	0
BRDN0000738300	0
BRDN0000738299	0
BRDN0000738298	0
BRDN0000738297	0
BRDN0000738296	0
BRDN0000738294	0
BRDN0000738293	0
BRDN0000738292	0
BRDN0000738291	0
BRDN0000738290	0
BRDN0000738289	0
BRDN0000738287	0
BRDN0000738286	0
BRDN0000738284	0
BRDN0000738282	0
BRDN0000738281	0
BRDN0000738280	0
BRDN0000738279	0
BRDN0000738278	0
BRDN0000738277	0
BRDN0000738276	0
BRDN0000738275	0
BRDN0000738274	0
BRDN0000738273	0
BRDN0000738272	0
BRDN0000738271	0
BRDN0000738270	0
BRDN0000738268	0
BRDN0000738267	0
BRDN0000738266	0
BRDN0000738265	0
BRDN0000738264	0
BRDN0000738263	0
BRDN0000738262	0
BRDN0000738260	0
BRDN0000738259	0
BRDN0000738258	0
BRDN0000738257	0
BRDN0000738256	0
BRDN0000738255	0
BRDN0000738254	0
BRDN0000738253	0
BRDN0000738252	0
BRDN0000738251	0
BRDN0000738249	0
BRDN0000738248	0
BRDN0000738247	0
BRDN0000738246	0
BRDN0000738245	0
BRDN0000738244	0
BRDN0000738243	0
BRDN0000738242	0
BRDN0000738241	0
BRDN0000738240	0
BRDN0000738239	0
BRDN0000738238	0
BRDN0000738237	0
BRDN0000738236	0
BRDN0000738235	0
BRDN0000738234	0
BRDN0000738233	0
Ms4a15	0
Tdpoz5	0
Tdpoz4	0
Spats1	0
Trmt11	0
Trmt12	0
Spats2	0
Cdh20	0
Prrc2b	0
Tnrc18	0
Cdh23	0
Cdh24	0
Pdcd6ip	0
Fam183b	0
Tceal3	0
Tceal1	0
Tceal7	0
Tceal6	0
Tceal5	0
Olfr32	0
Txnl4a	0
Olfr30	0
Olfr31	0
Six5	0
Rhoh	0
Parp8	0
Parp9	0
Parp4	0
Parp6	0
Slurp1	0
Parp3	0
Zcchc9	0
Zcchc8	0
Srsf11	0
Nrbf2	0
Cd38	0
Zcchc3	0
Enc1	0
Nkain4	0
Nkain3	0
Zcchc6	0
Zcchc5	0
Hgd	0
Lrp10	0
Lrp11	0
Tox	0
Rdh5	0
Cyp2j13	0
Cypt1	0
Gm17019	0
Cypt2	0
Dhdds	0
Boll	0
Tas1r1	0
Cnfn	0
Vti1a	0
Klhdc8b	0
Vti1b	0
Fam169a	0
Myh7b	0
Aco2	0
Smr2	0
Oit1	0
l7Rn6	0
Oit3	0
Klhl36	0
Klhl34	0
Klhl35	0
Klhl33	0
Klhl30	0
Klhl31	0
Gtf2ird1	0
Gtf2ird2	0
Ros1	0
Cass4	0
Tead3	0
Tead2	0
Tead1	0
Mtdh	0
Tcam1	0
Hmgcs1	0
Cir1	0
BRDN0000738333	0
BRDN0000738332	0
BRDN0000738331	0
BRDN0000738330	0
BRDN0000738329	0
BRDN0000738328	0
BRDN0000738327	0
BRDN0000738326	0
BRDN0000738325	0
BRDN0000738324	0
BRDN0000738323	0
BRDN0000738322	0
BRDN0000738321	0
BRDN0000738320	0
BRDN0000738319	0
BRDN0000738318	0
BRDN0000738317	0
BRDN0000738316	0
BRDN0000738315	0
BRDN0000738314	0
BRDN0000738313	0
BRDN0000738311	0
BRDN0000738310	0
BRDN0000738309	0
BRDN0000738308	0
BRDN0000738307	0
BRDN0000738305	0
BRDN0000738304	0
BRDN0000738303	0
BRDN0000738302	0
BRDN0000738301	0
BRDN0000738300	0
BRDN0000738299	0
BRDN0000738298	0
BRDN0000738297	0
BRDN0000738296	0
BRDN0000738293	0
BRDN0000738292	0
BRDN0000738291	0
BRDN0000738290	0
BRDN0000738289	0
BRDN0000738288	0
BRDN0000738287	0
BRDN0000738286	0
BRDN0000738285	0
BRDN0000738284	0
BRDN0000738283	0
BRDN0000738282	0
BRDN0000738281	0
BRDN0000738280	0
BRDN0000738279	0
BRDN0000738278	0
BRDN0000738277	0
BRDN0000738276	0
BRDN0000738275	0
BRDN0000738274	0
BRDN0000738273	0
BRDN0000738272	0
BRDN0000738271	0
BRDN0000738270	0
BRDN0000738269	0
BRDN0000738268	0
BRDN0000738267	0
BRDN0000738266	0
BRDN0000738265	0
BRDN0000738264	0
BRDN0000738263	0
BRDN0000738262	0
BRDN0000738259	0
BRDN0000738258	0
BRDN0000738257	0
BRDN0000738256	0
BRDN0000738255	0
BRDN0000738254	0
BRDN0000738253	0
BRDN0000738252	0
BRDN0000738251	0
BRDN0000738249	0
BRDN0000738248	0
BRDN0000738247	0
BRDN0000738245	0
BRDN0000738244	0

BRDN0000737903	0	BRDN0000738232	0	Fjx1	0	BRDN0000738243	0
BRDN0000737902	0	BRDN0000738231	0	Thg1l	0	BRDN0000738242	0
BRDN0000737901	0	BRDN0000738230	0	Stam2	0	BRDN0000738240	0
BRDN0000737900	0	BRDN0000738229	0	4921530L21Rik	0	BRDN0000738239	0
BRDN0000737899	0	BRDN0000738228	0	Azin2	0	BRDN0000738238	0
BRDN0000737898	0	BRDN0000738227	0	Hemgn	0	BRDN0000738237	0
BRDN0000737897	0	BRDN0000738226	0	Sept5	0	BRDN0000738236	0
BRDN0000737896	0	BRDN0000738225	0	Lrp1b	0	BRDN0000738235	0
BRDN0000737895	0	BRDN0000738224	0	Cldn14	0	BRDN0000738234	0
BRDN0000737894	0	BRDN0000738221	0	Rad1	0	BRDN0000738233	0
BRDN0000737892	0	BRDN0000738220	0	Sept4	0	BRDN0000738231	0
BRDN0000737891	0	BRDN0000738219	0	Serpinb1a	0	BRDN0000738230	0
BRDN0000737890	0	BRDN0000738218	0	Ccdc42b	0	BRDN0000738229	0
BRDN0000737889	0	BRDN0000738217	0	Chid1	0	BRDN0000738228	0
BRDN0000737888	0	BRDN0000738216	0	Serpinb1b	0	BRDN0000738227	0
BRDN0000737887	0	BRDN0000738215	0	Cideb	0	BRDN0000738226	0
BRDN0000737886	0	BRDN0000738214	0	Ewsr1	0	BRDN0000738223	0
BRDN0000737885	0	BRDN0000738213	0	Serpine2	0	BRDN0000738222	0
BRDN0000737884	0	BRDN0000738212	0	Kmt2c	0	BRDN0000738221	0
BRDN0000737883	0	BRDN0000738211	0	Cd300lh	0	BRDN0000738220	0
BRDN0000737882	0	BRDN0000738210	0	Pcnxl4	0	BRDN0000738219	0
BRDN0000737881	0	BRDN0000738209	0	Ahcyl1	0	BRDN0000738218	0
BRDN0000737880	0	BRDN0000738208	0	Pcnxl2	0	BRDN0000738217	0
BRDN0000737879	0	BRDN0000738207	0	Kmt2e	0	BRDN0000738216	0
BRDN0000737878	0	BRDN0000738206	0	Kmt2d	0	BRDN0000738214	0
BRDN0000737877	0	BRDN0000738205	0	Gtpbp3	0	BRDN0000738213	0
BRDN0000737876	0	BRDN0000738204	0	BC005624	0	BRDN0000738212	0
BRDN0000737875	0	BRDN0000738203	0	Sh2d1a	0	BRDN0000738211	0
BRDN0000737874	0	BRDN0000738201	0	Fbxo16	0	BRDN0000738210	0
BRDN0000737873	0	BRDN0000738199	0	Fbxo17	0	BRDN0000738209	0
BRDN0000737872	0	BRDN0000738198	0	Fbxo15	0	BRDN0000738208	0
BRDN0000737871	0	BRDN0000738197	0	Clcf1	0	BRDN0000738206	0
BRDN0000737870	0	BRDN0000738196	0	Ube2z	0	BRDN0000738205	0
BRDN0000737869	0	BRDN0000738195	0	BRDN0000737919	0	BRDN0000738204	0
BRDN0000737868	0	BRDN0000738194	0	BRDN0000737918	0	BRDN0000738202	0
BRDN0000737867	0	BRDN0000738193	0	Rilpl1	0	BRDN0000738201	0
BRDN0000737866	0	BRDN0000738192	0	Fdx1l	0	BRDN0000738200	0
BRDN0000737865	0	BRDN0000738191	0	BRDN0000737917	0	BRDN0000738199	0
BRDN0000737864	0	BRDN0000738190	0	Rilpl2	0	BRDN0000738198	0
BRDN0000737863	0	BRDN0000738189	0	Gm5113	0	BRDN0000738197	0
BRDN0000737861	0	BRDN0000738188	0	Mapk8ip1	0	BRDN0000738196	0
BRDN0000737860	0	BRDN0000738184	0	Gm5111	0	BRDN0000738195	0
BRDN0000737859	0	BRDN0000738182	0	Slc35f5	0	BRDN0000738194	0
BRDN0000737858	0	BRDN0000738181	0	Olfr399	0	BRDN0000738193	0
BRDN0000737856	0	BRDN0000738178	0	BRDN0000738152	0	BRDN0000738192	0
BRDN0000737855	0	BRDN0000738177	0	BRDN0000737646	0	BRDN0000738191	0
BRDN0000737854	0	BRDN0000738176	0	Slc35f1	0	BRDN0000738190	0
BRDN0000737853	0	BRDN0000738175	0	BRDN0000738155	0	BRDN0000738189	0
BRDN0000737852	0	BRDN0000738173	0	Gtpbp2	0	BRDN0000738188	0
BRDN0000737851	0	BRDN0000738172	0	Slc35f2	0	BRDN0000738187	0
BRDN0000737850	0	BRDN0000738171	0	Olfr390	0	BRDN0000738185	0
BRDN0000737849	0	BRDN0000738170	0	Ak2	0	BRDN0000738184	0
BRDN0000737848	0	BRDN0000738168	0	Ak1	0	BRDN0000738182	0
BRDN0000737847	0	BRDN0000738167	0	Olfr393	0	BRDN0000738181	0
BRDN0000737846	0	BRDN0000738166	0	Ak7	0	BRDN0000738180	0
BRDN0000737845	0	BRDN0000738165	0	Oxsm	0	BRDN0000738179	0
BRDN0000737844	0	BRDN0000738164	0	Ak5	0	BRDN0000738178	0
BRDN0000737843	0	BRDN0000738163	0	Olfr397	0	BRDN0000738177	0
BRDN0000737842	0	BRDN0000738162	0	Brdt	0	BRDN0000738176	0
BRDN0000737840	0	BRDN0000738161	0	Kctd10	0	BRDN0000738175	0
BRDN0000737839	0	BRDN0000738160	0	Fabp2	0	BRDN0000738174	0
BRDN0000737838	0	BRDN0000738159	0	Lzts2	0	BRDN0000738173	0
BRDN0000737837	0	BRDN0000738158	0	Lzts3	0	BRDN0000738172	0
BRDN0000737836	0	BRDN0000738157	0	Lzts1	0	BRDN0000738171	0
BRDN0000737835	0	BRDN0000738156	0	Acot13	0	BRDN0000738170	0
BRDN0000737834	0	BRDN0000738155	0	Unc5c	0	BRDN0000738168	0
BRDN0000737833	0	BRDN0000738154	0	4930453N24Rik	0	BRDN0000738167	0
BRDN0000737832	0	BRDN0000738153	0	Bsx	0	BRDN0000738166	0
BRDN0000737831	0	BRDN0000738152	0	Cx3cr1	0	BRDN0000738165	0
BRDN0000737830	0	BRDN0000738151	0	BRDN0000738272	0	BRDN0000738164	0
BRDN0000737829	0	BRDN0000738150	0	Mical1	0	BRDN0000738162	0
BRDN0000737828	0	BRDN0000738149	0	Tsc22d1	0	BRDN0000738161	0
BRDN0000737827	0	BRDN0000738148	0	Tsc22d3	0	BRDN0000738160	0
BRDN0000737826	0	BRDN0000738147	0	Uts2b	0	BRDN0000738158	0
BRDN0000737825	0	BRDN0000738146	0	Tsc22d4	0	BRDN0000738157	0
BRDN0000737824	0	BRDN0000738145	0	Gm21002	0	BRDN0000738155	0
BRDN0000737823	0	BRDN0000738144	0	Bsn	0	BRDN0000738154	0
BRDN0000737822	0	BRDN0000738143	0	Mical3	0	BRDN0000738153	0
BRDN0000737821	0	BRDN0000738142	0	Agtpbp1	0	BRDN0000738152	0
BRDN0000737820	0	BRDN0000738141	0	Dennd1c	0	BRDN0000738151	0
BRDN0000737819	0	BRDN0000738140	0	Dennd1b	0	BRDN0000738150	0
BRDN0000737818	0	BRDN0000738139	0	Dennd1a	0	BRDN0000738149	0

BRDN0000737816	0	BRDN0000738138	0	Vmn1r72	0	BRDN0000738148	0
BRDN0000737815	0	BRDN0000738137	0	Tacc2	0	BRDN0000738147	0
BRDN0000737814	0	BRDN0000738136	0	Hirip3	0	BRDN0000738145	0
BRDN0000737812	0	BRDN0000738135	0	Rps13	0	BRDN0000738144	0
BRDN0000737811	0	BRDN0000738134	0	Rps12	0	BRDN0000738143	0
BRDN0000737810	0	BRDN0000738133	0	Rps11	0	BRDN0000738142	0
BRDN0000737809	0	BRDN0000738132	0	Nap1l3	0	BRDN0000738141	0
BRDN0000737808	0	BRDN0000738130	0	Rps17	0	BRDN0000738140	0
BRDN0000737807	0	BRDN0000738129	0	Rps16	0	BRDN0000738139	0
BRDN0000737806	0	BRDN0000738128	0	Rps15	0	BRDN0000738138	0
BRDN0000737805	0	BRDN0000738127	0	Rps14	0	BRDN0000738137	0
BRDN0000737804	0	BRDN0000738125	0	Gla	0	BRDN0000738136	0
BRDN0000737802	0	BRDN0000738124	0	Rps19	0	BRDN0000738135	0
BRDN0000737801	0	BRDN0000738123	0	Rps18	0	BRDN0000738134	0
BRDN0000737800	0	BRDN0000738122	0	Rcl1	0	BRDN0000738132	0
BRDN0000737799	0	BRDN0000738121	0	Cercam	0	BRDN0000738131	0
BRDN0000737798	0	BRDN0000738120	0	Wdr62	0	BRDN0000738129	0
BRDN0000737797	0	BRDN0000738119	0	Zfp764	0	BRDN0000738127	0
BRDN0000737796	0	BRDN0000738118	0	Lin7b	0	BRDN0000738126	0
BRDN0000737794	0	BRDN0000738117	0	Sgca	0	BRDN0000738125	0
BRDN0000737793	0	BRDN0000738116	0	Wdr63	0	BRDN0000738124	0
BRDN0000737792	0	BRDN0000738115	0	BRDN0000738348	0	BRDN0000738123	0
BRDN0000737791	0	BRDN0000738114	0	Gls	0	BRDN0000738122	0
BRDN0000737790	0	BRDN0000738112	0	Gm10767	0	BRDN0000738121	0
BRDN0000737789	0	BRDN0000738111	0	Gm10094	0	BRDN0000738120	0
BRDN0000737788	0	BRDN0000738110	0	Tff1	0	BRDN0000738119	0
BRDN0000737787	0	BRDN0000738109	0	Nrn1l	0	BRDN0000738118	0
BRDN0000737786	0	BRDN0000738108	0	Wdr61	0	BRDN0000738117	0
BRDN0000737785	0	BRDN0000738107	0	Crlf3	0	BRDN0000738116	0
BRDN0000737784	0	BRDN0000738105	0	Crlf1	0	BRDN0000738115	0
BRDN0000737783	0	BRDN0000738104	0	Cd200r4	0	BRDN0000738114	0
BRDN0000737782	0	BRDN0000738103	0	Mpp1	0	BRDN0000738112	0
BRDN0000737781	0	BRDN0000738102	0	Kng1	0	BRDN0000738111	0
BRDN0000737780	0	BRDN0000738101	0	Tcea2	0	BRDN0000738109	0
BRDN0000737779	0	BRDN0000738100	0	Mpp6	0	BRDN0000738108	0
BRDN0000737778	0	BRDN0000738098	0	Zfp131	0	BRDN0000738106	0
BRDN0000737777	0	BRDN0000738097	0	Lppr4	0	BRDN0000738103	0
BRDN0000737776	0	BRDN0000738096	0	Lppr3	0	BRDN0000738102	0
BRDN0000737775	0	BRDN0000738095	0	Zxdc	0	BRDN0000738101	0
BRDN0000737774	0	BRDN0000738093	0	Lppr1	0	BRDN0000738100	0
BRDN0000737773	0	BRDN0000738092	0	Aes	0	BRDN0000738099	0
BRDN0000737772	0	BRDN0000738090	0	Col1a2	0	BRDN0000738098	0
BRDN0000737771	0	BRDN0000738089	0	Il13ra1	0	BRDN0000738097	0
BRDN0000737770	0	BRDN0000738088	0	Vps72	0	BRDN0000738096	0
BRDN0000737769	0	BRDN0000738087	0	Tuft1	0	BRDN0000738095	0
BRDN0000737767	0	BRDN0000738086	0	Dgcr8	0	BRDN0000738094	0
BRDN0000737766	0	BRDN0000738085	0	Ocm	0	BRDN0000738093	0
BRDN0000737765	0	BRDN0000738083	0	Fgf9	0	BRDN0000738092	0
BRDN0000737764	0	BRDN0000738082	0	Lrpap1	0	BRDN0000738091	0
BRDN0000737763	0	BRDN0000738081	0	Enpp6	0	BRDN0000738090	0
BRDN0000737762	0	BRDN0000738080	0	Dgcr2	0	BRDN0000738089	0
BRDN0000737761	0	BRDN0000738078	0	Rhof	0	BRDN0000738087	0
BRDN0000737760	0	BRDN0000738077	0	Otof	0	BRDN0000738086	0
BRDN0000737758	0	BRDN0000738076	0	Otog	0	BRDN0000738085	0
BRDN0000737757	0	BRDN0000738075	0	Olfr1507	0	BRDN0000738084	0
BRDN0000737756	0	BRDN0000738074	0	Olfr1506	0	BRDN0000738083	0
BRDN0000737755	0	BRDN0000738073	0	Olfr1505	0	BRDN0000738082	0
BRDN0000737754	0	BRDN0000738072	0	Olfr1504	0	BRDN0000738080	0
BRDN0000737753	0	BRDN0000738071	0	Olfr1502	0	BRDN0000738079	0
BRDN0000737752	0	BRDN0000738070	0	Olfr1501	0	BRDN0000738078	0
BRDN0000737751	0	BRDN0000738068	0	Olfr1500	0	BRDN0000738076	0
BRDN0000737750	0	BRDN0000738067	0	Tep1	0	BRDN0000738075	0
BRDN0000737749	0	BRDN0000738066	0	Otor	0	BRDN0000738074	0
BRDN0000737748	0	BRDN0000738065	0	Otos	0	BRDN0000738073	0
BRDN0000737747	0	BRDN0000738064	0	1810013L24Rik	0	BRDN0000738071	0
BRDN0000737746	0	BRDN0000738063	0	Olfr1509	0	BRDN0000738070	0
BRDN0000737745	0	BRDN0000738062	0	Nup210l	0	BRDN0000738069	0
BRDN0000737743	0	BRDN0000738061	0	Trmt61a	0	BRDN0000738068	0
BRDN0000737742	0	BRDN0000738060	0	Lrfn3	0	BRDN0000738067	0
BRDN0000737741	0	BRDN0000738059	0	Dcaf12	0	BRDN0000738066	0
BRDN0000737740	0	BRDN0000738058	0	Get4	0	BRDN0000738065	0
BRDN0000737739	0	BRDN0000738057	0	Lsp1	0	BRDN0000738064	0
BRDN0000737738	0	BRDN0000738056	0	Gm11710	0	BRDN0000738063	0
BRDN0000737737	0	BRDN0000738055	0	1810065E05Rik	0	BRDN0000738062	0
BRDN0000737736	0	BRDN0000738054	0	C8b	0	BRDN0000738061	0
BRDN0000737735	0	BRDN0000738052	0	Olfr135	0	BRDN0000738060	0
BRDN0000737733	0	BRDN0000738051	0	Olfr136	0	BRDN0000738059	0
BRDN0000737732	0	BRDN0000738050	0	Olfr641	0	BRDN0000738058	0
BRDN0000737731	0	BRDN0000738049	0	Olfr646	0	BRDN0000738057	0
BRDN0000737730	0	BRDN0000738048	0	Ovol2	0	BRDN0000738056	0
BRDN0000737729	0	BRDN0000738047	0	Ovol1	0	BRDN0000738055	0
BRDN0000737728	0	BRDN0000738046	0	Dkc1	0	BRDN0000738053	0

BRDN0000737726	0	BRDN0000738044	0	BRDN0000738334	0	BRDN0000738052	0
BRDN0000737725	0	BRDN0000738043	0	BRDN0000738335	0	BRDN0000738051	0
BRDN0000737724	0	BRDN0000738042	0	Olfr648	0	BRDN0000738050	0
BRDN0000737723	0	BRDN0000738040	0	Olfr649	0	BRDN0000738049	0
BRDN0000737722	0	BRDN0000738039	0	Olfr139	0	BRDN0000738048	0
BRDN0000737721	0	BRDN0000738038	0	BRDN0000738332	0	BRDN0000738047	0
BRDN0000737720	0	BRDN0000738037	0	BRDN0000738333	0	BRDN0000738046	0
BRDN0000737719	0	BRDN0000738036	0	Tm2d1	0	BRDN0000738044	0
BRDN0000737717	0	BRDN0000738035	0	Tm2d2	0	BRDN0000738043	0
BRDN0000737716	0	BRDN0000738034	0	Tm2d3	0	BRDN0000738042	0
BRDN0000737715	0	BRDN0000738033	0	Myt1l	0	BRDN0000738041	0
BRDN0000737713	0	BRDN0000738032	0	Dcaf15	0	BRDN0000738040	0
BRDN0000737712	0	BRDN0000738031	0	Nr4a2	0	BRDN0000738039	0
BRDN0000737711	0	BRDN0000738030	0	Nr4a3	0	BRDN0000738038	0
BRDN0000737710	0	BRDN0000738029	0	Camk4	0	BRDN0000738037	0
BRDN0000737709	0	BRDN0000738028	0	Fbxl5	0	BRDN0000738036	0
BRDN0000737708	0	BRDN0000738027	0	Fbxl4	0	BRDN0000738035	0
BRDN0000737707	0	BRDN0000738026	0	Fbxl7	0	BRDN0000738033	0
BRDN0000737706	0	BRDN0000738025	0	Tfip11	0	BRDN0000738032	0
BRDN0000737705	0	BRDN0000738024	0	Bgn	0	BRDN0000738031	0
BRDN0000737704	0	BRDN0000738023	0	Erf	0	BRDN0000738029	0
BRDN0000737703	0	BRDN0000738022	0	I830012O16Rik	0	BRDN0000738028	0
BRDN0000737702	0	BRDN0000738021	0	Idnk	0	BRDN0000738027	0
BRDN0000737701	0	BRDN0000738019	0	Erh	0	BRDN0000738026	0
BRDN0000737700	0	BRDN0000738018	0	Akr1c21	0	BRDN0000738025	0
BRDN0000737699	0	BRDN0000738017	0	Cd34	0	BRDN0000738024	0
BRDN0000737696	0	BRDN0000738016	0	Inpp5a	0	BRDN0000738023	0
BRDN0000737695	0	BRDN0000738015	0	Inpp5b	0	BRDN0000738022	0
BRDN0000737694	0	BRDN0000738014	0	Lbr	0	BRDN0000738021	0
BRDN0000737693	0	BRDN0000738013	0	Ubxn2b	0	BRDN0000738020	0
BRDN0000737692	0	BRDN0000738012	0	Inpp5e	0	BRDN0000738019	0
BRDN0000737691	0	BRDN0000738011	0	Ran	0	BRDN0000738018	0
BRDN0000737689	0	BRDN0000738010	0	Inpp5j	0	BRDN0000738017	0
BRDN0000737688	0	BRDN0000738009	0	Inpp5k	0	BRDN0000738016	0
BRDN0000737687	0	BRDN0000738008	0	Dzank1	0	BRDN0000738014	0
BRDN0000737686	0	BRDN0000738007	0	Cyp20a1	0	BRDN0000738013	0
BRDN0000737685	0	BRDN0000738006	0	Etohi1	0	BRDN0000738012	0
BRDN0000737684	0	BRDN0000738004	0	Mcpt4	0	BRDN0000738011	0
BRDN0000737683	0	BRDN0000738003	0	Il34	0	BRDN0000738010	0
BRDN0000737682	0	BRDN0000738002	0	Mcpt1	0	BRDN0000738009	0
BRDN0000737681	0	BRDN0000738001	0	Il33	0	BRDN0000738008	0
BRDN0000737680	0	BRDN0000738000	0	Mcpt2	0	BRDN0000738007	0
BRDN0000737679	0	BRDN0000737999	0	Eny2	0	BRDN0000738006	0
BRDN0000737678	0	BRDN0000737998	0	Lbh	0	BRDN0000738005	0
BRDN0000737676	0	BRDN0000737997	0	Carhsp1	0	BRDN0000738003	0
BRDN0000737675	0	BRDN0000737995	0	Mcpt9	0	BRDN0000738002	0
BRDN0000737674	0	BRDN0000737994	0	Marc1	0	BRDN0000738000	0
BRDN0000737673	0	BRDN0000737993	0	Has2	0	BRDN0000737999	0
BRDN0000737672	0	BRDN0000737992	0	Has3	0	BRDN0000737998	0
BRDN0000737671	0	BRDN0000737991	0	Ltbr	0	BRDN0000737997	0
BRDN0000737670	0	BRDN0000737990	0	Mut	0	BRDN0000737996	0
BRDN0000737669	0	BRDN0000737989	0	Tbl1x	0	BRDN0000737995	0
BRDN0000737668	0	BRDN0000737988	0	4930555G01Rik	0	BRDN0000737994	0
BRDN0000737667	0	BRDN0000737987	0	Obp2b	0	BRDN0000737993	0
BRDN0000737666	0	BRDN0000737986	0	Itgb6	0	BRDN0000737992	0
BRDN0000737664	0	BRDN0000737985	0	Vmn1r67	0	BRDN0000737991	0
BRDN0000737663	0	BRDN0000737984	0	Trat1	0	BRDN0000737990	0
BRDN0000737662	0	BRDN0000737983	0	Mal2	0	BRDN0000737989	0
BRDN0000737661	0	BRDN0000737982	0	Plekha4	0	BRDN0000737988	0
BRDN0000737660	0	BRDN0000737981	0	Plekha5	0	BRDN0000737987	0
BRDN0000737659	0	BRDN0000737980	0	Zfp511	0	BRDN0000737985	0
BRDN0000737658	0	BRDN0000737979	0	Pla2g1b	0	BRDN0000737984	0
BRDN0000737657	0	BRDN0000737978	0	Rheb	0	BRDN0000737983	0
BRDN0000737656	0	BRDN0000737977	0	Psme1	0	BRDN0000737982	0
BRDN0000737655	0	BRDN0000737976	0	Gm9268	0	BRDN0000737981	0
BRDN0000737654	0	BRDN0000737975	0	Snrnp40	0	BRDN0000737980	0
BRDN0000737653	0	BRDN0000737974	0	Hic2	0	BRDN0000737979	0
BRDN0000737652	0	BRDN0000737973	0	1700026D08Rik	0	BRDN0000737978	0
BRDN0000737651	0	BRDN0000737972	0	Snrnp48	0	BRDN0000737977	0
BRDN0000737650	0	BRDN0000737971	0	Irs1	0	BRDN0000737976	0
BRDN0000737649	0	BRDN0000737970	0	Irs2	0	BRDN0000737975	0
BRDN0000737648	0	BRDN0000737969	0	Irs3	0	BRDN0000737974	0
BRDN0000737647	0	BRDN0000737968	0	Tm4sf20	0	BRDN0000737973	0
BRDN0000737646	0	BRDN0000737967	0	Crip2	0	BRDN0000737972	0
BRDN0000737645	0	BRDN0000737966	0	Usp6nl	0	BRDN0000737971	0
BRDN0000737644	0	BRDN0000737965	0	Bdh1	0	BRDN0000737970	0
BRDN0000737642	0	BRDN0000737963	0	Syn2	0	BRDN0000737969	0
BRDN0000737641	0	BRDN0000737962	0	1700025G04Rik	0	BRDN0000737968	0
BRDN0000737640	0	BRDN0000737961	0	Ugt1a9	0	BRDN0000737967	0
BRDN0000737639	0	BRDN0000737960	0	Pla2g15	0	BRDN0000737966	0
BRDN0000737638	0	BRDN0000737958	0	Abca12	0	BRDN0000737965	0
BRDN0000737636	0	BRDN0000737957	0	Kazald1	0	BRDN0000737963	0

BRDN0000737635	0	BRDN0000737956	0	Abca14	0	BRDN0000737962	0
BRDN0000737634	0	BRDN0000737955	0	Abca15	0	BRDN0000737961	0
BRDN0000737633	0	BRDN0000737954	0	Abca16	0	BRDN0000737960	0
BRDN0000737632	0	BRDN0000737952	0	Abca17	0	BRDN0000737959	0
BRDN0000737631	0	BRDN0000737951	0	Tmsb4x	0	BRDN0000737958	0
BRDN0000737629	0	BRDN0000737950	0	Sdhaf2	0	BRDN0000737956	0
BRDN0000737628	0	BRDN0000737949	0	2310022B05Rik	0	BRDN0000737954	0
BRDN0000737627	0	BRDN0000737948	0	Tcp10a	0	BRDN0000737953	0
BRDN0000737625	0	BRDN0000737946	0	Mall	0	BRDN0000737951	0
BRDN0000737624	0	BRDN0000737945	0	Tcp10c	0	BRDN0000737950	0
BRDN0000737623	0	BRDN0000737944	0	Hexim1	0	BRDN0000737949	0
BRDN0000737622	0	BRDN0000737943	0	Ceacam14	0	BRDN0000737948	0
BRDN0000737621	0	BRDN0000737942	0	Hnrnpll	0	BRDN0000737946	0
BRDN0000737620	0	BRDN0000737941	0	Clic3	0	BRDN0000737945	0
BRDN0000737619	0	BRDN0000737939	0	Sbf1	0	BRDN0000737944	0
BRDN0000737618	0	BRDN0000737938	0	Samd9l	0	BRDN0000737943	0
BRDN0000737617	0	BRDN0000737937	0	Synm	0	BRDN0000737942	0
BRDN0000737615	0	BRDN0000737936	0	3110062M04Rik	0	BRDN0000737941	0
BRDN0000737614	0	BRDN0000737935	0	Tcstv3	0	BRDN0000737940	0
BRDN0000737613	0	BRDN0000737934	0	6430573F11Rik	0	BRDN0000737939	0
BRDN0000737612	0	BRDN0000737933	0	BRDN0000737403	0	BRDN0000737938	0
BRDN0000737611	0	BRDN0000737932	0	Nrgn	0	BRDN0000737937	0
BRDN0000737610	0	BRDN0000737931	0	Phf21a	0	BRDN0000737936	0
BRDN0000737609	0	BRDN0000737929	0	Sync	0	BRDN0000737935	0
BRDN0000737608	0	BRDN0000737928	0	Dclk3	0	BRDN0000737933	0
BRDN0000737607	0	BRDN0000737927	0	Phf21b	0	BRDN0000737931	0
BRDN0000737606	0	BRDN0000737926	0	Gpr88	0	BRDN0000737930	0
BRDN0000737605	0	BRDN0000737925	0	Gpr89	0	BRDN0000737929	0
BRDN0000737604	0	BRDN0000737924	0	Olfr1283	0	BRDN0000737928	0
BRDN0000737603	0	BRDN0000737923	0	Capsl	0	BRDN0000737926	0
BRDN0000737602	0	BRDN0000737922	0	Caps2	0	BRDN0000737925	0
BRDN0000737601	0	BRDN0000737921	0	Myoc	0	BRDN0000737924	0
BRDN0000737600	0	BRDN0000737920	0	Olfr1284	0	BRDN0000737923	0
BRDN0000737599	0	BRDN0000737918	0	Gpr83	0	BRDN0000737922	0
BRDN0000737598	0	BRDN0000737917	0	Gpr84	0	BRDN0000737920	0
BRDN0000737597	0	BRDN0000737915	0	Myof	0	BRDN0000737919	0
BRDN0000737596	0	BRDN0000737914	0	Pkm	0	BRDN0000737918	0
BRDN0000737595	0	BRDN0000737913	0	Hectd2	0	BRDN0000737917	0
BRDN0000737594	0	BRDN0000737912	0	Hectd3	0	BRDN0000737916	0
BRDN0000737593	0	BRDN0000737911	0	Hectd1	0	BRDN0000737915	0
BRDN0000737592	0	BRDN0000737910	0	Tgm5	0	BRDN0000737914	0
BRDN0000737591	0	BRDN0000737909	0	Mcc	0	BRDN0000737913	0
BRDN0000737590	0	BRDN0000737908	0	Gm13040	0	BRDN0000737912	0
BRDN0000737589	0	BRDN0000737907	0	Zc3hav1	0	BRDN0000737911	0
BRDN0000737588	0	BRDN0000737906	0	Gfra4	0	BRDN0000737910	0
BRDN0000737587	0	BRDN0000737905	0	Dpysl5	0	BRDN0000737908	0
BRDN0000737586	0	BRDN0000737904	0	Gfra1	0	BRDN0000737907	0
BRDN0000737585	0	BRDN0000737903	0	BC117090	0	BRDN0000737906	0
BRDN0000737584	0	BRDN0000737902	0	Gfra3	0	BRDN0000737905	0
BRDN0000737583	0	BRDN0000737900	0	Gfra2	0	BRDN0000737904	0
BRDN0000737582	0	BRDN0000737899	0	BRDN0000738198	0	BRDN0000737901	0
BRDN0000737581	0	BRDN0000737898	0	Asic2	0	BRDN0000737900	0
BRDN0000737580	0	BRDN0000737897	0	Krtap27-1	0	BRDN0000737899	0
BRDN0000737579	0	BRDN0000737896	0	BRDN0000738199	0	BRDN0000737898	0
BRDN0000737578	0	BRDN0000737895	0	Iars	0	BRDN0000737897	0
BRDN0000737577	0	BRDN0000737894	0	AF251705	0	BRDN0000737896	0
BRDN0000737576	0	BRDN0000737893	0	Olfr1065	0	BRDN0000737895	0
BRDN0000737575	0	BRDN0000737892	0	Prrg3	0	BRDN0000737894	0
BRDN0000737574	0	BRDN0000737891	0	Brd2	0	BRDN0000737893	0
BRDN0000737573	0	BRDN0000737890	0	Lactb	0	BRDN0000737892	0
BRDN0000737572	0	BRDN0000737889	0	Cd200	0	BRDN0000737891	0
BRDN0000737571	0	BRDN0000737888	0	Olfr883	0	BRDN0000737890	0
BRDN0000737570	0	BRDN0000737887	0	Arl6ip5	0	BRDN0000737889	0
BRDN0000737569	0	BRDN0000737886	0	Cd207	0	BRDN0000737888	0
BRDN0000737568	0	BRDN0000737885	0	Sertad1	0	BRDN0000737887	0
BRDN0000737567	0	BRDN0000737882	0	Sertad2	0	BRDN0000737886	0
BRDN0000737566	0	BRDN0000737881	0	Sertad3	0	BRDN0000737885	0
BRDN0000737565	0	BRDN0000737880	0	Antxrl	0	BRDN0000737884	0
BRDN0000737564	0	BRDN0000737879	0	Mad1l1	0	BRDN0000737883	0
BRDN0000737563	0	BRDN0000737878	0	Gfral	0	BRDN0000737882	0
BRDN0000737562	0	BRDN0000737877	0	4931408C20Rik	0	BRDN0000737880	0
BRDN0000737561	0	BRDN0000737876	0	Vmn2r112	0	BRDN0000737879	0
BRDN0000737560	0	BRDN0000737875	0	Prelid2	0	BRDN0000737878	0
BRDN0000737559	0	BRDN0000737873	0	Fmnl1	0	BRDN0000737877	0
BRDN0000737558	0	BRDN0000737872	0	Chrna4	0	BRDN0000737875	0
BRDN0000737557	0	BRDN0000737871	0	Zfp94	0	BRDN0000737874	0
BRDN0000737556	0	BRDN0000737870	0	Pik3r4	0	BRDN0000737873	0
BRDN0000737555	0	BRDN0000737869	0	Pik3r3	0	BRDN0000737872	0
BRDN0000737554	0	BRDN0000737867	0	BRDN0000738192	0	BRDN0000737871	0
BRDN0000737553	0	BRDN0000737866	0	Chrna3	0	BRDN0000737870	0
BRDN0000737552	0	BRDN0000737865	0	Krt81	0	BRDN0000737869	0
BRDN0000737551	0	BRDN0000737863	0	BRDN0000738193	0	BRDN0000737868	0

BRDN0000737550	0	BRDN0000737862	0	Zfp72	0	BRDN0000737867	0
BRDN0000737549	0	BRDN0000737861	0	Matn1	0	BRDN0000737866	0
BRDN0000737548	0	BRDN0000737860	0	BRDN0000737681	0	BRDN0000737865	0
BRDN0000737547	0	BRDN0000737859	0	Matn3	0	BRDN0000737863	0
BRDN0000737546	0	BRDN0000737858	0	Gnpnat1	0	BRDN0000737862	0
BRDN0000737545	0	BRDN0000737857	0	Matn4	0	BRDN0000737861	0
BRDN0000737544	0	BRDN0000737856	0	Olfr1101	0	BRDN0000737860	0
BRDN0000737543	0	BRDN0000737855	0	Pes1	0	BRDN0000737859	0
BRDN0000737542	0	BRDN0000737853	0	Styxl1	0	BRDN0000737857	0
BRDN0000737541	0	BRDN0000737851	0	Pet2	0	BRDN0000737856	0
BRDN0000737540	0	BRDN0000737850	0	Rab6a	0	BRDN0000737855	0
BRDN0000737539	0	BRDN0000737849	0	Rab6b	0	BRDN0000737854	0
BRDN0000737538	0	BRDN0000737848	0	Btbd10	0	BRDN0000737853	0
BRDN0000737536	0	BRDN0000737847	0	Enkur	0	BRDN0000737852	0
BRDN0000737535	0	BRDN0000737846	0	BRDN0000737682	0	BRDN0000737851	0
BRDN0000737534	0	BRDN0000737845	0	9830107B12Rik	0	BRDN0000737850	0
BRDN0000737533	0	BRDN0000737844	0	Btg1	0	BRDN0000737848	0
BRDN0000737532	0	BRDN0000737843	0	Ndufc1	0	BRDN0000737847	0
BRDN0000737531	0	BRDN0000737842	0	Ndufc2	0	BRDN0000737845	0
BRDN0000737530	0	BRDN0000737841	0	BRDN0000737687	0	BRDN0000737844	0
BRDN0000737529	0	BRDN0000737840	0	Arhgap17	0	BRDN0000737843	0
BRDN0000737528	0	BRDN0000737839	0	Arhgap15	0	BRDN0000737842	0
BRDN0000737527	0	BRDN0000737838	0	Arhgap12	0	BRDN0000737840	0
BRDN0000737526	0	BRDN0000737837	0	Arhgap10	0	BRDN0000737839	0
BRDN0000737525	0	BRDN0000737836	0	BRDN0000738345	0	BRDN0000737838	0
BRDN0000737524	0	BRDN0000737832	0	Arhgap19	0	BRDN0000737837	0
BRDN0000737523	0	BRDN0000737829	0	Krtap12-1	0	BRDN0000737836	0
BRDN0000737522	0	BRDN0000737828	0	Gm4745	0	BRDN0000737835	0
BRDN0000737521	0	BRDN0000737827	0	Ddx3y	0	BRDN0000737834	0
BRDN0000737520	0	BRDN0000737826	0	Sik3	0	BRDN0000737833	0
BRDN0000737519	0	BRDN0000737825	0	Sik2	0	BRDN0000737832	0
BRDN0000737518	0	BRDN0000737824	0	Nr1h2	0	BRDN0000737831	0
BRDN0000737517	0	BRDN0000737823	0	Emx2	0	BRDN0000737830	0
BRDN0000737516	0	BRDN0000737822	0	Rpl21	0	BRDN0000737829	0
BRDN0000737515	0	BRDN0000737821	0	BRDN0000737748	0	BRDN0000737828	0
BRDN0000737514	0	BRDN0000737820	0	Emx1	0	BRDN0000737827	0
BRDN0000737513	0	BRDN0000737819	0	Adam10	0	BRDN0000737825	0
BRDN0000737512	0	BRDN0000737818	0	Adam11	0	BRDN0000737823	0
BRDN0000737511	0	BRDN0000737817	0	BRDN0000737799	0	BRDN0000737822	0
BRDN0000737510	0	BRDN0000737816	0	Adam15	0	BRDN0000737821	0
BRDN0000737509	0	BRDN0000737815	0	Adam17	0	BRDN0000737820	0
BRDN0000737508	0	BRDN0000737814	0	Adam19	0	BRDN0000737819	0
BRDN0000737507	0	BRDN0000737813	0	Dedd	0	BRDN0000737817	0
BRDN0000737506	0	BRDN0000737812	0	Catsperb	0	BRDN0000737816	0
BRDN0000737505	0	BRDN0000737811	0	Vmn2r116	0	BRDN0000737815	0
BRDN0000737504	0	BRDN0000737810	0	A630095E13Rik	0	BRDN0000737814	0
BRDN0000737503	0	BRDN0000737809	0	Rnd3	0	BRDN0000737813	0
BRDN0000737502	0	BRDN0000737808	0	Sertm1	0	BRDN0000737812	0
BRDN0000737501	0	BRDN0000737806	0	Mthfd1l	0	BRDN0000737811	0
BRDN0000737500	0	BRDN0000737805	0	Olfr424	0	BRDN0000737810	0
BRDN0000737499	0	BRDN0000737804	0	Ldhb	0	BRDN0000737809	0
BRDN0000737498	0	BRDN0000737802	0	Exoc3l4	0	BRDN0000737808	0
BRDN0000737497	0	BRDN0000737801	0	Brms1l	0	BRDN0000737807	0
BRDN0000737496	0	BRDN0000737800	0	Hebp1	0	BRDN0000737805	0
BRDN0000737495	0	BRDN0000737798	0	Hebp2	0	BRDN0000737804	0
BRDN0000737494	0	BRDN0000737797	0	Olfr1373	0	BRDN0000737803	0
BRDN0000737493	0	BRDN0000737796	0	Zfc3h1	0	BRDN0000737802	0
BRDN0000737492	0	BRDN0000737795	0	Gm17359	0	BRDN0000737801	0
BRDN0000737491	0	BRDN0000737794	0	Adam1a	0	BRDN0000737800	0
BRDN0000737490	0	BRDN0000737793	0	Ddx39	0	BRDN0000737799	0
BRDN0000737489	0	BRDN0000737792	0	Catsper4	0	BRDN0000737798	0
BRDN0000737487	0	BRDN0000737791	0	Aff4	0	BRDN0000737797	0
BRDN0000737485	0	BRDN0000737790	0	3110082J24Rik	0	BRDN0000737796	0
BRDN0000737484	0	BRDN0000737789	0	Catsper1	0	BRDN0000737795	0
BRDN0000737483	0	BRDN0000737788	0	Catsper2	0	BRDN0000737794	0
BRDN0000737482	0	BRDN0000737787	0	Suv420h1	0	BRDN0000737793	0
BRDN0000737481	0	BRDN0000737786	0	Pnpla3	0	BRDN0000737792	0
BRDN0000737479	0	BRDN0000737784	0	Pnpla2	0	BRDN0000737791	0
BRDN0000737477	0	BRDN0000737783	0	Pnpla7	0	BRDN0000737790	0
BRDN0000737476	0	BRDN0000737782	0	Pnpla6	0	BRDN0000737786	0
BRDN0000737475	0	BRDN0000737781	0	Pnpla5	0	BRDN0000737785	0
BRDN0000737474	0	BRDN0000737780	0	Lcn12	0	BRDN0000737784	0
BRDN0000737473	0	BRDN0000737779	0	Gpr162	0	BRDN0000737783	0
BRDN0000737472	0	BRDN0000737778	0	Gpr160	0	BRDN0000737782	0
BRDN0000737471	0	BRDN0000737777	0	Qars	0	BRDN0000737781	0
BRDN0000737470	0	BRDN0000737776	0	Nbeal1	0	BRDN0000737780	0
BRDN0000737469	0	BRDN0000737775	0	Gpr165	0	BRDN0000737779	0
BRDN0000737468	0	BRDN0000737774	0	Slc39a12	0	BRDN0000737778	0
BRDN0000737467	0	BRDN0000737773	0	Slc39a10	0	BRDN0000737777	0
BRDN0000737466	0	BRDN0000737772	0	Slc39a11	0	BRDN0000737776	0
BRDN0000737465	0	BRDN0000737771	0	Dzip3	0	BRDN0000737774	0
BRDN0000737463	0	BRDN0000737770	0	Tbpl2	0	BRDN0000737773	0

BRDN0000737461	0	BRDN0000737769	0	Prmt6	0	BRDN0000737772	0
BRDN0000737460	0	BRDN0000737768	0	Nsun3	0	BRDN0000737771	0
BRDN0000737459	0	BRDN0000737766	0	Nsun2	0	BRDN0000737770	0
BRDN0000737458	0	BRDN0000737765	0	Nsun5	0	BRDN0000737769	0
BRDN0000737457	0	BRDN0000737764	0	Nsun4	0	BRDN0000737767	0
BRDN0000737456	0	BRDN0000737762	0	Nsun7	0	BRDN0000737766	0
BRDN0000737455	0	BRDN0000737761	0	Nsun6	0	BRDN0000737765	0
BRDN0000737454	0	BRDN0000737760	0	Apc2	0	BRDN0000737763	0
BRDN0000737453	0	BRDN0000737759	0	Nfasc	0	BRDN0000737762	0
BRDN0000737452	0	BRDN0000737757	0	Olfr960	0	BRDN0000737759	0
BRDN0000737451	0	BRDN0000737756	0	Prmt5	0	BRDN0000737758	0
BRDN0000737450	0	BRDN0000737754	0	Adrm1	0	BRDN0000737757	0
BRDN0000737449	0	BRDN0000737753	0	Suox	0	BRDN0000737756	0
BRDN0000737448	0	BRDN0000737752	0	Nyap2	0	BRDN0000737754	0
BRDN0000737447	0	BRDN0000737751	0	Pan3	0	BRDN0000737753	0
BRDN0000737446	0	BRDN0000737750	0	Hoxd8	0	BRDN0000737752	0
BRDN0000737445	0	BRDN0000737749	0	Hoxd9	0	BRDN0000737751	0
BRDN0000737444	0	BRDN0000737748	0	BRDN0000737752	0	BRDN0000737750	0
BRDN0000737443	0	BRDN0000737747	0	Hoxd4	0	BRDN0000737749	0
BRDN0000737442	0	BRDN0000737746	0	Eef1a1	0	BRDN0000737748	0
BRDN0000737441	0	BRDN0000737745	0	Tbata	0	BRDN0000737747	0
BRDN0000737440	0	BRDN0000737744	0	Tln2	0	BRDN0000737746	0
BRDN0000737439	0	BRDN0000737743	0	Hoxd1	0	BRDN0000737745	0
BRDN0000737438	0	BRDN0000737742	0	Ptger2	0	BRDN0000737744	0
BRDN0000737436	0	BRDN0000737741	0	Tln1	0	BRDN0000737743	0
BRDN0000737435	0	BRDN0000737740	0	Il1f8	0	BRDN0000737742	0
BRDN0000737434	0	BRDN0000737739	0	Trim59	0	BRDN0000737741	0
BRDN0000737433	0	BRDN0000737738	0	Trim58	0	BRDN0000737740	0
BRDN0000737432	0	BRDN0000737737	0	Golga7b	0	BRDN0000737739	0
BRDN0000737431	0	BRDN0000737736	0	Glyatl3	0	BRDN0000737737	0
BRDN0000737430	0	BRDN0000737735	0	Mybl1	0	BRDN0000737736	0
BRDN0000737428	0	BRDN0000737733	0	Trim54	0	BRDN0000737734	0
BRDN0000737427	0	BRDN0000737732	0	Trim56	0	BRDN0000737733	0
BRDN0000737426	0	BRDN0000737731	0	Il1f5	0	BRDN0000737732	0
BRDN0000737425	0	BRDN0000737730	0	Trim52	0	BRDN0000737731	0
BRDN0000737424	0	BRDN0000737729	0	Pank3	0	BRDN0000737730	0
BRDN0000737423	0	BRDN0000737728	0	Pank2	0	BRDN0000737729	0
BRDN0000737422	0	BRDN0000737727	0	BRDN0000737755	0	BRDN0000737728	0
BRDN0000737421	0	BRDN0000737726	0	Cdc42ep3	0	BRDN0000737726	0
BRDN0000737420	0	BRDN0000737725	0	Gab2	0	BRDN0000737724	0
BRDN0000737419	0	BRDN0000737724	0	Gareml	0	BRDN0000737723	0
BRDN0000737418	0	BRDN0000737723	0	Mrgprb4	0	BRDN0000737722	0
BRDN0000737417	0	BRDN0000737722	0	Rbm39	0	BRDN0000737721	0
BRDN0000737416	0	BRDN0000737721	0	BRDN0000737458	0	BRDN0000737720	0
BRDN0000737415	0	BRDN0000737720	0	Vmn1r107	0	BRDN0000737719	0
BRDN0000737414	0	BRDN0000737719	0	Dap3	0	BRDN0000737718	0
BRDN0000737413	0	BRDN0000737718	0	Trit1	0	BRDN0000737717	0
BRDN0000737412	0	BRDN0000737717	0	Tes	0	BRDN0000737716	0
BRDN0000737411	0	BRDN0000737716	0	BRDN0000737450	0	BRDN0000737715	0
BRDN0000737410	0	BRDN0000737715	0	Gab1	0	BRDN0000737714	0
BRDN0000737409	0	BRDN0000737714	0	BRDN0000737454	0	BRDN0000737713	0
BRDN0000737408	0	BRDN0000737713	0	BRDN0000737455	0	BRDN0000737712	0
BRDN0000737407	0	BRDN0000737712	0	Pdha1	0	BRDN0000737711	0
BRDN0000737406	0	BRDN0000737711	0	Arhgef37	0	BRDN0000737710	0
BRDN0000737405	0	BRDN0000737710	0	Gnl3	0	BRDN0000737709	0
BRDN0000737404	0	BRDN0000737709	0	Gm10375	0	BRDN0000737708	0
BRDN0000737403	0	BRDN0000737708	0	Fbxw10	0	BRDN0000737707	0
BRDN0000737402	0	BRDN0000737707	0	Gm10377	0	BRDN0000737706	0
BRDN0000737401	0	BRDN0000737706	0	Fbxw16	0	BRDN0000737705	0
BRDN0000737400	0	BRDN0000737705	0	Casz1	0	BRDN0000737704	0
BRDN0000737399	0	BRDN0000737704	0	BC024139	0	BRDN0000737703	0
BRDN0000737398	0	BRDN0000737703	0	Fbxw15	0	BRDN0000737702	0
BRDN0000737396	0	BRDN0000737702	0	Glis2	0	BRDN0000737701	0
BRDN0000737395	0	BRDN0000737701	0	Vmn1r103	0	BRDN0000737700	0
BRDN0000737394	0	BRDN0000737700	0	Fbxw18	0	BRDN0000737699	0
BRDN0000737393	0	BRDN0000737699	0	Fbxw19	0	BRDN0000737698	0
BRDN0000737392	0	BRDN0000737697	0	Tmem39b	0	BRDN0000737697	0
BRDN0000737391	0	BRDN0000737696	0	Pcp2	0	BRDN0000737696	0
BRDN0000737390	0	BRDN0000737695	0	Tmem39a	0	BRDN0000737695	0
BRDN0000737389	0	BRDN0000737694	0	Evi5l	0	BRDN0000737694	0
BRDN0000737388	0	BRDN0000737693	0	Prokr1	0	BRDN0000737693	0
BRDN0000737387	0	BRDN0000737692	0	Prokr2	0	BRDN0000737692	0
BRDN0000737386	0	BRDN0000737691	0	St3gal5	0	BRDN0000737691	0
BRDN0000737385	0	BRDN0000737690	0	Zfp689	0	BRDN0000737690	0
BRDN0000737384	0	BRDN0000737689	0	Gvin1	0	BRDN0000737689	0
BRDN0000737383	0	BRDN0000737688	0	Fnip1	0	BRDN0000737688	0
BRDN0000737382	0	BRDN0000737687	0	Fnip2	0	BRDN0000737687	0
BRDN0000737381	0	BRDN0000737686	0	1700012A03Rik	0	BRDN0000737686	0
BRDN0000737380	0	BRDN0000737685	0	Psph	0	BRDN0000737685	0
Brd9	0	BRDN0000737684	0	Zar1	0	BRDN0000737684	0
Brd8	0	BRDN0000737683	0	Zfyve16	0	BRDN0000737683	0
Brd7	0	BRDN0000737682	0	Lin37	0	BRDN0000737682	0

Brd4	0	BRDN0000737680	0	Mmadhc	0	BRDN0000737681	0
Brd3	0	BRDN0000737679	0	BRDN0000738250	0	BRDN0000737680	0
Brd2	0	BRDN0000737678	0	Sprr3	0	BRDN0000737679	0
Brcc3	0	BRDN0000737677	0	Ubtd2	0	BRDN0000737678	0
Brca2	0	BRDN0000737676	0	BRDN0000737538	0	BRDN0000737677	0
Brca1	0	BRDN0000737675	0	BRDN0000737852	0	BRDN0000737676	0
Brap	0	BRDN0000737673	0	BRDN0000737885	0	BRDN0000737675	0
Braf	0	BRDN0000737672	0	BRDN0000737697	0	BRDN0000737674	0
Bptf	0	BRDN0000737671	0	Agk	0	BRDN0000737673	0
Bpnt1	0	BRDN0000737670	0	Actr3b	0	BRDN0000737672	0
Bpifb9b	0	BRDN0000737669	0	Zfp354c	0	BRDN0000737671	0
Bpifb6	0	BRDN0000737668	0	Zfp354b	0	BRDN0000737670	0
Bpifb5	0	BRDN0000737667	0	Zfp354a	0	BRDN0000737669	0
Bpifb4	0	BRDN0000737666	0	Gbp10	0	BRDN0000737668	0
Bpifb2	0	BRDN0000737665	0	Zmat1	0	BRDN0000737667	0
Bpifb1	0	BRDN0000737664	0	Mum1	0	BRDN0000737666	0
Bpifa6	0	BRDN0000737663	0	Ric8	0	BRDN0000737665	0
Bpifa5	0	BRDN0000737662	0	Bub1b	0	BRDN0000737664	0
Bpifa3	0	BRDN0000737661	0	Sntg1	0	BRDN0000737663	0
Bpifa2	0	BRDN0000737660	0	Sntg2	0	BRDN0000737662	0
Bpi	0	BRDN0000737659	0	Cnksr3	0	BRDN0000737661	0
Bpgm	0	BRDN0000737658	0	Cnksr2	0	BRDN0000737660	0
Bora	0	BRDN0000737657	0	Zmat2	0	BRDN0000737659	0
Bop1	0	BRDN0000737656	0	Syndig1l	0	BRDN0000737658	0
Boll	0	BRDN0000737655	0	BRDN0000737532	0	BRDN0000737657	0
Bola3	0	BRDN0000737654	0	Larp6	0	BRDN0000737656	0
Bola2	0	BRDN0000737653	0	Zcchc2	0	BRDN0000737655	0
Bola1	0	BRDN0000737650	0	BRDN0000737894	0	BRDN0000737654	0
Bok	0	BRDN0000737649	0	Eif1b	0	BRDN0000737653	0
Bod1l	0	BRDN0000737648	0	Itgb1	0	BRDN0000737652	0
Bod1	0	BRDN0000737647	0	Eif1a	0	BRDN0000737651	0
Bnipl	0	BRDN0000737646	0	Mpg	0	BRDN0000737650	0
Bnip3l	0	BRDN0000737645	0	Dnajb1	0	BRDN0000737647	0
Bnip3	0	BRDN0000737644	0	BRDN0000738374	0	BRDN0000737646	0
Bnip2	0	BRDN0000737643	0	Itgb3	0	BRDN0000737645	0
Bnip1	0	BRDN0000737642	0	Ric3	0	BRDN0000737644	0
Bnc2	0	BRDN0000737641	0	Rbm47	0	BRDN0000737643	0
Bnc1	0	BRDN0000737640	0	Nom1	0	BRDN0000737642	0
Bmx	0	BRDN0000737639	0	Olfr600	0	BRDN0000737641	0
Bms1	0	BRDN0000737638	0	Elmsan1	0	BRDN0000737640	0
Bmpr1b	0	BRDN0000737637	0	Clec11a	0	BRDN0000737639	0
Bmp8b	0	BRDN0000737636	0	Tpra1	0	BRDN0000737638	0
Bmp8a	0	BRDN0000737635	0	Galntl5	0	BRDN0000737637	0
Bmp7	0	BRDN0000737634	0	Olfr1245	0	BRDN0000737636	0
Bmp6	0	BRDN0000737633	0	Olfr1246	0	BRDN0000737635	0
Bmp4	0	BRDN0000737632	0	Olfr1247	0	BRDN0000737634	0
Bmp3	0	BRDN0000737631	0	Olfr1241	0	BRDN0000737633	0
Bmp2	0	BRDN0000737630	0	Olfr1242	0	BRDN0000737632	0
Bmp15	0	BRDN0000737629	0	Olfr1243	0	BRDN0000737631	0
Bmp10	0	BRDN0000737628	0	Tada2a	0	BRDN0000737630	0
Bmp1	0	BRDN0000737627	0	Gorasp1	0	BRDN0000737629	0
Bmi1	0	BRDN0000737626	0	Exo1	0	BRDN0000737628	0
Blzf1	0	BRDN0000737625	0	Olfr1248	0	BRDN0000737627	0
Blvrb	0	BRDN0000737624	0	Olfr1249	0	BRDN0000737626	0
Blvra	0	BRDN0000737623	0	Mlana	0	BRDN0000737625	0
Bloc1s6	0	BRDN0000737622	0	Exo5	0	BRDN0000737623	0
Bloc1s5	0	BRDN0000737620	0	BRDN0000738312	0	BRDN0000737622	0
Bloc1s4	0	BRDN0000737619	0	Pigh	0	BRDN0000737620	0
Bloc1s3	0	BRDN0000737618	0	Map7d1	0	BRDN0000737619	0
Bloc1s2	0	BRDN0000737617	0	Mpo	0	BRDN0000737618	0
Bloc1s1	0	BRDN0000737616	0	Nos1ap	0	BRDN0000737617	0
Blnk	0	BRDN0000737615	0	Olig3	0	BRDN0000737616	0
Blmh	0	BRDN0000737614	0	Bsdc1	0	BRDN0000737615	0
Blm	0	BRDN0000737613	0	Pigk	0	BRDN0000737614	0
Blk	0	BRDN0000737611	0	Lrrn4cl	0	BRDN0000737613	0
Bivm	0	BRDN0000737610	0	Krtap17-1	0	BRDN0000737612	0
Birc7	0	BRDN0000737608	0	Gm11595	0	BRDN0000737611	0
Birc6	0	BRDN0000737607	0	Qtrtd1	0	BRDN0000737610	0
Birc5	0	BRDN0000737606	0	Arid5a	0	BRDN0000737608	0
Birc3	0	BRDN0000737605	0	Mms22l	0	BRDN0000737607	0
Birc2	0	BRDN0000737604	0	2310033P09Rik	0	BRDN0000737606	0
Bin3	0	BRDN0000737603	0	Ankrd46	0	BRDN0000737605	0
Bin2	0	BRDN0000737602	0	Zcchc7	0	BRDN0000737604	0
Bin1	0	BRDN0000737601	0	Ugt2b38	0	BRDN0000737603	0
Bik	0	BRDN0000737600	0	Cobl	0	BRDN0000737602	0
Bicd2	0	BRDN0000737599	0	Olfr926	0	BRDN0000737601	0
Bicc1	0	BRDN0000737598	0	Ugt2b34	0	BRDN0000737600	0
Bhmt2	0	BRDN0000737597	0	Ugt2b35	0	BRDN0000737599	0
Bhmt	0	BRDN0000737596	0	Olfr923	0	BRDN0000737598	0
Bhlhe41	0	BRDN0000737595	0	Olfr922	0	BRDN0000737597	0
Bhlhe40	0	BRDN0000737593	0	Olfr921	0	BRDN0000737595	0
Bhlhe23	0	BRDN0000737592	0	Olfr920	0	BRDN0000737594	0

Bhlha9	0	BRDN0000737591	0	Rgs21	0	BRDN0000737592	0
Bhlha15	0	BRDN0000737590	0	Rgs20	0	BRDN0000737591	0
Bgn	0	BRDN0000737589	0	Hspa5	0	BRDN0000737590	0
Bglap2	0	BRDN0000737588	0	Hspa4	0	BRDN0000737589	0
Bglap	0	BRDN0000737587	0	Rcan1	0	BRDN0000737588	0
Bfsp2	0	BRDN0000737586	0	Rcan3	0	BRDN0000737587	0
Bfsp1	0	BRDN0000737585	0	BRDN0000737884	0	BRDN0000737586	0
Bfar	0	BRDN0000737584	0	Peg12	0	BRDN0000737585	0
Bex6	0	BRDN0000737582	0	Plat	0	BRDN0000737583	0
Bex2	0	BRDN0000737581	0	Dnajb13	0	BRDN0000737582	0
Bex1	0	BRDN0000737580	0	Dnajb12	0	BRDN0000737581	0
Bet1l	0	BRDN0000737579	0	Dnajb11	0	BRDN0000737580	0
Bet1	0	BRDN0000737578	0	Tmsb10	0	BRDN0000737579	0
Best3	0	BRDN0000737577	0	Slc29a4	0	BRDN0000737578	0
Best2	0	BRDN0000737576	0	Fpr1	0	BRDN0000737577	0
Best1	0	BRDN0000737575	0	Parvb	0	BRDN0000737576	0
Bend7	0	BRDN0000737574	0	Fam83b	0	BRDN0000737575	0
Bend6	0	BRDN0000737573	0	Plaa	0	BRDN0000737574	0
Bend5	0	BRDN0000737572	0	Mov10l1	0	BRDN0000737573	0
Bend4	0	BRDN0000737571	0	Fam83g	0	BRDN0000737572	0
Bend3	0	BRDN0000737570	0	Parvg	0	BRDN0000737571	0
Begain	0	BRDN0000737569	0	Ctdsp2	0	BRDN0000737569	0
Becn1	0	BRDN0000737568	0	Olfr15	0	BRDN0000737568	0
Bean1	0	BRDN0000737567	0	Olfr16	0	BRDN0000737567	0
Bdp1	0	BRDN0000737565	0	Pgbd5	0	BRDN0000737566	0
Bdnf	0	BRDN0000737564	0	Olfr10	0	BRDN0000737564	0
Bdkrb2	0	BRDN0000737563	0	Vash1	0	BRDN0000737563	0
Bdkrb1	0	BRDN0000737562	0	Pgbd1	0	BRDN0000737562	0
Bdh2	0	BRDN0000737561	0	Caprin1	0	BRDN0000737561	0
Bdh1	0	BRDN0000737560	0	Olfr18	0	BRDN0000737560	0
Bcr	0	BRDN0000737559	0	Olfr19	0	BRDN0000737559	0
Bcorl1	0	BRDN0000737558	0	Pnma1	0	BRDN0000737558	0
Bcor	0	BRDN0000737557	0	Ptpn3	0	BRDN0000737557	0
Bco2	0	BRDN0000737556	0	Gm15292	0	BRDN0000737556	0
Bco1	0	BRDN0000737555	0	Mia2	0	BRDN0000737555	0
Bcl9l	0	BRDN0000737554	0	Flt4	0	BRDN0000737554	0
Bcl9	0	BRDN0000737553	0	Flt3	0	BRDN0000737553	0
Bcl7c	0	BRDN0000737551	0	Kcnj13	0	BRDN0000737552	0
Bcl7a	0	BRDN0000737550	0	Kcnj12	0	BRDN0000737551	0
Bcl6b	0	BRDN0000737549	0	Kcnj11	0	BRDN0000737550	0
Bcl6	0	BRDN0000737548	0	Kcnj10	0	BRDN0000737549	0
Bcl3	0	BRDN0000737547	0	Kcnj16	0	BRDN0000737548	0
Bcl2l15	0	BRDN0000737546	0	Kcnj15	0	BRDN0000737546	0
Bcl2l13	0	BRDN0000737545	0	Kcnj14	0	BRDN0000737545	0
Bcl2l12	0	BRDN0000737544	0	Rcn3	0	BRDN0000737544	0
Bcl2l11	0	BRDN0000737543	0	Pnma5	0	BRDN0000737543	0
Bcl2l10	0	BRDN0000737542	0	Krt1	0	BRDN0000737542	0
Bcl2l1	0	BRDN0000737541	0	B230118H07Rik	0	BRDN0000737541	0
Bcl2a1d	0	BRDN0000737540	0	Qsox2	0	BRDN0000737540	0
Bcl2a1c	0	BRDN0000737539	0	Prss30	0	BRDN0000737539	0
Bcl2a1b	0	BRDN0000737538	0	Tpd52l2	0	BRDN0000737537	0
Bcl2a1a	0	BRDN0000737537	0	Itga2b	0	BRDN0000737536	0
Bcl2	0	BRDN0000737536	0	Htra4	0	BRDN0000737535	0
Bcl11b	0	BRDN0000737535	0	Prss37	0	BRDN0000737533	0
Bcl11a	0	BRDN0000737534	0	Olfr56	0	BRDN0000737532	0
Bckdk	0	BRDN0000737532	0	Sash3	0	BRDN0000737529	0
Bckdha	0	BRDN0000737531	0	Htra2	0	BRDN0000737528	0
Bche	0	BRDN0000737530	0	Sash1	0	BRDN0000737527	0
Bcdin3d	0	BRDN0000737529	0	Qsox1	0	BRDN0000737526	0
Bccip	0	BRDN0000737527	0	Tsnax	0	BRDN0000737525	0
Bcat1	0	BRDN0000737526	0	Ugt2a3	0	BRDN0000737524	0
Bcas3	0	BRDN0000737525	0	Prss34	0	BRDN0000737523	0
Bcas2	0	BRDN0000737524	0	Ugt2a2	0	BRDN0000737520	0
Bcas1	0	BRDN0000737523	0	Il6	0	BRDN0000737519	0
Bcar3	0	BRDN0000737521	0	Il7	0	BRDN0000737518	0
Bcar1	0	BRDN0000737520	0	Slamf7	0	BRDN0000737517	0
Bcap31	0	BRDN0000737519	0	Il5	0	BRDN0000737516	0
Bcap29	0	BRDN0000737518	0	Il2	0	BRDN0000737515	0
Bcan	0	BRDN0000737516	0	Cpa4	0	BRDN0000737514	0
Bcam	0	BRDN0000737515	0	Cyb561d1	0	BRDN0000737513	0
BC147527	0	BRDN0000737514	0	Mpzl2	0	BRDN0000737510	0
BC117090	0	BRDN0000737513	0	Krt9	0	BRDN0000737509	0
BC107364	0	BRDN0000737511	0	Il9	0	BRDN0000737508	0
BC100530	0	BRDN0000737510	0	Klhl10	0	BRDN0000737507	0
BC100451	0	BRDN0000737509	0	2810403A07Rik	0	BRDN0000737506	0
BC094916	0	BRDN0000737508	0	Krt8	0	BRDN0000737505	0
BC089491	0	BRDN0000737507	0	Klhl15	0	BRDN0000737504	0
BC080695	0	BRDN0000737505	0	Rnaseh2c	0	BRDN0000737503	0
BC068281	0	BRDN0000737504	0	Klhl17	0	BRDN0000737501	0
BC061237	0	BRDN0000737503	0	Klhl18	0	BRDN0000737500	0
BC061212	0	BRDN0000737502	0	Cyp3a25	0	BRDN0000737499	0
BC055324	0	BRDN0000737501	0	BRDN0000737875	0	BRDN0000737497	0

BC055111	0	BRDN0000737500	0	Bclaf1	0	BRDN0000737496	0
BC052040	0	BRDN0000737499	0	Aen	0	BRDN0000737495	0
BC051665	0	BRDN0000737498	0	Ddx4	0	BRDN0000737494	0
BC051628	0	BRDN0000737497	0	Ddx5	0	BRDN0000737493	0
BC051142	0	BRDN0000737495	0	Ddx6	0	BRDN0000737492	0
BC051019	0	BRDN0000737494	0	Csnk1e	0	BRDN0000737491	0
BC049762	0	BRDN0000737493	0	Ddx1	0	BRDN0000737490	0
BC049730	0	BRDN0000737492	0	2410012M07Rik	0	BRDN0000737489	0
BC049715	0	BRDN0000737491	0	Speer3	0	BRDN0000737488	0
BC049635	0	BRDN0000737490	0	Speer2	0	BRDN0000737486	0
BC049352	0	BRDN0000737488	0	Zscan2	0	BRDN0000737485	0
BC048679	0	BRDN0000737486	0	Scaf8	0	BRDN0000737484	0
BC048609	0	BRDN0000737485	0	1300017J02Rik	0	BRDN0000737483	0
BC048562	0	BRDN0000737484	0	Olfr223	0	BRDN0000737482	0
BC048546	0	BRDN0000737483	0	Nutf2	0	BRDN0000737481	0
BC048507	0	BRDN0000737482	0	Rbl1	0	BRDN0000737480	0
BC048502	0	BRDN0000737481	0	Rbl2	0	BRDN0000737479	0
BC037034	0	BRDN0000737480	0	Slc29a3	0	BRDN0000737478	0
BC035044	0	BRDN0000737479	0	Fmn1	0	BRDN0000737477	0
BC031181	0	BRDN0000737476	0	Ilk	0	BRDN0000737476	0
BC030867	0	BRDN0000737475	0	Fmn2	0	BRDN0000737475	0
BC030500	0	BRDN0000737474	0	Adgb	0	BRDN0000737474	0
BC030336	0	BRDN0000737473	0	Fbxo36	0	BRDN0000737473	0
BC030307	0	BRDN0000737472	0	Ccdc53	0	BRDN0000737472	0
BC028528	0	BRDN0000737471	0	Fbxo30	0	BRDN0000737471	0
BC027231	0	BRDN0000737470	0	Fbxo31	0	BRDN0000737470	0
BC027072	0	BRDN0000737469	0	Fbxo32	0	BRDN0000737469	0
BC026585	0	BRDN0000737468	0	Fbxo33	0	BRDN0000737468	0
BC024978	0	BRDN0000737467	0	Fam136a	0	BRDN0000737467	0
BC022687	0	BRDN0000737466	0	Ccdc54	0	BRDN0000737464	0
BC021891	0	BRDN0000737465	0	BRDN0000738051	0	BRDN0000737463	0
BC017643	0	BRDN0000737464	0	Serpinb3d	0	BRDN0000737462	0
BC016579	0	BRDN0000737463	0	Raf1	0	BRDN0000737461	0
BC005624	0	BRDN0000737462	0	Fbxo39	0	BRDN0000737460	0
BC005561	0	BRDN0000737461	0	Serpinb3a	0	BRDN0000737459	0
BC005537	0	BRDN0000737460	0	Ces2e	0	BRDN0000737458	0
BC003331	0	BRDN0000737459	0	Ppp1r21	0	BRDN0000737457	0
Bbs9	0	BRDN0000737458	0	Pak7	0	BRDN0000737456	0
Bbs7	0	BRDN0000737457	0	Sema5b	0	BRDN0000737455	0
Bbs5	0	BRDN0000737456	0	Mtf1	0	BRDN0000737454	0
Bbs2	0	BRDN0000737455	0	Fam214a	0	BRDN0000737453	0
Bbs12	0	BRDN0000737454	0	Fam214b	0	BRDN0000737451	0
Bbs10	0	BRDN0000737453	0	Mtf2	0	BRDN0000737450	0
Bbs1	0	BRDN0000737451	0	Vkorc1	0	BRDN0000737448	0
Bbox1	0	BRDN0000737450	0	Ppl	0	BRDN0000737447	0
Bbip1	0	BRDN0000737448	0	4930402K13Rik	0	BRDN0000737446	0
Bbc3	0	BRDN0000737446	0	Ccdc58	0	BRDN0000737445	0
Baz2b	0	BRDN0000737444	0	BRDN0000737935	0	BRDN0000737444	0
Baz2a	0	BRDN0000737443	0	BRDN0000737934	0	BRDN0000737442	0
Baz1b	0	BRDN0000737441	0	BRDN0000737933	0	BRDN0000737441	0
Baz1a	0	BRDN0000737440	0	BRDN0000737932	0	BRDN0000737440	0
Bax	0	BRDN0000737439	0	BC107364	0	BRDN0000737439	0
Batf3	0	BRDN0000737438	0	Dlx4	0	BRDN0000737438	0
Batf2	0	BRDN0000737436	0	Dlx5	0	BRDN0000737437	0
Batf	0	BRDN0000737434	0	Mpp2	0	BRDN0000737436	0
Barx2	0	BRDN0000737433	0	Teddm1b	0	BRDN0000737435	0
Barx1	0	BRDN0000737431	0	Deb1	0	BRDN0000737434	0
Barhl2	0	BRDN0000737430	0	Dlx2	0	BRDN0000737433	0
Barhl1	0	BRDN0000737429	0	BRDN0000737938	0	BRDN0000737431	0
Bap1	0	BRDN0000737427	0	Trim66	0	BRDN0000737430	0
Banf1	0	BRDN0000737426	0	Oscp1	0	BRDN0000737429	0
Bambi	0	BRDN0000737425	0	Tmx2	0	BRDN0000737427	0
Bak1	0	BRDN0000737424	0	Myl6	0	BRDN0000737426	0
Baiap2l1	0	BRDN0000737423	0	Nrip1	0	BRDN0000737425	0
Baiap2	0	BRDN0000737422	0	Nrip2	0	BRDN0000737424	0
Bag6	0	BRDN0000737421	0	Nrip3	0	BRDN0000737423	0
Bag5	0	BRDN0000737419	0	Sec1	0	BRDN0000737422	0
Bag4	0	BRDN0000737418	0	Cxxc1	0	BRDN0000737421	0
Bag3	0	BRDN0000737416	0	Opa3	0	BRDN0000737418	0
Bag2	0	BRDN0000737414	0	Cxxc5	0	BRDN0000737417	0
Bag1	0	BRDN0000737411	0	6430548M08Rik	0	BRDN0000737416	0
Bach2	0	BRDN0000737410	0	Aars2	0	BRDN0000737415	0
Bach1	0	BRDN0000737409	0	N4bp2l1	0	BRDN0000737414	0
Bace2	0	BRDN0000737408	0	Dimt1	0	BRDN0000737413	0
Bace1	0	BRDN0000737406	0	Cycs	0	BRDN0000737412	0
Babam1	0	BRDN0000737405	0	Cd151	0	BRDN0000737411	0
Baat	0	BRDN0000737404	0	Vav1	0	BRDN0000737410	0
B9d2	0	BRDN0000737402	0	Zbp1	0	BRDN0000737409	0
B9d1	0	BRDN0000737401	0	Vav3	0	BRDN0000737408	0
B930041F14Rik	0	BRDN0000737400	0	Cyp4f40	0	BRDN0000737406	0
B4galt7	0	BRDN0000737399	0	Vgll1	0	BRDN0000737405	0
B4galt6	0	BRDN0000737398	0	Lancl2	0	BRDN0000737404	0

B4galt5	0
B4galt4	0
B4galt2	0
B4galt1	0
B4galnt4	0
B4galnt3	0
B4galnt2	0
B4galnt1	0
B430306N03Rik	0
B3gntl1	0
B3gnt9	0
B3gnt7	0
B3gnt6	0
B3gnt5	0
B3gnt2	0
B3glct	0
B3gat3	0
B3gat2	0
B3gat1	0
B3galt6	0
B3galt5	0
B3galt4	0
B3galt2	0
B3galt1	0
B3galnt2	0
B3galnt1	0
B230216G23Rik	0
B230118H07Rik	0
B020031M17Rik	0
B020004J07Rik	0
B020004C17Rik	0
Azin1	0
Azi2	0
Azgp1	0
Aym1	0
AY761185	0
AY358078	0
AY074887	0
Axl	0
Axin2	0
Axin1	0
Awat1	0
AW551984	0
AW549877	0
AW209491	0
AW146154	0
Avpr2	0
Avpr1b	0
Avpr1a	0
Avpi1	0
Avp	0
Avl9	0
Avil	0
Aven	0
AV320801	0
Aurkc	0
Aurkb	0
Aurkaip1	0
Aurka	0
Auh	0
AU040320	0
AU022751	0
AU022252	0
AU019823	0
AU018091	0
Atxn7l1	0
Atxn7	0
Atxn3	0
Atxn2l	0
Atxn2	0
Atxn1l	0
Atxn10	0
Atxn1	0
Atrx	0
Atrnl1	0
Atrip	0
Atraid	0
Atr	0
Atpaf2	0
Atpaf1	0
Atp9a	0
Atp8b5	0

BRDN0000737397	0
BRDN0000737396	0
BRDN0000737395	0
BRDN0000737394	0
BRDN0000737393	0
BRDN0000737392	0
BRDN0000737391	0
BRDN0000737390	0
BRDN0000737389	0
BRDN0000737388	0
BRDN0000737387	0
BRDN0000737384	0
BRDN0000737383	0
BRDN0000737380	0
Brd8	0
Brd3	0
Brd2	0
Brcc3	0
Brca2	0
Brca1	0
Brat1	0
Brap	0
Bptf	0
Bpifb9b	0
Bpifb6	0
Bpifb5	0
Bpifb2	0
Bpifa5	0
Bpifa3	0
Bpifa2	0
Bpifa1	0
Bphl	0
Bpgm	0
Bora	0
Boll	0
Bola1	0
Bod1l	0
Boc	0
Bnipl	0
Bnip3l	0
Bnip3	0
Bnip2	0
Bmyc	0
Bmx	0
Bms1	0
Bmpr1b	0
Bmpr1a	0
Bmper	0
Bmp8b	0
Bmp8a	0
Bmp7	0
Bmp5	0
Bmp4	0
Bmp3	0
Bmp2k	0
Bmp15	0
Bmp10	0
Bmp1	0
Bmf	0
Blzf1	0
Blvra	0
Bloc1s3	0
Bloc1s2	0
Bloc1s1	0
Blnk	0
Blcap	0
Bivm	0
Birc7	0
Birc5	0
Birc3	0
Bin3	0
Bin2	0
Bin1	0
Bicd2	0
Bicc1	0
Bhmt2	0
Bhmt	0
Bhlhe40	0
Bhlhe23	0
Bhlha15	0
Bgn	0
Bglap2	0

AI987944	0
Lancl1	0
Mcpt8	0
Rhox2g	0
D2Wsu81e	0
Adgrb1	0
Adgrb2	0
Kin	0
Cenpn	0
Nr3c1	0
Gm20738	0
Cenpk	0
Cenpj	0
Cenph	0
Rph3al	0
Cenpf	0
Cenpe	0
Nop2	0
Gm20736	0
Dgkb	0
Gne	0
Ucn2	0
Eprs	0
Cenpt	0
Cenpq	0
Cenpp	0
Nhlrc3	0
Nhlrc1	0
Nav2	0
Cyp2j6	0
Zfp110	0
Zfp111	0
Cyp2j8	0
Nudt3	0
Zfp114	0
Nudt5	0
Nudt6	0
Smpd5	0
Smpd4	0
Vmn2r110	0
Smpd1	0
Hand1	0
Smpd3	0
Smpd2	0
BRDN0000738132	0
Vmn2r117	0
BRDN0000738130	0
Cpvl	0
BRDN0000737663	0
Gm4559	0
BRDN0000737661	0
1700040L02Rik	0
Sdk1	0
BRDN0000738214	0
Idh2	0
Idh1	0
1700125H20Rik	0
Evx1	0
Evx2	0
Ythdf3	0
Ythdf2	0
Ythdf1	0
Ccdc28a	0
Snrpn	0
BRDN0000737974	0
Snrpb	0
Scgb2b27	0
Snrpa	0
Snrpf	0
Snrpg	0
Snrpe	0
C8g	0
Dgka	0
Csnka2ip	0
Suds3	0
BRDN0000737599	0
Lrfn2	0
Tpgs2	0
Mlc1	0
Olfr1013	0
Olfr118	0
Olfr119	0

BRDN0000737403	0
BRDN0000737402	0
BRDN0000737401	0
BRDN0000737400	0
BRDN0000737399	0
BRDN0000737398	0
BRDN0000737397	0
BRDN0000737396	0
BRDN0000737395	0
BRDN0000737394	0
BRDN0000737393	0
BRDN0000737392	0
BRDN0000737391	0
BRDN0000737390	0
BRDN0000737389	0
BRDN0000737388	0
BRDN0000737387	0
BRDN0000737386	0
BRDN0000737385	0
BRDN0000737384	0
BRDN0000737383	0
BRDN0000737382	0
BRDN0000737381	0
BRDN0000737380	0
Brd9	0
Brd3	0
Brd2	0
Brcc3	0
Brca2	0
Brca1	0
Brat1	0
Brap	0
Bptf	0
Bpnt1	0
Bpifb9b	0
Bpifb9a	0
Bpifb6	0
Bpifb5	0
Bpifb4	0
Bpifb2	0
Bpifb1	0
Bpifa5	0
Bpifa3	0
Bpi	0
Bop1	0
Bola3	0
Bola2	0
Bod1l	0
Boc	0
Bnip3l	0
Bnip3	0
Bnip1	0
Bnc2	0
Bmyc	0
Bmx	0
Bms1	0
Bmpr2	0
Bmpr1a	0
Bmp8b	0
Bmp8a	0
Bmp6	0
Bmp3	0
Bmp2k	0
Bmp2	0
Bmp1	0
Bmi1	0
Blzf1	0
Bloc1s5	0
Bloc1s4	0
Bloc1s2	0
Bloc1s1	0
Blnk	0
Blmh	0
Blm	0
Bivm	0
Birc7	0
Birc6	0
Birc5	0
Birc3	0
Bin3	0
Bin1	0
Bik	0

Atp8b4	0	Bglap	0	Olfr116	0	Bid	0
Atp8b1	0	Bfsp2	0	Olfr117	0	Bicd2	0
Atp8a2	0	Bfsp1	0	Olfr115	0	Bhmt	0
Atp8a1	0	Bex6	0	Olfr112	0	Bhlhe40	0
Atp7b	0	Bex4	0	Olfr113	0	Bhlhe23	0
Atp7a	0	Bex2	0	Olfr110	0	Bhlhb9	0
Atp6v1h	0	Bex1	0	Eme2	0	Bgn	0
Atp6v1g3	0	Bet1l	0	2410016O06Rik	0	Bglap2	0
Atp6v1g2	0	Bet1	0	BC051142	0	Bglap	0
Atp6v1g1	0	Best2	0	Poc1a	0	Bex6	0
Atp6v1f	0	Best1	0	Epx	0	Bex4	0
Atp6v1e1	0	Bend5	0	Ptn	0	Bex1	0
Atp6v1d	0	Bend4	0	Asns	0	Bet1	0
Atp6v1c2	0	Bend3	0	Lrfn1	0	Best3	0
Atp6v1c1	0	Begain	0	Epo	0	Best2	0
Atp6v1b2	0	Becn1	0	Ecsit	0	Best1	0
Atp6v1b1	0	Bean1	0	Nudt1	0	Bend5	0
Atp6v1a	0	Bdkrb1	0	Celf5	0	Bend4	0
Atp6v0e2	0	Bdh2	0	Fbxw7	0	Bend3	0
Atp6v0e	0	Bcr	0	Chmp2b	0	Begain	0
Atp6v0d2	0	Bcorl1	0	Lhfpl5	0	Becn1	0
Atp6v0d1	0	Bco2	0	Muc1	0	Bean1	0
Atp6v0c	0	Bcl7c	0	Slc2a13	0	Bdp1	0
Atp6v0b	0	Bcl7b	0	Slc2a12	0	Bdnf	0
Atp6ap1	0	Bcl7a	0	Slc2a10	0	Bdh2	0
Atp5sl	0	Bcl6	0	Pou6f1	0	Bdh1	0
Atp5s	0	Bcl2l2	0	Rcsd1	0	Bcr	0
Atp5o	0	Bcl2l15	0	Celf4	0	Bco2	0
Atp5l	0	Bcl2l14	0	Atr	0	Bco1	0
Atp5k	0	Bcl2l13	0	Zfp800	0	Bcl9l	0
Atp5j2	0	Bcl2l12	0	5830415F09Rik	0	Bcl9	0
Atp5j	0	Bcl2l10	0	Lrat	0	Bcl7b	0
Atp5h	0	Bcl2l1	0	Ptpdc1	0	Bcl3	0
Atp5g3	0	Bcl2a1d	0	Pthlh	0	Bcl2l15	0
Atp5g2	0	Bcl2a1c	0	Msn	0	Bcl2l14	0
Atp5g1	0	Bcl2a1a	0	Etnk2	0	Bcl2l13	0
Atp5e	0	Bcl2	0	Etnk1	0	Bcl2l12	0
Atp5d	0	Bcl11b	0	Ttc39c	0	Bcl2l11	0
Atp5c1	0	Bcl11a	0	Ttc39b	0	Bcl2l10	0
Atp5b	0	Bcl10	0	Ttc39a	0	Bcl2l1	0
Atp5a1	0	Bckdk	0	Msc	0	Bcl2a1d	0
Atp4b	0	Bche	0	Ttc39d	0	Bcl2a1c	0
Atp4a	0	Bccip	0	Ptov1	0	Bcl2a1b	0
Atp2c2	0	Bcas3	0	Llgl1	0	Bcl2a1a	0
Atp2b4	0	Bcar1	0	Gpat2	0	Bcl11b	0
Atp2b3	0	Bcap31	0	BRDN0000738246	0	Bcl10	0
Atp2b2	0	Bcan	0	Leo1	0	Bckdk	0
Atp2b1	0	Bcam	0	Ogn	0	Bckdha	0
Atp2a3	0	BC147527	0	Necab3	0	Bcdin3d	0
Atp2a1	0	BC107364	0	Necab2	0	Bccip	0
Atp1b4	0	BC100530	0	Necab1	0	Bcat1	0
Atp1b3	0	BC094916	0	Eif4h	0	Bcas3	0
Atp1b2	0	BC080695	0	Ptpn22	0	Bcas2	0
Atp1b1	0	BC068281	0	Slc9a5	0	Bcap31	0
Atp1a4	0	BC061212	0	Ptpn20	0	Bcan	0
Atp1a2	0	BC055324	0	Slc9a7	0	Bcam	0
Atp1a1	0	BC049762	0	Slc9a1	0	BC147527	0
Atp13a5	0	BC049730	0	Slc9a2	0	BC117090	0
Atp13a4	0	BC049715	0	Slc9a3	0	BC107364	0
Atp13a3	0	BC049352	0	Glra3	0	BC100530	0
Atp12a	0	BC048671	0	Noc3l	0	BC100451	0
Atp11c	0	BC048562	0	Glra1	0	BC089491	0
Atp11a	0	BC048546	0	Hhla1	0	BC080695	0
Atp10b	0	BC048502	0	Slc9a9	0	BC068281	0
Atp10a	0	BC048403	0	Pcdhga11	0	BC061237	0
Atox1	0	BC037034	0	Ap3s2	0	BC061212	0
Atoh7	0	BC035044	0	Olfr1491	0	BC055324	0
Atoh1	0	BC030867	0	Ankrd53	0	BC055111	0
Atn1	0	BC030336	0	Ap3s1	0	BC052040	0
Atmin	0	BC030307	0	Tns2	0	BC051628	0
Atm	0	BC029214	0	Ms4a7	0	BC049762	0
Atl3	0	BC028528	0	Ms4a5	0	BC049730	0
Atl2	0	BC027231	0	Ms4a2	0	BC049635	0
Atl1	0	BC027072	0	Ms4a3	0	BC049352	0
Atic	0	BC026585	0	Ms4a1	0	BC048679	0
Athl1	0	BC024978	0	Ubxn7	0	BC048671	0
Atg9a	0	BC024139	0	4833423E24Rik	0	BC048609	0
Atg7	0	BC022687	0	Rttn	0	BC048562	0
Atg5	0	BC021785	0	Invs	0	BC048546	0
Atg4c	0	BC017643	0	Olfr1494	0	BC048507	0
Atg4b	0	BC016579	0	Ankrd50	0	BC048502	0
Atg2b	0	BC005561	0	Sypl	0	BC048403	0

Atg16l1	0
Atg14	0
Atg13	0
Atg12	0
Atg101	0
Atg10	0
Atf7ip2	0
Atf7ip	0
Atf6b	0
Atf6	0
Atf5	0
Atf4	0
Atf3	0
Atf2	0
Atf1	0
Ate1	0
Atcay	0
Atad5	0
Atad3a	0
Atad2	0
Atad1	0
Asz1	0
Asxl3	0
Asxl2	0
Asxl1	0
Asun	0
Astn2	0
Astn1	0
Aspscr1	0
Asprv1	0
Asphd2	0
Asphd1	0
Asph	0
Aspg	0
Aspdh	0
Aspa	0
Asnsd1	0
Asns	0
Asna1	0
Asic5	0
Asic4	0
Asic2	0
Asic1	0
Ash2l	0
Ash1l	0
Asgr2	0
Ascl5	0
Ascl3	0
Ascl2	0
Ascl1	0
Ascc2	0
Ascc1	0
Asb9	0
Asb8	0
Asb6	0
Asb5	0
Asb4	0
Asb3	0
Asb2	0
Asb18	0
Asb17	0
Asb16	0
Asb15	0
Asb13	0
Asb10	0
Asb1	0
Asap3	0
Asap2	0
Asap1	0
Asah2	0
Arxes2	0
Arxes1	0
Arvcf	0
Arv1	0
Artn	0
Art5	0
Art3	0
Art2b	0
Art1	0
Arsk	0
Arsj	0
Arsi	0
BC005537	0
BC004004	0
BC003331	0
Bbx	0
Bbs9	0
Bbs7	0
Bbs4	0
Bbs12	0
Bbs10	0
Bbs1	0
Bbox1	0
Bbip1	0
Baz2b	0
Baz2a	0
Baz1b	0
Baz1a	0
Bax	0
Batf3	0
Batf2	0
Batf	0
Basp1	0
Barx1	0
Barhl2	0
Bank1	0
Banf1	0
Bak1	0
Baiap3	0
Baiap2l1	0
Bag6	0
Bag4	0
Bag2	0
Bad	0
Bach2	0
Bach1	0
Bace1	0
B9d2	0
B630005N14Rik	0
B4gat1	0
B4galt7	0
B4galt6	0
B4galt5	0
B4galt3	0
B4galt1	0
B4galnt4	0
B4galnt3	0
B4galnt1	0
B3gntl1	0
B3gnt9	0
B3gnt8	0
B3gnt7	0
B3gnt6	0
B3gnt5	0
B3gnt4	0
B3gnt3	0
B3glct	0
B3gat3	0
B3gat2	0
B3gat1	0
B3galt6	0
B3galt5	0
B3galt4	0
B3galt2	0
B3galnt1	0
B2m	0
B230217C12Rik	0
B230216G23Rik	0
B230118H07Rik	0
B020004J07Rik	0
B020004C17Rik	0
Azin2	0
Azin1	0
Azi2	0
Azgp1	0
Aym1	0
AY761185	0
AY761184	0
AY358078	0
AY074887	0
Axl	0
Axin2	0
Axin1	0
Awat2	0
Eif4b	0
Nab2	0
Hint3	0
BRDN0000737694	0
Cyp4f37	0
H60b	0
H60c	0
D930020B18Rik	0
BRDN0000738119	0
Ubtf	0
Fcho2	0
Fxr1	0
Fxr2	0
Zfp758	0
Zfp759	0
Prg2	0
Masp2	0
Gjb6	0
Gykl1	0
Tagap1	0
Hook2	0
Hook3	0
1110051M20Rik	0
Hook1	0
Khdc3	0
Iapp	0
Esp8	0
Usp17lb	0
Usp17la	0
Phospho2	0
Phospho1	0
Usp17ld	0
Edn1	0
Esp1	0
Edn3	0
Edn2	0
Esp4	0
Esp6	0
Srcap	0
Gpr37l1	0
Slc22a29	0
Lhcgr	0
Slc22a23	0
Slc22a22	0
Slc22a21	0
Slc22a20	0
Slc22a27	0
Slc22a26	0
Glud1	0
Foxp1	0
Ssu72	0
Igll1	0
Rbfox3	0
Cav3	0
Rbfox2	0
Zfp750	0
Rbm41	0
Rbm42	0
Rbm43	0
Rbm44	0
Rbm45	0
Espn	0
Larp7	0
Rbm48	0
Rbp1	0
Rps27a	0
Dpp8	0
Gm6812	0
Mkrn2os	0
Gm1661	0
Klrg2	0
Primpol	0
Dpp3	0
Dpp4	0
Dpp6	0
Dpp7	0
Olfr981	0
Sh2d5	0
Ankrd55	0
Sh2d7	0
Gm5549	0
Aktip	0
BC031181	0
BC030336	0
BC030307	0
BC029214	0
BC028528	0
BC027231	0
BC027072	0
BC026585	0
BC024978	0
BC024139	0
BC021891	0
BC021785	0
BC021614	0
BC017643	0
BC017158	0
BC016579	0
BC005624	0
BC005561	0
BC005537	0
BC004004	0
BC003965	0
Bbs9	0
Bbs7	0
Bbs5	0
Bbs2	0
Bbs10	0
Bbs1	0
Bbox1	0
Baz2a	0
Baz1b	0
Baz1a	0
Bax	0
Batf2	0
Batf	0
Barx1	0
Barhl1	0
Bard1	0
Bap1	0
Banf1	0
Bambi	0
Bak1	0
Baiap2l2	0
Baiap2l1	0
Baiap2	0
Bahd1	0
Bag6	0
Bag5	0
Bag3	0
Bag2	0
Bad	0
Bach2	0
Bach1	0
Bace2	0
Babam1	0
B9d1	0
B930041F14Rik	0
B630005N14Rik	0
B4galt7	0
B4galt6	0
B4galt5	0
B4galt4	0
B4galt3	0
B4galt1	0
B4galnt4	0
B4galnt3	0
B4galnt2	0
B430306N03Rik	0
B3gntl1	0
B3gnt7	0
B3gnt5	0
B3gnt4	0
B3gnt3	0
B3gnt2	0
B3gat2	0
B3gat1	0
B3galt6	0
B3galt5	0
B3galt4	0
B3galt2	0
B3galnt2	0
B3galnt1	0
B2m	0

Arsg	0	Awat1	0	2510003E04Rik	0	B230219D22Rik	0
Arsb	0	AW551984	0	Tpbpa	0	B230216G23Rik	0
Arsa	0	AW549877	0	Tmcc2	0	B020031M17Rik	0
Arrdc5	0	AW209491	0	Tmcc3	0	B020004C17Rik	0
Arrdc3	0	Avpr2	0	Oprd1	0	Azin2	0
Arrdc2	0	Avpr1a	0	Prl8a1	0	Azi2	0
Arrdc1	0	Avpi1	0	BRDN0000737547	0	Azgp1	0
Arrb2	0	Avp	0	Fsd1l	0	Aym1	0
Arrb1	0	Avl9	0	Sim2	0	AY761185	0
Arr3	0	Aven	0	Sim1	0	AY358078	0
Arpp21	0	Aurkc	0	Col4a3bp	0	Axin1	0
Arpp19	0	Aurkaip1	0	Mfsd7b	0	AW551984	0
Arpc5l	0	Aurka	0	Mfsd7a	0	AW549877	0
Arpc5	0	AU040320	0	1110004E09Rik	0	AW146154	0
Arpc4	0	AU022751	0	4931440F15Rik	0	Avpr2	0
Arpc3	0	AU021092	0	Col20a1	0	Avpr1a	0
Arpc2	0	AU019823	0	Slc39a8	0	Avil	0
Arntl	0	AU018091	0	Slc39a9	0	AV320801	0
Arnt2	0	Atxn7l3	0	Krt31	0	Aurkb	0
Arnt	0	Atxn7l1	0	Pcp4	0	Aurkaip1	0
Armt1	0	Atxn2	0	Macrod2	0	Aurka	0
Armcx6	0	Atxn1l	0	Macrod1	0	Auh	0
Armcx5	0	Atxn10	0	Slc39a1	0	AU040320	0
Armcx4	0	Atxn1	0	Slc39a2	0	AU022751	0
Armcx3	0	Atrnl1	0	Slc39a5	0	AU021092	0
Armcx2	0	Atrip	0	Ggnbp2	0	Atxn7l3b	0
Armcx1	0	Atraid	0	Slc39a7	0	Atxn7l3	0
Armc9	0	Atr	0	Sumf2	0	Atxn7l2	0
Armc7	0	Atpaf2	0	Ctsll3	0	Atxn7l1	0
Armc6	0	Atpaf1	0	Sumf1	0	Atxn7	0
Armc5	0	Atp9a	0	Glipr2	0	Atxn3	0
Armc4	0	Atp8b5	0	Glipr1	0	Atxn2l	0
Armc3	0	Atp8b4	0	Rpl24	0	Atxn2	0
Armc2	0	Atp8b3	0	Gys1	0	Atxn10	0
Armc12	0	Atp8b1	0	Gys2	0	Atxn1	0
Arl9	0	Atp8a1	0	Gm561	0	Atrnl1	0
Arl8a	0	Atp7b	0	Surf1	0	Atrn	0
Arl6ip6	0	Atp6v1h	0	Alg5	0	Atrip	0
Arl6ip5	0	Atp6v1g1	0	Surf4	0	Atraid	0
Arl6ip1	0	Atp6v1f	0	Surf6	0	Atr	0
Arl6	0	Atp6v1e2	0	Nipal1	0	Atpaf2	0
Arl5b	0	Atp6v1e1	0	Ddx58	0	Atp9b	0
Arl4d	0	Atp6v1d	0	Ddx59	0	Atp9a	0
Arl4c	0	Atp6v1c2	0	Pbp2	0	Atp8b5	0
Arl3	0	Atp6v1b2	0	Gpr141	0	Atp8b3	0
Arl2bp	0	Atp6v1b1	0	Bpgm	0	Atp8b2	0
Arl2	0	Atp6v1a	0	Gpr143	0	Atp8b1	0
Arl16	0	Atp6v0e2	0	Ddx52	0	Atp8a2	0
Arl15	0	Atp6v0d2	0	Ddx51	0	Atp8a1	0
Arl14epl	0	Atp6v0c	0	Ddx56	0	Atp7b	0
Arl14	0	Atp6v0b	0	Vmn1r196	0	Atp7a	0
Arl13b	0	Atp6v0a1	0	Ddx54	0	Atp6v1h	0
Arl13a	0	Atp6ap2	0	Ddx55	0	Atp6v1g3	0
Arl11	0	Atp6ap1l	0	Rgsl1	0	Atp6v1g2	0
Arl10	0	Atp6ap1	0	Whsc1l1	0	Atp6v1g1	0
Arid5b	0	Atp5sl	0	Vmn1r197	0	Atp6v1f	0
Arid5a	0	Atp5s	0	Dcaf10	0	Atp6v1e2	0
Arid4b	0	Atp5o	0	Hnrnpa2b1	0	Atp6v1e1	0
Arid3c	0	Atp5l	0	Myct1	0	Atp6v1d	0
Arid3b	0	Atp5k	0	Sipa1l1	0	Atp6v1c1	0
Arid3a	0	Atp5j2	0	Pstpip2	0	Atp6v1b2	0
Arid1a	0	Atp5j	0	Sipa1l2	0	Atp6v1b1	0
Arhgef9	0	Atp5h	0	AI464131	0	Atp6v1a	0
Arhgef7	0	Atp5g3	0	Atn1	0	Atp6v0e2	0
Arhgef6	0	Atp5g1	0	Blmh	0	Atp6v0d2	0
Arhgef5	0	Atp5e	0	Fundc2	0	Atp6v0d1	0
Arhgef40	0	Atp5d	0	Dennd5b	0	Atp6v0c	0
Arhgef39	0	Atp5c1	0	Fundc1	0	Atp6v0b	0
Arhgef37	0	Atp5b	0	Magi1	0	Atp6v0a4	0
Arhgef33	0	Atp5a1	0	Gm21637	0	Atp6v0a1	0
Arhgef3	0	Atp4a	0	Arhgap44	0	Atp6ap2	0
Arhgef18	0	Atp2c2	0	Hexa	0	Atp6ap1l	0
Arhgef17	0	Atp2c1	0	Esm1	0	Atp5s	0
Arhgef16	0	Atp2b4	0	Hexb	0	Atp5o	0
Arhgef15	0	Atp2b3	0	Krt32	0	Atp5k	0
Arhgef12	0	Atp2b2	0	Spsb4	0	Atp5j	0
Arhgef11	0	Atp2a1	0	Prph	0	Atp5h	0
Arhgef10l	0	Atp1b2	0	C5ar2	0	Atp5g3	0
Arhgef10	0	Atp1b1	0	C5ar1	0	Atp5g2	0
Arhgef1	0	Atp1a4	0	Dcaf7	0	Atp5e	0
Arhgdig	0	Atp13a4	0	Dcaf6	0	Atp5d	0
Arhgdib	0	Atp13a2	0	Flot2	0	Atp5c1	0

Arhgap9	0	Atp11b	0	Dcaf4	0	Atp5a1	0
Arhgap8	0	Atp10b	0	Nxnl1	0	Atp4b	0
Arhgap5	0	Atn1	0	Gabrb1	0	Atp4a	0
Arhgap42	0	Atmin	0	Gabrb2	0	Atp2c2	0
Arhgap40	0	Atm	0	Dcaf8	0	Atp2c1	0
Arhgap4	0	Atl3	0	Slc6a11	0	Atp2b4	0
Arhgap39	0	Atl1	0	Lrig2	0	Atp2b3	0
Arhgap36	0	Atic	0	Pdcd2l	0	Atp2b1	0
Arhgap35	0	Athl1	0	Trim75	0	Atp2a2	0
Arhgap32	0	Atg9b	0	Hoxb9	0	Atp2a1	0
Arhgap31	0	Atg7	0	Trafd1	0	Atp1b4	0
Arhgap28	0	Atg5	0	Trim72	0	Atp1b1	0
Arhgap26	0	Atg4d	0	Gm13290	0	Atp1a1	0
Arhgap25	0	Atg4c	0	Tmed1	0	Atp13a3	0
Arhgap24	0	Atg4b	0	Hoxb2	0	Atp13a2	0
Arhgap22	0	Atg16l2	0	Vmn1r198	0	Atp13a1	0
Arhgap19	0	Atg16l1	0	Gm7030	0	Atp11c	0
Arhgap18	0	Atg14	0	Arhgap42	0	Atp11a	0
Arhgap15	0	Atg13	0	Lins	0	Atp10b	0
Arhgap12	0	Atg12	0	Hoxb7	0	Atp10a	0
Arhgap11a	0	Atf7ip2	0	Hoxb4	0	Atox1	0
Arhgap10	0	Atf7ip	0	Hoxb5	0	Atoh7	0
Arhgap1	0	Atf6b	0	Gm3238	0	Atoh1	0
Arglu1	0	Atf4	0	Cd81	0	Atmin	0
Arg2	0	Atf3	0	Ctr9	0	Atm	0
Arg1	0	Atf2	0	Pcdhgc3	0	Atl3	0
Arfrp1	0	Atf1	0	Pcdhgc5	0	Atl2	0
Arfip2	0	Ate1	0	Wrap53	0	Atl1	0
Arfip1	0	Atcay	0	Thada	0	Atg9b	0
Arfgef3	0	Atat1	0	Hs3st3b1	0	Atg9a	0
Arfgef1	0	Atad3a	0	Stat6	0	Atg7	0
Arfgap2	0	Atad2b	0	Stat4	0	Atg5	0
Arf6	0	Atad1	0	Stat3	0	Atg4c	0
Arf5	0	Asz1	0	Sbpl	0	Atg3	0
Arf4	0	Asxl3	0	Lrrfip1	0	Atg2b	0
Arf3	0	Asun	0	Cep250	0	Atg2a	0
Arf1	0	Astn2	0	Ppfia4	0	Atg16l2	0
Arel1	0	Astn1	0	Olfr569	0	Atg14	0
Areg	0	Asrgl1	0	Olfr568	0	Atg13	0
Arcn1	0	Aspscr1	0	Itgb7	0	Atg12	0
Arc	0	Asphd2	0	BRDN0000737433	0	Atg101	0
Arap3	0	Asphd1	0	BRDN0000737430	0	Atg10	0
Arap2	0	Asph	0	BRDN0000737431	0	Atf7ip2	0
Arap1	0	Aspdh	0	Prp2	0	Atf7ip	0
Araf	0	Asnsd1	0	Sepsecs	0	Atf6b	0
Aqr	0	Asns	0	BRDN0000737434	0	Atf6	0
Aqp9	0	Asna1	0	BRDN0000737435	0	Atf4	0
Aqp7	0	Asl	0	Zyg11b	0	Atf2	0
Aqp5	0	Asic2	0	Nfe2	0	Atf1	0
Aqp2	0	Asic1	0	2510039O18Rik	0	Atcay	0
Aptx	0	Ash1l	0	Egfl7	0	Atat1	0
Aprt	0	Asgr2	0	B4galt2	0	Atad5	0
Appl2	0	Asf1b	0	Lin9	0	Atad3a	0
Appl1	0	Ascl5	0	Ctrl	0	Atad2	0
App	0	Ascl1	0	Ctrc	0	Atad1	0
Apopt1	0	Ascc3	0	Gm10354	0	Asz1	0
Apool	0	Ascc2	0	Xlr4b	0	Asxl3	0
Apon	0	Asb8	0	Kif26b	0	Asxl2	0
Apold1	0	Asb6	0	Rundc3a	0	Asxl1	0
Apol9b	0	Asb4	0	Olfr155	0	Asun	0
Apol9a	0	Asb3	0	Palm	0	Astn1	0
Apol7e	0	Asb2	0	Chmp3	0	Asrgl1	0
Apol7c	0	Asb18	0	Krtap7-1	0	Aspscr1	0
Apol7b	0	Asb17	0	Hmx1	0	Asprv1	0
Apol7a	0	Asb16	0	Ube4b	0	Aspn	0
Apol6	0	Asb14	0	Ube4a	0	Asphd2	0
Apol11a	0	Asb13	0	Hmx2	0	Aspg	0
Apol10a	0	Asb10	0	Defa22	0	Aspdh	0
Apoh	0	Asap3	0	Defa21	0	Asnsd1	0
Apof	0	Asap1	0	Gyk	0	Asns	0
Apod	0	Asah2	0	Pex19	0	Asna1	0
Apoc4	0	Asah1	0	Tpte	0	Asl	0
Apoc3	0	Arxes2	0	Tex261	0	Asic4	0
Apoc2	0	Arvcf	0	Rsph3a	0	Asic2	0
Apoc1	0	Arv1	0	Tex264	0	Asic1	0
Apobr	0	Art5	0	Pex10	0	Asgr2	0
Apobec4	0	Art3	0	Pex13	0	Asgr1	0
Apobec3	0	Art1	0	Pex12	0	Asf1b	0
Apobec2	0	Arsi	0	Pex14	0	Ascl5	0
Apobec1	0	Arsg	0	Tufm	0	Ascl4	0
Apoa5	0	Arrdc5	0	Dock7	0	Ascl2	0
Apoa4	0	Arrdc4	0	Tuba3a	0	Ascl1	0

Apoa2	0
Apoa1bp	0
Apoa1	0
Apmap	0
Aplp2	0
Aplp1	0
Aplnr	0
Aplf	0
Apitd1	0
Apip	0
Aph1c	0
Aph1b	0
Apex2	0
Apcs	0
Apcdd1	0
Apc2	0
Apc	0
Apbb3	0
Apbb2	0
Apbb1	0
Apba3	0
Apba2	0
Apba1	0
Apaf1	0
Ap5s1	0
Ap5m1	0
Ap5b1	0
Ap4m1	0
Ap4b1	0
Ap3s2	0
Ap3m2	0
Ap3m1	0
Ap3d1	0
Ap3b2	0
Ap3b1	0
Ap2s1	0
Ap2m1	0
Ap2b1	0
Ap2a2	0
Ap2a1	0
Ap1s3	0
Ap1s2	0
Ap1s1	0
Ap1m2	0
Ap1m1	0
Ap1b1	0
Ap1ar	0
Aox4	0
Aox3	0
Aox2	0
Aox1	0
Aoc2	0
Anxa9	0
Anxa8	0
Anxa7	0
Anxa6	0
Anxa5	0
Anxa3	0
Anxa2	0
Anxa11	0
Anxa10	0
Anxa1	0
Antxrl	0
Antxr2	0
Antxr1	0
Anpep	0
Anp32e	0
Anp32b	0
Ano9	0
Ano8	0
Ano7	0
Ano6	0
Ano5	0
Ano3	0
Ano1	0
Anln	0
Ankzf1	0
Ankub1	0
Anks6	0
Anks4b	0
Anks3	0
Anks1b	0
Arrdc3	0
Arrb1	0
Arpp21	0
Arpp19	0
Arpc5l	0
Arpc5	0
Arpc4	0
Arpc3	0
Arpc2	0
Arpc1b	0
Arntl2	0
Arnt2	0
Arnt	0
Armcx5	0
Armcx3	0
Armc9	0
Armc8	0
Armc7	0
Armc6	0
Armc4	0
Armc2	0
Armc10	0
Arl9	0
Arl6ip6	0
Arl6ip5	0
Arl6ip4	0
Arl6ip1	0
Arl6	0
Arl5c	0
Arl5a	0
Arl4d	0
Arl4c	0
Arl4a	0
Arl2bp	0
Arl2	0
Arl15	0
Arl14epl	0
Arl14	0
Arl13a	0
Arl11	0
Arl1	0
Arih2	0
Arih1	0
Arid5b	0
Arid5a	0
Arid4b	0
Arid3c	0
Arid3a	0
Arid2	0
Arid1b	0
Arid1a	0
Arhgef9	0
Arhgef6	0
Arhgef40	0
Arhgef39	0
Arhgef3	0
Arhgef2	0
Arhgef19	0
Arhgef18	0
Arhgef17	0
Arhgef12	0
Arhgef11	0
Arhgef10l	0
Arhgef10	0
Arhgef1	0
Arhgdig	0
Arhgap9	0
Arhgap44	0
Arhgap4	0
Arhgap39	0
Arhgap36	0
Arhgap35	0
Arhgap33	0
Arhgap31	0
Arhgap30	0
Arhgap29	0
Arhgap27	0
Arhgap26	0
Arhgap25	0
Arhgap24	0
Arhgap22	0
Arhgap21	0
Bloc1s6	0
Bloc1s5	0
Morc1	0
Bloc1s3	0
E030030I06Rik	0
Morc4	0
Mlxipl	0
Bmyc	0
Pappa2	0
BRDN0000738039	0
Pirt	0
Tmem150a	0
Fam46d	0
Emilin3	0
Gm6904	0
Btnl1	0
Rpp14	0
Ccnt2	0
Btnl4	0
Btnl6	0
Ypel5	0
Eif3i	0
Eif3k	0
Eif3m	0
D630045J12Rik	0
Ypel2	0
Eif3a	0
Eif3b	0
Eif3c	0
Npffr2	0
Eif3e	0
Eif3f	0
Eif3g	0
Atp8b3	0
Slc30a5	0
Atp8b1	0
3110002H16Rik	0
Adnp2	0
Cfl2	0
Fancd2	0
Atp8b4	0
Olfr1262	0
Olfr1260	0
Olfr1261	0
Wbscr16	0
1600002K03Rik	0
Olfr1264	0
Slitrk3	0
Myom3	0
Myom2	0
Myom1	0
Vmn1r119	0
Gm13889	0
Cyp21a1	0
Efna5	0
Pth	0
Efna3	0
Efna2	0
Efna1	0
Triap1	0
4930571K23Rik	0
Smok2b	0
Tomm20l	0
Icam4	0
Cyp2w1	0
Yeats4	0
Pitrm1	0
Tcf24	0
Tcf25	0
Vwf	0
Tcf20	0
Tcf21	0
Rrm2	0
Tcf23	0
Tex19.2	0
Shcbp1l	0
Smok2a	0
Jph4	0
Fam50a	0
Adam5	0
Klk13	0
Klk10	0
Ascc3	0
Ascc2	0
Ascc1	0
Asb9	0
Asb7	0
Asb5	0
Asb4	0
Asb3	0
Asb2	0
Asb18	0
Asb17	0
Asb14	0
Asb12	0
Asb10	0
Asb1	0
Asap3	0
Asah1	0
Arxes2	0
Arxes1	0
Artn	0
Art5	0
Art4	0
Art3	0
Art2b	0
Art1	0
Arsj	0
Arsb	0
Arsa	0
Arrdc5	0
Arrdc3	0
Arrdc2	0
Arrdc1	0
Arrb2	0
Arrb1	0
Arr3	0
Arpp19	0
Arpc5	0
Arpc3	0
Arpc2	0
Arpc1b	0
Arpc1a	0
Arntl2	0
Arntl	0
Arnt2	0
Arnt	0
Armt1	0
Armcx6	0
Armcx5	0
Armcx4	0
Armcx3	0
Armcx1	0
Armc9	0
Armc8	0
Armc7	0
Armc6	0
Armc4	0
Armc3	0
Armc2	0
Armc10	0
Arl8b	0
Arl6ip4	0
Arl5c	0
Arl5b	0
Arl4d	0
Arl4a	0
Arl3	0
Arl2	0
Arl16	0
Arl14epl	0
Arl14	0
Arl13b	0
Arl13a	0
Arl1	0
Arih1	0
Arid5b	0
Arid4a	0
Arid3c	0
Arid3b	0
Arid3a	0
Arid2	0
Arid1b	0
Arid1a	0

Anks1	0
Ankrd7	0
Ankrd66	0
Ankrd63	0
Ankrd60	0
Ankrd6	0
Ankrd55	0
Ankrd54	0
Ankrd53	0
Ankrd52	0
Ankrd50	0
Ankrd49	0
Ankrd45	0
Ankrd44	0
Ankrd42	0
Ankrd40	0
Ankrd39	0
Ankrd37	0
Ankrd36	0
Ankrd34c	0
Ankrd34b	0
Ankrd34a	0
Ankrd33b	0
Ankrd33	0
Ankrd32	0
Ankrd29	0
Ankrd28	0
Ankrd27	0
Ankrd26	0
Ankrd24	0
Ankrd23	0
Ankrd2	0
Ankrd17	0
Ankrd16	0
Ankrd13d	0
Ankrd13c	0
Ankrd13b	0
Ankrd13a	0
Ankrd12	0
Ankrd11	0
Ankrd1	0
Ankmy2	0
Ankle2	0
Ankle1	0
Ankk1	0
Ankib1	0
Ankfy1	0
Ankdd1b	0
Ankar	0
Ank3	0
Ank2	0
Ank1	0
Ank	0
Angptl7	0
Angptl6	0
Angptl4	0
Angptl3	0
Angptl2	0
Angptl1	0
Angpt4	0
Angpt2	0
Angel2	0
Angel1	0
Ang6	0
Ang5	0
Ang	0
Anapc7	0
Anapc5	0
Anapc4	0
Anapc2	0
Anapc13	0
Anapc11	0
Anapc10	0
Anapc1	0
Amz2	0
Amz1	0
Amy2a5	0
Amy2a4	0
Amy2a3	0
Amy2a2	0
Amtn	0
Amt	0
Arhgap17	0
Arhgap15	0
Arhgap12	0
Arhgap11a	0
Arhgap1	0
Arglu1	0
Arg2	0
Arg1	0
Arfrp1	0
Arfip2	0
Arfip1	0
Arfgef3	0
Arfgef2	0
Arfgef1	0
Arfgap3	0
Arfgap2	0
Arf6	0
Arf4	0
Arf1	0
Arel1	0
Areg	0
Arcn1	0
Arc	0
Arap1	0
Araf	0
Aqp9	0
Aqp4	0
Aqp3	0
Aqp2	0
Aqp12	0
Aqp11	0
Aptx	0
Aprt	0
Appl2	0
Appbp2	0
Apool	0
Apoo	0
Apon	0
Apol9a	0
Apol8	0
Apol7e	0
Apol7c	0
Apol7b	0
Apol7a	0
Apol6	0
Apol11b	0
Apol11a	0
Apol10a	0
Apoh	0
Apoc1	0
Apobr	0
Apobec3	0
Apobec2	0
Apoa5	0
Apmap	0
Aplp2	0
Aplp1	0
Aplnr	0
Aplf	0
Apitd1	0
Apip	0
Aph1c	0
Aph1b	0
Aph1a	0
Apex2	0
Apex1	0
Apc	0
Apbb1ip	0
Apbb1	0
Apba3	0
Apaf1	0
Ap5s1	0
Ap4s1	0
Ap4m1	0
Ap4e1	0
Ap4b1	0
Ap3s2	0
Ap3s1	0
Ap3m2	0
Ap3m1	0
Ap3d1	0
Ap3b1	0
Klk11	0
Parpbp	0
Bmp2k	0
Klk14	0
Klk15	0
Tnnt2	0
Krtap4-7	0
Rere	0
Fip1l1	0
Olfr901	0
Cmtm6	0
Olfr902	0
Ass1	0
Olfr904	0
Olfr907	0
Olfr906	0
Slco2b1	0
Phactr4	0
Pcdha3	0
Tusc1	0
Phactr3	0
Phactr2	0
4632428N05Rik	0
Larp4	0
Olfr743	0
Gripap1	0
Emg1	0
Slc51a	0
Sri	0
Olfr742	0
Aqp6	0
Aldh18a1	0
Olfr77	0
Olfr740	0
S100z	0
BRDN0000737740	0
Olfr747	0
Smim22	0
Lgals3bp	0
Rnf186	0
Elmod3	0
Elmod2	0
Sobp	0
BRDN0000738219	0
Ppp3ca	0
Olfr744	0
Kcnb1	0
Crebbp	0
Frmd4a	0
Zfp566	0
Tma16	0
Arfgap1	0
BRDN0000738379	0
BRDN0000738258	0
Ccpg1os	0
Prune2	0
Gm826	0
Lztfl1	0
Ngly1	0
Zfp872	0
Smn1	0
Oip5	0
E430018J23Rik	0
Gm773	0
Cyp27a1	0
Cutal	0
Hsf2	0
Rnf180	0
Spata31d1d	0
Rfxap	0
1810043H04Rik	0
Spib	0
Tmod1	0
Pmpcb	0
Setmar	0
Ccdc153	0
C330027C09Rik	0
Rnf183	0
Dhcr7	0
Ei24	0
Dym	0
Flrt2	0
Arhgef9	0
Arhgef7	0
Arhgef40	0
Arhgef39	0
Arhgef33	0
Arhgef3	0
Arhgef2	0
Arhgef19	0
Arhgef18	0
Arhgef17	0
Arhgef16	0
Arhgef12	0
Arhgef10l	0
Arhgef10	0
Arhgef1	0
Arhgdig	0
Arhgdia	0
Arhgap9	0
Arhgap8	0
Arhgap6	0
Arhgap5	0
Arhgap44	0
Arhgap42	0
Arhgap40	0
Arhgap4	0
Arhgap36	0
Arhgap35	0
Arhgap32	0
Arhgap31	0
Arhgap30	0
Arhgap29	0
Arhgap27	0
Arhgap26	0
Arhgap24	0
Arhgap22	0
Arhgap21	0
Arhgap19	0
Arhgap18	0
Arhgap17	0
Arhgap15	0
Arhgap11a	0
Arhgap10	0
Arglu1	0
Arg2	0
Arg1	0
Arfrp1	0
Arfip1	0
Arfgef3	0
Arfgef2	0
Arfgef1	0
Arfgap3	0
Arfgap2	0
Arfgap1	0
Arf6	0
Arf4	0
Arf1	0
Arel1	0
Areg	0
Arcn1	0
Arc	0
Arap1	0
Araf	0
Aqr	0
Aqp9	0
Aqp8	0
Aqp6	0
Aqp5	0
Aqp4	0
Aqp2	0
Aqp12	0
Aprt	0
Appl1	0
Appbp2	0
App	0
Apopt1	0
Apoo	0
Apon	0
Apom	0
Apold1	0
Apol9a	0
Apol8	0
Apol7c	0

Amph	0	Ap2m1	0	Tslp	0	Apol7b	0
Ampd3	0	Ap2a2	0	Gspt2	0	Apol7a	0
Ampd2	0	Ap1s3	0	Lamb3	0	Apol6	0
Ampd1	0	Ap1s2	0	Magix	0	Apol11b	0
Amotl2	0	Ap1s1	0	D430019H16Rik	0	Apol11a	0
Amotl1	0	Ap1m2	0	Ssb	0	Apol10b	0
Amn1	0	Ap1m1	0	Pcna	0	Apol10a	0
Amn	0	Ap1b1	0	Ppp1r12b	0	Apoh	0
Ammecr1l	0	Ap1ar	0	Taf6l	0	Apod	0
Amigo3	0	Aox4	0	Helt	0	Apoc1	0
Amica1	0	Aox3	0	Creld2	0	Apobec3	0
Amhr2	0	Aox1	0	Glyctk	0	Apobec2	0
Amfr	0	Aoc3	0	Adar	0	Apobec1	0
Amer3	0	Aoc2	0	Cat	0	Apob	0
Amer2	0	Aoah	0	Ly75	0	Apoa2	0
Amer1	0	Anxa9	0	Exosc8	0	Apoa1bp	0
Amelx	0	Anxa8	0	Adal	0	Apoa1	0
Amdhd2	0	Anxa6	0	Trmt112	0	Apmap	0
Amdhd1	0	Anxa5	0	Exosc2	0	Aplp2	0
Amd2	0	Anxa4	0	Exosc3	0	Aplp1	0
Amd1	0	Anxa10	0	Cfap52	0	Apln	0
Amacr	0	Antxr2	0	Sigmar1	0	Apitd1	0
Alyref2	0	Antxr1	0	Exosc4	0	Apip	0
Alyref	0	Anpep	0	Cad	0	Aph1c	0
Alx4	0	Anp32e	0	Sema3a	0	Aph1b	0
Alx3	0	Anp32b	0	Sema3c	0	Aph1a	0
Alx1	0	Anp32a	0	Sema3b	0	Apex2	0
Als2cr12	0	Ano8	0	Sema3e	0	Apex1	0
Als2cr11	0	Ano7	0	Zfp560	0	Apcdd1	0
Als2	0	Ano6	0	Sema3g	0	Apba2	0
Alpl	0	Ano5	0	Sema3f	0	Apaf1	0
Alpk3	0	Ano3	0	Fam71e1	0	Ap5z1	0
Alpk2	0	Ano10	0	Mzb1	0	Ap5s1	0
Alpk1	0	Ano1	0	Prn	0	Ap4m1	0
Alpi	0	Anln	0	Ifi203	0	Ap4e1	0
Aloxe3	0	Ankzf1	0	Ano10	0	Ap4b1	0
Alox8	0	Ankub1	0	BRDN0000737950	0	Ap3s2	0
Alox5ap	0	Anks6	0	BRDN0000737953	0	Ap3m2	0
Alox5	0	Anks1	0	Fam216a	0	Ap3m1	0
Alox15	0	Ankrd9	0	Arpc1b	0	Ap3b1	0
Alox12e	0	Ankrd7	0	BRDN0000737954	0	Ap2s1	0
Alox12b	0	Ankrd66	0	BRDN0000737957	0	Ap2m1	0
Alox12	0	Ankrd63	0	BRDN0000737956	0	Ap2a2	0
Allc	0	Ankrd60	0	Rnf220	0	Ap2a1	0
Alkbh8	0	Ankrd53	0	1700016K19Rik	0	Ap1s2	0
Alkbh7	0	Ankrd52	0	Rnf222	0	Ap1s1	0
Alkbh6	0	Ankrd49	0	Lpin1	0	Ap1m2	0
Alkbh5	0	Ankrd46	0	Prx	0	Ap1m1	0
Alkbh4	0	Ankrd45	0	Tcaim	0	Ap1g2	0
Alkbh2	0	Ankrd44	0	Kncn	0	Ap1b1	0
Alkbh1	0	Ankrd42	0	Crygs	0	Aox4	0
Alk	0	Ankrd40	0	Klhl1	0	Aox3	0
Alg9	0	Ankrd37	0	Ralbp1	0	Aox1	0
Alg8	0	Ankrd34c	0	Rsrc1	0	Aoc2	0
Alg6	0	Ankrd34a	0	Lrp1	0	Aoah	0
Alg5	0	Ankrd33b	0	Syap1	0	Anxa8	0
Alg3	0	Ankrd33	0	Ccr1l1	0	Anxa5	0
Alg2	0	Ankrd32	0	Pias2	0	Anxa3	0
Alg14	0	Ankrd29	0	Tsga13	0	Anxa2	0
Alg13	0	Ankrd28	0	Tsga10	0	Anxa10	0
Alg12	0	Ankrd24	0	Pias1	0	Antxrl	0
Alg11	0	Ankrd23	0	Nexn	0	Antxr1	0
Alg1	0	Ankrd22	0	Pias4	0	Anp32e	0
Aldoc	0	Ankrd2	0	Ptbp2	0	Anp32b	0
Aldob	0	Ankrd17	0	Nipal2	0	Anp32a	0
Aldoart2	0	Ankrd16	0	Csde1	0	Ano8	0
Aldoa	0	Ankrd13b	0	Gja10	0	Ano6	0
Aldh8a1	0	Ankrd13a	0	BRDN0000737896	0	Ano5	0
Aldh6a1	0	Ankrd12	0	Tmem87b	0	Ano4	0
Aldh4a1	0	Ankrd10	0	Tmem87a	0	Ano3	0
Aldh3b1	0	Ankrd1	0	Jmjd7	0	Ano10	0
Aldh3a1	0	Ankra2	0	Hipk3	0	Ano1	0
Aldh2	0	Ankmy1	0	Jmjd4	0	Ankub1	0
Aldh1l2	0	Ankle2	0	Olfr597	0	Anks6	0
Aldh1l1	0	Ankle1	0	Pcnp	0	Anks4b	0
Aldh1b1	0	Ankk1	0	Fbxo41	0	Anks1b	0
Aldh1a3	0	Ankib1	0	Hipk1	0	Anks1	0
Aldh18a1	0	Ankfy1	0	Itgb3bp	0	Ankrd7	0
Aldh16a1	0	Ankef1	0	Vmn1r91	0	Ankrd61	0
Alb	0	Ankar	0	Tk2	0	Ankrd54	0
Alas1	0	Ank3	0	Jmjd1c	0	Ankrd53	0
Alad	0	Ank2	0	Muc5b	0	Ankrd50	0

Aktip	0	Ank1	0	Mex3d	0	Ankrd49	0
Akt3	0	Ank	0	Mex3b	0	Ankrd46	0
Akt2	0	Angptl7	0	Jmjd8	0	Ankrd45	0
Akt1s1	0	Angptl6	0	Cdkal1	0	Ankrd42	0
Akt1	0	Angptl4	0	Utp23	0	Ankrd40	0
Akr1e1	0	Angptl3	0	Art1	0	Ankrd39	0
Akr1d1	0	Angptl2	0	Chchd2	0	Ankrd36	0
Akr1c6	0	Angpt4	0	Art3	0	Ankrd34b	0
Akr1c21	0	Angpt1	0	Art5	0	Ankrd34a	0
Akr1c19	0	Angel1	0	Birc7	0	Ankrd33b	0
Akr1c18	0	Ang6	0	Ghr	0	Ankrd33	0
Akr1c14	0	Ang4	0	Ppp4c	0	Ankrd32	0
Akr1c13	0	Ang2	0	Vmn1r95	0	Ankrd29	0
Akr1b8	0	Anapc5	0	Loh12cr1	0	Ankrd28	0
Akr1b3	0	Anapc4	0	Clec9a	0	Ankrd27	0
Akr1a1	0	Anapc2	0	1700003F12Rik	0	Ankrd24	0
Akp3	0	Anapc16	0	Rnf113a2	0	Ankrd22	0
Akirin2	0	Anapc13	0	Zfp174	0	Ankrd2	0
Akap8l	0	Anapc11	0	Pcnx	0	Ankrd17	0
Akap6	0	Anapc10	0	Vmn1r1	0	Ankrd16	0
Akap5	0	Anapc1	0	Oxgr1	0	Ankrd13c	0
Akap4	0	Amz1	0	Vmn1r3	0	Ankrd12	0
Akap3	0	Amy2a5	0	Vmn1r4	0	Ankrd11	0
Akap17b	0	Amy2a4	0	C2	0	Ankrd10	0
Akap14	0	Amy2a2	0	Vmn1r6	0	Ankrd1	0
Akap13	0	Amy1	0	Vmn1r7	0	Ankra2	0
Akap12	0	Amtn	0	Susd6	0	Ankmy2	0
Akap10	0	Amph	0	Commd8	0	Ankmy1	0
Akap1	0	Ampd2	0	Commd9	0	Ankle2	0
Ak5	0	Ampd1	0	Cd40lg	0	Ankhd1	0
Ak4	0	Amotl1	0	Ppp1cc	0	Ankfy1	0
AK010878	0	Amn	0	Pkn3	0	Ankar	0
Ajuba	0	Ammecr1l	0	Ppp1ca	0	Ank1	0
Aire	0	Amigo2	0	Commd3	0	Ank	0
Aip	0	Amigo1	0	Commd1	0	Angptl6	0
Aimp2	0	Amica1	0	Commd6	0	Angptl3	0
Aimp1	0	Amhr2	0	Commd7	0	Angptl2	0
Aim1l	0	Amfr	0	Commd4	0	Angpt4	0
Aim1	0	Amer1	0	Commd5	0	Angpt2	0
Aifm3	0	Amdhd2	0	BRDN0000737609	0	Angpt1	0
Aifm1	0	Amdhd1	0	Cnbp	0	Angel1	0
Aif1l	0	Amd1	0	Tkt	0	Ang6	0
Aif1	0	Amacr	0	Susd5	0	Ang4	0
AI987944	0	Alyref2	0	Olfr358	0	Ang2	0
AI846148	0	Alyref	0	Mptx1	0	Ang	0
AI661453	0	Alx3	0	Mptx2	0	Anapc7	0
AI607873	0	Als2cr12	0	Epha6	0	Anapc5	0
AI597479	0	Als2cr11	0	Lilra6	0	Anapc4	0
AI464131	0	Als2cl	0	Olfr355	0	Anapc2	0
AI429214	0	Alppl2	0	Olfr356	0	Anapc13	0
AI317395	0	Alpk1	0	Lilra5	0	Anapc11	0
AI314180	0	Alpi	0	BRDN0000738118	0	Anapc10	0
AI182371	0	Aloxe3	0	BRDN0000737604	0	Amz1	0
Ahsg	0	Alox8	0	Olfr352	0	Amy2a5	0
Ahsa2	0	Alox5ap	0	Prop1	0	Amy2a4	0
Ahr	0	Alox5	0	Olfr1095	0	Amy2a2	0
Ahnak	0	Alox15	0	Olfr1094	0	Amtn	0
Ahi1	0	Alox12e	0	Olfr1097	0	Amt	0
Ahdc1	0	Alox12	0	Epha4	0	Amph	0
Ahcyl2	0	Alkbh8	0	Hdhd1a	0	Ampd3	0
Ahcyl1	0	Alkbh6	0	Olfr1090	0	Ampd1	0
Ahctf1	0	Alkbh5	0	Haus6	0	Amn	0
Agxt	0	Alkbh3	0	Haus7	0	Ammecr1l	0
Agtr2	0	Alkbh1	0	Haus4	0	Ammecr1	0
Agtr1b	0	Alg9	0	Haus5	0	Amigo3	0
Agtpbp1	0	Alg8	0	Haus2	0	Amigo2	0
Agt	0	Alg5	0	Apool	0	Amh	0
Agrp	0	Alg3	0	Haus1	0	Amfr	0
Agrn	0	Alg14	0	BRDN0000738373	0	Amer1	0
Agr3	0	Alg13	0	Lpin2	0	Amd2	0
Agr2	0	Alg11	0	Insm2	0	Amd1	0
Agpat5	0	Alg10b	0	Myl6b	0	Amacr	0
Agpat2	0	Alg1	0	Iqsec1	0	Alyref2	0
Agpat1	0	Aldoa	0	Cox7a2	0	Alyref	0
Ago3	0	Aldh6a1	0	Olfr686	0	Alx3	0
Ago2	0	Aldh5a1	0	Spon2	0	Als2cr11	0
Ago1	0	Aldh4a1	0	Olfr685	0	Alppl2	0
Agmat	0	Aldh3b2	0	Bcl2a1a	0	Alpk2	0
Agl	0	Aldh3a1	0	Ubap2l	0	Alpk1	0
Agk	0	Aldh1l2	0	Bcl2a1c	0	Alpi	0
Aggf1	0	Aldh1l1	0	4921504E06Rik	0	Aloxe3	0
Agbl4	0	Aldh1b1	0	Rgag1	0	Alox5	0

Agbl3	0
Agbl2	0
Agap3	0
Agap2	0
Afp	0
Afmid	0
Afg3l1	0
Aff4	0
Aff3	0
Aff2	0
Aff1	0
Afap1l1	0
Afap1	0
AF366264	0
AF251705	0
AF067063	0
Aes	0
Aen	0
Aebp2	0
Adss	0
Adsl	0
Adrm1	0
Adrbk2	0
Adrbk1	0
Adrb3	0
Adrb1	0
Adra2c	0
Adra2b	0
Adra2a	0
Adra1b	0
Adra1a	0
Adprm	0
Adprhl2	0
Adprhl1	0
Adprh	0
Adpgk	0
Adora3	0
Adora2b	0
Adora2a	0
Adora1	0
Ado	0
Adnp2	0
Adnp	0
Adm2	0
Adm	0
Adk	0
Adipor2	0
Adipor1	0
Adipoq	0
Adi1	0
Adhfe1	0
Adh7	0
Adh6a	0
Adh4	0
Adh1	0
Adgrl4	0
Adgrl2	0
Adgrl1	0
Adgrg7	0
Adgrg6	0
Adgrg5	0
Adgrg3	0
Adgrg2	0
Adgrg1	0
Adgrf4	0
Adgrf3	0
Adgrf2	0
Adgre5	0
Adgre1	0
Adgrd1	0
Adgrb3	0
Adgrb2	0
Adgra3	0
Adgra1	0
Adgb	0
Add3	0
Add2	0
Add1	0
Adcyap1	0
Adcy9	0
Adcy8	0
Adcy7	0
Aldh1a7	0
Aldh1a3	0
Aldh1a1	0
Aldh18a1	0
Aldh16a1	0
Alcam	0
Alas2	0
Alas1	0
Alad	0
Akt3	0
Akt2	0
Akr7a5	0
Akr1e1	0
Akr1c6	0
Akr1c21	0
Akr1c18	0
Akr1c12	0
Akr1b7	0
Akr1b3	0
Akr1b10	0
Akp3	0
Akirin2	0
Akirin1	0
Akap9	0
Akap8l	0
Akap7	0
Akap6	0
Akap4	0
Akap3	0
Akap2	0
Akap14	0
Akap12	0
Akap1	0
Ak3	0
Ak1	0
Ajuba	0
Ajap1	0
Aire	0
Aipl1	0
Aip	0
Aimp2	0
Aimp1	0
Aim2	0
Aim1l	0
Aim1	0
Aifm2	0
Aif1	0
Aida	0
Aicda	0
AI987944	0
AI846148	0
AI837181	0
AI661453	0
AI607873	0
AI597479	0
AI593442	0
AI467606	0
AI464131	0
AI413582	0
AI317395	0
AI314180	0
AI182371	0
Ahr	0
Ahnak	0
Ahi1	0
Ahdc1	0
Ahcyl2	0
Ahcyl1	0
Ahcy	0
Ahctf1	0
Agtrap	0
Agtr2	0
Agtr1b	0
Agtr1a	0
Agt	0
Agrp	0
Agrn	0
Agr3	0
Agps	0
Agpat9	0
Agpat6	0
Agpat4	0
Nhsl1	0
Nhsl2	0
Ddah1	0
Olfr178	0
Scgb1b29	0
Mettl18	0
Emc8	0
1810046K07Rik	0
Elmo2	0
Ndel1	0
Emc3	0
Emc2	0
2210016L21Rik	0
6330416G13Rik	0
Mettl16	0
Plxnc1	0
V1ra8	0
Dpagt1	0
Pmaip1	0
Capza2	0
Olfr78	0
Unkl	0
Chmp4c	0
Saa4	0
Saa3	0
Saa2	0
Saa1	0
Kl	0
BRDN0000738026	0
BRDN0000738375	0
Agps	0
Mxd3	0
Chrna10	0
Gprc6a	0
Ky	0
Dedd2	0
Col12a1	0
Ranbp6	0
Emcn	0
Inppl1	0
Ranbp1	0
Lnp	0
Amt	0
BRDN0000738376	0
Serinc5	0
Zmynd15	0
Ranbp3	0
Zmynd12	0
Tie1	0
BRDN0000738239	0
Fkrp	0
Zfp820	0
Zfp821	0
Sestd1	0
Olfr995	0
Ccdc109b	0
9130019O22Rik	0
1700031F05Rik	0
C1ql4	0
A530099J19Rik	0
BRDN0000738377	0
Afg3l2	0
Atg12	0
Atg13	0
Atg14	0
Nfil3	0
Serinc2	0
Dapk3	0
Olfr1404	0
Gm20867	0
Rabggtb	0
Gm20865	0
Ugdh	0
Olfr1402	0
Pygo1	0
Peak1	0
Nkrf	0
Uba52	0
Rabggta	0
Rai2	0
Ccin	0
Dok6	0
Alox15	0
Alox12e	0
Alox12b	0
Alox12	0
Allc	0
Alkbh8	0
Alkbh7	0
Alkbh6	0
Alkbh5	0
Alkbh3	0
Alkbh2	0
Alkbh1	0
Alg8	0
Alg6	0
Alg5	0
Alg3	0
Alg2	0
Alg14	0
Alg13	0
Alg12	0
Alg11	0
Alg10b	0
Alg1	0
Aldoc	0
Aldob	0
Aldoa	0
Aldh8a1	0
Aldh6a1	0
Aldh5a1	0
Aldh4a1	0
Aldh3b1	0
Aldh3a1	0
Aldh2	0
Aldh1l2	0
Aldh1l1	0
Aldh1b1	0
Aldh1a2	0
Aldh1a1	0
Aldh18a1	0
Aldh16a1	0
Alas1	0
Akt3	0
Akt1s1	0
Akr7a5	0
Akr1e1	0
Akr1d1	0
Akr1cl	0
Akr1c6	0
Akr1c21	0
Akr1c18	0
Akr1c14	0
Akr1c13	0
Akr1c12	0
Akr1b7	0
Akr1b3	0
Akr1a1	0
Akp3	0
Akirin2	0
Akirin1	0
Akap9	0
Akap8l	0
Akap7	0
Akap6	0
Akap5	0
Akap4	0
Akap3	0
Akap17b	0
Akap13	0
Akap12	0
Akap10	0
Akap1	0
Ak8	0
Ak7	0
Ak5	0
Ak2	0
AK010878	0
Ajuba	0
Ajap1	0
Aire	0
Aip	0
Aim2	0
Aim1l	0

Adcy6	0	Agpat3	0	Gls2	0	Aim1	0
Adcy5	0	Agpat2	0	4933411G11Rik	0	Aifm3	0
Adcy2	0	Agpat1	0	Cd3d	0	Aifm2	0
Adcy1	0	Ago2	0	Crb1	0	Aifm1	0
Adck5	0	Ago1	0	Mycl	0	Aida	0
Adck1	0	Agmo	0	Tmem254c	0	AI846148	0
Adat3	0	Agl	0	Dok4	0	AI837181	0
Adat2	0	Agk	0	BRDN0000738230	0	AI597479	0
Adarb1	0	Aggf1	0	Ydjc	0	AI593442	0
Adar	0	Agfg1	0	Defb28	0	AI464131	0
Adap2	0	Agbl2	0	Pou4f3	0	AI413582	0
Adap1	0	Agap3	0	Eif4a1	0	AI314180	0
Adamtsl5	0	Agap2	0	Eif4a3	0	AI118078	0
Adamtsl4	0	Agap1	0	Eif4a2	0	Ahsg	0
Adamtsl1	0	Aga	0	Phykpl	0	Ahsa2	0
Adamts8	0	Afmid	0	Magea10	0	Ahr	0
Adamts6	0	Afm	0	Csnk1a1	0	Ahnak	0
Adamts5	0	Aff4	0	Por	0	Ahi1	0
Adamts4	0	Aff3	0	Jazf1	0	Ahdc1	0
Adamts20	0	Aff2	0	Gm2663	0	Ahcyl1	0
Adamts2	0	Aff1	0	Casc3	0	Ahcy	0
Adamts19	0	Afap1l2	0	Ppox	0	Agtrap	0
Adamts17	0	AF529169	0	Casc1	0	Agtr2	0
Adamts15	0	AF366264	0	Hnrnph1	0	Agtr1a	0
Adamts13	0	AF067063	0	Luzp1	0	Agtpbp1	0
Adamts12	0	Aes	0	Casc5	0	Agrp	0
Adamts10	0	Aen	0	Casc4	0	Agrn	0
Adamts1	0	Aebp2	0	Lasp1	0	Agr3	0
Adam9	0	Adtrp	0	Nhp2	0	Agr2	0
Adam7	0	Adssl1	0	Pawr	0	Agps	0
Adam6b	0	Adsl	0	Tcf3	0	Agpat9	0
Adam6a	0	Adrm1	0	Taok1	0	Agpat6	0
Adam4	0	Adrbk2	0	Crb3	0	Agpat5	0
Adam39	0	Adrb3	0	Taok3	0	Agpat3	0
Adam34	0	Adrb2	0	Dnmt3b	0	Agpat1	0
Adam33	0	Adrb1	0	Cyp4f18	0	Ago4	0
Adam32	0	Adra2b	0	Cyp24a1	0	Agmo	0
Adam30	0	Adra1d	0	4930563D23Rik	0	Agmat	0
Adam3	0	Adra1b	0	Axin2	0	Agl	0
Adam28	0	Adra1a	0	Axin1	0	Agk	0
Adam26b	0	Adprm	0	Cyp4f13	0	Aggf1	0
Adam25	0	Adprhl2	0	Cyp4f16	0	Agbl5	0
Adam24	0	Adprhl1	0	Cyp4f17	0	Agbl2	0
Adam22	0	Adpgk	0	Cyp4f14	0	Agap3	0
Adam20	0	Adora3	0	Cyp4f15	0	Agap2	0
Adam2	0	Adora2b	0	BRDN0000738234	0	Aga	0
Adam1b	0	Adora2a	0	Tnfrsf8	0	Aftph	0
Adam19	0	Ado	0	Tnfrsf9	0	Afp	0
Adam18	0	Adnp2	0	Zfp770	0	Afg3l2	0
Adam17	0	Adnp	0	Zfp771	0	Afg3l1	0
Adam15	0	Adm2	0	Zfp772	0	Aff4	0
Adam12	0	Adipor1	0	Zfp773	0	Aff2	0
Adam10	0	Adipoq	0	Zfp775	0	Afap1l2	0
Adal	0	Adig	0	Olfr1124	0	Afap1l1	0
Adad2	0	Adi1	0	Sept8	0	Afap1	0
Adad1	0	Adh7	0	Gm4846	0	AF067063	0
Ada	0	Adh6a	0	Rab21	0	Aes	0
Acyp2	0	Adh5	0	Cpt1c	0	Aen	0
Acyp1	0	Adh1	0	Rab23	0	Aebp2	0
Acy3	0	Adgrv1	0	Esr2	0	Adtrp	0
Acy1	0	Adgrl4	0	Mtmr12	0	Adsl	0
Acvrl1	0	Adgrl1	0	Krtap16-3	0	Adrbk1	0
Acvr2b	0	Adgrg6	0	Gm4841	0	Adrb3	0
Acvr2a	0	Adgrg5	0	Rab28	0	Adrb2	0
Acvr1c	0	Adgrg3	0	Mcidas	0	Adrb1	0
Acvr1b	0	Adgrg2	0	Ggct	0	Adra2c	0
Acvr1	0	Adgrf4	0	Cntnap1	0	Adra2b	0
Actrt3	0	Adgrf3	0	Mfn2	0	Adra1b	0
Actrt2	0	Adgrf1	0	Tcp1	0	Adra1a	0
Actr8	0	Adgre5	0	Cntnap2	0	Adprm	0
Actr6	0	Adgrd1	0	Cntnap4	0	Adprhl2	0
Actr5	0	Adgrb3	0	Dlst	0	Adprhl1	0
Actr3b	0	Adgrb2	0	Gm12695	0	Adpgk	0
Actr3	0	Adgra3	0	F13a1	0	Adora2a	0
Actr2	0	Adgra2	0	Steap3	0	Ado	0
Actr1b	0	Adgra1	0	Steap2	0	Adnp	0
Actr1a	0	Add2	0	Bzw1	0	Adm2	0
Actr10	0	Adcyap1r1	0	Steap4	0	Adk	0
Actn3	0	Adcy9	0	Bzw2	0	Adipor2	0
Actn2	0	Adcy8	0	Csf2rb	0	Adipor1	0
Actl7b	0	Adcy7	0	Gm13078	0	Adig	0
Actl7a	0	Adcy3	0	Csf2ra	0	Adhfe1	0

Actl6a	0	Adcy2	0	Sec23a	0	Adh7	0
Actl10	0	Adcy1	0	Sec23b	0	Adh6a	0
Actg2	0	Adck5	0	Peli3	0	Adh5	0
Actg1	0	Adck4	0	Zfp488	0	Adh1	0
Actb	0	Adck3	0	Tubal3	0	Adgrv1	0
Acta1	0	Adck2	0	Pamr1	0	Adgrl4	0
Acss3	0	Adck1	0	Crocc	0	Adgrl2	0
Acss2	0	Adat3	0	Pidd1	0	Adgrl1	0
Acss1	0	Adat2	0	LOC100048884	0	Adgrg7	0
Acsm5	0	Adarb2	0	Wbp1l	0	Adgrg5	0
Acsm4	0	Adarb1	0	Zfp420	0	Adgrg3	0
Acsm3	0	Adar	0	Samhd1	0	Adgrf5	0
Acsm2	0	Adap2	0	Gabpb2	0	Adgrf4	0
Acsm1	0	Adamtsl5	0	Sdcbp	0	Adgrf1	0
Acsl6	0	Adamtsl4	0	Trim29	0	Adgre5	0
Acsl5	0	Adamtsl3	0	Phc1	0	Adgre4	0
Acsl4	0	Adamtsl2	0	Olfr65	0	Adgre1	0
Acsl3	0	Adamts6	0	Upp1	0	Adgrd1	0
Acsl1	0	Adamts5	0	Mfng	0	Adgrb3	0
Acsf3	0	Adamts4	0	Hsph1	0	Adgra3	0
Acsf2	0	Adamts3	0	Mrpl55	0	Adgra1	0
Acsbg2	0	Adamts20	0	Duox1	0	Adgb	0
Acsbg1	0	Adamts2	0	Duox2	0	Add3	0
Acrbp	0	Adamts18	0	Egfr	0	Add1	0
Acr	0	Adamts17	0	Impdh1	0	Adcyap1r1	0
Acpt	0	Adamts15	0	Impdh2	0	Adcyap1	0
Acpp	0	Adamts14	0	Pelp1	0	Adcy9	0
Acp5	0	Adamts13	0	Rab2a	0	Adcy6	0
Acp1	0	Adamts10	0	Npc1	0	Adcy3	0
Acoxl	0	Adamdec1	0	Olfr66	0	Adcy1	0
Acox2	0	Adam9	0	Whamm	0	Adck4	0
Acox1	0	Adam7	0	Serpine1	0	Adck3	0
Acot9	0	Adam6b	0	Trim25	0	Adat3	0
Acot7	0	Adam6a	0	Olfr61	0	Adat2	0
Acot6	0	Adam5	0	Scgb1b24	0	Adat1	0
Acot5	0	Adam4	0	Jade3	0	Adarb2	0
Acot4	0	Adam39	0	AI467606	0	Adarb1	0
Acot2	0	Adam34	0	Jade1	0	Adap2	0
Acot12	0	Adam33	0	Mrpl51	0	Adap1	0
Acot11	0	Adam32	0	Sftpd	0	Adamtsl5	0
Acot10	0	Adam30	0	Elane	0	Adamtsl4	0
Acot1	0	Adam28	0	Trim27	0	Adamts8	0
Aco2	0	Adam25	0	Mrpl52	0	Adamts6	0
Aco1	0	Adam24	0	Ccl3	0	Adamts5	0
Acnat1	0	Adam23	0	Fam63b	0	Adamts4	0
Acmsd	0	Adam22	0	Mrpl53	0	Adamts3	0
Acly	0	Adam21	0	BRDN0000738120	0	Adamts20	0
Ackr4	0	Adam20	0	Olfr811	0	Adamts2	0
Ackr2	0	Adam2	0	Atoh1	0	Adamts19	0
Ackr1	0	Adam1b	0	G6pc	0	Adamts18	0
Acin1	0	Adam1a	0	Asphd1	0	Adamts17	0
Ache	0	Adam19	0	Fiz1	0	Adamts15	0
Acer3	0	Adam18	0	1810037I17Rik	0	Adamts14	0
Acer2	0	Adam17	0	Tanc2	0	Adamts13	0
Acer1	0	Adam11	0	4932414N04Rik	0	Adamts12	0
Ace3	0	Adam10	0	BRDN0000737637	0	Adamts1	0
Ace	0	Adal	0	Ccl7	0	Adam9	0
Acd	0	Adad1	0	Opalin	0	Adam7	0
Accsl	0	Ada	0	Apip	0	Adam5	0
Acbd7	0	Acyp2	0	Ccl4	0	Adam4	0
Acbd6	0	Acyp1	0	Rsl1	0	Adam33	0
Acbd5	0	Acy3	0	Olfr814	0	Adam32	0
Acat3	0	Acy1	0	H2afy2	0	Adam3	0
Acat2	0	Acvrl1	0	Fabp4	0	Adam29	0
Acat1	0	Acvr2b	0	Hs3st3a1	0	Adam28	0
Acap3	0	Acvr1b	0	Skint1	0	Adam26b	0
Acap2	0	Acvr1	0	Rab43	0	Adam25	0
Acap1	0	Actrt2	0	Skint3	0	Adam23	0
Acan	0	Actr8	0	Itpkb	0	Adam21	0
Acads	0	Actr6	0	Skint5	0	Adam20	0
Acadl	0	Actr5	0	Skint4	0	Adam2	0
Acad9	0	Actr3b	0	Skint7	0	Adam1b	0
Acad8	0	Actr2	0	9030624G23Rik	0	Adam1a	0
Acad11	0	Actr1b	0	Kcna6	0	Adam19	0
Acacb	0	Actr1a	0	Kcna7	0	Adam18	0
Acaca	0	Actr10	0	Kcna4	0	Adam15	0
Acaa2	0	Actn4	0	Kcna5	0	Adam12	0
Acaa1b	0	Actn3	0	Kcna2	0	Adam10	0
Abtb2	0	Actn2	0	Kcna1	0	Adal	0
Abtb1	0	Actn1	0	Irgc1	0	Adad2	0
Abt1	0	Actl9	0	Ccl9	0	Adad1	0
Abracl	0	Actl7b	0	Mpp3	0	Ada	0

Abra	0	Actl7a	0	BRDN0000738232	0	Acyp2	0
Abr	0	Actl6b	0	Pld5	0	Acyp1	0
Abo	0	Actl6a	0	Mrps9	0	Acy3	0
Ablim3	0	Actl11	0	H2-Q1	0	Acvrl1	0
Abl2	0	Actl10	0	Cfap44	0	Acvr2b	0
Abl1	0	Actg1	0	Fabp1	0	Acvr1b	0
Abi3bp	0	Acta2	0	Timeless	0	Acvr1	0
Abi3	0	Acta1	0	4930407I10Rik	0	Actrt3	0
Abhd8	0	Acss2	0	Gm2825	0	Actrt2	0
Abhd6	0	Acss1	0	Xntrpc	0	Actrt1	0
Abhd5	0	Acsm5	0	Meikin	0	Actr6	0
Abhd4	0	Acsm4	0	Ddx19a	0	Actr5	0
Abhd2	0	Acsl6	0	Ddx19b	0	Actr3	0
Abhd17c	0	Acsl5	0	H2-Q2	0	Actr2	0
Abhd17b	0	Acsl4	0	Itih3	0	Actr1b	0
Abhd17a	0	Acsl1	0	Pld1	0	Actr1a	0
Abhd15	0	Acsf3	0	Ephb3	0	Actr10	0
Abhd14b	0	Acsf2	0	Ephb1	0	Actn4	0
Abhd13	0	Acsbg1	0	Ephb6	0	Actn3	0
Abhd12b	0	Acrbp	0	Zak	0	Actn2	0
Abhd12	0	Acr	0	Ephb4	0	Actn1	0
Abhd11	0	Acpt	0	Aamdc	0	Actl9	0
Abcg8	0	Acp6	0	Lypd6b	0	Actl7b	0
Abcg5	0	Acp5	0	Mfsd11	0	Actl6b	0
Abcg4	0	Acp1	0	Mfsd10	0	Actl6a	0
Abcg3	0	Acox2	0	Cep76	0	Actl10	0
Abcg1	0	Acox1	0	Trp53inp1	0	Actg1	0
Abcf2	0	Acot9	0	Cep70	0	Actc1	0
Abcf1	0	Acot7	0	Cep72	0	Actb	0
Abce1	0	Acot6	0	Acot8	0	Acta2	0
Abcd4	0	Acot5	0	Fabp9	0	Acta1	0
Abcd2	0	Acot4	0	Cep78	0	Acss3	0
Abcc9	0	Acot2	0	Ppcdc	0	Acss2	0
Abcc8	0	Acot12	0	Vmp1	0	Acss1	0
Abcc6	0	Aco2	0	Acads	0	Acsm5	0
Abcc4	0	Aco1	0	Lrtm2	0	Acsm4	0
Abcc3	0	Acnat2	0	Sco1	0	Acsm3	0
Abcc2	0	Acnat1	0	2610528J11Rik	0	Acsm2	0
Abcc10	0	Acmsd	0	Igsf10	0	Acsm1	0
Abcc1	0	Acly	0	Igsf11	0	Acsl6	0
Abcb9	0	Ackr3	0	Traf3ip3	0	Acsl4	0
Abcb7	0	Ackr2	0	Mtcl1	0	Acsl1	0
Abcb6	0	Ackr1	0	Pctp	0	Acsf2	0
Abcb5	0	Ache	0	Col28a1	0	Acsbg2	0
Abcb4	0	Acer3	0	Adamts17	0	Acpt	0
Abcb1b	0	Acer2	0	Allc	0	Acpp	0
Abcb1a	0	Acer1	0	Acadl	0	Acp5	0
Abcb10	0	Ace3	0	Eef1e1	0	Acp1	0
Abca9	0	Ace2	0	BRDN0000737391	0	Acoxl	0
Abca8b	0	Ace	0	Trim11	0	Acox2	0
Abca6	0	Acd	0	Nkx1-1	0	Acox1	0
Abca5	0	Acbd7	0	Trim15	0	Acot9	0
Abca4	0	Acbd6	0	Trim14	0	Acot8	0
Abca2	0	Acbd5	0	Trim17	0	Acot6	0
Abca17	0	Acbd4	0	Trim16	0	Acot4	0
Abca16	0	Acbd3	0	Smad4	0	Acot13	0
Abca15	0	Acat3	0	Smad5	0	Acot12	0
Abca12	0	Acat1	0	1700074P13Rik	0	Acot11	0
Abca1	0	Acap1	0	Gm3259	0	Acot10	0
Abat	0	Acadvl	0	Atp6ap1	0	Acot1	0
AB124611	0	Acadsb	0	Nmrk2	0	Acnat2	0
Aatk	0	Acadm	0	Nmrk1	0	Acmsd	0
Aatf	0	Acadl	0	Smad3	0	Acly	0
Aasdhppt	0	Acad9	0	Tbc1d9b	0	Ackr4	0
Aasdh	0	Acad11	0	Nlrp14	0	Ache	0
Aarsd1	0	Acaca	0	Jup	0	Acer3	0
Aars2	0	Acaa1b	0	Pcdhga9	0	Acer2	0
Aars	0	Acaa1a	0	Casp14	0	Ace3	0
Aar2	0	Abtb1	0	Nlrp10	0	Ace	0
Aanat	0	Abt1	0	Nlrp12	0	Acd	0
Aamp	0	Abracl	0	Pcdhga2	0	Accsl	0
Aamdc	0	Abra	0	Pcdhga3	0	Accs	0
Aak1	0	Ablim3	0	Gdf15	0	Acbd7	0
Aagab	0	Ablim2	0	Svs5	0	Acbd6	0
Aaed1	0	Abl2	0	Pcdhga6	0	Acbd4	0
Aadat	0	Abl1	0	Olfr554	0	Acat3	0
Aadacl3	0	Abi3bp	0	Pcdhga4	0	Acat2	0
Aadacl2	0	Abhd6	0	Pcdhga5	0	Acat1	0
Aadac	0	Abhd5	0	Atp6v0b	0	Acap2	0
Aaas	0	Abhd4	0	Atp6v0c	0	Acap1	0
AA986860	0	Abhd3	0	Olfr543	0	Acan	0
AA792892	0	Abhd17b	0	BRDN0000737400	0	Acadsb	0

AA415398	0	Abhd16b	0	Olfr545	0	Acads	0
A930018P22Rik	0	Abhd16a	0	Olfr544	0	Acadm	0
A930009A15Rik	0	Abhd15	0	Olfr547	0	Acad8	0
A830080D01Rik	0	Abhd14b	0	Arvcf	0	Acacb	0
A830018L16Rik	0	Abhd14a	0	BRDN0000737414	0	Acaca	0
A730017C20Rik	0	Abhd12b	0	Acad9	0	Acaa1b	0
A630095N17Rik	0	Abhd12	0	BRDN0000737416	0	Acaa1a	0
A630095E13Rik	0	Abcg8	0	BRDN0000737417	0	Abtb2	0
A630076J17Rik	0	Abcg5	0	BRDN0000737410	0	Abt1	0
A630073D07Rik	0	Abcg4	0	Gm5941	0	Abo	0
A630033H20Rik	0	Abcg3	0	BRDN0000737412	0	Ablim2	0
A630023A22Rik	0	Abcg2	0	Cyp2a22	0	Abl1	0
A630001G21Rik	0	Abcg1	0	Frmpd4	0	Abi3bp	0
A530064D06Rik	0	Abcf2	0	Frmpd1	0	Abi3	0
A530032D15Rik	0	Abcf1	0	Cox8b	0	Abi1	0
A530016L24Rik	0	Abce1	0	Cox8a	0	Abhd5	0
A4gnt	0	Abcc6	0	BRDN0000738255	0	Abhd4	0
A4galt	0	Abcc5	0	BRDN0000737404	0	Abhd3	0
A430089I19Rik	0	Abcc4	0	Sytl5	0	Abhd17c	0
A430078G23Rik	0	Abcc3	0	Suclg2	0	Abhd17b	0
A430033K04Rik	0	Abcc10	0	Ctps	0	Abhd16b	0
A430005L14Rik	0	Abcc1	0	Nlrp1a	0	Abhd16a	0
A330050F15Rik	0	Abcb9	0	Rnps1	0	Abhd15	0
A2m	0	Abcb7	0	Sytl3	0	Abhd14a	0
A230065H16Rik	0	Abcb6	0	Sytl2	0	Abhd13	0
A230050P20Rik	0	Abcb5	0	Nr1i2	0	Abhd12b	0
A230046K03Rik	0	Abcb1b	0	Gm10334	0	Abhd12	0
A1cf	0	Abcb1a	0	Zfp85	0	Abhd11	0
A1bg	0	Abcb11	0	Elovl6	0	Abhd10	0
A130010J15Rik	0	Abca8b	0	Elovl7	0	Abcg5	0
a	0	Abca8a	0	Elovl4	0	Abcg4	0
9930111J21Rik1	0	Abca7	0	Gpld1	0	Abcg3	0
9930012K11Rik	0	Abca6	0	Elovl2	0	Abcg2	0
9830147E19Rik	0	Abca5	0	Elovl3	0	Abcg1	0
9830107B12Rik	0	Abca4	0	Elovl1	0	Abcf3	0
9530077C05Rik	0	Abca3	0	Ube2i	0	Abcf1	0
9530053A07Rik	0	Abca2	0	Ube2h	0	Abce1	0
9530003J23Rik	0	Abca17	0	Ube2k	0	Abcd4	0
9530002B09Rik	0	Abca14	0	Hopx	0	Abcd3	0
9430069I07Rik	0	Abca13	0	Ube2m	0	Abcd2	0
9430038I01Rik	0	Abca12	0	Ube2o	0	Abcc5	0
9430020K01Rik	0	Abat	0	Ube2n	0	Abcc4	0
9430016H08Rik	0	AB124611	0	Ube2a	0	Abcc2	0
9430007A20Rik	0	Aatk	0	Plaur	0	Abcc10	0
9330182O14Rik	0	Aass	0	Ube2c	0	Abcb9	0
9330182L06Rik	0	Aasdhppt	0	Cyp1a2	0	Abcb7	0
9330159F19Rik	0	Aasdh	0	Ube2f	0	Abcb6	0
9230112D13Rik	0	Aarsd1	0	Gstt4	0	Abcb5	0
9230110F15Rik	0	Aars2	0	Kbtbd13	0	Abcb1b	0
9230104L09Rik	0	Aars	0	Kbtbd12	0	Abcb11	0
9130409I23Rik	0	Aard	0	Kbtbd11	0	Abca9	0
9130401M01Rik	0	Aar2	0	Mpp4	0	Abca8b	0
9130204L05Rik	0	Aanat	0	Pstk	0	Abca8a	0
9130023H24Rik	0	Aamp	0	Gstt3	0	Abca6	0
9130019O22Rik	0	Aamdc	0	Ube2s	0	Abca5	0
9130011E15Rik	0	Aak1	0	Ube2u	0	Abca3	0
9130008F23Rik	0	Aaed1	0	Ube2t	0	Abca2	0
9030624J02Rik	0	Aadacl2	0	Ube2w	0	Abca17	0
9030624G23Rik	0	Aadac	0	Scamp3	0	Abca16	0
8430408G22Rik	0	Aaas	0	Lin7a	0	Abca13	0
8030462N17Rik	0	AA792892	0	Lin7c	0	Abca12	0
8030423J24Rik	0	AA415398	0	Acadvl	0	Abca1	0
8030411F24Rik	0	A930018P22Rik	0	Gm6760	0	Abat	0
7530416G11Rik	0	A930009A15Rik	0	Topbp1	0	AB124611	0
7420426K07Rik	0	A830080D01Rik	0	Trappc6a	0	Aatk	0
6820408C15Rik	0	A830010M20Rik	0	Tuba1c	0	Aasdhppt	0
6430573F11Rik	0	A730017C20Rik	0	Tuba1b	0	Aasdh	0
6430571L13Rik	0	A630095N17Rik	0	Tuba1a	0	Aarsd1	0
6430550D23Rik	0	A630095E13Rik	0	Scamp1	0	Aars	0
6430548M08Rik	0	A630073D07Rik	0	Trappc6b	0	Aard	0
6430531B16Rik	0	A630033H20Rik	0	BRDN0000738338	0	Aar2	0
6330416G13Rik	0	A630001G21Rik	0	Dcaf13	0	Aanat	0
6330409D20Rik	0	A530064D06Rik	0	Cc2d2a	0	Aamp	0
6030498E09Rik	0	A530032D15Rik	0	Dcaf11	0	Aak1	0
6030468B19Rik	0	A530016L24Rik	0	Srbd1	0	Aagab	0
6030458C11Rik	0	A4galt	0	Dcaf17	0	Aadacl3	0
6030419C18Rik	0	A430105I19Rik	0	Thnsl1	0	Aadacl2	0
5830473C10Rik	0	A430078G23Rik	0	Prrc1	0	Aaas	0
5830415F09Rik	0	A430033K04Rik	0	Dcdc2b	0	AA986860	0
5830411N06Rik	0	A430005L14Rik	0	5430402E10Rik	0	AA792892	0
5730559C18Rik	0	A3galt2	0	Hist1h4c	0	AA467197	0
5730507C01Rik	0	A330050F15Rik	0	Dcdc2c	0	AA415398	0

5730455P16Rik	0	A2m	0	Opn1sw	0	A930018P22Rik	0
5730409E04Rik	0	A230050P20Rik	0	Hist1h4b	0	A930009A15Rik	0
5530400C23Rik	0	A1cf	0	Trf	0	A830080D01Rik	0
5430435G22Rik	0	A1bg	0	Olfr150	0	A830018L16Rik	0
5430427O19Rik	0	A130010J15Rik	0	Olfr1280	0	A830010M20Rik	0
5430421N21Rik	0	a	0	Olfr1281	0	A730017C20Rik	0
5430419D17Rik	0	9930111J21Rik1	0	Fitm1	0	A630095N17Rik	0
5430401F13Rik	0	9930012K11Rik	0	Tcstv1	0	A530032D15Rik	0
5031414D18Rik	0	9830147E19Rik	0	Brd8	0	A4gnt	0
5031410I06Rik	0	9830107B12Rik	0	Brd9	0	A430105I19Rik	0
4933436I01Rik	0	9530068E07Rik	0	Olfr1286	0	A430089I19Rik	0
4933434E20Rik	0	9530003J23Rik	0	Olfr1287	0	A430078G23Rik	0
4933430I17Rik	0	9430069I07Rik	0	Olfr1288	0	A430033K04Rik	0
4933428G20Rik	0	9430038I01Rik	0	Olfr1289	0	A3galt2	0
4933427D14Rik	0	9430020K01Rik	0	Brd7	0	A2m	0
4933421I07Rik	0	9430016H08Rik	0	Kndc1	0	A230065H16Rik	0
4933416C03Rik	0	9430015G10Rik	0	BC048546	0	A230050P20Rik	0
4933415F23Rik	0	9330182O14Rik	0	Brd3	0	A230046K03Rik	0
4933412E24Rik	0	9330182L06Rik	0	Mansc4	0	A1bg	0
4933411K16Rik	0	9330159F19Rik	0	Olfr154	0	9930111J21Rik1	0
4933411G11Rik	0	9230112D13Rik	0	Heatr3	0	9830147E19Rik	0
4933409G03Rik	0	9230110F15Rik	0	Pprc1	0	9530077C05Rik	0
4933406M09Rik	0	9130409I23Rik	0	2610528A11Rik	0	9530068E07Rik	0
4933406J08Rik	0	9130401M01Rik	0	BC029214	0	9530053A07Rik	0
4933405O20Rik	0	9130204L05Rik	0	Calml3	0	9530003J23Rik	0
4933405L10Rik	0	9130011E15Rik	0	Iqsec2	0	9530002B09Rik	0
4933403O08Rik	0	9030624J02Rik	0	Mcmbp	0	9430069I07Rik	0
4933402N22Rik	0	9030624G23Rik	0	Rnf166	0	9430020K01Rik	0
4933402N03Rik	0	8430408G22Rik	0	Kif20a	0	9330182O14Rik	0
4933402J07Rik	0	8030423J24Rik	0	Cyp2u1	0	9330182L06Rik	0
4933402E13Rik	0	8030411F24Rik	0	Tm6sf1	0	9230112D13Rik	0
4932443I19Rik	0	7420461P10Rik	0	Olfr961	0	9230110F15Rik	0
4932438H23Rik	0	7420426K07Rik	0	Olfr159	0	9230110C19Rik	0
4932438A13Rik	0	6430573F11Rik	0	Olfr967	0	9230104L09Rik	0
4932429P05Rik	0	6430548M08Rik	0	Gm14548	0	9130409I23Rik	0
4932418E24Rik	0	6430531B16Rik	0	Olfr965	0	9130401M01Rik	0
4932414N04Rik	0	6330408A02Rik	0	2310061I04Rik	0	9130204L05Rik	0
4932411E22Rik	0	6330403A02Rik	0	Olfr969	0	9130011E15Rik	0
4931440F15Rik	0	6030468B19Rik	0	Olfr968	0	9130008F23Rik	0
4931431F19Rik	0	5830473C10Rik	0	Ttc21b	0	9030624J02Rik	0
4931429L15Rik	0	5830415F09Rik	0	Ttc21a	0	9030624G23Rik	0
4931429I11Rik	0	5730559C18Rik	0	Polr2m	0	8430408G22Rik	0
4931423N10Rik	0	5730507C01Rik	0	Gm6377	0	8030462N17Rik	0
4931417E11Rik	0	5430421N21Rik	0	Hspe1	0	8030423J24Rik	0
4931414P19Rik	0	5430419D17Rik	0	Ccr4	0	7420461P10Rik	0
4931406P16Rik	0	5430402E10Rik	0	Junb	0	6820408C15Rik	0
4930596D02Rik	0	5430401F13Rik	0	Phf20	0	6430573F11Rik	0
4930590J08Rik	0	5031414D18Rik	0	Phf23	0	6430571L13Rik	0
4930579F01Rik	0	5031410I06Rik	0	Slxl1	0	6330416G13Rik	0
4930578I06Rik	0	4933436I01Rik	0	Ccl11	0	6330409D20Rik	0
4930571K23Rik	0	4933434E20Rik	0	Phf11d	0	6030498E09Rik	0
4930568D16Rik	0	4933433C11Rik	0	Phf11b	0	6030468B19Rik	0
4930567H17Rik	0	4933430I17Rik	0	Phf11c	0	6030458C11Rik	0
4930564D02Rik	0	4933428G20Rik	0	Ccl17	0	6030419C18Rik	0
4930564B18Rik	0	4933427G17Rik	0	Fas	0	5830473C10Rik	0
4930563D23Rik	0	4933427D06Rik	0	Fap	0	5830415F09Rik	0
4930562C15Rik	0	4933413G19Rik	0	Kdm2a	0	5730559C18Rik	0
4930557A04Rik	0	4933411G11Rik	0	Fau	0	5730508B09Rik	0
4930555G01Rik	0	4933409G03Rik	0	Pex1	0	5730507C01Rik	0
4930550L24Rik	0	4933408B17Rik	0	Dyrk3	0	5730455P16Rik	0
4930550C14Rik	0	4933406M09Rik	0	Siglec15	0	5730409E04Rik	0
4930549C01Rik	0	4933406J08Rik	0	Ubtfl1	0	5530400C23Rik	0
4930544G11Rik	0	4933405O20Rik	0	Dyrk4	0	5430435G22Rik	0
4930539E08Rik	0	4933402N22Rik	0	Col3a1	0	5430427O19Rik	0
4930538K18Rik	0	4933402N03Rik	0	Prss45	0	5430419D17Rik	0
4930524N10Rik	0	4933402J07Rik	0	Ccr10	0	5430401F13Rik	0
4930523C07Rik	0	4933402E13Rik	0	Ulk2	0	5031414D18Rik	0
4930522H14Rik	0	4933402D24Rik	0	Ulk3	0	4933433C11Rik	0
4930519G04Rik	0	4932443I19Rik	0	Npy1r	0	4933430I17Rik	0
4930513O06Rik	0	4932438A13Rik	0	Rasgef1c	0	4933421I07Rik	0
4930505A04Rik	0	4932418E24Rik	0	Mpzl3	0	4933417A18Rik	0
4930503L19Rik	0	4932414N04Rik	0	Ulk4	0	4933415F23Rik	0
4930503E14Rik	0	4932411N23Rik	0	Mpzl1	0	4933412E24Rik	0
4930503B20Rik	0	4932411E22Rik	0	Clip2	0	4933409G03Rik	0
4930502E18Rik	0	4931440F15Rik	0	Serpina3j	0	4933408B17Rik	0
4930486L24Rik	0	4931431F19Rik	0	Spc24	0	4933406M09Rik	0
4930480E11Rik	0	4931429L15Rik	0	Odf3b	0	4933405O20Rik	0
4930474N05Rik	0	4931429I11Rik	0	Ifnb1	0	4933405L10Rik	0
4930468A15Rik	0	4931428F04Rik	0	Zfp953	0	4933402J07Rik	0
4930467E23Rik	0	4931417E11Rik	0	6030419C18Rik	0	4933402E13Rik	0
4930451I11Rik	0	4931414P19Rik	0	Slc26a10	0	4933402D24Rik	0
4930449I24Rik	0	4931408C20Rik	0	Slc26a11	0	4932443I19Rik	0

4930447C04Rik	0	4931406P16Rik	0	AU021092	0	4932438H23Rik	0
4930447A16Rik	0	4931406C07Rik	0	Serpina3m	0	4932438A13Rik	0
4930444G20Rik	0	4931406B18Rik	0	Vmn1r39	0	4932414N04Rik	0
4930433I11Rik	0	4930590J08Rik	0	Maged1	0	4931440F15Rik	0
4930432K21Rik	0	4930579G24Rik	0	Maged2	0	4931423N10Rik	0
4930430A15Rik	0	4930579F01Rik	0	Thy1	0	4931417E11Rik	0
4930427A07Rik	0	4930578I06Rik	0	Vill	0	4931414P19Rik	0
4930415O20Rik	0	4930571K23Rik	0	Trp53rk	0	4931409K22Rik	0
4930415L06Rik	0	4930568D16Rik	0	Ccr7	0	4931408C20Rik	0
4930412D23Rik	0	4930567H17Rik	0	Shox2	0	4931406C07Rik	0
4930407I10Rik	0	4930564D02Rik	0	Efcab9	0	4930579G24Rik	0
4930404N11Rik	0	4930563D23Rik	0	Gpm6b	0	4930578I06Rik	0
4930402H24Rik	0	4930562C15Rik	0	Efcab5	0	4930571K23Rik	0
4930402F06Rik	0	4930549C01Rik	0	Jsrp1	0	4930568D16Rik	0
4922502D21Rik	0	4930539E08Rik	0	Efcab6	0	4930564D02Rik	0
4921539E11Rik	0	4930538K18Rik	0	Efcab1	0	4930564B18Rik	0
4921536K21Rik	0	4930524B15Rik	0	Efcab3	0	4930563D23Rik	0
4921524L21Rik	0	4930522H14Rik	0	Efcab2	0	4930555G01Rik	0
4921524J17Rik	0	4930505A04Rik	0	Mau2	0	4930550L24Rik	0
4921517D22Rik	0	4930504O13Rik	0	2300009A05Rik	0	4930550C14Rik	0
4921511H03Rik	0	4930503L19Rik	0	Mroh2a	0	4930549C01Rik	0
4921509C19Rik	0	4930503E14Rik	0	Mroh2b	0	4930544G11Rik	0
4921507P07Rik	0	4930503B20Rik	0	5730559C18Rik	0	4930539E08Rik	0
4921504E06Rik	0	4930502E18Rik	0	Gm12169	0	4930538K18Rik	0
4833427G06Rik	0	4930486L24Rik	0	Mtmr7	0	4930524N10Rik	0
4833423E24Rik	0	4930467E23Rik	0	Ngf	0	4930523C07Rik	0
4833420G17Rik	0	4930452B06Rik	0	Fam212a	0	4930522H14Rik	0
4430402I18Rik	0	4930451I11Rik	0	Cdk11b	0	4930519G04Rik	0
3830417A13Rik	0	4930449I24Rik	0	Cd8a	0	4930505A04Rik	0
3830403N18Rik	0	4930447C04Rik	0	Prss39	0	4930504O13Rik	0
3425401B19Rik	0	4930447A16Rik	0	Pga5	0	4930503E14Rik	0
3300002I08Rik	0	4930444G20Rik	0	Lhb	0	4930503B20Rik	0
3110082J24Rik	0	4930435E12Rik	0	Fam198a	0	4930480E11Rik	0
3110079O15Rik	0	4930433I11Rik	0	Foxp3	0	4930468A15Rik	0
3110062M04Rik	0	4930430A15Rik	0	Fam198b	0	4930453N24Rik	0
3110057O12Rik	0	4930415L06Rik	0	Foxp4	0	4930452B06Rik	0
3110052M02Rik	0	4930407I10Rik	0	Stat5b	0	4930451I11Rik	0
3110043O21Rik	0	4930404N11Rik	0	Hip1	0	4930449I24Rik	0
3110040N11Rik	0	4930402K13Rik	0	Bcl11b	0	4930447A16Rik	0
3110009E18Rik	0	4930402H24Rik	0	Zer1	0	4930435E12Rik	0
3110007F17Rik	0	4930402F06Rik	0	Rbm24	0	4930433I11Rik	0
3110002H16Rik	0	4921539E11Rik	0	Dus4l	0	4930432K21Rik	0
3110001I22Rik	0	4921536K21Rik	0	Pde12	0	4930430F08Rik	0
3010026O09Rik	0	4921524L21Rik	0	Ssr1	0	4930430A15Rik	0
2900026A02Rik	0	4921524J17Rik	0	Snap47	0	4930427A07Rik	0
2810474O19Rik	0	4921517D22Rik	0	BC080695	0	4930415O20Rik	0
2810459M11Rik	0	4921511H03Rik	0	Rbm22	0	4930415F15Rik	0
2810408M09Rik	0	4921507P07Rik	0	1110008L16Rik	0	4930407I10Rik	0
2810408A11Rik	0	4921501E09Rik	0	Cspp1	0	4930404N11Rik	0
2810021J22Rik	0	4833439L19Rik	0	Ascl4	0	4930402K13Rik	0
2810007J24Rik	0	4833427G06Rik	0	Rreb1	0	4930402H24Rik	0
2810006K23Rik	0	4833423E24Rik	0	Rab1	0	4930402F06Rik	0
2810004N23Rik	0	4833420G17Rik	0	Papln	0	4922502D21Rik	0
2700097O09Rik	0	4732456N10Rik	0	Rab7	0	4921539E11Rik	0
2700089E24Rik	0	4430402I18Rik	0	Nrn1	0	4921536K21Rik	0
2700081O15Rik	0	3632451O06Rik	0	Rab9	0	4921524L21Rik	0
2700062C07Rik	0	3425401B19Rik	0	Cd80	0	4921524J17Rik	0
2700060E02Rik	0	3300002I08Rik	0	Cd83	0	4921511H03Rik	0
2700049A03Rik	0	3110082I17Rik	0	Cd82	0	4921509C19Rik	0
2700029M09Rik	0	3110062M04Rik	0	Cd84	0	4833427G06Rik	0
2610528J11Rik	0	3110052M02Rik	0	Ccs	0	4833423E24Rik	0
2610528A11Rik	0	3110040N11Rik	0	Cd86	0	4833420G17Rik	0
2610524H06Rik	0	3110021N24Rik	0	Rprd1a	0	4732456N10Rik	0
2610507B11Rik	0	3110009E18Rik	0	Taf4a	0	4632428N05Rik	0
2610318N02Rik	0	3110002H16Rik	0	Taf4b	0	3830417A13Rik	0
2610305D13Rik	0	3010026O09Rik	0	Rprd1b	0	3830406C13Rik	0
2610301B20Rik	0	2900026A02Rik	0	Cck	0	3830403N18Rik	0
2610034B18Rik	0	2900011O08Rik	0	Wdr53	0	3425401B19Rik	0
2610028H24Rik	0	2810474O19Rik	0	Clip1	0	3300002I08Rik	0
2610020H08Rik	0	2810459M11Rik	0	Pfdn1	0	3110082I17Rik	0
2610008E11Rik	0	2810428I15Rik	0	Zc3h8	0	3110079O15Rik	0
2610002M06Rik	0	2810417H13Rik	0	Fabp12	0	3110062M04Rik	0
2610002J02Rik	0	2810408M09Rik	0	Pfdn2	0	3110057O12Rik	0
2510009E07Rik	0	2810408A11Rik	0	Pfdn5	0	3110052M02Rik	0
2510003E04Rik	0	2810007J24Rik	0	Pfdn4	0	3110040N11Rik	0
2510002D24Rik	0	2810004N23Rik	0	Pithd1	0	3110035E14Rik	0
2410131K14Rik	0	2700097O09Rik	0	Pfdn6	0	3110021N24Rik	0
2410127L17Rik	0	2700094K13Rik	0	B020004J07Rik	0	3110007F17Rik	0
2410089E03Rik	0	2700081O15Rik	0	BRDN0000737972	0	3110002H16Rik	0
2410016O06Rik	0	2700062C07Rik	0	Zc3h3	0	3110001I22Rik	0
2410015M20Rik	0	2700060E02Rik	0	BRDN0000737970	0	2810474O19Rik	0
2410004B18Rik	0	2700049A03Rik	0	BRDN0000737977	0	2810459M11Rik	0

2410002F23Rik	0
2310079G19Rik	0
2310061N02Rik	0
2310061I04Rik	0
2310057N15Rik	0
2310057M21Rik	0
2310039H08Rik	0
2310036O22Rik	0
2310035C23Rik	0
2310034C09Rik	0
2310030G06Rik	0
2310022B05Rik	0
2310022A10Rik	0
2310014L17Rik	0
2310011J03Rik	0
2310009B15Rik	0
2310007L24Rik	0
2310003H01Rik	0
2310002L09Rik	0
2300009A05Rik	0
2300005B03Rik	0
2300003K06Rik	0
2210408I21Rik	0
2210407C18Rik	0
2210404O09Rik	0
2210018M11Rik	0
2210016F16Rik	0
2210010C04Rik	0
2010315B03Rik	0
2010300C02Rik	0
2010109I03Rik	0
2010107G23Rik	0
2010012O05Rik	0
2010005H15Rik	0
2010003K11Rik	0
2010001E11Rik	0
1810065E05Rik	0
1810062G17Rik	0
1810055G02Rik	0
1810046K07Rik	0
1810043H04Rik	0
1810043G02Rik	0
1810041L15Rik	0
1810037I17Rik	0
1810030O07Rik	0
1810026J23Rik	0
1810024B03Rik	0
1810022K09Rik	0
1810013L24Rik	0
1810011O10Rik	0
1810011H11Rik	0
1810009J06Rik	0
1810009A15Rik	0
1700129C05Rik	0
1700125H20Rik	0
1700123K08Rik	0
1700123I01Rik	0
1700122O11Rik	0
1700112E06Rik	0
1700109H08Rik	0
1700102P08Rik	0
1700101E01Rik	0
1700093K21Rik	0
1700092M07Rik	0
1700084C01Rik	0
1700080O16Rik	0
1700080E11Rik	0
1700074P13Rik	0
1700067P10Rik	0
1700066M21Rik	0
1700066B19Rik	0
1700061G19Rik	0
1700057G04Rik	0
1700056E22Rik	0
1700055N04Rik	0
1700042B14Rik	0
1700040L02Rik	0
1700037H04Rik	0
1700037C18Rik	0
1700034O15Rik	0
1700034J05Rik	0
1700034E13Rik	0
2610528J11Rik	0
2610528A11Rik	0
2610524H06Rik	0
2610507B11Rik	0
2610318N02Rik	0
2610305D13Rik	0
2610008E11Rik	0
2610002J02Rik	0
2510039O18Rik	0
2510009E07Rik	0
2510003E04Rik	0
2510002D24Rik	0
2410137M14Rik	0
2410131K14Rik	0
2410089E03Rik	0
2410016O06Rik	0
2410015M20Rik	0
2410012M07Rik	0
2410004P03Rik	0
2410002F23Rik	0
2310061I04Rik	0
2310057M21Rik	0
2310057J18Rik	0
2310050C09Rik	0
2310047M10Rik	0
2310039H08Rik	0
2310036O22Rik	0
2310035C23Rik	0
2310034C09Rik	0
2310033P09Rik	0
2310030G06Rik	0
2310022A10Rik	0
2310014L17Rik	0
2310011J03Rik	0
2310007B03Rik	0
2310003H01Rik	0
2300005B03Rik	0
2300003K06Rik	0
2300002M23Rik	0
2210408I21Rik	0
2210404O09Rik	0
2210018M11Rik	0
2210016F16Rik	0
2200002J24Rik	0
2200002D01Rik	0
2010300C02Rik	0
2010111I01Rik	0
2010109I03Rik	0
2010109A12Rik	0
2010107E04Rik	0
2010106E10Rik	0
2010005H15Rik	0
2010003K11Rik	0
2010002M12Rik	0
2010001E11Rik	0
1810065E05Rik	0
1810062G17Rik	0
1810046K07Rik	0
1810043G02Rik	0
1810041L15Rik	0
1810037I17Rik	0
1810026J23Rik	0
1810024B03Rik	0
1810022K09Rik	0
1810013L24Rik	0
1810011H11Rik	0
1810009J06Rik	0
1810009A15Rik	0
1700125H20Rik	0
1700123O20Rik	0
1700123K08Rik	0
1700123I01Rik	0
1700122O11Rik	0
1700112E06Rik	0
1700109H08Rik	0
1700084C01Rik	0
1700080O16Rik	0
1700074P13Rik	0
1700067K01Rik	0
1700066M21Rik	0
1700066B19Rik	0
1700061G19Rik	0
BRDN0000737976	0
Dynlt3	0
Zc3h6	0
Pih1d3	0
Pih1d1	0
Fetub	0
Eps15l1	0
Acyp1	0
Gm8677	0
Pts	0
Gca	0
Kdsr	0
Rwdd3	0
Rwdd1	0
Wfdc6a	0
Wfdc6b	0
Gtse1	0
Epn1	0
Pcdhb19	0
Epn3	0
Epn2	0
AA467197	0
Echs1	0
Cpe	0
Gabpb1	0
Decr2	0
Elp2	0
1700022I11Rik	0
Slc8a3	0
Amh	0
Rwdd2a	0
Gm20823	0
Amn	0
3425401B19Rik	0
Pcdhb18	0
Cd300lg	0
Lats2	0
Lats1	0
Cd300ld	0
Ranbp9	0
Cd300lb	0
Adgre4	0
4930451I11Rik	0
Tagln2	0
Tagln3	0
Tbc1d10c	0
Gck	0
Tbc1d10a	0
Ranbp2	0
Vmn1r113	0
Wisp1	0
Epm2aip1	0
5730409E04Rik	0
Phax	0
Fcgr2b	0
Abracl	0
Ikbkb	0
4930502E18Rik	0
Bmp4	0
Ppp6c	0
Ikbkg	0
Ikbke	0
Zc4h2	0
Alpi	0
Aldh1a7	0
Aldh1a1	0
Olfr376	0
Galnt14	0
Olfr374	0
Tek	0
Olfr372	0
Olfr373	0
Olfr370	0
Olfr371	0
Fam118a	0
Bcl10	0
Txndc17	0
Txndc11	0
Zp3r	0
Olfr378	0
Txndc12	0
Fhl2	0
2810428I15Rik	0
2810408M09Rik	0
2810408A11Rik	0
2810403A07Rik	0
2810007J24Rik	0
2810004N23Rik	0
2700097O09Rik	0
2700094K13Rik	0
2700089E24Rik	0
2700081O15Rik	0
2700062C07Rik	0
2610318N02Rik	0
2610305D13Rik	0
2610034B18Rik	0
2610028H24Rik	0
2610020H08Rik	0
2610015P09Rik	0
2610008E11Rik	0
2610002M06Rik	0
2510009E07Rik	0
2410137M14Rik	0
2410127L17Rik	0
2410089E03Rik	0
2410016O06Rik	0
2410015M20Rik	0
2410012M07Rik	0
2410004P03Rik	0
2410002F23Rik	0
2310079G19Rik	0
2310061N02Rik	0
2310057M21Rik	0
2310047M10Rik	0
2310034C09Rik	0
2310033P09Rik	0
2310030G06Rik	0
2310022B05Rik	0
2310022A10Rik	0
2310014L17Rik	0
2310011J03Rik	0
2310009B15Rik	0
2310007L24Rik	0
2310007B03Rik	0
2310003H01Rik	0
2310002L09Rik	0
2300005B03Rik	0
2300003K06Rik	0
2210407C18Rik	0
2210404O09Rik	0
2210018M11Rik	0
2210016L21Rik	0
2210010C04Rik	0
2200002D01Rik	0
2010315B03Rik	0
2010300C02Rik	0
2010111I01Rik	0
2010109I03Rik	0
2010109A12Rik	0
2010107E04Rik	0
2010106E10Rik	0
2010012O05Rik	0
2010005H15Rik	0
2010001E11Rik	0
1810065E05Rik	0
1810043G02Rik	0
1810041L15Rik	0
1810026J23Rik	0
1810024B03Rik	0
1810022K09Rik	0
1810013L24Rik	0
1810011H11Rik	0
1810009J06Rik	0
1700129C05Rik	0
1700125H20Rik	0
1700123O20Rik	0
1700123I01Rik	0
1700122O11Rik	0
1700113H08Rik	0
1700106J16Rik	0
1700101E01Rik	0
1700088E04Rik	0
1700084C01Rik	0
1700080O16Rik	0

1700031F05Rik	0
1700030K09Rik	0
1700030J22Rik	0
1700030F18Rik	0
1700029P11Rik	0
1700029J07Rik	0
1700029I15Rik	0
1700029H14Rik	0
1700029F12Rik	0
1700028P14Rik	0
1700028K03Rik	0
1700026L06Rik	0
1700025G04Rik	0
1700025F22Rik	0
1700024P16Rik	0
1700024G13Rik	0
1700023F06Rik	0
1700023E05Rik	0
1700022I11Rik	0
1700021K19Rik	0
1700021F07Rik	0
1700021F05Rik	0
1700020N15Rik	0
1700020D05Rik	0
1700020A23Rik	0
1700019O17Rik	0
1700019N19Rik	0
1700019L03Rik	0
1700019G17Rik	0
1700019D03Rik	0
1700019B03Rik	0
1700018F24Rik	0
1700018B08Rik	0
1700017D01Rik	0
1700017B05Rik	0
1700016K19Rik	0
1700016H13Rik	0
1700016D06Rik	0
1700015G11Rik	0
1700015F17Rik	0
1700013F07Rik	0
1700013D24Rik	0
1700012P22Rik	0
1700012L04Rik	0
1700012B09Rik	0
1700012A03Rik	0
1700011I03Rik	0
1700011H14Rik	0
1700011E24Rik	0
1700011A15Rik	0
1700010I14Rik	0
1700010B08Rik	0
1700008P02Rik	0
1700008O03Rik	0
1700008I05Rik	0
1700008F21Rik	0
1700007K09Rik	0
1700006A11Rik	0
1700003F12Rik	0
1700001O22Rik	0
1700001L19Rik	0
1700001K19Rik	0
1700001J03Rik	0
1700001C19Rik	0
1700001C02Rik	0
1600015I10Rik	0
1600014K23Rik	0
1600014C23Rik	0
1600014C10Rik	0
1600002K03Rik	0
1600002H07Rik	0
1500009L16Rik	0
1190003K10Rik	0
1190002N15Rik	0
1110059G10Rik	0
1110059E24Rik	0
1110051M20Rik	0
1110038F14Rik	0
1110037F02Rik	0
1110032F04Rik	0
1110032A03Rik	0
1110025L11Rik	0
1700056E22Rik	0
1700047I17Rik2	0
1700042B14Rik	0
1700037H04Rik	0
1700037C18Rik	0
1700034J05Rik	0
1700034E13Rik	0
1700031F05Rik	0
1700030J22Rik	0
1700030F18Rik	0
1700029P11Rik	0
1700029J07Rik	0
1700029I15Rik	0
1700029H14Rik	0
1700029F12Rik	0
1700028P14Rik	0
1700028K03Rik	0
1700026L06Rik	0
1700025G04Rik	0
1700025F22Rik	0
1700024P04Rik	0
1700024G13Rik	0
1700023F06Rik	0
1700023E05Rik	0
1700022I11Rik	0
1700021F05Rik	0
1700020L24Rik	0
1700019L03Rik	0
1700019G17Rik	0
1700019B03Rik	0
1700019A02Rik	0
1700017D01Rik	0
1700017B05Rik	0
1700016H13Rik	0
1700016D06Rik	0
1700016C15Rik	0
1700015F17Rik	0
1700015E13Rik	0
1700013H16Rik	0
1700013D24Rik	0
1700012P22Rik	0
1700012L04Rik	0
1700012B09Rik	0
1700012B07Rik	0
1700011L22Rik	0
1700011I03Rik	0
1700011H14Rik	0
1700011E24Rik	0
1700011A15Rik	0
1700010I14Rik	0
1700009N14Rik	0
1700008P02Rik	0
1700008I05Rik	0
1700007K13Rik	0
1700007K09Rik	0
1700007G11Rik	0
1700007B14Rik	0
1700003F12Rik	0
1700003E16Rik	0
1700001P01Rik	0
1700001O22Rik	0
1700001K19Rik	0
1700001F09Rik	0
1700001C02Rik	0
1600015I10Rik	0
1600014C23Rik	0
1600002K03Rik	0
1600002H07Rik	0
1500012F01Rik	0
1500009L16Rik	0
1300017J02Rik	0
1200014J11Rik	0
1190007I07Rik	0
1190003K10Rik	0
1190002N15Rik	0
1110051M20Rik	0
1110038F14Rik	0
1110037F02Rik	0
1110034G24Rik	0
1110025L11Rik	0
1110012L19Rik	0
1110008P14Rik	0
BRDN0000737622	0
BRDN0000737621	0
BRDN0000737620	0
BRDN0000737627	0
BRDN0000737626	0
Fhl4	0
1700009N14Rik	0
Itih1	0
D19Bwg1357e	0
BRDN0000737629	0
BRDN0000737628	0
Itih5	0
Itih4	0
Pcdh11x	0
Cacnb3	0
Siah1b	0
Siah1a	0
Ccdc144b	0
Psmd14	0
Psmd10	0
Psmd13	0
Psmd12	0
Fam134b	0
Nxf7	0
Odf3l2	0
Gsdmc4	0
Odf3l1	0
Olfr152	0
Olfr153	0
Hist1h4a	0
Tmem14c	0
Tmem14a	0
Hist1h4d	0
Hist1h4k	0
Hist1h4j	0
Hist1h4i	0
Hist1h4h	0
Hist1h4n	0
Hist1h4m	0
Calml4	0
Rnd1	0
Rnd2	0
Rsl1d1	0
Vat1	0
Josd1	0
Sit1	0
Fbxo45	0
Cpxcr1	0
Vps9d1	0
Ctla4	0
Tekt1	0
Rorc	0
Tekt2	0
Tekt5	0
Tekt4	0
Csgalnact1	0
Rorb	0
Cntrob	0
Ermap	0
Fbxo46	0
Gm9758	0
Sept11	0
Oosp1	0
Slc8a1	0
Olfr6	0
Decr1	0
Slc8a2	0
4931409K22Rik	0
Mms19	0
Bmp7	0
Bmp6	0
Bmp5	0
Bmp3	0
Bmp2	0
Bmp1	0
Acot5	0
Spert	0
Rxrg	0
Gm5885	0
Rxra	0
Rxrb	0
Chst4	0
1700074P13Rik	0
1700067K01Rik	0
1700066M21Rik	0
1700061G19Rik	0
1700057G04Rik	0
1700056E22Rik	0
1700042G07Rik	0
1700040L02Rik	0
1700037H04Rik	0
1700034J05Rik	0
1700031F05Rik	0
1700030K09Rik	0
1700029P11Rik	0
1700029I15Rik	0
1700029H14Rik	0
1700028P14Rik	0
1700028K03Rik	0
1700026L06Rik	0
1700026D08Rik	0
1700024P16Rik	0
1700024P04Rik	0
1700024G13Rik	0
1700022I11Rik	0
1700021K19Rik	0
1700021F05Rik	0
1700020N15Rik	0
1700020L24Rik	0
1700020D05Rik	0
1700019G17Rik	0
1700019A02Rik	0
1700018C11Rik	0
1700018B08Rik	0
1700017D01Rik	0
1700017B05Rik	0
1700016H13Rik	0
1700016D06Rik	0
1700016C15Rik	0
1700015G11Rik	0
1700015F17Rik	0
1700013H16Rik	0
1700013F07Rik	0
1700013D24Rik	0
1700012L04Rik	0
1700012B07Rik	0
1700011L22Rik	0
1700011I03Rik	0
1700011H14Rik	0
1700011A15Rik	0
1700010I14Rik	0
1700009N14Rik	0
1700008O03Rik	0
1700008I05Rik	0
1700008F21Rik	0
1700007K13Rik	0
1700007G11Rik	0
1700006E09Rik	0
1700003E16Rik	0
1700001P01Rik	0
1700001L19Rik	0
1700001K19Rik	0
1700001J03Rik	0
1700001F09Rik	0
1700001C02Rik	0
1600015I10Rik	0
1600014K23Rik	0
1600014C23Rik	0
1600014C10Rik	0
1600002K03Rik	0
1600002H07Rik	0
1500015O10Rik	0
1500009L16Rik	0
1190007I07Rik	0
1190005I06Rik	0
1190002N15Rik	0
1110065P20Rik	0
1110059G10Rik	0
1110059E24Rik	0
1110038F14Rik	0
1110037F02Rik	0
1110034G24Rik	0
1110032F04Rik	0
1110032A03Rik	0

1110017D15Rik	0	1110008L16Rik	0	Chst9	0	1110017D15Rik	0
1110008P14Rik	0	1110008F13Rik	0	Chst8	0	1110012L19Rik	0
1110008L16Rik	0	1110007C09Rik	0	Tmem145	0	1110008P14Rik	0
1110008F13Rik	0	1110004F10Rik	0	Tmem144	0	1110008L16Rik	0
1110004E09Rik	0	1110001J03Rik	0	Ikbkap	0	1110008F13Rik	0
1110001J03Rik	0	0610040J01Rik	0	Tdgf1	0	1110007C09Rik	0
0610040J01Rik	0	0610030E20Rik	0	Tmem141	0	1110004F10Rik	0
0610030E20Rik	0	0610011F06Rik	0	Chst1	0	1110004E09Rik	0
0610011F06Rik	0	0610010K14Rik	0	Chst3	0	1110001J03Rik	0
0610010F05Rik	0	0610010F05Rik	0	Rgs17	0	0610040J01Rik	0
0610010B08Rik	0	0610010B08Rik	0	Chst5	0	0610011F06Rik	0
0610009O20Rik	0	0610009B22Rik	0	Mndal	0	0610010K14Rik	0
0610007P14Rik	0	0610007P14Rik	0	Chst7	0	0610010B08Rik	0
