# Supplementary material for: The murine MHC-E molecule Qa-1b is surface displayed in a peptide-free conformation in homeostasis
Source: Front Immunol. 2026 Mar 9;17:1743362. doi: 10.3389/fimmu.2026.1743362 (PMC13006831; doi:10.3389/fimmu.2026.1743362)
Supplement: Supplementary Table 2 — Resource Table. [file DataSheet2.pdf]

Supplementary Table 2, Key resources table

Reagent or resource	Source/ Supplier	Identifier
**Antibodies**		
Anti-human β2-microglobulin (clone 2M2)	Biolegend	Cat: 316302
Anti-mouse CD11b PE-Cyanine7 (clone M1/70)	Thermo Fisher	Cat: 25-0112-82
Anti-mouse CD11c APC-Cyanine7 (clone N418)	BioLegend	Cat: 117324
Anti-mouse CD16/CD32 Fc block (clone 2.4G2)	BD Biosciences	Cat: 553142
Anti-mouse CD19 BV510 (clone 1D3)	BD Biosciences	Cat: 562956
Anti-mouse CD19 FITC (clone 1D3)	Thermo Fisher	Cat: 11-0193-85
Anti-mouse CD19 PE (clone 1D3)	Thermo Fisher	Cat: 12-0193-82
Anti-mouse CD3 BV510 (clone 145-2C11)	Biolegend	Cat: 100353
Anti-mouse CD3e FITC (clone 145-2C11)	Thermo Fisher	Cat: 11-0031-85
Anti-mouse CD4 BV605 (clone RM4-5	BioLegend	Cat: 100547
Anti-mouse CD45 AF700 (clone 30-F11)	BioLegend	Cat: 103128
Anti-mouse CD45.2 APC-eFluor780 (clone 104)	Thermo Fisher	Cat: 47-0454-82
Anti-mouse CD62L BV421 (clone MEL-14)	BioLegend	Cat: 104436
Anti-mouse CD8a AF700 (clone 53-6.7)	Thermo Fisher	Cat: 56-0081-82
Anti-mouse F4/80 PE (clone BM8)	BioLegend	Cat: 123110
Anti-mouse H-2L^{d}/H-2D^{b} PE (clone 28-14-8)	BioLegend	Cat: 114507
Anti-mouse I-A/I-E BV421 (clone M5/114.15.2)	BD Biosciences	Cat: 562564
Anti-mouse Ly-6C BV605 (Clone HK1.4)	BioLegend	Cat: 128036
Anti-mouse Ly-6G BV785 (clone 1A8)	BioLegend	Cat: 127645

Anti-mouse NK1-1 BV510 (clone PK137)	Biolegend	Cat: 108737
Anti-mouse NK1-1 BV650 (clone PK136)	BD Biosciences	Cat: 564143
Anti-mouse Siglec-F BV711 (clone E50-2440)	BD Biosciences	Cat: 740764
Anti-mouse Siglec-H BV650 (clone 440c)	BD Biosciences	Cat: 747672
Anti-mouse/human CD44 BV786 (clone IM7)	BioLegend	Cat: 103059
Anti-mouse Qa-1 ^{b} (clone 6A8.6F10.1A6)	BD Biosciences	Cat: 559827
Anti-mouse Qa-1^{b} biotin (clone 6A8.6F10.1A6)	BD Biosciences	Cat: 559829
EXX-1 antibody	Produced in house	N/A
Mouse IgG2A EXX1	ATUM	N/A
Mouse IgG2A EXX1 AF647	ATUM	N/A
Streptavidin APC	Invitrogen	Cat: 17-4317-82
Streptavidin PE-CF594	BD Biosciences	Cat: 562318

**Chemicals, peptides and recombinant proteins**		
Blasticidin	Thermo Fisher	Cat: A1113903
Bovine Serum Albumin	Sigma-Aldrich	Cat: A3912
BsmBI	NEB	Cat: R0739L
GroEL peptide (GMQFDRGYL)	Genscript	N/A
Lipofectamine 3000	Thermo Fisher	Cat: L3000008
MG132	Sigma-Aldrich	Cat: C2211
OVA peptide (SIINFEKL)	Produced in house	N/A
Penicillin-Streptomycin-Glutamine (100x)	Gibco	Cat: 10378016
Polybrene	Merck Millipore	Cat: TR-1003-G
Polyethyleneimine	Polyscience Inc.	Cat: 23966-1

Puromycin	Thermo Fisher	Cat: J672368EQ
Q001 peptide (AQAERTPEL)	Genscript	N/A
Qdm peptide (AMAPRTLLL)	Genscript	N/A
Recombinant murine IFN-γ	BioLegend	Cat: 575306
Sodium azide	In house pharmacy	N/A
T4 DNA ligase	Thermo Fisher	Cat: EL0011
Tilorone dihydrochloride	Sigma-Aldrich	Cat: 220957
**Critical commercial kits**		
Bionectics label-free microarray plates	Millipore-Sigma	Cat:RSNT-RSI96100N6-5
BirA Biotin Ligase Kit	Avidity	N/A
Click-it sDIBO alkyne kit for antibody labeling (Alexa Fluor 647)	Thermo Fisher	Cat: C20029
Isolate II genomic DNA kit	GC Biotech	Cat: BIO-52067
Nucleospin Plasmid Transfection Grade Kit	Macherey-Nagel	Cat: 740.490.250
SiteClick antibody azido modification kit	Thermo Fisher	Cat: S20026
Zombie Aqua Fixable Viability Kit	BioLegend	Cat: 423102
**Oligonucleotides (sgRNAs, primers, plasmids etc) all sequences 5' → 3'**		
CDS Codon-optimized Qa-1^b heavy chain for cell lines (*H2-T23*)	Twist Bioscience	N/A
Plasmid LentiCRISPRv2 puro	Addgene	Cat: 98290
Plasmid LentiCRISPRv2 blast	Addgene	Cat:83480
Plasmid Pax2	Addgene	Cat: 12260
Plasmid pMD2.G	Dr. T. Schumacher (Addgene)	Cat: 12259

Plasmid pCDH-CMV-MCS-EF1-Puro	Dr. T. Schumacher (System Biosciences)	N/A
Plasmid pET-21a(+)	Millipore-Sigma	Cat: 69740
Plasmid pMDLg/PRRE	Addgene	Cat: 12251
Plasmid pRSV-Rev	Addgene	Cat: 12253
Plasmid pCMV-VSV-G	Addgene	Cat: 8454
Plasmid pFUSE-mouse (m)IgG2A-Fc2	Invivogen	Cat: pfuse-mg2afc2
Genome-wide Calabrase activation library (sublibrary A+B)	Dr. J. Doench	Addgene cat: 1000000111
Genome-wide mouse CRISPR Brie knockout library	Dr. D Root and Dr. J Doench	Addgene cat: 73632
MCS T2A oligo (TTAATTAAGGTACCACCGGTAGAATTCAGATATCACTCGAGAGGATCCGGCGAGGGCAGGGGAAGTCTACTAACATGCGGGGACGTGGAGGAAAATCCCGGCCCAAACAAGATA)	Integrated DNA Technologies	N/A
NGS-Lib-Fwd-1 (AATGATACGGCGACCACCGAGATCTACACTCTTTCCCTACACGACGCTCTTCCGATCTTAAGTAGAGGCTTTATATATCTTGTGGAAAGGACGAAACACC)	Integrated DNA Technologies	N/A
NGS-Lib-Fwd-2 (AATGATACGGCGACCACCGAGATCTACACTCTTTCCCTACACGACGCTCTTCCGATCTATCATGCTTAGCTTTATATATCTTGTGGAAAGGACGAAACACC)	Integrated DNA Technologies	N/A
NGS-Lib-Fwd-3 (AATGATACGGCGACCACCGAGATCTACACTCTTTCCCTACACGACGCTCTTCCGATCTGATGCACATCTGCTTTATATATCTTGTGGAAAGGACGAAACACC)	Integrated DNA Technologies	N/A
NGS-Lib-Fwd-4 (AATGATACGGCGACCACCGAGATCTACACTCTTTCCCTACACGACGCTCTTCCGATCTCG	Integrated DNA Technologies	N/A

ATTGCTCGACGCTTTATATATCTTGTGGAAA GGACGAAACACC)		
NGS-Lib-Fwd-5 (AATGATACGGCGACCACCGAGATCTACAC TCTTTCCCTACACGACGCTCTTCCGATCTTC GATAGCAATTCGCTTTATATATCTTGTGGAA AGGACGAAACACC)	Integrated DNA Technologies	N/A
NGS-Lib-Fwd-6 (AATGATACGGCGACCACCGAGATCTACAC TCTTTCCCTACACGACGCTCTTCCGATCTAT CGATAGTTGCTTGCTTTATATATCTTGTGGA AAGGACGAAACACC)	Integrated DNA Technologies	N/A
NGS-Lib-Fwd-7 (AATGATACGGCGACCACCGAGATCTACAC TCTTTCCCTACACGACGCTCTTCCGATCTGA TCGATCCAGTTAGGCTTTATATATCTTGTGG AAAGGACGAAACACC)	Integrated DNA Technologies	N/A
NGS-Lib-Fwd-8 (AATGATACGGCGACCACCGAGATCTACAC TCTTTCCCTACACGACGCTCTTCCGATCTCG ATCGATTTGAGCCTGCTTTATATATCTTGTG GAAAGGACGAAACACC)	Integrated DNA Technologies	N/A
NGS-Lib-Fwd-9 (AATGATACGGCGACCACCGAGATCTACAC TCTTTCCCTACACGACGCTCTTCCGATCTAC GATCGATACACGATCGCTTTATATATCTTGT GGAAAGGACGAAACACC)	Integrated DNA Technologies	N/A
NGS-Lib-Fwd-10 (AATGATACGGCGACCACCGAGATCTACAC TCTTTCCCTACACGACGCTCTTCCGATCTTA CGATCGATGGTCCAGAGCTTTATATATCTTG TGGAAAGGACGAAACACC)	Integrated DNA Technologies	N/A
NGS-Lib-KO-Rev-1 (CAAGCAGAAGACGGCATACGAGATTCGCC TTGGTGACTGGAGTTCAGACGTGTGCTCTT CCGATCTCCGACTCGGTGCCACTTTTTCAA)	Integrated DNA Technologies	N/A
NGS-Lib-KO-Rev-2 (CAAGCAGAAGACGGCATACGAGATATAGC	Integrated DNA Technologies	N/A

GTCGTGACTGGAGTTCAGACGTGTGCTCTTCCGATCTCCGACTCGGTGCCACTTTTTCAA)		
NGS-Lib-KO-Rev-3 (CAAGCAGAAGACGGCATACGAGATGAAGAAGTGTGACTGGAGTTCAGACGTGTGCTCTTCCGATCTCCGACTCGGTGCCACTTTTTCAA)	Integrated DNA Technologies	N/A
NGS-Lib-KO-Rev-4 (CAAGCAGAAGACGGCATACGAGATATTCTAGGGTGACTGGAGTTCAGACGTGTGCTCTTCCGATCTCCGACTCGGTGCCACTTTTTCAA)	Integrated DNA Technologies	N/A
NGS-Lib-KO-Rev-5 (CAAGCAGAAGACGGCATACGAGATCGTTACCAGTGACTGGAGTTCAGACGTGTGCTCTTCCGATCTCCGACTCGGTGCCACTTTTTCAA)	Integrated DNA Technologies	N/A
NGS-Lib-KO-Rev-6 (CAAGCAGAAGACGGCATACGAGATGTCTGATGGTGACTGGAGTTCAGACGTGTGCTCTTCCGATCTCCGACTCGGTGCCACTTTTTCAA)	Integrated DNA Technologies	N/A
NGS-Lib-KO-Rev-7 (CAAGCAGAAGACGGCATACGAGATTTACGCACGTGACTGGAGTTCAGACGTGTGCTCTTCCGATCTCCGACTCGGTGCCACTTTTTCAA)	Integrated DNA Technologies	N/A
NGS-Lib-KO-Rev-8 (CAAGCAGAAGACGGCATACGAGATTTGAATAGGTGACTGGAGTTCAGACGTGTGCTCTTCCGATCTCCGACTCGGTGCCACTTTTTCAA)	Integrated DNA Technologies	N/A
NGS-Lib-SAM-Rev-1 (CAAGCAGAAGACGGCATACGAGATTCGCCTTGGTGACTGGAGTTCAGACGTGTGCTCTTCCGATCTGCCAAGTTGATAACGGACTAGCCTT)	Integrated DNA Technologies	N/A
NGS-Lib-SAM-Rev-2 (CAAGCAGAAGACGGCATACGAGATATAGCGTCGTGACTGGAGTTCAGACGTGTGCTCTTCCGATCTGCCAAGTTGATAACGGACTAGCCTT)	Integrated DNA Technologies	N/A
NGS-Lib-SAM-Rev-3 (CAAGCAGAAGACGGCATACGAGATGAAG	Integrated DNA Technologies	N/A

AAGTGTGACTGGAGTTCAGACGTGTGCTCTTCCGATCTGCCAAGTTGATAACGGACTAGCCTT)		
NGS-Lib-SAM-Rev-4 (CAAGCAGAAGACGGCATACGAGATATTCTAGGGTGACTGGAGTTCAGACGTGTGCTCTTCCGATCTGCCAAGTTGATAACGGACTAGCCTT)	Integrated DNA Technologies	N/A
NGS-Lib-SAM-Rev-5 (CAAGCAGAAGACGGCATACGAGATCGTTACCAGTGACTGGAGTTCAGACGTGTGCTCTTCCGATCTGCCAAGTTGATAACGGACTAGCCTT)	Integrated DNA Technologies	N/A
NGS-Lib-SAM-Rev-6 (CAAGCAGAAGACGGCATACGAGATGTCTGATGGTGACTGGAGTTCAGACGTGTGCTCTTCCGATCTGCCAAGTTGATAACGGACTAGCCTT)	Integrated DNA Technologies	N/A
NGS-Lib-SAM-Rev-7 (CAAGCAGAAGACGGCATACGAGATTTACGCACGTGACTGGAGTTCAGACGTGTGCTCTTCCGATCTGCCAAGTTGATAACGGACTAGCCTT)	Integrated DNA Technologies	N/A
NGS-Lib-SAM-Rev-8 (CAAGCAGAAGACGGCATACGAGATTTGAATAGGTGACTGGAGTTCAGACGTGTGCTCTTCCGATCTGCCAAGTTGATAACGGACTAGCCTT)	Integrated DNA Technologies	N/A
sgRNA Dnajc8 KO FW (CACCGTGGGTTCAGATTAAAGTAAG)	Sigma-Aldrich	N/A
sgRNA Dnajc8 KO RV (AAACCTTACTTTAATCTGAACCCAC)	Sigma-Aldrich	N/A
sgRNA Erap1 KO FW (CACCGAGGGCAGGTTCATCAAAGCA)	Sigma-Aldrich	N/A
sgRNA Erap1 KO RV (AAACTGCTTTGATGAACCTGCCCTC)	Sigma-Aldrich	N/A
sgRNA GanaB KO FW (CACCGAAGCTGCAGAGTGTCCAGCA)	Sigma-Aldrich	N/A

sgRNA GanaB KO RV (AAACTGCTGGACACTCTGCAGCTTC)	Sigma-Aldrich	N/A
sgRNA Gyk KO FW (CACCGAACGTGTCGAGCTGGTACCC)	Sigma-Aldrich	N/A
sgRNA Gyk KO RV (AAACGGGTACCAGCTCGACACGTTC)	Sigma-Aldrich	N/A
sgRNA Qa-1^b FW (CACCGGGCTATGTCATTCGCGGTCC)	Sigma-Aldrich	N/A
sgRNA Qa-1^b RV (AAACGGACCGCGAATGACATAGCCC)	Sigma-Aldrich	N/A
sgRNA Tap1 KO FW (CACCGCCTAGGACTAGGGGTCCGCG)	Sigma-Aldrich	N/A
sgRNA Tap1 KO RV (AAACCGCGGACCCCTAGTCCTAGGC)	Sigma-Aldrich	N/A
sgRNA Tap2 KO FW (CACCGGAGCACCTCAGTAGTCCGAG)	Sigma-Aldrich	N/A
sgRNA Tap2 KO RV (AAACCTCGGACTACTGAGGTGCTCC)	Sigma-Aldrich	N/A
sgRNA Tapbp KO FW (CACCGGATCGAGTGCTGGTTCGTGG)	Sigma-Aldrich	N/A
sgRNA Tapbp KO RV (AAACCCACGAACCAGCACTCGATCC)	Sigma-Aldrich	N/A

**Other reagents**		
FBS	Serana	Cat: S-FBS-SA-015
Hiload 26/600 Superdex 200	Cytivalifescience	Cat: 28989336
Hiload 26/600 Superdex 75	Cytivalifescience	Cat: 28989334
Iscove's Modified Dulbecco's Medium (IMDM)	Gibco	Cat: 12440
Lysis buffer (red blood cells)	In house pharmacy	N/A
Nuclease-free water	In house pharmacy	N/A
PBS	Fresenius Kabi	N/A

Protein A resin	Genscript	Cat: L00210
**Experimental models: Cell lines**		
B16F10	ATCC	Cat: CRL6475, RRID:CVCL_0159
Expi293F	Thermo Fisher Scientific	Cat: A14527, RRID:CVCL_D615
HEK293T	ATCC	RRID:CVCL_0063
K562	Yvonne Zoet	RRID:CVCL_0004
RAW264.7	Dr. F. Ossendorp	RRID:CVCL_0493
**Experimental models: organisms/strains**		
C57BL/6J	Charles River, The Netherlands	Cat: 632
Qa-1^{b} KO BL/6, B6.129S6-H2-$T23^{tm1Cant}$/J	Jackson Laboratories	Cat: 007907
BL21 E. Coli	NEB	Cat: C25271
STBL3 bacteria	Thermo Fisher	Cat: C737303
**Software and algorithms**		
BioRender	BioRender	http://biorender.com
FlowJo V.10	Treestar	https://www.flowjo.com/solutions/flowjo
GraphPad Prism V.10	GraphPad	http://www.graphpad.com
X500B QTOF	Sciex	https://sciex.com/products/mass-spectrometers/qtof-systems
OMIQ	Omiq Inc.	https://www.omiq.ai
PinAPL-Py	University of San Diego, California	http://pinapl-py.ucsd.edu
Resosens Ultra Mab-Pro Instrument	ResonantSensors inc.	https://www.resonantsensors.com/product

Resovu Software	ResonantSensors inc.	https://www.resonantsensors.com/product
