# Supplementary figures and images for: The murine MHC-E molecule Qa-1b is surface displayed in a peptide-free conformation in homeostasis
Source: Front Immunol. 2026 Mar 9;17:1743362. doi: 10.3389/fimmu.2026.1743362 (PMC13006831; doi:10.3389/fimmu.2026.1743362)

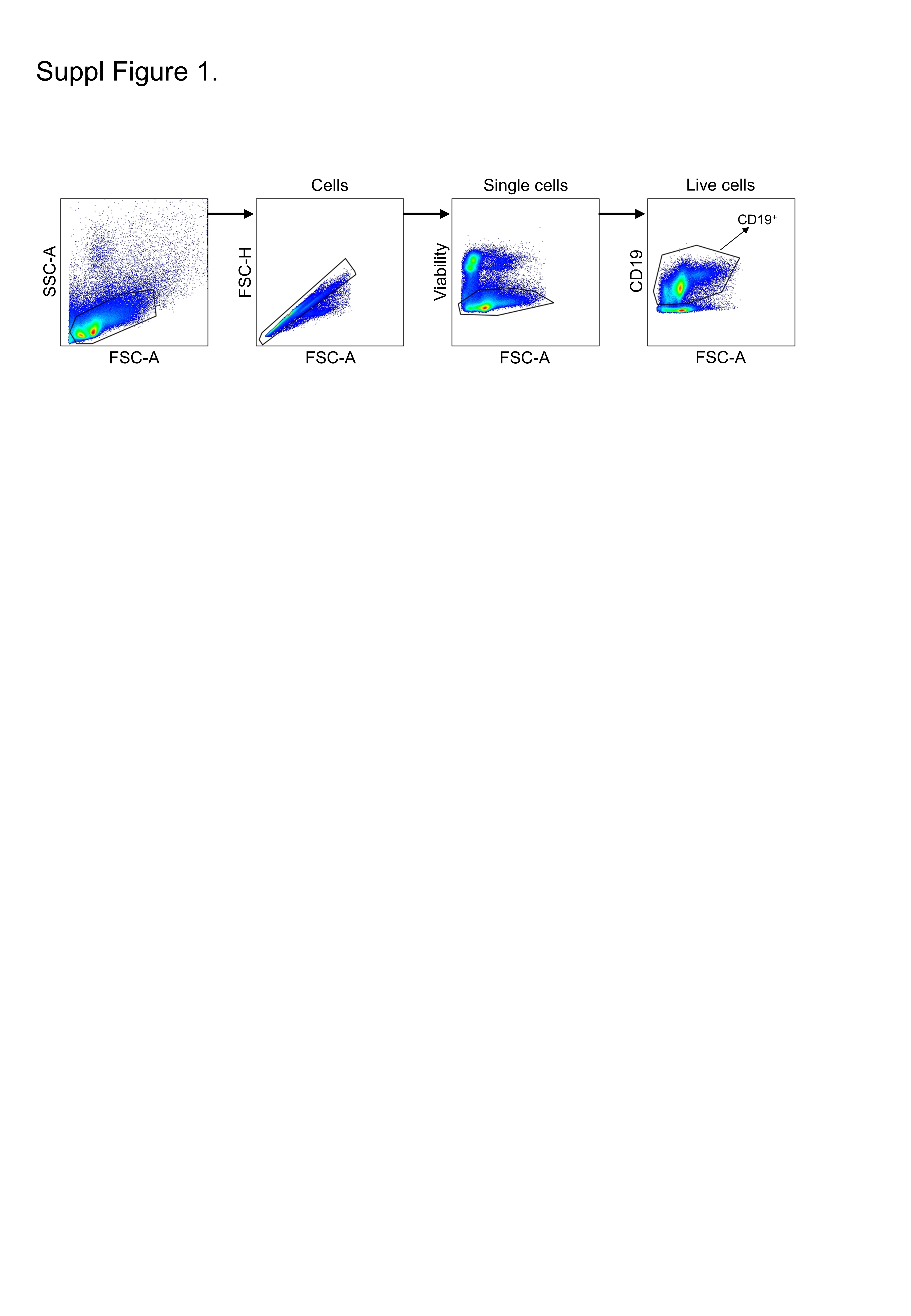

Supplement: Supplementary Figure 1 — Flow cytometry gating strategy. Flow cytometry pre-gating of the splenic B cells shown in Figure 1D. [file Image1.tif]

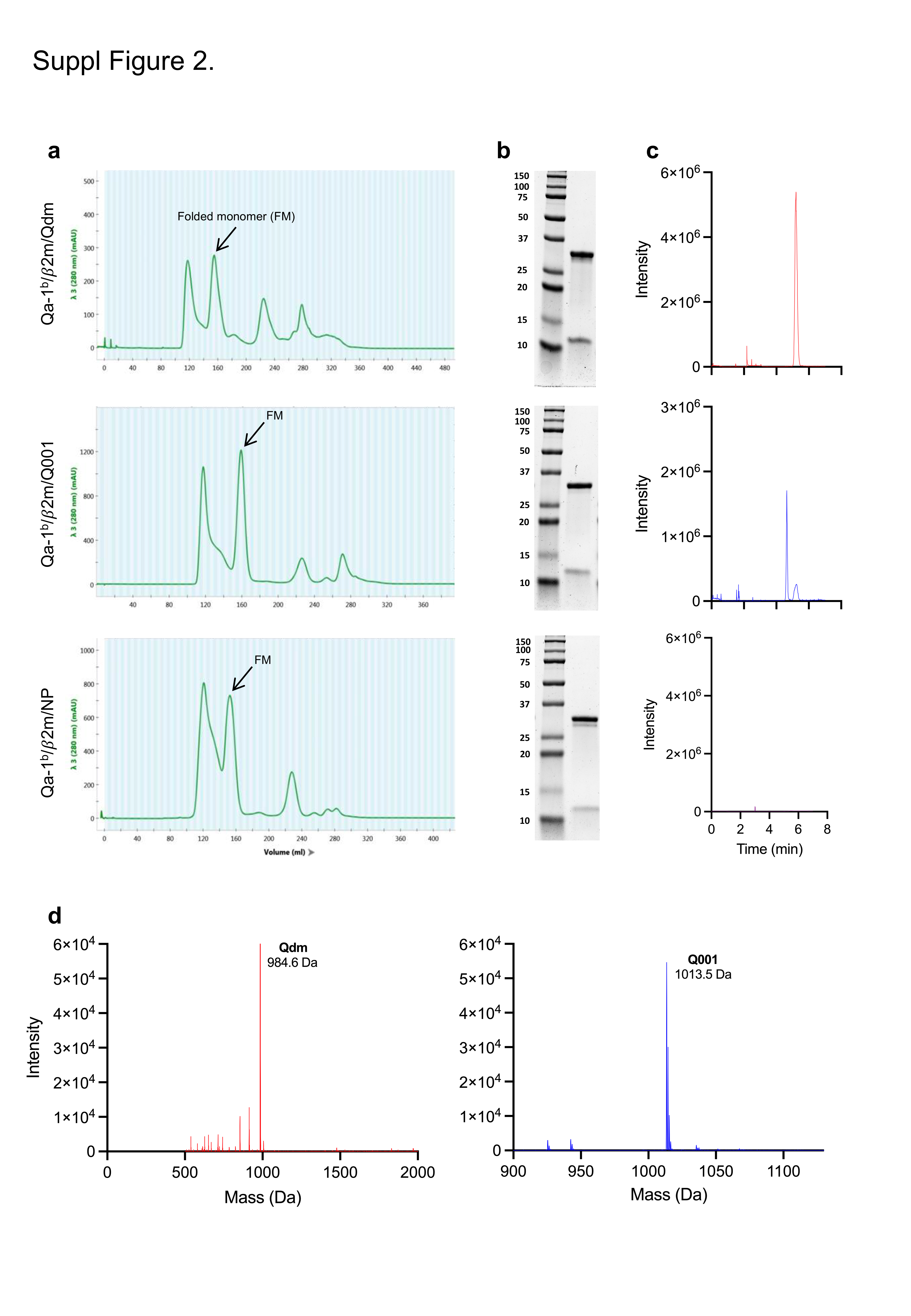

Supplement: Supplementary Figure 2 — Quality controls of protein production and formation of Qa-1b monomers. (A) Representative size-exclusion chromatography (SEC) profiles using Fast Performance Liquid Chromatography (FPLC) for purification of Qa-1b protein complexes. (B) SDS-PAGE analysis of purified correctly folded Qa-1b complexes under reducing conditions, confirming the presence of Qa-1b heavy chain (~34 kDa) and β2-microglobulin (~11 kDa) subunitsz. (C) Liquid chromatography (LC) chromatograms showing detection of peptides Qdm and Q001, but no detectable GroEL-derived peptide (NP). (D) Mass spectrometry analysis of selected LC fractions from (C), confirming the molecular masses corresponding to Qdm and Q001 peptides, validating their incorporation into Qa-1b complexes. [file Image2.tif]

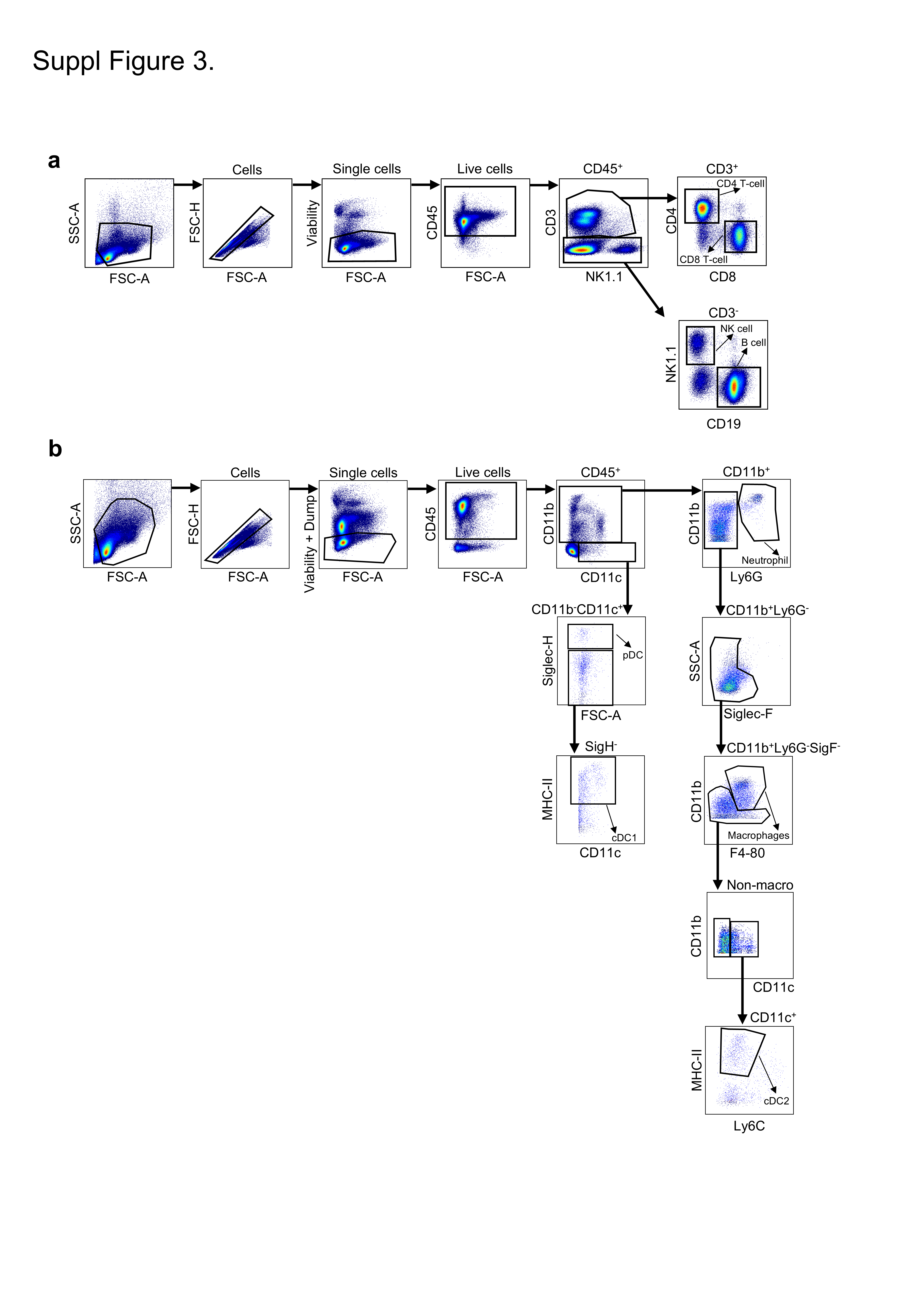

Supplement: Supplementary Figure 3 — Flow cytometry gating strategy. Flow cytometry pre-gating of the several splenocyte populations shown in Figure 6. (A) Lymphocyte staining panel and (B) myeloid cell staining panel, which included antibodies to CD3, CD19 and NK1.1 in the dump channel. [file Image3.tif]

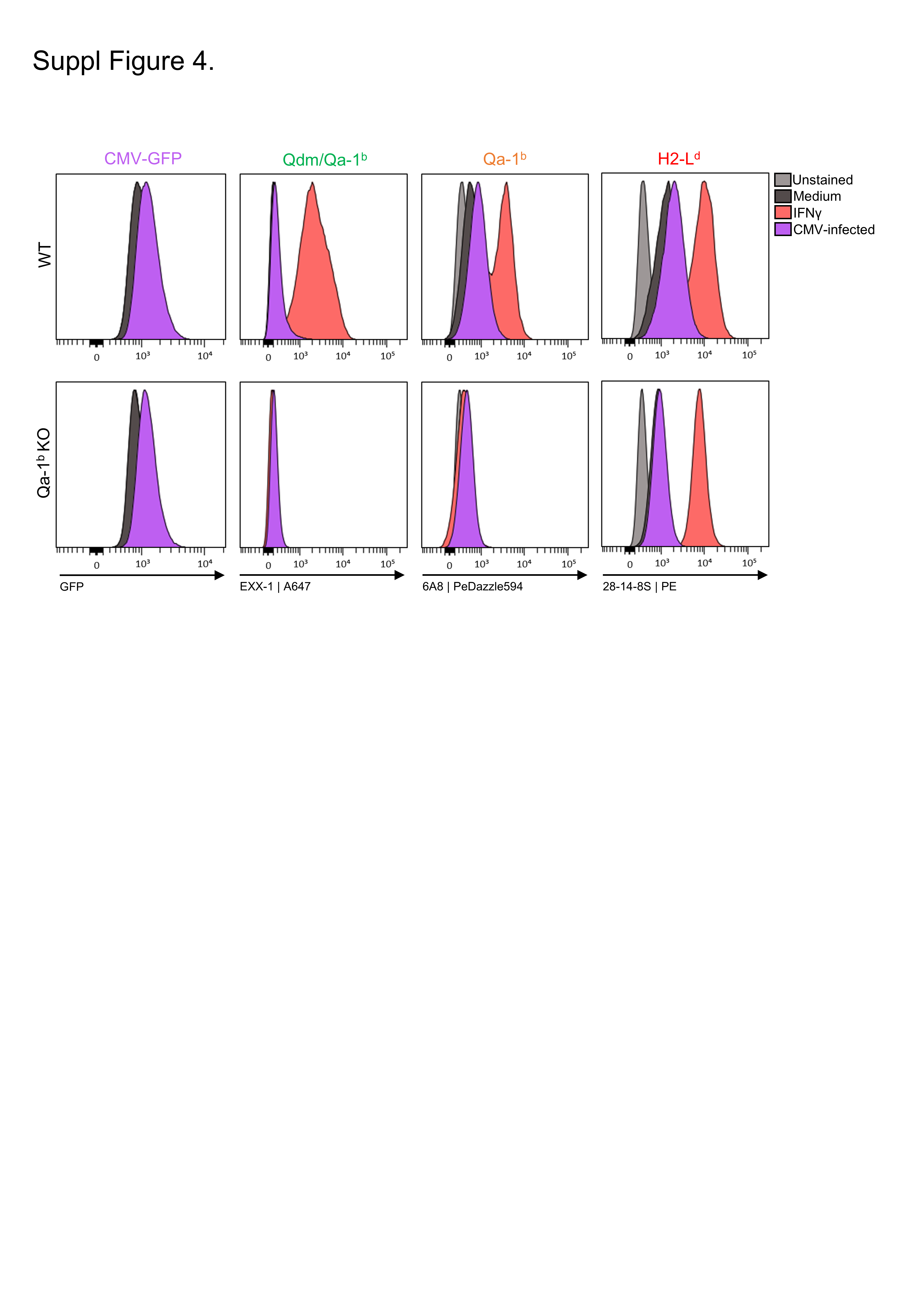

Supplement: Supplementary Figure 4 — Infection with MCMV increases staining with 6A8 antibody.RAW264.7 macrophages and their Qa-1b KO variants were infected by GFP-expressing recombinant MCMV at an MOI of 1 or incubated with 5 IU/mL IFNγ for 2 days. (B) Flow cytometry histograms of the RAW264.7 macrophages stained with EXX-1, 6A8 and 28-14-8S antibodies. [file Image4.tif]
